# Polyubiquitin gene *Ubb* is required for upregulation of Piwi protein level during mouse testis development

**DOI:** 10.1038/s41420-021-00581-2

**Published:** 2021-07-26

**Authors:** Bitnara Han, Byung-Kwon Jung, So-Hyun Park, Kyu Jin Song, Muhammad Ayaz Anwar, Kwon-Yul Ryu, Kwang Pyo Kim

**Affiliations:** 1 Department of Applied Chemistry, Institute of Natural Science, Global Center for Pharmaceutical Ingredient Materials, Kyung Hee University, Yongin, Republic of Korea; 2 Department of Life Science, University of Seoul, Seoul, Republic of Korea; 3 Department of Biomedical Science and Technology, Kyung Hee Medical Science Research Institute, Kyung Hee University, Seoul, Republic of Korea

**Keywords:** Proteomics, Proteomics

## Abstract

Testis development, including early embryonic gonad formation and late postnatal spermatogenesis, is essential for the reproduction of higher metazoans to generate fertile gametes, called sperm. We have previously reported that the polyubiquitin gene *Ubb* is required for fertility in both male and female mice. In particular, the *Ubb*-null male mice showed an azoospermia phenotype due to arrest of spermatogenesis at the pachytene stage. Here, we analyzed the whole testis proteome at postnatal day 20 to define the molecular mediators of the male-infertility phenotype caused by *Ubb* knockout. From the identified proteome, 564 proteins were significantly and differentially expressed in *Ubb-*knockout testes and, among these, 36 downregulated proteins were involved at different stages of spermatogenesis. We also found that levels of piRNA metabolic process-related proteins, including Piwil2 and Tdrd1, were downregulated in *Ubb*-null testes through functional gene ontology analysis. Further, protein–protein interaction mapping revealed that 24 testis development-related proteins, including Hsp90aa1, Eef1a1, and Pabpc1, were directly influenced by the depletion of ubiquitin. In addition, the reduced mRNA levels of these proteins were observed in *Ubb-*knockout testes, which closely resembled the global downregulation of piRNA-metabolic gene expression at the transcriptional and post-transcriptional levels. Together with proteomic and transcriptional analyses, our data suggest that *Ubb* expression is essential for the maintenance of testicular RNA-binding regulators and piRNA-metabolic proteins to complete spermatogenesis in mice.

## Introduction

Ubiquitin (Ub) is a highly conserved eukaryotic protein encoded by mono- and polyubiquitin genes in mammals [1–3]. In mice, two monoubiquitin genes (*Uba52* and *Uba80*) encode a single Ub fused with each ribosomal protein, and two polyubiquitin genes (*Ubb* and *Ubc*) comprise tandem repeats of 4 or 9 Ub-coding units, respectively [4]. Cellular Ub, which is synthesized *de novo*, is freely available for well-studied biochemical reactions defined as ubiquitination-catalyzed by E1, E2, and E3 enzymes [5]. So far, many studies have shown that hundreds of different intracellular proteins are monoubiquitinated or polyubiquitinated [6]. These complicated Ub signals are accurately recognized by receptor proteins that have Ub-binding domains, performing a wide range of biological functions, such as DNA repair, cell cycle regulation, protein degradation, and cell signal transduction [6]. Therefore, in order to maintain the intracellular Ub levels, monoubiquitin genes are always highly expressed, and polyubiquitin genes are also expressed at high levels, which may be regulated depending on the cellular condition [7–9]. In contrast, depletion of the intracellular Ub levels by knockout of *Uba52* or *Ubc* leads to failure in embryonic development in the early stages [10, 11].

From the early embryonic stage to postnatal adulthood, mouse testes continue to develop through several important steps [12]. In the early embryonic stage, migrating primordial germ cells (PGCs) arise from a specific region of the mesoderm and subsequently participate in sex determination [13]. In addition, testicular somatic cells differentiate into Sertoli cells and form embryonic gonads with PGCs [14]. After birth, the testicular somatic and germ cells proliferate and mature, and spermatogonia subsequently undergo spermatogenesis, including meiosis. Furthermore, epigenetic regulation, cell signaling, and endocrine systems are tightly controlled to achieve testicular development and successfully produce mature sperm [15].

Recent studies in the field of infertility have focused only on a single spermatogenic factor [16–18]. Few studies have profiled reproduction-related factors that are affected by *Ubb* at the protein level in mammalian testes. Lan-Tao et al. recently showed that the mutation in the D-box region of piwi-like protein (Piwil) reduced the binding of the Rnf8 during late sperm generation, resulting in degradation of Piwil and infertility in mice [17]. Li et al. confirmed that the interaction between Dazl and Pabpc1 using immunoprecipitation mass spectrometry [16]. The translation of many sperm-forming factors that are key to the progression of sperm formation is regulated by Pabpc1. In addition, the association between infertility and Dazl was confirmed at the single-protein level of cells arrested in the zygotene/pachytene-transition stage owing to the lack of Dazl. Huang et al. confirmed that 44 ubiquitinated proteins are associated with spermatogenesis using liquid chromatography–mass spectrometry (LC–MS) analysis of ubiquitinated peptides obtained through the affinity-enrichment method in the testes of adult buffalo [18].

We have previously reported that *Ubb-*knockout mice are viable but show two striking phenotypes: adult-onset hypothalamic neurodegeneration and infertility in both male and female mice [19, 20]. The infertility in *Ubb-*knockout mice is caused by the failure of gametes to form owing to developmental defects in the gonads following birth. In particular, the testes and ovaries of *Ubb-*knockout mice are smaller than those of wild-type (WT) mice, and the size differences increase as they become sexually mature [20]. Furthermore, meiotic cell cycle arrest in germ cells leads to azoospermia in *Ubb*-null male mice, whereas other somatic cells in the testes are normal. The expression level of *Ubb* in testicular germ cells is relatively higher than that in testicular somatic cells, leading to a more significant decrease in Ub levels [20]. Although there have been thorough investigations into the mechanism of developmental defects in *Ubb-*knockout testes, including transcriptome analysis, it is still unclear how reduced Ub levels lead to meiotic cell cycle arrest [21].

Here, we focused on WT and *Ubb*^−/−^ testes at postnatal day 20 (P20), which began to generate sperm, and at embryonic day 14.5 (E14.5) to investigate the molecular perturbation caused by cellular Ub deficiency. We used state-of-the-art mass spectrometry-based quantitative proteomics between WT and *Ubb*^−^^/^^*−*^ testes to identify key proteins related to meiotic cell cycle arrest in Ub-deficient spermatogonia. Our data provide a rich resource for profiling the mouse testis proteome and mapping the role of *Ubb* in various stages of spermatogenesis.

## Results

### Profiling of global proteins expressed in the testis of the *Ubb-* knockout mouse model

**Fig. 1:**
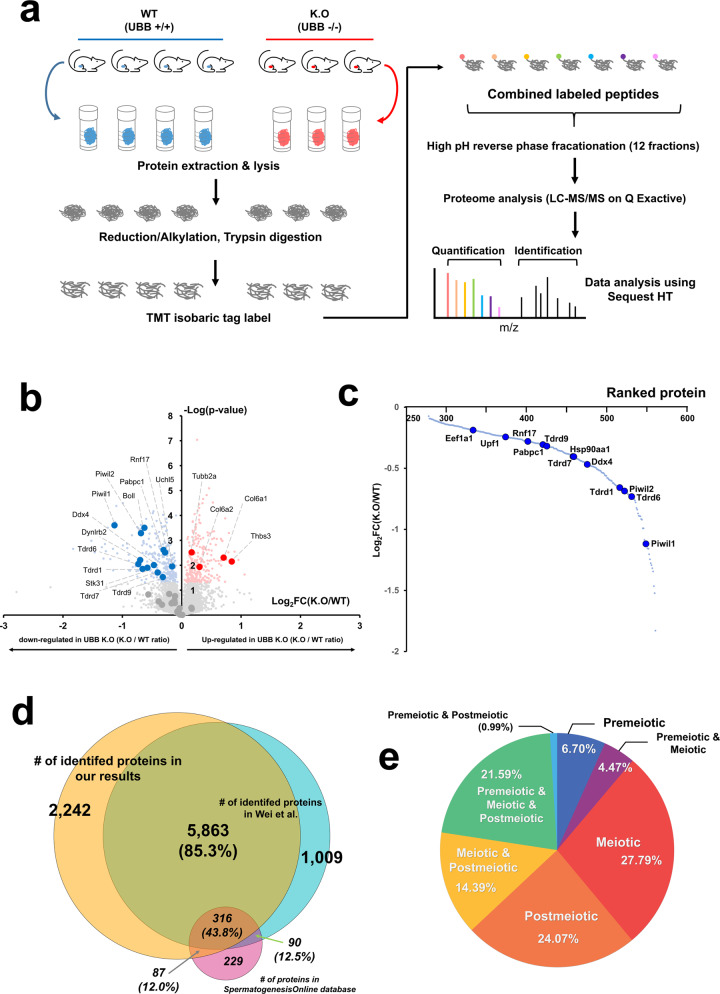
Overall experimental process and global proteome profiling results. **a** An overview of the method followed to process the tissue samples from the testis of four WT and three *Ubb*^−/−^ (KO) mice for proteome analysis. **b** The volcano plot representation for the identified proteome and differentially expressed proteins. Red and blue dots represent significantly up- and downregulated proteins, respectively, with statistical criteria (student’s *t*-test, *p*-value < 0.05; red is log2fc > 1, blue is downregulated). **c** Protein rank plot for downregulated proteins, based on their log2-transformed fold change. **d** Venn-diagram for proteins in Spermatogenesis database (red circle), proteins-identified in the current study by LC–MS/MS analysis (yellow circle), and proteins-identified in Wei et al., (blue circle). **e** Spermatogenesis Online database-based characterization of testicular cell type of profiled proteins.

Before proteomic analysis to identify the changes underlying *Ubb* disruption, we isolated testis tissues from four WT and three *Ubb*^−/−^ male mice at postnatal day 20. All seven samples were lysed, digested, labeled with TMT, and then pooled and analyzed using LC–MS/MS (Fig. 1a). A total of 8105 testicular proteins were identified, of which 6511 proteins could be quantified for relative comparison. Next, a two-sample *t*-test was performed and the proteins with a *p* value of < 0.05 and a ±1.2-fold change in their expression were considered significant and selected as DEPs. In total, 564 proteins were differentially expressed in *Ubb*^−/−^ testes compared with WT controls, including 277 upregulated and 287 downregulated proteins that satisfied the *p* value threshold (Fig. 1b, Supplementary table 2). To gain insights into the protein-ranked list influenced by *Ubb* disruption, the proteins that showed a difference in expression levels were ranked based on the value of log_2_ fold change (log_2_FC). Many of the proteins related to spermatogenesis (such as Piwil1, Tdrd6, Piwil2, Tdrd1, and Pabpc1) showed decreased expression levels in *Ubb*^−/−^ and were mostly ranked toward the edge (Fig. 1c). Comparing our data with that of Wei et al., we found that 5863 proteins matched with our data [22], and 403 proteins in the spermatogenesis database were identified [23] (Fig. 1d). Among the 403 spermatogenesis-related proteins, 108 proteins involved in the meiosis stage and 104 proteins involved in the post meiotic stage were identified using LC–MS/MS analysis (Fig. 1e, Supplementary table 3). Intriguingly, 36 proteins that were downregulated because of *Ubb* disruption were involved in various stages of spermatogenesis, consistent with our previous report on meiotic arrest due to Ub deficiency [16, 18, 21, 23, 24]. In addition, 11 proteins, including Eif4g3, Hsp90aa1, Tdrd9, and Sycp2, were involved with spermatocytes and showed abnormally lower levels of expression as compared to that of the WT control. The levels of eight proteins, including Ddx4, Tdrd6, Tdrd7, and Rnf17, involved in the spermatid stage of sperm maturation, were also downregulated (Supplementary table 3). Taken together, our proteomic profiling revealed that *Ubb* disruption perturbs a wide range of spermatogenesis-related biological functions.

### Functional enrichment gene ontology analysis of DEPs (WT vs. *Ubb-*knockout)

**Fig. 2:**
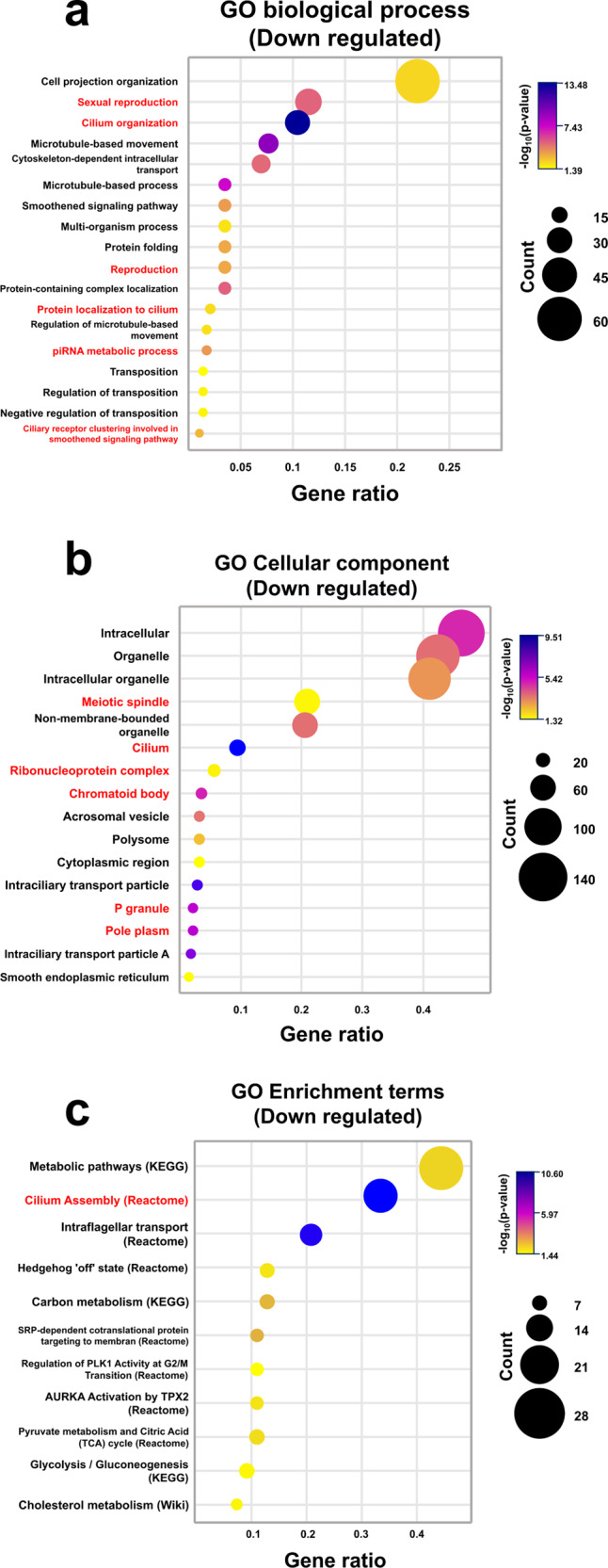
*Ubb* influence on the expression of spermatogenesis proteins. **a**, **b** Dot plot of gene ontology analysis results from downregulated DEPs enriched in biological processes and cellular components. Color indicates the g:scs correction -log(adjusted *p*-value), the circle size indicates the number of genes in that term, and the ratio of genes is in the category over total downregulated proteins. Categories related to spermatogenesis are shown in red. **c** Dot plot for Kegg pathway, reactome, and Wiki pathway-based categories enriched with downregulated proteins.

Next, we performed enrichment analysis to characterize the testicular proteins associated with *Ubb* disruption. GO analysis showed enrichment of DEPs in biological processes, cellular components, and various pathways in the *Ubb*-null state. For GO analysis, an open-source software, g:profiler, was used, and functional profiling of *Ubb*-related proteins was performed by statistical enrichment analysis sing the g:scs algorithm. Proteins with reduced expression owing to deletion of *Ubb* were primarily enriched in cilium-related biological processes, cellular components, and pathways, and the *p*-value obtained using g:SCS was very low. The enriched biological processes, such as sexual reproduction, cilium organization, and piRNA metabolic process, were actively downregulated in *Ubb*^−/−^ testes (Fig. 2a), whereas the upregulated proteins were mainly enriched in processes such as cellular component organization, developmental process, and cytoskeleton organization (Supplementary table 4). We also observed cellular components enriched in proteins with decreased abundance, including meiotic spindle, cilium, ribonucleoprotein complex, chromatoid body, and p-granule (Fig. 2b). Furthermore, the upregulated-proteins were associated with cell junctions, cell–cell contact zones, membrane regions, and extracellular matrix (Supplementary table 4). Notably, we observed decreased protein abundance-related to glycolysis/glucogenesis and cilium assembly. In addition, protein-enriched in Hedgehog “off”-state reactome encompassed the downregulated proteins (Fig. 2c). In contrast, upregulated proteins were enriched in extracellular matrix interaction, focal adhesion, and prostaglandin synthesis and regulation pathways. In addition, the proteins with increased abundance in our knockout model were related to the apoptotic execution phase and signaling by platelet-derived growth factor (PDGF) (Supplementary table 4).

### Interaction networks between spermatogenesis and *Ubb*-related testis proteins

**Fig. 3:**
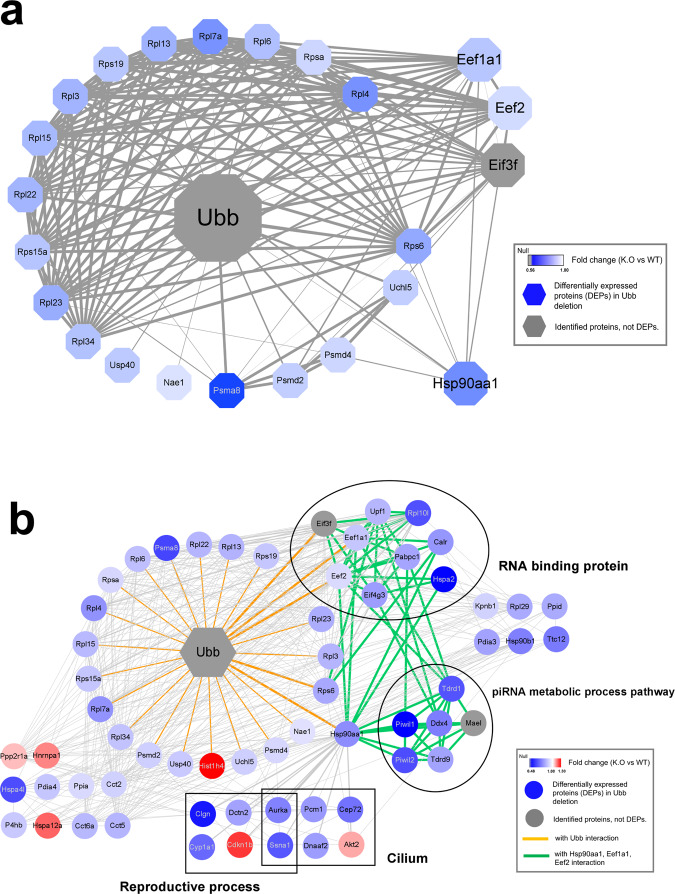
Network of proteins affected by *Ubb*. **a** Interaction between Ubb and significantly downregulated proteins because of the disruption of *Ubb*. In the interaction map, the thickness of the edge is proportional to the combined score. All interactions contribute to the combined score; higher scores correlate to more biologically meaningful evidence. The node color scale indicates the fold-change values, and nodes for proteins without significant differential expression are indicated with gray. **b** A network of Hsp90aa1, Eef1a1, and Eef2 proteins affected by the presence of *Ubb* and proteins related to fertility. The orange edges are the interaction between Ubb and the first shell, while green edges refer to the interacting partners of Hsp90aa1, Eef1, and Eif4g3. The proteins involved in RNA binding, piRNA metabolic process, cilia, and reproductive process are indicated with black circles and squares. This network has been created with STRING in cytoscape.

Gene ontology analysis showed that, *Ubb-*knockout affected the expression of proteins required for piRNA metabolism and sperm formation. To investigate the link between sperm formation and Ub, we constructed a protein-interaction network with the *Ubb*-encoded protein (Ubb) using the STRING database and Cytoscape software. Of the testis proteins identified by LC–MS/MS analysis, 296 interacted with Ubb (Supplementary table 5), of which 24 proteins (including Psma8, Hsp90aa1, Rps6, Eef1a1, Uchl5, and Eef2) are closely related to *Ubb* deletion (Fig. 3a). Interestingly, 24 DEPs that interact with Ubb were mainly enriched with molecular functions of RNA binding, organelle organization, cilium, reproduction, and ribonucleoprotein granules. In particular, it was found that Hsp90aa1, Eef1a1, Eef2, and Eif3f may be important for the maturation of meiotic cells and spermatocytes, through information from enriched biological processes, spermatogenesis online databases, and previous studies [18, 25–27] (Fig. 3b). From the protein–protein interaction network and proteome analysis, the following functional associations were observed: in our results, Hsp90aa1 protein interacting with Ubb, which upon deletion induces apoptosis of germ cells in adult mice, inhibits growth at the pachytene stage, and inhibits gamete formation [28], showed a significantly downregulated expression level in the Ubb-null model, and levels of proteins related to piRNA pathway components interacting with Hsp90aa1 were also affected by Ubb elimination (see Fig. 3b). Our results also implicated a wider role for Eef2, Eef1a1, and Eif3f, which interact with Ubb. Among the proteins that interact with these proteins, the expression levels of Hspa2 and Eif4g3 proteins were significantly reduced in the infertile group because of *Ubb-*knockout as compared to that in WT mice [29, 30].

### Altered expression of DEPs at the RNA level, including Piwil1 and Piwil2

Among the proteins affected by *Ubb* disruption, Hsp90aa1, Eef1a1, Eef2, and Eif3f are those that are largely related to spermatogenesis. All four proteins are associated with spermatogenesis markers, as reported in many studies [16, 18, 24]. In particular, levels of TDRD family proteins and Piwil1/2, which induce pachytene arrest when deficient in spermatocytes, were generally downregulated, and all these proteins interacted with the four key proteins mentioned above. Moreover, several DEPs in our data showed DNA-binding characteristics, including germ-cell-specific transcription factors, and misregulation of their gene expression may be critical for the failure of testicular development and growth arrest at the pachytene stage during meiosis in *Ubb*^−/−^ mice. In order to test this, we examined the altered expression of several up- and downregulated DEPs at the RNA level, based on the results of proteomic analysis. Upregulated proteins included extracellular matrix constituents and proteins related to PDGF signaling, which were highly expressed in testicular somatic cells, and downregulated proteins were related to piRNA metabolism and ciliogenesis, which are essential for meiotic progression of germ cells and spermatogenesis.

**Fig. 4:**
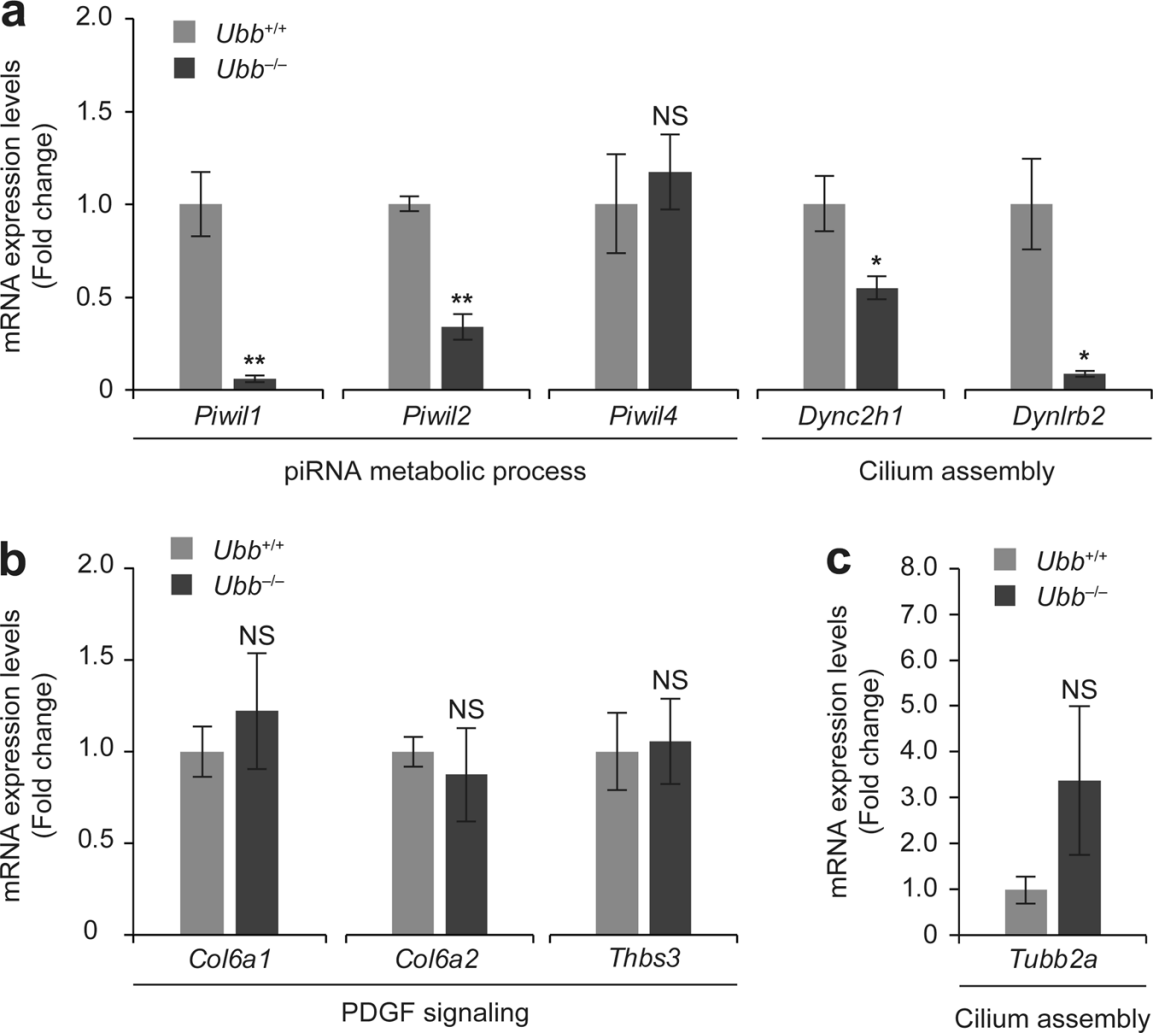
Altered gene expression of testicular DEPs. Based on proteomic analysis, several mRNA levels of *Ubb*^+/+^ (WT) and *Ubb*^−/−^ testis were determined using qRT-PCR. **a** Among downregulated proteins, expression levels of their genes involved in the piRNA metabolic process (*Piwil1*, *Piwil2*, and *Piwil4*) and cilium assembly (*Dync2h1* and *Dynlrb2*) were compared between *Ubb*^+/+^ and *Ubb*^−/−^ testis (*n* = 3 each). **b** Among upexpressed proteins, expression levels of their genes involved in PDGF signaling (*Col6a1*, *Col6a2*, and *Thbs3*) were compared between *Ubb*^+/+^ and *Ubb*^−/−^ testis (*n* = 3 each). **c** The expression level of *Tubb2a*, which was involved in cilium assembly, increased in *Ubb*^−/−^ testis as compared to that in WT testis (*n* = 3 each). All mRNAs used were isolated from *Ubb*^+/+^ or *Ubb*^−/−^ testes, which were collected from mice at postnatal day 20 (P20). All expression levels of genes were normalized to *Gapdh* level, and data are expressed as the mean ± SEM of indicated number of samples. *P < 0.05; **P < 0.01 vs. *Ubb*^*+/+*^. NS not significant.

We found that the RNA expression levels of *Piwil1* and *Piwil2*, which are involved in piRNA metabolism, and *Dync2h1* and *Dynlrb2*, involved in ciliogenesis, were significantly reduced in *Ubb*^−/−^ testes (Fig. 4a). However, there was no significant difference in the expression levels of the extracellular matrix constituents and the proteins involved in PDGF signaling, including *Col6a1* and *Thbs3*, between WT and *Ubb*^−/−^ testes (Fig. 4b). Their expression increased only at the protein level, which was probably due to the difference in the ratio of cellular composition in tissues by the relative increase in testicular somatic cell numbers and the decrease in germ-cell numbers in *Ubb*^−/−^ testes and the direct misregulation of the protein level owing to Ub deficiency. In fact, it was confirmed that the expression of *Tubb2a*, which was one of the upregulated proteins in *Ubb*^−/−^ testes, although it is involved in ciliogenesis, was also slightly, but not significantly, increased at the RNA level (Fig. 4c). Overall, our qRT-PCR results suggest that the expression of Piwil1 and Piwil2, which are highly expressed in the germ cells of developing testes, significantly decreased not only at the protein level but also at the RNA level in *Ubb*^−/−^ testes. Among the mouse Piwi protein family, Piwil1 is expressed only in differentiating sperm during the late stage of spermatogenesis, while Piwil2 is expressed throughout spermatogenesis [31]. Accordingly, the decreased expression of *Piwil2* may precede that of *Piwil1* during spermatogenesis in *Ubb*^−/−^ testes. In contrast to Piwil1/2, PDGF signaling proteins and extracellular matrix constituents, which are relatively highly expressed in testicular somatic cells (such as Sertoli cells and Leydig cells), were only increased at the protein level in *Ubb*^−/−^ testes.

### Increased expression of *Ubb* is essential for upregulation of Piwil2 level after birth

We found that levels of not only proteins essential for ciliogenesis (such as Dync2h1 and Dynlrb2) but also of late-expressed Piwi protein, especially Piwil1, decreased in *Ubb*^−/−^ testes. Because *Ubb*-null spermatogonia cannot progress into meiosis owing to pachytene arrest [20], downregulation of levels of these proteins at P20, when they are supposed to increase at the late stage of spermatogenesis, may result from the different populations of testicular germ cells between WT and *Ubb*^−/−^ testes. Furthermore, it is unclear whether *Ubb* expression is required for the upregulation of critical protein levels, including Piwil2, to pass the appropriate meiosis stages in testicular germ cells.

**Fig. 5:**
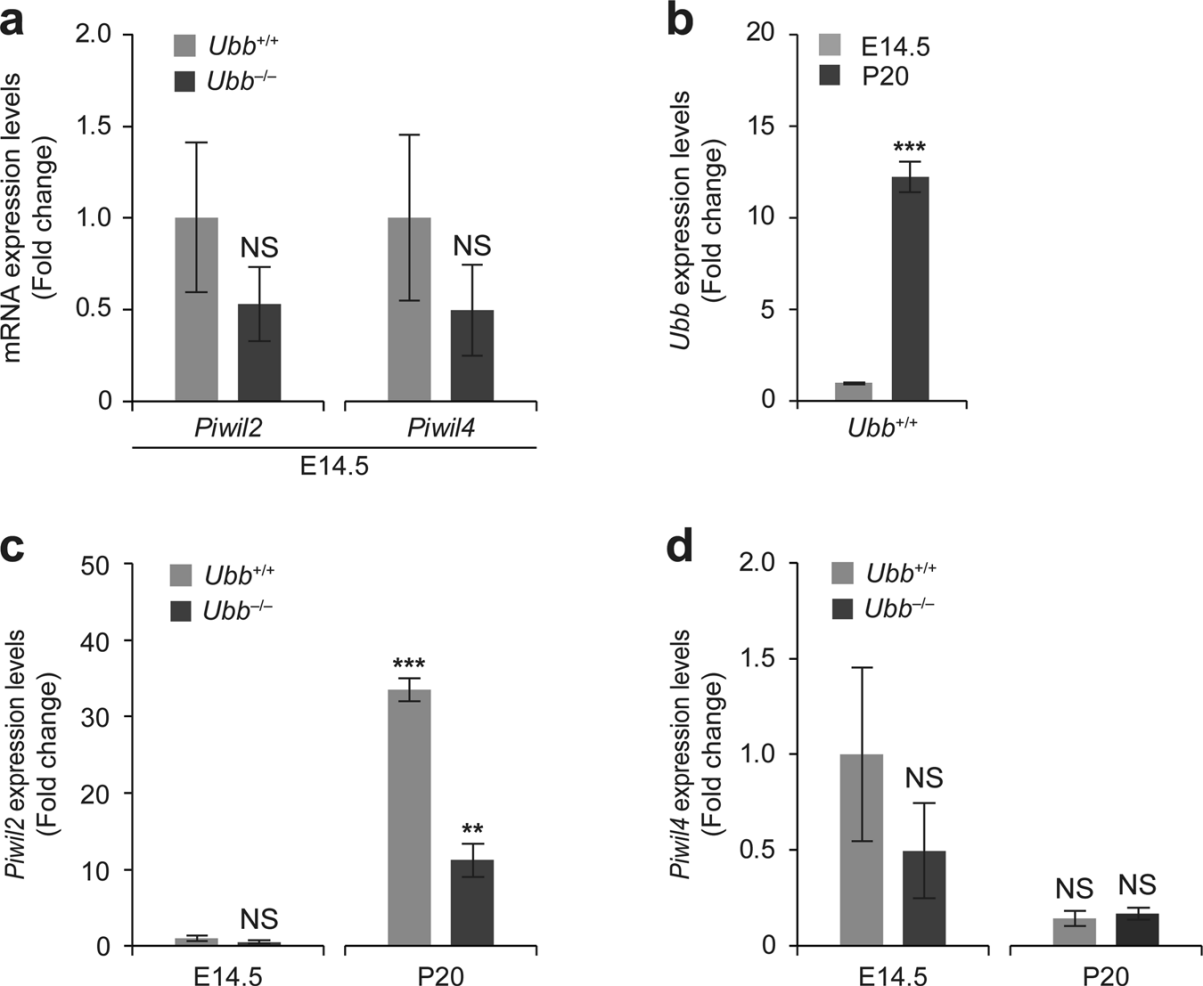
Increased expression levels of *Ubb* and *Piwi*l2 in postnatal development as compared to embryonic development. **a** Using mRNAs isolated from *Ubb*^+/+^ (WT) and *Ubb*^−/−^ gonadal ridges (*n* = 3 each) at 14.5 days of embryonic development (E14.5), mRNA levels of *Piwil2* and *Piwil4* were measured using qRT-PCR and normalized to *Gapdh* level. **b** *Ubb* expression levels of E14.5 and P20 *Ubb*^+/+^ testis (*n* = 3 each) were determined using qRT-PCR and normalized to *Gapdh* level. **c**, **d** For the relative comparison of mRNA levels between E14.5 gonadal ridges and P20 testis, the measured levels of *Piwil2* and *Piwil4* expression in *Ubb*^+/+^ and *Ubb*^−/−^ testis (*n* = 3 each) were presented as fold change relative to that in WT at E14.5. Data are expressed as the mean ± SEM of indicated number of samples. **P < 0.01; ***P < 0.001 vs. *Ubb*^*+/+*^ at E14.5. NS, not significant.

To investigate this, we isolated the gonadal ridges from WT and *Ubb-*null male embryos at 14.5 dpc and analyzed the RNA expression levels for *Piwil2* and *Piwil4*. In this period, *Piwil1* was not expressed, *Piwil2* was expressed at levels lower than that at P20, and *Piwil4* was highly expressed in the developing embryonic gonad [31]. We found that the expression levels of *Piwil2* and *Piwil4* were not significantly different between WT and *Ubb*^−/−^ gonadal ridges (Fig. 5a). Interestingly, the expression level of not only *Piwil4*, which is highly expressed from early embryonic development through birth, but also that of *Piwil2*, was not altered, despite *Ubb* disruption (Fig. 5a). Furthermore, we observed that *Ubb* and *Piwil2* expression was significantly upregulated in P20 testes as compared to that in embryonic gonadal ridges (Fig. 5b, c). This observation and our proteomic data raised the possibility that the infertility phenotype of *Ubb*^−/−^ male mice was caused by the misregulation or lack of dramatic upregulation of *Piwil2* expression during testis development between E14.5 and P20 (Fig. 5c). In fact, *Piwil2-*knockout mice also showed the pachytene-arrest phenotype during spermatogenesis [32]. Interestingly, *Piwil4* expression was downregulated during testis development, regardless of genotypes (Fig. 5d). Based on these results and proteomic data, we suggest that upregulation of *Ubb* expression during postnatal testicular development is required for meiotic progression and increased germ cell numbers through the upregulation of levels of proteins in the Piwi family and piRNA metabolism.

## Discussion

Polyubiquitin genes in mice are highly expressed in almost all cell types and throughout their life. When cells encounter specific environmental stimuli, such as oxidative stress, these genes are further upregulated to supplement readily available free Ub. Even under complex and elaborate processes such as embryonic development, the expression of polyubiquitin genes is essential for the differentiation of stem cells and tissue development. We previously reported that the straight phenotypes of the polyubiquitin gene *Ubb-*knockout mice are hypothalamic neurodegeneration and infertility in both male and female mice. Of the two polyubiquitin genes in mice, the expression levels of *Ubb* are relatively higher than that of *Ubc* in the germ cells of the testes; thus, Ub deficiency causes fatal defects in the pachytene stage during meiotic progression.

Here, we investigated the proteome of WT and *Ubb-*knockout testes at P20 to analyze the DEPs and their functions during the developmental processes. Contrary to our speculation that levels of a significant number of proteins were supposed to be upregulated in Ub-deficient testes, as the general function of Ub in cells is the degradation and quality control of proteins, only 562 proteins were significantly differentially expressed. We also found downregulated levels of a significant number of proteins (287), which were involved in piRNA metabolism and spermatogenesis. When a protein–protein interaction map was created, it showed that Ubb interacted with 267 proteins, of which, levels of 22 proteins were downregulated. Among the proteins with downregulated levels, Hsp90aa1, Eef2, Eef1a1, and Eif3f are critical and play different roles in spermatogenesis.

As reported previously, deletion of Hsp90aa1 induces apoptosis of germ cells in adult mice, arrests growth at the pachytene stage, and inhibits gamete formation [28, 33]. Also, Hsp90aa1 is associated with the biogenesis of Piwil through regulation of phosphorylation and piRNA loading into Piwil [25, 28]. In our results, the Hsp90aa1 protein that interacted with Ubb exhibited a significantly downregulated expression level in the *Ubb*-null model, and levels of proteins related to piRNA pathway components interacting with HSP90aa1 were also affected by *Ubb* elimination (see Fig. 3b). As shown in Fig. 3b, Hsp90aa1 closely interacted with Piwil1/2, which is involved in piRNA production, Ddx4, which is related to sperm motility, and piRNA pathway components, such as Tdrd family proteins, which are required for normal sperm formation, with a high combined score. In addition to Piwil1/2, Tdrd1, Tdrd9, and Hspa2 interacted with the Hsp90aa1 protein. These proteins are known to be essential for the maturation of germ cells into late spermatocytes and spermatids and, in the *Ubb*^−/−^ model, their levels were remarkably reduced [29, 34, 35] (Supplementary table 3). Conversely, levels of the Mov10l, which is highly expressed in normal pachytene cells and stabilizes the structure of Piwil protein [36, 37], and Fkbp6, which promotes recycling of Piwi protein, were not significantly different in the *Ubb* deletion model (see Supplementary table 2).

Our results also implicated a wider role for Eef2, Eef1a1, and Eif3f, which interact with Ubb. Eef2 and Eef1a1 interact with Ubb and show a significant difference in expression and are associated with Upf1 in one of the germ cell-specific perinuclear structures called chromatin bodies (CB) [38–40]. In addition, Rpl10l is essential for sperm formation, and its level was downregulated in the *Ubb*^−/−^ model. Rpl10l is a testis-specific retrogene and is an essential protein when transitioning from prophase to metaphase (meiosis I) in spermatocytes [41]. The level of Rpl10l, which is associated with all of Eef1a1, Eef2, and Eif3f, perpetuates transition from prophase to metaphase in spermatocytes, and also interacts with 60 s and 40 s ribosomal proteins downregulated in the *Ubb*^−/−^ model. The Rpl3/4/6 protein has also been shown to interact with the Pabpc1 protein, which regulates the translational activation of sperm-generating mRNA in the pachytene stage [42] (see Fig. 3a). Moreover, Eef1a1 interacted with Tdrd1, which regulates the recognition of piRNA mediators or cofactors for protein–protein interactions in the piRNA pathway by binding to Piwil2 of the Tudor family [34, 43, 44]. Eef1a1, Eef2, and Hsp90aa1 are commonly associated with Hspa2, which is also an essential protein in spermatogenesis and the development of spermatids [45]. Hspa2 interacts with Cdk1 and plays an essential role in the formation of the Cdk1/cyclin B1 complex in sperm cells; therefore, deficiency of Hspa2 indicated prophase arrest of pachytene spermatocytes during meiosis I [29].

Eif3f is one of the constituent proteins of the Piwi/Eif3f/Elavl1 supercomplex that regulates mRNA translation of Tudor domain family proteins in CB [42, 46, 47] and shows interactions with Ubb, but is independent of *Ubb* deletion [27]. Meanwhile, Elavl1, which interacts with Eif3f, also did not change its expression levels owing to the deletion of *Ubb*, but the expression of Eif4g3 and Pabpc1 proteins that form a supercomplex with Elavl1 was influenced by *Ubb*. Eif4g3 functions in the translation of Hspa2 and is required for male germ cells [30, 48]. Deletion of Eif4g3 affects Hspa2 translation and inhibits sperm formation during the meiosis stage, leading to infertility [30]. Pabpc1 showed the highest expression level in the pachytene and round-spermatid (RS) stages, and its deficiency can cause infertility [42]. Thus, our results showed a significant decrease in Pabpc1 protein level as compared to that in WT mice causing infertility because of *Ubb-*knockout, suggesting that this decrease in expression level may induce infertility along with changes in other protein levels. In addition, Pabpc1 interacts with the poly A tail of Dazl mRNA and acts as a translation regulator of proteins necessary for maturation in spermigonesis and spermatogonia, as well as in the meiosis stage [16].

Regarding pathways, expression of PDGF and extracellular matrix-related proteins was only different at the protein level, which could be linked to different numbers of cells, as *Ubb-*knockout caused a reduction in testis size. However, expression of piRNA metabolism and ciliogenesis-related genes was downregulated at the transcriptome level. This highlights the alteration of levels of proteins that regulate the transcription of proteins involved in piRNA metabolism and ciliogenesis. Piwi proteins are primarily involved in genome maintenance and regulation of the expression of various target genes [47]. Piwil2 expression was found to be downregulated at both the RNA and protein levels. This points to the plausible role of *Ubb*-mediated proteins in the transcriptional regulation of Piwil2. In summary, this study provided the proteome of *Ubb*^−/−^ testes and furnished a mechanism for *Ubb* knockout-mediated infertility. It also highlights the unappreciated role of Piwi-related proteins in spermatogenesis and testicular growth.

## Materials and methods

### Mouse

Mice were maintained in plastic cages with *ad libitum* access to food and water. *Ubb*^+/+^ and *Ubb*^−/−^ mice were obtained from the interbreeding of *Ubb*^+/−^ mice. All experiments were performed in accordance with the relevant guidelines and regulations approved by the University of Seoul Institutional Animal Care and Use Committee (UOS IACUC). All experimental protocols were approved by the UOS IACUC (UOS-170517-1, UOS IACUC-2020-03-A).

### Isolation testis and embryonic male gonad from *Ub*^+/+^ and *Ubb*^−/−^ mice

Testes were isolated from *Ubb*^+/+^ (WT) and *Ubb*^−/−^ mice at postnatal day 20 (P20) and placed in cold PBS to remove other adjacent tissues. Embryonic male gonads were isolated from WT and *Ubb*^−/−^ mice 14.5 days post coitum (dpc) with a dissection microscope and placed in cold PBS to remove other embryonic tissues. After isolation, P20 testes and E14.5 male gonads were immediately frozen in liquid nitrogen and stored at −80 °C for further experiments.

### Protein extraction and tryptic digestion

The left testis tissue was individually cryopulverized using a Cryoprep device (CP02; Covaris, USA). Each tissue piece was placed in a cryovial (430487; Covaris, USA) on dry ice, transferred to a Covaris tissue bag (TT1; Covaris, USA), placed in liquid nitrogen, and pulverized at an impact level of 3 for 30 s. The powder from each tissue was then placed in a sonication tube (002109; Covaris, USA) and mixed with 600 μL of lysis buffer (RIPA buffer [89900; Thermo Fisher Scientific, USA], 534 μL; 100 × Halt™ Protease Inhibitor Cocktail [78430; Thermo Fisher Scientific, USA], 6 μL; 10 × PhosSTOP™ [4906845001; Roche, Swiss], 60 μL). Tissue lysis was performed by sonication using a focused ultrasonicator (S220; Covaris, USA) at a setting of 2 W (intensity 5) for 5 s, followed by 36 W (intensity 10) for 20 s and 0 W (intensity 0) for 10 s. The sonication cycle was repeated 20 times at 16 °C. The homogenate was centrifuged at 16,000 × *g* at 4 °C for 10 min, and the supernatant was transferred to a new tube. Protein concentration was measured using the Pierce™ BCA Protein Assay Kit (23227; Thermo Fisher Scientific, USA). Each 80 µg of protein was digested individually using the Suspension-Trapping (S-Trap) filter (C02-mini-80; Protifi, USA) method [49]. Proteins from each sample were denatured and reduced with 5% sodium dodecyl sulfate (SDS) and 20 mM 1,4-dithiothreitol (DTT) (10708984001; Roche, Swiss), boiled at 95 °C for 10 min, and then alkylated with 40 mM iodoacetamide (IAA) (I16125-10 g; Sigma-Aldrich, USA) for 30 min at room temperature (RT) in the dark. Next, a final concentration of 1.2% phosphoric acid (695017-100 ML; Sigma-Aldrich, USA) and 6 volumes of binding buffer (90% methanol and 100 mM triethyl ammonium bicarbonate) were added to the sample and mixed gently to form colloidal protein particles. The colloidal protein solution was loaded onto the S-Trap filter and rotated at 4,000 × g for 30 s, and the flow-through was collected and reloaded into the filter. The filter was then washed 2–3 times with 400 μL of binding buffer. Seven samples were subjected to digestion with Pierce™ Trypsin Protease (90057; Thermo Fisher Scientific, USA) at a 1:20 enzyme-to-substrate ratio and incubated overnight at 37 °C. The peptides were eluted with three stepwise buffers: (1) 80 μL of 50 mM triethylammonium bicarbonate (TEAB) (90114; Thermo Fisher Scientific, USA), (2) 80 μL of 0.2% formic acid (56302-50 ML; Fluka, USA) in H_2_O, and (3) 80 μL of 50% acetonitrile and 0.2% formic acid, with centrifugation at 4000 × *g* for 30 s. The resulting tryptic peptides were dried using a Speed-Vac (Hypercool, Labex, Republic of Korea) and kept at −80 °C, until tandem mass isobaric tag (TMT) (A44520; Thermo Fisher Scientific, USA) labeling.

### TMT labeling of peptides and basic pH reverse-phase fractionation

Peptides were labeled with seven different mass tags among the Tandem Mass Tag (TMT) reagents (90111; Thermo Fisher Scientific, USA) as follows: the four peptide samples from WT testis tissue were labeled with 126 N, 127[N/C], and 128 N TMT reagents, and the three peptide samples from *Ubb*^−/−^ testis tissue were labeled with 128 C, 129 C, and 130 N TMT reagents. The prepared TMT reagent was transferred to the peptide sample, and the mixture was incubated for 1 h at RT following brief vortexing. The reaction was quenched with 5% hydroxylamine and incubated for 30 min at RT. All TMT-labeled peptides in the batch were pooled and concentrated by vacuum centrifugation. Labeled peptides were loaded on an analytical column (XBridge Peptide BEH C18 Column; 300 Å, 5 µm, 4.6 × 250 mm, 186003625; Waters™, USA) for fractionation. A gradient was generated using an Ultimate 3000 HPLC system (Dionex, USA) operated with solvent A (4.5 mM ammonium formate [17843-50 G; Fluka, USA] [pH 10] in 2% [vol/vol] acetonitrile); and solvent B (4.5 mM ammonium formate [pH 10] in 90% [vol/vol] acetonitrile). The gradient was as follows: 0–7 min, 0% B; 7–13 min, 16% B; 13–73 min, 40% B; 73–77 min, 44% B; 77–82 min, 60% B; 82–96 min, 60% B; 96–110 min, 0% B [50]. The separated peptides were collected every 1 min into 96 tubes and non-consecutively pooled into 12 fractions by combining eight parts. The resulting 12 fractions were desalted using a Pierce C18 spin column (89870; Thermo Fisher Scientific, USA) (Fig. 1a).

### LC–MS/MS analysis for global proteome

For LC–MS analysis, a nanoACQUITY UPLC system (Waters™, USA) was coupled to a Q Exactive Hybrid Quadrupole-Orbitrap Mass Spectrometer (Thermo Fisher Scientific, Germany) and equipped with a trap column (Acclaim™ PepMap™ 100 C18 LC Column, C18, 75 μm × 2 cm, 5 μm, 164564; Thermo Fisher Scientific, USA) for cleanup followed by an analytical column (EASY-Spray™ LC Columns, C18, 75 μm × 50 cm, 2 μm, ES803A; Thermo Fisher Scientific, USA). The peptides were separated using a mobile phase comprising solvent A (0.1% formic acid and 2% acetonitrile in water) and solvent B (0.1% formic acid in 90% acetonitrile). For global proteome-digested peptides, the labeled peptides were trapped using 100% solvent A for 4 min at a flow rate of 3 μL/min, and the optimized linear-gradient elution program was set as follows (T min:% of solvent B): 0:3, 4:3, 7:12, 143:40, 144:80, 154:80, 155:5, and 180:5. The flow rate was 300 nL/min throughout the run time. Full MS scans were acquired for the mass range of 350–1,800 m/z at a resolution of 70,000 in MS1 level and an automatic gain control (AGC) target of 1e6, and MS/MS analysis was performed by data-dependent acquisition in positive mode. The electric potential of electrospray ionization was set to 2.2 kV, and the temperature of the capillary was set to 250 °C. The top 12 precursor peaks were selected from the MS1 scan and separated for fragmentation. For MS2 acquisition with high-energy collisional dissociation (HCD), the resolution was set to 35,000 with a fixed first m/z of 100 m/z, an AGC target of 1e6, an isolation window of 2.0 m/z, an isolation offset of 0.5 m/z, and a normalized collision energy (NCE) of 32%. The charge states of unassigned, 1, or >6 were discarded, and dynamic exclusion for 30 s was enabled.

### Protein database searching and quantification of global proteomic data

The MS/MS spectra were searched against a composite database of Uniprot *Mus musculus* reference (May 2020; 21,989 entries) using the Sequest HT search engine. The search was restricted to fully tryptic peptides, allowing up to two missed cleavage sites. In modification, carbamidomethylation, alkylation of disulfide bonds in cysteine (+57.021 Da), TMT modification of lysine, and N-termination (+229.163 Da) were noted as static modifications. Oxidation of methionine (+15.995 Da) was used as a variable modification. The false-discovery rate (FDR) for peptide level was set to 0.01 for removing false-positive data and to quantify each reporter ion, using the “peptide and protein quantifier” method in Proteome Discoverer 2.4 (Thermo Fisher Scientific, USA).

### RNA isolation and quantitative reverse transcription–polymerase chain reaction

Quantitative reverse transcription–polymerase chain reaction (qRT-PCR) was performed as previously described, with slight modifications [11]. Briefly, total RNA was isolated from cells using Tri-reagent (Molecular Research Center, USA) following the manufacturer’s protocol, and 10 ng of total RNA was used as a template for reverse transcription using SuperScript reverse transcriptase (Enzynomics, Republic of Korea) and oligo-dT (Cosmogenetech, Republic of Korea). For qRT-PCR, we used SYBR-Green qPCR Mastermix (Enzynomics, Republic of Korea) and an iCycler system (Bio-Rad, USA). The mRNA expression levels of target genes, including *Ubb*, *Piwil1*, *Piwil2*, *Piwil4*, *Dync2h1*, *Dynlrb2*, and *Thbs3*, were normalized to *Gapdh* levels. The sequences of the primers used for qRT-PCR are listed in Supplementary table S1.

### Statistical analysis and gene enrichment analysis

For the enrichment pathway analysis of all identified proteins, we classified proteins with more than ±1.2-fold expression changes in a two-tailed Student’s t-test as differentially expressed proteins (DEPs) with a p-value < 0.05. Annotation enrichment processes were performed for the significantly upregulated or downregulated DEPs. Gene Ontology (GO) searches were performed to explore the biological processes and cellular components affected by *Ubb-*knockout using g:Profiler [51]. The GO biological process enhanced by DEPs was found to be FDR < 0.05. To reconstruct the network model for DEPs, protein–protein interaction information was collected from the STRING v11.0 public database [52]. The network model used Cytoscape to depict the enhanced process and interaction data.

## Supplementary information

Supplementary table 1 & information

Supplementary table 2

Supplementary table 3

Supplementary table 4

Supplementary table 5
