# Supplementary material for: Polyubiquitin gene Ubb is required for upregulation of Piwi protein level during mouse testis development
Source: Cell Death Discov. 2021 Jul 26;7:194. doi: 10.1038/s41420-021-00581-2 (PMC8313548; doi:10.1038/s41420-021-00581-2)
Supplement: Supplementary file 1 — Supplementary table 1 & information [file 41420_2021_581_MOESM1_ESM.pdf]

supplementary information

supplementary table s1

Primer sequence used in qRT-PCR

Genes		Sequence (5' – 3')
Gapdh	F	GGC ATT GCT CTC AAT GAC AA
	R	CTT GCT CAG TGT CCT TGC TG
Ubb	F	TCT GAG GGG TGG CTA TTA A
	R	TGC TTA CCA TGC AAC AAA AC
Piwil1	F	CAG GAT AGG ACT GGA CGC T
	R	TTT TCC AGT CAG CTC AGG TGT T
Piwil2	F	GGC TGG AAT AGG AGG GAA AGG
	R	GGC TGT AGA AAC GAC TGT TGG
Piwil4	F	CAC CCA GGA ACA TGA GTG GA
	R	GTA CGT CCA CCA TCA CCA GA
Thbs3	F	TTT CCA CGA GCA GCG TTC A
	R	GAA ACA GGG GTC AGC GTG AG
Col6a1	F	CGT TCT GAC ACT CAA CGG GA
	R	GAG ATA CCT GGC CGA CCT TG
Col6a2	F	AAG ACG TCC TTT GTC CAG ACC
	R	ACC TTG TGG AAG TTC TGC TCG
Dync2h1	F	AGC ATT GAG CCA TGT ACA AAA GT
	R	GCA CAT AGA AAG AAG GTG ACT CT
Dynlrb2	F	ACA AAG GGG TCA TCG GAA CG
	R	GGT GGA GAA GAC CCG CAT AC
Tubb2a	F	AGG CGG AGA GCA ACA TGA AT
	R	GAA AGA CCA TGC TGG AGG ACA
F: Forward, R: Reverse		

supplementary table s2

Differentially expressed protein list

supplementary table s3

Among the identified proteins, those that overlap with the proteins in the Spermatogenesis database

supplementary table s4

GO functional enrichment analysis of upregulated proteins. (biological process, cellular components, molecular functions, pathways)

supplementary table s5

Interaction of 296 proteins with the *Ubb* protein and interaction between *Ubb* and 24 significantly expressed proteins.
