# Supplementary material for: Polyubiquitin gene Ubb is required for upregulation of Piwi protein level during mouse testis development
Source: Cell Death Discov. 2021 Jul 26;7:194. doi: 10.1038/s41420-021-00581-2 (PMC8313548; doi:10.1038/s41420-021-00581-2)
Supplement: Supplementary file 2 — Supplementary table 2 [file 41420_2021_581_MOESM2_ESM.pdf]

Table of Contents

Accession	UniProt identifier
Gene name	Gene symbol
# AAs	Number of amino acids in the protein
MW [kDa]	Molecular weight of in the protein
calc. pI	Isoelectric point value of the protein
TMT reporter ion intensity_replicate1 (R1_WT 1 to 4)	Normalized data (Repoter ion intensity) for the first LC-MS/MS analysis of four wildtype biological replications
TMT reporter ion intensity_replicate1 (R1_K.O 1 to 3)	Normalized data (reporter ion intensity) for first LC-MS/MS analysis of biological replicates of three Ubb knockout models
TMT reporter ion intensity_replicate1 (R2_WT 1 to 4)	Normalized data (Repoter ion intensity) for the second LC-MS/MS analysis of four wildtype biological replications
TMT reporter ion intensity_replicate1 (R2_K.O 1 to 3)	Normalized data (reporter ion intensity) for second LC-MS/MS analysis of biological replicates of three Ubb knockout models
WT average	Average of Wildtype samples' repoter ion intensity of 2 technical replicate and 4 biological replicates
KO average	Average of Ubb K.O samples' repoter ion intensity of 2 technical replicate and 4 biological replicates
p-value (KO vs WT)	The p-value obtained by performing a student t-test for all WT and K.O replicates.
FC (KO vs WT)	Ratio of Ubb K.O reporter ion average intensity versus WT reporter ion average intensity

Supplemental Table S2a. The mouse testis global proteome identified by TMT labeled LC-MS/MS analysis

* **Four biological replicates for the wild type (WT) left testis sample, the three biological replicates for the *Ubb* knockout (KO) sample.**

Accession	Protein name (8105)	Gene name	# AAs	MW [kDa]	calc. pI	Normalized intensity_replicate1							Normalized intensity_replicate2						
						N R1 WT1	N R1 WT2	N R1 WT3	N R1 WT4	N R1 KO1	N R1 KO2	N R1 KO3	N R2 WT1	N R2 WT2	N R2 WT3	N R2 WT4	N R2 KO1	N R2 KO2	N R2 KO3
P62983	Ubiquitin-40S ribosomal protein S27a	Rps27a	156	17.9	9.64	1545232.2	1932845.4	2159847.0	2074326.2	2323548.9	2130184.9	1540229.6	2476117.7	1772729.4	2005154.0	2192629.5	2678535.3	2299303.7	2479711.8
P62984	Ubiquitin-60S ribosomal protein L40	Uba52	128	14.7	9.83	#N/A	#N/A	#N/A	#N/A	#N/A	#N/A	#N/A	#N/A	#N/A	#N/A	30995.8	32489.2	27224.2	29747.3
Q9JHU4	Cytoplasmic dynein 1 heavy chain 1	Dync1h1	4644	531.7	6.42	8593953.9	9407980.9	8911886.5	9595484.8	9155609.2	8605457.6	7999246.2	8404104.9	9066725.6	9480849.3	10472333.7	11181520.7	11115530.5	11137175.5
E9Q616	PDZ domain-containing protein	Ahnak	5656	603.9	6.3	4810917.8	4659813.8	4741093.9	4254823.6	5635599.9	6381408.6	6014657.8	5319506.6	4680857.8	4726571.2	4804142.4	4672162.6	4457975.6	4483863.9
P16546	Spectrin alpha chain, non-erythrocytic 1	Sptan1	2472	284.4	5.33	5969137.4	5596908.3	5864205.8	5519835.6	7047027.1	7199924.9	7888800.4	6385203.8	6101962.9	5990397.3	5002546.3	4951192.6	4831373.0	4531992.1
O08638	Myosin-11	Myh11	1972	226.9	5.45	7422812.6	6830466.7	7241997.1	6829413.2	7166400.4	7740170.9	9405202.4	10259393.1	7115841.7	6810269.9	8636019.3	7957564.2	8244860.4	7874221.6
Q9QXS1	Plectin	Plec	4691	533.9	5.96	3247727.6	3211396.1	3041303.0	3184490.6	3341545.3	3375758.9	3525077.2	3256118.3	3135233.2	3193201.9	3681554.5	3762934.1	3523290.6	3539919.0
E9PWQ3	Collagen, type VI, alpha 3	Col6a3	3284	353.7	6.93	9511701.6	8770166.6	8290812.4	8236407.2	11690208.2	15510425.3	17913532.5	12558382.6	9825657.0	8652350.8	11094857.5	9681556.2	9155942.6	9237310.8
P26039	Talin-1	Tln1	2541	269.7	6.18	2846205.2	2903610.1	2721761.5	2901274.8	2891495.1	2884207.4	3166599.3	2778839.3	2793309.4	2858939.8	2961828.8	2935747.0	2891896.6	2929721.8
Q8VDD5	Myosin-9	Myh9	1960	226.2	5.66	4247015.5	3808629.2	3859329.4	3621198.5	4156105.9	4381486.3	4425916.6	4429881.4	3832225.4	3894831.8	5415789.4	4809232.7	4904904.2	4612518.4
P19096	Fatty acid synthase	Fasn	2504	272.3	6.58	6434029.4	6012735.8	6123496.2	5729063.3	6343033.1	5734446.3	5636692.3	6253455.6	6280410.7	6303642.0	7066882.2	6797747.9	6681784.1	6315683.4
Q68FD5	Clathrin heavy chain 1	Cltc	1675	191.4	5.69	6101723.4	6320353.6	6622263.8	6581875.5	6435349.1	6090153.0	5958073.2	6294145.4	6149502.2	6112109.2	5317054.3	5417280.3	5549094.7	5585672.3
Q01853	Transitional endoplasmic reticulum ATPase	Vcp	806	89.3	5.26	15875962.6	15012440.7	16356218.1	15958419.7	16654526.8	15622290.3	16949382.3	15316005.3	16329273.0	16275640.5	13015005.0	12101875.8	13995327.1	13293901.7
Q64727	Vinculin	Vcl	1066	116.6	6	5141989.2	4509505.8	4793644.5	4269539.4	5294746.0	5612924.2	5803362.9	5329166.5	5330411.2	5033885.4	8148756.2	7090619.8	7657482.7	6709474.1
P07724	Serum albumin	Alb	608	68.6	6.07	145966599.7	107346867.1	93270972.8	89907750.3	71428625.0	113479186.4	137427621.7	146919542.0	97529427.9	84584707.6	123703844.5	92622743.5	75568126.7	74158429.1
Q62261	Spectrin beta chain, non-erythrocytic 1	Sptbn1	2363	274.1	5.58	4007683.7	3665751.6	3820316.0	3578622.6	4527672.5	4902621.0	5500410.8	4313605.5	3934266.0	3806894.5	3313956.7	3076437.3	3083670.9	3061110.8
Q8BTM8	Filamin-A	Flna	2647	281	6.04	2992215.3	3123494.2	2794876.1	3024093.9	2656430.2	3192230.5	3147439.6	3905042.8	2781428.0	2776222.0	3270223.1	3236919.5	2856239.9	3438533.3
A2ANY6	Midasin	Mdn1	5589	629.9	5.72	1399144.9	1529280.8	1485836.2	1501764.7	1546584.6	1512280.6	1306332.4	1374761.3	1470337.8	1548274.4	1954477.1	2028276.6	1969544.5	1899204.9
E9PVA8	eIF-2-alpha kinase activator GCN1	Gcn1	2671	292.8	7.36	2154899.4	2210385.4	2059066.5	2173557.6	1987504.4	1901566.6	1887548.0	1976215.8	2143936.5	2147884.7	1812356.8	1855934.2	1794888.2	1856139.9
Q6ZWQ0	Nesprin-2	Syne2	6874	782.2	5.33	2176090.4	2100149.4	2018870.8	2168540.3	2135851.2	2097277.8	2259647.5	1981710.3	2089700.0	2144307.8	2075667.4	1863013.0	2002484.7	1951862.2
P07901	Heat shock protein HSP 90-alpha	Hsp90aa1	733	84.7	5.01	21656138.2	25923418.9	28968897.6	28674698.1	22072551.8	20299244.5	17693574.9	22383325.5	23972617.0	24796123.1	24163059.4	27752851.7	29723517.5	30035073.1
Q6P4T2	U5 small nuclear ribonucleoprotein 200 kDa helicase	Snrnp200	2136	244.4	6.06	2928429.9	2842667.5	3025372.0	2895304.7	3066424.2	2963432.5	2868542.0	2848745.1	3053372.6	3035347.1	1751254.0	1877073.8	1857448.0	1871529.9
Q61879	Myosin-10	Myh10	1976	228.9	5.54	1106907.0	1087556.2	1174628.4	1056184.5	1164798.7	1342165.8	1104684.8	1153468.6	1058753.7	1076312.5	1011523.0	969437.2	1136247.4	966266.7
P58252	Elongation factor 2	Eef2	858	95.3	6.83	13798432.3	12955543.0	13773550.4	12199138.4	13034262.9	12045594.3	11409920.0	14028471.7	13073966.8	13523755.3	13576578.2	13033336.1	13678915.0	12645023.6
Q9WTQ5	A-kinase anchor protein 12	Akap12	1684	180.6	4.44	2364783.6	2197624.7	2230367.9	2186664.6	2490563.2	2378077.5	2710873.9	2295004.4	2407804.4	2336244.3	2533559.5	2420142.8	2500360.0	2394909.5
P63038	60 kDa heat shock protein, mitochondrial	Hspd1	573	60.9	6.18	6207655.3	5510454.2	5869690.7	5992167.0	5952223.0	5511836.6	5848891.5	5820727.2	5737936.0	5914594.2	5077971.2	5142683.2	4936544.9	5111549.5
P20029	Endoplasmic reticulum chaperone BiP	Hspa5	655	72.4	5.16	17947766.8	18396765.9	21826425.4	20279512.1	18654052.0	17635573.5	13064372.0	16834347.5	20315682.6	19500401.6	24185937.9	25267288.1	29702697.5	28536681.5
P11499	Heat shock protein HSP 90-beta	Hsp90ab1	724	83.2	5.03	13417421.5	12706382.5	13552094.8	11385505.7	12502112.5	12414850.5	12268200.9	13918425.9	13445950.5	12250074.6	12381928.6	11489516.1	12088030.6	10651322.4
Q01320	DNA topoisomerase 2-alpha	Top2a	1528	172.7	8.6	3849287.2	4405616.3	4879722.0	4528297.6	4586284.1	4501991.1	3146022.2	3521162.5	4501070.5	4531306.4	3629298.7	4123024.1	4615357.8	4328719.2
P08113	Endoplasmin	Hsp90b1	802	92.4	4.82	21520444.5	20811138.3	22067242.4	22685408.4	17863586.0	16174847.1	13749608.7	20077786.3	20006833.9	19807490.1	16647534.4	16852367.1	18131059.0	18377816.2
Q9QXZ0	Microtubule-actin cross-linking factor 1	Macf1	7354	831.4	5.43	1087412.2	1243789.1	1144533.2	1181161.2	1124007.9	1167382.1	1024390.8	1149277.3	1092457.8	1129587.2	1385150.9	1463456.7	1356374.1	1406870.6
Q99PV0	Pre-mRNA-processing-splicing factor 8	Prpf8	2335	273.4	8.84	3195101.4	3373154.3	3454199.0	3339838.9	3624755.7	3431560.1	3240323.3	3171183.5	3441155.4	3608646.6	3798301.3	3971200.3	4363160.4	3940768.4
P63017	Heat shock cognate 71 kDa protein	Hspa8	646	70.8	5.52	42489557.0	41728413.7	47663853.6	47369933.3	37957775.8	38342678.0	35762815.2	39621669.3	42757612.2	42204986.0	21166747.8	22963579.9	22759785.5	22523445.6
F6ZDS4	Nucleoprotein TPR	Tpr	2431	273.8	5.03	1995531.4	2084373.2	2133935.1	1985018.2	2350281.8	2287935.8	2226659.2	1943618.6	2110540.4	2140310.3	1951884.6	1769496.1	2102794.2	1812154.3
Q921I1	Serotransferrin	Tf	697	76.7	7.18	18052054.3	15528132.9	12409927.3	11742925.2	9164864.4	12186008.6	15448851.4	17106879.4	13072294.4	12586540.3	15809401.3	13200491.7	10943815.7	9917037.5
Q61316	Heat shock 70 kDa protein 4	Hspa4	841	94.1	5.24	4803261.6	4914840.1	5444884.8	5116087.2	4655107.4	4550035.7	4017508.3	4455801.4	4874975.7	4923712.8	3255323.3	3715472.9	3428116.0	3585248.9
P52480	Pyruvate kinase PKM	Pkm	531	57.8	7.47	10603233.6	10035795.6	9812508.2	9725653.6	9610913.0	8984534.4	9948870.5	10105001.3	10565707.9	10373060.1	11290900.9	10939671.4	10366156.6	10645716.8
E9Q7G0	Nuclear mitotic apparatus protein 1	Numa1	2094	235.5	5.87	1475650.5	1393743.7	1485911.3	1351610.3	1712358.1	1695772.3	1741321.9	1437047.3	1512711.8	1506812.0	1440550.6	1353283.5	1408663.5	1394315.0
Q02053	Ubiquitin-like modifier-activating enzyme 1	Uba1	1058	117.7	5.66	5676946.6	5334375.3	5644901.9	5294095.5	5699740.6	5706739.7	5485854.8	5401715.1	5795964.5	5767788.0	5789522.7	5589855.0	5663832.1	5564988.4
A2AGT5	Cytoskeleton-associated protein 5	Ckap5	2032	225.5	7.96	2136142.2	2432430.5	2352030.6	2318376.8	2324562.9	2458005.5	1953889.2	2101025.2	2370827.0	2382372.1	1956491.2	1977480.5	2115476.4	1944830.9
A2AN08	E3 ubiquitin-protein ligase UBR4	Ubr4	5180	571.9	6.06	1268441.5	1276099.2	1284489.8	1239156.5	1297137.0	1295407.6	1173628.5	1244240.1	1270126.2	1278216.4	1810007.5	1856367.8	1893347.8	1807664.0
P20152	Vimentin	Vim	466	53.7	5.12	25592389.1	18745434.1	22397594.8	18249835.0	29197988.1	28111948.7	28313568.2	25282451.8	22146762.1	20800002.0	29095622.8	20678684.9	25790200.9	20684115.8
P01027	Complement C3	C3	1663	186.4	6.73	3053994.3	2678749.9	1989054.7	2481027.2	1451281.5	1684589.6	2021505.8	2771012.2	2092323.5	2090475.7	4020480.8	3642334.5	2816293.1	3310080.2
Q9ERU9	E3 SUMO-protein ligase RanBP2	Ranbp2	3053	340.9	6.18	1752838.5	1812580.5	1822958.7	1751258.6	2025499.3	2009072.7	1834555.4	1656684.5	1870390.1	1878766.3	1241420.5	1313240.6	1268732.4	1263006.8
B2RQC6	CAD protein	Cad	2225	243.1	6.43	2600731.2	2212310.4	2417882.0	2187204.5	2376476.8	2128724.9	2105221.4	2545744.1	2147769.6	2277353.0	2207094.3	2228776.9	2244776.7	2117647.5
O55143	Sarcoplasmic/endoplasmic reticulum calcium ATPase 2	Atp2a2	1044	114.8	5.34	2003548.1	1990917.2	2093228.4	2085794.2	1964423.7	1951783.5	1899638.3	2154538.4	1982055.2	2092032.0	1731437.6	1821375.3	1888675.8	1855512.7
Q61554	Fibrillin-1	Fbn1	2873	312.1	4.92	1348284.9	1299205.6	1357596.1	1149424.6	1916768.2	2390390.9	2226629.3	1574195.2	1461308.8	1455670.3	1244948.7	1294435.3	1230204.3	1156435.8
Q8CGC7	Bifunctional glutamate/proline--tRNA ligase	Eprs1	1512	170	7.66	3177772.7	3212508.1	3484764.9	3192359.9	3486502.5	3345317.1	2960150.5	3252545.5	3307168.0	3348848.5	2387422.6	2471419.7	2627657.4	2440230.6
P14733	Lamin-B1	Lmnb1	588	66.7	5.16	4227527.7	3798467.2	4387229.4	3818788.2	5439972.5	5619759.6	7250892.7	4551022.3	4811446.1	4782212.0	3973778.8	3778066.7	3990786.2	3713820.5
P80314	T-complex protein 1 subunit beta	Cct2	535	57.4	6.4	2847009.7	2985123.8	3240600.3	3058482.2	2917689.0	2888495.9	2587103.7	2729412.1	3034906.7	3120502.5	2970295.9	3330986.6	3359567.7	3359869.2
P56480	ATP synthase subunit beta, mitochondrial	Atp5f1b	529	56.3	5.34	8753886.2	8601342.1	8569506.9	8427301.7	8797305.9	8660627.4	8585043.8	8853625.4	8542628.1	8541356.1	9174896.7	7981719.7	8581099.1	8930891.3
Q9CW03	Structural maintenance of chromosomes protein 3	Smc3	1217	141.5	7.18	1724954.0	1792687.7	1818978.8	1680780.7	2129925.2	2146805.0	1924087.9	1738131.0	1889162.2	1932188.3	1105279.5	1179008.5	1189994.5	1085268.5
P48722	Heat shock 70 kDa protein 4L	Hspa4l	838	94.3	5.74	1462708.1	1837054.2	1906894.2	2145347.5	1284250.4	1225545.0	1096300.0	1320504.5	1596089.8	1655013.7	1603271.2	2009039.4	2189899.9	2485612.8
P17156	Heat shock-related 70 kDa protein 2	Hspa2	633	69.6	5.67	5551653.6	7546019.4	8171347.0	10174692.0	4249231.9	3349965.0	2885415.8	4599145.5	6149626.2	7395757.4	5677697.8	8823440.3	8573508.7	10896138.8
Q45VK7	Cytoplasmic dynein 2 heavy chain 1	Dync2h1	4306	492	6.6	734678.7	940554.5	902014.9	1022613.4	666901.4	653830.1	626592.2	666270.6	786031.5	849884.9	833328.0	964925.6	978346.6	1045105.0
Q99KI0	Aconitate hydratase, mitochondrial	Aco2	780	85.4	7.93	4428428.1	4328664.5	4533011.3	4592196.2	4674405.8	4400343.0	4651803.6	4333735.5	4685404.9	4726687.4	4289982.4	4160758.6	4310281.2	4337347.8
O70133	ATP-dependent RNA helicase A	Dhx9	1380	149.4	6.83	2703802.3	2800682.0	3189387.4	2981815.2	3243039.6	3009347.7	2974725.6	2880528.6	3032560.0	3099481.4	2572891.6	2695720.2	3083971.1	2888403.6
Q9JKR6	Hypoxia up-regulated protein 1	Hyou1	999	111.1	5.19	2982517.1	3377990.3	3409532.9	3573736.1	2888870.5	2794009.0	2420022.3	2941000.6	3114729.1	3107687.0	4053392.1	4670975.7	4566944.6	5086528.6
P99024	Tubulin beta-5 chain	Tubb5	444	49.6	4.89	24427057.3	22805602.1	24157128.1	21770075.1	24896237.0	22860600.8	26010195.3	23949157.5	25284337.5	25509171.3	19819636.5	19026440.3	21324532.6	19887976.5
P27773	Protein disulfide-isomerase A3	Pdia3	505	56.6	6.21	11793947.3	12557654.4	12628327.2	12880700.4	10801025.0	10135504.6	9245376.6	12195260.8	11476079.8	11951953.2	11858738.7	13505229.8	12764719.5	14083099.5
Q61768	Kinesin-1 heavy chain	Kif5b	963	109.5	6.44	1306461.2	1301423.8	1252065.8	1242041.3	1283974.6	1230434.7	1270873.7	1275794.3	1270363.1	1205377.2	1631147.0	1493238.1	1574842.7	1576105.0
E9Q555	E3 ubiquitin-protein ligase RNF213	Rnf213	5152	584.4	6.8	1164619.7	1159027.0	1167785.5	1102047.7	1138971.4	1158912.7	1160710.1	1150665.5	1163375.0	1150871.7	915857.6	936601.5	842611.9	866950.8
P17182	Alpha-enolase	Eno1	434	47.1	6.8	8538577.3	7754701.1	8019383.8	7310036.6	8118630.4	8092731.7	9366960.0	8966643.3	8675451.1	8247025.2	7180948.8	6579146.1	6484999.5	6352241.1
Q7TMY8	E3 ubiquitin-protein ligase HUWE1	Huwe1	4377	482.3	5.22	1229661.7	1293401.6	1279181.2	1228174.5	1353161.0	1357566.9	1230833.4	1225720.7	1261469.7	1338630.1	1137797.3	1157362.9	1136566.7	1099423.7
P68372	Tubulin beta-4B chain	Tubb4b	445	49.8	4.89	1767866.8	1892665.8	1981153.3	1750842.1	2032510.2	2062619.9	2014331.6	1583418.0	2029000.3	1991433.0	1714677.1	1897701.3	1878340.9	1755127.9
Q8BMS1	Trifunctional enzyme subunit alpha, mitochondrial	Hadha	763	82.6	9.14	5050235.1	4716413.1	4709869.1	4874556.6	4978111.9	4632880.2	4823185.8	4829219.5	4801848.0	4913663.3	4292636.2	4170655.1	4176378.6	4174741.1
Q99MD9	Nuclear autoantigenic sperm protein	Nasp	773	83.9	4.37	2285047.3	2611925.8	3125549.8	2994904.3	2694705.8	2451415.9	2011558.4	2238093.0	2650263.4	2713972.9	2499506.2	3278010.3	3184826.8	3483495.9
E9Q0C6	Acrosomal protein KIAA1210	Kiaa1210	1637	180.9	8.7	2236241.0	2213030.9	1908237.3	2188313.2	1673044.4	1476728.0	2139909.3	1851389.6	2030278.1	1991851.7	2710239.7	2732216.1	2448797.2	2699572.7
P68369	Tubulin alpha-1A chain	Tuba1a	451	50.1	5.06	158267.1	164926.1	163251.6	166796.6	178561.6	164875.6	157036.3	161461.4	163497.3	167047.1	15884482.8	18843461.4	18327605.6	17029268.0
Q8BWT1	3-ketoacyl-CoA thiolase, mitochondrial	Acaa2	397	41.8	8.09	2047405.0	1815704.5	1769593.6	1773387.8	1789572.0	1828274.6	1831815.9	1849413.3	1785142.2	1763923.4	2769227.1	2365679.9	2341228.5	2425588.3
Q6ZQ38	Cullin-associated NEDD8-dissociated protein 1	Cand1	1230	136.2	5.78	3841927.8	3980145.8	4485174.3	4376087.7	3993221.6	3753487.4	3381082.8	3572726.7	4164169.1	4179279.9	3808642.2	4094262.1	4422414.7	4234160.7
Q6PDI5	Proteasome adapter and scaffold protein ECM29	Ecpas	1840	203.6	7.06	1105557.9	1199109.0	1160392.1	1100498.9	1191674.1	1159938.7	1055657.9	1111609.0	1194898.4	1213142.1	1168456.9	1223064.7	1150960.2	1126106.8
P40142	Transketolase	Tkt	623	67.6	7.5	4690905.9	3753078.2	4317999.4	3797941.0	5105321.8	5635586.8	6538781.7	5010903.6	4562761.5	4386633.9	5105763.6	4132026.3	4622279.0	4179927.1
P27546	Microtubule-associated protein 4	Map4	1125	117.4	4.98	1391792.8	1281900.5	1240952.6	1204967.8	1382663.6	1398824.1	1455202.6	1330849.1	1302555.5	1275810.3	1163713.9	1091543.6	1150135.1	1117199.1
Q8VDN2	Sodium/potassium-transporting ATPase subunit alpha-1	Atp1a1	1023	112.9	5.45	2156746.0	2114060.0	2121069.0	2050100.5	2300474.9	2314027.9	2470810.1	2228303.1	2138516.9	2120832.4	2465394.6	2416704.6	2300671.9	2267509.4
A2AX52	Collagen alpha-4(VI) chain	Col6a4	2309	250.6	6.81	1030298.2	1015013.7	1042200.1	946864.6	1234101.6	1360929.4	1536639.8	1055641.6	1095492.8	1112464.0	1042289.1	1001218.4	1052982.2	948242.5
Q8C7R4	Ubiquitin-like modifier-activating enzyme 6	Uba6	1053	117.9	6.11	1620199.6	1815665.5	1839693.0	1681219.1	1889145.7	1894483.5	1551687.9	1603683.3	1778641.1	1781219.8	1370110.5	1577609.9	1533745.2	1503640.9
Q6P5E4	UDP-glucose:glycoprotein glucosyltransferase 1	Uggt1	1551	176.3	5.62	1336360.3	1392160.6	1261273.9	1414284.1	1247269.4	1236516.3	1160535.6	1213090.9	1266001.8	1260280.0	2014068.6	2013635.8	1913456.7	2048181.7
A6H584	Collagen alpha-5(VI) chain	Col6a5	2640	289.4	6.57	1387733.1	1291991.4	1220259.6	1350823.6	1474848.5	2011392.7	2077915.9	1823643.9	1320414.6	1240047.3	1002243.0	967535.8	906115.9	982065.2
P60710	Actin, cytoplasmic 1	Actb	375	41.7	5.48	1710757.0	1856533.1	1817574.7	1617130.5	1715927.1	1658135.8	1601122.1	1630646.0	1677870.6	1717589.1	31749817.5	26021474.2	30510387.3	26452668.9
P47738	Aldehyde dehydrogenase, mitochondrial	Aldh2	519	56.5	7.62	7754950.0	6427521.6	7302304.7	7328522.8	7599637.2	6266521.1	7809128.8	7800657.1	7933268.7	7412746.8	4216522.8	4089161.7	3614291.7	4160804.4
P38647	Stress-70 protein, mitochondrial	Hspa9	679	73.4	6.07	3303376.9	3086547.4	3266114.4	3083822.4	3131862.4	3100784.1	2939519.9	3312141.5	3029325.8	2995539.6	2732620.1	2517160.5	2534333.7	2569972.5
P10126	Elongation factor 1-alpha 1	Eef1a1	462	50.1	9.01	17123090.3	17712562.2	19281153.7	17960838.4	16836593.2	15521294.7	14881956.0	18770295.6	17134056.7	16889431.9	17134199.5	17409046.6	20762090.9	18205493.6
Q9DBG3	AP-2 complex subunit beta	Ap2b1	937	104.5	5.38	2396879.9	2586350.7	2615149.2	2586998.4	2604367.5	2449974.2	2341998.1	2528494.3	2554142.6	2522456.2	1950933.1	1966214.9	1958384.0	1936567.0
P24549	Retinal dehydrogenase 1	Aldh1a1	501	54.4	7.8	7622384.4	6622506.0	6652972.2	6944751.5	5710414.4	5145233.9	7013635.0	7107135.6	6539031.6	6129215.5	9130091.4	7909683.7	7996301.0	8244074.7
P05214	Tubulin alpha-3 chain	Tuba3a	450	49.9	5.1	254801.2	328345.2	317918.3	325339.1	283092.1	283788.0	261998.2	269294.1	302493.9	348038.8	479242.3	602901.0	568527.0	589081.9

Q64511	DNA topoisomerase 2-beta	Top2b	1612	181.8	8.29	787596.6	875259.2	837870.1	812189.1	917164.3	902029.1	783588.0	776861.5	823154.8	855783.9	1220505.6	1280472.7	1188126.6	1241509.1
Q8BKC5	Importin-5	Ipo5	1097	123.5	4.93	1476144.1	1703386.6	1650010.4	1890897.0	1435147.8	1273423.9	1190786.7	1220347.2	1532564.0	1584119.2	1094925.1	1084520.8	1024357.3	1145647.1
Q9JKF1	Ras GTPase-activating-like protein IQGAP1	Iqgap1	1657	188.6	6.48	945674.6	933702.4	865133.8	903275.9	950354.2	984878.6	1009678.7	921529.6	888478.7	894557.3	929113.5	960151.2	905150.5	928545.9
Q6P5D8	Structural maintenance of chromosomes flexible hinge domain-containing protein 1	Smchd1	2007	225.5	7.24	1574123.5	1697039.2	1710790.2	1605251.0	1868118.2	1775104.2	1575549.6	1558968.3	1694916.1	1727591.8	1766174.2	1879387.8	1896659.0	1820864.2
Q6PB66	Leucine-rich PPR motif-containing protein, mitochondrial	Lrpprc	1392	156.5	6.83	2304354.0	2247280.9	2252849.7	2202373.9	2282199.6	2100171.1	2071755.5	2136131.7	2220211.2	2232366.6	2104813.2	1988712.4	2006527.3	1975627.6
P11103	Poly [ADP-ribose] polymerase 1	Parp1	1013	113	8.95	3631838.3	3406174.4	3955954.3	3472225.1	4143133.5	4138789.3	3605698.6	3793376.8	3648762.1	3610706.7	2386930.1	2203477.7	2334750.0	2223502.9
P80318	T-complex protein 1 subunit gamma	Cct3	545	60.6	6.7	3220710.8	3658404.1	3828147.1	3757692.2	3630802.8	3332793.9	3198085.7	3352161.5	3605266.7	3751061.4	4407585.5	5016788.4	5389426.2	5461516.7
P06801	NADP-dependent malic enzyme	Me1	572	63.9	7.44	4801291.8	4506347.9	4320147.9	5000250.0	3875603.1	3750358.1	3791255.4	4319996.2	3984620.0	4004720.5	5567369.7	4886134.4	4481885.3	5409108.2
Q9JKK8	Serine/threonine-protein kinase ATR	Atr	2635	300	7.53	1185192.3	1326166.6	1270603.1	1310361.5	1317169.2	1232201.7	1070001.9	1045011.3	1321620.2	1387351.6	821393.2	939221.8	961564.3	936029.8
Q6P5F9	Exportin-1	Xpo1	1071	123	6.07	2968389.1	3116261.2	3319826.7	3160259.9	3281361.2	3198876.9	2990189.5	2863819.6	3185057.9	3245040.9	2902811.2	3124289.9	3168369.5	3134627.5
Q99NB9	Splicing factor 3B subunit 1	Sf3b1	1304	145.7	7.09	1215533.9	1276447.4	1361149.9	1335589.4	1387544.6	1405117.4	1242044.5	1212025.3	1269974.0	1322113.4	1212586.4	1283107.3	1230335.8	1314337.8
Q3U1J4	DNA damage-binding protein 1	Ddb1	1140	126.8	5.26	3108213.9	3222385.3	3377434.8	3390299.7	3539634.2	3448702.0	3143352.0	2988172.8	3454527.2	3651130.9	3753050.1	3716882.7	4144453.1	3784349.7
P16858	Glyceraldehyde-3-phosphate dehydrogenase	Gapdh	333	35.8	8.25	13395349.1	12174538.1	13129774.3	11528651.6	13630509.8	13868333.3	15858472.9	13699149.8	13448719.4	13516086.8	13248489.8	10882177.7	13626679.6	10523796.1
P09103	Protein disulfide-isomerase	P4hb	509	57	4.88	6319033.5	6649488.7	6425708.6	6437165.3	6069710.0	6017154.4	5021813.9	6776764.3	6549544.7	6374175.6	6460687.0	6397681.3	6602443.0	6391810.6
Q91V92	ATP-citrate synthase	Acly	1091	119.7	7.44	2611099.5	2465459.4	2683589.3	2394088.5	2642213.4	2576492.8	2651289.7	2603755.3	2585547.9	2627342.1	3640165.7	3965740.9	3674006.3	3792810.0
Q64514	Tripeptidyl-peptidase 2	Tpp2	1262	139.8	6.58	2341274.8	2633708.3	2607692.4	2661361.0	2530139.2	2388645.8	2162074.2	2345846.3	2553386.8	2618178.5	2345090.6	2856388.6	2646434.9	2764883.0
P09405	Nucleolin	Ncl	707	76.7	4.75	7449076.9	7680371.4	8663927.4	6874027.8	9670562.3	9312495.5	7956438.1	7544174.0	8577663.5	8905038.0	10643829.3	11050461.5	12885882.5	10181783.6
O70318	Band 4.1-like protein 2	Epb41l2	988	109.9	5.43	2366344.4	2123858.7	2303765.0	2076396.1	2723997.2	2780369.7	2778646.3	2294853.4	2474992.8	2402351.3	1779973.8	1647281.4	1669173.8	1574589.2
P68373	Tubulin alpha-1C chain	Tuba1c	449	49.9	5.1	21513239.0	18942114.7	26725981.2	22728101.7	22799023.5	22303402.6	26813572.9	24532477.1	23939096.7	21163186.2	50446.7	40615.5	37169.2	39177.6
Q8CI94	Glycogen phosphorylase, brain form	Pygb	843	96.7	6.73	2952202.5	2696974.8	2745023.6	2528795.8	3209114.4	3359481.7	3935841.3	2942223.0	3245943.6	3167775.2	3162914.6	2692755.9	3055346.3	2537697.4
P14873	Microtubule-associated protein 1B	Map1b	2464	270.1	4.83	784004.0	763044.4	755297.4	798563.4	750363.7	778810.4	811295.3	776272.0	736524.7	731511.7	664705.3	653231.0	626678.7	689072.2
P70398	Probable ubiquitin carboxyl-terminal hydrolase FAF-X	Usp9x	2559	290.5	5.87	1883881.5	1849571.7	1782003.7	1734324.6	1796255.9	1740457.8	1602629.3	1669761.1	1820330.9	1929511.9	1522215.7	1548487.3	1598331.5	1514408.7
Q99PU5	Long-chain-fatty-acid--CoA ligase ACSBG1	Acsbg1	721	80.4	5.94	3133779.4	2867523.1	2803720.8	3037421.1	2842077.3	2709321.3	2969609.7	2916750.5	2864047.8	2847215.1	3275269.0	2952975.5	2852322.7	3169969.0
Q7TPR4	Alpha-actinin-1	Actn1	892	103	5.38	1300149.8	1279256.9	1325893.6	1249628.7	1332085.6	1499962.3	1656132.1	1485849.8	1287077.4	1230662.2	1859950.4	1699756.2	1877669.2	1695216.6
Q8VEK3	Heterogeneous nuclear ribonucleoprotein U	Hnrnpu	800	87.9	6.24	4067827.3	3963653.6	4458081.5	3927179.2	4377331.6	4427614.6	4117894.1	4058947.7	4040959.1	4088909.3	5020944.7	5532049.5	5171154.0	5258372.5
Q7TMM9	Tubulin beta-2A chain	Tubb2a	445	49.9	4.89	532714.1	515332.6	576899.1	491731.8	569300.9	654045.5	579019.9	630746.1	514971.2	550983.7	549875.5	545071.1	552586.5	518468.2
Q9CWF2	Tubulin beta-2B chain	Tubb2b	445	49.9	4.89	19273.9	12590.7	20425.2	13693.4	26386.4	22529.9	23261.9	30609.7	16401.2	21134.0	242909.1	322251.0	227634.7	260925.7
A0A0J9YUD5	Nucleoporin 205	Nup205	2061	233	6.62	1096079.4	1139505.7	1140727.7	1116365.7	1115498.1	1154320.1	1091691.8	1050806.2	1123680.7	1120564.5	970917.4	986938.5	992367.2	1000236.4
Q5SWU9	Acetyl-CoA carboxylase 1	Acaca	2345	265.1	6.39	1181222.7	1078510.7	1088315.9	1041750.5	1009013.7	946427.0	900595.4	1059965.5	1062624.7	1082476.4	761506.2	735044.3	700890.7	709821.8
P11983	T-complex protein 1 subunit alpha	Tcp1	556	60.4	6.16	3232414.2	3708889.7	3932411.2	3991782.9	3274748.2	3115931.0	2952398.9	3068756.2	3690959.8	3596149.0	2593092.2	2786635.2	2854379.3	2854755.3
S4R1W1	Glyceraldehyde-3-phosphate dehydrogenase	Gm3839	333	35.8	8.03	355300.5	393696.3	354786.9	348543.8	380779.1	349922.3	375888.4	376369.3	380889.1	371174.7	318504.4	284810.9	263469.7	303779.5
Q3TXS7	26S proteasome non-ATPase regulatory subunit 1	Psmd1	953	105.7	5.39	1185160.5	1279872.2	1234364.9	1273922.0	1242395.4	1188013.5	1103543.2	1135623.9	1227336.1	1259762.6	1359103.1	1409885.0	1314622.4	1443136.0
Q9Z1Q9	Valine--tRNA ligase	Vars1	1263	140.1	7.77	2049471.2	2099013.3	2227666.0	2072595.8	2344909.3	2154525.0	2014221.3	2008874.4	2153012.1	2298987.7	2016484.1	1943923.3	2151433.7	1894427.1
Q05793	Basement membrane-specific heparan sulfate proteoglycan core protein	Hspg2	3707	398	6.32	738000.0	728336.4	753586.4	729692.4	839346.9	853349.3	868006.6	829275.4	745832.3	736438.4	841473.1	835218.5	867204.2	805141.6
Q03265	ATP synthase subunit alpha, mitochondrial	Atp5f1a	553	59.7	9.19	8618693.3	8282976.9	8343536.4	8677752.0	8545185.8	8154169.9	8415219.2	8370005.6	8439725.8	8244810.9	5982463.8	6968072.0	6211177.3	6728558.7
P14824	Annexin A6	Anxa6	673	75.8	5.5	3543644.6	3475937.1	3420711.8	3177657.0	3885410.8	4282685.3	4261909.7	4105992.1	3706581.1	3635429.1	3043983.7	2635738.3	2721960.2	2460283.8
P68368	Tubulin alpha-4A chain	Tuba4a	448	49.9	5.06	192864.7	202069.6	204809.9	198218.9	215847.3	214558.0	328721.3	207224.8	217023.2	223904.5	327684.6	340887.9	359192.5	310160.2
P80316	T-complex protein 1 subunit epsilon	Cct5	541	59.6	6.02	2299724.4	3058054.3	2601149.0	3100871.0	2449359.0	2247058.4	2318961.5	2264034.1	2693078.4	2861732.5	2294415.4	2701475.9	2561758.8	2737414.3
P08003	Protein disulfide-isomerase A4	Pdia4	638	71.9	5.31	4563687.8	4647990.6	4361933.8	4639697.5	4169587.2	4160035.7	3711489.7	4350961.0	4477998.7	4436200.3	4395568.0	4603378.9	4358885.2	4664137.3
Q9ERD7	Tubulin beta-3 chain	Tubb3	450	50.4	4.93	807932.9	820138.3	734347.8	806473.0	790987.7	775467.8	728902.1	751743.3	767501.5	716681.7	1058839.4	1043666.7	963278.7	988833.8
P80315	T-complex protein 1 subunit delta	Cct4	539	58	8.02	2567640.4	2560510.3	2917680.6	2818585.5	2680228.4	2614265.9	2268153.6	2533698.5	2618007.5	2629242.7	2207260.7	2420399.8	2541320.5	2467290.9
P97479	Unconventional myosin-VIIa	Myo7a	2215	254.8	8.54	1283835.0	1266084.9	1186163.1	1238296.7	1206424.6	1135692.0	1256445.1	1241617.2	1266596.6	1207690.8	1449222.2	1466837.2	1363729.4	1378918.4
Q61598	Rab GDP dissociation inhibitor beta	Gdi2	445	50.5	6.25	1947579.4	1821063.7	1982185.9	1862771.1	1943068.1	1985774.5	2000893.7	2209014.8	1956973.2	1838750.1	2271932.2	2185353.5	2156118.7	2106625.6
Q8CIE6	Coatomer subunit alpha	Copa	1224	138.3	7.65	2939459.7	2712265.7	2805976.6	2784952.3	2671221.1	2578189.5	2492250.7	2850348.6	2695580.5	2744828.2	2385210.0	2336466.7	2376368.4	2364155.3
P14211	Calreticulin	Calr	416	48	4.49	10536577.9	12814824.5	12406007.0	12832542.8	10696271.7	9298609.7	8319271.9	10705401.7	12060588.5	11964208.9	12717962.5	13433688.7	15165733.6	14189756.6
Q91VI7	Ribonuclease inhibitor	Rnh1	456	49.8	4.78	816600.3	833785.9	806517.9	929899.6	929247.2	848032.7	907197.1	876844.7	877559.2	886152.4	1049997.5	957476.2	948948.3	1012547.3
Q8CAQ8	MICOS complex subunit Mic60	Immt	757	83.8	6.61	1814525.7	1706308.0	1805317.4	1795898.0	1809586.2	1650010.7	1694940.7	1658476.1	1686364.6	1688928.9	1643192.9	1442466.8	1676146.3	1557707.5
Q9ERK4	Exportin-2	Cse1l	971	110.4	5.77	1890090.6	2029721.8	2083110.3	2119776.7	2122904.4	1916847.6	1837708.2	1805072.2	2116074.9	2001856.1	2116572.8	2192920.0	2197127.1	2160240.0
Q60675	Laminin subunit alpha-2	Lama2	3118	343.6	6.09	1012038.2	980810.9	948892.5	948148.9	1020552.2	1019600.1	1074429.7	1002770.4	1039299.3	1017155.6	1012008.9	988222.2	1001956.3	950987.0
O35643	AP-1 complex subunit beta-1	Ap1b1	943	103.9	5.17	314652.9	362260.4	380153.5	344176.8	358236.6	332258.6	323439.9	320694.8	335707.5	337186.8	398799.1	436145.2	418002.0	422726.4
Q8BX70	Vacuolar protein sorting-associated protein 13C	Vps13c	3748	419.8	6.81	610814.0	595080.4	603598.8	558703.9	659661.4	655910.9	620052.5	549195.7	619561.8	628150.4	317154.1	349855.8	335809.3	341995.7
P31254	Ubiquitin-like modifier-activating enzyme 1 Y	Uba1y	1058	118	5.55	650974.6	734341.2	826013.6	645062.4	876105.8	787593.4	642147.8	672683.5	785902.4	812939.3	558420.8	637663.2	690503.6	579625.7
Q64737	Trifunctional purine biosynthetic protein adenosine-3	Gart	1010	107.4	6.68	2012198.6	1945483.5	1940167.6	1862440.2	1898495.9	1841151.9	1801557.7	1942073.1	1893384.4	1953657.8	1775049.3	1607622.6	1656976.0	1623483.8
Q9JIF7	Coatomer subunit beta	Copb1	953	107	6	1041475.3	1111147.0	1083133.9	1078169.9	1038508.0	1028388.6	990387.8	1054631.2	1075938.2	1075449.7	795264.3	810635.1	771306.5	815770.1
Q80X19	Collagen alpha-1(XIV) chain	Col14a1	1797	192.9	5.1	1114833.5	1049576.9	967748.9	1062088.9	1099357.5	1288621.6	1244480.3	1393562.8	1000740.3	1012285.1	1122040.9	973091.2	940917.3	983619.4
P42932	T-complex protein 1 subunit theta	Cct8	548	59.5	5.62	3998654.2	3812051.5	5202361.4	4741160.7	4214932.6	3955861.4	3663017.7	4265306.8	4504809.6	4248004.9	3493838.3	4284997.7	4966183.2	4430574.8
Q9D6F9	Tubulin beta-4A chain	Tubb4a	444	49.6	4.88	74995.8	96870.0	76163.9	92320.6	98317.9	89015.7	75021.4	72610.4	87469.1	95120.4	#N/A	#N/A	#N/A	#N/A
Q60597	2-oxoglutarate dehydrogenase, mitochondrial	Ogdh	1023	116.4	6.83	2083829.0	2032371.8	1829432.5	1960346.3	1988002.1	1989348.3	2055281.9	1956520.1	2017134.3	2031733.3	1648230.0	1609143.4	1427978.3	1586041.2
P62737	Actin, aortic smooth muscle	Acta2	377	42	5.39	14711964.5	13214156.4	14651088.1	13366435.2	12967037.3	13421814.0	15413201.3	17044913.7	12918230.6	13424681.1	1386432.5	1422367.1	1442753.8	1156138.4
P48678	Prelamin-A/C	Lmna	665	74.2	6.98	2560495.3	2268662.6	2469868.5	2339605.6	3145934.2	3615416.0	4375203.0	2830295.3	2641280.2	2588685.7	2221358.4	1902219.0	2122889.5	1897820.7
Q61292	Laminin subunit beta-2	Lamb2	1799	196.5	6.67	931072.9	881501.4	883769.3	857577.0	1022451.7	1043979.4	1170931.8	990874.7	941941.9	907781.4	767308.8	730073.3	699279.4	658624.1
P70168	Importin subunit beta-1	Kpnb1	876	97.1	4.78	2201235.3	2171033.9	2445334.4	2304745.3	2304413.1	2025176.5	1845573.0	2161513.7	2081734.2	2118084.7	2069763.9	2103834.0	2230275.9	2291941.9
P57780	Alpha-actinin-4	Actn4	912	104.9	5.41	989528.7	878892.8	805384.6	844623.2	840364.2	878939.1	924690.8	826816.8	896223.5	892267.5	665369.0	666896.2	651203.6	666170.7
Q60847	Collagen alpha-1(XII) chain	Col12a1	3120	340	5.64	705597.2	626391.4	537615.4	667194.7	537741.4	648492.7	929643.1	729381.9	669630.0	577773.7	993430.0	883009.8	774901.0	950305.5
P15626	Glutathione S-transferase Mu 2	Gstm2	218	25.7	7.39	8437229.4	7424850.2	9517203.3	8523397.1	9405998.4	9642010.5	10912972.4	10405980.8	8998011.9	7872197.4	10820071.1	9761846.9	12956243.6	11040593.8
Q9Z1F9	SUMO-activating enzyme subunit 2	Uba2	638	70.5	5.24	1378694.6	1543641.1	1557833.8	1532038.8	1568635.4	1541347.1	1405197.9	1394656.3	1532475.6	1608023.8	1441132.4	1618450.8	1708900.7	1722810.8
Q3UMC0	ATPase family protein 2 homolog	Spata5	893	97.2	8.24	995141.9	1020368.2	1011096.8	968834.3	1048767.6	1063050.3	958596.0	971188.6	1005583.7	1028654.8	673465.1	733107.3	703440.9	664770.3
O08810	116 kDa U5 small nuclear ribonucleoprotein component	Eftud2	971	109.3	5	1673357.2	1626234.9	1747292.1	1671687.1	1739856.9	1715758.0	1673681.5	1521193.6	1720375.4	1772803.7	1349775.2	1369251.3	1321101.2	1355560.2
Q6ZQ08	CCR4-NOT transcription complex subunit 1	Cnot1	2375	266.6	7.11	914457.5	955492.2	894894.4	943975.8	907603.0	889367.2	793533.3	791831.3	918658.5	949508.9	1174198.2	1259221.2	1287778.7	1275959.6
Q3THK7	GMP synthase [glutamine-hydrolyzing]	Gmps	693	76.7	6.73	1160959.1	1369913.2	1146920.4	1277619.0	1341505.8	1219148.2	1083867.9	1139709.4	1220020.2	1332793.7	1195128.2	1179022.6	1306920.9	1232253.8
Q9D0E1	Heterogeneous nuclear ribonucleoprotein M	Hnrnpm	729	77.6	8.63	3626686.7	4118839.7	4033843.4	4495005.3	3691095.7	3736540.9	3896584.0	3470290.8	3901646.1	4177633.0	2657295.0	2915305.4	3033166.6	2933384.5
Q920B9	FACT complex subunit SPT16	Supt16h	1047	119.7	5.66	1296311.0	1304484.0	1361589.3	1349240.5	1450265.1	1435342.0	1405548.2	1295669.9	1354609.9	1383523.2	1265604.7	1336997.8	1400750.6	1268629.6
Q6A4J8	Ubiquitin carboxyl-terminal hydrolase 7	Usp7	1103	128.4	5.5	1217072.9	1179441.0	1372888.4	1246842.5	1400847.8	1320132.4	1209760.7	1241158.5	1291744.2	1323164.6	965234.7	997041.6	1001451.8	996360.4
Q78PY7	Staphylococcal nuclease domain-containing protein 1	Snd1	910	102	7.43	1314581.3	1288845.2	1320906.8	1260859.4	1338368.1	1289147.9	1272828.5	1331384.8	1296826.5	1268845.9	982672.3	1080369.6	977824.3	990910.8
Q91VR5	ATP-dependent RNA helicase DDX1	Ddx1	740	82.4	7.21	1780549.8	1915123.5	1899408.6	1809416.6	1861453.3	1904649.9	1718364.7	1774632.6	1777307.7	1811399.2	1517444.3	1898066.7	1601424.1	1660124.0
Q7TPV4	Myb-binding protein 1A	Mybbp1a	1344	151.9	8.95	1126654.2	1181802.8	1243267.4	1078522.6	1308527.6	1272045.5	1139048.7	1189010.9	1163125.9	1215862.1	796996.3	1027815.2	833663.2	844643.9
P02468	Laminin subunit gamma-1	Lamc1	1607	177.2	5.21	1146978.9	1061457.9	1123100.3	1071113.4	1243604.9	1280762.4	1334557.1	1169309.5	1160306.7	1141565.3	779488.8	737459.0	685633.3	768611.1
Q9CWJ9	Bifunctional purine biosynthesis protein PURH	Atic	592	64.2	6.76	1448198.9	1444857.8	1261818.6	1357292.2	1328798.8	1361418.7	1399697.0	1334042.2	1411268.6	1465457.2	1905132.8	1877768.2	1848159.1	1726195.1
P80313	T-complex protein 1 subunit eta	Cct7	544	59.6	7.84	2973441.4	2754770.1	3318398.2	3193569.3	2834116.3	2751582.6	2591526.7	2836927.6	2972656.0	2994321.9	4305943.1	3986169.9	5145616.9	4547572.8
G5E870	E3 ubiquitin-protein ligase TRIP12	Trip12	2025	224	8.35	855463.0	1088672.2	872177.9	1042136.9	1007023.7	971794.8	780405.4	842461.8	945047.4	1019639.4	909950.6	1039380.1	974134.0	1034110.6
P27612	Phospholipase A-2-activating protein	Plaa	794	87.2	6.14	1022643.1	1042572.1	1102231.2	1078491.2	1123040.1	1038679.6	1015245.3	1085289.5	1010786.0	1026211.8	1428083.0	1539456.1	1523449.3	1561461.1
Q921M3	Splicing factor 3B subunit 3	Sf3b3	1217	135.5	5.26	2157921.5	2087855.4	2289508.1	2157710.1	2344090.8	2355328.3	2359934.0	2142663.1	2340355.2	2416533.0	1626085.9	1694684.1	1772598.1	1569567.2
Q689Z5	Protein strawberry notch homolog 1	Sbno1	1390	153.6	8.07	767081.4	828811.9	917768.6	829924.4	861086.2	828193.5	704714.9	712754.7	875380.3	894625.4	822914.8	938392.0	932781.4	871271.3
Q9QUR6	Prolyl endopeptidase	Prep	710	80.7	5.73	2766544.5	2654974.4	2417123.8	2391133.9	2649676.6	2768627.4	2461331.2	2530933.4	2608660.1	2630650.8	1864788.7	1770509.5	1897536.4	1701315.3
Q6PDQ2	Chromodomain-helicase-DNA-binding protein 4	Chd4	1915	217.6	5.81	1149873.3	1128228.0	1195078.2	1095174.7	1241113.5	1212469.9	1195795.1	1096720.0	1168049.0	1230568.5	1058550.2	983330.0	1100194.5	983916.8
P23116	Eukaryotic translation initiation factor 3 subunit A	Eif3a	1344	161.8	6.77	2138612.7	2125460.8	2197751.7	2162738.2	2177865.1	2069358.5	1993385.5	2096257.4	2140168.8	2216719.5	1369628.9	1494436.7	1526285.0	1505433.8
Q8K1M6	Dynamin-1-like protein	Dnm1l	742	82.6	7.05	1451119.4	1559110.1	1530849.0	1559630.4	1508911.8	1461890.9	1411006.0	1440302.3	1490087.1	1550673.7	1360941.9	1492498.0	1397123.5	1479963.8
Q04857	Collagen alpha-1(VI) chain	Col6a1	1025	108.4	5.36	3835958.6	3443018.5	3383384.7	3344364.3	4262573.6	5677270.8	6595082.8	4674249.9	3731973.5	3504036.9	3027336.6	2897555.0	2713118.7	2866433.0
O88685	26S proteasome regulatory subunit 6A	Psmc3	442	49.5	5.19	1398327.0	1489623.7	1397847.9	1466953.0	1389128.8	1394169.3	1310848.7	1348608.3	1395479.7	1431721.6	1252205.6	1419148.4	1400060.9	1449886.9
Q99PL5	Ribosome-binding protein 1	Rrbp1	1605	172.8	9.33	1259581.3	1219088.6	1168222.4	1188068.6	1060983.4	1070750.8	1075929.8	1269159.8	1039092.0	1067210.4	1489333.7	1563646.8	1442534.5	1520863.3
P50247	Adenosylhomocysteinase	Ahcy	432	47.7	6.54	8971122.8	7606402.4	9173839.3	7322080.0	10368407.4	10761163.6	14095372.0	9141502.7	9887132.0	9725874.3	7413043.8	6279592.9	7463727.4	5827897.8
Q9WU78	Programmed cell death 6-interacting protein	Pdcd6ip	869	96	6.52	1906153.3	1968621.7	1910455.9	1926213.8	1878884.6	1918693.6	1877483.5	1824459.8	1930618.3	1849956.2	1755209.8	1883524.4	1840241.0	1859239.8

Q91ZX7	Prolow-density lipoprotein receptor-related protein 1	Lrp1	4545	504.4	5.36	1086982.1	1002456.8	1095933.6	997271.1	1082596.6	1130274.6	1166058.2	1134343.4	1076871.3	1042967.1	1099369.6	1042131.6	1042625.0	963130.0
P17426	AP-2 complex subunit alpha-1	Ap2a1	977	107.6	7.03	1352210.0	1431349.8	1348067.2	1387239.5	1423343.3	1378205.1	1309046.5	1278401.4	1362996.7	1393049.6	1854793.8	1871331.8	1917421.9	1885244.3
Q60864	Stress-induced-phosphoprotein 1	Stip1	543	62.5	6.8	4222108.9	4586901.9	4575642.4	4777821.5	3989293.5	3914562.9	3690360.6	4077890.8	4279271.5	4279074.5	2942203.7	3382701.7	3686413.4	3559848.9
P05064	Fructose-bisphosphate aldolase A	Aldoa	364	39.3	8.09	2883156.7	2425410.2	2838478.0	2382750.1	3236259.1	2979329.9	2831274.2	3192503.7	2643989.7	2471557.8	3700928.8	3094094.2	3595429.0	3067876.0
P17563	Methanethiol oxidase	Selenbp1	472	52.5	6.29	1797791.6	1723892.3	1867455.0	1715186.7	2202051.1	2819293.4	3515309.9	1956207.1	2125220.7	2111034.8	1579457.7	1643890.7	1673151.4	1666694.7
P07356	Annexin A2	Anxa2	339	38.7	7.69	7190522.5	6301231.7	7297310.7	6170869.8	9180519.6	10166453.6	12161854.2	8509980.3	7633336.6	7247894.7	6859034.6	6314146.4	7332276.8	6291948.5
Q8BHN3	Neutral alpha-glucosidase AB	Ganab	944	106.8	6.06	1842665.7	1604122.7	1515166.7	1687108.5	1663220.8	1665353.6	1665164.9	1661365.2	1621049.3	1605443.3	2364703.8	2240218.6	2113850.1	2170802.3
Q99KK7	Dipeptidyl peptidase 3	Dpp3	738	82.8	5.38	2149224.0	2242652.7	2095805.1	2196307.6	2035651.9	2015442.0	2041820.8	1940668.5	2109176.5	2210674.5	1438781.2	1327841.7	1245883.3	1307914.3
P62259	14-3-3 protein epsilon	Ywhae	255	29.2	4.74	5080991.8	6128500.7	6160704.1	7045767.1	5979938.2	5634159.8	6081050.4	5475177.7	6959497.1	6350924.2	3518773.8	4658780.0	4489069.4	5461894.7
Q76MZ3	Serine/threonine-protein phosphatase 2A 65 kDa regulatory subunit A alpha isoform	Ppp2r1a	589	65.3	5.11	1194205.5	1227664.3	1198924.7	1176635.0	1347882.8	1278788.9	1261242.3	1245365.6	1220210.1	1179070.0	1085885.6	1115943.4	1092985.9	1084331.8
P59242	Cingulin	Cgn	1191	136.4	5.91	1175039.7	1081819.6	1086777.1	1032872.7	1128057.8	1126404.1	1174972.3	1062651.7	1076735.5	1103427.6	1194852.3	1006290.7	1081605.7	956979.1
P26041	Moesin	Msn	577	67.7	6.6	4217669.3	3972433.8	3792451.4	3829385.0	4016412.2	4299506.0	4591778.0	4291886.8	3932865.9	4068643.3	2542748.3	2297665.0	2242494.4	2231120.5
Q9D0I9	Arginine--tRNA ligase, cytoplasmic	Rars1	660	75.6	7.55	1233234.2	1279822.9	1311493.1	1268511.6	1301736.3	1290859.6	1140608.2	1266357.5	1289102.4	1302593.2	952819.5	1024925.6	1025055.3	1017707.1
Q922D8	C-1-tetrahydrofolate synthase, cytoplasmic	Mthfd1	935	101.1	7.14	1068947.4	958876.8	950950.0	924087.1	914265.3	950358.9	957025.9	945388.4	956387.3	966220.4	686126.4	714881.4	627624.4	701664.6
P60843	Eukaryotic initiation factor 4A-I	Eif4a1	406	46.1	5.48	1783048.9	1754292.0	1833823.6	1712191.8	1927339.8	1795399.3	1683017.1	1806850.2	1877820.1	1867651.0	2528285.9	2619268.9	2442229.8	2508602.5
E9QAE3	B-TFIID TATA-box-binding protein-associated factor 1	Btaf1	1848	206.9	6.43	720832.6	820555.4	771655.7	854793.3	751523.5	683596.7	618709.2	617010.8	768545.2	795685.0	586543.8	639737.3	587462.1	675237.2
Q9JIK5	Nucleolar RNA helicase 2	Ddx21	851	93.5	9.11	1127095.8	1089940.1	1052996.5	980534.4	1208646.5	1187762.2	1085784.9	1095423.2	1125086.0	1122484.2	1166259.9	1163249.2	1226655.5	1120997.4
Q9EPU0	Regulator of nonsense transcripts 1	Upf1	1124	123.9	6.61	1269642.4	1401097.8	1415209.9	1442902.9	1288638.3	1256389.9	1208587.7	1215187.5	1326294.1	1386014.0	1403333.8	1681183.2	1600010.8	1735735.3
P02469	Laminin subunit beta-1	Lamb1	1786	197	4.94	702721.8	610642.1	613271.3	587555.5	703580.0	729177.8	617633.4	731681.8	577223.0	630091.1	618781.3	572931.8	547609.1	549785.6
P28271	Cytoplasmic aconitate hydratase	Aco1	889	98.1	7.5	1471812.4	1329355.1	1355441.2	1391028.0	1385152.3	1379565.7	1514359.1	1451323.6	1379984.0	1372688.6	1029100.8	959935.2	968075.8	1125241.2
O88844	Isocitrate dehydrogenase [NADP] cytoplasmic	Idh1	414	46.6	7.17	3818342.1	3610334.3	3722130.6	3708714.9	3912687.0	3827461.4	4171326.5	3621586.9	3867873.5	3942943.3	3416856.1	3004990.0	3064401.0	3070508.3
P97807	Fumarate hydratase, mitochondrial	Fh	507	54.3	9.04	1388734.3	1564416.9	1468242.1	1426043.6	1511884.2	1503670.7	1357562.3	1565735.2	1504351.6	1489474.0	1512677.9	1469226.6	1402945.7	1446578.6
Q99MW1	Serine/threonine-protein kinase 31	Stk31	1018	114.9	5.39	867375.9	1076510.1	1053163.9	1159640.5	800255.7	781093.7	682736.1	809247.3	921866.4	972399.6	1372765.2	1748275.0	1535398.2	1915873.8
Q61656	Probable ATP-dependent RNA helicase DDX5	Ddx5	614	69.2	8.92	4841423.0	5114139.8	5271493.8	5250147.9	5371676.3	4755091.7	4669735.5	4724161.5	5085159.8	5185394.3	4682460.4	4837668.8	4941084.5	5105632.3
Q8VDM4	26S proteasome non-ATPase regulatory subunit 2	Psmd2	908	100.1	5.17	1908788.2	2224457.6	2015819.9	2191822.9	2037134.5	1912418.2	1763947.3	1865757.9	2113471.3	2234741.8	1790643.5	2181816.1	1870014.7	2210079.7
Q3UPL0	Protein transport protein Sec31A	Sec31a	1230	133.5	6.76	1292077.2	1208534.9	1298870.0	1329194.6	1161422.3	1209630.8	1142539.7	1266550.0	1225137.2	1171859.0	1615527.4	1645266.0	1523165.0	1633849.1
P26231	Catenin alpha-1	Ctnna1	906	100	6.23	833431.9	839406.0	717604.5	814808.9	866830.5	853147.6	896845.9	812352.1	789226.4	800325.0	1028588.9	1174582.6	954692.2	1006995.6
P19324	Serpin H1	Serpinh1	417	46.5	8.82	1574289.5	1576300.5	1277245.3	1511774.7	1070012.2	1176601.8	884077.3	1924832.6	1111405.1	1084103.3	2302805.8	2025225.1	1586038.6	1945008.7
Q8BL66	Early endosome antigen 1	Eea1	1411	160.8	5.77	376550.1	386069.3	379873.5	377857.1	404996.8	433049.9	372797.9	355568.6	363779.5	368037.7	591946.7	472192.0	646491.5	523632.0
P11276	Fibronectin	Fn1	2477	272.4	5.59	380526.4	394321.5	342374.4	329373.3	316859.1	361492.1	387073.9	426228.3	358760.2	388723.6	619731.4	588327.3	602077.2	516715.1
Q62318	Transcription intermediary factor 1-beta	Trim28	834	88.8	5.77	2512655.5	2428099.6	2845347.9	2625025.8	2770640.5	2623715.4	2563538.1	2542936.2	2551238.8	2555607.0	2249489.1	2213502.8	2479529.3	2379585.2
Q05920	Pyruvate carboxylase, mitochondrial	Pc	1178	129.6	6.71	1467125.5	1464056.7	1345536.4	1470941.2	1430442.1	1299576.9	1305468.2	1386599.8	1451553.7	1460228.8	1175718.3	1110990.8	1000323.8	1115109.3
P54276	DNA mismatch repair protein Msh6	Msh6	1358	151	6.73	714572.3	813020.0	862270.1	788803.8	844614.7	823681.1	736373.0	732768.3	846214.3	831215.5	857185.5	836610.0	885361.8	994613.2
B1AVB3	Scm polycomb group protein-like 2	Scml2	969	106.7	9.54	1479901.3	1665512.9	1626830.1	1459583.6	1651388.8	1603142.7	1272416.5	1189796.4	1653328.0	1703534.0	1939246.0	2077101.1	2046502.8	1857221.0
Q8C6K9	Collagen alpha-6(VI) chain	Col6a6	2265	246.2	6.83	692081.2	616421.2	617026.2	632335.4	772880.1	874632.4	892146.7	763350.6	650276.2	637170.2	444530.3	441319.5	399052.4	402449.5
P56399	Ubiquitin carboxyl-terminal hydrolase 5	Usp5	858	95.8	5.01	1077478.3	1145746.8	1150860.3	1185385.0	1101116.2	1056156.1	1055408.2	1049005.8	1142558.3	1146879.6	1133903.4	1126107.4	1255346.2	1212132.7
Q71LX4	Talin-2	Tln2	2375	253.5	5.8	193809.0	215230.9	189943.6	202247.3	214788.0	222569.4	202064.5	186919.5	203928.5	197014.9	297595.9	302300.3	274932.0	300107.0
Q91XV3	Brain acid soluble protein 1	Basp1	226	22.1	4.51	434870.6	562988.3	398394.4	515438.3	441430.4	490372.7	745603.7	443815.1	660236.9	715464.5	556360.1	437633.5	555515.1	417267.6
Q8BGQ7	Alanine--tRNA ligase, cytoplasmic	Aars1	968	106.8	5.67	847627.3	865036.0	891474.9	878966.4	837649.5	807052.5	796586.2	782099.0	872420.7	885309.8	1556503.0	1702844.7	1745803.4	1699178.4
D4AFX7	J domain-containing protein	Dnajc13	2248	254.9	6.77	745010.7	782895.4	809952.8	787895.5	916133.7	814691.5	722898.4	725608.4	770946.9	794982.2	834087.5	917586.3	828313.2	862895.2
Q8BMJ2	Leucine--tRNA ligase, cytoplasmic	Lars1	1178	134.1	7.05	677951.8	763853.3	806086.7	746176.0	786285.2	827637.4	695032.8	718561.1	798429.0	768787.9	954454.6	981098.8	1107408.0	997526.9
Q9JLV5	Cullin-3	Cul3	768	88.9	8.46	644264.3	759904.8	677360.2	696010.2	718844.1	680162.8	601632.8	641795.8	681038.5	735480.1	472377.5	567020.8	491670.1	527469.7
O08553	Dihydropyrimidinase-related protein 2	Dpysl2	572	62.2	6.38	1590396.1	1350482.4	1638766.2	1185713.0	1981784.3	2115471.8	1971205.9	1780387.6	1601484.5	1654126.8	1312941.0	1252977.5	1315399.4	1087838.6
Q8CDG1	Piwi-like protein 2	Piwil2	971	109.4	8.97	844742.2	1138166.7	1069652.6	1242557.7	810964.4	686885.9	612546.4	772785.3	923131.0	990069.1	1028953.2	1393437.1	1306154.2	1569281.7
Q9EQ20	Methylmalonate-semialdehyde dehydrogenase [acylating], mitochondrial	Aldh6a1	535	57.9	8.07	1675711.1	1638115.2	1653154.1	1679388.1	1671445.8	1637894.3	2146749.3	1729867.4	1662623.1	1612972.4	2281149.4	1860099.6	1981343.5	2011633.0
P30416	Peptidyl-prolyl cis-trans isomerase FKBP4	Fkbp4	458	51.5	5.72	3307216.2	3329636.7	3424131.9	3276899.7	3053814.1	3027993.4	2964407.1	3202249.6	3369805.2	3371624.9	2623041.4	2966487.3	2537714.1	2850120.3
P17427	AP-2 complex subunit alpha-2	Ap2a2	938	104	6.93	399953.8	380295.1	400296.7	394414.7	408017.0	403608.4	405267.3	394393.7	361348.4	369368.9	388387.6	409736.1	351347.0	372147.1
Q80X90	Filamin-B	Flnb	2602	277.7	5.71	428312.4	429834.7	459502.3	441846.3	440892.4	466768.9	466269.6	454546.4	418386.6	425788.9	415080.5	432722.8	383904.8	404584.9
O55201	Transcription elongation factor SPT5	Supt5h	1082	120.6	5.05	766016.3	820093.5	848063.5	815827.9	882123.4	847536.4	801172.2	728189.7	856097.2	873548.2	781258.5	830701.5	840705.6	850063.7
A2A432	Cullin-4B	Cul4b	970	110.6	8.37	1051064.0	1097686.0	1014310.3	1021715.8	1174308.5	1094454.4	1015856.8	958121.5	1077026.4	1138221.9	717676.2	639079.8	682076.8	605711.2
Q91VD9	NADH-ubiquinone oxidoreductase 75 kDa subunit, mitochondrial	Ndufs1	727	79.7	5.72	1264406.8	1355236.0	1296324.3	1334037.2	1265685.1	1198591.6	1179157.1	1199327.2	1300905.7	1291399.3	1758623.5	1705938.4	1773867.3	1671827.9
Q8BJ71	Nuclear pore complex protein Nup93	Nup93	819	93.2	5.72	1393993.4	1453637.6	1437626.5	1497515.1	1446271.3	1389091.0	1466864.7	1359649.9	1458438.1	1515542.5	897267.5	931069.6	927219.3	926528.5
Q8CGK3	Lon protease homolog, mitochondrial	Lonp1	949	105.8	6.57	1426629.8	1277734.2	1330148.7	1273577.8	1414528.2	1403535.0	1477034.2	1360487.9	1303058.1	1319863.4	864973.2	837245.9	784723.9	803773.6
O08528	Hexokinase-2	Hk2	917	102.5	6.11	1212971.1	1153754.4	1162398.4	1172865.2	1306802.5	1070754.7	1222576.5	1177270.2	1151468.1	1165695.5	914075.8	973531.2	948641.4	890347.6
Q8K4Z5	Splicing factor 3A subunit 1	Sf3a1	791	88.5	5.22	631724.2	603553.2	668595.4	660601.4	708080.0	631888.3	625865.4	593457.5	655760.4	634369.8	727672.3	790699.4	737821.2	810264.3
Q7TMK9	Heterogeneous nuclear ribonucleoprotein Q	Syncrip	623	69.6	8.59	1765857.6	1646307.3	2081368.4	1610230.8	1808092.0	1652593.3	1658657.9	1849821.5	1700384.4	1665024.0	697758.3	692444.0	716351.6	648904.7
Q8K310	Matrin-3	Matr3	846	94.6	6.25	1523605.2	1778789.5	1795139.7	1805619.5	1673129.8	1539188.0	1455277.1	1485059.9	1619890.6	1776188.7	1697495.9	1927360.4	1752936.1	1880911.3
Q8BML9	Glutamine--tRNA ligase	Qars1	775	87.6	7.31	984546.8	1004193.1	1066864.6	989543.1	1071741.9	1022442.9	928124.3	989935.8	1057255.0	1069707.7	1011481.2	1058136.0	1025018.2	1004632.9
Q5SUR0	Phosphoribosylformylglycinamidine synthase	Pfas	1337	144.5	5.67	819513.7	794154.5	838834.0	777962.6	876185.0	913989.4	895010.3	871737.2	870662.7	839744.6	620664.1	600401.6	585183.2	590813.6
P35564	Calnexin	Canx	591	67.2	4.64	2408261.0	2466027.0	2578803.1	2624652.7	2225447.6	2123908.1	2250300.5	2128574.9	2479316.8	2548557.8	2139421.9	2331554.3	2354762.2	2428564.9
P16125	L-lactate dehydrogenase B chain	Ldhb	334	36.5	6.05	9468777.7	7654299.1	8641362.2	7934194.9	9621160.6	9142156.2	10966122.8	9092595.2	9571948.4	9306458.6	10871606.0	10539574.1	9682965.4	9720399.7
Q8BY87	Ubiquitin carboxyl-terminal hydrolase 47	Usp47	1376	157.4	5.11	527065.2	555515.5	552564.0	568581.2	521235.3	510160.0	421247.3	449443.6	495666.7	531844.2	706984.1	784212.4	782884.5	779619.1
Q61496	ATP-dependent RNA helicase DDX4	Ddx4	702	76.4	6.09	1337999.4	1609565.7	1709830.8	1852131.2	1271258.1	1115367.8	979741.5	1207773.9	1399085.0	1457839.5	1002917.7	1211444.0	1152304.3	1286073.8
Q8K411	Presequence protease, mitochondrial	Pitrm1	1036	117.3	7.2	1222680.4	1301209.5	1311403.6	1302980.1	1316760.9	1232267.7	1446066.8	1125760.2	1287665.2	1301696.2	1464865.8	1456897.4	1353749.4	1385883.6
P48036	Annexin A5	Anxa5	319	35.7	4.96	6730076.1	5061076.7	6257644.4	5489059.5	6792270.5	8909609.1	9919556.6	8734019.5	5839485.2	5245896.9	5949792.4	5047132.2	5314196.1	5292387.4
P08249	Malate dehydrogenase, mitochondrial	Mdh2	338	35.6	8.68	7363381.7	6741792.8	7101393.2	6774553.5	7760676.1	7255428.1	7207197.5	7379019.4	7499292.6	7372875.3	7600138.2	6817639.0	7126983.1	6587917.9
Q91YQ5	Dolichyl-diphosphooligosaccharide--protein glycosyltransferase subunit 1	Rpn1	608	68.5	6.46	3246748.1	3639668.8	3438670.3	3678319.0	3318930.4	3113093.8	2801211.3	3184706.9	3308053.6	3388918.8	2002904.3	2230654.1	2032513.7	2196111.1
Q8VDJ3	Vigilin	Hdlbp	1268	141.7	6.87	1322841.1	1218873.6	1304627.8	1239625.4	1281767.3	1295646.4	1179095.7	1353438.9	1212275.8	1140877.0	1167424.3	1106108.6	1049228.6	1102565.8
O88738	Baculoviral IAP repeat-containing protein 6	Birc6	4882	531.8	6.07	247144.5	264441.6	237763.6	255603.8	257757.3	254080.8	227961.2	258191.0	248189.8	265372.1	234229.2	278186.3	219044.2	208760.6
Q9JMB7	Piwi-like protein 1	Piwil1	862	98.5	9.38	940463.3	1455697.0	1381408.3	1739382.5	703533.6	696778.0	578411.1	830834.2	1039000.1	1230966.7	973182.5	1462674.9	1455945.5	1877318.0
Q61543	Golgi apparatus protein 1	Glg1	1175	133.6	6.84	782858.3	761779.7	705368.7	710736.2	746930.2	738142.2	745081.8	687395.7	780914.5	797689.6	812866.5	746178.2	879518.4	723861.8
P23953	Carboxylesterase 1C	Ces1c	554	61	5.06	4166113.2	3298921.2	2903793.6	2673093.5	2227226.3	2721267.0	3265379.3	3723867.2	2757271.0	2661342.6	3288480.4	2798323.5	2395026.0	2410013.6
P42567	Epidermal growth factor receptor substrate 15	Eps15	897	98.4	4.6	1232599.4	1310170.7	1192251.8	1213461.1	1251951.3	1172070.9	1134649.9	1110376.8	1204585.0	1286719.8	911223.4	985238.2	938417.0	947328.6
P26043	Radixin	Rdx	583	68.5	6.2	1272611.1	1168202.0	1161195.8	1110183.5	1270325.5	1285250.5	1222098.9	1271194.7	1219333.6	1242807.9	1633052.3	1535566.3	1535154.7	1432424.9
Q60854	Serpin B6	Serpinb6	378	42.6	5.74	2999449.2	2334019.4	2737440.0	2227427.6	2854276.5	3030499.6	3278528.0	3021639.1	2800331.9	2709914.0	3845233.8	3157847.5	3427078.4	2940911.2
Q9JLN9	Serine/threonine-protein kinase mTOR	Mtor	2549	288.6	7.17	779475.5	792548.7	773589.5	743229.5	792091.7	784934.4	785543.2	773027.8	812271.0	787405.6	502596.6	530741.8	510096.4	508149.8
P50544	Very long-chain specific acyl-CoA dehydrogenase, mitochondrial	Acadvl	656	70.8	8.75	802429.7	851102.1	809856.9	820861.8	829167.9	728783.1	674567.9	757215.4	795223.5	739576.3	550377.4	575469.9	522236.9	566110.4
Q9CU62	Structural maintenance of chromosomes protein 1A	Smc1a	1233	143.1	7.64	705301.8	646925.7	685272.6	634949.5	767052.9	786346.2	815845.2	666190.7	697615.7	699645.3	244640.5	250531.8	242482.6	225393.3
P28665	Murinoglobulin-1	Mug1	1476	165.2	6.42	1277129.7	1397059.5	926733.5	1320274.4	700087.2	787769.4	877197.7	1050537.2	970194.2	845248.9	1060708.1	1214656.1	721168.7	1103766.4
Q05D44	Eukaryotic translation initiation factor 5B	Eif5b	1216	137.5	5.59	865376.4	909922.9	933943.1	905873.7	923352.2	955202.4	869109.0	821834.1	929755.7	948814.0	520981.5	530524.4	567987.0	526847.4
Q8BFR5	Elongation factor Tu, mitochondrial	Tufm	452	49.5	7.56	1875491.6	1919892.5	1793534.8	1915238.7	1976917.6	1907811.1	2028477.2	1968220.8	1981124.6	1994901.9	1455086.7	1556735.4	1552714.4	1586490.7
Q9Z1X4	Interleukin enhancer-binding factor 3	Ilf3	898	96	8.76	2059634.5	2169539.2	2236445.3	2166309.7	2274695.5	2119212.1	2119914.5	1987048.6	2170075.1	2266604.8	1266864.3	1276228.4	1292190.9	1284935.4
P42859	Huntingtin	Htt	3119	344.5	6.29	484582.2	508072.1	483212.0	501684.3	535389.8	498772.7	462339.5	474597.8	515369.9	521651.7	454254.0	447005.7	442055.8	423856.8
P80317	T-complex protein 1 subunit zeta	Cct6a	531	58	7.08	3240907.5	3482748.8	3846687.5	3787240.5	3268189.0	3008687.5	2678898.3	3070790.4	3425513.4	3467747.7	2611449.1	2981811.0	3007896.2	3026024.2
Q8VHM5	Heterogeneous nuclear ribonucleoprotein R	Hnrnpr	632	70.8	8.13	911107.4	787807.7	972591.7	837705.1	1008223.3	995548.7	1024084.4	965848.6	920972.4	861571.1	1827864.4	1839397.3	1953436.0	1770820.1
Q8C1A5	Thimet oligopeptidase	Thop1	687	78	6.06	2302288.5	2425108.1	2612014.5	2502191.5	2463232.9	2543115.0	2571926.7	2243271.9	2686815.4	2683708.5	1516659.4	1637829.6	1707086.5	1650990.8
Q8BU30	Isoleucine--tRNA ligase, cytoplasmic	Iars1	1262	144.2	6.55	1262625.3	1361463.2	1423437.3	1293862.0	1380364.1	1328220.9	1250766.7	1277148.2	1344989.0	1388934.0	967718.9	987294.9	947087.4	1041523.0
Q9CXY6	Interleukin enhancer-binding factor 2	Ilf2	390	43	5.26	1733672.1	1664406.8	1940117.3	1720451.2	2326849.5	1728975.9	1813462.5	1645574.2	1761169.1	1805647.9	1988493.1	2135645.0	2624650.8	2238772.8
Q8JZQ9	Eukaryotic translation initiation factor 3 subunit B	Eif3b	803	91.3	5.02	894073.3	929008.4	888274.7	886394.5	925815.8	870108.3	841118.0	828807.6	851945.7	881655.9	493226.2	489284.1	527210.5	509046.2
P17879	Heat shock 70 kDa protein 1B	Hspa1b	642	70.1	5.72	58054.1	48046.6	61949.0	58541.8	59206.5	67747.7	60506.8	52616.9	58469.9	62823.2	#N/A	#N/A	#N/A	#N/A
Q1HFZ0	RNA cytosine C(5)-methyltransferase NSUN2	Nsun2	757	85.4	6.58	467256.8	494128.1	487803.0	482866.6	527193.0	545744.6	479738.4	474577.8	520832.8	546456.4	739364.8	689482.1	708052.8	648908.5
P61979	Heterogeneous nuclear ribonucleoprotein K	Hnrnpk	463	50.9	5.54	4109348.2	4425193.2	4748226.7	4531458.9	4795365.3	4301851.4	4267792.7	4201873.7	4209713.4	4311734.3	5042310.9	4781463.8	5350624.4	4921273.7

P09411	Phosphoglycerate kinase 1	Pgk1	417	44.5	7.9	3027739.2	2628368.9	2678719.1	2679753.4	2463495.3	2584815.5	3108898.9	2893267.3	2680357.7	2673198.6	2252059.6	2278864.9	1975005.7	2169763.8
Q8QZT1	Acetyl-CoA acetyltransferase, mitochondrial	Acat1	424	44.8	8.51	2476757.3	2211272.0	2593443.3	2564299.3	2572053.7	2394195.5	2744747.0	2567827.3	2507492.4	2384757.3	1509436.5	1515430.5	1531419.0	1651154.9
O35945	Aldehyde dehydrogenase, cytosolic 1	Aldh1a7	501	54.6	7.71	313050.8	327791.8	275782.4	348411.7	243877.4	229184.4	240415.3	280911.5	251027.6	274427.5	149159.8	146203.5	122385.3	158759.5
Q9Z0X1	Apoptosis-inducing factor 1, mitochondrial	Aifm1	612	66.7	9.17	502499.4	513697.7	524439.8	522810.5	545665.8	536624.6	518623.4	504358.0	520692.6	538151.1	527180.1	683516.2	526490.4	595492.4
Q61147	Ceruloplasmin	Cp	1061	121.1	5.85	728995.5	643660.2	629287.5	621867.0	600174.5	653541.4	962263.2	732911.3	723803.1	738066.3	774088.8	689377.5	665684.0	629889.9
O35286	Pre-mRNA-splicing factor ATP-dependent RNA helicase DHX15	Dhx15	795	90.9	7.46	1774673.4	1917249.1	1684025.8	1789305.7	1822287.2	1723465.2	1702167.9	1617369.9	1869689.2	1931080.5	1380777.6	1342628.1	1374025.0	1278053.0
Q8R1A4	Dedicator of cytokinesis protein 7	Dock7	2130	241.3	6.71	682456.5	618504.8	631177.6	621867.7	632994.6	623054.1	661125.6	721308.6	648856.6	677600.6	392724.3	399110.4	382465.2	401520.5
Q922R8	Protein disulfide-isomerase A6	Pdia6	440	48.1	5.14	1734910.6	1836594.6	1932243.9	1864652.6	1762779.4	1659399.0	1369739.6	1699868.2	1698959.9	1710005.3	1714183.9	1664068.7	1923168.5	1702366.7
Q91ZW3	SWI/SNF-related matrix-associated actin-dependent regulator of chromatin subfamily A member 5	Smarca5	1051	121.6	8.15	1198350.8	1320709.2	1369027.5	1363324.2	1309096.9	1220811.5	1053112.2	1081379.2	1249189.6	1316774.6	1254070.6	1359679.7	1393827.5	1424631.7
P10649	Glutathione S-transferase Mu 1	Gstm1	218	26	7.94	13917698.9	10972054.6	12907830.9	11335557.8	16307366.6	18253821.0	18142733.0	14962428.1	13831827.8	12491612.7	11351983.3	9943307.0	11459634.5	10514509.9
Q99KE1	NAD-dependent malic enzyme, mitochondrial	Me2	589	65.8	7.61	980425.3	878981.3	906064.3	816457.5	1006503.9	948114.5	1026189.7	919442.9	1015985.5	1019908.6	1037723.6	911537.3	1109088.0	977618.1
P49718	DNA replication licensing factor MCM5	Mcm5	734	82.4	8.29	1326366.8	1283461.9	1338259.0	1218210.2	1414242.6	1439138.3	1200633.8	1263189.7	1253096.4	1331073.3	790418.6	1384018.6	1440121.6	732455.4
Q61696	Heat shock 70 kDa protein 1A	Hspa1a	641	70	5.72	294685.4	285909.5	300359.8	294229.5	306978.8	330533.0	521800.3	300149.3	376043.2	398606.8	799461.5	669991.3	813749.9	655644.7
Q922F4	Tubulin beta-6 chain	Tubb6	447	50.1	4.89	125686.3	114209.4	117290.9	109137.4	127239.7	125787.1	127002.9	109111.8	127304.7	123157.7	138738.2	150423.5	121170.9	141765.7
P29341	Polyadenylate-binding protein 1	Pabpc1	636	70.6	9.5	3746388.9	4119071.5	4367779.8	4633896.4	3538380.9	3259219.7	3111865.1	3614949.4	3832910.3	4064892.4	3216132.6	3509571.0	3696653.1	3856265.7
O54774	AP-3 complex subunit delta-1	Ap3d1	1199	135	7.37	689917.6	730212.9	634402.5	645691.1	670669.6	623427.5	563114.2	617156.3	659077.4	680572.4	449657.3	439928.0	425834.6	430652.0
P51660	Peroxisomal multifunctional enzyme type 2	Hsd17b4	735	79.4	8.57	2467312.5	2316039.4	2292106.7	2440821.1	2448385.2	2335304.8	2154417.5	2448980.5	2422251.2	2538284.0	3168549.4	2887812.2	3041762.9	3067165.5
Q6PIE5	Sodium/potassium-transporting ATPase subunit alpha-2	Atp1a2	1020	112.1	5.55	186225.6	184751.3	180218.1	196832.1	185682.3	194466.8	221338.9	178627.9	178170.6	183880.0	200391.0	204306.1	189193.8	212651.7
Q99JF8	PC4 and SFRS1-interacting protein	Psip1	528	59.7	9.13	1109432.5	1416053.4	1347711.6	1644946.5	976775.9	967829.2	974656.3	936367.7	1188813.3	1340611.2	880097.9	1225537.8	1152407.6	1449048.0
Q3U0V1	Far upstream element-binding protein 2	Khsrp	748	76.7	7.33	875117.3	949841.0	1035238.6	927347.6	1040156.5	1005569.4	1046999.1	888086.5	936608.3	989591.3	1285852.3	1432553.6	1403229.2	1452469.8
P68254	14-3-3 protein theta	Ywhaq	245	27.8	4.78	914446.2	987629.7	1145938.9	1092563.5	1119511.0	997351.9	971032.7	1143224.3	1055205.6	902266.1	891533.9	1049059.3	1095752.0	1108395.4
O08709	Peroxiredoxin-6	Prdx6	224	24.9	6.01	3937301.6	3351519.8	4654258.1	3525815.9	5083098.0	5170294.1	4984107.3	5085270.5	4295116.4	3831526.6	3718370.1	3142686.3	4012644.2	3281380.7
Q8R081	Heterogeneous nuclear ribonucleoprotein L	Hnrnpl	586	63.9	8.1	1473185.3	1711448.3	1680237.6	1777436.8	1707679.7	1567670.3	1848211.4	1457438.8	1817694.1	1855136.6	1483414.7	1630752.4	1936870.2	1610932.3
P97310	DNA replication licensing factor MCM2	Mcm2	904	102	5.72	782000.5	799150.2	795328.7	779198.6	839333.9	797132.5	674049.3	748344.6	793741.5	832337.7	654634.1	680737.5	682793.5	631153.6
Q6PDN3	Myosin light chain kinase, smooth muscle	Mylk	1941	212.8	6.25	707699.2	631833.8	672393.5	625632.2	679901.5	749703.0	860946.3	972192.7	741793.3	685009.0	1235755.6	1154285.6	1206158.2	1148112.0
P25206	DNA replication licensing factor MCM3	Mcm3	812	91.5	5.55	817970.4	870836.0	947417.6	839784.7	994313.9	919420.6	773466.1	892875.8	897781.9	901481.3	789244.6	863803.8	943362.8	839522.1
P61922	4-aminobutyrate aminotransferase, mitochondrial	Abat	500	56.4	8.09	1107677.6	978256.3	1132176.7	1021280.7	1295337.1	1189407.3	1430736.8	1024993.3	1213679.3	1212952.7	1596088.7	1522274.8	1607630.0	1574006.2
P10518	Delta-aminolevulinic acid dehydratase	Alad	330	36	6.79	1064074.0	1498104.5	1627939.7	1429930.3	1752949.5	1711897.8	1922545.5	1184237.1	1391974.1	1376693.1	1043660.3	1180576.6	1310063.6	1235596.0
Q8NZJ6	Eukaryotic translation initiation factor 4 gamma 1	Eif4g1	1600	176	5.4	468243.6	441297.7	466541.6	429104.2	454659.2	412636.9	394243.4	434042.8	420283.4	419751.7	819138.3	795352.8	771893.5	792045.6
P46471	26S proteasome regulatory subunit 7	Psmc2	433	48.6	5.95	928473.1	1023674.1	1035999.4	998845.1	1048580.7	1009382.8	958272.3	919963.0	1004851.0	997435.5	1343439.6	1436190.9	1447310.0	1475999.7
P43247	DNA mismatch repair protein Msh2	Msh2	935	104.1	5.96	875782.8	940313.6	935751.6	866506.5	1016446.6	941058.5	939889.1	822795.6	955297.7	1014953.7	712387.7	849328.1	817097.2	794343.3
Q9CUU3	Synaptonemal complex protein 2	Sycp2	1500	172	7.93	488899.5	719764.2	602340.4	703718.0	575773.4	518811.5	399698.6	414768.0	580334.1	637298.1	474849.1	517482.3	541818.6	525440.1
Q99JY0	Trifunctional enzyme subunit beta, mitochondrial	Hadhb	475	51.4	9.38	3112910.0	2881097.0	3139642.3	3038690.9	3453181.6	3077881.5	3099089.8	3130798.2	3188225.4	3232249.1	2212862.7	2169452.0	2533328.3	2290882.3
Q8VC30	Triokinase/FMN cyclase	Tkfc	578	59.7	6.92	1280641.3	1494932.0	1503060.4	1734492.0	1142876.0	955032.5	982185.9	1083784.5	1270997.4	1414879.5	832799.9	994454.1	1118748.2	1375731.5
Q9DBG6	Dolichyl-diphosphooligosaccharide--protein glycosyltransferase subunit 2	Rpn2	631	69	5.81	1297936.7	1306394.0	1237213.8	1325545.9	1271828.6	1093143.8	1225152.7	1155659.4	1262310.8	1316615.7	1360232.5	1455941.6	1268244.3	1508329.0
Q8CG48	Structural maintenance of chromosomes protein 2	Smc2	1191	134.2	8.41	573456.8	648481.6	589707.8	610066.4	663723.4	628638.3	538052.1	562799.9	610070.0	639022.4	402625.1	452855.2	409846.6	394241.9
Q9Z2V5	Histone deacetylase 6	Hdac6	1149	125.7	5.78	1046877.9	1008402.6	1020841.0	979996.5	1101161.9	1017328.8	845097.3	1001880.1	1028771.1	1055939.1	898275.7	989286.0	910970.2	965001.2
Q9R1T2	SUMO-activating enzyme subunit 1	Sae1	350	38.6	5.36	2258471.4	2623131.3	2819253.5	2879516.1	2788255.2	2607343.3	2462278.6	2135595.7	2832080.3	2928832.9	2005025.0	2200225.5	2237005.2	2285360.1
O88569	Heterogeneous nuclear ribonucleoproteins A2/B1	Hnrnpa2b1	353	37.4	8.95	8922382.1	9773960.0	10742461.3	10354500.5	10901154.4	10222393.5	10442491.5	9520119.2	10508977.3	9992822.2	9034578.8	10456425.7	10251340.1	9187180.6
P70296	Phosphatidylethanolamine-binding protein 1	Pebp1	187	20.8	5.4	4050713.0	3609494.2	4732785.7	3855652.2	4290957.3	4085760.2	4726305.7	4672583.6	4362358.8	4027816.0	4344418.9	3824550.0	4909521.1	4098969.4
P58281	Dynamin-like 120 kDa protein, mitochondrial	Opa1	960	111.3	7.55	1211182.9	1214385.6	1229471.3	1222029.9	1282986.0	1229955.6	1295308.1	1248149.3	1270730.5	1210482.1	1215471.6	1169320.1	1274237.9	1252467.3
Q3URD3	Sarcolemmal membrane-associated protein	Slmap	845	96.9	5.33	1648568.9	1535298.0	1711225.8	1134462.3	1332929.4	1537438.0	1764281.0	1788475.0	1729807.6	1610547.0	1285913.2	1173734.6	1270171.3	1232403.6
P26443	Glutamate dehydrogenase 1, mitochondrial	Glud1	558	61.3	8	1674859.6	1586962.7	1609377.8	1644598.1	1770940.2	1809378.5	2189117.7	1819007.9	1782750.9	1665080.4	714654.6	678544.5	675884.1	686258.2
Q9D6Z1	Nucleolar protein 56	Nop56	580	64.4	9.14	854151.0	855531.1	778306.4	814975.8	894938.1	851466.2	918285.4	776754.0	886967.1	954623.0	598340.3	644785.1	666917.9	614958.5
A8DUK4	GLOBIN domain-containing protein	Hbb-bs	147	15.7	7.69	11611064.4	16964930.4	10533957.1	18101494.4	15419562.9	12539063.9	20412564.3	22897636.0	13335513.5	17467604.8	12783267.1	11370531.9	11036606.1	15676221.8
Q9D8E6	60S ribosomal protein L4	Rpl4	419	47.1	11	2469331.3	2921602.0	2614891.1	2710050.5	2220621.4	2096336.2	1793319.2	2422215.8	2398104.7	2473760.1	2776545.5	3142156.3	3220432.6	3186683.2
P31001	Desmin	Des	469	53.5	5.27	554018.6	515511.3	478468.1	518703.5	550449.0	606055.8	701593.7	646140.6	519723.0	518727.9	521467.0	458427.4	434485.0	494139.6
B2RR83	3'-5' RNA helicase YTHDC2	Ythdc2	1445	161	8.51	398729.3	467328.9	440503.7	465717.9	427929.1	434577.7	358764.0	380664.4	435922.9	460902.9	461225.4	527708.2	497623.2	511621.2
P63101	14-3-3 protein zeta/delta	Ywhaz	245	27.8	4.79	3752514.3	3685837.2	4645311.3	4267048.7	4569166.1	4302445.4	4121807.6	4860414.8	4602536.8	3888392.3	3142419.4	3107761.1	4037910.2	3716612.8
P60122	RuvB-like 1	Ruvbl1	456	50.2	6.42	603311.7	667263.1	691055.4	744547.7	546311.7	563319.2	550465.7	542950.5	594171.5	621353.5	896258.1	964610.9	962372.7	1023006.6
Q9EQH3	Vacuolar protein sorting-associated protein 35	Vps35	796	91.7	5.44	682366.4	769047.1	751517.9	771960.4	717672.9	721962.1	637804.3	671562.0	732028.6	706408.9	825384.2	928509.4	842965.6	915356.9
Q00PI9	Heterogeneous nuclear ribonucleoprotein U-like protein 2	Hnrnpul2	745	84.9	4.89	1288299.1	1257654.1	1324917.1	1267142.2	1431932.0	1442300.4	1519410.8	1393504.8	1327804.3	1355105.6	1230433.6	1238301.2	1264832.5	1236077.0
P19137	Laminin subunit alpha-1	Lama1	3083	337.9	6.71	653321.3	640288.1	601738.1	633236.0	652094.8	678467.8	714986.0	600815.1	684761.4	699995.2	607767.6	703338.9	652822.0	695557.2
Q9D964	Glycine amidinotransferase, mitochondrial	Gatm	423	48.3	7.88	1014371.3	1006193.8	897504.5	875829.1	1020901.3	1038736.5	919080.8	1029809.8	945985.2	1018354.3	1361338.6	1358323.7	1282852.2	1189354.4
Q80W21	Glutathione S-transferase Mu 7	Gstm7	218	25.7	6.8	1711082.5	1434652.5	1983801.4	1698085.7	1702717.4	1684951.2	2061587.0	2413475.9	1671403.6	1544550.3	928547.4	934800.5	823432.4	1063802.5
P40124	Adenylyl cyclase-associated protein 1	Cap1	474	51.5	7.52	1574649.3	1732999.6	1596219.7	1661475.8	1519980.6	1521294.5	1627220.2	1557731.2	1570013.2	1573741.5	697299.9	731981.3	670168.0	688184.7
P10630	Eukaryotic initiation factor 4A-II	Eif4a2	407	46.4	5.48	5583103.4	6202460.6	6539480.7	6378208.1	5028281.0	5272327.0	5141585.8	5554621.1	5868234.8	6032258.9	4153478.2	4050556.1	4176694.7	3926699.5
P02088	Hemoglobin subunit beta-1	Hbb-b1	147	15.8	7.65	403298.1	70411.5	243148.2	350451.1	86576.5	66749.3	55213.5	70619.9	297613.7	124312.2	13207824.9	1353158.2	8660242.9	18841030.0
Q9D1A2	Cytosolic non-specific dipeptidase	Cndp2	475	52.7	5.66	955767.2	931037.2	955397.7	1006468.2	911448.0	864749.7	1076650.1	999720.6	958712.5	966041.4	699624.5	757189.3	621137.2	745338.6
A0A5F8MPH8	Tenascin XB	Tnxb	4356	473.8	5.39	324589.9	348515.0	368118.0	301152.0	427852.9	587061.0	566692.3	426658.3	381501.9	376281.0	307356.9	288676.0	300466.2	277728.7
P52293	Importin subunit alpha-1	Kpna2	529	57.9	5.68	1016911.0	1256404.1	1309576.2	1320357.8	1174767.6	1011407.4	798654.0	910518.3	1178751.0	1280087.0	1181108.3	1504601.2	1492682.8	1540477.8
O70311	Glycylpeptide N-tetradecanoyltransferase 2	Nmt2	529	60.4	7.8	922276.1	996804.2	983485.2	920477.2	1150599.3	1072912.9	924236.9	918514.5	1098514.3	1120645.4	1466712.1	1491913.6	1585137.0	1410237.9
P26040	Ezrin	Ezr	586	69.4	6.1	650198.3	709374.1	673797.7	717473.4	755366.0	802123.9	860362.9	720110.9	705273.4	719515.9	734509.2	768585.9	766454.0	784386.0
Q9D0F9	Phosphoglucomutase-1	Pgm1	562	61.4	6.57	2247207.1	1995638.0	2204614.1	1994525.9	2396503.4	2351483.6	2579667.1	2187642.1	2342284.3	2335773.5	1621800.3	1663156.0	1738882.8	1590788.9
P39447	Tight junction protein ZO-1	Tjp1	1745	194.6	6.64	556595.4	521807.8	552659.4	506993.4	603219.5	615473.9	640559.8	524876.1	556560.9	575283.4	259135.2	232569.5	217859.3	224180.6
Q62148	Retinal dehydrogenase 2	Aldh1a2	518	56.6	5.74	675101.5	774583.5	740055.9	745639.6	715854.6	688028.3	749424.6	638760.2	771949.4	779756.1	618463.7	688893.0	682192.7	678546.9
P32067	Lupus La protein homolog	Ssb	415	47.7	9.77	2047865.8	2167525.2	2265155.7	2077294.9	2319431.0	2233622.4	2041657.6	1981783.9	2299593.7	2314652.8	3538942.3	3515611.5	3922436.9	3366565.3
Q9JLI8	Squamous cell carcinoma antigen recognized by T-cells 3	Sart3	962	109.6	5.24	902959.6	912700.7	895687.4	884464.5	928789.2	935995.1	1020274.2	798389.2	962246.2	998204.9	538131.6	576590.2	546897.5	550865.9
P13864	DNA (cytosine-5)-methyltransferase 1	Dnmt1	1620	183.1	7.74	509346.0	516523.9	534073.5	523350.9	589009.2	575640.4	541726.9	519249.8	536848.8	545755.1	746921.1	776584.6	735293.2	752641.8
O08788	Dynactin subunit 1	Dctn1	1281	141.6	5.9	656064.7	682811.9	685768.2	710715.7	650100.5	618374.8	595376.9	619469.8	639318.3	656766.7	439113.3	460786.9	440162.4	471176.8
Q61753	D-3-phosphoglycerate dehydrogenase	Phgdh	533	56.5	6.54	2341477.2	2177165.4	2048195.2	2088007.4	1992088.6	1988474.4	2143702.6	2161334.4	2178598.5	2185670.2	1887920.8	1677542.9	1606187.2	1580185.3
Q8BIJ6	Isoleucine--tRNA ligase, mitochondrial	Iars2	1012	112.7	6.81	804642.2	789015.5	836571.3	809373.7	843438.6	795035.9	805831.0	786716.7	810521.8	822608.1	625141.5	597998.9	599245.9	610135.7
P08775	DNA-directed RNA polymerase II subunit RPB1	Polr2a	1970	217	7.37	595285.9	649875.3	583251.9	607645.2	623134.5	611579.2	616255.0	568094.7	627486.6	644130.0	457676.9	494175.7	474955.0	476089.9
P17225	Polypyrimidine tract-binding protein 1	Ptbp1	527	56.4	8.34	454976.7	460904.4	438827.9	428455.0	500698.4	475072.8	492469.7	437132.9	456774.4	463837.9	842745.3	757106.2	805421.6	745881.6
Q9R0P5	Destrin	Dstn	165	18.5	7.97	2470421.2	2360047.2	2206622.2	2230863.9	2363881.1	2559242.5	2743417.2	2504376.4	2390024.0	2354765.3	1409968.5	1431074.6	1300224.4	1400471.6
Q571H0	Nucleolar pre-ribosomal-associated protein 1	Urb1	2274	254.5	6.96	289084.8	330061.0	338256.7	332719.6	333177.4	306977.9	247742.1	276720.0	298303.9	313511.8	337489.4	369326.3	338704.0	363772.9
Q4VA53	Sister chromatid cohesion protein PDS5 homolog B	Pds5b	1446	164.3	8.5	465125.2	519756.9	489917.5	448939.4	587556.4	627165.4	529544.4	543071.3	510440.6	505481.4	1131042.6	1117990.0	1136082.0	1036861.8
Q8R0G9	Nuclear pore complex protein Nup133	Nup133	1155	128.5	5.2	454766.2	487715.0	449542.6	469557.8	479524.1	482283.1	449158.6	416735.0	484527.5	480622.4	324424.2	309081.5	300921.5	303210.7
Q69ZN7	Myoferlin	Myof	2048	233.2	6.16	225673.5	229990.7	223815.4	214969.5	270322.9	277675.8	288514.1	233044.0	244412.5	231350.5	251307.2	237947.9	256509.4	253546.1
Q61584	Fragile X mental retardation syndrome-related protein 1	Fxr1	677	76.2	6.98	590512.5	604109.4	609611.4	601205.9	605393.7	603015.5	542153.8	570943.6	575420.6	629589.8	911954.5	1093296.5	1103156.1	1085817.9
P23249	Putative helicase MOV-10	Mov10	1004	113.5	9.01	1453399.3	1528649.9	1366729.4	1378192.2	1374970.3	1338364.0	1319362.8	1407180.5	1428634.9	1451785.8	758934.7	736630.6	703045.8	685024.6
O35685	Nuclear migration protein nudC	Nudc	332	38.3	5.26	1837020.8	2375034.6	1749249.5	2281482.7	1670259.7	1588537.3	1769783.7	1736127.5	2121817.0	2125012.7	2320755.3	2216905.3	2572762.8	2471640.4
P18760	Cofilin-1	Cfl1	166	18.5	8.09	2343677.1	2231585.1	2367395.6	2226139.9	2522025.6	2387751.9	2248768.0	2501974.7	2491312.7	2228455.3	3739445.7	3406446.0	3776480.3	3530508.3
Q62167	ATP-dependent RNA helicase DDX3X	Ddx3x	662	73.1	7.18	1249222.8	1658978.3	1250979.1	1428299.0	1324164.5	1213279.0	957475.0	1267876.8	1254207.2	1421028.3	2097418.8	2011137.7	1866947.2	1980950.8
Q9CZU3	Exosome RNA helicase MTR4	Mtrex	1040	117.6	6.4	512971.2	514941.1	528094.4	509786.2	577128.4	575488.9	488742.7	459639.7	519222.9	541105.3	669172.9	677933.3	658808.7	610492.3
G5E829	Plasma membrane calcium-transporting ATPase 1	Atp2b1	1220	134.7	5.91	466315.3	461215.0	449682.3	414996.7	485492.8	533378.1	461825.3	464240.6	498075.3	471666.6	733063.5	694474.0	673089.9	683236.6
Q501J6	Probable ATP-dependent RNA helicase DDX17	Ddx17	650	72.4	8.59	502762.2	528499.2	606383.4	558111.3	553518.9	611207.3	522351.9	522403.9	565611.6	518065.7	381216.1	392633.7	419461.8	370231.7
Q6P1B1	Xaa-Pro aminopeptidase 1	Xpnpep1	623	69.5	5.54	2840157.1	2777635.4	2529340.2	2888832.2	2367763.6	2135705.6	2050505.3	2491376.6	2734785.4	2689240.7	1805823.1	1788757.0	1679588.9	1868611.1
Q91YR9	Prostaglandin reductase 1	Ptgr1	329	35.5	7.96	2202204.1	2297355.4	2187462.0	2240570.1	2352346.8	2270389.8	2521585.5	2263823.5	2356457.6	2322690.6	1946211.4	2015444.2	1873011.0	1986996.2
Q9CZS1	Aldehyde dehydrogenase X, mitochondrial	Aldh1b1	519	57.5	7.02	998522.8	905902.1	838982.5	980001.4	824312.3	850671.9	903670.3	849855.6	870159.5	890980.7	960134.7	870288.7	851407.3	875892.5
P00342	L-lactate dehydrogenase C chain	Ldhc	332	35.9	8.21	1828348.0	2921717.1	2693925.7	4473903.2	1023872.6	1049185.0	1089718.2	1477370.2	1828196.5	2319424.9	1184473.7	1953149.5	1817888.8	3066137.0
Q9WTM5	RuvB-like 2	Ruvbl2	463	51.1	5.64	999212.2	1046745.5	1083701.3	1138055.0	961788.4	945224.0	916369.1	935274.7	1000918.3	1076769.6	1174976.0	1204021.4	1214050.7	1283150.7

Q9DCD0	6-phosphogluconate dehydrogenase, decarboxylating	Pgd	483	53.2	7.23	2377544.2	2464797.9	2461064.9	2523335.6	2479982.7	2468317.1	2401967.1	2379587.3	2572382.6	2580086.5	3738011.6	3434153.6	3507745.4	3489624.8
Q3UJB0	SAP domain-containing protein	Sf3b2	878	98.1	5.62	1153082.3	1167960.8	1255021.8	1264501.0	1266569.4	1187171.0	1192019.8	1130585.8	1223039.2	1276896.9	1342841.6	1341680.1	1413490.4	1364716.8
P29758	Ornithine aminotransferase, mitochondrial	Oat	439	48.3	6.62	1822557.3	1509532.3	1404124.8	1545949.6	1410259.7	1375744.4	1429830.0	1815267.7	1376619.7	1327375.0	2323161.9	2088231.4	1747763.1	1796735.5
P37913	DNA ligase 1	Lig1	916	102.2	6.81	573341.6	661786.3	631039.6	656385.8	713267.9	714189.5	616288.7	589213.9	673434.0	673315.0	268966.8	321324.0	277918.4	292362.0
Q68FL6	Methionine--tRNA ligase, cytoplasmic	Mars1	902	101.4	7.14	1412853.2	1475395.7	1395056.8	1376220.9	1437150.6	1399403.2	1308555.8	1298925.1	1472513.6	1551428.0	1082958.5	1316734.4	1189378.9	1260079.3
Q9CPY7	Cytosol aminopeptidase	Lap3	519	56.1	7.72	1289718.8	1257895.5	1342237.2	1279306.7	1269412.0	1254290.7	1166919.9	1235887.3	1286896.9	1291158.5	548486.2	575910.3	542340.4	534520.7
P50516	V-type proton ATPase catalytic subunit A	Atp6v1a	617	68.3	5.58	830831.4	902114.8	827282.6	929252.5	834028.6	858849.5	858726.6	766298.4	830432.4	856400.0	750999.3	813145.3	735309.3	743232.9
Q8VHX6	Filamin-C	Flnc	2726	290.9	5.95	127288.0	133744.3	127206.1	131364.1	131347.9	144395.7	133743.0	155627.5	126143.4	130482.2	164663.1	170084.8	176680.5	162225.9
Q61699	Heat shock protein 105 kDa	Hsph1	858	96.3	5.53	684946.5	638402.7	694480.1	646640.9	614288.6	621298.9	621538.6	671212.5	661172.1	589469.9	307180.8	297037.9	300947.9	278711.9
P39054	Dynamin-2	Dnm2	870	98.1	7.43	837019.0	832174.4	860911.9	880611.8	819673.1	827769.3	762495.4	812872.9	851561.4	829758.9	521356.9	655880.7	530088.5	584459.5
Q9ESE1	Lipopolysaccharide-responsive and beige-like anchor protein	Lrba	2856	316.9	5.69	982807.8	936170.3	942890.0	913703.9	1090302.2	931612.4	1011548.8	955017.0	980196.4	952875.9	418604.2	394256.2	440314.3	396863.9
P45376	Aldo-keto reductase family 1 member B1	Akr1b1	316	35.7	7.18	12455057.0	11788166.9	13490969.6	12991827.3	14590923.7	14469566.5	17965148.3	13909045.1	15476921.1	14336501.3	12215444.0	11141658.2	11747726.3	11714027.2
P07759	Serine protease inhibitor A3K	Serpina3k	418	46.9	5.16	1741019.1	2136553.7	914659.0	2150845.1	775439.0	774529.1	840709.8	1139015.8	1195049.2	870476.3	1617327.3	2011996.5	562484.1	2125155.1
P46061	Ran GTPase-activating protein 1	Rangap1	589	63.5	4.68	709327.8	676265.5	666279.5	676384.9	730244.7	684276.7	689697.8	664549.6	689735.1	639190.0	394529.3	403146.0	370263.9	407329.1
Q61941	NAD(P) transhydrogenase, mitochondrial	Nnt	1086	113.8	7.64	900903.6	812445.5	791143.9	821342.6	826941.3	831611.7	989888.5	829758.4	839802.1	827372.8	812899.2	758790.4	699278.6	731190.5
Q9WTI7	Unconventional myosin-Ic	Myo1c	1063	121.9	9.35	653254.3	645015.0	607404.6	610974.2	687972.0	716965.3	767393.8	632242.6	658409.5	663699.4	866948.6	885178.2	837655.2	846048.9
P06745	Glucose-6-phosphate isomerase	Gpi	558	62.7	8.13	1723375.4	1563708.5	1783618.2	1557115.8	1864384.0	1843823.2	1795227.7	1741944.4	1824838.5	1649931.1	2169700.4	2242441.6	2213802.2	2124864.1
Q8K2B3	Succinate dehydrogenase [ubiquinone] flavoprotein subunit, mitochondrial	Sdha	664	72.5	7.37	1065873.0	1124628.8	1179081.6	1167985.8	1223107.9	1151944.5	1144927.8	1128746.9	1090404.7	1126977.5	1069183.0	1076505.7	1204584.3	1162545.9
Q61937	Nucleophosmin	Npm1	292	32.5	4.77	1588800.4	1609741.9	1894388.3	1732731.1	1929361.9	1890539.2	1760108.2	1689393.7	1931827.9	1732058.9	3631548.1	3204530.3	4273062.2	3614252.0
O08749	Dihydrolipoyl dehydrogenase, mitochondrial	Dld	509	54.2	7.9	1778646.1	1792563.3	1879842.6	1873657.5	1834714.2	1747699.7	1814413.8	1715356.3	1854365.7	1893284.4	1153775.1	1386955.2	1173241.1	1294188.3
Q9JHR7	Insulin-degrading enzyme	Ide	1019	117.7	6.54	1040297.9	1062657.8	906938.8	1029195.7	916024.9	925062.6	1024418.3	931200.7	968382.7	1058026.2	884774.9	801038.2	827368.6	828296.1
E9Q6R7	Utrophin	Utrn	3430	392.5	5.33	679047.6	683940.1	558399.2	618058.6	673572.0	689723.7	698469.8	589223.7	673264.7	698564.0	222298.6	224270.1	199240.8	211368.3
P24547	Inosine-5'-monophosphate dehydrogenase 2	Impdh2	514	55.8	7.28	1346121.3	1304104.5	1181271.3	1227067.4	1159656.7	1030559.8	980945.6	1222915.8	1198283.9	1239995.0	630034.6	658704.0	685584.2	617179.4
Q9JMA1	Ubiquitin carboxyl-terminal hydrolase 14	Usp14	493	56	5.24	371355.5	430113.2	338225.7	436773.5	370640.1	378836.0	385121.1	432448.3	394596.7	431271.2	732207.1	734753.3	650110.8	695170.0
Q569Z5	Probable ATP-dependent RNA helicase DDX46	Ddx46	1032	117.4	9.26	842776.8	899896.4	834560.3	915610.3	936487.1	887706.6	827659.3	790055.9	887425.0	918587.5	927645.7	874080.9	928539.7	886665.4
Q9D819	Inorganic pyrophosphatase	Ppa1	289	32.6	5.6	1559901.5	1687626.5	1922485.2	1573170.7	2088614.1	1857486.4	1768063.6	1725722.7	1908409.3	1937191.8	1218956.3	1286347.7	1451412.9	1235811.7
Q9DBF1	Alpha-aminoadipic semialdehyde dehydrogenase	Aldh7a1	539	58.8	7.47	1198613.3	1313293.1	1126281.8	1418896.6	1054570.9	1088139.3	1311697.5	1486392.2	1359723.3	1183036.3	960137.1	925008.2	858249.7	1173879.9
O35098	Dihydropyrimidinase-related protein 4	Dpysl4	572	61.9	6.98	633595.8	572587.0	561483.2	542579.3	603177.6	579334.9	612889.9	568811.3	564134.9	590385.2	560193.4	538371.0	540067.1	520544.8
Q3UJB9	Enhancer of mRNA-decapping protein 4	Edc4	1406	152.4	5.78	529316.0	589356.7	509753.4	624078.8	533855.0	514314.0	468173.0	475910.5	567349.9	590370.6	480135.5	532703.0	442772.3	502243.3
Q6A068	Cell division cycle 5-like protein	Cdc5l	802	92.1	8.02	551422.4	558162.8	567235.1	584984.0	583407.4	554776.4	557287.5	523591.8	543909.6	568266.2	600838.7	650581.5	592522.4	658018.7
Q62095	ATP-dependent RNA helicase DDX3Y	Ddx3y	658	73.4	7.53	115732.6	124297.5	119236.5	130878.9	113163.3	120093.9	115902.7	105604.6	134419.1	123328.9	109759.0	99747.5	110411.7	101549.4
P47791	Glutathione reductase, mitochondrial	Gsr	500	53.6	7.99	472318.9	461349.1	495414.7	426779.4	547643.4	551332.6	532091.1	487439.6	529688.7	526920.9	833709.2	830134.3	850170.2	806113.1
Q921H8	3-ketoacyl-CoA thiolase A, peroxisomal	Acaa1a	424	43.9	8.44	616382.5	653916.1	571604.1	598875.6	585473.4	608463.5	637133.3	637620.9	618579.3	579215.9	447651.1	414625.6	447592.8	398695.2
P11881	Inositol 1,4,5-trisphosphate receptor type 1	Itpr1	2749	313	6.04	717149.9	685123.5	657541.3	663066.5	704611.8	722634.3	714946.0	719233.4	654505.5	664289.3	702636.5	699248.7	642081.6	682527.6
P49717	DNA replication licensing factor MCM4	Mcm4	862	96.7	7.2	583650.3	654382.5	596013.6	606729.2	670719.8	717362.1	573850.4	572928.9	628870.0	662986.2	873612.9	882091.9	907419.2	796505.9
P68040	Receptor of activated protein C kinase 1	Rack1	317	35.1	7.69	3841079.3	3646193.4	3709292.9	3638415.4	3907226.6	3530148.8	3372522.7	3793775.3	3808205.3	3701531.4	3866556.3	3968873.6	3814143.3	3853824.7
Q6P4S8	Integrator complex subunit 1	Ints1	2195	245	6.28	165395.9	169330.7	175382.7	173185.0	189242.3	183135.9	167311.6	158177.6	181336.2	182253.6	222733.6	269845.0	238195.3	217226.1
Q80U72	Protein scribble homolog	Scrib	1612	174	5.12	449949.4	463728.4	468527.1	458119.3	485665.8	477892.5	439121.2	440690.9	463924.4	478488.0	113156.5	167692.7	119743.9	135711.5
Q7TT50	Serine/threonine-protein kinase MRCK beta	Cdc42bpb	1713	194.6	6.46	818545.3	765874.9	747548.1	792554.4	768411.0	741788.5	787805.4	719566.4	764767.1	780473.4	807355.1	818429.9	740141.2	886693.8
P62196	26S proteasome regulatory subunit 8	Psmc5	406	45.6	7.55	1108088.0	1316362.1	1130433.8	1259217.2	1219399.8	1202955.3	973338.8	1037803.6	1185445.8	1222623.2	697781.2	530798.2	671870.5	693572.4
P24527	Leukotriene A-4 hydrolase	Lta4h	611	69	6.42	1084722.4	1099481.4	1130837.1	1141791.1	1098366.8	1128618.8	1398584.6	1068109.0	1137145.3	1081562.3	1311698.8	1256764.4	1234609.5	1203317.4
Q8R429	Sarcoplasmic/endoplasmic reticulum calcium ATPase 1	Atp2a1	994	109.4	5.22	37050.5	43967.7	38440.2	44304.1	37064.7	41603.9	34309.3	127460.9	42582.7	40006.6	176957.6	186095.3	166704.6	179349.3
Q9JIF0	Protein arginine N-methyltransferase 1	Prmt1	371	42.4	5.43	1624191.1	1577554.1	1712420.8	1589449.0	1679641.0	1535793.8	1464869.7	1614884.9	1588379.9	1659984.1	1826905.1	1658914.3	1904062.0	1618571.4
Q99K85	Phosphoserine aminotransferase	Psat1	370	40.4	8.03	2920880.4	2497793.4	2286176.9	2472922.1	2329923.0	2311006.3	2095141.4	2628018.8	2404488.2	2404823.3	2541487.5	2388066.9	2134437.5	2276784.6
Q9R0L6	Pericentriolar material 1 protein	Pcm1	2025	228.7	5.01	863933.0	1163145.5	1074359.4	1218237.1	917450.4	792029.6	739244.1	735456.5	1049992.6	1156359.8	715639.8	835557.5	825225.2	874346.9
Q9D8N0	Elongation factor 1-gamma	Eef1g	437	50	6.74	4013602.9	3957013.8	4096573.7	4065868.0	3828772.7	3657549.4	3237617.8	3807262.0	3679307.2	3706985.4	4569078.1	4773697.6	4909440.5	4865574.4
Q6DFZ1	SEC7 domain-containing protein	Gbf1	1861	206.7	5.8	426052.3	450705.7	426234.8	454707.4	427778.6	422109.1	366496.4	387464.0	427734.8	452337.3	278113.0	399402.9	338218.5	396658.8
Q9CYG7	Mitochondrial import receptor subunit TOM34	Tomm34	309	34.3	9.14	1151236.2	1231781.5	1356679.1	1269244.9	1219118.1	1015155.7	872346.2	1251995.7	1203676.2	1118280.8	878517.9	932021.5	910773.0	968996.9
Q9Z2I9	Succinate--CoA ligase [ADP-forming] subunit beta, mitochondrial	Sucla2	463	50.1	7.01	2532907.5	2432940.7	2624166.8	2840243.4	2287658.5	2155725.8	2243455.2	2366339.4	2444597.8	2315480.7	1636921.4	1619333.8	1666548.5	1857440.6
Q9Z1Z0	General vesicular transport factor p115	Uso1	959	106.9	4.93	1168268.6	1325546.5	1155446.6	1272194.8	1210204.9	1279077.9	1050859.4	1041005.0	1203220.9	1142122.6	626515.5	712312.5	658116.9	734764.5
Q7TMB8	Cytoplasmic FMR1-interacting protein 1	Cyfip1	1253	145.1	6.9	499338.9	471644.1	422781.9	459567.4	440366.7	452914.8	438380.8	428052.0	440028.6	459829.2	294517.5	311022.3	300652.6	293791.3
B1AY13	Ubiquitin carboxyl-terminal hydrolase 24	Usp24	2617	293.8	6.19	489832.0	516438.1	527571.4	520620.4	510723.1	503227.8	452340.0	459223.2	519148.0	507706.6	413374.2	472102.9	452532.0	447647.6
O55029	Coatomer subunit beta'	Copb2	905	102.4	5.3	1707926.0	1709110.5	1663602.8	1744739.6	1572388.2	1495794.6	1496571.9	1631618.3	1644258.5	1648538.8	1151615.2	1199660.9	1095031.2	1150933.4
Q99P88	Nuclear pore complex protein Nup155	Nup155	1391	155	6.15	724896.5	632785.4	639816.1	612335.9	657943.7	639478.3	667891.8	608219.4	636748.6	662858.5	489878.7	479119.1	533112.7	469884.0
Q9JLJ2	4-trimethylaminobutyraldehyde dehydrogenase	Aldh9a1	494	53.5	6.98	1620147.4	1604724.9	1438885.3	1515976.0	1506199.0	1579979.7	1526632.4	1457494.8	1624491.4	1518625.4	1620554.2	1659622.1	1683198.3	1654670.2
Q8VDP4	Cell cycle and apoptosis regulator protein 2	Ccar2	922	102.9	5.25	707088.5	815199.8	818255.8	813224.3	715294.2	685180.1	687597.0	618547.8	745675.1	804119.4	392613.3	430718.2	453342.5	470859.7
E9QA15	Caldesmon 1	Cald1	768	89.2	5.5	591440.0	533902.2	612689.9	532390.4	642199.7	697801.0	842797.9	677474.0	669854.5	569300.0	733912.8	654995.8	747523.0	609748.7
Q61838	Pregnancy zone protein	Pzp	1495	165.7	6.68	894446.8	795289.4	559540.8	742145.6	465556.2	523573.1	680708.0	755155.6	610429.8	662139.1	951462.2	774749.4	598976.6	782795.6
P61222	ATP-binding cassette sub-family E member 1	Abce1	599	67.3	8.34	651672.0	701723.1	683245.6	633579.8	678055.4	707737.9	647945.2	642476.4	664766.6	687368.1	550503.1	583248.4	632465.7	579199.1
Q11011	Puromycin-sensitive aminopeptidase	Npepps	920	103.3	5.9	1571144.6	1509572.7	1439682.5	1468599.1	1523717.9	1495757.7	1582108.4	1462391.5	1530930.0	1515291.0	1585370.4	1534355.9	1400638.5	1507582.9
Q61033	Lamina-associated polypeptide 2, isoforms alpha/zeta	Tmpo	693	75.1	8.05	357693.8	338200.1	383585.8	317390.6	422562.0	445553.5	451277.2	399870.3	375656.7	379194.7	607279.4	604277.5	632643.4	567815.9
Q91W50	Cold shock domain-containing protein E1	Csde1	798	88.7	6.37	1234425.9	1246445.7	1233407.1	1292723.8	1211364.3	1054871.3	945117.6	1179472.0	1139768.7	1208083.8	623963.5	696221.7	609006.3	641530.4
Q9Z0U1	Tight junction protein ZO-2	Tjp2	1167	131.2	6.79	478136.2	418878.4	473724.8	398700.3	524711.0	519551.5	535156.6	459074.2	487385.0	453429.9	272181.4	248990.7	279469.8	234651.7
P58771	Tropomyosin alpha-1 chain	Tpm1	284	32.7	4.74	3949321.8	3240727.1	3947940.1	3198800.0	3495480.5	3976026.9	4696501.2	5866476.6	3605609.6	3289737.7	1294311.3	1286971.3	1395074.1	1356138.0
Q80TP3	E3 ubiquitin-protein ligase UBR5	Ubr5	2792	308.2	5.85	499716.7	559848.0	543253.8	540362.1	548341.2	512544.0	510523.2	475743.6	510878.0	537934.9	190250.9	208245.2	210377.5	199117.6
P17751	Triosephosphate isomerase	Tpi1	299	32.2	5.74	2511660.7	2030565.8	1847527.3	2163530.0	1981981.4	2501422.6	3129332.0	2805895.4	2153407.4	2295046.2	1806920.5	1377191.8	1616160.5	1682765.7
Q9Z204	Heterogeneous nuclear ribonucleoproteins C1/C2	Hnrnpc	313	34.4	5.05	1973811.1	1987264.9	1912077.5	1965551.1	2040844.1	1927073.2	1931469.5	1773767.0	2004941.6	2046280.4	1247989.6	1199615.9	1411499.4	1229096.4
Q8C878	NEDD8-activating enzyme E1 catalytic subunit	Uba3	462	51.7	5.45	974386.4	1040788.8	1142460.3	1129406.3	1037414.9	999841.0	1020425.5	1022291.2	1102338.2	1088833.6	448726.4	475723.9	450162.5	481057.8
P48962	ADP/ATP translocase 1	Slc25a4	298	32.9	9.72	3079331.1	2847526.5	2860955.5	2927617.1	2682660.4	2639019.2	3015559.0	3457682.8	2712643.8	2429250.6	2479397.0	2597353.6	2261152.3	2636474.4
Q8BMF4	Dihydrolipoyllysine-residue acetyltransferase component of pyruvate dehydrogenase complex, mitochondrial	Dlat	642	67.9	8.57	1128598.6	1288052.9	1279644.9	1447724.0	1031368.4	1009389.6	961662.1	1008566.3	1086728.7	1205003.5	1238276.6	1313032.9	1255865.9	1423437.3
P55096	ATP-binding cassette sub-family D member 3	Abcd3	659	75.4	9.26	817922.7	753198.7	715202.3	746609.5	740369.2	688992.3	685640.1	760631.5	752730.4	726006.6	1192299.5	1245290.7	1027200.6	1164851.6
O35841	Apoptosis inhibitor 5	Api5	504	56.8	5.92	951518.9	963744.1	1075587.1	1037479.1	1059281.8	929892.3	961927.6	873102.9	1030790.8	1036224.7	920649.9	981496.5	1085658.3	1069422.4
Q3UDE2	Tubulin--tyrosine ligase-like protein 12	Ttll12	639	74	5.63	520856.6	497407.1	492326.7	463226.0	530313.2	534426.2	518192.0	486354.0	478475.7	477893.7	707230.1	700526.9	670783.2	697642.5
Q60932	Voltage-dependent anion-selective channel protein 1	Vdac1	296	32.3	8.43	1939997.2	1740704.0	1857917.8	1860191.3	1796851.4	1757912.1	1929653.6	2067546.6	1629929.6	1561689.1	1735747.0	1811658.5	1522020.5	1843029.6
Q6NS46	Protein RRP5 homolog	Pdcd11	1862	207.6	8.75	590575.6	562083.9	590291.0	551624.8	645268.0	600019.8	562137.1	566244.3	590715.8	595576.3	307578.6	304637.4	319676.5	280830.0
Q9D0K2	Succinyl-CoA:3-ketoacid coenzyme A transferase 1, mitochondrial	Oxct1	520	56	8.53	730121.6	695897.6	692832.3	665314.0	791468.5	793306.5	707451.5	798114.3	719155.6	742830.0	952872.6	869038.6	875162.8	818896.4
Q9CQN1	Heat shock protein 75 kDa, mitochondrial	Trap1	706	80.2	6.68	896088.6	896097.9	852658.7	867200.3	767277.3	785015.2	729725.5	806197.9	823810.3	873101.9	572574.2	609110.0	556128.0	608783.0
P54869	Hydroxymethylglutaryl-CoA synthase, mitochondrial	Hmgcs2	508	56.8	8.41	2558604.6	2392782.9	1728406.0	2643456.3	1897078.8	1710684.7	2057826.3	2077480.8	1898830.9	1941620.2	2576143.4	2311424.4	1777545.2	2604867.2
P05202	Aspartate aminotransferase, mitochondrial	Got2	430	47.4	9	2144347.1	1841244.3	2097144.3	2144710.9	2106571.5	2163091.1	2507600.0	2625531.7	2059727.3	1946707.5	2340730.4	1920014.5	2082105.2	2183817.3
Q9D0R2	Threonine--tRNA ligase 1, cytoplasmic	Tars1	722	83.3	7.36	703494.7	730820.2	721260.6	706341.4	717997.4	691878.0	650322.0	716171.3	703366.8	735679.9	502422.9	528361.8	502685.0	506172.7
P62192	26S proteasome regulatory subunit 4	Psmc1	440	49.2	6.21	1540196.4	1694622.9	1668148.1	1716843.4	1635961.2	1483208.4	1457762.1	1438014.6	1631681.7	1694937.6	1049035.8	1074226.3	1144790.2	1197059.1
Q8BMA6	Signal recognition particle subunit SRP68	Srp68	625	70.5	8.57	417481.3	421378.9	457113.8	423010.8	447453.7	458577.1	438202.6	424939.7	421289.5	462972.6	302970.7	296180.7	273782.6	293414.9
P46935	E3 ubiquitin-protein ligase NEDD4	Nedd4	887	102.6	5.26	1376905.6	1202388.6	1243127.9	1150938.5	1249217.5	1305034.3	1459904.9	1400345.1	1224999.5	1188766.1	1261953.4	1004063.4	1141377.9	1014207.3
O55131	Septin-7	Septin7	436	50.5	8.57	619077.8	583503.1	554900.9	570863.8	614081.2	639607.9	558915.3	584464.5	565960.3	574786.0	253046.8	241649.9	222906.9	232478.1
P18654	Ribosomal protein S6 kinase alpha-3	Rps6ka3	740	83.6	6.89	677099.8	674480.6	587207.2	601303.2	648603.6	650187.2	661377.9	636315.1	658103.9	660182.5	992887.8	915901.5	906937.1	878074.9
P28660	Nck-associated protein 1	Nckap1	1128	128.7	6.62	625872.8	618884.2	602566.4	602590.5	651197.5	659158.3	689146.3	660954.2	631970.1	630768.8	534253.3	458756.6	437719.3	424843.3
Q8C7X2	ER membrane protein complex subunit 1	Emc1	997	111.5	7.43	537096.3	552483.9	555624.5	544289.7	561245.8	552079.8	502048.3	516280.7	563987.0	563838.9	351543.3	389461.4	402510.7	381559.3
P21619	Lamin-B2	Lmnb2	596	67.3	5.5	265525.8	253333.7	253877.4	234491.6	304524.8	296763.0	347745.8	250854.9	268382.9	269032.3	484353.5	448910.5	475832.9	482395.5
Q91VC3	Eukaryotic initiation factor 4A-III	Eif4a3	411	46.8	6.73	1674211.5	1608075.0	1736661.9	1651516.3	1749640.8	1633186.4	1603322.1	1599957.6	1645845.6	1736633.8	1848418.5	1661303.9	1766601.7	1764393.5
P51881	ADP/ATP translocase 2	Slc25a5	298	32.9	9.73	5166442.0	5036077.7	4381831.0	5173627.6	3837298.3	3376558.3	4277309.6	5056979.0	4502924.9	4401689.2	7172004.8	5754722.3	5426591.9	6086754.1
P97311	DNA replication licensing factor MCM6	Mcm6	821	92.8	5.45	660938.2	691274.0	703109.1	642282.9	791598.2	756072.2	650527.4	668374.3	696118.5	704709.1	617067.1	697834.2	678628.6	597262.8
Q922B2	Aspartate--tRNA ligase, cytoplasmic	Dars1	501	57.1	6.49	1750644.8	1887457.8	1894484.4	1958233.1	1782991.9	1787669.4	1630281.5	1877991.3	1887885.0	1926111.4	2161497.8	2301448.8	2366984.5	2283103.5

E9Q3L2	Phosphatidylinositol 4-kinase alpha	Pi4ka	2105	236.9	7.06	443191.5	456347.1	439637.1	398114.2	468974.8	470810.1	458546.1	443140.2	428614.4	421194.7	307824.0	288766.1	336005.6	280623.6
Q9QZE5	Coatomer subunit gamma-1	Copg1	874	97.5	5.35	362848.1	382018.3	323812.3	369574.2	343141.2	348615.4	318441.5	352860.3	354533.6	369951.2	300044.3	315591.0	281874.9	305329.1
O88342	WD repeat-containing protein 1	Wdr1	606	66.4	6.6	958008.4	858261.9	929841.2	936849.4	927593.1	963819.7	990186.4	947069.3	952544.3	921337.7	671482.8	621461.7	636662.3	580084.9
Q9WVK4	EH domain-containing protein 1	Ehd1	534	60.6	6.83	878542.7	968928.2	813800.4	896259.7	903746.6	1009140.6	956461.9	932653.0	899699.9	909427.1	654680.4	678520.6	589597.2	650518.7
Q8CIB5	Fermitin family homolog 2	Fermt2	680	77.8	6.7	324321.1	365608.1	349643.4	349161.4	380438.5	404951.6	456806.6	374926.0	369559.4	354588.4	359956.8	326369.3	346977.5	317040.7
Q9DBJ1	Phosphoglycerate mutase 1	Pgam1	254	28.8	7.18	2430890.0	2251239.6	2393581.3	2369498.6	2435884.0	2421886.9	2483724.5	2595460.6	2307949.3	2293532.0	1418740.0	1413740.5	1512458.0	1335222.9
Q9QXK3	Coatomer subunit gamma-2	Copg2	871	97.6	5.8	743159.3	776510.1	744411.2	803635.8	713939.3	690510.0	697866.8	662654.8	754610.4	765789.2	492318.8	656667.3	525195.3	578978.4
P59328	WD repeat and HMG-box DNA-binding protein 1	Wdhd1	1117	124.2	5.63	361883.1	368895.3	400273.5	363902.2	488890.7	464781.2	395619.7	382339.9	395775.8	393379.6	291988.8	304722.2	314454.4	296214.5
O35660	Glutathione S-transferase Mu 6	Gstm6	218	25.6	6.24	3883006.8	3336100.2	3708777.6	3443709.1	4183831.4	3572106.8	4451610.1	3852038.2	4209320.8	3977289.8	5256795.3	4135494.8	5220948.7	4536952.4
P50396	Rab GDP dissociation inhibitor alpha	Gdi1	447	50.5	5.08	220159.7	200272.1	205233.6	210853.2	230976.7	226619.8	213546.2	203329.8	211804.5	209370.6	736831.6	702561.2	666797.2	679162.7
Q6PFD9	Nuclear pore complex protein Nup98-Nup96	Nup98	1816	197.1	6.18	532435.8	590635.6	567109.4	560502.7	637246.9	608743.5	592653.8	516763.1	604828.7	580497.8	656699.3	671939.2	663240.7	664662.9
Q9ESZ8	General transcription factor II-I	Gtf2i	998	112.2	6.55	500909.1	444546.9	395339.3	469216.8	467694.1	441368.2	458888.8	450054.6	438297.9	487289.7	858206.8	841957.4	817027.1	819141.8
Q80UM3	N-alpha-acetyltransferase 15, NatA auxiliary subunit	Naa15	865	100.9	7.62	955705.1	1018800.2	977581.8	986385.2	988416.2	925531.1	818249.9	915189.5	964507.6	1035276.8	1509302.5	1646630.9	1658685.6	1675067.7
P16381	Putative ATP-dependent RNA helicase Pl10	D1Pas1	660	73.1	7.18	678732.6	846871.5	849953.3	966205.8	583365.1	512723.9	447762.1	576424.9	734312.8	810683.5	180090.6	195805.9	199921.2	222169.1
Q6Q477	Plasma membrane calcium-transporting ATPase 4	Atp2b4	1205	133	6.13	470195.1	493324.6	495328.7	520027.8	494150.5	543888.4	611698.4	457216.2	501895.2	517089.8	172144.6	195510.1	180131.8	200341.6
P82198	Transforming growth factor-beta-induced protein ig-h3	Tgfbi	683	74.6	7.06	797225.9	671587.7	721749.2	634734.7	1084656.2	1404095.3	1312295.7	864373.4	801776.4	819390.1	699516.1	665030.2	681389.7	655782.1
Q3UYV9	Nuclear cap-binding protein subunit 1	Ncbp1	790	91.9	6.64	554651.4	538543.3	530132.8	603087.9	513201.7	508948.9	503413.1	477087.3	568613.3	614050.5	384900.1	438730.2	373587.2	398093.5
P06728	Apolipoprotein A-IV	Apoa4	395	45	5.47	1280296.3	1163833.2	1154460.4	1004569.6	904937.3	1211061.0	1096098.6	1416217.5	1116780.0	1387826.8	1413064.4	1304311.5	1316434.0	1153610.0
Q9CZ13	Cytochrome b-c1 complex subunit 1, mitochondrial	Uqcrc1	480	52.8	6.21	1308668.6	1237598.9	1452255.0	1346078.9	1402387.7	1287713.7	1239971.5	1346342.8	1368430.9	1259263.9	1511373.9	1565502.2	1590980.0	1687003.8
G3X9B1	HEAT repeat-containing 1	Heatr1	2143	241.9	6.98	530626.2	547581.7	525034.7	518597.5	550855.6	539637.3	497956.8	502238.1	550463.2	544708.6	495020.8	503042.1	491406.8	488521.8
Q93092	Transaldolase	Taldo1	337	37.4	7.03	2053686.2	2052577.6	2167753.5	1927301.8	2539479.9	2439436.5	2650222.7	2156397.0	2219330.6	2254721.9	2850321.0	3217477.0	2778900.1	2516873.0
Q9D8W5	26S proteasome non-ATPase regulatory subunit 12	Psmd12	456	52.9	7.06	868578.3	962626.8	815750.7	925487.5	837971.8	765160.2	769179.9	812306.6	896779.3	941297.9	572491.7	605470.3	536038.5	584767.6
P08752	Guanine nucleotide-binding protein G(i) subunit alpha-2	Gnai2	355	40.5	5.45	1060279.3	1158558.9	1033523.7	1156109.0	1175409.3	1160908.6	1430370.2	1035272.7	1265966.9	1325575.9	783629.3	696128.5	705232.7	737719.9
P67778	Prohibitin	Phb	272	29.8	5.76	1686992.7	1527994.9	1701801.2	1682798.3	1807800.5	1685934.5	1730853.1	1727843.6	1675255.4	1491159.1	1753125.4	1664442.0	1800021.0	1769817.5
Q3UEB3	Poly(U)-binding-splicing factor PUF60	Puf60	564	60.2	5.29	982008.3	939739.9	951524.4	950746.7	963250.7	910977.8	940140.4	883376.7	961681.7	997058.3	627093.0	604992.5	592184.7	632777.5
Q9WTX6	Cullin-1	Cul1	776	89.6	8	716625.4	749831.6	806840.0	735610.8	830280.5	806760.7	644428.2	722342.0	764512.8	766809.9	615717.2	700133.6	617235.0	663075.0
P17918	Proliferating cell nuclear antigen	Pcna	261	28.8	4.77	1896584.8	2288024.8	2492813.8	2054344.8	2714793.9	2488984.7	1900927.7	2041726.6	2519710.6	2490744.2	2240492.3	2316696.2	2672424.7	2259516.1
Q9Z1N5	Spliceosome RNA helicase Ddx39b	Ddx39b	428	49	5.67	2813745.4	2901526.2	2970611.6	2890029.5	3227066.8	2965722.2	2910295.8	2830420.5	3163773.7	3242314.0	2091335.6	2006748.4	2651512.4	2225624.8
P17742	Peptidyl-prolyl cis-trans isomerase A	Ppia	164	18	7.9	5950221.6	5976208.1	6394093.7	5890021.7	5539178.8	5449482.7	5208214.9	5658030.4	5839168.2	6173467.6	5949023.8	6189734.4	6032933.1	6250497.1
Q810A7	ATP-dependent RNA helicase DDX42	Ddx42	929	101.9	6.98	532389.4	682335.7	557192.2	654245.7	550154.1	548854.0	552920.2	471942.8	629754.6	734046.7	315997.4	362457.2	356881.8	360070.3
Q3ULD5	Methylcrotonoyl-CoA carboxylase beta chain, mitochondrial	Mccc2	563	61.3	8	525427.0	520552.2	467940.1	531255.2	517456.6	529782.2	530046.5	513013.3	510807.3	502802.1	389469.7	336883.6	369293.6	368907.1
Q8BG05	Heterogeneous nuclear ribonucleoprotein A3	Hnrnpa3	379	39.6	9.01	1606819.8	1619029.9	1869150.2	1633846.6	1753171.4	1810145.2	1806119.7	1646522.1	1692148.1	1754317.1	1134030.8	1071003.2	1341228.4	1144567.7
Q8BHG1	Nardilysin	Nrdc	1161	132.8	4.87	872454.6	890267.2	819848.8	862008.5	760201.5	795704.6	707342.2	792531.9	863497.4	824765.5	849506.8	759879.6	743033.1	746216.9
Q9WUB3	Glycogen phosphorylase, muscle form	Pygm	842	97.2	7.11	402122.0	395699.0	370916.4	439544.8	338516.3	382132.4	375412.9	475359.6	387144.6	390388.2	388531.4	357261.2	311183.8	371363.9
Q8CFQ3	RNA helicase aquarius	Aqr	1481	170.2	6.27	266265.8	285167.8	263807.4	267030.0	314712.6	277265.6	280982.7	264279.6	293843.2	293747.9	320703.0	320616.6	312860.2	333766.2
Q80X82	Symplekin	Sympk	1288	142.5	6.05	510522.4	593597.4	532655.5	532083.8	570424.0	555015.4	517447.3	502162.7	555330.7	567089.9	244505.4	293123.8	288704.8	290375.7
A6H5Y3	Methionine synthase	Mtr	1253	139	5.57	522257.7	569094.3	585371.7	600960.4	559862.0	564044.1	599461.3	539416.1	560280.8	607797.3	559408.7	599920.3	546766.7	573018.3
Q8BG32	26S proteasome non-ATPase regulatory subunit 11	Psmd11	422	47.4	6.48	841176.6	924839.5	855252.5	902850.7	879229.0	873236.6	830797.0	808689.5	833343.5	863949.5	1550059.2	1780962.3	1863058.9	1885098.3
P62814	V-type proton ATPase subunit B, brain isoform	Atp6v1b2	511	56.5	5.81	366352.5	397190.4	390691.2	418134.3	397435.7	392059.2	374092.8	368569.1	384218.3	392771.4	401342.3	425658.8	385156.4	400506.1
Q99LF4	RNA-splicing ligase RtcB homolog	Rtcb	505	55.2	7.23	850228.5	862243.6	880585.1	868971.1	837620.4	873561.5	822177.9	853185.0	844067.7	868553.9	1096676.1	920061.1	1078549.6	1017160.6
Q7TNP2	Serine/threonine-protein phosphatase 2A 65 kDa regulatory subunit A beta isoform	Ppp2r1b	601	65.9	5.1	339340.3	347134.7	393323.0	363893.2	379528.9	366538.3	334880.3	358169.9	416359.1	386122.9	299119.7	367278.2	357117.3	392501.7
Q8VIJ6	Splicing factor, proline- and glutamine-rich	Sfpq	699	75.4	9.44	1265018.2	1230541.9	1267763.4	1233081.4	1465275.8	1462990.8	1376131.5	1241996.2	1292671.3	1283930.7	1986951.2	1723979.7	2129288.4	1755458.7
Q6WKZ8	E3 ubiquitin-protein ligase UBR2	Ubr2	1755	199	6.33	359585.1	396358.5	390575.2	367109.5	420193.1	412585.7	351844.8	380694.5	375869.2	396567.8	299623.9	350592.1	306297.3	308743.5
Q91X72	Hemopexin	Hpx	460	51.3	7.8	3691496.3	3949405.7	3229074.7	3201851.1	1973948.6	2433327.1	3272381.1	3644753.7	3991539.9	3955206.3	3756012.5	3920029.3	3033649.3	2808396.7
O35969	Guanidinoacetate N-methyltransferase	Gamt	236	26.3	5.71	1077365.1	981726.6	1071959.6	984799.2	1139931.8	1037720.8	1150160.8	1123464.3	1137518.0	1079374.4	1521292.6	1464375.1	1616501.6	1590294.9
Q8BWY3	Eukaryotic peptide chain release factor subunit 1	Etf1	437	49	5.71	453095.0	431130.7	417790.3	404765.1	483143.8	540149.9	472394.0	448799.7	437850.2	427815.3	1052702.2	1116196.4	1122612.8	1137682.5
E9Q557	Desmoplakin	Dsp	2883	332.7	6.8	266933.4	311840.9	255590.0	284181.1	492299.0	381188.2	298636.3	306934.1	306390.3	307853.6	305775.3	364139.4	332518.1	376684.1
P55937	Golgin subfamily A member 3	Golga3	1487	167.1	5.4	150750.8	181293.3	162010.6	176530.2	181024.0	169359.8	142034.1	156741.3	159087.8	164087.0	262345.5	308538.6	286048.9	299337.8
P12970	60S ribosomal protein L7a	Rpl7a	266	30	10.56	1810894.5	1859670.8	1759379.5	1778991.4	1527763.0	1431138.2	1356633.8	1535431.7	1564815.2	1662242.4	2200984.0	2361792.2	1969026.5	2115391.6
Q9CZD3	Glycine--tRNA ligase	Gars1	729	81.8	6.65	733700.8	792138.1	722968.2	787114.5	817519.2	740785.3	630275.5	648252.0	729697.3	800076.6	1075652.1	994731.9	1141432.1	1012232.4
Q02788	Collagen alpha-2(VI) chain	Col6a2	1034	110.3	6.42	2214686.2	2007530.4	1914838.0	1870800.7	2734225.2	3742360.6	4419421.3	3019097.8	2269370.7	2104407.9	2954257.7	2204867.5	2234714.6	2451124.1
P12382	ATP-dependent 6-phosphofructokinase, liver type	Pfkl	780	85.3	7.17	650293.3	689179.4	599862.4	666944.6	652561.2	638605.1	668332.8	613021.7	653963.8	658138.2	736502.6	694085.6	661612.6	725362.0
Q9EPL8	Importin-7	Ipo7	1038	119.4	4.82	471781.6	488434.5	486672.1	523340.6	493252.4	467798.6	450825.6	471840.5	507744.2	487322.9	610262.5	638184.5	623220.3	607685.1
Q9DCL9	Multifunctional protein ADE2	Paics	425	47	7.23	626246.7	837672.7	657652.1	646529.9	774238.5	743899.4	611747.0	707273.3	665186.2	749132.9	837330.2	804725.5	878084.0	836925.1
Q8BH64	EH domain-containing protein 2	Ehd2	543	61.1	6.51	1296910.7	1248983.0	1133939.1	1163160.4	1649242.9	1498772.7	1802953.3	1404300.7	1306915.6	1284105.2	905168.5	772783.9	889306.6	741909.3
Q61425	Hydroxyacyl-coenzyme A dehydrogenase, mitochondrial	Hadh	314	34.4	8.65	3735341.1	2758856.1	3471738.7	2800077.7	3526386.6	3324257.6	3324941.9	3606984.2	3096786.4	2923444.4	3998784.9	3326057.5	3379250.4	3331719.2
Q9Z2W0	Aspartyl aminopeptidase	Dnpep	473	52.2	7.25	614185.3	699617.6	606916.7	731801.9	593712.5	563622.7	530509.8	521486.9	646356.6	665038.9	459043.8	556838.5	450951.9	556861.3
P14685	26S proteasome non-ATPase regulatory subunit 3	Psmd3	530	60.7	8.44	723497.7	790629.8	757319.4	771240.2	740375.8	725013.2	682002.2	716363.1	733880.5	772124.2	1254263.6	1427382.3	1314594.7	1360946.2
Q9EPK7	Exportin-7	Xpo7	1087	123.7	6.38	1013632.2	1037651.0	1025402.2	1012263.7	1124554.0	1101125.3	1064026.5	937818.8	1049633.5	1076480.9	724654.8	748689.4	747304.5	718661.4
Q61578	NADPH:adrenodoxin oxidoreductase, mitochondrial	Fdxr	494	54.2	8.66	1739714.4	1532849.6	1414223.3	1632735.6	1295410.1	1454579.7	1341625.8	1754570.9	1658719.7	1507869.5	1011171.8	918822.8	753491.9	886386.4
Q9DBR1	5'-3' exoribonuclease 2	Xrn2	951	108.6	7.59	642748.6	636770.9	707220.1	650759.2	820700.6	756880.9	700920.9	722346.8	661440.0	663319.3	537633.1	517414.9	546782.4	531173.2
Q8QZY1	Eukaryotic translation initiation factor 3 subunit L	Eif3l	564	66.6	6.44	815979.9	873781.2	858852.7	862039.6	846801.3	790964.9	807201.3	801284.4	840634.1	870428.8	664009.3	634953.9	659807.6	668868.6
Q99PU8	ATP-dependent RNA helicase DHX30	Dhx30	1217	136.6	8.75	358433.1	429989.6	404695.0	408278.1	393801.4	386735.0	336541.4	354007.5	408178.9	438097.4	477937.8	499662.1	490192.0	527552.8
Q8BK67	Protein RCC2	Rcc2	520	55.9	8.72	701034.8	724551.7	707669.8	727920.4	808946.7	795311.8	698793.0	735382.4	731950.1	794254.4	1126460.4	1162250.9	1071153.5	1063544.2
P20918	Plasminogen	Plg	812	90.7	6.6	1014447.7	928506.0	820702.4	793995.8	651690.5	794993.4	948987.9	852453.6	801622.1	810549.9	702574.7	628692.4	565493.4	571222.9
B2RXS4	Plexin-B2	Plxnb2	1842	206.1	5.87	324969.0	341727.2	300847.0	316198.4	345087.4	366762.8	362416.1	312342.1	304120.1	346310.0	316360.8	306937.1	286211.9	275749.6
Q61781	Keratin, type I cytoskeletal 14	Krt14	484	52.8	5.17	315527.5	354792.5	315847.0	427236.5	696434.9	469503.7	385769.5	357968.7	456652.9	441657.8	388207.1	422800.6	400408.0	433984.8
P60670	Nuclear protein localization protein 4 homolog	Nploc4	608	68	6.46	349826.0	377863.4	381122.7	390334.7	401275.0	400898.3	394525.2	335949.9	382923.0	394306.9	475742.1	513646.2	524281.5	512965.3
P17710	Hexokinase-1	Hk1	974	108.2	6.8	300393.6	282418.0	276276.2	277206.1	281347.4	294425.4	305470.0	270683.8	276814.6	309612.3	521356.5	513078.9	505160.7	517244.4
P28658	Ataxin-10	Atxn10	475	53.7	5.25	494267.0	532002.8	487594.6	515163.3	589535.5	484814.6	484805.9	476264.5	521456.5	494250.6	1385799.9	1468230.1	1452572.1	1485494.7
Q99MR6	Serrate RNA effector molecule homolog	Srrt	875	100.4	5.97	451528.4	512013.7	454881.5	480242.8	476175.8	457103.8	454366.0	417771.4	470120.5	491235.8	472590.3	517318.4	440285.3	456269.1
Q8VCW8	Medium-chain acyl-CoA ligase ACSF2, mitochondrial	Acsf2	615	67.9	8.18	433511.8	449922.5	427331.4	457205.8	592672.4	419932.2	563461.0	399367.7	458785.2	458340.9	770704.3	772589.4	755669.9	771574.3
Q6DFW4	Nucleolar protein 58	Nop58	536	60.3	8.34	612620.6	571134.7	594653.6	593331.1	696605.4	692588.5	746535.2	617769.4	639755.3	636721.9	449535.9	466430.3	512315.4	438661.1
O88544	COP9 signalosome complex subunit 4	Cops4	406	46.3	5.83	581387.7	660216.8	650790.3	669038.5	643181.2	609830.9	542875.6	590279.3	615182.4	592758.7	684973.3	715538.6	715060.0	739528.7
Q921F2	TAR DNA-binding protein 43	Tardbp	414	44.5	6.7	591236.3	547123.7	690783.2	597580.7	636034.4	641213.5	613084.2	602324.7	622756.9	614330.3	421200.0	365778.9	508123.2	416450.9
P07758	Alpha-1-antitrypsin 1-1	Serpina1a	413	46	5.72	1205868.7	1419616.5	1043314.7	1317267.5	508765.6	509986.1	1325753.1	953487.5	1199745.2	512924.5	2919953.0	2321550.4	2250758.2	2809807.5
O35129	Prohibitin-2	Phb2	299	33.3	9.83	1425241.2	1387308.9	1485052.5	1312888.0	1523354.8	1451517.4	1445753.4	1523599.3	1402113.9	1367970.2	2320651.0	2262071.7	2295674.2	2260770.3
P23475	X-ray repair cross-complementing protein 6	Xrcc6	608	69.4	6.79	850143.5	841142.8	899056.7	824905.0	915905.6	955290.8	898254.4	854428.4	877853.6	840091.5	1063253.6	1046141.8	1161629.5	1101680.6
Q8BTI8	Serine/arginine repetitive matrix protein 2	Srrm2	2703	294.7	12.03	368454.6	421315.8	400375.3	384882.8	423305.7	400642.7	379397.2	361305.9	397656.2	405088.6	244843.8	272210.1	255330.9	271298.0
P06151	L-lactate dehydrogenase A chain	Ldha	332	36.5	7.74	1477648.5	1282161.9	1279396.3	1389051.4	1195327.7	1303679.4	1355340.6	1494407.6	1276445.4	1209298.7	1966416.7	2140969.1	1794951.2	2293527.5
P13707	Glycerol-3-phosphate dehydrogenase [NAD(+)], cytoplasmic	Gpd1	349	37.5	7.17	482090.9	468242.2	357399.5	499717.8	403385.5	350744.5	379146.7	417467.4	415245.5	414173.4	840621.5	906594.5	588278.0	891704.1
Q9Z2I8	Succinate--CoA ligase [GDP-forming] subunit beta, mitochondrial	Suclg2	433	46.8	7.02	909732.4	813305.6	814536.8	866338.6	910661.0	897808.3	919174.3	746205.8	861887.0	826532.9	974369.2	752670.5	907731.9	835915.9
Q91YT0	NADH dehydrogenase [ubiquinone] flavoprotein 1, mitochondrial	Ndufv1	464	50.8	8.21	954314.0	976961.7	992117.5	976254.2	1008414.9	913208.9	937370.9	971793.5	960249.1	931981.6	516434.5	551107.9	567430.5	583046.4
Q06890	Clusterin	Clu	448	51.6	5.67	837097.7	759874.5	777447.8	702085.7	789059.6	750118.0	736487.0	981198.9	904713.5	836149.1	1255978.7	1113723.7	1198580.1	981545.3
Q9Z277	Tyrosine-protein kinase BAZ1B	Baz1b	1479	170.5	8.6	513351.2	512220.8	545566.7	506194.8	542926.5	536032.6	472876.3	479695.8	489297.1	507966.1	477608.4	506540.8	493590.2	470169.3
Q7TPH6	E3 ubiquitin-protein ligase MYCBP2	Mycbp2	4749	520.9	7.09	201003.7	249190.3	251508.4	270919.1	261652.9	241534.8	220188.0	227462.0	252871.5	254920.1	212594.1	221761.8	232359.0	214731.1
Q99K01	Pyridoxal-dependent decarboxylase domain-containing protein 1	Pdxdc1	787	87.3	5.48	413038.7	454392.4	463453.3	472567.3	466603.0	432377.6	390269.7	423105.8	444279.5	456833.6	565891.7	638463.0	634583.9	667602.5
Q9JHI5	Isovaleryl-CoA dehydrogenase, mitochondrial	Ivd	424	46.3	8.29	1324876.4	1254491.7	1357481.7	1321115.5	1315615.3	1247182.2	1637190.0	1272173.7	1327390.0	1324664.0	965953.8	915098.3	948993.7	918947.0
P51150	Ras-related protein Rab-7a	Rab7a	207	23.5	6.7	815246.3	756679.2	801147.1	769528.0	766676.4	748948.2	747037.6	787563.1	770026.4	782041.0	791728.3	728118.5	730284.2	723130.0
P68510	14-3-3 protein eta	Ywhah	246	28.2	4.89	1149194.9	1282266.0	1178222.5	1290662.9	1263239.0	1265334.8	1329418.8	1243255.1	1411888.0	1266430.0	796159.9	869585.6	748618.2	811548.3
Q9Z315	U4/U6.U5 tri-snRNP-associated protein 1	Sart1	806	90.8	5.82	430174.1	439593.8	453345.0	442150.5	458629.5	455470.0	468853.6	413660.1	444474.0	460672.6	238046.0	262364.4	248267.2	241833.0
A0A286YCV9	Kinesin family member 13B	Kif13b	1847	204.8	5.8	965066.2	1004870.6	939681.3	956668.6	961638.9	952813.2	908507.4	948072.0	958181.8	1017577.9	393172.8	401279.2	412082.1	397994.7

P09470	Angiotensin-converting enzyme	Ace	1312	150.8	6.55	410944.9	391674.4	336828.0	373312.2	432851.5	417131.4	446948.6	390288.4	413953.0	377814.7	445193.0	436582.9	403814.6	441305.8
Q9JMH9	Unconventional myosin-XVIIIa	Myo18a	2050	232.6	6.28	486270.4	496671.9	461771.7	478768.9	509539.3	531851.2	465075.0	482219.8	464259.7	496721.3	200401.5	217376.7	182028.1	185503.4
Q07417	Short-chain specific acyl-CoA dehydrogenase, mitochondrial	Acads	412	44.9	8.47	530074.8	492578.0	499960.7	503735.6	515754.1	535433.7	580076.0	529146.0	500599.4	485180.6	480092.1	473625.8	428902.0	467429.3
P24270	Catalase	Cat	527	59.8	7.88	281739.0	289428.7	290682.5	279939.3	329536.4	285582.2	299036.2	289833.4	301071.1	301918.7	258000.9	259314.8	242842.0	244357.1
Q00623	Apolipoprotein A-I	Apoa1	264	30.6	5.73	4816882.0	4718665.8	4498780.1	4249554.1	3679081.3	4194477.4	4116932.6	5216624.2	4427936.3	4451301.3	4240644.0	4101398.6	4063681.9	3560999.3
Q9CZ30	Obg-like ATPase 1	Ola1	396	44.7	7.81	1026457.8	1171178.2	1218072.3	1215548.4	1088971.8	957012.0	886120.7	1027582.5	1150986.3	1163445.0	1313349.6	1169074.9	1073720.5	1143512.8
Q6ZWX6	Eukaryotic translation initiation factor 2 subunit 1	Eif2s1	315	36.1	5.08	1514198.2	1707723.9	1695081.1	1612049.5	1629303.2	1529123.0	1414177.4	1472853.4	1703494.7	1819682.3	1764575.4	1721528.1	1825568.9	1728789.5
P99029	Peroxiredoxin-5, mitochondrial	Prdx5	210	21.9	8.85	2227715.8	1946879.9	2136724.1	2105163.8	2144330.4	2165507.6	2317440.6	2382605.5	2171940.2	2091504.3	2616317.9	2321211.1	2566201.1	2526604.9
P15864	Histone H1.2	H1-2	212	21.3	11	365690.7	383506.4	357170.5	326591.5	382989.2	426681.3	465713.6	325635.4	388116.6	439779.2	1838208.8	1586169.8	2263048.1	1463462.0
Q62351	Transferrin receptor protein 1	Tfrc	763	85.7	6.57	782585.7	830898.3	906192.0	771477.1	921848.9	886092.4	629593.1	762492.1	911978.8	974596.2	728192.4	756725.3	805506.9	715993.3
Q99MV7	RING finger protein 17	Rnf17	1640	185.5	5.48	510400.6	658692.5	632506.2	683289.0	533244.2	507329.9	418580.1	499753.3	544981.8	618352.3	575379.3	632833.3	555318.8	612449.3
P62334	26S proteasome regulatory subunit 10B	Psmc6	389	44.1	7.49	1004869.5	1021073.3	906340.4	975367.5	973200.4	899672.6	831467.9	899673.8	880623.0	955762.4	866445.4	885573.4	826999.5	896058.3
Q9DB77	Cytochrome b-c1 complex subunit 2, mitochondrial	Uqcrc2	453	48.2	9.25	1458346.5	1460513.0	1533137.6	1512395.0	1466416.9	1456866.0	1307206.8	1441488.4	1455182.0	1499030.3	1055663.0	1010207.4	1190304.9	1047252.1
P28654	Decorin	Dcn	354	39.8	8.68	1538862.5	1388049.3	1378081.0	1346723.9	1949078.8	2589292.1	3607859.3	2273507.9	1661247.6	1534850.4	1237025.5	991121.2	1051225.6	1141360.7
P62806	Histone H4	H4f16	103	11.4	11.36	7913561.7	7861604.8	11894701.6	8115915.7	12691977.7	12091207.8	10505625.4	9852323.8	9803202.3	8737986.3	10202160.4	10038471.0	16100789.5	10059550.4
Q91YR7	Pre-mRNA-processing factor 6	Prpf6	941	106.7	8.09	440572.0	443524.5	458436.5	451460.1	458982.1	436645.3	423474.6	419981.3	444281.3	478208.4	635465.5	670809.4	617068.8	668113.3
Q8R0Y6	Cytosolic 10-formyltetrahydrofolate dehydrogenase	Aldh1l1	902	98.6	5.91	346735.8	342171.0	337581.8	351256.3	342346.5	305362.8	380181.1	296669.0	340919.6	354185.5	339619.0	361408.2	312075.2	334850.5
P63158	High mobility group protein B1	Hmgb1	215	24.9	5.74	3747946.9	3727706.3	4369859.6	3681071.6	4950586.5	5242919.3	5646480.9	4540435.6	4299145.1	4301704.2	2281767.5	2572654.7	2832808.1	2282917.3
P52431	DNA polymerase delta catalytic subunit	Pold1	1105	123.7	7.61	479741.3	509278.0	498729.0	491741.5	526811.1	531749.2	488756.4	469004.2	521193.0	530318.4	632701.4	743737.7	623017.4	702609.0
Q8BX17	Gem-associated protein 5	Gemin5	1502	166.5	6.71	546554.4	578729.8	579106.8	565138.1	568792.4	577857.7	480249.7	525211.3	571434.9	562587.7	435582.4	455111.3	448264.9	431327.8
Q61555	Fibrillin-2	Fbn2	2907	313.6	4.84	212831.6	198286.9	214307.7	200510.1	249610.8	308385.3	293688.8	233577.5	232885.7	236796.4	254614.1	207395.5	198424.4	197977.2
Q3V3R1	Monofunctional C1-tetrahydrofolate synthase, mitochondrial	Mthfd1l	977	105.7	7.02	348065.8	337561.4	299913.6	343764.6	327047.8	328238.8	350423.1	341168.3	324387.6	364107.2	427417.6	427961.0	383908.5	428175.8
Q8VDM6	Heterogeneous nuclear ribonucleoprotein U-like protein 1	Hnrnpul1	859	95.9	6.58	1030711.7	1117582.7	1027999.3	1138027.0	988260.4	1010154.5	1065309.0	912956.4	1060231.1	1101443.3	816451.8	841288.1	812536.7	845717.9
Q6P542	ATP-binding cassette sub-family F member 1	Abcf1	837	94.9	6.51	421132.5	449589.1	415457.3	425479.2	397380.3	392698.1	349127.7	420980.2	401546.3	410176.9	344719.3	336769.7	332694.2	339827.0
Q61391	Neprilysin	Mme	750	85.6	5.81	649444.3	609088.9	593128.1	564003.3	717180.3	743458.4	689928.5	664369.0	631775.7	639016.6	895766.6	782107.6	877250.4	751265.3
Q8BMK4	Cytoskeleton-associated protein 4	Ckap4	575	63.7	5.64	890450.6	797889.7	810885.1	832769.0	828687.8	844840.5	699150.5	989634.2	718604.2	758436.8	524171.1	522215.9	495063.2	526555.7
Q9QZQ1	Afadin	Afdn	1820	206.4	6.32	276451.3	282183.2	260538.1	279198.7	274354.4	267938.8	261110.0	239821.0	272640.4	289923.2	476584.2	504008.0	474545.7	476274.5
Q8JZN5	Complex I assembly factor ACAD9, mitochondrial	Acad9	625	68.7	7.46	444462.0	602582.8	493071.9	626134.5	417161.4	401671.7	440800.9	414287.1	528028.7	625302.7	321887.0	307896.6	321625.2	307062.1
Q9D5V6	Synapse-associated protein 1	Syap1	365	41.3	4.54	342090.2	318314.6	357488.7	330046.4	346273.1	341361.1	328698.6	328216.2	329099.9	298646.5	448584.7	551651.4	443806.9	510754.8
P52825	Carnitine O-palmitoyltransferase 2, mitochondrial	Cpt2	658	73.9	8.37	1466383.3	1385168.0	1507181.6	1523292.3	1444631.6	1421240.7	1376694.7	1373691.6	1396262.3	1420154.5	1200516.6	1435148.6	1227756.2	1467559.4
O08759	Ubiquitin-protein ligase E3A	Ube3a	870	99.8	5.08	287818.1	291681.4	295083.0	288622.2	298794.1	305661.6	309899.7	291891.6	287631.6	295422.0	135084.0	146970.7	140833.2	145718.8
Q9D5V5	Cullin-5	Cul5	780	90.9	7.81	414205.1	497527.9	443328.6	446132.3	479864.7	515098.5	434710.9	429214.6	455172.5	467754.0	416758.0	415607.8	466775.3	416506.3
Q9Z1T1	AP-3 complex subunit beta-1	Ap3b1	1105	122.7	5.66	785699.9	837481.9	784771.3	800113.7	790291.1	791207.3	755278.8	763625.9	821110.1	831144.1	783264.1	847917.0	784698.2	805430.1
P97371	Proteasome activator complex subunit 1	Psme1	249	28.7	5.97	917363.4	892969.1	954522.7	912642.5	1056506.2	957231.4	1037660.1	1017732.0	958751.6	922487.2	1226760.1	1219425.1	1407726.7	1235699.5
P10852	4F2 cell-surface antigen heavy chain	Slc3a2	526	58.3	5.91	582598.6	642063.3	544041.0	616054.2	558654.4	493226.0	523411.9	483043.2	539494.1	543174.1	655564.7	669623.3	610151.0	651182.9
P35700	Peroxiredoxin-1	Prdx1	199	22.2	8.12	11249646.7	10665007.2	12666743.7	11014075.7	12563812.4	12285994.8	11019774.3	11699030.0	12189340.6	12203534.0	11941904.1	11392096.6	14112651.0	11548528.8
P70698	CTP synthase 1	Ctps1	591	66.6	6.58	907712.9	949124.9	1058048.1	915867.0	1026555.3	988537.7	805346.5	871032.8	988650.0	954391.2	967764.4	1068819.8	1264830.8	1044799.6
P14576	Signal recognition particle 54 kDa protein	Srp54	504	55.7	8.75	396339.6	394784.4	401850.8	393217.9	407890.5	425473.7	384885.6	383867.3	391611.1	390703.9	425444.0	402908.6	431644.4	377753.2
P62141	Serine/threonine-protein phosphatase PP1-beta catalytic subunit	Ppp1cb	327	37.2	6.19	552443.8	593167.2	659398.1	602471.7	610273.5	616280.9	621052.3	590223.1	599231.2	598839.1	722347.6	808903.5	832079.2	818182.7
P22599	Alpha-1-antitrypsin 1-2	Serpina1b	413	45.9	5.54	4245444.5	3999603.6	3126747.7	3522046.2	1752096.8	2172629.3	4508791.3	3768122.3	3565190.0	2193362.1	1637753.0	1358659.6	1265384.6	1180263.2
Q8JZQ2	AFG3-like protein 2	Afg3l2	802	89.5	8.6	909606.2	894226.3	937332.5	869664.6	919902.6	869689.9	890535.0	851694.8	893516.1	891484.5	697723.7	705565.6	649725.3	743421.3
Q62188	Dihydropyrimidinase-related protein 3	Dpysl3	570	61.9	6.49	439142.7	434634.7	407839.5	404790.9	445136.6	531678.9	571197.2	500271.2	454998.7	460104.6	247076.6	240217.4	242480.2	213849.0
Q8BFZ9	Erlin-2	Erlin2	340	37.8	5.5	925378.5	862674.3	914884.3	916498.1	948807.0	917076.0	887706.8	886753.7	887292.8	909048.1	1290586.8	1292293.1	1209551.8	1315388.8
Q6PR54	Telomere-associated protein RIF1	Rif1	2419	266.1	5.57	437777.7	453152.6	441377.1	436951.9	422279.1	418885.1	407786.4	418495.3	463863.7	464185.2	312733.2	268964.6	317131.0	277670.8
Q8K224	RNA cytidine acetyltransferase	Nat10	1024	115.3	8.32	625375.4	618665.0	576921.5	575163.0	596112.9	592112.1	532527.2	555898.6	611470.6	622038.8	324252.4	341984.9	346666.2	326077.0
P43277	Histone H1.3	H1-3	221	22.1	11.03	3357985.6	2703161.4	4210587.5	2690909.6	4451280.9	4774183.5	4022723.4	3965772.2	3593569.2	4046748.9	4502604.2	3469749.3	5606156.8	3395977.9
Q8K0E8	Fibrinogen beta chain	Fgb	481	54.7	7.08	530741.8	551407.4	484444.8	524822.5	368370.1	394631.9	539691.6	542301.2	514756.8	532722.8	518021.8	471885.8	397413.9	434501.2
E9Q0S6	Tensin 1	Tns1	1888	201.3	7.75	290655.4	299507.7	298133.6	283968.5	343669.5	343659.4	392779.2	321490.2	306415.2	301226.0	544498.5	474396.2	527388.9	492774.6
Q9Z1Q5	Chloride intracellular channel protein 1	Clic1	241	27	5.17	736730.4	679591.4	622488.4	670751.1	704657.3	733018.9	760851.6	782552.1	688532.3	634109.9	635333.7	588172.8	544632.3	580007.4
Q9QXX4	Calcium-binding mitochondrial carrier protein Aralar2	Slc25a13	676	74.4	8.6	365624.7	384874.3	428368.6	389891.4	394736.7	406413.0	414719.7	389692.2	370252.9	392383.4	531269.9	530501.9	507084.2	501961.6
P10493	Nidogen-1	Nid1	1245	136.5	5.44	656018.2	583239.2	599522.2	559239.4	736411.5	840540.8	824601.6	735827.8	660843.4	679095.3	941458.5	882869.6	882999.3	876991.8
Q9CZU6	Citrate synthase, mitochondrial	Cs	464	51.7	8.57	1679406.4	1642496.2	1876212.4	1693745.8	1866028.0	1612153.8	1579884.6	1869826.7	1798021.8	1732537.2	2156473.4	2235949.5	2402306.5	2204454.6
Q62465	Synaptic vesicle membrane protein VAT-1 homolog	Vat1	406	43.1	6.37	738115.2	782012.5	821807.0	694337.2	891330.2	950345.3	986944.6	832720.6	841191.2	812843.4	592618.6	593180.5	506239.3	532886.9
P18242	Cathepsin D	Ctsd	410	44.9	7.15	1680661.2	1808187.9	1848282.4	1805588.2	2339250.7	2331796.5	3167162.7	1876759.7	1982685.8	1920910.3	1707099.2	1540981.1	1781924.3	1690882.9
P97742	Carnitine O-palmitoyltransferase 1, liver isoform	Cpt1a	773	88.2	8.62	781996.5	729297.5	738486.3	719951.3	774012.2	816951.4	868021.2	797849.8	800854.2	726588.1	1320560.0	1246336.5	1332054.4	1357753.0
P61205	ADP-ribosylation factor 3	Arf3	181	20.6	7.43	4444109.5	4513626.7	4572042.6	4613619.9	4182546.1	4336301.0	4257854.7	4328502.4	4374521.1	4634767.9	4587660.9	4667938.3	4634701.3	4828292.3
Q6DIC0	Probable global transcription activator SNF2L2	Smarca2	1577	180.1	7.2	287489.3	304106.7	284110.0	343149.1	302733.8	308803.5	282373.1	276203.3	276095.8	303767.0	391432.9	385756.4	377056.0	391451.7
E9Q3G8	Nucleoporin 153	Nup153	1462	151.9	8.84	268033.7	290130.5	255485.3	286263.9	304108.3	303395.1	286005.9	242291.5	303341.3	286603.0	523501.4	466234.3	564808.3	459526.9
P18293	Atrial natriuretic peptide receptor 1	Npr1	1057	119	6.89	281682.7	292650.9	285007.6	275838.3	341709.6	358392.0	329800.1	325099.5	294526.9	307064.8	286144.1	261634.2	267839.7	255960.3
Q9R1C7	Pre-mRNA-processing factor 40 homolog A	Prpf40a	953	108.4	7.69	432475.1	421019.6	503625.8	441885.2	529327.6	523067.7	491311.2	479350.2	471546.1	473385.1	351340.7	366447.7	383280.9	310121.7
Q8BP47	Asparagine--tRNA ligase, cytoplasmic	NARS1	559	64.2	5.86	474549.6	508761.9	499113.4	532944.4	533826.7	562185.4	512015.0	478459.4	520135.7	529603.6	277426.5	294054.3	270282.5	289969.2
Q5SSW2	Proteasome activator complex subunit 4	Psme4	1843	211.1	7.01	212370.2	251384.2	231781.1	254015.5	225347.5	223713.7	207881.0	203689.0	231090.2	236575.6	228749.0	277209.1	220341.7	259149.0
Q80UG5	Septin-9	Septin9	583	65.5	8.9	1348858.0	1248083.1	1286094.2	1277258.1	1286970.1	1251578.0	1350487.0	1261085.3	1222078.3	1167531.5	491488.3	434144.6	451894.0	422980.1
Q6ZPJ3	(E3-independent) E2 ubiquitin-conjugating enzyme UBE2O	Ube2o	1288	140.7	5.06	450265.6	456312.3	459760.3	443062.5	511727.8	459654.5	444378.6	448724.5	464407.4	495024.3	93259.2	97204.4	116017.3	96457.7
P27641	X-ray repair cross-complementing protein 5	Xrcc5	732	83	5.16	263245.9	279799.3	275711.6	275387.4	306301.2	312448.8	306110.7	270997.3	288975.2	275190.8	526726.6	589914.7	567338.1	585266.1
Q99LC5	Electron transfer flavoprotein subunit alpha, mitochondrial	Etfa	333	35	8.38	675982.2	635280.0	587016.9	665989.6	611280.4	571715.1	654001.7	665269.6	573841.9	556293.9	590722.5	630370.1	564446.3	653019.4
Q9DBR7	Protein phosphatase 1 regulatory subunit 12A	Ppp1r12a	1029	114.9	5.49	526519.5	531347.9	537070.6	512942.2	599784.7	520028.5	579408.7	513553.6	528357.4	520747.4	719854.2	740281.9	728283.6	730718.4
Q60930	Voltage-dependent anion-selective channel protein 2	Vdac2	295	31.7	7.49	1162374.6	1224025.7	1323809.0	1426798.5	1149962.0	1034160.8	1191154.7	1139883.7	1251583.6	1178301.1	1983580.6	2022408.2	2157348.5	2297962.3
Q6EDY6	F-actin-uncapping protein LRRC16A	Carmil1	1374	151.8	7.8	462824.8	455440.5	447724.2	432399.8	447422.2	473360.5	447777.5	441471.7	474936.4	468298.1	707242.1	602261.2	609494.8	605303.0
P26369	Splicing factor U2AF 65 kDa subunit	U2af2	475	53.5	9.09	1111787.1	1107227.4	1186046.8	1091516.1	1273255.1	1241619.0	1126005.2	1029224.2	1109386.9	1146562.1	763205.8	687512.1	796552.7	710907.0
Q5XJY5	Coatomer subunit delta	Arcn1	511	57.2	6.21	779377.1	750849.9	739065.5	796827.8	743549.5	748873.0	692085.7	790550.4	746903.3	753903.0	885246.2	852841.5	719559.3	842793.0
P58774	Tropomyosin beta chain	Tpm2	284	32.8	4.7	644080.1	677738.0	606523.4	602287.6	640916.0	677541.2	762333.2	851472.0	658345.7	636812.3	2408320.1	2253524.0	2393953.0	2200510.7
Q8R016	Bleomycin hydrolase	Blmh	455	52.5	6.48	962827.2	919901.7	936670.6	882927.0	977927.9	972110.2	966370.5	922956.0	923521.6	921698.3	1315897.1	1395168.9	1404474.5	1418163.7
P54775	26S proteasome regulatory subunit 6B	Psmc4	418	47.4	5.21	709662.2	825623.7	770425.8	835230.6	793934.3	709896.0	713975.0	706152.5	810711.5	852857.2	618473.3	860033.7	690493.6	801590.9
Q8BGC0	HIV Tat-specific factor 1 homolog	Htatsf1	757	86.2	4.4	1401010.0	1392943.2	1337701.3	1315139.8	1271031.4	1170390.5	1166556.3	1316573.9	1391485.1	1438646.2	1190804.6	1073300.4	1153496.2	1059488.4
Q8BYA0	Tubulin-specific chaperone D	Tbcd	1196	133.2	6.51	885786.0	714924.4	712073.0	719729.2	789016.7	761492.9	788403.6	807991.4	844496.7	836905.2	157657.7	145713.8	136060.2	154374.7
F8VQC1	Signal recognition particle subunit SRP72	Srp72	671	74.6	9.23	463965.7	293070.9	275109.7	434333.6	284292.9	268214.1	237206.3	412744.3	439793.9	286197.2	324986.6	341956.4	347742.8	357425.3
Q8CFI7	DNA-directed RNA polymerase II subunit RPB2	Polr2b	1174	133.8	6.87	204094.4	241874.9	227370.8	225713.7	231644.8	221748.1	193637.0	207273.3	221859.1	233919.5	674147.2	686549.4	712987.1	652308.4
P63276	40S ribosomal protein S17	Rps17	135	15.5	9.85	914341.8	1102742.3	960920.8	986532.9	974964.9	867287.3	869583.0	976537.7	902481.4	992079.5	971163.2	838352.4	976291.2	875709.2
Q6A026	Sister chromatid cohesion protein PDS5 homolog A	Pds5a	1332	150.2	7.85	192658.4	194536.5	191405.8	194125.2	216741.9	191900.8	216243.4	193959.6	196627.0	188658.8	420927.6	421437.1	519791.5	502243.5
P54071	Isocitrate dehydrogenase [NADP], mitochondrial	Idh2	452	50.9	8.69	1246813.6	1178390.5	1019518.1	1193158.6	1149239.0	1267372.0	1460947.5	1288149.7	1186885.8	1135349.6	1512762.5	1250354.2	1265800.9	1286116.7
P14206	40S ribosomal protein SA	Rpsa	295	32.8	4.87	2020216.9	2085151.5	1633396.1	1899432.9	1778173.1	1724932.3	1552268.7	1586715.2	1818203.7	2002562.5	1821294.6	1778550.4	1909677.8	1832432.4
P32261	Antithrombin-III	Serpinc1	465	52	6.46	1982146.5	1982431.6	1515580.0	1729292.9	1254561.9	1441514.8	1939189.4	1831957.9	1542499.8	1544774.9	1994181.6	1877470.1	1451422.9	1569451.1
A8C756	Thyroid adenoma-associated protein homolog	Thada	1938	217.2	6.25	267133.8	235043.8	229225.7	245544.1	227964.4	237416.7	201445.1	221254.0	238411.0	248889.3	332204.9	340119.4	312346.2	318321.9
Q5SYD0	Unconventional myosin-Id	Myo1d	1006	116	9.41	432300.1	455952.6	436687.7	449978.0	489796.8	484193.4	554374.4	452703.6	467339.9	478435.4	535882.8	541994.0	525556.2	555356.3
Q9CPV4	Glyoxalase domain-containing protein 4	Glod4	298	33.3	5.47	1098078.6	1150073.4	1023776.2	1089113.2	1161898.8	1170818.5	1109929.3	1043374.4	1152397.8	1204619.7	1365354.5	1479255.7	1373306.9	1411709.4
Q91V61	Sideroflexin-3	Sfxn3	321	35.4	9.51	915148.6	817847.9	782391.4	845132.5	779474.0	782039.6	894999.9	868090.4	778884.5	738680.9	570147.4	557538.8	599196.9	541968.2
P46460	Vesicle-fusing ATPase	Nsf	744	82.6	6.95	814638.8	975900.0	881428.0	932500.9	961252.6	921718.3	880110.2	767668.6	922775.7	954081.9	984605.6	954754.6	1031152.2	946601.8
Q91ZJ5	UTP--glucose-1-phosphate uridylyltransferase	Ugp2	508	56.9	7.61	1332325.8	1400509.3	1451975.2	1343195.7	1458697.4	1509680.5	1359249.9	1369276.0	1390163.4	1416582.5	954254.3	956584.5	960400.3	885803.9
P43274	Histone H1.4	H1-4	219	22	11.11	1260009.0	940836.7	1264545.5	908552.5	1431178.5	1588292.7	1611152.6	1213076.5	1238119.8	1218410.4	592648.0	621931.0	620380.2	600235.2
P60335	Poly(rC)-binding protein 1	Pcbp1	356	37.5	7.09	1378303.8	1580958.9	1308219.6	1548865.6	1052763.8	1280130.3	1520386.7	1404153.5	1379245.1	1390432.2	1111324.7	1228813.9	1193554.4	1301996.9
Q91VW5	Golgin subfamily A member 4	Golga4	2238	257.4	5.36	208930.4	219150.4	197810.2	217740.5	216498.0	197471.4	182288.5	195578.0	204235.3	204262.9	179479.0	193665.6	163389.7	190828.4

Q60668	Heterogeneous nuclear ribonucleoprotein D0	Hnrnpd	355	38.3	7.81	2907525.9	3000653.2	3136059.2	2898440.6	3490765.8	3192938.9	3313645.1	2970936.6	3254002.5	3172520.4	4754076.9	3983805.4	4970168.0	3952901.0
Q8R5M8	Cell adhesion molecule 1	Cadm1	456	49.8	5.03	437569.1	398484.5	355640.3	375103.9	379107.9	360365.3	373952.4	358512.6	401206.0	406589.7	196468.4	183075.5	211440.2	169836.7
Q02248	Catenin beta-1	Ctnnb1	781	85.4	5.86	537907.7	499485.6	523108.5	533185.5	487304.5	546494.0	543287.4	551642.0	501225.3	481733.1	305762.9	308853.8	290224.0	302440.0
P48774	Glutathione S-transferase Mu 5	Gstm5	224	26.6	7.21	1501191.5	1703421.1	2077655.6	2120761.7	1617038.8	1657086.4	1667258.2	1722358.1	1718752.0	1753302.9	2337379.9	2619276.5	2909967.2	3313651.9
Q8BND3	WD repeat-containing protein 35	Wdr35	1181	133.9	6.34	445098.0	514086.3	536379.7	602603.0	417566.5	394113.5	350991.0	403499.0	441728.8	479797.6	342326.1	403002.1	345065.8	440622.9
Q8C854	Myelin expression factor 2	Myef2	591	63.3	8.87	1024453.0	1148790.5	1132403.9	1190822.3	1129256.4	1076433.0	1022559.1	972904.8	1112332.1	1125454.6	977120.6	1069399.9	1070149.8	1122079.6
Q99PA7	MAGE domain-containing protein	[illegible]30550L24[illegible]	320	35	5.14	898171.1	896396.1	1025389.5	869250.6	891629.3	837386.6	836149.1	934353.2	885183.2	959024.3	567802.4	473358.9	506548.6	473239.7
Q3V132	ADP/ATP translocase 4	Slc25a31	320	35.2	9.58	3517974.7	4223731.2	4672342.8	4677661.2	3944071.8	3644334.4	2865156.5	3303731.3	4324182.2	4438780.2	2244432.9	2462233.5	2811133.0	2781802.6
Q62448	Eukaryotic translation initiation factor 4 gamma 2	Eif4g2	906	102	7.14	565642.5	622945.1	607500.4	609399.4	611370.5	577646.9	538271.2	527416.7	563956.3	564725.8	594726.7	639965.1	609552.0	636391.3
Q9WUA2	Phenylalanine--tRNA ligase beta subunit	Farsb	589	65.7	7.12	871455.1	983871.3	946832.6	971338.9	898727.8	838379.2	869642.4	818531.3	951693.9	980228.4	1348361.9	1525640.1	1466313.6	1479470.7
P42208	Septin-2	Septin2	361	41.5	6.55	616947.2	572358.0	608022.3	544562.3	629882.6	682012.9	673437.4	624070.5	596319.3	565262.3	895187.1	794262.6	852943.3	786111.6
Q00612	Glucose-6-phosphate 1-dehydrogenase X	G6pdx	515	59.2	6.49	1370413.7	1435948.3	1233101.7	1327142.5	1269120.8	1155207.8	1277789.1	1343521.9	1370600.0	1377784.6	915810.8	919725.6	864422.7	847103.1
P14869	60S acidic ribosomal protein P0	Rplp0	317	34.2	6.25	1477307.0	1697246.5	1465582.9	1674767.9	1502918.7	1452497.7	1555254.8	1449093.6	1668062.7	1701904.4	2309087.7	2403052.2	2234294.2	2456804.2
Q920F6	Structural maintenance of chromosomes protein 1B	Smc1b	1248	144.4	7.12	622348.8	653508.5	667188.9	614541.3	755081.0	750309.4	639769.8	639352.5	677378.5	657163.0	348094.3	434971.0	387576.9	390273.0
Q9JIX8	Apoptotic chromatin condensation inducer in the nucleus	Acin1	1338	150.6	5.91	480538.8	403006.9	521447.0	430250.1	419476.2	381782.0	356209.2	366263.7	384923.2	419086.9	424431.7	437281.8	462892.2	461337.9
E9Q7L0	Transket_pyr domain-containing protein	Ogdhl	1029	116.5	6.84	232775.3	247570.9	220042.2	261071.1	229443.6	239423.5	244231.4	212040.7	232692.8	253509.9	257386.1	286095.8	222283.6	290577.9
Q9CZ44	NSFL1 cofactor p47	Nsfl1c	370	40.7	5.15	524725.6	517073.5	538729.6	603736.6	561877.6	525612.8	591260.6	526140.4	560415.1	576551.3	381066.1	346137.9	338497.0	425586.3
Q91WJ8	Far upstream element-binding protein 1	Fubp1	651	68.5	7.93	341297.0	395119.5	395835.6	393021.1	383712.8	390042.6	393546.7	320559.0	422816.3	411553.5	1015924.7	1017993.0	1087205.7	970192.8
Q61191	Host cell factor 1	Hcfc1	2045	210.3	7.18	501621.6	517121.2	503482.7	468919.1	573961.6	576673.0	566885.1	494515.6	529415.2	515918.7	590042.8	666988.0	622321.4	600206.1
Q99JY9	Actin-related protein 3	Actr3	418	47.3	5.88	750816.4	699070.4	731189.5	692010.2	724167.8	685170.4	701787.4	792538.8	665960.7	656691.7	416700.9	385781.4	444179.9	403097.2
Q9WV92	Band 4.1-like protein 3	Epb41l3	929	103.3	5.31	323490.7	301941.1	337470.8	308485.4	350463.8	347610.5	363631.8	312169.1	344170.2	323964.6	457929.2	483824.9	411701.2	453528.4
Q9DCN2	NADH-cytochrome b5 reductase 3	Cyb5r3	301	34.1	8.38	671455.8	642684.7	628186.2	663784.8	634186.1	619644.4	650218.1	601463.3	649165.3	683527.0	577140.6	548150.5	663933.2	588490.0
P18653	Ribosomal protein S6 kinase alpha-1	Rps6ka1	724	81.5	8.1	162449.8	196081.3	180112.4	168201.8	171641.7	193365.4	175375.7	152948.0	160367.7	176129.3	130693.2	151978.8	153503.8	149598.8
Q01405	Protein transport protein Sec23A	Sec23a	765	86.1	7.08	351912.7	366238.6	332206.7	344002.5	332196.9	368532.6	341905.7	326550.9	340807.8	353272.2	347327.6	373296.2	339969.8	351599.9
P21614	Vitamin D-binding protein	Gc	476	53.6	5.5	753336.7	559819.7	552485.3	514459.7	285532.5	367038.8	599437.6	745327.4	482296.8	379842.4	756579.8	869757.2	650490.8	762770.8
Q61881	DNA replication licensing factor MCM7	Mcm7	719	81.2	6.37	519422.5	564091.0	579164.6	501789.8	578306.4	563083.5	493077.8	545046.8	496767.3	518062.8	591430.5	649144.9	616597.2	619302.5
Q8VI75	Importin-4	Ipo4	1082	119.2	5.03	490442.7	549360.6	491942.2	547857.1	525897.2	478518.5	480936.3	450448.8	535331.2	514351.6	587346.1	578283.4	592175.2	581913.7
P42227	Signal transducer and activator of transcription 3	Stat3	770	88	6.3	547173.7	528641.3	523133.9	532029.8	501325.6	518795.3	554071.4	533371.3	521745.4	503263.4	342375.2	348470.5	332962.4	338948.6
Q99020	Heterogeneous nuclear ribonucleoprotein A/B	Hnrnpab	285	30.8	7.91	2686214.7	2509191.6	2653946.3	2399575.1	2862182.9	2833724.2	2513421.9	2602634.4	2588412.1	2914869.5	5628777.7	4862940.9	5536616.1	4725114.0
Q61183	Poly(A) polymerase alpha	Papola	739	82.3	7.34	336333.8	326704.2	354845.1	347171.7	358356.3	322287.1	296079.3	343946.7	351086.2	357177.8	357324.2	368556.0	380683.9	357099.4
Q8BRT1	CLIP-associating protein 2	Clasp2	1286	140.7	8.63	273247.8	273047.1	261225.5	252734.0	289568.2	286893.4	280208.5	244850.0	270661.3	283957.3	52020.3	65315.1	53910.8	60918.9
Q14BI7	ATP-dependent RNA helicase TDRD9	Tdrd9	1383	155.9	6.7	389383.8	435353.9	427025.7	473519.6	388125.6	334459.7	331866.9	356489.7	376951.4	432161.9	489429.7	602458.3	558356.3	641309.2
Q9CR16	Peptidyl-prolyl cis-trans isomerase D	Ppid	370	40.7	7.43	667935.9	762912.4	731841.7	782481.3	620307.7	630198.2	575542.3	640642.9	679622.8	683290.5	636824.5	683249.0	683335.8	717980.6
P26638	Serine--tRNA ligase, cytoplasmic	Sars1	512	58.4	6.3	1057696.7	1130341.6	927397.5	1073126.2	1028253.4	939704.1	1058960.3	989027.9	1062333.6	1011777.0	433304.7	467767.0	406623.8	458573.0
Q9QYJ0	DnaJ homolog subfamily A member 2	Dnaja2	412	45.7	6.48	602709.8	824061.4	608952.8	770272.5	782691.4	704403.9	600461.8	708279.0	745671.2	828653.1	351457.2	378130.1	375215.8	372285.3
P97429	Annexin A4	Anxa4	319	35.9	5.57	844576.8	853466.4	769823.5	773221.5	880486.3	815456.4	1049094.2	806341.3	855246.4	905908.7	666353.0	659169.3	645262.9	597472.6
Q9CRC8	Leucine-rich repeat-containing protein 40	Lrrc40	602	68	6.89	701533.0	622316.0	626518.9	702871.9	690539.3	677145.7	665696.1	776121.0	659037.5	600254.2	361286.4	376093.1	351995.4	362812.9
Q8CG47	Structural maintenance of chromosomes protein 4	Smc4	1286	146.8	7.3	487219.0	503019.3	472128.0	465988.1	494720.9	488240.0	456293.1	424693.8	499120.6	518936.7	756033.6	821823.5	805928.0	782416.4
Q9CSH3	Exosome complex exonuclease RRP44	Dis3	958	108.8	7.53	440542.2	462038.5	474384.3	440643.4	532976.2	522179.7	456444.8	445022.9	493508.9	488042.9	359210.4	424565.5	386921.0	400086.0
P47856	Glutamine--fructose-6-phosphate aminotransferase [isomerizing] 1	Gfpt1	697	78.5	6.84	259428.0	291924.3	263441.8	275949.5	280775.5	266106.0	228815.9	251864.5	269110.2	281766.6	520513.1	606170.8	556471.7	636920.3
Q6NWV3	Intraflagellar transport protein 122 homolog	Ift122	1182	134.7	6.98	368238.9	413521.4	402853.9	468748.3	351728.9	325795.1	302050.1	320427.7	385593.4	406463.4	271075.3	296887.9	287900.4	321925.2
Q99P72	Reticulon-4	Rtn4	1162	126.5	4.54	197859.5	199370.5	195936.0	224560.0	212717.2	180541.1	185611.7	184454.8	184098.0	202942.1	197216.5	221650.8	209055.4	212569.9
P62082	40S ribosomal protein S7	Rps7	194	22.1	10.1	697093.1	768061.5	650402.6	738286.0	681013.2	702642.3	722723.1	684047.7	656342.7	689322.2	807838.9	619204.7	829333.4	758601.4
P28352	DNA-(apurinic or apyrimidinic site) lyase	Apex1	317	35.5	7.91	457596.6	435129.8	478303.7	421262.0	504435.3	530807.0	549282.0	473033.4	484350.1	491168.6	827064.2	911289.0	862425.6	858673.7
O70194	Eukaryotic translation initiation factor 3 subunit D	Eif3d	548	63.9	6.05	640570.5	675373.6	586345.5	677793.1	573730.1	539348.9	570373.3	576312.6	626907.5	687157.8	478496.7	455223.6	483643.3	476668.9
Q99LX0	Parkinson disease protein 7 homolog	Park7	189	20	6.77	1037515.1	965768.6	1191437.1	1021132.2	1104858.2	1068822.5	1037417.6	1128097.1	1080489.4	1068069.8	1064788.2	1039415.7	1195713.3	1096979.9
P30681	High mobility group protein B2	Hmgb2	210	24.1	7.31	4940644.3	6245010.3	6446315.3	6175991.0	6143964.2	5482022.6	4552098.6	4966731.9	5959863.3	6051628.8	2493356.2	3066972.8	3418333.0	3194443.0
P54823	Probable ATP-dependent RNA helicase DDX6	Ddx6	483	54.2	8.66	309220.0	307906.2	277960.6	296069.6	294534.9	289290.9	298603.4	266440.9	315169.1	318730.3	781619.5	697233.0	866887.0	744341.1
D3Z2H9	Tropomyosin 3, related sequence 7	Tpm3-rs7	248	29	4.81	515966.9	494816.1	490712.3	503359.4	477941.8	452422.8	567917.9	478973.9	462511.6	465878.2	669045.1	771826.7	713605.0	693088.5
Q9QYB5	Gamma-adducin	Add3	706	78.7	5.95	441747.1	453588.6	429411.7	421076.9	497389.3	509085.1	539738.5	438567.5	478818.4	485668.5	437144.5	472659.6	439037.9	468501.4
Q8BZ98	Dynamin-3	Dnm3	863	97.1	8.35	75115.7	84682.8	74840.9	89673.0	66642.1	63297.1	57646.9	68925.7	70845.5	76650.2	372456.5	344567.7	278801.2	352581.6
Q5XG71	Small subunit processome component 20 homolog	Utp20	2788	317.5	8.09	288609.8	308652.3	309536.4	330588.8	313110.8	308109.4	265142.2	280403.6	307899.1	320211.2	145383.1	140869.2	140862.9	143626.8
Q924C1	Exportin-5	Xpo5	1204	136.9	5.82	288247.8	288291.1	303588.5	292148.1	308517.7	301655.4	276757.0	268528.0	296199.4	313131.0	425824.0	475500.0	431808.1	427927.5
P14152	Malate dehydrogenase, cytoplasmic	Mdh1	334	36.5	6.58	4613469.1	4263899.3	4054514.1	4085155.2	4453377.1	4563684.6	4737182.0	4615624.6	4283199.7	4197227.3	4329867.6	3357962.6	3519210.5	3501311.8
Q99PT1	Rho GDP-dissociation inhibitor 1	Arhgdia	204	23.4	5.2	893429.3	837351.9	782964.5	778755.9	895826.3	890886.5	870976.3	850956.4	903600.1	938873.2	1633336.2	1504367.8	1696181.4	1465872.4
P09055	Integrin beta-1	Itgb1	798	88.2	5.94	1169426.0	916236.8	951135.7	935938.5	1036225.8	1077886.9	1476071.3	1130742.0	929303.8	1032271.9	485840.3	483610.9	437435.6	392505.1
P07742	Ribonucleoside-diphosphate reductase large subunit	Rrm1	792	90.2	6.7	716561.6	767962.2	803165.2	733109.5	900147.8	833935.9	835033.2	801926.6	810391.7	865650.9	712818.3	682259.6	814042.9	598682.5
P62897	Cytochrome c, somatic	Cycs	105	11.6	9.58	2189084.3	2018501.8	2203275.7	2039493.9	2148260.5	2150782.6	2036746.9	2235284.4	2147640.0	2142831.8	5434476.4	4550302.0	4982634.0	4388911.1
Q922U2	Keratin, type II cytoskeletal 5	Krt5	580	61.7	7.75	544696.2	569489.8	580592.9	661473.4	1034745.9	801888.2	634931.1	668461.9	667714.5	629270.4	675215.3	701915.9	694566.9	855495.9
Q6URW6	Myosin-14	Myh14	2000	228.4	5.55	134656.2	141835.8	131934.6	127789.5	140508.4	135919.0	135877.6	128638.4	149687.7	139188.8	156244.7	179206.6	133653.6	161535.2
Q91YI0	Argininosuccinate lyase	Asl	464	51.7	6.96	430391.1	391949.3	398748.6	408532.4	452581.1	502163.1	585943.2	474649.5	452365.3	441315.8	778058.1	728269.0	683383.4	758865.6
Q6PB44	Tyrosine-protein phosphatase non-receptor type 23	Ptpn23	1692	185.1	6.8	250532.2	294279.6	268198.0	260885.6	286555.8	281663.1	273773.0	271954.9	265203.9	266485.3	149548.9	174169.1	164513.6	153811.5
Q01768	Nucleoside diphosphate kinase B	Nme2	152	17.4	7.5	233137.3	547339.1	204177.7	380820.9	336869.0	276793.8	237629.0	265377.3	299796.8	364614.2	206785.9	406434.2	182383.9	303830.6
Q8C4J7	Transducin beta-like protein 3	Tbl3	801	88.2	6.81	242909.9	267454.9	272513.2	234328.9	280645.5	280268.5	229053.0	261210.8	264904.4	300270.8	277081.3	306215.8	258340.7	291128.7
Q99JX7	Nuclear RNA export factor 1	Nxf1	618	70.3	8.73	362947.7	414375.3	381997.6	414729.7	398107.0	465422.7	383718.9	343060.3	413811.1	403474.8	319970.5	329726.0	335701.1	336963.9
Q505F5	Leucine-rich repeat-containing protein 47	Lrrc47	581	63.6	8.1	495115.8	490133.8	477322.0	488346.5	485868.3	455610.7	411595.9	445235.1	471289.0	492749.0	640456.9	652192.1	641860.9	639053.4
P62908	40S ribosomal protein S3	Rps3	243	26.7	9.66	4368943.6	4158660.6	4390067.5	4164958.5	4206740.4	4178128.0	3875746.7	4472977.3	4064616.2	4061455.6	5662079.4	5562528.3	5859867.3	5478276.9
P62874	Guanine nucleotide-binding protein G(I)/G(S)/G(T) subunit beta-1	Gnb1	340	37.4	6	370774.5	344848.6	393202.1	352082.4	402039.2	408836.8	391583.8	362807.0	381943.0	387743.5	160407.9	175182.7	180293.8	166237.8
Q91Z31	Polypyrimidine tract-binding protein 2	Ptbp2	531	57.5	8.66	965600.7	1328699.7	1290440.8	1609836.3	850357.0	736925.3	619471.4	853363.6	1057104.0	1191831.5	900155.7	1192345.0	1272040.6	1479417.5
P70399	TP53-binding protein 1	Tp53bp1	1969	212.6	4.63	147759.7	149450.6	131710.7	166622.5	140176.3	144693.4	164718.0	148296.6	127333.2	159279.1	111709.4	112958.4	102407.4	121509.7
Q00897	Alpha-1-antitrypsin 1-4	Serpina1d	413	46	5.44	101137.4	111934.8	142142.7	100410.7	102808.0	102718.5	147996.0	94369.1	137974.4	106538.9	1150851.5	1103531.6	1542093.5	1106378.0
Q99KJ8	Dynactin subunit 2	Dctn2	402	44.1	5.26	614702.0	574827.1	644926.0	655176.0	524454.9	498455.4	469400.3	550000.3	554183.0	571863.7	499024.5	499537.0	540457.1	613550.8
Q8BRF7	Sec1 family domain-containing protein 1	Scfd1	639	72.3	6.38	528031.1	493373.7	549952.1	479551.5	435179.5	434368.0	420582.8	444119.5	448258.5	474687.1	576901.7	557106.5	597881.0	606403.0
Q5PSV9	Mediator of DNA damage checkpoint protein 1	Mdc1	1707	184.6	5.19	426864.2	477812.0	473333.9	479878.5	433440.2	421209.5	355454.3	372942.1	425554.1	468958.6	285514.3	291967.1	304848.3	306388.9
Q8BMG7	Rab3 GTPase-activating protein non-catalytic subunit	Rab3gap2	1366	152.4	6.23	275530.6	282156.1	259016.9	275523.1	270085.6	264065.6	238146.7	251753.1	276263.6	290172.2	338726.1	364980.3	342253.6	372010.0
P63330	Serine/threonine-protein phosphatase 2A catalytic subunit alpha isoform	Ppp2ca	309	35.6	5.54	528268.6	503019.2	518653.5	485756.5	471148.2	460710.9	492017.0	479693.4	509737.9	540711.4	191419.1	197582.4	194794.3	175669.1
P25444	40S ribosomal protein S2	Rps2	293	31.2	10.24	1361725.5	1260058.8	1841323.0	1464050.3	1458510.0	1379220.3	1353712.7	1654440.6	1403340.4	1319570.9	3371715.5	3079469.2	4295340.4	3253534.4
D3Z0M9	DEAD (Asp-Glu-Ala-Asp) box polypeptide 23	Ddx23	819	95.4	9.58	839226.6	837628.9	922473.8	848815.8	936042.7	940645.9	824761.3	865720.8	879591.8	916462.1	431965.3	442686.0	473184.4	452702.9
Q61595	Kinectin	Ktn1	1327	152.5	5.86	313078.5	294356.9	275833.0	279226.3	289586.1	301798.5	293484.8	273168.1	294490.0	296738.5	200614.8	150776.9	185022.6	169653.0
Q9Z1Z2	Serine-threonine kinase receptor-associated protein	Strap	350	38.4	5.12	891594.3	921231.2	931837.6	920670.3	931997.6	906018.2	862249.9	870851.1	962187.1	940286.3	541255.2	593044.1	615112.8	526964.3
Q9QY81	Nuclear pore membrane glycoprotein 210	Nup210	1886	204	6.65	194295.5	202652.2	190812.0	189657.6	226955.8	215881.7	225248.7	188403.2	208671.7	206528.5	315474.3	328578.3	314195.6	327559.9
B2RXC6	DNA-directed RNA polymerase subunit	Polr3a	1390	155.6	8.5	500638.5	493742.2	537375.7	513943.4	546613.0	571121.7	527859.3	499401.4	559919.6	553315.6	292131.7	298844.6	319093.5	290602.0
Q8R1B4	Eukaryotic translation initiation factor 3 subunit C	Eif3c	911	105.5	5.78	1408899.8	1443835.9	1490303.7	1362966.7	1259128.0	1199198.1	1169304.3	1347664.1	1373906.8	1428451.8	972948.2	853040.4	883407.5	900449.8
Q9EQH2	Endoplasmic reticulum aminopeptidase 1	Erap1	930	106.5	6.2	421802.5	381614.8	322487.9	377638.1	362263.8	364348.5	377882.5	370766.8	379105.9	384789.8	318191.8	302064.2	283477.0	297333.8
Q6PAV2	Probable E3 ubiquitin-protein ligase HERC4	Herc4	1057	118.3	6.29	438120.1	435326.1	430435.3	441727.0	440301.8	534525.8	495137.6	452546.2	513229.6	476099.0	305747.7	342187.8	309322.0	344377.9
P62715	Serine/threonine-protein phosphatase 2A catalytic subunit beta isoform	Ppp2cb	309	35.6	5.43	235925.5	220075.9	222147.1	217395.3	263666.9	240658.4	222195.2	238886.0	237337.4	233812.2	634948.8	598475.3	607801.4	611931.3
Q8VCA8	Secernin-2	Scrn2	425	46.6	5.71	287440.0	271514.3	294324.5	291312.7	310008.2	304820.7	338391.7	290822.7	307082.9	307731.2	543477.5	535994.0	506290.1	497865.6
P54822	Adenylosuccinate lyase	Adsl	484	54.8	7.27	560817.1	574950.5	624004.4	567899.2	648584.5	571890.6	605730.2	553020.4	582483.1	611555.1	359217.8	429984.0	435325.1	424322.2
Q99MV1	Tudor domain-containing protein 1	Tdrd1	1172	129.6	5.81	569428.2	702333.8	635488.0	789028.1	437114.2	373858.3	371134.6	492940.8	536948.6	637034.1	323383.3	391979.7	390797.1	453077.7
Q9CY58	Plasminogen activator inhibitor 1 RNA-binding protein	Serbp1	407	44.7	8.54	781650.4	801903.6	814934.6	840982.5	789215.4	765681.6	758673.9	779517.9	745054.4	770997.3	964960.7	1004952.1	972386.9	1008458.0
A6X935	Inter alpha-trypsin inhibitor, heavy chain 4	Itih4	942	104.6	6.4	392932.0	389524.8	367243.8	351577.9	262403.9	320884.8	418833.5	389977.3	368625.5	344826.6	418182.9	440576.5	355366.6	398151.9
Q8CDN6	Thioredoxin-like protein 1	Txnl1	289	32.2	4.96	1078794.1	1100689.5	1172960.8	1032831.7	1265036.1	1252374.4	1051760.6	1075752.9	1175432.2	1193202.1	1429200.3	1493359.3	1510979.3	1480338.0

Q91ZA3	Propionyl-CoA carboxylase alpha chain, mitochondrial	Pcca	724	79.9	7.25	410496.9	404036.1	379311.0	422936.0	392944.8	372620.7	405983.3	409752.1	385257.4	371081.5	525591.0	518289.1	486938.3	524767.6
Q91VA7	Isocitrate dehydrogenase [NAD] subunit, mitochondrial	Idh3b	384	42.2	8.6	797698.8	815110.1	848281.8	831664.8	871845.1	739051.4	841595.7	803829.4	863218.6	868305.3	784536.0	655602.9	736027.7	724486.8
Q6P9P6	Kinesin-like protein KIF11	Kif11	1052	118	6	408331.0	502954.5	449398.6	430420.9	487060.8	474985.3	394623.3	405072.2	448021.3	460694.4	194496.0	203012.3	214779.6	194843.0
Q61990	Poly(rC)-binding protein 2	Pcbp2	362	38.2	6.79	189447.4	329249.5	221750.3	256306.2	183948.3	229097.8	162535.9	226327.4	216354.1	223400.4	156001.7	179360.0	185176.7	187865.3
Q6GQT9	Nodal modulator 1	Nomo1	1214	133.3	6.09	665764.2	650714.7	660298.5	699717.6	653991.9	630554.0	687169.7	656181.8	631489.2	652383.9	647910.0	705697.0	581245.3	634489.4
D3YXK2	Scaffold attachment factor B1	Safb	937	105	5.35	320216.5	389153.3	385844.3	417126.3	401147.8	384346.8	373944.1	326770.7	375209.9	394130.9	431343.8	437005.0	500504.0	475733.1
P63005	Platelet-activating factor acetylhydrolase IB subunit alpha	Pafah1b1	410	46.6	7.37	1543005.6	1655798.5	1713057.6	1757288.1	1626809.7	1591414.7	1361078.9	1425423.6	1635077.9	1637662.9	1456849.4	1623622.9	1516837.0	1556502.0
Q60931	Voltage-dependent anion-selective channel protein 3	Vdac3	283	30.7	8.79	1043695.9	1078778.4	1082318.7	1080470.3	1106854.1	1081529.7	1195297.9	1012248.7	1094229.1	1108886.5	2123898.2	2071988.0	2131320.2	2186690.7
Q9JI11	Serine/threonine-protein kinase 4	Stk4	487	55.5	5.19	458208.4	507232.8	455657.3	484012.4	391384.5	373003.1	342955.5	402353.7	462588.3	453473.3	307640.8	327186.8	323436.3	331374.3
P62880	Guanine nucleotide-binding protein G(I)/G(S)/G(T) subunit beta-2	Gnb2	340	37.3	6	586383.9	546863.5	588526.3	559257.1	685272.3	675891.0	767889.4	604632.8	636888.4	637776.9	632578.9	595350.7	604943.3	607436.5
Q924M7	Mannose-6-phosphate isomerase	Mpi	423	46.5	5.95	346135.2	384660.4	356723.1	426967.6	311490.3	299791.7	307102.0	324890.3	351799.0	369383.7	384579.7	402904.9	376003.8	427407.8
Q99L45	Eukaryotic translation initiation factor 2 subunit 2	Eif2s2	331	38.1	5.8	893109.6	914693.7	923535.5	822431.1	928419.0	890271.0	738598.0	788809.6	923015.7	898141.9	476167.3	554598.5	494322.7	540403.8
P21981	Protein-glutamine gamma-glutamyltransferase 2	Tgm2	686	77	5.1	685152.1	661829.1	602593.1	650338.7	570807.9	689273.9	695077.4	707202.1	624361.3	585674.0	472620.6	430918.6	421604.4	438860.8
Q9WV80	Sorting nexin-1	Snx1	522	58.9	5.22	412664.1	420361.8	388512.0	432318.0	410284.1	428961.9	389137.3	433356.4	392105.0	399157.0	482681.2	499612.0	479190.1	508577.2
Q8CDG3	Deubiquitinating protein VCIP135	Vcpip1	1220	134.4	7.17	374462.8	360661.1	320163.3	380043.6	319337.6	314176.7	315801.3	312746.9	347532.7	347677.9	509117.1	526170.1	478957.9	510652.5
P55302	Alpha-2-macroglobulin receptor-associated protein	Lrpap1	360	42.2	7.87	2410334.3	2455026.2	2210406.2	2338695.6	2270308.4	1991687.3	2177801.4	2053515.4	2367738.0	2374012.3	3154382.9	3747082.2	3083012.0	3737550.1
P50580	Proliferation-associated protein 2G4	Pa2g4	394	43.7	6.86	831041.5	923580.9	861406.5	931374.0	823465.6	852750.2	818337.1	802526.0	869367.6	945264.3	1516913.4	1473056.5	1497710.4	1524576.7
Q8VDW0	ATP-dependent RNA helicase DDX39A	Ddx39a	427	49	5.68	288290.8	341451.4	330125.1	370128.8	308905.4	289031.3	283818.3	294415.8	339853.7	351017.9	214239.6	219703.1	203382.7	222742.4
Q6PHQ9	Polyadenylate-binding protein	Pabpc4	660	72.2	9.39	274912.5	250361.7	288298.7	231879.7	287540.6	267693.1	255140.9	288342.4	273945.5	258985.2	417654.8	387647.0	399066.6	374837.7
O54724	Caveolae-associated protein 1	Cavin1	392	43.9	5.52	1433861.5	1399393.9	1406530.5	1363145.0	1739424.5	1758090.6	1934887.2	1457974.5	1428671.8	1463785.0	1710866.4	1528098.3	1609436.3	1510643.3
Q04447	Creatine kinase B-type	Ckb	381	42.7	5.67	690499.1	575837.9	522634.2	597604.0	486953.1	661016.5	769203.4	845768.2	592791.0	535255.1	592472.4	635313.6	500784.0	579981.1
Q91WQ3	Tyrosine--tRNA ligase, cytoplasmic	Yars1	528	59.1	7.01	1425362.1	1384060.7	1481841.5	1414282.5	1468869.6	1400647.8	1295639.0	1524654.1	1400873.4	1390327.1	957258.4	972410.9	938288.4	993620.0
Q8BH59	Calcium-binding mitochondrial carrier protein Aralar1	Slc25a12	677	74.5	8.25	162751.3	152778.6	141626.1	149921.6	150418.9	158181.9	177434.2	165744.5	166765.2	152507.7	206841.6	175064.6	157778.0	159854.4
Q5SX39	Myosin-4	Myh4	1939	222.7	5.74	291673.1	259341.2	265294.1	253467.2	254712.4	239134.1	229616.0	1609199.3	239551.5	257075.6	136486.3	159999.1	126227.3	144113.1
Q78ZA7	Nucleosome assembly protein 1-like 4	Nap1l4	375	42.7	4.67	546456.4	685223.8	646171.4	689541.4	610693.3	549053.4	529018.1	512976.9	647816.8	713087.1	717542.0	1179101.3	854483.7	930981.7
Q8BJY1	26S proteasome non-ATPase regulatory subunit 5	Psmd5	504	55.9	5.21	679006.0	640436.3	614568.5	621480.6	664382.4	665745.8	652077.4	603862.2	646564.6	660660.4	495377.6	550741.0	495388.0	494132.4
Q921G7	Electron transfer flavoprotein-ubiquinone oxidoreductase, mitochondrial	Etfdh	616	68	7.58	383838.4	397834.8	366186.2	379980.2	422464.9	413220.7	420862.5	393643.9	374250.0	389803.5	528946.5	435757.3	475884.1	442693.7
Q80X50	Ubiquitin-associated protein 2-like	Ubap2l	1107	116.7	7.11	425799.7	464880.1	451661.8	412577.1	434274.4	431029.7	374763.3	441767.4	422406.6	430224.1	525806.0	508773.6	542536.3	478984.0
Q8R3C0	Mini-chromosome maintenance complex-binding protein	Mcmbp	642	72.8	5.66	284398.8	304128.6	281374.9	294678.9	352985.8	351544.5	321366.4	293110.2	308284.4	323956.9	341543.2	386046.3	354223.8	329741.2
Q9EPU4	Cleavage and polyadenylation specificity factor subunit 1	Cpsf1	1441	160.7	6.39	477309.0	524074.3	474267.2	500073.7	515704.5	507029.8	503761.3	437294.2	488226.0	521381.1	594478.5	568887.2	597625.5	582790.6
O54988	STE20-like serine/threonine-protein kinase	Slk	1233	141.4	5.14	412519.9	458657.7	451537.6	413230.2	527975.9	516062.3	455739.6	565427.1	478584.3	640791.1	94656.4	98623.6	110279.3	101556.9
Q5I043	Ubiquitin carboxyl-terminal hydrolase 28	Usp28	1051	119.2	5.19	184102.6	176165.0	175879.1	162704.0	204169.7	200316.0	193209.0	167739.8	186655.5	192203.0	404633.8	373120.8	399830.3	369179.4
Q8R3B1	1-phosphatidylinositol 4,5-bisphosphate phosphodiesterase delta-1	Plcd1	756	85.8	6.21	346310.1	305442.6	280058.1	295195.1	304623.8	291985.2	328456.5	277396.2	310296.0	322420.0	397075.7	377674.8	347738.6	343258.9
Q9QZ82	Cholesterol side-chain cleavage enzyme, mitochondrial	Cyp11a1	526	60.3	9.39	662381.1	596310.1	411166.0	807643.2	353340.0	344760.5	341078.9	448890.2	411325.6	463809.2	375819.4	377512.5	283075.9	383545.2
P27659	60S ribosomal protein L3	Rpl3	403	46.1	10.21	2491440.0	2782772.7	2686416.2	2784923.6	2379824.7	2262914.0	2006405.7	2368204.6	2510784.2	2666354.9	2558233.9	2903530.3	2810862.5	2868406.1
P35123	Ubiquitin carboxyl-terminal hydrolase 4	Usp4	962	108.3	5.64	270393.8	273939.4	291725.4	275330.5	217650.0	205605.3	195162.2	226466.0	247414.0	237858.9	380160.2	405812.8	369676.1	433186.6
Q99MN1	Lysine--tRNA ligase	Kars1	595	67.8	5.94	970031.1	870452.3	853743.3	948986.5	845877.1	818673.4	828114.9	754610.8	926054.2	993753.2	913444.8	949464.5	986955.1	1062403.6
P51174	Long-chain specific acyl-CoA dehydrogenase, mitochondrial	Acadl	430	47.9	8.31	711579.6	596586.2	657158.4	798457.8	712760.4	656300.1	598513.9	580592.0	563991.0	566044.4	411059.5	414214.3	415405.5	472254.8
Q8R326	Paraspeckle component 1	Pspc1	523	58.7	6.67	2284824.6	2053255.0	2703583.8	2186359.9	2519658.7	2418579.2	1933452.4	2383740.2	2227578.4	2167748.3	1269893.6	1374040.2	1473527.4	1527569.5
Q8BJS4	SUN domain-containing protein 2	Sun2	731	81.6	7.02	366760.9	364579.3	334805.0	338713.0	393134.9	381877.4	395596.8	369142.6	379141.6	366618.1	393422.2	382854.8	366754.2	367889.0
P37040	NADPH--cytochrome P450 reductase	Por	678	77	5.53	626785.2	601582.4	582841.7	618500.6	579119.3	589695.4	607236.2	567797.6	617629.2	611420.0	883078.8	909993.1	810949.3	849478.1
Q60520	Paired amphipathic helix protein Sin3a	Sin3a	1274	145	7.25	234096.7	230811.8	228274.0	220929.1	242902.0	249237.6	228186.5	233023.4	222529.4	230399.2	266513.0	277447.7	272278.1	273287.3
O35855	Branched-chain-amino-acid aminotransferase, mitochondrial	Bcat2	393	44.1	8.29	1449497.6	1617247.1	1388574.9	1353615.7	1478306.4	1524031.3	1442560.3	1455672.7	1419523.1	1459992.9	1190387.8	1238549.5	1158648.3	1187325.9
Q8BP92	Reticulocalbin-2	Rcn2	320	37.2	4.42	126687.6	139431.6	184986.5	144204.8	153109.2	126248.4	121086.8	125990.1	141970.7	136590.1	93005.4	124906.5	115564.6	131949.5
P47740	Aldehyde dehydrogenase family 3 member A2	Aldh3a2	484	53.9	8.35	903539.2	792849.8	859192.1	775518.9	925091.4	994581.1	1080528.6	936536.0	862340.3	883360.9	811131.2	740836.1	800446.9	689978.6
Q0HA38	Tetratricopeptide repeat protein 21B	Ttc21b	1315	150.7	7.14	300461.0	379911.4	358348.0	402875.7	282112.7	288179.9	273938.7	280068.5	308028.9	339975.7	390517.4	511466.9	469238.5	565069.6
Q99KQ4	Nicotinamide phosphoribosyltransferase	Nampt	491	55.4	7.15	819717.2	788370.1	829567.2	864614.3	762565.8	725660.8	753533.3	721146.0	822129.4	796436.9	1175002.9	892654.7	1181594.1	1194396.0
P49312	Heterogeneous nuclear ribonucleoprotein A1	Hnrnpa1	320	34.2	9.23	683481.3	626365.6	606707.2	573711.9	672463.4	688874.5	727434.0	617873.5	629515.3	663656.1	658891.4	605983.4	732743.8	584782.6
Q99LI7	Cleavage stimulation factor subunit 3	Cstf3	717	82.8	8.12	278892.7	365367.2	305904.0	331680.5	340579.8	320460.2	268228.9	283875.7	324288.9	354421.5	272788.2	278956.1	267447.4	281773.0
O88448	Kinesin light chain 2	Klc2	599	66.6	7.21	319987.8	318108.0	295022.2	316284.2	305736.9	281963.7	308393.6	298705.0	321556.9	316664.0	550699.1	464781.0	470236.6	461265.2
Q9D051	Pyruvate dehydrogenase E1 component subunit beta, mitochondrial	Pdhb	359	38.9	6.87	436178.1	510593.7	590681.6	638381.4	397443.2	369259.5	345107.9	405351.3	459279.8	502848.8	701097.6	823314.4	793196.0	883576.8
Q9WV55	Vesicle-associated membrane protein-associated protein A	Vapa	249	27.8	8.4	1273408.5	1279689.0	1132805.0	1143376.8	1111504.1	1112742.5	1151254.2	1130398.2	1209261.3	1197808.9	1401207.2	1679128.0	1432536.3	1405698.1
Q05CL8	La-related protein 7	Larp7	570	64.8	9.54	436522.3	532180.8	614436.2	638751.3	519526.2	449642.1	366382.1	406118.0	515492.1	530334.5	428968.4	429850.1	380460.1	420685.4
Q921X9	Protein disulfide-isomerase A5	Pdia5	517	59.2	7.5	642567.7	577036.1	630050.2	549356.7	586630.6	546133.2	575466.3	600915.7	604322.6	560607.0	1848956.8	1546226.3	1590469.0	1607207.5
Q3TEA8	Heterochromatin protein 1-binding protein 3	Hp1bp3	554	60.8	9.7	1390835.8	1178421.1	1440843.8	1260461.6	1948919.8	1906150.7	1976842.2	1590026.3	1494260.0	1514077.2	991261.5	876914.4	954332.3	915238.0
Q91WM2	Haloacid dehalogenase-like hydrolase domain-containing 5	Hdhd5	419	46.3	7.88	775515.3	713115.9	726632.0	709090.0	785498.9	792811.0	807879.7	695275.2	753755.4	745554.4	645316.7	591489.3	619208.2	590278.1
P70699	Lysosomal alpha-glucosidase	Gaa	953	106.2	5.83	249243.3	239493.4	243037.2	243014.8	296250.4	289648.0	263639.0	241787.2	257599.6	256174.1	215678.1	201802.7	200688.4	210697.0
P45377	Aldose reductase-related protein 2	Akr1b8	316	36.1	6.37	3317066.2	2769737.4	2769981.3	2710377.9	2910408.0	2846380.1	3296879.6	2831161.1	2943370.2	2975340.2	3712623.8	3266250.6	3454017.6	3380716.3
Q60714	Long-chain fatty acid transport protein 1	Slc27a1	646	71.2	8.38	280988.9	317046.7	269281.3	283693.4	324397.6	291913.1	313065.0	287415.3	287254.5	314108.6	172466.2	189739.3	179248.7	182935.8
Q99L13	3-hydroxyisobutyrate dehydrogenase, mitochondrial	Hibadh	335	35.4	8.13	291776.8	316702.6	290573.1	298576.3	306738.3	295179.9	276719.4	261182.4	319498.5	327104.9	364048.7	378695.2	360677.3	362550.4
Q61166	Microtubule-associated protein RP/EB family member 1	Mapre1	268	30	5.22	865653.8	862686.7	871891.6	863140.8	1006666.6	939511.0	1009337.3	858394.0	952419.4	957919.4	1498182.8	1846632.8	1512606.3	1907259.9
P52194	Calmegin	Clgn	611	69.4	4.68	426542.1	611646.4	577485.9	866436.8	333866.2	335400.2	258794.9	410221.0	462808.1	531541.4	502029.4	752438.7	784917.8	1039568.0
P13439	Uridine 5'-monophosphate synthase	Umps	481	52.3	6.61	497004.0	533144.6	530513.4	502128.6	515977.9	523601.2	490769.6	501904.4	533091.9	578570.4	621465.5	627745.8	608228.4	614120.1
P68037	Ubiquitin-conjugating enzyme E2 L3	Ube2l3	154	17.9	8.51	1017632.6	986914.0	1034254.9	1115946.7	980278.9	920559.2	816539.3	936679.1	1058284.6	1032861.3	412741.5	448962.5	395888.7	419070.6
Q8BVI4	Dihydropteridine reductase	Qdpr	241	25.6	7.81	1302675.7	1367908.3	1264659.8	1304582.2	1304015.6	1348006.5	1597336.1	1341954.3	1533212.7	1466502.1	932117.2	985659.9	822587.2	918045.9
P51885	Lumican	Lum	338	38.2	6.43	1256541.3	969705.5	1171207.9	961347.3	1707151.2	2359516.4	3090799.8	1980817.9	1233756.0	1162230.2	1785363.5	1481923.9	1616977.0	1477985.7
Q9QYR9	Acyl-coenzyme A thioesterase 2, mitochondrial	Acot2	453	49.6	7.36	595579.6	506745.1	551707.9	420209.7	655588.7	660683.9	789235.7	576676.9	619177.9	635905.0	179994.5	176913.4	164038.9	166873.5
O35857	Mitochondrial import inner membrane translocase subunit TIM44	Timm44	452	51.1	8.13	559230.4	549610.0	615004.3	552639.5	612251.2	561048.2	487268.0	557424.7	551315.8	560936.7	479546.4	448105.8	432867.1	447321.7
Q8CGF7	Transcription elongation regulator 1	Tcerg1	1100	123.7	8.65	681251.7	596897.9	587194.9	645829.7	657833.1	642776.7	616257.1	534690.8	678313.2	665533.7	1011494.6	1026315.6	1011974.3	989434.4
Q3TLH4	Protein PRRC2C	Prrc2c	2846	310.7	9.1	521497.8	527031.7	514465.6	515815.0	531980.4	521073.3	552392.2	504719.0	519777.2	540513.7	331626.1	415058.0	315484.8	348542.5
P28474	Alcohol dehydrogenase class-3	Adh5	374	39.5	7.25	1673668.0	1617759.0	1609947.8	1650983.9	1641457.5	1588313.4	1908387.2	1732220.3	1781058.2	1776265.1	1024459.4	1002123.9	996800.2	989809.1
Q9ES00	Ubiquitin conjugation factor E4 B	Ube4b	1173	133.2	6.07	203597.0	220169.8	209549.1	229479.6	198995.7	187966.6	189703.8	177112.6	202998.5	223927.2	288025.6	311754.5	273074.3	302703.7
Q3TCH7	Cullin-4A	Cul4a	759	87.7	8.35	454790.6	562751.0	546650.6	619708.5	517448.3	469487.8	434427.7	454744.7	584812.5	591126.0	285965.0	350989.9	331813.9	384504.4
Q9D892	Inosine triphosphate pyrophosphatase	Itpa	198	21.9	5.87	249654.4	386409.9	254573.2	370879.4	352150.3	290912.4	231720.9	292902.8	291871.7	364088.4	362865.7	614978.3	361590.6	488242.4
Q3UKJ7	WD40 repeat-containing protein SMU1	Smu1	513	57.5	7.18	300495.4	334464.4	299460.1	306168.7	333669.1	306317.8	331421.0	305460.1	297380.0	320212.0	369504.0	334679.2	373231.8	381749.7
B1AZI6	THO complex subunit 2	Thoc2	1594	182.7	8.44	394770.1	360785.6	363615.8	355642.3	413067.8	420045.0	389357.3	358460.9	386458.9	389523.4	660007.3	655151.1	640976.1	643718.8
Q91Z53	Glyoxylate reductase/hydroxypyruvate reductase	Grhpr	328	35.3	7.65	492864.3	451141.7	458162.2	493606.4	511782.3	469379.5	522301.3	504297.7	497490.1	484034.0	924282.4	841212.4	825606.5	829580.3
Q61035	Histidine--tRNA ligase, cytoplasmic	Hars1	509	57.4	6	1170760.6	1144793.6	1135656.5	1121354.9	1167987.2	1068430.2	1003113.2	1114618.4	1010163.6	1101846.0	1261529.6	1214492.1	1202893.2	1194183.2
Q8VCH0	3-ketoacyl-CoA thiolase B, peroxisomal	Acaa1b	424	44	8.51	34176.0	36552.3	31931.3	32171.7	35739.7	39923.3	38025.1	37000.9	44334.4	35138.7	23122.7	26168.5	25492.6	24572.5
Q04690	Neurofibromin	Nf1	2841	319.4	7.39	193214.3	190244.3	208714.8	181309.8	212639.4	217782.6	202078.5	181848.7	201786.3	194404.4	175804.2	170174.0	168899.0	199308.3
Q9JJ28	Protein flightless-1 homolog	Flii	1271	144.7	6.06	182873.5	191687.1	173557.1	188646.3	182298.1	189747.9	167901.1	175142.4	187968.8	195921.3	167739.8	203179.9	196020.1	188856.0
O35864	COP9 signalosome complex subunit 5	Cops5	334	37.5	6.54	524733.5	546536.8	589788.6	566480.3	515444.9	501547.7	499322.7	528088.5	539240.9	566104.9	289982.3	322686.4	360099.0	327192.7
A2AKX3	Probable helicase senataxin	Setx	2646	297.4	6.83	437168.5	548532.7	543324.9	536830.5	504311.5	464224.0	350053.4	444212.8	478041.5	504541.5	342796.5	392794.6	375502.5	381594.8
Q80VD1	Protein FAM98B	Fam98b	429	45.3	8.5	264475.8	257645.5	241815.6	254499.6	248203.4	233603.2	227348.7	217564.3	248915.6	245508.8	493922.4	478459.3	504654.3	467946.4
Q3TDN2	FAS-associated factor 2	Faf2	445	52.4	5.47	225083.2	225992.2	199605.9	215231.6	201965.0	194215.9	204634.9	191092.9	215347.7	216416.8	103695.7	131633.7	106107.3	108670.4
P83741	Serine/threonine-protein kinase WNK1	Wnk1	2377	250.8	6.43	674971.2	704446.2	752868.0	700750.6	713711.2	705459.7	628775.5	601098.3	689591.1	683653.9	753422.9	818454.1	751032.4	815924.8
Q8BFY9	Transportin-1	Tnpo1	898	102.3	4.98	691835.0	714597.1	690795.5	748796.4	742021.2	713945.7	702055.6	666897.6	686990.9	707713.5	454386.6	441914.2	464011.6	458930.0
Q9D154	Leukocyte elastase inhibitor A	Serpinb1a	379	42.5	6.21	579342.9	529462.2	535419.0	489565.8	583955.4	756623.1	661561.2	621871.2	528689.7	509116.4	508598.7	467013.5	439465.0	449291.4
P05063	Fructose-bisphosphate aldolase C	Aldoc	363	39.4	7.12	396135.9	425199.5	388022.6	428983.0	449961.6	470272.3	423116.6	397448.8	404794.4	404680.3	551285.2	528836.8	593440.4	537799.1
Q9CWS0	N(G),N(G)-dimethylarginine dimethylaminohydrolase 1	Ddah1	285	31.4	5.97	729964.3	751461.9	725286.8	740117.4	864694.8	913248.9	966946.1	739408.5	795967.4	782845.3	349785.1	393208.6	320941.2	336701.8
P15116	Cadherin-2	Cdh2	906	99.7	4.78	278384.8	275687.8	278710.4	241721.7	333602.5	353631.1	313335.0	272803.3	283720.4	288490.4	169418.6	154106.3	152629.6	143465.2
P47857	ATP-dependent 6-phosphofructokinase, muscle type	Pfkm	780	85.2	8	332394.8	305890.4	345551.5	317015.8	328817.3	314620.0	282036.1	329673.3	312718.2	323597.5	317529.7	311421.2	309105.7	323758.9

P22892	AP-1 complex subunit gamma-1	Ap1g1	822	91.3	6.8	1003869.8	1059436.8	1068547.1	1081687.0	1039564.1	998591.5	990549.6	951063.9	1104064.2	1083685.1	707190.1	798420.7	815369.2	761735.9
Q9Z2X1	Heterogeneous nuclear ribonucleoprotein F	Hnrnpf	415	45.7	5.49	1867263.1	1711119.7	1871356.4	1933956.3	1935630.1	1994636.8	1911019.8	1760970.5	1797245.8	1835821.5	1340723.1	1614404.6	1554920.0	1684926.7
Q9QYI3	DnaJ homolog subfamily C member 7	Dnajc7	494	56.4	6.49	400106.5	460764.8	463787.8	491232.9	393395.3	347694.2	326370.5	342598.4	421452.0	453136.1	444711.8	452014.9	481794.5	464111.5
Q9CQV8	14-3-3 protein beta/alpha	Ywhab	246	28.1	4.83	1149760.9	1091018.6	1107008.0	1077839.4	1300730.1	1121005.2	1055016.6	1233870.2	1190747.5	1048152.2	813899.0	778139.7	871695.6	842539.1
O35737	Heterogeneous nuclear ribonucleoprotein H	Hnrnph1	449	49.2	6.3	97446.4	113686.5	103234.1	100464.5	110747.7	103684.2	95375.5	92116.2	114661.6	108887.0	312989.5	388442.0	429653.8	412707.8
O35382	Exocyst complex component 4	Exoc4	975	110.5	6.49	388639.1	370673.5	341351.4	342697.2	391064.6	379088.2	419802.4	376610.5	352741.8	343161.5	353982.9	332559.2	323717.4	356969.0
O35639	Annexin A3	Anxa3	323	36.4	5.76	577249.1	536169.5	565599.8	489330.0	688121.9	744111.2	902310.0	621886.9	668662.2	626823.6	868530.5	751604.8	829725.5	673210.7
Q02257	Junction plakoglobin	Jup	745	81.7	6.14	773012.1	724992.9	723686.0	735300.0	1048624.1	947684.1	926667.4	767876.8	810729.7	797783.7	608048.6	591391.7	579603.6	583325.1
P28656	Nucleosome assembly protein 1-like 1	Nap1l1	391	45.3	4.46	581074.7	611155.7	601670.7	649198.0	562125.2	528240.4	482686.8	504223.6	558929.0	598608.4	528486.5	589639.5	619857.2	621042.7
P41216	Long-chain-fatty-acid--CoA ligase 1	Acsl1	699	77.9	7.15	577667.7	541040.7	532919.2	600073.9	639898.1	528353.2	568520.3	576835.0	554803.9	574159.0	550095.3	537868.6	530148.6	554482.3
Q61301	Catenin alpha-2	Ctnna2	953	105.2	5.71	102471.9	127305.6	106761.8	105741.0	119946.6	139751.9	127850.7	106834.0	112955.3	115456.5	236495.0	246711.6	231158.4	211573.4
Q8BH04	Phosphoenolpyruvate carboxykinase [GTP], mitochondrial	Pck2	640	70.5	7.28	291139.7	293400.9	269398.9	283400.5	314621.9	348257.5	328829.9	290722.7	286904.8	299010.2	353047.6	331497.3	320377.4	338367.5
Q9R0P9	Ubiquitin carboxyl-terminal hydrolase isozyme L1	Uchl1	223	24.8	5.24	273614.8	299332.7	397019.7	317855.2	351723.0	320146.1	325301.5	368734.9	314399.8	294920.8	408848.4	448240.3	461998.2	399228.3
O09106	Histone deacetylase 1	Hdac1	482	55	5.48	440716.0	450553.1	440101.6	461735.8	444844.3	487499.2	459739.3	407628.7	446377.5	453327.6	325156.8	352423.0	316757.3	348951.8
Q62383	Transcription elongation factor SPT6	Supt6h	1726	199	4.93	504865.2	477872.1	496009.6	474182.6	526281.8	506457.9	546339.1	494654.0	563339.4	554421.9	547404.8	518880.1	536360.8	502153.7
A6H8H2	DENN domain-containing protein 4C	Dennd4c	1906	211.3	6.4	261039.4	278108.2	271563.8	270675.2	269336.4	263323.3	270213.0	243095.2	264205.9	274264.6	270427.2	276931.6	278578.9	272063.5
P62821	Ras-related protein Rab-1A	Rab1A	205	22.7	6.21	1226185.4	1330934.6	1310365.6	1336334.3	1201029.8	1280875.9	1183015.2	1199603.4	1143189.1	1239186.9	1145028.9	1224702.6	1235088.7	1218969.3
Q7TSQ8	Pyruvate dehydrogenase phosphatase regulatory subunit, mitochondrial	Pdpr	878	99.2	6.35	386933.1	387641.2	343533.9	404338.9	371068.7	354205.2	396731.9	334767.2	401205.3	378133.8	265656.1	252628.0	226237.6	234071.5
Q5SW19	Clustered mitochondria protein homolog	Cluh	1315	148	6.02	363937.8	365403.1	340429.3	369397.5	339657.7	333537.9	338375.2	335944.6	337626.2	345048.2	414617.9	420262.7	371556.7	406405.0
Q91VU7	Pseudouridylate synthase 7 homolog	Pus7	660	74.7	5.87	230672.9	212369.9	239167.2	193031.1	233248.1	257821.7	257628.7	236328.2	272286.2	248898.3	214168.4	222513.9	210529.8	198359.4
Q8C3Y4	Kinetochore-associated protein 1	Kntc1	2207	250.2	6	189113.3	199769.5	197197.1	234293.8	196632.1	223237.1	203775.1	180351.2	224289.9	212841.2	136308.3	163093.9	148452.6	153146.7
A2A9R3	Melanoma-associated antigen B4	Mageb4	556	60.9	9.16	560352.7	476772.3	511638.7	461829.2	552141.4	564614.2	516129.3	538267.7	542289.0	510424.1	526100.7	576020.3	556478.2	472610.1
Q9QY76	Vesicle-associated membrane protein-associated protein B	Vapb	243	26.9	7.78	792388.6	800545.9	645796.5	686506.4	663967.3	682954.0	648095.5	716603.2	671495.1	644643.8	1727440.0	1633558.6	1512437.2	1680546.2
Q9CQE8	RNA transcription, translation and transport factor protein	RTRAF	244	28.1	6.89	602073.0	650346.1	699623.5	707332.6	662505.4	625693.7	615054.7	598882.5	662718.9	659569.1	290311.5	322668.1	335095.9	366224.5
Q61464	Zinc finger protein 638	Znf638	1960	218	6.89	218461.9	234916.7	230420.9	260116.6	238653.5	263849.3	224634.1	197940.8	209813.9	208489.7	239481.9	256330.0	279443.4	252322.5
Q9CQF9	Prenylcysteine oxidase	Pcyox1	505	56.5	6.92	250503.0	234091.6	226579.4	221638.9	242677.3	239903.8	238314.2	229577.6	241796.9	232319.0	415220.3	387076.3	352854.5	365631.8
Q6ZQ93	Ubiquitin carboxyl-terminal hydrolase 34	Usp34	3582	408	5.68	173830.6	165223.8	148298.0	154569.6	159546.2	170366.3	159927.9	150470.7	161985.0	165135.3	241827.9	254001.5	264243.8	247920.7
P63037	DnaJ homolog subfamily A member 1	Dnaja1	397	44.8	7.08	206559.1	239963.8	231446.4	226973.3	203226.2	188109.2	171648.3	199826.6	233350.4	228102.0	520812.1	444639.4	606729.2	474681.1
Q8K3G9	DCC-interacting protein 13-beta	Appl2	662	73.8	5.03	436740.4	433939.6	449338.7	451023.6	480215.6	469550.9	458627.2	447369.7	473371.0	474147.4	542432.6	517969.8	470938.9	504998.9
Q91V41	Ras-related protein Rab-14	Rab14	215	23.9	6.21	770428.6	870707.1	805590.5	838757.6	825911.9	846568.6	761944.9	797753.6	828695.0	819697.7	777665.3	769979.1	831998.8	781766.4
Q921M7	CYFIP-related Rac1 interactor B	Cyrib	324	36.8	6.06	449042.5	474948.3	447396.2	452041.6	449483.4	434817.2	407973.6	407554.8	433416.4	445063.7	396690.2	519938.4	408854.1	453378.7
E9PUR0	ANK_REP_REGION domain-containing protein	Ankhd1	2548	269.5	5.87	279697.2	283998.2	302438.8	280848.4	297549.7	294770.4	277180.9	266117.8	293246.0	285424.6	143414.4	151177.5	143559.9	142342.8
O55234	Proteasome subunit beta type-5	Psmb5	264	28.5	7.02	435933.2	424888.0	479262.1	451326.4	487889.1	473355.4	483417.1	435906.7	475416.7	435316.1	290736.0	292953.5	319819.4	337788.3
Q9CYR6	Phosphoacetylglucosamine mutase	Pgm3	542	59.4	6.2	821365.8	769809.3	739007.1	754734.8	732663.6	761870.7	805490.6	765489.2	774329.8	758455.0	692325.2	725897.7	633954.7	705324.1
Q6KCD5	Nipped-B-like protein	Nipbl	2798	315.3	7.91	254606.0	290409.4	300688.4	292887.5	317287.3	315124.3	287801.7	259186.2	321374.6	313470.4	334427.2	330149.2	352975.2	336737.7
Q8BSL7	ADP-ribosylation factor 2	Arf2	181	20.7	6.58	184513.0	203874.9	185257.7	185329.8	215496.7	215275.8	181934.3	174677.5	209457.6	213364.2	73113.1	77563.2	77102.6	74940.4
Q6NZC7	SEC23-interacting protein	Sec23ip	998	110.7	5.94	990274.1	918006.7	1033329.1	1038725.2	898254.9	884610.1	857176.5	918937.5	945142.4	905764.7	1015769.4	1067244.7	1068115.2	1138074.3
Q9D4E6	Polyadenylate-binding protein	Pabpc6	643	71	9.26	199967.3	247921.6	258822.5	273700.4	215675.1	194693.4	210747.9	183776.0	227136.6	252107.7	113052.5	123333.5	120443.3	139612.4
P24369	Peptidyl-prolyl cis-trans isomerase B	Ppib	216	23.7	9.55	6578760.3	5770242.3	6218967.6	6097651.5	5699771.4	6008729.2	5243393.9	6465729.7	5754693.0	5745596.4	4800864.2	4871011.5	4340830.6	5036358.5
P56959	RNA-binding protein FUS	Fus	518	52.6	9.36	645047.4	599660.9	734627.8	604195.2	743000.4	785677.8	690719.4	635511.1	666108.2	677151.0	533434.7	480169.6	592731.2	477427.3
P42225	Signal transducer and activator of transcription 1	Stat1	749	87.1	5.58	567092.7	549541.3	517179.2	546884.0	579719.7	582601.3	540702.1	522991.1	531740.1	528788.2	340290.1	374679.2	303885.6	330885.2
P84084	ADP-ribosylation factor 5	Arf5	180	20.5	6.79	237675.0	304944.4	214784.0	285667.7	217537.8	239555.8	269993.7	212620.7	276901.3	289900.0	590070.2	580263.3	567473.9	560515.8
Q9DCD2	Pre-mRNA-splicing factor SYF1	Xab2	855	99.9	6.23	318957.0	323564.5	317616.2	335100.5	309176.8	297339.1	314187.6	348109.6	335126.2	346419.3	300079.2	282160.6	285805.4	286091.4
P27046	Alpha-mannosidase 2	Man2a1	1150	131.5	8.03	257346.2	231633.8	224584.2	223914.1	220680.0	217467.2	205051.5	250211.5	205780.0	196320.7	234942.1	253567.0	202998.0	236133.6
P59325	Eukaryotic translation initiation factor 5	Eif5	429	48.9	5.52	935050.5	1155501.1	1144553.3	1161448.2	1189550.8	1115334.8	922733.5	904016.8	1113078.3	1182772.3	558730.7	586695.9	643069.1	600101.6
Q8C0I1	Alkyldihydroxyacetonephosphate synthase, peroxisomal	Agps	645	71.6	7.5	327152.6	345739.4	300418.6	325881.1	295415.2	301040.0	289313.0	394631.8	300585.0	298811.4	328776.4	357533.5	321171.3	325020.1
O08784	Treacle protein	Tcof1	1320	134.9	9.35	207719.7	183316.1	202107.7	192070.5	195619.5	212583.8	226643.4	191084.7	197271.9	193939.8	444216.5	400486.3	417744.5	354530.2
P62702	40S ribosomal protein S4, X isoform	Rps4x	263	29.6	10.15	2053224.2	2114746.3	2713869.6	2172965.6	2255704.9	2048382.7	1815196.1	2527188.9	2029709.0	2067708.4	4284968.0	4166058.6	4448404.5	4378124.6
P63087	Serine/threonine-protein phosphatase PP1-gamma catalytic subunit	Ppp1cc	323	37	6.54	71755.2	66942.9	77794.1	76411.4	69497.8	67730.1	63196.1	65913.7	67214.5	61215.7	290877.2	273157.8	253795.3	277718.7
Q99MD6	Thioredoxin reductase 3	Txnrd3	652	71.3	8.09	639451.4	733333.8	790471.3	908598.1	625801.3	561216.0	518476.9	531662.9	677008.3	730420.0	828832.7	975296.4	1092458.9	1108157.1
Q08943	FACT complex subunit SSRP1	Ssrp1	708	80.8	6.76	749135.8	852360.0	729008.5	782968.9	790911.7	776742.1	814878.1	666054.4	833796.0	884917.0	1672390.2	1699088.5	1783351.6	1596358.2
E9PVZ8	Golgi autoantigen, golgin subfamily b, macrogolgin 1	Golgb1	3238	369.9	5.05	287043.1	310717.7	283724.6	302722.7	299410.7	293696.6	266998.4	294244.2	299341.4	314959.0	232397.5	231641.3	238970.3	232669.5
Q99JI6	Ras-related protein Rap-1b	Rap1b	184	20.8	5.78	447744.8	478482.9	476153.8	473851.2	499078.0	511665.0	609157.5	493327.5	524439.0	494112.8	1372101.1	1274699.9	1230280.7	1137352.8
Q9JHW4	Selenocysteine-specific elongation factor	Eefsec	583	63.5	8.29	416455.8	440132.2	395146.0	384359.1	419572.1	393058.8	382703.0	368176.3	410895.1	434611.6	272056.6	286163.4	277707.6	267568.1
Q7TSC1	Protein PRRC2A	Prrc2a	2158	229.1	9.39	138444.2	156737.9	156308.4	151350.1	157026.0	146027.2	128804.2	145759.2	153072.6	153913.1	105072.0	130630.2	129436.7	124724.1
Q922Q8	Leucine-rich repeat-containing protein 59	Lrrc59	307	34.9	9.52	922488.9	951508.6	951256.8	938648.3	866179.4	840643.8	752720.0	917108.0	822028.9	892473.4	1067012.9	1033999.4	1016460.7	1027013.9
O35226	26S proteasome non-ATPase regulatory subunit 4	Psmd4	376	40.7	4.79	573173.5	690636.8	614461.7	637384.8	636418.4	541087.4	558946.5	580111.2	589013.2	597207.0	705857.1	565884.5	635228.8	639797.6
Q9EPE9	Manganese-transporting ATPase 13A1	Atp13a1	1200	132.3	8.03	390503.9	400357.5	345676.7	382088.7	344706.3	347911.1	345032.6	351978.6	396264.1	413917.5	309430.9	310525.2	278125.5	273810.6
Q921F4	Heterogeneous nuclear ribonucleoprotein L-like	Hnrnpll	591	64.1	5.85	820139.1	848796.2	881802.7	866567.4	826893.1	835775.0	800225.4	794071.8	855959.5	850451.3	1044203.4	950137.6	1068878.7	1102398.5
Q99JI4	26S proteasome non-ATPase regulatory subunit 6	Psmd6	389	45.5	5.52	617257.6	630925.0	589491.2	643228.3	658655.8	653934.8	666253.7	561648.2	624252.1	622935.6	415241.9	462233.5	442140.1	457774.8
O35343	Importin subunit alpha-3	Kpna4	521	57.9	4.94	151130.1	184237.0	167386.6	187978.8	165232.5	162100.8	151336.5	140206.5	197233.9	191555.0	35366.8	38480.3	40291.4	42054.2
Q9D554	Splicing factor 3A subunit 3	Sf3a3	501	58.8	5.34	288662.2	324544.1	299699.1	323865.0	336754.9	349876.7	281850.2	276424.1	307239.6	333616.2	718755.1	665484.7	854089.6	717275.8
P15532	Nucleoside diphosphate kinase A	Nme1	152	17.2	7.37	890464.0	991557.9	992531.7	1051371.5	1034136.0	1109184.5	895574.3	963200.8	940106.6	887474.7	685479.0	780540.4	720931.4	745051.7
Q7TT37	Elongator complex protein 1	Elp1	1333	149.5	6	664081.7	634490.7	657892.9	620773.0	705977.4	673394.0	604494.5	641663.4	635323.6	640539.8	431195.7	435513.8	426952.9	406312.8
Q9R1P4	Proteasome subunit alpha type-1	Psma1	263	29.5	6.46	1150706.1	1126065.6	1435514.7	1320194.5	1308245.7	1264491.0	1144965.1	1189901.9	1296815.3	1262138.2	1145110.7	1091882.1	1554563.4	1303211.5
P23198	Chromobox protein homolog 3	Cbx3	183	20.8	5.22	570541.6	627257.9	557484.6	563892.6	620471.9	674697.2	585926.1	584067.6	591912.9	595429.1	473201.8	534995.3	506423.6	507487.1
Q61830	Macrophage mannose receptor 1	Mrc1	1456	164.9	6.83	351790.2	346997.7	232872.4	382511.1	224830.6	238930.9	248865.7	291133.1	230182.1	243729.0	390056.9	462085.3	286200.9	401036.7
Q9R0H0	Peroxisomal acyl-coenzyme A oxidase 1	Acox1	661	74.6	8.48	305109.1	327239.3	273449.8	292282.6	321484.2	293649.9	297921.1	284095.7	295796.9	294467.4	189978.2	248847.8	210547.8	227908.4
Q99NB1	Acetyl-coenzyme A synthetase 2-like, mitochondrial	Acss1	682	74.6	6.98	327770.7	325771.5	344848.5	324800.7	322341.9	302204.1	321242.4	334525.1	311429.2	314558.5	316799.0	288188.0	303920.2	291449.3
Q07113	Cation-independent mannose-6-phosphate receptor	Igf2r	2483	273.6	5.71	368180.5	367652.2	383135.5	378857.0	376371.6	386572.7	372590.1	407126.1	358921.2	391463.0	266071.7	260330.6	296665.6	280631.0
Q7M6U3	Inactive serine/threonine-protein kinase TEX14	Tex14	1450	162.4	5.49	88560.0	96628.1	79650.4	100202.5	98091.7	92375.9	86689.7	83717.6	89298.9	94083.8	114777.1	154279.2	124219.4	139695.7
Q9R1P0	Proteasome subunit alpha type-4	Psma4	261	29.5	7.72	1442376.9	1497683.8	1588610.7	1621938.6	1541686.3	1462776.6	1479426.4	1427153.3	1492253.5	1543323.2	1676812.7	1877829.0	1868523.7	1854087.5
P43275	Histone H1.1	H1-1	213	21.8	10.93	1731959.3	1973123.9	2341576.2	1671101.1	2356611.4	2207389.0	1846313.7	1671945.5	2274918.1	2083810.9	1679406.4	1930753.6	2075913.9	1681788.0
Q6NV83	U2 snRNP-associated SURP motif-containing protein	U2surp	1029	118.2	8.47	293625.1	321960.6	332066.8	312240.5	343759.4	321356.1	289239.4	292330.4	310051.9	310310.1	417135.3	469089.1	434755.7	438509.1
Q9QZQ8	Core histone macro-H2A.1	Macroh2a1	372	39.7	9.8	661354.4	684243.0	695412.5	650654.8	756140.6	767403.8	808549.3	707676.0	680339.0	683746.1	485054.3	488770.2	475216.6	484820.5
Q3UJD6	Ubiquitin carboxyl-terminal hydrolase 19	Usp19	1360	150.5	6.38	455201.3	471462.6	462519.0	447693.1	407402.6	400492.1	374675.7	409771.1	460266.8	445616.7	236913.1	256334.2	221506.8	246046.3
Q5SX40	Myosin-1	Myh1	1942	223.2	5.76	108997.6	111588.1	108447.7	110682.3	102760.6	121015.3	109525.4	119569.0	110706.9	111608.7	61820.9	61976.6	49981.9	60967.8
P16015	Carbonic anhydrase 3	Ca3	260	29.3	7.37	431318.6	444772.0	278772.9	331421.8	944922.7	255838.4	561716.8	407770.3	297718.0	335281.9	308043.1	265188.0	172349.4	225397.2
Q9JII6	Aldo-keto reductase family 1 member A1	Akr1a1	325	36.6	7.39	1424807.5	1277390.2	1488037.8	1318808.1	1534166.0	1500367.4	1722801.0	1466770.0	1643511.0	1618498.5	1875529.1	1484186.7	2019915.4	1736506.6
Q91W90	Thioredoxin domain-containing protein 5	Txndc5	417	46.4	5.78	783698.7	792917.8	666218.3	767492.1	592812.4	639203.7	623750.4	804341.9	646090.0	648290.0	784897.7	719061.5	672083.9	750531.4
Q8C0C7	Phenylalanine--tRNA ligase alpha subunit	Farsa	508	57.6	8.28	841081.8	885847.4	915836.6	909118.4	804061.3	829045.7	728494.0	797215.2	890537.3	899277.6	704221.8	773180.5	851808.0	790847.3
P02089	Hemoglobin subunit beta-2	Hbb-b2	147	15.9	8.05	401546.4	158189.0	271587.4	570244.1	149710.1	144549.9	132502.7	152671.9	429162.6	171986.8	3075251.0	418645.4	1870618.6	3639816.3
P43276	Histone H1.5	H1-5	223	22.6	10.92	1361966.9	1086427.4	1438961.0	1056720.8	1651154.8	1753203.3	2100222.7	1438786.1	1396552.8	1471901.7	1619838.3	1277609.5	1568564.9	1203774.0
P31324	cAMP-dependent protein kinase type II-beta regulatory subunit	Prkar2b	416	46.1	4.98	138067.2	129654.5	87275.8	120239.7	117251.6	141659.1	107278.7	134705.3	108633.6	116713.9	489031.8	425847.2	381902.4	415253.6
P53994	Ras-related protein Rab-2A	Rab2a	212	23.5	6.54	870330.5	896113.8	875993.7	957988.2	799803.2	758894.7	751785.1	784736.5	847010.4	886168.6	1132395.1	1200671.1	1253767.6	1346810.1
Q9DB20	ATP synthase subunit O, mitochondrial	Atp5po	213	23.3	9.99	1759638.4	1786346.8	1856283.1	1894211.7	1826384.2	1606959.0	1749767.1	1872446.5	1834499.5	1677535.1	1412219.8	1478425.4	1472699.9	1528789.1
O35887	Calumenin	Calu	315	37	4.67	1295499.3	1273138.4	1198194.7	1182594.5	1193833.9	1210565.9	1154616.6	1367008.2	1217198.5	1145955.5	1095683.1	988607.3	980412.5	955942.5
Q9CZN7	Serine hydroxymethyltransferase, mitochondrial	Shmt2	504	55.7	8.47	635710.2	647671.2	593194.7	607502.7	639028.0	653289.9	616215.9	681568.3	612127.1	615315.6	760606.2	816665.2	732763.3	752343.1
Q8VEE4	Replication protein A 70 kDa DNA-binding subunit	Rpa1	623	69	7.91	477993.0	510713.9	496815.9	478922.9	518618.0	502427.3	440733.4	456037.8	485428.1	513623.1	552020.9	602791.9	585389.9	572602.0
Q8CIM8	Integrator complex subunit 4	Ints4	964	108.1	6.42	232313.6	239641.9	245109.6	241811.1	268693.4	280980.7	271234.8	233370.6	238950.5	260789.9	87088.2	99482.0	81561.1	85832.0
P70388	DNA repair protein RAD50	Rad50	1312	153.4	6.95	477916.2	495719.1	520360.7	459793.2	563431.2	582804.6	535223.5	473597.2	532126.3	510679.6	593522.4	591438.7	635639.1	534346.5
Q61503	5'-nucleotidase	Nt5e	576	63.8	6.64	192640.2	192165.0	156643.4	159412.3	181891.3	191354.8	201303.6	169912.2	205316.4	183067.9	179898.5	172204.5	140882.1	161773.2

Q9JHW2	Omega-amidase NIT2	Nit2	276	30.5	6.9	642719.0	670220.9	694679.9	617164.9	774654.8	756832.5	778731.2	681895.6	740834.7	730969.9	729301.4	687749.3	729004.8	706491.8
Q6ZQH8	Nucleoporin NUP188 homolog	Nup188	1759	196.6	7.01	209002.2	234273.7	218838.3	242556.1	249017.4	209324.4	201222.4	241416.0	224571.8	234868.0	309555.8	318050.6	319375.9	313787.4
Q80U95	Ubiquitin-protein ligase E3C	Ube3c	1083	123.9	6.39	223378.0	220026.4	213618.6	210554.1	220164.8	206232.8	202070.5	197954.6	225129.0	228683.5	244065.8	228397.5	214761.3	212792.5
Q8BUR4	Dedicator of cytokinesis protein 1	Dock1	1865	214.9	7.62	113628.3	118225.6	124674.0	118872.2	126202.0	136146.2	135643.3	131358.2	118567.7	114742.9	122419.1	111827.2	111028.6	110636.7
P50431	Serine hydroxymethyltransferase, cytosolic	Shmt1	478	52.6	6.95	568480.3	597438.2	607292.1	583197.6	632553.3	606215.3	575208.4	547113.6	629373.2	639853.3	722209.4	685656.4	737475.5	748818.2
Q6P2K6	Serine/threonine-protein phosphatase 4 regulatory subunit 3A	Ppp4r3a	820	93.8	4.89	420010.4	426336.6	505458.7	427891.4	539888.5	509580.6	466274.5	433510.8	453158.9	449766.9	488559.9	473084.8	496183.0	476720.9
P11679	Keratin, type II cytoskeletal 8	Krt8	490	54.5	5.82	579907.5	436381.7	607002.9	426968.7	575586.3	662200.4	714815.0	505696.6	563639.6	469376.4	323734.5	355751.9	298394.0	432002.6
Q9EQU5	Protein SET	Set	289	33.4	4.32	594782.3	570826.4	536089.7	551873.2	610420.6	661795.1	783820.2	555835.0	631434.6	596490.8	1034868.1	902270.8	904880.9	860248.1
Q9R0B6	Laminin subunit gamma-3	Lamc3	1581	172.2	6.96	120240.9	119927.6	112022.0	112553.3	119352.8	130267.6	122121.1	119662.9	118161.1	120024.8	146588.5	168919.2	149068.5	157608.4
P39061	Collagen alpha-1(XVIII) chain	Col18a1	1774	182.1	5.62	522956.0	462940.9	475063.7	441958.6	502785.3	511354.3	505081.8	547828.5	431981.6	449892.0	666439.3	701463.4	519689.7	582266.7
Q9QUM9	Proteasome subunit alpha type-6	Psma6	246	27.4	6.76	719202.7	796402.1	775381.3	734892.0	872837.0	735376.6	644207.1	745984.9	674300.1	692067.6	715857.5	782505.6	819693.7	783837.2
P47962	60S ribosomal protein L5	Rpl5	297	34.4	9.77	860911.1	929167.2	877303.1	900098.6	822056.7	819632.2	739500.1	787005.0	898215.8	861015.3	1006202.4	1325204.1	1152236.4	1251786.2
P84091	AP-2 complex subunit mu	Ap2m1	435	49.6	9.54	697471.0	687063.6	701215.9	665714.2	719761.8	666675.1	642637.1	648214.5	686680.3	696758.8	640233.5	618540.1	654745.2	627586.6
Q9Z2I0	Mitochondrial proton/calcium exchanger protein	Letm1	738	82.9	6.52	612302.9	649397.1	572693.5	613306.2	649588.1	604771.4	584454.7	580544.4	621791.4	604158.7	343124.3	357770.7	387729.0	359844.5
Q9QX47	Protein SON	Son	2444	265.5	5.6	245050.8	297392.9	266302.2	267710.1	267068.3	314345.6	272515.4	257083.0	275653.3	307176.7	351523.5	373901.0	368879.5	365528.8
P32233	Developmentally-regulated GTP-binding protein 1	Drg1	367	40.5	8.9	414986.5	391309.7	352168.9	368940.8	352414.3	349476.3	327846.0	394157.9	368693.5	370846.3	511846.5	549222.3	489503.4	500599.9
P14148	60S ribosomal protein L7	Rpl7	270	31.4	10.89	2194178.4	1970674.0	2519419.9	2111659.8	2216885.5	2017357.7	1487403.5	2286013.9	2072545.5	2027263.7	3664547.5	3766873.8	4087852.6	3715005.2
Q8BWF0	Succinate-semialdehyde dehydrogenase, mitochondrial	Aldh5a1	523	55.9	8.25	237826.0	229387.5	228068.6	216685.2	239488.8	238091.2	250304.3	228775.7	249735.7	259353.6	235506.2	221829.5	232176.1	219716.1
O54879	High mobility group protein B3	Hmgb3	200	23	8.37	1397232.2	1125617.3	1336752.3	977063.4	1626012.0	1788164.0	1774240.2	1447025.9	1500332.4	1566028.3	1053170.0	804935.6	1030148.9	710038.1
Q9EQK5	Major vault protein	Mvp	861	95.9	5.59	230170.2	236929.6	205425.2	220324.1	207903.0	215859.9	231034.6	208617.2	221667.5	219234.2	139489.4	153126.9	117272.2	134616.2
Q9QYC0	Alpha-adducin	Add1	735	80.6	5.9	465481.9	427307.8	454853.3	418747.0	511693.9	508729.1	544766.1	479583.8	477962.1	444539.5	356329.9	330404.2	345992.2	331029.7
Q9Z0W3	Nuclear pore complex protein Nup160	Nup160	1402	158.1	5.52	270955.0	305438.5	271828.1	282190.3	297320.6	306424.9	264966.4	261294.5	280615.3	288713.0	325115.5	346152.7	339982.4	303168.0
B9EJR8	Dynein assembly factor 5, axonemal	Dnaaf5	853	93.8	6.15	279083.9	334109.1	297582.9	338305.0	299827.3	288158.1	267355.7	278044.0	324348.5	340416.0	257272.5	278635.3	256973.6	270385.7
G3X972	Sec24-related gene family, member C (S. cerevisiae)	Sec24c	1096	118.5	6.84	678570.8	626005.5	667926.1	674862.1	592750.2	617220.3	640821.9	603041.3	618985.2	621611.7	604348.5	625059.5	507066.9	642439.8
P16332	Methylmalonyl-CoA mutase, mitochondrial	Mmut	748	82.8	6.89	480090.8	462506.0	454828.1	470202.4	481804.5	480091.7	502100.5	484073.9	445637.2	453756.2	221079.9	230097.8	197430.4	221309.3
Q8VEM8	Phosphate carrier protein, mitochondrial	Slc25a3	357	39.6	9.26	1815005.8	1581737.3	1581767.4	1531359.2	1538783.8	1512948.8	1484247.5	1780001.8	1705023.5	1563896.9	1101443.1	1071821.9	932568.7	1096342.0
Q8BK64	Activator of 90 kDa heat shock protein ATPase homolog 1	Ahsa1	338	38.1	5.53	737993.2	746605.2	774491.3	753397.5	671596.3	609310.2	634488.8	656313.3	715274.0	737713.8	260182.9	258744.0	283327.8	263940.4
Q8CAA7	Glucose 1,6-bisphosphate synthase	Pgm2l1	621	70.2	6.49	850727.6	848917.1	859181.2	903205.4	880233.9	872228.6	813679.0	790720.9	828846.0	881586.5	515507.6	552303.8	596767.8	598794.0
P19221	Prothrombin	F2	618	70.2	6.43	624781.8	508734.1	513039.7	459593.5	473718.8	429003.5	568868.6	577278.5	493205.3	507198.5	462290.5	434339.2	351297.9	386774.1
Q9CYH2	Peroxiredoxin-like 2A	Prxl2a	218	24.4	9.17	1406806.4	1339276.4	1473723.2	1209114.9	1395259.8	1539763.4	1741455.1	1291033.6	1475108.7	1352139.6	1123641.4	1142222.9	1238800.0	1193859.3
P51859	Hepatoma-derived growth factor	Hdgf	237	26.3	4.83	676241.9	752301.5	765415.7	842641.9	711892.8	827526.3	995555.9	729323.7	794883.4	828891.9	570121.7	626948.9	663422.2	622854.2
P43406	Integrin alpha-V	Itgav	1044	115.3	5.63	353351.7	344327.4	363314.9	326614.9	349066.0	365032.1	366592.0	335005.4	370858.2	357040.3	176628.8	187833.8	171871.6	167620.5
Q9D662	Protein transport protein Sec23B	Sec23b	767	86.4	6.96	200293.7	182611.1	179964.3	178154.0	183607.3	175897.8	158058.2	185995.6	191082.1	171824.0	207244.5	220008.4	215628.2	230516.9
P10107	Annexin A1	Anxa1	346	38.7	7.37	330261.7	330133.3	323743.3	314800.7	390571.0	460654.5	446531.3	420021.1	328527.9	321041.2	461582.3	479106.1	487067.7	452655.4
Q99MR8	Methylcrotonoyl-CoA carboxylase subunit alpha, mitochondrial	Mccc1	717	79.3	7.83	520445.9	482075.8	494427.5	475744.0	522289.5	485145.4	599027.8	495712.0	511362.7	505191.1	573005.8	509122.7	509988.8	511925.9
Q9DBC3	Cap-specific mRNA (nucleoside-2'-O-)-methyltransferase 1	Cmtr1	837	95.6	7.27	469416.0	452460.6	494030.8	443494.5	541472.2	534657.6	504665.9	456145.0	481249.1	505927.6	296926.6	287554.9	292689.6	288766.4
Q8CJF7	Protein ELYS	Ahctf1	2243	247.5	6.55	172496.7	157594.0	175185.8	149423.2	181995.1	172181.0	160801.6	160146.4	154485.1	151941.4	292660.7	266491.0	269154.1	281286.6
A2AWA9	Rab GTPase-activating protein 1	Rabgap1	1064	120.7	5.25	470519.1	480222.2	526728.2	520049.7	494923.2	527946.7	477397.2	484620.1	498487.5	499003.0	228423.2	247650.0	242224.6	221123.3
E9Q411	Neuroblastoma-amplified sequence	Nbas	2356	265.6	5.95	148328.2	154435.5	161737.6	180813.7	149679.8	148154.4	135656.6	141546.1	146764.6	146352.6	129650.1	143450.6	125089.2	140889.6
P19157	Glutathione S-transferase P 1	Gstp1	210	23.6	7.87	416876.5	855481.8	362212.2	605766.0	617021.5	495523.3	367403.3	509579.7	495614.0	620890.3	657367.7	747098.9	612852.1	748747.1
P32921	Tryptophan--tRNA ligase, cytoplasmic	Wars1	481	54.3	6.89	352744.9	367255.1	335721.9	394084.2	335349.2	357502.1	339767.9	370370.4	330199.3	346923.5	379389.7	413801.5	396719.3	436902.4
Q9CZW5	Mitochondrial import receptor subunit TOM70	Tomm70	611	67.5	7.53	738273.7	850907.7	854887.1	906428.0	785672.2	762784.9	721968.5	744685.3	765863.1	793274.6	1023820.2	1215218.6	1196012.4	1226330.4
O08547	Vesicle-trafficking protein SEC22b	Sec22b	215	24.7	8.51	795895.1	869516.1	871980.2	833690.7	776806.2	906638.0	825670.2	854913.7	907367.7	834712.8	725611.4	763319.5	765434.6	814561.9
Q6ZQI3	Malectin	Mlec	291	32.3	6.05	656564.8	657387.9	679431.5	667702.4	631195.0	669856.7	645309.6	626198.2	667831.2	670535.3	791011.5	799300.5	805871.6	765824.0
Q6A0A9	Constitutive coactivator of PPAR-gamma-like protein 1	FAM120A	1112	121.6	8.92	205789.1	210597.5	188694.0	208299.1	186658.5	179569.5	203363.0	224814.0	197202.0	182223.5	229699.1	221721.5	196682.3	227677.9
P48758	Carbonyl reductase [NADPH] 1	Cbr1	277	30.6	8.31	828192.1	841422.3	836488.4	884627.7	854141.4	893899.7	968345.1	884875.6	886626.4	888866.4	902373.4	835307.2	779334.5	830779.7
Q8BI72	CDKN2A-interacting protein	Cdkn2aip	563	59.7	9.16	102949.7	114581.3	113266.0	121274.9	107667.9	107320.1	97908.5	109104.2	109630.9	114584.7	97802.0	95069.6	94273.8	92476.0
Q922P9	Putative oxidoreductase GLYR1	Glyr1	546	59.7	9.22	152452.2	174596.0	150940.4	169243.1	178213.1	188219.8	135046.8	183195.1	164812.9	179625.4	198410.7	261380.2	232315.3	227271.2
P70333	Heterogeneous nuclear ribonucleoprotein H2	Hnrnph2	449	49.2	6.3	655252.5	738258.5	580837.5	632130.4	740035.3	700869.5	790604.7	613576.8	728515.7	788135.4	147822.2	141392.0	152788.4	135910.2
Q9QWL7	Keratin, type I cytoskeletal 17	Krt17	433	48.1	5.06	1752342.3	1933125.4	1807111.0	1874363.7	4022794.5	2176408.1	1944449.5	2015001.1	2123372.3	1959319.8	1631414.2	1730384.4	1566684.7	1685786.0
Q8CC88	von Willebrand factor A domain-containing protein 8	Vwa8	1905	213.3	6.6	320843.8	321983.9	284033.3	294969.0	298066.0	330136.3	309724.7	292237.5	294548.7	307328.2	368333.4	396698.2	361220.8	368751.5
Q64331	Unconventional myosin-VI	Myo6	1265	146.3	8.85	436437.4	433109.3	371706.6	417447.2	386355.6	396035.3	441749.4	394560.6	412103.7	421029.9	564576.5	505696.9	441680.1	521291.8
Q9D7N9	Adipocyte plasma membrane-associated protein	Apmap	415	46.4	6.32	604078.2	643210.3	557866.0	602165.9	569397.6	551773.7	570143.6	500421.9	577136.4	617133.0	674694.1	679226.8	659294.9	685007.0
P35550	rRNA 2'-O-methyltransferase fibrillarin	Fbl	327	34.3	10.24	908542.7	740440.8	875403.2	751435.7	933329.0	889608.9	955608.1	856472.5	885694.8	866984.1	449601.9	424040.7	474203.3	464669.0
Q6ZPE2	Myotubularin-related protein 5	Sbf1	1867	208.6	7.12	193232.4	190197.6	192223.2	183528.7	227335.4	210726.0	195892.4	182056.0	199859.3	196259.0	197467.9	210834.8	201475.2	193588.2
P15508	Spectrin beta chain, erythrocytic	Sptb	2128	245.1	5.33	101827.4	170983.7	97676.5	124909.8	146069.8	147218.3	118919.1	113644.6	109894.7	142189.3	104727.1	103141.1	85913.3	100123.2
P21550	Beta-enolase	Eno3	434	47	7.18	186716.2	166396.4	241286.9	151577.6	257841.8	194104.0	268120.7	246899.1	205330.9	211644.4	62052.5	71890.2	71148.9	75198.5
P21107	Tropomyosin alpha-3 chain	Tpm3	285	33	4.72	1224865.1	1110434.9	1069140.6	1069637.0	1127511.7	1147186.6	1238256.0	1186469.5	1039884.5	1061126.6	501891.3	489238.4	490043.9	505272.0
Q3TKT4	Transcription activator BRG1	Smarca4	1613	181.3	8	64085.2	71676.4	68436.2	61906.1	72611.7	78981.0	75581.1	75772.8	75130.9	72907.4	85763.5	102822.6	87824.3	88349.3
Q8BH74	Nuclear pore complex protein Nup107	Nup107	926	106.7	5.43	334370.1	354311.9	346219.4	352461.3	397044.6	387212.4	356333.2	344978.2	367033.5	371316.1	263724.0	306206.3	294768.4	281758.5
Q61739	Integrin alpha-6	Itga6	1091	122.1	7.03	442076.2	414138.2	403494.5	390429.7	451171.4	442415.8	418795.2	430057.0	393885.5	414386.2	156795.2	185626.9	173765.7	160817.5
P98192	Dihydroxyacetone phosphate acyltransferase	Gnpat	678	76.8	8.13	547928.9	573436.9	578192.0	567623.2	558839.7	530374.0	501255.8	530773.3	569505.4	533275.6	210201.9	237324.9	194192.8	221740.9
G3UW82	Myosin, heavy polypeptide 2, skeletal muscle, adult	Myh2	1942	223.1	5.77	113847.3	142159.8	97188.3	110987.4	111612.7	107595.6	100773.5	135848.0	88704.3	120035.1	#N/A	#N/A	#N/A	#N/A
P50446	Keratin, type II cytoskeletal 6A	Krt6a	553	59.3	7.94	400014.4	420339.2	455021.2	359604.1	1038317.4	520753.9	429848.0	459382.8	459760.8	338757.4	313161.9	324968.0	329000.6	332583.4
Q64378	Peptidyl-prolyl cis-trans isomerase FKBP5	Fkbp5	456	50.9	7.8	322611.7	353140.4	301604.0	326918.2	333643.2	340398.1	284280.4	331845.2	322709.4	308102.7	236126.0	241923.6	248220.9	213672.0
Q9DBC7	cAMP-dependent protein kinase type I-alpha regulatory subunit	Prkar1a	381	43.2	5.35	584514.2	668014.3	650022.1	689822.3	580480.5	537057.2	468570.1	574340.5	628097.5	621810.4	814424.5	781545.0	880574.5	831769.9
P37804	Transgelin	Tagln	201	22.6	8.81	664328.7	617881.8	660583.2	623171.7	540575.4	683363.7	793525.1	954083.5	656114.5	593429.7	938458.0	914814.1	978579.5	910922.7
Q80VL1	Tudor and KH domain-containing protein	Tdrkh	560	62.1	4.94	249726.3	277037.6	273365.2	274205.9	254955.9	261198.6	210256.3	226148.2	260428.5	289791.9	184734.4	215796.3	236424.8	225428.3
Q9Z2U0	Proteasome subunit alpha type-7	Psma7	248	27.8	8.46	632104.5	536832.5	718705.1	554726.2	643200.5	614128.0	449979.2	669470.4	496654.4	493314.4	728052.7	673235.1	786102.1	670514.5
O35344	Importin subunit alpha-4	Kpna3	521	57.7	4.94	206453.5	309987.5	249448.9	273056.6	255264.9	315157.7	196873.4	254316.2	242138.0	265718.8	258635.0	285446.0	267084.2	253219.6
Q6PDG5	SWI/SNF complex subunit SMARCC2	Smarcc2	1213	132.5	5.59	311911.2	305205.5	296007.2	346219.3	303408.6	324510.6	302829.0	297687.4	292375.8	305383.7	436476.9	426012.8	443279.2	413373.8
P06909	Complement factor H	Cfh	1234	139	6.99	265811.4	251483.8	220247.0	221316.8	211785.5	226219.3	231469.1	267158.3	217170.2	247078.6	170028.2	169566.6	137667.3	155416.9
P70335	Rho-associated protein kinase 1	Rock1	1354	158.1	5.86	302257.7	307846.5	279084.6	303451.1	297784.5	329152.2	309438.0	289854.3	302865.2	314692.9	241066.6	239808.2	247305.6	251508.9
Q61074	Protein phosphatase 1G	Ppm1g	542	58.7	4.39	529090.4	594328.3	615465.6	602274.5	560114.8	586911.1	548117.6	587499.0	553908.1	561726.3	289314.8	314323.6	321999.6	310037.9
P30999	Catenin delta-1	Ctnnd1	938	104.9	6.87	358244.3	347201.1	400886.9	371918.5	398770.3	392296.4	409389.6	377311.2	358706.4	387572.4	467335.0	385299.9	411575.7	401899.2
P13020	Gelsolin	Gsn	780	85.9	6.18	660176.3	591341.5	628840.7	563949.4	621911.0	694220.6	634480.4	686904.2	603291.2	552189.3	1084370.0	918346.6	856782.3	759677.0
Q99KV1	DnaJ homolog subfamily B member 11	Dnajb11	358	40.5	6.32	455689.9	486558.4	492407.9	476698.5	431296.1	417480.7	358708.2	425444.9	467065.3	483548.3	307674.9	346170.5	325293.1	337692.5
B2RY56	RNA-binding protein 25	Rbm25	838	99.5	6.32	464513.1	548211.1	494313.0	498654.1	537336.6	504044.0	465455.8	455596.3	490109.9	508454.2	499077.9	504384.3	531288.3	511047.1
Q8CIN4	Serine/threonine-protein kinase PAK 2	Pak2	524	57.9	5.77	506592.9	444066.2	512751.5	420958.8	532795.8	497540.5	465160.6	531518.0	473252.9	449409.6	311100.0	277351.4	318723.6	251689.2
Q9DA79	Dipeptidase 3	Dpep3	493	54.2	6.48	1347531.1	1765352.8	2061665.9	2272771.5	1603104.0	1316571.5	1002645.7	1121287.6	1714859.3	1634143.8	1052928.5	1328679.2	1687405.6	1910668.7
Q925I1	ATPase family AAA domain-containing protein 3	Atad3	591	66.7	9.29	723183.8	735116.5	717135.7	727456.8	759334.6	713608.4	729494.2	656773.5	713929.9	699446.9	839040.1	868421.9	862002.5	893555.8
Q8K010	5-oxoprolinase	Oplah	1288	137.5	6.28	254300.6	224194.2	215218.0	194812.4	236172.6	270154.9	219818.9	259531.5	213161.5	202273.4	143662.2	123125.1	146171.9	135075.3
P62962	Profilin-1	Pfn1	140	14.9	8.28	1963507.4	2113483.1	2017407.5	2117604.4	1944745.3	1947638.2	1881632.5	1915200.5	1996225.6	2001932.8	4381699.9	4460271.1	4081969.9	4710784.2
P99027	60S acidic ribosomal protein P2	Rplp2	115	11.6	4.54	449091.7	586012.1	652112.7	635690.6	580654.2	466925.7	566258.0	676520.3	725965.6	480372.2	195564.8	247597.9	241029.5	249112.4
P51410	60S ribosomal protein L9	Rpl9	192	21.9	9.95	780492.9	793163.6	825508.4	779450.9	770538.1	759167.5	684574.0	776267.2	776648.4	771849.6	1086265.8	1108618.2	1083068.0	1061868.0
Q9DCX2	ATP synthase subunit d, mitochondrial	Atp5pd	161	18.7	5.69	793280.8	907841.4	1100562.8	1011116.8	1066169.7	947763.7	981616.5	1176039.4	941539.4	871731.0	1026277.6	1081406.5	1106409.4	1132412.2
Q8K0U4	Heat shock 70 kDa protein 12A	Hspa12a	675	74.8	6.77	258056.6	234246.1	260778.8	231206.9	268016.7	306454.1	333158.3	269659.7	258144.8	243872.8	301905.1	286856.4	261442.9	302162.3
Q91YH5	Atlastin-3	Atl3	541	60.5	6.1	704236.8	682311.4	620243.0	628978.5	573130.9	597570.0	660412.4	693330.3	643815.5	639680.5	693779.5	679609.3	651115.3	603393.8
P60229	Eukaryotic translation initiation factor 3 subunit E	Eif3e	445	52.2	6.04	695948.8	582323.8	727275.6	679029.3	744792.7	629672.9	553632.9	699049.5	660714.0	659552.0	367045.5	393037.0	357574.0	397668.4
Q6PCM1	Lysine-specific demethylase 3A	Kdm3a	1323	147.8	7.74	476637.7	556041.2	563255.5	588300.4	504511.9	492756.5	422157.1	469574.6	516256.1	571014.3	157147.6	172725.8	153618.6	157364.1
Q9DBG5	Perilipin-3	Plin3	437	47.2	5.62	327240.2	306518.8	293253.6	286910.7	348144.3	347877.1	364421.9	311114.6	298747.7	308605.4	483382.4	527254.8	484108.9	467497.0
Q9CQA3	Succinate dehydrogenase [ubiquinone] iron-sulfur subunit, mitochondrial	Sdhb	282	31.8	8.68	983021.3	1007070.6	1229639.6	1116350.5	894549.8	863284.0	848153.6	884437.4	940552.0	994446.4	620985.2	612133.4	710254.6	659448.4

A2A5R2	Brefeldin A-inhibited guanine nucleotide-exchange protein 2	Arfgef2	1792	202.1	6.55	366713.1	373932.0	360117.3	349569.2	355349.3	368326.2	341007.9	327834.6	356727.8	364051.2	93929.4	97955.1	93583.6	101509.4
Q99J36	THUMP domain-containing protein 1	Thumpd1	350	38.9	6.07	595960.9	525474.8	656020.0	533201.2	678181.3	672090.9	597377.2	598442.1	568201.4	526885.6	1640960.2	1470140.6	1881628.0	1444916.2
Q8BWW9	Serine/threonine-protein kinase N2	Pkn2	983	111.5	6.32	525980.6	532982.1	498395.6	507230.1	521288.3	529352.7	520768.0	500215.3	545814.5	523329.8	626149.1	570336.2	570340.3	559739.5
P62137	Serine/threonine-protein phosphatase PP1-alpha catalytic subunit	Ppp1ca	330	37.5	6.33	69532.6	69788.1	70019.3	72070.4	70866.7	69351.4	71982.5	75194.9	61615.9	60744.1	590236.9	599086.2	585409.6	586399.1
P53995	Anaphase-promoting complex subunit 1	Anapc1	1944	215.9	6.35	260479.5	286088.3	267760.7	251937.7	282460.0	290109.5	272794.0	264960.7	264174.7	273888.5	316250.2	308203.4	336332.8	293630.9
P97386	DNA ligase 3	Lig3	1015	113	8.98	294320.4	297314.1	367262.4	310019.6	357329.2	335229.8	277127.4	282477.2	309626.2	343168.6	364463.8	404491.5	425599.9	435214.4
Q91WM1	Spermatid perinuclear RNA-binding protein	Strbp	672	73.7	8.72	456143.0	509956.4	545628.2	523172.4	446859.2	459294.1	384621.3	390704.4	463835.8	509865.1	504338.0	580296.9	560019.2	610783.5
P05132	cAMP-dependent protein kinase catalytic subunit alpha	Prkaca	351	40.5	8.79	846138.1	786927.5	778715.1	788015.0	788900.5	782987.3	665868.9	731500.8	715706.7	672901.8	558768.3	481955.6	464619.8	484832.0
Q8VBW6	NEDD8-activating enzyme E1 regulatory subunit	Nae1	534	60.2	5.52	312334.2	300521.5	305116.5	287230.5	279004.2	276436.2	281148.7	282277.9	300839.2	306749.2	274188.6	276256.6	288464.1	271444.3
Q9DAR7	m7GpppX diphosphatase	Dcps	338	39	6.48	831233.9	808445.0	826428.2	767704.4	838772.2	891359.6	937064.8	805902.7	886384.6	850732.4	746702.0	654342.5	703310.2	689939.2
P62918	60S ribosomal protein L8	Rpl8	257	28	11.03	341895.8	315552.0	431080.9	354933.3	409038.9	368309.3	331431.0	416164.3	349446.2	321076.2	252590.1	260839.0	344254.9	306193.9
O88487	Cytoplasmic dynein 1 intermediate chain 2	Dync1i2	612	68.4	5.29	160321.9	160256.4	160234.4	176085.3	165796.3	172814.4	150273.5	157327.4	158079.0	165441.7	76084.3	90135.7	82942.3	92095.7
Q91YW3	DnaJ homolog subfamily C member 3	Dnajc3	504	57.4	5.85	482728.7	519607.3	521437.5	521256.7	472999.8	487191.2	383042.5	420547.1	428533.9	455200.5	346807.6	369611.1	399599.8	391971.3
P21271	Unconventional myosin-Vb	Myo5b	1818	210.4	7.11	492951.7	494194.9	478858.9	462147.3	450773.9	432677.9	422274.2	468992.1	461655.6	476820.1	538396.3	570033.7	516897.7	540794.5
P46664	Adenylosuccinate synthetase isozyme 2	Adss2	456	50	6.38	356489.6	411919.3	368395.3	365928.9	436440.5	414423.6	402975.3	377627.5	398864.5	387765.6	381436.1	371693.2	410215.7	391146.0
P45952	Medium-chain specific acyl-CoA dehydrogenase, mitochondrial	Acadm	421	46.5	8.37	764519.6	810049.8	788624.8	779352.7	813016.7	783387.6	803944.3	754803.5	792375.9	800927.2	678634.2	586704.5	672937.3	617652.9
Q8VH51	RNA-binding protein 39	Rbm39	530	59.4	10.1	393969.3	383078.6	393991.1	374442.9	375082.0	379373.9	404659.7	370480.3	425221.0	387277.5	668784.7	639361.2	581421.1	601341.1
Q60902	Epidermal growth factor receptor substrate 15-like 1	Eps15l1	907	99.2	5.02	209735.3	220001.2	209371.4	224005.9	231440.9	227427.3	207558.4	214534.5	211997.5	209724.7	249141.3	256882.7	259344.0	250932.1
E9PYG6	RAS p21 protein activator 1	Rasa1	1038	115.4	6.6	272281.0	280121.8	281010.2	289510.0	279917.5	272747.6	256278.0	251460.6	262669.9	268920.5	250980.9	269082.5	262948.8	271116.4
Q9DCJ9	N-acetylneuraminate lyase	Npl	320	35.1	8.03	648160.1	637647.2	625356.4	677804.3	589054.8	542057.5	742152.1	582186.8	640312.8	621829.2	785514.3	755024.3	758944.8	794284.6
B2RX14	Terminal uridylyltransferase 4	Tut4	1644	184.5	8.19	156196.6	155710.5	172684.4	168801.3	186829.0	186252.9	173887.2	149875.9	177816.7	163108.5	182165.2	192028.7	182922.1	168025.2
P29699	Alpha-2-HS-glycoprotein	Ahsg	345	37.3	6.51	1757806.9	1550030.2	1506092.7	1476467.2	1311691.6	1550086.1	1669053.1	1585962.1	1549560.3	1500500.0	976676.8	1230287.8	748282.6	859205.9
Q9CWK8	Sorting nexin-2	Snx2	519	58.4	5.12	343546.5	341165.0	353907.2	338531.0	340233.5	315773.6	252587.5	320767.3	314043.8	326058.4	699615.3	682687.2	645877.0	660407.8
P53395	Lipoamide acyltransferase component of branched-chain alpha-keto acid dehydrogenase complex, mitochondrial	Dbt	482	53.2	8.6	620534.1	567308.8	640871.3	566507.6	799265.6	770695.4	671947.5	666825.6	647771.5	651329.9	714959.4	639716.0	706968.5	591583.4
G3X9K3	Brefeldin A-inhibited guanine nucleotide-exchange protein 1	Arfgef1	1846	208.4	5.86	147460.5	167470.6	168083.7	148102.1	194564.0	169040.9	177964.6	149828.0	178422.1	165405.3	306494.4	307330.3	350297.9	326377.0
Q569Z6	Thyroid hormone receptor-associated protein 3	Thrap3	951	108.1	10.17	604934.4	582788.7	601638.4	600664.1	601128.2	605735.5	590754.7	572382.1	583782.1	609888.0	617830.1	729005.0	697979.8	698872.7
P24472	Glutathione S-transferase A4	Gsta4	222	25.5	7.39	3554230.8	3466076.8	4026323.0	3571374.9	4052997.1	3358120.2	4117751.5	4099445.7	3947936.7	3638922.5	5914853.2	4881754.0	6259712.3	5412054.4
Q9D7H3	RNA 3'-terminal phosphate cyclase	RtcA	366	39.2	7.9	144614.5	169657.0	173932.4	174768.4	165124.9	155008.8	146322.9	147441.3	170911.6	165130.4	378147.2	484358.7	350510.0	448106.4
Q91V76	Ester hydrolase C11orf54 homolog	MGI:191823	315	35	6.29	600083.6	579731.8	544921.3	566474.6	602643.5	608166.4	657302.6	557775.1	566667.4	573127.2	638624.5	507616.0	680001.6	556628.3
P97822	Acidic leucine-rich nuclear phosphoprotein 32 family member E	Anp32e	260	29.6	3.88	446041.8	426779.9	482063.3	435740.7	556659.2	505721.8	598400.7	476809.0	510888.8	523464.8	551310.1	524158.7	557456.6	540847.8
Q8BGH2	Sorting and assembly machinery component 50 homolog	Samm50	469	51.8	6.8	515181.3	524281.9	479462.7	523173.2	558535.7	500034.1	507655.6	504675.1	520013.9	569276.3	588965.9	562348.8	585901.8	600385.8
Q9QYB1	Chloride intracellular channel protein 4	Clic4	253	28.7	5.59	140332.9	145719.4	135704.8	138024.6	143186.7	136915.3	124020.8	139428.1	145752.3	127424.3	489684.8	498999.5	508494.7	507184.7
Q61194	Phosphatidylinositol 4-phosphate 3-kinase C2 domain-containing subunit alpha	Pik3c2a	1686	190.6	8.02	254057.8	285333.8	301412.7	265980.1	280819.7	316116.3	248738.7	244007.2	279273.9	290216.4	310856.5	317622.0	311384.3	329127.3
Q9JHQ5	Leucine zipper transcription factor-like protein 1	Lztfl1	299	34.8	5.17	279950.1	320544.0	379319.5	410232.9	279408.1	271921.8	219003.0	277508.8	300246.0	286524.9	357840.9	650102.2	422897.6	560420.0
P22907	Porphobilinogen deaminase	Hmbs	361	39.3	6.87	440866.1	493432.0	400122.2	476205.7	472149.3	508188.8	464369.4	439054.0	461962.3	462053.1	238212.3	318946.4	244490.8	273973.4
Q8R050	Eukaryotic peptide chain release factor GTP-binding subunit ERF3A	Gspt1	636	68.6	5.21	979173.6	987794.0	950211.3	987917.5	867351.0	875088.8	879003.5	971720.8	980548.7	969567.4	551115.9	558274.0	571436.5	575817.2
P32020	Non-specific lipid-transfer protein	Scp2	547	59.1	7.44	454010.8	570182.4	403362.1	550828.9	475190.4	407210.3	577725.8	473802.0	610977.1	630662.6	576919.3	604336.5	544876.9	611521.2
Q9Z1A1	PB1 domain-containing protein	Tfg	397	43	5.1	217612.7	235555.7	200609.6	206627.7	214877.2	221888.8	212334.6	212866.9	221866.6	214297.4	134263.7	172041.3	133441.3	145648.7
Q62209	Synaptonemal complex protein 1	Sycp1	993	115.9	5.85	393580.0	479917.3	500340.8	464345.5	525082.8	496183.0	345578.7	399540.8	480200.9	459377.5	272667.1	306019.3	339634.2	293526.9
Q99KP3	Lambda-crystallin homolog	Cryl1	319	35.2	5.86	199435.6	216073.2	223249.3	208678.2	266116.8	256338.6	325866.9	262437.2	216554.8	242336.4	310647.8	294757.3	289729.9	290095.6
P35979	60S ribosomal protein L12	Rpl12	165	17.8	9.42	1421929.2	1296606.9	1517103.2	1435571.1	1493957.6	1394834.1	1250226.2	1490714.6	1274717.8	1401950.1	1279764.2	1299749.7	1345852.2	1225851.3
Q9Z1R2	Large proline-rich protein BAG6	Bag6	1154	121	5.71	314849.8	309671.4	326074.2	301319.1	322490.7	308481.1	325826.4	319201.1	312431.1	322366.4	431395.8	420853.6	390691.7	453726.7
Q9Z2C8	Y-box-binding protein 2	Ybx2	360	38.2	10.95	286635.6	308908.9	331621.0	466371.8	255076.3	232113.5	193152.6	232962.1	293650.3	302825.6	146250.9	223972.2	238535.6	291598.1
Q61233	Plastin-2	Lcp1	627	70.1	5.33	328555.2	303506.2	226487.0	320765.7	242884.6	273617.6	345493.7	328059.7	247968.7	249574.4	338791.7	319181.7	287370.7	365723.2
E9PY46	Intraflagellar transport protein 140 homolog	Ift140	1464	165.8	5.91	265718.1	311892.0	278918.6	312053.5	258143.6	266618.0	276196.6	273881.5	257927.7	279977.1	218543.7	254036.9	212832.9	276880.3
Q6PAR5	GTPase-activating protein and VPS9 domain-containing protein 1	Gapvd1	1458	162.3	5.19	227281.6	243734.4	257916.5	262727.6	221230.6	220810.7	205303.9	208437.2	226162.9	241745.0	221199.3	260816.4	236577.7	242506.4
Q91YE6	Importin-9	Ipo9	1041	116	4.81	539660.2	574119.3	489038.4	577199.6	520031.6	506013.2	428767.4	521054.6	460518.0	507414.2	365788.7	386134.4	382179.3	381000.7
Q6P9R2	Serine/threonine-protein kinase OSR1	Oxsr1	527	58.2	6.43	589672.7	603849.3	611390.9	555080.8	707816.5	660954.5	650355.8	607603.5	646154.2	644256.2	407420.0	374240.5	407668.3	359510.9
Q8R180	ERO1-like protein alpha	Ero1a	464	54.1	6.54	529151.2	627694.5	569022.4	564570.2	595800.9	577950.1	506895.3	498025.6	580674.3	606402.0	479238.6	559904.1	537075.5	480118.2
Q99KC8	von Willebrand factor A domain-containing protein 5A	Vwa5a	793	87.1	6.58	451448.7	440049.1	392585.0	411016.6	440678.7	501287.9	544069.9	477363.9	433005.3	433282.1	460940.9	447259.3	398499.4	437626.1
Q9DC51	Guanine nucleotide-binding protein G(i) subunit alpha	Gnai3	354	40.5	5.69	296877.2	291751.7	295559.7	315247.1	266858.8	279346.4	258120.4	293518.4	277789.2	272810.5	74685.8	83804.6	76745.1	73832.0
Q62420	Endophilin-A1	Sh3gl2	352	39.9	5.39	485837.4	417498.0	411696.9	393167.0	425396.8	399479.3	479125.7	469056.5	400003.1	398647.1	301511.2	325118.3	275960.8	296532.7
Q922D4	Serine/threonine-protein phosphatase 6 regulatory subunit 3	Ppp6r3	844	94.6	4.58	456187.9	489444.0	437056.7	484591.6	458222.6	418861.7	412913.7	381068.7	470928.6	474761.6	331787.8	402469.4	346851.2	381480.7
Q9DB29	Isoamyl acetate-hydrolyzing esterase 1 homolog	Iah1	249	28	5.52	464552.0	491997.8	511715.1	488211.7	552376.6	494653.1	486710.8	478578.9	500837.2	502026.5	487650.4	619177.2	570709.1	562072.1
P0DP26	Calmodulin-1	Calm1	149	16.8	4.22	924858.8	1115167.8	1147692.2	1165907.0	920066.0	788575.9	830072.1	942169.9	1120189.9	999143.8	1302054.3	1564077.6	1696365.4	1614426.1
P97351	40S ribosomal protein S3a	Rps3a	264	29.9	9.73	4050154.1	4347006.5	4368966.3	4400440.5	3909617.3	3694900.1	3348354.8	4180037.1	3991668.8	4014715.4	1449784.5	1500409.0	1668006.7	1504652.4
Q99LG2	Transportin-2	Tnpo2	887	100.4	4.98	231338.9	232606.7	212586.7	247199.9	253534.4	231685.8	255429.6	256784.5	258517.1	239733.5	198061.0	188444.8	202679.8	207536.7
Q80UM7	Mannosyl-oligosaccharide glucosidase	Mogs	834	91.8	9	302162.0	341806.8	294297.6	319972.9	325196.2	345253.3	307762.2	308759.9	315625.6	336651.5	283461.4	344476.7	287625.6	290635.3
Q8K1R3	Polyribonucleotide nucleotidyltransferase 1, mitochondrial	Pnpt1	783	85.6	8.03	349139.8	291847.2	307044.0	333769.3	286322.9	271303.2	330335.6	293980.8	347990.2	327085.7	299786.5	304303.3	312076.2	305537.7
Q8VEH6	COBW domain-containing protein 1	Cbwd1	393	43.7	4.89	88136.0	107014.4	106240.0	101777.7	109367.4	100797.6	71915.1	83695.6	100113.9	97580.0	178274.3	202731.1	174031.0	193316.5
Q8VD65	Phosphoinositide 3-kinase regulatory subunit 4	Pik3r4	1358	152.5	7.12	182370.8	226789.1	176289.9	179847.0	199777.6	212081.4	192407.5	169003.9	195440.4	189891.8	229207.7	238015.2	215288.2	228004.9
Q9D4H1	Exocyst complex component 2	Exoc2	924	103.9	7.18	329670.8	310739.6	309543.6	314052.4	315946.4	317335.5	306236.9	310243.8	322845.3	310839.5	425803.5	395059.3	379771.5	401924.0
Q9Z1D1	Eukaryotic translation initiation factor 3 subunit G	Eif3g	320	35.6	5.9	391057.3	351124.7	407841.2	363773.8	390241.2	357829.8	323970.5	370751.7	353081.0	394572.6	260867.1	244422.1	238821.5	257103.7
Q9D8Y0	EF-hand domain-containing protein D2	Efhd2	240	26.8	5.06	1324986.4	1377905.4	1358393.3	1381218.5	1290763.5	1273942.3	1699878.1	1536572.6	1599232.6	1375632.6	440163.5	361106.3	497287.4	343493.3
P83917	Chromobox protein homolog 1	Cbx1	185	21.4	4.93	227905.8	237778.9	234361.4	217362.9	279308.6	289080.5	259258.0	218858.0	233276.9	257214.5	167765.5	153047.7	157723.3	155669.3
Q8BFZ3	Beta-actin-like protein 2	Actbl2	376	42	5.49	36142.2	41206.9	50853.9	40657.7	34674.1	39089.6	38370.4	41855.7	35121.5	39428.3	#N/A	#N/A	#N/A	#N/A
Q9D1G1	Ras-related protein Rab-1B	Rab1b	201	22.2	5.73	297000.5	275031.6	313262.4	275638.7	329385.5	305560.9	317006.1	320251.6	303499.7	294753.3	158507.7	155176.6	162010.6	133682.4
O55137	Acyl-coenzyme A thioesterase 1	Acot1	419	46.1	6.58	91811.3	107607.3	82168.0	80499.5	94453.2	102612.0	97393.0	85350.0	103020.4	105133.7	602576.9	515847.3	565829.2	508505.4
P70206	Plexin-A1	Plxna1	1894	211	6.9	344221.2	328540.9	321261.4	342915.6	314559.0	324531.0	308447.0	310374.5	322718.4	367702.9	455465.2	552702.3	479308.1	487658.6
Q8VHE0	Translocation protein SEC63 homolog	Sec63	760	87.8	5.38	557106.1	589259.6	604719.1	602969.4	566149.2	524878.0	488383.4	527545.7	586267.1	624464.7	389231.3	406681.3	404955.8	418061.0
Q9R190	Metastasis-associated protein MTA2	Mta2	668	75	9.67	389066.3	381751.5	349633.5	371892.3	383251.6	380238.4	365053.3	362317.5	376785.1	417735.7	652198.1	694864.5	661246.7	669804.9
Q3UW53	Protein Niban 1	Niban1	926	102.6	4.78	940481.0	891874.1	756503.3	823295.5	837337.4	779954.4	865174.8	877103.3	747808.6	767061.6	182519.6	185130.3	149358.1	160248.3
Q99L47	Hsc70-interacting protein	St13	371	41.6	5.26	1100903.0	1446698.1	1491254.2	1706482.8	1061327.0	910789.9	762079.6	1028864.2	1243574.5	1335475.1	1496307.1	2165139.0	2336814.1	2596884.7
Q9EP71	Ankycorbin	Rai14	979	108.8	6.27	132205.6	151940.1	142254.6	150411.1	149185.6	146151.2	149901.8	134088.3	148443.3	136453.4	257722.3	275022.3	245502.4	281590.5
Q9CQM9	Glutaredoxin-3	Glrx3	337	37.8	5.59	691932.1	786227.4	760617.3	823172.3	657037.2	903314.7	870084.7	691987.1	748143.2	721131.6	993577.5	1159298.0	968500.6	1065942.1
Q80TV8	CLIP-associating protein 1	Clasp1	1535	169.1	9.03	156433.5	159454.4	149627.7	156572.6	158732.4	158354.9	155661.0	132526.0	153885.2	163828.2	178203.5	168124.1	160058.8	171558.7
P70288	Histone deacetylase 2	Hdac2	488	55.3	5.91	685695.8	633470.2	661799.8	600694.6	673399.1	687343.8	654272.8	643747.7	670972.2	708232.5	291324.4	303622.2	280721.0	271024.5
Q9JHS4	ATP-dependent Clp protease ATP-binding subunit clpX-like, mitochondrial	Clpx	634	69.2	7.71	284283.0	258436.5	277916.9	271971.4	283612.8	259443.8	245541.2	248814.5	284351.7	282143.2	210492.7	250184.8	210011.8	221297.3
P97313	DNA-dependent protein kinase catalytic subunit	Prkdc	4128	471.2	7.12	210119.7	218646.5	205576.4	224074.3	213177.4	204685.4	197302.0	193511.4	215760.0	222117.8	128287.1	144846.3	136721.0	138456.2
Q9CQ65	S-methyl-5'-thioadenosine phosphorylase	Mtap	283	31	7.14	322172.0	329279.7	293417.4	309959.7	296251.1	304988.7	341903.7	319387.9	328934.4	338655.0	386490.3	370603.8	364181.6	352704.0
B2RY04	Dedicator of cytokinesis protein 5	Dock5	1868	214.3	7.55	335160.4	303862.3	301465.4	298839.5	349526.8	352333.4	318515.3	332845.7	347462.0	321925.1	151013.2	160738.2	141975.3	133800.8
Q60865	Caprin-1	Caprin1	707	78.1	5.25	739732.4	794845.8	816761.7	807557.9	718703.8	703751.1	685371.9	789349.3	752661.1	758344.7	975639.0	986738.7	968794.5	1016037.3
Q7TNL5	Serine/threonine-protein phosphatase 2A 56 kDa regulatory subunit	Ppp2r5d	595	69.1	7.96	137372.4	135846.4	136328.6	112236.4	203084.1	139756.5	146295.4	120774.6	133966.2	148879.5	149555.4	144671.0	127663.1	139895.5
Q99MN9	Propionyl-CoA carboxylase beta chain, mitochondrial	Pccb	541	58.4	7.66	390451.6	357309.6	338105.6	368006.3	350363.4	351136.7	348919.4	353251.6	317752.0	321241.0	362640.4	428278.1	328525.9	405686.5
O08756	3-hydroxyacyl-CoA dehydrogenase type-2	Hsd17b10	261	27.4	8.41	424758.8	446567.3	450525.4	472900.9	449871.1	440216.2	422691.4	400605.7	469347.6	419272.5	110634.7	110619.0	108340.8	104297.3
P61982	14-3-3 protein gamma	Ywhag	247	28.3	4.89	279575.0	291067.7	268006.4	288692.0	312197.3	355298.8	306773.3	313661.9	300312.3	270461.5	102180.2	100916.8	108897.8	91941.5
O35074	Prostacyclin synthase	Ptgis	501	57	6.73	857906.6	837257.5	754859.0	717824.1	680858.8	845823.6	953122.6	878525.6	765415.9	742345.7	502492.1	516257.6	493896.3	464177.5
P04186	Complement factor B	Cfb	761	85	7.37	331449.2	360717.1	310709.9	331660.2	256596.2	269325.0	367766.7	371196.1	339635.4	345920.3	94207.8	109554.1	77973.6	78711.7
Q91YP2	Neurolysin, mitochondrial	Nln	704	80.4	6.44	778841.8	827845.3	761266.2	814108.7	813030.1	779504.6	770519.5	731504.9	805751.7	822933.9	881644.0	914005.1	864800.0	882681.5
Q8K2Q9	Shootin-1	Shtn1	631	71.3	5.44	206530.0	186115.6	186188.4	168265.4	210472.7	184900.0	209989.0	201407.9	196749.9	189458.0	421007.9	364192.2	396677.8	363008.0
P28738	Kinesin heavy chain isoform 5C	Kif5c	956	109.2	6.19	225664.9	226292.9	223746.0	214154.0	269501.9	255897.2	227735.3	231528.3	240145.1	235846.7	141231.7	131548.9	138784.1	120986.6
Q9DBS5	Kinesin light chain 4	Klc4	619	68.6	6.09	257907.9	248059.0	261880.5	248210.4	273770.9	270257.8	275923.4	251588.2	251248.1	247487.5	204148.3	196960.1	189931.9	199156.3

P35487	Pyruvate dehydrogenase E1 component subunit alpha, testis-specific form, mitochondrial	Pdha2	391	43.4	8.5	543341.6	667526.5	719226.1	869813.0	442838.4	356370.3	290236.2	440744.8	557486.8	663521.0	393108.3	444113.6	490702.0	569190.1
Q8CCJ3	E3 UFM1-protein ligase 1	Ufl1	793	89.5	6.67	381263.9	396816.2	372007.8	409331.1	351427.4	344139.1	349897.4	369097.5	373866.5	399751.5	336911.5	376886.1	342293.5	378015.4
P61202	COP9 signalosome complex subunit 2	Cops2	443	51.6	5.53	765895.1	750083.6	853791.1	751746.5	891668.2	851300.9	710772.8	786821.4	782355.2	762049.0	625924.5	557278.4	748143.5	631574.9
Q9JMH6	Thioredoxin reductase 1, cytoplasmic	Txnrd1	613	67	7.44	194454.7	216824.8	205210.9	194577.9	203219.5	198601.8	207700.9	195944.9	195679.2	213445.4	411986.8	411472.4	387686.3	394952.4
Q60848	Lymphocyte-specific helicase	Hells	821	95.1	8	193505.4	256479.7	215919.7	207215.5	243353.4	267164.9	202329.0	204380.2	222373.0	234240.4	241079.9	238590.0	262963.3	214101.5
O88990	Alpha-actinin-3	Actn3	900	103	5.45	94916.9	109191.9	76836.9	100494.9	92869.0	83606.1	85057.5	114412.0	87833.0	83576.3	131131.2	158471.5	104026.8	152620.9
P47199	Quinone oxidoreductase	Cryz	331	35.2	8.07	547330.6	543557.8	504398.8	535501.4	542131.2	565029.8	606067.3	518916.2	537188.0	518510.5	262570.0	300720.4	260533.4	297984.6
O08677	Kininogen-1	Kng1	661	73.1	6.54	1159774.8	1038344.1	827054.4	851829.6	626096.7	835004.4	1043828.0	898156.6	997882.4	1106859.9	1053984.8	938849.1	869607.3	828783.5
Q62393	Tumor protein D52	Tpd52	224	24.3	4.72	95867.3	105780.7	94079.2	101667.4	99529.9	97714.0	92706.2	114322.2	96005.9	91848.5	254373.8	291612.4	251801.7	283972.4
Q99K48	Non-POU domain-containing octamer-binding protein	Nono	473	54.5	8.95	470336.9	505679.0	425726.7	495687.9	476905.5	522541.9	703471.6	432812.3	501856.9	604255.3	495665.5	475604.0	459495.7	404586.6
Q80X68	Citrate synthase	Csl	466	52.3	8.79	114828.4	137255.4	128691.5	172299.6	82317.3	91067.9	78966.0	81276.7	107219.2	129794.3	177449.0	215541.9	227198.6	272790.1
P47758	Signal recognition particle receptor subunit beta	Srprb	269	29.6	9.28	246170.7	236652.1	253247.9	242450.2	227123.6	212795.3	187747.2	222047.9	229893.6	224696.0	338605.7	368564.7	332651.7	356124.6
Q61823	Programmed cell death protein 4	Pdcd4	469	51.7	5.16	246863.9	253514.5	240441.2	241332.0	260486.2	250781.6	243230.5	222277.4	255685.6	245305.6	299677.4	328375.9	375691.5	391496.4
Q8K297	Procollagen galactosyltransferase 1	Colgalt1	617	71	7.28	585233.5	587927.1	479161.7	509414.3	446701.1	402535.1	330604.0	514924.7	430677.9	460855.8	1197670.0	1032626.3	882240.1	891565.1
Q64525	Histone H2B type 2-B	Hist2h2bb	126	13.9	10.32	7541778.9	7950253.8	7703642.9	6445466.8	9652096.3	10442238.0	9713473.8	6631756.3	8822035.8	8901946.8	4040269.2	4261715.4	4655838.7	3285854.2
Q6ZQ73	Cullin-associated NEDD8-dissociated protein 2	Cand2	1235	135.5	5.58	173345.5	190096.5	175015.6	182162.2	194247.8	198819.4	163737.0	167669.6	170138.3	174179.9	137036.6	150858.7	136880.2	132990.0
Q6ZQ88	Lysine-specific histone demethylase 1A	Kdm1a	853	92.8	6.52	197496.0	212155.9	196675.6	220749.5	203806.7	214816.1	212566.0	200273.2	187347.2	189674.2	41621.3	50018.2	46870.3	50149.8
P97384	Annexin A11	Anxa11	503	54	7.66	916156.0	917229.5	953603.1	887622.5	1059002.9	1022246.7	1007210.3	906641.2	948044.2	999495.5	1015294.3	1019937.3	1022394.6	992473.2
Q02819	Nucleobindin-1	Nucb1	459	53.4	5.07	228092.1	297574.0	266523.2	303469.0	284211.9	255747.1	209151.0	233230.1	242152.0	253353.3	206682.6	214230.5	245753.9	227479.1
Q8C2Q3	RNA-binding protein 14	Rbm14	669	69.4	9.67	687835.9	756933.6	886573.0	834065.0	794206.8	732040.5	707138.7	795084.6	761065.0	791401.7	911024.7	958799.0	971169.9	995770.7
Q8BX02	KN motif and ankyrin repeat domain-containing protein 2	Kank2	843	90.2	5.55	179580.0	167141.9	177384.9	157694.2	212208.0	224774.1	233740.7	183698.2	179962.4	177507.9	148884.7	143566.3	150465.6	167894.9
Q8BVE3	V-type proton ATPase subunit H	Atp6v1h	483	55.8	6.61	521265.5	611275.0	552346.4	637551.3	517817.7	552794.1	539074.0	501680.4	524475.0	542858.6	698030.2	671126.2	687566.5	690212.3
Q9DCW4	Electron transfer flavoprotein subunit beta	Etfb	255	27.6	8.1	1779577.9	1727169.3	1540427.2	1707583.0	1668308.5	1644288.5	1890157.6	1723935.3	1679177.2	1582799.9	1116489.0	1168588.5	919900.0	1117047.0
Q80XI3	Eukaryotic translation initiation factor 4 gamma 3	Eif4g3	1579	174.8	5.53	270425.8	332163.4	275455.2	333870.6	242266.6	255518.4	221444.0	245259.0	258234.7	275585.1	346313.2	395163.0	382239.2	455640.3
Q9WVJ2	26S proteasome non-ATPase regulatory subunit 13	Psmd13	376	42.8	5.71	1224831.3	1195791.7	1171155.5	1218353.4	1128225.8	1107600.8	988152.5	1038482.5	1118618.3	1140923.4	762758.7	783635.1	693601.1	844025.4
Q9Z0N2	Eukaryotic translation initiation factor 2 subunit 3, Y-linked	Eif2s3y	472	51.1	8.5	779231.9	702948.5	847426.4	706084.9	809825.0	756733.4	754734.0	756784.9	777611.8	783165.4	1101777.0	1000926.5	1066089.4	938462.8
P60824	Cold-inducible RNA-binding protein	Cirbp	172	18.6	9.61	567406.0	499448.7	906725.5	606788.8	909342.2	661426.2	522543.5	634269.9	713451.0	819252.3	592786.8	574498.5	892688.1	628225.5
P35278	Ras-related protein Rab-5C	Rab5c	216	23.4	8.41	838553.4	852404.2	826368.1	829152.3	855387.6	875577.5	853087.5	820122.3	867390.6	859028.3	708109.8	693832.6	658903.8	697020.4
P63242	Eukaryotic translation initiation factor 5A-1	Eif5a	154	16.8	5.24	2432483.1	2554566.6	2366591.4	2584466.1	2305059.9	2164444.4	2058400.4	2117963.6	2536640.6	2641169.0	1550590.4	2257924.1	1408628.2	1958946.6
Q9D8U8	Sorting nexin-5	Snx5	404	46.8	6.62	740286.6	761627.3	697068.5	767640.8	768842.7	760939.4	723668.4	755181.6	757808.2	716850.9	437825.1	468843.9	408202.7	476396.3
Q6P1F6	Serine/threonine-protein phosphatase 2A 55 kDa regulatory subunit B alpha isoform	Ppp2r2a	447	51.7	6.2	540068.5	532969.7	581238.7	524480.4	559461.8	566048.4	561446.8	551435.7	597655.7	599053.1	585650.6	592559.7	527537.2	586933.5
O09061	Proteasome subunit beta type-1	Psmb1	240	26.4	7.81	818610.9	760480.0	712827.1	855068.0	621226.6	680022.2	631381.4	686785.4	716121.6	743461.9	1242350.9	1216265.7	1250501.9	1282683.4
Q99KP6	Pre-mRNA-processing factor 19	Prpf19	504	55.2	6.61	1029140.3	1059877.1	1170247.6	1064008.9	1260904.6	1229149.8	1195426.3	1278309.8	1209990.3	1174616.3	1377654.7	1504823.3	1436100.6	1456516.0
P99026	Proteasome subunit beta type-4	Psmb4	264	29.1	5.64	397939.5	486419.9	418990.3	435745.3	428855.1	408129.1	365623.5	403693.0	398835.2	444966.7	395987.7	393337.6	417254.1	432766.7
Q99LE6	ATP-binding cassette sub-family F member 2	Abcf2	628	71.7	7.05	286422.5	277292.1	254836.4	277001.3	248729.6	256048.4	247703.7	243016.8	272109.7	284157.2	356269.7	392124.2	375925.3	372675.5
Q64213	Splicing factor 1	Sf1	653	70.4	8.98	206924.0	239835.2	212421.2	219196.1	234947.1	231957.9	198035.7	203766.6	210727.3	223370.0	185449.5	178685.0	173600.2	186716.1
O70423	Membrane primary amine oxidase	Aoc3	765	84.5	6.42	763556.9	755710.1	717992.9	746336.3	828028.0	848236.0	804705.1	764987.5	806735.0	761574.5	463342.1	400677.6	322517.8	349764.2
Q99K51	Plastin-3	Pls3	630	70.7	5.62	134432.7	122054.8	123148.0	141271.4	123673.8	153410.8	173560.3	162497.8	128406.1	137409.7	126007.4	116508.1	121544.0	120525.9
Q9CXW3	Calcyclin-binding protein	Cacybp	229	26.5	7.87	904580.3	1044617.2	1139390.8	1107923.7	1029132.0	920279.0	784990.4	905547.8	1074869.5	1042376.4	657797.4	750822.8	727276.6	732898.4
Q9JLQ0	CD2-associated protein	Cd2ap	637	70.4	6.38	320125.0	323086.4	285242.7	286818.4	304283.7	313445.7	314584.6	297738.0	321228.2	324818.5	613939.1	603168.8	586225.7	572604.1
Q99NH0	Ankyrin repeat domain-containing protein 17	Ankrd17	2603	274	6.52	227596.2	232900.5	235328.6	233713.7	243303.0	243539.9	201896.9	232522.9	219307.8	215740.5	165502.6	191697.8	178885.2	189871.1
Q9CX34	Protein SGT1 homolog	Sugt1	336	38.1	5.45	662612.7	555605.3	591090.5	544641.3	556012.9	501804.2	593817.0	532234.5	548221.1	560242.7	185158.7	200056.2	190397.8	215692.3
Q9CXT8	Mitochondrial-processing peptidase subunit beta	Pmpcb	489	54.6	6.99	323921.9	320609.7	290211.5	339732.8	311589.1	303649.3	281858.8	289378.0	308814.4	321835.4	303878.6	288505.8	291578.4	303384.8
Q8CHP8	Glycerol-3-phosphate phosphatase	Pgp	321	34.5	5.35	707413.4	718687.7	784308.2	843269.9	606700.3	582267.7	576225.8	635088.4	718354.6	703120.3	634798.2	665844.8	662650.8	717557.7
Q9JI10	Serine/threonine-protein kinase 3	Stk3	497	56.8	4.98	163995.4	159220.6	153986.5	161433.8	169557.5	187612.7	161817.3	170360.9	150827.6	173310.2	335853.0	277693.7	244542.2	262309.1
P57759	Endoplasmic reticulum resident protein 29	Erp29	262	28.8	6.15	1213860.7	1349492.2	1178666.7	1409097.1	1113995.1	1026280.9	954588.7	1234374.3	1140039.2	1095728.0	1090140.3	1220372.5	1153098.7	1227624.7
Q99JB2	Stomatin-like protein 2, mitochondrial	Stoml2	353	38.4	8.87	115229.4	111784.9	108120.2	117815.3	108491.8	117898.1	107854.0	113967.7	107921.1	99843.6	293539.1	277060.4	310725.3	290102.2
Q9D1D4	Transmembrane emp24 domain-containing protein 10	Tmed10	219	24.9	6.7	711405.1	734324.4	723070.9	729361.0	691095.6	720689.5	657693.1	685676.4	704392.5	686177.4	471624.5	579674.7	509038.7	582461.8
O70251	Elongation factor 1-beta	Eef1b	225	24.7	4.69	670252.2	867789.1	616568.9	698721.9	635561.6	611640.2	511107.7	751710.7	626255.5	650340.7	521308.3	488113.3	489814.7	524300.0
O08529	Calpain-2 catalytic subunit	Capn2	700	79.8	4.96	298181.3	283884.2	244397.4	268153.5	261375.0	329698.8	310793.2	293769.6	263587.5	272125.7	247524.9	263250.3	230532.0	221958.4
Q8BH95	Enoyl-CoA hydratase, mitochondrial	Echs1	290	31.5	8.48	1306380.7	1191803.0	1237892.9	1179109.0	1277005.1	1208003.9	1322438.1	1241184.5	1198203.0	1192042.9	1652080.2	1457167.9	1415083.9	1410707.2
Q6PGC1	ATP-dependent RNA helicase DHX29	Dhx29	1365	153.9	7.94	288300.1	286704.6	295732.3	271707.0	295279.5	300745.6	252535.6	296249.3	291222.2	289299.4	370068.9	365719.7	352345.5	345212.2
Q9EP69	Phosphatidylinositide phosphatase SAC1	Sacm1l	587	66.9	7.3	611528.3	594385.5	520938.5	575808.8	574670.2	552897.7	529306.2	537848.9	573647.9	582428.4	531782.1	528815.6	476135.8	532875.5
Q9WTX5	S-phase kinase-associated protein 1	Skp1	163	18.7	4.54	1035559.9	1076455.1	1189702.6	1092651.3	1134507.6	1052330.7	943834.4	1139317.0	1158372.1	1023852.0	860367.2	1079271.8	1046028.3	1125003.2
Q6ZPR5	Sphingomyelin phosphodiesterase 4	Smpd4	823	93.2	8.13	318910.5	345909.8	337621.9	324003.8	355720.1	356159.4	290668.2	301535.6	328007.4	322148.1	407528.8	416055.7	419183.6	415786.4
Q9DAW6	U4/U6 small nuclear ribonucleoprotein Prp4	Prpf4	521	58.3	7.28	447128.3	479628.3	480950.0	462204.6	469675.8	457320.0	442360.9	427594.6	491680.3	479918.7	448038.0	474817.2	467573.1	429007.9
P28653	Biglycan	Bgn	369	41.6	7.27	273823.6	290663.2	250615.1	270780.4	278444.6	276438.6	264386.6	282853.3	259743.0	295373.6	812760.8	838807.6	781637.3	812084.3
Q9D4H8	Cullin-2	Cul2	745	86.8	7.01	240905.2	277726.0	234785.7	265241.7	229690.7	271843.6	268707.6	222942.4	271364.4	290444.0	252472.8	259699.7	298563.7	292696.8
Q00915	Retinol-binding protein 1	Rbp1	135	15.8	5.25	902421.8	776919.7	826337.7	727120.5	904157.9	1049000.7	1373094.6	964182.7	943007.9	924977.3	751628.3	701177.8	697318.2	629071.0
Q3UHD6	Sorting nexin-27	Snx27	539	61	6.4	517959.2	593671.1	600356.8	595743.9	510700.9	481326.1	415975.7	494027.6	562884.8	575949.0	836563.5	971014.6	930353.6	951806.1
P35585	AP-1 complex subunit mu-1	Ap1m1	423	48.5	7.3	320688.9	362540.2	382794.7	359975.8	372813.6	377175.1	299900.3	334838.8	359349.6	361934.1	211683.6	232570.0	225600.3	224885.0
Q9Z0R4	Intersectin-1	Itsn1	1714	194.2	7.91	233913.9	242560.0	218209.9	213491.0	242225.7	236891.4	256287.9	239459.0	225593.6	251882.1	187835.3	195422.8	205288.3	192568.3
Q9Z2U1	Proteasome subunit alpha type-5	Psma5	241	26.4	4.79	622993.2	609731.5	650564.9	657026.8	664170.4	625251.7	604521.8	617659.5	656089.5	619868.3	453326.6	446084.1	473073.4	488474.7
Q68FG2	Spectrin beta chain	Sptbn2	2388	270.8	5.86	156031.0	165033.6	160802.8	147083.4	159959.3	172667.9	149046.9	156291.8	149714.7	151176.0	126959.1	131709.8	123175.0	115280.0
Q9CQ19	Myosin regulatory light polypeptide 9	Myl9	172	19.8	4.92	2209119.5	2417183.5	2209302.7	2450340.5	2710708.9	2664645.0	2598101.1	3372652.0	2484045.3	2491343.2	513501.4	450454.2	527842.1	457423.9
Q6ZWV3	60S ribosomal protein L10	Rpl10	214	24.6	10.08	208096.8	246084.1	236839.6	231425.2	203197.0	197555.6	225620.5	187200.3	226708.9	249690.4	1721060.4	1711269.6	1747211.1	1633746.6
P63085	Mitogen-activated protein kinase 1	Mapk1	358	41.2	6.98	1198011.5	1116626.9	1029983.5	1038059.5	1219088.3	1228142.0	1185984.0	1063443.4	1179601.2	1181599.4	884089.8	935107.6	810171.6	890626.9
Q6PDM2	Serine/arginine-rich splicing factor 1	Srsf1	248	27.7	10.36	1237469.9	1362532.4	1431868.3	1372595.2	1362466.8	1313798.8	1342180.3	1285102.8	1369312.9	1373357.1	1191566.1	1223513.7	1307410.3	1264001.3
O08663	Methionine aminopeptidase 2	Metap2	478	52.9	5.82	356956.1	376895.6	350243.9	365894.3	347529.8	337368.2	313248.7	337846.6	335642.8	351814.2	424613.3	430383.7	408971.6	438705.7
O70252	Heme oxygenase 2	Hmox2	315	35.7	5.87	515174.8	539160.2	546043.1	541810.1	578823.1	613050.0	504311.9	464601.5	565402.0	559619.9	585589.8	647741.3	673307.3	661082.0
Q6IFX2	Keratin, type I cytoskeletal 42	Krt42	452	50.1	5.16	549586.0	607109.8	492907.3	595325.0	1022608.2	632841.6	575196.0	539078.8	627764.9	582529.3	442688.1	461839.5	392731.5	430164.5
Q64010	Adapter molecule crk	Crk	304	33.8	5.55	216763.8	202948.9	225158.2	219034.4	243261.7	254144.7	263657.8	221013.9	229643.8	216559.2	180959.2	181663.6	153891.6	171893.9
Q8VEH3	ADP-ribosylation factor-like protein 8A	Arl8a	186	21.4	7.77	422752.3	630432.3	398225.8	400772.6	446702.1	401732.6	427542.0	406129.6	388541.6	424905.7	670280.9	942816.5	603902.2	799103.8
Q9EP89	Serine beta-lactamase-like protein LACTB, mitochondrial	Lactb	551	60.7	8.9	424639.9	418523.1	427422.2	429303.2	383099.7	378393.2	405171.3	415475.0	380666.1	357238.3	177291.4	170226.1	180520.5	161466.8
Q80Y81	Zinc phosphodiesterase ELAC protein 2	Elac2	831	92.7	7.34	556032.6	526899.2	563645.2	552001.5	567403.4	531111.8	527078.5	534751.4	534504.3	562629.4	676035.9	678194.9	752778.0	641462.0
P47757	F-actin-capping protein subunit beta	Capzb	277	31.3	5.74	1273652.8	1211551.1	1241446.2	1216643.7	1212214.0	1169357.9	1264938.9	1324861.1	1236186.4	1233956.3	773191.6	827171.0	847420.0	839784.7
P97449	Aminopeptidase N	Anpep	966	109.6	5.9	193546.9	175371.4	155255.2	185815.2	186721.3	214769.6	258864.0	195027.2	164323.0	194680.2	195777.2	185899.5	212965.1	199498.7
Q8K1X1	WD repeat-containing protein 11	Wdr11	1223	135.9	7.11	475936.7	504176.7	513827.5	520069.3	485687.7	456627.2	420078.9	448640.3	493380.9	516874.5	322381.0	385818.5	432187.9	381695.4
Q9WUM4	Coronin-1C	Coro1c	474	53.1	7.08	743374.4	750814.4	724496.6	717616.8	769074.8	759715.5	730722.2	695824.5	758802.2	777339.3	548314.2	537718.9	559780.9	525842.4
Q8K2Z4	Condensin complex subunit 1	Ncapd2	1392	155.6	6.38	281202.2	304292.5	278827.3	295026.1	308325.5	302344.0	272159.2	263360.6	286552.7	309140.9	255799.6	264950.3	223512.9	240778.8
P63154	Crooked neck-like protein 1	Crnkl1	690	83.4	6.93	408560.5	398827.5	418132.6	412324.7	418122.8	404758.5	373978.5	390461.4	416906.7	427168.3	298826.1	279091.2	305006.1	280428.0
Q78IK4	MICOS complex subunit Mic27	Apool	265	29.2	9.31	506370.1	521138.5	460614.9	458578.3	518985.5	514035.9	513372.2	472470.4	497741.0	515501.0	517458.9	509375.5	506208.2	495442.4
Q9EQQ9	Protein O-GlcNAcase	Oga	916	103.1	4.92	139352.3	132300.0	158444.1	142403.8	197289.6	186963.3	182440.1	134886.5	154749.0	170864.0	152633.5	154580.5	155498.5	149900.8
P31230	Aminoacyl tRNA synthase complex-interacting multifunctional protein 1	Aimp1	310	34	8.35	683665.6	641628.2	670996.1	637201.3	636228.7	612838.2	572173.5	597415.4	645844.3	655580.9	446421.5	425514.4	458288.4	413997.1
Q7TPD0	Integrator complex subunit 3	Ints3	1041	117.9	5.8	283861.1	258411.8	263506.6	266655.7	290321.2	264883.5	274491.8	264445.9	270898.5	265411.6	268471.1	234068.5	235222.6	222023.9
Q8CAY6	Acetyl-CoA acetyltransferase, cytosolic	Acat2	397	41.3	7.5	52879.8	58927.1	62656.5	59492.2	58311.8	67190.7	55164.7	60404.1	59583.1	50664.6	68990.9	86610.1	68631.8	71828.9
Q9CVB6	Actin-related protein 2/3 complex subunit 2	Arpc2	300	34.3	7.36	856970.0	908984.4	781653.7	922065.0	844535.0	839729.2	821863.6	831776.7	872628.2	865786.4	1339231.8	1389917.3	1218268.7	1336135.4
P53810	Phosphatidylinositol transfer protein alpha isoform	Pitpna	271	31.9	6.37	889355.8	811665.2	899905.8	851808.7	881448.0	922978.6	831753.3	879708.4	871396.7	795654.6	834518.0	764951.2	796066.6	839359.3
P70302	Stromal interaction molecule 1	Stim1	685	77.5	6.54	902788.0	909865.0	796792.6	832789.9	786638.4	741841.8	684203.7	806878.3	794800.2	806440.6	831866.9	892503.9	719535.4	741600.6
Q9JJK2	LanC-like protein 2	Lancl2	450	50.7	7.28	209049.3	217932.2	188214.5	219483.6	181392.6	177302.1	187573.5	174171.2	196284.8	212614.9	241069.9	292860.3	308986.5	343753.5
Q62141	Paired amphipathic helix protein Sin3b	Sin3b	1098	126.3	7.06	218312.6	202443.3	213242.7	202700.4	196767.2	193657.5	195506.2	204504.3	215259.2	211160.0	157481.8	139690.5	171307.6	162488.6
Q60737	Casein kinase II subunit alpha	Csnk2a1	391	45.1	7.74	1082992.2	1216597.1	1124307.3	1133620.2	1193149.8	1185727.5	1139420.7	1163084.2	1106299.7	1096996.5	847059.0	706078.6	754050.9	917307.3

Q64518	Sarcoplasmic/endoplasmic reticulum calcium ATPase 3	Atp2a3	1038	113.6	5.85	65754.9	57704.1	60422.6	57129.9	68927.8	73541.7	79949.8	72510.6	58149.6	70268.4	73980.8	72039.0	70822.9	68834.4
Q8C570	mRNA export factor	Rae1	368	40.9	7.83	530266.7	556141.5	627691.0	545612.2	718483.2	611523.1	568361.7	614253.3	657342.0	601541.8	934372.9	970477.9	1007450.3	958546.3
Q9JL35	High mobility group nucleosome-binding domain-containing protein 5	Hmgn5	406	45.3	4.37	303739.8	243565.4	306401.8	243753.6	344835.6	345089.8	404684.8	284817.2	330250.6	344580.9	217757.9	210114.0	197859.8	188412.6
Q9CZC8	Secernin-1	Scrn1	414	46.3	4.79	522602.7	514533.5	488361.6	483922.1	489773.5	473936.1	462156.2	504754.3	514152.8	520991.9	539540.4	471993.4	487762.1	451404.1
E9PVX6	Proliferation marker protein Ki-67	Mki67	3177	350.7	9.72	221729.3	254237.3	235874.2	241577.2	253379.4	241769.3	225715.2	232003.0	230553.2	221443.2	160693.0	163333.6	148336.2	150077.3
Q61171	Peroxiredoxin-2	Prdx2	198	21.8	5.41	6061638.0	5051846.7	5694730.8	5195795.8	5908170.4	6289777.8	6527975.0	6379105.7	5889320.6	5558924.0	6133145.1	5076351.2	5780339.5	5033050.1
O35381	Acidic leucine-rich nuclear phosphoprotein 32 family member A	Anp32a	247	28.5	4.07	1100614.9	983222.9	1249940.3	976108.8	1459524.1	1456921.6	1586442.2	1291100.6	1208352.1	1209702.3	1448478.4	1220693.1	1530144.1	1493532.2
Q9WUM5	Succinate--CoA ligase [ADP/GDP-forming] subunit alpha, mitochondrial	Suclg1	346	36.1	9.39	1333948.9	1273690.9	1309435.6	1402087.0	1248570.0	1148228.7	1319457.8	1305261.8	1292554.5	1287436.0	1133922.2	1015202.1	1121105.6	1204521.4
P57776	Elongation factor 1-delta	Eef1d	281	31.3	5.02	446852.8	421843.9	462182.5	436688.7	431520.7	419169.4	410184.6	409759.7	409127.8	414554.4	847530.4	872842.2	905287.0	891682.4
P97377	Cyclin-dependent kinase 2	Cdk2	346	39	8.54	594383.6	615313.0	742594.8	628644.2	661639.4	607284.7	506890.4	635279.9	686922.4	660299.1	547999.2	616595.5	652003.3	617330.5
Q99KH8	Serine/threonine-protein kinase 24	Stk24	431	47.9	5.43	383637.6	335649.4	377411.5	308596.1	396527.1	402274.0	402226.0	382690.6	370631.2	344145.1	405529.5	367746.1	405266.4	367135.6
Q64105	Sepiapterin reductase	Spr	261	27.9	5.74	238338.3	248019.5	198269.5	233402.9	237283.5	253862.6	237609.1	219096.9	218924.3	241011.7	260736.3	282768.6	267799.5	262386.1
O88532	Zinc finger RNA-binding protein	Zfr	1074	116.8	9.04	402174.8	415835.5	416849.9	409041.5	406243.3	420131.9	380793.8	372933.8	408515.9	415127.8	114244.5	198709.3	144863.0	145213.4
P70696	Histone H2B type 1-A	H2bc1	127	14.2	10.29	1643682.0	3287594.0	3099616.2	3379387.5	2641724.9	2228952.8	2104629.0	1868777.0	3137332.1	3204238.8	5642840.8	7785792.4	7332469.9	7626034.2
Q9DC69	NADH dehydrogenase [ubiquinone] 1 alpha subcomplex subunit 9, mitochondrial	Ndufa9	377	42.5	9.74	308597.3	332213.5	323864.9	346752.8	321607.6	306528.1	343586.8	301995.4	329992.7	344374.4	737587.7	628613.3	684241.2	784970.2
Q9CQC6	Basic leucine zipper and W2 domain-containing protein 1	Bzw1	419	48	5.92	1813907.4	1766398.8	1937085.1	1805156.5	2167625.6	1901031.5	1732137.2	1792484.7	1833743.5	1838468.0	1476244.5	1562722.4	1750583.7	1518622.8
Q8BWM0	Prostaglandin E synthase 2	Ptges2	384	43.3	9	176089.6	163294.3	143482.9	159685.7	147773.7	150875.8	160198.2	151555.0	156074.6	158452.0	280987.2	274646.0	259272.1	268994.2
O88958	Glucosamine-6-phosphate isomerase 1	Gnpda1	289	32.5	6.6	1154028.8	1174512.4	1113338.0	1255894.6	1079071.6	1100062.1	1186293.1	1076472.8	1123750.5	1120038.0	1127126.5	966794.4	1098751.9	1024175.8
Q8K019	Bcl-2-associated transcription factor 1	Bclaf1	919	105.9	9.99	472977.8	450971.8	632461.1	509242.4	696251.5	645750.1	564705.7	573611.5	502988.1	524814.6	365118.7	394140.6	362578.2	372616.7
Q3THS6	S-adenosylmethionine synthase isoform type-2	Mat2a	395	43.7	6.48	1875610.8	1808954.8	1531670.0	1694533.2	1615645.8	1654502.8	1770896.8	1528844.4	1738246.6	1762590.8	1666998.4	1480938.6	1482677.7	1506500.0
Q8CGY8	UDP-N-acetylglucosamine--peptide N-acetylglucosaminyltransferase 110 kDa subunit	Ogt	1046	116.9	6.7	172548.2	173387.1	172952.7	173916.0	182207.7	186654.1	160737.3	181304.8	178733.2	175265.4	297205.1	291186.3	270899.9	278309.3
P70261	Paladin	Pald1	859	96.7	6.58	243281.9	253294.3	237224.2	234389.7	300683.0	269796.5	285033.6	228418.1	286749.4	269130.1	327076.8	335439.3	314359.6	342188.9
Q8R1Q8	Cytoplasmic dynein 1 light intermediate chain 1	Dync1li1	523	56.6	6.42	235924.3	270443.3	257848.3	260827.9	239116.0	250949.7	233135.4	247734.7	253311.9	268668.1	633489.9	614491.2	605535.8	598924.7
Q99M31	Heat shock 70 kDa protein 14	Hspa14	509	54.6	5.92	298589.9	325429.3	325899.1	341306.7	336847.9	305275.2	271709.8	274429.7	311861.6	337840.4	304172.0	343678.1	322265.5	334509.9
Q9CZR2	N-acetylated-alpha-linked acidic dipeptidase 2	Naalad2	740	82.7	8.37	157423.5	136234.3	153099.9	138797.1	157365.9	167266.7	195551.1	153054.9	145673.4	154693.3	335197.0	275744.1	287438.6	267296.0
O35250	Exocyst complex component 7	Exoc7	697	79.9	6.98	303691.0	308961.1	305621.1	300892.3	322143.8	300055.2	289384.6	285888.8	304146.9	303889.3	378735.4	379755.0	327067.7	365971.3
Q8BGD9	Eukaryotic translation initiation factor 4B	Eif4b	611	68.8	5.67	151402.1	129942.5	161144.6	133886.2	147060.7	149070.5	128783.1	148681.8	145591.5	146556.7	338147.3	390654.6	375661.0	361745.5
Q9JMI7	Testis-expressed protein 101	Tex101	250	27	6.47	13097122.6	13926204.9	14951400.4	19390384.4	11589333.5	10934913.5	8314283.5	11414244.9	15518824.7	17089491.2	2506671.8	3369350.2	3945902.1	3584594.7
Q8R146	Acylamino-acid-releasing enzyme	Apeh	732	81.5	5.59	297143.0	283956.4	280220.7	279244.0	283520.0	306232.0	300994.2	270043.6	297824.8	298457.5	473209.1	490743.2	420144.5	476255.2
O55023	Inositol monophosphatase 1	Impa1	277	30.4	5.19	1199422.6	1077644.8	1006390.1	1096481.8	1014014.5	1003249.7	1075725.9	1037813.6	1074171.8	1047121.4	1236063.1	1131985.8	1015648.1	1162132.1
Q9CR68	Cytochrome b-c1 complex subunit Rieske, mitochondrial	Uqcrfs1	274	29.3	8.7	582780.5	583482.3	620461.2	554860.3	569738.7	612712.0	592003.5	567287.7	598194.7	642547.6	927492.5	1173704.4	929077.4	993572.3
Q8BKG3	Inactive tyrosine-protein kinase 7	Ptk7	1062	117.5	6.84	476470.1	425438.5	454086.7	393533.2	525530.5	587961.7	548643.8	525555.1	480244.9	475814.1	357756.1	347261.7	361442.8	345658.1
Q9QZE7	Translin-associated protein X	Tsnax	290	32.9	6.55	336330.1	345387.8	408444.3	364606.6	378128.3	358016.4	332160.4	342943.1	368526.6	372562.2	274719.4	299269.1	334292.0	294503.2
Q9ER72	Cysteine--tRNA ligase, cytoplasmic	Cars1	831	94.8	6.76	257873.8	287012.4	290356.8	300886.6	279374.8	276968.4	263440.9	266303.2	288049.0	290962.2	394782.1	424036.5	363041.8	397348.1
Q9D020	Cytosolic 5'-nucleotidase 3A	Nt5c3a	331	37.2	6.65	402165.3	420390.3	443910.1	402632.5	458595.4	423720.2	451134.0	392014.1	427782.9	430460.2	781419.2	794894.5	761246.0	730139.9
P62960	Y-box-binding protein 1	Ybx1	322	35.7	9.88	297412.3	282654.1	311983.9	306664.3	245565.5	238150.8	224063.7	283249.2	309945.0	300327.7	126132.7	129584.2	125207.8	127105.0
P97315	Cysteine and glycine-rich protein 1	Csrp1	193	20.6	8.57	307387.0	284154.9	263265.6	259850.6	282212.5	350927.1	379396.9	357706.5	309458.3	281147.3	135305.9	143120.6	129724.4	136816.3
Q9Z0N1	Eukaryotic translation initiation factor 2 subunit 3, X-linked	Eif2s3x	472	51	8.4	137282.1	130351.7	130832.9	142376.6	129487.8	134186.2	113670.1	134815.3	141955.0	115336.6	570335.6	609207.7	684481.1	612805.5
P97372	Proteasome activator complex subunit 2	Psme2	239	27	5.76	235860.2	227800.2	224605.2	205608.3	253946.8	229664.8	256564.1	250170.9	229551.8	222672.4	141469.2	144162.4	158096.3	154431.8
Q9R099	Transducin beta-like protein 2	Tbl2	442	49.6	9.04	420480.9	450128.4	471025.7	443631.4	418489.3	413449.7	330184.3	415114.4	434181.8	430070.4	368534.6	427891.0	344434.6	354758.3
Q9JKB1	Ubiquitin carboxyl-terminal hydrolase isozyme L3	Uchl3	230	26.1	5.05	738500.9	843999.5	862489.7	964179.2	824775.3	799395.3	780910.3	729399.2	892015.0	888711.2	415360.6	526922.7	600067.3	612362.3
Q61081	Hsp90 co-chaperone Cdc37	Cdc37	379	44.6	5.34	283049.7	283046.0	252104.3	276850.3	255141.7	261914.1	245472.6	261815.4	256080.1	269860.4	313363.8	316403.1	296613.9	282464.4
O08599	Syntaxin-binding protein 1	Stxbp1	594	67.5	6.96	164846.6	177638.1	187650.3	176758.7	189584.3	183920.5	161977.5	169187.6	177491.2	180791.7	100152.3	110450.7	99272.4	106112.4
P10711	Transcription elongation factor A protein 1	Tcea1	301	33.9	8.38	416914.2	465968.7	421444.2	441172.0	430189.4	465916.5	484654.7	425724.3	481270.3	471177.5	133548.7	128218.6	127893.7	114585.6
Q6ZQ58	La-related protein 1	Larp1	1072	121.1	8.79	248369.1	343464.4	275475.6	306597.6	264371.4	256924.0	246727.1	268701.8	294650.3	281094.7	335104.1	383123.2	366928.2	405419.5
Q9WTU6	Mitogen-activated protein kinase 9	Mapk9	423	48.2	5.97	1068743.2	1047080.8	1048354.6	1044013.9	1075173.4	1136282.6	1071751.6	1016384.1	1086743.8	1068348.8	857474.9	844751.6	622124.7	717684.0
Q9JHU9	Inositol-3-phosphate synthase 1	Isyna1	557	60.9	6.42	1107877.1	1236870.8	1107365.2	1437091.8	891693.9	994841.3	1278051.8	1015363.0	1291126.1	1216890.8	854769.9	997267.3	899468.3	1033228.4
Q8K124	Pleckstrin homology domain-containing family O member 2	Plekho2	495	53.8	5.5	254051.5	258406.4	222388.3	234649.9	257526.0	260240.2	254613.9	239157.0	252612.2	262867.8	362775.6	346760.3	350739.0	321782.9
Q8JZK9	Hydroxymethylglutaryl-CoA synthase, cytoplasmic	Hmgcs1	520	57.5	5.99	223594.8	229368.5	217204.6	197788.0	238587.9	240418.5	177580.7	210568.1	225277.1	208573.8	213668.1	289822.7	294966.0	286804.0
Q99MX1	Ubiquitin carboxyl-terminal hydrolase 26	Usp26	835	95.4	7.78	482704.7	510715.3	523853.7	454986.0	609047.2	565967.8	458134.4	461196.3	540228.3	553157.7	308933.9	348553.8	353855.8	303687.7
Q9WVA4	Transgelin-2	Tagln2	199	22.4	8.24	480379.1	545012.3	465315.9	512290.0	536582.3	670859.9	719296.2	584252.3	511735.0	499177.7	543399.4	557145.1	498812.4	498272.8
Q3U319	E3 ubiquitin-protein ligase BRE1B	Rnf40	1001	113.9	6.48	197091.0	216865.9	203061.8	189660.4	226618.1	217895.5	218861.3	199884.9	217589.0	219896.9	134481.0	137100.0	159109.2	136546.0
Q99JX4	Eukaryotic translation initiation factor 3 subunit M	Eif3m	374	42.5	5.74	635089.3	593991.8	510298.5	570991.8	516479.4	552920.4	587143.9	551590.3	589184.5	572573.6	457822.5	426794.8	478618.3	407920.8
O35691	Pinin	Pnn	725	82.4	7.01	393690.1	407604.6	420270.0	403579.9	446814.6	439080.8	427903.9	399864.5	413888.1	417569.3	362314.4	392035.7	363056.8	365614.3
Q8C1B7	Septin-11	Septin11	431	49.7	6.68	556782.7	508549.3	548982.5	505901.8	620781.2	591453.0	594299.6	602272.4	574166.4	542297.7	839428.8	601319.3	757687.3	635225.1
Q9CQW2	ADP-ribosylation factor-like protein 8B	Arl8b	186	21.5	8.43	838899.7	793298.2	904453.7	880392.3	836590.1	833857.1	710171.0	898743.9	835975.2	716376.4	320122.9	374776.4	310950.2	350400.2
Q61553	Fascin	Fscn1	493	54.5	6.89	398566.4	324410.5	305086.3	336455.3	320053.9	314513.0	309329.7	324481.7	332128.2	346354.8	110447.0	120376.6	104222.6	101984.9
Q69ZR2	E3 ubiquitin-protein ligase HECTD1	Hectd1	2618	289.9	5.41	383966.0	455349.8	375397.6	412921.6	431852.0	462956.1	393515.4	373561.3	391369.5	412864.5	344884.0	376485.1	325220.8	376001.3
Q8VBT9	Tether containing UBX domain for GLUT4	Aspscr1	550	59.8	6.96	260659.5	218157.5	229231.8	216473.1	227384.6	219223.9	211361.5	172787.7	255991.0	253464.9	312956.3	320291.7	331638.0	344876.1
Q8CH09	SURP and G-patch domain-containing protein 2	Sugp2	1067	118	8.31	246708.7	366015.6	352308.8	425366.1	267135.7	226380.1	197961.6	250660.1	323824.0	369079.6	317337.1	379820.7	296683.5	414731.9
P61407	Tudor domain-containing protein 6	Tdrd6	2134	237.8	5.36	200212.9	282468.4	277130.0	390536.4	163021.7	177319.0	148029.0	187467.6	224665.1	253058.1	147641.5	175496.4	209408.6	263874.4
P45591	Cofilin-2	Cfl2	166	18.7	7.88	1034845.9	1225987.3	1182644.0	1234810.5	1039129.8	1080294.8	947190.1	945584.4	1140316.1	1166087.1	874355.4	1011267.1	1039129.0	1056776.1
P97434	Myosin phosphatase Rho-interacting protein	Mprip	1024	116.3	6.21	196116.1	204581.0	210713.6	212257.1	223401.5	236519.2	226510.8	198938.8	208620.8	198676.5	125671.4	102334.6	115565.9	117624.8
O88322	Nidogen-2	Nid2	1403	153.8	5.38	301085.7	220738.3	268147.2	228866.7	328236.3	344038.4	351969.0	324762.6	278775.2	272363.4	451679.1	406260.4	445614.5	379084.5
Q3TIV5	Zinc finger CCCH domain-containing protein 15	Zc3h15	426	48.3	5.3	278614.1	295024.5	270333.1	277524.5	279479.9	286342.3	246155.8	264147.2	276031.3	271235.2	206922.8	213307.0	198900.0	213068.2
Q3UXL1	Aldo_ket_red domain-containing protein	Akr1cl	322	36.8	7.61	739725.3	686749.4	713663.5	720668.0	741594.5	703389.8	738719.8	661787.3	731413.3	726530.2	1439417.6	1304012.8	1348908.0	1570158.8
Q80XP9	Serine/threonine-protein kinase WNK3	Wnk3	1757	193.9	6.02	111857.9	117052.9	91568.8	87353.7	94072.4	108098.5	111028.5	92292.6	103676.6	90123.9	180243.5	194448.3	176827.2	178929.3
Q6PE01	U5 small nuclear ribonucleoprotein 40 kDa protein	Snrnp40	358	39.3	8.1	330866.8	354221.1	346128.7	337066.0	385793.6	379143.8	347676.9	324455.9	356628.2	357897.4	316807.2	337990.1	379340.6	324883.1
Q5H8C4	Vacuolar protein sorting-associated protein 13A	Vps13a	3166	359.2	6.19	286796.3	328687.6	328636.1	375821.7	289957.2	285626.6	281237.9	286973.9	318624.8	321682.2	352401.4	351648.4	377875.6	447748.9
Q8C2E7	WASH complex subunit 5	Washc5	1159	134	7.12	166861.4	158556.3	150079.8	153889.9	175522.7	159931.1	144826.9	139424.2	159998.4	171478.2	181598.2	185715.7	171617.2	176492.7
Q9D4D4	Transketolase-like protein 2	Tktl2	627	68.4	6.96	498065.3	659385.1	715905.6	753735.0	410953.8	329880.8	280761.2	439632.2	595513.1	645543.0	453353.7	696833.8	776350.0	806031.3
O70310	Glycylpeptide N-tetradecanoyltransferase 1	Nmt1	496	56.9	8	216284.7	264174.5	219565.5	260933.7	248692.9	249811.5	210583.6	238269.8	228841.0	243233.1	227213.5	234865.8	251691.5	241523.6
Q62418	Drebrin-like protein	Dbnl	436	48.7	4.92	599896.6	629932.8	547644.7	583957.8	565717.8	551686.3	560302.1	586441.3	591407.1	617437.6	302491.7	315691.7	270130.9	280556.9
Q3B7Z2	Oxysterol-binding protein 1	Osbp	805	88.7	7.2	382991.6	398730.4	388896.4	374684.5	401522.4	384216.7	390483.2	363881.9	406969.1	413764.4	369391.9	359832.2	377386.8	347942.0
Q9CR62	Mitochondrial 2-oxoglutarate/malate carrier protein	Slc25a11	314	34.1	9.94	859255.6	858198.5	754544.1	813751.4	812677.4	807073.8	829696.2	842788.1	762463.9	832942.6	530227.8	531381.3	471593.2	512614.1
Q9DC50	Peroxisomal carnitine O-octanoyltransferase	Crot	612	70.2	6.73	409028.5	452497.0	374587.8	401586.8	477387.8	444178.7	372768.0	434181.9	429831.8	440925.7	241985.2	236754.1	225538.4	236158.7
O88531	Palmitoyl-protein thioesterase 1	Ppt1	306	34.5	8	720394.9	716028.9	716901.7	667689.4	694349.0	594624.0	752668.9	746480.2	679803.9	692574.6	1131439.1	916794.1	1135349.5	1091224.1
P54103	DnaJ homolog subfamily C member 2	Dnajc2	621	71.7	8.7	330770.6	367565.4	364088.1	361304.4	370636.6	339593.6	301466.3	335549.1	333302.9	380551.3	335046.4	340736.7	364880.0	341926.8
P53026	60S ribosomal protein L10a	Rpl10a	217	24.9	9.98	2309020.6	2554646.5	2613569.7	2627116.4	2597515.9	2557386.7	2627078.8	2508754.8	2654862.1	2730961.8	2528812.9	3273915.3	2544775.9	2904925.4
P57784	U2 small nuclear ribonucleoprotein A'	Snrpa1	255	28.3	8.62	463258.4	509707.8	514054.4	541909.5	551997.2	508334.1	510054.0	496263.0	520087.7	525130.8	414894.5	427293.0	437572.8	436051.3
Q7TQH0	Ataxin-2-like protein	Atxn2l	1049	110.6	8.85	416204.8	455609.1	479462.4	468657.5	418934.9	392817.1	351689.2	384694.3	424190.4	428759.3	386333.1	405859.0	370427.2	376001.7
Q04750	DNA topoisomerase 1	Top1	767	90.8	9.33	756741.0	793053.2	697702.8	769421.1	649464.2	649754.3	630113.5	541316.8	699023.2	724869.3	575934.8	537460.5	559610.2	549398.3
Q61024	Asparagine synthetase [glutamine-hydrolyzing]	Asns	561	64.2	6.58	634050.8	652377.7	630331.4	652467.1	591558.1	558584.6	576709.3	658620.6	601817.8	613855.2	712317.2	752403.9	651160.1	672965.5
Q7TQK1	Integrator complex subunit 7	Ints7	966	106.8	8.22	216589.1	247353.7	227369.0	233530.5	258044.7	245448.0	213316.7	224970.8	221321.7	238799.9	162927.2	168297.9	163289.0	156999.5
Q9DBP5	UMP-CMP kinase	Cmpk1	196	22.2	5.83	594832.8	588572.1	550864.3	575487.1	624710.6	629783.0	607826.2	597218.4	617319.8	621210.4	473531.8	447085.1	447424.3	430329.4
P27661	Histone H2AX	H2ax	143	15.1	10.74	1041827.7	1363972.7	1163697.3	1288671.5	1299588.4	1280150.4	1122817.4	1037582.9	1363200.6	1540813.5	2116800.7	2482946.1	2636897.3	2705895.2
Q3TZZ7	Extended synaptotagmin-2	Esyt2	845	94.1	7.75	197436.0	198864.0	170432.5	180583.3	221345.5	218736.7	216979.0	208731.2	195992.9	197424.6	163153.4	165591.6	161062.7	168280.3
Q9D2U9	Histone H2B type 3-A	Hist3h2ba	126	14	10.37	254853.0	319837.0	337869.1	278704.5	368327.8	359235.3	296835.1	278561.5	317697.0	328445.3	#N/A	#N/A	#N/A	#N/A
Q148V7	RAB11-binding protein RELCH	Relch	1216	134.5	5.34	258059.2	275212.7	243185.9	264648.7	252606.5	250740.7	212677.2	218087.1	246146.6	243934.1	172148.9	181697.8	174642.1	192952.6
P61164	Alpha-centractin	Actr1a	376	42.6	6.64	562406.4	626110.5	595097.0	656529.7	542899.3	496606.4	484543.5	546890.3	589576.4	606112.0	387229.1	397574.9	397495.8	446829.9
Q9WUP7	Ubiquitin carboxyl-terminal hydrolase isozyme L5	Uchl5	329	37.6	5.33	414945.3	455174.2	443616.1	455919.3	423897.1	372027.2	369673.5	397883.7	464623.0	454083.5	375467.8	428434.4	458443.2	421708.5
P61965	WD repeat-containing protein 5	Wdr5	334	36.6	8.27	781662.8	775538.3	967619.6	752989.4	1057969.1	1027312.4	923050.1	878828.6	883270.5	912389.7	437780.0	489479.9	580308.1	493368.0

P52196	Thiosulfate sulfurtransferase	Tst	297	33.4	7.85	3316673.7	2633870.4	2824607.4	2788069.9	2890746.0	2732060.8	2962568.8	2943208.7	2851095.3	2729500.9	3091381.8	2869780.0	2706751.8	2876796.4
P05201	Aspartate aminotransferase, cytoplasmic	Got1	413	46.2	7.14	452067.2	514146.6	432072.7	430602.2	445863.5	450415.0	448973.1	454843.6	440602.8	464592.2	622698.8	555881.8	541374.8	528137.2
P46735	Unconventional myosin-Ib	Myo1b	1107	128.5	9.26	524674.1	461833.3	458720.4	486578.7	547664.6	507275.4	500003.5	541904.7	486561.7	528058.5	417325.3	400338.4	385540.9	412760.8
P11031	Activated RNA polymerase II transcriptional coactivator p15	Sub1	127	14.4	9.6	731627.7	736733.7	787881.3	725073.9	817552.9	792601.7	609617.0	742910.5	768401.2	785217.0	983480.7	882281.0	1076273.2	961876.3
Q99M87	DnaJ homolog subfamily A member 3, mitochondrial	Dnaja3	480	52.4	9.22	218580.7	193288.9	205086.6	218540.8	207230.9	210176.1	193386.8	200741.6	196089.7	221572.7	162127.3	163499.0	171840.7	166494.4
Q80U93	Nuclear pore complex protein Nup214	Nup214	2085	212.8	7.08	187416.6	173289.2	175415.0	177417.0	202686.9	194465.7	206512.5	177337.1	212840.5	179296.0	152250.4	156797.6	156105.1	150740.0
P54227	Stathmin	Stmn1	149	17.3	5.97	788611.8	907259.3	1075861.7	975191.8	855095.0	853724.5	884038.8	820112.1	952803.6	962509.8	963869.7	1160151.0	1028710.4	1217562.8
P35922	Synaptic functional regulator FMR1	Fmr1	614	68.9	7.62	342829.0	368286.8	344218.3	352812.1	393224.8	367232.7	320067.8	315477.9	349781.4	369890.3	241974.0	304887.2	233140.2	256395.4
Q9Z2K1	Keratin, type I cytoskeletal 16	Krt16	469	51.6	5.2	106568.7	125580.5	99172.8	108727.5	349369.9	116691.3	100835.6	101030.8	130989.0	114092.7	53579.7	63035.7	62613.1	55208.3
Q99LD8	N(G),N(G)-dimethylarginine dimethylaminohydrolase 2	Ddah2	285	29.6	6.01	811602.7	737264.3	733078.2	702153.0	846892.4	846094.7	793588.0	835323.8	789894.7	740368.1	658191.1	572765.1	597497.0	556228.7
Q03734	Serine protease inhibitor A3M	Serpina3m	418	47	6.1	16813.4	22272.9	16393.5	19976.9	17204.4	20147.8	18715.9	16285.9	24734.0	19119.3	41459.9	33479.9	45505.5	35682.4
Q80YD1	ATP-dependent RNA helicase SUPV3L1, mitochondrial	Supv3l1	779	87	7.84	258712.3	321410.1	259659.9	319729.5	302811.2	267435.0	258150.8	252161.7	290971.4	300365.6	229562.7	274573.2	251746.6	251433.1
Q60710	Deoxynucleoside triphosphate triphosphohydrolase SAMHD1	Samhd1	658	75.8	7.93	389014.8	377414.6	327661.3	370165.3	356436.7	338910.3	385526.9	344020.8	381599.6	392956.6	417528.6	425197.2	350760.6	422441.7
Q6NZR5	Superkiller viralicidic activity 2-like (S. cerevisiae)	Skiv2l	1244	137.4	6.28	136997.1	152152.9	135713.1	145180.0	141601.4	138720.5	125288.5	144250.8	140523.1	132228.8	59146.3	85630.9	54684.2	70314.2
B1AR17	Chromodomain helicase DNA-binding protein 3	Chd3	2055	232.6	6.27	31514.8	26221.3	37224.7	27802.4	31669.0	36119.5	31337.9	33649.0	32765.4	36709.5	51796.8	54224.6	53281.9	53445.3
Q91WK2	Eukaryotic translation initiation factor 3 subunit H	Eif3h	352	39.8	6.67	280036.1	309767.4	298997.9	305837.5	322114.2	298686.0	283694.2	259121.0	297603.5	301594.8	510784.3	536802.6	532132.9	511049.5
Q80X81	Acetyl-Coenzyme A acetyltransferase 3	Acat3	397	41.4	7.94	769099.6	589759.3	764076.0	749006.8	711965.4	728584.4	706173.7	721909.4	770939.9	726412.8	383838.7	347141.5	402665.5	417499.7
P46638	Ras-related protein Rab-11B	Rab11b	218	24.5	5.94	1718819.7	1701817.2	2053598.0	1808547.2	1820066.4	1782249.0	1732058.3	1934599.7	1741143.1	1784470.1	1828495.4	1796020.0	2061700.0	1784419.3
P54923	[Protein ADP-ribosylarginine] hydrolase	Adprh	362	40	5.76	410989.6	475930.4	466761.2	485651.0	420141.8	418302.6	417855.0	390854.7	467857.0	496438.1	573036.0	689912.1	670611.4	734991.7
Q8VCG1	dUTPase domain-containing protein	Dut	200	21.2	9.58	230771.5	245647.0	250129.8	256725.0	256306.6	276636.5	287633.5	248302.2	264729.8	278052.8	1118316.5	1522325.6	1445952.2	1568277.6
Q8VDQ1	Prostaglandin reductase 2	Ptgr2	351	38	5.41	521854.9	558528.1	525444.9	554466.4	575785.5	566508.8	568663.0	499289.0	545326.6	579292.9	281637.9	305389.2	289333.9	319790.1
O08795	Glucosidase 2 subunit beta	Prkcsh	521	58.8	4.46	576386.7	538358.1	552873.7	514094.0	526120.4	553855.5	484910.4	569540.2	512077.1	511477.0	933999.1	871556.7	834370.5	901532.5
Q9D1Q6	Endoplasmic reticulum resident protein 44	Erp44	406	46.8	5.27	394837.4	427175.0	390554.3	409625.8	430986.7	432504.3	404237.7	412512.9	417857.6	374212.2	426154.7	744272.3	409513.0	597311.3
Q8BJW6	Eukaryotic translation initiation factor 2A	Eif2a	581	64.4	8.91	416658.0	569862.0	420744.8	490942.9	526312.8	481509.7	400062.9	454431.3	465785.4	527376.9	376161.2	378846.4	406129.1	377581.2
Q8R2G4	Ecto-ADP-ribosyltransferase 3	Art3	371	42	6.02	316041.7	382471.0	385258.2	518401.4	320375.3	285968.2	292828.7	279084.3	321698.2	326769.8	381226.5	446758.4	469617.5	531879.2
Q6PDL0	Cytoplasmic dynein 1 light intermediate chain 2	Dync1li2	492	54.2	6.28	140085.1	157499.1	152077.1	153812.9	161778.8	156160.8	137838.2	159394.7	154851.1	156413.2	753775.6	756845.3	788710.5	794990.2
E9PV24	Fibrinogen alpha chain	Fga	789	87.4	6.11	1063356.2	1031833.8	764984.3	861962.2	513902.5	562504.0	958236.6	841190.7	848118.2	957211.2	669751.9	730624.7	525180.7	657867.5
Q8R2Y0	Monoacylglycerol lipase ABHD6	Abhd6	336	38.2	8.47	1020417.4	900862.0	836083.4	887258.1	758249.5	749976.2	714414.0	867870.8	859893.2	869942.8	463536.6	423625.0	465632.2	413246.0
P12367	cAMP-dependent protein kinase type II-alpha regulatory subunit	Prkar2a	401	45.4	4.89	146414.9	148744.5	144385.4	147596.1	166352.8	179379.2	150770.7	134329.4	147250.9	153465.7	220514.7	224827.9	218353.9	192916.2
Q6P5B0	RRP12-like protein	Rrp12	1295	143	8.91	192839.7	198771.5	179523.1	195008.2	184001.4	190416.2	171796.0	175959.2	170162.8	215928.3	268220.1	276676.5	284183.2	255550.6
Q9WUL7	ADP-ribosylation factor-like protein 3	Arl3	182	20.5	7.24	632213.3	736950.1	747827.2	888477.9	561007.6	567359.5	560460.8	577507.3	627990.8	657815.5	356281.0	372648.6	381288.5	435760.4
Q91VR2	ATP synthase subunit gamma, mitochondrial	Atp5f1c	298	32.9	9.01	1650630.5	1487231.0	1639920.1	1748611.9	1587305.3	1454122.5	1624978.3	1695556.8	1578766.3	1544400.8	1359309.0	1227230.2	1341196.5	1332090.3
P22315	Ferrochelatase, mitochondrial	Fech	420	47.1	8.91	579949.9	537989.3	519771.6	576684.3	564557.7	573810.2	561873.5	522288.9	575072.3	555634.0	530322.7	455964.8	477136.6	490285.8
Q9CXI5	Mesencephalic astrocyte-derived neurotrophic factor	Manf	179	20.4	8.07	1858626.5	1801961.5	2120086.9	1999712.2	1781771.2	1673096.3	1383960.7	1748912.4	1796085.6	1827460.6	934759.1	1443682.6	1079404.1	1302376.6
P49135	General transcription and DNA repair factor IIH helicase subunit XPB	Ercc3	783	89.1	7.18	334741.2	336237.2	326831.4	325929.9	306978.0	320522.6	278155.5	264478.3	314250.4	334602.5	270077.5	298456.0	255079.8	265239.5
Q80TL7	Protein MON2 homolog	Mon2	1715	189	6.13	156234.6	142886.5	145932.7	140961.8	138376.3	149857.2	134056.5	134201.9	147860.2	151593.8	324442.8	369667.4	330274.4	352336.0
Q99KK2	N-acylneuraminate cytidylyltransferase	Cmas	432	48	8.1	814195.7	734982.3	752308.4	714942.8	722240.6	768159.9	973501.4	805033.3	866332.8	804794.9	996804.7	884261.4	960364.7	952800.7
P60766	Cell division control protein 42 homolog	Cdc42	191	21.2	6.55	1307355.3	1134314.1	1148604.0	1149422.4	1206834.8	1241511.3	1331593.5	1267868.6	1198233.1	1210779.1	466992.9	481489.8	465959.3	439395.1
Q8BHB4	WD repeat-containing protein 3	Wdr3	942	105.7	6.64	328288.3	342504.0	336330.3	326590.8	367961.7	349081.9	321366.7	330670.2	336861.9	364628.9	162734.1	175469.1	188662.7	163786.4
Q9Z160	Conserved oligomeric Golgi complex subunit 1	Cog1	980	109	7.18	178002.3	211290.0	161525.6	187065.2	174302.9	168820.3	153529.3	151948.4	184286.4	195412.6	145456.8	155415.9	148838.2	156818.9
Q8K2V1	Serine/threonine-protein phosphatase 4 regulatory subunit 1	Ppp4r1	951	106.2	4.81	177336.3	163085.6	172055.1	169653.1	170605.2	158012.1	161771.5	181912.5	161527.7	160765.1	56279.4	64509.1	53947.5	54221.1
Q8JZM7	Parafibromin	Cdc73	531	60.5	9.61	287765.2	326250.8	298629.5	305940.4	339336.0	323523.8	268856.2	302067.2	308647.4	319447.9	400715.9	366443.1	427401.0	396530.8
P14602	Heat shock protein beta-1	Hspb1	209	23	6.55	1055074.7	1022660.1	1522831.5	1088797.9	1647338.2	1465642.3	1773825.9	1095774.1	1328696.2	1365659.4	615436.5	623634.4	665815.4	658774.3
Q3U9G9	Delta(14)-sterol reductase LBR	Lbr	626	71.4	9.36	265610.7	330433.4	310956.7	320451.5	340281.7	369180.5	372636.3	311945.6	366983.0	382794.2	370415.6	403209.1	414095.9	408325.6
Q9JLV6	Bifunctional polynucleotide phosphatase/kinase	Pnkp	522	57.2	7.9	317560.2	337051.4	313864.1	323413.6	321658.7	314159.4	341141.9	301603.1	324689.5	325214.4	440373.0	471519.2	443016.3	463116.5
Q3THE2	Myosin regulatory light chain 12B	Myl12b	172	19.8	4.84	474841.4	463347.8	535769.9	475626.9	574614.0	606298.7	590699.4	579732.2	519097.2	501511.9	1464198.8	1304474.3	1586962.8	1444599.9
P70227	Inositol 1,4,5-trisphosphate receptor type 3	Itpr3	2670	304.1	6.54	139066.6	131452.0	131248.0	145555.2	159571.7	151179.3	140826.5	119475.4	124973.1	136225.8	57349.5	54693.7	50387.2	57165.8
P61161	Actin-related protein 2	Actr2	394	44.7	6.74	518071.8	498565.3	525931.8	503167.9	539937.1	537358.0	490969.3	571782.3	510670.9	475323.6	537796.8	556014.2	525999.4	548341.6
Q8R0X7	Sphingosine-1-phosphate lyase 1	Sgpl1	568	63.6	9.1	145780.1	146591.5	163590.7	113812.8	171223.8	162936.3	155445.7	153078.5	144120.9	126835.2	481835.6	501145.2	473455.4	439728.9
P42125	Enoyl-CoA delta isomerase 1, mitochondrial	Eci1	289	32.2	8.98	900966.7	1063929.7	838550.4	897015.0	1134242.7	1125737.6	1040691.0	1061861.9	970515.3	908696.0	938898.1	903767.1	950656.1	825836.9
Q922U1	U4/U6 small nuclear ribonucleoprotein Prp3	Prpf3	683	77.4	9.5	638725.1	661447.3	658983.2	640343.2	677559.7	673354.2	657231.1	641271.0	653448.0	683327.3	577562.9	643330.0	633661.8	604688.2
E9Q9W4	Long-chain fatty acid transport protein 6	Slc27a6	619	70.1	7.72	422750.2	396981.9	329925.6	391231.3	332775.8	364331.6	322812.5	374236.3	351715.4	330511.8	476634.9	453174.4	416967.3	445287.4
Q91WG4	Elongator complex protein 2	Elp2	831	93	5.82	170472.4	202205.6	187710.6	200495.5	175175.8	178948.5	175493.0	165618.5	186544.9	200584.5	451545.0	423650.0	416705.8	411829.8
P70336	Rho-associated protein kinase 2	Rock2	1388	160.5	5.99	385439.4	393476.4	384107.4	358172.8	415117.1	443400.9	387376.0	378088.2	381315.0	370941.2	324270.7	350096.3	305687.4	317283.6
Q99MX0	Transketolase-like protein 1	Tktl1	595	65.2	6.02	186481.5	208995.2	236580.3	182777.0	246186.3	247456.5	150786.3	171303.0	264215.9	273198.1	115994.5	140154.3	159807.7	115098.3
Q62348	Translin	Tsn	228	26.2	6.44	351526.1	303969.9	399320.2	390944.7	346484.0	327363.6	353884.6	338274.1	337270.5	355694.0	572216.1	595917.7	762762.7	638955.8
P47753	F-actin-capping protein subunit alpha-1	Capza1	286	32.9	5.55	196636.1	200018.7	197200.9	206275.0	181689.1	173989.9	189096.6	181937.0	213255.9	211174.9	671442.6	709388.5	689446.3	656214.5
Q8BVQ5	Protein phosphatase methylesterase 1	Ppme1	386	42.2	5.97	377268.3	446749.6	477636.9	468345.6	441053.5	406035.6	359703.3	434768.4	455754.6	504878.8	312756.7	335509.1	323256.9	327839.3
Q80ZX0	Sec24-related gene family, member B (S. cerevisiae)	Sec24b	1251	135.5	6.96	191075.9	199151.2	193349.0	197613.6	188825.5	196767.0	194516.7	187188.1	195459.8	203312.5	149044.6	140957.2	155840.5	135132.4
E9Q4X2	UDP-glucose glycoprotein glucosyltransferase 2	Uggt2	1504	172.7	6.2	311573.0	326397.8	333722.1	342819.8	264746.2	267533.3	258752.7	254599.8	306949.8	332305.5	271354.2	308369.4	322317.6	370166.5
P54731	FAS-associated factor 1	Faf1	649	73.8	4.86	309109.1	331674.9	305180.6	334445.1	337822.6	317756.9	287169.2	291056.4	338104.2	329231.3	270165.7	278874.9	250772.3	279844.6
Q791V5	Mitochondrial carrier homolog 2	Mtch2	303	33.5	8.25	459418.0	532143.8	473714.8	492563.2	503210.3	455062.6	439302.8	470518.9	459374.0	489261.4	486039.2	550421.0	463943.6	537310.9
Q7TQI3	Ubiquitin thioesterase OTUB1	Otub1	271	31.3	4.94	1110414.0	1161877.8	1331461.8	1180476.7	1253360.6	1144470.2	1116880.1	1161585.0	1242661.2	1283026.8	846313.8	963644.7	1101504.3	986643.3
P35293	Ras-related protein Rab-18	Rab18	206	23	5.36	530680.7	540749.5	570498.2	561171.3	490762.5	434327.9	425356.4	468937.7	506187.8	534835.6	819425.5	966815.1	814579.5	855204.1
Q9R1P1	Proteasome subunit beta type-3	Psmb3	205	22.9	6.55	533365.1	518931.2	545206.3	558832.6	569921.3	532401.7	533651.6	496250.1	568227.3	582830.4	324297.9	363984.9	390505.7	369431.4
Q8BI84	Transport and Golgi organization protein 1 homolog	Mia3	1930	213.5	4.75	264300.9	125659.1	135404.3	154152.1	104541.4	99925.8	100904.4	115471.0	116866.8	114963.0	46829.7	58822.9	48336.6	56790.3
E9QAT4	Protein transport protein Sec16A	Sec16a	2357	254	5.81	161598.0	163549.5	168763.6	163689.3	173494.7	168643.0	175419.4	144865.0	164842.8	171669.5	209855.3	215047.7	192419.5	208530.6
Q3U7R1	Extended synaptotagmin-1	Esyt1	1092	121.5	5.95	337433.8	323045.9	337438.1	311086.5	360832.5	384447.7	421741.4	383317.4	337196.8	343522.5	440896.6	482141.3	463240.6	507249.9
P62754	40S ribosomal protein S6	Rps6	249	28.7	10.84	801547.1	772728.0	727516.3	754201.8	637383.4	662575.5	543360.1	663726.7	695331.5	726851.7	892019.9	937453.6	844454.5	803846.7
P47911	60S ribosomal protein L6	Rpl6	296	33.5	10.7	2229486.9	2587745.0	2495860.3	2574689.5	2302988.0	2141878.4	1926177.1	2240849.9	2328919.9	2373640.8	2574402.4	2874126.1	2797670.7	2746648.9
Q8C0M9	Isoaspartyl peptidase/L-asparaginase	Asrgl1	326	33.9	7.65	525341.6	616036.8	645862.7	746526.9	480545.0	443415.4	423420.6	484124.9	527661.7	573377.0	558998.0	646163.2	614609.7	654868.6
Q9Z1W9	STE20/SPS1-related proline-alanine-rich protein kinase	Stk39	556	60.3	6.29	187549.8	207033.3	171974.8	199649.0	176365.2	180737.4	153545.1	187187.3	169527.1	193214.9	148233.5	152418.8	150316.8	167658.4
Q9WV60	Glycogen synthase kinase-3 beta	Gsk3b	420	46.7	8.78	303397.9	344357.4	297056.3	313594.8	313943.4	352884.9	303909.0	299441.8	322523.6	314008.3	83729.4	90296.5	99112.1	90390.2
A2A8L1	Chromodomain-helicase-DNA-binding protein 5	Chd5	1946	222.4	6.24	11252.8	13032.6	15854.1	14774.1	17320.8	15999.1	11178.6	13266.1	13143.6	16549.0	#N/A	#N/A	#N/A	#N/A
Q99J77	Sialic acid synthase	Nans	359	40	7.06	269566.8	268191.4	264783.1	274332.4	293176.7	292833.4	281676.9	253747.2	288246.3	266258.1	372522.2	430218.5	390757.1	367637.2
Q04736	Tyrosine-protein kinase Yes	Yes1	541	60.6	6.64	214215.5	231241.2	188121.5	193941.7	220539.5	225745.3	198283.6	192039.9	202150.2	225523.4	154052.7	156863.1	161726.8	147302.2
Q9QYE6	Golgin subfamily A member 5	Golga5	729	82.3	6.23	276741.1	302458.0	347685.4	317078.9	311197.2	300348.1	281493.9	283592.3	288618.1	295446.7	342881.2	374420.1	368955.9	368349.3
Q62077	1-phosphatidylinositol 4,5-bisphosphate phosphodiesterase gamma-1	Plcg1	1302	149.6	5.8	132830.6	115158.1	126101.7	108748.8	151298.1	136786.1	149617.1	134290.6	130862.0	122900.1	210699.3	205530.2	218356.8	176937.0
Q9ESX5	H/ACA ribonucleoprotein complex subunit DKC1	Dkc1	509	57.4	9.28	394825.5	385146.3	348031.9	323007.1	378942.9	381226.2	419517.2	334016.8	378508.4	357966.2	529390.2	464671.7	466059.6	447158.8
Q8BQZ4	Ral GTPase-activating protein subunit beta	Ralgapb	1484	165.1	6.77	245643.5	245333.8	225312.7	232008.1	257569.5	274469.5	222414.6	229589.5	260684.7	255270.3	217917.2	226322.5	225825.3	208759.2
P61087	Ubiquitin-conjugating enzyme E2 K	Ube2k	200	22.4	5.44	369834.1	410745.3	392133.5	392531.2	416390.7	431985.6	405006.7	341107.4	442527.4	459994.6	693914.6	759123.7	856719.5	778643.1
Q9D379	Epoxide hydrolase 1	Ephx1	455	52.5	8.35	786911.2	883974.8	697507.7	838625.8	700106.5	675552.3	806150.6	670261.0	743047.5	718220.7	342169.3	297505.8	271081.0	357284.7
Q2TPA8	Hydroxysteroid dehydrogenase-like protein 2	Hsdl2	490	54.2	6.74	364524.5	379768.0	373507.3	385920.4	406958.3	360725.3	395025.1	393246.9	390223.9	372344.4	246666.4	223306.9	213646.0	217176.2
P62071	Ras-related protein R-Ras2	Rras2	204	23.4	6.01	364589.9	322966.3	347700.1	322248.7	402539.6	409396.4	398514.1	368922.5	373630.9	359962.9	495101.0	448183.3	449779.5	413471.2
Q9CXF4	TBC1 domain family member 15	Tbc1d15	671	76.5	5.3	292419.7	345247.0	317495.0	340383.7	314689.5	280348.7	286371.8	297481.1	307465.5	315055.5	108033.5	130488.0	119636.3	126171.0
Q6PGB8	Probable global transcription activator SNF2L1	Smarca1	1046	121.6	8.27	182681.1	187205.8	175660.6	185150.7	175925.5	177210.3	189551.2	182366.2	177392.7	177015.2	150680.7	143728.9	142867.0	144603.0
Q60973	Histone-binding protein RBBP7	Rbbp7	425	47.8	5.05	651801.2	565091.6	622367.1	572776.3	652540.0	705169.6	731119.9	657277.3	633089.4	578828.5	1214236.9	1181051.2	1145513.5	1153623.8
P16045	Galectin-1	Lgals1	135	14.9	5.49	738331.2	1643352.5	655655.0	1080097.1	1336025.0	1201807.8	726929.9	1132557.8	950404.4	1260641.6	3982787.4	5544708.6	3478385.7	3273137.4
Q8CIG8	Protein arginine N-methyltransferase 5	Prmt5	637	72.6	6.42	534775.7	636996.3	623990.6	651529.4	560418.9	485570.2	466370.1	496274.3	575185.3	626634.5	614920.5	598669.8	676043.3	658561.7
Q8CEC6	Peptidylprolyl isomerase domain and WD repeat-containing protein 1	Ppwd1	646	73.3	7.15	146468.6	145947.9	171385.9	140350.5	174234.9	176647.1	158315.7	141507.1	152454.8	160854.5	198816.7	211602.5	200121.5	204761.5
P49722	Proteasome subunit alpha type-2	Psma2	234	25.9	7.43	260224.8	268806.9	274532.6	269531.8	279423.7	256879.9	248001.6	253633.3	261922.3	267657.6	397363.7	373413.9	502077.0	429336.4
Q6P549	Phosphatidylinositol 3,4,5-trisphosphate 5-phosphatase 2	Inppl1	1257	138.9	6.54	282232.5	284313.6	276421.6	289076.6	268005.0	288245.4	269617.3	242906.3	294012.2	305057.1	181624.7	218946.3	190227.9	186158.1

D3Z5G7	Carboxylic ester hydrolase	Ces1b	567	62.2	5.22	107331.0	111208.5	103473.6	94788.3	95903.0	98534.7	86837.0	94236.5	111124.6	96711.7	175426.4	179172.3	168971.8	155429.5
P05480	Neuronal proto-oncogene tyrosine-protein kinase Src	Src	541	60.6	7.84	382553.6	372150.5	440272.5	411856.3	448982.9	457178.4	431537.9	405287.6	428666.5	417063.3	255932.7	224013.1	218530.8	215683.6
Q71RI9	Kynurenine--oxoglutarate transaminase 3	Kyat3	455	51.1	8.37	273272.2	305716.2	267282.4	327060.2	280050.9	333143.5	343574.8	268569.3	279010.4	261044.4	344055.8	433428.8	341056.9	370640.7
Q8K212	Phosphofurin acidic cluster sorting protein 1	Pacs1	961	104.8	7.74	273644.7	304077.0	316422.8	311286.8	316400.5	283783.8	300081.1	286183.2	296059.8	288504.9	194906.0	201098.8	172472.1	190780.2
Q99KY4	Cyclin-G-associated kinase	Gak	1305	143.6	5.73	140658.7	169433.9	163523.2	159276.2	176667.7	174265.0	152450.7	154528.2	158139.3	165534.7	267323.4	304740.9	269206.7	264601.4
Q01730	Ras suppressor protein 1	Rsu1	277	31.5	8.88	427812.9	426397.8	402590.4	408306.5	464086.1	529664.1	592220.5	498441.9	444740.7	424067.9	221712.9	210335.9	251439.9	211252.1
O70503	Very-long-chain 3-oxoacyl-CoA reductase	Hsd17b12	312	34.7	9.52	853899.2	824326.6	745052.6	771491.9	742051.0	727351.1	720466.2	812227.5	763974.4	761977.5	863610.8	819550.2	743863.3	785398.2
Q8JZU2	Tricarboxylate transport protein, mitochondrial	Slc25a1	311	33.9	9.89	818277.9	831066.6	887710.0	765292.3	844049.9	737857.7	1087121.9	839923.7	884929.4	875359.4	621275.2	623839.6	716093.2	683179.5
Q3UM45	Protein phosphatase 1 regulatory subunit 7	Ppp1r7	361	41.3	4.92	527344.4	663450.9	550110.1	643451.1	515717.1	509317.4	594450.1	470808.2	605581.5	648582.0	507248.9	525536.9	477366.8	551567.3
P16460	Argininosuccinate synthase	Ass1	412	46.6	8.22	1159695.3	1111563.1	789351.3	1492000.6	777426.0	796296.4	929817.0	843274.5	794596.8	876166.7	731226.0	721496.4	436249.2	986504.7
Q6PGF7	Exocyst complex component 8	Exoc8	716	81	5.4	105755.8	113514.6	90461.9	109869.9	107479.4	117563.9	96746.3	102140.1	101651.3	101514.5	527485.9	536235.8	482398.7	504525.6
Q8C5W3	Tubulin-specific chaperone cofactor E-like protein	Tbcel	424	48	5.53	225009.3	236587.0	231832.0	244813.0	217416.1	242076.2	209956.7	229407.1	238428.4	219877.2	81454.1	77081.6	87609.5	82536.9
Q9R0E1	Multifunctional procollagen lysine hydroxylase and glycosyltransferase LH3	Plod3	741	84.9	6.23	101407.4	124795.4	98486.8	104063.7	103166.4	101531.3	100402.0	98836.0	98742.3	104494.1	168877.2	167267.4	155346.4	157557.3
P62242	40S ribosomal protein S8	Rps8	208	24.2	10.32	1304017.7	1460632.7	1502813.1	1416153.1	1168134.8	1202114.0	983491.3	1248089.7	1247421.8	1324590.8	793327.0	955056.3	750052.9	912544.3
Q9CQ62	2,4-dienoyl-CoA reductase, mitochondrial	Decr1	335	36.2	8.95	717898.5	627720.3	574604.6	638137.0	634452.3	739406.3	673703.3	635585.0	671090.7	644265.7	746456.0	642820.5	645796.3	674154.5
O35459	Delta(3,5)-Delta(2,4)-dienoyl-CoA isomerase, mitochondrial	Ech1	327	36.1	7.71	825429.6	743374.1	731394.1	679895.4	735665.2	686389.8	750723.0	757648.5	788732.2	743035.6	984451.0	889932.4	914437.5	836597.4
Q3U2P1	Protein transport protein Sec24A	Sec24a	1090	118.7	7.83	404826.9	394771.2	389032.3	364475.7	379453.7	362155.0	320344.9	376116.6	370211.3	367197.1	227743.8	217488.4	200868.8	213464.0
Q9CZX8	40S ribosomal protein S19	Rps19	145	16.1	10.4	1204611.6	1314692.3	1334570.2	1320687.6	1194130.4	1098807.3	1041124.9	1190685.3	1201126.4	1237830.4	1274193.8	1332688.6	1413074.6	1362502.3
Q9DC23	DnaJ homolog subfamily C member 10	Dnajc10	793	90.5	6.96	267862.6	329787.1	300752.9	339579.2	288397.7	278573.1	252371.6	294006.6	283216.1	295581.1	347471.1	355923.0	326898.7	410706.7
P46467	Vacuolar protein sorting-associated protein 4B	Vps4b	444	49.4	7.11	471565.0	418964.5	520058.9	450961.1	536786.2	553124.5	484071.7	480490.3	461345.6	475953.8	275551.7	293437.7	280091.7	304190.4
P97496	SWI/SNF complex subunit SMARCC1	Smarcc1	1104	122.8	5.73	150669.4	161366.0	151084.3	173689.6	152098.2	150275.5	140552.6	155861.9	147507.7	173571.1	270482.0	277416.6	277330.2	266790.5
P26516	26S proteasome non-ATPase regulatory subunit 7	Psmd7	321	36.5	6.77	463179.6	456947.4	471864.8	459617.3	459570.5	465466.6	462734.5	445018.5	477718.1	470880.9	908679.5	865269.9	804988.9	968103.2
Q9Z130	Heterogeneous nuclear ribonucleoprotein D-like	Hnrnpdl	301	33.5	7.31	315607.3	366941.0	344462.0	343007.3	403564.9	353524.3	321899.8	318779.0	363876.7	366343.4	496139.6	571255.5	521264.3	539520.0
Q3UQN2	F-BAR domain only protein 2	Fcho2	809	88.7	6.89	231403.1	241231.2	224175.9	234836.1	249382.8	249311.9	230122.6	237672.8	266976.5	244473.0	160002.8	177452.0	148133.0	158100.8
Q80U87	Ubiquitin carboxyl-terminal hydrolase 8	Usp8	1080	122.5	8.47	195267.1	207691.7	231374.1	217607.6	202554.7	207423.7	198596.6	173347.9	201366.8	217263.7	127185.5	149555.5	118593.4	145110.7
P61750	ADP-ribosylation factor 4	Arf4	180	20.4	7.14	1066537.6	1148310.6	1045391.7	1144617.8	1098016.3	1111525.3	1050172.1	1020031.4	1114110.0	1105970.7	1170080.8	1216494.3	1251870.4	1188092.2
Q91VM9	Inorganic pyrophosphatase 2, mitochondrial	Ppa2	330	38.1	6.98	536484.5	545328.7	626260.3	563418.9	648790.8	604580.4	682220.3	551073.0	640764.0	604353.2	350399.3	310919.5	344361.6	340387.7
Q9DBL1	Short/branched chain specific acyl-CoA dehydrogenase, mitochondrial	Acadsb	432	47.8	7.87	368698.7	322413.4	352369.3	318346.9	415974.5	348628.2	383989.9	436777.6	389204.7	302771.0	456651.2	404718.2	395480.6	418245.2
B9EJ86	Oxysterol-binding protein-related protein 8	Osbpl8	889	101.2	6.96	219785.8	235206.4	243453.1	226619.2	247116.5	228557.8	197027.3	199521.9	222761.3	226403.1	178195.8	217937.8	218828.8	197554.7
Q6PD26	GPI transamidase component PIG-S	Pigs	555	61.7	6.93	276809.2	310257.7	284171.1	290327.5	280640.5	279468.8	243605.3	267312.7	268710.3	294096.8	478376.5	471406.6	474789.2	464422.1
Q05816	Fatty acid-binding protein 5	Fabp5	135	15.1	6.54	560829.3	540751.3	460809.2	519404.5	474000.5	470605.8	496068.9	476717.6	497405.7	515026.3	490758.0	403730.1	397041.8	426130.4
P42232	Signal transducer and activator of transcription 5B	Stat5b	786	89.9	6.05	169348.9	164555.5	162085.0	167779.9	170167.1	179245.6	183242.3	162066.5	170637.5	160424.0	251112.6	255744.9	242139.7	240685.6
Q9CSN1	SNW domain-containing protein 1	Snw1	536	61.4	9.48	795892.2	760199.6	881133.8	792600.4	707721.4	758276.0	916195.2	779206.4	799863.7	794411.3	444417.6	448515.0	484061.6	450930.6
Q5DTM8	E3 ubiquitin-protein ligase BRE1A	Rnf20	973	113.5	5.96	262526.9	269779.1	254085.9	281397.0	296874.5	319616.5	282671.8	263039.3	271389.6	275109.7	131173.3	123778.9	116376.3	132539.9
O08583	THO complex subunit 4	Alyref	255	26.9	11.15	229474.2	230983.2	275988.8	245207.4	273840.8	265410.9	275325.6	251335.7	257009.2	245793.7	344337.9	404930.9	325216.4	379629.1
Q8BP48	Methionine aminopeptidase 1	Metap1	386	43.2	7.17	341423.8	403149.3	352422.9	395148.2	353053.2	364603.2	328400.7	334292.1	372761.0	388383.2	254858.0	280072.0	252881.2	287179.5
Q8K4G1	Latent-transforming growth factor beta-binding protein 4	Ltbp4	1666	178.5	5.47	84922.7	89050.3	98922.0	82867.1	107128.2	109981.1	95406.6	108162.9	90100.2	94909.1	65323.5	74711.0	75314.5	66944.0
O70566	Protein diaphanous homolog 2	Diaph2	1098	124.8	6.92	523723.4	437958.0	456454.0	447082.8	479931.6	459340.5	470835.9	480944.6	462268.7	462453.2	518523.3	434665.3	441058.6	477422.0
Q80YR5	Scaffold attachment factor B2	Safb2	991	111.8	6.38	455054.0	592109.9	422014.0	471894.0	467417.6	468244.7	452410.1	451473.9	422364.7	453787.5	129047.1	136423.8	135523.0	119754.6
P97494	Glutamate--cysteine ligase catalytic subunit	Gclc	637	72.5	5.9	490385.7	486619.5	462931.0	498706.6	453399.6	480041.3	447255.8	458532.0	476654.8	518572.8	349630.7	356353.1	346760.4	326041.3
Q8BP00	IQ calmodulin-binding motif-containing protein 1	Iqcb1	598	68.7	9.31	459268.0	450915.4	448047.1	425948.8	466148.3	476134.5	447445.9	411554.6	499141.0	475552.6	354985.4	384211.8	371634.4	349099.9
Q8K298	Anillin	Anln	1121	122.7	6.98	181762.0	207819.4	228899.1	221276.9	189665.9	255152.2	180229.9	185634.5	199758.2	193324.8	129045.9	138336.0	168410.9	151837.8
P40336	Vacuolar protein sorting-associated protein 26A	Vps26a	327	38.1	6.57	226137.3	269993.7	272347.2	288873.3	266554.0	253725.5	220240.0	239881.4	262189.5	261183.4	681180.1	718123.3	743788.7	759377.1
P62830	60S ribosomal protein L23	Rpl23	140	14.9	10.51	850290.8	878287.9	787422.0	885918.0	726662.1	742550.2	721541.3	789623.2	805114.3	811505.3	830814.4	1006954.7	760346.5	945095.4
Q9Z103	Activity-dependent neuroprotector homeobox protein	Adnp	1108	124.2	6.86	157075.5	151214.0	150585.8	156109.7	148207.4	163885.9	150706.0	137363.6	156512.7	135478.6	133970.6	132389.7	141091.6	128750.4
P49586	Choline-phosphate cytidylyltransferase A	Pcyt1a	367	41.6	7.03	408248.5	444249.3	474657.6	465724.4	564871.3	618785.7	684165.1	458084.8	522804.4	508312.4	460055.4	425161.4	475512.3	436507.7
P58389	Serine/threonine-protein phosphatase 2A activator	Ptpa	323	36.7	6.39	812558.0	960078.5	1053769.4	1100024.1	872363.0	876612.2	877831.0	788037.0	939631.4	1049016.8	798671.6	869480.2	956589.5	942077.5
Q9DA97	Septin-14	44088	430	49.8	7.27	301811.6	273553.5	308083.8	286352.0	287123.4	268294.7	280032.4	266690.7	298676.4	300036.0	564585.0	510934.7	552311.3	442335.0
P29533	Vascular cell adhesion protein 1	Vcam1	739	81.3	5.3	326635.6	269991.5	248251.2	303734.3	256807.5	238766.7	257110.8	304346.0	252910.0	273590.4	245848.9	202729.4	188117.7	230072.1
Q8K0D5	Elongation factor G, mitochondrial	Gfm1	751	83.5	6.92	221705.5	233914.9	231000.3	234755.1	219137.8	205604.5	202310.2	210578.9	223869.2	228087.3	125756.3	146688.2	117580.0	139345.5
P46978	Dolichyl-diphosphooligosaccharide--protein glycosyltransferase subunit STT3A	Stt3a	705	80.5	8.1	708451.8	692488.1	559796.1	727543.2	598934.5	594840.4	544389.1	517942.8	685623.9	683591.6	547572.2	515660.4	499612.6	554174.3
Q60972	Histone-binding protein RBBP4	Rbbp4	425	47.6	4.89	538739.0	543077.2	540832.0	529391.0	584185.0	519159.5	559418.3	548362.7	573380.4	544281.4	165991.2	222279.8	186061.7	200491.8
Q9WTP7	GTP:AMP phosphotransferase AK3, mitochondrial	Ak3	227	25.4	8.84	774108.0	694036.6	705365.7	738413.1	775359.8	768836.4	860256.1	728542.8	828583.2	774703.0	1136084.5	1094704.7	1071022.9	1090130.3
Q9EQP2	EH domain-containing protein 4	Ehd4	541	61.4	6.76	236811.0	243177.6	209329.0	232462.5	229798.9	226727.1	235246.0	210769.9	213511.1	232420.2	249330.7	236245.4	249834.6	232578.2
O70435	Proteasome subunit alpha type-3	Psma3	255	28.4	5.44	971642.4	1328227.9	922606.8	1158450.4	1078449.8	1063241.5	1047185.5	928540.2	1126531.5	1252420.0	438778.9	570573.8	441331.9	519314.3
A2AIV2	Protein virilizer homolog	Virma	1811	201.3	4.96	117890.1	147832.0	122762.9	105019.1	128818.0	136777.2	116749.3	115325.1	128822.4	137641.3	98574.7	84672.4	100272.7	92391.0
Q8CEE6	PAS domain-containing serine/threonine-protein kinase	Pask	1383	151.2	5.07	424404.5	490054.8	444752.8	495927.7	422743.9	400921.1	357962.7	365354.9	421379.1	495549.3	235237.2	315774.6	277852.2	297954.5
Q6P2B1	Transportin-3	Tnpo3	923	104.1	5.57	231795.1	274621.5	253222.4	266503.4	247905.8	248815.1	221293.7	234655.4	232595.1	256625.4	320409.5	353904.1	332596.6	357551.4
Q6WVG3	BTB/POZ domain-containing protein KCTD12	Kctd12	327	35.9	5.81	89620.0	132821.3	83031.1	93118.3	100173.5	114213.3	83813.1	118191.6	90022.2	93587.4	198980.1	228717.3	188008.7	203976.5
P28741	Kinesin-like protein KIF3A	Kif3a	701	80.1	6.54	233691.8	295212.8	267183.6	352386.0	221832.8	204831.0	193100.1	207162.7	243375.7	269344.0	147139.1	180383.0	177578.9	185758.3
Q8K3H0	DCC-interacting protein 13-alpha	Appl1	707	79.3	5.41	244377.6	225792.1	221330.8	237286.1	224633.2	223387.8	234315.0	232087.7	241347.5	227056.2	394297.0	474448.0	367013.1	423445.4
P70362	Ubiquitin recognition factor in ER-associated degradation protein 1	Ufd1	307	34.5	6.7	961325.1	889061.9	1007014.8	964589.0	1042467.1	1040838.2	1023121.5	911745.9	1031156.7	970647.4	521547.0	560125.2	520339.4	524328.2
Q9WVA3	Mitotic checkpoint protein BUB3	Bub3	326	36.9	6.84	836301.3	846522.3	985890.0	898797.2	927072.5	841181.9	822235.2	797072.3	920862.8	948900.5	897359.0	935070.7	991465.2	965493.7
Q99KK9	Histidine--tRNA ligase, mitochondrial	Hars2	505	56.9	8.32	216462.2	193978.5	213464.5	182192.7	209087.4	214304.8	206337.8	197599.1	191025.4	192229.2	306374.0	331042.0	299338.1	320716.9
P70268	Serine/threonine-protein kinase N1	Pkn1	946	104.3	6.27	321996.0	411943.7	325769.0	316102.6	341871.8	347721.5	323374.2	334192.8	332104.7	320015.2	449640.2	435695.5	422055.9	425326.2
Q9D902	General transcription factor IIE subunit 2	Gtf2e2	292	33	9.64	483451.1	535443.5	569099.2	508687.9	522058.4	511779.2	442810.9	495531.1	552135.8	583385.1	369920.1	405281.3	386488.8	362900.0
Q65Z40	Wings apart-like protein homolog	Wapl	1200	134	5.41	255610.7	270269.2	271819.3	251515.8	306254.3	285981.1	282067.5	234346.0	262545.4	268796.8	116353.1	130914.6	132377.5	146264.0
Q8R418	Endoribonuclease Dicer	Dicer1	1916	216.7	5.82	108769.7	119144.6	90455.5	94679.9	109855.5	107155.4	84651.1	103583.1	111870.8	104688.3	112786.7	146908.5	127109.2	127719.7
Q60715	Prolyl 4-hydroxylase subunit alpha-1	P4ha1	534	60.9	5.9	752058.2	687262.5	622931.7	638317.8	617507.5	578660.2	555528.5	676126.0	611711.9	648685.8	597017.7	554830.1	510318.0	498095.0
Q3UVL4	Vacuolar protein sorting-associated protein 51 homolog	Vps51	782	86.1	6.24	297247.1	311465.6	272375.9	296449.2	271368.3	275983.2	294615.8	271887.6	293557.9	293726.4	175226.9	230417.3	175682.2	197424.0
P42669	Transcriptional activator protein Pur-alpha	Pura	321	34.9	6.44	217997.9	194641.6	202855.6	205990.1	212988.4	222072.2	234136.7	206900.5	215596.8	211675.6	516814.1	481370.8	469471.8	491899.2
Q810B6	Rabankyrin-5	Ankfy1	1169	128.6	5.91	263213.5	277662.3	241084.4	252921.3	285571.3	283071.8	263787.5	258927.7	264775.8	284373.5	162481.1	181574.3	144226.9	166391.0
Q3UHX2	28 kDa heat- and acid-stable phosphoprotein	Pdap1	181	20.6	7.39	276936.3	390046.3	425818.5	262033.4	440226.6	298871.8	264943.6	284862.3	271785.0	420688.1	389665.3	388456.3	428958.4	387951.7
G3UXL2	Pribosyltran_N domain-containing protein	Prps1l3	318	34.8	6.98	809665.4	800781.9	794425.8	844578.6	852531.2	790701.8	677642.5	792859.3	847496.1	854687.8	958747.2	1017723.8	991843.9	980037.3
Q58A65	C-Jun-amino-terminal kinase-interacting protein 4	Spag9	1321	146.1	5.15	181801.5	210440.1	173357.3	184801.4	202108.2	187550.5	177073.8	174948.4	180198.7	186348.4	187694.4	223846.8	179041.1	187668.6
O54734	Dolichyl-diphosphooligosaccharide--protein glycosyltransferase 48 kDa subunit	Ddost	441	49	5.83	2640573.4	2524090.5	2916771.7	2625000.9	2882669.9	2768392.8	2087637.1	2850933.4	2488895.3	2392224.7	2041422.9	2129861.4	2354617.4	2282736.7
Q9CRT8	Exportin-T	Xpot	963	109.7	5.25	116146.1	117616.4	110242.2	127782.0	123219.5	121270.7	114400.7	105138.1	122229.8	119227.5	102593.3	101258.2	106690.2	103918.3
Q9CQL1	Protein mago nashi homolog 2	Magohb	146	17.1	6.39	496391.9	548680.7	577871.8	518072.1	576750.0	563913.7	513779.6	490086.0	562541.1	602214.0	638787.0	617415.1	781922.9	662233.9
Q05A80	Caprin-2	Caprin2	1031	114.4	6.81	167288.3	180806.4	178760.8	169666.8	195456.4	203767.0	160418.2	165077.3	174419.1	199145.2	94502.4	111372.9	129740.3	102864.4
Q7TNV0	Protein DEK	Dek	380	43.1	6.86	371463.1	349733.4	361363.9	363281.3	391599.8	405876.9	387105.6	337788.3	370008.2	375446.4	446442.3	492384.1	461395.8	476197.4
Q9CYZ2	Tumor protein D54	Tpd52l2	220	24	6.15	330726.4	344261.0	305495.2	348391.2	341267.8	337875.5	332507.0	334645.4	347792.2	325096.0	488728.1	451372.3	474953.7	447378.8
Q8K363	ATP-dependent RNA helicase DDX18	Ddx18	660	74.1	9.52	177453.3	187112.6	187625.7	177901.5	197779.5	184013.0	167685.1	170988.4	186726.5	197163.5	227321.8	221919.0	236378.3	213097.3
Q8C129	Leucyl-cystinyl aminopeptidase	Lnpep	1025	117.2	5.96	172668.9	179354.4	210106.8	180226.3	193043.3	186195.4	196060.8	182767.6	181427.2	200296.8	255496.4	279086.7	248579.3	252774.3
Q8C052	Microtubule-associated protein 1S	Map1s	973	102.9	7.02	157606.5	158844.1	137250.9	135294.4	147455.9	151573.0	129851.3	143115.6	146350.0	158209.2	128684.2	139216.0	126910.1	122777.1
Q9DAE2	RRM domain-containing protein	Rbmxl2	385	41.9	10.27	437978.5	536958.6	543068.0	652465.4	435788.8	367302.5	336247.7	380672.3	475017.5	499955.4	819696.4	1107512.9	982694.8	1215775.2
Q8R4N0	Citramalyl-CoA lyase, mitochondrial	Clybl	338	37.5	8.54	180805.2	176298.5	183595.2	191760.2	196597.5	206280.5	204119.8	190745.7	177546.3	177640.1	161025.3	153905.8	154726.5	173321.0
Q3TJZ6	Protein FAM98A	Fam98a	515	55	8.95	89869.3	101683.4	93497.7	104377.7	99845.1	93146.3	87941.1	86377.2	95814.5	91918.7	194816.2	238937.0	187690.7	212972.6
P85094	Isochorismatase domain-containing protein 2A	Isoc2a	206	22.4	8.02	77594.2	55365.3	57918.7	52532.7	59334.9	60820.0	61368.1	73525.2	66840.8	46384.2	137650.6	136619.2	142637.5	136667.6
Q9DCT2	NADH dehydrogenase [ubiquinone] iron-sulfur protein 3, mitochondrial	Ndufs3	263	30.1	7.17	546159.6	546419.6	530247.0	548829.0	525133.8	487932.4	503013.5	502948.7	557836.9	520675.7	644349.5	597101.3	687593.4	609433.2
Q62419	Endophilin-A2	Sh3gl1	368	41.5	5.72	641004.0	774023.4	707951.1	769920.8	636560.2	587720.3	552889.8	593844.9	683627.9	787974.1	287714.3	295390.4	286438.8	322105.6
P70670	Nascent polypeptide-associated complex subunit alpha, muscle-specific form	Naca	2187	220.4	9.35	399804.6	418310.0	391656.0	410031.4	340761.8	332487.7	306000.1	318964.2	370044.3	336095.0	373170.0	412058.9	348622.0	380354.0
P47963	60S ribosomal protein L13	Rpl13	211	24.3	11.55	1764206.6	1546787.4	1799319.3	1679813.2	1290419.6	1299255.6	1253030.4	1624872.0	1456606.2	1439080.2	1255567.0	1260666.9	1210212.1	1241346.7

Q91Z67	SLIT-ROBO Rho GTPase-activating protein 2	Srgap2	1071	120.7	6.64	67064.9	68766.9	68381.9	73883.4	74006.6	74103.5	64434.0	67576.2	67390.5	73204.4	140909.4	163062.3	136709.4	153117.1
P32037	Solute carrier family 2, facilitated glucose transporter member 3	Slc2a3	493	53.4	4.98	830348.4	1344751.9	1410483.8	2077640.2	441136.6	239320.0	265752.4	542523.0	868922.7	1148840.8	541887.7	781997.3	809709.9	1178569.6
Q3TIX9	U4/U6.U5 tri-snRNP-associated protein 2	Usp39	564	65.1	8.9	379337.8	425678.9	420644.2	412846.8	425550.4	431459.9	383990.6	366804.5	426815.1	456230.4	225280.4	249517.3	286588.8	363736.2
Q6ZPY7	Lysine-specific demethylase 3B	Kdm3b	1562	170.8	6.92	219913.1	220493.5	222529.3	238831.2	222705.4	226993.6	219284.3	195893.5	219552.0	245321.9	46480.1	48004.6	49702.3	45343.2
Q99J62	Replication factor C subunit 4	Rfc4	364	39.8	6.7	779432.4	815662.7	845892.1	785709.0	866709.7	876165.1	730451.6	745592.4	775799.3	802194.3	534439.8	500098.0	586149.0	488039.9
P55264	Adenosine kinase	Adk	361	40.1	6.21	942294.9	871577.3	784887.0	736226.1	957490.4	1016876.7	949677.2	941582.4	890598.1	806682.8	1270164.6	1085365.5	1103125.0	1069834.6
Q8CD92	Tetratricopeptide repeat protein 27	Ttc27	847	96.4	5.77	183992.6	194006.5	193822.8	187377.8	186959.1	183796.5	158401.5	178625.8	195956.5	187699.1	527490.6	461201.5	437504.4	406273.5
Q8CJG0	Protein argonaute-2	Ago2	860	97.2	9.19	128293.8	134484.9	119130.8	119450.6	132196.9	134556.9	122002.8	120941.7	127988.0	130314.2	200796.4	176807.9	191665.8	167693.8
O54781	SRSF protein kinase 2	Srpk2	681	76.7	4.91	293017.6	273811.9	268295.7	253926.4	261545.1	241402.1	246817.0	266974.9	271627.3	275441.4	169016.2	194465.4	155282.6	189126.4
Q66JS6	Eukaryotic translation initiation factor 3 subunit J-B	Eif3j2	263	29.5	4.81	294456.9	326175.9	295211.0	321978.5	306779.6	324419.6	287405.6	284378.7	324747.2	327690.5	139706.3	133043.9	146680.5	123235.7
P70372	ELAV-like protein 1	Elavl1	326	36.1	9.04	962977.3	1046699.5	893097.6	1046754.5	926737.0	927904.5	920337.5	817009.2	989950.3	1031944.2	1030876.6	1059313.3	1124095.4	1178660.4
Q8BY71	Histone acetyltransferase type B catalytic subunit	Hat1	416	49.2	6.33	411734.1	457252.0	452213.1	442846.7	449243.7	396388.7	355655.3	402788.5	422061.2	461705.5	568119.0	626096.2	666676.4	641502.7
Q8BIW1	Exopolyphosphatase PRUNE1	Prune1	454	50.2	5.11	501788.9	514107.5	489151.5	499620.1	513775.7	463203.0	504974.5	477685.9	513368.9	499788.1	414690.2	378963.6	388745.8	393295.7
Q01339	Beta-2-glycoprotein 1	Apoh	345	38.6	8.22	598380.8	567977.4	523894.3	487961.1	551892.8	566480.4	575776.4	576849.7	550634.9	558773.4	659711.8	593286.8	553349.1	499297.6
Q8VHK9	ATP-dependent DNA/RNA helicase DHX36	Dhx36	1001	113.8	8.29	236816.6	264429.2	249349.5	239887.7	248303.1	251263.3	227238.4	214748.2	264924.4	266405.6	289239.4	281612.7	285453.8	268896.5
P20108	Thioredoxin-dependent peroxide reductase, mitochondrial	Prdx3	257	28.1	7.58	1325806.4	1222099.1	1133285.6	1193043.9	1221483.4	1295735.1	1464950.9	1178574.7	1202820.3	1172879.0	689850.9	570921.1	635119.6	638846.8
Q9ET01	Glycogen phosphorylase, liver form	Pygl	850	97.4	7.09	166981.2	172576.9	149306.0	145987.5	158368.3	191742.0	208268.9	175287.6	155240.7	159011.3	218886.3	189056.1	191447.7	183865.6
Q9DBR3	Armadillo repeat-containing protein 8	Armc8	673	75.3	6.73	89471.9	118998.9	111776.2	118080.1	101898.8	103883.5	89659.4	92639.2	103002.3	105375.7	161652.6	195876.6	168864.0	190149.0
Q9DC61	Mitochondrial-processing peptidase subunit alpha	Pmpca	524	58.2	6.83	265627.7	243872.5	241487.5	250634.5	277298.7	297007.6	224792.1	302564.3	244177.4	246457.6	354076.3	382314.9	314895.7	393187.5
Q63932	Dual specificity mitogen-activated protein kinase kinase 2	Map2k2	401	44.4	7.05	324204.2	299644.5	318573.4	293971.4	325218.1	313890.5	280647.6	311808.0	292337.1	308390.6	599323.6	581694.7	546456.8	581189.8
Q922R5	Serine/threonine-protein phosphatase 4 regulatory subunit 3B	Ppp4r3b	820	93.9	4.86	47300.4	62970.5	51117.8	62308.5	57154.4	55274.7	45267.6	48650.7	55800.1	58673.5	167074.7	213590.0	211988.7	214551.6
Q9CXS4	Centromere protein V	Cenpv	252	27.5	9.79	759126.4	563229.2	692038.4	598295.9	619698.5	621170.0	733137.7	591922.7	612923.1	607659.0	819883.5	741603.4	815927.9	802228.9
Q8BSY0	Aspartyl/asparaginyl beta-hydroxylase	Asph	741	83	5.08	218046.2	211457.1	198720.8	200050.6	198254.4	216309.3	220936.0	222711.8	198661.6	212947.9	321163.7	290936.1	303220.2	298697.4
Q62009	Periostin	Postn	838	93.1	7.53	267331.5	196003.4	168009.0	255021.6	189064.9	214003.8	200083.4	274800.8	260922.9	260339.5	181758.0	157802.2	181433.9	173657.0
P57722	Poly(rC)-binding protein 3	Pcbp3	371	39.3	7.52	152113.5	175620.9	175752.2	217961.0	160221.1	156889.2	129816.6	132827.3	159986.0	182444.6	172802.8	250075.1	223008.6	241811.7
Q62446	Peptidyl-prolyl cis-trans isomerase FKBP3	Fkbp3	224	25.1	9.28	1377029.1	1342970.7	1518897.8	1374293.7	1432512.1	1302970.5	1245256.3	1263035.2	1515318.3	1475794.0	402927.1	400029.3	555792.5	404698.0
P15105	Glutamine synthetase	Glul	373	42.1	7.08	744863.6	694255.4	612350.9	761828.7	826294.8	707192.6	630738.2	639839.4	692420.4	731653.1	182568.2	183311.0	153809.0	213138.4
Q7M6Y3	Phosphatidylinositol-binding clathrin assembly protein	Picalm	660	71.5	7.91	329801.5	319263.9	325801.8	315071.3	330485.5	340910.6	318703.0	326431.8	328805.7	299672.4	439848.5	501411.8	406935.0	456919.1
Q9D0B6	Protein PBDC1	Pbdc1	198	22.2	4.55	149326.3	169354.1	170342.5	155938.3	187163.6	176630.9	158234.3	147190.6	174811.8	185235.8	249775.2	298730.0	291310.5	287999.8
P62751	60S ribosomal protein L23a	Rpl23a	156	17.7	10.45	557250.8	589007.8	578581.5	557894.9	545876.0	527128.2	436983.6	517794.7	506203.2	534532.2	1305171.4	2090688.8	1389331.7	1752417.9
Q9D172	Glutamine amidotransferase-like class 1 domain-containing protein 3A, mitochondrial	Gatd3a	266	28.1	8.78	263830.0	229454.9	262019.9	241392.3	243724.9	266042.4	264899.6	253152.1	253776.8	249892.9	211996.9	186195.8	199055.8	190039.1
Q6IRU2	Tropomyosin alpha-4 chain	Tpm4	248	28.5	4.68	685572.5	685120.4	555059.7	624433.9	699525.1	848468.6	788023.7	741657.8	628794.5	622392.6	453956.1	566388.0	463472.2	554003.8
Q9WUZ9	Ectonucleoside triphosphate diphosphohydrolase 5	Entpd5	427	47.1	5.29	269726.5	277704.5	244885.7	252318.2	222359.3	232772.3	219482.9	250012.1	246277.2	250080.3	164125.7	181407.5	159704.2	175985.3
O35593	26S proteasome non-ATPase regulatory subunit 14	Psmd14	310	34.6	6.52	1043332.6	1110890.8	982708.2	1020526.2	1154034.7	1035161.2	941388.6	912909.0	1031128.3	985509.6	753208.0	895164.3	741045.5	866177.7
Q8BZY3	DEAD (Asp-Glu-Ala-Asp) box polypeptide 19b	Ddx19b	494	55.6	8.54	122951.1	143343.9	128033.1	153487.6	119003.6	124700.3	120644.4	112222.1	129057.1	137431.5	77147.1	72778.1	54151.1	64271.5
Q9D706	RNA polymerase II-associated protein 3	Rpap3	660	74.1	7.99	305419.2	346404.3	326791.8	359789.3	298554.9	288887.1	251565.6	261886.3	329751.8	337054.0	312136.7	334036.5	308129.7	312101.3
Q99MV5	RNA helicase Mov10l1	Mov10l1	1187	132.7	7.21	169471.4	214058.3	220561.1	208574.9	204395.9	193704.4	140631.9	170586.4	199561.3	198823.4	264171.4	279329.3	285305.6	274029.4
Q8CGZ0	Calcium homeostasis endoplasmic reticulum protein	Cherp	936	106.1	9.14	398641.1	474948.8	458239.4	487568.9	503144.2	478174.9	404417.0	450633.3	432701.2	458340.4	360256.3	422210.8	398155.6	440227.5
Q91WC0	Actin-histidine N-methyltransferase	Setd3	594	67.1	5.6	570346.9	595677.9	528779.9	553649.4	525595.6	564410.1	503885.1	499043.3	532429.7	552787.0	321642.1	344822.0	345511.0	334130.3
Q99KD5	Protein unc-45 homolog A	Unc45a	944	103.4	6.34	138617.4	149451.3	125589.2	135729.9	133290.9	139777.0	139483.2	124997.0	118984.9	126370.7	145173.8	122061.1	131134.8	123599.0
Q9ER00	Syntaxin-12	Stx12	274	31.2	5.44	329737.2	381784.0	399663.4	394278.4	389406.5	353539.1	325010.5	340329.3	393522.6	392698.6	485082.9	452416.3	467059.0	402064.8
Q60598	Src substrate cortactin	Cttn	546	61.2	5.4	493038.7	444291.6	453258.5	494216.0	471585.2	483103.3	492762.8	476227.0	465176.0	465029.8	995562.2	938237.1	1004519.2	979118.5
O55060	Thiopurine S-methyltransferase	Tpmt	240	27.6	6.44	715290.8	653357.0	720402.4	718154.8	704420.3	642032.8	760715.9	601815.2	675672.7	713401.1	790528.4	687951.5	748571.6	698332.1
P58871	182 kDa tankyrase-1-binding protein	Tnks1bp1	1720	181.7	4.88	141706.9	127484.3	123057.0	139364.5	139245.8	163228.6	138048.6	140864.8	123890.2	130141.9	173191.2	164534.1	167569.4	165613.9
Q9QY06	Unconventional myosin-IXb	Myo9b	2114	238.7	8.63	173827.4	194371.3	145794.0	165135.2	165206.3	159077.6	158834.6	154411.8	168499.5	174264.2	76333.8	80723.1	74005.8	80399.6
P47754	F-actin-capping protein subunit alpha-2	Capza2	286	32.9	5.85	391684.4	408660.0	384270.2	399990.4	394438.9	459550.2	399672.4	390809.6	397388.5	394915.5	313590.2	325061.6	345635.6	331020.1
A0A286YCX6	Trafficking protein particle complex 8	Trappc8	1437	160.8	7.01	122077.3	111910.1	110952.0	116692.4	115063.4	93275.0	101278.5	96312.5	105484.2	113970.7	269540.4	271370.5	249728.9	265159.4
Q9CQ60	6-phosphogluconolactonase	Pgls	257	27.2	5.85	265846.1	263920.8	263212.9	287888.0	260365.1	256330.3	247085.4	256287.4	278367.6	274719.9	250558.5	241771.7	247327.4	254054.6
P09528	Ferritin heavy chain	Fth1	182	21.1	5.88	146093.7	206989.6	167019.7	173069.8	191661.3	170512.6	148541.0	179705.7	176405.0	175840.1	218751.9	223294.9	279787.5	234143.9
O08807	Peroxiredoxin-4	Prdx4	274	31	7.15	496354.3	653773.0	674381.1	622085.8	690927.1	652854.0	549753.5	594498.5	638456.0	644809.3	616138.4	814786.1	700634.0	692206.4
Q9QXG4	Acetyl-coenzyme A synthetase, cytoplasmic	Acss2	701	78.8	6.64	817681.1	951662.6	942056.2	937629.5	897027.7	916512.9	784452.4	767092.5	1001513.6	959403.6	536782.6	587592.8	605579.5	585491.9
O88543	COP9 signalosome complex subunit 3	Cops3	423	47.8	6.65	223866.7	239956.9	242269.8	250295.1	232735.6	216793.6	208547.3	211086.2	218850.2	215221.8	179277.5	180167.2	173407.7	180263.9
P35279	Ras-related protein Rab-6A	Rab6a	208	23.6	5.54	527125.3	549464.6	510389.6	536550.3	498592.8	510347.9	490299.4	495237.7	526770.7	549214.8	324970.3	365261.4	375813.3	369781.4
Q9Z1M8	Protein Red	Ik	557	65.6	6.64	1095372.6	1094155.7	1056307.9	1077418.3	1121822.6	1056600.9	1012676.0	946049.2	1127281.0	1131230.9	743389.6	729933.0	799269.1	697653.5
Q80WJ7	Protein LYRIC	Mtdh	579	63.8	9.33	165139.8	174615.5	149683.4	167794.8	173772.2	160544.7	169182.9	163928.8	174099.4	177266.8	206213.2	211550.8	206482.0	200168.8
O35350	Calpain-1 catalytic subunit	Capn1	713	82.1	5.87	198060.5	213319.3	192764.4	196636.6	201456.7	231231.8	233690.9	230631.3	196222.0	186705.1	212597.2	197427.6	199458.5	190852.8
Q80TM9	Nischarin	Nisch	1593	174.9	5.16	239633.1	229162.9	210879.2	214982.5	224467.4	234976.0	232333.2	212677.4	220923.3	249949.9	156628.4	152428.2	151384.3	154207.1
Q3TW96	UDP-N-acetylhexosamine pyrophosphorylase-like protein 1	Uap1l1	507	56.6	5.43	236509.3	218339.1	224528.4	248914.9	253150.4	268647.1	289438.3	240311.0	240048.7	250251.3	404532.5	399024.3	415158.5	380502.5
O08811	General transcription and DNA repair factor IIH helicase subunit XPD	Ercc2	760	86.8	6.92	267472.7	346757.2	270509.4	311663.4	278439.7	294028.5	261569.4	270284.9	271092.9	313122.1	243628.5	294586.7	258694.8	264883.3
O54692	Centromere/kinetochore protein zw10 homolog	Zw10	779	88	5.92	92704.9	100831.2	93266.3	94392.7	92819.9	97444.1	87430.8	84942.5	89082.0	90398.8	250293.8	262126.3	247432.6	278371.6
Q8CI51	PDZ and LIM domain protein 5	Pdlim5	591	63.3	8.25	204536.5	221688.0	176704.7	205469.7	207690.5	220452.8	225005.7	206731.7	213901.1	215083.2	198044.1	196401.5	196308.6	188079.2
Q8C754	Vacuolar protein sorting-associated protein 52 homolog	Vps52	723	82	5.9	70487.0	70998.7	75978.7	73634.8	83323.2	83817.9	79461.5	78618.4	73219.8	76308.6	199571.9	216096.7	203254.6	206743.2
O08716	Fatty acid-binding protein 9	Fabp9	132	15	7.74	383879.4	638697.1	660077.5	764523.9	301615.3	228441.5	167297.6	326235.4	498848.2	560509.7	678123.7	914644.3	1053104.0	1254807.1
Q8BMP6	Golgi resident protein GCP60	Acbd3	525	60.1	5.11	261163.2	259336.9	239068.5	257519.8	246934.3	241172.5	196141.3	225062.3	255892.0	252081.8	391684.9	404319.4	371481.4	396081.4
P70122	Ribosome maturation protein SBDS	Sbds	250	28.8	8.76	559482.4	616545.0	554854.9	630910.6	566370.5	604068.3	660532.6	612400.3	607201.1	609023.7	1027131.2	1093828.7	1056911.1	1155504.9
Q8BGS1	Band 4.1-like protein 5	Epb41l5	731	81.6	6.39	247351.0	242658.8	234045.7	220332.8	254926.9	216009.4	243286.3	224126.6	242309.4	245627.7	153485.5	158062.7	142277.2	146289.6
Q06138	Calcium-binding protein 39	Cab39	341	39.8	6.89	574505.1	678781.0	580647.3	673808.9	643364.7	619962.0	572185.9	551831.5	638053.2	667917.6	411113.3	454398.6	435454.5	470014.6
F8VPU6	USP domain-containing protein	Usp9y	2556	292.1	6.07	75095.0	89474.3	76731.6	83259.8	87352.2	88860.7	74199.7	76139.7	80286.7	83640.6	41705.9	48654.2	38833.0	42151.5
D3YZP9	Coiled-coil domain-containing protein 6	Ccdc6	469	52.9	7.34	351684.7	346027.6	369337.8	332083.8	394161.1	384977.6	374866.0	367395.7	364629.2	373907.7	311339.1	320554.4	313467.5	317026.3
A2AAE1	Transmembrane protein KIAA1109	Kiaa1109	5005	555	6.61	84476.6	96247.9	76139.3	91270.7	86793.0	91735.6	72880.1	83091.3	82370.0	99438.2	118733.7	136248.7	119673.0	120140.9
Q920E5	Farnesyl pyrophosphate synthase	Fdps	353	40.6	5.66	216931.5	207240.2	186053.7	260248.1	181495.7	163740.9	159127.0	233148.9	163486.4	163815.8	368689.8	274115.0	283171.5	343195.4
Q62417	Sorbin and SH3 domain-containing protein 1	Sorbs1	1290	143	8.25	102473.9	108577.9	107666.6	96732.9	115765.8	122061.2	124200.0	106270.1	97689.6	109525.6	68457.7	66958.9	77536.6	73368.7
Q91YP0	L-2-hydroxyglutarate dehydrogenase, mitochondrial	L2hgdh	464	50.9	8.29	248518.6	245301.1	291988.0	286430.8	248490.3	256645.3	260874.0	273356.0	272871.0	291105.4	115979.0	116092.9	131043.4	134632.2
O54833	Casein kinase II subunit alpha'	Csnk2a2	350	41.2	8.56	858559.1	1011361.9	998088.2	1011568.4	929723.1	949578.0	951716.5	857550.6	1001892.9	1009839.8	551366.6	646443.0	695715.5	707181.3
Q61235	Beta-2-syntrophin	Sntb2	520	56.3	8.69	380610.8	389757.5	365878.0	343288.9	375476.2	381531.9	387513.4	333790.0	359651.2	395769.0	540277.7	508556.9	535465.9	498146.4
Q2NL51	Glycogen synthase kinase-3 alpha	Gsk3a	490	51.6	8.81	218362.7	241368.8	214676.2	243564.7	214575.0	220496.6	203991.1	203566.7	217979.0	225218.2	146786.1	167462.0	158387.0	170691.3
Q8K2V6	Importin-11	Ipo11	975	112.3	5.26	277512.4	302788.6	262048.5	290266.1	303939.4	289507.0	248688.2	247213.7	320918.7	297366.8	418902.0	428318.3	376014.8	466622.0
Q8BTZ4	Anaphase-promoting complex subunit 5	Anapc5	740	83	6.71	246667.5	219636.4	242340.8	219573.9	262875.3	277575.4	232963.7	248297.6	235802.0	227939.5	140927.5	169739.1	140115.7	147076.5
Q8CH18	Cell division cycle and apoptosis regulator protein 1	Ccar1	1146	132	5.76	733691.5	753311.7	715422.7	764388.1	754952.2	786430.0	814292.8	685303.4	788893.6	818421.6	570708.8	599492.1	607282.9	636896.9
Q99JR1	Sideroflexin-1	Sfxn1	322	35.6	9.23	376529.0	343743.9	272267.1	360447.3	294186.4	318547.5	336244.8	311511.3	305146.4	280607.2	626058.2	605563.5	577660.3	585553.4
Q9WVH9	Fibulin-5	Fbln5	448	50.2	4.7	983883.2	1009662.8	1550678.3	1009178.5	1482524.8	1438587.1	1616001.7	1716972.3	1145817.4	1040480.8	337992.0	334589.2	397159.5	313849.5
P70303	CTP synthase 2	Ctps2	586	65.5	6.49	123360.3	124448.1	119560.7	114532.4	131078.8	133488.9	143615.1	135532.2	132000.3	124780.6	247756.4	261619.6	229216.2	235790.4
P70281	Synaptonemal complex protein 3	Sycp3	254	29.3	8.68	1070071.7	1452009.2	1463845.3	1450353.4	1490212.5	1329737.5	795547.9	913108.5	1413048.1	1421641.5	792824.9	1030566.5	1185269.4	1084441.1
Q8K183	Pyridoxal kinase	Pdxk	312	35	6.29	299748.7	253895.3	218187.3	263321.9	238518.6	293207.8	363337.4	288079.4	271082.6	255946.6	257052.3	267410.0	254968.5	272111.3
Q9WVR4	Fragile X mental retardation syndrome-related protein 2	Fxr2	673	73.7	6.23	173987.3	164641.2	161520.4	158479.1	168413.9	168088.7	161387.4	147074.9	172843.1	170105.2	80631.7	76509.1	89192.2	76974.4
Q80UP5	Ankyrin repeat domain-containing protein 13A	Ankrd13a	588	67.1	5.08	249151.7	238984.1	227339.4	265461.3	190801.0	194929.1	188433.2	214718.9	218239.7	235742.0	116849.1	131422.6	120952.1	125168.3
Q9WUR2	Enoyl-CoA delta isomerase 2, mitochondrial	Eci2	391	43.2	8.92	682514.7	686242.3	588878.4	673333.0	663188.2	591735.3	698589.2	609259.3	661216.3	661860.3	438168.6	435314.1	407400.5	404112.0
Q91YP3	Deoxyribose-phosphate aldolase	Dera	318	35	8.72	127398.7	168422.1	129851.8	128637.4	144857.9	147295.8	142840.9	125898.5	131182.0	147819.2	178973.7	190836.2	166826.9	170374.5
Q9D2G2	Dihydrolipoyllysine-residue succinyltransferase component of 2-oxoglutarate dehydrogenase complex, mitochondrial	Dlst	454	49	8.95	384975.6	512901.2	391900.2	430195.3	464539.1	440907.9	424381.8	419598.7	414064.4	455276.5	321073.6	379862.4	304220.4	336038.2
P51855	Glutathione synthetase	Gss	474	52.2	5.8	582853.4	599296.7	640408.7	611991.8	619385.5	611605.1	644344.8	524863.9	610674.2	634007.2	390610.0	396910.2	379168.0	387422.4
Q9WTX2	Interferon-inducible double-stranded RNA-dependent protein kinase activator A	Prkra	313	34.3	8.43	155804.4	181870.6	189087.2	152937.7	187526.6	192011.6	163426.3	191797.3	142487.8	157840.2	542536.0	581005.0	543724.2	560535.1
Q61490	CD166 antigen	Alcam	583	65.1	6.15	267391.6	206448.6	252756.2	173295.2	292209.3	293040.5	245422.1	288090.4	255686.0	276366.2	249163.4	233791.6	239918.9	210646.2

Q9CX86	Heterogeneous nuclear ribonucleoprotein A0	Hnrnpa0	305	30.5	9.31	298967.9	363608.2	291760.2	342796.8	337098.7	327797.7	348297.7	332795.2	296105.4	334488.2	1360105.7	1210639.9	641102.1	1385055.4
P38060	Hydroxymethylglutaryl-CoA lyase, mitochondrial	Hmgcl	325	34.2	8.41	299966.6	294172.8	289481.8	311342.6	303093.1	331716.1	293824.6	270225.7	301628.2	281395.5	216996.7	237746.6	220098.4	243179.0
P63028	Translationally-controlled tumor protein	Tpt1	172	19.5	4.86	962928.8	974609.1	1211542.4	1052941.0	1144924.2	1105299.1	1130427.3	1189023.3	1122292.2	1114934.3	1238251.2	1244143.7	1238148.3	1244756.1
Q3V1L4	Cytosolic purine 5'-nucleotidase	Nt5c2	560	64.8	6.21	394604.3	427504.3	438391.2	388303.4	444433.5	438704.4	356541.8	381476.2	422669.5	429256.2	398209.4	441155.0	528600.6	461765.2
Q9JK53	Prolargin	Prelp	378	43.3	9.54	631501.9	595828.9	579156.1	545569.2	746835.8	830103.5	1162870.4	637506.3	687078.3	674835.2	743877.3	704307.3	722049.4	705969.0
Q64368	Deleted in azoospermia-like	Dazl	298	33.3	8.48	246399.0	345555.7	249904.7	274875.5	306430.8	280944.2	225200.6	259102.0	251820.8	293010.6	217124.2	300654.7	234022.3	254342.7
P62631	Elongation factor 1-alpha 2	Eef1a2	463	50.4	9.03	10462.1	29825.9	9046.7	14281.3	16471.3	17547.9	8835.6	13085.7	10794.3	13397.8	26603.6	41772.8	26062.8	32050.1
Q6Y685	Transforming acidic coiled-coil-containing protein 1	Tacc1	774	83.9	5.03	488009.6	454993.9	431323.7	466808.9	450105.6	398701.3	428941.0	426682.1	513782.0	439859.9	197689.1	176328.2	203697.8	184502.0
P97855	Ras GTPase-activating protein-binding protein 1	G3bp1	465	51.8	5.59	517663.7	515067.2	511667.0	489686.1	480470.5	429261.6	392907.9	484626.2	469191.8	490870.5	441051.7	415103.8	447003.2	456061.9
E9Q5G3	Kinesin-like protein KIF23	Kif23	953	108.7	8.66	115746.0	116894.8	122992.6	111267.4	137099.9	134091.5	108079.1	113093.3	122230.1	118125.3	70693.8	80344.4	69885.6	71851.6
Q8R3N6	THO complex subunit 1	Thoc1	657	75.4	4.97	232370.9	223443.9	225729.8	219213.5	228057.5	257111.5	248802.0	218715.2	241086.3	228406.2	243831.8	236883.7	236262.4	240527.4
Q9R0X4	Acyl-coenzyme A thioesterase 9, mitochondrial	Acot9	439	50.5	8.59	359511.5	377666.9	367444.5	397087.9	412847.7	422350.2	382598.6	396545.5	403337.1	381472.0	346265.2	361619.8	371709.8	366973.5
P00493	Hypoxanthine-guanine phosphoribosyltransferase	Hprt1	218	24.6	6.68	830357.3	792366.0	875171.3	801785.2	919165.5	914538.1	853762.9	796944.8	902147.6	870129.2	611114.2	663809.1	531456.3	570837.2
Q8R574	Phosphoribosyl pyrophosphate synthase-associated protein 2	Prpsap2	369	40.9	7.17	334250.5	327086.8	325914.2	298655.3	326495.7	310719.1	293538.4	290029.4	319024.9	337830.5	169502.3	200077.2	180023.0	177809.7
Q91WC3	Long-chain-fatty-acid--CoA ligase 6	Acsl6	697	78	7.34	221079.3	270360.0	225133.0	263782.3	235181.6	243152.9	244497.1	223246.1	254469.7	239577.2	328611.9	339799.9	315782.6	311478.2
P31938	Dual specificity mitogen-activated protein kinase kinase 1	Map2k1	393	43.4	6.7	246235.4	258275.4	229881.7	239296.9	263209.3	261428.1	248636.6	246465.3	251742.1	245216.1	236550.8	224683.1	215836.0	194719.8
Q91W96	Anaphase-promoting complex subunit 4	Anapc4	807	91.6	5.4	196658.6	184481.6	188867.3	178257.7	196304.7	193265.5	189837.5	179118.5	180925.1	199177.0	141313.8	142120.7	158942.7	146461.3
Q8C547	HEAT repeat-containing protein 5B	Heatr5b	2070	224.2	7.14	256268.3	295991.8	298663.7	296427.6	295545.6	286028.5	280579.1	265451.2	294463.3	303826.1	144257.8	160096.2	187452.5	164691.4
P51949	CDK-activating kinase assembly factor MAT1	Mnat1	309	35.8	5.82	149468.2	169133.4	173433.9	177971.1	161938.8	157762.1	135469.0	158163.1	166982.6	166907.6	217482.2	243236.5	201381.9	232100.9
Q8R317	Ubiquilin-1	Ubqln1	582	61.9	4.94	298543.5	276531.4	301259.7	302822.1	287326.9	272870.3	279029.6	281167.3	291843.1	275960.5	148831.1	169481.2	165823.1	164884.9
Q8K4X7	1-acyl-sn-glycerol-3-phosphate acyltransferase delta	Agpat4	378	43.8	8.38	787706.7	642110.3	737935.6	629616.9	702186.1	718773.5	748858.1	853637.6	664088.4	645146.4	673133.2	647307.1	621654.9	612323.1
Q8R5I6	Glutathione S-transferase mu 4	Gstm4	218	25.5	6.93	175394.8	191780.8	173623.1	185999.5	192947.1	183466.5	196993.5	185808.8	189700.8	185010.9	226093.3	215425.5	201985.3	225843.0
Q9Z1G3	V-type proton ATPase subunit C 1	Atp6v1c1	382	43.9	7.46	349443.8	352046.2	349331.7	385458.9	388821.8	367089.0	334181.1	343349.8	366121.6	352899.7	349568.7	399014.1	363378.9	376348.3
O55135	Eukaryotic translation initiation factor 6	Eif6	245	26.5	4.74	429089.4	431020.5	466448.1	415118.6	449465.4	436475.1	436662.3	405303.9	473196.3	496982.0	307111.3	308674.0	315427.6	290579.4
G3X8U3	Queuosine salvage protein	:10016F16I	338	38.6	5.64	393689.0	349763.7	357138.1	353784.2	373682.1	364315.8	378171.2	340910.5	369355.9	382680.1	397190.9	407480.1	414712.9	409298.1
E9Q6J5	Biorientation of chromosomes in cell division protein 1-like 1	Bod1l	3032	327.3	5.33	259553.2	264297.3	251276.1	256597.3	271563.7	286996.5	250442.1	224639.1	256543.3	274519.6	105942.8	106508.2	107264.1	111205.2
Q80YV3	Transformation/transcription domain-associated protein	Trrap	2565	291.4	8.48	208611.2	212285.3	199456.3	211836.4	195119.4	198001.7	190020.3	186191.1	204007.4	210002.1	348386.1	378640.6	342933.5	359104.8
P19001	Keratin, type I cytoskeletal 19	Krt19	403	44.5	5.39	265374.2	309758.5	293919.6	350780.4	304682.5	325122.9	379113.4	328409.1	315354.2	291872.2	104093.2	130546.8	113614.4	138637.4
P23591	GDP-L-fucose synthase	Tsta3	321	35.9	6.74	434451.9	532618.2	449190.0	505109.2	456989.1	443457.2	450520.7	422384.9	458847.5	516778.9	408731.8	497899.9	428115.9	481984.8
Q9QUI0	Transforming protein RhoA	Rhoa	193	21.8	6.1	504301.5	425973.4	435947.8	426991.5	407879.3	488631.4	544891.9	475585.8	439253.5	401462.6	561293.7	551348.1	498128.8	525885.5
Q80UJ7	Rab3 GTPase-activating protein catalytic subunit	Rab3gap1	981	110.1	5.73	446896.4	429771.0	420260.5	431053.1	404424.1	408814.8	394402.6	415674.2	411929.3	417526.2	294683.5	301485.3	267803.2	293198.0
Q9Z108	Double-stranded RNA-binding protein Staufen homolog 1	Stau1	487	53.9	9.52	157532.6	172166.9	146074.4	157325.1	148474.3	150305.1	148907.1	141490.8	148569.9	160303.6	130703.1	128543.3	125183.6	118858.7
P28667	MARCKS-related protein	Marcksl1	200	20.2	4.61	106536.4	92126.8	107419.9	88858.1	122931.4	110681.9	96820.7	116785.4	105836.3	95641.6	105419.4	94338.1	100980.0	86073.1
Q60605	Myosin light polypeptide 6	Myl6	151	16.9	4.65	1704598.7	1305705.8	1333203.5	1715135.0	1280670.7	1218154.4	1616528.3	2147050.7	1390267.4	1500033.5	543519.4	715834.7	542261.3	594138.3
Q8K4B0	Metastasis-associated protein MTA1	Mta1	715	80.7	9.29	128729.3	130534.6	126013.5	124349.3	132161.2	133822.2	109385.8	112433.8	122102.3	127536.7	430529.7	485438.4	392403.5	428527.2
O70250	Phosphoglycerate mutase 2	Pgam2	253	28.8	8.5	100485.0	140734.5	184920.0	260690.8	75175.7	122067.7	74732.9	108696.5	123672.8	101185.4	74856.6	113676.3	115713.4	163326.0
Q9JII5	DAZ-associated protein 1	Dazap1	406	43.2	8.56	316973.4	371038.3	318200.3	367553.3	344447.7	324509.7	329030.7	285751.4	332375.1	345968.1	289878.8	293887.7	286547.1	278979.7
Q9D0M1	Phosphoribosyl pyrophosphate synthase-associated protein 1	Prpsap1	356	39.4	7.2	238610.8	240731.5	279065.0	222678.1	276427.6	263119.3	231911.8	250872.7	239100.3	255100.2	305508.1	395573.3	295082.8	384841.3
Q3UQ44	Ras GTPase-activating-like protein IQGAP2	Iqgap2	1575	180.4	5.64	135712.8	164426.6	118797.7	151696.2	143745.3	133658.3	119906.5	140901.7	133014.9	129072.6	98762.7	115369.7	94004.2	102808.0
Q3UM29	Conserved oligomeric Golgi complex subunit 7	Cog7	770	86	5.38	130843.1	176246.5	153580.5	168957.5	156235.4	160595.5	141704.4	151973.3	156493.5	165519.7	217303.0	220978.9	202041.6	238421.4
P08228	Superoxide dismutase [Cu-Zn]	Sod1	154	15.9	6.51	384347.2	313814.4	365463.9	348336.3	431586.1	472231.1	408824.4	426667.1	377975.5	367126.9	923699.4	1446230.9	898675.6	1101241.7
Q8K1H1	Tudor domain-containing protein 7	Tdrd7	1086	122.1	6.7	535005.1	664123.4	624088.6	749304.9	523333.5	521733.0	463036.6	494333.9	550407.7	565946.0	423029.6	515498.3	465050.4	570924.6
P32507	Nectin-2	Nectin2	530	57.3	4.86	355470.6	416973.2	362872.3	385702.7	412355.9	419392.0	359816.1	407601.8	398266.7	437979.3	187441.0	214236.9	181753.6	180869.3
Q9CZR8	Elongation factor Ts, mitochondrial	Tsfm	324	35.3	7.06	126368.2	130985.6	113604.3	125984.8	128624.8	119928.7	114742.9	114423.1	127881.5	124994.9	211888.3	229720.3	201019.5	217396.7
P42230	Signal transducer and activator of transcription 5A	Stat5a	793	90.8	6.39	85227.5	91013.1	79114.8	89305.5	99262.9	99085.2	94536.1	98840.2	84374.7	93036.0	67832.7	80095.5	52100.2	53928.4
Q80W22	Threonine synthase-like 2	Thnsl2	483	54.2	6.39	123911.5	114352.4	98484.7	114751.6	119721.1	110343.6	120170.3	108038.4	123334.9	121015.7	146778.4	134258.7	120562.6	146397.4
O88477	Insulin-like growth factor 2 mRNA-binding protein 1	Igf2bp1	577	63.4	9.2	274556.5	284812.2	285513.1	270579.5	293507.9	267298.2	258845.2	262945.9	286492.3	327737.4	185797.5	268224.1	169602.1	210404.9
P23506	Protein-L-isoaspartate(D-aspartate) O-methyltransferase	Pcmt1	227	24.6	7.65	291871.5	365820.0	252384.7	328878.3	353140.5	317115.4	300718.9	272223.3	285545.9	304442.0	291849.8	522991.1	277261.1	447703.4
O70209	PDZ and LIM domain protein 3	Pdlim3	316	34.3	7.91	190364.6	198106.3	159046.9	202913.2	183428.7	197699.1	174551.9	201962.9	166220.1	178639.5	124490.4	144350.7	122389.2	118677.6
Q9EPK6	Nucleotide exchange factor SIL1	Sil1	465	52.4	5.27	378385.8	513241.8	446262.0	539782.7	406422.9	344994.1	251771.4	428350.0	424083.8	404643.3	193659.3	214722.2	190679.1	216869.2
Q9QYA2	Mitochondrial import receptor subunit TOM40 homolog	Tomm40	361	37.9	7.74	232286.5	217043.4	222478.6	220077.5	231354.8	224624.1	213917.6	207922.5	215377.8	204050.7	253348.0	263887.8	264012.5	271611.8
O70551	SRSF protein kinase 1	Srpk1	648	73	6.19	358329.0	387160.2	348088.2	371148.3	340295.1	362147.7	324176.1	314047.4	361189.6	376910.2	1145544.9	1121578.1	1136612.8	1150906.3
Q9QXD6	Fructose-1,6-bisphosphatase 1	Fbp1	338	36.9	6.57	82490.8	122065.0	156847.2	215519.4	61834.0	58848.5	52066.5	61202.1	73625.1	117035.5	206629.1	218001.8	217700.0	227802.9
Q3TIX6	Far upstream element (FUSE)-binding protein 3	Fubp3	575	61.9	8.38	685742.6	898489.3	613956.2	675738.9	697696.4	750998.8	627599.9	740411.2	666203.1	669511.5	324983.6	326509.5	279734.2	324891.3
Q8BUK6	Protein Hook homolog 3	Hook3	718	83.2	5.19	187503.5	227479.5	170865.4	187582.4	198951.6	197858.1	185956.9	170211.7	182236.5	206541.0	445186.7	437952.7	382590.8	402846.6
Q61136	Serine/threonine-protein kinase PRP4 homolog	Prpf4b	1007	116.9	10.23	221941.6	212607.1	233150.3	235260.0	225162.6	234756.3	205669.4	212327.9	229410.9	222351.0	284052.2	300833.2	349505.0	342479.2
P70195	Proteasome subunit beta type-7	Psmb7	277	29.9	7.99	1206928.0	1267405.1	1215625.5	1270159.6	1290888.4	1195181.5	1195873.4	1235912.4	1227809.4	1317883.8	489008.1	449695.5	511904.0	463124.2
Q63844	Mitogen-activated protein kinase 3	Mapk3	380	43	6.61	771297.4	695352.0	661115.7	661797.0	735085.7	738277.5	698572.6	698119.8	718899.7	683795.6	1139641.0	1144643.2	1001905.1	1044962.0
Q9CPU0	Lactoylglutathione lyase	Glo1	184	20.8	5.47	1335613.1	1170427.2	1251048.2	1115093.1	1326554.7	1340873.9	1405192.0	1255940.2	1213459.1	1231880.2	1551554.3	1103158.7	1351356.2	1091369.8
Q8R2Y2	Cell surface glycoprotein MUC18	Mcam	648	71.5	5.83	210055.5	230105.3	211099.4	190795.4	264863.9	256725.5	230188.6	221040.1	217163.2	211195.0	350542.9	370225.9	381275.4	352565.9
Q8C0L8	Conserved oligomeric Golgi complex subunit 5	Cog5	829	91.3	6.27	215908.9	235835.0	243638.1	261868.1	226638.4	218579.3	185207.4	226741.2	214244.9	219612.5	155508.3	180746.4	162582.4	177564.1
Q8CCK0	Core histone macro-H2A.2	Macroh2a2	372	40.1	9.69	305543.9	283949.4	308925.9	273129.3	323829.3	351498.4	395257.4	327664.4	317633.6	315116.8	747907.7	727031.2	665356.6	644118.9
Q6P9L6	Kinesin-like protein KIF15	Kif15	1387	160	6.05	111707.6	144387.3	121592.8	137606.3	121041.3	116556.9	92512.2	95007.3	131880.8	127714.5	107030.2	141681.9	140372.8	142160.4
P01029	Complement C4-B	C4b	1738	192.8	7.53	270485.1	339336.7	269660.9	335392.4	272168.7	295714.2	316870.3	281333.9	291928.2	316869.9	301563.4	297146.2	233142.8	257043.1
Q3URQ0	Testis-expressed protein 10	Tex10	928	105.1	9.16	518750.1	549566.8	499366.0	511383.9	521379.4	525175.1	490620.6	482122.9	507206.8	517229.2	134541.7	137181.5	152610.6	133726.5
Q8BY89	Choline transporter-like protein 2	Slc44a2	706	80.1	8.79	545309.6	508314.9	553439.2	539411.0	589518.8	582643.3	658615.1	551580.4	581973.8	580766.3	864711.0	781667.6	788541.4	778289.1
Q6NZF1	Zinc finger CCCH domain-containing protein 11A	Zc3h11a	792	86.4	8.13	254468.9	234013.0	257072.5	231330.7	309576.6	286241.4	284917.6	264059.5	262874.1	263375.4	280414.4	258189.6	290406.8	254109.7
O35134	DNA-directed RNA polymerase I subunit RPA1	Polr1a	1717	194	6.93	172289.2	182249.3	161639.5	162706.7	213681.2	199675.2	181463.8	169382.1	185216.8	196660.8	131483.3	146152.7	145384.9	142957.0
Q8BTS4	Nuclear pore complex protein Nup54	Nup54	510	55.7	7.02	81961.5	89906.0	97543.7	92949.6	95204.3	93954.7	89953.0	90351.5	84424.5	92801.8	195294.3	214238.5	219056.3	231030.7
Q8BGE6	Cysteine protease ATG4B	Atg4b	393	44.3	5.07	116008.6	132227.8	128060.3	123821.8	139204.6	139726.3	119328.9	141162.5	129619.4	127865.7	117178.3	125885.1	109569.0	131242.6
Q8K2T8	RNA polymerase II-associated factor 1 homolog	Paf1	535	60.5	4.65	318934.5	344936.4	368550.0	341416.6	403444.0	390510.9	348569.9	329550.7	382615.0	375245.0	156230.1	166832.6	151866.3	150578.2
D3Z7P3	Glutaminase kidney isoform, mitochondrial	Gls	674	73.9	7.99	139425.0	130009.1	120487.0	120451.8	145700.0	139031.2	119139.8	127729.4	119482.0	117659.0	78557.0	87309.2	81123.3	88470.8
Q8K1J6	CCA tRNA nucleotidyltransferase 1, mitochondrial	Trnt1	434	49.9	8.56	894187.8	943990.2	1001917.7	937193.1	1012929.5	1014524.7	923098.6	864107.0	915349.2	966158.5	359549.9	406164.7	410935.9	390775.1
Q9DBH5	Vesicular integral-membrane protein VIP36	Lman2	358	40.4	6.95	1159752.3	984621.0	1011386.0	1026698.0	928317.8	985553.8	845409.9	1131151.6	973563.5	951334.2	1173415.4	1171310.6	1123312.1	1186503.2
Q99KN9	Clathrin interactor 1	Clint1	631	68.5	6.25	188480.7	220492.6	213781.5	202414.7	215715.2	230844.2	177454.6	219606.3	187497.6	189646.0	359374.8	438068.6	370782.0	412119.3
Q9CZ42	ATP-dependent (S)-NAD(P)H-hydrate dehydratase	Naxd	343	36.7	7.77	408406.0	434772.4	405028.6	417852.2	423645.7	394598.4	385517.1	368731.0	434230.9	425838.1	442405.1	453270.4	408046.2	447981.0
P62281	40S ribosomal protein S11	Rps11	158	18.4	10.3	1863445.1	1513846.4	2025313.5	1502158.4	1736630.0	1723696.8	1253336.9	2092787.8	1550810.2	1459283.3	4027921.7	3593175.8	4164484.2	3635111.3
Q3UFY7	7-methylguanosine phosphate-specific 5'-nucleotidase	Nt5c3b	300	34.4	6.24	479551.4	471569.1	479077.2	482696.7	550074.9	529719.1	520559.3	477528.6	541521.7	485900.3	130404.4	123606.4	129741.5	114348.2
Q8JZX4	Splicing factor 45	Rbm17	405	45.3	5.82	158971.8	184357.6	172893.9	164715.2	191158.3	186463.9	165078.2	174479.6	178904.2	178696.8	319153.5	300477.3	311872.8	310309.9
Q4VA55	PWWP domain-containing DNA repair factor 3B	Pwwp3b	681	76.9	4.93	265526.6	229377.7	279432.8	243037.7	300362.4	248587.4	195513.6	310085.8	246743.8	265340.8	438713.4	394327.1	377953.0	361242.2
Q8BHD7	Polypyrimidine tract-binding protein 3	Ptbp3	523	56.7	9.13	112876.0	126120.7	116474.4	127800.9	117591.9	132479.0	136148.2	123055.2	129312.0	146529.3	94131.3	90479.7	87656.5	101296.4
P34152	Focal adhesion kinase 1	Ptk2	1052	119.2	6.62	384108.6	427785.2	424295.0	385991.9	465113.7	469760.9	375320.7	399648.9	432009.7	427054.6	280321.6	307502.0	283916.9	282025.2
P54728	UV excision repair protein RAD23 homolog B	Rad23b	416	43.5	4.83	443093.3	445120.2	432427.9	436908.0	430246.8	438421.6	400287.2	376158.9	416929.8	423736.8	464336.1	561976.5	475482.9	543179.5
Q9R0B9	Procollagen-lysine,2-oxoglutarate 5-dioxygenase 2	Plod2	737	84.4	6.89	476283.9	517955.0	362444.5	503682.8	416693.0	378673.6	353896.4	469589.4	425376.8	410561.9	324567.8	304630.5	273651.4	317622.9
Q61655	ATP-dependent RNA helicase DDX19A	Ddx19a	478	53.9	6.67	190279.3	183815.7	161035.9	201975.8	174403.0	164699.3	173595.7	163606.6	179486.9	190899.4	283215.3	299854.5	268944.5	302910.1
Q9Z110	Delta-1-pyrroline-5-carboxylate synthase	Aldh18a1	795	87.2	7.55	235458.7	260634.2	214000.6	241218.7	232872.2	228244.1	248789.1	246961.4	250100.4	246484.0	235639.3	231186.3	232638.9	210848.8
Q8BPN8	DmX-like protein 2	Dmxl2	3032	338	6.42	153371.9	187166.7	172491.3	174288.1	184836.5	161069.9	152533.1	152491.9	166497.7	175518.1	161238.6	173515.1	173005.8	175190.6
Q5SUF2	Luc7-like protein 3	Luc7l3	432	51.4	9.77	726431.4	781038.6	765275.4	801182.2	796728.6	775196.7	763438.9	664306.1	787869.7	772527.9	532184.3	501309.8	574132.9	523760.1
E9Q5E2	THO complex subunit 2-like	Thoc2l	1589	182.6	8.56	67797.9	78375.2	84024.9	87318.0	71116.7	71079.7	54715.0	64025.3	72660.5	80671.5	128205.0	155959.7	129566.3	125053.9
O55022	Membrane-associated progesterone receptor component 1	Pgrmc1	195	21.7	4.7	502210.5	456629.3	378730.6	469479.8	392101.8	408985.8	476038.4	433764.9	450991.2	401144.0	676614.8	657783.7	562359.9	745315.7
Q3UA06	Pachytene checkpoint protein 2 homolog	Trip13	432	48.3	5.86	577704.7	601236.8	573759.7	535352.6	646426.6	617183.9	552411.0	523917.1	596648.2	595738.0	364387.9	336119.9	364133.3	322803.5
P19426	Negative elongation factor E	Nelfe	375	42.5	9.38	83654.6	90760.3	105909.8	87515.5	80913.3	88567.3	73574.0	88468.5	93686.4	86674.1	139935.3	141996.2	147467.5	152703.2

O35658	Complement component 1 Q subcomponent-binding protein, mitochondrial	C1qbp	278	31	4.92	972618.6	898335.4	933382.6	853263.1	1002257.1	971066.7	950515.4	950222.1	935608.7	934299.1	228750.3	224678.2	215177.1	217660.3
P56960	Exosome component 10	Exosc10	887	100.9	8.29	173589.6	182638.8	196094.5	173037.6	208875.3	189565.7	176332.2	161628.8	185756.1	197101.4	220547.7	221138.1	220572.8	218774.9
Q8VEG4	Exonuclease 3'-5' domain-containing protein 2	Exd2	650	74.3	7.78	100661.1	117806.5	113945.7	124411.9	100830.7	102058.8	90618.7	95285.7	113030.6	116801.4	55592.2	60195.8	55623.8	58910.3
Q811U4	Mitofusin-1	Mfn1	741	83.7	6.51	47815.8	50288.2	55171.3	51193.3	50579.9	46101.9	38009.3	47107.8	49675.5	44325.8	180568.4	206681.5	209205.7	211582.1
Q9QZM0	Ubiquilin-2	Ubqln2	638	67.3	5.22	202158.4	184903.9	194214.7	166045.5	183414.2	179288.7	184324.5	193408.6	173732.8	188216.8	153560.3	159911.6	155776.7	165646.0
Q9Z329	Inositol 1,4,5-trisphosphate receptor type 2	Itpr2	2701	307.3	6.44	300120.6	328992.1	269379.4	294532.1	265173.2	285069.2	297628.9	258085.4	263464.4	279344.4	136684.6	120391.5	119120.9	112122.2
Q9WVE8	Protein kinase C and casein kinase substrate in neurons protein 2	Pacsin2	486	55.8	5.2	248290.6	274822.2	278534.1	236830.0	314442.8	375898.8	302095.1	292515.2	268684.0	273534.1	348022.3	351838.7	321022.4	361066.3
Q91XI1	tRNA-dihydrouridine(47) synthase [NAD(P)(+)]-like	Dus3l	637	71	7.97	189053.2	193725.1	193658.0	183664.6	203759.4	194243.3	206059.1	175627.1	196282.8	210853.9	225577.5	224989.6	228836.0	231967.8
Q80W54	CAAX prenyl protease 1 homolog	Zmpste24	475	54.7	6.95	375797.2	397737.0	373440.4	363449.6	364154.5	373016.0	381272.3	416670.7	373644.4	370712.4	453891.2	429276.7	382082.5	402978.7
Q8CCF0	U4/U6 small nuclear ribonucleoprotein Prp31	Prpf31	499	55.4	5.67	409303.4	405725.8	389432.8	423742.7	416567.0	399659.7	420433.5	388488.4	439631.7	448296.7	468862.9	487598.3	492781.9	487676.0
Q3TPX4	Exocyst complex component 5	Exoc5	708	81.7	6.71	328050.6	364542.1	327814.6	356008.7	349611.4	343145.9	319583.0	332299.7	342533.4	349154.1	212333.6	246399.2	182969.4	220523.7
Q922H2	[Pyruvate dehydrogenase (acetyl-transferring)] kinase isozyme 3, mitochondrial	Pdk3	415	47.9	8.82	317627.3	335511.7	314957.8	320715.5	319496.2	303312.9	338494.0	326554.4	350546.0	364045.6	762441.9	633929.6	781792.5	616525.4
Q9JIH2	Nuclear pore complex protein Nup50	Nup50	466	49.5	6.24	163022.6	152450.9	157687.3	159014.4	167477.4	166562.6	161293.7	139938.4	167041.1	163771.3	350400.8	326731.2	388589.0	336023.1
Q8VDR9	Dedicator of cytokinesis protein 6	Dock6	2080	233.1	6.8	150050.2	150520.1	155813.1	161457.8	153654.6	155651.2	159119.8	136331.2	154101.4	166771.0	159462.5	181035.9	176682.7	175961.0
Q3THK3	General transcription factor IIF subunit 1	Gtf2f1	508	57.2	7.01	157513.1	163298.9	165097.6	153404.6	173095.1	167397.6	150754.5	155933.8	155803.1	162785.9	217048.5	227772.2	232086.5	217877.3
Q8VD75	Huntingtin-interacting protein 1	Hip1	1029	115.1	5.43	122219.5	152045.9	112833.1	125007.3	128069.9	136158.1	119568.1	125655.0	119098.8	137080.7	373114.5	382139.5	349452.4	372766.6
P21279	Guanine nucleotide-binding protein G(q) subunit alpha	Gnaq	359	42.1	5.68	786542.1	624722.9	760720.0	632652.3	903758.3	906602.5	824102.8	831834.2	734665.2	697466.8	104929.1	109458.5	101801.5	104234.7
Q9DBD0	Inhibitor of carbonic anhydrase	Ica	700	76.7	7.25	218517.7	210644.0	163426.3	174771.8	146632.3	177582.8	213888.7	185216.1	195129.9	189110.7	263021.4	270951.6	273000.8	242159.4
Q3U186	Probable arginine--tRNA ligase, mitochondrial	Rars2	578	65.3	8.02	350759.4	384469.1	374788.6	395129.1	368490.1	338002.7	346497.8	329769.9	362586.6	379906.0	343197.9	320399.7	314662.4	354583.1
Q9DB05	Alpha-soluble NSF attachment protein	Napa	295	33.2	5.45	383082.7	473928.7	440884.6	466514.0	450190.4	447605.8	365128.4	431536.2	415190.2	387915.7	526001.1	519869.9	543633.9	534800.4
Q61335	B-cell receptor-associated protein 31	Bcap31	245	27.9	8.7	247044.9	254650.8	253836.1	240825.9	272678.3	246767.2	277060.3	258220.2	258829.6	270521.8	217322.0	225534.1	197375.6	237635.6
Q9D0I8	mRNA turnover protein 4 homolog	Mrto4	239	27.5	8.54	463256.4	548582.1	482667.6	522861.7	523398.9	474116.0	410254.5	429511.5	547117.0	548139.8	439150.4	527770.6	437358.0	460182.9
Q6PAM1	Alpha-taxilin	Txlna	554	62.3	6.74	227485.0	255728.5	317115.7	258455.9	324317.0	283492.0	260772.7	281990.1	270393.5	255010.9	141606.5	142204.4	140677.4	149735.7
Q62311	Transcription initiation factor TFIID subunit 6	Taf6	678	72.6	8.6	351606.2	361474.5	387318.9	369575.1	406257.7	408928.3	329179.8	310731.2	372866.4	372165.5	119200.9	140074.8	126548.6	131025.9
Q9EQF6	Dihydropyrimidinase-related protein 5	Dpysl5	564	61.5	7.09	487475.2	458373.9	434858.4	505453.2	396793.4	386809.1	335712.8	421739.5	412726.7	421651.5	209778.5	232568.1	190158.8	203684.3
Q8K448	ATP-binding cassette sub-family A member 5	Abca5	1642	185.8	7.2	122987.0	158385.2	162336.3	186233.7	138536.2	140698.9	114640.2	119620.1	141725.7	148352.0	101175.9	141334.3	128629.6	134824.3
Q3UHU5	Microtubule cross-linking factor 1	Mtcl1	1945	213.7	6.14	331567.5	286448.0	315615.3	286851.2	319743.3	324132.3	345092.7	287106.8	316121.1	319685.3	162741.5	212375.8	158002.0	174943.3
Q3TAQ9	WD_REPEATS_REGION domain-containing protein	Wdr36	899	99.7	7.11	242509.1	263062.3	256175.3	236160.9	276458.2	262755.7	225900.4	245172.9	247404.2	255812.5	86438.8	88288.8	81162.1	79193.3
Q8R127	Saccharopine dehydrogenase-like oxidoreductase	Sccpdh	429	47.1	8.6	647177.4	771664.6	824322.1	943093.9	553247.4	530486.3	435559.5	546490.7	633472.3	689416.4	888586.2	1162221.2	1147949.2	1440211.5
Q9QYJ3	DnaJ homolog subfamily B member 1	Dnajb1	340	38.1	8.63	563686.0	564899.2	571261.5	604507.0	567078.5	573340.1	562370.3	510441.4	568976.6	585285.7	561672.9	562720.5	629015.1	597256.1
Q9WV54	Acid ceramidase	Asah1	394	44.6	8.46	255171.6	221658.6	209783.5	242314.7	218276.3	215435.4	251368.5	218842.4	235520.6	231133.2	181984.2	197822.9	189263.0	198334.3
Q9D6J6	NADH dehydrogenase [ubiquinone] flavoprotein 2, mitochondrial	Ndufv2	248	27.3	7.4	187945.4	206366.2	174269.4	182700.3	186737.5	184784.9	170906.9	176245.3	188381.2	192532.0	117355.0	183879.3	158840.0	173983.6
Q9DBD5	Proline-, glutamic acid- and leucine-rich protein 1	Pelp1	1123	118	4.36	213317.2	210305.6	236560.6	218015.9	248415.6	233169.4	238573.3	220997.5	228762.3	235724.6	93977.2	109228.5	106807.4	96891.3
O88587	Catechol O-methyltransferase	Comt	265	29.5	5.83	108394.2	113624.5	97159.5	107945.0	104596.4	109730.9	124750.2	108167.9	101757.9	103341.6	216716.7	192027.5	187291.9	213155.7
Q9R0P3	S-formylglutathione hydrolase	Esd	282	31.3	7.12	504015.9	426037.9	433336.5	410070.8	482533.8	538812.6	519789.4	601663.6	455789.8	451210.7	1195671.4	1276553.8	1312628.9	1130422.3
F7BJB9	MORC family CW-type zinc finger protein 3	Morc3	942	106.5	5.68	97696.5	119875.3	101737.3	116728.6	111767.8	113390.7	111914.5	104257.6	120705.1	120456.6	80634.1	88364.2	74629.8	83962.6
P11440	Cyclin-dependent kinase 1	Cdk1	297	34.1	8.43	346126.1	443919.3	400759.4	420431.7	386447.5	319237.4	271087.1	330236.1	396132.0	383259.7	821564.8	979889.1	867983.5	952558.8
O35654	DNA polymerase delta subunit 2	Pold2	469	51.3	5.78	165638.3	164196.7	157369.6	168630.7	167931.9	168234.8	136057.9	146533.4	164707.8	165992.6	161242.8	187033.5	176674.1	171228.0
P62835	Ras-related protein Rap-1A	Rap1a	184	21	6.67	65297.7	55054.5	45463.3	57112.0	55678.6	65154.5	47370.0	35955.8	26864.9	30282.9	621124.7	588899.4	586452.9	593516.6
P14131	40S ribosomal protein S16	Rps16	146	16.4	10.21	3012993.4	2766565.7	3016396.3	2711256.5	3000729.4	2798046.7	2514969.2	2973531.7	2880254.2	2795255.0	2967193.7	3010310.8	3103976.6	2920831.5
Q7TSV4	Phosphoglucomutase-2	Pgm2	620	68.7	6.14	114296.6	115222.0	101761.1	102705.4	133288.8	145723.6	146815.7	126368.7	114805.2	120739.0	54917.2	45887.7	45555.0	42103.3
O70305	Ataxin-2	Atxn2	1285	136.4	9.55	146045.1	129115.8	133273.0	135357.3	140909.9	121356.5	113725.7	119889.0	137739.2	141422.8	128691.2	133448.2	125500.4	126800.9
Q8R1T1	Charged multivesicular body protein 7	Chmp7	451	50.6	5.22	63610.8	58994.6	57345.6	56733.2	60268.2	70002.3	54633.9	60841.9	53352.3	59583.0	92612.1	106664.4	86718.8	95715.5
Q9Z2N8	Actin-like protein 6A	Actl6a	429	47.4	5.6	190379.6	190347.9	217499.6	218119.3	225115.9	206770.1	202677.2	200405.5	197526.7	207869.1	348419.3	403794.3	399920.4	437181.8
Q6NXI6	Regulation of nuclear pre-mRNA domain-containing protein 2	Rprd2	1469	156.5	7.59	129817.6	144653.2	140507.4	133470.5	162907.0	161682.5	155074.2	138490.8	140651.7	141200.9	86665.7	88558.6	108268.7	89592.1
Q64012	RNA-binding protein Raly	Raly	312	33.2	8.84	950144.1	751323.5	1010942.5	737425.8	1178195.1	1001979.7	869483.8	984791.8	840744.7	874956.9	687073.7	620400.6	639014.1	711208.4
Q6PGL7	WASH complex subunit 2	Washc2	1334	145.2	4.77	58522.3	77357.5	55988.4	62590.9	58760.9	63464.0	65040.1	59431.2	65119.5	81232.0	74075.0	76643.0	68876.2	73710.7
Q61466	SWI/SNF-related matrix-associated actin-dependent regulator of chromatin subfamily D member 1	Smarcd1	515	58.2	9.25	248760.9	251604.1	253059.7	244096.4	272065.0	279222.8	245645.4	254359.9	260740.2	262155.1	122956.2	125912.0	105565.0	121045.0
Q6P8X1	Sorting nexin-6	Snx6	406	46.6	6.16	143339.3	195554.6	156843.8	156762.3	151884.4	157451.1	139851.7	169566.3	144304.2	140931.4	254365.9	275193.5	259780.5	270740.8
Q07133	Histone H1t	H1-6	208	21.5	11.71	147525.5	194275.6	205730.6	226046.3	131590.8	114452.1	92545.4	117542.0	151296.1	177243.9	265232.8	373246.4	393079.1	373700.7
Q9DAW9	Calponin-3	Cnn3	330	36.4	5.72	283256.3	277344.3	309977.4	269406.5	337279.7	365292.6	380448.6	318041.2	319568.6	306968.5	260642.0	221346.2	208684.6	207526.2
Q9R0N0	Galactokinase	Galk1	392	42.3	5.26	526992.2	531834.4	489267.1	521017.4	481067.3	489182.4	443763.0	490903.5	503423.8	543826.2	689773.1	714476.2	692451.9	681139.6
O08648	Mitogen-activated protein kinase kinase kinase 4	Map3k4	1597	179.7	6.43	275630.8	267854.0	228629.7	244792.3	247788.8	248907.2	238954.1	250041.4	257318.1	265728.5	120331.3	127523.8	115830.2	107157.5
P25976	Nucleolar transcription factor 1	Ubtf	765	89.5	5.76	311638.6	278011.6	311735.6	286229.9	331249.7	321636.0	347580.5	320452.4	315425.2	318680.0	444063.6	392671.0	400677.9	384626.8
Q3U3T8	WD repeat-containing protein 62	Wdr62	1523	167.2	5.48	221443.9	243502.2	209813.9	256110.2	212582.1	208951.9	196564.9	182117.9	213381.9	238582.3	245800.3	249849.3	271739.9	310192.3
Q9D0L7	Armadillo repeat-containing protein 10	Armc10	306	33.3	7.99	354212.9	339283.8	372458.8	335247.4	381258.3	393010.7	392287.0	370095.4	378783.8	354929.5	333354.4	323032.6	383838.5	340748.4
Q8VI36	Paxillin	Pxn	591	64.4	6.02	324850.1	297709.4	308499.5	303069.9	331134.4	327065.0	321384.9	305189.3	302796.1	308741.6	77427.8	76412.9	80152.6	74648.5
P39688	Tyrosine-protein kinase Fyn	Fyn	537	60.6	6.67	222713.5	218829.8	197757.7	213572.6	237713.3	230029.5	219864.5	209479.5	221567.7	222459.4	364591.7	377129.8	359084.1	358344.3
Q9EPN1	Neurobeachin	Nbea	2936	326.5	6.2	73832.1	78661.7	71246.0	78395.4	72271.4	84308.3	70347.9	73542.7	82763.5	78645.5	#N/A	#N/A	#N/A	#N/A
P62748	Hippocalcin-like protein 1	Hpcal1	193	22.3	5.5	490390.8	492098.6	459437.9	446474.2	563505.8	607283.5	594100.7	517966.0	523625.0	515275.4	403060.8	326344.9	379111.4	318629.5
Q9EPC1	Alpha-parvin	Parva	372	42.3	5.95	1074006.9	1172547.3	1112275.8	1098555.4	1314620.0	1463104.7	1459715.4	1256888.0	1200338.7	1273211.0	433314.3	420956.3	415519.5	412754.0
Q91YR1	Twinfilin-1	Twf1	350	40.1	6.67	386601.2	418743.7	380404.7	423514.9	393592.2	381722.9	358651.1	377210.2	402064.5	401457.4	798260.1	898697.0	759203.5	934897.7
Q9DBE8	Alpha-1,3/1,6-mannosyltransferase ALG2	Alg2	415	47.4	7.97	213795.8	258607.8	201583.4	234250.0	223740.0	208697.2	199770.6	207582.6	224218.0	230800.8	354056.9	348049.0	346908.7	338122.8
Q810D6	Glutamate-rich WD repeat-containing protein 1	Grwd1	446	49.2	4.81	124568.3	135651.5	119391.1	124517.9	119555.6	119538.6	113354.7	120620.1	125114.3	125363.0	239094.4	269186.7	262094.8	236218.5
Q8BZM1	Glomulin	Glmn	596	67.7	5.06	288023.6	317910.1	312803.8	316273.2	329840.1	295792.8	278194.0	274655.6	313130.7	345982.9	141731.2	148333.1	160188.7	154308.7
P97868	E3 ubiquitin-protein ligase RBBP6	Rbbp6	1790	199.5	9.63	225134.5	252900.9	252164.4	252033.5	260900.8	257566.8	244490.5	218352.9	252836.0	263521.6	259925.5	247612.4	259819.4	251589.1
Q9CYA6	Zinc finger CCHC domain-containing protein 8	Zcchc8	709	78	4.73	108383.2	133599.1	113122.2	120649.9	126871.2	119262.5	109437.3	96449.7	118985.8	129206.0	104672.0	107445.8	99149.8	103540.6
Q8K2F0	Bromodomain-containing protein 3	Brd3	726	79.7	9.36	409710.3	375478.9	381099.5	365893.9	411127.6	400357.9	461735.9	386630.5	385962.1	416084.7	584066.0	588975.3	605204.8	597892.4
Q8BH69	Selenide, water dikinase 1	Sephs1	392	42.9	5.97	313881.4	350686.4	292986.8	305964.4	347649.5	354671.8	331459.8	321320.8	332722.6	338035.1	361498.9	328136.3	326275.9	302526.4
Q9CW46	Ribonucleoprotein PTB-binding 1	Raver1	748	79.3	8.72	159146.5	165627.9	170759.3	170217.1	154698.5	159076.1	148167.5	166276.5	157800.8	152718.9	150463.4	147014.8	126379.9	133047.6
Q91X78	Erlin-1	Erlin1	348	39.2	7.21	104567.8	127651.5	124544.4	138784.6	124863.5	110975.5	99941.7	107784.2	115954.9	119687.9	246486.6	267662.7	287015.0	287052.3
P86048	60S ribosomal protein L10-like	Rpl10l	214	24.5	10.11	252198.1	391565.1	351889.4	442282.1	265885.5	211101.0	173503.5	235740.4	335978.0	354927.5	558918.6	672571.6	736485.5	715416.3
Q6TXD4	Dynamin-binding protein	Dnmbp	1580	177.2	5.41	106608.9	121381.9	94632.1	101565.8	103668.3	105891.7	101221.6	92595.1	99306.2	111841.2	59054.8	61089.9	50776.4	55397.7
Q8BVY0	Ribosomal L1 domain-containing protein 1	Rsl1d1	452	50.4	9.98	162985.3	160105.7	179143.9	155573.8	165294.4	162265.4	120797.0	147844.7	162059.8	161011.8	124615.7	139430.9	109974.3	121794.2
Q9CQQ7	ATP synthase F(0) complex subunit B1, mitochondrial	Atp5pb	256	28.9	9.06	598653.1	564755.8	676301.2	623717.0	691400.5	655046.6	556548.6	657053.3	616538.3	556554.9	700076.6	681985.4	724014.3	723060.7
O88986	2-amino-3-ketobutyrate coenzyme A ligase, mitochondrial	Gcat	416	44.9	7.33	170005.3	197336.7	160666.6	186375.6	196851.1	184085.3	173304.9	172938.1	194284.0	197100.2	313973.8	328799.8	333086.8	331125.5
A0A2I3BRL8	RRM domain-containing protein	Gm7324	388	42.2	9.99	611288.8	567127.0	572214.4	591059.9	604174.4	638213.0	693599.7	552374.6	600466.5	590632.5	834912.7	746591.8	817310.2	776170.0
Q8CG76	Aflatoxin B1 aldehyde reductase member 2	Akr7a2	367	40.6	8.12	1311894.4	1223178.9	1336946.7	1252291.4	1489487.2	1418988.1	1497574.3	1308956.3	1388326.0	1359718.0	974746.5	912183.9	1039812.7	964540.4
Q922Q1	Mitochondrial amidoxime reducing component 2	Mtarc2	338	38.2	8.68	886751.3	766364.6	784069.5	846207.5	946799.9	862239.3	981365.5	872464.3	762047.8	800619.7	1225930.2	1103686.2	1091287.8	1213296.3
Q8K268	ATP-binding cassette sub-family F member 3	Abcf3	709	79.8	6.16	135592.3	152644.6	132715.0	149754.6	142952.2	133454.9	119865.7	124318.0	146199.4	161578.3	297193.2	316688.4	290593.9	313066.5
Q69ZS7	HBS1-like protein	Hbs1l	682	75.1	6.46	129847.8	129234.4	132632.4	131284.2	133387.6	137252.6	114995.6	128574.3	126071.0	134286.0	141485.1	171835.6	152346.6	161924.9
D3Z3N4	Heterogeneous nuclear ribonucleoprotein H3	Hnrnph3	346	36.8	6.87	567816.7	676452.2	670997.9	632624.1	665805.8	629990.2	544318.4	578384.1	618104.2	636388.6	440572.6	473303.0	458754.9	448765.1
Q62376	U1 small nuclear ribonucleoprotein 70 kDa	Snrnp70	448	52	9.94	620692.7	629057.1	716219.3	660628.1	757019.5	746212.0	658962.5	645806.5	693242.4	666281.8	444495.7	447355.9	524765.4	478103.7
Q9D4J1	EF-hand domain-containing protein D1	Efhd1	240	27	6.02	237626.2	241892.4	231527.3	269861.8	168013.3	171771.9	193094.7	218097.3	197782.4	191405.7	356445.1	347820.5	324117.3	361559.6
Q91V64	Isochorismatase domain-containing protein 1	Isoc1	297	32	7.39	190807.1	182171.0	188127.4	169969.8	231979.0	215533.6	232081.7	200335.4	198576.6	200231.0	501792.7	381243.6	444128.5	417641.0
Q9CQD1	Ras-related protein Rab-5A	Rab5a	215	23.6	8.15	191715.8	210252.9	216135.1	202213.4	201740.8	208292.3	201068.3	198659.5	235690.1	230500.6	91237.5	110522.5	97571.0	94515.4
Q60770	Syntaxin-binding protein 3	Stxbp3	592	67.9	8.02	194286.0	191708.9	155019.2	195970.9	188775.3	200228.5	190104.1	160498.3	189418.1	179160.0	118726.3	125664.3	137626.5	102924.9
Q6GYP7	Ral GTPase-activating protein subunit alpha-1	Ralgapa1	2035	229.2	6.1	170380.9	213779.7	184881.5	190556.7	158483.9	180073.2	181175.5	160282.2	196799.5	202381.7	134024.9	129851.3	129178.0	124201.9
Q61550	Double-strand-break repair protein rad21 homolog	Rad21	635	72	4.64	247829.8	261828.0	266133.1	265809.8	309499.0	322424.0	312736.6	280069.6	270859.7	269146.3	119426.0	132757.2	111903.2	119004.4
Q8BVP2	L-lactate dehydrogenase	Ldhal6b	382	42	9.16	375640.4	573839.8	557739.4	800623.8	242924.7	217465.3	184661.1	302657.6	432136.7	463960.0	243365.1	365384.1	419656.3	625798.0
Q8CH02	SURP and G-patch domain-containing protein 1	Sugp1	643	72.6	7.64	103849.5	112277.4	107991.8	110985.7	134081.5	119141.9	97198.4	94380.4	105874.2	114602.2	165832.3	203114.6	170423.8	189928.7
Q9DBG7	Signal recognition particle receptor subunit alpha	Srpra	636	69.6	8.95	214329.0	228567.8	193853.9	198213.8	197889.4	217372.9	200038.5	177346.7	218916.7	222535.1	238874.9	253486.3	247707.1	261249.9

P86049	Probable RNA-binding protein 46	Rbm46	533	60	7.88	383333.9	444484.5	416104.7	441452.6	446976.6	432342.8	397510.0	447189.7	428455.6	459731.9	301779.3	329609.5	323976.6	327763.0
Q922J3	CAP-Gly domain-containing linker protein 1	Clip1	1391	155.7	5.24	413329.4	481851.9	448646.1	438770.9	486917.4	507737.1	506198.3	419915.4	454349.3	456444.0	246467.0	288837.3	232719.3	253624.2
Q923D5	WW domain-binding protein 11	Wbp11	641	69.8	8.4	228007.4	263180.6	259160.7	272737.2	261281.7	253728.2	244059.7	232775.8	259638.7	250389.0	205940.1	211230.1	236283.7	233658.9
Q923D2	Flavin reductase (NADPH)	Blvrb	206	22.2	7.01	388482.2	413865.5	369511.1	472378.3	345043.7	348861.0	386324.9	364838.1	397775.1	415858.2	490392.0	588170.4	455020.0	638389.0
Q9D0G0	28S ribosomal protein S30, mitochondrial	Mrps30	442	49.9	9.38	56780.7	71355.4	60211.5	64531.1	61139.6	62778.5	51757.7	65859.9	54393.1	55452.2	75334.3	94941.0	81985.4	84948.7
E9Q7L1	Urb2 domain-containing protein	Urb2	1524	171.5	7.34	195579.0	242951.4	212781.1	224700.2	209898.2	212957.7	176833.2	185077.4	220036.1	225428.4	136398.2	150255.1	142357.9	140251.1
Q9CZW4	Long-chain-fatty-acid--CoA ligase 3	Acsl3	720	80.4	8.54	274597.4	331118.8	370888.9	336757.5	336105.5	326094.8	284742.0	325590.7	320425.1	336737.0	296207.2	331314.5	351217.5	339444.3
Q8CEC0	Nuclear pore complex protein Nup88	Nup88	753	84.9	6.27	187657.2	191468.8	189199.1	174963.0	195855.9	196573.3	188528.4	175469.0	176301.9	190079.7	230365.8	334230.6	236788.3	248522.9
Q8CHY6	Transcriptional repressor p66 alpha	Gatad2a	629	67.3	9.89	159907.0	182739.3	141803.8	170059.4	163136.8	163313.0	145784.2	161338.1	168574.1	171368.5	95965.0	97139.4	96001.1	97904.7
Q3UPH1	Protein PRRC1	Prrc1	443	46.3	5.95	63593.6	72843.4	67226.1	76870.2	57156.0	64682.1	62451.9	65896.6	62598.3	67597.3	59461.9	75667.2	64221.6	76400.1
Q6IR34	G-protein-signaling modulator 1	Gpsm1	673	74.3	6.23	75216.0	81702.0	72512.8	83819.4	71627.4	77306.9	63993.4	61251.6	83846.8	77532.7	132223.5	121484.6	128992.8	126727.2
Q99LI8	Hepatocyte growth factor-regulated tyrosine kinase substrate	Hgs	775	86	6.16	230188.1	243770.2	230941.1	228354.3	235126.5	256058.0	225800.9	221918.8	241197.5	232309.9	129510.6	128031.3	116543.7	121961.3
Q8K2C7	Protein OS-9	Os9	672	76.1	4.84	337450.2	402800.1	352177.0	420390.9	354011.9	324232.0	337323.6	313349.2	366456.7	394495.4	132666.0	167490.8	140412.8	172510.4
Q8CI11	Guanine nucleotide-binding protein-like 3	Gnl3	538	60.7	9.11	209759.6	214571.0	191244.8	214218.4	196522.5	201594.9	185660.2	184437.3	205148.2	215525.6	73694.3	75091.3	86883.6	78431.0
Q921T2	Torsin-1A-interacting protein 1	Tor1aip1	595	66.7	7.05	218664.5	297325.7	230116.5	255026.1	301391.2	260106.4	347071.3	218146.4	301147.8	319042.5	213649.7	280686.2	200958.0	224907.9
Q4VBE8	WD repeat-containing protein 18	Wdr18	431	47.2	6.89	566634.9	528804.2	586545.8	531684.4	664137.5	668799.3	619360.7	627494.9	603681.6	651330.9	296513.8	272880.5	298826.7	288165.9
Q8BL99	Protein dopey-1	Dop1a	2399	269.1	6.24	89963.0	87330.5	98419.2	98039.8	105629.2	103846.7	100122.4	92756.4	103169.5	109525.7	70134.9	70049.6	80715.6	84720.2
G3X8X0	DEAH (Asp-Glu-Ala-His) box polypeptide 16	Dhx16	1044	119	6.61	312408.9	344036.5	320793.8	344340.8	355219.9	350838.2	335447.0	305914.1	321401.8	333861.5	113085.3	118764.5	131044.3	126788.8
O70422	General transcription factor IIH subunit 4	Gtf2h4	463	52.2	9.04	190199.1	214338.0	227885.5	219086.2	205441.1	203837.4	187681.9	174286.9	199592.2	228205.7	84098.7	81222.2	101369.2	95335.5
Q922K7	Probable 28S rRNA (cytosine-C(5))-methyltransferase	Nop2	793	86.7	9.22	490508.0	519934.0	489623.7	498973.8	521954.3	572876.5	507624.8	532920.8	512266.5	516169.6	250476.2	263102.0	256817.8	226467.3
Q9D0M3	Cytochrome c1, heme protein, mitochondrial	Cyc1	325	35.3	9.16	368744.0	385113.2	310733.7	365025.5	348849.9	360426.7	334180.6	342408.4	373464.8	352078.6	424093.6	588281.4	509709.2	621821.5
P49615	Cyclin-dependent-like kinase 5	Cdk5	292	33.3	7.66	298122.4	310403.9	302765.9	334109.6	344053.5	313334.6	366457.2	325736.5	329902.3	259671.1	1109328.6	1103016.2	1040316.1	1108692.6
P26350	Prothymosin alpha	Ptma	111	12.2	3.79	447700.5	387936.1	875764.0	289698.5	384386.6	698758.4	254683.5	777909.8	348588.2	247631.0	423149.4	284961.4	476361.6	318680.2
P11930	Nucleoside diphosphate-linked moiety X motif 19	Nudt19	357	40.3	6.68	253125.0	226430.9	267571.3	253277.1	257413.4	252068.7	238801.7	247400.5	250434.6	247713.9	154373.2	149935.1	167279.7	140790.5
P51655	Glypican-4	Gpc4	557	62.5	6.33	464885.0	404551.3	440131.1	419382.2	461406.7	472280.1	510239.6	402504.3	443463.5	402112.7	480234.5	482081.7	504747.2	479624.5
P01869	Ig gamma-1 chain C region, membrane-bound form	Ighg1	393	43.4	6.44	229290.1	205996.9	241177.0	244400.0	284102.4	225986.6	232936.0	238673.5	240155.7	233041.0	294118.7	281129.4	299884.2	286535.6
Q91W89	Alpha-mannosidase 2C1	Man2c1	1039	115.6	6.51	208791.5	238102.2	232045.2	237898.1	240327.5	235399.2	236800.4	223282.7	221302.7	239275.6	141664.1	117302.0	123464.5	141483.7
Q9JJU8	SH3 domain-binding glutamic acid-rich-like protein	Sh3bgrl	114	12.8	4.92	270912.6	274409.4	282605.9	285023.6	303924.8	348199.5	374142.8	331022.9	323737.9	288388.7	155768.2	158894.5	140248.9	155420.2
P68181	cAMP-dependent protein kinase catalytic subunit beta	Prkacb	351	40.7	8.56	91739.6	80702.5	86079.6	87322.0	86908.1	83912.9	78779.5	99359.9	86583.9	86440.6	336040.0	294641.8	283759.7	281117.8
Q8R5L3	Vam6/Vps39-like protein	Vps39	886	101.6	6.99	121760.0	132740.7	123970.1	131152.7	133150.3	130422.3	127653.3	128535.0	129733.5	129965.6	146129.3	152926.4	130913.2	144646.9
P21278	Guanine nucleotide-binding protein subunit alpha-11	Gna11	359	42	5.97	483463.2	422839.4	390288.9	443362.5	389126.4	368541.3	384869.1	374178.7	432879.6	410197.0	800441.9	721973.8	823041.8	709299.0
Q64152	Transcription factor BTF3	Btf3	204	22	9.52	241609.3	234746.4	220531.1	216863.3	235446.6	239119.9	247197.3	237084.1	257742.7	246709.1	772726.5	716284.0	727566.3	721010.5
Q6NZB0	DnaJ homolog subfamily C member 8	Dnajc8	253	29.8	9.06	626023.7	607905.7	626113.8	647978.6	602923.5	599244.7	685094.4	573092.7	651151.9	649730.6	800307.4	990427.7	856128.5	876758.3
Q6NZN0	RNA-binding protein 26	Rbm26	1012	114.1	9.16	139971.9	151379.4	147320.3	142531.3	150598.1	151690.3	136582.0	141230.7	145532.8	141583.4	436267.3	395031.1	417880.5	414014.2
Q8VE88	Protein FAM114A2	Fam114a2	497	54	4.93	274971.1	322877.5	313484.9	310760.1	295614.5	268441.5	248001.2	258051.3	297478.5	324317.3	193465.6	210400.9	211226.5	200041.9
E9PZJ8	Activating signal cointegrator 1 complex subunit 3	Ascc3	2198	250.4	7.02	519300.1	469063.3	505531.5	533286.3	469146.8	531511.7	482281.0	473441.7	539652.0	513600.5	255559.1	283957.3	249297.1	278531.9
Q3TMX7	Sulfhydryl oxidase 2	Qsox2	692	77.7	8.59	104100.9	103522.4	107519.9	87178.7	96066.9	91622.7	86509.1	97841.3	91172.5	102093.2	205439.8	216845.4	200444.0	209791.0
Q3UVK0	Endoplasmic reticulum metallopeptidase 1	Ermp1	898	100.1	7.49	128398.7	137800.6	124306.8	123546.0	132078.8	124028.5	115199.5	121245.6	125044.5	122621.4	145951.3	167293.0	143746.5	146431.7
Q8BJ05	Zinc finger CCCH domain-containing protein 14	Zc3h14	735	82.4	7.37	98148.7	108931.6	116290.4	122746.9	100624.6	103128.5	94446.4	99157.3	108199.2	110956.9	62847.7	38838.5	58190.5	43199.3
F8VPK0	Tetratricopeptide repeat domain 37	Ttc37	1563	173.8	7.12	116820.9	133482.4	132921.8	135503.1	125959.2	131443.6	129288.4	125689.5	123233.9	134657.0	102020.3	108443.1	106744.2	109662.6
Q9D394	Protein RUFY3	Rufy3	469	53	5.49	196023.3	206656.5	175216.9	216646.3	188535.1	231189.0	176352.1	172379.1	199410.2	196251.7	206706.5	191446.3	182307.6	192521.4
Q8QZS1	3-hydroxyisobutyryl-CoA hydrolase, mitochondrial	Hibch	385	43	8.06	308435.5	330670.4	264961.6	319369.2	295028.1	300586.3	301154.9	274294.8	306151.7	303184.6	381392.0	384381.1	339727.2	357206.0
Q9Z1G4	V-type proton ATPase 116 kDa subunit a isoform 1	Atp6v0a1	839	96.4	6.76	99273.0	145642.2	79632.6	112391.7	152800.1	124051.6	107290.3	130513.2	108519.8	120179.7	240650.4	248532.9	286540.5	258379.2
Q8R3S6	Exocyst complex component 1	Exoc1	894	101.8	6.52	332985.4	299190.3	316107.8	297477.0	358533.9	318890.7	308177.5	358729.3	316093.9	285355.0	512375.7	490179.8	444384.1	452265.1
P54320	Elastin	Eln	860	71.9	10.43	45724.0	50997.7	49296.4	47056.0	53846.1	52814.4	51649.5	54926.7	51899.9	51425.8	26779.7	32052.9	26670.4	24352.6
P50136	2-oxoisovalerate dehydrogenase subunit alpha, mitochondrial	Bckdha	442	50.3	8.06	65514.0	69325.3	65170.7	64330.9	69227.2	70531.6	80444.9	75612.5	72100.7	65532.9	49918.3	50002.6	41705.3	40253.6
P35486	Pyruvate dehydrogenase E1 component subunit alpha, somatic form, mitochondrial	Pdha1	390	43.2	8.19	309499.0	280055.6	273425.1	270950.2	296952.9	313160.7	320599.5	313742.8	277854.2	292962.6	237162.1	230549.9	231477.3	236678.9
Q8R151	NFX1-type zinc finger-containing protein 1	Znfx1	1909	218.7	7.47	111996.1	149496.9	125499.6	132110.5	132683.9	169330.5	114771.4	160239.3	138587.4	147663.9	112772.3	97272.8	93403.6	103845.0
P62889	60S ribosomal protein L30	Rpl30	115	12.8	9.63	676162.6	725826.8	704727.7	704624.1	738898.3	711144.2	615480.6	714050.4	687845.4	686398.5	1189662.7	1203287.3	1290089.2	1226212.8
Q8K4Z3	NAD(P)H-hydrate epimerase	Naxe	282	31	7.69	504632.7	569293.0	543499.0	596942.6	537038.3	519375.6	546485.2	507494.2	571225.1	584826.6	419641.1	466828.7	503223.9	485113.0
Q6NVF9	Cleavage and polyadenylation specificity factor subunit 6	Cpsf6	551	59.1	7.15	258640.3	292072.9	269300.1	265030.3	311689.8	307654.2	254578.2	275525.2	255783.4	279116.0	231960.0	267735.6	295974.8	270586.8
P62827	GTP-binding nuclear protein Ran	Ran	216	24.4	7.49	3028406.8	3301223.7	3832593.5	3506246.2	3103248.5	3001424.9	2782629.0	3175671.2	3344738.0	3248950.9	4545573.7	4821363.6	5444126.1	5247857.3
O09159	Lysosomal alpha-mannosidase	Man2b1	1013	114.6	8.13	166762.5	163008.5	155115.9	172500.9	182876.4	173243.1	169484.5	181303.1	173968.0	159993.3	165283.0	168516.2	175576.3	172873.9
Q9D0L8	mRNA cap guanine-N7 methyltransferase	Rnmt	465	53.3	6.48	151331.7	192412.3	152297.1	163231.4	200386.3	192988.1	149930.4	163441.8	171310.2	183934.0	166140.1	153789.1	169125.4	171056.4
Q3UTJ2	Sorbin and SH3 domain-containing protein 2	Sorbs2	1180	132.3	8.53	105146.8	105154.4	101437.3	96073.6	110709.9	123822.9	131729.3	122191.3	109087.6	104154.6	287885.8	256620.2	278009.1	250220.0
Q923T9	Calcium/calmodulin-dependent protein kinase type II subunit gamma	Camk2g	529	59.6	7.58	655803.8	680137.9	654760.7	692196.3	693145.1	671922.9	672679.2	680085.9	673189.3	649340.9	549777.0	539863.1	543781.2	576338.6
Q8CBE3	WD repeat-containing protein 37	Wdr37	496	55	7.23	490586.3	540620.4	483114.6	514381.2	498680.3	485808.1	435054.6	461800.7	488925.2	529107.2	813106.1	795867.0	751232.0	782053.0
Q91XL9	Oxysterol-binding protein-related protein 1	Osbpl1a	950	107.7	6.44	212177.9	195659.1	190499.2	191954.7	188870.1	200052.0	192640.2	179703.6	179662.0	199270.0	148543.0	150873.4	141891.1	154032.8
Q9D1M0	Protein SEC13 homolog	Sec13	322	35.5	5.38	242940.4	345645.0	258131.8	358478.1	271949.3	280177.4	317187.7	261013.8	322634.6	350823.8	348337.7	331474.3	358023.3	343188.0
P70697	Uroporphyrinogen decarboxylase	Urod	367	40.7	6.65	109527.4	124011.0	108750.2	136247.8	129220.0	125620.1	113537.3	123192.2	132931.0	122450.6	450375.2	433850.1	394488.7	445387.8
Q9D6R2	Isocitrate dehydrogenase [NAD] subunit alpha, mitochondrial	Idh3a	366	39.6	6.73	797114.4	791418.4	800879.7	815404.7	818708.9	829376.1	826330.8	792311.3	909242.6	900882.2	988634.6	920791.9	925916.2	916390.5
Q9JHJ0	Tropomodulin-3	Tmod3	352	39.5	5.14	330468.8	380533.9	324876.5	365653.9	357211.0	375985.9	383722.5	326335.5	355322.3	354535.3	246828.0	262344.1	240849.6	251891.1
P48024	Eukaryotic translation initiation factor 1	Eif1	113	12.7	7.44	206626.3	194573.4	203435.9	207155.7	201093.6	207482.5	197865.5	216110.3	214928.7	215612.2	175909.4	199304.7	200264.3	184303.2
A2A4P0	ATP-dependent RNA helicase DHX8	Dhx8	1244	142.5	8.12	143206.0	164195.4	152361.9	148920.9	157588.0	176796.9	155087.5	127148.7	149221.6	152320.2	207690.7	234663.4	225694.4	224977.6
Q60739	BAG family molecular chaperone regulator 1	Bag1	355	39.7	8.46	646952.4	570189.2	701832.4	533821.5	685645.7	680118.5	627779.5	660169.3	592988.2	621345.4	673031.9	771269.6	634083.8	678326.8
P19639	Glutathione S-transferase Mu 3	Gstm3	218	25.7	7.77	174736.4	176419.3	187826.9	160811.8	196391.0	157560.6	212158.5	153473.2	179779.3	192691.8	345190.9	308588.3	359821.5	278498.7
F8VPP8	Zinc finger CCCH type-containing 7B	Zc3h7b	982	110.2	7.42	232129.5	213018.7	211828.9	195849.4	238981.5	234955.5	237811.5	227069.4	234712.5	221408.5	152935.9	162345.9	144790.4	140346.9
Q8BYJ6	TBC1 domain family member 4	Tbc1d4	1307	147.4	7.33	195174.3	188358.6	177387.5	178456.7	184017.2	174736.0	208750.0	176679.2	183749.1	183362.2	172785.8	177337.3	166679.4	163319.3
P47802	Metaxin-1	Mtx1	317	35.6	6.18	209537.8	213012.5	223728.5	198474.7	217888.0	224042.7	218390.0	228266.3	212571.0	215410.1	234163.1	213310.1	239854.0	252687.6
Q8C3R1	BRCA1-associated ATM activator 1	Brat1	822	89	5.31	361508.9	294194.0	330681.8	309423.3	395422.4	362406.9	384925.4	299053.1	329097.1	318876.4	247774.7	235377.1	242705.7	242189.5
Q8BG51	Mitochondrial Rho GTPase 1	Rhot1	631	72.2	6.49	332809.5	358442.5	348346.4	335353.4	337079.4	352743.6	334046.0	317306.3	334864.2	354918.5	159596.5	173408.5	186681.0	192377.1
Q9JIW9	Ras-related protein Ral-B	Ralb	206	23.3	6.62	371929.6	427776.3	354137.0	379851.1	410916.5	403224.9	390119.9	391664.4	374822.1	402556.5	727206.2	683697.0	701509.9	691931.6
Q8K284	General transcription factor 3C polypeptide 1	Gtf3c1	2101	237.3	7.25	378352.7	400442.9	332295.2	373434.6	370209.8	389062.6	365057.0	326401.4	383285.7	381400.9	308920.9	276360.1	289734.8	268180.9
Q91Z49	UAP56-interacting factor	Fyttd1	317	35.9	11.84	357730.0	487352.6	464377.2	563784.2	396179.0	342578.1	288981.1	358131.3	443222.7	451717.5	610842.8	697864.6	606408.4	698208.1
P51954	Serine/threonine-protein kinase Nek1	Nek1	1203	136.6	5.45	233808.9	241150.9	274389.5	250104.3	264843.2	244878.8	231874.0	219589.7	248963.8	270942.9	131353.2	139535.7	139506.1	131565.8
Q61749	Translation initiation factor eIF-2B subunit delta	Eif2b4	524	57.6	9.25	284174.0	311710.8	318020.9	303477.4	314710.7	300138.7	267981.8	289723.5	295799.5	313679.4	41618.3	38706.6	40836.6	38942.0
Q8R5C5	Beta-centractin	Actr1b	376	42.3	6.4	20344.1	19480.5	24008.9	22373.6	24407.9	20824.5	22536.2	25768.0	21785.9	17778.8	20652.9	25513.1	25146.0	24806.2
Q8BH61	Coagulation factor XIII A chain	F13a1	732	83.2	5.92	193636.2	190064.7	143882.5	178932.2	175411.7	190325.8	162764.9	175120.0	165591.4	165568.3	1161965.5	1015384.0	975570.1	1125318.4
Q9WV02	RNA-binding motif protein, X chromosome	Rbmx	391	42.3	10.05	25404.4	31186.9	27298.7	31451.3	27947.9	29770.0	25907.4	28182.6	26026.6	27375.9	425054.0	549331.7	436902.3	513216.4
Q9Z2D6	Methyl-CpG-binding protein 2	Mecp2	484	52.3	9.96	269051.7	238583.7	253208.2	252193.2	305946.3	317269.9	407769.5	268070.1	282988.4	259215.7	177726.3	173044.3	175475.7	177894.8
Q8VCM7	Fibrinogen gamma chain	Fgg	436	49.4	5.86	673548.0	742250.5	518691.0	649136.1	428654.0	465028.0	722847.2	626453.4	659720.7	682537.4	990892.9	1197688.3	754541.8	997449.1
Q9JJA4	Ribosome biogenesis protein WDR12	Wdr12	423	47.3	5.6	228908.5	335998.8	281738.9	337475.2	282536.3	278038.8	263079.5	259106.9	322249.1	355161.5	305552.5	320627.3	324276.0	326648.4
Q80YQ2	Mediator of RNA polymerase II transcription subunit 23	Med23	1367	156	7.31	138574.9	149485.6	136113.4	129144.7	143442.3	165637.4	149917.2	122436.9	139738.8	168292.8	75084.9	77189.6	84348.8	74639.0
Q8VBZ3	Cleft lip and palate transmembrane protein 1 homolog	Clptm1	664	75.2	6.3	167749.5	211858.9	163572.5	174812.9	182365.6	193073.6	163783.8	193625.8	176194.3	194529.9	106379.9	111756.6	108123.7	97189.1
Q99LD4	COP9 signalosome complex subunit 1	Gps1	471	53.4	6.84	347460.3	400826.3	397418.6	432824.8	363587.8	366528.7	333383.2	349254.8	387992.6	373160.1	557343.1	624398.1	673834.5	641799.7
Q9WUK2	Eukaryotic translation initiation factor 4H	Eif4h	248	27.3	7.23	167774.9	200284.8	218930.2	238912.1	158504.6	173290.4	161796.7	155196.0	175175.1	203174.1	270677.3	268198.0	309735.8	316319.4
Q61026	Nuclear receptor coactivator 2	Ncoa2	1462	158.4	6.68	207318.5	211096.1	192000.6	205432.0	198812.9	197667.2	203091.0	190561.2	198521.1	206593.8	83790.6	77986.1	66820.5	65819.5
P02535	Keratin, type I cytoskeletal 10	Krt10	570	57.7	5.11	1525307.8	1754287.9	1715421.7	2349627.3	2936227.1	3785344.7	2261208.0	2207119.2	2431790.9	2094357.5	2023590.5	2134093.3	2092826.2	2617704.3
Q3UHJ0	AP2-associated protein kinase 1	Aak1	959	103.3	6.7	100827.3	103721.2	116517.0	94833.7	111414.4	109778.8	100424.3	104797.5	110568.7	115055.5	140879.6	145546.6	142375.5	148058.8
Q8BMJ3	Eukaryotic translation initiation factor 1A, X-chromosomal	Eif1ax	144	16.5	5.24	584083.2	679254.7	595263.2	597356.8	638743.7	656251.8	625436.6	602191.5	624922.4	631589.3	148022.7	149917.6	139223.4	147935.6
P62270	40S ribosomal protein S18	Rps18	152	17.7	10.99	3035376.0	3171080.1	3036055.2	2990694.9	2883375.1	2913396.4	2682342.2	2964428.2	2983288.1	3065271.0	1836776.3	1841666.3	1910671.4	1824611.6
B1AR69	Myosin, heavy polypeptide 13, skeletal muscle	Myh13	1938	223.4	5.57	7047.0	8996.3	8426.9	8007.2	7379.3	7010.9	8147.3	7291.3	7148.7	6329.9	#N/A	#N/A	#N/A	#N/A

Q99LC8	Translation initiation factor eIF-2B subunit alpha	Eif2b1	305	33.8	8.32	372944.8	405075.2	376399.9	349358.4	408090.7	380308.9	334554.3	388021.7	400391.8	424497.4	268284.6	272832.2	245967.6	243992.4
Q8R480	Nuclear pore complex protein Nup85	Nup85	656	74.7	5.57	190543.5	208484.6	192818.6	191595.8	220874.4	221046.7	203926.5	187797.3	189448.0	197338.4	140222.6	139722.2	134497.2	131200.5
P35235	Tyrosine-protein phosphatase non-receptor type 11	Ptpn11	593	68	7.3	381168.0	374784.0	357002.0	369045.7	375250.6	402533.7	387539.8	387908.0	372101.7	372409.0	246749.6	251151.7	233212.1	241122.7
Q8CHC4	Synaptojanin-1	Synj1	1574	172.5	6.89	144206.7	149703.4	149177.4	144255.5	135800.1	142743.6	127001.3	118864.7	204970.6	145296.4	198351.8	199294.1	219158.0	209120.3
P63328	Serine/threonine-protein phosphatase 2B catalytic subunit alpha isoform	Ppp3ca	521	58.6	5.86	137208.5	146537.3	141847.6	156456.5	153240.6	162197.0	145409.2	156658.3	143807.0	149976.9	391652.1	345880.4	383318.1	349617.6
O08901	Mitotic checkpoint serine/threonine-protein kinase BUB1	Bub1	1058	119.5	6.1	360758.4	405577.3	363175.4	388985.6	391927.5	433001.7	335705.0	323339.0	398689.9	411672.4	333404.1	375795.6	366250.9	355754.9
O35465	Peptidyl-prolyl cis-trans isomerase FKBP8	Fkbp8	402	43.5	5.16	333581.9	322040.8	338618.3	327749.7	353371.6	317109.5	323034.5	310425.9	314350.2	324533.1	265341.5	277991.5	260923.6	264973.7
Q8BIK4	Dedicator of cytokinesis protein 9	Dock9	2055	235.2	7.25	108855.7	104142.0	110150.7	96332.0	120633.3	128618.1	122994.4	107284.5	103889.9	108983.2	229831.5	228191.1	215358.3	208169.4
Q8K296	Myotubularin-related protein 3	Mtmr3	1196	133.8	5.83	239438.1	220778.0	226420.2	238690.8	231885.8	230484.1	232360.5	202396.6	226969.4	249880.8	118862.5	129784.5	116600.6	114279.6
Q9Z2G6	Protein sel-1 homolog 1	Sel1l	790	88.3	5.57	71716.6	88094.6	82165.0	92353.1	77990.1	84694.4	76024.4	69517.5	79967.5	82033.8	149663.1	133873.9	161001.3	136147.0
Q9QYS9	Protein quaking	Qki	341	37.6	8.56	398917.1	367885.1	363423.6	368742.7	386835.1	397266.0	374154.6	348239.8	384932.7	399904.1	329743.9	343984.2	324947.3	345430.7
Q9WTP6	Adenylate kinase 2, mitochondrial	Ak2	239	26.5	7.39	347610.9	353693.9	373782.5	405086.2	359098.9	403143.2	341198.7	330051.1	364942.4	370986.8	383629.1	400361.2	389110.5	410240.6
Q03147	Cyclin-dependent kinase 7	Cdk7	346	38.9	8.47	226323.7	250320.3	269949.8	249245.4	256180.8	247600.2	231070.6	221244.5	254510.3	260789.9	196968.6	189772.1	205188.1	212140.2
A6H630	Damage-control phosphatase ARMT1	Armt1	439	50.5	5.92	694621.6	866580.3	1091413.6	1228716.1	503773.7	440085.2	366233.9	554009.0	747424.1	827049.4	517283.6	720181.0	681272.8	758479.2
Q9QXL2	Kinesin-like protein KIF21A	Kif21a	1672	186.4	6.27	499682.8	390870.6	367728.9	379087.8	329728.0	304134.0	325012.0	407992.2	350257.5	355646.9	204421.5	178701.4	185920.4	180922.1
P50518	V-type proton ATPase subunit E 1	Atp6v1e1	226	26.1	8.43	151025.6	158347.3	143375.0	171286.8	167461.6	156851.2	185878.4	143562.4	157838.6	158131.0	398502.5	449355.5	423265.1	468128.7
P36916	Guanine nucleotide-binding protein-like 1	Gnl1	607	68.7	5.68	391815.6	494236.9	400509.4	454905.2	447968.7	460029.7	349056.8	402657.6	418854.0	435312.9	210458.9	237059.6	207955.8	245885.6
Q60960	Importin subunit alpha-5	Kpna1	538	60.1	5.01	225323.1	217947.8	229330.5	221152.4	239010.2	249710.0	210695.1	238684.6	242708.1	224136.1	248229.4	291162.2	287569.3	282401.2
Q8BGC4	Prostaglandin reductase-3	Zadh2	377	40.5	7.42	320844.6	336094.6	253460.6	303189.3	315816.6	327839.1	254672.9	314828.1	301030.2	301640.9	280712.6	421197.5	266078.2	326623.0
P35505	Fumarylacetoacetase	Fah	419	46.1	7.18	150183.9	185346.5	148413.3	173395.3	164772.8	184038.2	212396.2	190668.1	172300.7	172672.2	243315.7	302866.7	216261.7	265898.8
Q9Z2M7	Phosphomannomutase 2	Pmm2	242	27.6	6.42	282308.2	289481.4	261040.7	244208.0	311658.8	277209.8	375907.9	242230.2	274259.2	304356.3	207992.3	183679.9	196442.2	175567.8
Q99LC3	NADH dehydrogenase [ubiquinone] 1 alpha subcomplex subunit 10, mitochondrial	Ndufa10	355	40.6	7.78	456498.9	500629.7	464494.5	480244.3	475183.7	515018.1	454294.8	452472.9	458136.9	466494.5	381621.1	409664.3	377821.8	423539.8
Q9QZD9	Eukaryotic translation initiation factor 3 subunit I	Eif3i	325	36.4	5.64	551197.4	617269.5	690364.1	677472.3	602650.9	547296.3	554219.5	607857.1	594248.2	642314.5	428050.0	437949.8	504796.8	473146.8
Q8BWQ6	VPS35 endosomal protein sorting factor-like	Vps35l	963	109	7.31	259110.3	304198.6	263907.6	314253.9	293819.7	305856.3	305191.4	246931.9	285018.1	303550.8	79697.6	80750.7	86626.1	79042.9
Q8BHC4	Dephospho-CoA kinase domain-containing protein	Dcakd	231	26.5	9.58	634447.6	660850.5	618026.0	688334.4	689442.7	708352.1	670353.3	654227.9	697610.1	667693.7	671030.8	696204.8	657547.6	670973.2
G5E895	Aldo-keto reductase family 1, member B10 (aldose reductase)	Akr1b10	316	35.8	7.28	523657.8	516515.2	466134.2	493178.8	534498.4	549476.6	609268.3	517782.4	498354.7	497775.2	519431.3	455432.0	393866.4	451009.0
Q920A5	Retinoid-inducible serine carboxypeptidase	Scpep1	452	50.9	5.66	637209.1	682397.4	558961.2	682159.4	551622.3	619886.1	692364.8	629689.3	620250.9	604488.2	391350.2	416604.8	337977.6	398181.4
B1AUN2	Eukaryotic translation initiation factor 2B, subunit 3	Eif2b3	452	50.5	6.74	325051.9	335797.8	359145.8	328644.4	362988.0	373958.0	349477.2	315288.4	352975.6	356142.2	271941.4	305647.2	281263.4	291663.2
Q80V62	Fanconi anemia group D2 protein homolog	Fancd2	1450	163.5	6.32	126281.6	152006.0	131509.4	136092.8	148185.5	139943.8	136375.2	130437.7	136541.7	153729.1	192482.6	207756.2	190371.1	203351.6
Q9CPP6	NADH dehydrogenase [ubiquinone] 1 alpha subcomplex subunit 5	Ndufa5	116	13.4	8.1	429925.0	426451.3	422774.3	432103.0	465248.6	391580.1	409993.9	403038.4	447743.9	434227.5	252758.1	282332.0	271170.8	314835.9
Q9DCH4	Eukaryotic translation initiation factor 3 subunit F	Eif3f	361	38	5.58	377676.8	391243.6	380523.4	378768.5	371869.2	354631.3	335807.3	344105.1	368680.5	378211.0	330692.2	329203.3	368378.2	341643.6
Q9CU65	Zinc finger MYM-type protein 2	Zmym2	1376	154.5	6.37	225958.1	249661.4	249180.9	268400.5	240065.3	257126.4	230050.1	242624.0	234265.8	255066.8	161310.7	177539.2	187706.2	173134.0
Q9CS42	Ribose-phosphate pyrophosphokinase 2	Prps2	318	34.8	6.61	207438.4	210647.8	201090.9	211634.7	187623.4	190738.9	204428.0	205331.5	221732.2	210804.4	221129.2	236300.2	204938.9	230126.9
Q80X41	Serine/threonine-protein kinase VRK1	Vrk1	440	49.7	8.94	262104.2	303800.7	297121.6	309748.7	302391.9	292896.8	263275.3	255420.4	287865.3	297539.9	619096.9	690648.3	738120.4	725561.9
O88848	ADP-ribosylation factor-like protein 6	Arl6	186	20.9	8.25	326233.0	373461.9	357854.2	366903.7	349933.4	341573.7	319957.8	314311.8	339187.4	354866.2	299188.5	311350.8	317170.1	321145.8
O35469	3 beta-hydroxysteroid dehydrogenase/Delta 5-->4-isomerase type 6	Hsd3b6	373	42	6.35	953458.9	945940.7	504695.6	1340221.5	404092.1	384152.1	382551.5	579299.7	477461.5	453312.0	787508.6	809703.4	418039.7	967248.4
E9Q394	A-kinase anchor protein 13	Akap13	2776	303.8	5.38	80175.4	85801.6	71962.0	70261.1	81215.9	82379.6	79308.9	74303.5	79837.2	78246.8	186557.8	205951.9	191471.6	190655.4
Q99J79	DNA damage-binding protein 2	Ddb2	432	48.3	9.17	176468.9	205252.0	186605.9	175870.1	198243.8	204672.5	173372.1	166459.5	193561.0	203533.0	80130.6	114256.1	76187.5	96004.1
Q6ZQF0	DNA topoisomerase 2-binding protein 1	Topbp1	1515	168.8	6.67	228145.4	243152.5	246809.8	239912.1	229451.8	209370.7	203580.7	190070.7	242388.2	265854.3	159033.7	175687.8	159903.4	159172.1
Q8VDS4	Regulation of nuclear pre-mRNA domain-containing protein 1A	Rprd1a	312	35.7	7.58	348098.7	368017.1	375260.7	369057.1	387428.7	368924.7	348541.6	327421.1	384637.9	390960.4	385814.8	428775.8	393019.5	413699.4
Q8CGN5	Perilipin-1	Plin1	517	55.6	7.01	176570.5	172639.3	138736.9	184582.0	182711.9	135996.4	146695.4	156558.7	157777.0	153805.2	104005.5	107386.5	92615.6	103247.2
Q9JK81	UPF0160 protein MYG1, mitochondrial	Myg1	380	42.7	7.02	248454.3	257468.2	264248.7	237078.4	257824.7	277931.6	263548.9	249269.9	243172.5	271600.8	271888.2	252980.3	281232.5	251465.4
Q5U458	DnaJ homolog subfamily C member 11	Dnajc11	559	63.2	8.32	571424.8	594195.2	586536.3	588193.4	631649.5	586337.5	572923.9	582808.3	648172.4	612326.1	368124.1	404160.6	444199.5	422431.5
P62301	40S ribosomal protein S13	Rps13	151	17.2	10.54	2934977.7	2342056.9	3074806.8	2568644.7	2793354.8	2880791.6	2585896.7	3088171.6	2740333.9	2601328.5	2807011.9	2460328.9	3199123.9	2753439.1
Q8K057	Intraflagellar transport protein 80 homolog	Ift80	777	87.8	7.94	226161.9	263160.5	258068.2	295149.7	213498.3	196085.0	195045.0	207510.8	239278.0	238569.2	192331.9	256800.2	227107.0	270794.3
Q4FZC9	Nesprin-3	Syne3	975	112	6.33	207543.0	222684.7	213317.6	207138.6	239097.4	239944.4	233926.9	219848.4	214214.5	208443.4	150929.8	161396.9	166399.0	142532.2
P61759	Prefoldin subunit 3	Vbp1	196	22.4	6.28	520321.3	539234.5	612040.2	584945.6	624926.6	596204.4	468095.2	569328.8	609093.7	579756.9	684760.0	773034.8	771657.4	695762.3
Q9DBT5	AMP deaminase 2	Ampd2	798	92	6.23	223641.2	233454.0	224997.9	218563.9	243841.3	269375.9	229227.1	224864.9	219218.7	238420.6	318003.0	286815.4	306106.8	284138.0
Q99JT2	Serine/threonine-protein kinase 26	Stk26	416	46.6	5.24	182606.2	191536.3	167235.3	161337.4	184568.5	200822.1	222857.8	160073.0	172081.0	190649.4	#N/A	#N/A	#N/A	#N/A
Q9CRC9	Glucosamine-6-phosphate isomerase 2	Gnpda2	276	31.1	6.9	218408.3	201011.7	217230.7	197087.1	245655.4	239947.7	248767.4	224692.3	234885.7	215746.9	560792.3	553491.4	584072.1	567464.3
Q61247	Alpha-2-antiplasmin	Serpinf2	491	54.9	6.3	580306.9	546989.1	493942.4	482247.0	413129.6	477859.8	521529.9	529886.8	488705.4	509523.2	553931.8	540923.1	439040.4	458571.6
Q921E2	Ras-related protein Rab-31	Rab31	194	21.3	7.4	298313.4	287539.5	268197.3	267593.9	276481.9	288880.3	283929.5	252774.8	293011.6	299365.7	300772.1	306505.1	273187.1	286925.1
D3YW29	RNA exonuclease 5	Rexo5	759	86.4	8.98	73915.9	90989.0	83498.3	81208.9	81085.7	86137.8	67735.9	66463.1	81258.5	80792.4	170759.3	202215.9	195776.6	191908.1
E9Q912	RAP1, GTP-GDP dissociation stimulator 1	Rap1gds1	607	66	5.35	149141.3	151452.5	137984.2	140715.5	149963.8	143675.6	128548.1	151516.4	139325.2	134873.4	168781.3	176290.9	157258.3	158385.3
S4R1W5	RNA-binding motif protein 6	Rbm6	1118	128.3	6.16	154215.6	138812.3	143235.8	136075.0	151139.4	153660.4	134400.9	131978.5	152179.3	155709.1	237706.3	300855.5	233931.4	263401.6
Q60899	ELAV-like protein 2	Elavl2	360	39.6	9.13	162772.4	181104.4	191764.5	161627.8	205488.1	202055.5	161873.5	155058.1	184144.7	177280.0	207299.3	250714.7	201882.3	252201.6
O88622	Poly(ADP-ribose) glycohydrolase	Parg	969	109.3	6.9	122288.2	139543.2	120500.6	135285.1	119107.4	138493.1	114481.6	111907.2	128159.1	135373.0	233535.4	260771.2	255455.8	268564.7
Q9D906	Ubiquitin-like modifier-activating enzyme ATG7	Atg7	698	77.5	6.4	201003.5	193312.3	193369.2	189784.7	188813.5	201154.7	179089.4	189191.6	187948.5	183071.2	185411.1	220080.3	204370.5	224874.1
Q8C4Y3	Negative elongation factor B	Nelfb	580	65.6	6.02	332515.1	341413.6	297215.6	327879.6	319696.7	346699.5	330065.8	286283.1	342508.6	332935.6	529000.7	565043.3	536749.1	553174.1
Q5FWK3	Rho GTPase-activating protein 1	Arhgap1	439	50.4	6.44	383568.4	392939.0	365624.0	419548.6	462342.4	432550.6	411774.1	442595.5	442161.5	430845.7	1215668.7	1167073.7	1115016.9	1180957.3
Q8BH15	CCR4-NOT transcription complex subunit 10	Cnot10	744	81.8	7.68	156654.7	177410.6	164720.2	166092.1	179267.2	176990.9	166172.8	172211.9	177463.4	177333.4	112852.9	129918.8	120466.4	129256.6
O88851	Putative hydrolase RBBP9	Rbbp9	186	20.9	5.97	423575.5	380170.0	429547.3	396514.8	444981.8	476942.8	443071.6	417888.0	427090.0	425198.9	308988.2	293011.2	305639.6	282150.5
Q8R5H1	Ubiquitin carboxyl-terminal hydrolase 15	Usp15	981	112.3	5.17	78583.7	71740.1	78946.6	72962.5	71371.1	75660.5	61488.4	75821.8	72110.5	70149.3	92482.3	88783.4	82222.9	97912.2
P97447	Four and a half LIM domains protein 1	Fhl1	280	31.9	8.37	175745.8	174430.7	206459.8	168407.7	226178.0	225257.1	232908.7	219520.8	193277.3	190606.4	175761.5	156643.9	175403.8	186199.1
Q8K1C0	Protein angel homolog 2	Angel2	544	62.4	8.21	98555.3	124461.1	100789.9	111396.6	93315.2	94211.4	87937.6	92617.5	97938.5	103455.4	133115.5	144073.6	127415.6	141535.0
Q3UQ84	Threonine--tRNA ligase, mitochondrial	Tars2	723	81.6	7.87	292441.7	295336.7	273583.0	278697.3	271620.4	258513.7	270811.7	271257.5	271795.1	267998.5	272523.1	274573.9	269900.8	278861.9
Q9DBR0	A-kinase anchor protein 8	Akap8	687	76.2	5.14	309842.0	313092.5	352201.9	303434.0	369433.2	352770.6	344685.8	300388.3	350969.4	346914.2	259960.6	255171.9	268194.2	267008.3
O55222	Integrin-linked protein kinase	Ilk	452	51.3	8.07	442734.9	413586.4	371705.7	378628.5	423794.6	460285.3	551764.9	372216.8	438946.7	437329.2	490536.1	519995.1	407857.3	476890.5
O35646	Calpain-6	Capn6	641	74.5	7.17	216853.0	212168.1	217366.2	198347.0	224670.3	215677.4	202606.5	209519.7	206590.7	224857.6	215129.2	201894.2	193365.3	192490.2
Q9CWU9	Nucleoporin Nup37	Nup37	326	36.7	6.09	649052.4	686313.5	806760.7	723337.6	770117.2	782209.5	738797.8	704908.1	793918.4	788232.6	259413.2	266317.9	267705.8	261935.7
Q62245	Son of sevenless homolog 1	Sos1	1319	150.8	6.89	202589.5	213016.6	182861.2	204206.8	172208.0	151155.7	165438.0	168816.7	188896.9	239345.4	179364.4	182713.4	173370.0	167760.1
Q9CRB9	MICOS complex subunit Mic19	Chchd3	227	26.3	8.37	326061.4	331674.9	380879.8	352201.7	387641.6	365009.6	359140.6	349526.2	342577.3	325891.0	110794.9	110260.4	108028.1	97938.0
Q9Z247	Peptidyl-prolyl cis-trans isomerase FKBP9	Fkbp9	570	63	5.21	438854.7	360871.5	410422.2	363584.7	429773.6	365892.6	349145.3	477508.4	341959.4	368105.2	417998.6	412722.3	370398.4	406727.0
Q9ESW4	Acylglycerol kinase, mitochondrial	Agk	421	46.9	8.4	294525.9	261954.4	292616.9	292236.1	300961.8	293420.6	286785.0	309637.2	299989.1	279800.9	174923.5	178341.2	168424.0	185884.4
Q8R2N2	U3 small nucleolar RNA-associated protein 4 homolog	Utp4	686	76.9	8.87	94112.8	115592.3	101465.8	91662.3	108139.4	108715.1	96888.0	109200.5	95286.3	100993.4	178789.7	172868.2	164422.4	162023.7
Q8CI95	Oxysterol-binding protein-related protein 11	Osbpl11	751	83.6	7.01	341056.3	320384.5	342175.7	306291.3	365508.9	353228.8	420203.4	338296.8	341459.2	328525.3	650291.7	628971.5	623876.7	617315.6
Q9Z0P5	Twinfilin-2	Twf2	349	39.4	6.8	379977.4	365303.3	333568.5	341996.7	329525.0	326495.9	363919.9	346144.5	377590.6	362526.2	141633.6	144936.0	138905.5	133271.7
P51432	1-phosphatidylinositol 4,5-bisphosphate phosphodiesterase beta-3	Plcb3	1234	139.4	5.94	301196.8	273172.4	266123.8	294856.0	273119.2	271373.3	282933.7	258878.0	262499.6	259622.8	208772.9	231716.9	199392.4	224056.5
Q9ESB3	Histidine-rich glycoprotein	Hrg	525	59.1	7.66	322486.9	328503.2	294284.3	283006.1	281001.8	333512.9	333553.7	281030.5	318990.3	391719.5	256084.8	243410.4	254429.8	217451.9
Q6PF93	Phosphatidylinositol 3-kinase catalytic subunit type 3	Pik3c3	887	101.4	6.73	284909.0	268834.8	250085.8	266927.3	254783.1	281219.4	244992.8	254989.1	261987.1	251284.6	529376.3	503431.3	504699.1	506309.0
P62264	40S ribosomal protein S14	Rps14	151	16.3	10.05	276162.9	333301.0	265175.4	303461.8	287204.2	346086.1	268278.4	258874.7	269827.6	297897.0	333740.7	400185.8	309130.8	329072.2
Q9D824	Pre-mRNA 3'-end-processing factor FIP1	Fip1l1	581	64.9	5.77	75874.0	97264.8	76497.4	90515.8	87520.0	72407.7	81962.8	73139.5	81161.3	88168.9	92796.9	101768.6	103413.8	95737.1
Q2YDW2	Protein misato homolog 1	Msto1	556	61.2	6.49	152801.5	141339.1	132529.5	134187.7	119832.9	144571.5	137955.6	134995.4	129675.9	135758.7	237070.9	224084.4	225072.2	239570.3
P08122	Collagen alpha-2(IV) chain	Col4a2	1707	167.2	8.48	179114.1	200417.9	188196.4	178553.8	246904.6	230141.1	218969.0	224332.0	196251.1	212908.9	294663.4	292939.4	286048.3	277463.5
P27601	Guanine nucleotide-binding protein subunit alpha-13	Gna13	377	44	8.21	514542.5	481718.5	412347.0	467894.1	476693.1	433005.9	472305.8	454632.2	482409.5	474743.4	427701.6	500242.5	432165.6	498853.0
Q91WN1	DnaJ homolog subfamily C member 9	Dnajc9	259	30	5.94	232138.3	255154.9	281144.3	256061.0	306732.0	303471.5	249470.3	244197.7	272042.2	268910.1	414639.5	439941.7	419152.0	395429.4
Q91YN5	UDP-N-acetylhexosamine pyrophosphorylase	Uap1	522	58.6	6.49	90109.3	102688.5	84218.3	108702.3	90401.4	94039.6	84424.4	92394.8	81582.3	94782.2	174414.5	182885.4	169257.1	173304.6
Q01149	Collagen alpha-2(I) chain	Col1a2	1372	129.5	9.19	256000.9	193753.2	199946.3	189877.1	133626.9	159445.1	139019.8	275609.5	182376.3	169315.5	38940.7	37303.0	40642.1	42988.3
Q8VDH1	F-box only protein 21	Fbxo21	627	72.1	6.06	217669.5	201848.7	194129.7	205530.4	201230.7	202111.4	194497.2	184761.2	209787.3	216036.7	273222.9	235653.9	271442.4	228832.0
O54941	SWI/SNF-related matrix-associated actin-dependent regulator of chromatin subfamily E member 1	Smarce1	411	46.6	4.88	200406.8	196034.0	224176.1	199117.2	220174.9	219657.1	194858.0	191411.5	209219.6	213284.1	170168.6	178392.5	220212.5	177989.5
P26645	Myristoylated alanine-rich C-kinase substrate	Marcks	309	29.6	4.34	362842.5	274875.0	342676.6	256676.9	260974.6	252275.1	266149.8	366955.0	266392.1	260413.3	169143.0	110688.1	153534.3	92865.9
Q6A028	Switch-associated protein 70	Swap70	585	69	6.05	207847.4	203748.3	200409.8	197576.6	214702.9	217414.5	204710.0	202075.0	196140.2	206681.7	234308.0	230931.1	236564.5	208441.9

A2AQ25	Sickle tail protein	Skt	1946	212.9	7.77	267156.0	242950.2	239088.3	234264.5	239633.0	270325.8	261876.9	247618.0	251802.7	244389.8	149442.5	188080.3	149214.4	168709.6
O55126	Protein NipSnap homolog 2	Nipsnap2	281	32.9	9.26	979327.8	803366.2	877620.0	918737.3	1020265.8	964119.4	1109410.5	950102.1	994165.9	956960.9	666850.9	542600.6	713055.6	541395.7
P62965	Cellular retinoic acid-binding protein 1	Crabp1	137	15.6	5.38	663539.6	662549.6	886811.0	546424.7	943715.8	939046.9	619350.6	806355.8	761359.6	759879.3	400568.2	1099110.0	360659.3	613561.4
O70400	PDZ and LIM domain protein 1	Pdlim1	327	35.8	6.84	120188.5	134312.2	99618.8	130139.3	101635.2	119277.6	111891.5	124901.4	102969.9	110434.0	148325.3	150186.1	109563.5	162851.6
Q99PL6	UBX domain-containing protein 6	Ubxn6	442	49.8	8.54	126224.1	139751.6	138140.5	142714.0	141303.3	143451.5	127823.1	122645.4	136020.5	143543.2	157770.7	192382.4	181696.4	182984.3
O88796	Ribonuclease P protein subunit p30	Rpp30	268	29.5	9.06	242790.0	259445.9	255505.5	232716.7	291703.1	293438.1	275321.6	242683.0	274371.4	283276.4	535085.9	518039.1	535560.4	505285.3
Q9ES28	Rho guanine nucleotide exchange factor 7	Arhgef7	862	97	6.8	236875.9	276085.1	251504.4	248200.3	266830.8	258906.0	246177.2	259461.3	246477.8	276702.6	336929.5	357612.7	370726.7	332415.3
Q8R0F6	Integrin-linked kinase-associated serine/threonine phosphatase 2C	Ilkap	392	42.7	7.36	317127.4	367695.5	298991.6	343699.2	325889.1	359649.1	311346.7	314757.0	332461.0	341088.8	378209.2	408884.3	387960.6	399713.6
Q9JI78	Peptide-N(4)-(N-acetyl-beta-glucosaminyl)asparagine amidase	Ngly1	651	74.2	6.89	216054.5	218393.6	242189.5	230701.8	230884.9	207340.5	193127.3	188645.9	217407.2	235705.5	196257.6	167267.6	137618.3	141358.6
D3Z7X0	Acyl-Coenzyme A dehydrogenase family, member 12	Acad12	555	61.4	9.36	273222.4	311095.5	252083.4	280154.7	287058.2	278208.5	292213.0	292036.1	254106.0	282492.0	724313.0	656755.7	644285.0	659444.8
Q61263	Sterol O-acyltransferase 1	Soat1	540	63.8	9.01	605111.1	590690.1	547274.4	619244.2	450830.6	437117.4	424259.9	551895.3	505134.2	494339.9	780346.8	707776.9	566529.5	733777.9
P61290	Proteasome activator complex subunit 3	Psme3	254	29.5	5.95	656697.8	735805.7	649538.7	697930.9	667787.7	642899.7	590933.4	635495.5	666485.0	677896.0	608854.2	673924.8	639932.1	722536.8
Q91WD5	NADH dehydrogenase [ubiquinone] iron-sulfur protein 2, mitochondrial	Ndufs2	463	52.6	6.99	496234.2	537670.2	506405.0	521717.7	501183.3	506757.8	485822.5	511954.3	506696.4	508557.4	520308.2	521020.3	580224.1	517249.7
P41233	Phospholipid-transporting ATPase ABCA1	Abca1	2261	253.7	6.81	155671.2	193796.2	160896.8	204711.1	149639.3	157811.4	153706.1	161609.1	160019.4	166218.0	314355.7	309098.3	222990.5	293100.5
Q69ZR9	Protein TASOR	Tasor	1610	181.3	5.67	103052.8	113457.7	118399.9	104949.0	106087.0	109911.7	118550.5	90481.7	100763.8	108081.1	125165.1	142522.9	138015.1	140203.3
P61211	ADP-ribosylation factor-like protein 1	Arl1	181	20.4	5.72	241865.5	272499.9	262022.4	267236.0	250814.2	260097.0	234848.9	248515.8	265867.1	250663.2	290210.3	332045.5	303478.8	333728.4
Q9ET47	Espin	Espn	871	94.4	7.69	745180.8	866070.9	717917.9	950102.5	528460.1	443054.4	589302.3	672372.4	870701.2	781360.4	1335736.4	1541661.7	1193722.3	1642489.7
P24788	Cyclin-dependent kinase 11B	Cdk11b	784	91.5	5.39	234985.8	259667.2	231728.6	237282.3	238715.6	242025.2	192740.1	201329.5	254597.0	253496.3	300033.0	333579.7	313329.2	336103.4
Q8CFX1	GDH/6PGL endoplasmic bifunctional protein	H6pd	789	88.9	6.93	203386.2	231866.3	146225.5	206242.9	165526.1	173990.9	191190.6	171741.4	174712.0	183407.1	216624.2	266670.9	183664.0	248111.6
A2AQ19	RNA polymerase-associated protein RTF1 homolog	Rtf1	715	80.7	8.16	179839.2	202184.8	165544.9	160269.7	190040.0	201772.3	164201.3	191736.2	163457.0	171487.0	174584.6	178279.7	177878.2	169271.3
P61089	Ubiquitin-conjugating enzyme E2 N	Ube2n	152	17.1	6.57	722494.6	724191.0	725067.9	722423.0	701504.6	609888.9	559330.6	673759.3	668891.1	672253.5	1533468.5	1829667.7	1771432.8	1808306.7
Q61510	E3 ubiquitin/ISG15 ligase TRIM25	Trim25	634	71.7	8.28	194637.0	182919.4	148610.8	167843.9	173358.0	174057.2	180199.9	181317.5	169634.8	180823.1	286170.9	294987.9	308869.5	297763.3
Q9CQH7	Transcription factor BTF3 homolog 4	Btf3l4	158	17.3	6.35	77812.1	100108.1	87709.4	94715.1	84519.2	77975.7	80781.9	79346.8	94883.4	99506.6	487752.4	507233.0	509061.0	529715.5
P08030	Adenine phosphoribosyltransferase	Aprt	180	19.7	6.79	597260.5	685216.8	615071.8	629686.5	672608.7	696781.8	771652.2	605389.3	687808.3	723206.7	199910.2	205864.6	207711.6	199540.7
Q9CXF7	Chromodomain-helicase-DNA-binding protein 1-like	Chd1l	900	101.4	6.64	197380.1	203313.3	199635.3	216193.1	201765.1	210080.4	184580.7	181542.4	202652.7	209249.0	214531.5	238418.4	213907.8	234071.8
Q9ER67	MAGE domain-containing protein	Maged2	616	65.4	9.14	290317.3	278030.7	255257.8	275539.1	205481.9	189662.0	206389.7	258400.6	239540.7	261742.2	522636.6	534027.5	521963.5	486370.6
Q91V12	Cytosolic acyl coenzyme A thioester hydrolase	Acot7	381	42.5	8.68	215370.7	258236.9	267923.0	269165.2	239978.9	220114.1	188481.6	214894.4	238185.0	263361.1	263562.9	285158.2	283469.8	291607.2
Q91WP6	Serine protease inhibitor A3N	Serpina3n	418	46.7	5.82	413006.4	384420.3	361534.6	337371.1	427543.8	447257.0	514198.2	401546.8	617639.8	402091.4	551229.8	585099.5	492937.9	530486.6
Q9D2N9	Vacuolar protein sorting-associated protein 33A	Vps33a	598	67.5	7.08	124351.1	106153.0	121309.0	105057.4	114370.9	120405.4	121151.8	115838.5	114146.8	119404.5	114540.4	122789.8	108384.8	114956.7
Q3V0K9	Plastin-1	Pls1	630	70.4	5.38	138638.1	122476.0	130084.7	115350.9	149219.8	140046.4	134684.0	124861.6	129698.0	135051.9	184993.4	191904.7	175402.8	173752.0
Q0VGB7	Serine/threonine-protein phosphatase 4 regulatory subunit 2	Ppp4r2	417	46.5	4.56	187055.5	208574.5	207175.9	214202.3	227480.8	227863.0	189722.8	192131.6	212577.5	209621.5	147378.1	176396.2	158436.4	167609.5
O54984	ATPase GET3	Get3	348	38.8	4.91	469452.9	426979.8	463759.1	457221.2	461528.4	395549.4	422123.2	441290.3	443977.1	455368.8	669702.4	608529.9	616490.7	652526.8
Q9R0Q7	Prostaglandin E synthase 3	Ptges3	160	18.7	4.55	2134734.8	2072715.5	2401425.5	2502736.0	2098773.0	1932429.1	1705729.1	1913860.7	2347074.0	2360896.3	1794173.7	1790516.4	1996525.6	2042670.7
Q9EPR5	VPS10 domain-containing receptor SorCS2	Sorcs2	1159	128.8	7.34	167362.7	143351.2	170599.3	143037.5	148077.2	159416.9	166424.3	160972.6	149649.1	132898.2	173514.4	175897.2	167337.0	153455.6
Q571I9	Aldehyde dehydrogenase family 16 member A1	Aldh16a1	802	84.7	6.32	138317.0	135682.0	117211.0	123814.7	134650.1	151746.6	123244.7	128701.0	139569.4	134515.2	130494.3	143255.4	135204.7	129173.2
Q61792	LIM and SH3 domain protein 1	Lasp1	263	30	7.05	505759.7	611856.4	434561.2	481475.0	437818.9	437882.6	470816.5	437908.5	463730.3	481985.8	784451.7	763863.5	790453.6	760608.0
Q80XK6	Autophagy-related protein 2 homolog B	Atg2b	2075	231.3	5.88	344792.3	359075.1	340810.5	348589.6	328690.9	345198.2	319027.4	299099.8	343212.8	345203.3	117742.8	153828.8	132099.0	147144.9
Q62000	Mimecan	Ogn	298	34	5.74	563556.9	531868.7	533281.6	575962.1	654864.7	803458.5	1036264.6	664940.0	603278.1	598409.4	393987.4	371795.7	398729.5	396547.0
Q8K0C9	GDP-mannose 4,6 dehydratase	Gmds	372	42	7.03	224813.7	246355.4	232873.5	225092.4	261134.7	250594.3	242836.2	217593.7	240541.4	267236.6	236904.7	239089.6	280760.5	221706.9
P34022	Ran-specific GTPase-activating protein	Ranbp1	203	23.6	5.22	636851.5	599511.1	696564.9	687745.6	482668.1	514265.3	472101.1	597557.4	596178.0	550858.1	807061.6	783987.2	875111.1	950485.7
Q99LR1	Lysophosphatidylserine lipase ABHD12	Abhd12	398	45.2	8.72	265108.7	327339.3	249176.2	270899.6	280639.9	274438.5	235030.3	274203.2	260949.0	302029.5	386050.2	395678.4	342588.4	325404.7
Q6ZWN5	40S ribosomal protein S9	Rps9	194	22.6	10.65	4634215.8	4470283.9	4992573.8	4494784.6	4366432.7	4133467.5	3309771.4	4952311.8	4382339.4	4299359.8	6181358.7	5996146.0	6735204.0	6108671.0
Q64521	Glycerol-3-phosphate dehydrogenase, mitochondrial	Gpd2	727	80.9	6.61	186203.6	187631.7	158667.3	175889.4	174121.2	178206.4	175892.6	175894.7	179520.0	189027.1	164643.4	167111.8	155481.8	164194.2
Q91XQ0	Dynein heavy chain 8, axonemal	Dnah8	4731	540.9	6.18	117838.0	165160.6	133260.3	180364.1	98153.3	104656.9	83193.9	93479.1	117641.1	137049.2	85205.5	100707.1	91948.8	116516.1
Q8C8U0	Liprin-beta-1	Ppfibp1	969	108.5	5.49	120600.1	128259.6	107404.9	97020.7	134822.9	128032.4	118668.3	118278.2	117917.4	113490.6	250571.4	308361.1	212874.3	273025.5
Q9JK23	Proteasome assembly chaperone 1	Psmg1	289	33.1	6.37	168998.3	190223.3	189833.3	187159.8	176578.2	170377.4	167364.9	171557.4	184116.1	172144.8	296158.1	353156.6	395326.6	351346.1
F8VQ70	U1-type domain-containing protein	Scaper	1398	157.7	7.31	119210.0	126350.6	137735.4	141162.5	123290.0	146909.5	149318.6	112871.8	125391.4	122880.1	149480.7	191426.1	168132.1	155510.1
A2A8Z1	Oxysterol-binding protein-related protein 9	Osbpl9	736	83.1	6.07	714191.1	702559.3	685441.7	651492.6	667457.6	640495.6	724445.6	627182.8	685328.3	707692.7	432363.9	506291.3	455162.2	458196.9
Q8BH57	WD repeat-containing protein 48	Wdr48	676	76	7.17	346463.4	328291.8	352860.2	364863.0	325282.0	324706.5	326683.6	352826.0	316577.7	328324.8	291724.6	333262.8	317483.4	337437.5
Q61702	Inter-alpha-trypsin inhibitor heavy chain H1	Itih1	907	101	6.96	186617.5	196610.2	158348.6	171223.5	145871.0	175917.4	166166.2	198082.1	170134.9	188055.6	170875.3	166890.2	145014.0	145067.5
Q80Y44	Probable ATP-dependent RNA helicase DDX10	Ddx10	875	100.7	7.75	107216.7	113217.6	109281.6	107265.8	107190.9	116601.6	98961.1	108066.3	109628.0	110272.8	403248.3	359179.8	355709.7	330896.9
Q8BGR9	Ubiquitin-like domain-containing CTD phosphatase 1	Ublcp1	318	36.8	6.46	755894.9	848923.6	756508.5	823395.8	787460.0	812359.4	659536.0	699056.5	803854.7	879657.8	616447.0	634433.0	758585.2	638281.5
Q99LH1	Nucleolar GTP-binding protein 2	Gnl2	728	83.3	9.2	305060.6	288375.7	286629.1	291424.5	263625.0	237019.5	253535.6	227894.4	263107.4	296370.6	91441.6	102735.9	103874.9	101160.7
O35295	Transcriptional activator protein Pur-beta	Purb	324	33.9	5.43	322913.9	263520.0	284034.9	251095.0	326206.7	322684.7	333398.5	331480.9	271670.0	280768.1	374354.9	371286.9	338433.3	359018.2
Q60967	Bifunctional 3'-phosphoadenosine 5'-phosphosulfate synthase 1	Papss1	624	70.7	6.77	141093.3	137222.1	143938.1	148945.8	147641.6	155278.1	144604.1	138677.1	141803.2	147414.9	35103.5	38687.5	35218.0	36228.4
Q9DCS3	Enoyl-[acyl-carrier-protein] reductase, mitochondrial	Mecr	373	40.3	9.07	114593.5	121117.1	123125.0	125613.9	122615.8	119344.0	112018.3	127789.2	121876.8	113169.6	232146.5	249053.4	229352.0	241230.3
P62996	Transformer-2 protein homolog beta	Tra2b	288	33.6	11.25	649240.5	670271.2	675975.7	657503.5	681471.9	647171.1	682772.8	609899.5	666231.6	666667.7	1690169.8	1659417.6	1905032.9	1878242.6
Q3V3R4	Integrin alpha-1	Itga1	1179	130.7	6.2	286182.4	258862.0	284520.3	264781.9	296709.1	290301.0	365890.5	295236.8	275922.5	259116.9	324301.1	364993.3	341428.4	331645.6
J3QN89	WD_REPEATS_REGION domain-containing protein	Aamp	436	46.9	4.41	213744.6	226630.7	236633.4	243697.1	225199.2	216851.4	205865.0	199722.4	228029.0	239598.7	66682.1	85066.1	74006.7	64877.6
P10605	Cathepsin B	Ctsb	339	37.3	5.91	292764.8	287809.9	291844.5	300896.6	379752.0	346417.1	440963.8	337925.2	312899.1	330238.9	535409.1	467499.7	508834.3	466513.4
Q8R3I3	Conserved oligomeric Golgi complex subunit 6	Cog6	657	73	6.14	273809.3	327548.6	268678.1	322357.4	257243.2	250414.8	227510.8	239222.5	269048.5	291509.1	204147.3	240028.9	211161.3	241870.9
Q8R123	FAD synthase	Flad1	492	54.7	6.64	144684.6	164054.4	124585.8	169596.3	146686.7	137330.3	150945.5	141454.9	164059.0	163828.3	172289.6	188552.1	169319.9	166532.3
Q9EQ61	Pescadillo homolog	Pes1	584	67.8	6.84	270307.4	271716.7	260364.3	255603.6	254663.5	257410.6	259236.9	280885.0	277366.7	294095.3	288743.9	327142.1	299237.8	296218.4
Q64512	Tyrosine-protein phosphatase non-receptor type 13	Ptpn13	2453	270.2	6.37	215757.3	223014.9	192919.1	226294.0	209548.4	225220.1	251836.4	218888.6	209611.1	230544.1	130749.8	127736.7	119686.2	120490.5
Q8BMD8	Calcium-binding mitochondrial carrier protein SCaMC-1	Slc25a24	475	52.9	7.43	574930.3	526321.7	509566.3	512412.4	567359.4	607508.6	653080.4	600705.0	586789.4	479123.9	309322.6	292796.2	283879.0	264646.1
O70433	Four and a half LIM domains protein 2	Fhl2	279	32.1	7.3	366524.7	348549.0	386177.1	335123.8	416037.5	415399.4	376413.2	389157.3	379243.8	382394.5	266193.5	256955.7	275764.3	254020.2
Q8C6E0	Cilia- and flagella-associated protein 36	Cfap36	343	39.6	4.93	51602.7	68877.9	81002.4	120755.8	48188.5	47588.8	40368.9	52832.1	55192.9	61375.9	280353.4	403212.5	376459.0	502407.9
Q921W4	Quinone oxidoreductase-like protein 1	Cryzl1	348	38.7	5.97	246688.2	253523.6	239089.8	262300.2	224509.4	216055.8	204168.2	235905.8	234261.0	256194.1	270809.4	307416.9	284498.4	306409.3
F6TQW2	Immunoglobulin heavy constant gamma 2C	Ighg2c	404	44.2	6.9	213003.2	205526.6	212017.2	196795.4	209033.6	204641.3	220306.4	223639.7	198807.2	214761.1	296566.9	246655.4	354825.5	347624.1
O88520	Leucine-rich repeat protein SHOC-2	Shoc2	582	64.9	8.46	97813.0	105965.1	103118.7	101729.9	114922.5	111094.3	94004.2	104064.5	100855.4	100399.9	230455.0	226687.1	218977.3	225271.8
Q11136	Xaa-Pro dipeptidase	Pepd	493	55	5.78	402977.1	411176.4	400614.5	435161.1	429320.2	401708.7	406841.3	404283.0	430208.2	407350.8	298399.7	284703.5	262347.4	289188.5
Q3UIA2	Rho GTPase-activating protein 17	Arhgap17	846	92.1	7.56	137266.8	152531.7	127730.4	136685.3	143011.0	139651.3	129944.3	131977.3	143985.8	151506.8	88748.0	69971.6	76318.4	67280.0
Q61001	Laminin subunit alpha-5	Lama5	3718	403.8	6.73	80969.4	70630.9	71236.5	67395.8	76280.7	93870.2	90083.3	86816.1	84109.5	82756.6	69165.5	70757.0	78287.9	70396.3
Q9D2M8	Ubiquitin-conjugating enzyme E2 variant 2	Ube2v2	145	16.4	8.09	180931.0	174157.1	195783.3	179477.1	206935.0	221499.4	208174.1	184573.3	182697.1	180603.8	317833.1	311035.8	301817.2	312153.0
Q8CG46	Structural maintenance of chromosomes protein 5	Smc5	1101	128.7	8.56	115666.2	139972.6	136705.3	155182.2	125066.1	134358.2	115691.7	107128.1	118662.1	131456.4	107715.4	116563.7	119128.3	123782.9
Q9CPR4	60S ribosomal protein L17	Rpl17	184	21.4	10.18	820382.0	831045.0	894775.4	832155.3	870412.5	788147.3	663815.3	867699.1	818027.1	787756.0	978454.2	1168366.7	965610.6	1068497.5
Q9Z2L6	Multiple inositol polyphosphate phosphatase 1	Minpp1	481	54.5	7.49	339245.3	382353.2	315818.6	383218.5	306389.6	318347.5	268161.0	312734.2	346655.5	364952.1	291239.8	320602.5	295697.9	324506.6
Q8C9B9	Death-inducer obliterator 1	Dido1	2256	247	7.91	263234.6	304041.6	299266.7	298542.8	291604.0	304378.9	273484.9	260094.7	298720.0	297181.7	154704.0	150008.2	163683.8	160396.8
O70378	ER membrane protein complex subunit 8	Emc8	207	23.3	6.15	230213.3	235908.8	243675.8	227762.4	230435.7	245669.9	248560.1	231909.1	238604.0	246121.6	392482.3	402862.0	392645.7	403621.0
Q8BFP9	[Pyruvate dehydrogenase (acetyl-transferring)] kinase isozyme 1, mitochondrial	Pdk1	434	49	8.19	149249.5	147816.2	137121.7	142192.7	149003.3	148646.3	155458.1	144294.2	145637.4	156230.0	190741.6	203902.5	172059.7	187994.8
Q9Z2W1	Serine/threonine-protein kinase 25	Stk25	426	48.1	6.81	254109.4	261080.0	224449.9	225400.5	259863.0	244259.5	228164.3	226297.1	271657.9	264921.1	199039.8	208333.0	208539.2	207271.1
Q80SW1	S-adenosylhomocysteine hydrolase-like protein 1	Ahcyl1	530	58.9	6.89	211367.2	240607.9	213973.6	231907.8	225831.7	213133.3	224000.7	194743.2	216308.4	235001.8	438080.0	500331.6	423412.5	423875.8
Q5SFM8	RNA-binding protein 27	Rbm27	1060	118.5	9.19	413780.2	391145.1	352167.7	399651.0	355250.8	357394.9	320857.5	336790.4	371975.4	421945.0	197676.7	245402.7	212697.7	235922.0
Q9CY64	Biliverdin reductase A	Blvra	295	33.5	7.02	679547.9	687466.4	630766.4	651006.3	719690.0	649672.1	690013.2	670914.6	732684.1	745284.2	709463.7	708436.2	682773.8	681117.1
Q8VDG5	Phosphopantothenate--cysteine ligase	Ppcs	311	33.8	6.55	581125.9	636085.8	552076.2	622454.7	532048.2	536586.6	608044.5	596117.3	618605.7	586453.2	647800.6	571628.6	571230.9	607055.3
Q8CE96	tRNA (adenine(58)-N(1))-methyltransferase non-catalytic subunit TRM6	Trmt6	497	55.5	6.95	334315.2	318234.4	315688.3	308766.5	360717.2	350599.5	323771.2	315529.2	333581.2	357911.2	310005.1	340013.6	331211.9	320835.2
P00329	Alcohol dehydrogenase 1	Adh1	375	39.7	8.1	453323.0	412855.6	342692.5	354524.7	418296.2	421256.3	493753.6	455098.4	370080.3	411628.7	903766.5	802293.5	744293.0	779433.7
Q9CWZ7	Gamma-soluble NSF attachment protein	Napg	312	34.7	5.41	692591.1	816817.5	790027.1	838557.5	678029.3	676689.4	618919.2	673374.9	759425.7	803326.9	268681.8	302873.2	284647.3	299311.0
Q6P6M7	O-phosphoseryl-tRNA(Sec) selenium transferase	Sepsecs	504	55.3	8.06	115362.9	105208.1	134988.5	115843.1	135558.0	131881.1	118299.2	114364.7	138502.5	126055.5	159390.6	183617.3	189785.2	198075.9
Q80YV4	4'-phosphopantetheine phosphatase	Pank4	820	91.5	6.71	216305.4	234162.7	218874.7	243059.5	232299.9	216732.5	213194.7	213349.3	233901.8	239258.1	205575.6	246237.8	222224.0	223775.1
Q9CQI3	Glia maturation factor beta	Gmfb	142	16.7	5.16	1061065.9	1283866.2	1470752.9	1467614.6	1302198.4	1056117.5	1014099.5	1155209.8	1354630.4	1364349.0	1305709.8	1436108.2	1721596.1	1615415.3
Q9CQC9	GTP-binding protein SAR1b	Sar1b	198	22.4	6.11	197226.1	314850.9	187458.7	281886.7	282505.0	230334.2	201326.0	227907.1	237911.7	269291.2	238831.7	276925.5	248322.4	259676.5

Q80Y84	Lysine-specific demethylase 5B	Kdm5b	1544	175.4	6.43	41813.6	48183.2	45902.4	46202.3	45382.2	49159.4	44157.1	42697.6	47023.0	49728.0	40211.8	57779.1	49668.1	49410.9
Q9CQW1	Synaptobrevin homolog YKT6	Ykt6	198	22.3	6.35	661375.5	689087.9	606435.4	668996.0	639814.1	627842.2	581928.9	597421.6	653413.4	708708.9	1157011.1	1129826.4	1254336.4	1073536.1
Q8K3C3	Protein LZIC	Lzic	190	21.5	4.96	226936.3	291607.9	274144.9	292004.6	271957.9	268537.2	248172.9	266360.4	274884.1	296786.7	252044.7	256097.2	318503.4	291117.8
Q6P9K8	Caskin-1	Caskin1	1431	150.4	9.17	116408.4	125737.0	115581.5	119934.6	118233.3	125517.7	128248.0	119407.3	135828.1	126183.1	137884.2	140476.0	125666.8	130338.0
Q6Y7W8	GRB10-interacting GYF protein 2	Gigyf2	1291	149.1	5.57	143867.6	137708.4	153146.9	136015.4	143195.0	128572.0	142033.8	137153.4	146884.2	147857.9	121236.3	129279.2	149916.1	126444.2
Q64433	10 kDa heat shock protein, mitochondrial	Hspe1	102	11	8.35	9109874.3	8655632.5	9190362.6	8609650.2	9623801.9	8498666.2	8778523.4	8974820.1	9029185.3	9197464.1	8462111.3	7854896.1	9054219.4	7715078.5
O09130	NFATC2-interacting protein	Nfatc2ip	412	45.1	6.67	469496.3	443553.1	361359.3	447320.2	355120.3	350096.4	341677.6	422453.2	418234.2	403020.5	387098.9	373690.0	320058.7	390583.4
Q60676	Serine/threonine-protein phosphatase 5	Ppp5c	499	56.8	6.2	232719.1	283759.6	244882.8	308080.8	246496.1	230000.7	215075.0	218218.0	241735.8	260756.8	471985.8	571716.6	593683.9	650318.0
Q99M51	Cytoplasmic protein NCK1	Nck1	377	42.9	6.47	144730.9	159662.6	186687.2	178209.8	153686.9	167170.9	136498.1	148269.5	152391.4	156780.2	143947.2	169912.9	183958.3	174407.1
P14094	Sodium/potassium-transporting ATPase subunit beta-1	Atp1b1	304	35.2	8.65	868570.9	913767.5	1000781.9	840368.8	994931.2	1058718.1	1089652.2	1006835.5	909215.2	970078.6	851168.7	903790.7	875413.0	803662.6
O35345	Importin subunit alpha-7	Kpna6	536	59.9	4.94	238149.9	241442.5	234204.4	261064.6	234262.4	209938.0	225946.6	214995.2	229491.6	242315.9	166532.9	176369.9	159559.8	174482.2
P62267	40S ribosomal protein S23	Rps23	143	15.8	10.49	463687.3	862071.9	486295.0	591930.7	603067.6	512237.2	486143.9	539507.4	524678.2	644459.0	598494.9	646399.4	561923.0	589391.3
Q64324	Syntaxin-binding protein 2	Stxbp2	593	66.3	6.74	124292.2	168951.6	140357.6	154633.7	140581.1	156102.7	123681.4	143174.9	146602.4	137293.7	38184.7	48419.7	43490.4	48645.0
E9Q2E4	HECT domain-containing protein	Hectd4	4418	483.8	6.06	160648.5	162882.8	167947.6	158709.7	168533.9	153321.1	156482.7	168295.5	158241.5	161709.7	77967.7	94255.9	73744.0	76141.7
P69566	Ran-binding protein 9	Ranbp9	653	71	6.84	288005.5	298272.8	293493.8	279248.5	267791.3	274454.2	256666.3	268188.3	287818.6	255555.6	77788.5	81768.9	77877.5	88184.8
Q99K41	EMILIN-1	Emilin1	1017	107.5	5.3	157253.2	127978.1	139399.7	131755.7	155989.2	179964.4	199542.3	156142.9	153632.2	147744.2	212530.3	221937.3	169122.8	203225.9
Q9D0F3	Protein ERGIC-53	Lman1	517	57.8	6.34	302517.7	273163.4	298866.7	306874.9	290876.5	284303.2	289105.8	285396.3	294199.2	284062.3	515248.7	427637.4	502477.1	422434.1
Q6PCM2	Integrator complex subunit 6	Ints6	883	99.6	8.78	232577.5	234295.6	232363.5	229803.6	262191.6	229757.5	224322.9	216203.9	235515.1	242523.8	156436.5	182075.0	207108.7	163846.4
Q91VE0	Long-chain fatty acid transport protein 4	Slc27a4	643	72.3	8.59	193085.9	175494.4	170577.7	165717.7	168430.5	162032.5	170778.3	165900.4	165631.4	173770.0	178938.8	181224.6	174567.2	171211.9
P53811	Phosphatidylinositol transfer protein beta isoform	Pitpnb	271	31.5	6.95	76944.0	81047.7	67808.2	74980.6	75085.3	78515.0	86849.1	74568.3	72196.0	72044.8	339728.5	348463.8	302999.3	354785.3
Q61187	Tumor susceptibility gene 101 protein	Tsg101	391	44.1	6.71	178297.9	182525.4	195439.6	180092.9	183197.5	213614.9	202886.3	182774.4	180899.5	162489.4	189924.8	219705.7	219235.4	225792.2
Q9ERG0	LIM domain and actin-binding protein 1	Lima1	753	84	6.6	111708.1	117226.3	113485.5	115180.0	106344.2	109911.2	117536.4	125969.5	109413.5	110251.0	216375.9	240088.8	209058.2	231336.4
P29391	Ferritin light chain 1	Ftl1	183	20.8	6	288990.7	351991.9	324330.0	294021.8	345469.0	307596.0	299176.5	286656.7	305406.9	329834.6	419130.9	423787.9	452379.5	446261.5
P57716	Nicastrin	Ncstn	708	78.4	6.09	285834.1	273973.6	281872.2	281073.9	279357.8	252487.1	264352.4	291018.1	273323.6	251079.6	267905.1	272699.4	253289.7	265728.9
P67871	Casein kinase II subunit beta	Csnk2b	215	24.9	5.55	330953.8	367491.3	419781.9	400559.2	398443.7	353235.6	378570.3	358889.6	401514.6	378900.2	668751.6	674601.5	725397.4	692166.2
Q62159	Rho-related GTP-binding protein RhoC	Rhoc	193	22	6.58	36127.7	35739.7	34031.7	33259.8	39948.6	38135.1	47414.6	43127.4	40823.1	40346.9	62901.4	57027.5	54452.7	57693.0
B7ZMP1	Xaa-Pro aminopeptidase 3	Xpnpep3	506	56.6	7.68	152591.4	174129.1	153349.0	155287.0	163927.2	147717.8	138772.8	138599.2	159226.6	170196.6	122480.0	130266.6	127197.5	137407.5
Q921S7	39S ribosomal protein L37, mitochondrial	Mrpl37	423	48.3	8.84	108896.4	104004.3	96756.7	103090.9	102948.6	98693.2	94610.7	91784.5	104834.0	109963.3	216842.8	223019.3	226237.8	234825.5
P97825	Jupiter microtubule associated homolog 1	Jpt1	154	16.1	5.31	329879.7	374830.0	340371.2	380270.9	329754.7	326797.2	309689.5	325476.5	359126.9	381489.7	62782.0	89039.2	82377.5	86504.0
Q6IR42	Zinc finger CW-type PWWP domain protein 1	Zcwpw1	630	70.5	5.45	329534.2	358665.3	333317.0	345816.3	376042.0	429070.4	315447.0	304883.9	346768.5	362451.7	146273.2	170912.7	172536.2	176236.2
Q9DBN5	Lon protease homolog 2, peroxisomal	Lonp2	852	94.5	7.23	267903.1	270404.1	242950.1	233627.0	280960.5	255127.6	222145.1	255851.4	259151.0	279314.3	144869.5	150141.5	146025.4	139266.2
Q8BKX6	Serine/threonine-protein kinase SMG1	Smg1	3658	409.5	6.4	171732.5	163032.5	157969.7	168033.6	167921.8	169144.8	157317.2	152579.7	163389.2	160422.5	91388.3	93858.6	82197.0	92466.3
Q9DB73	NADH-cytochrome b5 reductase 1	Cyb5r1	305	34.1	8.87	371089.1	330834.6	303386.4	335065.8	319692.9	319011.1	331881.2	322288.9	314551.3	343458.9	325311.3	361466.9	311937.7	335734.6
Q0VBL3	RNA-binding protein 15	Rbm15	962	105.7	10.08	478295.6	577770.6	593553.6	420292.7	376598.6	367467.9	354829.6	323821.9	393209.0	397580.4	193948.1	206687.3	201125.7	204625.3
Q9JLB0	MAGUK p55 subfamily member 6	Mpp6	553	62.6	6.4	251766.0	210645.6	244570.6	223334.1	265515.4	259820.5	243588.5	227312.4	237874.6	237628.5	390512.7	448389.0	405349.6	423427.0
Q505D1	Serine/threonine-protein phosphatase 6 regulatory ankyrin repeat subunit A	Ankrd28	1053	112.8	6.27	164829.4	153524.9	139497.3	154921.6	141165.1	140482.0	137164.1	138424.3	139254.0	153647.4	331279.7	336571.0	318195.5	334330.5
Q8BXQ2	GPI transamidase component PIG-T	Pigt	582	65.7	8.4	538805.7	582991.1	541895.1	568685.1	498760.8	502675.1	461398.1	458784.5	513168.0	547587.0	577709.6	575026.1	581720.8	552871.9
Q9JKC8	AP-3 complex subunit mu-1	Ap3m1	418	46.9	6.93	348415.5	373299.0	344818.0	359138.4	349644.6	345305.3	319585.1	338726.9	351398.7	342837.0	258606.2	273670.2	249947.7	247801.1
Q9D1E6	Tubulin-folding cofactor B	Tbcb	244	27.4	5.24	675206.0	783300.1	830651.9	791329.6	641535.7	697760.7	643147.8	717339.7	699760.0	770162.7	493425.3	460479.1	564718.0	494793.4
E9PWW9	PHD-type domain-containing protein	Rsf1	1441	161.7	5.02	72538.2	92258.3	71021.9	70786.3	84639.9	80121.4	70867.9	67513.3	73819.7	80101.7	84279.9	89102.7	100174.3	88637.5
Q62523	Zyxin	Zyx	564	60.5	6.4	508328.5	464922.6	398010.2	444208.7	432981.2	451414.8	468086.6	453676.9	425794.8	426809.5	589404.9	568160.1	545844.3	530332.1
P62915	Transcription initiation factor IIB	Gtf2b	316	34.8	8.35	395090.3	423064.0	412821.2	419059.4	434110.0	430335.6	389109.1	414693.1	386156.7	441810.3	216347.1	260508.4	210162.1	250444.1
Q9JKB3	Y-box-binding protein 3	Ybx3	361	38.8	9.69	40267.2	47579.1	48632.8	47845.0	39584.7	38187.1	41774.7	37458.2	37833.7	42870.4	366830.5	423051.2	449380.6	588351.3
Q61474	RNA-binding protein Musashi homolog 1	Msi1	362	39.1	7.87	271073.7	292442.4	278552.3	264043.5	267987.0	260291.8	284617.6	239919.9	259029.0	285976.8	219252.6	195300.0	202294.0	194885.6
Q9D1P4	Cysteine and histidine-rich domain-containing protein 1	Chordc1	331	37.3	7.9	475287.5	548836.9	463866.5	570874.8	414794.5	433843.9	423034.1	444261.4	465544.2	446302.7	610184.4	647032.5	589439.2	675659.3
Q3TMP1	General transcription factor IIIC, polypeptide 3	Gtf3c3	882	100.6	5.15	96730.6	92613.9	89514.3	86924.2	103130.8	106790.9	96030.1	93937.1	96735.8	108000.3	109150.7	91863.6	79078.7	85146.6
Q99MP8	BRCA1-associated protein	Brap	591	66.9	5.64	72002.9	91050.8	85012.8	85228.9	96815.2	96980.7	79317.3	85225.8	85860.3	84600.7	50107.6	51242.3	51754.9	54143.8
P63280	SUMO-conjugating enzyme UBC9	Ube2i	158	18	8.66	600655.1	680167.9	784183.6	702105.5	764216.6	666138.6	661132.4	693317.4	753944.3	739037.1	612838.8	642858.8	697091.5	663944.0
Q8BU33	2-hydroxyacyl-CoA lyase 2	Ilvbl	632	68.1	8.69	314497.3	310651.7	269979.8	311972.0	280657.3	299585.4	270083.9	296923.2	290752.4	301579.0	466677.0	440582.0	424252.9	430115.8
Q61207	Prosaposin	Psap	557	61.4	5.19	213989.5	214336.2	207168.9	214516.6	213491.2	188032.0	198925.4	203459.0	201853.6	197357.7	98199.2	101598.5	91634.3	97138.9
Q8CIH9	Amidophosphoribosyltransferase	Ppat	517	57.4	6.96	496531.5	483958.9	477203.6	453228.6	487291.2	484977.0	496533.0	452239.7	496966.9	515831.3	147599.9	167170.7	148082.3	158843.6
Q61216	Double-strand break repair protein MRE11	Mre11	706	80.2	6.01	271683.0	280195.4	286484.7	264874.2	311335.3	312210.5	279922.4	278948.5	285355.3	285603.6	165503.1	180933.3	187594.0	180777.9
Q80W00	Serine/threonine-protein phosphatase 1 regulatory subunit 10	Ppp1r10	888	94.3	9.16	149732.3	132548.5	110704.9	125209.6	114348.9	117683.8	104620.1	100973.2	172093.6	119809.3	133169.8	145711.0	139477.8	141710.6
P0C7L0	WAS/WASL-interacting protein family member 3	Wipf3	485	49.4	10.18	130029.4	123156.1	116368.5	114283.2	124896.1	125948.4	129656.8	119951.0	121484.8	126741.3	319081.4	294682.1	273389.2	266826.8
Q08091	Calponin-1	Cnn1	297	33.3	8.97	150066.0	151505.9	180498.7	173268.8	178591.4	231767.1	305390.0	547695.5	174729.3	160779.9	206842.6	208484.8	217156.6	225853.1
Q9WVJ3	Carboxypeptidase Q	Cpq	470	51.8	6.21	214677.4	279893.1	245882.9	210796.3	237323.7	320286.7	341607.1	199976.0	262257.4	253285.0	117476.5	126256.7	130680.4	123820.1
P04117	Fatty acid-binding protein, adipocyte	Fabp4	132	14.6	8.4	366357.3	254770.4	171579.0	179643.4	1475056.5	180714.5	624416.6	283476.7	181880.3	198036.1	458549.4	433033.6	293234.0	336057.9
Q9QXB9	Developmentally-regulated GTP-binding protein 2	Drg2	364	40.7	8.88	325525.6	334419.4	355576.8	356209.7	369366.3	369414.1	322664.4	355189.0	336104.0	347936.5	236353.7	237982.6	244843.7	221638.3
A0A1L1SU27	Pleckstrin homology domain-containing, family G (with RhoGef domain) member 4	Plekhg4	1217	134.8	7.25	186238.1	200442.8	182286.1	188781.9	171869.2	155900.7	145297.9	159807.0	165584.4	173877.4	100363.1	109165.3	107965.2	101683.4
Q8VDG3	Poly(A)-specific ribonuclease PARN	Parn	624	71.5	5.97	263295.7	258739.0	231191.5	249056.8	254940.1	256899.8	258605.8	233509.3	247572.8	256414.5	337278.5	296904.4	332183.5	374036.9
Q14AT2	Testis-expressed protein 11	Tex11	947	109.6	8.34	192926.5	240509.4	211975.9	207409.3	244700.5	243686.7	201994.5	192553.6	227533.4	248765.8	176821.7	170351.2	179349.1	154382.2
P08556	GTPase NRas	Nras	189	21.2	5.17	145244.3	156995.6	156538.0	155023.6	158541.1	155186.8	148229.6	147634.1	153058.8	154067.3	105959.6	112510.2	128979.1	123885.0
Q99J45	Nuclear receptor-binding protein	Nrbp1	535	59.8	5.08	122857.8	137836.0	126545.7	135707.3	134556.1	150227.1	126667.7	121930.9	137354.2	141359.4	130288.9	187982.5	129603.3	150669.8
Q99KF1	Transmembrane emp24 domain-containing protein 9	Tmed9	235	27.1	8.41	1393506.8	1637255.4	1446205.4	1570041.7	1326447.7	1286351.3	1524296.1	1299353.2	1536592.7	1626614.6	1592393.3	1545674.2	1478770.8	1490374.5
O55026	Ectonucleoside triphosphate diphosphohydrolase 2	Entpd2	495	54.3	8.37	323866.6	300964.9	302218.8	301839.4	370094.7	397192.3	384766.1	312994.8	341521.6	341070.7	119876.7	132369.2	109695.4	116157.5
P17183	Gamma-enolase	Eno2	434	47.3	5.11	8931.2	8973.1	4964.2	8551.7	9459.4	7864.5	9031.9	5815.7	6269.9	10839.5	7069.2	4940.2	7611.9	4390.4
E9Q5C9	Nucleolar and coiled-body phosphoprotein 1	Nolc1	702	73.7	9.36	525678.7	609132.2	521419.7	603395.6	607823.0	552200.2	598292.1	493795.5	588255.9	642317.9	304972.4	308864.5	335415.2	304862.2
Q9D3R3	Centrosomal protein of 72 kDa	Cep72	646	72.4	6.74	238609.6	360270.1	321926.0	432578.9	269540.1	227555.0	192772.8	236213.0	316312.1	342703.5	269882.2	319835.1	295179.4	331789.7
Q9WVM1	Rac GTPase-activating protein 1	Racgap1	628	70.1	8.51	258624.1	299406.7	260634.8	259597.6	284496.3	280211.8	233643.1	226047.9	269407.8	278233.7	213451.4	233905.6	217591.2	211758.9
Q99J09	Methylosome protein 50	Wdr77	342	36.9	5.27	234899.5	296264.5	268147.1	315060.8	219231.1	224576.8	202909.1	206156.9	259788.5	272204.3	219834.1	254824.7	225011.1	270771.4
P50171	Estradiol 17-beta-dehydrogenase 8	Hsd17b8	259	26.6	6.54	159267.4	164099.9	155107.8	170138.2	155621.3	150053.4	166971.7	141817.4	159380.9	159324.1	120587.7	123221.4	132361.3	132235.5
Q3TCN2	Putative phospholipase B-like 2	Plbd2	594	66.2	6.13	252504.4	259971.5	256436.8	265587.8	277289.3	237712.3	255187.0	247077.5	264079.2	267987.2	419513.9	460977.1	442922.1	437810.1
P61021	Ras-related protein Rab-5B	Rab5b	215	23.7	8.13	142791.3	197052.0	132296.7	164160.5	158336.1	154793.1	150044.1	133627.0	173044.8	175229.7	202197.6	185709.3	200103.1	198537.5
Q8K368	Fanconi anemia group I protein homolog	Fanci	1330	149.2	6.16	125239.7	137116.9	121756.7	123215.9	121211.2	126594.3	112461.1	113241.5	132043.9	120808.2	157787.3	180524.0	145128.4	167468.4
Q8BPU7	Engulfment and cell motility protein 1	Elmo1	727	83.9	6.28	159210.9	169253.2	156497.8	158153.7	169396.5	165532.4	159390.8	171598.9	168741.1	158113.6	267750.4	268294.2	256817.8	252627.7
Q9JK48	Endophilin-B1	Sh3glb1	365	40.8	6.04	349546.4	387814.0	383519.2	419650.8	352119.4	338426.8	323644.9	336654.5	382052.9	369037.0	448567.5	478103.7	513926.6	562999.7
A2AT37	Regulator of nonsense transcripts 2	Upf2	1269	147.5	5.64	500472.9	551325.5	541484.9	555481.7	529085.9	520670.7	468735.1	492617.4	516504.7	537077.3	324190.9	352958.8	343321.6	331195.7
P49817	Caveolin-1	Cav1	178	20.5	6.02	482811.6	509147.8	439061.5	489588.2	569711.9	570596.5	657551.9	523594.2	511202.5	493679.4	345624.9	300975.1	394229.3	319312.4
Q80X98	DEAH (Asp-Glu-Ala-His) box polypeptide 38	Dhx38	1228	140.5	6.49	172949.6	164563.5	172303.1	166365.7	178632.5	163525.1	151357.3	165309.8	169333.4	170669.2	171523.8	173587.7	170316.8	179653.6
P24815	3 beta-hydroxysteroid dehydrogenase/Delta 5-->4-isomerase type 1	Hsd3b1	373	42	8.63	547946.6	538603.4	491101.7	586273.5	467496.0	451779.1	507615.6	513346.2	550251.3	535484.3	144209.0	155022.7	125743.0	169244.2
Q6ZQL4	WD repeat-containing protein 43	Wdr43	677	75.3	5.38	195858.5	227774.5	198069.2	211052.4	235913.1	231200.3	208277.5	196809.6	216226.9	225291.8	238156.1	247732.4	229537.1	237533.6
P61027	Ras-related protein Rab-10	Rab10	200	22.5	8.38	397398.8	368383.6	385239.2	353750.3	413953.9	452186.7	406663.6	405342.3	384615.6	380705.9	446997.2	401400.1	445954.9	382767.6
Q3UHX0	Nucleolar protein 8	Nol8	1147	128.6	6.55	153183.5	159251.6	138276.2	131300.3	135556.1	136551.3	118750.0	122728.0	156204.3	142515.0	88509.2	118402.5	93690.4	96003.8
Q91WU5	Arsenite methyltransferase	As3mt	376	41.8	5.86	202743.7	238570.5	210566.9	221516.7	221536.0	245598.3	213591.4	200174.9	218046.7	207142.8	465131.0	493805.9	391716.3	451731.1
P28740	Kinesin-like protein KIF2A	Kif2a	705	79.7	6.73	618351.9	642399.3	656517.4	629247.6	591442.3	657353.0	560697.8	527285.7	693223.5	669651.9	612074.8	711335.1	718312.8	693838.7
Q9CQE1	Protein NipSnap homolog 3B	Nipsnap3b	247	28.3	9.48	282628.5	278189.0	260601.3	280387.6	281539.4	276190.9	292851.2	277943.4	290319.0	233845.4	195473.7	266495.1	174578.4	219851.7
Q3THG9	Alanyl-tRNA editing protein Aarsd1	Aarsd1	412	44.9	6.42	346128.1	344394.6	375608.3	340645.4	352532.7	307071.5	286021.6	359328.7	321973.5	338097.7	417719.6	470523.1	453886.5	442438.8
O08808	Protein diaphanous homolog 1	Diaph1	1255	139.3	5.57	248888.8	270481.8	237722.9	254451.0	265473.2	269822.3	256628.7	267877.0	266374.1	251969.6	172025.0	203560.4	167822.2	174074.9
Q91YD9	Neural Wiskott-Aldrich syndrome protein	Wasl	501	54.2	7.93	316226.0	316128.6	317008.0	301282.2	314700.2	303675.7	318081.1	321107.0	309865.5	296178.6	1090996.5	983962.2	902111.9	973447.1
P51863	V-type proton ATPase subunit d 1	Atp6v0d1	351	40.3	5	258043.9	259657.7	290328.3	257652.9	311417.4	288345.3	268609.0	282419.5	285224.5	290801.1	440881.3	440721.0	422453.9	473663.9
Q8CHT0	Delta-1-pyrroline-5-carboxylate dehydrogenase, mitochondrial	Aldh4a1	562	61.8	8.24	274504.5	263350.2	252058.5	271651.5	264566.8	262235.7	313469.7	260731.1	260476.6	242208.4	198810.2	183586.1	203647.9	196105.5
Q9D7B6	Isobutyryl-CoA dehydrogenase, mitochondrial	Acad8	413	45	8.13	106271.0	104364.8	109881.5	107069.5	109146.1	108065.7	103809.4	100155.3	103936.1	97265.9	777542.7	670951.3	754041.6	719281.8

Q80TR8	DDB1- and CUL4-associated factor 1	Dcaf1	1506	168.8	5.08	208500.6	206196.5	201121.2	204085.8	204362.4	208918.4	203016.5	182915.8	221950.7	210374.3	258794.7	292240.8	296797.0	266036.7
Q9EQ06	Estradiol 17-beta-dehydrogenase 11	Hsd17b11	298	32.9	8.66	591287.5	527738.1	575226.6	509274.7	538447.8	534415.8	624582.1	577108.5	554642.2	564025.7	428135.4	392152.9	406145.7	391734.4
Q9QUR8	Semaphorin-7A	Sema7a	664	74.9	7.74	349862.0	353057.3	344627.1	320919.4	377313.4	392784.7	355968.2	353841.9	332720.8	377462.3	229096.3	240976.9	231360.5	230862.8
Q99JW2	Aminoacylase-1	Acy1	408	45.8	6.32	393113.7	479627.2	352720.5	402267.6	436792.5	471546.8	398281.6	446840.7	408540.1	425007.6	327331.5	336203.3	317996.1	314964.5
P58742	Aladin	Aaas	546	59.4	6.87	534762.0	580618.9	502730.6	530564.4	571918.7	562824.1	513607.5	528537.4	564377.2	552495.8	306080.1	336686.9	305186.5	335414.4
Q9QY15	ATP-dependent RNA helicase DDX25	Ddx25	484	54.8	5.99	84236.4	121985.1	117830.0	152364.2	90805.3	95728.0	79851.3	83614.1	104656.6	116898.7	216633.6	295268.9	280895.8	368539.6
P00405	Cytochrome c oxidase subunit 2	Mtco2	227	26	4.73	1140087.4	1258149.3	1269783.6	1358182.5	1222560.7	1209221.3	1169433.1	1150758.3	1228880.8	1189562.7	511338.6	548590.6	859434.7	759788.4
Q8BH43	Wiskott-Aldrich syndrome protein family member 2	Wasf2	497	54	5.53	108739.5	117287.9	110168.5	111909.1	113938.6	113757.2	108425.3	114625.2	103723.9	115506.9	119082.1	126223.1	118659.4	120092.0
Q8BSQ9	Protein polybromo-1	Pbrm1	1634	187.1	6.9	200047.9	189278.8	216019.9	185704.4	214540.4	226756.7	226678.1	222266.6	183935.7	216857.9	371464.5	257653.8	378139.1	316825.3
Q8CI71	Syndetin	Vps50	964	111.1	6.07	333943.6	341186.9	382761.2	357726.6	372488.6	354085.4	325596.2	341148.0	366897.8	354015.6	158119.6	161757.7	156503.1	160490.5
B9EKI3	TATA element modulatory factor	Tmf1	1091	121.7	4.87	58439.4	71565.7	72758.1	68262.4	63235.1	60594.3	57785.3	71382.3	60417.6	73643.3	122499.3	133615.0	126177.0	137764.4
B2RXC1	Trafficking protein particle complex subunit 11	Trappc11	1133	128.3	7.58	250447.1	265365.1	206598.1	241001.4	228685.6	235360.2	211943.0	205095.3	221597.7	245741.9	251301.0	261768.3	259376.3	266849.3
Q8QZY9	Splicing factor 3B subunit 4	Sf3b4	424	44.3	8.56	214227.4	219144.2	215077.8	216225.9	213859.1	207123.4	201063.8	204242.5	211217.5	214279.0	160547.3	159723.9	162206.6	165872.0
Q9D880	Mitochondrial import inner membrane translocase subunit TIM50	Timm50	353	39.8	8.13	233538.0	255813.6	271796.8	265582.9	282845.3	268864.9	257291.7	257053.6	264528.5	247509.1	244841.2	276075.1	265806.5	279023.8
Q9CZ04	COP9 signalosome complex subunit 7a	Cops7a	275	30.2	7.87	287689.9	299123.3	305024.0	331344.8	288664.5	267644.8	275163.4	275046.6	299461.6	302828.2	351048.1	360674.3	429427.6	407027.4
Q8VCT4	Carboxylesterase 1D	Ces1d	565	61.7	6.61	99111.0	98588.3	81740.4	174230.6	147077.0	114508.7	168831.5	177448.4	84754.7	95027.9	68609.1	72195.1	63569.7	116036.1
A3KMP2	Tetratricopeptide repeat protein 38	Ttc38	465	52.2	6.3	187340.3	189429.0	201669.7	187929.3	217545.3	215202.1	253517.0	218304.8	205946.1	208609.8	168414.1	188580.5	163822.2	174004.5
Q8BT07	Centrosomal protein of 55 kDa	Cep55	462	53.9	7.94	328653.8	368360.2	335947.0	352287.5	314309.0	340982.6	327721.1	314247.2	342870.2	371530.3	388736.9	458210.0	430614.8	443266.0
Q5SRX1	TOM1-like protein 2	Tom1l2	507	55.6	4.82	97320.3	93573.2	85171.3	91832.0	91705.4	105165.7	82684.2	84633.8	84397.7	97300.8	202471.1	207461.6	176651.9	189965.9
A2ATU0	Probable 2-oxoglutarate dehydrogenase E1 component DHKTD1, mitochondrial	Dhtkd1	921	102.7	6.9	120492.1	127209.5	135947.3	131496.0	147283.9	143823.9	154201.0	133604.5	140186.3	139820.6	182792.6	175497.0	197518.9	172796.5
P11352	Glutathione peroxidase 1	Gpx1	201	22.3	7.21	389702.2	392883.2	340145.7	392293.0	405848.1	431467.5	496767.0	410221.0	396106.6	386068.8	518802.6	487945.8	422963.8	480376.0
Q6PGB6	N-alpha-acetyltransferase 50	Naa50	169	19.4	8.81	283437.0	312287.3	303105.0	269609.8	317538.7	285987.5	284090.0	277947.6	301548.9	288271.8	640538.8	631104.5	707638.4	643856.1
Q920Q6	RNA-binding protein Musashi homolog 2	Msi2	346	36.9	8.47	142155.3	118134.1	124172.5	129856.6	122873.9	127466.5	143047.5	133330.7	119752.9	129870.7	124533.7	126327.0	114872.2	111561.4
P56376	Acylphosphatase-1	Acyp1	99	11.2	9.04	913992.8	1206760.8	1219985.6	1742120.0	546555.7	560959.2	587257.6	770716.9	946115.5	1013064.4	795382.5	1151402.7	1165764.8	1594155.6
Q8BMQ2	General transcription factor 3C polypeptide 4	Gtf3c4	817	91.6	6.73	96912.4	106766.5	66214.0	64414.7	103320.3	75689.3	71082.3	66996.6	70686.3	71451.9	81947.8	77414.5	74443.4	77698.5
Q9D5T0	ATPase family AAA domain-containing protein 1	Atad1	361	40.7	6.9	175365.7	177438.8	150898.8	175588.0	152486.4	151380.3	144588.3	161051.0	165845.3	162483.3	121218.1	118206.6	107804.6	112979.2
Q922Y1	UBX domain-containing protein 1	Ubxn1	297	33.6	5.26	369936.3	400512.5	355256.7	409355.2	327723.4	308779.1	316912.7	333810.7	361644.5	394699.6	347386.3	366085.4	356800.5	368511.8
Q8BGZ7	Keratin, type II cytoskeletal 75	Krt75	551	59.7	8.31	252176.5	236985.1	245402.8	337155.7	450734.4	453440.9	359922.9	302174.3	322112.4	337836.0	304475.0	290865.7	285010.2	345721.6
Q8BGW1	Alpha-ketoglutarate-dependent dioxygenase FTO	Fto	502	58	5.12	200570.7	229520.2	188175.6	210399.3	242719.8	237620.0	199987.8	218377.1	213063.1	225000.6	110681.7	126108.9	110243.4	124338.7
P08226	Apolipoprotein E	Apoe	311	35.8	5.68	566380.3	479629.2	451976.6	419383.8	394403.2	488977.4	450401.2	498828.5	447719.4	523940.3	521428.4	516481.7	430416.9	454945.3
Q3V089	RNA-binding protein 44	Rbm44	1013	112.2	5.86	108629.2	150367.9	169734.4	166645.1	111988.6	102038.4	104008.8	103170.0	119522.6	127891.9	139261.6	147242.6	161464.2	163415.3
Q8K2C9	Very-long-chain (3R)-3-hydroxyacyl-CoA dehydratase 3	Hacd3	362	43.1	9.13	546853.8	564138.2	542205.6	617777.4	516537.3	501944.6	498465.8	557888.2	519325.7	499684.8	740132.3	734257.4	730638.5	783644.2
Q80VC9	Calmodulin-regulated spectrin-associated protein 3	Camsap3	1252	135.1	8.43	225763.1	182113.1	186186.5	190621.0	188603.9	200305.0	181423.3	208254.4	192964.3	179037.3	192307.8	189500.8	214138.2	174765.5
P11087	Collagen alpha-1(I) chain	Col1a1	1453	137.9	5.85	335234.4	353433.9	330587.7	348723.8	342353.4	356649.8	343018.3	315772.5	347290.4	358594.6	111321.7	112809.9	112512.8	114371.0
E9PV38	Carboxylic ester hydrolase	Ces2g	560	62.7	6.04	416512.4	431449.8	401733.9	373688.0	420602.1	458522.6	577254.2	385702.8	421036.0	424938.5	325877.4	301817.1	250299.3	282248.2
Q3TYA6	M-phase phosphoprotein 8	Mphosph8	858	97.4	5.85	294598.6	300235.4	297536.1	309485.1	325424.6	346604.3	302246.4	297803.8	300742.6	306421.8	435322.3	488614.2	486232.4	483796.8
Q9D287	Pre-mRNA-splicing factor SPF27	Bcas2	225	26.1	5.66	223068.5	244519.0	270028.2	272982.1	302902.2	263649.2	252730.1	255402.3	276869.7	268841.1	529110.5	548343.9	656665.9	613556.7
Q80UU9	Membrane-associated progesterone receptor component 2	Pgrmc2	217	23.3	5.15	196908.2	185248.4	204437.7	206330.0	188236.6	189841.2	177947.4	196492.1	204584.5	194597.5	281061.3	278454.8	240914.6	294964.8
Q9JJI8	60S ribosomal protein L38	Rpl38	70	8.2	10.1	1319796.5	1317714.5	1323880.3	1299703.0	1320304.8	1327145.6	1215346.0	1308039.5	1347547.7	1290982.9	1790988.7	2031512.8	1779475.0	1862316.0
P97823	Acyl-protein thioesterase 1	Lypla1	230	24.7	6.62	153091.0	181961.8	157333.0	180569.1	158879.6	154026.5	149555.2	150523.7	168643.6	172225.1	70069.8	74004.6	77766.5	72443.3
Q9CT10	Ran-binding protein 3	Ranbp3	491	52.5	5.12	170177.2	185451.6	210990.5	182621.7	200798.1	220098.4	188317.1	208049.6	192335.0	193575.3	141591.7	150433.4	131137.5	149914.3
Q3UUQ7	GPI inositol-deacylase	Pgap1	922	104.5	8.91	174098.8	195131.3	183062.2	198000.4	193757.4	211703.1	195750.3	211296.2	190104.5	177038.2	119487.1	122365.9	128564.6	122744.8
P47809	Dual specificity mitogen-activated protein kinase kinase 4	Map2k4	397	44.1	8.07	180069.6	188366.1	173037.6	175531.5	192853.3	193062.6	175045.6	181265.1	182139.6	165913.7	320841.9	315940.3	276213.7	300483.7
Q9QZ59	Doublesex- and mab-3-related transcription factor 1	Dmrt1	374	39.4	6.64	229227.8	230518.2	228164.1	202403.0	269245.7	257558.3	219541.4	239492.0	227542.3	224248.7	121236.7	105077.7	124863.9	108055.7
P59997	Lysine-specific demethylase 2A	Kdm2a	1161	132.6	7.44	162042.9	148076.2	183994.5	146102.8	186143.5	190453.5	165335.0	172946.4	173516.5	156804.4	88924.0	84207.1	66012.3	70128.6
Q9D5T7	HORMA domain-containing protein 1	Hormad1	392	44.9	8.41	261683.3	363418.4	349982.5	322405.8	406874.1	339926.0	213776.3	250628.9	359142.2	390088.8	348456.3	380901.6	442020.7	402492.5
Q9CQT1	Methylthioribose-1-phosphate isomerase	Mri1	369	39.4	5.91	150329.8	149619.8	142583.0	138946.1	165619.2	160634.5	158956.3	150969.8	146651.4	151574.7	207109.2	197911.0	205078.8	200378.5
P54729	NEDD8 ultimate buster 1	Nub1	614	70.3	5.88	199149.8	176640.9	203927.3	198285.2	216263.1	236372.8	188711.7	214670.2	185116.0	205435.0	215361.3	237918.8	200768.9	232756.0
Q8BZW8	NHL repeat-containing protein 2	Nhlrc2	725	78.4	5.54	272400.7	271384.1	301920.6	287545.7	324568.3	316843.5	295014.0	285702.2	310341.9	315327.0	169020.0	165246.7	151320.5	171982.5
Q8CCB4	Vacuolar protein sorting-associated protein 53 homolog	Vps53	832	94.4	6.61	438945.8	420456.3	461221.5	409880.2	461859.2	478991.4	411350.4	420357.2	433793.0	445315.7	128350.0	152916.0	139248.4	137856.2
Q9D0D5	General transcription factor IIE subunit 1	Gtf2e1	440	49.6	4.86	401795.3	412738.2	439564.9	416493.2	419236.0	372563.7	338894.8	372148.0	416555.9	415765.6	202126.0	227506.0	248014.4	239931.4
O88879	Apoptotic protease-activating factor 1	Apaf1	1249	140.9	6.43	128203.7	146421.9	123975.6	125053.5	134923.0	136245.5	128246.2	121803.7	119535.1	134401.8	139052.8	128144.3	115489.5	122599.8
Q9CQ80	Vacuolar protein-sorting-associated protein 25	Vps25	176	20.7	6.34	480518.3	546812.0	489854.9	540784.4	526778.0	546631.6	535554.2	471154.7	545590.6	532034.1	497894.0	533120.7	566403.5	585309.7
Q9ERS2	NADH dehydrogenase [ubiquinone] 1 alpha subcomplex subunit 13	Ndufa13	144	16.8	9.48	305273.7	320487.9	262363.5	305901.6	297047.3	292511.6	267026.9	268632.9	329715.8	304391.0	159307.3	169458.5	146497.7	154707.5
Q69Z89	Ras-associating and dilute domain-containing protein	Radil	1099	120.3	6.57	229154.8	274784.2	277178.7	281791.7	233002.8	196987.0	137579.0	198038.6	237415.9	252253.5	137224.3	193444.5	145722.1	151429.1
P09671	Superoxide dismutase [Mn], mitochondrial	Sod2	222	24.6	8.62	1556789.6	1306835.2	1484490.0	1473756.6	1324163.8	1358137.3	1477623.7	1449446.8	1398375.1	1434605.7	1518249.6	1391809.6	1484155.3	1439170.4
Q9CYN2	Signal peptidase complex subunit 2	Spcs2	226	25	8.57	644799.7	734826.9	648371.5	735846.3	635789.6	603890.0	747286.9	625396.6	690633.4	690542.0	2208330.7	2128622.5	2197765.0	2252919.0
Q60780	Growth arrest-specific protein 7	Gas7	421	48.1	7.83	205633.9	187718.2	200831.8	208121.2	207621.7	219448.7	204155.4	204305.7	205651.2	215399.7	235077.0	269663.0	234245.2	260752.3
A2APB8	Targeting protein for Xklp2	Tpx2	745	85.8	9.38	145132.4	136170.8	187549.5	169466.5	138766.4	119629.3	118260.0	138664.3	140108.8	145263.6	176887.1	187738.9	165994.0	188704.4
Q63850	Nuclear pore glycoprotein p62	Nup62	526	53.2	5.31	191895.2	229494.2	234399.7	231498.9	279650.4	261179.3	267265.6	222270.5	236412.9	231033.1	151775.2	177186.1	180697.9	174266.9
O55106	Striatin	Strn	780	85.9	5.27	150890.6	154593.5	164940.2	142372.6	184001.6	177509.2	169384.3	173394.2	155281.5	166182.1	310087.3	322144.8	305693.2	313914.2
Q8VCI0	Phospholipase B-like 1	Plbd1	550	63	8.87	176896.7	181823.4	151819.4	117359.7	224321.7	195487.1	244079.3	193768.8	173517.9	172896.1	79049.4	79117.9	60424.9	52682.2
Q8K337	Type II inositol 1,4,5-trisphosphate 5-phosphatase	Inpp5b	993	112.7	5.64	159745.0	150557.2	154190.4	148463.6	152805.2	133707.7	154149.5	144735.6	159351.4	172201.5	231373.9	189722.3	204070.5	210403.2
Q8R4X3	RNA-binding protein 12	Rbm12	992	102.7	8.32	157997.4	176007.7	163944.8	161761.8	206518.7	170319.4	175987.2	162194.5	173903.3	179097.1	213207.6	187312.8	199493.3	187405.0
P63325	40S ribosomal protein S10	Rps10	165	18.9	10.15	380520.1	415715.1	354873.9	383196.8	362652.7	378428.2	319118.8	353367.6	359373.1	395950.8	649985.3	864894.4	646127.3	668044.4
Q61576	Peptidyl-prolyl cis-trans isomerase FKBP10	Fkbp10	581	64.7	5.64	420992.8	345712.2	364114.7	319785.9	357481.9	329153.9	355086.6	445099.3	334457.9	346504.5	452141.3	471731.3	407150.2	409279.4
Q920I9	WD repeat-containing protein 7	Wdr7	1489	163.3	6.9	135016.4	131174.4	111452.5	128215.6	122387.9	119139.5	111143.5	110110.8	109695.1	128910.9	196544.5	213778.3	171401.7	188358.1
Q99K67	Alpha-aminoadipic semialdehyde synthase, mitochondrial	Aass	926	102.9	6.87	68561.2	68595.8	62738.1	59990.6	67006.5	62283.6	71015.7	62311.3	58192.3	70129.1	239995.1	243857.1	234221.3	256244.2
Q78JE5	F-box only protein 22	Fbxo22	402	44.2	7.61	374247.1	382739.8	352480.0	347897.1	400188.9	410820.5	386405.2	356922.0	374839.3	417334.7	250312.7	255859.8	238155.6	251695.4
Q62018	RNA polymerase-associated protein CTR9 homolog	Ctr9	1173	133.3	6.49	309590.7	333075.7	293907.7	332371.4	320031.9	294354.4	358166.6	307896.7	320388.8	392092.5	312168.0	340683.6	380495.1	345201.8
Q60749	KH domain-containing, RNA-binding, signal transduction-associated protein 1	Khdrbs1	443	48.3	8.72	575518.1	601797.2	613568.0	631804.2	623158.2	631630.1	526415.6	584230.1	622067.8	612620.0	390478.5	298677.4	387587.3	366191.9
P97470	Serine/threonine-protein phosphatase 4 catalytic subunit	Ppp4c	307	35.1	5.06	164579.8	167979.3	159708.7	178074.0	166172.7	162696.6	161098.6	153170.1	163846.3	175068.6	125550.3	152753.5	122850.5	125396.6
Q07076	Annexin A7	Anxa7	463	49.9	6.18	206489.5	187441.6	190798.9	181318.7	181219.8	194459.0	236275.9	223441.9	196473.7	178061.4	374409.6	387420.0	379490.6	382380.7
D3YYU8	Obscurin-like protein 1	Obsl1	1804	197.8	5.41	166457.5	160107.3	147940.8	144496.4	141435.8	157802.6	147266.8	149141.8	139743.1	150037.4	182410.2	176858.3	152541.7	166081.7
P36536	GTP-binding protein SAR1a	Sar1a	198	22.4	6.93	64073.8	53548.8	59896.7	55954.4	48270.6	51526.9	48795.9	60762.5	54835.9	51918.8	49997.0	51528.9	49792.9	47809.8
Q9CYA0	Protein disulfide isomerase Creld2	Creld2	350	38.2	4.58	151403.2	169683.5	174393.8	180947.8	148151.4	163985.6	135805.5	156449.4	169109.3	159159.8	264120.2	346555.7	329518.5	342712.6
Q99ME9	Nucleolar GTP-binding protein 1	Gtpbp4	634	74.1	9.52	229540.5	234947.5	234805.1	235356.0	230610.1	253533.7	203896.0	212416.2	228912.5	238286.0	287094.6	291191.9	294421.9	300232.9
Q9CSU0	Regulation of nuclear pre-mRNA domain-containing protein 1B	Rprd1b	326	36.9	5.97	91849.8	99140.2	94970.3	178755.2	103639.1	90822.5	94857.1	91147.6	127994.4	99911.9	249385.9	230356.3	247582.9	242172.7
Q80W14	Pre-mRNA-processing factor 40 homolog B	Prpf40b	870	99.2	6.96	107751.2	116004.1	111730.4	109636.3	115369.9	111064.8	101513.3	97443.9	120129.8	126481.4	79450.0	85079.8	87956.1	85933.0
O35218	Cleavage and polyadenylation specificity factor subunit 2	Cpsf2	782	88.3	5.11	328547.2	374693.4	350744.0	377409.5	359190.6	363996.4	332539.7	320968.9	367523.4	407092.4	345542.0	376362.1	397257.0	366672.4
Q3V1H1	Cytoskeleton-associated protein 2	Ckap2	664	74	9.23	109446.7	134405.4	141839.4	127339.7	107539.2	112609.6	98414.8	86895.0	132426.1	137500.1	182459.4	299914.2	248308.4	274891.6
Q8C4Q6	Axin interactor, dorsalization-associated protein	Aida	305	34.9	6.74	203235.2	215100.3	179083.9	195450.4	177113.5	203327.3	220048.3	190126.3	196975.8	204793.9	178905.7	208838.5	158759.0	178688.8
E9Q1Z0	IF rod domain-containing protein	Krt90	538	58.2	8.25	487870.2	538144.8	452041.7	542396.3	584247.5	556801.5	469958.4	507034.1	523867.8	555217.0	100317.9	118812.1	102489.1	121193.4
P58044	Isopentenyl-diphosphate Delta-isomerase 1	Idi1	227	26.3	6.16	409135.8	468881.5	503286.5	562444.4	486041.2	444444.9	376025.8	378984.8	477393.5	488174.7	336387.8	449522.9	405900.2	440214.3
Q9CYI4	Putative RNA-binding protein Luc7-like 1	Luc7l	371	43.9	9.88	270148.5	269056.1	279144.9	264806.6	279803.8	283844.4	267700.2	250693.6	287929.6	275950.5	148021.7	152918.6	163353.2	147415.3
Q8BX10	Serine/threonine-protein phosphatase PGAM5, mitochondrial	Pgam5	288	32	9.04	508401.8	546537.8	570920.7	551856.4	566784.9	548427.3	516792.8	483682.9	555675.8	595878.8	336437.6	346790.9	375376.7	340451.5
Q9CY27	Very-long-chain enoyl-CoA reductase	Tecr	308	36.1	9.55	258358.1	295974.8	247171.7	303703.2	314562.1	277656.8	248477.8	291196.0	259586.6	302089.2	480593.7	509791.4	544228.2	558995.6
P63024	Vesicle-associated membrane protein 3	Vamp3	103	11.5	8.5	923361.0	1028849.7	1020672.8	999337.2	999015.4	952662.3	901835.6	939643.5	1030504.8	1048147.3	715052.9	768996.2	756016.5	756781.4
Q99PP7	E3 ubiquitin-protein ligase TRIM33	Trim33	1142	123.8	6.74	201948.9	221628.4	208478.9	204569.6	214363.7	214409.7	213457.9	199334.0	204555.4	211102.2	203246.5	195576.2	198878.4	196978.1
O35435	Dihydroorotate dehydrogenase (quinone), mitochondrial	Dhodh	395	42.7	9.55	108438.5	122948.5	110555.2	110368.3	120480.0	123351.3	118522.8	111734.8	116660.5	113599.6	264462.0	227436.3	224106.8	240852.5
P59470	DNA-directed RNA polymerase III subunit RPC2	Polr3b	1133	127.6	8.5	94504.5	102745.8	91108.2	90432.4	100458.7	106538.2	85981.2	90628.0	80606.7	102646.2	203798.6	223039.5	220168.0	197180.1
Q8CHG3	GRIP and coiled-coil domain-containing protein 2	Gcc2	1679	194.3	5.12	148212.1	136203.9	122021.5	129807.3	133513.2	124652.9	121791.3	124513.8	135456.5	131676.3	134950.8	148975.3	139512.6	148077.6

P47811	Mitogen-activated protein kinase 14	Mapk14	360	41.3	5.88	312640.5	291478.2	263915.6	262670.8	267415.6	285788.1	254315.2	271789.6	269367.7	274670.1	191893.7	186733.0	187392.8	179575.3
Q8BG67	Protein EFR3 homolog A	Efr3a	819	92.6	6.83	138155.8	123725.3	131538.7	125754.0	143811.9	153292.3	152835.7	137123.5	149074.2	136748.5	286816.1	278141.6	273304.2	285048.4
Q8CGF1	Rho GTPase-activating protein 29	Arhgap29	1266	142.3	7.17	242967.1	294353.2	267230.1	269730.8	262422.9	277315.5	263073.2	221152.0	292442.1	279453.7	118752.7	119887.9	109340.2	115838.6
O35551	Rab GTPase-binding effector protein 1	Rabep1	862	99.5	5.01	181782.7	193295.8	169967.4	174999.5	171514.6	178294.5	158742.9	161244.4	176106.4	160125.9	218326.4	239979.3	212879.2	236629.0
Q9ERE7	LRP chaperone MESD	Mesd	224	25.2	6.34	880124.5	806925.6	738770.3	817330.0	765707.9	728468.0	687977.1	782735.4	739367.9	728971.9	255867.4	281646.0	253167.5	275587.9
Q8VIG3	Radial spoke head 1 homolog	Rsph1	301	34.2	4.53	436332.6	468870.8	494568.5	466077.9	399063.2	282811.7	275860.5	432044.1	474076.5	497618.1	110275.2	134082.5	145635.8	151960.8
Q6A009	E3 ubiquitin-protein ligase listerin	Ltn1	1767	198.8	6.55	292233.6	304321.9	283021.0	280490.2	312992.9	284446.4	280176.8	269928.8	288005.0	302634.5	327964.6	323378.5	300423.0	306909.4
P62717	60S ribosomal protein L18a	Rpl18a	176	20.7	10.71	4840446.3	4692453.7	5085405.3	4746279.7	4264863.9	4467116.2	3645971.1	4715847.0	4664198.3	4753320.5	1293390.6	1497915.7	1509481.3	1485829.5
P97927	Laminin subunit alpha-4	Lama4	1816	201.7	6.21	248439.9	246449.1	240198.1	239416.3	323763.4	316038.8	312605.6	295775.4	251605.4	239258.8	50386.0	67427.9	53119.8	52057.6
Q8VBV7	COP9 signalosome complex subunit 8	Cops8	209	23.2	5.2	224198.2	263117.8	240167.8	281762.5	252637.9	230991.0	220658.4	219660.4	251971.5	267315.9	402255.4	429101.3	441645.2	457827.4
Q99J99	3-mercaptopyruvate sulfurtransferase	Mpst	297	33.1	6.47	393047.3	380450.5	425336.7	377790.6	464921.7	435130.2	462776.9	405791.2	428797.8	421977.5	382207.0	359434.1	398892.7	367168.6
Q8C5H8	NAD kinase 2, mitochondrial	Nadk2	452	50.8	8.07	133023.2	137548.2	141435.1	125450.8	139585.5	129605.1	135674.8	118389.6	136260.4	136766.2	198923.1	206634.8	210378.9	195025.5
Q78JW9	Ubiquitin domain-containing protein UBFD1	Ubfd1	368	40.1	8.85	308368.2	360170.7	308324.7	319645.3	334424.4	339818.8	307418.0	305045.2	342520.9	353265.9	325100.7	346421.4	357738.1	336579.9
Q99LP6	GrpE protein homolog 1, mitochondrial	Grpel1	217	24.3	8.38	295874.9	306489.4	280699.9	301999.4	298919.6	273779.3	254965.0	296353.9	294665.6	273688.1	396328.6	391161.1	366765.0	399082.5
P01942	Hemoglobin subunit alpha	Hba	142	15.1	8.22	4436741.2	2784843.3	2915853.6	4675912.9	3423494.7	3023212.3	4846923.4	5334181.7	4133974.5	3419210.8	2085275.1	1318907.3	1276620.4	2460217.5
Q80W47	WD repeat domain phosphoinositide-interacting protein 2	Wipi2	445	48.4	5.86	226463.6	224259.3	228373.6	223189.0	233951.3	243464.3	201588.3	221519.1	234127.8	220144.3	135104.7	157513.6	139899.7	135581.2
Q3U487	E3 ubiquitin-protein ligase HECTD3	Hectd3	861	97.3	5.47	95791.8	133958.0	101513.6	104525.2	136330.7	128574.0	97859.2	120561.2	120370.0	127031.3	66474.4	69503.3	63403.2	66025.8
P10833	Ras-related protein R-Ras	Rras	218	23.7	6.79	225624.7	252035.6	220993.3	217023.2	261895.3	284936.4	265766.2	235089.5	227347.2	252783.6	188514.2	201985.4	208698.5	207683.0
Q6VH22	Intraflagellar transport protein 172 homolog	Ift172	1749	197.4	6.02	220585.5	272521.5	241177.0	285263.2	207800.6	195247.7	184008.2	203432.3	231656.4	265421.3	249968.0	269023.4	286508.4	339231.8
Q9R0E2	Procollagen-lysine,2-oxoglutarate 5-dioxygenase 1	Plod1	728	83.5	6.54	245063.9	251668.1	221828.7	227975.2	242723.2	233717.5	211799.7	244470.0	235403.5	246562.3	293274.6	306604.5	286325.4	292395.8
Q8BKS9	Pumilio homolog 3	Pum3	647	72.8	9.66	158882.3	175300.1	154331.6	174237.9	172271.0	157264.8	138017.3	147652.6	167306.1	167797.3	146745.6	148564.8	143098.5	142103.4
Q8BHS3	Pre-mRNA-splicing factor RBM22	Rbm22	420	46.9	8.54	114194.7	126299.6	114712.8	116827.9	115860.8	117401.4	121858.6	108249.6	116941.4	126308.0	107858.1	111901.9	111705.7	104206.0
Q91W86	Vacuolar protein sorting-associated protein 11 homolog	Vps11	941	107.7	7.01	98392.0	103617.0	107820.8	103463.2	109682.3	113895.8	100428.8	109227.7	95268.7	102350.1	172167.5	194252.0	162658.1	177383.8
Q9D1J3	SAP domain-containing ribonucleoprotein	Sarnp	210	23.5	6.65	801404.2	787951.9	829355.5	838082.0	839778.8	772682.3	864083.0	778148.7	827390.8	910736.7	750545.9	849085.9	901500.3	772497.5
Q61036	Serine/threonine-protein kinase PAK 3	Pak3	559	62.4	5.4	219523.9	208512.8	214447.3	198502.7	224218.7	215463.5	200713.7	200487.6	212734.5	229121.9	551600.9	465738.8	517224.2	473735.3
Q921G8	Gamma-tubulin complex component 2	Tubgcp2	905	103.2	6.77	120522.5	130098.9	121646.2	126420.6	118918.6	116755.8	109079.1	103596.3	119226.5	123202.3	381871.9	397828.2	389572.8	385482.4
Q91W39	Nuclear receptor coactivator 5	Ncoa5	579	65.3	9.82	231344.3	241659.4	232418.9	255110.9	217648.0	232228.7	212246.7	231264.8	214006.8	234986.3	252576.9	280722.0	264774.1	285853.1
Q9CXR1	Dehydrogenase/reductase SDR family member 7	Dhrs7	338	38.1	8.32	233398.6	225900.1	205081.5	220946.5	215903.1	198543.4	219764.3	216130.3	219518.5	218962.9	401807.1	456445.0	378017.6	413298.8
Q62048	Astrocytic phosphoprotein PEA-15	Pea15	130	15	5.02	301675.0	279227.2	291943.2	303682.8	294677.2	324696.6	341659.2	382094.2	338227.3	256291.8	230865.1	210506.5	203663.3	236495.2
Q3TTP0	Testicular spindle-associated protein SHCBP1L	Shcbp1l	639	70.9	5.2	133774.7	159547.4	171191.9	213996.0	97801.5	98097.6	97024.0	107669.6	136083.7	143931.5	192405.6	298612.2	303906.1	415483.9
P49443	Protein phosphatase 1A	Ppm1a	382	42.4	5.36	517531.1	542426.4	494799.4	479591.1	613167.8	591357.1	546827.5	575426.3	521014.6	554815.2	327952.8	319480.4	335415.1	302451.0
E9Q735	Ubiquitin conjugation factor E4 A	Ube4a	1028	118.1	5.33	123408.5	141133.4	123287.1	137951.7	116302.7	123092.8	108280.2	114501.8	126773.6	134569.3	111047.8	108517.0	109572.6	100551.9
Q8BTY8	Sec1 family domain-containing protein 2	Scfd2	684	74.7	6.81	172634.9	206374.6	168336.3	190897.8	174698.1	167639.6	162065.9	165612.4	171680.7	172503.6	193718.6	208651.9	177841.5	200051.9
Q8BX09	Retinoblastoma-binding protein 5	Rbbp5	538	59.1	5.1	358454.3	325871.6	244099.3	332489.9	371275.1	369070.0	357311.5	315521.3	327783.1	333408.9	229482.6	238762.2	245758.9	253715.4
O35326	Serine/arginine-rich splicing factor 5	Srsf5	269	30.9	11.56	883389.9	874441.2	1068315.9	1016282.4	1130694.8	923280.4	961861.2	894648.7	996336.0	1031338.2	805959.3	881197.2	918710.3	911538.8
Q9D968	Host cell factor 2	Hcfc2	722	79.4	8.35	154675.0	174174.2	185187.2	171036.3	187978.0	167952.3	152841.7	149464.7	169442.6	173821.7	61432.0	68747.5	69878.9	66347.5
P06684	Complement C5	C5	1680	188.8	6.81	186629.6	201732.8	186907.7	183650.9	183844.1	194483.4	200878.9	192515.9	185410.9	203191.3	79184.9	87232.2	77398.6	67990.7
Q00422	GA-binding protein alpha chain	Gabpa	454	51.3	5	669013.6	693830.2	743773.4	672563.2	836989.3	847825.8	753011.4	677927.2	727941.8	731910.1	197784.1	189608.3	222538.0	179584.0
Q9WUK4	Replication factor C subunit 2	Rfc2	349	38.7	6.44	130834.9	150229.3	133270.0	132065.1	154439.8	145606.9	127283.8	130217.7	133900.1	141901.9	189838.6	223555.7	205197.1	197425.6
Q9DBY8	Nuclear valosin-containing protein-like	Nvl	855	94.4	6.35	106071.8	116497.1	97966.4	94834.1	120976.0	124267.1	107806.5	102802.7	107792.7	119915.0	89346.8	114556.6	95692.6	100648.3
Q7JJ13	Bromodomain-containing protein 2	Brd2	798	88	9.13	254471.5	250974.9	269984.7	260293.1	268490.4	280519.3	243562.0	225728.5	247432.4	261482.9	257522.8	273390.2	260142.9	264713.2
P30677	Guanine nucleotide-binding protein subunit alpha-14	Gna14	355	41.5	6.3	52326.6	49437.0	46210.1	42767.7	49590.6	51387.3	69822.0	45220.6	49357.9	50172.9	382107.0	317752.0	376292.8	308146.5
Q8K592	Anti-Muellerian hormone type-2 receptor	Amhr2	568	61.2	5.43	122109.3	132627.9	119518.2	145730.8	130878.3	110603.2	121949.0	113833.8	119933.0	134561.1	179764.4	166064.1	145897.2	175817.7
Q8BG81	Polymerase delta-interacting protein 3	Poldip3	420	46.1	10.05	391625.5	375375.6	396668.8	391700.0	410099.7	401872.8	422980.0	392357.0	416706.9	418071.8	385408.5	448360.6	381938.5	388310.5
Q8BP40	Lysophosphatidic acid phosphatase type 6	Acp6	418	47.6	7.72	146203.4	138075.1	145971.4	141653.4	180796.5	159643.9	153960.4	133890.4	158597.9	141624.5	170125.7	159384.1	169684.1	147784.9
Q9Z321	DNA topoisomerase 3-beta-1	Top3b	862	96.9	7.93	269911.3	310218.1	306363.2	308420.5	298428.8	282362.4	245563.9	273775.3	289008.9	296102.8	150422.5	155157.3	161985.3	156358.1
Q8VDL4	ADP-dependent glucokinase	Adpgk	496	53.9	5.62	292876.1	320573.4	302186.0	361882.5	246886.3	237592.7	213472.4	235138.3	259617.6	298918.0	287670.5	299694.0	242318.9	294574.4
Q99104	Unconventional myosin-Va	Myo5a	1853	215.4	8.63	121322.6	143968.4	132580.4	141240.4	141729.9	142141.4	116275.7	133459.6	132438.2	129361.2	121670.4	121035.6	113719.3	108222.5
Q9JLT4	Thioredoxin reductase 2, mitochondrial	Txnrd2	524	56.6	8.46	169095.3	133382.3	153685.1	142821.1	200938.5	180976.2	169392.9	156180.8	156076.8	141065.3	117066.3	99968.3	117879.2	120316.7
Q8BSE0	Regulator of microtubule dynamics protein 2	Rmdn2	410	47	7.56	159204.8	158042.1	132378.1	155093.5	158672.2	140895.6	144460.3	138673.9	143761.8	143342.8	293681.6	266561.9	211242.4	254773.9
Q91XW8	Inactive peptidyl-prolyl cis-trans isomerase FKBP6	Fkbp6	327	37.1	8	632665.7	642921.6	660314.6	672535.2	646443.3	633393.5	566416.6	582147.3	657942.1	697097.6	591659.3	583387.2	536246.0	561423.4
Q8BRN9	Coiled-coil and C2 domain-containing protein 1B	Cc2d1b	848	93	5.41	206086.2	199301.7	200494.9	208556.4	204001.8	212534.5	210987.1	197145.8	199080.1	206737.2	202565.9	188525.1	189545.3	196167.1
Q9CWX9	Probable ATP-dependent RNA helicase DDX47	Ddx47	455	50.6	9.1	111295.8	116753.7	108387.6	105892.7	117392.7	110073.6	101929.4	97264.9	106203.3	118929.1	89189.5	87616.0	92103.2	86679.7
Q6P069	Sorcin	Sri	198	21.6	5.59	395537.7	370401.8	365952.3	333667.8	413442.5	422228.1	456360.6	402350.2	396917.0	388189.9	415317.7	407538.2	395306.8	395574.8
Q91VJ4	Serine/threonine-protein kinase 38	Stk38	465	54.1	7.34	159645.8	158833.6	135856.4	150352.9	149910.0	149176.8	146939.0	130443.5	140226.3	150027.9	131370.2	145660.5	145291.0	134334.1
O89017	Legumain	Lgmn	435	49.3	6.39	152237.0	157516.8	159634.3	165072.3	169639.0	176183.0	184984.7	176091.1	172175.1	163831.7	102469.1	88856.9	78718.2	84417.6
Q9CR57	60S ribosomal protein L14	Rpl14	217	23.5	11.02	403840.5	453232.9	374024.1	454815.3	353738.1	347456.2	371478.6	376466.0	433115.3	466157.2	804637.4	901700.0	893852.9	903666.4
P48428	Tubulin-specific chaperone A	Tbca	108	12.8	5.27	429914.4	477291.8	561338.4	514489.4	446442.9	421289.9	406746.0	471810.1	486046.2	459737.2	279605.1	316091.5	336010.9	348438.4
Q8K0C4	Lanosterol 14-alpha demethylase	Cyp51a1	503	56.7	8.41	279228.4	277930.9	232623.5	310345.2	237142.0	215656.9	224258.3	258741.5	232900.0	257310.1	227885.9	236492.9	214745.8	264885.9
P62843	40S ribosomal protein S15	Rps15	145	17	10.39	542042.1	592324.2	520567.3	595234.2	521413.6	535912.4	597747.9	523677.2	575619.1	610930.4	196492.8	165606.6	218076.1	128029.5
Q8K299	Scavenger receptor class A member 5	Scara5	491	53.6	6.76	205995.8	175966.8	179316.3	177710.1	158931.9	143996.8	149491.2	181318.5	173906.0	176922.7	166118.3	150448.5	134458.3	164581.8
P32883	GTPase KRas	Kras	189	21.6	6.77	48054.3	42328.3	50733.6	44339.3	55850.6	51443.6	51597.5	46212.9	48742.9	49028.3	246944.4	256163.2	260489.6	239845.4
Q9QUJ7	Long-chain-fatty-acid--CoA ligase 4	Acsl4	711	79	8.28	121528.5	123806.6	122527.0	119868.8	131380.0	121317.5	118039.3	113914.3	120355.9	132960.8	197503.8	209582.5	171293.9	196071.9
Q8BTV2	Cleavage and polyadenylation specificity factor subunit 7	Cpsf7	471	52	8	171663.3	166951.0	191433.1	164026.3	193274.7	164775.5	163860.5	153347.2	176963.4	183951.1	167028.3	166109.4	181736.5	162510.7
Q8VCM8	Nicalin	Ncln	563	62.9	6.49	114821.1	116233.8	108362.0	120988.8	112155.5	119185.3	113822.0	105554.8	111721.1	120011.4	275968.0	282774.7	269029.5	279821.6
V9GX06	Gp_dh_C domain-containing protein	Gm11214	183	19.9	7.42	40616.5	34880.0	26569.4	37274.1	36065.3	37185.4	36808.9	30758.9	33011.1	39623.7	#N/A	#N/A	#N/A	#N/A
P00920	Carbonic anhydrase 2	Ca2	260	29	7.01	187439.2	143396.6	116240.4	214392.1	140030.2	157945.9	263542.7	191175.2	197085.0	173304.7	424706.2	368965.5	348953.8	440599.0
Q9JJ78	Lymphokine-activated killer T-cell-originated protein kinase	Pbk	330	36.7	5.12	552489.8	710445.4	767359.2	828756.0	520668.0	486513.0	437252.7	501756.9	640024.1	686628.3	427714.4	605912.6	627512.7	727122.1
Q62388	Serine-protein kinase ATM	Atm	3066	349.2	6.86	196698.1	196312.4	174429.8	169484.2	194718.9	186861.8	171026.8	171416.3	198829.4	188108.2	157307.2	149798.1	135597.4	135958.8
Q8VDC0	Probable leucine--tRNA ligase, mitochondrial	Lars2	902	101.4	8.19	173837.2	193018.0	186081.2	178311.2	204238.9	197100.6	176766.6	179467.7	173856.9	176975.9	106530.7	103227.7	104138.4	96941.3
Q6DVA0	LEM domain-containing protein 2	Lemd2	511	57.5	9.01	386138.8	350556.6	354064.7	332093.6	331972.7	319716.9	353595.0	349377.2	336120.8	331447.7	271736.2	247553.2	254318.0	248089.5
Q8VHI3	GDP-fucose protein O-fucosyltransferase 2	Pofut2	429	49.4	6.48	163622.4	147040.3	150584.2	131978.3	139827.2	150213.8	125772.9	144301.5	129516.3	140867.2	144947.6	135741.9	127672.7	148983.9
P19783	Cytochrome c oxidase subunit 4 isoform 1, mitochondrial	Cox4i1	169	19.5	9.23	1157078.1	1366188.9	1555799.0	1604585.3	1222301.1	1093310.6	1263097.6	1355909.6	1412260.9	1431542.7	1239803.0	1288858.1	1397127.6	1426571.2
Q8K4P0	pre-mRNA 3' end processing protein WDR33	Wdr33	1330	145.2	9.13	282375.4	384867.5	299498.1	352073.9	342834.2	325268.6	295660.4	287744.8	307619.8	354769.2	86321.5	96769.6	89459.0	100900.3
Q99P31	Hsp70-binding protein 1	Hspbp1	357	39.1	5.36	100971.1	131414.1	95113.1	114928.4	125946.7	111908.4	100466.3	104254.1	100737.4	115855.3	151651.2	142604.9	120287.3	146373.6
P62317	Small nuclear ribonucleoprotein Sm D2	Snrpd2	118	13.5	9.91	1352932.9	1359770.0	1398745.4	1449928.8	1547530.5	1387776.0	1272872.4	1344655.2	1323433.9	1396074.4	1184610.5	1391165.9	1389460.5	1378235.2
Q7TQK5	Coiled-coil domain-containing protein 93	Ccdc93	629	72.6	8.29	124892.7	129638.7	124080.7	126959.0	139565.0	134762.3	142226.8	117579.0	132101.8	128087.6	208677.3	201535.5	188312.2	247491.2
Q9ERI6	Retinol dehydrogenase 14	Rdh14	334	36.3	8.18	163048.6	153773.9	165588.2	154310.8	170358.4	162221.7	183547.5	150905.5	153623.6	168055.5	107072.5	108209.2	113485.6	116523.3
Q9D3E6	Cohesin subunit SA-1	Stag1	1258	144.3	5.59	274653.6	281863.8	325370.0	279155.6	366900.0	402634.0	375467.1	315991.3	315960.6	298490.7	620992.4	571581.3	584245.3	553370.4
Q8R1U1	Conserved oligomeric Golgi complex subunit 4	Cog4	785	88.6	5.15	114996.6	122684.1	110782.5	119631.5	113812.3	104597.6	95983.8	105841.3	111542.9	113542.5	164238.1	181802.7	182682.1	181239.9
Q5DTX6	Junctional protein associated with coronary artery disease	Jcad	1320	144.7	6.52	353204.4	334718.9	300198.9	329612.0	304151.8	319434.9	310644.4	285527.9	281697.6	305457.9	167878.8	144052.8	152042.3	153788.5
Q9ERR7	Selenoprotein F	Selenof	162	17.8	5.35	344110.3	371945.2	347845.0	349264.4	369491.9	364870.2	311318.0	345371.7	348047.3	344656.2	391496.0	429564.9	370128.4	398537.6
Q8VCH8	UBX domain-containing protein 4	Ubxn4	506	56.4	6.61	391844.2	474867.2	387321.6	427374.5	457626.5	388424.2	361446.9	384601.1	408285.4	469194.8	160651.0	202847.6	148244.7	180046.6
Q3UMY5	Echinoderm microtubule-associated protein-like 4	Eml4	988	110	6.54	206092.5	211008.0	194707.6	203783.6	219531.3	218781.7	206517.1	208910.8	206401.7	207223.4	344706.7	305238.3	316504.2	298526.9
P31750	RAC-alpha serine/threonine-protein kinase	Akt1	480	55.7	5.9	106974.6	95902.7	99523.3	104961.2	102437.2	105159.3	102952.0	108673.0	97751.8	100458.8	246658.1	259300.8	261915.5	227543.4
Q61686	Chromobox protein homolog 5	Cbx5	191	22.2	5.86	221363.6	217952.4	179094.9	217809.2	210532.8	210804.4	224397.8	196151.7	188287.4	222420.9	298948.7	288488.5	300109.9	265402.1
Q61703	Inter-alpha-trypsin inhibitor heavy chain H2	Itih2	946	105.9	7.27	203625.8	187484.7	157870.9	156269.3	121496.6	152782.2	158614.4	199875.3	142427.5	162675.4	124934.2	145026.1	125124.1	125707.1
Q8C050	Ribosomal protein S6 kinase alpha-5	Rps6ka5	863	96.5	7.34	162941.2	164598.4	149857.3	162375.7	163564.4	159369.4	152208.3	152495.2	159859.1	173379.0	131264.1	139042.0	130853.7	149990.6
Q8VCN9	Tubulin-specific chaperone C	Tbcc	341	38.1	5.3	77165.3	87300.8	69667.6	78919.3	76305.7	81278.8	73213.9	69546.0	77888.7	80079.7	77456.4	75743.1	66402.3	75902.0
Q8R164	Valacyclovir hydrolase	Bphl	291	32.8	8.94	288139.0	280905.4	262802.1	289498.6	320663.7	331894.8	333905.4	309636.0	294228.1	303727.5	304509.9	263291.0	282397.4	282622.1
Q8VHN8	Tudor-interacting repair regulator protein	Nudt16l1	211	23.4	9.26	343026.1	389744.3	378957.7	419081.6	366070.5	346012.5	332813.1	316305.2	359402.7	387181.6	312269.1	313959.5	312276.4	360771.6
P84244	Histone H3.3	H3-3b	136	15.3	11.27	4012058.4	3827432.4	4849838.8	3734155.2	5988248.6	5606963.1	4886687.6	4120445.5	4553513.3	5148382.2	43550.3	60494.3	55147.1	28603.7

Q8CCN5	Breast carcinoma-amplified sequence 3 homolog	Bcas3	928	101	6.7	267005.7	271034.6	264467.4	273296.6	267909.6	266521.4	244243.1	262937.7	262834.1	264204.6	127409.8	125676.7	126168.6	123281.1
B1ART2	UBA domain-containing protein	Vps13d	4390	490.7	6.55	120751.1	132071.2	135351.8	131666.3	142533.3	132342.9	131880.1	128857.9	126786.7	133817.0	180864.4	243181.4	177036.4	184963.2
Q61151	Serine/threonine-protein phosphatase 2A 56 kDa regulatory subunit epsilon isoform	Ppp2r5e	467	54.7	6.95	175573.1	178100.2	174436.1	174003.6	189362.3	189653.4	182041.5	172210.5	181809.9	184388.8	110631.7	142165.3	103191.1	108028.8
P63323	40S ribosomal protein S12	Rps12	132	14.5	7.24	1429900.9	1713954.4	1480782.4	1701428.3	1352769.1	1295535.2	1406078.8	1397077.4	1604121.1	1636337.7	842479.8	1061047.9	757451.8	863735.9
F8VQ05	FRY-like transcription coactivator	Fryl	3007	337.6	5.63	345531.2	373446.8	312084.8	355873.8	312282.7	358513.7	334542.8	307193.9	333413.8	335914.2	197055.1	203097.7	165835.6	206603.2
Q6PNC0	DmX-like protein 1	Dmxl1	3013	335.8	6.42	112404.0	113660.2	94761.0	106309.0	103442.4	105885.6	96958.3	99229.0	111913.1	108195.0	114663.0	115162.3	115757.5	107785.2
Q8R3G1	Nuclear inhibitor of protein phosphatase 1	Ppp1r8	351	38.5	7.37	261139.3	246279.6	258816.7	236184.6	257761.1	251859.5	240066.0	228707.7	257191.4	287839.1	172391.1	159364.5	176428.7	162635.9
Q8VE97	Serine/arginine-rich splicing factor 4	Srsf4	489	55.9	11.4	374930.9	645002.1	450718.0	549690.3	560622.3	505426.7	425441.3	435775.9	513928.0	580410.4	123338.1	137935.5	155946.7	140316.3
Q8CI43	Myosin light chain 6B	Myl6b	207	22.7	5.53	216433.5	288213.6	257334.5	338568.9	209919.9	202392.1	226119.0	191058.2	249482.2	241968.8	147119.2	191554.6	171412.7	213916.0
Q8R0A0	General transcription factor IIF subunit 2	Gtf2f2	249	28.4	9.22	542848.0	537652.1	578272.2	573904.2	604925.2	578255.3	503851.8	512238.4	549652.8	572947.3	276681.2	262744.7	272501.5	255938.0
Q9R1T4	Septin-6	Septin6	434	49.6	6.43	79561.2	89482.6	81418.5	83557.3	82313.7	88945.9	85699.1	80553.5	82702.1	88017.0	107553.8	98245.0	91890.2	103175.7
P47934	Carnitine O-acetyltransferase	Crat	626	70.8	8.44	155046.1	148067.6	171153.4	176807.4	134081.9	136901.0	133585.6	152421.6	157713.7	152495.5	259267.7	277673.9	286200.8	294666.9
Q6R0H7	Guanine nucleotide-binding protein G(s) subunit alpha isoforms XLas	Gnas	1133	121.4	4.81	401482.3	357279.6	388734.5	346065.5	464531.6	458447.1	476450.1	397974.5	399153.8	395312.2	552053.8	490606.4	478295.7	476154.4
P45878	Peptidyl-prolyl cis-trans isomerase FKBP2	Fkbp2	140	15.3	8.88	795540.6	806171.4	757628.5	845996.3	736558.1	744559.2	713226.0	814034.3	755621.6	754480.5	453139.3	486393.1	418773.6	445476.2
P47968	Ribose-5-phosphate isomerase	Rpia	303	32.4	8.02	94563.8	143961.8	106252.9	113237.1	117985.8	138452.5	91306.6	122276.5	107254.3	117300.1	99217.3	123594.6	114542.7	129643.0
E9PXF8	Myotubularin-related protein 13	Sbf2	1872	210.3	7.28	167376.6	173159.0	153474.0	157169.0	185001.3	196942.2	165763.6	149240.2	159172.9	171756.2	60795.2	67314.2	56865.0	68949.7
P62869	Elongin-B	Elob	118	13.2	5.01	1284373.5	1381161.4	1655644.7	1420280.9	1455499.7	1296892.5	1054577.0	1359210.4	1334413.6	1350408.2	1231706.6	1466568.8	1531743.5	1538214.0
O89079	Coatomer subunit epsilon	Cope	308	34.5	5.06	221422.6	243926.9	250029.1	256092.6	252625.2	235614.8	215639.3	224184.3	270537.9	252824.3	277638.3	289369.0	276070.7	298104.4
P59016	Vacuolar protein sorting-associated protein 33B	Vps33b	617	70.5	6.86	156477.6	161961.7	162945.4	158895.3	161116.4	170626.4	147748.0	150150.5	161750.3	174161.8	316245.9	332564.5	349832.7	373622.1
P33174	Chromosome-associated kinesin KIF4	Kif4	1231	139.4	6.68	100546.7	110402.6	106394.2	103717.8	129180.4	126815.0	108285.7	99085.4	108035.9	106045.3	121052.0	130769.4	129465.4	133391.5
P49138	MAP kinase-activated protein kinase 2	Mapkapk2	386	44	8.57	526094.8	506069.7	493636.9	496488.3	471128.2	459367.6	457177.4	494802.2	503243.2	492750.4	383505.8	342143.6	333589.3	307889.6
Q91YN9	BAG family molecular chaperone regulator 2	Bag2	210	23.5	6.42	546059.6	619156.7	545523.1	610386.3	489175.0	450371.6	512940.4	546588.7	573967.8	611597.9	291940.1	268284.7	256034.0	267430.6
Q99LB6	Methionine adenosyltransferase 2 subunit beta	Mat2b	334	37.4	6.95	449438.1	446591.5	417695.4	426369.6	483029.8	473407.6	505510.8	408596.9	461518.9	484137.9	310122.9	322067.6	290622.0	302424.6
Q9WVQ5	Methylthioribulose-1-phosphate dehydratase	Apip	241	26.9	6.9	281425.8	315030.0	257922.3	299569.4	309840.2	302215.1	280320.8	280815.6	277542.2	300192.9	172656.4	181435.5	188234.5	181161.8
Q9D3R9	Transcription initiation factor TFIID subunit 7-like	Taf7l	471	52.9	5.12	129560.4	139752.3	132367.1	126933.9	149832.1	150953.1	122900.6	118301.4	140189.3	136567.2	180047.3	210946.1	200644.4	179874.7
P21300	Aldo-keto reductase family 1 member B7	Akr1b7	316	36	7.25	339168.4	371135.0	309482.8	337400.9	311073.4	299444.1	331133.4	358176.9	323418.5	367388.6	67187.6	60331.3	54116.3	60528.5
Q9CZY3	Ubiquitin-conjugating enzyme E2 variant 1	Ube2v1	147	16.3	7.96	667284.5	698219.0	645077.1	710686.7	668257.6	650177.7	672185.9	635898.1	681260.6	704063.5	250675.7	340500.6	242081.6	290640.9
O09167	60S ribosomal protein L21	Rpl21	160	18.6	10.49	1121620.6	1018763.5	1215450.9	1121617.7	1125683.3	1105723.3	966678.5	1221027.0	1107491.6	1017621.1	1213218.7	1194547.3	1324100.4	1286099.8
Q8VDQ8	NAD-dependent protein deacetylase sirtuin-2	Sirt2	389	43.2	5.35	243151.2	270978.1	258201.8	275646.4	266939.0	268430.9	262427.0	237044.0	261748.7	263046.4	434608.4	477326.8	466212.4	498905.9
P97393	Rho GTPase-activating protein 5	Arhgap5	1501	172	6.34	232474.9	258125.4	215515.2	222362.6	226118.2	234811.1	229721.8	211537.9	240477.3	257531.5	336360.2	346678.8	319511.0	317788.1
Q8BH24	Transmembrane 9 superfamily member 4	Tm9sf4	643	74.6	7.23	212407.0	363189.1	223987.5	261574.7	275710.4	345800.5	197767.1	282572.1	256229.4	286617.7	339309.6	579116.9	275576.9	340603.2
Q8BL97	Serine/arginine-rich splicing factor 7	Srsf7	267	30.8	11.9	1235686.7	1282015.1	1446993.9	1286772.6	1376518.2	1353215.9	1365613.3	1247404.4	1334050.1	1397249.8	568394.8	662748.0	655690.2	678692.8
Q9R0A0	Peroxisomal membrane protein PEX14	Pex14	376	41.2	5.11	638561.3	665464.7	654322.4	637863.9	627977.7	537507.3	607829.9	609238.3	616820.5	649680.7	566273.1	583547.7	549932.2	596824.1
Q3V0C5	Ubiquitin carboxyl-terminal hydrolase 48	Usp48	1052	120.6	6.25	126180.7	126567.4	133550.7	117087.9	149902.4	157326.2	138953.8	129728.9	135693.0	128761.3	306481.4	297374.8	305390.6	285243.7
P04104	Keratin, type II cytoskeletal 1	Krt1	637	65.6	8.15	657978.7	619571.3	685133.9	688258.7	1448200.2	1618493.0	662688.8	1106090.5	851892.4	876339.7	5017891.7	5623963.9	5124516.6	7819241.4
Q8C0L0	Thioredoxin-related transmembrane protein 4	Tmx4	335	37.1	4.37	347078.9	339185.8	393902.5	322200.1	390435.4	429120.7	341447.5	339805.6	337332.1	364796.8	733030.7	703714.2	749562.6	771258.5
A2BH40	AT-rich interactive domain-containing protein 1A	Arid1a	2283	241.9	6.68	193061.6	191686.2	193937.5	199404.3	194236.7	201143.4	202048.0	187385.6	193261.5	194690.1	112484.9	112432.6	114618.7	102040.8
P61226	Ras-related protein Rap-2b	Rap2b	183	20.5	4.81	259929.5	253789.2	251297.8	234333.5	283223.3	292489.3	288057.3	269064.9	267886.4	268144.9	395902.3	369085.3	366798.3	348169.2
Q9DCS9	NADH dehydrogenase [ubiquinone] 1 beta subcomplex subunit 10	Ndufb10	176	21	8.03	325033.3	383621.2	363539.6	379457.4	406686.3	375312.1	353341.5	388272.4	374210.3	358735.0	347735.6	369777.3	333272.6	379836.8
O08579	Emerin	Emd	259	29.4	5.01	186925.8	151355.0	206344.9	172247.7	162405.0	191378.4	202052.1	193091.8	181921.9	155688.1	280867.0	272449.4	273078.4	269746.1
Q9D0B0	Serine/arginine-rich splicing factor 9	Srsf9	222	25.6	8.65	616487.4	639717.4	618213.5	601452.8	665617.2	598431.0	599421.8	565576.7	650623.2	683836.9	855231.1	840539.2	856431.6	808843.0
Q9Z0H4	CUGBP Elav-like family member 2	Celf2	508	54.2	8.76	272367.2	278126.9	269162.2	273702.4	297986.9	276026.3	251812.2	261481.8	262312.8	270206.9	425530.8	482998.2	465337.7	446190.8
Q8BGT5	Alanine aminotransferase 2	Gpt2	522	57.9	8	471105.0	498083.8	353180.0	509138.9	354761.0	319536.2	336026.6	385124.4	386098.2	397802.4	277248.9	288170.5	235220.8	296151.2
Q8VDC1	FYVE and coiled-coil domain-containing protein 1	Fyco1	1437	162.2	4.98	137226.4	148602.6	131935.7	137694.9	130332.2	137153.8	130433.8	132182.7	133114.7	144573.3	88187.9	103505.4	82130.9	90828.8
Q60823	RAC-beta serine/threonine-protein kinase	Akt2	481	55.7	6.37	334517.8	295646.5	337998.6	312884.3	357237.4	381663.8	347968.8	342235.1	320407.4	321536.2	321839.7	333789.0	326137.5	295202.3
Q6QI06	Rapamycin-insensitive companion of mTOR	Rictor	1708	191.4	7.23	87977.8	89646.4	101649.7	91921.7	96763.8	100697.7	96371.4	91679.1	94828.6	88101.9	290143.7	281676.8	250573.7	272369.1
Q52KI8	Serine/arginine repetitive matrix protein 1	Srrm1	946	106.8	11.87	261731.2	246810.5	232100.4	242137.7	205623.1	186254.2	204067.8	199068.3	214974.1	232225.4	669814.5	684633.8	732599.3	722689.1
P70318	Nucleolysin TIAR	Tial1	392	43.4	7.99	517280.5	544539.9	549119.9	529601.3	529966.3	530169.2	524174.7	498890.5	574876.4	567631.0	467850.3	497846.3	457937.0	459134.5
Q5U4D9	THO complex subunit 6 homolog	Thoc6	341	37.3	7.12	236157.8	232957.4	241300.4	232480.2	259864.1	254212.8	267364.9	239854.9	244913.0	248449.8	218882.1	228049.7	208367.6	197089.6
O09172	Glutamate--cysteine ligase regulatory subunit	Gclm	274	30.5	5.52	418636.8	423367.3	485672.6	446284.2	456774.9	501023.8	388613.4	419185.2	414873.6	438370.3	378354.7	480579.0	414038.2	478129.4
Q9CWH6	Proteasome subunit alpha type-8	Psma8	250	27.8	8.69	113866.9	154445.9	207987.8	180435.2	115475.3	79891.2	76934.8	112509.8	131339.4	133914.0	77432.9	98819.4	96788.9	115528.8
P70700	DNA-directed RNA polymerase I subunit RPA2	Polr1b	1135	128.1	7.58	135339.5	133323.3	150732.6	138788.0	163070.9	155640.5	131685.6	134262.2	142164.8	145582.8	96392.1	105936.0	95983.5	93193.4
Q8CI32	BAG family molecular chaperone regulator 5	Bag5	447	50.9	6.05	130729.7	143319.9	143821.9	150190.2	136042.5	133690.6	136444.3	128579.4	129916.8	135827.2	73333.3	67415.8	67158.7	72054.6
Q68FL4	Putative adenosylhomocysteinase 3	Ahcyl2	613	66.9	7.36	256851.7	258968.8	225883.0	248508.6	238070.0	240846.6	249964.9	218709.9	237613.5	234439.4	127202.7	145371.3	118047.1	111568.5
Q8CI04	Conserved oligomeric Golgi complex subunit 3	Cog3	820	93.2	5.78	147307.7	161351.6	154373.5	158316.3	154533.3	142338.9	132823.2	140891.7	141250.3	147771.3	337890.3	356097.5	334049.0	348146.5
Q8K205	Processing of 1, ribonuclease P/MRP family, (S. cerevisiae)	Pop1	1045	117.3	9.11	73705.5	77657.6	79835.2	83792.2	93566.8	81112.8	69541.7	76967.2	83670.9	79731.0	108579.8	126681.2	112491.3	119566.8
Q922V4	Pleiotropic regulator 1	Plrg1	513	56.9	9.17	424461.1	323864.0	364178.4	453022.1	329647.1	318851.7	305596.1	309017.8	405689.3	333118.2	373268.1	354590.3	374110.9	392539.3
Q921L5	Conserved oligomeric Golgi complex subunit 2	Cog2	731	82	6.21	137770.1	176094.7	169207.2	155152.3	143404.5	150223.4	148379.2	163478.5	157409.7	160856.4	58598.3	91489.8	77533.9	86456.5
Q64674	Spermidine synthase	Srm	302	34	5.5	310689.6	287700.6	261797.5	254834.7	258275.7	254373.7	251517.2	249924.5	269128.7	262058.8	181897.6	170292.7	169104.7	186892.3
Q8C5D8	E3 SUMO-protein ligase PIAS2	Pias2	621	68.4	7.65	60272.6	75345.6	61143.5	64727.8	74865.9	67704.5	58135.5	65140.6	57290.0	70505.2	55275.6	50562.2	54755.7	60212.4
Q9CQF3	Cleavage and polyadenylation specificity factor subunit 5	Nudt21	227	26.2	8.82	876563.8	963118.5	1090845.7	1010590.4	1076609.3	966159.8	1112997.0	968076.1	1156445.5	1070183.8	818414.7	869583.4	1029748.0	870579.2
Q8BQ46	TAF15 RNA polymerase II, TATA box binding protein (TBP)-associated factor	Taf15	557	58.6	8.4	180981.9	190876.3	187796.6	193682.9	195762.1	180336.9	178446.2	166279.1	183821.1	182448.3	122109.5	119308.1	146346.3	141574.4
P47941	Crk-like protein	Crkl	303	33.8	6.74	250957.7	245245.1	241090.4	242908.8	241905.5	245525.8	256164.8	224528.4	251227.0	271533.9	301713.2	298141.1	287968.4	276852.5
Q9QZ88	Vacuolar protein sorting-associated protein 29	Vps29	182	20.5	6.79	183299.0	227808.8	176815.1	194103.9	213683.7	205444.5	154507.3	181484.3	188955.3	197335.1	673809.6	852372.8	807107.1	801626.6
Q60634	Flotillin-2	Flot2	428	47	5.2	131092.0	133373.8	161913.1	116642.2	165276.4	155442.5	160704.8	138358.6	134094.9	130305.2	95849.4	110382.6	103411.6	106104.6
Q9JI46	Diphosphoinositol polyphosphate phosphohydrolase 1	Nudt3	168	19	6.34	913670.9	928147.7	839107.4	909991.2	963842.7	1013313.0	1010509.2	872582.1	903665.1	1010834.7	305577.9	289356.7	298715.7	320228.1
A2A8L5	Receptor-type tyrosine-protein phosphatase F	Ptprf	1898	211.4	6.65	67884.1	69191.8	68669.5	68265.3	78746.5	70652.4	75268.5	72411.1	74906.7	70622.1	51920.8	52701.1	49130.5	44919.3
P61620	Protein transport protein Sec61 subunit alpha isoform 1	Sec61a1	476	52.2	8.06	493691.4	551165.4	455966.2	480140.0	467431.5	469627.9	436883.5	469103.8	529903.4	449565.1	230230.8	238512.4	269264.5	237858.6
Q8BW75	Amine oxidase [flavin-containing] B	Maob	520	58.5	8.29	299539.5	266057.4	255805.8	323426.7	272172.9	334858.9	362253.6	352143.4	265623.9	246758.6	240539.9	223936.4	207269.9	238446.4
Q9CZM2	60S ribosomal protein L15	Rpl15	204	24.1	11.62	678955.7	731798.0	804929.7	766425.3	640850.0	626445.0	594048.8	766524.2	694426.7	696797.9	828084.3	883582.6	864346.5	931199.5
Q14DK4	Glycerol-3-phosphate acyltransferase 2, mitochondrial	Gpat2	801	89.1	6.99	50312.4	62343.6	50112.2	54609.2	53035.3	52644.6	45618.8	49730.3	47607.4	50696.7	45230.1	50077.8	41729.0	47014.8
Q04207	Transcription factor p65	Rela	549	60.2	5.76	109038.6	118231.9	108741.2	111491.9	106008.9	123153.6	113260.0	105348.0	128491.6	110804.8	152349.7	172772.8	151928.5	168995.1
P62849	40S ribosomal protein S24	Rps24	133	15.4	10.78	327356.4	401655.8	324619.9	375787.3	370685.6	351116.2	343153.4	350496.3	354217.9	380471.0	540644.7	975587.5	490315.3	655091.8
Q9Z2Z9	Glutamine--fructose-6-phosphate aminotransferase [isomerizing] 2	Gfpt2	682	77	7.15	145983.7	129480.2	127264.6	135422.4	138468.3	138148.8	131452.1	122098.3	133721.5	139173.0	189690.3	212955.3	178459.5	178542.0
Q9D967	Magnesium-dependent phosphatase 1	Mdp1	164	18.6	6.8	332851.4	381722.7	380720.6	370582.5	386886.8	387464.6	407617.6	356786.5	420659.0	381231.3	275235.7	334398.0	275248.7	315327.3
Q7TQ95	Endoplasmic reticulum junction formation protein lunapark	Lnpk	425	47.5	5.27	89920.0	98695.2	94955.4	109191.0	92718.8	86158.7	83223.1	80859.0	95856.3	98453.3	172244.2	96102.8	92594.1	93787.1
Q62087	Serum paraoxonase/lactonase 3	Pon3	354	39.3	5.74	249179.5	226088.9	173215.3	239380.4	186661.4	188602.1	205316.3	183982.8	222071.1	216413.2	110786.2	133546.3	85226.0	130158.1
Q61771	Kinesin-like protein KIF3B	Kif3b	747	85.2	7.69	288059.8	325207.9	326102.5	348557.2	264300.6	261843.4	256509.2	282951.6	300319.2	310425.0	105969.9	83159.3	127735.3	107569.4
Q3UHB1	5'-nucleotidase domain-containing protein 3	Nt5dc3	546	63.1	8.56	260492.1	279665.5	222595.4	234581.5	251029.6	280744.7	219740.1	275979.0	217470.1	247068.1	247439.5	238695.5	193422.3	223375.1
Q8C863	E3 ubiquitin-protein ligase Itchy	Itch	864	98.9	6.28	136078.3	143366.9	126480.9	116683.1	130968.8	136640.6	123163.6	118306.2	127921.7	131323.4	185327.6	202141.4	196681.4	208925.3
Q9DB41	Mitochondrial glutamate carrier 2	Slc25a18	320	34.1	9.19	95154.9	95918.1	87183.4	96790.7	89523.4	92102.3	81266.4	92027.6	81365.6	90150.8	98236.6	100404.0	98384.6	103734.4
Q8BZH4	Pogo transposable element with ZNF domain	Pogz	1409	154.8	7.52	129945.2	134351.7	131515.2	118458.3	150764.6	137747.2	133939.5	138126.3	132042.1	143553.1	44302.2	43418.8	41574.8	46879.8
Q9CRB6	Tubulin polymerization-promoting protein family member 3	Tppp3	176	19	9.11	529730.6	453699.5	574912.2	358692.7	755468.3	1403828.5	1050678.2	652495.3	615175.2	594767.9	239485.9	271557.2	196731.1	216892.2
Q9CXW2	28S ribosomal protein S22, mitochondrial	Mrps22	359	41.2	8.56	197254.8	189968.0	185477.8	175731.3	183872.1	177004.2	181570.2	175469.0	194126.1	181772.4	68390.6	68484.2	70876.1	75025.2
O70439	Syntaxin-7	Stx7	261	29.8	5.78	96199.3	84376.5	95680.0	90675.9	94546.6	88422.7	98752.8	96005.5	97014.6	86040.2	259756.2	286294.3	246746.6	290963.6
Q8C5L7	RNA-binding protein 34	Rbm34	442	49.1	10.01	213935.5	225292.2	181056.5	210922.7	196339.6	195127.3	182261.5	182141.2	189147.3	213247.2	102706.6	113747.3	114541.6	95724.0
Q80VQ1	Leucine-rich repeat-containing protein 1	Lrrc1	524	59.4	5.16	32133.9	35757.0	28863.8	34478.7	35748.8	37256.9	40694.0	35432.5	26417.4	38693.4	47233.3	47827.2	38360.9	41969.7
Q99N96	39S ribosomal protein L1, mitochondrial	Mrpl1	336	37.6	8.72	140109.9	165434.0	148478.9	155809.8	161888.6	171628.4	159265.7	158069.0	153435.5	172064.4	144699.7	185115.9	145685.9	150953.9
Q8BQM4	HEAT repeat-containing protein 3	Heatr3	679	74.3	5.01	204782.0	217194.5	205247.1	213985.8	229666.4	239520.0	207006.6	202005.9	209631.6	216252.7	68394.0	73804.6	66717.2	74073.7
Q99MI1	ELKS/Rab6-interacting/CAST family member 1	Erc1	1120	128.3	5.87	118744.3	114971.3	107677.5	105849.9	126015.5	113394.0	110454.7	121376.5	116008.7	116725.0	67210.6	68277.4	69010.3	56862.3
Q8BFU2	Histone H2A type 3	H2aw	130	14.1	11.05	147088.7	151475.4	147580.5	132620.9	176840.1	169419.6	163715.7	147860.5	154667.8	156398.3	204746.0	210431.3	211649.1	216532.4
O55098	Serine/threonine-protein kinase 10	Stk10	966	111.8	7.15	158923.1	173484.1	155251.9	159475.9	176779.8	165697.0	178666.1	165158.5	185913.2	171623.6	271774.3	239211.2	257407.0	227849.4

Q9Z2A9	Glutathione hydrolase 5 proenzyme	Ggt5	573	61.6	8.5	197680.2	171493.7	158312.3	185573.6	219418.2	251003.1	310832.8	196141.5	169585.3	174107.1	211352.4	184048.8	187205.9	188161.1
Q99L43	Phosphatidate cytidylyltransferase 2	Cds2	444	51.3	7.05	384308.4	412041.7	405464.1	360146.0	472703.8	461588.4	439967.6	427804.8	447167.6	392331.5	163478.0	149883.6	149762.9	160354.3
Q9CQG2	RNA N6-adenosine-methyltransferase METTL16	Mettl16	553	62.3	7.36	185506.2	196289.6	182426.5	171739.0	175075.1	179054.4	165955.8	182018.2	189299.6	183781.8	150740.6	145250.0	127935.0	151042.8
Q9JHS9	Spliceosome-associated protein CWC15 homolog	Cwc15	229	26.6	5.71	298207.1	307064.4	307842.2	286744.6	308220.7	299930.4	268788.5	273063.7	291035.8	306472.0	138377.0	139046.1	125306.9	142208.0
Q8BUV3	Gephyrin	Gphn	769	83.2	5.6	107623.9	110859.2	99641.8	102247.2	104151.8	98213.5	95251.2	102441.2	107322.3	102520.4	126804.0	133945.4	140266.5	147876.4
P84096	Rho-related GTP-binding protein RhoG	Rhog	191	21.3	8.12	163486.9	169883.1	141972.3	148628.5	144489.7	168162.7	165546.8	157141.8	151876.5	148148.3	452555.5	435065.8	437714.7	420304.6
O08582	GTP-binding protein 1	Gtpbp1	668	72.3	8.29	187781.2	205181.6	198756.2	206031.7	198465.0	203010.9	186045.0	192150.8	193749.1	203701.1	221096.0	245406.7	237966.4	221633.9
P97760	DNA-directed RNA polymerase II subunit RPB3	Polr2c	275	31.4	4.92	190470.6	209800.1	205873.3	212996.8	187740.3	190088.1	176579.8	176871.3	193827.1	199017.0	60295.4	58883.4	59571.6	65346.0
Q9CRA5	Golgi phosphoprotein 3	Golph3	298	33.7	6.44	70332.7	89555.2	67541.1	78723.1	72683.6	74254.3	64566.9	70545.1	66139.4	74778.3	177340.7	180728.9	169367.9	187401.8
Q8K2Z2	Pre-mRNA-processing factor 39	Prpf39	665	77.9	5.53	293007.7	308870.3	353635.8	318640.6	303730.1	321789.4	276662.7	302918.6	304070.6	309111.4	247226.7	236141.9	250305.7	246817.2
Q8BP27	Swi5-dependent recombination DNA repair protein 1 homolog	Sfr1	319	35.2	5.15	284903.3	268570.8	277741.3	267389.8	297136.8	291318.6	258561.4	256716.6	265735.8	281311.9	745335.9	761498.1	756147.0	684343.9
Q8BPB5	EGF-containing fibulin-like extracellular matrix protein 1	Efemp1	493	54.9	5.14	121835.1	130977.4	122501.3	113559.8	133404.5	151178.4	144281.3	128460.7	120164.2	111050.2	151842.5	152871.4	146112.3	151579.6
Q14C51	Pentatricopeptide repeat domain-containing protein 3, mitochondrial	Ptcd3	685	77.7	5.88	224083.2	219995.9	199105.9	223075.7	222196.9	208174.5	222748.0	205441.8	217243.7	235517.7	131895.3	145064.4	137545.0	143126.5
Q149F3	Eukaryotic peptide chain release factor GTP-binding subunit ERF3B	Gspt2	632	69.1	5.2	157075.4	145358.2	148683.6	140554.1	145675.9	153626.4	147919.1	160204.6	144375.2	158303.0	113689.4	117074.8	113643.2	107971.3
P97820	Mitogen-activated protein kinase kinase kinase kinase 4	Map4k4	1233	140.5	7.47	283930.4	288016.6	261547.5	275486.5	300860.2	299830.2	296892.0	281472.7	287258.2	287226.3	294641.1	305044.9	270896.0	268567.6
Q70IV5	Synemin	Synm	1561	173.1	5.12	222714.7	218854.8	203885.7	200260.6	251639.9	265480.1	285246.1	234546.9	231265.6	226660.1	222118.6	215340.4	217275.1	206677.6
Q5DTI8	Extended synaptotagmin-3	Esyt3	891	100	8.59	90477.9	71825.6	83897.0	68452.4	78762.8	82091.8	89512.5	78095.5	88661.6	96036.1	276774.4	288115.8	283367.9	244777.4
A2ADY9	Protein DDI1 homolog 2	Ddi2	399	44.6	5.05	212989.9	236982.4	223099.4	227111.4	253210.7	254901.8	214293.1	232422.2	229471.7	229544.2	192440.0	204422.0	183943.5	190907.3
P35821	Tyrosine-protein phosphatase non-receptor type 1	Ptpn1	432	49.6	6.16	184200.1	185618.1	189282.0	171682.3	189263.7	187816.6	179515.3	193776.4	199736.8	189802.6	99172.1	110570.3	90384.8	96051.0
P59672	Ankyrin repeat and SAM domain-containing protein 1A	Anks1a	1150	125.2	6.05	256268.8	270788.1	235034.6	265870.5	241783.2	259635.5	234774.6	262869.5	239615.5	265474.8	59799.9	113087.6	58676.8	76359.1
Q8BIQ5	Cleavage stimulation factor subunit 2	Cstf2	580	61.3	6.83	290261.5	342669.1	399814.6	368839.0	354607.4	331460.6	287464.3	302017.2	343718.5	335668.9	339104.8	365658.8	379261.0	372794.2
O88441	Metaxin-2	Mtx2	263	29.7	5.63	266528.0	292176.9	339077.5	332085.3	303287.5	289784.4	256944.9	292595.0	279938.5	289278.3	304662.0	343496.0	366166.9	368624.0
Q9QXK7	Cleavage and polyadenylation specificity factor subunit 3	Cpsf3	684	77.5	5.6	325102.6	365282.9	330506.0	315102.2	352975.8	370132.5	311957.7	317042.5	319035.2	333313.5	539780.0	569913.9	533661.1	531969.1
P00015	Cytochrome c, testis-specific	Cyct	105	11.7	9.51	354977.4	447892.5	536075.2	600738.3	305134.3	261247.3	249420.2	333040.1	423984.5	459253.2	436075.2	566132.6	490454.8	647807.1
A0A0U1RNG7	BTB domain-containing protein	Kctd14	287	33	8.13	316035.2	300049.9	291496.7	267097.8	308083.4	293785.5	286269.6	270367.4	314410.1	314206.4	343839.8	326753.0	369105.3	332231.9
Q99L48	60S ribosomal export protein NMD3	Nmd3	503	57.6	7.17	217878.8	234994.2	215650.5	229786.5	229269.2	231207.2	185120.4	186664.6	209021.7	218672.1	208030.3	230589.8	227979.3	211268.7
P84104	Serine/arginine-rich splicing factor 3	Srsf3	164	19.3	11.65	714739.8	755892.4	684923.0	800863.9	738948.1	767438.5	695369.3	644068.4	726245.3	730359.0	1192697.0	1287371.0	1442808.0	1310873.5
P58854	Gamma-tubulin complex component 3	Tubgcp3	905	103.4	8.32	147849.8	165995.6	164643.0	145234.2	169744.1	158810.9	156040.9	148893.6	147915.0	164343.0	201400.0	191839.0	194379.9	195679.1
Q9D071	MMS19 nucleotide excision repair protein homolog	Mms19	1031	113	6.21	175895.4	181628.3	169565.7	176577.5	173893.3	181884.6	162062.4	174051.4	159692.4	163818.2	240898.9	253299.6	231651.5	250776.4
Q61733	28S ribosomal protein S31, mitochondrial	Mrps31	384	43.9	8.51	366316.6	415275.6	371088.2	398094.1	421581.5	385023.5	368388.2	375342.0	411799.5	428540.7	71801.7	84873.9	79424.7	86862.4
Q9JKV1	Proteasomal ubiquitin receptor ADRM1	Adrm1	407	42	5.07	211293.8	236123.6	211194.5	217132.0	212937.5	199300.7	205470.1	200901.3	215602.9	216624.9	72260.6	72351.3	66491.9	68209.0
Q9D0S9	Histidine triad nucleotide-binding protein 2, mitochondrial	Hint2	163	17.3	9.82	272500.0	273550.0	277970.9	264332.9	278317.9	302980.4	317870.4	272897.3	288363.0	277532.8	279655.5	278986.3	273035.4	254596.4
Q9D517	1-acyl-sn-glycerol-3-phosphate acyltransferase gamma	Agpat3	376	43.3	8.51	397505.7	395376.9	335987.3	379870.5	321498.2	321657.7	351798.9	328981.6	365089.8	356931.7	256532.1	274467.4	234868.9	262952.4
P97370	Sodium/potassium-transporting ATPase subunit beta-3	Atp1b3	278	31.8	8.51	321627.2	283809.6	294683.3	295938.0	295174.9	278059.1	311208.1	281600.1	266099.4	267069.7	528041.5	603249.9	510260.6	693407.5
Q8K1C9	Leucine-rich repeat-containing protein 41	Lrrc41	807	88.1	8.31	42539.7	44481.3	43509.6	41256.4	56775.8	46846.4	43719.6	42729.6	47631.6	49093.2	46870.4	51446.2	48543.3	53476.2
Q3TDQ1	Dolichyl-diphosphooligosaccharide--protein glycosyltransferase subunit STT3B	Stt3b	823	93.2	8.95	903830.6	923120.1	932131.1	915636.4	991725.9	1001705.1	871316.8	883763.7	942429.8	906402.7	869749.3	889827.6	970386.0	945028.2
Q1PSW8	E3 ubiquitin-protein ligase TRIM71	Trim71	855	92	7.53	130956.3	128868.4	127835.0	120963.6	133534.5	129468.4	122346.9	120959.7	128039.9	148753.8	133202.0	133346.9	107359.1	127026.3
Q9CX56	26S proteasome non-ATPase regulatory subunit 8	Psmd8	353	39.9	9.58	702152.2	669832.8	662866.8	695051.6	636739.8	638592.3	644651.0	616636.3	656174.8	686722.0	779187.2	849676.5	878223.1	902006.6
P97452	Ribosome biogenesis protein BOP1	Bop1	732	82.5	6.28	246239.7	246241.5	239234.4	231496.4	254034.5	246784.5	224383.3	210356.9	250867.9	255690.5	106749.6	134676.4	112535.4	106294.0
Q4U2R1	E3 ubiquitin-protein ligase HERC2	Herc2	4836	527.1	6.27	129630.3	135024.5	166971.9	137138.6	154403.4	134852.8	117148.7	127197.7	143118.7	120581.9	272779.5	328219.8	276443.2	329693.1
Q8VHR5	Transcriptional repressor p66-beta	Gatad2b	594	65.4	9.7	146677.8	142257.8	153009.1	128163.9	195526.5	185561.8	181121.1	166266.0	154789.2	154783.0	148926.3	128208.8	140870.2	129490.9
Q8C5Q4	G-rich sequence factor 1	Grsf1	479	53	6.67	102792.8	116878.7	110679.7	106525.7	129997.1	121577.3	112690.9	122765.0	109298.2	127614.3	176296.5	186819.5	175747.9	172974.6
Q8CJ40	Rootletin	Crocc	2009	226.8	5.55	193151.8	188543.7	199903.3	189202.0	221908.4	201426.5	206440.8	182886.7	194649.7	195299.1	268796.5	241549.1	272638.5	229517.1
P97355	Spermine synthase	Sms	366	41.3	5.06	254209.4	266753.7	272546.4	257216.9	270412.1	248478.0	257124.1	253696.0	258370.8	267871.4	224515.1	308979.5	241026.5	253408.7
Q8BJ64	Choline dehydrogenase, mitochondrial	Chdh	596	66.4	8.51	223123.7	201992.4	229030.1	180852.0	269858.5	291230.8	366343.9	252980.2	252779.9	255399.0	207911.2	209158.1	198321.5	194958.7
P18572	Basigin	Bsg	389	42.4	5.85	361397.0	443082.0	390821.9	412699.5	400780.8	416189.8	406958.3	388650.6	441859.2	458640.3	214874.8	264477.3	238254.7	285522.8
Q9ERA6	Tuftelin-interacting protein 11	Tfip11	838	96.2	5.9	73447.6	78660.8	74667.5	79070.3	81424.2	87865.6	80415.0	78870.3	76782.5	80363.0	166979.3	159784.4	175678.1	152165.9
Q9R0Q3	Transmembrane emp24 domain-containing protein 2	Tmed2	201	22.7	5.17	43526.9	43933.8	45721.6	49519.0	49748.4	56421.7	44493.2	50200.1	50410.7	47598.3	73115.4	104894.3	66013.2	79272.8
O35130	Ribosomal RNA small subunit methyltransferase NEP1	Emg1	244	27	8.91	58835.9	71627.7	67523.7	65854.5	63702.0	79199.2	57970.8	62398.9	64475.3	71827.3	103590.0	98934.0	94224.4	97876.8
Q8CBY8	Dynactin subunit 4	Dctn4	467	53	7.72	294983.1	292207.7	258283.2	291635.4	277640.3	287434.0	269999.5	264800.5	265520.1	292467.5	72965.1	82410.7	81338.0	78305.6
Q9WUQ2	Prolactin regulatory element-binding protein	Preb	417	45.4	8.76	183731.3	192607.5	179115.9	187504.0	182607.0	181575.6	187400.0	182661.3	181523.3	199814.9	351201.7	343857.3	328444.8	332472.7
Q8BG30	Negative elongation factor A	Nelfa	530	57.5	9.11	68511.3	88475.5	75691.5	94240.4	77293.8	62018.7	67924.2	61099.6	78793.9	85146.0	132313.7	121207.5	130314.9	126686.9
Q61599	Rho GDP-dissociation inhibitor 2	Arhgdib	200	22.8	5.11	120061.4	122192.0	82217.1	110401.6	103620.3	120399.0	142549.6	116528.6	86330.0	98430.2	287449.6	273021.4	214839.0	247982.8
O70576	Cohesin subunit SA-3	Stag3	1240	141.1	6.29	97156.6	99935.8	96192.0	99724.7	99345.8	97473.9	88073.1	84243.9	100976.7	107543.0	184322.5	200841.6	189403.2	194118.8
A2AF47	Dedicator of cytokinesis protein 11	Dock11	2073	237.6	7.78	77405.8	90316.0	83816.7	88698.3	95399.5	93277.3	92381.0	85030.8	90884.8	94052.3	98820.2	96133.9	102535.1	95426.3
P59017	Bcl-2-like protein 13	Bcl2l13	434	46.7	4.59	122210.3	135630.1	133100.7	129951.0	137723.3	144955.4	122732.1	120875.6	130867.6	135072.6	147686.0	166997.5	142672.9	164667.3
Q3UH60	Disco-interacting protein 2 homolog B	Dip2b	1574	171	8.09	198384.4	175651.1	174827.7	195052.9	178544.2	175304.1	203722.6	196160.5	188480.4	195560.4	155349.4	169497.3	129024.6	144948.9
B2RSH2	Guanine nucleotide-binding protein G(i) subunit alpha-1	Gnai1	354	40.3	5.97	330780.4	401032.5	361677.7	405808.3	331028.5	323092.3	324053.3	317349.9	351667.3	376498.7	91796.1	112230.3	96499.8	87358.9
Q9WUA3	ATP-dependent 6-phosphofructokinase, platelet type	Pfkp	784	85.4	7.11	76011.3	77730.1	60693.4	90731.8	58898.5	61346.0	58705.9	57150.1	73641.2	74775.4	92736.9	121654.6	99106.7	111514.9
P10639	Thioredoxin	Txn	105	11.7	4.92	735498.3	753309.6	714455.8	721975.6	709577.3	753440.0	844384.6	867547.8	745769.7	646756.0	851410.2	770840.0	773304.5	716381.3
Q8CHI8	E1A-binding protein p400	Ep400	3072	337	9.14	85144.2	85718.1	80204.5	79770.3	91259.7	83585.3	79230.7	84500.1	78479.3	92597.4	284640.9	280843.2	277527.4	274827.8
P12815	Programmed cell death protein 6	Pdcd6	191	21.9	5.4	376832.4	384684.7	427876.9	387587.8	439139.8	415988.7	449521.8	431221.6	387728.9	397618.5	1180701.0	1123576.3	1242597.7	1131317.4
A2AKG8	Focadhesin	Focad	1798	198.8	6.74	196585.2	215960.0	195664.9	197343.0	195571.9	207477.2	172147.2	200370.7	196859.1	188489.0	127981.6	144857.4	117854.8	123057.3
Q9QZB7	Actin-related protein 10	Actr10	417	46.2	7.61	69976.4	68849.4	58681.1	63300.2	60061.6	55512.1	57484.5	59617.0	68472.6	64519.6	102859.6	101768.7	96399.6	110811.0
Q8K2A7	Integrator complex subunit 10	Ints10	710	82	7.33	207799.8	227995.4	241591.0	226457.3	250199.9	244675.7	227689.8	216396.1	235590.3	244557.8	293290.6	306999.7	273756.4	287398.9
Q8VEJ9	Vacuolar protein sorting-associated protein 4A	Vps4a	437	48.9	7.8	214083.2	225827.3	207354.3	222788.5	206536.6	203096.9	193339.1	188037.4	231173.8	213663.6	242287.2	284114.6	241281.7	241378.0
P18155	Bifunctional methylenetetrahydrofolate dehydrogenase/cyclohydrolase, mitochondrial	Mthfd2	350	37.8	8.88	526919.1	511076.9	465418.3	478375.7	459258.4	418443.1	411360.9	504273.6	496362.3	504565.3	310308.7	288713.1	272242.8	288016.9
Q9QXE7	F-box-like/WD repeat-containing protein TBL1X	Tbl1x	527	56.8	5.72	302277.1	287607.1	339894.9	310613.2	355189.2	354115.0	343278.9	359426.9	299877.1	313875.2	110848.4	113735.9	129498.8	121413.8
Q3V1V3	ESF1 homolog	Esf1	845	98	5.05	346338.4	369788.7	376172.3	345916.0	396435.7	401362.1	368900.2	342037.1	405777.1	404704.4	154417.0	165431.9	174684.8	155939.2
Q9Z2Z6	Mitochondrial carnitine/acylcarnitine carrier protein	Slc25a20	301	33	9.11	369029.6	388376.9	393312.0	378846.5	395763.1	377183.4	361716.4	352733.3	373116.1	388908.2	336240.4	350475.3	319278.5	349301.8
Q9CQR6	Serine/threonine-protein phosphatase 6 catalytic subunit	Ppp6c	305	35.1	5.69	502396.0	602429.4	610972.7	566318.5	581484.1	552703.5	467943.3	490135.3	562819.5	595238.0	546238.6	558794.7	577160.9	538898.4
P23492	Purine nucleoside phosphorylase	Pnp	289	32.3	6.16	128164.4	140743.8	120891.4	145969.3	127770.7	142588.7	141959.6	138580.2	124122.5	124718.1	194809.3	237359.2	192995.1	232464.3
Q61210	Rho guanine nucleotide exchange factor 1	Arhgef1	920	102.7	5.6	133400.9	140699.3	125170.6	118108.5	131633.8	141222.2	135591.7	140339.2	131027.1	137335.7	147781.1	173650.2	129421.7	158455.0
A0A2R8VHP3	IF rod domain-containing protein	Gm5478	535	57.9	6.2	136947.9	101681.6	99882.8	128871.8	222865.2	255764.2	121510.4	131507.1	136282.4	158695.3	122002.6	100245.3	59203.7	84081.2
P56812	Programmed cell death protein 5	Pdcd5	126	14.3	5.68	1083024.0	1481985.5	1291279.3	1453927.3	1253980.0	1144671.3	1214780.7	1124649.9	1510345.3	1470169.3	589327.8	709185.7	751360.6	746407.3
P31786	Acyl-CoA-binding protein	Dbi	87	10	8.82	138345.4	176403.7	188193.8	156955.4	171704.5	153725.4	153881.4	181272.4	139165.4	148296.5	146899.0	144298.2	173334.1	162614.4
Q9DBE9	pre-rRNA 2'-O-ribose RNA methyltransferase FTSJ3	Ftsj3	838	95.5	8.38	58518.7	67921.4	59621.8	56173.0	64402.5	58031.9	51569.9	57286.1	61340.5	61217.8	10011.7	13725.7	12795.0	12965.3
Q8BMS9	Ras association domain-containing protein 2	Rassf2	326	37.9	7.94	174123.1	151106.5	181590.3	163968.6	186787.6	183000.4	148356.3	190232.6	165236.2	162736.0	198150.4	204236.1	182202.1	181354.5
Q04997	Inhibin alpha chain	Inha	366	39.5	8.13	296912.9	303410.7	257640.7	291050.1	209249.7	189327.6	223899.5	286576.6	264342.4	242229.3	172900.0	165578.7	164569.5	152703.5
E9PZP8	HECT and RLD domain-containing E3 ubiquitin protein ligase family member 1	Herc1	4859	532.3	6.04	96604.3	97256.0	90289.5	91889.5	92937.7	95920.6	83897.0	87613.8	91499.1	98135.0	79070.0	69905.8	66013.5	73103.1
Q62189	U1 small nuclear ribonucleoprotein A	Snrpa	287	31.8	9.8	743679.9	835386.7	801968.0	794122.8	877775.7	844767.3	815742.7	735169.6	807021.8	850008.9	940143.9	956927.3	1154927.9	979622.1
Q9WUM3	Coronin-1B	Coro1b	484	53.9	5.78	698943.9	725051.9	669800.1	681798.4	606085.0	639377.3	554378.7	647159.8	618629.6	647461.4	514159.2	488748.2	486411.4	489435.1
Q9CWN7	CCR4-NOT transcription complex subunit 11	Cnot11	505	54.9	6.55	102537.0	146042.2	122028.8	136477.4	137167.9	122382.2	105163.0	119073.0	118191.5	125494.7	68931.8	80794.9	62181.6	70262.6
Q9CWZ3	RNA-binding protein 8A	Rbm8a	174	19.9	5.72	89613.5	76600.0	81494.4	85092.0	87487.6	91256.7	89885.9	73932.3	92361.7	91279.3	202754.8	199092.1	178067.0	195719.9
Q8R080	G2 and S phase-expressed protein 1	Gtse1	741	78.7	9.39	78060.2	89424.1	82716.8	93436.6	89630.4	81406.7	72451.9	76624.6	95719.3	89835.7	65052.3	59463.0	43958.4	47716.4
Q8BFS6	Serine/threonine-protein phosphatase CPPED1	Cpped1	312	35.2	5.34	281861.0	257218.8	254099.8	259558.9	339624.4	330532.9	376279.6	288300.5	303800.4	273550.7	176617.9	174226.3	169025.6	171848.9
Q6VSS7	Homeobox domain-containing protein	Rhox8	320	35.9	3.94	1221475.4	1021048.3	1183715.6	918999.4	1549946.9	1209316.1	1412954.3	1216589.4	1444866.5	1435732.5	774648.4	694083.2	763737.2	628061.1
Q80U49	Centrosomal protein of 170 kDa protein B	Cep170b	1574	170.7	6.87	131935.6	160381.7	135011.8	137051.0	131590.4	132568.9	131378.6	122094.2	144659.8	135846.5	188198.4	209533.5	187119.6	211297.7
A3KGS3	Ral GTPase-activating protein subunit alpha-2	Ralgapa2	1872	210.2	6.21	117414.0	118228.3	111915.3	126324.6	123489.7	126190.6	94252.2	115399.1	113460.0	112276.9	90955.3	101044.0	78550.8	82066.1
O08915	AH receptor-interacting protein	Aip	330	37.6	6.4	105472.7	111676.8	105175.1	105180.0	119779.5	116416.5	105748.2	106793.9	107454.6	120561.4	114907.2	119782.1	127178.4	126246.5
Q3TDD9	Protein phosphatase 1 regulatory subunit 21	Ppp1r21	780	88.3	6.9	244371.5	248517.2	242624.8	227921.8	269549.1	257803.9	241366.1	251560.9	237089.4	239871.4	141947.0	142724.2	135596.3	136293.6
Q8K4Q0	Regulatory-associated protein of mTOR	Rptor	1335	149.4	6.87	123551.1	124827.7	119166.8	125984.9	126052.6	137965.5	127650.4	119278.4	127808.0	123191.5	131622.3	150174.0	126406.6	139360.4

E9PYH6	Histone-lysine N-methyltransferase SETD1A	Setd1a	1716	185.9	5.06	40140.2	50113.6	63465.0	44950.7	58664.3	59239.9	52659.4	45712.4	60148.9	52695.6	119332.1	109862.2	105666.9	109916.3
P30115	Glutathione S-transferase A3	Gsta3	221	25.3	8.73	825458.8	719429.5	810222.2	717949.4	880124.4	817544.3	1070121.8	875280.7	819678.4	812742.8	815641.6	863147.1	880827.0	911389.5
Q60649	Caseinolytic peptidase B protein homolog	Clpb	677	76	8.51	305437.5	294295.6	297832.2	316771.1	296606.2	293027.7	336334.1	298833.3	261306.1	269107.5	252423.6	244930.1	249398.3	252927.6
Q80WC7	Arf-GAP domain and FG repeat-containing protein 2	Agfg2	479	48.9	9.1	341078.9	354967.4	315305.0	336402.6	354530.2	311908.7	329167.5	320013.3	335409.3	354260.2	152747.1	142732.5	147550.3	146742.9
Q8CI33	CWF19-like protein 1	Cwf19l1	537	60.2	7.14	251632.2	258407.8	265804.1	255522.6	303640.0	337480.2	311650.8	237396.0	296260.8	305051.4	165808.3	161217.6	163879.9	158199.4
Q6NWW9	Fibronectin type III domain-containing protein 3B	Fndc3b	1207	132.7	6.29	101208.7	97234.4	89182.4	89820.2	91175.5	83878.1	85237.2	106932.1	97381.3	85840.0	36865.7	40552.1	38465.3	43119.8
Q6PHZ2	Calcium/calmodulin-dependent protein kinase type II subunit delta	Camk2d	499	56.3	7.25	248418.1	227472.0	194700.1	223165.3	217169.3	241647.0	279197.0	219434.0	216466.0	234045.9	48393.0	47262.1	48017.3	42847.2
Q6PGH1	Protein BUD31 homolog	Bud31	144	17	8.82	114712.1	140610.0	132374.3	115948.7	129698.0	134204.8	112122.1	167618.1	119466.9	126110.5	116765.4	116959.8	104273.7	97142.3
Q8K409	DNA polymerase beta	Polb	335	38.3	8.85	202895.4	211856.7	201862.2	205004.8	185387.9	203062.5	181454.5	175626.6	201964.8	209725.1	157372.2	163333.9	158216.7	172745.4
A2AFI6	Transmembrane 9 superfamily member	Gm364	671	77.2	6.48	511591.8	607015.3	673367.8	542963.2	725563.1	714309.5	565875.7	580346.1	677075.1	683761.5	392579.2	447872.4	571296.7	430156.4
P41105	60S ribosomal protein L28	Rpl28	137	15.7	12.02	140083.7	152349.8	147298.1	147863.3	141754.5	138542.3	126353.8	141220.4	142011.0	146335.5	806972.8	719707.6	802395.2	749196.5
Q91ZV0	Melanoma inhibitory activity protein 2	Mia2	1396	156.4	4.55	89182.8	113405.5	103925.9	112666.6	117754.6	109761.0	102355.9	113231.0	104221.1	105288.8	110591.0	115639.2	109132.3	117635.1
Q9ET30	Transmembrane 9 superfamily member 3	Tm9sf3	587	67.5	7.21	379549.7	378372.8	373921.7	391218.1	365597.7	358685.5	302048.5	355000.6	353176.5	363905.9	280005.0	288362.4	286081.3	296393.7
Q8CHH9	Septin-8	Septin-8	429	49.8	6	116192.0	132028.7	114898.8	110866.6	127195.5	133556.1	130425.7	113872.1	115009.1	112073.3	375418.8	344020.2	317320.7	342266.6
A2AKQ8	Tetratricopeptide repeat protein 30A2	Ttc30a2	664	76.1	5.16	31642.4	33973.1	35702.9	38091.3	27458.1	34425.3	30151.4	31419.5	36676.0	31218.6	#N/A	#N/A	#N/A	#N/A
Q8BYL4	Tyrosine--tRNA ligase, mitochondrial	Yars2	472	52.6	9.16	176760.1	190041.1	171079.7	170069.9	183535.3	180366.8	152188.6	168030.0	175139.3	170186.5	154753.0	163115.7	166881.3	164506.7
Q8BT60	Copine-3	Cpne3	533	59.5	5.78	479198.6	474404.3	488865.6	495269.8	518957.1	552916.0	477562.2	473177.8	504208.4	501485.5	751651.0	749977.9	788666.1	753303.5
Q8BH97	Reticulocalbin-3	Rcn3	328	38	4.88	281627.3	366535.7	195494.8	274605.3	279832.0	317971.5	228862.8	237322.2	263867.2	294468.2	179581.8	173590.1	147541.7	175835.6
Q9JIQ3	Diablo homolog, mitochondrial	Diablo	237	26.8	6.37	280712.7	345796.2	364366.4	420564.9	290146.8	285155.1	272934.7	279961.8	343070.9	352647.3	164224.5	192813.4	192933.2	226150.6
Q9EST5	Acidic leucine-rich nuclear phosphoprotein 32 family member B	Anp32b	272	31.1	4.01	906239.7	932024.8	979433.7	951149.0	1155341.8	1026796.8	964756.4	955327.3	946992.1	966587.0	672207.2	694809.6	683328.5	701974.6
Q99JX3	Golgi reassembly-stacking protein 2	Gorasp2	451	47	4.79	282875.9	302635.5	268546.2	322354.8	252677.6	252915.8	242791.3	227766.1	297886.7	287116.8	638642.2	592750.4	622000.5	604580.9
Q9D0J4	ADP-ribosylation factor-like protein 2	Arl2	184	20.9	5.96	103609.0	112704.0	95814.0	98133.4	98664.6	102033.4	95713.3	89885.2	104157.3	110245.0	133620.1	145555.8	126557.0	130262.4
P81117	Nucleobindin-2	Nucb2	420	50.3	5.15	379421.0	379159.0	329151.0	428651.0	303280.9	302745.5	310605.9	373153.4	286207.8	290036.5	382230.7	410834.5	334185.2	440026.6
P97290	Plasma protease C1 inhibitor	Serping1	504	55.5	6.29	225875.3	224529.8	176329.6	216090.3	201647.7	226871.0	197834.2	222059.3	221258.5	215504.1	263904.6	242045.0	235990.4	216228.5
Q80V26	Golgi-resident adenosine 3',5'-bisphosphate 3'-phosphatase	Impad1	356	38.6	6.47	290923.4	318916.9	261002.0	263473.9	272759.2	267465.6	260911.4	262104.8	259519.5	269628.2	108817.1	127161.6	115633.3	125706.1
Q80TN4	DnaJ homolog subfamily C member 16	Dnajc16	772	89.1	7.55	125812.4	123714.1	122260.6	123004.2	121817.9	116705.4	109010.3	122751.5	126406.7	122210.6	111478.0	112826.4	102366.4	117926.9
Q8BK63	Casein kinase I isoform alpha	Csnk1a1	337	38.9	9.57	291569.3	300820.1	298959.7	303923.3	302222.5	293434.4	261720.6	262670.8	306658.7	287777.2	185626.9	187148.1	172590.2	193516.5
Q3UMB9	WASH complex subunit 4	Washc4	1173	136.3	7.37	108134.7	98427.8	100308.4	99424.5	106623.2	120110.3	102348.7	102563.7	102578.2	96601.1	173124.3	175785.1	169329.6	174321.2
Q8R2Y8	Peptidyl-tRNA hydrolase 2, mitochondrial	Ptrh2	181	19.5	7.42	153690.6	145100.0	156597.5	158320.9	166329.7	166398.1	165697.5	149493.6	149958.1	156336.5	247895.6	208678.9	260040.3	247433.5
P52503	NADH dehydrogenase [ubiquinone] iron-sulfur protein 6, mitochondrial	Ndufs6	116	13	8.65	211892.7	192219.7	197885.7	200219.9	186049.2	192178.2	175422.3	199474.3	188470.1	191223.8	251426.5	195359.0	217605.8	214018.0
Q8C166	Copine-1	Cpne1	536	58.8	5.66	271530.7	242686.2	303743.6	227207.3	330054.6	260271.3	283901.2	259980.9	272585.4	291482.6	248058.5	289169.0	295745.2	273148.6
Q69ZF3	Non-lysosomal glucosylceramidase	Gba2	918	103.2	5.59	133770.8	133068.7	137014.3	141773.2	135045.9	133101.8	122562.8	139643.6	138697.2	134464.5	101523.8	104100.5	101482.5	100394.3
Q8CD10	Calcium uptake protein 2, mitochondrial	Micu2	432	49.4	9.45	151783.7	167507.8	146099.2	162544.9	165524.3	155895.8	176318.4	159821.9	150234.8	166416.0	353324.1	318848.0	337010.4	345669.8
Q91YJ2	Sorting nexin-4	Snx4	450	51.7	5.8	157359.8	147731.9	135503.4	149198.5	145588.7	137848.4	130230.9	141117.9	149274.4	153484.2	106465.2	114723.9	108872.1	115036.8
Q9Z2G9	Oxidoreductase HTATIP2	Htatip2	242	26.9	8.59	122605.4	132529.8	139919.5	151141.7	133488.4	126759.3	114964.8	111074.6	132719.1	135385.9	352235.4	296803.6	370051.8	446285.0
Q9CZX0	Elongator complex protein 3	Elp3	547	62.3	8.9	424800.7	447123.2	439197.3	416409.0	447001.1	442114.3	409934.0	434318.3	436246.4	436714.7	366609.5	385121.4	375240.1	363818.2
P41241	Tyrosine-protein kinase CSK	Csk	450	50.7	7.06	297597.1	321881.0	274628.0	280015.5	297152.3	316932.1	266198.9	280785.0	303730.1	282408.1	111952.5	113791.7	100923.1	111303.7
Q80TT8	Cullin-9	Cul9	1865	209	5.63	77359.7	102304.8	77324.8	94882.8	75513.8	71506.4	61907.3	67383.5	84206.9	91671.2	74019.7	88337.0	81010.7	89595.4
Q922Q4	Pyrroline-5-carboxylate reductase 2	Pycr2	320	33.6	7.77	534657.5	546495.8	466115.6	557870.7	558228.6	551899.6	553241.9	531598.4	549786.4	541537.0	221082.1	220379.4	205989.1	241979.2
Q8VED5	Keratin, type II cytoskeletal 79	Krt79	531	57.5	7.69	772665.8	914374.0	893150.6	944695.1	2031512.2	1500498.2	838881.6	1020521.0	1056914.9	996168.9	137996.1	143233.1	143372.6	142023.1
Q9CRD2	ER membrane protein complex subunit 2	Emc2	297	34.9	6.81	165758.5	196290.1	181559.6	159093.3	190212.4	210802.7	163498.8	193845.0	191788.7	175479.6	156424.0	155038.7	160375.1	144084.0
A0A0U1RNF2	Leucine carboxyl methyltransferase 1	Lcmt1	332	38.1	5.74	533841.9	507217.7	478373.0	477139.0	501393.0	460490.8	466004.4	424383.3	482976.1	517799.7	161530.4	204530.0	174423.4	166255.9
O08738	Caspase-6	Casp6	276	31.6	6.89	308103.1	274266.9	271563.4	263793.1	309713.6	300998.0	305096.6	288775.1	273865.6	283799.5	445432.8	440496.5	401718.0	427476.3
Q8CC70	Cilia- and flagella-associated protein 300	Cfap300	267	30.9	6.92	117660.0	178556.2	144406.0	205664.9	98057.3	95289.4	100396.2	107327.0	119506.1	130630.0	194446.8	237762.0	245491.0	267359.7
B2RPU8	SCAN domain containing 3	Zbed5	756	84.2	8.44	189626.7	235953.2	202722.2	215066.6	219202.8	239016.2	212190.3	214802.4	201402.1	215827.5	464737.8	442388.8	513867.1	391763.7
Q9EQ08	Heparan N-sulfatase	Sgsh	502	56.7	6.37	273794.7	293454.0	273748.5	273945.8	302644.1	316095.3	329038.2	276131.0	288688.4	281932.7	258561.7	237729.4	259405.3	243920.7
Q60759	Glutaryl-CoA dehydrogenase, mitochondrial	Gcdh	438	48.6	8.73	869942.5	870144.5	827489.6	820720.6	884875.7	846391.5	903240.6	853807.6	895115.1	892464.6	627154.5	614492.9	621085.9	601212.7
Q8R3F5	Malonyl-CoA-acyl carrier protein transacylase, mitochondrial	Mcat	381	41.9	8.1	96459.6	133016.9	89040.5	104985.0	130480.8	113244.0	106176.0	100654.2	103287.2	111307.4	77627.1	66413.1	80733.6	77614.7
Q9JHK4	Geranylgeranyl transferase type-2 subunit alpha	Rabggta	567	64.9	5.8	428224.2	422569.2	380167.1	407548.8	400632.1	423674.3	364941.4	366469.6	396909.2	422025.8	229530.9	235680.7	212567.2	222623.7
Q8WTY4	Anamorsin	Ciapin1	309	33.4	5.2	452646.4	492455.7	528298.4	568873.0	439492.3	463637.3	381270.6	425177.4	491202.4	483985.6	201632.4	225042.7	228721.7	221856.9
Q8CGU1	Calcium-binding and coiled-coil domain-containing protein 1	Calcoco1	691	77.2	4.82	141558.1	158164.4	160569.2	177030.2	150239.8	141838.2	129384.6	126061.9	147069.0	146761.1	181811.5	183037.7	182405.7	165735.7
Q9CQA9	Cancer-related nucleoside-triphosphatase homolog	Ntpcr	190	20.7	9.72	145381.9	169944.3	148915.0	171504.7	152989.4	147497.1	129956.9	139020.1	141711.4	148528.9	49498.5	74784.5	65364.1	74638.0
Q9CWY8	Ribonuclease H2 subunit A	Rnaseh2a	301	33.5	4.98	85118.1	85703.1	84219.5	75159.3	91145.1	81701.8	81124.7	87756.7	91741.9	87430.0	382575.4	386841.6	382709.9	365123.6
Q80ZJ1	Ras-related protein Rap-2a	Rap2a	183	20.6	4.82	110945.4	102070.1	106997.8	100785.4	110849.8	122913.3	111121.8	97493.5	105257.4	101181.4	240282.0	229119.7	247072.1	220739.8
Q8CFE2	Histone PARylation factor 1	Hpf1	346	39.3	7.69	249033.3	230502.1	249508.9	246913.9	259039.8	250193.4	230072.9	251310.4	243161.4	241236.5	149484.4	153061.9	148497.2	158490.8
Q8CI59	Metalloreductase STEAP3	Steap3	488	54.7	9.22	320711.1	291950.4	305644.8	274966.0	381322.8	432165.3	395797.5	296515.7	307356.9	297785.0	272231.2	258897.1	239460.5	267202.7
A2AR02	Peptidyl-prolyl cis-trans isomerase G	Ppig	752	88.3	10.27	325938.3	341882.4	336682.1	354217.3	350626.1	347080.8	332944.3	313209.5	347133.3	352566.4	322363.2	321481.6	325131.6	328827.3
Q9Z0R9	Acyl-CoA 6-desaturase	Fads2	444	52.4	8.82	1440620.0	1084024.3	1142927.8	1267952.8	706779.3	682137.6	758755.9	1206634.2	949908.8	877875.1	677958.4	561594.7	531065.3	636787.0
Q9Z2D8	Methyl-CpG-binding domain protein 3	Mbd3	285	32.1	5.82	111254.0	138199.4	142195.0	128610.1	145710.8	153364.4	127357.5	136869.9	132192.5	128490.2	100530.5	109927.5	101289.7	102755.5
Q8BP67	60S ribosomal protein L24	Rpl24	157	17.8	11.25	1314079.3	1297592.6	1378065.3	1273025.4	1148452.5	1114859.7	900623.5	1170941.2	1088095.2	1135889.0	1808228.1	1976303.1	1965501.9	2022616.2
Q91ZR1	Ras-related protein Rab-4B	Rab4b	213	23.6	6.06	303786.7	309535.3	264223.2	297438.1	253521.1	254398.8	251127.8	256476.5	290801.1	289395.6	256096.8	261463.0	207994.0	250958.3
Q80Y17	Lethal(2) giant larvae protein homolog 1	Llgl1	1036	112.5	6.48	71061.2	124948.2	68507.4	80315.2	102503.5	137612.8	85368.4	129522.1	83660.5	99756.2	94086.3	102687.0	87017.4	94414.8
Q6P9R4	Rho guanine nucleotide exchange factor 18	Arhgef18	1405	155.9	6.54	207223.2	232594.9	179188.2	221671.4	205205.6	211534.7	239856.1	167642.5	246036.0	232444.8	184129.7	209640.4	197184.3	191965.4
Q8R1F1	Protein Niban 2	Niban2	749	84.8	5.94	150664.5	132945.9	150239.3	128193.0	157184.8	174855.2	157566.7	162055.7	136087.1	141173.2	543112.0	530371.3	520366.5	490201.4
Q924Z6	Exportin-6	Xpo6	1125	128.6	6.37	81522.6	107391.8	87605.0	103968.9	96858.6	106917.0	93556.9	88109.7	87937.0	94785.4	129536.8	142705.3	128979.1	151401.3
Q9D855	Cytochrome b-c1 complex subunit 7	Uqcrb	111	13.5	9.11	337060.3	337717.9	437541.2	387829.4	353803.1	330306.2	315283.3	366750.9	390292.2	356213.5	243991.9	252428.6	276722.0	260536.9
Q9R1P3	Proteasome subunit beta type-2	Psmb2	201	22.9	7.02	892699.0	882763.1	961374.3	939023.6	887280.3	882630.3	798978.9	883098.8	927927.5	891330.5	966494.5	1090273.5	1030389.4	1017110.8
Q8BWR8	Rhophilin-2	Rhpn2	686	76.9	6.8	300768.4	373498.9	308121.9	318244.8	348788.2	347644.9	290286.7	328980.3	315657.3	356250.7	219177.4	201933.1	192425.0	200753.7
O88485	Cytoplasmic dynein 1 intermediate chain 1	Dync1i1	628	70.7	5.12	168795.1	162391.4	154805.7	165921.6	145030.5	130815.5	144762.6	123470.4	141586.8	151935.0	158103.0	196179.0	140790.3	174449.8
P97379	Ras GTPase-activating protein-binding protein 2	G3bp2	482	54.1	5.62	302452.4	337147.1	303006.0	317533.4	310094.6	316114.7	325230.9	289985.5	309962.0	296681.1	534176.2	527068.8	493557.2	527828.6
P61971	Nuclear transport factor 2	Nutf2	127	14.5	5.38	627403.4	649131.2	592639.4	598919.5	620391.0	607623.6	566424.1	583223.6	640045.7	648936.4	289440.0	326865.5	279697.2	333788.3
Q9QZA0	Carbonic anhydrase 5B, mitochondrial	Ca5b	317	36.6	7.31	536566.9	521335.4	576282.3	504561.9	597801.2	520026.0	558655.0	547113.2	540885.7	578465.2	277669.2	280477.6	291842.3	286886.4
Q8VD66	(Lyso)-N-acylphosphatidylethanolamine lipase	Abhd4	342	38.8	7.59	608356.2	505178.7	594831.1	606286.7	377393.1	329205.8	315497.7	403102.7	612196.3	525122.6	268668.4	231568.0	217613.3	217590.7
Q3ULJ0	Glycerol-3-phosphate dehydrogenase 1-like protein	Gpd1l	351	38.2	6.77	467504.8	452008.4	374444.1	482646.2	426053.5	451747.8	572751.9	407093.3	425168.4	462005.8	498268.0	392056.1	391860.3	444841.1
O88545	COP9 signalosome complex subunit 6	Cops6	324	35.9	5.73	263257.3	280025.1	293619.3	288305.0	302069.3	286093.7	277089.3	275170.4	297559.9	293455.6	461785.9	500266.0	452343.1	499901.6
Q9WV91	Prostaglandin F2 receptor negative regulator	Ptgfrn	879	98.7	6.61	152035.7	150610.1	162012.4	151414.6	170651.8	222384.1	202449.6	173437.7	150032.4	150865.9	280366.9	286489.9	291362.0	274000.1
Q9JMG7	Hepatoma-derived growth factor-related protein 3	Hdgfl3	202	22.4	8.4	471481.8	501466.9	488672.4	454893.1	521912.8	589674.5	660944.7	460794.2	520561.1	561397.6	482972.4	558508.7	446586.4	472283.7
Q91WM3	U3 small nucleolar RNA-interacting protein 2	Rrp9	475	52.1	7.88	151891.6	128982.2	144756.1	127263.3	147732.0	148957.5	139728.4	129803.2	152418.3	144909.5	245806.2	241394.6	224840.7	238753.9
Q8CI08	SLAIN motif-containing protein 2	Slain2	581	62.3	9.47	37280.3	43094.2	47401.5	44308.9	36178.4	37452.3	36153.0	45930.8	39742.8	43249.7	135089.3	114704.7	125228.5	130183.2
O89001	Carboxypeptidase D	Cpd	1377	152.3	6.18	121003.2	114436.0	111032.5	116060.7	127692.1	130003.3	123406.2	116723.9	102361.8	105509.8	255260.4	241292.3	243292.3	276098.9
P61358	60S ribosomal protein L27	Rpl27	136	15.8	10.56	1574292.8	1540505.6	1565849.3	1556207.9	1616978.8	1572204.4	1385252.9	1617727.6	1614055.4	1664350.8	1360026.4	1570267.6	1298238.9	1358940.5
O08917	Flotillin-1	Flot1	428	47.5	7.15	130025.7	151838.4	109894.1	135929.1	122168.7	111385.6	106224.5	114611.7	128101.9	138554.7	123141.1	131718.3	111777.5	111169.0
Q8R307	Vacuolar protein sorting-associated protein 18 homolog	Vps18	973	110.1	6.09	538775.9	531535.8	529332.7	509742.6	533785.5	522782.1	482804.6	554173.6	518238.2	529637.9	39885.0	44222.5	41731.6	41504.5
Q5NCJ1	Rap guanine nucleotide exchange factor (GEF) 6	Rapgef6	1609	180.1	6.47	160567.0	146700.6	141495.1	139926.0	140446.1	140360.4	154497.3	135426.9	143220.4	159290.2	223440.4	245836.9	223101.2	247844.8
Q3TC72	Fumarylacetoacetate hydrolase domain-containing protein 2A	Fahd2	313	34.7	8.16	96786.9	106810.0	108954.8	105659.4	102869.7	100925.0	90795.5	94091.4	95112.0	96979.4	203370.2	268150.9	218054.8	272163.3
P01837	Immunoglobulin kappa constant	Igkc	107	11.9	5.9	172270.6	137053.4	151318.4	163533.1	176201.3	133984.9	168583.1	172966.6	148871.9	167861.5	56597.4	42987.9	49017.0	51402.3
P35285	Ras-related protein Rab-22A	Rab22a	194	21.8	8.15	115921.8	112934.3	107764.3	120865.0	120492.1	113662.0	101566.5	113078.4	133503.5	131182.4	32911.9	32756.6	32784.3	32223.5
Q8CCP0	Nuclear export mediator factor Nemf	Nemf	1064	121.1	6.8	151466.9	156929.2	125873.7	143183.1	134517.8	129609.1	128131.2	124741.7	130277.8	143595.6	124896.5	158882.3	131915.2	148742.0
Q9Z1S0	Mitotic checkpoint serine/threonine-protein kinase BUB1 beta	Bub1b	1052	118.3	5.43	153483.4	165051.3	190566.8	173029.9	159720.6	152072.9	143985.8	152751.9	159505.9	135853.6	175689.0	183483.6	175008.8	181716.8
Q8K327	Chromosome alignment-maintaining phosphoprotein 1	Champ1	802	87.5	7.85	128829.1	129009.3	117993.1	121378.0	130214.5	122659.1	128015.9	119652.7	125481.1	133871.8	162251.6	159739.9	151330.5	142351.4
Q8CH72	E3 ubiquitin-protein ligase TRIM32	Trim32	655	72	6.9	62151.8	68217.4	55785.1	61270.5	58804.8	54653.1	53158.7	54196.4	60519.8	64330.5	126970.7	151895.9	125011.8	150648.7
Q9EPB4	Apoptosis-associated speck-like protein containing a CARD	Pycard	193	21.4	5.43	227978.2	229604.4	210150.2	235873.1	226962.4	239284.2	263634.0	266229.6	238459.1	213033.8	266622.8	226337.9	249013.2	246869.1

O35598	Disintegrin and metalloproteinase domain-containing protein 10	Adam10	749	83.9	7.94	159427.9	159974.5	134927.3	131509.1	151765.0	156516.0	141215.3	150152.1	153671.6	153298.3	57567.1	45500.0	56524.6	47711.0
P01901	H-2 class I histocompatibility antigen, K-B alpha chain	H2-K1	369	41.3	6.39	64994.6	86171.8	81847.3	82032.0	84078.3	78143.8	69798.9	77945.6	76384.7	73886.5	74146.9	69939.9	62399.8	75447.2
P31428	Dipeptidase 1	Dpep1	410	45.7	6.37	234951.6	240011.4	215437.5	235656.1	236607.0	267699.5	272701.6	209341.8	230890.2	212705.0	280983.2	276973.7	237365.2	259642.7
Q08288	Cell growth-regulating nucleolar protein	Lyar	388	43.7	9.5	146061.3	230794.6	207375.5	283377.8	161189.7	139881.8	109067.7	134407.4	188751.0	206710.2	63189.5	66725.2	80382.8	104890.4
Q8BHL5	Engulfment and cell motility protein 2	Elmo2	732	83.8	5.95	92227.2	94169.9	91288.2	100305.9	95218.1	110620.9	88956.2	87439.1	90040.0	81479.5	86178.5	86711.2	90749.3	84702.5
P01867	Ig gamma-2B chain C region	Igh-3	404	44.2	6.52	254443.4	273097.4	248032.8	249495.1	272108.8	238389.7	244085.8	235407.6	252760.5	279313.2	288443.8	268812.8	272795.5	243763.7
Q923E4	NAD-dependent protein deacetylase sirtuin-1	Sirt1	737	80.3	4.73	265132.2	268006.8	280936.8	294100.0	280779.4	278058.2	243476.6	238464.2	290898.6	280848.4	102081.5	111838.3	124110.3	118414.3
O54962	Barrier-to-autointegration factor	Banf1	89	10.1	6.09	350773.5	411935.5	421405.2	400972.8	449355.8	409810.1	320071.4	458987.7	423926.1	375488.7	281941.0	434486.3	366512.5	432061.5
P00375	Dihydrofolate reductase	Dhfr	187	21.6	8.53	397902.4	398901.7	364681.2	377746.3	406288.2	386012.8	378110.4	365472.6	409637.7	431399.8	760770.0	756526.2	769770.0	721994.4
Q6PHQ8	N-alpha-acetyltransferase 35, NatC auxiliary subunit	Naa35	725	83.3	7.3	372481.5	408524.8	357663.2	386663.5	393525.5	375636.1	357082.1	346850.0	389388.7	387019.8	216562.7	238594.7	213254.1	245427.9
O70152	Dolichol-phosphate mannosyltransferase subunit 1	Dpm1	260	29.2	9.51	186532.2	195212.3	191656.8	194401.4	186146.6	194069.3	187232.5	191693.7	190365.0	199434.4	263729.3	283554.9	286112.6	270177.9
Q64523	Histone H2A type 2-C	H2ac20	129	14	10.9	714734.6	688957.2	911663.3	673340.1	954550.1	1004563.0	1600279.0	735110.7	919652.1	895463.2	5718030.6	4358815.6	7670310.5	4584503.8
Q8BZR9	Nuclear cap-binding protein subunit 3	Ncbp3	615	70	5.8	88804.0	97424.3	101746.9	91288.2	111097.2	106674.3	95636.9	91927.6	98648.8	102311.2	217420.4	205704.0	211317.0	216990.9
O70492	Sorting nexin-3	Snx3	162	18.7	8.66	1095390.9	1033255.9	973431.6	1038529.2	1030155.1	1011333.2	969207.1	966283.9	1014475.4	1041608.5	793708.7	723162.1	605862.5	702875.4
Q8BMC4	Nucleolar protein 9	Nop9	636	70	7.27	129991.3	155537.8	139186.4	156496.6	141186.7	144936.2	124168.2	137117.8	149134.9	152695.0	151914.9	150162.8	163549.7	157499.8
Q8VD46	Ankyrin repeat, SAM and basic leucine zipper domain-containing protein 1	Asz1	475	53	6.24	567458.4	663665.6	544908.7	711312.8	516955.1	476625.1	456022.3	466758.0	579694.1	641589.1	205326.8	258420.1	229516.1	269168.1
P35761	Dual specificity protein kinase TTK	Ttk	856	96.2	8.19	92423.0	115614.5	109169.9	112129.3	96210.2	117043.3	77294.9	85837.8	128084.1	119724.4	71866.0	89476.2	80024.1	86519.6
P08730	Keratin, type I cytoskeletal 13	Krt13	437	47.7	4.86	269301.5	32595.6	37218.9	33805.8	39316.6	43715.5	41182.2	28130.9	34051.7	35698.6	8787.2	10549.9	6484.3	9550.4
P67984	60S ribosomal protein L22	Rpl22	128	14.8	9.19	1194581.6	1268247.1	1163648.8	1368527.6	1082268.0	1040257.6	1131828.4	1026129.8	1361042.2	1320045.8	1059727.7	1218825.3	1093354.8	1212259.3
Q91Y86	Mitogen-activated protein kinase 8	Mapk8	384	44.2	7.69	169483.6	187361.5	173321.6	171452.5	203682.6	201907.3	175778.2	171731.2	183540.0	197032.7	93031.5	103504.6	87199.9	89355.5
Q6P6L6	Gem (Nuclear organelle) associated protein 4	Gemin4	1058	120.1	5.15	244941.9	265817.5	262276.6	245882.8	268778.1	248272.2	220167.3	227750.7	249960.8	272473.3	478603.3	453888.6	487203.0	449038.1
Q5PRF0	HEAT repeat-containing protein 5A	Heatr5a	2038	219.8	6.76	92397.8	120741.7	122481.6	144177.5	87723.0	90781.1	81224.3	83277.4	104414.6	103664.1	39721.4	36098.6	27875.8	36233.7
P62245	40S ribosomal protein S15a	Rps15a	130	14.8	10.13	1813554.6	1800170.7	1862837.4	1813921.9	1486702.8	1657167.9	1435427.8	1856268.7	1572084.2	1782297.3	1518874.7	1423934.7	1608775.1	1330090.1
P51125	Calpastatin	Cast	788	84.9	5.52	216282.4	218974.2	179825.9	216504.6	201975.9	210798.3	195469.0	197670.4	231531.8	211041.7	66611.8	53809.2	73437.4	61178.7
Q9Z0H3	SWI/SNF-related matrix-associated actin-dependent regulator of chromatin subfamily B member 1	Smarcb1	385	44.1	6.23	74507.7	69833.8	68656.0	69301.9	72153.0	71756.0	72736.2	72854.4	72047.0	70248.0	88940.9	82978.6	82367.6	82091.8
Q9EQC5	N-terminal kinase-like protein	Scyl1	806	89.1	6.44	196215.4	219288.2	205047.6	212677.7	204178.6	199764.6	196191.4	190221.7	192266.4	216055.7	184694.8	176649.8	172842.2	172987.1
Q91WE2	PSME3-interacting protein	Psme3ip1	254	28.7	5.14	346022.4	385188.7	367423.5	369697.5	372133.7	362674.2	344020.0	318203.1	358200.2	391001.0	399433.9	453381.5	453521.7	441436.0
O88447	Kinesin light chain 1	Klc1	541	61.4	5.68	180613.1	177186.6	195728.1	185104.9	205888.3	207397.4	189161.2	195808.5	179972.0	170254.0	200697.1	191031.4	209024.2	186148.5
P62331	ADP-ribosylation factor 6	Arf6	175	20.1	8.95	589821.3	547883.9	571993.0	556156.7	586612.0	605086.9	636909.4	559119.3	584409.7	578752.6	422640.8	405795.9	318454.0	412710.0
O88327	Alpha-catulin	Ctnnal1	731	81.4	6.55	283310.1	244311.9	209978.1	218079.9	237412.2	246729.1	261560.6	241450.2	229501.6	228469.6	92941.1	81079.3	93928.0	75681.1
Q9WVM3	Anaphase-promoting complex subunit 7	Anapc7	565	63	5.72	166704.0	183690.4	161901.5	164753.9	168452.9	171590.4	157390.6	158963.7	166785.1	175522.6	547703.8	590866.4	589097.8	553540.1
Q9D2E2	Target of EGR1 protein 1	Toe1	511	56.8	6.64	146187.3	154482.0	185665.0	166320.3	198442.2	199614.3	196517.7	146394.1	176819.1	201712.3	124170.5	142437.4	156591.9	127784.0
Q9JM14	5'(3')-deoxyribonucleotidase, cytosolic type	Nt5c	200	23.1	5.49	98343.7	96779.5	92457.8	85827.4	88843.7	85786.0	97109.3	87676.4	104988.8	100292.6	244768.3	243397.9	250176.8	239243.1
Q8VE37	Regulator of chromosome condensation	Rcc1	421	44.9	8.1	740723.9	782678.4	775715.1	722017.6	834098.2	857663.6	789889.4	705237.0	804846.6	822269.7	379508.3	373671.9	306830.4	352970.0
Q99LC9	Peroxisome assembly factor 2	Pex6	981	104.5	7.34	119429.4	116309.6	107973.0	103008.5	109746.2	114717.9	100597.9	101119.5	109247.1	114220.4	87347.4	83326.2	102232.5	93276.3
Q9JMB0	G kinase-anchoring protein 1	Gkap1	366	41.7	8.82	173215.8	199853.0	201856.6	201949.1	182416.3	183066.9	186248.9	173421.7	197255.5	198743.2	63833.1	82737.9	69880.5	77229.3
Q6ZPF4	Formin-like protein 3	Fmnl3	1028	117.1	6.67	115023.4	118227.8	119354.5	98756.7	132428.8	133608.0	107001.6	114748.2	100269.2	110073.4	164145.5	166369.4	162550.8	154956.2
Q9DC48	Pre-mRNA-processing factor 17	Cdc40	579	65.4	7.06	245139.4	280494.2	263697.7	280637.2	276496.9	279692.5	260459.5	245321.6	265855.7	287685.5	103912.4	119504.2	95850.8	94303.2
Q60648	Ganglioside GM2 activator	Gm2a	193	20.8	5.9	1585897.4	1495350.3	1759874.6	1364081.5	1864672.1	1549062.3	1589159.2	1671932.8	1616868.9	1600939.3	1210955.6	1161091.3	1292505.7	1031574.5
Q9QZD8	Mitochondrial dicarboxylate carrier	Slc25a10	287	31.7	9.32	343726.9	281328.9	306078.1	342243.2	314076.6	232505.0	248085.1	303417.7	282221.8	285910.2	254329.5	238893.8	227307.7	277860.2
Q9DBL7	Bifunctional coenzyme A synthase	Coasy	563	62	7.11	392590.5	373417.4	377629.0	388519.7	387119.2	397341.2	370862.7	387416.4	373971.5	388036.0	300976.4	306980.0	275176.2	284176.3
Q64127	Transcription intermediary factor 1-alpha	Trim24	1051	116.6	7.06	39620.4	42307.2	31861.1	33140.9	37730.1	32908.0	37253.2	36974.5	36603.0	38958.9	67443.0	74890.6	65184.9	63671.6
Q80WQ2	Protein VAC14 homolog	Vac14	782	88	6.13	243145.1	239803.8	234999.4	242139.9	237053.8	235542.7	247329.8	226912.0	269852.8	236312.1	148790.5	145654.2	150096.8	141634.3
Q811D0	Disks large homolog 1	Dlg1	905	100.1	5.8	150419.6	162753.8	141589.6	160699.1	169064.3	170863.1	174664.6	144003.4	150220.5	163235.2	184473.7	169435.6	175000.0	161031.6
Q6DID3	SR-related and CTD-associated factor 8	Scaf8	1268	139.5	8.24	125803.6	198795.9	143979.3	160003.1	167370.8	208301.2	134875.8	165381.1	151472.1	166506.2	115003.5	110600.3	107349.3	112019.6
O88712	C-terminal-binding protein 1	Ctbp1	441	47.7	6.77	194036.4	381204.0	189204.9	262135.7	394390.2	311887.3	196492.7	280385.4	239012.9	286869.2	410740.1	443930.1	425536.6	420560.0
Q99K28	ADP-ribosylation factor GTPase-activating protein 2	Arfgap2	520	56.6	8.18	309057.6	352298.1	341250.4	333777.9	339072.7	314568.7	304436.1	358013.1	328136.0	347839.4	301327.4	285704.5	292624.0	305552.2
P63044	Vesicle-associated membrane protein 2	Vamp2	116	12.7	8.13	173716.7	211157.5	246299.1	217973.4	199981.6	187248.7	160005.6	178807.2	211113.9	236056.6	494603.4	570454.2	597696.2	594697.6
Q99LM2	CDK5 regulatory subunit-associated protein 3	Cdk5rap3	503	57	4.83	407330.3	481191.9	492828.1	499843.2	486472.4	473066.9	482242.4	474140.2	478459.7	454567.6	219953.7	251458.9	241362.7	250641.7
Q6KAR6	Exocyst complex component 3	Exoc3	755	86.4	6.2	297701.9	287059.1	269003.5	274693.7	294788.1	302107.6	274721.4	268617.3	273435.8	270802.0	315089.6	319418.8	279677.5	307033.4
Q8N7N5	DDB1- and CUL4-associated factor 8	Dcaf8	591	66	5.87	100761.5	115032.3	114708.1	111512.2	107934.0	110027.7	99792.2	108500.9	107010.6	119870.9	184059.5	225513.4	205812.2	222170.8
Q60996	Serine/threonine-protein phosphatase 2A 56 kDa regulatory subunit gamma isoform	Ppp2r5c	524	60.8	6.64	73021.4	83971.3	81137.7	79856.4	79581.7	82821.0	78787.0	74928.8	67221.6	79160.9	61494.6	65369.7	67898.9	71790.1
P39053	Dynamin-1	Dnm1	867	97.7	7.74	142118.8	139991.2	113868.4	126553.5	139178.9	146315.7	124125.9	119201.4	122945.9	140502.2	66200.3	64515.7	54495.7	58417.3
Q9R0I7	YLP motif-containing protein 1	Ylpm1	1386	155	6.65	135558.6	124007.9	138888.8	133626.8	143937.8	139324.9	133678.0	134053.8	126356.8	127584.7	89268.3	77345.9	71516.8	78588.2
P97450	ATP synthase-coupling factor 6, mitochondrial	Atp5pf	108	12.5	9.36	132891.5	334889.3	161269.8	174747.5	136879.4	262418.8	198903.8	224622.1	203159.7	113389.1	72937.2	71004.6	85654.9	76198.8
O55047	Serine/threonine-protein kinase tousled-like 2	Tlk2	718	82.2	8.27	275640.8	289830.2	292200.5	292268.8	288728.2	329565.5	329128.5	335251.1	295915.1	288203.8	275785.8	272088.8	304716.7	280470.9
P36993	Protein phosphatase 1B	Ppm1b	390	42.8	5.19	126164.1	138787.7	131029.3	131035.7	137383.4	139213.2	126553.6	128271.2	139181.8	134855.8	260540.4	282248.5	267779.2	257006.7
Q8BMK0	Centrosomal protein of 85 kDa	Cep85	761	85.3	6.24	75276.4	79327.0	63984.5	65015.2	75609.8	66646.5	64439.2	58648.7	66136.2	70276.6	205636.9	226768.8	199640.7	229390.2
Q8C8N2	Protein SCAI	Scai	606	70.2	8.6	57383.5	59949.7	66406.4	57050.0	80506.9	74804.7	70826.3	62195.7	69012.4	70701.5	117010.6	121942.6	119253.4	111911.4
O35215	D-dopachrome decarboxylase	Ddt	118	13.1	6.54	1849917.5	2534351.5	2535968.4	3035120.9	1473648.2	1213055.4	1625648.0	1546342.3	2146802.2	2356040.8	1437544.0	1934442.9	2264337.3	2715607.4
Q91Z96	BMP-2-inducible protein kinase	Bmp2k	1138	126.1	6.8	164933.6	172915.8	144440.6	167477.4	136349.2	148891.9	168892.6	155741.9	169346.2	171855.5	69667.8	78668.2	66311.1	76068.7
Q4ZGD9	Nuclear RNA export factor 3	Nxf3	553	63.6	8.13	342176.6	387786.9	278656.5	338821.1	320470.4	303732.9	305948.5	300353.4	340037.2	343698.9	369129.9	359286.5	314718.1	345406.1
Q8BFV2	PCI domain-containing protein 2	Pcid2	399	46.1	8.53	110590.1	138442.0	128484.9	125731.4	147072.6	136535.3	121548.9	116145.5	131519.1	120725.3	155560.5	169446.5	161408.1	147928.0
Q9ER88	28S ribosomal protein S29, mitochondrial	Dap3	391	44.7	8.94	206821.7	188651.0	191384.5	202000.8	222980.8	227347.1	218130.6	197649.3	207177.9	190781.9	103787.2	87993.3	98405.2	95414.0
Q3UA37	Glutamine-rich protein 1	Qrich1	777	86.5	5.96	132777.8	131016.1	140003.1	142773.0	150116.5	157358.6	141740.5	144250.2	135173.2	141381.6	118161.8	139719.4	131239.1	124480.4
Q8VE99	Coiled-coil domain-containing protein 115	Ccdc115	180	19.7	8.81	160515.4	158423.4	159714.9	146916.0	162128.3	156365.3	143467.1	143874.3	163391.4	156283.1	177659.6	197444.2	190244.4	200208.3
Q9D3U0	tRNA pseudouridine synthase Pus10	Pus10	527	59.7	6.49	401871.6	426197.7	436227.1	442021.6	435636.5	440707.4	414924.5	394223.0	433418.8	477380.6	265924.3	166821.4	180399.1	172072.9
Q9D6S7	Ribosome-recycling factor, mitochondrial	Mrrf	262	29	9.85	165489.8	157888.5	174065.1	158679.4	158419.4	166521.1	167435.0	158852.0	164290.9	163344.8	78618.4	81821.4	72808.0	66010.8
O89020	Afamin	Afm	608	69.3	5.78	334647.8	313830.7	244865.3	273304.9	213873.3	256623.8	243035.0	312765.2	230129.4	229316.2	360218.3	352475.2	289649.2	328748.3
Q8BKT7	THO complex subunit 5 homolog	Thoc5	683	78.6	7.12	286546.9	279780.9	246467.2	272121.4	262918.3	245710.5	285416.6	242415.4	283296.1	296465.0	247859.0	286359.0	253477.2	246378.5
Q9D771	Proton-activated chloride channel	Pacc1	350	40.2	7.77	143206.7	137145.2	130186.5	136808.4	136364.1	155168.3	134444.6	149289.4	147003.3	137023.3	403557.7	380741.4	400455.9	377144.4
Q8C650	Septin-10	Septin10	452	52.4	6.6	22756.4	25971.6	23780.7	22567.9	22788.3	21109.1	18168.5	17621.0	23788.3	23599.9	24418.8	30560.7	37746.6	36554.2
Q9D7X8	Gamma-glutamylcyclotransferase	Ggct	188	21.2	5.67	307202.8	324213.3	289795.4	331902.4	287365.4	274458.0	260403.8	278965.0	302448.8	300532.1	598096.5	651759.1	581731.1	671177.9
P62320	Small nuclear ribonucleoprotein Sm D3	Snrpd3	126	13.9	10.32	621428.3	544113.7	677959.4	684067.2	618674.4	574507.2	563810.5	538006.8	621562.3	573429.5	620633.2	650918.9	654706.8	654877.5
Q91YE5	Bromodomain adjacent to zinc finger domain protein 2A	Baz2a	1889	209.5	6.51	60115.1	72448.5	58174.4	59781.6	62911.9	67304.5	44623.1	54455.7	58524.3	57650.3	225083.2	318595.2	263874.6	265653.7
Q8C796	REST corepressor 2	Rcor2	523	57.9	9.01	146953.1	164876.4	152699.4	155284.6	173293.7	157642.5	147661.6	149074.5	154938.5	153543.7	166142.2	153247.6	154666.0	142679.4
Q9WV32	Actin-related protein 2/3 complex subunit 1B	Arpc1b	372	41	8.35	508025.7	384311.8	431685.6	416089.3	545134.4	623961.1	655023.9	436207.8	492425.6	512211.7	1054918.0	812358.7	919947.5	794475.0
Q9D1H9	Microfibril-associated glycoprotein 4	Mfap4	257	28.9	5.44	1062052.6	867890.6	1438375.8	1017078.4	1644613.2	2218745.0	3543805.0	1430524.2	1582163.9	1240217.3	953256.9	660381.6	1141223.3	684278.8
Q68FF6	ARF GTPase-activating protein GIT1	Git1	770	85.2	6.93	237224.3	203497.4	226169.6	206883.4	205051.7	222257.6	208987.3	219569.9	200745.4	223826.8	229081.6	207791.3	210512.6	215407.4
Q8CHW4	Translation initiation factor eIF-2B subunit epsilon	Eif2b5	717	80	5.07	129978.1	138457.7	144190.2	140394.0	146586.8	136049.7	139318.3	150386.9	142269.6	129789.5	206128.5	220420.6	213545.9	230862.3
Q8R010	Aminoacyl tRNA synthase complex-interacting multifunctional protein 2	Aimp2	320	35.4	7.83	182346.2	194865.3	189605.8	189943.0	184083.2	174990.5	164915.1	164471.6	194808.0	205404.1	272291.5	291895.9	254037.9	269652.5
P97346	Nucleoredoxin	Nxn	435	48.3	4.93	99299.0	106859.8	102061.4	126630.7	105254.3	102426.7	90167.9	104024.7	106611.2	104986.4	78602.2	95269.0	88384.5	98185.7
Q64133	Amine oxidase [flavin-containing] A	Maoa	526	59.6	7.81	220137.6	208642.2	195427.5	207037.5	183492.6	195665.9	196134.9	174079.2	219772.7	242559.6	236693.9	224701.7	213583.6	251839.8
Q9Z0P4	Paralemmin-1	Palm	383	41.6	4.84	87781.1	88863.4	89529.9	91741.5	128209.7	144692.1	135673.4	104202.2	97384.5	95498.0	153120.1	133863.6	133316.1	135784.8
Q8BFW7	Lipoma-preferred partner homolog	Lpp	613	65.8	7.37	41312.3	44114.0	39767.2	39672.4	43927.1	44401.0	51862.8	42899.6	39040.0	37244.8	194116.4	178264.0	189723.8	185406.7
O54826	Protein AF-10	Mllt10	1068	113	8.19	27634.1	37946.1	39520.9	41880.5	26238.6	26041.7	24176.6	22799.5	36833.7	32553.0	51934.9	55899.8	55158.4	58114.4
Q9D0M0	Exosome complex exonuclease RRP42	Exosc7	291	31.8	5.19	155558.5	152875.2	147363.2	150489.9	162549.4	151979.8	162636.9	151165.4	169081.7	180069.5	163793.1	188563.2	177784.3	152647.7
Q8VCD5	Mediator of RNA polymerase II transcription subunit 17	Med17	649	72.4	7.3	88720.9	116946.7	107095.2	104898.4	97964.3	108355.6	97852.6	102538.2	102694.8	106759.1	261932.6	292062.3	339027.5	318355.4
Q61545	RNA-binding protein EWS	Ewsr1	655	68.4	9.33	479762.2	550543.2	591636.8	631064.4	590020.5	552721.8	561117.7	511908.7	585741.7	600210.6	476689.2	418635.9	581814.1	487798.4
O08797	SERPIN domain-containing protein	Serpinb9	374	42.2	5.4	129817.6	153346.2	107955.6	152092.7	116187.6	148205.1	157381.7	150565.6	107940.9	123014.3	105908.8	148255.7	104698.1	136192.6
Q8VDF2	E3 ubiquitin-protein ligase UHRF1	Uhrf1	782	88.2	8.31	217836.8	211673.6	172837.2	223269.2	132370.9	146852.6	168052.1	205965.3	191424.7	162802.7	194108.6	210333.4	150119.0	196814.2
Q9EPJ9	ADP-ribosylation factor GTPase-activating protein 1	Arfgap1	414	45.3	5.57	67839.0	70674.2	70892.7	67751.3	70429.1	70977.2	68618.9	75789.7	62808.7	64404.3	595675.9	549129.3	489031.8	521565.8

P02463	Collagen alpha-1(IV) chain	Col4a1	1669	160.6	8.24	154369.9	155073.4	151541.4	150139.7	215177.5	183528.8	174722.9	157193.4	170279.7	189122.3	70865.7	96070.6	77894.5	79615.5
Q6PA06	Atlastin-2	Atl2	583	66.2	5.43	149857.4	160341.8	137799.0	155472.8	122592.8	125405.9	130094.5	132539.7	139406.2	145815.9	246688.8	253889.1	247955.5	260257.5
Q9D8S3	ADP-ribosylation factor GTPase-activating protein 3	Arfgap3	523	57.4	8.47	262374.0	330250.5	306043.7	357323.2	269341.3	229609.4	203422.7	254907.5	268833.5	266762.1	78970.3	87673.2	87327.4	90412.1
Q8C170	Unconventional myosin-IXa	Myo9a	2542	291.9	8.97	81348.6	89940.9	86207.6	93336.5	90976.1	101127.6	92236.0	94210.6	102995.4	93904.1	119503.7	145682.5	131133.7	148340.8
Q9WUD1	STIP1 homology and U box-containing protein 1	Stub1	304	34.9	6.01	128876.2	141967.9	139720.9	144558.8	136745.8	126057.5	121442.9	133805.7	139622.1	135673.2	258706.1	252289.2	243389.1	251003.3
Q9JKY0	CCR4-NOT transcription complex subunit 9	Cnot9	299	33.6	8.03	116631.4	125787.2	115604.3	112833.6	111050.7	104544.5	100587.1	101945.3	109902.7	123579.0	68770.8	59586.5	62930.0	59026.8
Q80Y98	Phospholipase DDHD2	Ddhd2	699	79.5	5.31	45421.9	49937.3	45293.3	51980.2	48324.2	46147.6	42393.1	45904.5	50903.7	45287.5	191344.9	203152.6	175959.6	178349.6
B2RRE7	OTU domain-containing protein 4	Otud4	1107	123	6.76	118891.1	139244.0	139684.6	133787.4	142576.4	151127.3	125190.0	136265.3	141278.0	149163.8	65792.5	83657.9	63960.9	67663.6
Q3U4S0	Pantothenate kinase 2	Pank2	443	48.6	8.44	109499.4	128193.6	107744.8	125770.5	123634.4	118891.0	98974.6	111124.9	116546.1	125651.6	159363.6	163668.0	162978.7	159217.7
Q9CX00	IST1 homolog	Ist1	362	39.4	5.44	483026.8	500895.2	501214.6	477978.7	531677.1	507083.3	507588.0	481049.5	480250.5	481893.8	627944.3	627841.9	626686.2	636184.4
Q91WG2	Rab GTPase-binding effector protein 2	Rabep2	554	62.1	4.89	222323.4	211091.6	210050.4	213323.5	211408.5	206930.4	223085.6	217536.5	213662.7	208790.1	121424.9	121181.1	117784.5	118888.9
Q61112	45 kDa calcium-binding protein	Sdf4	361	42	4.96	93637.5	101671.1	85522.4	93991.4	89882.8	88662.3	80313.2	89328.4	91555.4	97951.1	53784.1	57324.9	54313.8	62792.1
P83510	Traf2 and NCK-interacting protein kinase	Tnik	1323	150.3	7.27	273330.0	322437.2	268307.6	284878.0	302381.3	322936.0	305127.6	286085.3	295030.3	304554.2	189150.2	209150.9	189768.5	195225.6
Q8C0J2	Autophagy-related protein 16-1	Atg16l1	607	68.1	6.38	209933.8	211599.9	213976.2	220642.4	206580.4	241947.2	225795.9	214975.2	236969.2	250234.9	251223.4	246669.5	262200.1	267771.0
Q3UJU9	Regulator of microtubule dynamics protein 3	Rmdn3	470	52	5.21	76868.9	103450.3	96172.3	103549.6	89536.9	89817.8	82944.0	89350.6	89401.1	100163.3	115056.9	119197.9	111621.1	127586.2
O70591	Prefoldin subunit 2	Pfdn2	154	16.5	6.58	320781.4	357104.2	366889.0	359966.8	401404.7	371480.8	334940.2	369041.7	406677.6	370892.3	423643.6	497891.9	471012.7	382259.2
Q9Z0R6	Intersectin-2	Itsn2	1659	188.8	8.13	189233.2	183731.2	162842.1	167907.0	176192.4	172615.5	175449.2	195874.9	175663.4	184197.3	113023.1	106496.7	100862.5	105796.6
Q9Z1K5	E3 ubiquitin-protein ligase ARIH1	Arih1	555	64	5.08	158287.0	186961.9	119751.3	158899.9	145234.8	148099.9	127936.4	131798.6	147284.2	161177.7	187863.5	186223.2	188676.9	190779.5
Q8R124	Kelch-like protein 36	Klhl36	613	69.9	5.9	135755.7	140415.4	138665.4	147777.8	134000.5	121531.9	131483.8	138940.6	147029.6	128066.4	101555.9	94210.0	98518.2	97493.4
Q9ESU6	Bromodomain-containing protein 4	Brd4	1400	155.8	9.19	128665.5	152402.2	139665.0	135792.6	163219.1	152877.0	163641.6	136642.4	146858.2	148700.1	262156.3	290856.8	293779.7	254771.6
Q8K1A6	Coiled-coil and C2 domain-containing protein 1A	Cc2d1a	943	103.6	7.84	243197.5	252420.3	233692.0	236800.4	248962.0	247067.4	228405.5	247625.6	228118.4	243108.3	137832.5	143866.4	141322.2	147586.4
Q69Z99	Zinc finger protein 512	Znf512	562	63.9	9.51	111319.8	98704.1	112539.1	98878.8	137033.3	137925.4	134859.8	119752.8	117639.0	119984.8	71984.7	61982.6	87952.5	57855.8
Q64442	Sorbitol dehydrogenase	Sord	357	38.2	7.02	415595.6	433677.0	423420.6	436399.0	446116.3	405943.9	428479.0	401025.1	440627.7	424385.1	147697.4	121091.1	105222.4	119120.2
Q99L04	Dehydrogenase/reductase SDR family member 1	Dhrs1	313	34	8.35	142855.0	148684.8	142663.1	155826.0	128663.6	114328.8	129863.0	147198.1	223951.0	138058.0	220882.0	231696.8	202350.2	233591.2
Q9D1G5	Leucine-rich repeat-containing protein 57	Lrrc57	239	26.7	8.35	150069.4	143625.9	134863.2	147660.1	142362.2	146006.6	170890.3	139981.5	140060.8	147162.8	111115.7	101360.6	99391.4	107537.7
Q99PG2	Opioid growth factor receptor	Ogfr	633	70.6	4.77	242262.8	234677.4	250174.7	240759.3	244771.5	235580.2	213603.2	206293.4	228467.7	244903.8	252404.3	198341.4	179950.5	201764.8
Q9D1H7	Golgi to ER traffic protein 4 homolog	Get4	327	36.5	5.41	304030.1	295349.3	270225.2	324198.1	287350.3	289981.1	310339.0	298231.5	292986.7	296642.1	261051.0	352388.0	249452.3	259943.4
Q7TSH4	Centriolar coiled-coil protein of 110 kDa	Ccp110	1004	111.1	8.85	87863.9	90900.1	100060.1	90301.9	95962.6	89122.0	79366.9	88489.6	89917.9	90189.2	147610.3	130899.0	130733.9	136816.5
Q497V5	S1 RNA-binding domain-containing protein 1	Srbd1	982	110.2	8.78	257743.0	254123.6	233933.4	232743.4	248526.5	263326.7	232907.3	215564.5	245221.1	265915.1	397679.9	384784.1	396804.3	400355.1
Q8CH25	SAFB-like transcription modulator	Sltm	1031	116.9	7.72	232122.7	241811.1	248550.1	260278.4	251604.3	210318.7	206753.9	195908.9	224583.7	219908.8	135859.8	135463.1	128373.6	165082.7
Q9D8B3	Charged multivesicular body protein 4b	Chmp4b	224	24.9	4.82	491227.7	535299.5	529580.3	560326.2	549047.4	514258.6	519238.6	523918.7	553931.1	546333.1	318129.8	344008.7	401177.1	408226.9
Q8CCS6	Polyadenylate-binding protein 2	Pabpn1	302	32.3	5.17	307007.4	530892.2	320928.9	370388.5	384276.7	493979.9	322407.8	388154.3	348814.1	363883.4	1051950.7	1014328.8	1187041.0	1070704.9
Q91XD6	Vacuolar protein-sorting-associated protein 36	Vps36	386	43.7	7.15	148833.9	163840.0	168687.5	162934.8	171858.4	142189.4	149599.9	151306.7	148962.0	162327.4	127133.0	135477.3	115597.0	125605.5
P97770	THUMP domain-containing protein 3	Thumpd3	505	56.4	7.43	180112.5	181019.3	180870.3	187215.6	161330.4	169207.0	144865.7	161005.8	178539.4	181464.0	187329.8	64980.1	67573.4	68280.8
Q8BK58	HSPB1-associated protein 1	Hspbap1	483	54.4	5.27	156344.0	155782.7	162263.9	144726.0	172701.9	178415.8	150163.5	158420.0	145348.3	164620.3	154393.1	142672.1	146386.9	130780.7
Q3UV70	[Pyruvate dehydrogenase [acetyl-transferring]]-phosphatase 1, mitochondrial	Pdp1	538	61.1	6.67	194921.5	187038.5	196542.8	203143.6	217503.9	217656.5	221241.9	201748.7	224084.5	206610.6	114410.8	105972.1	109706.9	115239.5
Q8C078	Calcium/calmodulin-dependent protein kinase kinase 2	Camkk2	588	64.6	5.9	98368.8	84638.2	99539.0	86357.2	81675.4	88567.3	85923.6	92143.1	97506.8	88657.6	364945.7	323181.1	343749.0	321906.3
Q91VH2	Sorting nexin-9	Snx9	595	66.5	5.52	202265.4	207198.3	170477.6	202358.2	188328.1	201305.2	200815.0	178620.8	184843.2	196213.1	346741.7	324116.1	332556.8	312440.5
Q3TWW8	Serine/arginine-rich splicing factor 6	Srsf6	339	39	11.46	1616314.0	1617701.2	1598261.3	1672245.7	1654421.3	1566016.2	1631679.0	1524018.9	1711238.9	1659544.1	579356.8	615001.0	550277.6	578610.7
P10648	Glutathione S-transferase A2	Gsta2	222	25.5	8.81	901809.2	1033126.0	1162680.7	1109287.9	1064284.8	863877.2	1412962.8	1019208.3	1156572.9	1185859.2	1535975.2	1798095.6	2305111.5	1969449.9
O08912	Polypeptide N-acetylgalactosaminyltransferase 1	Galnt1	559	64.2	7.71	138838.1	135015.9	119911.5	130641.7	128924.0	124490.6	121344.4	122549.0	130521.7	128156.5	88276.7	93380.0	85994.4	79693.2
Q3TUU5	Testis-expressed protein 30	Tex30	225	25.2	8.75	1405337.1	2043127.1	2235484.4	2128222.5	1906357.4	1700732.0	1137187.4	1380987.9	2125903.6	2209389.2	1752332.6	2133802.7	2183609.2	2207345.8
O88974	Histone-lysine N-methyltransferase SETDB1	Setdb1	1307	144.5	5.99	172421.5	175174.5	196153.7	181174.7	182282.8	183916.6	156023.2	173268.2	181395.7	178963.9	276923.9	272250.0	267902.6	270014.6
Q9Z1K6	E3 ubiquitin-protein ligase ARIH2	Arih2	492	57.7	5.69	107325.9	105883.1	120055.3	110407.9	114788.9	102028.6	90637.9	90023.7	117047.9	107246.1	108504.5	123355.1	125995.4	133758.0
Q8C8R3	Ankyrin-2	Ank2	3898	426	5.17	126893.6	116253.6	109398.9	114253.6	106131.6	102610.2	108800.8	111099.3	108001.5	104539.2	135931.5	119845.7	127800.1	120574.5
Q8BWZ3	N-alpha-acetyltransferase 25, NatB auxiliary subunit	Naa25	972	111.6	6.52	115340.0	131225.4	114863.7	133106.2	112009.4	119636.0	110277.5	111515.3	121107.0	126500.7	185871.3	237154.7	205610.7	211571.6
P21995	Embigin	Emb	330	37	6.02	183764.8	191169.3	169400.0	181121.9	167882.5	153564.9	167879.9	169723.6	171338.9	146606.4	530414.2	565652.9	477568.9	534412.4
Q8BGU5	Cyclin-Y	Ccny	341	39.4	7.2	111359.8	129038.6	119673.7	123560.0	130208.5	129089.8	114918.2	123782.5	120453.1	120695.1	98908.9	93701.9	89781.5	91129.4
Q9D6Y9	1,4-alpha-glucan-branching enzyme	Gbe1	702	80.3	6.43	240916.6	284605.8	248145.9	254937.1	262790.2	295768.1	271384.1	268645.9	282919.1	282206.9	122714.1	165892.5	122664.2	131434.8
P35980	60S ribosomal protein L18	Rpl18	188	21.6	11.78	668653.4	819349.6	673295.3	752010.9	757019.1	682556.2	687863.2	598789.0	740634.6	832501.9	1017207.8	1134532.1	1057914.6	1083635.1
Q9JJY4	Probable ATP-dependent RNA helicase DDX20	Ddx20	825	91.7	6.74	88630.4	110265.7	98788.9	91238.6	98915.1	101654.6	94747.5	83338.2	95750.5	104626.8	283178.2	301626.5	266100.8	268057.1
Q8BRH0	Protein O-mannosyl-transferase TMTC3	Tmtc3	920	104.1	8.53	154015.1	144634.7	134880.4	131882.4	145039.0	158953.6	165390.8	149226.0	148663.1	139339.3	263077.6	258750.9	248580.1	250458.6
Q3TTY5	Keratin, type II cytoskeletal 2 epidermal	Krt2	707	70.9	8.06	781348.0	769103.8	850216.7	819252.7	1245934.1	1309103.9	960266.2	1011252.2	1052083.1	931291.0	863956.4	707336.3	748915.5	843357.3
O88643	Serine/threonine-protein kinase PAK 1	Pak1	545	60.7	5.74	110039.2	112838.7	109323.8	106274.2	115366.0	118770.3	103423.2	105242.5	102807.2	104302.6	95146.2	105142.8	93520.2	91973.3
Q7TNC4	Putative RNA-binding protein Luc7-like 2	Luc7l2	392	46.6	10.1	207360.6	202427.4	220668.6	213135.1	225030.5	242665.7	227101.3	208468.3	217364.8	227433.8	937394.7	945134.5	1049311.3	1014274.5
P84099	60S ribosomal protein L19	Rpl19	196	23.5	11.47	331569.5	353173.3	354333.0	343022.5	352656.1	318114.9	283249.9	346188.8	339786.2	329737.9	953935.7	867845.7	1100751.5	1041072.2
Q8K2Q7	BRO1 domain-containing protein BROX	Brox	411	46.2	7.69	360630.9	328264.7	394114.8	302700.5	401863.1	393207.5	357542.2	373127.6	342210.0	334933.7	405749.3	372159.7	416654.2	361344.0
Q6P8J2	Diamine acetyltransferase 2	Sat2	170	19.3	6.04	374796.1	332199.8	317194.9	400570.3	342215.5	330763.6	357505.7	326763.8	329423.7	319819.3	427227.1	416413.6	395440.8	417931.9
Q9WTX8	Mitotic spindle assembly checkpoint protein MAD1	Mad1l1	717	83.5	5.66	218013.2	238136.5	200170.8	215472.6	242489.0	230678.7	219646.7	206285.1	222154.3	232795.6	77717.1	96390.4	85677.3	87871.4
Q7TNL7	Dual specificity protein phosphatase	Dusp9	452	48.6	5.16	51255.1	63331.5	40360.2	54363.9	47402.4	53436.1	39494.0	47799.8	47214.9	48792.3	83092.1	87165.9	71634.3	82597.1
E9Q8I7	RING-type domain-containing protein	Nfxl1	918	101.4	8.4	143256.0	161751.6	150936.6	148154.3	156840.8	141313.0	125815.0	119673.6	152276.7	152193.6	198603.2	177316.1	176933.7	198471.4
Q62203	Splicing factor 3A subunit 2	Sf3a2	475	49.9	9.54	616342.2	646735.1	654736.5	632172.6	727150.1	629213.4	620344.8	598840.2	629480.4	643223.3	182735.6	210731.1	226967.6	213545.7
P54116	Erythrocyte band 7 integral membrane protein	Stom	284	31.4	6.93	120263.4	156386.8	142639.0	172118.4	124038.1	117810.5	102441.5	135564.7	123209.0	128327.3	148585.7	136534.6	129959.6	142706.9
P53564	Homeobox protein cut-like 1	Cux1	1515	165.5	6.32	147045.6	123870.9	143623.9	125471.8	149778.2	140061.7	156973.7	138749.7	139751.1	132998.3	129383.5	99038.1	119868.5	99617.4
P61082	NEDD8-conjugating enzyme Ubc12	Ube2m	183	20.9	7.69	115929.5	119654.8	131812.3	118730.9	105158.1	102514.1	95976.3	115222.7	115248.4	103548.5	422949.2	455951.3	425954.5	494402.7
P62254	Ubiquitin-conjugating enzyme E2 G1	Ube2g1	170	19.5	5.3	168420.5	163858.4	165250.5	162402.2	168467.4	178415.5	171918.2	162833.5	176684.2	170661.6	108099.6	108747.8	106271.8	90804.1
A6H5Z3	Exocyst complex component 6B	Exoc6b	810	94.1	6.39	183539.4	231304.1	179643.9	182299.2	204846.3	217659.0	178632.0	190058.7	175038.5	200364.7	89294.7	90196.0	82333.5	90732.0
Q3UGF1	WD repeat-containing protein 19	Wdr19	1341	151.4	6.57	250398.8	307817.7	289666.7	334777.2	259121.8	251981.7	235888.0	230592.1	261929.5	294543.0	231447.2	274151.0	272868.8	303771.2
P59900	EMILIN-3	Emilin3	758	82.3	6.93	39970.7	39891.3	49876.7	42179.3	66060.2	75882.2	64958.2	49609.2	49606.2	49695.8	48243.4	45923.5	40542.7	46105.0
Q8C0D5	Elongation factor-like GTPase 1	Efl1	1127	125.7	6.16	144407.7	161586.1	163660.7	145844.7	152299.4	144160.3	147847.3	148005.5	158115.8	163867.0	187373.5	237299.7	203938.9	201725.9
Q925E7	Serine/threonine-protein phosphatase 2A 55 kDa regulatory subunit B delta isoform	Ppp2r2d	453	51.9	6.49	169776.8	172297.9	185441.4	165433.6	191255.6	192669.8	186884.5	175596.7	189531.2	182519.2	206359.1	182430.6	220010.7	187818.5
P63001	Ras-related C3 botulinum toxin substrate 1	Rac1	192	21.4	8.5	323720.9	313061.7	307963.7	303624.5	311526.4	327721.0	318353.6	302174.2	308712.0	322876.2	520950.9	513540.1	490693.0	472374.9
Q3UGR5	Haloacid dehalogenase-like hydrolase domain-containing protein 2	Hdhd2	259	28.7	6.05	151042.7	171379.9	135054.6	154670.5	218587.8	162035.6	152653.3	160960.6	157921.9	154225.8	414445.0	427810.9	385052.0	398543.6
Q9WTK3	Glycosylphosphatidylinositol anchor attachment 1 protein	Gpaa1	621	67.9	8.4	303728.8	277605.1	288811.4	277019.2	288590.4	276496.2	228698.2	274959.8	281061.3	270812.0	226236.9	281907.1	257686.4	266289.1
Q6PIP5	NudC domain-containing protein 1	Nudcd1	582	66.7	5.3	449683.5	501644.8	430409.2	441283.9	450706.4	451285.9	418957.4	416965.2	444728.0	477729.6	125746.9	136641.7	134703.4	124503.2
O35405	5'-3' exonuclease PLD3	Pld3	488	54.4	6.52	205861.6	204459.7	177628.5	194568.0	181090.2	169302.4	164266.4	177026.2	180607.2	189874.6	330216.4	365107.9	230256.5	308653.3
Q9CZP5	Mitochondrial chaperone BCS1	Bcs1l	418	47.4	7.93	207600.4	219502.8	193264.5	181004.7	217853.8	218878.3	175986.5	198758.2	201661.7	189576.6	227345.7	249296.3	215979.2	236268.9
Q60949	TBC1 domain family member 1	Tbc1d1	1255	141.9	7.24	155642.5	170760.3	169724.8	167348.0	173686.1	177934.7	146730.6	151126.1	160414.9	169924.5	95341.8	129408.6	109692.8	130919.0
Q5VCS6	Tudor domain-containing protein 5	Tdrd5	1040	115.9	8.12	168359.4	218532.6	183173.4	210199.2	175859.5	150280.2	129130.7	161253.9	171739.3	181826.0	76164.6	89460.8	84941.9	97636.7
Q5SWD9	Pre-rRNA-processing protein TSR1 homolog	Tsr1	803	92	6.99	170831.2	194629.6	180539.1	163985.9	206799.4	220892.7	176231.9	203908.3	180037.6	195096.7	168202.5	178981.2	167224.3	164210.5
Q9CQU0	Thioredoxin domain-containing protein 12	Txndc12	170	19	5.26	415028.8	474938.9	440653.6	462283.1	427025.0	371161.9	407898.3	404614.5	457148.2	459609.0	413366.6	390618.2	456509.0	423769.5
Q8CBW3	Abl interactor 1	Abi1	481	52.3	7.64	150691.8	166040.4	152114.0	145549.1	152666.2	161475.2	151367.1	160766.2	152883.8	145035.9	281879.2	230936.4	251289.9	228634.4
Q5SVQ0	Histone acetyltransferase KAT7	Kat7	613	70.6	8.72	60594.1	70272.7	63381.7	63160.8	71523.3	69490.6	66368.1	61826.6	73262.7	70108.3	71250.8	80771.0	86340.2	83663.5
Q99LS3	Phosphoserine phosphatase	Psph	225	25.1	6.14	299153.8	276203.8	311129.1	297612.7	339849.7	350029.9	344666.8	298874.8	314514.3	320334.5	58229.1	65070.7	72021.4	72658.9
Q99MR3	Solute carrier family 12 member 9	Slc12a9	914	96.3	7.02	274108.2	347241.5	240212.8	270642.5	327886.4	332220.8	227599.7	302160.0	279212.0	316320.8	130306.3	153391.9	143644.6	175712.4
P35283	Ras-related protein Rab-12	Rab12	243	27.3	8.41	294818.7	322521.6	276095.4	291295.5	266340.6	271671.8	267816.2	284723.8	272842.3	296796.7	192574.1	168757.8	179952.0	178474.3
Q8R1X6	Spartin	Spart	671	72.6	5.86	87804.4	86469.4	95956.9	90164.6	90613.5	98433.4	98661.2	88466.2	80353.1	78424.2	127284.1	140319.6	122118.4	123445.3
Q9D281	Protein Noxp20	Fam114a1	569	61	4.56	155436.4	145957.7	144081.4	143295.6	142534.8	148018.6	154842.2	159351.8	137760.4	136565.6	261383.3	251239.9	240984.3	244174.5
Q8R2E9	ERO1-like protein beta	Ero1b	467	53.5	8.09	360781.2	422960.8	394167.2	410210.6	331260.4	315714.8	263256.9	294120.5	375542.7	380783.1	118328.2	150183.7	137370.6	149872.2
Q9D620	Rab11 family-interacting protein 1	Rab11fip1	645	70.6	9.47	262835.0	249138.3	238938.7	263031.1	238351.6	252497.2	246417.2	272049.1	261126.6	213856.9	92050.6	96230.8	81780.0	84539.8
Q6NSR8	Probable aminopeptidase NPEPL1	Npepl1	524	55.9	6.84	161294.6	169762.4	149329.2	162741.5	172207.9	181602.5	169960.0	142913.3	179721.6	151484.2	71396.3	74086.3	58802.5	64310.7
O35900	U6 snRNA-associated Sm-like protein LSm2	Lsm2	95	10.8	6.52	313088.6	247005.6	362858.9	259344.8	355142.9	351678.1	270206.6	317745.9	289010.1	274635.3	750840.6	701135.8	875524.6	776663.9

Q9CZJ2	Heat shock 70 kDa protein 12B	Hspa12b	685	76.1	8.47	49260.5	47951.3	49625.5	50291.6	53618.2	58272.5	56759.4	53051.4	49177.0	53698.6	47682.8	52530.4	55181.5	52773.3
O88325	Alpha-N-acetylglucosaminidase	Naglu	739	82.5	6.61	232611.1	265234.7	219711.6	256152.6	213966.5	221934.3	237216.0	223938.5	218076.0	229509.0	119684.5	125769.9	106919.8	133090.8
Q99K70	Ras-related GTP-binding protein C	Rragc	398	44.1	5.1	511791.5	557849.5	491246.8	540036.5	484132.8	483557.7	457480.3	451330.0	494388.1	515572.2	269555.4	265907.9	276570.8	245365.1
Q91XL3	UDP-glucuronic acid decarboxylase 1	Uxs1	420	47.5	8.94	40182.3	47359.1	44800.2	35924.0	39306.1	36216.6	36321.2	39126.9	38998.2	41019.9	76237.4	71625.6	63318.5	64236.2
Q62219	Transforming growth factor beta-1-induced transcript 1 protein	Tgfb1i1	461	50.1	6.73	26222.0	25454.8	26937.6	21690.7	26898.6	25693.2	24490.8	28178.3	28793.4	26344.3	86496.2	92646.4	78196.3	86391.7
Q9D358	Low molecular weight phosphotyrosine protein phosphatase	Acp1	158	18.2	6.74	232688.9	226420.0	234381.3	234406.4	225593.4	216470.6	197737.5	218704.6	214539.6	230283.8	266318.0	312601.4	264252.3	326777.8
Q99NB3	Ig-like domain-containing protein	Tcam1	548	60.7	5.26	148643.7	164595.5	177101.2	199169.6	131393.7	134272.7	121298.6	113143.2	144370.0	168903.8	210545.8	261026.6	259223.2	282142.3
Q5SW75	Protein phosphatase Slingshot homolog 2	Ssh2	1423	158.1	5.67	64682.6	68972.9	75426.1	78597.7	64880.4	61253.0	64877.9	61458.6	65374.4	64738.4	128437.1	151924.0	140580.2	161771.7
Q5SUE7	Adenosine deaminase domain-containing protein 1	Adad1	619	67.8	8.85	161690.5	177480.8	182556.8	195387.1	146957.1	142017.3	118909.7	141231.2	163835.5	174324.4	108736.2	152698.9	149816.8	157516.9
P50543	Protein S100-A11	S100a11	98	11.1	5.45	103642.6	94157.5	102969.4	85238.6	123774.9	127817.6	127377.1	117193.1	113872.3	99739.8	908606.7	861925.3	997739.3	819851.3
Q9D7V1	SH2 domain-containing protein 4A	Sh2d4a	421	48.4	8.91	103115.9	107189.0	110674.9	105615.8	124034.5	130112.4	119710.0	106219.8	120710.6	117231.1	85294.6	93402.6	97998.8	84990.9
E9Q4N7	AT-rich interactive domain-containing protein 1B	Arid1b	2244	237.6	6.73	103389.3	99066.4	104212.7	102436.3	129706.3	141249.9	123628.0	111779.9	110832.8	105597.7	39886.3	42084.8	45757.5	37400.9
Q9CQH3	NADH dehydrogenase [ubiquinone] 1 beta subcomplex subunit 5, mitochondrial	Ndufb5	189	21.7	9.41	276413.1	327053.4	321608.3	350935.8	332975.2	272807.5	314305.3	305604.4	330127.9	343462.8	250124.9	271663.8	257893.8	283387.3
G5E8V9	ADP-ribosylation factor-interacting protein 1	Arfip1	373	41.5	6.76	521168.4	511837.4	580327.7	561882.1	593888.7	651080.9	526989.4	574930.7	545331.0	570149.8	216124.3	202243.0	222347.1	221233.4
Q9D868	Peptidyl-prolyl cis-trans isomerase H	Ppih	188	20.5	8.09	624124.9	698283.5	771909.1	677233.7	728481.8	699291.8	600641.1	625954.6	715138.0	751458.4	440562.3	518206.0	552642.7	531809.4
Q8CG72	ADP-ribose glycohydrolase ARH3	Adprs	370	39.4	4.96	401744.1	413589.8	437649.1	413915.4	474270.4	456607.6	430242.3	444963.7	432259.9	408007.2	251390.4	246678.2	237430.4	256451.0
Q8BGT7	Survival of motor neuron-related-splicing factor 30	Smndc1	238	26.7	7.24	108584.5	127056.8	120692.3	118372.2	120519.7	127722.7	127019.2	119015.5	130114.6	132260.1	105649.8	115166.8	97062.0	110901.9
Q9ES56	Trafficking protein particle complex subunit 4	Trappc4	219	24.4	6.21	525063.6	554893.2	583070.7	574444.9	560153.9	504765.3	499367.6	498378.0	533000.5	559389.6	618611.3	713399.1	664698.7	698661.7
P12787	Cytochrome c oxidase subunit 5A, mitochondrial	Cox5a	146	16.1	6.54	1078986.1	1221150.8	1318803.8	1458708.5	1218122.8	1077424.5	1241250.5	1268131.0	1504068.2	1303757.9	1529176.5	1587210.1	1888376.3	1985228.8
O88811	Signal transducing adapter molecule 2	Stam2	523	57.4	5.07	127428.2	127932.5	131111.2	129826.1	127755.6	135973.5	133067.3	118480.6	131845.4	129075.7	242979.3	256594.0	241637.6	247352.8
P11438	Lysosome-associated membrane glycoprotein 1	Lamp1	406	43.8	8.4	538950.7	771896.5	505095.3	627915.0	644621.5	538586.9	448716.7	546716.9	568856.0	619659.3	610598.5	572535.6	738487.7	638866.4
Q9D6T0	Nitric oxide synthase-interacting protein	Nosip	301	33.2	8.62	266380.6	277526.8	283889.9	248973.5	294277.4	285806.8	312709.5	283925.7	296002.0	294264.7	247156.1	253254.8	257647.3	230611.4
Q9QZW0	Phospholipid-transporting ATPase 11C	Atp11c	1129	129.2	7.3	124290.1	135591.4	126082.1	126337.9	126991.2	127640.4	119862.2	120301.7	136627.0	138411.2	94068.1	132845.0	108894.9	124000.2
P39749	Flap endonuclease 1	Fen1	378	42.3	8.34	263041.3	310847.9	277222.4	272128.6	342714.6	334271.0	309428.3	275015.3	304857.4	305564.1	262795.2	288512.3	257859.6	267702.8
Q9JJV2	Profilin-2	Pfn2	140	15	6.99	336040.4	329816.5	338844.5	383409.7	304983.3	310371.1	284153.7	289298.6	314469.6	342885.6	512551.0	542276.5	545584.0	549597.5
Q9CXG3	Peptidyl-prolyl cis-trans isomerase-like 4	Ppil4	492	57.2	6.11	128943.3	139726.2	135250.9	133256.5	130006.5	128936.8	115889.1	118881.0	133727.8	134290.4	110974.3	121005.1	118077.4	127209.2
Q8BH55	Threonine synthase-like 1	Thnsl1	747	83	7.2	89285.5	90940.8	91484.2	91258.3	82603.4	78601.4	72133.3	72847.8	92697.0	92911.9	160510.2	179743.4	177023.6	199984.0
Q09XV5	Chromodomain-helicase-DNA-binding protein 8	Chd8	2582	290.7	6.42	159028.8	202476.2	157007.5	193098.3	192645.7	219162.5	165179.3	192998.4	190096.8	200234.7	122826.1	131642.0	123700.4	118982.3
Q9DCE5	p21-activated protein kinase-interacting protein 1	Pak1ip1	382	42.1	8.47	226155.9	240362.8	205603.5	206399.0	211999.5	210413.9	203991.9	212589.3	221175.2	227260.6	314074.8	311323.0	339770.0	308233.3
E9PX68	FHA domain-containing protein	Slc4a1ap	744	82.8	5.03	142555.1	181852.8	158025.3	155912.5	171549.4	166001.7	134088.1	151514.7	154698.4	174215.4	348685.1	378968.3	399327.2	365200.8
Q63918	Caveolae-associated protein 2	Cavin2	418	46.7	5.21	424953.2	370136.8	421099.1	344680.1	601186.8	504379.5	695096.5	480200.2	425650.8	460564.1	249887.2	199076.1	227934.4	207180.7
Q3UMU9	Hepatoma-derived growth factor-related protein 2	Hdgfl2	669	74.2	8.66	70377.4	124445.1	84692.1	135021.1	77061.0	79036.8	117258.5	67555.4	113311.0	131461.4	93621.9	129134.4	95439.4	124436.7
Q61239	Protein farnesyltransferase/geranylgeranyltransferase type-1 subunit alpha	Fnta	377	44	4.93	286337.1	318529.1	304671.5	306766.8	307940.7	291026.2	287031.3	294952.5	321519.1	319980.6	123636.1	140248.4	128081.1	134080.6
Q8R3D1	TBC1 domain family member 13	Tbc1d13	400	46.4	5.36	140310.6	156313.9	159937.8	149849.2	153767.8	167518.1	146102.9	149848.8	156871.1	153741.7	225396.1	229282.1	221074.2	212704.5
Q91Y44	Bromodomain testis-specific protein	Brdt	956	107.2	8.97	196981.6	229598.0	199505.1	241511.7	203495.6	220730.6	167469.6	204597.5	232571.4	245679.7	352690.8	460158.9	437215.3	470497.6
Q8BWW4	La-related protein 4	Larp4	719	79.7	6.39	183949.9	216528.3	199727.7	200275.3	193223.4	192885.9	157660.3	169951.8	207689.6	209186.9	73895.5	94017.8	84750.2	76224.3
P58802	TBC1 domain family member 10A	Tbc1d10a	500	56.2	7.85	170055.9	176154.7	174448.2	169500.8	189574.5	184946.1	168148.5	173668.8	170873.1	173252.1	291874.6	302200.1	309895.2	310794.3
P35601	Replication factor C subunit 1	Rfc1	1131	125.9	9.33	157850.2	149855.5	156505.1	155201.4	166354.4	163529.2	141430.9	142556.0	157800.1	154443.8	63142.5	71428.7	70774.4	76254.8
Q9WUN2	Serine/threonine-protein kinase TBK1	Tbk1	729	83.4	6.87	77724.2	76324.6	79433.3	69540.9	90163.8	92275.5	73963.3	80760.1	79943.2	78686.5	95939.5	109843.5	95968.9	93320.8
Q8BGA5	KRR1 small subunit processome component homolog	Krr1	380	43.5	9.79	774148.5	769860.8	851065.5	750494.1	898684.5	873235.8	822139.6	821879.0	839518.7	858790.2	400626.8	381313.9	385478.8	384621.0
Q14AX6	Cyclin-dependent kinase 12	Cdk12	1484	163.6	9.47	145180.2	154459.8	130881.0	135100.1	136307.1	139080.6	142014.2	124109.7	133060.7	141202.4	84278.7	83232.4	83043.9	89321.2
Q9R0U0	Serine/arginine-rich splicing factor 10	Srsf10	262	31.3	11.27	359246.1	376990.0	423431.4	394526.9	441790.6	401782.7	372300.2	364493.1	403077.5	392117.0	320050.7	365618.6	330159.3	313774.2
Q9EQ00	Ropporin-1-like protein	Ropn1l	218	24.5	6.34	320257.5	489093.3	545517.8	701835.7	309293.3	253888.1	238783.2	302982.7	373322.8	387344.8	138807.2	208951.7	209098.7	268552.0
Q08093	Calponin-2	Cnn2	305	33.1	7.62	357224.9	294342.0	340443.3	301970.1	316686.4	331337.5	339462.4	352697.3	319138.6	329284.6	471259.7	484619.2	509744.9	509715.8
Q8CDM8	Protein FAM160B1	Fam160b1	764	86	5.25	226613.9	220590.5	237648.4	226296.8	236664.0	234974.0	231371.6	216657.1	219754.1	219170.7	364816.4	332447.5	343504.4	371632.5
G3X9J0	Signal-induced proliferation-associated 1-like protein 3	Sipa1l3	1776	194.9	8.32	342434.9	331703.9	294753.3	307729.5	287164.0	286308.1	277825.3	285163.1	315790.8	323502.9	229011.5	239236.6	210601.6	233648.5
Q69ZQ2	Pre-mRNA-splicing factor ISY1 homolog	Isy1	285	33	5.17	137746.9	165461.5	145547.2	155868.8	162406.7	159819.4	168699.5	131503.4	161400.5	168027.6	118804.0	132160.7	135471.8	126652.3
Q9QZ06	Toll-interacting protein	Tollip	274	30.3	5.17	246481.1	270738.3	235760.7	256847.0	243288.3	244536.1	221057.6	238095.9	245273.0	257093.0	358518.2	340134.0	331646.3	349807.1
Q9JK38	Glucosamine 6-phosphate N-acetyltransferase	Gnpnat1	184	20.8	7.69	129033.2	147042.9	147522.2	151031.6	142436.8	128494.9	127936.2	135433.1	150115.1	151707.0	51305.0	58250.7	44169.3	46597.2
Q8R1S0	Ubiquinone biosynthesis monooxygenase COQ6, mitochondrial	Coq6	476	51.4	7.17	223961.1	231964.3	237783.6	237324.8	244222.8	237404.4	230031.6	217742.6	242217.9	234048.4	277638.9	281945.2	278546.3	285235.7
P19253	60S ribosomal protein L13a	Rpl13a	203	23.4	11.02	749261.5	1034941.4	943498.7	821581.9	791774.9	753606.9	599642.5	843165.2	814640.2	849418.7	1440675.5	1659905.8	1476714.1	1656992.7
O89053	Coronin-1A	Coro1a	461	51	6.48	214627.0	209726.9	159351.4	210455.8	171615.6	192228.9	189360.9	190049.6	193842.4	198351.0	406696.3	380510.9	319844.2	389205.4
Q8BVU0	DISP complex protein LRCH3	Lrch3	778	86.3	7.03	170029.0	196585.0	186948.1	185114.8	163621.8	172633.3	173465.9	165985.7	165472.0	184134.8	274270.4	275538.2	267103.4	314754.0
P62900	60S ribosomal protein L31	Rpl31	125	14.5	10.54	770948.1	1092837.4	656926.1	982106.1	1064213.8	873387.0	605695.8	835777.1	806962.7	977725.6	1031926.6	1445689.0	1025791.8	869733.0
P97930	Thymidylate kinase	Dtymk	212	23.9	7.9	398773.1	400819.4	407149.5	411827.3	374339.7	350028.9	351484.3	382157.0	379527.1	394422.9	654284.5	747364.3	697287.0	808147.2
Q80XL6	Acyl-CoA dehydrogenase family member 11	Acad11	779	87.3	8.43	235768.3	233716.6	230885.2	234531.7	229510.3	221852.8	218752.4	228802.0	230622.2	236327.2	628107.3	723533.3	602863.0	656266.4
Q9QUR7	Peptidyl-prolyl cis-trans isomerase NIMA-interacting 1	Pin1	165	18.4	8.79	568983.6	532937.0	584706.2	555690.9	543056.4	498987.2	472186.7	517513.7	565532.4	574075.0	448667.4	456986.6	473576.7	446365.5
Q8BU31	Ras-related protein Rap-2c	Rap2c	183	20.7	4.94	122245.2	115583.0	115679.6	116153.7	129149.0	129244.4	120572.8	117358.7	116319.6	122988.7	56539.5	55470.4	48205.3	65151.4
Q6P3A8	2-oxoisovalerate dehydrogenase subunit beta, mitochondrial	Bckdhb	390	42.9	6.7	169293.6	152131.4	130620.5	126337.4	139785.5	142939.4	189093.7	153217.0	140431.8	147210.6	99610.6	107932.9	94879.9	99008.9
Q8C0E3	E3 ubiquitin-protein ligase TRIM47	Trim47	641	69.9	6.4	134065.5	118252.9	128715.6	110715.4	133539.2	131444.1	131492.3	138232.8	127830.6	115365.4	74276.6	72686.0	75504.5	77553.1
Q8VC88	Grancalcin	Gca	220	24.6	5.07	674432.5	726242.3	672958.6	698256.5	667616.9	590760.8	585068.1	748953.2	658406.5	667872.6	1129975.4	1048384.0	1071239.6	1124358.9
O55128	Histone deacetylase complex subunit SAP18	Sap18	153	17.6	9.35	54379.0	54484.2	51623.9	51805.5	54365.4	54121.9	50207.8	51490.0	51809.4	53673.2	348952.5	348041.5	390385.1	348212.8
Q9JLQ2	ARF GTPase-activating protein GIT2	Git2	708	78.7	7.72	18299.9	26513.2	23580.5	18473.3	22978.8	24707.3	19032.1	20819.3	21700.5	27344.0	50830.9	61281.2	48703.9	54877.7
Q8BH58	TIP41-like protein	Tiprl	271	31.2	5.63	64174.5	63411.6	62726.0	62648.5	59534.7	66384.6	63693.9	75077.2	58627.3	55379.7	101885.3	110451.1	112253.6	118295.8
Q8BQ47	Protein canopy homolog 4	Cnpy4	245	28.1	4.77	97163.8	109406.2	89739.1	95068.0	122369.8	111565.6	107919.9	104476.1	114531.2	108771.0	332060.6	341468.0	285394.5	301555.8
P53996	Cellular nucleic acid-binding protein	Cnbp	178	19.6	7.58	41796.5	66043.3	41167.2	48804.8	56493.0	43297.4	35750.7	42296.9	44130.6	54000.7	30927.5	64662.6	61061.3	43180.0
Q9CWL8	Beta-catenin-like protein 1	Ctnnbl1	563	64.9	5.07	258995.0	264447.8	270678.1	272008.3	273679.6	254735.3	234326.1	236928.1	259315.3	268469.5	496686.3	504292.1	481694.2	505639.8
Q9WUU9	Germinal-center associated nuclear protein	Mcm3ap	1971	217.2	6.61	110076.6	115001.3	93806.3	99941.9	115744.4	111247.1	102199.7	107586.2	112844.4	110381.4	246787.7	271641.9	239397.2	266613.3
Q9D666	SUN domain-containing protein 1	Sun1	913	101.9	6.81	74110.0	67336.1	61512.1	67337.8	66131.3	74300.9	69526.9	65676.2	74769.3	71450.6	130241.8	127972.5	142304.3	133533.5
Q8C3P7	N6-adenosine-methyltransferase subunit METTL3	Mettl3	580	64.6	6.49	111170.3	105496.9	110047.6	103992.0	108339.4	113168.9	102804.1	106267.6	100818.0	107437.0	105837.7	118656.6	104950.9	121753.1
Q5SVR0	TBC1 domain family member 9B	Tbc1d9b	1263	141.7	5.29	233754.8	250718.7	222000.5	243160.7	246597.3	265375.9	236630.8	235290.1	228649.7	238156.8	456347.4	436737.6	398652.1	418141.9
Q8R2R3	Alpha- and gamma-adaptin-binding protein p34	Aagab	316	34.5	4.64	57043.3	50680.5	49535.9	40515.1	50967.0	52484.3	46900.6	43608.1	48222.8	52581.5	104783.8	162352.0	108110.0	166116.6
Q3UX10	Tubulin alpha chain-like 3	Tubal3	446	50	5.58	44712.9	47300.8	39157.3	41114.1	43912.2	42333.8	43280.5	46998.1	51313.4	42448.9	24017.0	22607.4	22242.0	21481.3
P63034	Cytohesin-2	Cyth2	400	46.6	5.63	146408.3	137850.6	132805.0	139824.5	148337.6	129997.7	124428.7	162894.4	147735.5	144728.4	84763.4	84506.3	82875.2	92286.8
Q925J9	Mediator of RNA polymerase II transcription subunit 1	Med1	1575	167	8.73	120732.3	124926.3	113887.4	118356.9	123567.3	119812.5	107626.4	106405.5	124209.8	129632.2	185114.3	188605.3	186983.0	185701.2
P29416	Beta-hexosaminidase subunit alpha	Hexa	528	60.6	6.54	173769.4	194078.6	164225.4	192169.6	169013.3	190502.7	184867.2	167887.5	172802.3	180750.3	119262.2	189589.0	140251.4	158159.2
Q3TKR3	NACHT, LRR and PYD domains-containing protein 4C	Nlrp4c	982	112.6	6.92	248600.5	242003.4	245871.4	208931.0	272808.3	278772.0	284052.2	237140.9	254091.7	258006.9	529713.8	512734.5	542028.3	493004.6
P18872	Guanine nucleotide-binding protein G(o) subunit alpha	Gnao1	354	40.1	5.53	74219.4	82307.3	70523.9	84483.7	68265.0	68770.1	66944.2	71159.0	71714.0	74810.3	128572.1	130579.2	130452.4	151201.2
Q8JZP9	GAS2-like protein 1	Gas2l1	678	72.4	10.01	210834.8	235016.8	193033.8	209084.1	196858.8	190272.6	186196.5	189747.8	213817.2	220744.6	90217.3	97749.0	76147.4	95067.9
P27786	Steroid 17-alpha-hydroxylase/17,20 lyase	Cyp17a1	507	57.6	7.18	267794.2	238441.0	127895.0	418520.8	116963.7	136677.0	121679.9	148395.8	144080.7	148911.0	241748.4	200453.2	112397.6	316492.2
Q9DBS1	Transmembrane protein 43	Tmem43	400	44.8	7.36	131581.0	134539.1	159881.7	132746.1	157269.2	162514.7	140316.5	150618.6	153012.3	137306.1	255849.7	242589.9	297838.9	234875.5
B2RT41	Zinc finger, C3H1-type-containing	Zfc3h1	1992	224.9	8.15	140710.7	137438.8	133569.7	132440.0	149364.2	153660.2	125679.0	131715.1	134357.0	145978.1	109839.0	113585.7	121092.8	119569.8
Q9JI39	ATP-binding cassette sub-family B member 10, mitochondrial	Abcb10	715	77.1	9.6	330422.8	303916.3	297767.0	312046.1	301239.0	295649.8	293296.8	307131.0	322937.3	297830.6	363289.7	328941.8	289224.6	328506.9
Q05186	Reticulocalbin-1	Rcn1	325	38.1	4.84	213401.8	190939.0	160961.9	176619.8	200846.1	232733.4	208202.5	172825.7	191645.8	199196.3	556200.6	501500.6	383439.3	546250.0
Q8BLF1	Neutral cholesterol ester hydrolase 1	Nceh1	408	45.7	7.05	103216.8	132310.6	98302.2	104129.7	118286.3	144491.4	121901.9	124687.4	112301.4	112090.2	69269.8	68968.7	65821.6	70824.8
Q9Z0S1	3'(2'),5'-bisphosphate nucleotidase 1	Bpnt1	308	33.2	5.82	206379.5	194258.2	184324.3	216418.0	177937.8	187395.6	192033.5	175999.3	203492.6	196153.1	207285.0	191275.9	182967.1	183808.6
P24452	Macrophage-capping protein	Capg	352	39.2	7.2	434351.7	377038.3	347307.9	341073.5	365664.0	452693.7	581897.6	462246.6	374612.4	355678.0	108082.4	104655.6	90017.6	95816.7
Q9DBB4	N-alpha-acetyltransferase 16, NatA auxiliary subunit	Naa16	864	101.2	8.35	100283.2	98001.4	109295.1	96969.5	103633.9	119366.3	94705.4	152570.5	107329.4	107627.0	123457.9	112618.2	117055.8	100713.9
Q4VAA2	Protein CDV3	Cdv3	281	29.7	6.1	128749.8	135657.0	121353.6	115578.1	144777.6	129421.5	130125.2	119073.0	126283.5	130665.1	534850.9	512657.3	532709.4	494908.5
Q8R313	Exocyst complex component 6	Exoc6	802	93	6.15	249479.3	227468.5	214681.7	218690.3	242061.1	234715.1	234024.2	235758.8	234892.2	230887.0	163217.8	193105.1	151037.6	169906.5
Q8BHG2	CXXC motif containing zinc binding protein	Czib	160	18	5.12	407801.2	415940.8	407546.1	436308.2	399494.7	419990.8	391726.2	403748.8	431885.6	423933.8	428627.8	452606.2	472115.0	470537.3
Q9DC29	ATP-binding cassette sub-family B member 6, mitochondrial	Abcb6	842	93.7	8.05	141922.0	125221.8	151443.6	150567.1	135588.9	137491.4	125896.0	138086.0	131139.9	138707.5	60921.9	46717.7	54881.8	54237.6

Q8R3N1	Nucleolar protein 14	Nop14	860	98.7	7.59	139767.7	142615.8	119873.5	123019.2	143822.0	141585.1	123246.4	124183.0	125220.7	135221.2	63398.4	68262.6	56849.0	54451.6
Q61037	Tuberin	Tsc2	1814	201.9	6.98	139686.8	143662.1	126374.7	131829.7	131128.3	131799.9	132035.6	133426.3	137418.4	139998.3	55867.8	77167.2	55229.2	50252.8
Q8VE47	Ubiquitin-like modifier-activating enzyme 5	Uba5	403	44.8	4.96	195009.7	207875.2	180710.6	195519.6	191816.9	194625.1	185775.0	198600.1	189900.0	189044.6	133839.0	109979.3	106240.7	114228.9
P63321	Ras-related protein Ral-A	Rala	206	23.5	7.11	345640.4	362767.5	311716.2	337362.9	362160.3	322657.6	338411.5	299848.4	348808.5	347215.4	365306.1	442812.7	341298.8	393335.2
Q8BJU0	Small glutamine-rich tetratricopeptide repeat-containing protein alpha	Sgta	315	34.3	5.06	262095.0	297554.4	285707.2	305628.9	255333.4	276262.8	280626.3	261412.0	282434.1	281603.0	206117.4	212018.4	193913.9	226343.3
Q925B0	PRKC apoptosis WT1 regulator protein	Pawr	333	35.9	5.92	89378.3	94940.3	94857.5	93411.7	99075.4	100765.3	126325.7	97152.3	92090.3	94576.3	531625.7	234324.1	236015.0	253897.5
Q9EQI8	39S ribosomal protein L46, mitochondrial	Mrpl46	283	32.1	7.4	250487.6	230363.5	222129.7	229055.3	236856.2	232140.6	240899.8	230600.5	250933.8	241644.5	78701.3	81978.1	79372.5	83393.0
A7UAK5	6PF2K domain-containing protein	Pfkfb3	555	63.6	8.25	191286.2	185368.5	187487.8	181589.1	217647.7	210957.9	236138.7	193150.2	202104.7	195400.1	142185.1	145734.6	154145.4	137685.2
Q62036	Centrosomal protein of 131 kDa	Cep131	1060	120.2	8.72	131662.3	143750.7	131014.3	141276.6	130258.4	135118.2	138048.7	129407.3	134269.9	129659.8	201893.5	193497.9	208489.0	204408.4
Q8CGC4	Protein LSM14 homolog B	Lsm14b	385	42.3	9.63	79550.4	101925.0	102195.7	107999.1	93567.8	87438.2	89005.6	82951.7	97653.5	100521.0	457504.8	494059.5	504718.4	494561.9
Q8CIV8	Tubulin-specific chaperone E	Tbce	524	59	6.29	81274.2	85516.3	82064.7	86956.3	88057.4	87423.7	76450.8	81899.1	86185.5	85512.8	102578.3	118148.2	107822.9	105866.0
Q8K1E0	Syntaxin-5	Stx5	355	39.7	8.92	141088.8	171654.8	138404.1	144747.0	138130.4	139875.5	118020.3	117125.0	142602.0	155772.9	88085.8	92269.6	87078.9	92830.7
Q9D2H9	Dynein assembly factor 1, axonemal	Dnaaf1	634	69.7	4.67	110985.2	139299.4	132326.6	157540.7	100929.7	97082.9	100467.9	104571.6	120128.6	115948.0	114806.5	164793.7	204640.1	280894.1
Q03141	MAP/microtubule affinity-regulating kinase 3	Mark3	753	84.3	9.51	242181.8	212458.3	272688.2	215814.0	263295.1	258236.8	221907.2	230998.8	261987.9	249506.6	223502.5	242612.0	212277.3	207451.8
Q56A08	G-patch domain and KOW motifs-containing protein	Gpkow	488	53.8	8.07	206018.8	217996.7	218245.5	208805.0	228476.7	239875.5	208318.1	193474.8	213627.3	232673.1	417052.0	404043.7	477754.4	424132.9
Q8K021	Secretory carrier-associated membrane protein 1	Scamp1	338	38	7.71	191359.0	210431.9	184933.0	203276.7	195147.1	191768.2	222864.4	183031.4	194933.4	188601.9	280790.6	286399.3	263006.5	277321.0
Q9D338	39S ribosomal protein L19, mitochondrial	Mrpl19	292	33.6	9.44	88037.4	122362.5	96390.7	92596.7	104076.3	99667.3	94076.8	109024.8	95125.8	105527.2	131730.9	118154.9	130709.1	129440.0
D3Z6P9	DEAD (Asp-Glu-Ala-Asp) box polypeptide 43	Ddx43	646	72.2	9.11	212080.6	240255.6	219671.9	259688.1	204931.3	213686.6	198513.6	182016.7	225829.2	245816.6	156519.1	189898.9	181189.2	202729.3
P62488	DNA-directed RNA polymerase II subunit RPB7	Polr2g	172	19.3	5.54	371411.4	397076.2	393652.1	373312.1	408574.9	403203.8	416117.4	362971.6	400083.4	407521.9	397353.7	382091.1	444049.8	388686.1
Q921J2	GTP-binding protein Rheb	Rheb	184	20.4	5.92	548165.9	580913.7	596047.7	591302.3	663830.3	626466.2	557573.9	533178.0	622685.0	599746.8	289254.8	313292.8	322619.4	308126.0
Q8BPY9	Fidgetin-like protein 1	Fignl1	683	74.8	6.44	227376.8	314502.2	246835.1	259716.5	249706.9	261593.5	185173.0	213294.8	277405.3	260024.8	120155.9	123043.4	128253.2	109983.3
P46412	Glutathione peroxidase 3	Gpx3	226	25.4	8.22	221022.9	225111.9	206783.7	194824.8	251871.0	271485.0	308155.5	221769.4	211305.8	220642.9	184309.5	185006.4	180906.0	181693.9
Q80TA9	Ectopic P granules protein 5 homolog	Epg5	2572	290.6	6.37	64905.6	74732.1	58751.7	57513.2	71778.6	71563.9	67073.7	57327.9	62706.0	70373.3	110976.4	106308.6	93621.9	105495.1
Q8VE70	Programmed cell death protein 10	Pdcd10	212	24.7	8.19	567641.3	556659.2	550759.2	528178.0	526114.4	508340.7	567678.8	526336.1	558439.9	534899.6	478330.0	567423.7	537902.5	551845.3
P60330	Separin	Espl1	2118	232.9	6.9	143784.5	161581.1	136792.1	151480.7	157669.1	145265.3	126720.0	132379.9	148085.4	130516.8	79917.6	72036.6	82028.6	84515.4
P70271	PDZ and LIM domain protein 4	Pdlim4	330	35.5	8.05	81753.1	85070.1	76638.1	85890.0	86894.5	92647.6	86638.7	86490.9	83377.2	82752.9	62605.0	64214.6	70816.9	49984.0
Q9Z0H1	WD repeat-containing protein 46	Wdr46	622	69	9.77	205264.2	205640.8	206717.2	172066.1	211601.9	221159.2	186119.8	200930.2	189743.4	191520.5	183028.5	209033.1	184138.7	186637.0
Q9EQS3	c-Myc-binding protein	Mycbp	103	12	5.91	473693.2	565128.8	663338.0	593036.4	604276.6	520213.9	494137.0	577576.8	630304.2	550237.1	194923.9	205007.5	209882.9	216464.6
P01865	Ig gamma-2A chain C region, membrane-bound form	Igh-1a	398	43.9	6.29	385550.7	208917.8	162454.0	161560.8	188657.7	222670.0	318871.2	387253.3	171516.2	150940.9	416736.6	267982.9	259587.6	197609.6
Q2TBE6	Phosphatidylinositol 4-kinase type 2-alpha	Pi4k2a	479	54.2	8.05	145296.8	150643.5	151746.7	126678.4	160913.2	163810.9	146029.7	138033.2	140137.1	134016.8	184213.9	196321.4	190154.8	183614.6
Q8R332	Nucleoporin p58/p45	Nup58	587	59.4	9.29	156576.8	175319.5	170975.4	179220.1	186745.0	177314.8	158138.5	148732.4	158508.8	178308.5	97822.3	133816.6	102815.5	110696.1
P02772	Alpha-fetoprotein	Afp	605	67.3	5.92	114228.9	112418.7	83269.7	79204.5	124991.7	136634.8	77866.5	165894.7	92991.2	112496.4	99086.7	94203.7	88803.6	73944.5
Q9CPX6	Ubiquitin-like-conjugating enzyme ATG3	Atg3	314	35.8	4.72	310408.3	329034.3	355106.8	350473.7	316114.6	334536.0	269675.9	327651.4	330897.5	309963.1	114074.3	139557.3	133382.8	146677.4
Q8R5K4	Nucleolar protein 6	Nol6	1152	129.1	6.79	136480.2	168373.0	138263.6	139182.4	171850.0	156830.8	123441.4	138405.3	134773.8	145952.5	209249.7	207119.3	161770.7	197389.4
Q7TPS5	C2 domain-containing protein 5	C2cd5	1016	111.6	5.78	380782.5	519495.6	402995.4	401018.9	433484.8	388822.8	332424.3	313036.7	447992.5	494036.5	76040.2	83954.7	88713.8	90096.3
Q61102	ATP-binding cassette sub-family B member 7, mitochondrial	Abcb7	752	82.5	9.32	94760.8	99232.8	98909.6	98466.7	108079.4	111946.5	85583.1	98992.3	101488.4	103626.0	100242.3	112197.7	90484.3	99177.5
Q06180	Tyrosine-protein phosphatase non-receptor type 2	Ptpn2	406	47.3	8.37	132331.7	154322.9	132504.9	148263.7	147363.5	134346.0	128870.7	126143.6	130491.1	138781.5	203474.8	232818.9	236224.5	218910.3
Q8JZN7	Mitochondrial Rho GTPase 2	Rhot2	620	69	6	91977.1	110799.2	131800.4	103732.3	109363.2	140850.0	115709.8	117576.3	109899.9	101734.4	144834.7	151039.1	134741.5	135220.8
Q9Z129	ATP-dependent DNA helicase Q1	Recql	648	72.4	8.34	268161.9	282800.7	282464.1	275861.9	315649.6	302783.0	314675.9	275675.7	317391.8	298813.4	212586.8	237269.8	228125.4	209536.8
P58404	Striatin-4	Strn4	760	81.6	5.38	53736.6	43348.0	47139.1	48602.7	53526.1	46602.6	45659.2	47112.7	45550.5	47886.6	84337.7	89503.4	94653.4	89100.7
Q9JJA2	Conserved oligomeric Golgi complex subunit 8	Cog8	640	71.6	5.19	266893.5	237538.3	268338.2	277477.5	263688.9	261996.0	244148.6	233602.7	254317.9	242598.9	157089.7	193247.6	189959.8	201571.4
Q99J47	Dehydrogenase/reductase SDR family member 7B	Dhrs7b	323	35	9.63	305986.0	330731.6	238450.2	276182.8	277514.0	254575.4	255865.0	236468.9	266824.7	286098.4	145118.2	138453.3	123193.7	140148.7
Q9D0W5	Peptidyl-prolyl cis-trans isomerase-like 1	Ppil1	166	18.2	7.99	312897.2	281691.6	331256.4	290803.6	346043.6	311419.9	325501.1	305146.7	311586.6	334930.0	374473.1	356027.8	401066.2	342933.0
Q8C092	Transcription initiation factor TFIID subunit 5	Taf5	801	87	5.64	120778.6	128237.0	123259.8	130977.4	125984.9	130587.4	131867.8	122290.7	137422.0	135335.0	270471.4	288509.2	286813.8	284362.7
P55258	Ras-related protein Rab-8A	Rab8a	207	23.7	9.07	387286.6	416804.2	417668.1	446299.2	417380.3	422206.6	426941.2	424091.6	396054.2	389155.1	843269.5	954185.3	925211.1	1047560.2
E9PWG6	Cnd3 domain-containing protein	Ncapg	1004	112.8	5.55	226783.9	237764.5	257019.6	211936.0	212649.3	221048.1	211112.9	231217.8	216752.9	222229.0	179481.5	181628.1	172386.6	168487.7
E0CZ22	Maestro heat-like repeat family member 1	Mroh1	1640	181.7	6.39	182724.3	219057.0	204106.9	199330.2	211905.1	204835.4	189736.1	177543.7	207042.5	220274.1	117028.0	133756.4	145414.0	121860.4
Q6NXH9	Keratin, type II cytoskeletal 73	Krt73	539	58.9	8.09	751978.8	346396.5	348603.1	404836.0	513698.3	533249.5	429919.5	404942.4	440919.1	488664.0	501888.8	291521.6	284285.1	338147.0
P24668	Cation-dependent mannose-6-phosphate receptor	M6pr	278	31.2	5.39	388058.9	465530.5	359766.2	385635.4	409438.7	389612.7	392391.6	376144.8	405990.5	412553.1	243275.9	242562.3	280030.3	237013.1
Q9ER69	Pre-mRNA-splicing regulator WTAP	Wtap	396	44.2	5.25	138719.1	138856.3	138752.0	138640.5	153633.2	145909.6	141582.6	133664.1	143148.1	137298.8	121485.7	122209.9	120549.5	118608.2
Q8K2I3	Dimethylaniline monooxygenase [N-oxide-forming] 2	Fmo2	535	60.9	8.47	235344.7	209357.3	201404.7	204682.3	250004.1	347404.8	455810.2	285302.5	231773.2	212774.9	194892.6	211239.9	182397.0	193770.2
P70404	Isocitrate dehydrogenase [NAD] subunit gamma 1, mitochondrial	Idh3g	393	42.8	9.01	253129.6	347272.4	237541.5	249018.3	257317.0	233089.2	245794.8	233680.7	255770.9	241474.1	649264.3	734756.9	688204.1	669497.8
Q9WU42	Nuclear receptor corepressor 2	Ncor2	2472	269.6	7.44	167487.2	167925.0	170897.1	169310.9	183623.2	188888.9	192813.0	184036.0	172979.2	186185.1	64062.5	59763.0	61780.9	63889.1
Q8BFR4	N-acetylglucosamine-6-sulfatase	Gns	544	61.1	8.24	638901.6	677042.3	641989.1	716694.9	619322.9	609986.6	678459.0	600883.2	659273.1	634374.3	655061.8	718993.5	626438.5	706498.9
Q9QXM1	Junction-mediating and -regulatory protein	Jmy	983	110.5	6.13	171361.3	181523.8	174530.2	175905.0	167643.5	162553.8	157526.7	166445.8	176040.6	173903.8	181526.9	205412.9	150099.0	168879.7
Q5U430	E3 ubiquitin-protein ligase UBR3	Ubr3	1889	212.6	5.9	182381.8	189280.2	172538.8	171669.3	201372.8	196517.7	181819.1	163934.9	175067.1	179205.5	78457.1	93506.0	82461.8	77873.0
Q80XS6	Protein Smaug homolog 2	Samd4b	687	75	6.74	154212.4	179540.9	169532.3	175489.3	182839.6	166087.3	154665.3	147922.6	157577.1	163812.0	108107.9	99060.3	98926.2	92464.9
Q6PHN9	Ras-related protein Rab-35	Rab35	201	23	8.29	223247.2	216961.3	245618.2	188101.5	268052.8	279000.2	242236.6	238575.8	211200.9	231098.5	176737.9	186167.7	158107.9	156552.1
Q9JKY7	Cytochrome P450 CYP2D22	Cyp2d22	500	56.4	6.98	178292.1	165631.4	142454.5	162629.6	176019.6	172332.5	229505.0	159719.8	161806.8	165763.9	71851.3	85690.8	93639.9	86532.9
A2A484	Zinc finger, MYND-type-containing 8	Zmynd8	1235	136.9	7.52	41267.9	36214.7	39177.9	39218.8	39819.4	48869.0	44523.8	43075.5	40893.5	42015.7	97425.4	86358.9	111256.5	95783.3
Q8K2T1	NmrA-like family domain-containing protein 1	Nmral1	309	34.4	6.86	311367.7	309479.2	302469.0	288005.1	314204.2	317711.1	296151.3	299079.5	317328.2	309300.4	168380.4	170388.8	145531.5	151580.4
O35594	Intraflagellar transport protein 81 homolog	Ift81	676	79.2	8.75	97789.7	153101.6	112128.5	143838.1	109516.2	113094.4	87226.5	107588.7	115812.8	127389.0	113360.6	134450.4	122063.0	126381.3
Q6DFV5	Probable helicase with zinc finger domain	Helz	1964	219.7	7.4	217136.5	208868.6	215306.3	203540.0	216746.8	209588.7	201573.2	197611.2	221096.5	218053.2	88678.4	89621.0	86035.2	89164.2
Q99JR8	SWI/SNF-related matrix-associated actin-dependent regulator of chromatin subfamily D member 2	Smarcd2	531	59	9.64	348508.6	338760.5	360267.9	319163.1	392625.8	392651.7	348015.6	331491.5	356165.0	354772.8	397277.5	405935.5	371369.8	397768.4
Q8R3C6	Probable RNA-binding protein 19	Rbm19	952	106	6.57	33271.9	32037.6	34402.3	28377.7	30636.1	38591.9	31468.2	29781.7	29276.7	34720.5	97185.3	108029.9	94591.2	99025.9
Q8K2K6	Arf-GAP domain and FG repeat-containing protein 1	Agfg1	561	58	8.63	460967.4	524917.5	442704.6	483404.4	408931.0	405267.5	356450.9	404815.3	480040.9	515243.1	124475.8	132423.7	121223.1	125087.2
Q60575	Kinesin-like protein KIF1B	Kif1b	1816	204	5.62	190459.4	175115.4	182743.5	168369.5	199731.9	200948.9	178347.5	181819.7	172498.3	192011.1	133108.3	155690.6	157026.6	160988.5
D3YUJ3	CYCLIN domain-containing protein	Ccnyl1	367	41.6	6.34	393832.7	475724.8	485783.7	482956.7	410355.1	401549.3	357863.6	373291.0	448185.4	464192.6	345558.2	424482.0	408677.9	408510.3
Q9JLB2	MAGUK p55 subfamily member 5	Mpp5	675	77.2	6.09	110782.6	113966.7	107028.8	115936.8	125593.5	127140.2	106152.1	101009.3	110085.4	106811.8	86382.8	90749.8	86114.3	85203.2
Q8CHD8	Rab11 family-interacting protein 3	Rab11fip3	1047	118.5	4.39	162913.6	151944.0	157065.5	151622.7	140957.6	157660.8	134720.0	156847.8	149241.3	150171.3	169377.7	147922.6	145223.8	150034.4
Q8BKX1	Brain-specific angiogenesis inhibitor 1-associated protein 2	Baiap2	535	59.2	9.04	153681.4	187297.8	193475.7	186901.8	194779.3	211138.6	163162.3	168382.2	192632.6	195472.7	62658.8	84008.1	75755.1	96727.3
Q6DID5	PWWP domain-containing DNA repair factor 3A	Pwwp3a	682	76	8.46	70234.5	80087.6	90820.0	87019.3	69468.6	52881.8	49387.1	63096.2	64991.5	76477.4	323197.4	366328.1	352722.6	393043.6
P58021	Transmembrane 9 superfamily member 2	Tm9sf2	662	75.3	7.43	287768.9	299588.6	310367.7	327409.4	282019.4	286308.0	255597.2	279852.6	290803.7	300817.2	230318.4	247094.9	222421.7	266821.1
Q8VCT3	Aminopeptidase B	Rnpep	650	72.4	5.35	124690.4	113862.5	91303.9	142588.2	108536.6	106056.5	107599.3	142273.7	94129.4	102615.2	208980.5	236801.5	174264.5	225477.4
Q9CQ92	Mitochondrial fission 1 protein	Fis1	152	17	8.53	271238.0	270680.3	276503.0	293490.4	277221.7	261534.0	251924.3	272563.3	264738.2	263990.4	198562.3	209455.2	203885.2	217118.1
Q8C5N3	Pre-mRNA-splicing factor CWC22 homolog	Cwc22	908	104.7	8.09	192037.2	197780.1	200371.3	206321.4	203642.3	201161.2	181131.7	190172.1	206778.5	210587.7	255273.5	228417.5	253225.1	238743.5
Q6A065	Centrosomal protein of 170 kDa	Cep170	1588	174.9	7.17	191635.2	209919.5	195492.4	195643.9	210493.0	205678.2	191572.0	185920.0	206362.5	203728.7	119536.0	131837.1	124573.4	124164.3
P61028	Ras-related protein Rab-8B	Rab8b	207	23.6	9.07	195474.3	206015.7	198591.1	204017.1	186487.3	187349.6	175605.5	177962.1	195609.9	200160.9	45015.7	56130.3	52108.3	49301.3
Q9D483	DNA-directed RNA polymerase III subunit RPC3	Polr3c	533	60.7	7.58	103155.2	104773.7	97443.7	102886.7	114716.8	118357.5	102472.0	109659.1	102968.9	105089.0	72057.0	76019.7	73027.9	74209.1
Q7TMQ7	WD repeat-containing protein 91	Wdr91	748	83.4	6.64	175912.0	189828.3	160799.1	181988.1	166529.2	175622.8	166768.4	162564.5	159768.5	160345.0	164539.7	187510.4	148751.1	175026.0
Q78PG9	Coiled-coil domain-containing protein 25	Ccdc25	208	24.5	6.95	198867.7	184395.0	190257.1	180903.8	188035.0	178641.8	218317.9	203313.7	190068.2	200315.4	625464.0	665620.2	622094.4	631528.0
Q8R238	Serine dehydratase-like	Sdsl	329	34.7	6.43	96042.6	179774.2	80565.3	98958.0	115986.8	139612.5	92710.1	118763.2	107203.2	110101.1	91786.5	92863.5	91405.1	89749.5
Q9DCV4	Regulator of microtubule dynamics protein 1	Rmdn1	305	35	8.7	327170.5	397089.3	342229.2	386113.0	376291.7	363327.4	370117.2	360947.3	341423.5	379048.3	155140.7	161268.5	144005.4	167371.5
Q9CWP4	Lymphocyte antigen 6K	Ly6k	154	17.1	8.13	99723.1	126431.4	119494.4	122141.4	113719.4	107939.8	83437.7	94986.6	112367.6	118654.2	501042.8	703495.8	614905.9	677087.4
Q3UIL6	Pleckstrin homology domain-containing family A member 7	Plekha7	1118	126.7	9.39	30047.8	40439.3	34443.4	42672.9	38463.3	42513.8	38329.1	41160.3	40839.9	36201.1	256194.2	307844.9	269271.2	300690.0
Q9DAI2	Intraflagellar transport protein 22 homolog	Ift22	185	20.8	5.25	310914.7	377299.9	360367.9	428569.8	248210.9	252279.7	231099.6	266279.5	315830.0	350594.5	153550.6	192750.2	185049.7	237016.3
Q924K8	Metastasis-associated protein MTA3	Mta3	591	67	8.88	88361.0	120484.5	115494.6	110773.5	132366.7	118042.7	112432.0	117912.5	125603.2	116240.3	211721.2	224465.1	192693.8	218985.4
Q640M1	U3 small nucleolar RNA-associated protein 14 homolog A	Utp14a	767	87.2	9.2	249337.7	275634.4	233246.4	246643.6	289722.3	258328.6	247326.5	239749.5	267602.5	274028.9	254669.1	263972.7	219751.5	248706.4
Q6P1H6	Ankyrin repeat and LEM domain-containing protein 2	Ankle2	964	106.1	7.37	198221.7	194235.4	167998.9	189881.9	172629.2	167435.6	182422.3	172619.4	195741.3	188994.7	110240.0	112760.0	97320.1	100840.7
Q8BS45	Intraflagellar transport protein 56	Ttc26	554	64.1	6.8	86017.9	102578.4	98090.5	104498.9	89874.0	84800.8	75471.1	76489.3	97457.1	112104.4	216150.8	269340.9	255171.9	290911.3
Q3UFS0	Protein zyg-11 homolog B	Zyg11b	744	83.9	6.87	45929.2	41367.9	50535.0	43670.7	45005.9	48600.1	42993.8	43188.8	46332.6	47581.0	148500.4	148652.5	139544.7	142835.0
Q8VBT0	Thioredoxin-related transmembrane protein 1	Tmx1	278	31.4	5.29	166058.8	186961.8	184734.1	181773.1	189973.6	174073.6	151850.6	162167.3	170549.3	174772.2	204464.2	243769.7	224708.4	249395.6

O88792	Junctional adhesion molecule A	F11r	300	32.4	6.77	156601.5	124674.9	172226.2	113348.0	204601.5	220967.5	201086.0	172258.9	169511.1	171292.6	113693.2	114692.5	115187.4	101767.7
Q562E2	BTB/POZ domain-containing protein KCTD19	Kctd19	927	104.8	6.62	134145.8	142835.3	158231.8	155546.2	144698.1	139976.0	114543.8	123130.7	141377.0	135456.7	50448.3	64972.6	62117.4	73269.4
Q6NXL1	Sec24-related gene family, member D (S. cerevisiae)	Sec24d	1032	112.6	7.18	279318.0	280448.7	293859.5	279921.2	306983.8	312169.8	295453.8	335548.9	270154.6	278578.3	200930.1	198922.2	173144.6	194561.9
O88379	Bromodomain adjacent to zinc finger domain protein 1A	Baz1a	1555	178.3	6.54	104759.7	132314.9	106871.0	156938.6	90872.1	94383.0	79550.8	97311.5	102805.4	108712.6	157417.1	193273.7	162884.9	198803.2
A2APV2	Formin-like protein 2	Fmnl2	1086	123	7.53	172732.4	179421.3	161356.1	166968.6	206858.9	195375.1	214089.2	176795.6	182135.6	177351.5	232267.3	211444.9	194335.5	206462.2
Q9CQ26	STAM-binding protein	Stambp	424	48.5	6.64	121876.9	132783.6	125908.6	140226.4	115665.0	121855.1	112045.3	124564.6	125912.5	122705.6	249934.3	260952.5	245161.7	257540.0
Q5SSZ5	Tensin-3	Tns3	1440	155.5	6.65	143293.3	132174.4	146689.7	145054.0	136243.7	140700.0	152053.5	131104.5	138496.8	142545.3	54856.4	50440.3	49780.3	52793.6
Q61129	Complement factor I	Cfi	603	67.2	7.46	236138.6	215096.9	198353.7	214465.3	172735.6	198789.7	226114.4	204466.3	218328.4	201827.8	30059.4	33891.4	22712.3	24627.9
Q8CD15	Ribosomal oxygenase 2	Riox2	465	53.5	6.38	219116.6	202043.7	226277.0	180198.2	253833.1	256536.0	242317.8	249324.1	233816.5	219097.4	505442.5	494573.0	512667.0	475466.0
Q9CQA5	Mediator of RNA polymerase II transcription subunit 4	Med4	270	29.8	5.05	158731.7	164698.6	176657.4	165015.6	166026.5	162071.2	159426.7	147820.0	157229.3	155464.5	64309.3	72041.7	67099.3	63990.7
Q8BTZ7	Mannose-1-phosphate guanyltransferase beta	Gmppb	360	39.9	6.74	312226.8	330159.5	323809.3	320240.5	334882.5	338517.6	288410.3	312837.8	299869.7	313241.4	274287.3	300923.7	271994.4	309025.4
Q3SXD3	5'-deoxynucleotidase HDDC2	Hddc2	199	22.7	5.02	80245.9	76655.8	79724.2	84658.7	87386.1	83720.7	78324.7	73710.6	91701.0	79850.1	77069.0	87905.6	79222.1	78819.5
Q9QY53	Nephrocystin-1	Nphp1	687	77	5.25	199925.4	242373.0	201546.1	227378.2	192777.9	192815.1	183082.3	194579.9	195554.9	218974.9	162753.9	189037.8	185187.0	202454.1
Q8BXN9	Transmembrane protein 87A	Tmem87a	555	63.3	6.67	37115.3	36009.7	35644.8	40173.0	46922.1	51861.1	41890.9	42918.5	38217.0	36735.5	49434.0	54329.1	48886.5	43706.3
Q9CRA8	Exosome complex component RRP46	Exosc5	235	25.2	7.56	106176.0	132009.8	114068.9	122217.7	124862.6	134177.9	107253.2	111888.9	125882.3	113549.4	27093.8	36516.7	28098.3	32445.6
Q8R3B7	Bromodomain-containing protein 8	Brd8	951	102.5	4.72	110847.5	123721.4	128476.4	125509.9	124457.9	122251.7	102391.6	112497.3	123717.1	120964.6	114711.0	110063.6	134829.3	120894.7
Q8CDM1	ATPase family AAA domain-containing protein 2	Atad2	1040	117.9	6.89	104853.7	134716.1	116200.2	106512.0	130628.8	122203.6	105211.2	112966.9	109083.2	103257.1	162677.4	172186.4	141087.6	159269.2
A2RSJ4	UHRF1-binding protein 1-like	Uhrf1bp1l	1457	161.8	6.61	280547.5	285027.0	293133.4	280066.2	305013.5	305598.5	231356.2	266955.9	281420.6	285593.1	124625.6	149502.2	119552.4	127255.5
P58242	Acid sphingomyelinase-like phosphodiesterase 3b	Smpdl3b	456	51.6	6.43	355161.0	247961.6	287788.5	234222.0	326764.8	342746.0	373830.4	257150.1	264868.4	277377.7	182484.5	174251.3	161983.7	154944.7
Q8R404	MICOS complex subunit MIC13	Micos13	119	13.4	8.63	194474.9	215535.8	225765.1	224746.5	201558.1	210658.3	189866.8	217831.7	218648.3	232998.9	716277.4	711282.4	732629.2	737261.1
Q8CJ19	[F-actin]-monooxygenase MICAL3	Mical3	1993	223.6	5.47	177363.7	148641.3	143565.1	147344.1	151137.5	159592.2	179433.2	146400.7	153936.6	158510.7	72038.8	72485.0	72676.4	75439.5
P07309	Transthyretin	Ttr	147	15.8	6.16	1894280.4	1682030.3	1389673.3	1534166.1	710493.0	858988.1	1375256.0	1769917.1	1319652.8	1050777.5	1407400.0	1412210.1	1185492.5	1290919.1
Q3U2A8	Valine--tRNA ligase, mitochondrial	Vars2	1060	118.4	7.14	267735.3	241923.5	253661.4	234485.6	265200.6	286081.4	256692.3	275829.0	245290.3	256054.3	223289.5	220655.3	206787.1	202264.2
Q791T5	Mitochondrial carrier homolog 1	Mtch1	389	41.5	9.32	293010.2	286800.4	272309.2	282806.7	299257.1	291119.0	285642.3	328919.0	271652.6	289862.1	243228.0	255887.9	230957.4	229999.2
A2AHC3	Calmodulin-regulated spectrin-associated protein 1	Camsap1	1581	175.8	6.95	80795.1	81840.6	71261.0	73176.7	83425.5	72935.7	76698.3	72873.1	76241.5	93882.3	97131.7	92963.0	96696.4	85321.2
F8VPN4	Amylo-1,6-glucosidase, 4-alpha-glucanotransferase	Agl	1532	174.2	6.74	100044.7	109340.1	92892.8	106148.5	104180.7	99402.2	97035.6	98460.7	109317.9	107873.6	167138.9	162125.1	152499.5	162972.1
Q8BFQ4	WD repeat-containing protein 82	Wdr82	313	35.1	7.69	675783.0	650142.2	624519.4	647372.3	623839.5	652248.3	656678.6	624594.8	646496.9	664404.5	243606.2	229443.7	255356.3	246228.3
Q9CYR0	Single-stranded DNA-binding protein, mitochondrial	Ssbp1	152	17.3	9.92	302121.1	331484.4	337497.1	312131.5	360314.4	356434.4	333580.2	339776.6	351422.3	338849.3	573649.0	566885.9	584760.2	552983.0
Q3UKC1	Tax1-binding protein 1 homolog	Tax1bp1	814	93.6	5.33	208178.7	221761.0	239764.0	227508.1	226631.9	217316.8	177264.4	200165.5	217506.8	214680.0	200919.0	233303.9	227418.7	232114.7
Q8R3P6	Integrator complex subunit 14	Ints14	515	57.2	5.17	59741.8	68207.6	63889.1	65214.7	63736.8	68739.5	59771.3	58617.1	62663.0	66364.8	50307.2	39703.4	41144.6	47904.9
Q91UZ1	1-phosphatidylinositol 4,5-bisphosphate phosphodiesterase	Plcb4	1175	134.4	6.9	95594.8	94932.0	84882.5	90417.3	90040.5	96359.4	102970.6	91884.3	91089.7	91879.6	140450.3	136857.0	117166.4	139219.2
P29387	Guanine nucleotide-binding protein subunit beta-4	Gnb4	340	37.4	6.16	35453.5	32070.1	30391.3	23932.1	37967.0	41945.9	47460.4	35636.8	32696.1	31896.3	27791.1	24966.9	23183.0	24347.1
P68433	Histone H3.1	H3c10	136	15.4	11.12	108678.2	204336.2	133197.7	154823.1	155747.3	146064.1	205689.1	113202.3	194032.1	213153.8	1930536.3	2623407.4	2056731.0	2489153.2
Q5SUQ9	CST complex subunit CTC1	Ctc1	1212	133.9	8.12	87042.9	70268.9	79430.7	79247.7	87552.1	92805.0	94440.1	74678.4	78324.9	99948.0	123423.6	152300.9	89281.5	133859.0
P58069	Ras GTPase-activating protein 2	Rasa2	847	96.3	7.59	238201.8	245605.0	259850.7	252459.4	266978.5	256901.0	247745.7	216705.2	246439.6	261185.5	161861.7	167261.1	173097.8	148704.9
Q06770	Corticosteroid-binding globulin	Serpina6	397	44.7	5.24	301742.8	257040.0	235643.6	240938.8	185580.5	233019.4	247705.0	258680.6	201169.5	221109.3	512857.7	443787.3	397059.7	442729.9
Q9CRY7	Lysophospholipase D GDPD1	Gdpd1	314	35.8	8.31	340145.4	393337.9	394873.4	377324.7	421370.4	392712.9	364457.6	337205.8	398152.5	412972.7	707323.5	716759.8	717908.4	734320.3
Q8C5P7	Testis development-related protein	Tdrp	182	20.2	5.73	167459.5	167744.9	181651.7	158519.0	165072.4	165470.8	171092.0	172235.0	173175.8	156454.7	184188.7	209754.2	200570.7	201060.7
Q6PCL9	Poly(A) polymerase gamma	Papolg	739	82.9	9.16	94575.7	120203.1	107508.9	110514.7	123726.9	135847.8	104667.4	101730.6	108621.8	114543.7	45726.1	45909.5	48080.0	42925.8
Q3KNM2	E3 ubiquitin-protein ligase MARCHF5	Marchf5	278	31.2	8.7	273595.1	311958.8	278432.1	289534.8	311290.0	325325.1	280424.9	278793.4	292079.5	295266.1	219800.4	219524.2	217102.4	230917.9
Q99J38	Tetratricopeptide repeat protein 30A1	Ttc30a1	664	76.2	5.11	130724.3	148758.9	155976.3	165239.1	115651.1	115603.7	116619.8	128690.0	133425.9	148497.7	46562.3	54484.8	36603.3	46146.6
P35282	Ras-related protein Rab-21	Rab21	222	24.1	7.94	110429.0	128388.1	116212.4	133735.0	99955.0	129377.5	102146.0	110583.2	109254.3	106461.0	109057.0	107258.7	115761.3	108806.9
Q9WU20	Methylenetetrahydrofolate reductase	Mthfr	654	74.5	5.26	148708.7	139754.4	158470.2	141611.6	164915.3	193203.1	206679.2	161378.5	174506.6	149775.6	148143.5	149079.9	181852.1	150819.8
E9PZM7	RING-type domain-containing protein	Scaf11	1456	162	6.77	101688.3	97875.0	102101.0	99021.1	124449.2	110660.4	111693.3	114114.2	112945.9	106457.1	109798.8	104493.6	116888.4	87367.7
Q91XU0	ATPase WRNIP1	Wrnip1	660	71.7	6.18	257289.3	254775.7	212397.7	236213.3	243449.8	256178.4	234114.9	194959.5	260919.7	270133.8	58442.3	64804.5	62404.1	64211.6
P47739	Aldehyde dehydrogenase, dimeric NADP-preferring	Aldh3a1	453	50.4	6.95	172959.1	132114.0	135203.7	133607.2	163180.8	175452.2	173078.0	189565.5	138453.3	140686.3	192147.4	165353.1	163117.6	166358.7
Q9DCV7	Keratin, type II cytoskeletal 7	Krt7	457	50.7	5.87	82901.6	79516.6	72296.0	81821.3	65927.8	111433.5	107495.0	111425.0	71374.9	64531.7	453379.0	507151.0	417714.3	505404.1
Q60631	Growth factor receptor-bound protein 2	Grb2	217	25.2	6.32	165569.0	127961.4	163355.4	134394.5	186220.3	189429.5	151131.4	160370.0	159811.6	135758.0	163512.8	198827.5	167586.0	150403.2
Q9QZ23	NFU1 iron-sulfur cluster scaffold homolog, mitochondrial	Nfu1	255	28.5	5.03	249156.0	254435.6	259951.4	262798.7	258831.8	245612.8	222360.7	243534.3	258641.7	265019.7	424312.9	478936.4	441340.9	448390.3
Q9WVL0	Maleylacetoacetate isomerase	Gstz1	216	24.3	7.85	69907.8	70994.7	66366.5	62884.5	79171.5	85700.9	75469.3	67147.1	70711.2	77850.5	110293.1	116433.5	99338.8	109004.6
P70460	Vasodilator-stimulated phosphoprotein	Vasp	375	39.6	8.53	310920.1	301514.8	298324.9	289551.0	334984.6	323942.5	327684.0	305511.4	303011.4	309148.2	330299.0	350543.5	330440.6	332939.8
O35083	1-acyl-sn-glycerol-3-phosphate acyltransferase alpha	Agpat1	285	31.7	9.14	113303.9	126482.6	108213.5	116460.1	116221.5	121618.2	106449.6	105925.3	119250.8	119300.1	260336.4	306670.3	257593.5	301004.8
Q9ERF3	WD repeat-containing protein 61	Wdr61	305	33.8	5.36	277443.9	264121.2	274091.1	275408.2	280864.0	259988.2	251064.3	265152.3	277026.9	260656.3	151799.1	163278.9	153968.9	160621.4
Q80XQ2	TBC1 domain family member 5	Tbc1d5	815	91.8	6.79	58167.3	58380.1	63022.0	65964.7	60586.1	54803.8	54157.0	55395.4	56679.0	54908.7	185603.7	199753.5	175637.6	187407.5
Q9D9V3	Ethylmalonyl-CoA decarboxylase	Echdc1	322	35.4	7.01	164408.5	201739.4	162426.4	183398.6	169293.5	171676.2	178768.2	167206.5	153617.6	158922.8	94592.3	97456.0	91278.7	108586.1
Q8K0L9	Zinc finger and BTB domain-containing protein 20	Zbtb20	741	81	6.55	187162.1	200830.7	180586.4	183381.0	206801.0	216508.4	204593.3	206879.1	191776.5	203956.7	114628.2	112904.6	104200.6	109526.0
Q9Z1A9	TBC1 domain family member 8	Tbc1d8	1134	130	5.48	82886.1	80218.0	75322.6	83461.8	85776.6	80841.2	66576.7	76436.2	81607.0	80008.9	90528.8	104474.5	87635.6	90503.7
Q3UV17	Keratin, type II cytoskeletal 2 oral	Krt76	594	62.8	8.43	442885.8	504566.1	424424.3	602537.8	799512.2	791689.8	594743.1	599732.0	711386.5	650799.1	79969.6	80665.3	77177.5	94799.4
Q6P9Q6	FK506-binding protein 15	Fkbp15	1216	132.9	5.07	98881.8	118368.1	98238.9	100201.1	107104.5	121092.4	99190.4	117055.4	98954.3	100729.7	272065.0	280669.9	268536.8	280877.6
P35831	Tyrosine-protein phosphatase non-receptor type 12	Ptpn12	775	86.5	5.99	181873.2	207253.4	190743.1	187356.8	199872.7	197739.1	178718.1	168076.4	184650.0	187145.8	103153.3	105381.1	105821.1	100868.3
Q9R0Q6	Actin-related protein 2/3 complex subunit 1A	Arpc1a	370	41.6	8.18	290058.0	300644.1	275364.4	298322.9	277894.6	270318.6	234589.3	287744.8	275801.8	285930.5	264237.9	244079.1	253448.7	243941.8
P52479	Ubiquitin carboxyl-terminal hydrolase 10	Usp10	792	87	5.17	116463.5	119487.0	113450.3	127411.7	113054.3	115012.4	114128.3	117456.7	121054.6	125438.7	157307.7	167391.1	158312.6	164499.9
O70493	Sorting nexin-12	Snx12	165	19.1	7.34	376169.3	347626.8	327142.6	348840.6	376901.6	396576.4	369442.5	354401.9	378315.5	369169.5	384888.3	402910.3	409264.3	401904.6
Q62315	Protein Jumonji	Jarid2	1234	137.4	9.42	182036.7	163864.4	176567.7	160774.5	178496.0	181681.0	162388.1	159758.2	182277.7	173672.2	110979.9	114086.2	125207.2	103931.8
A7MCT6	Ethanolamine kinase 2	Etnk2	385	44.5	7.21	198611.6	189390.8	183255.1	193800.3	196637.5	190120.5	179061.0	195464.7	191544.8	194092.3	199660.1	220492.1	184360.1	202337.5
E9PYR1	F-box domain-containing protein	Fbxl18	771	84.4	8.22	70109.0	86530.4	81093.5	77130.6	86200.8	75264.1	79449.6	81069.8	81842.7	70487.4	43226.5	46009.5	40703.1	45574.6
Q3UNZ8	Quinone oxidoreductase-like protein 2	Cryzl2	350	37.8	8.9	49901.0	54366.2	38940.8	53641.7	51178.0	47325.7	50016.9	44520.8	52206.2	47269.0	21898.5	26619.1	21493.4	24786.6
Q6PCP5	Mitochondrial fission factor	Mff	291	32.9	6.83	78988.1	90134.3	83706.2	88427.8	68970.9	86899.1	73067.1	71537.2	86628.4	81948.8	85154.1	94725.2	89269.0	89579.7
P07214	SPARC	Sparc	302	34.4	4.86	348448.9	373333.3	306671.9	376518.0	340395.2	300446.2	276292.3	346793.9	323858.6	332881.3	307762.9	375536.6	255529.2	328277.4
Q9EQ28	DNA polymerase delta subunit 3	Pold3	462	50.8	9.32	133034.1	147653.1	133106.6	132127.3	156023.1	153818.3	126795.5	125743.4	141105.5	148095.3	131472.8	121015.5	117937.2	119045.8
Q61205	Platelet-activating factor acetylhydrolase IB subunit gamma	Pafah1b3	232	25.8	6.93	624380.2	554714.1	612213.6	510004.2	552395.0	593799.9	696103.4	639604.3	610783.3	575901.1	776492.7	751707.6	793777.1	759259.3
E9Q3P4	Centromere protein F	Cenpf	2997	342.3	5.16	85219.0	106949.6	104392.3	136660.5	104575.5	113859.2	88892.4	87558.7	96183.9	111868.9	110732.6	126246.8	126924.1	131404.4
Q9CWW6	Peptidyl-prolyl cis-trans isomerase NIMA-interacting 4	Pin4	131	13.8	9.77	329178.5	335191.1	314328.0	320119.8	357998.9	346156.5	349577.6	339014.9	334783.4	354846.0	625300.8	638420.7	662114.2	634384.1
Q8BVU5	ADP-ribose pyrophosphatase, mitochondrial	Nudt9	350	38.6	6.76	430609.7	383747.9	436075.1	418825.5	434523.1	431947.1	437430.3	445475.5	427016.2	402458.4	476165.8	439598.7	403635.4	457426.8
Q922L6	Negative elongation factor D	Nelfcd	591	66.2	5.16	85229.4	92967.5	86085.5	92999.6	94139.7	94732.1	90501.9	80807.3	93783.7	98658.9	101927.3	106525.7	96538.7	106647.8
P16406	Glutamyl aminopeptidase	Enpep	945	107.9	5.44	217566.1	214043.9	246572.9	219591.0	279922.4	310302.5	350074.4	260245.0	250753.4	250044.9	117053.2	140428.2	130770.9	123259.6
Q61142	Spindlin-1	Spin1	262	29.6	6.96	123317.4	129609.6	142530.3	109856.9	154776.6	156893.8	156309.5	139294.4	139971.3	134848.1	165757.2	149952.2	195175.2	161670.0
Q8BIJ7	RUN and FYVE domain-containing protein 1	Rufy1	712	80.3	5.68	259810.3	284467.2	280109.2	279081.4	296323.9	265171.1	274484.6	240177.9	283799.6	289492.9	119359.3	110902.1	99670.6	109716.9
Q3TX08	tRNA (guanine(26)-N(2))-dimethyltransferase	Trmt1	663	72.3	8	258367.2	273195.2	277794.0	249521.7	277442.2	301223.0	266607.1	257792.6	287176.2	271215.0	96925.9	105251.6	102941.9	88243.1
Q7TSH6	SR-related and CTD-associated factor 4	Scaf4	1183	129	9.64	197633.1	193308.1	181833.4	196670.3	179563.2	179271.9	177152.2	176458.9	181849.1	190132.0	87001.8	99939.5	71619.4	90492.6
Q64310	Surfeit locus protein 4	Surf4	269	30.4	7.78	69597.7	71442.6	65529.8	69238.4	70576.6	75683.7	72573.5	62024.1	75532.0	71434.1	200865.6	203730.1	184580.7	207537.3
Q9QYF1	Retinol dehydrogenase 11	Rdh11	316	35.1	8.91	265903.0	261712.3	341469.6	375735.4	196577.4	185705.8	212390.5	214045.6	288781.6	281282.9	352682.7	473031.4	414363.7	451111.1
Q9D168	Integrator complex subunit 12	Ints12	461	48.5	9.69	203374.9	167446.6	228992.9	176479.8	221129.3	211074.5	203450.3	170556.1	202238.5	209422.2	341075.0	307207.2	381046.5	333114.7
Q5XG69	Soluble lamin-associated protein of 75 kDa	Fam169a	665	73.2	4.68	137329.4	153216.9	157660.0	174256.4	161291.4	166485.4	187851.9	165380.8	147315.2	154208.8	122293.0	141980.2	121468.6	144540.8
Q8VE62	Polyadenylate-binding protein-interacting protein 1	Paip1	400	45.7	4.55	450498.9	502058.9	478305.0	547684.7	427648.6	357748.3	343705.7	419319.3	450994.5	447131.0	555132.6	620054.0	609676.3	679178.6
Q9DBZ5	Eukaryotic translation initiation factor 3 subunit K	Eif3k	218	25.1	4.93	171615.2	260576.0	162070.7	234986.9	211972.9	198047.5	160062.8	177402.3	196086.4	227672.0	133078.8	186203.3	122926.9	136080.2
Q69ZA1	Cyclin-dependent kinase 13	Cdk13	1511	164.5	9.69	158234.0	184204.7	168249.6	189671.4	159659.8	151595.4	149771.4	155363.1	153838.7	164185.2	211029.0	238737.8	212494.2	217843.6
P28659	CUGBP Elav-like family member 1	Celf1	486	52.1	8.46	498952.6	489968.5	468157.6	486822.0	516012.2	466953.9	449922.4	473879.7	489902.3	510223.5	427638.7	400399.9	339457.4	378217.3
Q9CQB5	CDGSH iron-sulfur domain-containing protein 2	Cisd2	135	15.2	9.51	255214.7	308184.6	281888.9	281617.2	279363.8	281728.4	253678.8	279854.0	276648.7	273866.4	315104.2	325752.7	344377.5	326829.8
O88967	ATP-dependent zinc metalloprotease YME1L1	Yme1l1	715	80	8.97	472314.7	490391.3	471837.3	487529.5	466486.8	521906.1	435694.7	476603.5	479276.3	469522.8	168176.8	161365.7	148363.3	155368.4
O55125	Protein NipSnap homolog 1	Nipsnap1	284	33.3	9.44	263622.3	253483.4	272806.4	238495.2	277822.3	288265.4	308512.5	271416.1	278967.6	268822.1	199545.4	181522.5	195704.5	169495.5
Q9JMK2	Casein kinase I isoform epsilon	Csnk1e	416	47.3	9.66	115749.4	128769.5	125974.2	124165.9	141643.1	120685.5	106585.1	127714.2	135754.5	136074.1	#N/A	#N/A	#N/A	#N/A

Q9JKF7	39S ribosomal protein L39, mitochondrial	Mrpl39	336	38.5	7.94	237111.0	235799.5	221972.7	232653.3	228625.1	220490.3	207221.0	223007.3	218813.7	237002.9	496912.3	516072.7	507090.2	485708.0
Q6SKR2	Methyltransferase N6AMT1	N6amt1	214	23	5.62	142264.3	137591.5	142784.9	131527.8	151439.1	163269.0	155024.4	142918.3	142292.8	146822.2	111363.1	117146.3	122777.0	105418.1
Q9CRD0	OCIA domain-containing protein 1	Ociad1	247	27.6	7.81	292723.0	281030.7	285443.2	270977.7	277245.6	282073.5	265081.2	264766.5	293787.0	294762.8	240652.3	259868.7	300808.5	274521.5
Q9CWK3	CD2 antigen cytoplasmic tail-binding protein 2	Cd2bp2	342	37.7	4.6	390367.2	405922.3	453775.5	443630.7	424374.4	408698.7	367927.3	356614.1	400016.8	432666.4	218226.6	212508.9	209422.4	209287.2
Q9D0R4	Probable ATP-dependent RNA helicase DDX56	Ddx56	546	61.2	9.17	68833.7	88091.4	67374.3	73999.9	80473.3	76526.1	66199.7	68046.1	73396.5	80727.5	35684.4	39078.9	47375.1	39354.1
Q9WVP6	Poly(A) polymerase beta	Papolb	641	72.2	6.74	1040799.3	971831.8	993802.6	945149.6	1024331.8	1031246.0	983464.5	925636.0	1000767.9	1057353.7	995739.1	959271.0	874094.0	908082.2
Q9CXY9	GPI-anchor transamidase	Pigk	395	44.9	6.54	146199.2	150969.1	135492.7	144373.5	143929.1	137638.2	124420.8	138320.7	152659.6	142256.4	241448.7	252064.4	232928.6	240227.2
Q9QYF9	Protein NDRG3	Ndrg3	375	41.5	5.25	121574.1	114905.5	107186.4	106512.5	114146.9	114371.9	113683.7	105794.8	113146.3	107629.8	440346.0	403988.3	406183.5	406445.2
Q9D8B4	NADH dehydrogenase [ubiquinone] 1 alpha subcomplex subunit 11	Ndufa11	141	15	8.35	451675.0	447292.0	456497.0	446049.4	472208.1	446393.1	420236.3	456297.6	452255.7	446164.6	178642.3	210142.2	194048.0	206741.7
P53702	Cytochrome c-type heme lyase	Hccs	272	31	7.12	21681.1	17222.5	25130.1	21578.9	27694.9	21889.8	19518.3	24301.6	22662.8	23907.2	132808.3	166163.5	136658.9	145812.9
Q91VT4	Carbonyl reductase family member 4	Cbr4	236	25.4	9.69	498173.8	548764.7	622144.4	556833.6	571318.5	487372.1	478339.3	471124.2	550849.6	567314.6	499343.1	467476.0	472049.7	503212.2
O08804	SERPIN domain-containing protein	Serpinb6b	377	42.5	5.26	214631.4	216199.9	180822.5	199204.1	206259.6	231268.8	236327.3	211044.9	194757.4	199592.0	140580.0	139340.1	117842.8	130321.4
Q8K354	Carbonyl reductase [NADPH] 3	Cbr3	277	30.9	6.57	73963.2	72573.7	68911.8	73504.7	88432.9	95321.5	101710.8	78458.7	73542.3	75960.8	12131.4	13506.7	20412.6	17547.4
Q8BZX4	Splicing regulatory glutamine/lysine-rich protein 1	Srek1	494	56.7	10.48	89226.5	96975.8	86019.8	100032.6	87413.8	103552.4	91269.3	90372.6	92051.7	89534.0	58771.8	69411.2	72480.7	68468.6
Q99LJ6	Glutathione peroxidase 7	Gpx7	186	21	8.27	279980.2	258368.0	248483.5	239119.3	290753.1	288777.2	266738.9	311052.8	235545.5	248364.5	195033.1	214065.1	180558.4	198992.1
Q8VDI7	Ubiquitin-associated domain-containing protein 1	Ubac1	409	45.5	4.97	103404.6	113111.6	104508.4	122284.9	116375.3	119809.9	105719.8	101912.9	104877.4	111988.8	141974.7	165405.1	156640.3	157823.8
P70429	Ena/VASP-like protein	Evl	414	44.3	8.85	307608.0	282514.2	281661.9	296558.6	277884.7	268666.4	297998.8	275013.0	275169.9	295627.3	139536.9	145794.3	152774.9	143004.4
Q8BHA3	D-aminoacyl-tRNA deacylase 2	Dtd2	168	18.2	7.91	140373.4	115367.3	143592.9	133972.7	117532.8	110502.4	107767.3	113532.8	121800.1	128036.5	154060.8	147053.9	134171.5	141313.0
Q4LDD4	Arf-GAP with Rho-GAP domain, ANK repeat and PH domain-containing protein 1	Arap1	1452	162.2	6.18	163347.3	167832.7	187016.5	173817.4	184011.5	203309.2	176015.1	191492.1	157121.5	165943.4	160213.1	155040.6	148133.5	162916.0
Q60875	Rho guanine nucleotide exchange factor 2	Arhgef2	985	111.9	7.25	102527.1	89404.8	106010.4	110421.3	119020.0	106781.0	104052.3	95995.7	94302.1	94748.2	92233.1	98088.0	86094.4	90460.8
Q6Q899	Antiviral innate immune response receptor RIG-I	Ddx58	926	105.9	6.65	176345.0	181560.8	147296.5	200515.9	151746.9	168745.0	178260.0	168693.5	146068.4	162710.6	182146.5	179750.4	160132.8	182208.0
Q5SV85	Synergin gamma	Synrg	1306	139.5	5.03	91838.7	102156.4	101407.6	99441.9	93768.3	91049.0	86484.7	95366.4	104058.8	106403.5	131619.7	135389.3	138094.2	138533.6
Q91ZU6	Dystonin	Dst	7393	833.7	5.31	54984.0	57393.4	62314.6	64350.5	67003.8	57312.1	58211.1	70051.2	57864.3	64189.8	#N/A	#N/A	#N/A	#N/A
Q91VE6	MKI67 FHA domain-interacting nucleolar phosphoprotein	Nifk	317	36.2	10.24	108164.1	103288.4	104389.4	89846.3	115160.5	96223.4	87170.1	99804.2	106163.6	107353.6	105946.0	123505.4	134453.3	117353.4
Q9CR00	26S proteasome non-ATPase regulatory subunit 9	Psmd9	222	24.7	6.43	188504.5	206630.0	200281.5	203043.9	191295.8	212767.4	199678.9	200315.0	209405.3	212091.3	681329.9	755648.4	666642.5	722092.5
Q9CY73	39S ribosomal protein L44, mitochondrial	Mrpl44	333	37.5	8.51	386286.2	461541.1	309631.1	393005.8	365163.1	369279.5	363598.8	380065.2	388615.5	421342.5	74887.4	87571.2	68262.0	72021.4
P17439	Lysosomal acid glucosylceramidase	Gba	515	57.6	7.75	101747.2	102607.7	81481.8	98061.9	79361.9	85516.0	79975.0	93754.1	92827.1	78988.9	210277.3	201264.5	170288.3	193489.0
Q9CY50	Translocon-associated protein subunit alpha	Ssr1	286	32	4.45	272133.4	311706.9	249681.8	269725.6	285901.0	261910.4	249057.0	236347.5	270140.4	290299.6	240502.7	225638.0	240540.7	224943.2
O70480	Vesicle-associated membrane protein 4	Vamp4	141	16.3	7.36	177004.4	199690.7	219087.1	229254.1	193946.3	180796.2	169486.1	167693.7	183786.9	190789.7	19783.3	34086.6	23147.2	32420.5
Q8CIM3	D-2-hydroxyglutarate dehydrogenase, mitochondrial	D2hgdh	535	58.5	7.37	82423.2	93975.4	83939.8	80747.2	85080.2	81726.7	86162.2	82039.0	94634.4	100561.3	100459.0	94295.6	95988.8	102279.9
Q8BZQ7	Anaphase-promoting complex subunit 2	Anapc2	837	95.2	5.31	183813.8	182538.7	179946.9	180241.8	191627.3	196069.6	173469.7	176306.6	184228.9	183364.8	188211.1	221792.1	196375.0	196698.3
Q8BK72	28S ribosomal protein S27, mitochondrial	Mrps27	415	47.7	5.5	252129.9	244145.8	240835.2	263110.5	256580.9	239970.9	245118.3	237069.1	264200.4	233332.9	171350.0	161900.5	179734.5	172043.6
Q8K3Z9	Nuclear envelope pore membrane protein POM 121	Pom121	1200	120.9	10.2	141263.1	143639.4	142864.5	133517.6	157208.9	149269.3	144488.4	136354.3	144903.4	144449.0	54601.2	54999.7	71059.1	64029.6
Q9Z1J3	Cysteine desulfurase, mitochondrial	Nfs1	459	50.5	8.16	147585.6	176111.0	149638.8	145626.6	166406.9	156599.0	154237.1	141341.0	164088.8	164134.3	210937.8	216411.2	204810.9	203185.6
Q64435	UDP-glucuronosyltransferase 1-6	Ugt1a6	531	60.4	8.62	130208.6	138044.1	109239.1	147038.8	119154.3	151968.4	154764.1	137430.9	122690.6	121927.1	77412.9	79716.4	62587.9	78864.5
Q3UE37	Ubiquitin-conjugating enzyme E2 Z	Ube2z	356	38.3	5.62	135455.7	144480.3	135219.3	127770.6	136377.8	151284.2	138036.2	145564.3	135029.7	126236.4	292570.2	297390.8	288081.6	283361.9
P06797	Cathepsin L1	Ctsl	334	37.5	6.83	222143.5	223316.3	204179.3	204824.7	194954.9	163659.0	191629.3	223419.1	228421.2	222327.6	143318.8	154674.8	140807.5	144469.7
A2AL36	Centriolin	Cntrl	2334	268.7	5.52	172468.4	197368.7	200149.7	194828.5	206178.5	227037.6	170447.8	164564.4	205496.7	218535.1	124004.8	126634.5	132332.5	125675.6
P97461	40S ribosomal protein S5	Rps5	204	22.9	9.72	439453.1	418013.4	469941.1	435865.8	410574.3	417518.3	389396.7	460467.7	435266.3	423334.6	251288.1	250879.2	265418.7	237321.4
Q9DBX3	Sushi domain-containing protein 2	Susd2	820	90.6	6.62	300448.5	232045.1	259795.4	230513.3	306222.2	336554.9	410976.8	291341.8	294280.9	263642.3	260803.3	256030.4	260912.0	239151.0
Q60692	Proteasome subunit beta type-6	Psmb6	238	25.4	5.11	127360.3	119710.4	132191.8	128244.6	127737.5	120278.7	118137.6	131642.9	128557.5	137080.5	197266.6	213547.2	196360.4	214880.4
Q9CQE7	Endoplasmic reticulum-Golgi intermediate compartment protein 3	Ergic3	383	43.2	6.47	132213.1	134718.2	138650.9	145006.8	136915.7	152568.3	132643.4	133601.4	140795.0	139824.9	179957.2	186777.9	186630.8	183441.2
P97477	Aurora kinase A	Aurka	395	44.7	9.38	256138.2	333147.0	349895.0	400214.4	260447.5	236368.2	193347.0	216830.6	312691.1	320248.9	213076.7	279565.2	272351.2	268813.0
Q8BXA5	Cleft lip and palate transmembrane protein 1-like protein	Clptm1l	539	62.1	8.84	131614.5	145574.5	125589.5	151305.9	131054.7	130584.8	122321.3	120403.7	141536.8	131248.4	89834.8	101194.6	90458.5	89391.8
Q99LI2	Chloride channel CLIC-like protein 1	Clcc1	539	60.6	5.68	109876.5	123182.8	116035.2	126375.2	138706.8	121871.3	107782.1	103658.9	122471.1	123305.8	147238.7	128920.6	131503.1	141233.9
Q99ME2	WD repeat-containing protein 6	Wdr6	1125	121.8	6.98	31977.6	39316.4	37535.3	43641.5	36051.8	36185.0	32376.3	35358.9	39900.6	39017.6	145650.2	161152.2	153927.7	152100.6
Q9R0L7	A-kinase anchor protein 8-like	Akap8l	642	71.4	5.05	226661.1	301955.2	285927.4	295851.2	325110.6	307061.4	249874.7	248882.8	290109.6	281473.5	150939.7	167130.2	158644.1	176126.1
Q8K3G5	Inactive serine/threonine-protein kinase VRK3	Vrk3	453	50.8	8.57	102061.7	113454.4	113479.8	118287.4	132016.6	120845.0	126275.3	113492.5	116150.8	119776.5	48777.5	52795.8	54993.6	61061.1
P62911	60S ribosomal protein L32	Rpl32	135	15.9	11.33	322648.8	407122.2	342328.2	401644.7	332376.9	311904.2	327657.5	282735.3	370979.3	402516.1	341153.5	485544.1	314686.5	492886.8
Q921E6	Polycomb protein EED	Eed	441	50.2	7.03	267074.4	263173.1	283027.1	239010.6	250197.2	239713.4	208309.2	235520.6	244746.2	245320.5	230554.2	256183.8	242322.4	218142.0
Q8BVA4	Leiomodin-1	Lmod1	595	66.3	9.19	332094.6	335124.9	292630.5	286426.2	314631.1	340640.7	358235.5	296444.6	337977.5	303141.0	305532.0	322631.5	276540.4	282268.6
Q8VE19	GATOR complex protein MIOS	Mios	875	98.3	6.71	30377.0	36640.0	26795.3	31134.6	34217.4	36226.3	27057.0	29192.0	31766.6	32610.7	159678.1	157670.6	149140.2	171399.4
O35638	Cohesin subunit SA-2	Stag2	1231	141.2	5.43	149006.1	155352.6	170792.5	158084.9	221738.9	216229.3	232507.5	152799.8	172441.1	174887.9	172547.4	150906.4	164855.4	154635.6
Q9R1Z8	Vinexin	Sorbs3	733	82.3	9.2	209649.9	214214.7	232972.6	223369.0	213101.3	212333.8	237329.7	206154.5	200575.3	231431.9	278227.3	323769.6	279650.9	345644.8
Q9Z1P6	NADH dehydrogenase [ubiquinone] 1 alpha subcomplex subunit 7	Ndufa7	113	12.6	10.17	87080.6	82184.7	101512.6	96300.1	97337.0	82844.9	85692.1	100892.5	87527.9	91145.2	122179.4	139695.9	147317.1	138928.6
Q8C0J6	Ankyrin repeat domain-containing protein SOWAHC	Sowahc	512	54.9	6	97440.1	84288.8	97915.8	95956.5	102684.8	90226.4	94942.5	94845.9	101559.8	101121.0	52393.0	58298.5	51743.4	53083.1
Q8BIL5	Protein Hook homolog 1	Hook1	728	84.4	5.22	177916.8	176093.7	183341.8	189519.0	169061.3	171809.4	165127.8	163998.4	173249.2	165696.6	80630.9	101460.7	90364.2	99265.2
A2BE28	Ribosomal biogenesis protein LAS1L	Las1l	776	89.4	4.44	153204.0	167146.4	164054.3	159803.3	172500.8	170148.0	170744.4	152738.2	170467.9	167297.3	298409.0	302315.4	287354.4	274567.2
P62852	40S ribosomal protein S25	Rps25	125	13.7	10.11	2252549.1	2358583.9	2374886.2	2349486.8	2019946.6	1905093.7	1810776.0	2171048.8	2058046.3	2233788.2	3334926.8	3526960.7	3438169.3	3407210.7
Q3TLP5	Enoyl-CoA hydratase domain-containing protein 2, mitochondrial	Echdc2	296	31.8	8.72	164930.2	190813.8	158018.3	168019.9	176464.5	181997.7	189850.7	160986.9	169194.9	179941.7	149795.0	220869.9	143124.3	167765.3
Q8VE22	28S ribosomal protein S23, mitochondrial	Mrps23	177	20.3	8.59	267722.9	289317.3	280759.1	282217.7	308691.2	261385.7	267878.0	290184.9	301787.5	297348.5	321156.6	328999.4	298479.1	323546.1
P05784	Keratin, type I cytoskeletal 18	Krt18	423	47.5	5.33	99279.5	106356.9	105940.3	150961.6	102721.8	162541.0	254772.7	183107.7	107926.9	100178.6	38191.1	36239.8	37751.3	53399.4
Q62433	Protein NDRG1	Ndrg1	394	43	6.1	155515.6	248839.9	141671.5	176734.7	147875.3	173439.2	194274.6	166760.6	188390.8	189598.6	173508.1	191180.5	174430.5	173187.8
P35762	CD81 antigen	Cd81	236	25.8	5.83	172361.2	158764.1	163304.0	156488.7	169128.4	172778.1	164440.8	159090.0	173790.4	143186.6	329487.1	328977.7	295729.1	305284.7
Q7TMC8	L-fucose kinase	Fcsk	1090	119.2	6.6	234032.5	236484.4	270512.0	230113.9	269113.7	263363.1	235314.3	249606.3	228105.5	235823.1	146785.2	145266.4	146775.7	144765.3
Q80YR4	E3 ubiquitin-protein ligase ZNF598	Znf598	908	99.1	8.34	82320.8	76898.3	67996.7	67877.0	76880.8	72601.8	73151.4	75903.5	82553.5	78036.6	128284.1	129271.6	111776.1	137473.9
Q62101	Serine/threonine-protein kinase D1	Prkd1	918	102	6.58	157115.3	156684.9	155423.2	131322.3	161580.2	166519.4	168129.3	158151.7	166053.6	159874.9	301289.1	274927.8	254662.9	255086.4
Q8C156	Condensin complex subunit 2	Ncaph	731	82.3	4.96	73175.5	90850.8	67894.5	78914.8	89750.5	93251.6	82254.6	74062.5	80611.6	86895.4	88366.5	96996.4	83134.6	92707.0
Q80V86	Integrator complex subunit 8	Ints8	995	113.3	6.96	188297.0	237423.0	176656.7	194818.3	218477.7	217722.7	191600.2	197158.1	208993.5	226231.7	130990.5	142033.3	123733.6	126225.8
P52430	Serum paraoxonase/arylesterase 1	Pon1	355	39.5	5.22	140356.8	134367.9	107746.5	112264.8	98428.0	106935.8	134303.2	123254.9	111385.8	99845.5	218519.2	209653.6	174262.4	198038.5
Q8CHT3	Integrator complex subunit 5	Ints5	1018	108.3	7.06	90102.4	98464.7	98522.2	89306.8	111458.1	109274.7	96030.5	89683.8	102754.0	98692.8	119742.8	132776.6	129375.2	121709.9
Q91VK1	Basic leucine zipper and W2 domain-containing protein 2	Bzw2	419	48	6.68	143221.2	163419.3	158100.0	151163.8	159011.2	167080.2	161770.3	161510.1	164044.8	154204.4	457198.0	566992.5	433597.0	482946.3
Q922S8	Kinesin-like protein KIF2C	Kif2c	721	81	7.97	88738.5	103350.6	97957.3	91699.4	101890.9	97350.0	90703.8	85247.6	105753.6	99921.9	188742.0	224524.7	215641.7	225968.8
Q9CYN9	Renin receptor	Atp6ap2	350	39.1	5.54	84786.0	79552.6	75219.0	77149.4	83960.5	80281.8	72522.8	78934.1	78378.1	79425.5	62723.9	60542.5	68926.3	67001.8
Q91YR5	eEF1A lysine and N-terminal methyltransferase	:EF1AKNM	698	78.7	6.83	107596.1	111215.2	110367.1	97490.9	116338.9	106718.8	102905.9	97420.9	110384.9	118011.6	230721.8	226671.4	215118.4	217406.4
Q922Q9	Chitinase domain-containing protein 1	Chid1	393	44.9	7.97	174654.8	166638.0	173773.7	169981.3	170125.2	181332.1	160637.6	176605.2	162811.2	165196.9	181161.0	184888.3	189212.0	185018.0
P97298	Pigment epithelium-derived factor	Serpinf1	417	46.2	6.98	221054.3	213513.5	181026.2	183853.5	164734.4	194226.1	155991.2	239332.2	162807.3	178310.6	220115.1	251528.4	178489.3	211856.6
Q78T81	Protein FAM102A	Fam102a	392	42.8	8.56	50172.6	40898.9	41639.9	36584.1	39739.7	40068.1	37553.3	48971.2	41540.4	41135.9	142395.6	146775.0	152803.7	132900.9
Q5BL07	Peroxisome biogenesis factor 1	Pex1	1284	141.3	6.35	143482.5	149107.9	127165.0	155423.8	132462.6	152507.1	131657.8	126128.4	152239.5	162331.2	385894.0	462928.4	391287.8	418984.1
Q8C804	Spindle and centriole-associated protein 1	Spice1	860	95.6	7.05	142507.9	171324.3	132666.9	146950.5	139329.5	145363.4	128354.1	136126.1	142755.7	150505.7	205242.0	238964.5	202895.8	235075.2
P13405	Retinoblastoma-associated protein	Rb1	921	105.3	7.93	246891.7	237982.5	272516.3	218109.7	273897.3	271004.0	228004.1	266537.8	239309.8	242232.2	277738.5	214520.6	226127.8	202091.9
Q9ES46	Beta-parvin	Parvb	365	41.6	6.33	213180.0	228759.8	181612.7	218016.4	204746.2	218894.8	220926.3	197085.9	223344.1	219351.1	139120.5	154527.2	133951.3	142682.8
Q5SSH7	Zinc finger ZZ-type and EF-hand domain-containing protein 1	Zzef1	2924	328.1	6.11	86422.8	91733.6	76088.6	84574.9	79588.6	80895.9	84982.3	77146.7	83097.7	87353.5	156610.8	167795.2	146033.9	161866.1
O88456	Calpain small subunit 1	Capns1	269	28.4	5.63	754664.4	679654.3	669297.8	682980.8	698680.5	777179.3	827929.3	786718.2	647240.0	651846.2	560496.5	547870.6	493592.3	524757.4
D3YZZ5	GOLD domain-containing protein	Tmed7	224	25.2	6.89	227813.2	294447.5	291778.9	292286.2	277578.1	272450.1	244067.0	280227.0	264648.2	279418.7	327395.7	320775.7	341122.3	321948.9
Q8R2U0	Nucleoporin SEH1	Seh1l	360	39.7	8.05	160458.8	183075.7	148755.6	192951.9	186331.5	209545.5	169600.5	154958.2	180023.4	179432.7	339539.0	325209.5	311360.4	335519.2
Q8BV79	TPR and ankyrin repeat-containing protein 1	Trank1	2999	343.1	7.81	180061.9	171416.0	172216.4	161162.4	171530.3	173192.2	180103.2	166175.7	183770.1	189352.4	122511.3	127723.5	119763.5	108664.4
Q0P678	Zinc finger CCCH domain-containing protein 18	Zc3h18	948	105.6	7.8	323156.8	341933.2	372237.8	346089.3	361179.0	349845.1	347885.9	301896.0	341555.5	367224.6	185844.4	199555.7	185192.6	190092.4
E9Q4Z2	Acetyl-CoA carboxylase 2	Acacb	2448	275.6	6.32	363094.9	371898.7	372758.6	369027.4	369250.3	349281.5	340228.7	339462.3	375411.8	383761.3	337981.1	347031.8	355213.2	317894.5
Q9QZ73	DCN1-like protein 1	Dcun1d1	259	30.1	5.34	243655.8	224796.8	254349.0	240094.0	253524.0	250334.5	246680.6	239536.6	222569.3	234715.5	471922.0	423767.9	426902.9	424453.2
Q8VCF0	Mitochondrial antiviral-signaling protein	Mavs	503	53.4	6.37	111908.3	101283.8	84814.6	93038.7	107747.3	106502.5	103097.6	104214.4	108039.4	102064.9	85210.4	74123.8	76267.8	69319.1
P61255	60S ribosomal protein L26	Rpl26	145	17.2	10.55	902544.5	976928.5	920907.5	930793.4	849304.1	814845.7	821307.6	859038.5	906300.8	973448.2	1225987.1	1911797.3	1343168.3	1560318.1

Q3UXZ9	Lysine-specific demethylase 5A	Kdm5a	1690	192.1	6.39	96879.6	131385.7	112134.3	101496.3	131614.4	127445.8	104905.4	103437.6	118432.3	122879.8	156859.5	165800.0	155056.7	143897.5
Q64430	Copper-transporting ATPase 1	Atp7a	1491	161.9	6.62	259872.5	224739.3	247390.5	220516.4	267464.1	250628.8	246484.2	235673.1	253321.8	243369.0	260923.6	261302.6	266780.7	237269.6
Q9R0M6	Ras-related protein Rab-9A	Rab9a	201	22.9	5.66	165860.6	181501.3	163184.8	181068.6	177240.5	161511.1	157322.2	157147.2	163042.0	171013.3	461199.4	461447.3	486629.6	452619.6
Q8K1N4	Spermatogenesis-associated serine-rich protein 2	Spats2	545	58.9	9	211194.8	212998.1	234202.2	211232.1	211275.9	214331.9	179839.0	208748.7	202649.8	217398.3	125361.2	143677.8	133029.7	146329.9
A2AG06	Meiosis-specific coiled-coil domain-containing protein MEIOC	Meioc	965	108.8	6.89	107612.7	133764.9	131625.1	143204.0	127450.7	128611.1	100550.1	102262.2	124637.6	129563.7	70584.3	88035.4	84183.7	76577.7
Q9JJG0	Transforming acidic coiled-coil-containing protein 2	Tacc2	1149	124.1	5.06	163493.7	167063.7	190528.7	152658.6	186535.4	161448.1	185621.0	152921.5	179089.8	168672.8	268454.3	262526.9	244171.3	245861.2
Q3ULJ3	Smg4_UPF3 domain-containing protein	Upf3a	422	48.7	9.35	88151.6	89909.4	93829.6	101755.9	84420.3	90634.0	79209.8	89376.8	88758.3	92098.4	#N/A	#N/A	#N/A	#N/A
Q8VE09	Tetratricopeptide repeat protein 39C	Ttc39c	580	65.4	6.87	307519.0	283535.5	281613.7	278961.0	319017.0	325412.1	343921.7	273687.3	296836.1	325943.7	240037.1	233492.8	248889.3	223167.2
Q9CQ45	Neudesin	Nenf	171	18.9	5.27	693581.5	714335.6	674304.8	663660.1	586376.9	522145.7	515778.0	767053.1	636395.6	610743.6	306384.5	288138.6	290502.7	294419.7
Q8BHJ5	F-box-like/WD repeat-containing protein TBL1XR1	Tbl1xr1	514	55.6	5.63	109810.8	113016.4	117509.5	117202.3	114036.6	114100.8	106835.8	109101.4	115501.1	118358.9	#N/A	#N/A	#N/A	#N/A
Q8C0L9	Glycerophosphocholine phosphodiesterase GPCPD1	Gpcpd1	675	76.5	5.58	145112.4	159138.6	143167.8	145423.2	145169.1	147723.9	124580.1	130671.1	150004.3	157584.6	119418.9	120409.7	110467.7	112937.6
Q80UW8	DNA-directed RNA polymerases I, II, and III subunit RPABC1	Polr2e	210	24.6	5.95	254263.7	290759.3	278364.2	262834.2	293465.6	284167.7	258691.1	258353.0	292641.0	281031.6	236305.7	267468.3	272426.0	249845.8
Q03173	Protein enabled homolog	Enah	802	85.8	7.72	155394.0	126280.8	135514.6	133093.2	150975.7	149885.7	142906.7	144810.6	142055.5	138809.6	222857.1	265773.9	257189.9	254020.7
Q9QVP9	Protein-tyrosine kinase 2-beta	Ptk2b	1009	115.7	6.35	78559.6	81909.1	80280.6	84525.9	98664.5	94357.5	97994.3	81815.2	82526.0	89567.6	35357.6	31885.7	25908.3	30732.5
Q3U821	WD repeat-containing protein 75	Wdr75	830	94	6.18	145529.7	165206.4	144173.3	129313.8	181465.1	168331.2	149747.8	150924.0	150065.7	171940.4	164384.4	174549.1	158317.5	151236.8
Q9EP97	Sentrin-specific protease 3	Senp3	568	64.4	8.75	222141.6	264735.6	233739.4	233664.8	261962.5	252915.0	223830.5	233350.7	242125.7	255679.1	149996.8	158077.3	158264.3	158983.5
P57746	V-type proton ATPase subunit D	Atp6v1d	247	28.4	9.45	221946.9	239420.8	229684.3	225728.9	212797.1	209673.6	201950.4	205392.0	228237.9	231395.5	169323.3	172992.8	166305.6	178470.6
Q8BHN1	Gamma-taxilin	Txlng	524	60.3	8.12	105704.0	122520.4	105646.6	110480.9	123753.5	114545.9	101455.4	102866.8	118498.6	114231.1	247414.0	255294.7	291319.1	240872.7
O88508	DNA (cytosine-5)-methyltransferase 3A	Dnmt3a	908	101.6	6.65	130045.4	146215.4	134514.4	139348.6	154020.4	145500.7	125609.6	148982.6	136189.4	150709.9	541747.8	485647.9	468103.9	464155.1
Q9EP53	Hamartin	Tsc1	1161	128.7	6.37	147729.6	123062.1	121370.2	116730.2	126465.0	139580.9	134362.5	131793.2	120987.9	122799.9	74965.2	56567.0	64716.7	51894.2
Q9Z0J0	NPC intracellular cholesterol transporter 2	Npc2	149	16.4	7.68	220279.4	160037.5	199394.1	248258.6	237579.4	284897.4	276628.8	252336.5	229425.0	231739.7	342469.1	348345.3	337623.0	332398.8
B1AZP2	Disks large-associated protein 4	Dlgap4	992	108	7.08	149007.2	146346.4	143885.9	144490.2	144028.5	148074.8	141106.4	132876.9	142867.6	144900.5	314645.7	310344.1	267740.3	292916.0
Q9JKX4	Protein AATF	Aatf	526	59.4	4.93	72893.9	81426.1	72240.9	72513.0	70540.5	70431.5	68092.3	60247.6	72757.2	69978.3	89201.3	101206.3	86272.1	91788.7
Q70FJ1	A-kinase anchor protein 9	Akap9	3797	435.9	5.03	58868.2	57189.7	57801.1	58683.3	63034.6	61822.0	62403.4	60256.2	55073.4	53920.4	115677.4	140900.1	133738.5	138600.7
Q8BM72	Heat shock 70 kDa protein 13	Hspa13	471	51.7	5.63	247819.3	261967.4	240370.2	238171.9	222823.0	235370.9	227606.9	244773.1	234885.0	243927.7	107068.7	127409.1	102313.6	113391.4
Q9CPT4	Myeloid-derived growth factor	Mydgf	166	18	6.79	649940.9	644062.2	624412.6	655012.0	532856.9	575704.5	517277.6	670125.1	623768.3	590929.2	636579.0	617376.1	608411.2	615237.6
Q9DAU1	Protein canopy homolog 3	Cnpy3	276	30.5	5.62	313532.9	333824.9	304726.5	342590.8	265831.7	285514.2	280992.4	302435.6	323264.1	310274.7	169056.7	166018.7	178307.2	187442.9
Q62093	Serine/arginine-rich splicing factor 2	Srsf2	221	25.5	11.85	1323940.5	1599829.2	1664415.0	1493536.9	1809233.5	1709079.1	1560213.0	1588770.4	1587314.5	1638692.4	1206216.0	1341164.8	1232024.4	1204520.4
Q8BHW2	Protein OSCP1	Oscp1	379	43.2	7.2	131016.2	153490.0	139307.3	173264.6	131731.5	129333.0	108616.0	127280.6	139824.0	149636.9	229103.5	273157.2	290082.3	318024.4
Q9QYG0	Protein NDRG2	Ndrg2	371	40.8	5.4	50492.1	44821.7	48755.4	46682.7	46033.6	48523.2	55755.1	45216.1	41959.6	49383.2	232623.7	233014.4	199651.6	242176.4
Q91YE7	RNA-binding protein 5	Rbm5	815	92.3	6.21	332048.1	363674.8	323765.2	337341.5	284964.5	297032.6	287412.9	270536.5	332687.4	333878.0	450572.0	465262.0	444077.0	483849.9
Q920L1	Acyl-CoA (8-3)-desaturase	Fads1	447	52.3	9.29	540785.0	489150.4	405974.1	498173.1	386010.7	402051.6	389817.6	482265.3	400405.0	398605.0	88283.1	78598.4	81434.0	93170.4
Q6ZQM8	UDP-glucuronosyltransferase 1-7C	Ugt1a7c	531	59.7	8.35	277292.1	274231.4	214309.2	290562.5	235117.2	231737.6	259090.1	277264.1	228791.1	245390.8	605871.5	563657.8	414627.4	654913.0
Q60953	Protein PML	Pml	885	98.2	5.63	100683.1	104051.7	109072.1	109928.7	107566.9	115440.3	122494.7	110880.7	102818.0	113405.9	57798.2	51857.2	48574.3	60017.8
Q8C1D8	Protein IWS1 homolog	Iws1	766	85.2	4.67	152817.7	187498.9	146746.4	173811.5	171991.4	166206.9	134346.3	150861.7	163087.8	178082.8	261602.5	314166.8	290305.8	286576.8
Q9D7X3	Dual specificity protein phosphatase 3	Dusp3	185	20.5	6.54	254228.2	254860.5	186795.3	226326.4	241463.8	240625.9	207291.8	209082.8	222010.8	254375.8	204586.6	213886.9	182824.8	198312.4
Q9DC28	Casein kinase I isoform delta	Csnk1d	415	47.3	9.74	40980.1	42365.2	39356.5	34604.0	36067.5	40481.7	37345.6	40213.1	40408.9	38333.7	101730.7	87990.3	94974.5	100328.9
P70349	Histidine triad nucleotide-binding protein 1	Hint1	126	13.8	6.87	339590.4	530852.4	415428.6	407332.1	402928.1	433542.9	346604.4	381615.9	362709.4	394444.3	1339763.4	1465430.8	1733964.0	1498915.2
Q8BIP0	Aspartate--tRNA ligase, mitochondrial	Dars2	653	74.1	6.98	254709.7	250268.9	251410.7	251142.7	255180.4	256900.7	239277.8	235666.0	239373.1	254296.2	606866.0	654918.8	644197.9	658106.2
Q7TNB8	Protein strawberry notch homolog 2	Sbno2	1349	149.2	6.65	40119.1	43265.2	43401.3	39778.0	44537.7	41268.3	40231.1	34610.9	44745.1	47875.5	#N/A	#N/A	#N/A	#N/A
Q68FH4	N-acetylgalactosamine kinase	Galk2	458	50.5	6.9	192011.6	196073.9	170273.8	171358.2	194447.4	188173.4	196101.7	172619.5	167199.0	194789.3	237119.4	217634.3	224777.2	214110.2
Q9Z127	Large neutral amino acids transporter small subunit 1	Slc7a5	512	55.8	7.9	209923.4	211491.6	211130.6	201325.9	215508.8	205517.0	226052.4	203269.6	223359.9	218456.6	69397.0	70785.8	72363.7	68796.9
Q8CGF6	WD repeat-containing protein 47	Wdr47	920	102.2	5.97	214105.8	233021.4	233050.8	220199.6	231537.3	229675.8	209714.3	195561.5	233424.6	221905.5	313824.0	363237.5	353895.1	342943.5
Q9WU63	Heme-binding protein 2	Hebp2	205	23	4.48	158731.7	159395.4	136299.0	142733.3	137566.2	142835.6	159038.5	148277.2	161088.6	157162.5	129212.6	120265.9	125691.4	119853.2
Q920Q4	Vacuolar protein sorting-associated protein 16 homolog	Vps16	839	94.9	7.01	192030.1	179647.5	181355.4	192313.4	200606.5	191847.7	189622.8	183290.3	188073.8	192213.8	131098.6	152841.4	122889.2	124891.9
Q9Z148	Histone-lysine N-methyltransferase EHMT2	Ehmt2	1263	138	5.83	103867.1	103483.6	85611.0	89341.4	103308.3	98564.2	100869.6	93272.4	96855.2	104997.6	254234.4	261945.5	228683.8	239535.7
O88665	Bromodomain-containing protein 7	Brd7	651	74	6.38	28837.8	29923.7	29393.1	30020.7	25766.7	30435.9	23862.5	23990.5	26749.3	29385.7	21280.9	25232.0	24374.1	27054.3
Q3TJD7	PDZ and LIM domain protein 7	Pdlim7	457	50.1	8.47	411921.3	427421.8	390118.2	396152.6	495182.4	490704.0	509085.1	584503.0	419969.4	408622.1	359803.8	333153.7	346768.1	318795.5
Q80XN0	D-beta-hydroxybutyrate dehydrogenase, mitochondrial	Bdh1	343	38.3	9.01	323689.5	294451.8	284401.7	267167.8	330550.7	354293.2	288773.4	308972.5	299986.0	290640.1	835284.1	767379.0	675322.8	656239.0
Q9ERB0	Synaptosomal-associated protein 29	Snap29	260	29.6	5.38	66283.0	61552.2	72933.0	68994.3	66295.0	70384.9	67969.0	67674.6	67807.2	61840.0	85578.7	111884.1	102805.7	104370.8
Q9D0F6	Replication factor C subunit 5	Rfc5	339	38.1	7.81	142461.9	146478.6	143641.9	146168.9	150904.4	159127.5	150946.1	141466.0	142406.9	155273.5	124838.9	141463.2	133504.9	138370.0
Q62384	Zinc finger protein ZPR1	Zpr1	459	50.7	4.78	155995.8	126146.8	150165.4	137985.9	143136.2	145015.2	143392.8	146232.0	138760.6	136675.2	206651.8	171542.6	189442.5	182226.8
Q921N6	Probable ATP-dependent RNA helicase DDX27	Ddx27	760	85.9	9.25	321126.0	381373.1	307221.9	322840.2	326868.7	343277.8	267947.7	335083.8	321751.9	321124.3	186933.2	239587.4	175062.2	206314.8
P61406	Telomerase-binding protein EST1A	Smg6	1418	160.4	6.77	71823.7	78190.9	75849.6	78463.9	78008.8	76949.4	70649.5	72436.9	78642.7	71957.4	165252.7	172881.6	181597.2	149775.7
Q9QZN4	F-box only protein 6	Fbxo6	295	34.5	8.18	193300.4	214716.3	202170.2	207750.1	231666.6	220148.0	208666.0	183707.5	207931.6	222847.2	169455.1	193500.5	184056.7	169858.4
Q7TQK4	Exosome complex component RRP40	Exosc3	274	29.5	8.06	97240.0	108719.2	101872.0	94106.6	118667.9	110532.1	103221.7	106700.3	103132.3	101231.0	132353.9	122914.9	133448.7	126526.6
Q99JF5	Diphosphomevalonate decarboxylase	Mvd	401	44	6.3	292753.0	366331.1	289772.4	336804.8	315017.5	300984.4	267600.6	276105.8	325508.2	393837.4	257300.1	426764.4	272945.3	342579.0
Q922H4	Mannose-1-phosphate guanyltransferase alpha	Gmppa	420	46.2	7.62	173065.3	154650.2	164703.0	180888.0	146910.6	181087.9	148985.7	169634.5	143921.0	147622.3	208857.8	243688.8	221018.6	233404.7
P16254	Signal recognition particle 14 kDa protein	Srp14	110	12.5	10.17	455545.5	489323.6	527690.6	451087.8	501958.3	480945.3	436933.3	459640.1	516221.0	496631.9	274333.8	336844.7	309178.2	342726.2
Q62422	Osteoclast-stimulating factor 1	Ostf1	215	23.8	5.68	257310.5	285547.5	267535.0	284603.2	283810.7	291522.2	246586.9	291586.7	280915.6	264797.8	161511.2	152825.4	114276.7	153001.6
Q9WUU7	Cathepsin Z	Ctsz	306	34	6.6	363752.5	307701.3	313213.2	309449.3	386033.4	403286.3	454413.7	331806.3	333050.0	355496.5	22250.9	54236.3	19665.4	40034.7
Q3U0V2	Tumor necrosis factor receptor type 1-associated DEATH domain protein	Tradd	310	34.6	5.2	127043.0	136255.9	110481.3	123687.0	119280.6	126938.4	129025.4	127547.7	130013.9	122880.6	66339.6	70539.3	67514.4	73057.6
O70325	Phospholipid hydroperoxide glutathione peroxidase	Gpx4	197	22.2	8.46	185107.9	222716.7	186604.4	192280.1	182278.6	179157.2	165392.8	173184.5	167232.5	185693.0	714169.0	748185.2	748616.9	825595.2
Q8CB77	Elongin-A	Eloa	773	87.1	9.61	213399.6	229476.6	227558.8	233839.7	225971.5	212331.7	210712.5	198696.8	230197.3	232352.6	243628.3	227573.8	247110.6	235317.8
Q3UHH1	Zinc finger SWIM domain-containing protein 8	Zswim8	1832	196.9	6.84	154232.1	155932.9	146366.4	150718.6	139782.6	163275.4	141080.3	145169.7	142591.6	153083.7	92259.5	95244.3	85563.1	88002.5
P61924	Coatomer subunit zeta-1	Copz1	177	20.2	4.81	179239.4	203637.9	197350.6	197005.4	199565.5	183719.2	179952.6	188519.2	199995.1	202147.6	275083.9	266449.1	296623.5	268249.5
P14115	60S ribosomal protein L27a	Rpl27a	148	16.6	11.12	724711.1	919018.1	969125.0	894946.1	797661.8	800224.2	896765.1	958352.8	845700.0	848024.2	1349629.2	1507621.7	1805130.9	1549385.2
Q6ZQK5	Arf-GAP with coiled-coil, ANK repeat and PH domain-containing protein 2	Acap2	770	87.2	6.68	231239.6	220143.0	229268.1	242332.1	246244.1	245679.0	241518.7	242098.7	234160.1	238433.2	98568.6	88674.5	81503.4	81283.7
Q8K2G4	Bardet-Biedl syndrome 7 protein homolog	Bbs7	715	80.3	5.81	78683.7	95402.4	81599.4	94295.4	80669.9	76979.7	71923.6	71796.7	77975.7	85670.2	57266.6	58972.7	56797.7	59022.9
O88708	Origin recognition complex subunit 4	Orc4	433	50	7.15	144227.2	160256.4	154675.4	150041.7	172213.8	168458.0	158643.0	146697.0	152174.3	166799.9	98953.9	128710.1	107182.7	112315.5
Q3USH5	Splicing factor, suppressor of white-apricot homolog	Sfswap	945	104.1	8.05	161234.3	161486.2	160767.9	156718.1	161496.6	151927.2	160583.3	141837.9	155073.6	168201.8	148560.0	175407.8	150683.4	164504.3
P53986	Monocarboxylate transporter 1	Slc16a1	493	53.2	7.47	447284.5	387714.3	436147.2	395588.7	577456.4	584305.3	475392.8	459823.0	404067.6	365655.7	257272.7	245542.2	253668.3	238595.1
Q9D3D9	ATP synthase subunit delta, mitochondrial	Atp5f1d	168	17.6	5.08	31561.6	32590.8	21203.3	25766.3	24111.2	19983.4	16792.3	24927.1	15536.3	20651.9	229336.6	200657.5	235215.7	217579.4
Q9CQI7	U2 small nuclear ribonucleoprotein B''	Snrpb2	225	25.3	9.72	209979.4	212624.3	219396.9	218138.7	218234.1	197916.4	186703.1	224318.5	216272.9	196550.1	89286.0	77182.1	89249.7	73337.5
Q9D0T1	NHP2-like protein 1	Snu13	128	14.2	8.46	649786.7	601105.4	641052.4	589076.2	687301.1	717467.0	806279.4	715258.9	690259.1	654633.6	347652.0	327776.5	384696.3	332166.4
Q3TQQ9	Uncharacterized protein C1orf112 homolog	IGI:359055	903	101.2	6.84	99765.2	116965.1	104563.3	127209.3	116429.3	122136.4	102033.4	104394.0	125196.9	123704.5	117427.0	136314.0	134382.9	122237.7
Q8R1F5	Putative hydroxypyruvate isomerase	Hyi	277	30.4	6.43	124896.5	132938.4	109659.7	104749.7	132901.0	111641.5	146998.4	104807.2	129992.0	122726.9	96612.5	116389.4	80013.9	94168.2
Q62252	Sperm surface protein Sp17	Spa17	149	17.3	4.83	57672.1	76400.8	79414.1	103888.1	72978.7	64583.3	62882.4	63290.0	70884.5	71214.3	122262.8	150650.6	148486.4	160176.6
P34914	Bifunctional epoxide hydrolase 2	Ephx2	554	62.5	6.19	168178.7	172084.0	159209.9	178531.4	173368.3	171275.0	178327.6	186688.1	163020.2	176865.4	133164.8	141934.3	134482.1	137453.6
Q9CY66	H/ACA ribonucleoprotein complex subunit 1	Gar1	231	23.5	11.02	507889.8	468102.0	508646.3	461746.8	567266.0	539608.0	537117.4	483030.0	499816.7	493237.9	504259.1	442956.5	478110.2	425843.7
Q8CFJ7	Solute carrier family 25 member 45	Slc25a45	288	31.7	9.1	464242.9	495663.1	478113.3	498404.6	478260.7	449460.9	461059.6	460771.2	485971.2	474102.3	357565.4	350919.7	356106.0	347169.6
Q8VCD6	Receptor expression-enhancing protein 2	Reep2	254	28.4	9.41	34745.8	35910.2	29917.2	25738.0	29718.0	36152.2	28832.7	33179.2	28972.5	29603.3	220821.6	208466.9	208025.4	198995.7
Q99K23	Ufm1-specific protease 2	Ufsp2	461	52.5	6.76	141661.9	145825.8	142584.8	147435.7	140361.5	123324.5	128144.9	135758.1	144106.7	152361.4	121561.1	197726.7	218231.5	206156.1
Q6P5G6	UBX domain-containing protein 7	Ubxn7	467	52.1	5.03	92310.9	94622.8	98843.8	99388.2	109007.8	109530.1	102260.2	90109.1	96350.7	97479.2	139417.1	148150.5	140076.2	128768.8
Q9JK83	Partitioning defective 6 homolog beta	Pard6b	371	41	5.59	144455.6	149560.8	123317.8	137518.5	126570.9	122840.4	128023.7	125851.6	114893.4	142132.4	66871.9	59511.7	69586.7	51282.2
Q9WVB0	RNA-binding protein with multiple splicing	Rbpms	197	21.8	8.07	170248.5	175758.5	191284.4	171445.5	230856.5	263916.3	217176.4	207690.7	215882.8	191881.8	134599.7	159766.5	152608.1	149118.6
P10922	Histone H1.0	H1-0	194	20.8	10.9	1562111.1	1234667.7	1459435.7	1280312.7	1479201.5	1609814.8	1422676.8	1624988.0	1326494.7	1366685.3	1885994.9	1459470.7	1872476.0	1438260.5
P56391	Cytochrome c oxidase subunit 6B1	Cox6b1	86	10.1	8.72	678031.0	682672.7	694345.0	678219.5	733665.3	678755.1	732852.2	726492.3	721341.0	727501.4	736574.0	757043.3	777901.0	718668.5
P48453	Serine/threonine-protein phosphatase 2B catalytic subunit beta isoform	Ppp3cb	525	59.1	5.91	333856.7	335155.9	338125.2	324043.8	357744.1	359039.3	335908.7	343771.1	380663.9	365329.2	149250.5	140301.1	138441.1	141075.9
Q8CHR6	Dihydropyrimidine dehydrogenase [NADP(+)]	Dpyd	1025	111.2	7.27	154666.0	169084.7	149750.8	151475.2	182403.0	166976.8	161390.5	149687.9	151092.2	158753.2	130507.8	141622.1	139575.8	136150.8
P83940	Elongin-C	Eloc	112	12.5	4.78	212059.1	208820.6	229674.1	205721.1	222909.6	219891.2	198015.6	202436.0	218946.5	222151.1	239076.3	324922.0	300762.6	310767.1
Q64520	Guanylate kinase	Guk1	198	21.9	6.55	75038.1	60147.5	70960.8	78710.1	77022.3	82855.3	72190.3	71503.4	72403.5	65080.4	69027.8	77482.8	61577.9	70043.8

Q80UK8	Integrator complex subunit 2	Ints2	1198	133.4	6.11	185031.5	182810.5	188823.7	182286.4	196798.2	194739.2	177480.2	172310.5	185456.9	207930.5	163763.4	160446.6	163946.3	159713.8
Q8VEH8	Endoplasmic reticulum lectin 1	Erlec1	483	54.9	6.25	98751.0	110470.2	100889.1	114620.3	105031.5	105789.1	98201.8	100532.4	102690.7	114268.1	142813.4	152579.1	155020.2	162521.1
Q99KB8	Hydroxyacylglutathione hydrolase, mitochondrial	Hagh	309	34.1	7.75	120906.4	127628.3	154872.8	160174.0	109828.1	110134.1	102851.8	122117.5	123542.5	126801.4	59528.1	62958.9	70003.3	74430.5
Q8VI33	Transcription initiation factor TFIID subunit 9	Taf9	264	29	7.91	174509.8	193866.6	198101.7	194044.1	210021.0	182282.3	168342.7	171864.0	199238.5	200984.6	183376.6	186243.1	185813.7	185099.6
E9Q1P8	Interferon regulatory factor 2-binding protein 2	Irf2bp2	570	59.3	8.69	54812.2	54692.4	55052.0	52165.7	58852.4	61744.0	60365.5	54004.1	51735.7	55920.2	48301.9	51408.3	53761.3	47985.5
Q9Z120	tRNA (guanine-N(7)-)-methyltransferase	Mettl1	268	30.6	7.34	75011.4	79995.8	74873.7	76984.2	89697.5	85254.8	78216.6	78503.7	78135.5	79412.0	112992.5	110323.1	102565.4	98661.3
Q8BYB9	Protein O-glucosyltransferase 1	Poglut1	392	46.4	8.85	159973.8	182459.6	162492.4	168122.7	180361.7	199547.5	177209.6	176415.8	180586.2	181002.6	250930.6	251868.4	235777.3	219557.9
Q8C0E2	Vacuolar protein sorting-associated protein 26B	Vps26b	336	39.1	7.37	336018.4	311809.9	311501.2	291695.1	339024.8	348465.1	355332.8	327632.9	328971.5	314469.7	417524.0	400730.2	391173.9	362617.4
Q8K2X3	CST complex subunit STN1	Stn1	378	43.5	5.77	274158.8	279876.3	264502.4	244646.6	282903.0	302758.5	318672.6	250843.6	292375.9	295568.3	51821.3	71080.0	60014.6	58729.2
Q9Z1T6	1-phosphatidylinositol 3-phosphate 5-kinase	Pikfyve	2097	236.7	6.68	250619.8	274660.6	267888.1	239285.1	317849.1	312347.6	291438.6	271369.1	283878.0	291806.0	34290.4	31727.6	30117.9	30330.4
Q3UM18	Large subunit GTPase 1 homolog	Lsg1	644	73.1	6.51	133215.2	160094.6	131266.2	147622.7	138961.1	138651.4	125698.2	134058.5	140221.8	143794.1	227888.0	238401.1	202969.4	237955.4
Q9D024	Coiled-coil domain-containing protein 47	Ccdc47	483	55.8	4.84	236742.1	239651.8	221264.9	225709.9	241299.9	199923.6	206821.3	218671.9	231874.7	235701.8	131948.5	144796.9	143310.8	141630.9
P56395	Cytochrome b5	Cyb5a	134	15.2	5.07	541832.8	755568.1	481819.0	549236.2	604040.8	568305.9	499254.6	513258.1	544233.1	632915.0	1042850.4	1110699.9	896793.4	988720.5
Q8K114	Integrator complex subunit 9	Ints9	658	74	6.4	298503.6	303738.6	288045.4	294214.9	286730.4	295365.2	277299.2	269480.1	299505.9	320116.5	222256.0	204486.8	199045.7	219169.6
Q5F2E8	Serine/threonine-protein kinase TAO1	Taok1	1001	116	7.55	147755.2	164211.8	125571.4	138278.3	147596.8	147281.2	128779.2	135140.8	132876.5	139267.5	235405.8	237187.1	197112.3	211084.5
P40630	Transcription factor A, mitochondrial	Tfam	243	28	9.69	663259.0	537304.4	641862.9	555175.6	679504.1	668354.6	726123.3	639200.0	641207.8	593325.4	600488.3	539019.5	548139.2	522967.8
Q8VEH5	EPM2A-interacting protein 1	Epm2aip1	606	70.1	5.87	110340.9	116758.9	104790.5	109935.4	114015.5	126345.8	121072.9	111446.3	118097.2	121700.1	77132.4	80270.4	77147.9	66992.7
Q61206	Platelet-activating factor acetylhydrolase IB subunit beta	Pafah1b2	229	25.6	5.92	229211.7	219528.0	222383.7	211103.1	267902.4	248895.0	205217.7	227985.1	224370.5	247971.6	1646245.9	1587050.7	1758987.1	1596464.0
O70456	14-3-3 protein sigma	Sfn	248	27.7	4.78	46546.1	55737.0	51774.4	52865.8	47608.3	57850.2	51347.3	41877.6	48858.3	45946.6	38060.1	40996.9	33580.6	33854.3
Q03958	Prefoldin subunit 6	Pfdn6	127	14.4	8.88	650404.1	720118.5	652130.8	665890.1	711279.5	655368.1	603622.1	683953.5	685538.9	702427.8	544497.7	619718.4	635028.1	615410.5
Q9JI13	Something about silencing protein 10	Utp3	469	53.4	5.54	88413.5	82653.3	87983.2	82510.6	96009.4	91134.3	76362.6	84209.4	85621.1	87313.9	42238.0	51688.5	48529.8	45912.0
Q8VE11	Myotubularin-related protein 6	Mtmr6	617	70.9	7.83	89703.4	118478.3	102323.2	122877.2	110698.2	104083.3	88905.9	96964.2	96399.5	105786.2	139292.8	152725.3	133934.8	150337.3
G5E897	Protein O-glucosyltransferase 3	Poglut3	503	57.7	7.74	339877.0	279317.9	281495.3	284114.6	260027.0	317792.5	296381.7	338002.2	290754.0	290968.3	101867.5	100186.7	88652.6	97439.3
Q8R3V5	Endophilin-B2	Sh3glb2	400	44.5	5.82	93126.4	114180.0	99590.7	106665.1	112288.3	119645.9	95462.9	101010.7	104111.2	104228.9	217733.1	227401.5	212480.5	220443.9
Q64008	Ras-related protein Rab-34	Rab34	259	29.1	8.27	136457.7	132201.4	123163.2	133816.1	149811.6	147786.8	142765.6	141053.6	142393.0	139847.5	179145.9	169619.8	179156.7	150707.0
Q8BWR2	PITH domain-containing protein 1	Pithd1	211	24.2	5.74	121153.3	154952.6	129570.7	133167.6	134967.3	134675.9	120550.4	128208.8	128360.4	140099.7	147297.1	201382.6	154227.6	179968.7
Q61211	Eukaryotic translation initiation factor 2D	Eif2d	570	62.8	8.24	63555.9	75400.9	51139.4	66102.3	72509.2	65334.1	55762.0	58297.0	53807.3	67681.2	46454.2	55587.6	42305.1	47851.7
Q9CXJ1	Probable glutamate--tRNA ligase, mitochondrial	Ears2	523	58.3	8.75	119518.0	112347.0	120285.3	113998.9	119452.8	112897.4	103337.4	105723.2	121491.7	122502.0	78629.2	102950.6	71054.3	84367.9
P05977	Myosin light chain 1/3, skeletal muscle isoform	Myl1	188	20.6	5.03	68433.9	80003.3	79898.7	78442.5	85559.3	80143.8	76423.5	140739.4	81122.5	73443.1	50850.6	51795.7	44476.2	45741.6
Q8BJD1	Inter-alpha-trypsin inhibitor heavy chain H5	Itih5	952	106.7	8.16	95055.5	105500.5	95159.5	98907.8	112579.5	116160.5	105825.0	110113.5	93077.4	103669.0	271712.0	207292.4	258620.6	222672.4
P56546	C-terminal-binding protein 2	Ctbp2	445	48.9	6.95	131300.9	129795.3	140593.1	123699.7	156565.7	202840.7	186651.5	172549.0	140412.0	140801.3	220034.3	186933.2	201536.5	180509.2
Q62028	Secretory phospholipase A2 receptor	Pla2r1	1487	170.4	6.62	68455.7	70160.9	57431.2	59929.4	68639.6	81230.1	77961.9	65084.8	57910.0	62990.9	39524.0	37783.9	38134.3	35783.6
P35288	Ras-related protein Rab-23	Rab23	237	26.7	6.79	140090.0	150317.0	148559.9	158740.8	156334.4	168923.9	146840.1	158984.0	158838.0	151166.5	181439.7	190726.6	179807.3	187905.6
B2RUM3	CHCH domain-containing protein	AU015836	263	30.5	3.76	257876.7	209879.3	315589.7	219170.8	332539.8	311270.9	338570.7	272969.6	282010.4	246968.4	431999.0	386799.3	463186.6	387372.0
Q9D753	Exosome complex component RRP43	Exosc8	276	29.9	5.2	129965.9	129474.9	126983.2	129471.4	141117.6	144960.9	123877.0	126187.6	141349.8	134462.6	76974.1	106107.3	72133.9	105514.7
Q7TSI3	Serine/threonine-protein phosphatase 6 regulatory subunit 1	Ppp6r1	856	94.5	4.64	225120.2	237828.6	215404.9	230805.8	214622.0	201622.7	181577.8	203626.1	217111.0	229888.6	135388.2	131533.1	131080.3	124179.7
Q6NZQ2	Probable ATP-dependent RNA helicase DDX31	Ddx31	687	76.9	9.74	205475.7	221088.2	209045.7	200122.8	233385.3	217943.4	196274.3	181936.9	200979.2	232807.6	79565.0	83686.1	82456.7	82791.8
Q6PE54	Probable ATP-dependent RNA helicase DHX40	Dhx40	779	88.5	8.63	201916.1	240064.3	199967.4	212838.5	226550.6	232404.7	205798.9	184655.9	207725.9	241564.7	145986.1	203657.0	160948.6	168469.6
Q9D787	RING-type E3 ubiquitin-protein ligase PPIL2	Ppil2	521	59	8.38	298141.9	335480.0	331009.7	309421.5	336778.8	331588.3	328828.0	305718.8	339350.8	359869.0	273550.7	276319.2	261527.0	290896.3
P52795	Ephrin-B1	Efnb1	345	37.8	9.03	81147.5	86589.7	75448.9	79377.5	76624.9	82807.8	84039.9	83015.2	82060.0	82189.2	174270.5	151095.3	171762.2	138640.8
Q9R0Y5	Adenylate kinase isoenzyme 1	Ak1	194	21.5	5.81	98902.6	101683.8	88410.9	88593.1	98783.2	113059.8	148877.6	109881.4	94157.9	93005.4	139345.1	131687.3	117442.7	136207.9
Q62193	Replication protein A 32 kDa subunit	Rpa2	270	29.7	6.15	270885.1	337213.8	302238.2	306378.9	307662.9	284350.1	269524.0	275178.5	290159.0	323209.6	645296.1	741224.8	594590.3	684955.8
Q9JMD0	BUB3-interacting and GLEBS motif-containing protein ZNF207	Znf207	495	52.8	9.1	251774.4	273821.5	243513.2	279315.2	231744.7	236041.1	216248.8	223004.2	253226.6	261658.3	375480.2	353412.8	320132.4	373340.2
Q3TKY6	Spliceosome-associated protein CWC27 homolog	Cwc27	469	53.5	5.53	215358.3	219683.9	220302.4	207495.1	228880.3	222805.2	223855.3	196715.0	236269.9	241921.4	485237.3	474634.1	402101.8	455277.2
Q8C165	N-fatty-acyl-amino acid synthase/hydrolase PM20D1	Pm20d1	503	55.6	6.43	86020.9	74775.0	86572.2	71504.7	97873.7	100708.6	97281.6	79520.5	93201.7	82095.7	112152.9	102954.0	104765.7	90328.4
O70348	Decapping and exoribonuclease protein	Dxo	397	45.3	8.06	129962.6	123160.0	108554.4	116685.5	118735.8	109472.2	105575.7	106972.9	125822.5	134673.5	219746.8	211289.8	180556.6	207980.9
Q3UN02	Lysocardiolipin acyltransferase 1	Lclat1	376	44.4	8.53	469442.0	397989.8	411071.1	415717.7	377147.2	356217.1	472835.3	376498.1	422648.4	449278.8	202997.2	224783.8	213711.3	235036.0
Q9WU40	Inner nuclear membrane protein Man1	Lemd3	921	100.2	7.55	53258.4	56720.1	57173.2	55831.5	57770.2	67605.1	57040.8	55347.9	55336.7	62388.7	222814.9	260966.5	210693.2	233963.5
Q9JM96	Cdc42 effector protein 4	Cdc42ep4	349	37.8	5.36	58930.9	74371.4	60735.0	64805.1	68495.3	69130.7	69545.3	56922.2	67149.6	66426.3	85160.7	86792.4	70830.1	83094.5
Q8BYK6	YTH domain-containing family protein 3	Ythdf3	585	63.9	9.04	283200.5	243301.8	258828.0	274158.7	268598.2	263852.1	280818.9	255434.3	273418.1	271647.0	218937.0	171060.7	182641.5	172101.0
Q8VBV3	Exosome complex component RRP4	Exosc2	293	32.6	7.5	178068.6	153560.0	154153.2	174568.3	143138.5	134424.2	140352.8	135782.9	182911.3	155619.8	104972.4	110465.8	106948.5	106370.1
Q6PE15	Mycophenolic acid acyl-glucuronide esterase, mitochondrial	Abhd10	297	33	8.79	434103.4	474745.9	461630.3	460434.7	486848.0	478546.7	467519.8	450483.2	511820.1	491624.4	347617.6	349981.6	351903.8	344394.1
Q8VHY0	Chondroitin sulfate proteoglycan 4	Cspg4	2327	252.2	5.44	117887.5	127875.3	132303.4	115391.9	122414.5	135642.0	131943.1	118400.7	112540.9	139410.9	211712.8	202432.6	183929.2	209030.3
Q7TN29	Stromal membrane-associated protein 2	Smap2	428	46.5	8.87	127017.0	151095.3	148388.7	148672.0	129455.7	129678.7	113644.5	132992.4	138287.6	130330.3	41835.8	43011.4	46216.5	45433.2
Q9DB27	Malignant T-cell-amplified sequence 1	Mcts1	181	20.5	8.82	391477.4	384762.2	357324.4	379333.4	371190.8	417172.0	439128.2	467724.1	381867.0	367156.7	345237.8	329507.3	302383.1	313922.3
P70452	Syntaxin-4	Stx4	298	34.1	6.14	176490.3	205207.2	162990.8	199713.2	193794.1	182110.3	201997.6	181432.4	182641.3	190288.5	222278.6	220626.5	197731.9	242460.5
Q9JKK7	Tropomodulin-2	Tmod2	351	39.5	5.35	127352.5	131979.4	140114.1	135399.8	149946.2	149575.3	166935.8	147072.7	126350.9	138088.9	49467.7	47480.2	45582.7	42012.4
P11859	Angiotensinogen	Agt	477	52	5.44	65823.7	71009.4	58006.9	60345.3	60409.7	74878.9	60291.7	69996.4	66923.2	66336.0	66358.1	63540.5	57239.9	59329.5
Q6NVE8	WD repeat-containing protein 44	Wdr44	915	101.5	5.24	107720.1	111940.3	115937.9	114359.0	119149.0	125390.0	114126.6	106580.3	119484.0	119419.6	119490.4	120942.5	123060.1	117622.1
Q9CXL3	Uncharacterized protein C7orf50 homolog	MGI:192046	195	22.2	9.39	59734.7	65229.0	68423.1	60601.8	70201.2	74714.4	79011.1	69632.5	69744.5	70192.5	171328.1	147279.8	189726.6	156202.7
Q99J95	Cyclin-dependent kinase 9	Cdk9	372	42.7	8.79	57914.4	57442.0	49872.5	51846.1	61469.5	64529.8	51077.0	60036.8	49241.5	57401.4	428513.2	491692.1	420465.2	431473.0
Q7TNG5	Echinoderm microtubule-associated protein-like 2	Eml2	649	70.7	6.28	93269.5	103121.2	92518.4	98288.5	98090.8	102642.0	95528.2	94335.4	102138.3	92926.7	253717.8	238527.2	246526.2	236469.0
Q8R3H9	Tetratricopeptide repeat protein 4	Ttc4	386	44.3	5.22	181282.4	198269.8	236475.0	208501.6	217482.7	230729.3	196313.4	194339.1	225441.4	201609.1	43306.4	48585.8	51146.6	50905.5
Q9JK30	Origin recognition complex subunit 3	Orc3	715	82.3	7.4	114394.3	176008.9	116176.1	130315.3	155313.0	139867.3	120991.4	135935.3	139332.2	154799.1	70969.9	80688.3	83243.2	70332.0
Q9CQF0	39S ribosomal protein L11, mitochondrial	Mrpl11	192	20.7	9.73	118614.8	136719.0	133217.6	131831.1	143640.1	129940.8	122162.7	139136.0	123651.0	143811.3	57915.5	61131.9	55318.0	48274.1
Q7TSE6	Serine/threonine-protein kinase 38-like	Stk38l	464	53.7	6.96	155461.4	162342.3	122416.4	167459.7	171980.4	159363.0	150147.4	155724.6	159844.1	165880.5	238844.3	264776.3	256322.3	228863.7
Q9DCL8	Protein phosphatase inhibitor 2	Ppp1r2	206	23.1	4.83	24030.3	28332.6	24474.4	25964.3	23900.2	24512.2	19794.3	21855.0	23551.6	25328.9	74446.6	87407.1	84910.2	109131.3
Q61687	Transcriptional regulator ATRX	Atrx	2476	278.4	6.68	61388.3	56744.7	57686.8	59169.6	67876.3	76285.8	77731.8	67443.3	62271.4	66681.5	235463.1	243654.7	203100.3	215280.3
Q99PP2	Zinc finger protein 318	Znf318	2237	246.2	7.01	159730.2	175022.6	183691.9	190265.0	202792.9	191981.1	156111.3	154412.5	171315.1	179322.8	186961.3	201299.7	189857.5	182899.0
Q3TBW2	39S ribosomal protein L10, mitochondrial	Mrpl10	262	29.4	9.69	53896.3	57432.4	56917.3	60208.7	59566.3	63803.8	58524.4	59133.3	60510.5	63550.9	137039.7	112908.0	101658.0	90749.6
P36552	Oxygen-dependent coproporphyrinogen-III oxidase, mitochondrial	Cpox	443	49.7	8.53	293687.4	307296.1	289668.1	291608.2	269502.9	277161.3	279353.4	298199.7	288368.8	292584.9	266744.3	336878.9	268640.2	290539.8
E9QAS7	IPPc domain-containing protein	Inpp5a	422	48.8	7.09	127048.5	133026.3	122151.5	130666.5	140291.8	153732.1	139628.7	131774.5	133927.0	126704.2	163635.7	184861.3	182630.9	172034.3
Q811P8	Rho GTPase-activating protein 32	Arhgap32	2089	229.6	6.86	141159.7	149563.3	140761.3	134271.7	158908.1	150107.7	155248.9	142787.0	154647.3	159432.8	#N/A	#N/A	#N/A	#N/A
Q8R4R6	Nucleoporin NUP35	Nup35	325	34.8	9.25	159406.7	166358.5	172891.0	164654.3	186591.1	190487.7	176901.9	166320.6	165163.9	168381.4	172054.8	175882.5	177942.1	166641.4
Q60596	DNA repair protein XRCC1	Xrcc1	631	68.9	6.33	182398.6	190069.4	211650.7	206465.7	194220.6	183692.0	160881.4	192250.2	181978.2	188599.2	141688.2	150550.1	164833.8	152597.4
P51807	Dynein light chain Tctex-type 1	Dynlt1	113	12.5	5.08	87654.6	96327.6	111588.5	102023.8	89204.5	81795.5	83191.0	95787.0	81815.8	92755.1	37404.6	48074.1	44769.8	54209.2
P63011	Ras-related protein Rab-3A	Rab3a	220	25	5.03	192510.7	172998.5	162167.1	162802.8	175477.9	186238.0	235229.7	165402.7	182133.8	179676.7	168865.4	133811.6	151420.6	144348.3
P08074	Carbonyl reductase [NADPH] 2	Cbr2	244	25.9	9.01	573298.7	594296.2	424359.2	638884.5	434838.6	433705.3	427225.7	507922.5	456417.0	435170.5	666906.0	589300.5	426594.4	682959.7
Q00493	Carboxypeptidase E	Cpe	476	53.2	5.19	147801.5	129070.5	148472.1	140279.9	155922.4	145541.1	145237.8	152715.1	151287.2	139977.2	159442.5	164138.1	169338.7	167669.1
Q9D495	Synaptonemal complex central element protein 1	Syce1	329	38.2	6.38	250209.0	322953.8	273295.7	295447.2	298289.6	283738.6	234868.9	233582.3	304433.0	319574.4	181604.5	201029.4	196396.8	196957.8
Q8R2Q4	Ribosome-releasing factor 2, mitochondrial	Gfm2	779	86.1	6.43	161406.4	168519.8	154514.9	160917.6	159376.4	158330.9	146273.2	157446.8	163501.9	171687.6	104096.1	101598.6	95159.5	101648.6
Q8K0V4	CCR4-NOT transcription complex subunit 3	Cnot3	751	81.9	6.2	303601.7	322562.5	302211.6	321452.1	313689.8	314345.7	265070.6	301030.4	321682.3	290913.9	207628.6	277316.1	227187.3	240785.0
Q9CPN8	Insulin-like growth factor 2 mRNA-binding protein 3	Igf2bp3	579	63.5	8.87	142364.3	155626.8	140943.6	136495.8	147121.1	148921.4	127629.4	144587.7	148863.0	161019.9	165826.3	173461.7	159258.2	169937.8
Q8BLN5	Lanosterol synthase	Lss	733	83.1	6.4	213619.4	239565.9	207478.2	229240.1	222189.4	236947.4	210584.3	225191.4	222063.4	232231.3	291121.4	266700.4	267466.0	293603.4
A2RSY6	TRMT1-like protein	Trmt1l	728	80.8	7.5	134503.0	123649.1	134790.1	134977.0	142146.2	138168.7	127455.8	121117.4	133334.0	138334.9	85651.9	86182.2	82632.0	83045.8
Q9D2D7	Zinc finger protein 687	Znf687	1237	130.3	8.12	34518.5	34902.9	34941.2	33297.2	38512.0	38772.9	36904.2	36723.5	34198.5	36652.7	72933.4	72235.8	65080.1	58937.1
Q9D8B7	Junctional adhesion molecule C	Jam3	310	34.8	7.03	225766.6	234464.1	245326.7	221780.5	235314.1	238047.7	184373.9	213385.6	224073.9	230110.2	183943.0	206483.2	221526.7	206504.2
Q9JLR1	Protein transport protein Sec61 subunit alpha isoform 2	Sec61a2	476	52.2	8.06	43704.5	63561.2	75019.4	58794.5	70478.4	57993.5	52649.5	51876.5	65750.4	65511.7	112150.1	115463.7	114827.7	100789.6
Q00519	Xanthine dehydrogenase/oxidase	Xdh	1335	146.5	7.56	107552.2	123914.7	89864.3	112756.2	120427.5	116018.3	172675.7	119181.7	109167.9	103685.8	133551.6	127800.7	118688.1	125712.5
Q8CFE3	REST corepressor 1	Rcor1	480	52.7	7.03	70559.8	76209.1	65687.3	66221.5	65766.6	63368.3	66982.1	63419.6	60308.7	69964.5	35197.1	35780.2	31561.1	36197.8
Q99MK8	Beta-adrenergic receptor kinase 1	Grk2	689	79.6	7.28	98775.4	112328.5	103530.1	100454.2	112979.4	111808.9	95492.9	97724.0	102180.3	108601.2	194170.2	220931.2	186836.7	201506.8
Q69ZF7	Metal transporter CNNM4	Cnnm4	771	86.6	6.07	182530.4	185602.2	169910.0	181650.0	176460.4	188220.0	169640.7	166522.1	174259.8	185263.3	369539.5	400764.5	373234.1	389395.8

P11404	Fatty acid-binding protein, heart	Fabp3	133	14.8	6.57	1212778.2	1211539.0	874002.7	1137832.6	1067598.7	1443801.8	1351310.5	1114822.1	1122900.0	1033742.5	797864.7	716955.7	651316.3	761229.6
Q91YT7	YTH domain-containing family protein 2	Ythdf2	579	62.2	8.79	415446.2	502687.9	482001.8	491471.4	406896.5	368964.3	398523.9	414704.8	521054.7	470401.1	71631.5	80422.2	81710.7	76107.7
Q9R013	Cathepsin F	Ctsf	462	51.6	6.55	116040.5	113377.8	129892.5	127397.4	151306.5	157529.3	197905.9	135464.6	130273.9	110486.3	156703.5	163116.4	141323.0	153778.5
P08121	Collagen alpha-1(III) chain	Col3a1	1464	138.9	6.52	36991.1	38203.3	42671.8	37480.0	38589.1	50456.6	42780.6	66388.1	45598.1	44157.8	97202.4	96295.6	80734.1	82218.4
P52912	Nucleolysin TIA-1	Tia1	386	42.8	7.74	140393.6	165578.1	160661.2	141093.6	150661.6	151163.5	136216.1	162824.0	137374.3	149592.9	275737.2	321136.6	286771.7	315909.8
Q9R0A1	Chloride channel protein 2	Clcn2	908	99.4	8.56	88666.8	80569.6	81048.0	81528.1	75090.7	77990.1	74007.1	73838.1	83045.6	87822.2	178006.2	171125.8	165597.1	167501.6
Q8R1V4	Transmembrane emp24 domain-containing protein 4	Tmed4	227	26	8.18	576992.1	649688.7	611456.7	750297.0	510678.7	467428.2	472585.8	531983.7	560298.1	610803.8	376308.8	444586.2	397215.0	427061.7
Q9D9K3	Cell death regulator Aven	Aven	342	37.2	4.98	39936.6	48556.1	37178.8	33480.5	33495.3	25016.3	31599.5	24445.1	41183.0	39724.6	122877.1	167111.6	164042.2	176534.3
Q9CR61	NADH dehydrogenase [ubiquinone] 1 beta subcomplex subunit 7	Ndufb7	137	16.3	8.18	107814.4	130523.3	107354.4	111375.4	109066.7	102723.6	95034.6	104443.9	114753.6	115957.5	87814.2	100928.7	91213.4	89190.1
Q3UDP0	WD repeat-containing protein 41	Wdr41	460	51.5	5.15	95135.6	103453.6	105768.5	104581.6	89823.2	85617.8	82158.1	93333.3	92643.7	91566.4	72792.7	73718.2	71285.7	71765.3
Q5SSI6	U3 small nucleolar RNA-associated protein 18 homolog	Utp18	552	61.2	8.78	139393.5	128846.9	137706.1	118271.2	140539.3	156847.9	114204.7	126158.4	120888.3	134747.4	166746.8	178381.1	174060.9	168945.5
Q6NXJ0	Protein WWC2	Wwc2	1187	132.5	5.71	92085.2	99104.4	89450.9	94166.5	90043.1	93286.6	85155.9	91102.7	91134.1	99438.7	54915.7	64184.5	58628.1	58780.2
Q4KMM3	Oxidation resistance protein 1	Oxr1	866	95.9	5.33	60141.4	65715.6	66263.7	62659.5	66747.6	64066.5	62768.8	62404.4	68208.6	63296.0	84829.6	82642.5	72556.0	79126.8
Q9CZT8	Ras-related protein Rab-3B	Rab3b	219	24.7	5.11	155022.2	132552.5	131532.7	112204.6	164788.3	160577.1	194186.0	149908.8	131834.4	139736.3	239608.1	218228.0	210506.7	193701.5
O09131	Glutathione S-transferase omega-1	Gsto1	240	27.5	7.36	450676.1	445893.3	463264.0	413105.1	482648.6	476606.8	485848.0	493351.8	471628.2	486214.1	649558.2	588437.6	611649.2	551514.6
Q9ESV0	ATP-dependent RNA helicase DDX24	Ddx24	857	96.4	9.36	188310.9	199393.0	191813.3	191073.8	182890.1	189255.7	163045.0	179159.3	183608.6	188348.8	129527.0	132497.1	119688.4	120537.4
Q8K1R7	Serine/threonine-protein kinase Nek9	Nek9	984	107.1	5.63	148015.4	154806.1	163474.0	155744.3	162137.2	165109.6	155676.6	156673.7	155396.8	161893.6	314738.4	330862.0	304667.7	315313.5
Q99JR6	Nicotinamide/nicotinic acid mononucleotide adenylyltransferase 3	Nmnat3	245	27.7	8.56	94146.0	110598.2	100771.2	95207.2	103106.6	110414.2	102745.7	100305.2	95494.4	98675.8	96310.3	98721.9	89900.3	109812.7
Q5F2E7	Nuclear fragile X mental retardation-interacting protein 2	Nufip2	692	75.6	8.7	55202.0	52221.9	56594.1	52973.1	57885.7	55826.3	46100.2	62093.0	47241.1	49666.2	130931.8	134971.4	138577.3	130511.5
Q8K358	Phosphatidylinositol glycan anchor biosynthesis class U protein	Pigu	434	49.8	7.72	256949.7	306612.2	272481.2	286995.2	271766.6	263994.1	251417.8	268943.7	296149.8	279996.4	183865.2	194759.7	200300.9	192678.4
Q9D7S9	Charged multivesicular body protein 5	Chmp5	219	24.6	4.79	132510.4	145425.6	137829.1	134002.9	142465.2	144289.4	134719.7	133535.3	134063.9	146941.0	219137.0	212806.2	216406.1	178952.0
F8VPZ3	Ubiquitin-specific peptidase 32	Usp32	1604	181.6	6.51	90144.1	105968.4	104166.9	96433.3	99514.1	106258.0	91543.6	86668.6	95853.6	106786.7	100525.5	108334.0	103440.0	105965.3
Q91Z38	Tetratricopeptide repeat protein 1	Ttc1	292	33.2	5.01	113850.6	119552.0	111434.7	100908.3	107105.7	100584.4	94737.8	95687.8	104949.5	103349.0	269755.6	256914.1	300377.6	258476.5
Q9CX80	Cytoglobin	Cygb	190	21.5	6.8	199562.6	203513.6	217180.3	190279.7	207618.5	261709.6	339454.7	242103.0	227769.8	220945.4	63237.6	73266.8	67677.1	67349.7
Q91X20	Set1/Ash2 histone methyltransferase complex subunit ASH2	Ash2l	623	68.2	5.69	250124.8	201085.7	229299.2	243323.5	241605.6	245590.1	268353.8	245673.8	248778.5	241157.1	330823.7	351662.6	346494.2	373456.9
O88271	Craniofacial development protein 1	Cfdp1	295	32.9	4.86	271091.9	246530.2	239603.3	233793.6	247067.8	249971.4	260288.2	234484.3	244450.2	253305.1	708904.5	830644.4	775665.8	761288.6
Q6P8I4	PEST proteolytic signal-containing nuclear protein	Pcnp	178	19	7.49	711203.7	718547.7	717032.9	706544.8	665310.9	715750.9	690112.9	649256.2	705017.1	708660.8	494109.0	484245.2	538883.6	486735.7
E9Q9B7	ANK_REP_REGION domain-containing protein	Kidins220	1793	199.1	6.67	128730.7	141678.5	121228.9	129067.6	132630.6	138357.0	125350.9	114963.0	138361.0	135133.0	154007.4	154195.1	140494.0	140531.7
Q9D0C4	tRNA (guanine(37)-N1)-methyltransferase	Trmt5	501	56.8	7.91	94483.6	130603.4	112643.2	115246.3	92503.6	96132.9	76688.4	97374.8	107915.6	113824.3	47406.3	42409.4	43380.0	45985.9
Q9JM76	Actin-related protein 2/3 complex subunit 3	Arpc3	178	20.5	8.59	630468.7	660386.6	644928.7	625508.7	624614.3	638824.2	724804.3	676029.8	652592.9	620425.1	628742.7	638726.5	663942.2	671784.7
Q62432	Mothers against decapentaplegic homolog 2	Smad2	467	52.2	6.58	143151.9	154671.2	215568.3	143336.3	172543.4	174381.4	160033.1	145459.1	155047.4	152594.1	182241.3	179574.5	171071.5	192481.5
Q61458	Cyclin-H	Ccnh	323	37.5	7.11	162991.1	184144.1	172790.2	173234.0	174559.3	165481.3	168864.1	160251.7	173688.8	178968.4	47968.3	60984.8	49999.4	55780.6
Q3TUH1	Phosphatidate cytidylyltransferase, mitochondrial	Tamm41	337	37.8	8.98	117572.7	131677.4	127503.9	123359.2	125584.1	123380.5	118987.2	120984.5	124197.0	132423.7	208484.2	216893.0	202934.5	221803.0
Q0P5W1	Vacuolar protein sorting-associated protein 8 homolog	Vps8	1427	161	5.58	278461.0	252475.7	259790.4	256188.0	263941.3	266821.6	276140.6	259320.5	264083.9	261232.8	157056.1	159543.3	158146.6	150247.6
Q6IME9	Keratin, type II cytoskeletal 72	Krt72	520	56.7	7.4	178111.4	214669.9	180380.8	211523.5	254407.3	261217.1	211462.3	202868.6	226673.6	227344.2	33882.5	31456.3	37294.0	41803.6
P04919	Band 3 anion transport protein	Slc4a1	929	103.1	5.45	253808.3	201441.7	182160.3	270775.4	208910.5	208367.9	293667.0	244569.3	248092.7	245435.0	245965.1	211953.1	187914.4	267339.1
P18608	Non-histone chromosomal protein HMG-14	Hmgn1	96	10.1	9.76	49089.9	41155.0	50271.0	42702.1	55542.3	56605.5	61655.7	36208.1	49311.8	47868.8	64362.5	58314.3	72199.0	61342.8
Q9CR86	Calcium-regulated heat stable protein 1	Carhsp1	148	16.1	8.21	382020.2	489843.4	269351.7	377011.8	413024.3	344230.4	369273.0	344335.3	415845.2	404632.8	412072.5	385458.3	373551.6	346689.4
Q99LG4	Tetratricopeptide repeat protein 5	Ttc5	440	48.8	6.29	212782.4	242533.2	220794.4	239323.6	199571.5	202892.9	198107.7	202206.3	194832.7	238140.1	79761.9	77560.3	71228.9	77048.5
Q6PFR5	Transformer-2 protein homolog alpha	Tra2a	281	32.3	11.28	121468.8	201227.7	124811.9	140207.0	170549.5	158666.4	129755.1	147931.4	135665.9	184473.8	173433.3	198913.5	192483.7	169090.1
Q8BJL0	SWI/SNF-related matrix-associated actin-dependent regulator of chromatin subfamily A-like protein 1	Smarcal1	910	100.8	8.9	164741.2	153194.3	125941.9	150293.9	139466.9	143444.6	137622.8	124392.3	142293.1	158658.6	145363.1	151043.3	135688.3	116887.0
Q8CDA1	Phosphatidylinositide phosphatase SAC2	Inpp5f	1132	127.5	7.11	154307.1	181852.9	158053.1	151818.2	185950.0	202636.4	162618.2	174779.0	171450.5	180612.5	162445.5	199654.6	161341.3	191683.6
Q61704	Inter-alpha-trypsin inhibitor heavy chain H3	Itih3	889	99.3	6.05	167379.3	158682.6	155820.9	153009.5	163838.9	170202.0	176760.3	160220.8	193040.9	193442.7	148128.5	152919.6	142588.2	144884.0
Q91VN6	Probable ATP-dependent RNA helicase DDX41	Ddx41	622	69.8	6.7	122139.2	127814.7	135277.3	141875.3	131691.7	128548.7	132823.7	118519.8	136325.7	134982.1	312238.4	321184.8	301962.4	338281.0
Q8BTU1	Cilia- and flagella-associated protein 20	Cfap20	193	22.7	9.76	241240.9	236728.9	281210.6	262416.8	251996.2	301771.6	261404.4	282278.0	251820.4	230974.8	249243.9	266965.7	316880.1	295883.7
O88384	Vesicle transport through interaction with t-SNAREs homolog 1B	Vti1b	232	26.7	8.79	110083.8	159805.7	113697.6	123261.2	122474.6	172387.4	103787.2	126956.5	110055.5	109005.8	220934.5	256034.4	222685.7	219610.8
Q8K2F8	Protein LSM14 homolog A	Lsm14a	462	50.5	9.52	425384.0	460757.3	460184.3	431369.5	456483.0	466893.0	399997.1	386135.9	421374.8	412510.6	399840.4	415873.7	385483.7	406896.7
Q6P5E6	ADP-ribosylation factor-binding protein GGA2	Gga2	603	66	7.3	203359.6	166511.7	188552.5	201703.7	203665.7	200276.3	181894.8	181866.2	185316.6	195473.4	28916.6	38356.4	27769.4	36199.5
Q6P3D0	U8 snoRNA-decapping enzyme	Nudt16	195	21.8	7.12	230541.3	228640.9	241367.9	236438.6	259021.3	259989.3	281908.7	246378.7	251777.4	248177.0	133341.8	138058.1	148968.5	146614.2
Q9D869	Calcineurin B homologous protein 2	Chp2	196	22.6	5.72	178726.9	191183.4	179032.5	173479.5	219268.1	210562.0	206482.9	172013.0	191267.4	203181.1	222641.5	212227.7	216898.0	212825.3
Q8C0L6	Peroxisomal N(1)-acetyl-spermine/spermidine oxidase	Paox	504	55.4	5.11	251726.9	263658.9	216277.3	258561.4	245179.4	228459.6	246177.3	247183.4	258705.1	266738.8	366452.3	375142.4	281469.7	314004.0
Q9CPR5	39S ribosomal protein L15, mitochondrial	Mrpl15	295	33.5	10.07	411892.5	357412.5	340038.9	344070.2	349154.4	356406.8	349682.8	348440.2	358179.3	378426.2	147000.3	133764.4	139947.9	130401.0
Q9D2N4	Dystrobrevin alpha	Dtna	746	84	6.76	464682.6	419575.0	420624.8	396812.2	473963.7	510585.4	497409.3	483172.8	443504.9	417654.0	120560.8	118940.5	114752.0	133511.4
O54916	RalBP1-associated Eps domain-containing protein 1	Reps1	795	86.5	5.58	150896.5	141653.3	151025.9	127853.7	167238.8	151635.4	161023.0	138395.5	133952.1	143682.9	163119.7	190570.5	155552.9	165928.5
P60840	Alpha-endosulfine	Ensa	121	13.3	7.24	54734.9	60280.9	51853.6	54112.0	60458.1	56833.2	53622.6	56277.9	55680.3	53735.4	104176.4	108792.4	127161.3	108237.9
O88696	ATP-dependent Clp protease proteolytic subunit, mitochondrial	Clpp	272	29.8	7.47	148230.2	139755.6	130644.0	143055.6	135705.3	124092.5	132536.8	132893.1	142042.8	136232.0	432361.9	427328.3	369447.6	427574.2
Q9ERA0	Alpha-globin transcription factor CP2	Tfcp2	502	57	5.54	117876.5	128202.8	101495.4	127145.8	121579.1	109027.3	105912.8	105962.2	116405.2	117576.6	79637.4	86983.9	88950.5	92374.7
Q8BQ30	Phostensin	Ppp1r18	594	65.6	5.39	119193.4	117592.2	126477.5	97633.7	119167.2	101553.1	110660.2	111217.9	100893.5	109248.2	254145.5	250758.1	267262.6	243977.2
Q91WT7	Aldo_ket_red domain-containing protein	Akr1c14	323	37.2	7.2	449002.0	421572.0	462604.3	387593.3	508464.1	471741.3	571134.1	422392.2	501131.3	521308.3	127897.1	126980.6	132285.1	125461.1
Q91YJ5	Translation initiation factor IF-2, mitochondrial	Mtif2	727	81.2	7.24	304155.7	294612.5	272957.0	264145.3	279157.4	273447.8	218007.3	262388.5	276452.1	271090.7	212202.0	247981.0	231621.2	264735.7
P30412	Peptidyl-prolyl cis-trans isomerase C	Ppic	212	22.8	7.5	184921.0	185572.5	180532.5	167980.8	202371.4	193661.3	177373.9	240993.2	162910.4	185176.8	502099.1	462001.6	394014.7	414206.3
P83887	Tubulin gamma-1 chain	Tubg1	451	51.1	6.02	148424.8	175581.5	149529.8	160174.9	166034.8	163816.2	143295.7	151824.8	167515.6	154935.8	263021.2	275962.5	251527.7	261348.2
Q80UK0	SEC14 domain and spectrin repeat-containing protein 1	Sestd1	696	79.3	5.1	118293.4	110893.1	116186.7	114699.1	112447.7	122059.5	111222.1	121124.4	112218.4	104815.7	98035.3	103812.7	101521.0	95230.9
P28028	Serine/threonine-protein kinase B-raf	Braf	804	88.7	7.65	89885.6	109228.2	108844.9	101927.4	106513.5	107630.0	99300.3	90163.1	108333.7	111188.6	#N/A	#N/A	#N/A	#N/A
Q6PGH2	Jupiter microtubule associated homolog 2	Jpt2	190	20	8.62	310476.7	264880.2	268944.6	253929.8	284475.9	301419.3	285976.9	283218.2	274975.1	360188.0	247644.3	246477.7	276642.3	228350.3
P52875	Transmembrane protein 165	Tmem165	323	34.8	7.46	59050.0	48631.2	52182.9	53708.3	46849.9	49741.9	51051.3	49686.4	48322.4	50020.6	155250.2	152761.5	139298.3	137915.3
P70671	Interferon regulatory factor 3	Irf3	419	46.8	5.43	95666.4	113160.4	88653.7	97140.2	101661.3	117329.3	93506.5	101650.8	103695.8	99278.8	53375.2	63221.8	53941.9	49874.5
Q8K3K8	Optineurin	Optn	584	67	5.26	169761.3	148335.2	157433.7	148382.9	169473.1	156830.3	169839.1	154767.3	166035.3	165811.5	303084.5	302517.3	305803.0	286788.0
Q9R1C6	Diacylglycerol kinase epsilon	Dgke	564	63.6	7.44	154286.9	143744.4	132831.2	141022.9	122927.4	126437.1	123687.8	108070.0	138116.1	150662.1	81715.2	94343.5	93501.6	88062.1
Q9D0D3	Poly(A) RNA polymerase, mitochondrial	Mtpap	585	65.2	8.88	306082.9	320862.8	311358.0	329483.1	318442.1	330945.3	314151.9	315240.9	328350.9	314098.3	118103.5	130146.6	126750.6	121816.1
Q9R1Q9	V-type proton ATPase subunit S1	Atp6ap1	463	51	5.92	151916.1	195622.5	163717.7	178493.3	188784.1	194832.9	153471.4	152249.9	174712.2	178479.8	177560.4	173801.9	174616.0	163522.6
Q8BVP5	Casein kinase I isoform gamma-2	Csnk1g2	415	47.6	9.06	197132.6	184837.3	201369.3	179288.6	199922.6	218521.4	204140.9	194586.1	206229.9	192814.4	#N/A	#N/A	#N/A	#N/A
P60762	Mortality factor 4-like protein 1	Morf4l1	362	41.5	9.32	202439.8	218572.2	182674.4	206054.4	205218.9	198968.8	197668.5	177007.4	204042.3	216204.5	110259.8	126916.4	110631.7	119685.1
Q80ZX8	Sperm-associated antigen 1	Spag1	901	100.6	5.62	140428.9	162434.4	145609.6	187327.8	122689.6	121560.2	106269.0	119031.9	127066.3	141251.7	97591.1	120069.5	109689.2	136613.3
Q9CYL5	Golgi-associated plant pathogenesis-related protein 1	Glipr2	154	17.1	9.51	57869.2	57665.7	63945.0	49302.2	79844.1	80702.7	90014.8	65591.1	64253.6	64193.1	46316.1	40497.5	45786.6	42463.5
Q9JJ11	Transforming acidic coiled-coil-containing protein 3	Tacc3	631	70.6	4.61	211720.3	246608.4	273764.4	267978.9	256741.9	245805.2	204890.0	210169.7	259479.6	259206.4	402083.4	406930.8	438861.1	423087.5
Q9JIY5	Serine protease HTRA2, mitochondrial	Htra2	458	49.3	9.6	183447.2	201214.9	163026.5	201400.5	156012.4	182658.7	160596.6	154451.2	174111.7	143395.0	198662.8	204266.2	189587.8	202425.4
Q99LD9	Translation initiation factor eIF-2B subunit beta	Eif2b2	351	38.9	6.24	428378.1	471474.1	474726.7	460959.6	397349.5	430384.1	397268.3	397057.0	420234.7	433246.5	381747.0	415101.9	385800.5	388855.3
Q9D8X1	Copper homeostasis protein cutC homolog	Cutc	272	29	7.34	281733.0	292514.6	320053.3	331085.0	312225.7	329062.8	330137.5	276420.0	319497.4	332295.0	136975.4	147779.9	153920.5	141628.2
P63073	Eukaryotic translation initiation factor 4E	Eif4e	217	25	6.15	181488.8	238642.7	198339.7	203353.9	202449.5	194242.1	212324.0	222483.9	198901.2	196336.0	246447.9	461757.0	227328.3	353229.8
Q8R1N4	NudC domain-containing protein 3	Nudcd3	363	40.9	5.26	78129.0	117222.2	85095.7	94347.6	102871.1	88736.7	81417.7	78155.1	96937.9	104516.5	126147.0	140052.8	131769.3	143373.4
Q5SNZ0	Girdin	Ccdc88a	1873	215.8	6.24	106123.6	138535.0	107958.7	121241.8	120786.2	130639.5	96498.4	122560.1	113685.6	129704.3	25754.5	25843.5	21768.9	39679.3
J3QNT6	S1-like domain-containing protein	Gm5039	144	16.5	5.35	264810.8	323245.9	256224.6	308065.2	215865.3	250404.7	270357.7	252935.7	267787.5	262990.8	#N/A	#N/A	#N/A	#N/A
Q9CXW4	60S ribosomal protein L11	Rpl11	178	20.2	9.6	899361.1	861306.1	905555.7	836514.9	856585.7	834749.1	799178.7	899677.9	818393.5	887985.5	1050081.8	1131167.5	1063734.6	1048796.9
Q09143	High affinity cationic amino acid transporter 1	Slc7a1	622	67	7.06	203720.2	188128.2	177763.2	193112.9	130152.0	127752.8	125594.1	181090.3	174614.5	168610.1	310513.9	236431.3	276902.9	289028.5
Q99M01	Phenylalanine--tRNA ligase, mitochondrial	Fars2	451	52.3	7.17	70251.4	66560.8	77291.1	71096.1	77151.3	71269.5	63557.8	71517.6	77311.4	82434.1	105658.9	120149.4	117382.1	105977.8
Q9D2C2	Protein SAAL1	Saal1	474	52.7	4.53	171449.4	202682.5	181090.7	200902.0	195929.8	187891.1	165202.3	165090.8	175902.1	196614.7	139504.8	168206.2	137200.4	157396.4
Q8C8T8	Pre-rRNA-processing protein TSR2 homolog	Tsr2	191	20.9	4.23	244089.0	221251.5	252855.2	230957.7	238405.8	267892.5	234945.7	238139.1	241728.5	251843.6	277351.0	254224.2	267408.8	242555.3
E9Q166	Bromo domain-containing protein	Atad2b	1460	164.6	6.96	59373.6	84766.7	67335.5	69543.6	74118.4	78190.1	73063.8	68234.3	75183.2	71734.8	57857.7	70442.1	51668.0	62996.0
P09242	Alkaline phosphatase, tissue-nonspecific isozyme	Alpl	524	57.5	7.01	184512.0	196792.3	171770.4	217337.6	137226.4	154421.7	156108.2	152554.3	162545.0	168673.8	247622.4	278023.3	223161.4	279864.0
A6H6A9	Rab GTPase-activating protein 1-like	Rabgap1l	815	92.3	5.31	52933.0	57375.2	47914.3	52107.5	63134.0	61752.4	54157.4	42641.5	53839.8	56913.9	282892.6	330481.5	318041.5	349545.3
Q80TE0	RNA polymerase II-associated protein 1	Rpap1	1409	155.2	6.65	282371.7	279077.1	233030.0	296182.0	269497.7	253632.0	272414.9	277412.5	229732.0	303728.5	102139.4	102433.7	124809.4	108782.0

Q05512	Serine/threonine-protein kinase MARK2	Mark2	776	86.3	9.67	229217.1	204379.5	193146.0	195957.7	214365.3	221065.3	231720.0	197748.0	215462.4	240819.7	172803.6	190262.4	179083.9	194609.8
Q9D8M4	60S ribosomal protein L7-like 1	Rpl7l1	246	28.5	10.64	421114.6	378057.6	429941.7	371757.4	458454.4	439160.5	446334.2	436044.3	409551.9	459178.7	283256.0	301903.3	285417.4	297319.3
Q501J7	Phosphatase and actin regulator 4	Phactr4	694	76.6	6.48	59865.0	57627.3	56869.4	58892.5	60378.3	63183.9	59265.6	55895.3	59657.9	61060.2	128894.3	133647.3	124760.6	120973.8
Q8VHH5	Arf-GAP with GTPase, ANK repeat and PH domain-containing protein 3	Agap3	910	97.9	7.75	160487.5	156280.3	160118.7	145797.7	162681.3	166258.6	154014.8	156870.9	156792.4	156332.8	78462.9	81076.6	88621.3	80259.8
Q99JW4	LIM and senescent cell antigen-like-containing domain protein 1	Lims1	325	37.2	8.05	419402.9	342511.8	337899.4	348452.0	347705.4	379047.8	392954.3	382964.6	351484.5	336598.0	560853.4	610114.0	535212.6	549633.0
Q91WG5	5'-AMP-activated protein kinase subunit gamma-2	Prkag2	566	62.9	9.36	122963.0	117262.6	119901.3	108468.7	119602.1	127592.0	118036.8	105135.5	106625.4	128395.7	#N/A	#N/A	#N/A	#N/A
P49025	Citron Rho-interacting kinase	Cit	2055	235.2	6.54	117365.3	110178.5	117034.1	120550.9	122987.5	113470.2	111550.1	122954.9	117019.1	112174.7	137556.2	163894.7	162975.5	167311.7
P29788	Vitronectin	Vtn	478	54.8	5.88	100748.1	92959.4	91937.3	86980.8	70540.9	74985.0	75323.6	93291.6	76241.3	75972.8	169292.2	175553.2	146891.4	161618.1
Q8CIQ7	Dedicator of cytokinesis protein 3	Dock3	2027	232.8	6.99	157814.6	159066.0	142271.1	147083.5	134993.0	128892.2	122934.4	136399.0	145751.2	141118.3	95833.3	107957.8	94221.2	96329.7
Q8K301	Probable ATP-dependent RNA helicase DDX52	Ddx52	598	67.4	9.57	283726.7	269420.4	294410.1	266714.9	282148.0	279210.7	258905.9	272420.0	299805.2	294359.9	134895.6	135117.2	145812.7	139223.5
Q4PZA2	Endothelin-converting enzyme 1	Ece1	769	87	5.91	298030.1	269799.1	273169.4	274383.2	304374.0	332840.9	292371.5	276204.6	261660.6	277272.6	81100.5	83286.0	69145.9	87516.6
Q9D0I4	Syntaxin-17	Stx17	301	33.2	6.74	88735.2	97831.6	94688.5	89855.2	98191.5	86206.2	82497.9	94544.3	91754.4	85193.0	502185.8	547076.1	523035.5	536474.4
Q3UQ28	Peroxidasin homolog	Pxdn	1475	165	7.14	155043.0	163411.8	147927.2	147070.1	155695.9	171497.3	144630.0	157080.5	155767.3	158363.3	37874.1	35779.4	35839.9	35722.9
Q8CHU3	Epsin-2	Epn2	595	63.4	8.16	99692.1	107903.9	110142.6	110212.9	116632.8	109210.8	107024.6	98365.7	111397.8	109267.9	370334.5	381182.0	365255.5	353699.5
P54726	UV excision repair protein RAD23 homolog A	Rad23a	363	39.7	4.58	9565.6	16429.2	9421.7	11196.7	9384.2	13720.2	9488.7	8084.0	9516.2	9898.1	34090.7	35849.7	34285.0	35175.0
Q8JZM0	Dimethyladenosine transferase 1, mitochondrial	Tfb1m	345	38.9	9.47	344638.9	361010.4	343666.3	348103.8	344416.5	349514.9	295239.0	306881.7	333041.5	359684.3	358371.3	408494.1	336988.9	427705.3
P62075	Mitochondrial import inner membrane translocase subunit Tim13	Timm13	95	10.5	8.18	247205.3	259083.9	274557.3	289847.6	255162.5	207635.6	222264.3	265751.4	266646.7	246936.2	328684.2	375144.4	360811.8	373949.6
Q8K0T2	Cytoplasmic dynein 2 light intermediate chain 1	Dync2li1	351	39.4	8.09	126353.6	133321.3	130024.0	139257.6	102705.0	106935.0	105282.2	112478.5	119696.7	122159.3	156572.9	186410.7	169101.1	188385.6
Q61845	Meiosis-expressed gene 1 protein	Meig1	88	10.8	9.1	911492.2	1264447.4	1551521.0	1551953.3	978703.2	705699.5	646979.0	1124628.3	1175567.2	1259470.6	326016.3	417876.0	474638.9	482033.2
Q9EQG9	Ceramide transfer protein	Cert1	624	71.1	5.44	146467.7	147424.8	158005.5	143880.7	176457.7	170740.0	165416.2	159792.1	166489.9	160388.6	129065.4	135099.0	142288.9	121802.9
O88561	Solute carrier family 27 member 3	Slc27a3	667	72.9	7.65	495005.4	575489.2	386864.9	497153.0	422637.1	499225.5	356483.2	470570.3	458370.4	493184.0	278508.7	254500.9	333434.4	256048.7
A0A140LIU8	Predicted gene, 18336	Gm18336	765	78.7	4.25	119027.8	123216.2	115672.3	110043.6	114645.2	108352.2	105622.7	108407.0	113937.2	120339.2	130651.3	124924.4	111688.1	103192.6
Q3U0M1	Trafficking protein particle complex subunit 9	Trappc9	1148	128.2	6.47	70777.2	106764.7	84386.9	92360.5	97128.1	93995.1	79632.2	100054.6	74276.8	83276.1	142046.4	143904.9	135497.8	141650.9
Q80Y55	BSD domain-containing protein 1	Bsdc1	427	46.9	4.4	88894.8	100203.8	91342.4	97137.0	86048.5	90487.0	83108.7	81100.5	86871.7	86844.6	152576.4	163571.5	158381.8	167661.4
Q8BIA4	F-box/WD repeat-containing protein 8	Fbxw8	598	67.9	6.16	100951.2	99189.5	78408.1	91750.9	93442.7	97828.2	89107.4	80534.4	103892.3	89361.0	84492.7	101489.4	98895.8	91871.8
Q61586	Glycerol-3-phosphate acyltransferase 1, mitochondrial	Gpam	827	93.6	7.84	275948.9	251342.1	267076.3	246727.8	302928.6	254400.3	248277.4	241765.0	249615.5	255077.5	98474.7	100803.3	100942.4	89577.3
P61961	Ubiquitin-fold modifier 1	Ufm1	85	9.1	9.31	211525.7	224443.8	216421.5	222888.9	218533.3	197093.2	198134.9	212320.1	237689.7	237892.0	54958.6	71995.9	62207.2	68711.8
Q8CI75	DIS3-like exonuclease 2	Dis3l2	870	97.7	5.9	227436.0	234505.5	217483.6	220934.0	253043.4	245252.6	224792.5	230517.9	228478.5	227425.1	241950.1	253733.6	241274.7	233316.8
P59708	Splicing factor 3B subunit 6	Sf3b6	125	14.6	9.38	180468.6	183913.7	220785.4	210243.4	228649.6	207497.7	205979.6	209915.5	224274.6	208744.5	140082.2	119379.5	152380.4	144350.4
O09044	Synaptosomal-associated protein 23	Snap23	210	23.2	4.98	188778.6	187862.6	193709.4	203180.6	198247.8	215253.8	211274.5	190440.5	208541.5	178995.6	154447.2	121956.5	174553.0	144037.0
Q8C6G8	WD repeat-containing protein 26	Wdr26	641	70.5	6.16	293117.0	308407.2	310375.8	331346.9	283563.7	307779.9	287706.5	284378.1	312003.4	296014.8	105811.1	123905.2	104146.8	103632.3
Q8K2D3	Enhancer of mRNA-decapping protein 3	Edc3	508	55.9	7.09	50542.1	54178.1	57178.6	60432.7	57100.9	53889.3	50313.5	46097.2	57329.3	53473.8	71345.4	78445.4	85918.5	82430.1
Q8BYC6	Serine/threonine-protein kinase TAO3	Taok3	898	105.3	7.36	77081.5	74447.9	66469.8	78824.0	63121.8	64098.2	53872.4	62122.0	76323.7	69489.3	397748.3	368083.2	296370.8	305591.2
P70290	55 kDa erythrocyte membrane protein	Mpp1	466	52.2	7.2	148210.9	156574.4	136361.9	156145.8	165146.3	163360.8	164268.9	166237.9	150789.4	148787.4	70598.6	75762.0	58116.4	71670.5
Q6IE82	Protein Jade-3	Jade3	823	93.4	7.02	149301.7	161570.9	158667.6	149388.3	158199.0	155247.3	152295.1	147735.4	145994.5	152990.8	79468.7	76852.2	67019.2	66493.9
Q6ZQK0	Condensin-2 complex subunit D3	Ncapd3	1506	169.3	7.15	46333.5	57769.9	48655.7	47124.1	54153.7	57825.3	47629.0	44219.6	49501.6	56345.9	64729.5	64195.7	69799.3	69248.5
Q3UU96	Serine/threonine-protein kinase MRCK alpha	Cdc42bpa	1719	195.4	6.51	70759.6	69015.0	63789.8	65973.3	68721.4	70674.9	68068.8	65889.3	70217.8	65953.2	589942.7	578265.0	511654.0	578136.2
A2CG49	Kalirin	Kalrn	2964	336.8	6.07	118127.6	111961.9	106811.9	101740.7	122685.8	124875.5	115404.0	114128.8	113661.8	113386.2	83377.2	78034.8	82870.0	78721.2
Q9DCT8	Cysteine-rich protein 2	Crip2	208	22.7	8.63	321910.9	331445.6	337423.3	364828.1	356078.1	339528.4	327999.1	305401.0	330365.0	351169.5	321186.5	352335.4	351297.2	313750.9
Q8K3W0	BRISC and BRCA1-A complex member 2	Babam2	383	43.5	5.94	184332.2	222960.9	203729.6	208481.3	216540.9	216643.3	199303.3	202599.7	210721.7	223278.1	217249.9	236028.7	258557.7	236307.5
O88291	DBIRD complex subunit ZNF326	Znf326	580	65.2	5.19	146444.2	152154.5	156147.5	170237.8	155861.2	145249.0	142581.2	140414.9	143680.8	158345.8	195401.4	179169.0	268416.4	206393.1
O70475	UDP-glucose 6-dehydrogenase	Ugdh	493	54.8	7.56	286902.9	276424.2	256367.1	281827.1	280474.8	309154.2	292875.6	327593.7	252902.4	260100.4	69154.1	67512.8	62070.3	64631.1
Q6IRU5	Clathrin light chain B	Cltb	229	25.2	4.63	241604.3	230992.6	231646.4	223201.1	247344.9	227048.5	210548.7	236648.0	232568.0	246505.8	177227.9	187472.4	170494.2	159646.5
Q9WV70	Nucleolar complex protein 2 homolog	Noc2l	747	85.4	5.97	191854.7	214324.2	201986.2	204452.7	223521.4	224155.3	195099.4	201178.5	208651.7	211887.2	224549.5	223117.2	226295.2	219485.2
Q9WVL3	Solute carrier family 12 member 7	Slc12a7	1083	119.4	6.51	163958.2	162491.9	164752.2	161679.9	176805.7	192260.3	222049.3	170189.0	180322.9	170766.4	69904.2	76390.1	62168.9	70282.1
P11157	Ribonucleoside-diphosphate reductase subunit M2	Rrm2	390	45.1	5.45	108636.5	130449.4	117050.9	110046.1	147665.3	135777.7	124611.4	138468.9	121510.5	125932.5	38218.8	50717.7	39607.8	41192.0
P50096	Inosine-5'-monophosphate dehydrogenase 1	Impdh1	514	55.2	6.8	52333.6	45742.1	45864.3	42102.5	51626.3	47279.9	45087.4	46455.7	46734.2	50957.2	30747.7	30570.8	28279.5	25059.7
Q8BFY6	Peflin	Pef1	275	29.2	6.3	115899.5	123402.1	115146.0	123832.9	108241.2	106976.3	120211.8	106856.9	128831.4	122868.2	25959.0	27942.1	30519.2	25769.3
Q99MZ7	Peroxisomal trans-2-enoyl-CoA reductase	Pecr	303	32.4	8.29	152634.8	131291.0	147055.9	147808.7	153191.4	148972.5	181457.0	140126.7	155411.9	141892.4	296674.6	288684.9	318970.0	275986.0
Q8BU03	Periodic tryptophan protein 2 homolog	Pwp2	919	102.8	6.23	196669.1	206620.0	171273.1	180413.2	202742.7	204541.8	176902.2	165588.6	193936.8	207013.5	58041.2	55845.0	52670.4	51523.2
Q8C176	Transcription initiation factor TFIID subunit 2	Taf2	1104	126.9	8.1	92624.6	106026.7	93385.7	88565.0	94310.2	93932.2	85382.6	75585.4	86991.4	97096.9	98949.1	118368.5	97859.1	107842.3
Q8K0Q5	Rho GTPase-activating protein 18	Arhgap18	663	74.9	6.73	202569.7	197881.0	181156.2	189211.9	201244.8	208738.3	214913.2	196966.3	201290.7	204249.4	132146.7	129143.0	111321.6	120319.4
Q99KG3	RNA-binding protein 10	Rbm10	930	103.4	5.92	54265.2	41594.4	59177.4	41029.5	63150.9	62710.1	67539.3	63122.4	50781.5	47535.8	186263.5	197116.3	191663.9	172974.8
Q3TIR3	Synembryn-A	Ric8a	530	59.8	5.68	126168.8	128473.8	158371.9	115813.3	113029.5	119924.9	201639.4	111722.2	173853.6	108499.1	28572.4	35660.1	29925.9	36475.0
Q6PIU9	Uncharacterized protein FLJ45252 homolog	-	354	37.7	5.07	125383.6	139782.3	138954.4	126873.9	137555.0	140604.2	126456.4	134631.3	131380.0	126370.1	42821.2	47260.0	45696.5	46178.4
P56380	Bis(5'-nucleosyl)-tetraphosphatase [asymmetrical]	Nudt2	147	17	6.28	204195.7	205239.9	165096.3	196751.7	177152.3	194056.4	175611.4	177112.6	193771.4	210713.4	442448.5	404140.0	416273.2	433704.3
Q9DBA9	General transcription factor IIH subunit 1	Gtf2h1	547	61.8	8.76	126543.5	145112.5	148649.4	142766.6	127089.6	133854.5	121268.8	120411.5	138811.9	150541.7	269530.8	283507.9	284260.3	292344.7
Q9JHI7	Exosome complex component RRP45	Exosc9	438	48.9	5.11	82769.1	85681.1	90796.1	100390.3	113735.5	117417.5	92571.8	96217.3	98635.9	94886.2	149250.8	153440.3	155005.7	142152.9
Q9QXT0	Protein canopy homolog 2	Cnpy2	182	20.8	5.07	551968.7	494183.4	500195.8	487498.9	494866.5	538887.7	488730.7	557552.7	510292.1	482998.4	604918.3	538673.4	530984.4	517195.4
Q8CE33	Kelch-like protein 11	Klhl11	709	80.4	6.01	100842.0	114495.0	112514.1	93642.8	108018.8	114800.5	97326.4	112082.3	113284.7	112456.1	200320.3	203323.8	171977.0	188666.5
Q9CR58	Kidney mitochondrial carrier protein 1	Slc25a30	291	32.3	9.38	170050.1	176960.8	164671.2	180814.6	162608.2	150460.8	149858.1	159905.6	161485.9	175830.4	144545.1	130362.4	143158.1	142883.9
Q9D5P4	Adenosine deaminase domain-containing protein 2	Adad2	478	51	8.27	148562.2	182846.0	177157.0	207881.3	107337.7	94152.4	87685.1	104359.8	144376.1	182177.3	124579.8	166591.4	154452.4	182835.7
P12023	Amyloid-beta A4 protein	App	770	86.7	4.79	203948.9	201030.3	221523.5	222563.0	197794.1	160634.8	170228.3	183741.8	198273.1	201184.6	108471.2	117997.3	114805.2	99905.9
P62340	TATA box-binding protein-like protein 1	Tbpl1	186	20.9	9.54	121325.3	144734.3	132143.7	145239.7	148442.0	125481.9	123464.5	118745.0	131052.1	138111.9	79430.1	88261.9	91726.4	97126.2
Q8C9H6	Striatin-interacting proteins 2	Strip2	844	96.2	5.77	7589.8	5036.7	4608.1	7159.8	6342.0	9998.1	8476.2	6481.1	2597.3	6823.4	146893.0	123104.4	135564.5	125179.1
E9Q8D0	DnaJ homolog subfamily C member 21	Dnajc21	531	61.7	5.9	91188.1	118690.7	118214.8	122475.5	113594.0	125738.2	90241.0	95385.5	118440.0	125927.0	185950.1	184037.5	178357.5	170725.8
Q61189	Methylosome subunit pICln	Clns1a	236	26	4.12	444793.6	427211.3	474960.9	409122.6	433592.6	459327.6	411119.9	420926.8	468555.5	500032.8	127972.4	147511.1	159857.7	166424.4
O88307	Sortilin-related receptor	Sorl1	2215	246.9	5.54	56598.5	54062.7	55073.2	51664.3	58760.1	51039.6	55481.6	57684.5	55683.6	60991.9	47353.1	47135.4	38632.8	35739.4
Q9D513	Meiosis-specific with OB domain-containing protein	Meiob	470	52.9	6.96	239572.5	266424.8	244590.2	253331.1	292171.1	301902.0	267770.4	232756.9	274625.6	269433.2	130829.3	139204.0	139509.2	133745.3
Q9Z0J1	Reversion-inducing cysteine-rich protein with Kazal motifs	Reck	971	106	6.71	43332.5	32590.9	38267.3	34714.5	45448.4	46935.1	51379.3	44400.6	38479.9	37783.4	119572.4	127545.4	143680.4	131970.4
Q8BR63	Protein FAM177A1	Fam177a1	207	23.6	4.59	53626.3	61462.2	65941.3	71725.9	64530.8	54374.7	58207.4	49655.0	58544.5	61560.7	50245.2	58130.1	66469.4	85981.8
Q8VE73	Cullin-7	Cul7	1689	192.2	6.29	152115.1	143517.5	130971.2	134904.0	152623.6	158477.8	150398.9	144162.4	141526.5	143286.0	134152.1	173559.8	135083.6	132009.2
Q64261	Cyclin-dependent kinase 6	Cdk6	326	37	6.62	109823.1	115912.2	95713.0	100926.2	124471.0	140580.3	109694.9	130241.5	105086.3	106867.3	92224.3	89433.1	86627.6	96722.2
Q9D8T2	Gasdermin-D	Gsdmdc1	487	53.2	5.1	121333.7	123772.1	111374.2	111566.9	133389.3	130503.5	147410.7	112550.6	127735.8	126601.1	96707.9	102116.8	108398.4	94964.9
Q6ZPU9	KIF-binding protein	Kifbp	617	71	5.48	34331.2	44067.1	33507.0	42244.6	41797.8	44676.5	37302.6	38186.5	37080.4	39012.2	195999.1	240235.9	187626.4	178633.6
P27048	Small nuclear ribonucleoprotein-associated protein B	Snrpb	231	23.6	10.9	342282.2	355352.1	317596.8	348189.9	355354.2	338528.1	364046.5	329211.3	360157.7	374760.1	896860.8	879005.0	873557.1	881372.6
Q8R5J9	PRA1 family protein 3	Arl6ip5	188	21.5	9.61	211236.7	212554.4	190309.6	204697.2	214334.0	229334.5	235130.7	205769.5	198686.0	210133.1	244104.4	247047.0	232462.7	244402.8
Q91VS7	Microsomal glutathione S-transferase 1	Mgst1	155	17.5	9.66	1575915.0	1462373.6	1370662.4	1435751.3	1432885.9	1420025.2	1486417.6	1516328.0	1425907.4	1385990.5	2014608.6	1914376.7	1922154.3	1919427.6
Q9DCC4	Pyrroline-5-carboxylate reductase 3	Pycr3	274	28.7	7.27	235383.9	249199.9	230070.2	218747.6	242824.9	265531.4	273051.4	237275.4	253479.8	263436.2	419483.6	378145.4	401386.3	355002.8
Q9WTQ8	Mitochondrial import inner membrane translocase subunit Tim23	Timm23	209	22	9.13	221399.5	221911.4	212917.1	221892.7	199060.6	193736.9	193542.7	201694.1	220151.6	202350.4	171232.0	172363.4	158576.9	156213.2
O89110	Caspase-8	Casp8	480	55.3	5.25	214165.9	185780.8	210828.3	191391.1	220659.1	209051.2	215373.9	214353.7	202766.4	205905.8	225681.4	235189.8	217828.6	207460.5
O08914	Fatty-acid amide hydrolase 1	Faah	579	63.2	7.87	94847.5	102831.8	85138.9	99176.8	90688.9	100077.3	96808.6	92605.1	84076.5	97577.8	24665.3	21979.9	19630.4	18812.8
Q9D7N3	28S ribosomal protein S9, mitochondrial	Mrps9	390	44.9	8.81	134020.7	145234.7	135121.9	140042.4	122836.3	133005.3	140849.6	127399.5	123193.6	135971.1	163992.0	173475.7	148087.8	166773.7
P70441	Na(+)/H(+) exchange regulatory cofactor NHE-RF1	Slc9a3r1	355	38.6	5.9	64525.6	82367.6	68854.7	101796.8	59967.7	90985.2	73115.1	75951.1	71149.5	60090.2	95776.9	123128.6	91866.2	122634.3
Q3V063	TAFII55_N domain-containing protein	Taf7l2	378	42.8	5.02	105210.4	161049.7	159208.7	178223.0	125472.2	114766.8	93470.1	107473.8	149422.6	158081.0	60603.3	73784.4	72657.7	75470.3
Q69ZX6	ATPase MORC2A	Morc2a	1030	117.3	8.34	52255.1	48220.4	44416.5	44028.4	55206.3	55346.5	47328.4	45483.5	48498.6	52290.5	90565.8	95991.6	100868.9	94429.3
Q921Q3	Chitobiosyldiphosphodolichol beta-mannosyltransferase	Alg1	482	54.4	8.37	122829.8	125422.9	136099.5	142890.6	122352.9	115288.9	97997.6	116271.3	126184.2	127429.1	76450.4	87826.3	76071.1	84445.3
Q9DB16	Calcium-binding protein 39-like	Cab39l	337	39.1	8.31	58756.3	59121.0	57608.5	51906.9	64997.2	64663.2	52590.9	53323.4	55632.8	60683.9	182510.6	171100.0	185199.5	173558.6
O55229	Choline/ethanolamine kinase	Chkb	394	45.1	5.41	269715.1	302083.2	255059.6	306562.7	282346.9	281682.5	272566.4	252706.0	286054.1	329469.1	52431.6	60645.9	50098.0	51184.6
Q8VEJ4	Notchless protein homolog 1	Nle1	485	53.1	7.17	59827.3	71784.9	58988.7	60621.9	65687.6	74254.9	65146.6	55759.8	73431.2	68607.0	78895.2	75372.8	80602.8	77063.4
Q9CPQ8	ATP synthase subunit g, mitochondrial	Atp5mg	103	11.4	9.74	735750.8	806309.0	846055.8	843940.1	829492.8	756770.6	784888.0	898831.6	793303.2	734123.9	364846.5	365605.5	369743.7	364096.5
Q9D0M5	Dynein light chain 2, cytoplasmic	Dynll2	89	10.3	7.37	890557.5	838456.3	969967.2	873907.6	984654.0	947934.0	1016233.7	952166.1	886032.3	910557.8	958755.4	965903.3	1048568.5	1013374.9

Q9R207	Nibrin	Nbn	751	83.7	6.68	205324.7	224539.6	230180.9	209459.1	243166.0	244470.5	223234.9	210706.5	232565.1	229636.3	139333.6	146725.4	175936.1	151339.2
A2BDX3	Adenylyltransferase and sulfurtransferase MOCS3	Mocs3	460	49.3	7.49	79254.8	79994.0	83267.4	89670.0	72242.3	72254.6	80431.8	77071.1	81264.4	73264.1	61866.2	60687.4	64489.1	56509.3
Q924W5	Structural maintenance of chromosomes protein 6	Smc6	1097	127.1	7.17	62986.9	71545.3	78942.5	81967.7	69303.6	68640.0	64828.2	65468.8	69475.9	73676.3	77261.2	77541.7	69852.9	70211.9
Q80UV9	Transcription initiation factor TFIID subunit 1	Taf1	1891	214.3	5.11	141675.5	194214.1	145987.3	150316.2	167669.9	175711.8	158464.2	135894.5	163084.3	165845.5	65145.7	81581.2	75556.1	70398.2
Q9R0P4	Small acidic protein	Smap	181	20	4.82	69569.5	89087.5	65829.1	71681.6	70505.3	71478.8	65050.2	64037.1	73691.0	89150.1	78682.1	76489.3	93920.3	78215.2
Q8VIN1	Phosphatidylethanolamine-binding protein 2	Pbp2	187	21.2	8.37	37965.9	45394.0	44228.4	56217.0	42768.4	38577.9	42168.1	34559.2	43705.3	41336.3	67805.1	85835.7	99308.5	115858.8
Q9CRB2	H/ACA ribonucleoprotein complex subunit 2	Nhp2	153	17.2	8.41	209294.1	216857.6	202631.2	217640.1	231993.2	234333.2	212259.3	197714.8	226726.3	226592.7	143939.5	142514.0	137601.2	137312.9
Q61103	Zinc finger protein ubi-d4	Dpf2	391	44.2	6.47	40511.1	43642.4	42721.6	38840.1	44313.0	42802.5	48906.9	44232.3	39743.8	41147.8	150048.7	164675.8	153469.1	134489.7
O35613	Death domain-associated protein 6	Daxx	739	81.4	4.7	88832.7	100323.2	100551.6	96570.5	123847.9	90091.5	79190.4	86173.3	91712.4	97700.0	47848.9	51274.1	53051.9	54997.8
Q61398	Procollagen C-endopeptidase enhancer 1	Pcolce	468	50.1	8.41	286026.2	325822.5	257084.1	307461.4	261375.7	304340.0	244099.6	326997.5	254332.5	266959.6	205684.8	206331.0	203658.9	210954.5
Q8C4B4	Protein unc-119 homolog B	Unc119b	251	28.3	5.72	111801.1	127945.4	113953.9	105505.3	118357.9	122632.6	106693.9	119185.0	103462.6	112626.8	180694.9	174273.6	187702.2	170848.4
Q8CHK3	Lysophospholipid acyltransferase 7	Mboat7	473	53.4	8.69	77924.8	79368.4	77817.4	73252.0	78599.6	85720.5	78774.5	77503.6	70634.7	72751.2	39970.3	38622.2	35252.4	35624.9
Q9QXY9	Peroxisomal biogenesis factor 3	Pex3	372	42.2	7.77	138614.1	152204.5	169980.6	157548.6	168527.3	157719.9	133168.8	147559.0	154081.9	153727.0	164714.3	179753.2	169316.1	171167.5
Q9CQJ8	NADH dehydrogenase [ubiquinone] 1 beta subcomplex subunit 9	Ndufb9	179	22	7.8	156502.4	159844.6	141001.2	169178.6	161394.0	156058.2	164048.8	161854.7	149667.2	158304.2	110283.9	130169.9	116723.7	130961.6
Q8K3J1	NADH dehydrogenase [ubiquinone] iron-sulfur protein 8, mitochondrial	Ndufs8	212	24	6.21	153772.1	156068.0	168007.5	161791.8	168005.6	171079.7	175150.2	163218.8	158957.1	161602.9	284721.2	290072.8	271988.9	293520.8
Q640N1	Adipocyte enhancer-binding protein 1	Aebp1	1128	128.3	5.08	80070.1	75183.1	71889.2	76710.1	70126.8	83139.6	93485.9	72142.1	80995.7	82324.2	25395.9	37574.8	35124.8	30517.6
Q9D0D4	Probable dimethyladenosine transferase	Dimt1	313	35.3	9.99	99958.2	121215.2	107032.3	114489.4	120831.3	111699.8	110454.9	115401.1	118141.3	116846.2	239310.2	239107.5	231562.6	243356.3
Q6S5J6	Krev interaction trapped protein 1	Krit1	736	83.9	8.38	181616.5	185759.9	183554.0	177976.6	208416.5	200029.1	184719.8	177489.2	185612.4	196066.0	92034.1	102764.2	100491.5	97630.2
Q8R0P4	Mth938 domain-containing protein	Aamdc	122	13.2	7.99	178400.9	196989.3	210928.6	231206.0	198404.1	188701.4	175935.3	197386.3	211248.8	190448.9	303310.0	342662.9	366785.2	369645.2
O70309	Integrin beta-5	Itgb5	798	87.9	6.16	107217.4	86554.4	87583.4	88600.0	82098.4	83691.8	85174.5	97098.7	103471.4	96109.1	169364.7	160209.6	172407.9	151333.8
Q921K8	TRPM8 channel-associated factor 2	Tcaf2	919	101.5	6.52	72378.1	79201.7	66672.2	70154.0	74471.7	72618.5	64838.9	76261.2	65806.5	69521.5	#N/A	#N/A	#N/A	#N/A
Q8R3F9	Speckle targeted PIP5K1A-regulated poly(A) polymerase	Tut1	869	94.5	5.81	82152.6	76334.8	74061.2	77885.2	80003.6	78030.9	78090.9	77015.7	62676.3	69692.5	59675.1	59272.8	59451.0	54916.9
Q9CQN7	39S ribosomal protein L41, mitochondrial	Mrpl41	135	15.3	9.82	209439.1	206383.1	206523.1	206485.4	196619.4	175923.8	179137.9	195288.9	206226.0	218658.7	216580.4	238352.0	217006.5	220994.8
Q99K74	Mediator of RNA polymerase II transcription subunit 24	Med24	987	109.9	6.83	66084.2	67218.4	75479.4	71154.0	78588.1	73750.8	65830.7	73990.3	73645.0	69952.2	136312.6	143339.1	120482.6	139619.2
Q80X85	28S ribosomal protein S7, mitochondrial	Mrps7	242	28	9.94	156974.0	162687.8	166139.8	163131.0	164434.1	146322.0	135059.2	148118.1	159137.3	156143.2	117539.5	133663.4	113606.2	124337.7
Q8VCX5	Calcium uptake protein 1, mitochondrial	Micu1	477	54.3	8.59	337503.2	325880.7	312929.5	344314.9	298044.3	290096.7	325838.6	324548.4	314886.5	309771.9	309790.3	297788.3	307018.1	294059.3
O54931	A-kinase anchor protein 2	Akap2	893	98.5	5.21	89794.1	85318.0	83543.7	81349.6	87261.7	90256.5	84355.9	88823.1	90061.7	90396.4	96281.0	90147.1	84725.3	94784.2
P23780	Beta-galactosidase	Glb1	647	73.1	7.47	192130.1	192045.4	157059.1	204500.2	167682.1	164144.1	172452.5	166320.1	162571.2	165763.0	140805.6	132717.5	135056.3	150775.0
P59999	Actin-related protein 2/3 complex subunit 4	Arpc4	168	19.7	8.43	588103.4	573876.8	589436.2	589360.1	580805.8	620291.3	484320.2	654486.6	548681.3	536426.3	240565.2	246196.0	219326.7	226415.2
Q9QWR8	Alpha-N-acetylgalactosaminidase	Naga	415	47.2	6.44	163381.8	173821.6	171150.3	170668.9	217452.4	208811.6	224238.3	182249.6	184341.3	173954.9	385690.9	541258.7	381404.0	409131.2
P51175	Protoporphyrinogen oxidase	Ppox	477	50.8	8.84	223850.1	211845.3	230193.0	232567.7	250292.0	259495.2	243610.0	230906.8	228283.5	227748.8	384653.5	362207.0	373966.5	369177.6
Q6PHU5	Sortilin	Sort1	825	91.1	5.88	237747.8	227748.4	237951.7	236204.2	240888.5	243656.5	203297.7	243384.6	243949.9	233408.8	52235.7	62644.1	53430.4	65270.4
Q9R1X4	Protein timeless homolog	Timeless	1197	137.4	5.49	187555.8	189266.0	174386.5	185245.6	189542.1	194292.9	183824.8	172738.4	188472.2	197358.2	140858.6	161851.0	140392.6	145977.5
Q920Q8	Influenza virus NS1A-binding protein homolog	Ivns1abp	642	71.5	5.47	150918.7	153270.2	159378.0	155711.3	147603.9	152012.7	136112.7	135411.4	151418.0	155031.3	77036.7	66025.7	74740.5	64582.0
P97798	Neogenin	Neo1	1493	163.1	6.47	55356.7	68752.0	46642.7	60127.1	50117.7	55940.9	54020.1	44023.1	52567.9	62690.5	22626.7	19645.4	19538.6	23211.7
P61963	DDB1- and CUL4-associated factor 7	Dcaf7	342	38.9	5.52	318948.3	335192.9	340210.9	327159.5	351938.0	334099.0	301947.6	314917.5	302016.3	335683.8	312483.9	362135.2	343875.2	344216.5
Q6P9R1	ATP-dependent RNA helicase DDX51	Ddx51	639	70.3	9.47	168245.2	169398.7	169192.8	156171.0	199178.1	194150.0	173335.7	173268.6	169643.9	176406.8	227900.7	233942.3	211392.2	216105.3
Q91VH6	Protein MEMO1	Memo1	297	33.7	7.14	104903.7	115373.3	101133.6	118110.0	92073.4	107791.6	93476.4	105330.6	114198.6	100146.4	144187.7	148618.4	123570.0	160838.4
Q62425	Cytochrome c oxidase subunit NDUFA4	Ndufa4	82	9.3	9.52	616761.5	576483.9	668543.4	610097.8	670690.6	625222.9	640230.6	707476.5	621894.4	595191.6	893255.7	968714.2	1182615.2	1090241.6
Q9DCM2	Glutathione S-transferase kappa 1	Gstk1	226	25.7	8.88	160898.8	183862.2	162675.4	165552.0	191592.6	194180.2	205894.4	173532.1	180453.7	175564.5	114524.5	129736.8	117188.5	123910.8
Q62074	Protein kinase C iota type	Prkci	595	68.2	5.85	97609.8	104323.0	103440.2	92990.3	107254.9	98296.5	104571.4	103416.5	88333.5	95626.9	71408.5	83540.8	70592.2	71902.3
Q91VC7	Protein phosphatase 1 regulatory subunit 14A	Ppp1r14a	147	16.6	7.34	31834.5	29148.2	33592.9	31451.7	39449.5	39749.1	39162.3	35262.8	35796.3	31474.5	64258.7	74112.3	56439.7	70834.8
Q3U5Q7	UMP-CMP kinase 2, mitochondrial	Cmpk2	447	50	7.27	87299.7	86948.9	77737.7	83840.3	94510.7	96276.8	113539.6	86406.3	99740.6	89041.6	133891.7	117505.4	105790.5	115753.3
Q91YM2	Rho GTPase-activating protein 35	Arhgap35	1499	170.3	6.61	302010.8	322943.5	258828.7	300188.4	294487.3	274869.8	271648.3	253237.6	294305.6	326236.8	212900.0	216385.4	224583.0	203048.7
Q8R2M2	Deoxynucleotidyltransferase terminal-interacting protein 2	Dnttip2	758	84.2	6.44	100580.0	115415.3	112139.3	107655.2	139663.2	117995.3	118800.9	110497.2	118518.1	119039.5	224241.7	221247.3	232368.0	217020.1
Q9DB15	39S ribosomal protein L12, mitochondrial	Mrpl12	201	21.7	9.29	412713.6	391994.8	455589.2	377980.5	423470.0	391130.8	334467.9	452396.6	388352.3	324000.7	157830.6	177772.3	158260.3	162805.4
Q69ZP3	Probable hydrolase PNKD	Pnkd	385	43	8.94	118274.7	121041.9	90791.3	107655.2	103283.0	93886.1	102348.2	96938.4	106648.8	113062.4	150224.1	164908.5	120903.3	140125.5
Q9R1J0	Sterol-4-alpha-carboxylate 3-dehydrogenase, decarboxylating	Nsdhl	362	40.7	7.85	186934.2	185255.2	176535.9	207050.1	158776.7	136039.9	134421.7	144445.8	168408.7	161770.8	133475.1	143519.8	116012.6	169046.6
P50285	Dimethylaniline monooxygenase [N-oxide-forming] 1	Fmo1	532	59.9	8.47	60854.0	55239.9	58831.3	53414.9	55624.5	64339.8	77686.0	67693.1	58600.5	54245.3	76275.0	74545.3	74930.4	74165.3
Q3TSG4	RNA demethylase ALKBH5	Alkbh5	395	44.4	9.09	43995.8	56574.8	49914.0	48173.0	57938.6	50587.1	52918.3	44897.3	55692.0	57315.9	60350.4	61366.7	53729.1	62872.9
Q920A7	AFG3-like protein 1	Afg3l1	789	87	8.85	698909.7	634765.9	643032.5	633071.5	619238.1	606691.5	612556.8	655024.1	632093.0	646768.2	101069.0	138037.2	104305.4	117771.7
C0HK80	Adipocyte-related X-chromosome expressed sequence 2	Arxes2	180	20.1	9.6	836556.4	776595.0	779659.5	767089.6	827432.4	838686.2	817764.1	855051.9	802964.7	729848.7	898996.9	795769.5	806124.7	774624.1
Q9CQ10	Charged multivesicular body protein 3	Chmp3	224	25.2	5.06	225769.6	244774.4	228206.6	237791.4	248145.2	227039.2	204328.2	219783.6	231118.5	233654.3	280617.6	306779.3	297682.6	281411.6
Q8BND5	Sulfhydryl oxidase 1	Qsox1	748	82.7	7.17	240624.1	230044.1	200161.8	205334.0	193817.8	220249.9	222242.0	227117.9	212587.5	223414.7	312390.6	305776.7	274016.1	284847.7
Q8BR90	UPF0600 protein C5orf51 homolog	MGI:214623	294	33.5	5.19	339525.9	311960.6	329822.5	260583.3	328859.8	340253.1	333293.1	383516.5	302061.7	279577.1	183860.2	195569.1	198560.0	204094.4
Q80ZM5	H15 domain-containing protein	H1f10	188	20.1	11.22	446239.6	475195.9	519012.1	480447.0	566768.3	539698.5	534343.9	463154.0	497080.4	505373.5	447580.4	489033.2	571098.0	444814.8
Q9CYW4	Haloacid dehalogenase-like hydrolase domain-containing protein 3	Hdhd3	251	28	6.8	180072.7	296337.5	343545.4	340565.8	364182.4	333383.1	381977.8	198061.5	260477.8	243777.5	352277.3	445942.9	402375.1	447048.5
P10404	MLV-related proviral Env polyprotein	-	641	69.6	7.96	139239.8	138735.3	115273.4	158577.0	144219.3	137274.5	165716.9	151068.0	125186.7	127069.7	94096.6	100094.9	80507.3	117623.0
Q3U0P5	Ectonucleoside triphosphate diphosphohydrolase 6	Entpd6	455	49.8	8.62	66411.4	65919.2	64513.4	70214.4	55322.5	47549.0	52433.1	51216.9	62243.1	64694.4	178310.1	190387.4	183810.6	193796.0
Q9Z2C4	Myotubularin-related protein 1	Mtmr1	669	75.3	6.8	63095.9	74831.1	74074.0	64517.4	66349.0	67736.6	62737.9	63612.5	68705.0	64278.2	205959.7	207798.2	180349.0	197361.9
Q9Z2A0	3-phosphoinositide-dependent protein kinase 1	Pdpk1	559	63.7	7.37	123143.7	124756.8	126601.2	120545.2	123179.2	122329.3	124813.3	112333.4	121885.5	116817.9	301380.1	289344.2	260126.0	306934.7
Q9CR41	Huntingtin-interacting protein K	Hypk	129	14.7	4.93	26855.8	32057.9	31595.4	35603.2	30339.2	31129.8	27697.3	26990.4	32371.3	26378.1	133276.3	145608.7	157417.2	153266.7
Q9Z2Y8	Pyridoxal phosphate homeostasis protein	Plpbp	274	30	8.27	632054.3	684001.0	670233.5	697812.6	622023.6	638151.9	667713.1	613805.9	645681.9	698127.8	274560.1	281170.1	287385.4	272950.2
Q9WTS2	Alpha-(1,6)-fucosyltransferase	Fut8	575	66.5	7.52	405435.8	426814.0	401307.9	410889.3	442899.9	392934.2	413372.4	379834.6	397556.0	411178.2	84789.6	89573.1	84712.4	86170.1
Q8C7E9	Cleavage stimulation factor subunit 2 tau variant	Cstf2t	632	65.8	7.25	12903.9	12075.5	12069.5	13602.3	10261.9	9053.7	9827.0	9391.1	6367.8	10152.8	76601.6	86987.8	88186.2	103133.7
P26618	Platelet-derived growth factor receptor alpha	Pdgfra	1089	122.6	5.15	147561.2	127128.8	121628.8	123220.2	144777.7	156154.2	175365.7	132256.8	124314.0	126603.2	234579.2	220850.7	243473.7	219725.0
Q61160	FAS-associated death domain protein	Fadd	205	22.9	6.04	187747.0	186458.9	185369.2	167088.3	200616.1	202162.2	208527.9	201247.7	199532.8	191806.5	259074.1	252129.0	264467.3	250300.9
Q04692	SWI/SNF-related matrix-associated actin-dependent regulator of chromatin subfamily A containing DEAD/H box 1	Smarcad1	1021	116.4	5.68	127781.9	140663.0	135986.0	138330.2	137006.9	141056.1	138892.8	121879.5	146667.7	150805.9	59303.2	69022.7	64727.5	63269.8
Q0KL02	Triple functional domain protein	Trio	3102	347.6	6.35	158547.4	201717.9	148325.8	178327.3	181954.3	179150.0	157248.5	151466.8	179162.3	185650.6	168236.5	180514.2	155875.8	160715.0
E9Q7E2	AT-rich interactive domain-containing protein 2	Arid2	1828	195.9	7.47	146200.7	150536.0	148453.6	153933.0	146621.0	144065.6	134508.8	137140.5	145716.4	146538.5	97495.9	104190.8	111614.0	108992.5
P52760	2-iminobutanoate/2-iminopropanoate deaminase	Rida	135	14.2	8.68	393504.8	383851.1	378079.4	358566.3	401628.3	361057.0	381679.9	376135.0	406306.4	374300.4	306589.0	263762.4	292455.9	264546.2
Q8R323	Replication factor C subunit 3	Rfc3	356	40.5	8.38	156566.2	154927.5	154782.7	136596.0	162407.1	158933.1	152043.6	147026.7	170131.2	155363.9	214165.2	196299.8	205579.1	199358.2
P58501	PAX3- and PAX7-binding protein 1	Paxbp1	919	104.8	5.67	126942.1	144333.5	134124.7	142135.0	147428.8	139064.2	141515.5	128061.1	139300.5	138458.7	189193.0	197949.7	179766.9	189958.3
G3X9Z4	PCF11 cleavage and polyadenylation factor subunit	Pcf11	1553	172.6	8.43	116661.4	131864.6	125337.9	133064.2	127128.9	116593.7	110043.9	106744.2	105970.1	125776.7	199707.1	213375.5	205511.2	239019.5
P43025	Tetranectin	Clec3b	202	22.2	5.66	440986.8	448869.0	362611.0	403190.7	358151.3	490359.0	397083.7	543929.2	381831.0	382523.7	300493.2	323818.1	264009.7	300440.9
Q9CWG8	Protein arginine methyltransferase NDUFAF7, mitochondrial	Ndufaf7	436	48.4	6.95	96702.1	104378.4	102407.7	102161.2	98978.6	99391.8	93971.0	86572.1	114527.6	103255.0	51409.2	62512.2	56362.0	58936.1
Q8BV13	COP9 signalosome complex subunit 7b	Cops7b	264	29.7	6.32	261124.5	267128.6	255999.6	284275.6	278241.6	294953.0	275313.4	259675.6	267859.6	266408.6	266690.5	296636.8	298329.8	302716.4
Q91WS0	CDGSH iron-sulfur domain-containing protein 1	Cisd1	108	12.1	9.06	1143068.9	1270308.1	1257612.9	1552480.7	900291.6	872033.4	862255.8	1087248.5	1162562.3	1118811.2	215585.9	188693.5	246396.9	220101.8
P63168	Dynein light chain 1, cytoplasmic	Dynll1	89	10.4	7.4	606002.2	1359638.6	430300.8	924089.7	807058.8	571686.4	422671.7	567893.5	705975.6	894674.5	274170.2	277548.3	277174.2	330269.4
Q7TPM6	Fibronectin type III and SPRY domain-containing protein 1	Fsd1	496	55.5	6.67	122952.5	128068.7	116724.1	118222.5	128546.6	123725.7	117937.5	115301.8	125088.9	127999.5	82721.5	79677.3	77688.5	77539.9
Q8VDG6	Mitogen-activated protein kinase kinase kinase 21	Map3k21	1002	110	8.19	188380.2	226653.6	224391.3	218550.4	203247.7	185097.6	177214.5	173335.4	207479.3	215565.0	224790.2	180549.9	214241.3	183878.9
Q8BTY1	Kynurenine--oxoglutarate transaminase 1	Kyat1	424	47.5	6.95	178926.7	206163.3	185212.5	204632.5	198065.0	197266.0	180595.7	174642.8	206115.0	186134.8	168514.1	191825.5	188149.6	191410.1
Q91VU0	Protein FAM3C	Fam3c	227	24.7	8.29	296214.8	242960.1	210555.7	246532.5	249856.3	248003.2	241577.6	256448.2	273034.3	279079.2	137096.0	149661.5	128974.1	144478.3
Q59J78	NADH dehydrogenase [ubiquinone] 1 alpha subcomplex assembly factor 2	Ndufaf2	168	19.6	8.25	223196.6	281864.3	227784.0	280144.9	204724.2	220474.5	187735.9	197397.5	221323.9	220733.7	170543.7	188709.1	140564.9	139814.0
P21126	Ubiquitin-like protein 4A	Ubl4a	157	17.8	8.44	508100.0	454455.2	432533.6	477130.9	473254.4	456649.7	443073.8	482974.1	500408.1	464160.1	348501.0	346424.0	309686.7	329933.4
P97765	WW domain-binding protein 2	Wbp2	261	28	6.33	450158.8	470480.3	424432.7	490847.8	442634.5	414532.1	474202.4	407538.7	437348.7	482085.8	390446.3	414715.0	399974.7	453582.5
G5E8K5	Ankyrin-3	Ank3	1961	213.9	6.7	124451.4	125090.3	128142.9	131569.7	155932.4	167211.2	170470.1	157405.4	141470.5	128705.3	154210.7	143965.6	133903.2	138846.2
Q8BU11	TOX high mobility group box family member 4	Tox4	619	65.9	5.01	95364.9	80052.1	91937.6	77638.9	92513.5	93573.8	91878.3	91437.4	82187.4	91746.3	594244.5	570940.2	613041.1	596663.1
Q9DAP0	Leucine-rich repeat-containing protein 46	Lrrc46	323	36	5.39	146884.7	176073.7	166582.2	186559.4	137344.4	130667.4	125748.9	126994.0	158447.8	149464.4	43682.6	41150.8	43504.3	100771.3
Q8C033	Rho guanine nucleotide exchange factor 10	Arhgef10	1345	147.9	5.73	182270.0	138338.5	146191.1	139597.1	153412.5	150963.9	139770.5	141370.7	129854.8	166441.5	148000.9	144276.9	127118.9	140243.4
Q9DC40	Telomere length regulation protein TEL2 homolog	Telo2	840	93.3	5.25	64642.1	68676.8	54100.9	65826.4	63509.7	63920.7	55756.5	57018.3	57635.6	63118.3	114599.6	150022.7	109012.7	121937.9
Q61127	NGFI-A-binding protein 2	Nab2	525	56.5	6.86	225456.3	235841.6	204909.8	223341.7	232361.5	259049.5	250053.1	245141.4	234965.1	231461.6	180894.6	174677.3	172405.8	159504.0

Q8C3F2	Constitutive coactivator of PPAR-gamma-like protein 2	Fam120c	1091	119.6	8.46	253132.5	269348.6	249623.9	241599.1	253115.2	252420.8	247663.9	243181.3	250681.3	264177.7	46908.5	58717.8	49274.6	50205.6
Q9DB25	Dolichyl-phosphate beta-glucosyltransferase	Alg5	324	36.8	8.6	255732.0	238348.4	249364.4	243189.2	244838.1	233716.5	224824.1	218864.7	230744.5	235273.7	292582.9	284813.1	296674.5	277540.8
Q99JW5	Epithelial cell adhesion molecule	Epcam	315	35	5.95	127474.1	105299.0	121016.6	150277.7	125790.9	183224.9	286592.3	241999.4	137252.9	113845.2	107520.7	100820.9	101670.3	120725.6
A2RSQ1	Beta-galactosidase-1-like protein 3	Glb1l3	649	73.9	7.62	926954.8	1021538.9	667180.0	920248.2	570277.2	544067.0	562143.7	869058.5	652076.4	620453.6	420591.0	421483.5	304233.5	408879.5
Q8BXZ1	Protein disulfide-isomerase TMX3	Tmx3	456	51.8	5.16	837804.9	791113.6	841606.1	833791.3	802868.8	782893.8	877090.9	804953.4	813708.8	732218.0	537589.5	522171.8	523541.6	545777.5
Q9CQJ4	E3 ubiquitin-protein ligase RING2	Rnf2	336	37.6	6.84	456598.7	435535.2	443573.4	420964.0	477628.6	455686.0	459700.8	439463.9	446149.3	465887.8	354273.5	336787.9	352770.7	350130.7
Q9JLZ3	Methylglutaconyl-CoA hydratase, mitochondrial	Auh	314	33.4	9.51	583025.7	510204.2	450516.2	603513.7	542425.9	523229.5	586452.3	615736.7	486361.1	589758.2	540863.5	556195.5	499246.0	545011.8
P48725	Pericentrin	Pcnt	2898	329.3	5.36	32462.8	50462.5	33804.6	35781.2	39763.8	38400.0	35009.1	38145.2	38782.7	38963.2	79707.4	88777.1	91099.0	97752.5
P12246	Serum amyloid P-component	Apcs	224	26.2	6.35	479817.2	530476.7	391778.1	438343.1	363761.6	335732.3	495026.9	399925.3	571156.4	507395.5	351973.7	336768.8	291253.2	311694.2
Q61735	Leukocyte surface antigen CD47	Cd47	303	33.1	8.63	265401.5	246808.5	261758.8	262329.0	305423.9	311106.2	325725.5	279245.4	273017.7	256086.8	295792.8	290621.3	273591.4	306530.8
Q68FM7	Rho guanine nucleotide exchange factor (GEF) 11	Arhgef11	1552	171.7	5.58	205759.6	216563.5	210929.2	196419.7	208338.9	193371.7	194475.9	181624.3	197816.5	220564.4	222622.7	220716.2	201803.6	214101.5
Q8K4L3	Supervillin	Svil	2170	243	6.87	104359.9	92360.2	96323.3	95789.3	80850.5	101741.2	106325.7	100063.6	94832.3	87609.2	325664.9	297517.2	280053.7	282646.1
Q9D2V7	Coronin-7	Coro7	922	100.7	5.77	37974.1	34890.0	37561.5	35794.2	34742.4	36363.6	38446.5	33141.5	35489.8	37525.2	98092.7	101818.8	92569.9	103203.6
Q80TJ7	Histone lysine demethylase PHF8	Phf8	1023	113.5	8.44	328308.7	326437.7	340670.7	302658.0	395198.8	415092.1	444629.1	341712.9	391378.1	400903.8	56846.2	67495.3	55555.8	55158.5
Q8R060	Protein zwilch homolog	Zwilch	589	66.8	6.02	127410.4	155174.4	154845.4	158597.8	144517.7	143708.6	108355.1	126929.9	152140.8	156703.1	57671.0	66936.3	65489.3	69214.0
Q3TLD5	Unconventional prefoldin RPB5 interactor	Uri1	531	59	5.07	165704.1	185069.8	172931.2	169106.6	184851.3	190206.6	182184.5	154996.4	175282.3	165963.1	117686.1	128998.9	121271.6	114844.3
Q8CFE4	SCY1-like protein 2	Scyl2	930	103.3	8.02	134711.0	174433.1	136098.7	155843.4	157626.7	171499.9	121623.8	134690.0	131704.5	147711.7	109410.7	111205.7	112365.4	119294.9
Q922J9	Fatty acyl-CoA reductase 1	Far1	515	59.4	9.19	334211.9	318057.5	318552.8	310536.4	339736.2	289651.4	288469.1	335894.7	323124.5	333180.6	59919.3	65203.2	65182.6	66328.6
Q8BU14	Translocation protein SEC62	Sec62	398	45.6	7.31	612140.3	650307.4	566461.6	623488.9	522396.6	514833.8	473413.0	540956.4	561142.1	538159.9	402216.0	444460.0	399254.0	408776.9
Q8R570	Synaptosomal-associated protein 47	Snap47	413	46.5	5.76	36855.8	43294.9	36933.9	39243.8	42581.0	40073.6	41912.5	39580.3	42714.6	42389.8	111150.1	119049.7	111102.1	124211.1
P16675	Lysosomal protective protein	Ctsa	474	53.8	5.86	99382.4	140879.5	103057.1	112515.6	116755.3	110852.3	101219.9	97113.4	108680.9	121957.8	51507.9	55351.8	51297.6	50429.0
Q02780	Nuclear factor 1 A-type	Nfia	532	58.5	8.56	385948.5	421338.7	343675.1	385016.8	461625.0	504464.0	684739.2	421107.6	464795.2	492868.1	217512.0	239183.5	180854.7	215237.5
Q6QD59	Vesicle transport protein SEC20	Bnip1	228	26.2	8.75	160450.6	187809.6	168309.2	162259.8	178937.6	172172.8	170797.9	161966.5	173953.9	175337.8	169562.2	186293.0	169493.2	168890.0
Q9D023	Mitochondrial pyruvate carrier 2	Mpc2	127	14.3	10.61	425550.5	509676.4	493872.0	514151.0	525822.8	495198.1	453281.9	452367.0	523545.3	516612.5	886706.9	1015629.8	1109389.5	1171994.7
Q9DBX2	Phosducin-like protein	Pdcl	301	34.4	4.87	140732.6	141037.8	128958.1	131251.5	141261.3	146731.4	134241.0	129648.5	131092.1	132857.1	81509.8	79706.2	74265.8	83266.5
P62774	Myotrophin	Mtpn	118	12.9	5.52	594270.8	592796.6	760889.9	633641.5	841363.5	759773.7	552229.2	885337.4	684442.7	529597.7	761622.0	876400.5	794899.7	946841.4
P83877	Thioredoxin-like protein 4A	Txnl4a	142	16.8	5.85	165367.7	212754.7	225510.2	208592.7	205258.9	226264.5	206332.0	194249.8	201184.1	214817.1	83833.5	76663.8	72447.6	78844.1
Q9ERG2	Striatin-3	Strn3	796	87.1	5.29	100757.3	103274.9	99588.8	105851.1	100570.0	109078.0	101728.7	93557.6	100377.4	98342.6	379494.8	406192.4	379017.6	386506.6
Q8BVN9	Protein maelstrom homolog	Mael	434	49.3	8.46	86380.6	98274.8	96964.4	92511.0	92226.8	86296.1	82037.5	85073.6	94034.4	90049.8	120538.3	130107.7	125343.1	139997.2
Q06185	ATP synthase subunit e, mitochondrial	Atp5me	71	8.2	9.35	345719.4	378086.9	286226.3	388479.6	288722.2	234802.9	220814.2	202560.2	327353.0	310788.7	1768290.6	1659913.6	2002867.4	1929939.3
H3BKQ3	Rab-GAP TBC domain-containing protein	Evi5l	813	93.4	5.25	77769.2	96092.2	79266.6	84037.6	81327.8	79507.3	84096.5	74069.1	83152.4	87114.4	62103.4	62392.9	53295.3	61067.6
Q9QYH6	Melanoma-associated antigen D1	Maged1	775	85.6	7.5	137274.8	151309.4	139413.0	142506.9	158554.6	149367.8	138515.8	143358.7	148724.9	159417.8	203534.0	194632.5	149087.0	177399.9
Q8BZ09	Mitochondrial 2-oxodicarboxylate carrier	Slc25a21	298	33.2	9.72	325337.7	354325.6	319513.8	317700.1	316109.9	302668.2	347329.2	293021.4	332869.9	316630.3	526334.5	508919.2	467935.2	473582.3
Q6GV12	3-ketodihydrosphingosine reductase	Kdsr	332	35.9	7.9	151443.1	159517.9	135099.0	135215.1	151237.4	156850.5	142103.3	135517.1	147802.4	157949.9	177770.6	155000.1	171818.6	155076.4
Q8BGZ4	Cell division cycle protein 23 homolog	Cdc23	597	68.5	7.18	148545.5	168533.4	129527.2	136829.0	158105.7	150634.4	138330.3	134079.6	134308.0	147343.3	409147.0	396035.8	382778.3	380477.3
Q9CXZ1	NADH dehydrogenase [ubiquinone] iron-sulfur protein 4, mitochondrial	Ndufs4	175	19.8	9.99	269217.7	307354.8	270845.3	299357.5	312059.3	324635.8	299120.1	322748.9	296464.4	289881.8	328829.0	301738.3	370015.5	316969.0
Q9Z0K8	Pantetheinase	Vnn1	512	57.1	5.94	127225.4	118970.7	124525.7	125388.8	171262.9	207047.1	191561.5	154908.5	136607.6	132571.5	87400.9	87770.5	76906.2	85919.1
Q3U3R4	Lipase maturation factor 1	Lmf1	574	65.8	9.29	56267.6	65856.3	52589.1	69420.5	55678.9	52233.3	59732.4	54311.1	56347.2	60056.2	131089.2	146370.6	141240.9	132516.4
Q924T3	DNA repair protein XRCC4	Xrcc4	326	37	5.19	131690.9	176058.0	167988.1	182262.3	129790.6	125313.8	114371.8	124850.7	144991.4	159416.1	233198.6	246358.1	258177.5	288176.0
Q8VDK1	Deaminated glutathione amidase	Nit1	323	35.7	7.94	59011.5	83418.9	81533.4	78616.1	80821.3	85555.4	62232.9	79745.7	73479.2	77087.2	20437.2	21941.3	22456.8	20726.2
Q6PDY2	2-aminoethanethiol dioxygenase	Ado	256	28.4	5.97	88802.7	81945.2	92146.5	77178.0	85812.0	84201.4	96109.0	92345.5	84061.4	78533.5	81236.2	74449.6	66873.2	74902.4
Q61806	X-linked lymphocyte-regulated protein 3C	Xlr3c	226	26.2	8.75	123754.3	142149.6	141940.7	142320.2	152252.9	153932.6	133705.1	128684.3	144486.4	136687.9	120970.1	139762.6	141736.4	139655.8
O09012	Peroxisomal targeting signal 1 receptor	Pex5	639	70.7	4.55	83512.7	91995.9	83584.3	83166.1	92147.8	93064.9	85119.2	74887.6	81968.1	81549.2	111925.8	135171.0	134197.5	167560.7
Q91YJ3	Thymocyte nuclear protein 1	Thyn1	226	26.2	9.11	344493.0	335869.0	365680.4	334225.9	370122.3	356548.2	349422.8	343546.0	363469.0	357737.6	166451.0	179236.8	185758.3	175317.7
Q5ND34	WD repeat-containing protein 81	Wdr81	1934	211.8	5.77	55535.4	62785.4	51087.4	50192.1	63023.4	61145.3	47006.8	55856.1	54564.1	60051.4	110305.0	125269.1	98910.3	114128.0
Q811G0	Protein PTHB1	Bbs9	885	99	6.13	100929.6	127171.7	111530.0	123292.0	122225.4	116839.5	111097.7	114480.7	112473.5	116945.4	71736.0	83051.7	71178.1	83159.0
P52840	Sulfotransferase 1A1	Sult1a1	291	34	8.1	307700.3	351218.5	255015.5	310043.8	387442.6	307696.1	315077.8	290896.9	315408.5	353101.9	394908.8	381252.9	344552.3	407371.7
Q8BFT2	HAUS augmin-like complex subunit 4	Haus4	363	42.2	5.81	132821.2	120132.2	118273.1	146222.5	122298.9	125354.4	134691.0	135298.5	149803.6	125347.8	153419.1	183820.3	178496.6	186981.8
P59326	YTH domain-containing family protein 1	Ythdf1	559	60.8	8.95	225717.4	249166.3	256889.2	237966.1	271954.4	274864.5	261513.5	263528.3	253448.3	243430.8	221732.2	195070.6	214046.4	205419.8
Q8VDM1	Zinc finger CCCH-type with G patch domain-containing protein	Zgpat	511	56.4	5.33	90215.2	95192.6	82857.3	91873.9	99331.1	93424.0	94941.3	80814.7	97698.6	96321.6	130743.9	125427.9	119159.2	135304.5
Q8BMS4	Ubiquinone biosynthesis O-methyltransferase, mitochondrial	Coq3	370	40.9	7.96	226123.3	222346.8	234974.7	253518.6	230250.3	217540.8	221223.2	207719.0	237053.8	241921.7	237916.7	251023.9	261476.4	253786.2
Q9ESK9	RB1-inducible coiled-coil protein 1	Rb1cc1	1588	182.2	5.49	55971.0	56473.0	51438.1	56023.4	50909.3	65028.0	53816.1	46463.6	60109.4	59235.8	71588.0	74332.4	71809.4	75523.8
Q07813	Apoptosis regulator BAX	Bax	192	21.4	4.98	123134.3	213841.9	127659.8	150065.4	165097.1	192450.9	129050.3	189467.0	142934.2	175688.7	87047.9	104135.9	88696.1	88694.2
Q9CPP0	Nucleoplasmin-3	Npm3	175	19	4.82	96359.9	100694.7	84374.9	89717.1	82937.5	82489.5	77421.0	76995.1	94819.0	94485.1	52157.7	55123.4	46194.4	53572.6
Q8BFU3	RING finger protein 214	Rnf214	668	73.6	6.23	524250.3	542402.0	555403.2	512343.8	549543.3	551293.1	547765.4	479209.4	542955.6	542045.3	208373.4	194620.4	189659.5	198487.0
Q9Z0E1	Meiosis 1 arrest protein	M1ap	529	58.6	6.73	113498.7	137477.6	135392.9	117868.1	142490.8	152982.0	114131.0	124354.6	148573.7	145310.0	77119.2	88744.6	81795.3	75369.5
Q8VCR7	Protein ABHD14B	Abhd14b	210	22.4	6.27	160310.2	175693.6	129091.1	171468.0	135997.0	152587.3	198624.8	151755.2	164628.5	154128.4	203434.0	196284.5	176029.0	195284.1
Q99LJ1	Tissue alpha-L-fucosidase	Fuca1	452	52.2	6.95	175343.5	183581.8	152494.3	181604.9	137639.9	155936.0	172905.4	131711.9	164269.1	162385.9	191497.1	181649.3	180081.4	200407.2
Q14CH7	Alanine--tRNA ligase, mitochondrial	Aars2	980	106.7	6.18	67032.8	78387.3	63576.5	60479.7	58766.4	63400.9	56117.6	58160.4	61435.1	70053.9	53097.9	58332.3	54654.6	48516.2
Q80VJ3	2'-deoxynucleoside 5'-phosphate N-hydrolase 1	Dnph1	173	19	5.38	210983.2	237722.8	210322.9	241230.6	208771.6	191483.6	192801.8	202783.0	226023.8	234225.0	141417.0	163748.0	161580.5	154720.2
Q5U3K5	Rab-like protein 6	Rabl6	725	79.8	5.53	33640.0	36148.9	40530.4	34451.2	37482.4	32291.4	35434.4	31794.8	35742.9	34811.5	23167.4	24083.7	23426.1	24890.1
Q9JJL8	Serine--tRNA ligase, mitochondrial	Sars2	518	58.3	7.9	191862.8	213550.0	169861.6	198942.6	195651.5	191162.4	180002.4	177207.9	204678.1	210813.9	197197.4	250087.1	181799.5	186622.2
Q80XU3	Nuclear ubiquitous casein and cyclin-dependent kinase substrate 1	Nucks1	234	26.3	5.14	95750.0	85908.7	105490.7	93778.0	110713.9	116654.2	118697.1	93811.1	108000.4	87315.0	97417.2	75814.8	90940.1	69495.8
Q3THW5	Histone H2A.V	H2az2	128	13.5	10.58	275650.1	351617.4	303067.0	287535.8	350290.9	338338.1	373576.7	296479.3	278046.1	315822.8	390063.4	389281.4	454431.1	392402.1
O35075	Vacuolar protein sorting-associated protein 26C	Vps26c	297	32.9	7.68	110300.3	107508.2	90086.2	109247.9	104484.7	96148.7	98922.5	93239.1	100923.7	105639.7	146570.7	154508.9	149690.3	150842.2
Q920R0	Alsin	Als2	1651	182.5	6.3	50754.0	58905.3	48527.9	43120.3	59351.5	50506.1	51023.7	45417.8	51131.3	56053.3	146075.6	163395.8	158359.5	154319.1
Q99J39	Malonyl-CoA decarboxylase, mitochondrial	Mlycd	492	54.7	8.94	151136.4	167987.7	131903.7	148496.9	154665.3	160019.1	142573.6	131336.9	125469.3	141455.9	22762.3	27150.3	18960.1	26253.2
P01898	H-2 class I histocompatibility antigen, Q10 alpha chain	H2-Q10	325	37.2	5.25	129328.9	125146.1	99653.2	125565.4	95173.6	97757.8	137495.2	107443.5	104852.3	102862.7	176239.2	167220.4	152860.1	153621.1
P97412	Lysosomal-trafficking regulator	Lyst	3788	425	6.6	119870.9	122064.4	113809.4	112653.4	121115.8	127105.1	113668.2	108901.8	124473.4	126260.6	51212.7	49289.0	43376.4	48269.1
P49452	Centromere protein C	Cenpc	906	102.1	9.67	101096.5	127261.5	102428.3	123105.0	106664.2	111317.3	92404.6	94261.7	108532.4	116564.8	209931.4	265373.8	221967.5	249902.6
Q62165	Dystroglycan	Dag1	893	96.8	8.44	65612.4	58961.1	71704.4	65500.6	69650.1	67966.8	77842.4	65700.9	68739.2	71501.1	102676.4	104880.7	105031.6	104255.7
E2JF22	Piezo-type mechanosensitive ion channel component 1	Piezo1	2547	291.8	7.5	171837.1	201302.7	190119.4	179606.7	195525.9	205812.8	178930.0	180796.8	188533.5	189184.6	68611.9	62487.0	66965.8	61645.5
Q505B8	Synaptonemal complex central element protein 2	Syce2	171	19.5	5.2	178119.2	195064.4	222184.5	194651.9	207777.0	187841.5	153738.3	187631.9	185401.4	176514.6	288165.0	337625.9	349179.7	351400.8
Q61371	Intraflagellar transport protein 88 homolog	Ift88	824	92.9	5.9	119602.4	142513.5	141856.2	163672.7	117551.6	102386.7	95272.2	111832.1	123365.4	133453.7	62342.2	68861.0	72971.6	76977.5
Q8BMB3	Eukaryotic translation initiation factor 4E type 2	Eif4e2	245	28.2	8.87	306767.3	335169.4	301682.3	308409.0	324881.7	327266.7	291477.6	308892.4	325516.1	318902.2	272433.0	270323.7	256348.9	272628.9
B2KF50	Chorein_N domain-containing protein	Uhrf1bp1	1429	156.9	6.21	84185.7	87039.5	75584.3	89783.2	82543.6	81972.8	80939.1	74882.6	73596.8	80388.3	435984.9	465297.3	450227.7	426515.7
A0A1Y7VJN6	Immunoglobulin heavy constant gamma 3	Ighg3	399	44	7.14	193132.0	111000.7	86259.0	161942.6	126651.5	102503.2	90679.1	193129.0	102113.9	94373.6	358693.2	109365.4	70676.7	244968.7
Q8BGF3	WD repeat-containing protein 92	Wdr92	357	39.8	8.15	379862.1	439610.9	416529.4	517545.4	311149.1	297601.3	292140.0	302307.7	369764.3	415482.2	237259.8	276484.4	278966.3	301346.3
P55144	Tyrosine-protein kinase receptor TYRO3	Tyro3	880	96.1	5.92	87875.7	73202.3	67412.9	84518.3	72941.0	76361.9	69678.9	65384.3	72891.0	70134.3	41164.4	45824.0	41003.3	44332.3
Q8C7V3	U3 small nucleolar RNA-associated protein 15 homolog	Utp15	528	59.3	8.79	250352.1	261991.8	214617.5	194072.1	233639.1	234671.4	216713.9	198680.1	213889.8	245671.7	140583.1	156667.5	141911.5	160317.4
Q9DBN9	Probable ATP-dependent RNA helicase DDX59	Ddx59	619	68.2	6.98	77280.4	72144.2	75640.5	77249.5	75925.0	79444.4	74414.3	62262.0	76655.7	79359.1	180525.5	197297.5	187749.8	184866.5
Q3TL44	NLR family member X1	Nlrx1	975	107.8	7.37	159547.7	274628.9	148715.3	224531.2	234748.9	206565.3	152640.4	189632.0	194429.3	239481.0	97655.2	106566.3	99749.0	97076.4
Q8CGC6	RNA-binding protein 28	Rbm28	750	84.2	9.54	124821.2	128678.8	116900.3	129004.3	132606.8	113180.9	110711.5	128812.1	132635.0	133842.6	308212.6	309110.8	289128.3	284994.7
P55821	Stathmin-2	Stmn2	179	20.8	8.32	162968.1	152675.6	220230.1	149785.0	188958.4	157745.6	195327.0	152486.7	182396.1	200267.6	#N/A	#N/A	#N/A	#N/A
Q8K2J0	1-phosphatidylinositol 4,5-bisphosphate phosphodiesterase delta-3	Plcd3	785	88.6	6.83	136942.5	146798.1	147206.3	140100.2	150201.8	146330.6	139184.9	139983.8	134017.6	142694.8	179262.3	197699.9	187241.8	185060.0
Q5XG73	Acyl-CoA-binding domain-containing protein 5	Acbd5	508	56.6	5.41	138809.3	142714.3	127057.7	121761.7	143261.5	142629.1	117342.5	132400.7	127231.8	130685.6	131800.6	110173.4	114309.9	104069.7
Q8BZF8	Phosphoglucomutase-like protein 5	Pgm5	567	62.2	7.18	73992.5	82673.1	77422.7	85530.7	81239.5	100976.0	110124.3	137328.0	82716.4	80102.7	85149.1	82616.7	79886.8	91245.5
P62315	Small nuclear ribonucleoprotein Sm D1	Snrpd1	119	13.3	11.56	268267.2	294627.3	343714.4	299774.9	360356.5	314233.0	294267.3	293141.6	300659.4	314874.1	533779.8	570405.6	646549.3	590131.8
Q8K1N1	Calcium-independent phospholipase A2-gamma	Pnpla8	776	87.3	9.23	269893.6	256969.5	247295.8	255617.7	252229.7	232452.6	229283.7	259950.2	244305.1	260627.4	440782.2	466438.2	430778.9	423364.0
Q8CIF4	Biotinidase	Btd	520	58.1	5.8	130333.7	125475.8	112263.8	117130.2	122953.9	152850.1	135731.5	130468.9	116352.3	121884.4	138458.0	140595.9	151631.2	133592.6
Q3TIU4	2',5'-phosphodiesterase 12	Pde12	608	67.5	6.92	460832.8	453953.4	422172.7	414891.7	379063.1	417595.4	410613.1	486835.7	452986.1	433214.2	193481.1	210656.3	202659.0	205435.1
Q9QZH3	Peptidyl-prolyl cis-trans isomerase E	Ppie	301	33.4	5.6	86928.1	89808.0	87820.1	88611.3	106814.8	103031.7	90989.7	88760.9	97351.0	103767.2	50582.7	56882.7	56669.3	55956.8

Q8BG95	Protein phosphatase 1 regulatory subunit 12B	Ppp1r12b	976	109	5.74	6032.3	9975.4	13417.7	9956.8	12244.7	15282.5	16154.9	13461.1	12094.6	8056.5	705911.6	801037.6	691584.0	745905.4
P62746	Rho-related GTP-binding protein RhoB	Rhob	196	22.1	5.24	102851.7	105066.1	80520.1	99542.8	90247.5	93601.1	104660.3	94547.2	90392.9	97121.9	74500.7	81935.5	53936.3	69315.0
Q8BUI3	Leucine-rich repeat and WD repeat-containing protein 1	LRWD1	648	71.5	7.87	137334.9	161435.9	135223.9	153146.7	145011.4	143597.2	137978.1	136649.3	151800.3	151960.6	74005.6	96587.3	72206.6	74073.4
Q4FK66	Pre-mRNA-splicing factor 38A	Prpf38a	312	37.4	10.01	190885.4	216530.8	207662.9	213070.9	216224.8	294323.8	192356.7	188456.4	212135.0	229022.7	202771.6	205119.7	191734.6	199462.5
Q9ERE3	Serine/threonine-protein kinase Sgk3	Sgk3	496	57.1	7.15	154535.0	153080.0	165618.1	156406.5	149358.3	147065.8	155142.4	143902.6	152792.4	146578.5	190318.2	213417.4	183869.5	204410.0
Q3TPE9	Ankyrin repeat and MYND domain-containing protein 2	Ankmy2	440	48.7	6.67	79284.0	69567.7	70891.6	61361.5	63847.6	67293.0	66270.7	81111.1	77588.4	63965.3	76387.4	68914.9	65275.4	60090.4
P52432	DNA-directed RNA polymerases I and III subunit RPAC1	Polr1c	346	39.1	5.21	359799.5	350475.6	332264.2	349762.7	363227.3	349436.8	331556.1	317123.6	347024.0	363335.6	331684.1	339865.6	325287.5	313156.3
Q8C6B0	Methyltransf_11 domain-containing protein	Mettl7a1	244	28.1	8.31	202839.6	204739.4	180799.1	219213.3	188088.6	194798.4	228063.2	176514.1	197913.6	208224.7	49815.1	46348.3	52078.6	57789.8
Q9EQ80	NIF3-like protein 1	Nif3l1	376	41.7	6.76	390057.1	399416.3	368932.3	392542.3	389934.5	361469.4	383840.8	362127.2	407835.1	402831.8	265321.4	272337.7	270790.5	275096.8
Q62158	Zinc finger protein RFP	Trim27	513	58.5	6.39	103268.8	122647.2	101327.0	119749.2	113958.2	99984.3	95353.6	93285.7	113009.6	104563.0	178873.6	207006.9	178356.0	207510.2
Q9D898	Actin-related protein 2/3 complex subunit 5-like protein	Arpc5l	153	17	6.8	242168.4	258274.9	233595.1	237247.1	245379.0	250890.3	256601.4	227296.9	260651.8	260504.2	238160.6	242683.0	233488.9	242036.5
Q9Z1Q2	Phosphatidylserine lipase ABHD16A	Abhd16a	558	63	8.25	150389.0	149703.1	155489.3	136797.7	156956.1	133273.9	153089.6	130942.8	154391.3	162767.5	160501.4	177481.7	172110.7	172112.4
Q8K3A9	7SK snRNA methylphosphate capping enzyme	Mepce	666	72	9.25	76603.0	69847.9	74178.5	67821.2	82132.4	80366.1	80333.7	75145.6	75735.2	66204.1	82488.0	76780.3	98072.6	82826.2
D3YTP3	Metaxin	Mtx3	312	34.9	7.99	229158.3	240112.1	225919.9	237568.8	238183.7	241839.7	241150.5	232796.4	240800.0	242681.9	296891.1	274292.6	266288.4	253442.4
P97454	Mothers against decapentaplegic homolog 5	Smad5	465	52.1	7.71	445830.9	437564.7	464158.7	403428.0	479133.7	479620.3	473840.6	429371.9	432761.3	454473.8	#N/A	#N/A	#N/A	#N/A
P08032	Spectrin alpha chain, erythrocytic 1	Spta1	2415	279.7	5.03	77907.7	86161.0	66870.0	89360.0	79488.8	89542.0	96146.7	80466.6	81735.9	82702.1	98296.0	103560.0	81655.4	96086.2
P59481	VIP36-like protein	Lman2l	347	39.9	9.41	775728.8	764496.1	795839.9	797333.5	762873.3	808909.0	728316.5	763065.0	797525.5	768372.0	294761.1	275416.1	299548.9	264656.8
P31266	Recombining binding protein suppressor of hairless	Rbpj	526	58.5	8.13	88880.7	94088.4	94061.3	94417.3	110970.9	110358.0	92405.5	98719.0	101246.2	97866.2	#N/A	#N/A	#N/A	#N/A
Q60809	CCR4-NOT transcription complex subunit 7	Cnot7	285	32.7	4.84	160204.5	152399.3	149012.8	163183.8	149717.2	139596.0	149511.4	153098.0	148090.1	157031.2	44158.3	40692.9	37014.5	38832.2
P59235	Nucleoporin Nup43	Nup43	380	42	5.39	175994.3	187894.3	186775.3	178422.5	212729.4	224910.8	196879.9	169139.7	190797.6	208986.9	52145.1	53026.8	51804.5	52720.7
P97363	Serine palmitoyltransferase 2	Sptlc2	560	62.9	8.18	236721.0	269274.3	240408.2	256628.3	265434.7	247554.7	209575.3	230888.1	246393.4	257110.7	58967.3	59021.4	68145.7	61827.9
Q91ZT8	Ankyrin repeat and SOCS box protein 9	Asb9	290	31.6	7.56	197558.3	209186.9	165638.8	175908.3	204303.0	174135.5	176847.9	187568.0	158710.1	172652.3	473783.8	459968.4	417558.3	480762.2
Q91VA6	Polymerase delta-interacting protein 2	Poldip2	368	41.8	8.63	480727.4	512513.7	482493.7	471475.5	502281.3	502547.2	420671.8	423640.3	498170.4	523295.4	144900.6	155374.6	139516.7	144191.7
Q8BLY2	Threonine--tRNA ligase 2, cytoplasmic	Tars3	790	91.3	7.53	76253.1	88499.6	85585.8	79878.2	84741.4	87709.9	75704.3	71545.3	78564.4	97838.1	76019.6	86477.6	84409.5	86520.2
O88286	Protein Wiz	Wiz	1684	184.2	7.01	141550.0	135576.1	139094.2	150133.7	141707.0	138542.3	145134.3	134455.1	142848.6	142377.8	189692.2	209740.6	110123.9	111907.2
Q3UUI3	Acyl-coenzyme A thioesterase THEM4	Them4	230	26	9.64	75207.5	75749.5	77425.6	94334.0	71983.3	64102.1	69352.7	73648.8	75108.7	80847.2	101448.1	122163.7	120813.3	140606.8
Q9CWU2	Palmitoyltransferase ZDHHC13	Zdhhc13	622	70.8	8.19	176977.7	196436.8	182941.6	200426.4	190800.7	193978.0	161951.0	183563.6	176604.3	190757.6	104774.5	107853.0	105502.0	126593.8
Q6VNB8	WD repeat and FYVE domain-containing protein 3	Wdfy3	3508	392.1	6.8	108848.4	110046.8	98808.8	106015.2	112834.5	115982.1	108282.3	115264.3	105819.2	108456.0	76391.9	81643.4	70442.4	78912.7
Q99M28	RNA-binding protein with serine-rich domain 1	Rnps1	305	34.2	11.84	97156.2	123972.2	86588.6	111115.7	102139.7	97152.7	93196.0	98484.2	100925.8	112993.3	620032.7	657568.3	842156.5	711534.6
Q921J4	Ubiquitin-conjugating enzyme E2 S	Ube2s	223	24.2	7.91	72819.3	88603.8	95092.9	79374.7	91678.1	90079.8	71895.5	74155.7	85964.7	82568.6	338008.2	401232.2	365254.9	386457.8
Q9D7A6	Signal recognition particle 19 kDa protein	Srp19	144	16.2	9.96	152958.6	164452.3	145896.9	141034.6	153547.5	151828.0	140702.8	141581.8	163351.8	163814.0	149266.7	149814.5	154598.0	152754.4
P13634	Carbonic anhydrase 1	Ca1	261	28.3	6.96	99592.4	95815.1	104506.8	96112.1	122308.3	120788.3	169658.7	111409.1	111746.7	114550.1	38354.8	40850.3	47493.4	47662.5
P70257	Nuclear factor 1 X-type	Nfix	488	53.4	8.68	205676.1	230196.5	250502.7	211331.2	302313.6	312263.6	280036.4	240014.2	222966.9	222767.5	108348.9	10854.6	9317.3	9801.7
Q3UIU2	NADH dehydrogenase [ubiquinone] 1 beta subcomplex subunit 6	Ndufb6	128	15.5	9.79	720218.8	744963.9	761416.1	797596.1	820235.0	757652.0	787965.6	762760.1	786930.6	752799.3	483586.4	512190.7	498157.7	533606.2
Q62210	Baculoviral IAP repeat-containing protein 2	Birc2	612	69.6	6.54	90053.5	92977.0	87783.4	85555.5	110207.0	96052.8	88150.0	81429.2	95325.1	95050.9	92103.0	106768.2	106112.7	102247.1
O88874	Cyclin-K	Ccnk	554	61.3	8.41	81978.7	96131.9	115955.2	92695.1	103672.4	101321.5	80928.5	93816.4	108471.7	91666.1	144783.2	134434.2	150898.4	144395.4
Q9JIS8	Solute carrier family 12 member 4	Slc12a4	1085	120.5	6.67	102761.7	106255.5	98312.4	103052.4	98200.7	99531.0	100358.7	90840.6	100732.5	115303.2	448563.1	448372.6	439663.7	435496.4
Q9Z0Y1	Dynactin subunit 3	Dctn3	186	21	6.06	219455.7	214108.4	232161.6	220857.5	234975.0	237647.3	175431.5	227280.8	188361.3	208591.2	272777.5	283013.2	300230.3	298987.1
P70351	Histone-lysine N-methyltransferase EZH1	Ezh1	747	85.1	7.58	155028.1	176939.2	167732.2	168135.7	185043.2	182921.3	165882.3	155768.7	169600.4	186664.2	172187.5	178357.4	183030.5	169473.1
Q9Z0E0	Neurochondrin	Ncdn	729	78.8	5.54	244926.0	286212.3	239839.8	254853.9	275632.0	240031.6	217906.4	229627.4	249219.5	270003.9	64908.5	92376.6	77463.9	73632.7
Q8BH48	Ubiquitin-associated protein 1	Ubap1	502	55	5.07	99070.5	108847.0	115560.2	106484.3	119193.3	119939.9	102126.8	106034.0	103630.5	120171.4	391321.9	414219.9	409858.1	388956.4
Q61880	Meiotic recombination protein DMC1/LIM15 homolog	Dmc1	340	37.8	6.04	150478.2	174645.5	145015.8	156746.6	168748.8	180958.2	141552.6	137751.3	167513.6	166722.0	143834.4	144982.3	151176.2	147351.9
P97376	Protein FRG1	Frg1	258	29.1	8.92	255722.2	254950.7	272054.8	217794.7	311703.3	278855.7	275454.7	271016.3	291275.4	296419.1	171997.2	176360.4	165628.9	157857.7
P46414	Cyclin-dependent kinase inhibitor 1B	Cdkn1b	197	22.2	7.02	49156.4	58913.1	49429.1	51643.1	56110.6	59941.0	69434.2	52124.7	66546.0	54835.5	56647.6	56126.1	54094.1	51268.2
Q9CXI0	2-methoxy-6-polyprenyl-1,4-benzoquinol methylase, mitochondrial	Coq5	327	37.3	7.49	126610.6	122655.0	123004.0	122395.6	120364.1	114393.6	121161.8	104405.5	127157.4	127385.6	70814.9	69109.4	72140.9	71074.3
Q9JIM1	Equilibrative nucleoside transporter 1	Slc29a1	460	50.2	8.31	219220.8	182476.7	175803.5	176986.1	197930.5	207436.4	209070.0	162789.7	196265.8	197679.1	192302.8	192854.3	194566.5	209585.3
E9Q238	ANK_REP_REGION domain-containing protein	Kank1	1360	148.2	5.57	180871.2	182477.5	151443.7	165695.4	168072.0	162156.4	164466.4	154724.1	171244.5	173258.1	79116.9	76305.1	70560.9	69851.5
Q3TNH5	Cotranscriptional regulator FAM172A	Fam172a	417	48	5.97	161338.6	152642.4	158124.0	145273.4	149607.6	157113.6	146690.8	145612.9	155116.0	155349.8	120777.0	153760.9	129161.1	143523.5
Q3TZX8	Polynucleotide 5'-hydroxyl-kinase NOL9	Nol9	714	80.8	9.45	142560.2	147151.2	144801.1	164988.8	139655.1	133796.0	130255.3	139147.2	148774.2	140973.1	375763.2	346632.1	406796.6	318611.7
Q8C779	RPA-related protein RADX	Radx	850	96.8	8.73	301090.1	288472.1	296353.4	289567.1	310344.7	284706.6	264949.1	276523.3	282167.6	298815.6	70809.0	73339.0	75870.5	63323.9
P21460	Cystatin-C	Cst3	140	15.5	9	244137.9	262668.1	227722.5	231102.1	225785.8	230812.5	233401.5	242864.5	253721.9	249903.3	61309.6	59383.5	62411.3	61136.5
Q8BZA9	Fructose-2,6-bisphosphatase TIGAR	Tigar	269	29.2	8.18	188905.2	221707.4	187942.7	238886.4	181901.8	165581.8	176159.5	188474.2	208485.1	187836.8	154689.6	171359.8	137608.8	172146.6
Q8R5M0	PDZ domain-containing protein GIPC3	Gipc3	297	32.1	5.73	128715.4	141440.2	105127.1	133211.8	121801.0	139571.3	122770.4	136384.8	114481.2	118162.6	344387.1	307620.1	327632.0	325325.5
Q6P9J9	Anoctamin-6	Ano6	911	106.2	6.76	197524.1	233187.9	186564.9	236300.0	237740.3	226797.9	235369.1	222606.5	227720.7	268454.5	306716.3	297103.9	305172.6	298752.6
Q9D1C8	Vacuolar protein sorting-associated protein 28 homolog	Vps28	221	25.4	5.54	217599.2	203592.8	253351.3	226332.5	260562.5	201823.8	219900.1	224060.5	230781.6	216580.8	238479.9	241598.7	223593.3	260960.5
Q9CR67	Transmembrane protein 33	Tmem33	247	28	9.66	237047.0	421840.0	324760.0	456421.1	271297.5	255210.3	311555.3	273270.0	402336.0	411008.1	121117.6	132754.2	147362.1	147732.1
Q08509	Epidermal growth factor receptor kinase substrate 8	Eps8	821	91.7	7.65	95407.3	97980.1	91239.2	102251.5	89967.5	93698.4	89899.7	85747.3	98138.2	98340.4	35918.8	35184.6	37623.2	37831.0
P28867	Protein kinase C delta type	Prkcd	674	77.5	7.39	155761.8	159459.4	127362.7	151044.6	154292.8	155767.7	150127.2	143419.1	150877.2	153899.4	86523.8	97356.2	90430.8	90523.0
Q8R4H2	Rho guanine nucleotide exchange factor 12	Arhgef12	1543	172.2	5.74	87616.5	85586.1	83973.9	71490.8	92561.3	100543.2	85395.7	92380.6	79598.2	87083.9	212209.0	209972.5	198269.9	202712.9
Q9D6H2	Intraflagellar transport protein 25 homolog	Hspb11	143	16.3	5.16	69636.8	80107.8	75305.9	79574.3	77180.7	73267.9	74177.7	68020.3	84687.5	78114.9	150375.2	186705.7	155629.9	191254.4
Q8CBC4	Consortin	Cnst	711	76.8	4.59	330845.5	343437.0	316919.1	334713.9	332492.0	335578.4	286418.5	299530.6	327200.6	304075.2	232643.7	257559.5	195785.8	240411.2
Q9Z2D1	Myotubularin-related protein 2	Mtmr2	643	73.2	7.25	135729.1	130715.8	134347.2	128086.9	146545.9	136770.4	133411.9	132210.7	130282.5	134907.4	226447.4	221111.7	198075.2	219984.6
P40936	Indolethylamine N-methyltransferase	Inmt	264	29.4	6.39	74353.2	74067.8	62769.8	65729.1	78674.1	88703.0	229528.9	98228.0	72523.7	68654.7	63128.6	70446.4	62798.6	58377.1
P08207	Protein S100-A10	S100a10	97	11.2	6.77	1190077.6	1312794.0	1429967.6	1364735.8	1744865.8	1948096.5	2715600.1	1690074.4	1751684.8	1426005.4	1229980.0	1341222.2	1310289.7	1383036.5
P28652	Calcium/calmodulin-dependent protein kinase type II subunit beta	Camk2b	542	60.4	7.28	70291.6	67584.1	75266.2	72366.7	90890.8	92111.3	75882.9	74969.5	84062.2	86716.6	#N/A	#N/A	#N/A	#N/A
Q9D832	DnaJ homolog subfamily B member 4	Dnajb4	337	37.8	8.59	104988.9	134891.5	118986.5	109222.9	114188.2	132667.2	140202.9	113311.9	115578.3	110711.0	190689.4	201178.8	173928.5	199207.1
P49182	Heparin cofactor 2	Serpind1	478	54.5	7.34	343266.8	322627.7	276066.1	298255.5	265492.3	313615.3	314001.5	279834.8	322758.1	298467.1	193445.8	194699.2	158753.9	164590.6
A2ASQ1	Agrin	Agrn	1950	207.4	6.32	164525.7	154364.3	159714.6	164286.9	157990.5	169262.7	190037.9	150077.1	165852.2	171430.5	150386.7	159554.1	132554.9	150779.3
A2A6Q5	Cell division cycle protein 27 homolog	Cdc27	825	91.8	7.02	198908.7	199860.1	205666.4	206160.1	205903.5	213561.4	202220.9	208200.0	192067.1	189805.3	72552.3	83843.1	85854.1	83415.8
Q9DCJ5	NADH dehydrogenase [ubiquinone] 1 alpha subcomplex subunit 8	Ndufa8	172	20	8.46	843016.6	792887.4	891806.5	820469.2	896863.9	802475.5	873689.1	917290.2	863621.0	829809.9	278679.6	262641.0	274469.8	271578.3
Q8BJ03	Cytochrome c oxidase assembly protein COX15 homolog	Cox15	413	45.8	9.79	28952.3	34612.1	23533.9	28079.3	33281.3	35974.3	25985.0	31155.0	29761.7	28132.4	78500.4	77665.7	76322.0	79103.3
Q6NS57	Mitogen-activated protein kinase-binding protein 1	Mapkbp1	1503	162.8	6.71	193712.4	206528.2	214590.3	213984.9	197546.6	180022.3	156320.5	191747.0	189642.3	218634.6	66042.9	66820.0	78151.3	62371.1
Q9ERN0	Secretory carrier-associated membrane protein 2	Scamp2	329	36.4	6.32	476452.0	433948.1	480233.6	468568.5	492890.3	484196.2	493923.4	462387.5	435164.0	436359.4	726577.2	629552.4	622736.0	642903.3
Q00724	Retinol-binding protein 4	Rbp4	201	23.2	5.99	456573.8	436720.9	400779.1	395842.3	357881.7	346793.3	411759.6	430035.6	408972.8	387728.1	196579.4	185724.5	196144.5	183875.7
Q62421	Endophilin-A3	Sh3gl3	347	38.9	5.12	175166.9	193840.7	197231.8	239463.3	174650.3	151901.5	165057.7	167609.6	187308.8	186211.7	93513.9	106669.2	96590.4	117124.7
A3KGB4	TBC1 domain family member 8B	Tbc1d8b	1114	127.8	5.49	295822.9	374231.0	314326.8	358266.0	358193.9	346055.5	290030.9	299584.9	332485.4	351066.0	186448.8	210228.0	196790.3	204429.3
Q8BWR4	Ubiquitin carboxyl-terminal hydrolase 40	Usp40	1235	139.9	5.63	182373.8	194472.1	170239.3	178452.7	181185.2	166370.1	152474.8	150026.3	164997.2	176521.4	176701.4	204114.2	168237.5	212871.7
Q9JJX7	Tyrosyl-DNA phosphodiesterase 2	Tdp2	370	41	5.5	75952.3	118788.5	76017.4	79812.3	91031.2	112104.9	80905.2	89407.0	90764.1	84773.0	71866.5	65570.0	78124.8	65358.4
Q9CR60	Vesicle transport protein GOT1B	Golt1b	138	15.4	10.58	232352.6	250451.9	203154.1	246116.8	220642.5	206988.6	210723.7	191770.9	249980.2	227841.5	306265.3	290141.0	272375.3	340285.6
Q9DC33	High mobility group protein 20A	Hmg20a	346	39.9	6.4	45308.1	51548.5	46836.2	48949.2	51185.3	49118.3	49789.5	41932.3	53357.4	51555.4	271437.0	289968.0	247889.2	274649.3
Q8BLH5	Probable threonine protease PRSS50	Prss50	439	48.6	9.66	699775.7	854440.5	746169.2	787127.6	716947.7	786912.9	584990.6	580770.6	778984.8	778817.2	1124647.6	1213050.6	1407282.9	1289902.8
P47955	60S acidic ribosomal protein P1	Rplp1	114	11.5	4.32	182582.6	183321.7	195122.3	201481.7	176321.7	181877.5	161233.4	173216.5	181934.6	196690.6	149731.8	129364.4	147234.8	148870.2
P03888	NADH-ubiquinone oxidoreductase chain 1	Mtnd1	318	36	6.93	188539.8	186908.0	163633.4	181202.0	176014.4	174681.1	175170.3	182285.8	172606.1	170151.4	168515.7	155264.4	155107.4	169825.8
Q9JHP7	Protein O-glucosyltransferase 2	Poglut2	502	57.9	7.9	163318.1	145008.3	141057.9	135617.9	133808.0	143360.1	144623.9	146961.2	141517.8	139301.7	122683.4	106027.7	107304.3	103709.4
Q8VE80	THO complex subunit 3	Thoc3	351	38.7	6.09	637581.8	607482.5	619987.5	595979.9	628751.4	631691.2	639587.0	556886.2	624593.6	679616.9	516908.2	554615.3	531128.5	543466.3
Q3UVG3	Protein FAM91A1	Fam91a1	837	93.4	6.35	331539.0	357982.7	338141.4	334064.0	370939.4	336591.3	338705.0	305847.5	338954.2	356490.1	177619.5	175707.8	160917.8	172589.5
Q9JI44	DNA methyltransferase 1-associated protein 1	Dmap1	468	53.1	9.5	111018.5	130710.4	118787.1	120479.5	118333.5	118359.1	112554.2	112398.0	117076.2	131102.2	152067.3	145931.9	133770.2	151693.6
Q99LL5	Periodic tryptophan protein 1 homolog	Pwp1	501	55.6	4.82	48981.3	48968.6	47656.1	49309.9	48029.7	58027.2	50873.2	43694.5	48839.3	49869.5	169014.6	170362.4	169233.3	169953.5
Q80SY5	Pre-mRNA-splicing factor 38B	Prpf38b	542	63.7	10.54	312733.4	309760.2	262925.1	308243.4	263507.9	273100.3	285470.5	258294.7	306797.9	318010.3	328135.2	319250.8	326973.0	340811.4
Q6PEB6	MOB-like protein phocein	Mob4	225	26	5.78	93182.7	67547.9	69927.8	59504.2	65429.2	59474.7	54687.1	62604.9	63419.6	61117.7	32345.1	36315.4	33580.2	34649.9
Q9Z2A5	Arginyl-tRNA--protein transferase 1	Ate1	516	59.1	8.19	150742.8	172407.0	148597.7	173738.7	148243.4	148800.0	154710.0	137654.7	154440.4	170567.7	98378.8	91893.4	113523.6	110885.2
P21956	Lactadherin	Mfge8	463	51.2	6.52	127408.8	127492.7	124006.8	117551.9	122767.5	128789.3	122004.5	125849.0	115482.9	113450.3	133119.5	123100.1	112776.8	131112.5

P60867	40S ribosomal protein S20	Rps20	119	13.4	9.94	742024.1	654657.1	775990.5	651172.6	794540.9	722978.6	588878.0	869659.3	614801.4	612657.4	891266.4	839060.9	888240.8	866077.7
Q8BTV1	Tumor suppressor candidate 3	Tusc3	347	39.5	10.11	212529.0	310953.5	259711.4	309185.7	282578.4	244574.0	205694.2	228405.3	279440.1	306878.6	409793.8	434665.7	426731.7	464601.6
Q9D1R9	60S ribosomal protein L34	Rpl34	117	13.3	11.47	1162375.8	1303675.0	1464573.2	1312585.5	1249374.1	1143164.9	1019868.1	1385679.8	1241223.0	1208509.4	1108009.8	1256598.0	1179755.4	1228490.5
Q99N57	RAF proto-oncogene serine/threonine-protein kinase	Raf1	648	72.9	9.16	46106.7	59741.5	55880.6	56217.2	55596.9	58645.7	46171.1	54651.5	54524.2	57065.5	52472.7	53207.4	54765.4	46096.1
Q9D0P8	Intraflagellar transport protein 27 homolog	Ift27	186	20.8	5.2	93568.6	114082.9	100210.6	138268.1	87889.0	76134.7	85756.1	74703.7	98315.9	105428.3	99776.9	114031.8	111976.7	123848.6
Q8K2L8	Trafficking protein particle complex subunit 12	Trappc12	797	87.6	4.81	522356.3	542484.5	502522.7	530593.9	505065.3	513490.8	487195.8	510628.9	513961.5	496335.8	218531.9	290958.2	226897.7	238929.8
Q5SU73	Coilin	Coil	570	61.9	9	153229.7	157317.3	159563.2	157866.6	174113.9	147798.5	145537.7	136979.6	154253.0	152897.6	105262.2	120093.4	117598.2	101357.7
Q3UR70	Transforming growth factor-beta receptor-associated protein 1	Tgfbrap1	860	97.2	6.7	108428.9	111340.7	96505.2	103151.6	99367.2	95716.4	96774.1	107137.6	99479.2	103155.0	233335.8	257210.5	227108.6	248258.2
P18052	Receptor-type tyrosine-protein phosphatase alpha	Ptpra	829	93.6	7.05	114186.3	115910.1	131359.2	126291.7	128692.2	126800.4	127403.0	118714.4	110489.0	124760.4	75587.2	85649.3	79619.5	83497.6
Q91XU3	Phosphatidylinositol 5-phosphate 4-kinase type-2 gamma	Pip4k2c	421	47.3	6.89	411440.7	467041.4	396281.0	406954.8	433520.5	423719.0	387181.5	415783.7	400349.5	428371.5	699738.6	703613.3	700597.1	747178.3
Q6P2L7	Protein CASC4	Casc4	435	49.4	5.45	198976.8	203765.7	206845.8	202721.2	207286.7	214383.0	204230.0	210602.4	195039.2	198651.1	95716.2	99374.4	93986.9	98570.9
Q9DC63	F-box only protein 3	Fbxo3	480	55.2	5.02	183334.8	236892.7	176604.4	209999.4	202288.3	191255.8	166152.3	178887.0	199221.3	213835.2	203429.3	214566.4	208326.0	227456.9
Q8K1Z0	Ubiquinone biosynthesis protein COQ9, mitochondrial	Coq9	313	35.1	5.92	287912.0	356308.0	328744.6	322083.0	320274.6	321267.7	288046.0	327857.7	329648.1	321761.5	144290.1	146338.2	151124.8	164404.8
Q8BWU5	Probable tRNA N6-adenosine threonylcarbamoyltransferase	Osgep	335	36.3	6.24	173996.2	197915.3	197770.1	195164.2	199029.2	207120.9	193159.7	195310.2	195571.1	196868.0	169524.1	191521.6	189313.5	198038.8
Q65CL1	Catenin alpha-3	Ctnna3	895	99.7	6.52	41469.2	51680.1	43335.8	44617.4	43934.9	43834.4	41595.5	40417.4	39051.9	42482.6	17241.9	16796.9	18229.4	16746.1
O89086	RNA-binding protein 3	Rbm3	153	16.6	7.5	103625.5	95640.8	139667.1	95438.5	143664.5	127596.4	116790.1	130450.5	107257.6	113406.9	37777.6	29656.2	33568.1	28676.3
P62855	40S ribosomal protein S26	Rps26	115	13	11	1756859.6	1912512.5	2090043.4	1887024.8	1762863.4	1605273.5	1507876.0	1826974.3	1663805.8	1680351.7	2305640.1	2643667.8	2804034.9	2587860.5
Q922X9	Protein arginine N-methyltransferase 7	Prmt7	692	78.3	5.74	165880.0	194567.3	191897.4	187468.2	193513.6	185195.3	154775.3	151129.9	181405.5	196678.9	209633.6	241885.3	253056.4	248034.4
Q922E4	Ethanolamine-phosphate cytidylyltransferase	Pcyt2	404	45.2	6.58	119256.6	106984.3	107949.5	123551.5	92694.4	82038.7	102383.3	100806.5	111212.4	98163.1	91570.9	106888.3	107243.5	116424.8
Q91W18	Tudor domain-containing protein 3	Tdrd3	743	82.2	9.11	74493.9	107251.5	72601.9	86842.5	85995.0	92105.0	68659.1	88087.2	83879.7	89789.1	39553.9	44533.8	43197.5	46677.1
Q9JI75	Ribosyldihydronicotinamide dehydrogenase [quinone]	Nqo2	231	26.2	7.01	264067.0	238699.2	249361.5	241691.6	265169.9	274185.6	309918.3	262225.9	244006.1	252600.6	298219.3	264888.3	269637.7	253551.6
Q9DCT6	Chromatin complexes subunit BAP18	Bap18	171	18	7.33	90279.0	125907.8	129719.4	107386.4	131381.0	126004.0	111596.0	129349.8	131749.2	124851.1	143888.5	159253.0	151233.1	141529.0
O89112	Glutathione S-transferase LANCL1	Lancl1	399	45.3	7.77	322030.0	366012.3	341965.2	403747.2	319147.8	311841.5	291080.9	283226.2	353242.2	359865.8	164153.8	183359.9	174342.5	174226.4
Q8BGQ1	Spermatogenesis-defective protein 39 homolog	Vipas39	491	56.6	7.17	319746.1	355304.1	360695.4	384775.7	320261.0	347822.4	303207.7	310865.5	367467.0	370966.9	197804.9	211706.2	218723.7	208408.5
E9Q512	GRIP domain-containing protein	Trip11	1976	226.3	5.26	197718.3	191208.8	178112.3	190336.6	201294.9	188325.4	180404.7	184134.1	188094.6	179124.0	150225.7	147789.2	143761.4	147661.7
Q9CY57	Chromatin target of PRMT1 protein	Chtop	249	26.6	12.23	384108.3	414503.6	450950.9	442529.9	415098.9	392346.1	399352.1	415401.7	448515.6	419242.8	504843.1	597257.8	569389.5	604849.7
Q9QUH0	Glutaredoxin-1	Glrx	107	11.9	8.37	300603.4	300162.8	252025.3	293356.9	285359.9	309186.9	328172.8	308333.1	269309.1	263448.8	484603.6	476892.4	521805.7	526721.7
Q9DAC5	Cor1 domain-containing protein	00013H16l	291	33.6	4.65	97820.0	121036.7	119880.8	105694.2	143406.3	126555.9	101053.4	94772.8	118457.1	114190.9	109287.8	138917.9	139529.2	121598.9
P70166	Cytoplasmic polyadenylation element-binding protein 1	Cpeb1	561	61.9	7.72	92596.6	108120.5	90826.2	91681.9	101653.9	99124.5	86613.0	81819.0	97336.1	99647.7	10751.9	10571.7	8791.1	8894.6
Q6R891	Neurabin-2	Ppp1r9b	817	89.5	4.92	124287.4	122621.4	113875.4	130006.7	115502.7	110417.8	121876.8	122473.3	116154.9	119947.4	279538.8	271428.2	237613.4	224531.6
O08967	Cytohesin-3	Cyth3	399	46.3	5.54	206952.2	174512.5	147592.8	159468.4	173482.8	161978.8	175813.7	129177.5	154757.4	209513.2	#N/A	#N/A	#N/A	#N/A
O55033	Cytoplasmic protein NCK2	Nck2	380	42.9	6.95	185163.2	190282.6	166926.0	175630.4	166231.4	159539.7	172278.1	151860.9	181485.4	165678.5	5287.8	6242.8	3669.2	4534.6
Q9CR39	WD repeat domain phosphoinositide-interacting protein 3	Wdr45b	344	38	7.56	184869.6	189853.3	167010.2	179822.4	207085.7	201304.2	184175.8	186742.9	174386.4	182511.6	201954.2	193238.7	198744.2	185178.6
Q91V57	N-chimaerin	Chn1	459	53.2	6.76	135650.6	149505.0	124403.8	128676.2	140386.4	131979.3	123498.4	123805.1	139655.0	149522.7	110856.7	111568.1	109222.4	111607.3
Q9R020	Zinc finger Ran-binding domain-containing protein 2	Zranb2	330	37.3	9.89	542499.8	623392.6	627390.5	593159.4	581527.4	602121.4	543335.7	579911.1	576528.1	601533.5	337551.2	364273.6	372468.1	355936.2
Q7TPS0	Ribosomal protein S6 kinase alpha-6	Rps6ka6	764	86.5	6.68	241310.0	262498.6	212524.1	213835.8	265949.1	286209.3	210980.9	210772.5	218140.7	215348.6	78742.8	97982.9	101919.2	101317.1
Q60695	Ral guanine nucleotide dissociation stimulator-like 1	Rgl1	768	86.3	6.09	108623.4	113770.4	102709.5	100429.9	111909.7	108262.6	110867.1	103326.9	104888.4	104052.6	74515.6	66537.2	74759.1	63222.8
O55057	Retinal rod rhodopsin-sensitive cGMP 3',5'-cyclic phosphodiesterase subunit delta	Pde6d	150	17.3	5.67	150766.4	164844.0	172665.3	173736.7	159935.0	154755.6	153658.7	141963.9	165620.8	165091.6	586213.8	673206.7	600884.6	699877.6
Q80VP1	Epsin-1	Epn1	575	60.2	4.81	64069.2	76458.4	62473.9	77582.7	77901.2	70503.8	72986.4	74554.8	77471.8	86841.8	80801.4	83297.5	73641.9	83804.1
P50427	Steryl-sulfatase	Sts	624	66.5	8.5	307886.3	365853.4	236204.7	306943.0	314472.5	256815.2	289832.2	297693.5	310761.8	329815.2	329144.1	321491.3	250355.1	314461.8
Q9DC16	Endoplasmic reticulum-Golgi intermediate compartment protein 1	Ergic1	290	32.5	7.06	29481.4	48970.5	38314.8	39060.7	36964.7	40054.8	30590.2	42170.7	36042.6	39067.3	441696.6	415564.2	378385.2	401393.5
Q9D8V0	Minor histocompatibility antigen H13	Hm13	378	41.7	6.04	558909.5	562765.7	472803.1	519153.6	488486.4	450131.4	496846.4	495556.0	497736.4	532624.8	467263.4	462288.5	418992.4	439261.1
Q91V01	Lysophospholipid acyltransferase 5	Lpcat3	487	56.1	8.56	291598.1	379407.5	321476.3	390683.2	324162.9	304376.7	272044.1	281863.3	331339.1	367961.4	305617.9	367228.1	336971.7	382205.1
Q8BU04	Putative E3 ubiquitin-protein ligase UBR7	Ubr7	425	48	4.75	382436.3	444736.4	428232.2	426468.9	458336.9	419418.3	375348.4	358677.2	428790.2	483147.0	168544.5	178838.7	171091.2	179170.5
Q9DAZ9	Abscission/NoCut checkpoint regulator	Zfyve19	389	43.2	6.54	49926.6	52134.5	49418.0	52119.5	50457.9	58348.1	48289.1	48961.9	50327.4	47207.3	151445.3	171087.0	150992.4	170212.3
Q3U308	Cytoplasmic tRNA 2-thiolation protein 2	Ctu2	514	56.1	7.08	69283.7	74251.6	78411.3	73678.3	79414.3	76036.4	79903.4	79077.4	79713.1	78192.1	90163.9	108254.3	99238.1	105961.6
Q8BMI0	F-box only protein 38	Fbxo38	1194	133.8	6.09	78670.4	84949.0	74520.0	80673.7	80608.2	83121.6	79399.6	82064.3	74917.2	88288.9	82950.7	84046.3	82309.8	82208.6
E9Q4P1	WD repeat and FYVE domain-containing protein 1	Wdfy1	410	46.2	7.34	189716.4	187141.5	185961.3	179751.3	203788.7	199806.2	172791.4	183332.8	184973.2	177399.0	197406.7	256754.7	249723.9	264156.3
Q7TSG2	RNA polymerase II subunit A C-terminal domain phosphatase	Ctdp1	960	104.5	5.39	94079.0	106963.1	89980.6	103196.8	107707.5	116225.2	136671.9	117129.9	114371.6	116139.6	88673.9	87614.0	76692.7	82022.2
Q5I012	Putative sodium-coupled neutral amino acid transporter 10	Slc38a10	1090	117.1	5.96	251914.5	236734.2	230810.7	228270.7	273118.7	277551.2	246680.2	254150.0	256520.7	246655.9	331008.2	359362.3	349779.9	339564.0
Q9ERD6	Ras-specific guanine nucleotide-releasing factor RalGPS2	Ralgps2	590	65.5	8.73	171425.3	184387.5	197909.7	181936.8	159885.3	169276.6	134756.9	161283.7	178185.9	178309.9	138021.1	152957.4	138087.5	146447.8
Q9D8T7	SRA stem-loop-interacting RNA-binding protein, mitochondrial	Slirp	112	12.6	9.82	330748.3	322011.7	314939.4	315100.1	334196.5	315372.2	282184.3	307695.4	328759.4	310976.6	311931.4	384706.3	338459.9	369982.9
P49442	Inositol polyphosphate 1-phosphatase	Inpp1	396	43.3	5.01	122643.8	113537.8	103029.0	118656.3	118059.7	138167.3	139417.0	117319.0	122625.4	115865.9	76605.7	84635.7	80234.3	77546.3
Q3UQA7	Selenoprotein H	Selenoh	116	13	9.8	338639.9	319023.8	314112.8	281649.9	353821.1	363481.5	363676.2	323501.7	360416.5	355335.1	468907.1	467242.5	468406.1	396861.0
E0CZ26	Potassium channel tetramerisation domain-containing 18	Kctd18	429	46.9	8.35	100730.8	119138.3	115285.0	109633.0	115582.4	115310.8	114939.0	95279.4	104753.8	132276.4	123630.3	234018.0	139424.4	180024.6
P97390	Vacuolar protein sorting-associated protein 45	Vps45	570	65	8.25	284505.3	279763.1	222981.4	255101.3	263721.1	269495.5	269415.2	229692.1	269908.1	264376.8	147131.9	158299.5	145427.7	152871.0
Q9Z2R6	Protein unc-119 homolog A	Unc119	240	27	6.15	327962.6	377397.7	350463.1	334814.5	364765.2	351300.5	318114.3	325764.9	345569.4	354841.2	107840.6	107612.4	118100.8	111274.2
E9Q0F0	IF rod domain-containing protein	Krt78	1068	112.2	7.97	776398.9	453076.2	498621.6	339523.9	545837.1	500520.8	519714.4	378096.3	582186.8	377243.5	536886.3	410417.7	412958.4	317607.4
Q80V94	AP-4 complex subunit epsilon-1	Ap4e1	1122	124.8	5.72	49751.9	56061.5	49611.5	52800.1	56777.7	54186.5	52807.1	50971.4	50155.5	49540.6	68474.5	67050.0	65968.6	72241.1
P59108	Copine-2	Cpne2	548	61	5.96	190394.9	198625.1	188149.2	193636.5	200496.7	222972.7	206062.6	205140.4	194932.7	201236.2	250051.9	255470.9	235774.8	232296.7
Q8R344	Coiled-coil domain-containing protein 12	Ccdc12	166	18.9	7.21	104246.6	115650.0	136015.0	124922.5	124137.3	123050.1	113019.4	108437.0	113506.9	132806.0	254438.8	302179.4	263626.4	268480.1
P53762	Aryl hydrocarbon receptor nuclear translocator	Arnt	791	86.9	6.64	55209.6	59907.2	56051.5	47754.2	63466.6	62982.5	61205.9	60656.9	60545.8	63106.1	#N/A	#N/A	#N/A	#N/A
Q9D7M1	Glucose-induced degradation protein 8 homolog	Gid8	228	26.8	4.97	89550.7	121644.2	101784.9	111538.4	119156.4	97534.4	71219.0	106323.7	111382.0	108060.5	33321.6	34742.9	36097.6	42987.2
Q8C3S2	Transport and Golgi organization protein 6 homolog	Tango6	1079	118.7	6.32	227658.4	207301.9	201047.3	198728.5	202586.6	199478.5	215441.8	200369.1	198054.7	220542.9	142155.2	129453.6	131126.7	137476.5
Q9QXS6	Drebrin	Dbn1	706	77.2	4.49	53810.1	60062.6	55100.2	51173.7	51990.6	55345.6	47589.2	53792.2	57224.8	57354.7	95997.4	98857.1	85727.5	92682.8
A6H611	Mitochondrial intermediate peptidase	Mipep	711	80.8	7.15	128445.4	140117.8	133930.6	128428.3	144043.6	136190.5	140878.8	124395.9	131095.0	144891.1	124813.8	140671.2	127515.2	137208.2
Q5DU37	Zinc finger FYVE domain-containing protein 26	Zfyve26	2529	282.8	6.21	94702.5	96744.2	95727.2	89488.9	100784.6	95058.8	86213.3	102391.4	93648.0	101690.2	121278.9	118905.7	123548.6	116655.2
P52332	Tyrosine-protein kinase JAK1	Jak1	1153	133.3	7.59	194564.0	196417.0	173253.2	187192.6	196192.5	206783.0	227081.7	184671.4	180667.9	188295.6	119220.3	127683.6	124201.7	128733.4
Q07456	Protein AMBP	Ambp	349	39	6.32	482583.2	496907.2	399538.0	512570.7	420933.2	452907.2	500844.5	473743.9	390148.4	516946.2	80085.9	82690.4	72837.7	71775.4
P61957	Small ubiquitin-related modifier 2	Sumo2	95	10.9	5.5	1346818.2	1049643.3	2219193.7	1215545.9	1379728.2	1185366.7	1002353.7	1735368.7	1417388.1	1210924.5	1935624.9	1675386.3	3024989.0	2089068.5
Q8BM39	Pre-mRNA-splicing factor 18	Prpf18	342	39.9	8.15	170460.2	180173.6	163492.5	176641.4	182498.2	162081.4	152667.4	161006.1	176837.4	178540.7	#N/A	#N/A	#N/A	#N/A
D3YX43	V-set and immunoglobulin domain-containing protein 10	Vsig10	558	60.5	4.7	168752.1	161502.1	182359.8	148154.8	185250.6	191814.0	209277.5	173494.0	161577.3	171488.8	210127.2	169972.6	172203.4	165306.0
Q80TE4	Signal-induced proliferation-associated 1-like protein 2	Sipa1l2	1722	189.3	6.79	76367.5	77515.2	68523.9	73874.8	74478.3	75144.6	67187.9	70004.8	78012.2	68930.1	54910.5	65073.0	58520.5	59649.3
O35114	Lysosome membrane protein 2	Scarb2	478	54	5.1	574440.7	550527.3	537381.5	558536.3	601238.7	589371.7	756099.0	587801.7	568352.4	555975.8	519125.9	532207.6	477187.4	544368.4
Q9QVN7	Transcription elongation factor A protein 2	Tcea2	299	33.6	9.13	190555.9	244239.9	227773.5	258658.2	152796.6	151972.0	127756.5	188279.0	170429.5	200540.7	128311.2	154851.2	134860.6	166179.2
P48377	MHC class II regulatory factor RFX1	Rfx1	963	103.6	6.34	61690.7	65910.9	63935.9	60504.4	73649.0	77754.3	71982.2	68091.4	73676.3	65008.9	75977.9	83783.3	82502.8	87535.7
P07310	Creatine kinase M-type	Ckm	381	43	7.06	209046.4	173065.2	146052.5	151167.5	167147.3	212039.2	220912.6	519691.0	159173.7	155970.0	425222.3	356962.3	303807.6	335433.7
B2RXQ2	Ppfia1 protein	Ppfia1	1266	142.6	6.25	141538.0	138469.6	151554.7	149328.8	159893.6	144138.6	152664.8	150769.9	128623.9	142133.0	335867.8	343034.0	330471.6	343044.9
S4R1M9	Oxysterol-binding protein-related protein 10	Osbpl10	766	83.8	8.28	79955.2	81584.2	72962.9	61116.9	77791.3	76740.0	72233.4	65907.6	84569.4	79446.3	51580.5	61307.9	50801.3	58254.0
Q9CQJ6	Density-regulated protein	Denr	198	22.2	5.3	170510.4	157785.3	177575.4	153913.4	174080.6	172066.8	135380.8	182088.1	148792.4	157417.2	191600.9	145897.3	197860.1	168209.6
Q6PD03	Serine/threonine-protein phosphatase 2A 56 kDa regulatory subunit alpha isoform	Ppp2r5a	486	56.3	7.03	99241.1	105376.1	94608.3	106087.0	115805.2	120414.6	129935.7	115888.7	98419.2	103522.1	218543.0	231811.9	216428.0	214386.4
Q8VDS8	Syntaxin-18	Stx18	334	38.4	5.77	213499.8	243691.1	212417.2	240021.4	203804.0	208536.0	183653.2	222877.6	218744.0	208282.9	133435.4	151674.4	141717.4	151775.1
Q9ES74	Serine/threonine-protein kinase Nek7	Nek7	302	34.5	8.25	112108.4	116212.8	99053.8	98314.2	110701.7	106258.8	103182.4	101859.6	98751.5	104364.0	341702.3	307326.4	310993.2	313123.7
Q80U19	Disheveled-associated activator of morphogenesis 2	Daam2	1115	128.3	6.92	266439.8	252250.9	218826.1	257537.0	215675.2	202608.7	231137.5	217806.7	237933.1	251564.7	206702.1	239765.9	200448.5	242810.2
Q8BM55	Transmembrane protein 214	Tmem214	687	76.4	9.29	212392.4	210834.9	201228.5	210389.1	222328.8	191933.3	193647.4	211985.5	196530.1	193616.4	86772.2	87370.8	96622.4	93274.0
Q9CZV8	F-box/LRR-repeat protein 20	Fbxl20	436	48.4	7.49	81983.4	100266.3	86693.3	89518.3	92394.4	93090.3	78715.1	80204.7	87925.5	91212.6	35150.6	38147.8	36944.7	35223.1
Q8BHF7	CDP-diacylglycerol--glycerol-3-phosphate 3-phosphatidyltransferase, mitochondrial	Pgs1	553	62.5	8.9	106065.4	113887.6	115913.4	116200.6	120546.0	118835.6	117545.1	111284.1	109077.2	115484.5	122401.5	168748.5	118547.6	155079.0
Q99LN9	Deoxyhypusine hydroxylase	Dohh	302	32.9	4.84	105772.3	114414.9	92355.1	100393.4	101712.9	98311.0	94335.6	92580.4	88772.2	104716.6	74319.4	81202.3	82721.9	85344.0
Q9ET54	Palladin	Palld	1408	152	6.25	80671.5	103378.8	91329.0	77593.3	91059.9	94807.1	87209.4	96380.7	92561.2	88971.7	153828.1	134845.8	125499.8	123606.7
Q8BWY9	Protein CIP2A	Cip2a	907	102	6.28	247132.8	287273.9	276078.9	269391.2	308815.1	344651.5	266209.2	279406.0	259672.3	266066.4	124295.7	121821.9	129284.0	114391.9
Q8BGS0	Protein MAK16 homolog	Mak16	296	35.2	5.31	58202.5	63190.7	52316.1	60874.4	56845.0	62235.7	61521.0	48445.3	60290.2	57470.4	115102.2	126436.4	120129.5	119178.4
P45481	Histone lysine acetyltransferase CREBBP	Crebbp	2441	265.3	8.54	76412.3	81412.1	74115.5	76315.5	93441.0	89967.7	85580.7	82383.2	80052.3	76983.3	309353.3	21943.1	18014.8	35240.8

Q8C167	Prolyl endopeptidase-like	Prepl	725	83.1	6.65	276265.2	327695.5	292890.1	337784.3	253795.2	245350.1	252341.3	255770.4	314202.7	332172.1	178987.9	203196.2	170199.0	180581.5
Q3UHC7	Disabled homolog 2-interacting protein	Dab2ip	1189	131.6	8.72	245918.6	231908.8	251250.8	235315.1	306851.1	308164.8	298724.9	251973.7	252019.1	253685.3	194340.4	192174.2	210936.0	186895.0
Q80WE4	Kinesin-like protein KIF20B	Kif20b	1774	203.4	5.78	71385.2	84534.3	67702.3	68573.4	80912.3	69671.5	64441.8	65583.9	73639.4	82779.0	62865.9	60013.0	62085.4	57860.0
O55236	mRNA-capping enzyme	Rngtt	597	68.6	8.16	42679.5	60813.5	47898.6	51803.1	51091.8	51558.9	48789.1	49840.1	53834.4	58898.3	249196.0	261703.8	246177.6	287030.8
Q0VEE6	Zinc finger protein 800	Znf800	662	74.8	9.42	174835.1	193729.1	175445.4	202550.3	173945.3	179919.7	154709.1	165788.5	193939.3	181138.4	315844.0	383015.0	395958.5	359654.7
O54825	Bystin	Bysl	436	49.8	7.78	28293.9	28848.0	24674.3	26491.1	28723.4	26848.6	26117.3	27922.4	29291.6	26457.4	137097.5	153515.4	147118.4	153591.0
Q9CPW4	Actin-related protein 2/3 complex subunit 5	Arpc5	151	16.3	5.67	461884.9	465697.5	432068.0	437697.4	454340.1	421826.0	423507.8	450173.9	459247.8	440578.7	497484.9	453828.5	521049.9	487027.7
P35276	Ras-related protein Rab-3D	Rab3d	219	24.4	4.93	199117.0	198718.7	203288.2	211833.7	179980.6	187568.3	181713.2	193563.5	178467.0	189103.2	197486.4	189404.5	155871.7	160414.1
Q6ZWM4	U6 snRNA-associated Sm-like protein LSm8	Lsm8	96	10.4	4.48	203172.4	215028.8	195244.8	220936.0	225605.0	237894.5	201901.9	186475.4	213719.0	193277.0	606418.5	612048.3	624230.5	619252.7
Q9CQ75	NADH dehydrogenase [ubiquinone] 1 alpha subcomplex subunit 2	Ndufa2	99	10.9	9.99	570952.8	538412.2	587059.7	579454.0	580773.9	553739.5	553661.5	622041.6	579114.2	537880.9	397441.9	402081.1	383597.7	376764.1
O35490	Betaine--homocysteine S-methyltransferase 1	Bhmt	407	45	7.9	115002.0	124288.4	95331.3	141571.4	90759.3	105408.6	101502.2	95954.7	98468.3	104890.2	163128.1	180842.2	156718.8	207008.0
Q923G2	DNA-directed RNA polymerases I, II, and III subunit RPABC3	Polr2h	150	17.1	4.68	119170.4	128257.4	134518.2	126019.7	142466.9	143076.9	131441.1	135761.7	136075.1	134199.6	101474.3	107259.0	130201.4	107311.8
P70255	Nuclear factor 1 C-type	Nfic	439	48.7	8.32	194765.8	197039.2	206875.0	165769.1	242054.6	278446.5	273458.7	213203.0	196621.7	207236.3	278769.1	301400.4	286485.2	262759.3
Q9CYI0	Protein Njmu-R1	MGI:191784	393	44.4	5.05	102100.1	111921.7	106334.1	113474.8	110666.1	125647.6	101201.2	98438.3	110746.4	109955.0	103403.4	103447.0	108926.7	120454.0
Q9D164	FXYD domain-containing ion transport regulator 6	Fxyd6	94	10.4	5.11	40718.4	36291.2	36274.7	37042.8	39829.0	41312.2	39859.3	36717.3	38199.1	39773.2	19986.7	20352.8	17114.0	15823.6
Q80YV2	Nuclear-interacting partner of ALK	Zc3hc1	501	55.2	5.38	46901.5	50829.6	52096.4	52086.9	59146.6	53192.1	52436.5	47624.5	50105.4	52232.9	68360.1	69019.2	70917.7	75412.3
Q6TEK5	Vitamin K epoxide reductase complex subunit 1-like protein 1	Vkorc1l1	176	19.8	8.95	569194.4	650936.1	613960.9	624332.1	586215.5	542680.6	564769.2	563941.1	606049.5	598811.7	304367.8	350294.1	329933.3	346550.7
Q08857	Platelet glycoprotein 4	Cd36	472	52.7	8.35	185843.9	188332.6	138818.6	166818.1	439748.5	162079.2	190606.9	173548.6	154969.5	179177.9	134899.8	130250.0	113266.4	121187.2
Q8CHQ0	F-box only protein 4	Fbxo4	385	43.7	6.11	174380.7	177226.5	160413.8	159961.4	164742.2	177709.1	147054.6	185598.2	163528.7	170743.0	111459.8	162506.0	88476.2	117369.9
Q3TGF2	Protein FAM107B	Fam107b	131	15.6	8.31	138153.2	152735.6	178998.1	189380.5	128269.6	120780.9	122350.0	128908.1	147058.4	166698.9	108792.7	168340.3	200091.6	208882.3
Q9CXK8	60S ribosome subunit biogenesis protein NIP7 homolog	Nip7	180	20.4	8.24	89278.2	93723.5	80485.0	93525.4	95663.3	89746.9	90342.7	82195.3	89804.6	95591.5	106277.2	120442.2	129026.4	106557.4
Q08024	Core-binding factor subunit beta	Cbfb	187	22	5.77	48877.9	50823.5	50898.1	55274.8	57804.2	59716.2	63961.9	48051.1	67635.5	53591.7	414643.3	406765.1	413219.4	403614.0
E9PUA5	Kinesin-like protein	Kifc5b	672	73.6	8.81	188255.9	199731.6	209198.6	204935.5	214206.4	209443.0	185128.7	177706.9	206525.8	210463.5	#N/A	#N/A	#N/A	#N/A
Q8BJ37	Tyrosyl-DNA phosphodiesterase 1	Tdp1	609	68.6	7.81	103586.6	135212.6	114662.6	120524.4	119634.6	105774.1	132885.8	97528.7	127083.8	139279.5	65675.8	64390.6	71924.4	71260.4
Q9WVS8	Mitogen-activated protein kinase 7	Mapk7	806	87.7	5.76	85220.0	85146.8	87488.1	77329.2	92445.9	81050.6	75220.7	87837.6	85361.0	86227.4	113315.4	127439.4	109521.4	120162.3
Q8BHX3	Borealin	Cdca8	289	32.2	10.13	54537.3	63329.5	61865.8	62421.0	60966.4	60457.4	53757.1	49324.4	61434.9	57006.3	116973.3	129764.5	127906.8	120399.5
Q922W5	Pyrroline-5-carboxylate reductase 1, mitochondrial	Pycr1	309	32.4	6.86	108740.8	107672.8	100031.3	112274.1	108024.3	113390.9	100056.2	105635.2	117332.2	111210.2	#N/A	#N/A	#N/A	#N/A
B8ZXI1	Queuine tRNA-ribosyltransferase accessory subunit 2	Qtrt2	415	46.3	6.16	45607.5	44490.2	42625.9	40360.2	49738.2	49036.5	47501.9	38339.0	45271.8	50699.1	131549.6	138631.0	139657.2	127884.8
Q9CWU0	Putative ATP-dependent RNA helicase TDRD12	Tdrd12	1215	137.5	6.54	85190.9	100283.9	88085.2	101015.0	89097.2	80572.0	77501.5	74291.2	99628.9	112370.1	129826.4	127343.1	126910.9	130964.6
Q6PGF3	Mediator of RNA polymerase II transcription subunit 16	Med16	828	91.7	7.75	116971.8	136314.0	116376.7	149781.2	154442.6	155606.1	126433.8	115551.6	132690.2	140136.1	80784.3	83971.0	77038.6	73961.1
Q8CBC7	Putative tRNA (cytidine(32)/guanosine(34)-2'-O)-methyltransferase	Ftsj1	324	35.6	5.71	90100.9	95320.0	90951.1	96187.1	86875.3	84695.0	84735.1	92886.9	89942.5	89830.0	186186.3	182313.8	149162.2	167118.3
O88597	Beclin-1	Becn1	448	51.6	4.93	88719.3	115720.0	103911.8	101853.0	97546.8	104126.6	81099.2	92694.4	101173.9	98217.9	108847.3	121473.0	108803.1	121318.7
Q9D1M4	Eukaryotic translation elongation factor 1 epsilon-1	Eef1e1	174	19.8	8.59	278170.1	326031.8	330542.3	332653.4	353061.2	300029.8	337334.2	284594.3	365136.8	336384.9	753966.2	871841.7	855951.3	849206.6
P19536	Cytochrome c oxidase subunit 5B, mitochondrial	Cox5b	128	13.8	8.38	216837.0	179762.4	208857.2	209063.1	199983.1	180477.3	173310.6	208128.7	191312.7	184327.4	69523.8	69205.1	78693.4	74267.7
Q61334	B-cell receptor-associated protein 29	Bcap29	240	27.9	9.72	324744.1	364703.8	435412.4	420316.3	348381.1	296728.2	286958.8	293350.1	341082.0	358174.2	439243.6	530241.0	503211.9	552491.7
Q80VA0	N-acetylgalactosaminyltransferase 7	Galnt7	657	75.4	7.93	135074.0	159256.5	119261.6	133326.7	137918.7	133754.1	120065.6	124766.1	126243.3	140936.4	104100.9	97717.5	94603.8	103239.3
Q64471	Glutathione S-transferase theta-1	Gstt1	240	27.4	7.27	552374.9	501336.8	536588.3	539624.3	603237.0	581784.4	795386.6	520687.9	556421.9	558334.4	228876.8	204309.5	229041.9	207201.3
P09602	Non-histone chromosomal protein HMG-17	Hmgn2	90	9.4	9.99	72423.9	62718.4	76523.3	60193.7	106138.4	109195.3	93545.0	72712.7	78995.1	76747.7	189263.8	153474.2	213526.9	145487.5
Q99NF8	Ran-binding protein 17	Ranbp17	1088	124	6.38	258302.4	288440.0	277841.2	301934.5	265097.5	259648.8	246397.0	233268.0	274288.4	275244.9	190586.3	194239.4	172298.4	200849.0
Q8BKE9	Intraflagellar transport protein 74 homolog	Ift74	600	69.3	6	278478.8	307403.1	270234.7	321511.8	265947.7	254002.7	247742.5	240645.8	290906.3	292070.7	112694.1	141026.5	132178.0	147331.2
Q9JIA7	Sphingosine kinase 2	Sphk2	617	65.6	6.57	74228.9	66129.1	69860.2	68204.6	64814.7	73821.7	69754.0	68253.1	76781.2	73721.7	66493.7	63454.2	67278.9	65453.1
Q924T2	28S ribosomal protein S2, mitochondrial	Mrps2	291	32.3	9.14	148798.4	153941.2	151682.6	163404.1	159676.5	151594.0	149142.5	152688.6	165441.5	163085.1	125265.4	132924.1	129700.9	139155.8
O54901	OX-2 membrane glycoprotein	Cd200	278	31.2	8.79	142155.8	161990.8	122941.8	113363.4	158703.1	176046.8	184257.4	131034.4	138943.2	148459.2	234243.2	236494.8	224318.8	205043.0
Q8K2H2	Deubiquitinase OTUD6B	Otud6b	294	33.7	5.53	66031.4	90708.2	72891.2	73892.4	74820.6	79532.1	74087.1	79152.1	73121.6	81365.6	267130.0	231041.7	230735.8	219875.3
Q80XP8	Protein FAM76B	Fam76b	339	38.5	9.29	37878.5	40318.7	40945.4	37754.8	49532.8	40662.0	47837.4	41381.4	44752.9	45189.1	55814.7	48015.2	52214.4	44463.4
Q8BHZ0	CYFIP-related Rac1 interactor A	Cyria	323	37.3	6.01	32139.3	38406.6	28737.3	35311.0	36046.0	40172.9	36572.6	32037.8	28007.9	38136.0	82990.1	87682.4	72586.8	82282.1
Q3UJP5	Protein C8orf37 homolog	MGI:191440	209	23.8	6.86	75108.3	82659.8	85764.8	83863.7	83347.7	86707.0	90482.8	78885.4	78926.4	87119.5	1115477.6	1352886.2	1288691.9	1283816.0
Q9D6K8	FUN14 domain-containing protein 2	Fundc2	151	16.6	9.7	437950.2	439115.6	389629.1	392034.7	411948.5	400747.2	414897.6	456430.9	391761.9	394104.1	357346.1	349189.5	326835.1	331795.8
Q91V09	WD repeat-containing protein 13	Wdr13	485	53.6	9.14	68931.6	67210.9	61635.0	71859.0	62732.1	75282.7	103394.7	93695.0	72146.5	70477.0	145329.2	143250.6	124089.7	155326.3
Q9D786	HAUS augmin-like complex subunit 5	Haus5	619	69.5	8.43	199352.8	232224.7	235893.4	234288.3	253869.5	236899.1	192795.0	186103.9	221578.1	247070.2	37082.4	35970.7	25615.3	27999.2
Q9Z2L7	Cytokine receptor-like factor 3	Crlf3	442	49.5	5.03	46729.2	53770.3	44190.1	50642.1	57569.1	54069.8	53625.6	41905.1	43166.9	60026.9	215624.3	195188.7	237069.0	187788.4
Q9CXJ4	Mitochondrial potassium channel ATP-binding subunit	Abcb8	717	78	9.07	49753.8	63512.6	51629.7	65522.4	57023.7	58716.4	51647.5	56993.1	54754.6	63177.1	118728.3	119556.4	121903.3	129184.4
Q8VC16	Leucine-rich repeat-containing protein 14	Lrrc14	493	54.9	6.67	44860.1	57627.3	45063.7	53628.6	52739.0	54450.2	41272.3	46099.2	46331.3	50053.3	159234.7	171142.9	164970.0	203366.4
Q9Z1X2	Phosphatidylserine synthase 2	Ptdss2	473	55	7.09	162793.4	193524.6	165203.7	199867.4	163109.0	158127.4	160391.9	161210.7	178834.1	169429.5	173095.0	186167.1	174296.8	177872.5
P62627	Dynein light chain roadblock-type 1	Dynlrb1	96	11	7.25	141932.3	120518.7	164307.9	140619.7	133371.2	140520.4	115653.1	174278.4	142565.0	114053.0	83357.8	74781.3	100614.1	89235.5
Q62313	Trans-Golgi network integral membrane protein 1	Tgoln1	353	37.8	5.34	627049.3	699900.0	664691.4	653320.5	645300.8	620250.0	553250.2	610552.3	640785.5	621579.4	384876.6	401780.6	400358.6	409395.5
Q9R226	KH domain-containing, RNA-binding, signal transduction-associated protein 3	Khdrbs3	346	38.8	8.1	337207.5	492812.3	407939.6	520971.1	349253.3	329995.8	308074.4	326031.3	378307.5	430483.4	124690.1	168225.8	154962.0	186551.0
Q80UE6	Serine/threonine-protein kinase WNK4	Wnk4	1222	132.3	5.52	114893.1	105794.4	102731.5	109738.6	107638.4	103599.5	109339.0	105714.5	105658.1	104407.6	235896.1	276169.9	212393.6	268148.6
Q9WVF7	DNA polymerase epsilon catalytic subunit A	Pole	2283	261.9	6.44	131389.6	133324.8	124969.6	121063.2	126453.1	135933.6	127700.1	112448.5	130972.5	133288.1	137342.9	151427.8	134852.3	142608.2
Q9R061	Cytosolic Fe-S cluster assembly factor NUBP2	Nubp2	275	29.5	6.52	139418.3	125649.5	132885.4	132817.7	149488.4	138366.6	138790.4	134729.4	138338.4	132482.6	158193.7	175572.1	160991.4	170620.3
Q8BHK9	DNA excision repair protein ERCC-6-like	Ercc6l	1240	138.8	5.21	81323.6	85756.5	75428.2	85049.5	86636.0	89163.9	83006.0	77622.6	97946.6	102905.7	79528.4	87194.4	93817.0	79439.6
P11531	Dystrophin	Dmd	3678	425.6	5.94	84666.0	96940.2	75202.3	95760.4	79135.8	82093.4	72239.7	76826.9	83401.2	89601.0	60035.8	84866.2	56922.6	75550.0
Q9WVJ9	EGF-containing fibulin-like extracellular matrix protein 2	Efemp2	443	49.4	4.89	66321.0	60992.0	69864.9	62353.5	66240.9	82502.2	83867.9	82168.7	68494.7	62535.6	38171.9	39332.1	36332.7	27036.9
Q9CQZ5	NADH dehydrogenase [ubiquinone] 1 alpha subcomplex subunit 6	Ndufa6	131	15.3	10.11	95981.8	102807.2	96064.1	95673.6	105822.9	111310.8	91757.7	111807.8	92731.6	92393.9	216092.0	228744.9	239034.0	222464.9
P59114	mRNA (2'-O-methyladenosine-N(6)-)-methyltransferase	Pcif1	706	80.5	7.64	101253.8	115153.4	113263.1	96834.2	107854.0	115691.4	113784.3	104528.7	106251.6	111889.9	167565.0	185577.8	176690.0	173088.5
P97493	Thioredoxin, mitochondrial	Txn2	166	18.2	7.9	151877.5	145099.9	150425.3	156545.2	157404.9	159552.8	164185.8	159203.0	158127.8	157251.3	279054.9	262039.4	244784.6	272712.5
O70585	Dystrobrevin beta	Dtnb	659	74.4	8.62	251208.8	262116.9	239647.4	250359.6	282140.2	283056.4	285982.2	236675.1	270964.3	272743.7	196206.1	199376.3	188035.7	190211.1
Q8K157	Aldose 1-epimerase	Galm	342	37.8	6.74	82149.9	93008.9	86357.5	86826.8	98778.0	105854.7	119867.9	100603.2	93500.2	94783.8	60342.2	56989.0	47677.0	60137.8
Q8CHG7	Rap guanine nucleotide exchange factor 2	Rapgef2	1496	166.3	6.61	118079.6	131753.9	98778.7	109316.5	126704.3	134480.3	111496.8	122118.4	127043.9	131591.1	17901.9	13178.6	14013.1	15061.8
P56183	Ribosomal RNA processing protein 1 homolog A	Rrp1	494	54.7	5.17	133490.9	135321.1	128347.8	129989.1	132973.6	143414.6	117838.6	125162.7	120700.2	124103.3	108223.4	117865.1	114917.0	114688.3
P26262	Plasma kallikrein	Klkb1	638	71.3	8.02	220263.7	213368.5	186442.0	192609.8	165776.2	179407.6	184774.6	209601.2	172270.3	185533.5	58873.1	57365.2	50627.6	52904.6
Q8CAK1	Putative transferase CAF17 homolog, mitochondrial	Iba57	358	38.4	9.01	30334.5	41106.0	35936.0	44225.9	26795.9	27125.1	25385.1	26945.1	31701.7	34431.1	468614.0	492492.8	407127.0	476433.1
Q9CXV9	DCN1-like protein 5	Dcun1d5	237	27.6	5.44	295288.9	334852.9	344182.0	328457.4	343550.3	332688.4	281011.5	286745.6	321527.6	341548.7	222912.4	252045.9	235916.2	269771.3
Q8BJZ4	28S ribosomal protein S35, mitochondrial	Mrps35	320	36	8.59	189352.0	186877.7	165474.0	173965.8	165850.1	165106.7	166490.4	168026.6	179134.4	181941.4	253717.8	262221.1	229420.8	240301.5
Q9Z1B5	Mitotic spindle assembly checkpoint protein MAD2A	Mad2l1	205	23.6	5.2	224553.7	254749.3	247223.3	255157.3	269153.4	250544.7	221035.7	227152.8	247888.3	246242.5	340979.9	389219.5	371750.9	373028.7
Q8VC42	Regulator of MON1-CCZ1 complex	Rmc1	657	74.9	7.83	149997.5	112158.3	96011.5	104871.4	101762.7	104933.3	95851.7	95796.7	105130.5	102121.6	330954.7	375255.6	313948.6	340184.5
Q8R5A6	TBC1 domain family member 22A	Tbc1d22a	516	59.3	6.46	110135.3	126505.5	128412.6	107089.5	129299.6	126511.5	112804.5	124298.7	119283.4	130160.9	152245.2	153301.1	156246.5	153369.4
Q8C7Q4	RNA-binding protein 4	Rbm4	361	40	7.06	168869.4	170293.4	159677.3	159085.5	181594.3	180782.1	161159.9	160528.5	169091.3	176661.9	#N/A	#N/A	#N/A	#N/A
Q9CQQ8	U6 snRNA-associated Sm-like protein LSm7	Lsm7	103	11.6	5.27	70784.4	70607.3	75693.1	78413.1	72485.3	80035.5	76471.4	70728.6	85561.0	79460.1	819264.2	822979.9	921174.5	786774.2
P13595	Neural cell adhesion molecule 1	Ncam1	1115	119.4	4.83	101538.8	85674.6	104614.8	103904.0	95599.3	116639.0	116604.8	130505.3	92502.8	90226.2	65203.8	64838.6	62009.4	67687.4
Q7TNG8	Probable D-lactate dehydrogenase, mitochondrial	Ldhd	484	51.8	6.62	68087.4	58296.2	55541.9	66486.3	59383.3	56785.9	57383.6	66557.6	52453.2	58064.2	146319.9	141394.7	124417.3	137744.2
Q3V0G7	GTPase-activating Rap/Ran-GAP domain-like protein 3	Garnl3	1038	115.4	7.17	66760.5	71200.8	72695.0	57609.4	72972.6	67903.8	57911.6	69631.2	68131.4	67368.1	373306.6	373040.1	347088.9	354458.2
Q8R088	Golgi phosphoprotein 3-like	Golph3l	285	32.9	5.99	90934.0	97100.1	71628.1	89139.3	89184.8	85850.4	84631.9	90132.3	85760.1	94799.5	19694.6	21562.6	18778.7	20459.0
Q8CIF6	SID1 transmembrane family member 2	Sidt2	832	94.4	7.21	187510.9	188195.9	187769.9	171775.5	206637.9	208469.8	216700.0	193705.2	193526.9	183060.1	268913.9	231672.3	236124.1	235023.6
Q9CY97	RNA polymerase II subunit A C-terminal domain phosphatase SSU72	Ssu72	194	22.5	5.21	118749.1	125148.1	132247.3	120570.4	141477.2	138485.3	105250.2	117656.4	131811.8	132821.0	102236.4	112437.9	120376.8	109351.0
Q9DCZ4	MICOS complex subunit Mic26	Apoo	198	22.6	9.25	278864.6	291416.5	259814.1	286391.7	311811.7	273315.7	260500.5	254772.8	294250.1	299110.9	161144.7	181246.4	157014.2	169615.3
Q9CXN7	Phenazine biosynthesis-like domain-containing protein 2	Pbld2	288	32	5.34	105223.1	113391.6	126608.4	113151.8	135160.5	141985.6	190656.9	120781.9	116454.2	120158.3	98813.5	95868.2	100234.3	94307.8
P56212	cAMP-regulated phosphoprotein 19	Arpp19	112	12.3	9.09	409934.3	397205.8	464805.3	366654.0	413414.0	401245.2	402265.8	444950.1	423543.6	412132.9	268735.6	289942.4	274745.9	263912.7
Q91WG8	Bifunctional UDP-N-acetylglucosamine 2-epimerase/N-acetylmannosamine kinase	Gne	722	79.1	6.81	46679.7	57646.9	44761.6	59047.0	49134.9	51602.7	47594.4	48073.5	44492.3	55997.1	247288.1	250417.7	232797.9	263732.8
Q8BPG6	Inactive C-alpha-formylglycine-generating enzyme 2	Sumf2	308	34.7	7.11	181994.3	157988.5	141845.4	157798.5	155352.1	164389.7	159039.7	155558.7	159919.7	170722.1	130299.9	113893.1	89940.4	108219.9
Q9D883	Splicing factor U2AF 35 kDa subunit	U2af1	239	27.8	8.81	287668.0	300589.2	251433.0	267547.0	260572.2	272910.1	251130.7	238654.9	270907.5	284109.8	378615.6	420900.6	411510.3	445976.3
Q3UMW7	MAP kinase-activated protein kinase 3	Mapkapk3	384	43.3	8.09	69708.6	65288.8	65389.7	61035.8	67844.9	65425.1	62945.2	61184.5	65773.1	61277.4	127340.7	131780.8	121538.4	113260.7

Q8R1K1	Ubiquitin-associated domain-containing protein 2	Ubac2	345	39	9.44	147394.8	161444.0	128116.1	162007.7	132972.8	127598.4	123966.3	135029.8	140678.0	141383.3	142246.0	152275.5	146888.3	158965.9
Q8R3L2	Transcription factor 25	Tcf25	676	76.6	6.51	124269.4	121896.6	108873.0	103871.4	119726.6	120508.2	125286.0	130738.7	103587.5	119032.2	269105.2	284986.8	235371.3	277162.7
Q80X73	Protein pelota homolog	Pelo	385	43.3	5.99	141592.7	144740.5	140764.7	146395.0	134222.5	125011.7	124886.2	132423.6	146055.4	147573.1	405515.8	455862.5	402370.4	448443.0
Q8CGB6	Tensin-2	Tns2	1400	151.9	8.48	153685.8	167940.0	154383.1	145868.0	178710.2	178591.5	200421.6	158723.5	157899.0	167791.5	152776.6	173826.8	151044.0	156424.0
Q05BC3	Echinoderm microtubule-associated protein-like 1	Eml1	814	89.6	7.02	52770.2	52141.1	54563.0	46482.3	53183.2	60161.2	65899.3	55379.9	53360.7	47066.6	60232.8	54057.3	55823.3	58936.8
Q8BZ36	RAD50-interacting protein 1	Rint1	792	90	5.14	18005.1	22260.5	18480.8	19251.5	27316.5	22004.5	24500.1	17799.5	16125.5	17951.9	145674.7	155275.9	143315.8	161070.7
Q6NZK8	Protein tyrosine phosphatase domain-containing protein 1	Ptpdc1	747	83.9	6.34	163083.4	162244.9	155822.0	183731.2	158290.2	152267.8	146739.2	129528.5	154000.4	166995.5	151325.9	163832.9	199230.4	209699.4
Q9QYI4	DnaJ homolog subfamily B member 12	Dnajb12	376	42	8.51	120272.6	119407.0	124280.0	117899.3	130931.7	114904.2	101724.7	118857.3	122575.1	120166.1	124990.1	132035.4	103350.5	120292.5
Q8VCG3	WD repeat-containing protein 74	Wdr74	384	42.6	7.78	137435.5	136548.8	123660.5	135278.4	141900.3	134195.8	123774.8	127297.1	130636.6	135632.2	110306.3	105359.4	114011.1	109931.7
Q8BKH7	Target of rapamycin complex 2 subunit MAPKAP1	Mapkap1	522	59	7.53	70168.3	93253.5	69929.5	82077.7	100186.7	162662.1	74581.9	82633.4	67550.5	93332.5	182969.7	258880.5	202914.9	224280.6
Q6DFV1	Condensin-2 complex subunit G2	Ncapg2	1138	130.8	6.93	100193.0	117354.0	96747.9	95305.6	111335.0	117971.3	97863.9	98498.2	101960.7	106194.8	128085.0	128979.9	127044.5	125462.5
O54865	Guanylate cyclase soluble subunit beta-1	Gucy1b1	620	70.6	5.31	116779.2	120491.6	111278.9	123464.6	122928.3	122255.5	128590.9	113629.6	116290.6	109693.2	30544.5	34206.4	31654.6	34605.7
Q8BGI5	Peroxisome assembly protein 26	Pex26	305	34	6.8	90463.0	115418.0	74230.0	87549.6	86109.0	83670.9	80750.9	80881.5	89455.0	104385.7	364445.5	381835.5	302631.7	378748.6
Q8BX80	Cytosolic endo-beta-N-acetylglucosaminidase	Engase	734	82.9	6.65	69793.6	108496.0	59837.7	78005.3	58974.3	72782.6	68210.4	62209.7	67562.0	73668.7	85564.9	104424.9	78926.7	101558.0
Q7TN22	Thioredoxin domain-containing protein 16	Txndc16	820	92	5.31	116992.2	140097.7	121667.9	132732.8	144519.9	118813.1	128741.7	125001.8	127887.7	143051.1	58853.6	64661.0	61647.4	56380.3
Q8BX94	Oxysterol-binding protein-related protein 2	Osbpl2	484	55.3	6.2	113202.8	116424.5	99970.5	113567.5	118515.8	117256.9	110639.5	108673.7	105208.8	99907.7	59896.9	67184.3	63760.9	59099.9
P70188	Kinesin-associated protein 3	Kifap3	793	91.2	5.11	86414.2	102402.6	99108.8	108622.6	80454.1	82689.4	76689.8	79698.7	96388.4	98551.1	227906.1	277675.8	248778.6	301092.9
P97821	Dipeptidyl peptidase 1	Ctsc	462	52.3	6.89	48012.9	58338.6	46690.7	60899.5	47248.4	52609.9	47964.6	59130.9	40648.6	41827.7	40193.0	43765.8	33033.8	40619.5
Q9CQI6	Coactosin-like protein	Cotl1	142	15.9	5.4	906573.0	850387.9	742417.9	868184.9	682318.4	841426.6	932677.8	790440.5	750456.2	695387.7	400472.7	311603.4	328147.1	341364.9
Q9CR26	Vacuolar protein sorting-associated protein VTA1 homolog	Vta1	309	33.9	6.13	422405.7	454768.9	439865.2	448708.3	421329.8	379755.9	378677.2	476376.9	439683.9	443367.2	276285.7	275502.3	250242.3	277510.7
Q64314	Hematopoietic progenitor cell antigen CD34	Cd34	382	41	5.3	692172.7	549195.5	483917.6	526482.2	627472.6	721292.1	881653.7	599307.0	522396.8	527077.7	879926.2	777479.3	649981.9	759982.0
Q9DBM1	G patch domain-containing protein 1	Gpatch1	930	102.9	6.98	34075.3	40306.8	27823.7	31104.6	38131.9	35069.4	33227.3	37330.2	35135.7	37845.3	71501.7	70224.5	79007.5	68892.7
Q810Q5	Normal mucosa of esophagus-specific gene 1 protein	Nmes1	83	9.6	9.89	227742.2	336818.4	326452.4	385897.1	238192.2	209242.3	197578.1	241501.8	257742.8	275331.7	248316.7	325221.0	329103.8	456818.6
Q8BUE4	Ferroptosis suppressor protein 1	Aifm2	373	40.6	8.98	182741.3	146258.9	182898.0	167952.8	151791.4	156523.7	154072.6	150496.4	136932.6	132479.0	265931.1	278758.2	252142.8	261756.5
P49446	Receptor-type tyrosine-protein phosphatase epsilon	Ptpre	699	80.6	7.12	127288.4	116564.0	104447.3	132000.7	131408.6	109616.0	113205.6	103730.9	130366.2	125435.8	158016.2	170483.3	145020.1	162807.5
P47713	Cytosolic phospholipase A2	Pla2g4a	748	85.2	5.43	162023.4	183513.6	157392.9	169751.2	159976.5	150755.6	145448.0	160474.2	161626.0	183303.7	148262.8	156354.2	147620.6	148930.4
Q9JKK1	Syntaxin-6	Stx6	255	29	4.92	164897.3	162756.0	176620.3	176361.6	195387.6	190409.2	192095.2	175463.2	182441.4	177723.8	115943.9	111245.9	101246.6	101829.6
Q8BPB0	MOB kinase activator 1B	Mob1b	216	25.1	6.73	516319.3	524407.8	553422.2	527694.3	555418.1	579116.6	520149.4	508318.7	526979.2	539398.5	428009.4	437687.2	406243.3	418331.5
Q91VY9	Zinc finger protein 622	Znf622	476	53.4	6.1	103769.1	109286.2	107990.8	102994.0	104486.1	104392.1	103523.9	95247.8	108279.9	109679.5	105405.9	111882.3	107687.2	113825.5
Q91WE6	Threonylcarbamoyladenosine tRNA methylthiotransferase	Cdkal1	578	65.2	7.65	36744.1	33151.4	35558.7	39028.5	38599.1	36894.6	31988.2	36376.1	31348.2	35004.9	28404.9	29965.5	29228.5	35507.4
Q8VDP6	CDP-diacylglycerol--inositol 3-phosphatidyltransferase	Cdipt	213	23.6	8.27	230491.6	201382.1	206779.8	223371.1	209080.4	206023.6	197837.0	204361.7	200244.9	199952.7	186176.5	186202.2	186503.9	185557.8
Q9D7A8	Armadillo repeat-containing protein 1	Armc1	282	31.2	5.57	304325.8	329145.6	290516.5	293080.6	292485.2	284668.0	282775.0	292545.7	288320.4	301035.6	772042.4	825120.5	808926.0	809094.5
Q9JJR2	X-linked lymphocyte-regulated protein 5C	Xlr5c	179	20.7	7.03	75787.3	83888.2	78919.8	80076.7	86623.8	74462.2	69754.1	71979.8	80327.9	82001.2	226555.4	248914.9	237975.0	226445.0
Q9D710	Thioredoxin-related transmembrane protein 2	Tmx2	295	33.9	8.75	268449.3	266513.9	289561.0	263939.2	263814.6	242095.9	238305.0	265347.4	248373.3	248387.2	255094.3	300137.7	246830.0	276838.4
Q9QXK2	E3 ubiquitin-protein ligase RAD18	Rad18	509	57.4	7.24	193793.7	209898.4	205112.8	224640.8	200454.8	200633.6	165603.5	185721.6	217054.3	200851.2	581175.3	833147.6	771576.4	877454.3
P35290	Ras-related protein Rab-24	Rab24	203	23.1	6.23	157132.5	179806.2	156691.7	175641.0	157422.9	157236.7	151743.0	143719.9	163928.7	180189.6	121010.2	131240.0	140470.0	148632.2
Q62241	U1 small nuclear ribonucleoprotein C	Snrpc	159	17.4	9.67	311809.7	313197.3	404138.2	372884.0	389936.0	358884.2	367117.5	356900.4	357274.9	360986.2	223735.0	223188.8	277045.9	245753.3
Q91WR3	Activating signal cointegrator 1 complex subunit 2	Ascc2	749	85.6	5.03	47489.4	51007.9	45651.4	46538.6	46938.5	44484.1	45668.1	45247.0	43324.7	52724.8	31430.3	35906.7	39309.6	32713.2
Q9Z0E6	Guanylate-binding protein 2	Gbp2	589	66.7	5.71	55422.3	64456.8	57460.4	70001.7	61792.5	75490.3	82783.1	68899.0	68243.4	62136.8	70701.0	66175.2	69113.3	58810.2
Q00993	Tyrosine-protein kinase receptor UFO	Axl	888	98.1	5.74	48476.3	41415.9	39122.5	36903.3	39781.5	46673.1	41940.4	42082.7	42878.4	42785.4	15326.9	11777.1	13876.0	12190.4
Q99MJ9	ATP-dependent RNA helicase DDX50	Ddx50	734	82.1	9.25	132618.9	133662.1	118233.4	119901.0	136935.9	155155.3	130802.3	133165.0	133104.8	130308.9	259831.8	232982.2	242392.6	238646.3
Q60680	Inhibitor of nuclear factor kappa-B kinase subunit alpha	Chuk	745	84.7	6.77	145160.8	160658.3	150092.5	133856.9	150220.6	151431.5	132206.6	133259.1	141950.7	152936.9	46300.7	49579.5	44106.3	42672.4
Q9WU56	tRNA pseudouridine synthase A	Pus1	423	47.5	8.9	145001.2	175268.8	149777.4	154768.6	166486.9	163781.0	162753.8	153621.7	161017.7	164898.9	126142.5	133601.7	136212.6	126246.4
P83870	PHD finger-like domain-containing protein 5A	Phf5a	110	12.4	8.41	442358.4	490159.1	514786.0	508213.8	535220.1	508288.2	480404.8	461662.9	502613.9	525778.5	164466.3	177098.4	189958.2	181300.7
P98078	Disabled homolog 2	Dab2	766	82.3	6.1	209943.8	219678.3	152788.1	194136.2	154967.9	168851.2	166092.5	196447.5	160259.5	164222.5	410528.6	421016.6	335174.7	389243.4
O35609	Secretory carrier-associated membrane protein 3	Scamp3	349	38.4	7.64	24096.6	21195.5	19950.0	25071.9	21518.8	20663.7	27486.3	25088.4	24910.7	21116.9	96807.0	94368.7	93822.5	89976.5
Q8BRK9	Alpha-mannosidase 2x	Man2a2	1152	130.6	6.87	69979.0	63836.2	74234.3	62437.4	66339.3	67978.1	68598.8	72210.7	67548.3	68506.2	75075.0	85448.7	71210.0	82625.1
B2RWS6	Histone acetyltransferase p300	Ep300	2412	263.1	8.54	16223.6	10208.6	14864.4	9806.9	17254.6	15714.0	12733.5	10780.4	12971.9	7602.9	108464.0	108221.8	108903.1	102029.2
E9QAP7	TAFH domain-containing protein	Taf4	1042	105.2	9.86	131148.2	134825.8	145738.0	119901.0	154350.9	183892.7	150744.7	142522.0	137523.9	139626.2	107655.0	106821.7	114732.6	83148.9
Q8BUN5	Mothers against decapentaplegic homolog 3	Smad3	425	48.1	7.15	76943.5	91604.7	68065.0	51765.3	175575.0	122170.7	102230.6	77765.5	82051.0	118671.2	72460.5	79568.3	74561.4	59428.6
Q9QZI9	Serine incorporator 3	Serinc3	472	52.6	7.23	18646.1	23822.5	19554.0	21819.9	24453.8	23710.5	20172.0	19700.5	22866.0	18954.9	232050.6	213389.7	206598.3	225273.5
Q9DC71	28S ribosomal protein S15, mitochondrial	Mrps15	258	29.4	10.13	96609.9	134563.4	110201.3	108877.0	114343.8	125728.0	116260.8	113596.9	124200.3	136887.5	177442.0	177108.4	160070.4	164096.1
P48455	Serine/threonine-protein phosphatase 2B catalytic subunit gamma isoform	Ppp3cc	513	58.7	7.25	156960.0	177232.3	178423.1	226022.8	139304.0	130195.3	131020.9	136194.7	152152.2	157524.5	21185.3	25985.6	25458.7	34340.2
Q8C5P5	5'-nucleotidase domain-containing protein 1	Nt5dc1	467	53.1	6.33	77995.6	98228.7	96999.9	93609.0	91259.5	88634.4	79660.8	79712.9	80160.4	89722.7	117351.7	137988.6	147492.6	158109.5
Q80Y56	Rabenosyn-5	Rbsn	783	88.4	5.3	50895.8	57414.8	52063.1	49890.3	50384.2	52212.4	52816.1	57965.1	56202.8	51020.8	46902.8	51658.4	46519.0	49085.2
Q9R0P6	Signal peptidase complex catalytic subunit SEC11A	Sec11a	179	20.6	9.33	1314782.6	1298475.9	1397911.1	1395321.2	1288491.6	1306001.9	1316582.1	1305789.5	1326128.0	1346192.3	921399.5	937929.5	962959.7	945799.3
Q9EST4	Proteasome assembly chaperone 2	Psmg2	264	29.5	6.05	258222.8	351709.0	271874.7	324209.0	328325.9	294978.9	251996.4	264192.6	310797.4	347530.7	248961.0	269101.5	284383.5	281833.9
Q99LY9	NADH dehydrogenase [ubiquinone] iron-sulfur protein 5	Ndufs5	106	12.6	8.92	454908.7	487151.0	447433.1	479563.3	458052.0	448357.5	458487.3	427610.4	474460.4	500010.4	429882.6	424418.1	411207.3	418592.9
Q9QZR0	E3 ubiquitin-protein ligase RNF25	Rnf25	456	51.2	6.29	165897.8	173801.2	162116.7	178557.7	163594.2	167653.8	157372.3	165269.2	160542.0	166726.0	34938.4	37321.3	34749.3	42366.0
Q6ZPZ3	Zinc finger CCCH domain-containing protein 4	Zc3h4	1304	140.9	6.27	35457.8	41479.9	38097.7	32974.2	36257.2	36541.4	33675.9	36688.7	36035.7	33299.0	21335.4	17479.5	23018.0	22863.9
Q9CQU3	Protein RER1	Rer1	196	23	9.51	675664.7	655827.3	705598.2	715818.4	695538.1	681576.7	618213.6	730416.8	682551.3	629030.5	767476.8	762982.9	780369.6	787203.6
Q9WV68	Peroxisomal 2,4-dienoyl-CoA reductase	Decr2	292	31.3	8.81	129407.8	140641.3	130466.5	138703.9	132412.5	131329.2	142944.5	132119.9	141344.2	133359.2	86745.9	78867.7	81601.2	95812.9
P33610	DNA primase large subunit	Prim2	505	58.4	8.32	107731.0	109966.4	102706.7	100364.9	131368.8	139841.4	115211.8	122310.0	109063.3	115240.0	113276.1	103257.7	102822.9	92920.0
O55042	Alpha-synuclein	Snca	140	14.5	4.77	239892.3	195287.9	257752.3	210202.1	245170.4	211357.7	233290.4	254470.8	250484.6	226428.3	343148.9	306658.9	272274.6	354803.2
E9Q774	A kinase (PRKA) anchor protein 11	Akap11	1895	208.7	5.36	75066.4	73168.4	78744.9	71372.9	78342.4	81803.9	75783.9	75860.8	78940.4	78039.0	136972.8	139568.5	125492.8	131325.3
Q9CR95	Adaptin ear-binding coat-associated protein 1	Necap1	275	29.6	6.38	182709.8	209524.2	168850.5	192639.2	188783.5	180128.8	175165.2	173871.8	174520.9	193651.0	170507.0	153293.6	146626.6	143944.1
Q5KU39	Vacuolar protein sorting-associated protein 41 homolog	Vps41	853	98.5	5.81	256637.0	289408.0	243358.3	259676.5	274603.6	271832.3	245131.1	244551.6	258493.7	279534.4	188941.5	199511.9	178449.3	191132.0
Q91XL1	LRRCT domain-containing protein	Lrg1	342	37.4	6.61	156470.4	147538.9	144421.6	146399.8	120040.2	144603.4	218643.9	154025.4	194949.4	182546.8	267836.1	274607.6	259387.7	252744.3
Q8BTH8	Casein kinase I isoform gamma-1	Csnk1g1	459	52.7	9.01	164480.5	165888.4	163977.3	169201.0	178479.4	182811.7	169546.4	163493.4	167386.3	169345.5	19560.8	19478.0	18474.8	19705.4
Q8BTY2	Sodium bicarbonate cotransporter 3	Slc4a7	1034	116.4	6.24	50721.3	56221.6	47211.7	50660.4	61730.1	50527.4	47096.0	46469.6	53749.9	44507.1	81103.3	84964.0	86109.8	90049.5
Q8BH66	Atlastin-1	Atl1	558	63.3	6.49	56798.1	71827.5	66834.2	71546.6	61920.6	66621.6	56625.8	57066.3	64822.1	66295.8	#N/A	#N/A	#N/A	#N/A
Q8K4M5	COMM domain-containing protein 1	Commd1	188	21	7.59	175807.0	182752.6	163358.0	175436.3	170691.7	177508.4	169393.7	162911.6	163959.4	173298.4	95437.9	91304.6	98668.6	92114.7
Q6Y5D8	Rho GTPase-activating protein 10	Arhgap10	786	89.3	7.18	253439.5	243308.3	227899.2	231649.3	277669.8	261188.9	279994.2	244800.9	251277.6	250817.7	198552.4	197184.1	184788.6	187362.0
P62305	Small nuclear ribonucleoprotein E	Snrpe	92	10.8	9.44	289651.0	316717.1	310570.9	289839.8	329919.9	327920.4	295376.3	293965.2	325150.3	308676.2	274835.1	275871.2	268555.4	247886.5
P83093	Stromal interaction molecule 2	Stim2	746	83.9	6.79	86097.7	108487.7	82053.0	81732.8	101747.8	124936.6	103379.9	111212.7	100600.7	99013.7	77520.3	79545.0	64669.0	75138.2
Q3UPF5	Zinc finger CCCH-type antiviral protein 1	Zc3hav1	946	106.6	8.27	113221.5	116906.9	113366.0	111782.7	122173.2	125028.8	121220.6	116030.9	113938.3	116112.6	127248.0	127202.9	132340.2	115178.8
Q9D0J8	Parathymosin	Ptms	101	11.4	4.22	219327.2	183589.8	190399.5	196230.6	238409.0	280962.2	296556.4	264770.3	214696.7	205345.4	269332.3	224218.7	213942.2	219927.4
P28798	Progranulin	Grn	589	63.4	6.8	213243.2	203269.9	200528.4	210814.7	199664.1	197857.3	218000.3	197260.8	211110.1	198500.4	217120.5	207455.4	195851.0	212410.1
Q9DCS2	Methyltransferase-like 26	Mettl26	204	22.7	6.52	55364.8	55358.9	42170.4	58427.3	63178.4	59451.8	56587.1	59057.4	50292.0	57759.6	186662.2	199475.8	170122.8	169470.7
Q8CC86	Nicotinate phosphoribosyltransferase	Naprt	538	58.2	6.46	187912.8	172826.6	163181.8	154052.3	177389.5	195924.4	198570.1	170733.7	167336.4	190256.3	291566.5	284603.8	240942.0	252779.4
Q8R0H9	ADP-ribosylation factor-binding protein GGA1	Gga1	635	69.9	5.27	163895.2	179130.5	164905.5	170363.4	151101.2	157660.2	162908.5	152328.1	165856.6	160653.4	212351.4	252076.7	218629.7	238165.2
Q9QXA1	Cysteine and histidine-rich protein 1	Cyhr1	311	35.7	7.68	127252.5	136919.9	135789.9	106275.0	138480.2	126267.5	119279.4	110215.2	128560.0	142236.4	151196.7	148086.2	131687.6	149002.3
Q8BMZ5	tRNA-splicing endonuclease subunit Sen34	Tsen34	316	34.2	7.05	131630.7	140759.3	127781.4	138107.0	134689.8	152316.1	130303.6	124904.5	133086.5	131225.1	103169.1	118174.2	108423.6	116399.3
Q5M8N4	Epimerase family protein SDR39U1	Sdr39u1	293	31.4	9.31	191456.3	194171.8	183527.6	213783.0	180907.8	179399.5	183201.8	164919.8	191461.3	204942.7	224825.5	228104.2	232873.5	225666.6
Q9CW79	Golgin subfamily A member 1	Golga1	758	87.3	5.52	110349.5	138267.7	123254.9	141515.0	121799.8	114646.2	113558.2	119928.7	112893.4	122728.9	133785.5	147803.6	140497.1	151986.7
Q8CF89	TGF-beta-activated kinase 1 and MAP3K7-binding protein 1	Tab1	502	54.6	5.52	52212.6	49546.5	43887.3	51213.3	45468.6	49537.9	47117.9	48591.4	50385.4	51925.5	181091.3	181564.2	179994.5	160226.6
C0HKD9	Microfibrillar-associated protein 1B	Mfap1b	439	51.9	4.98	148556.6	119059.5	157430.2	122812.5	162857.4	158891.0	110958.3	154424.1	126739.3	120364.9	146811.8	136341.2	145133.5	116594.8
Q2VPQ9	Chromatin modification-related protein MEAF6	Meaf6	191	21.6	9.32	342294.4	373026.4	346742.9	362408.2	392081.3	397294.5	369738.1	375314.7	364340.1	355864.2	204977.3	209717.1	205591.0	207721.0
Q3TNA1	Xylulose kinase	Xylb	551	59.5	6.9	116592.4	134917.3	126081.3	128068.7	136995.0	132570.6	168405.7	123285.9	124181.8	131770.1	72172.5	76477.9	69716.1	69227.6
O88455	7-dehydrocholesterol reductase	Dhcr7	471	53.9	8.35	292058.0	283564.5	246509.8	299828.2	264112.0	257247.4	246311.0	250289.2	270436.4	271947.7	83391.4	82269.6	81212.3	96601.8
O35682	Myeloid-associated differentiation marker	Myadm	320	35.3	8.31	144223.2	146036.3	123685.5	144320.9	133660.7	150360.3	151389.3	140566.4	129237.1	147697.3	81487.1	90113.6	78139.2	80211.4
Q3TYS2	Cytochrome b-245 chaperone 1	Cybc1	187	20.9	8.59	123559.4	123702.2	117886.8	119669.9	126689.3	125249.4	128802.0	130394.6	120073.8	122546.3	739154.7	675144.1	649840.9	674391.9

D3YV10	Coiled-coil domain-containing protein 13	Ccdc13	709	79.7	9.01	71934.4	81693.4	81537.2	102122.2	70120.0	70488.8	68901.1	65460.6	74873.5	83308.6	55350.6	65606.7	67202.4	77989.8
Q8C0D4	Rho GTPase-activating protein 12	Arhgap12	838	95.3	7.59	132472.1	142140.4	126061.1	123457.8	131542.2	126600.7	123843.5	115536.0	116854.5	134124.7	111989.8	108432.5	97128.1	103170.1
Q6DFW5	Phospholipid-transporting ATPase	Atp11b	1175	133.5	6.92	173066.5	159477.4	164862.9	177044.9	167088.1	178717.9	172417.4	165203.9	175406.8	149509.5	134667.3	154011.9	138927.0	149287.3
Q6ZWU9	40S ribosomal protein S27	Rps27	84	9.5	9.45	337721.3	358753.7	370917.5	336084.7	352357.9	347006.4	301667.2	329021.7	348584.3	368636.1	#N/A	#N/A	#N/A	#N/A
Q99M71	Mammalian ependymin-related protein 1	Epdr1	224	25.5	7.58	76317.3	62121.7	76079.0	62671.4	90148.9	98793.8	85818.4	81422.1	78639.6	79185.0	50864.0	39388.0	49534.9	40514.3
Q91V83	TELO2-interacting protein 1 homolog	Tti1	1085	120.7	6.1	143905.3	148659.8	141910.6	141678.2	135357.6	150686.3	130635.6	128183.2	142455.5	152669.8	22917.9	30690.3	28512.2	25078.1
Q99JT9	1,2-dihydroxy-3-keto-5-methylthiopentene dioxygenase	Adi1	179	21.5	5.5	288599.1	339524.8	287520.9	310627.8	354434.7	364197.3	377832.2	300100.9	391010.0	365045.9	47104.1	109755.3	62914.5	77178.5
Q99KN2	Probable cytosolic iron-sulfur protein assembly protein CIAO1	Ciao1	339	37.6	4.88	32535.8	34729.0	35238.1	32708.6	30858.5	36832.7	27889.4	32985.2	30412.9	32039.9	98623.0	100703.9	102621.4	100045.5
Q8BVL3	Sorting nexin-17	Snx17	470	52.8	7.49	105167.2	100268.6	97465.6	95728.3	104595.0	103818.0	98533.7	92095.4	93386.7	88990.2	63267.0	65545.5	60444.3	62629.7
Q9JL70	Fanconi anemia group A protein homolog	Fanca	1439	161.1	7.2	34203.9	36811.0	39459.3	39468.0	38681.4	43872.4	35170.9	38663.8	40107.2	39123.8	1774.9	6294.0	3223.3	5521.4
Q99N94	39S ribosomal protein L9, mitochondrial	Mrpl9	265	30.2	10.08	199446.1	201243.0	191083.0	199676.5	201165.4	208160.4	191882.9	188539.1	205971.2	216844.3	173104.6	198111.2	169685.4	175501.0
Q9D0F1	Kinetochore protein NDC80 homolog	Ndc80	642	73.9	5.95	70491.1	80292.5	91876.2	99190.2	72392.7	70501.4	61122.4	64020.6	79659.6	87952.0	109878.8	111684.7	136900.1	121103.4
Q9D3P8	Plasminogen receptor (KT)	Plgrkt	147	17.3	9.5	339729.3	320046.5	369628.9	344946.7	374413.2	361385.1	378299.1	419920.9	354190.7	337546.9	223347.9	236036.5	248773.2	237472.0
Q8VEC4	Calcium homeostasis modulator protein 2	Calhm2	323	35.8	8.28	95763.9	89655.9	75133.2	75392.9	86613.0	92061.0	85608.4	77698.3	95615.5	93029.2	#N/A	#N/A	#N/A	#N/A
P23242	Gap junction alpha-1 protein	Gja1	382	43	8.76	146217.2	127514.4	142970.2	122613.2	147049.4	137254.7	139012.6	141965.2	138341.7	139082.6	69334.4	69505.5	78612.3	65194.2
Q6VVW5	Atrial natriuretic peptide receptor 2	Npr2	1047	117	6.87	129660.3	123343.6	138359.3	132763.4	148626.0	167220.3	148379.4	146437.7	134919.3	137427.6	#N/A	#N/A	#N/A	#N/A
Q9JJW6	Aly/REF export factor 2	Alyref2	218	23.7	10.04	26573.2	26876.0	28747.2	27873.8	23633.0	28093.2	29217.1	26150.9	27845.4	27037.3	#N/A	#N/A	#N/A	#N/A
Q9CQJ2	PIH1 domain-containing protein 1	Pih1d1	290	32.2	5.21	162601.5	155838.5	180092.0	165992.7	148944.5	153890.5	146021.3	169804.3	161121.3	158917.3	131573.2	128249.0	132486.8	131896.2
P20060	Beta-hexosaminidase subunit beta	Hexb	536	61.1	8.12	169045.3	165966.6	118578.7	182363.3	140914.8	163607.4	198740.7	158153.1	140491.9	148748.8	170835.0	159687.7	138970.3	173861.6
Q6ZWY3	40S ribosomal protein S27-like	Rps27l	84	9.5	9.45	210174.2	263291.4	227126.1	234689.9	254964.0	247621.7	195542.3	230684.8	237856.7	245801.2	809178.0	749328.8	856428.4	737132.9
Q3U3W5	Protein arginine N-methyltransferase 9	Prmt9	846	94.2	5.36	170999.5	154571.7	157288.1	150778.7	163809.8	149365.3	165265.0	127667.4	166066.3	172110.1	80951.5	91265.7	90592.1	98330.7
Q9Z2H5	Band 4.1-like protein 1	Epb41l1	879	98.3	5.62	228888.1	240629.9	197922.2	215839.2	240973.2	242375.5	247316.2	197706.6	218388.4	218404.6	150776.6	157070.1	139633.3	145476.0
Q9DBH0	NEDD4-like E3 ubiquitin-protein ligase WWP2	Wwp2	870	98.7	7.11	57694.5	79020.4	62795.1	73231.2	61074.3	65372.2	46970.4	59363.0	160179.0	70650.6	#N/A	#N/A	#N/A	#N/A
Q9D6N5	Dr1-associated corepressor	Drap1	205	22.3	5.26	153128.0	170564.3	153210.6	191092.0	137255.2	139185.2	128153.6	128096.5	139802.7	154538.9	513631.5	583396.4	580505.5	684395.2
Q3TC93	HCLS1-binding protein 3	Hs1bp3	395	43.7	4.98	251436.4	232047.5	202341.0	222601.6	222505.7	219967.3	233870.4	206144.2	217629.3	241831.5	17212.8	23249.9	19672.8	20922.2
Q8VD76	General transcription factor IIH subunit 3	Gtf2h3	309	34.2	6.76	228265.7	211303.4	248182.5	207136.9	218619.3	221219.3	196072.3	181358.7	218090.0	204845.9	365471.7	365683.4	415308.7	383674.3
P46718	Programmed cell death protein 2	Pdcd2	343	38.3	5.38	122829.2	125808.6	129617.7	119036.4	116267.2	123150.5	109844.6	126434.9	122612.8	120519.6	61288.3	69362.6	63064.8	63007.0
Q9R059	Four and a half LIM domains protein 3	Fhl3	289	31.8	6.2	290161.1	292717.1	278001.2	304075.0	323732.2	276164.6	295346.3	285309.7	325980.4	292101.0	154318.3	141895.7	166732.1	149103.5
Q9ERL7	Glia maturation factor gamma	Gmfg	142	16.7	5.71	69434.3	63010.6	51062.5	64290.3	60126.7	63679.9	64339.7	60579.8	61530.6	65104.8	239897.9	250475.9	201890.7	288399.1
Q8BG40	Katanin p80 WD40 repeat-containing subunit B1	Katnb1	658	72.6	7.27	85794.0	106867.6	91629.2	91893.0	96853.7	88864.8	76824.9	80345.1	86919.7	98066.9	106725.5	130470.3	92532.3	101830.6
Q7M6Z4	Kinesin-like protein KIF27	Kif27	1394	158.9	7.14	46626.2	54506.3	42752.2	56177.9	44225.1	49178.8	45558.4	45540.1	43326.6	46091.5	127752.3	130339.3	127969.3	150384.1
Q80XC6	Nuclear exosome regulator NRDE2	Nrde2	1172	133.4	7.93	52233.0	51454.5	45967.0	43672.3	46445.3	49972.3	52084.7	44758.7	50596.4	53993.1	40669.2	38772.0	37580.7	39125.0
Q60632	COUP transcription factor 1	Nr2f1	422	46.1	8.25	133040.2	132722.2	136239.0	128469.6	148679.3	170777.2	187880.6	156981.6	139622.4	131732.2	234359.0	200651.7	248044.0	192930.5
Q9QYR6	Microtubule-associated protein 1A	Map1a	2776	300	5	71182.2	70293.6	63336.2	68371.3	64560.3	70705.4	67454.0	67955.4	68668.0	61666.6	79477.2	81239.0	62300.5	75830.1
A2A5N3	Polyadenylate-binding protein	Pabpc1l	607	67.4	8.51	14956.6	16729.2	15167.0	13974.0	14093.8	16467.0	15135.5	18003.5	17573.9	15436.6	#N/A	#N/A	#N/A	#N/A
P83882	60S ribosomal protein L36a	Rpl36a	106	12.4	10.58	185000.7	238797.9	195370.4	234276.7	169965.7	164734.5	203643.1	171727.8	229675.3	233038.3	293600.7	301741.3	246359.2	297353.3
Q8CJG1	Protein argonaute-1	Ago1	857	97.2	9.16	83346.3	90926.3	84847.5	85484.8	95797.0	97391.6	101506.4	84362.2	88552.0	101223.5	50156.8	49370.1	48646.5	48014.7
Q91VX2	Ubiquitin-associated protein 2	Ubap2	1132	117.9	7.72	68309.3	70006.0	71797.3	65467.2	68390.8	67686.0	68074.9	63662.5	66606.6	71433.0	97139.7	95061.3	103233.2	102345.8
Q61559	IgG receptor FcRn large subunit p51	Fcgrt	365	40.1	5.29	74452.2	66910.3	70329.9	62863.5	77231.7	79595.8	84601.0	77044.0	64518.8	65103.0	58804.9	50680.5	50997.0	45018.9
P61022	Calcineurin B homologous protein 1	Chp1	195	22.4	5.1	121484.1	145368.7	120635.8	133125.9	124172.9	131057.1	121296.2	116826.2	127063.6	129685.2	134584.4	126853.3	117898.1	137867.3
P48193	Protein 4.1	Epb41	858	95.9	5.6	42306.1	39341.1	44366.1	35718.5	42889.6	51092.8	52229.4	39839.5	49777.3	46165.2	50992.3	55327.5	53985.5	55443.6
H3BJD6	Protein phosphatase 1, regulatory subunit 9A	Ppp1r9a	1292	144.3	5.55	100032.2	108376.9	118095.8	104555.7	119186.8	106191.4	113870.2	114586.9	111558.1	116304.1	54735.2	63919.3	54899.4	61023.9
Q9D1P0	39S ribosomal protein L13, mitochondrial	Mrpl13	178	20.7	9.35	131151.5	151168.0	129556.7	141934.2	142650.2	145049.7	131982.8	134629.7	132055.0	151639.8	217408.4	216561.6	218799.9	214851.7
Q9EQN3	TSC22 domain family protein 4	Tsc22d4	387	40	8.13	61817.5	80582.4	68482.7	69721.1	90851.7	73527.4	74228.3	66006.1	67500.2	67039.9	724010.8	751653.3	767280.9	803655.6
Q8C9S4	Coiled-coil domain-containing protein 186	Ccdc186	917	104.8	5.53	150570.2	150243.4	152419.4	144530.8	153594.9	165607.4	151444.5	146474.4	141379.0	148383.3	55201.8	50026.6	49499.1	50610.5
Q91YU8	Suppressor of SWI4 1 homolog	Ppan	470	52.7	10.32	227141.5	241991.5	236421.1	229270.2	252193.1	239051.5	214511.3	233593.8	234837.5	251612.2	119781.2	109411.3	109167.9	103349.8
Q8K339	DNA/RNA-binding protein KIN17	Kin	391	44.7	9.04	150408.5	160579.4	160338.3	155845.3	162876.3	156236.6	149076.3	141222.0	154210.6	165534.8	94844.1	98550.8	95636.5	101724.0
Q8BUH8	Sentrin-specific protease 7	Senp7	1037	116.3	6.52	46203.1	52539.5	40462.4	44475.8	53729.5	47727.3	49763.2	45645.3	51073.7	48013.3	47775.1	41576.4	44450.4	39737.8
A0A075B5V1	Ig-like domain-containing protein	Ighv1-31	98	11	8.73	143278.7	112934.0	173207.9	177680.2	160468.2	112660.1	152155.1	160233.6	158752.6	151534.3	128316.2	104580.6	150394.8	139842.3
P62743	AP-2 complex subunit sigma	Ap2s1	142	17	6.18	156480.9	191567.5	160730.2	189932.1	169988.7	184299.8	174749.3	153222.2	176214.9	190460.5	448325.6	467239.5	441248.7	492876.6
Q6P1G0	HEAT repeat-containing protein 6	Heatr6	1184	128.8	7.24	49654.6	54261.7	50068.0	42530.8	47641.2	48914.4	47376.8	42760.9	48962.7	47484.2	60926.9	65020.1	59782.6	58510.6
Q8C3X2	Coiled-coil domain-containing protein 90B, mitochondrial	Ccdc90b	256	29.6	8.53	97719.0	106452.5	98475.4	103150.3	109010.6	109193.8	109732.2	93057.4	108928.6	103257.5	220088.7	245063.7	235534.3	248593.8
Q9JKY5	Huntingtin-interacting protein 1-related protein	Hip1r	1068	119.4	6.52	132456.4	147578.0	136294.2	126698.5	140901.5	143939.6	130484.2	126095.9	132820.6	142353.7	186985.9	216751.7	215771.0	214435.3
Q3UL36	Arginine and glutamate-rich protein 1	Arglu1	271	32.9	10.36	428188.5	454400.0	466989.5	447282.0	440053.3	452016.6	410500.2	397774.4	438022.9	440176.9	156786.8	139270.1	170613.8	144607.6
Q3UHQ6	Protein dopey-2	Dop1b	2295	257.3	6.13	126633.9	132700.6	111300.4	122739.8	112508.0	128052.6	122586.9	120339.6	120618.2	119501.3	81129.6	72525.5	75558.1	89727.8
P70279	Surfeit locus protein 6	Surf6	355	41.2	10.52	429006.6	385190.1	338172.0	353237.8	377395.1	432872.6	391882.2	379804.0	379634.4	392141.9	140708.4	152658.2	134446.0	127137.5
Q922E6	FAST kinase domain-containing protein 2, mitochondrial	Fastkd2	689	78.9	8.9	56342.6	53434.2	56347.2	52566.9	55499.9	55670.7	48589.6	46702.9	48774.2	55831.0	131942.7	147432.7	119282.1	135151.1
Q8BNU0	Armadillo repeat-containing protein 6	Armc6	468	50.7	6	103232.5	141444.7	101801.6	114508.7	116673.2	114134.0	99056.1	102038.7	109299.5	112264.1	114509.1	109443.9	106887.9	114836.7
Q923B1	Lariat debranching enzyme	Dbr1	550	62.3	5.22	209433.1	223873.1	226489.9	222704.7	239547.1	258140.1	254375.1	234377.5	235772.9	231592.3	236459.0	257561.7	242777.4	238114.3
Q9D1H6	NADH dehydrogenase [ubiquinone] 1 alpha subcomplex assembly factor 4	Ndufaf4	173	20.1	9.39	238779.7	232568.2	236120.0	252347.0	230683.2	232823.1	242578.6	224181.0	257289.2	242205.9	132063.7	120026.3	125700.7	128823.5
O35379	Multidrug resistance-associated protein 1	Abcc1	1528	171.1	7.36	43757.3	43966.2	38964.5	45105.6	39041.5	42084.8	37664.8	35971.2	40608.9	40091.1	59201.4	64684.5	56283.5	55111.4
Q66JX5	FGFR1 oncogene partner	Fgfr1op	399	42.7	4.81	135330.9	137547.6	104067.3	141711.8	102390.2	115350.4	103487.1	105462.8	104133.6	101893.7	122429.3	102703.7	118718.9	134344.1
Q8BTW3	Exosome complex component MTR3	Exosc6	273	28.4	6.11	79308.0	80679.7	88391.9	68058.9	90105.5	88087.4	82130.5	84728.1	81654.3	84159.6	80467.4	104270.3	90212.9	93431.1
Q80VP0	Tectonin beta-propeller repeat-containing protein 1	Tecpr1	1166	130.2	6.48	116164.1	128725.8	114997.3	132546.1	110256.5	113862.6	110079.8	103362.7	115519.3	106749.5	78230.9	77525.2	78765.7	81803.6
Q6PB93	Polypeptide N-acetylgalactosaminyltransferase 2	Galnt2	570	64.5	8.53	221416.2	206816.8	191466.7	192466.3	213205.1	219810.5	185659.2	229923.2	181563.0	195488.5	195416.0	192477.1	188873.2	179662.0
Q3TIR1	Trafficking protein particle complex subunit 13	Trappc13	417	46.4	5.49	239953.7	277138.6	283450.9	287008.0	283009.9	260854.4	252903.0	248412.6	273814.7	287154.0	210063.2	204592.0	209441.4	207781.2
Q6ZPY2	Protein SMG5	Smg5	1017	114	6	109764.0	115330.8	101977.3	100618.4	114271.6	113400.5	104987.8	101340.9	102267.1	116935.4	139203.7	146493.0	135373.1	142271.7
P01654	Ig kappa chain V-III region PC 2880/PC 1229	-	111	12	5.34	106541.7	131916.7	102189.3	100489.4	103263.7	127794.6	147039.5	98087.3	109788.6	113622.4	#N/A	#N/A	#N/A	#N/A
Q8BH79	Anoctamin-10	Ano10	659	76.1	6.7	73965.1	71885.9	93391.5	77375.9	76530.3	78427.1	85662.4	91562.0	85152.2	77498.3	114241.9	116974.2	110987.0	115646.2
A2RT18	Testis-expressed gene 16	Tex16	1139	127.5	6.38	64534.4	76129.7	67495.9	70857.7	71609.5	77953.3	61971.6	62000.3	72125.3	75411.6	63017.1	55520.6	56391.6	57315.0
Q9CR81	Testis-expressed protein 12	Tex12	123	13.9	5.59	114605.7	170294.4	166251.3	159431.1	152574.1	159561.6	108361.7	135434.7	180721.5	154219.4	147315.8	195449.4	180394.6	188807.8
Q7TMF3	NADH dehydrogenase [ubiquinone] 1 alpha subcomplex subunit 12	Ndufa12	145	17.1	9.36	227673.8	249928.3	233190.7	238930.1	208441.1	220049.4	211931.6	211283.0	223898.0	223417.7	237299.1	229056.9	263934.8	243253.3
Q8CIG3	Lysine-specific histone demethylase 1B	Kdm1b	826	92.6	7.74	78651.5	76721.7	76953.0	75657.6	89402.7	94741.0	80890.5	77648.6	82314.5	87376.4	#N/A	#N/A	#N/A	#N/A
Q9JJT0	RNA 3'-terminal phosphate cyclase-like protein	Rcl1	373	40.8	9.26	265849.9	249754.7	229813.6	236625.2	278800.9	265122.4	231415.8	231030.5	246575.1	275258.1	145693.7	150902.9	150031.5	135174.9
Q9Z2Q6	Septin-5	Septin5	369	42.7	6.67	121410.5	126095.7	130982.6	125578.7	129672.8	149576.0	146074.4	127496.5	127161.0	126475.9	57390.9	59363.5	60109.4	53420.3
P60521	Gamma-aminobutyric acid receptor-associated protein-like 2	Gabarapl2	117	13.7	8.1	634590.8	669309.5	621922.1	682903.0	534861.9	572302.8	535021.0	583385.2	567445.6	593792.5	407705.6	429610.4	447245.0	458636.7
Q9D842	Aprataxin and PNK-like factor	Aplf	499	54.9	5.21	39191.1	41049.5	49484.4	40272.3	50284.8	43922.7	38295.1	37186.3	35287.1	42094.6	99780.2	111142.0	102673.2	108711.9
P56371	Ras-related protein Rab-4A	Rab4a	218	24.4	6.07	114363.9	106393.7	100376.1	109136.2	102439.9	101254.4	107341.1	106823.4	112602.6	110102.8	61969.2	75196.7	68714.5	65535.4
Q9DBB8	Trans-1,2-dihydrobenzene-1,2-diol dehydrogenase	Dhdh	333	36.3	6.44	28658.0	32553.7	28535.8	36933.5	29961.9	30701.7	28656.4	28235.0	30806.7	32971.2	52905.5	48595.9	44328.0	51112.8
Q9CQU5	ZW10 interactor	Zwint	252	28.7	8.43	626766.4	593785.0	663503.0	605863.0	632054.3	594300.2	536605.3	642054.0	614039.8	589957.2	738150.9	675321.8	769544.5	683903.7
Q9JIG7	Coiled-coil domain-containing protein 22	Ccdc22	627	70.8	6.01	222572.7	161033.5	133838.8	148076.2	155147.0	148536.3	153573.6	173636.1	148785.2	134739.0	107674.0	99592.1	100903.3	96124.3
Q8C013	Trophoblast glycoprotein-like	Tpbgl	384	41.1	7.39	127969.4	135476.2	107831.4	135949.2	118820.5	122692.0	112110.1	111979.3	125187.8	138009.6	128535.9	154369.1	111478.4	119690.2
A2CG63	AT-rich interactive domain-containing protein 4B	Arid4b	1314	147.6	5.06	115008.9	122807.4	104506.4	113540.7	110096.4	122178.4	111459.1	108250.5	121793.2	113396.1	47247.3	44092.4	45426.7	38379.6
Q9JHW9	Aldehyde dehydrogenase family 1 member A3	Aldh1a3	512	56.1	6.57	8880.8	8354.4	5390.9	5852.0	7633.5	7559.3	11951.5	8109.0	4412.9	8020.4	12823.0	5824.3	6558.3	8582.7
Q91YD3	mRNA-decapping enzyme 1A	Dcp1a	602	65.2	6.99	155871.1	167920.2	161924.7	181327.0	177570.6	158382.9	150209.7	149714.3	147384.0	150408.7	65714.4	96111.8	93561.8	110086.3
Q5DW34	Histone-lysine N-methyltransferase EHMT1	Ehmt1	1296	141.9	5.8	108759.4	101931.6	110853.7	92573.0	116318.2	118042.8	111011.6	100895.0	102383.0	125941.6	64218.2	65418.0	60514.8	60010.8
Q9DD03	Ras-related protein Rab-13	Rab13	202	22.8	9.5	78986.1	79329.7	84898.1	77383.0	86863.1	76371.4	87752.9	86375.6	74131.1	79938.4	49737.9	51949.7	47977.0	55757.5
Q99LC2	Cleavage stimulation factor subunit 1	Cstf1	431	48.4	6.58	601375.0	591588.5	672444.0	599930.3	692419.6	708310.6	597864.7	668205.4	651196.8	668945.5	189764.7	204433.9	205727.3	203391.9
Q9D735	Telomerase RNA component interacting RNase	Trir	173	18.4	9.67	144330.4	151810.9	135787.7	134785.9	131503.7	123612.2	123284.5	111138.4	144943.0	155899.3	510596.2	534663.6	615995.2	564810.9
A0A1B0GR91	Predicted gene 4779	Gm4779	561	60	8.5	298434.3	320939.7	306377.1	289674.3	341902.4	390526.8	320027.1	276866.5	319265.2	342904.3	160976.6	146381.3	251219.1	150544.2
Q8BKZ9	Pyruvate dehydrogenase protein X component, mitochondrial	Pdhx	501	54	7.75	317817.6	290120.4	274622.5	331110.0	280556.6	253091.9	264595.2	265848.2	281357.6	320955.6	167673.4	145710.8	170070.0	168586.4
D3Z6Q9	Bridging integrator 2	Bin2	489	52.5	5.34	310365.4	323480.9	320897.7	349543.7	291481.0	311536.8	292653.4	306207.0	294990.5	318186.9	155204.1	190048.9	183707.7	199903.2

P55012	Solute carrier family 12 member 2	Slc12a2	1205	131	7.33	59427.3	61939.5	53574.2	56252.7	62266.9	60404.5	53064.7	60003.7	61360.2	60058.1	65112.4	67015.1	57395.8	62942.8
Q8CIP4	MAP/microtubule affinity-regulating kinase 4	Mark4	752	82.6	9.67	72091.9	78799.3	66848.3	64189.5	63823.8	56802.6	62059.2	60822.2	52500.6	66798.3	333915.7	288708.7	290459.9	286688.1
Q99KW3	TRIO and F-actin-binding protein	Triobp	2014	223.2	8.06	129811.9	125848.0	122758.3	125227.1	136333.8	156685.2	150666.8	133912.3	137604.1	121007.3	66533.8	57868.9	68194.8	58872.6
Q9D7P6	Iron-sulfur cluster assembly enzyme ISCU, mitochondrial	Iscu	168	18.1	9.29	249985.4	246245.8	262149.6	261977.5	258531.2	251890.2	249670.5	231253.5	244769.1	247397.8	92817.9	103090.5	83911.5	89452.1
O55055	tRNA (cytosine(38)-C(5))-methyltransferase	Trdmt1	415	46.8	6.7	54461.5	67037.9	55980.5	61155.7	68433.3	67380.5	46817.1	50895.6	59063.1	55118.4	160402.9	169818.9	196937.2	197051.6
Q9CQZ6	NADH dehydrogenase [ubiquinone] 1 beta subcomplex subunit 3	Ndufb3	104	11.7	9.04	77222.4	81823.6	81793.9	80795.0	80609.8	81396.6	87825.0	89137.3	90895.5	91864.2	153845.1	188324.1	182232.6	192605.5
Q9JM13	Rab5 GDP/GTP exchange factor	Rabgef1	491	56.8	6.79	126557.9	143531.7	110657.3	134377.0	118272.5	126260.2	121586.3	116053.0	123585.8	133703.2	150280.1	202591.3	159055.4	193398.8
A1A5B6	TBC1 domain family member 25	Tbc1d25	742	82.5	6.79	90540.1	89598.9	105409.7	96786.4	98830.9	107033.8	104635.6	91488.6	94633.3	102970.4	56263.0	64054.5	49830.4	52987.4
P21447	ATP-dependent translocase ABCB1	Abcb1a	1276	140.6	8.85	229146.4	253284.1	219224.8	239969.0	247103.6	263125.8	247425.4	255728.6	211705.1	240587.7	295482.2	331444.2	304049.2	342722.6
Q8C0Z1	Protein FAM234A	Fam234a	555	60.5	5.68	175396.2	213022.4	181345.1	183910.5	203304.5	214128.0	213163.4	183022.2	200401.2	205963.7	188868.3	200667.4	194323.6	176144.9
Q9D7J9	Enoyl-CoA hydratase domain-containing protein 3, mitochondrial	Echdc3	300	32.4	8.34	233177.9	236130.5	234587.9	223938.6	239719.4	232825.1	218308.6	226983.7	222913.0	234180.1	216214.4	221529.4	227579.4	235681.7
Q8C0V0	Serine/threonine-protein kinase tousled-like 1	Tlk1	766	86.6	8.79	314709.5	357908.6	274448.9	339093.6	333493.3	360190.8	344149.8	315964.1	351918.7	303486.9	#N/A	#N/A	#N/A	#N/A
Q91YK2	Ribosomal RNA processing protein 1 homolog B	Rrp1b	724	80.5	9.72	163149.7	170680.7	168918.6	158677.8	176222.5	182892.8	155404.2	166582.9	185730.4	180522.3	150778.5	169609.5	156018.7	161081.0
Q8BPM2	Mitogen-activated protein kinase kinase kinase kinase 5	Map4k5	847	95	7.83	59381.0	72805.6	56809.9	64896.7	67313.4	65010.1	64315.7	62174.2	55909.6	68319.2	191920.6	214158.0	176325.9	211869.4
Q8BGX2	Mitochondrial import inner membrane translocase subunit Tim29	Timm29	266	29.4	6.73	61143.3	67109.5	67497.2	61133.5	67441.5	63221.7	59798.3	54275.0	53075.9	57906.5	191044.1	214432.8	195201.8	201354.4
O35488	Very long-chain acyl-CoA synthetase	Slc27a2	620	70.4	8.9	32133.8	38578.0	32589.6	31489.1	35421.6	38061.8	26517.6	28969.8	35306.4	34947.8	67615.5	88243.5	74482.8	73762.3
Q8K135	Dyslexia-associated protein KIAA0319-like protein	Kiaa0319l	1048	115.2	6.16	100082.8	109734.8	111216.6	93508.0	107218.2	112304.5	95807.2	97388.3	109166.5	112187.6	96896.5	112902.1	104490.8	103555.2
Q8K194	U4/U6.U5 small nuclear ribonucleoprotein 27 kDa protein	Snrnp27	155	18.9	11.62	324700.6	357007.9	376269.3	344153.3	373232.8	370123.2	324433.7	324700.6	375089.3	350708.5	72756.1	81696.0	86033.4	82151.3
Q9CQC7	NADH dehydrogenase [ubiquinone] 1 beta subcomplex subunit 4	Ndufb4	129	15.1	9.89	72309.3	82405.4	71217.2	78535.3	76872.7	63802.7	65672.9	70832.4	79903.6	78658.7	45987.4	44134.9	59352.9	49888.0
Q9D4H2	GRIP and coiled-coil domain-containing protein 1	Gcc1	778	87.6	5.54	208101.4	244538.1	179947.7	212490.2	214329.1	214623.1	175928.5	198912.6	191114.4	212862.4	197070.9	221011.6	198433.6	214943.8
Q9D7W5	Mediator of RNA polymerase II transcription subunit 8	Med8	268	29.2	6.95	191602.2	203977.5	191958.7	184316.9	194429.8	186468.6	174130.3	175754.8	196909.6	191407.6	218818.3	205177.5	199860.5	211009.5
E9Q5F9	Histone-lysine N-methyltransferase SETD2	Setd2	2537	285.5	6.3	75739.9	87479.7	70614.2	73072.5	87266.1	77594.0	72600.9	71914.1	80426.5	85187.2	129875.3	152657.8	115026.5	139934.1
Q9CQR4	Acyl-coenzyme A thioesterase 13	Acot13	140	15.2	8.82	145189.5	141146.0	127356.8	136859.1	144522.3	138664.0	188341.3	136626.2	142435.5	138909.7	82389.9	115299.4	67775.9	99635.9
Q921G6	Leucine-rich repeat and calponin homology domain-containing protein 4	Lrch4	680	73.1	7.53	11696.1	16881.1	16087.7	14310.9	14088.2	16408.7	16364.8	15035.3	16475.8	17991.8	304482.6	399977.9	337988.5	366640.6
Q9WUB0	RanBP-type and C3HC4-type zinc finger-containing protein 1	Rbck1	508	57.5	6.19	25485.1	28821.0	28789.1	24074.4	30328.5	29840.9	29840.5	24794.2	26693.0	32033.1	98753.5	94416.1	97695.3	99212.1
Q8BGB7	Enolase-phosphatase E1	Enoph1	257	28.6	4.92	244108.7	243829.2	244507.0	244340.4	242703.6	258012.1	263249.6	225293.5	253134.9	280085.5	199939.1	199218.2	202068.0	208874.8
Q91W34	RUS1 family protein C16orf58 homolog	/IGI:238457	466	50.4	7.15	68566.6	66921.4	64742.6	63707.9	61192.1	65271.9	62938.9	66017.0	62468.1	67231.2	43911.6	50285.1	38894.7	44913.8
P62069	Ubiquitin carboxyl-terminal hydrolase 46	Usp46	366	42.4	6.83	215657.2	209139.6	204565.9	198635.9	213271.7	208746.9	204518.6	209025.7	209022.1	211685.8	164596.9	136921.1	142699.4	141562.2
Q99LI5	Zinc finger protein 281	Znf281	893	96.6	8.56	131047.5	149232.3	156988.0	147243.5	151151.2	135313.8	148011.0	128049.0	134141.6	140458.1	#N/A	#N/A	#N/A	#N/A
Q9CZJ1	Probable U3 small nucleolar RNA-associated protein 11	Utp11	253	30.5	10.26	364154.9	337003.6	352782.7	339482.2	374171.3	381460.2	349232.8	344560.3	372821.9	373453.4	155033.3	155690.1	162946.6	148898.2
O09164	Extracellular superoxide dismutase [Cu-Zn]	Sod3	251	27.4	6.84	164208.5	174588.0	148801.0	165317.8	157423.7	162028.3	162071.2	160519.2	168542.9	177918.2	134447.8	127682.1	103559.5	131198.3
P27600	Guanine nucleotide-binding protein subunit alpha-12	Gna12	379	44.1	9.83	132356.2	135675.0	139775.0	122538.3	158438.3	163124.7	146119.2	133671.8	139259.2	130365.4	91590.0	89877.1	88186.9	76357.8
Q9WUV0	Origin recognition complex subunit 5	Orc5	435	50.2	8.06	120542.6	148595.6	141454.3	149245.6	152482.5	149030.9	133012.8	127499.9	150205.5	155989.9	58414.4	64277.6	59034.7	62155.7
Q9D7I5	Phospholysine phosphohistidine inorganic pyrophosphate phosphatase	Lhpp	270	29.1	5.1	119185.7	89391.7	105254.1	87858.3	108326.3	114736.8	114773.9	108008.3	90787.1	83902.4	194854.9	157364.5	173936.9	155018.0
Q08879	Fibulin-1	Fbln1	705	78	5.16	42837.3	39322.9	35076.8	38708.7	33975.2	46173.9	43241.8	41751.2	43732.8	41399.8	33688.9	41160.7	37900.7	37457.6
Q80XI4	Phosphatidylinositol 5-phosphate 4-kinase type-2 beta	Pip4k2b	416	47.3	7.33	167195.6	160405.3	165023.1	152910.0	168551.7	169828.0	172446.1	161219.9	159960.9	165817.5	49293.9	49508.1	50119.1	54027.9
Q8BH60	Golgi-associated PDZ and coiled-coil motif-containing protein	Gopc	463	50.6	6.25	217131.4	237066.0	199080.5	212113.3	192355.6	226137.9	195053.3	176378.8	193232.4	226684.6	146881.8	166892.8	160416.5	152703.6
Q99NB8	Ubiquilin-4	Ubqln4	596	63.5	5.03	42510.0	42357.4	47571.9	38584.5	39818.8	38917.3	36581.3	39984.5	47675.7	34131.4	22362.2	20429.8	21991.5	16427.5
Q9JK42	[Pyruvate dehydrogenase (acetyl-transferring)] kinase isozyme 2, mitochondrial	Pdk2	407	46	6.61	101561.1	107307.5	85621.5	107553.9	111326.5	120796.4	100361.3	98249.7	98885.5	96642.0	82739.9	87783.2	79969.6	79976.0
Q924D0	Reticulon-4-interacting protein 1, mitochondrial	Rtn4ip1	396	43.3	9.2	110864.3	123988.0	120805.9	120122.5	109814.9	114067.4	107262.1	111089.0	128379.7	115107.1	333082.1	338959.3	318491.2	349247.0
O89023	Tripeptidyl-peptidase 1	Tpp1	562	61.3	6.57	80717.0	85168.1	76332.5	77633.8	65981.7	69304.0	64614.9	65239.8	81484.0	67440.2	97137.5	111563.7	95726.2	98845.3
P02798	Metallothionein-2	Mt2	61	6.1	7.83	19002.9	11542.3	12533.4	13677.3	7201.1	12545.1	4228.3	8297.3	8761.9	10240.7	46158.6	52140.7	45754.7	45497.0
Q91X84	CREB-regulated transcription coactivator 3	Crtc3	619	67	6.83	101273.1	112778.8	97805.5	104383.3	114314.1	112782.4	106343.1	96927.1	110737.7	102554.4	#N/A	#N/A	#N/A	#N/A
Q9CQ22	Ragulator complex protein LAMTOR1	Lamtor1	161	17.7	5.15	298990.4	302352.2	294444.0	312038.1	309548.8	299717.1	287718.1	294674.0	306173.5	290450.4	181479.1	199083.4	182956.3	191022.1
P84089	Enhancer of rudimentary homolog	Erh	104	12.3	5.92	106084.4	109103.0	155980.4	134700.1	139576.1	113274.0	115312.0	134409.7	130635.2	115206.5	345968.8	335068.5	392617.5	367412.3
Q810J8	Zinc finger FYVE domain-containing protein 1	Zfyve1	777	86.9	7.34	165589.1	274142.7	177544.6	187177.6	218194.4	254569.5	150724.1	218480.8	184208.2	224083.2	1831904.2	1694939.3	1712239.1	1791318.4
Q921C3	Bromodomain and WD repeat-containing protein 1	Brwd1	2304	259.1	8.4	92812.3	98554.0	103343.2	87359.8	112381.8	106130.1	92867.7	95859.7	95609.4	98775.6	#N/A	#N/A	#N/A	#N/A
Q8VC60	Beta-galactosidase-1-like protein	Glb1l	646	73.2	7.28	117677.3	132412.9	109719.2	129611.1	112395.5	131169.2	112267.3	109771.4	116379.7	126162.0	106374.9	126013.6	115680.9	119032.3
Q3V1T4	Prolyl 3-hydroxylase 1	P3h1	739	83.6	5.14	225253.5	240817.2	200670.4	227289.3	215639.8	231821.2	208794.9	243567.0	214991.8	218803.1	144983.3	150260.6	128407.3	157063.4
Q61133	Glutathione S-transferase theta-2	Gstt2	244	27.6	7.55	104560.4	112477.3	109968.0	105667.5	127456.9	125287.9	141106.1	106280.6	124201.0	121916.7	282551.3	316315.5	266451.6	274057.9
Q99LB2	Dehydrogenase/reductase SDR family member 4	Dhrs4	279	29.9	9.38	243704.2	250274.1	239857.4	278494.7	245965.0	244941.0	242577.2	240236.8	249370.3	255837.1	525322.8	525886.0	448080.5	547992.7
Q9DBG1	Sterol 26-hydroxylase, mitochondrial	Cyp27a1	533	60.7	9.14	238457.6	253263.6	199177.6	235363.6	210510.3	227661.2	212193.6	193662.9	222766.1	200851.5	154895.5	151174.9	130974.1	149740.4
P26883	Peptidyl-prolyl cis-trans isomerase FKBP1A	Fkbp1a	108	11.9	8.16	646972.7	747247.6	619148.3	669101.8	1047974.4	769428.1	682574.9	689901.7	811893.2	737790.1	1109840.3	1194964.0	1113168.2	1116107.5
Q9CQY5	Magnesium transporter protein 1	Magt1	335	37.9	9.76	414561.6	412084.1	366626.6	382798.8	432036.4	425988.8	426316.7	421832.1	385449.6	410121.5	162487.8	140748.2	128285.6	140372.3
Q9CWM4	Prefoldin subunit 1	Pfdn1	122	14.2	8.32	538293.8	598862.3	628176.0	598754.3	586586.8	555565.9	501754.6	586267.4	659985.0	579803.4	552993.9	597983.5	667446.7	588001.2
A2RTL5	Arginine/serine-rich coiled-coil protein 2	Rsrc2	376	43.9	11.46	25586.2	25911.5	27397.0	27890.4	24783.1	32520.0	23785.1	27315.3	29970.0	26681.5	26584.5	25424.0	26573.7	23447.1
P58801	Receptor-interacting serine/threonine-protein kinase 2	Ripk2	539	60.4	7.2	316852.6	330228.8	319856.3	314993.4	325288.7	347887.8	300935.0	299865.5	315838.2	327283.5	189613.1	188988.5	178377.0	169011.4
Q64669	NAD(P)H dehydrogenase [quinone] 1	Nqo1	274	30.9	8.72	435043.0	381994.8	377312.1	429011.2	387405.2	438281.9	572075.2	433952.6	422497.8	429506.8	#N/A	#N/A	#N/A	#N/A
Q9CPS7	RNA-binding protein PNO1	Pno1	248	27.4	9.79	117296.5	139541.7	116800.8	120471.8	128805.3	132581.1	124086.7	112781.8	134787.5	132169.0	68208.0	86319.3	71726.1	79425.3
Q6PAL8	DENN domain-containing protein 5A	Dennd5a	1287	146.6	6.7	39213.7	41881.2	35784.3	37303.5	43068.4	43623.5	41194.3	40017.7	41469.2	39737.6	74252.7	79032.6	70291.4	74654.1
Q9Z207	Protein diaphanous homolog 3	Diaph3	1171	133.6	7.61	51118.8	53937.1	59872.3	57578.7	57769.7	52166.7	53105.8	58928.8	49319.8	54924.4	56805.8	68797.1	66980.2	74855.5
Q9CR98	Protein FAM136A	Fam136a	138	15.7	7.61	202762.3	216935.8	251832.6	220125.6	236650.9	226838.2	224826.0	222961.1	253606.4	226098.6	248410.8	242540.4	265655.0	244015.4
B1B1A0	Lethal(3)malignant brain tumor-like protein 4	L3mbtl4	621	70.9	7.68	135818.5	125996.1	120915.5	132435.7	127984.7	132038.5	125719.5	120473.7	122351.8	131353.7	407427.9	387033.1	387186.7	381994.0
Q99KL7	Ras-related protein Rab-28	Rab28	221	24.7	5.67	149003.7	165674.7	153517.7	153480.2	154831.6	167206.3	134985.6	142323.5	165993.7	155081.7	49983.2	56103.8	55656.0	52046.3
Q62426	Cystatin-B	Cstb	98	11	7.39	103010.6	115632.5	95578.5	101352.3	94323.8	98505.9	103715.8	97686.4	108585.4	121920.2	166220.0	186067.1	168811.9	169838.0
Q99MQ4	Asporin	Aspn	373	42.5	8.57	215866.3	209648.5	185066.6	199193.4	232935.1	266971.6	312148.9	301487.7	212872.5	203921.6	178330.0	167727.9	163423.8	165935.5
Q80WW9	DDRGK domain-containing protein 1	Ddrgk1	315	36	5.35	14673.7	19119.9	16900.0	15779.1	13939.0	14733.5	10705.8	15500.9	16743.1	15969.2	5908.1	8997.1	8795.5	10416.1
Q8R0S1	Cyclic AMP-dependent transcription factor ATF-7	Atf7	413	44.6	8.35	92773.3	88828.7	90440.1	80826.7	95294.5	100811.9	105131.7	83123.1	91071.9	87062.8	48393.8	51643.8	46159.7	38817.5
Q8BNV1	tRNA (uracil-5-)-methyltransferase homolog A	Trmt2a	574	63.3	7.25	133983.1	119189.2	123944.0	123285.4	145839.7	121799.9	121043.8	115663.9	129110.0	151623.2	153133.7	151879.0	154759.7	141873.6
Q99KW9	T-cell immunomodulatory protein	Itfg1	610	67.4	5.73	123703.0	125561.9	124058.7	109077.5	140999.4	129850.9	130476.9	120851.7	124761.0	124067.4	109691.9	110979.4	97841.9	98289.6
Q60990	RNA-binding motif protein, Y chromosome, family 1 member B	Rbmy1b	380	43.1	10.3	381400.2	394683.7	402778.4	368279.9	387633.1	382496.1	355222.9	350880.0	387363.4	440138.9	212931.6	223920.5	217340.2	229227.8
Q8VCY6	U3 small nucleolar RNA-associated protein 6 homolog	Utp6	597	70.4	7.71	265618.1	254642.8	263467.3	267642.3	291097.2	278499.5	237877.1	259994.4	256107.3	265265.4	229282.0	220639.0	280059.5	213853.6
Q3UGS4	Mapk-regulated corepressor-interacting protein 1	Mcrip1	97	11.1	9.14	258890.3	273457.5	275870.3	275186.1	263300.4	255861.3	238032.0	260723.4	271085.6	271352.6	253079.6	236118.4	255766.9	271867.7
Q9CQ48	NudC domain-containing protein 2	Nudcd2	157	17.6	5.07	283279.1	255009.1	272891.5	271160.7	275322.1	257452.9	256596.2	291686.8	292353.3	289806.7	71483.8	66788.5	66196.0	74253.7
Q9Z0V7	Mitochondrial import inner membrane translocase subunit Tim17-B	Timm17b	172	18.3	9.03	26664.2	29994.2	26173.9	25195.8	29114.9	25751.5	29615.9	25100.3	29945.2	29259.1	45854.8	43165.2	41160.8	41844.8
Q8BIV3	Ran-binding protein 6	Ranbp6	1105	124.5	5.03	26981.9	26946.5	27753.8	25232.8	27801.0	26836.2	24749.3	21100.9	23239.9	28181.1	18175.6	20876.2	18435.4	17736.0
Q8JZV9	3-hydroxybutyrate dehydrogenase type 2	Bdh2	245	26.7	7.99	145751.9	139963.3	163038.9	151437.1	177858.4	174827.3	193364.5	163717.7	165448.9	149998.5	105936.6	99645.7	115536.0	94601.9
P0C0A3	Charged multivesicular body protein 6	Chmp6	200	23.4	5.44	42349.5	36923.2	39736.7	35603.2	35499.6	41254.2	40195.6	34599.1	39956.1	40988.9	70148.9	86253.4	80967.0	84485.3
Q8BK35	Ribosome biogenesis protein NOP53	Nop53	484	55.8	10.35	65480.9	68712.4	59951.2	61250.3	61728.7	60844.6	56469.8	54973.9	64429.3	71926.1	98215.3	103619.8	99930.7	100260.3
Q6PGF5	Bms1-type G domain-containing protein	Bms1	1284	145.3	6.27	484896.7	548217.6	558387.6	531097.1	504627.2	503107.4	361338.5	483734.1	470053.5	531897.4	108815.6	109567.7	108192.1	103880.7
A2BH01	Cor1 domain-containing protein	Gm14692	229	26.4	8.79	156580.9	196446.3	156106.3	167573.9	180570.4	171560.0	137459.5	142925.1	159136.5	183235.2	99687.1	122340.3	123294.3	124714.6
Q91YE3	Egl nine homolog 1	Egln1	400	43.1	8.29	42253.3	38756.0	33116.4	36718.6	41162.7	38166.0	39432.8	89773.2	42176.9	36793.3	136682.4	129209.0	129878.5	126135.6
Q8CES0	N-alpha-acetyltransferase 30	Naa30	364	39.4	5.64	83945.1	111442.7	109345.1	99336.6	100304.1	100044.7	92619.5	90391.7	90446.9	108339.0	164886.7	153079.7	153306.5	159144.4
Q9CQF4	Mitochondrial transcription rescue factor 1	Mtres1	240	27.8	9.36	279971.7	297948.0	274472.2	276951.9	273940.6	275189.2	262641.7	256490.7	275663.7	270714.3	110799.7	111661.1	101836.5	115691.6
Q9CWR2	Histone-lysine N-methyltransferase SMYD3	Smyd3	428	49.1	7.08	90943.0	127508.8	90351.7	111513.0	121637.1	110935.5	81349.9	107237.2	104078.4	110509.7	172333.3	181187.1	180731.3	156722.4
Q91VW3	SH3 domain-binding glutamic acid-rich-like protein 3	Sh3bgrl3	93	10.5	5.14	38587.3	34515.0	42703.9	34794.0	33914.7	37936.7	39229.6	40604.5	31624.7	28976.8	49841.7	49634.0	50920.4	44387.4
Q921M4	Golgin subfamily A member 2	Golga2	999	113.2	5	172611.9	199639.3	195067.7	197359.9	170793.6	162324.6	142727.5	160833.4	172987.5	191850.8	100598.3	116329.8	119298.1	121588.7
Q99ML0	Zinc finger MYND domain-containing protein 10	Zmynd10	440	50.6	6.54	126504.5	170811.1	143779.8	167062.5	138024.6	125854.1	106100.2	126417.4	130813.1	140095.1	277623.2	401203.8	344262.2	460366.8
Q810V0	U3 small nucleolar ribonucleoprotein protein MPP10	Mphosph1(	681	78.7	4.87	72643.0	63080.6	61746.0	64555.6	65300.4	69716.7	76534.0	59875.7	70989.3	66562.2	56188.6	46839.2	43773.9	47124.1
Q3UYG1	Coiled-coil domain-containing protein 160	Ccdc160	323	37.4	9.32	682434.2	549538.6	617730.8	526811.1	762066.3	706912.1	741345.3	672498.4	667486.0	636882.3	140491.5	134700.9	141467.0	145330.3
P97287	Induced myeloid leukemia cell differentiation protein Mcl-1 homolog	Mcl1	331	35.2	6.2	181272.5	213268.0	168754.7	179340.2	223207.1	205293.0	212312.9	198449.0	216813.5	199575.9	13305.0	15169.7	16089.6	13562.0
Q6P9Q4	FH1/FH2 domain-containing protein 1	Fhod1	1197	129.5	6.28	51091.3	48592.2	53015.0	53120.5	56282.6	55672.2	55875.5	57790.4	45649.3	51670.0	97966.8	117529.1	107151.5	113092.7

Q8R4Y4	Stabilin-1	Stab1	2571	276.1	6.65	240724.1	278793.2	188669.9	256138.7	210031.5	224830.3	200400.7	207304.1	207146.0	222525.2	106449.0	123688.7	100571.9	126041.8
Q9CQ71	Replication protein A 14 kDa subunit	Rpa3	121	13.6	4.84	335861.9	386979.0	386292.4	379431.1	353679.3	344333.5	325052.4	338717.2	363150.0	322074.2	123758.9	126713.4	114866.6	123875.5
Q8VEE1	LIM and cysteine-rich domains protein 1	Lmcd1	365	41	7.93	50615.9	69412.8	46748.1	66187.0	45111.3	47565.1	35739.3	46479.2	50098.4	50831.5	175206.5	203978.5	136455.4	178814.1
Q9CR02	Translation machinery-associated protein 16	Tma16	221	25.8	9.19	387973.7	369895.7	312171.3	383608.4	419173.6	378882.7	327808.2	300852.0	341870.9	366656.1	443046.7	517481.3	468979.3	498104.7
Q9ESN9	C-Jun-amino-terminal kinase-interacting protein 3	Mapk8ip3	1337	147.5	5.45	69880.0	81992.0	66186.3	81551.9	73554.0	67451.7	64919.8	66397.3	68741.2	73213.2	87046.1	98998.3	87085.5	90582.9
Q3TE80	RIKEN cDNA 2410002F23 gene	10002F23I	284	32.4	6.32	365967.2	339599.3	321598.7	343756.5	374546.5	328444.1	338893.7	343306.9	364427.7	357957.9	438103.0	460547.4	425314.2	497212.4
Q9JJF3	Ribosomal oxygenase 1	Riox1	603	67.5	8.66	127930.5	164104.9	137455.0	150878.0	144795.4	148581.1	130779.8	138418.4	136737.3	155206.1	149995.8	137938.2	143880.9	144945.0
A2ACQ1	DEAH (Asp-Glu-Ala-His) box polypeptide 35	Dhx35	703	78.7	8.35	68144.2	79860.2	85032.5	87302.3	73885.7	63532.0	58510.5	67232.2	75787.2	71711.3	35237.3	33065.2	36647.5	35826.0
Q9QZH6	Evolutionarily conserved signaling intermediate in Toll pathway, mitochondrial	Ecsit	435	49.8	6.6	97545.3	120015.0	101527.4	101438.0	107082.0	110430.8	93096.4	101504.3	98318.8	100565.4	70807.1	64945.2	67395.6	67518.1
Q9D1R2	Protein KTI12 homolog	Kti12	351	38.4	6.92	57878.2	65629.8	56949.4	61439.5	61471.6	57214.2	57419.2	61637.4	55487.4	64815.6	100947.3	113355.0	98050.8	104184.9
Q8CFI0	E3 ubiquitin-protein ligase NEDD4-like	Nedd4l	1004	115.3	6.15	126864.6	133176.0	105983.9	127668.2	123146.0	122246.4	127105.2	110885.0	116730.7	132903.7	55606.2	61590.0	61101.1	62653.1
Q9DAS9	Guanine nucleotide-binding protein G(I)/G(S)/G(O) subunit gamma-12	Gng12	72	8	8.97	241715.3	250756.3	255264.0	218209.4	277638.6	272801.7	304296.7	265741.3	287983.0	261419.2	101813.4	109328.8	111417.5	104712.6
Q04735	Cyclin-dependent kinase 16	Cdk16	496	55.9	7.87	183055.4	179618.5	154658.7	167639.7	172928.3	159707.7	168154.4	158370.6	155043.3	168298.1	222188.9	229615.5	225264.6	224949.9
Q9D4F8	Gamma-tubulin complex component 4	Tubgcp4	667	76.1	6.65	191531.0	171462.2	184647.7	169177.2	186383.6	173691.3	178892.3	178954.6	181095.9	195311.2	92534.2	86594.4	80742.8	89180.6
Q3UFM5	Nucleolar MIF4G domain-containing protein 1	Nom1	854	95.9	8.87	506286.7	493340.1	532176.1	464924.4	543828.1	515486.3	457475.6	493994.2	490289.5	505720.0	164860.3	177893.5	172761.9	166658.3
Q8CE46	Pseudouridylate synthase 7 homolog-like protein	Pus7l	702	79.1	7.81	116386.6	117044.5	103856.0	107503.6	113733.2	115607.0	109453.4	111225.7	110048.0	111483.1	11949.2	11226.5	11110.0	8637.6
Q9D1J1	Adaptin ear-binding coat-associated protein 2	Necap2	266	28.6	7.94	304335.4	229745.5	234320.7	238275.5	214680.2	191271.4	198452.3	200071.2	196565.7	209009.0	115109.2	113364.7	108531.7	106338.5
Q8BP56	Protein-glucosylgalactosylhydroxylysine glucosidase	Pgghg	690	76.4	5.33	167818.7	143898.6	151902.3	147241.3	180397.2	188974.7	265669.7	177574.7	175093.3	164938.8	148687.9	140617.4	138089.8	135073.3
O35071	Kinesin-like protein KIF1C	Kif1c	1100	122.4	7.03	13676.7	11934.9	13185.2	12110.3	12094.1	13014.5	11128.9	13000.0	12088.6	10330.2	#N/A	#N/A	#N/A	#N/A
P70236	Dual specificity mitogen-activated protein kinase kinase 6	Map2k6	334	37.4	7.39	148185.0	151789.2	155400.6	146362.6	148127.4	158344.9	126542.6	143789.3	146597.8	157271.7	111144.4	96582.6	99662.5	97377.9
Q8BRG8	Transmembrane protein 209	Tmem209	561	62.9	8.73	43328.4	38381.4	50858.8	40512.3	45506.1	54542.6	49088.3	45959.7	42934.9	38577.3	45389.8	39287.9	46843.2	41516.6
P52623	Uridine-cytidine kinase 1	Uck1	277	31	7.88	199762.0	177773.0	184497.2	171140.7	229322.5	244155.7	271377.9	199605.1	212947.3	197619.0	303444.9	297200.5	320946.2	279074.0
P62313	U6 snRNA-associated Sm-like protein LSm6	Lsm6	80	9.1	9.58	783991.6	837169.5	902716.9	855292.7	934531.2	944110.5	906943.4	862002.4	968721.0	895933.5	1044847.8	1742573.9	1530120.3	1889529.2
Q9D0R8	Protein LSM12 homolog	Lsm12	195	21.7	7.74	415701.3	512214.7	475619.4	492907.5	494319.8	454287.9	462549.2	431384.6	497050.4	487015.5	485432.7	528594.0	543614.8	564833.6
Q00547	Hyaluronan mediated motility receptor	Hmmr	794	91.7	5.52	185449.8	182660.1	172305.0	225809.5	171922.2	157972.7	129922.0	124717.4	229941.3	182513.1	89949.6	105084.9	101262.8	95538.6
Q9QZ05	eIF-2-alpha kinase GCN2	Eif2ak4	1648	186.4	6.32	81513.1	78911.0	76784.0	69587.6	81246.2	88129.2	84882.1	78833.6	76425.4	87354.9	253464.4	271737.9	264440.2	250702.1
P79568	Ig-like domain-containing protein	H2-Q6	326	37.4	6.24	82685.3	86891.9	76843.3	77200.0	87105.1	87770.4	86424.2	81634.6	82624.4	79310.3	#N/A	#N/A	#N/A	#N/A
E9Q4Y8	RIKEN cDNA 8030474K03 gene	30474K03I	554	58.7	6.55	142178.6	126438.9	122735.7	126727.1	125821.1	125112.9	151274.4	123671.7	120706.0	137594.1	231116.6	258014.5	249736.6	235646.8
Q9WTN0	Geranylgeranyl pyrophosphate synthase	Ggps1	300	34.7	6.46	223593.6	272046.1	226232.1	253091.3	262240.0	258716.0	248337.5	221975.9	240170.0	240871.5	528857.8	570868.4	491041.5	579541.2
Q8CGB3	Uveal autoantigen with coiled-coil domains and ankyrin repeats	Uaca	1411	160.7	7.2	67692.1	63144.6	67780.2	58113.2	85333.6	80492.3	88054.1	77821.9	75835.2	66693.4	65379.5	69743.7	69343.3	61024.1
Q91YN1	Protein FAM118A	Fam118a	357	40.4	6.25	38590.0	48551.0	44277.2	43106.1	41797.1	44479.6	36113.0	36093.4	47355.0	49579.5	57116.2	64106.9	54139.8	60529.9
Q6PER3	Microtubule-associated protein RP/EB family member 3	Mapre3	281	31.9	5.54	47290.9	54199.2	49472.7	49037.4	50236.4	48397.0	42831.1	46094.5	52576.0	52336.1	55968.8	48933.7	50598.3	66049.5
P55772	Ectonucleoside triphosphate diphosphohydrolase 1	Entpd1	510	57.2	5.86	80124.6	91467.1	78790.2	91165.4	85992.7	106036.3	117887.6	91634.3	82028.3	85591.5	168813.4	187354.5	147031.3	172786.1
Q5NCE8	Magnesium transporter MRS2 homolog, mitochondrial	Mrs2	434	49.3	7.53	80280.2	90217.5	75976.1	85778.0	72429.9	72561.0	67100.0	74815.9	78906.4	85939.8	67650.5	72404.8	62339.8	64174.8
Q6NV99	HAUS6_N domain-containing protein	Haus6	933	105.5	6.67	134142.8	145143.2	156001.2	157296.5	174721.8	164207.8	129169.0	134256.6	146921.9	160914.1	205279.1	217501.7	165506.0	202320.3
Q99KR3	Endoribonuclease LACTB2	Lactb2	288	32.7	6.33	316976.4	361503.0	344435.1	366818.4	348659.9	378278.8	404530.8	328036.0	350483.4	377231.8	312196.0	336121.5	310921.7	333173.1
P17095	High mobility group protein HMG-I/HMG-Y	Hmga1	107	11.6	10.32	139102.9	203454.4	178146.3	178489.5	173587.9	159348.5	181222.4	143253.6	204873.0	213093.6	116401.9	139648.0	123199.0	132691.9
Q8R2U6	Diphosphoinositol polyphosphate phosphohydrolase 2	Nudt4	179	20.1	6.35	63006.7	62800.6	63603.9	55691.4	64579.4	69867.0	60149.1	60695.1	57513.2	59999.9	133308.6	143431.0	138150.0	146986.2
Q3U7U3	F-box only protein 7	Fbxo7	523	57.6	5.83	401211.5	407947.6	396012.3	399665.2	405142.1	408976.2	376394.0	363130.9	408704.5	402470.2	17184.9	18526.2	23259.8	22083.8
O70274	Protein tyrosine phosphatase type IVA 2	Ptp4a2	167	19.1	8.37	209594.3	193079.5	214379.2	211820.4	223889.2	239550.7	239492.5	217194.4	206541.4	191646.7	126980.9	150016.1	124618.9	129394.1
Q9Z172	Small ubiquitin-related modifier 3	Sumo3	110	12.4	5.96	399255.7	354038.5	446697.6	366416.5	424445.6	454011.8	483982.6	427502.1	434260.2	396248.3	1167294.8	975072.2	1227984.2	1066724.6
Q8BIH0	Histone deacetylase complex subunit SAP130	Sap130	1057	111.2	9.91	85887.9	89946.4	87539.4	90512.3	87612.0	96844.2	88521.7	86629.7	86092.5	94090.1	82326.0	80357.2	83645.5	79651.9
Q9DCJ1	Target of rapamycin complex subunit LST8	Mlst8	326	35.8	5.86	57967.2	66445.8	53510.4	64060.5	62100.8	56726.0	55818.0	57627.8	55791.8	61195.1	32967.6	39142.9	32531.3	31832.2
Q9QYY0	GRB2-associated-binding protein 1	Gab1	695	76.8	5.67	31018.4	30454.7	33556.0	27987.8	37010.8	40590.4	38395.3	30918.2	33582.3	30855.0	45934.3	46247.1	44290.0	42464.9
Q91V36	Nuclear receptor-binding protein 2	Nrbp2	499	57.3	6.35	79238.1	78276.6	74972.9	75682.0	81577.5	86183.6	86350.4	76553.1	80009.1	82753.1	234927.2	282450.2	246937.0	235227.9
Q61072	Disintegrin and metalloproteinase domain-containing protein 9	Adam9	845	92	7.47	38172.6	43111.2	35420.7	39816.1	40360.6	41320.5	37275.9	45051.4	41002.3	43938.7	44231.6	38197.4	46326.7	45180.3
Q80ZI6	E3 ubiquitin-protein ligase LRSAM1	Lrsam1	727	83.9	6.14	102343.6	107292.6	93725.4	96984.1	102464.3	97479.8	103595.2	93976.5	96291.4	103839.1	93402.4	90764.5	85612.2	91202.3
Q6ZWV7	60S ribosomal protein L35	Rpl35	123	14.5	11.05	1162954.4	1269983.4	1369116.9	1235246.6	1185049.9	1058066.2	964544.6	1235088.5	1165732.6	1137139.2	672893.5	753933.0	883896.7	741662.2
Q8VE18	Protein SMG8	Smg8	991	109.6	7.77	53138.1	63617.5	53903.9	54865.7	56303.7	59701.8	50682.3	56815.3	53857.4	59093.4	57426.8	79665.0	80063.3	78813.7
Q3TJB1	Zinc finger, MYM domain-containing 1	Zmym1	983	110.3	6.42	191152.2	201378.6	208696.5	199565.3	212173.5	203968.2	168891.4	169868.6	208105.5	215183.8	215918.2	234997.6	256112.1	257442.5
Q9D8M7	PHD finger protein 10	Phf10	497	55.8	6.61	129545.3	159700.3	166093.5	165876.3	146275.7	159444.1	147868.8	140146.2	144542.7	155126.0	#N/A	#N/A	#N/A	#N/A
P0C027	Diphosphoinositol polyphosphate phosphohydrolase 3-alpha	Nudt10	164	18.6	5.69	184886.9	167691.3	178242.3	161696.1	184669.0	182192.1	167125.8	202265.9	171526.8	163196.7	133677.6	148726.2	134663.2	137832.4
Q3TXU5	Deoxyhypusine synthase	Dhps	369	40.6	5.67	82822.4	73328.1	76353.7	73966.6	96041.1	86631.3	81137.6	74769.9	87470.4	82772.2	177635.6	177137.0	194262.5	169945.0
Q6PD19	Armadillo-like helical domain-containing protein 3	Armh3	689	78.6	6.6	152139.6	160995.0	161914.7	162459.7	160705.8	168283.6	157111.2	147438.3	164199.4	171193.2	295026.2	304112.1	250276.8	258043.1
Q6ZQ03	Formin-binding protein 4	Fnbp4	1031	111.2	4.73	60432.5	72083.8	65693.4	65698.0	93176.8	75983.6	64981.3	66369.9	74694.4	73071.9	154399.7	185872.8	162078.9	177059.0
Q8R2W9	Pantothenate kinase 3	Pank3	370	41.1	6.55	31937.0	37594.5	31964.4	36461.2	33520.5	35039.5	30171.1	30238.2	33723.6	31308.0	48894.4	54999.1	46205.4	47424.0
Q4VC33	E3 ubiquitin-protein transferase MAEA	Maea	396	45.3	8.69	157343.0	200224.8	166824.8	193441.6	172741.4	151723.3	174290.0	157021.0	177906.4	170859.1	126789.0	150660.0	133886.0	135659.8
Q8C079	Striatin-interacting protein 1	Strip1	837	95.5	6.25	39442.1	48992.5	41407.2	44456.7	44681.2	44604.6	39532.6	39681.7	43240.4	41641.5	47593.3	54670.8	50897.1	53475.4
Q9D8C9	Purine nucleoside phosphorylase	Pnp2	305	33.9	5.81	278082.4	297937.6	302878.6	1192634.7	824431.9	336677.4	245459.3	274629.0	288581.5	334028.2	#N/A	#N/A	#N/A	#N/A
Q8C522	Endonuclease domain-containing 1 protein	Endod1	501	55.2	6.16	20453.9	18639.6	18924.7	18054.2	19715.6	19439.4	16410.4	20918.0	19476.5	17133.9	144152.2	148401.3	137057.9	139336.8
O54946	DnaJ homolog subfamily B member 6	Dnajb6	365	39.8	9.36	245647.6	271468.3	296843.3	280542.3	259783.5	250499.5	262698.9	221350.4	271101.1	323811.9	262977.5	319068.5	321132.4	353826.6
Q8K4L0	ATP-dependent RNA helicase DDX54	Ddx54	874	97.7	10.01	100434.8	144868.7	103837.9	105340.6	129399.4	154545.5	85443.2	124894.9	102976.0	114987.3	118286.0	154904.3	124857.6	124625.0
Q64337	Sequestosome-1	Sqstm1	442	48.1	5.21	58306.5	64767.7	62432.8	62815.3	54555.8	60706.4	52879.7	49293.4	55916.8	63807.2	26592.0	30363.6	35206.5	34791.0
Q9D6X5	Solute carrier family 52, riboflavin transporter, member 3	Slc52a3	460	49.5	6.68	538655.0	559495.4	504230.8	493232.9	586626.0	472921.8	510395.2	522693.4	580450.6	515794.5	439282.7	412714.8	400024.2	353714.8
O55013	Trafficking protein particle complex subunit 3	Trappc3	180	20.3	4.96	222514.9	264663.3	244448.2	274233.0	265967.3	262320.6	236540.2	259217.3	251172.4	242456.7	229207.9	261154.8	252800.1	270324.4
Q91ZM2	SH2B adapter protein 1	Sh2b1	756	79.6	5.25	73307.5	80303.6	77686.3	67078.3	83406.3	83635.5	68824.0	64265.2	79996.7	84940.1	65327.5	79579.5	72370.1	69548.3
Q9ER58	Testican-2	Spock2	423	46.8	4.87	217937.5	147628.8	170594.2	122488.0	158606.3	131079.6	173486.9	174072.9	142587.0	149364.1	29228.3	39642.0	40101.0	40104.7
Q9CPQ3	Mitochondrial import receptor subunit TOM22 homolog	Tomm22	142	15.5	4.34	51695.5	75093.4	59139.6	71145.8	63503.5	65898.0	54331.4	70505.7	67180.2	67709.0	42120.2	51554.3	44675.5	54461.2
Q7TML3	Solute carrier family 35 member F2	Slc35f2	375	41.6	5.74	108527.3	122164.0	107521.0	138811.0	104828.3	103613.7	115796.1	120604.5	116841.7	111339.1	#N/A	#N/A	#N/A	#N/A
E9Q5K9	YTH domain-containing protein 1	Ythdc1	736	85.6	6.16	214629.3	236307.0	254311.1	235391.6	232934.8	237202.1	229313.5	236937.4	206467.3	239914.4	114577.6	138709.5	131741.1	135589.1
Q99K95	Replication termination factor 2	Rtf2	307	33.9	8.81	14850.9	21009.9	19931.4	17659.6	16566.6	22542.2	19356.5	19303.7	19244.6	17199.5	45482.9	47328.9	36828.2	39540.2
Q6NSU3	Glycosyltransferase 8 domain-containing protein 1	Glt8d1	371	42	9.41	188819.2	201938.6	218202.5	199377.5	186842.6	154566.9	154040.0	161960.6	176160.7	203647.7	390356.7	452210.6	443934.1	468798.5
Q9D6K9	Ceramide synthase 5	Cers5	414	48.1	8.07	480841.6	511038.0	449922.3	467441.6	477598.3	448536.8	381444.7	482845.4	479643.4	481928.5	482598.1	384434.9	417588.4	427598.9
Q9D7Z3	Nucleolar protein 7	Nol7	254	29	9.61	169384.6	185829.8	157212.0	165096.3	218797.1	205408.8	197207.2	199387.5	183942.7	202629.5	158991.0	158756.4	176455.0	160609.2
Q8VCN5	Cystathionine gamma-lyase	Cth	398	43.5	7.65	134919.4	136771.8	106652.5	135480.3	121546.8	116445.1	106728.7	103845.3	125093.8	132666.0	110474.4	112374.2	92240.1	110296.1
Q921Y2	U3 small nucleolar ribonucleoprotein protein IMP3	Imp3	184	21.8	9.5	92659.3	93504.9	80999.3	81506.1	94882.9	96944.6	94396.6	93911.3	84542.3	101776.5	269764.9	274530.3	255775.9	240907.7
Q3TYX3	SET and MYND domain-containing protein 5	Smyd5	416	47.1	5.21	144313.8	144895.9	155353.8	152708.7	143290.0	120027.3	148017.4	123445.6	150518.0	152644.6	392073.9	411640.6	349529.7	410152.4
Q9WVG6	Histone-arginine methyltransferase CARM1	Carm1	608	65.8	6.77	263516.9	267917.7	228585.7	244631.7	234930.9	278728.8	250499.2	260687.7	247522.0	237645.8	215361.5	227917.8	212474.0	223392.5
Q60751	Insulin-like growth factor 1 receptor	Igf1r	1373	155.7	5.74	81946.7	79277.9	76917.5	71207.5	94112.4	91889.8	99301.6	78838.2	81564.9	81459.9	65730.7	72753.8	66335.8	61513.5
Q9R0Q1	Synaptotagmin-like protein 4	Sytl4	673	76	8.84	95518.3	88552.8	114579.6	90574.9	119425.5	114881.6	111950.4	118118.6	108181.6	108177.9	199846.9	198170.6	185654.2	191622.3
Q8BIW9	Chromosome transmission fidelity protein 18 homolog	Chtf18	969	108.1	6.8	54798.0	59598.5	53836.8	59806.6	56553.3	56747.0	52763.4	50326.9	50987.2	54562.4	117277.2	147995.8	97902.3	115885.0
A1L314	Macrophage-expressed gene 1 protein	Mpeg1	713	78.3	5.92	45946.4	76133.8	43280.8	56155.5	67031.9	50828.4	45611.1	51925.5	56211.1	57118.9	38183.4	42112.6	43138.4	46573.0
Q62186	Translocon-associated protein subunit delta	Ssr4	172	18.9	5.78	196844.8	203402.6	190494.8	213235.1	190409.4	190272.6	240770.9	217921.3	206653.1	204358.9	286445.8	327062.7	272270.1	303237.8
O08997	Copper transport protein ATOX1	Atox1	68	7.3	6.51	1863602.6	2179670.4	1780496.6	2302608.9	1493553.4	1352482.9	1408089.4	1804996.9	1805849.1	1677694.6	2161308.0	2438118.4	2524357.3	2739123.0
Q921V5	Alpha-1,6-mannosyl-glycoprotein 2-beta-N-acetylglucosaminyltransferase	Mgat2	442	51	8.59	66470.2	65061.4	72641.8	61105.4	59033.3	72705.2	62575.8	60129.6	56907.7	55060.3	344041.6	336731.6	362139.5	363059.0
Q9DCT1	1,5-anhydro-D-fructose reductase	Akr1e2	301	34.4	7.33	740218.2	763376.6	698063.5	819472.9	674997.9	682400.8	690432.7	662347.1	764853.6	825836.8	819425.1	807458.6	689603.2	1002957.7
Q80Y14	Glutaredoxin-related protein 5, mitochondrial	Glrx5	152	16.3	6.55	104906.6	107183.7	129035.2	130355.0	128742.2	110764.7	97720.8	124140.4	119270.3	106815.6	118545.7	147467.5	132156.1	141420.4
Q3TZM9	GDP-Man:Man(3)GlcNAc(2)-PP-Dol alpha-1,2-mannosyltransferase	Alg11	492	55.2	8.29	101840.6	114059.1	103128.7	111518.6	105398.6	97826.5	101826.1	95651.3	102910.4	111740.5	563290.8	620279.1	587333.2	621612.5
Q8C163	Nuclease EXOG, mitochondrial	Exog	368	41.4	8.12	94092.5	100955.0	88037.8	100468.9	96821.0	104956.0	99186.3	96246.0	94378.2	89506.5	63669.7	70751.5	52998.4	64246.4
Q60592	Microtubule-associated serine/threonine-protein kinase 2	Mast2	1734	190.4	7.74	101552.9	93654.3	103606.1	99471.9	103371.3	96810.2	96435.2	92669.2	100254.6	98328.1	156403.2	158939.6	139669.9	148035.2
P61079	Ubiquitin-conjugating enzyme E2 D3	Ube2d3	147	16.7	7.8	67543.7	109365.4	73069.4	108658.4	79280.4	71121.1	84707.0	69497.4	93837.4	102297.2	#N/A	#N/A	#N/A	#N/A
O08532	Voltage-dependent calcium channel subunit alpha-2/delta-1	Cacna2d1	1103	124.6	5.3	30548.8	36006.8	34713.5	29673.1	41391.3	45152.1	49411.8	45902.8	39427.0	37507.6	#N/A	#N/A	#N/A	#N/A

Q8BHE8	m-AAA protease-interacting protein 1, mitochondrial	Maip1	291	33	9.14	312507.3	317867.5	337023.3	344317.1	349346.4	336939.8	358546.4	329966.6	362388.8	325153.5	236978.9	236383.2	266492.9	272003.8
Q8BJF9	Charged multivesicular body protein 2b	Chmp2b	213	23.9	8.78	1016532.7	972338.8	862027.6	877615.2	653923.7	802421.7	1048475.2	1012867.7	902716.2	882287.9	548434.1	620131.0	665136.8	726827.9
Q9D6L8	Peptidyl-prolyl cis-trans isomerase-like 3	Ppil3	161	18.1	6.79	210068.4	184104.8	192470.2	182990.5	199094.8	203298.0	180074.8	262986.2	210828.1	193749.2	257739.3	262799.6	282072.8	280555.5
P62307	Small nuclear ribonucleoprotein F	Snrpf	86	9.7	4.67	596529.7	647519.7	680623.9	689717.0	653676.4	607899.1	623291.8	671456.8	625106.7	625447.1	262466.5	266769.5	290533.1	289349.5
Q9D198	Pre-mRNA-splicing factor SYF2	Syf2	242	28.7	8.66	161030.7	173325.1	157613.6	163241.8	181634.5	203938.3	163319.2	154678.6	165520.9	186791.9	133604.0	155545.8	145402.6	151275.4
Q9QZS3	Protein numb homolog	Numb	653	70.8	8.65	110078.9	109808.7	124873.8	110756.4	127044.2	133310.9	116984.2	119518.4	116724.6	120777.9	41463.1	58358.2	56273.7	53820.4
Q8CJ53	Cdc42-interacting protein 4	Trip10	603	68.4	5.9	92350.8	91688.4	98886.5	95879.1	103300.3	100277.1	101021.9	103691.8	91555.6	98176.3	59435.1	57566.7	58878.5	50012.7
Q9DCT5	Stromal cell-derived factor 2	Sdf2	211	23.1	7.33	80610.4	75362.7	80064.2	83941.7	77532.0	76462.1	74297.3	87317.9	66533.5	68706.2	271603.2	242480.9	216581.8	245310.9
Q61249	Immunoglobulin-binding protein 1	Igbp1	340	38.9	6.18	155255.2	158450.0	140550.0	161223.3	137071.9	149137.9	145231.1	145102.9	151875.5	152911.4	153495.2	166498.8	154894.1	163905.1
Q8VI84	Nucleolar complex protein 3 homolog	Noc3l	807	93.2	9.09	95910.4	98766.0	96158.6	93720.9	106084.9	110587.8	96646.0	96670.6	94355.8	102480.3	92724.6	114227.6	95382.9	118812.1
Q91W36	Ubiquitin carboxyl-terminal hydrolase 3	Usp3	520	58.8	8.28	133705.9	129592.5	138537.7	111401.0	140733.4	168853.4	121509.9	125228.7	134075.3	144303.9	75041.5	82975.2	89459.7	74668.0
Q99KR7	Peptidyl-prolyl cis-trans isomerase F, mitochondrial	Ppif	206	21.7	9.16	1655993.9	1709808.0	1509711.6	1756020.1	1609907.9	1610569.3	1820074.0	1419475.9	1878448.1	1866272.1	445208.5	487786.8	414592.5	454099.0
Q9CQK7	RWD domain-containing protein 1	Rwdd1	243	27.8	4.26	220293.7	220231.9	211804.8	217296.9	229512.9	216123.5	203415.3	207801.2	242910.8	238796.1	496486.9	467341.5	473248.2	451235.6
Q6P5G3	MBT domain-containing protein 1	Mbtd1	631	70.6	7.77	107791.8	145138.7	102446.8	134546.0	123036.5	123822.2	80066.8	105330.9	121871.4	132238.0	250384.2	259898.3	231655.0	242594.3
P28184	Metallothionein-3	Mt3	68	7	7.47	165163.0	95963.9	104582.7	116912.1	133864.0	171955.3	163213.6	157422.3	113666.1	112364.6	522528.1	301579.8	358048.2	323641.5
Q8VCS3	Glycosaminoglycan xylosylkinase	Fam20b	409	46.6	6.95	125065.6	127424.3	106738.6	119297.0	109894.8	113125.9	114048.4	112389.0	109826.3	112879.5	146591.8	179225.9	145405.9	180253.3
O35239	Tyrosine-protein phosphatase non-receptor type 9	Ptpn9	593	67.9	8.1	91415.2	86629.4	85222.8	86249.6	82803.8	87137.2	91057.0	85143.3	86913.7	85929.3	441145.1	420736.2	382338.7	399229.7
Q9DCR2	AP-3 complex subunit sigma-1	Ap3s1	193	21.7	5.39	412439.4	414086.5	378715.8	407206.6	382403.6	373616.2	316284.4	342340.9	388756.6	385421.6	257801.5	257473.0	244365.6	246633.8
P12804	Fibroleukin	Fgl2	432	48.9	7.25	105651.0	111622.3	86303.0	104502.9	113927.1	116432.8	144157.4	87933.6	105070.4	94841.7	92180.5	82097.0	82657.7	99825.2
Q8BR92	Paralemmin-2	Palm2	376	42.1	5.15	100129.7	92105.9	116199.3	93970.1	122346.8	122506.5	110680.9	114441.4	118308.0	109485.8	192136.4	192794.7	206886.3	187428.4
Q3ULF4	Paraplegin	Spg7	781	85.9	9.04	89538.4	88822.7	85002.6	84651.3	91784.7	82865.1	88754.1	82772.6	96071.5	100914.4	68636.4	67129.0	69261.9	66613.8
Q8BL95	Cilia- and flagella-associated protein 298	Cfap298	290	33.2	6.67	104346.3	124088.7	110981.5	133042.3	91024.3	94148.0	80269.2	88503.7	104035.2	108938.1	124378.1	146823.3	122933.7	141517.3
P16330	2',3'-cyclic-nucleotide 3'-phosphodiesterase	Cnp	420	47.1	8.97	261746.3	267470.6	248707.9	247919.3	292518.2	294327.5	297154.3	263038.3	271006.5	271348.9	376868.0	330586.3	329625.5	328025.6
O09174	Alpha-methylacyl-CoA racemase	Amacr	381	41.7	7.4	469202.4	454906.3	446441.2	474134.4	480881.1	573243.2	534991.3	441867.1	513996.7	475563.4	220721.8	226649.6	226310.1	222960.7
Q91YM4	FAST kinase domain-containing protein 4	Tbrg4	630	71.5	8.34	227174.2	229445.4	247430.9	221242.5	249947.3	235030.1	217463.4	218954.5	235663.5	245339.5	107874.1	130251.8	109812.5	115027.5
Q62073	Mitogen-activated protein kinase kinase kinase 7	Map3k7	579	64.2	6.67	89265.5	95372.1	88010.1	91962.5	87909.6	85863.9	83235.6	80828.6	81283.1	100976.0	95790.9	112771.6	98215.5	105671.6
Q80U78	Pumilio homolog 1	Pum1	1189	126.5	6.86	66887.6	65719.4	73254.7	65462.3	76093.0	76726.9	71284.8	59893.9	69375.0	69283.0	242903.3	247228.6	228069.0	228686.0
G3XA57	Rab11 family-interacting protein 2	Rab11fip2	512	58.2	9.45	84299.3	88830.1	87473.4	75464.2	92306.5	83341.0	94958.0	81438.3	89272.8	90177.4	95992.9	102137.4	91810.1	96172.7
Q9D903	Probable rRNA-processing protein EBP2	Ebna1bp2	306	34.7	10.08	267909.7	338725.5	330245.2	360164.8	280453.6	234129.6	223420.8	240726.7	254707.4	294463.9	303845.3	331925.5	283730.3	297522.5
Q7TSY8	Shugoshin 2	Sgo2	1164	130.2	8.76	84008.8	114443.9	97438.3	95501.2	83502.9	71664.1	58693.2	73188.4	92858.5	95063.6	91250.8	110865.6	115374.8	111539.9
Q91UZ5	Inositol monophosphatase 2	Impa2	290	31.7	6.23	77170.5	86983.0	83167.4	78992.9	98349.2	98967.6	93804.4	93052.0	89060.4	91239.1	255778.1	270790.9	258470.0	257533.9
E9Q6E5	RRM domain-containing protein	Srsf11	511	56.7	10.7	231692.6	245502.9	265140.4	286273.2	236651.7	219546.5	227499.7	208858.6	235220.2	230981.6	168776.7	180303.5	172408.8	186039.5
Q61390	T-complex protein 1 subunit zeta-2	Cct6b	531	58.1	7.36	165522.4	206009.5	213361.5	249810.5	151752.3	140513.5	128881.9	150160.1	171503.1	182093.5	3206.7	6766.9	4684.1	8898.0
P35846	Folate receptor alpha	Folr1	255	29.4	6.62	296861.8	290062.6	279383.9	295907.5	279405.1	277318.2	352771.4	310554.7	293070.6	299993.8	86279.7	66694.6	82002.6	66002.2
Q8BGH4	Receptor expression-enhancing protein 1	Reep1	201	22.3	9.5	213424.4	241077.5	203887.4	208104.9	191462.2	178821.9	189861.4	201522.9	191681.6	187391.7	137601.5	136664.2	110847.5	111314.2
Q9D2V5	Protein AAR2 homolog	Aar2	384	43.4	5.48	16123.4	20143.6	18510.3	19353.4	16592.9	20177.9	16120.5	15372.7	20334.5	17753.1	50186.6	51652.5	58563.6	54799.2
Q8R1N0	Zinc finger protein 830	Znf830	363	40.6	5.4	29054.3	28618.9	25444.3	28077.0	26481.1	27599.1	29482.7	26685.8	29733.3	29815.5	277011.4	279611.6	279336.3	257202.9
P59759	Myocardin-related transcription factor B	Mrtfb	1080	117.5	6.16	105477.9	98442.9	89912.6	91057.2	94797.8	95982.0	99372.7	86471.1	97536.1	85442.9	393667.7	369137.0	335638.9	373445.2
Q0GNC1	Inverted formin-2	Inf2	1273	138.5	5.21	13167.9	5177.3	7517.6	7915.5	4862.2	9959.7	4060.1	7973.6	4766.8	3665.7	14895.0	23887.6	13684.7	15326.2
P12265	Beta-glucuronidase	Gusb	648	74.1	6.7	88582.4	99950.6	84039.0	87323.8	89046.9	96605.0	94403.3	95841.5	88558.4	91171.9	157779.9	151651.3	153174.1	151496.6
Q9QYI5	DnaJ homolog subfamily B member 2	Dnajb2	324	35.6	5.91	74752.0	86010.0	86248.8	89379.7	71633.7	72694.9	82945.0	86074.8	84884.9	81550.2	61210.1	68245.6	76904.0	80295.0
Q61581	Insulin-like growth factor-binding protein 7	Igfbp7	281	29	8.31	187478.2	233913.4	181274.1	211862.2	182243.1	187591.6	165888.5	198557.6	184524.6	201872.0	300551.9	320603.0	279443.8	287184.3
Q80X95	Ras-related GTP-binding protein A	Rraga	313	36.5	7.72	237821.2	210261.2	200886.8	197185.4	203010.5	205156.5	220431.3	200484.5	228229.2	209227.1	233602.5	211905.2	250333.5	203718.6
Q9DB43	Zinc finger protein-like 1	Zfpl1	310	34.1	8.19	348559.5	349601.1	373448.2	381336.9	321139.5	296764.8	288410.6	306191.1	312067.9	340843.9	277207.8	313548.5	292377.2	327723.1
Q8R409	Protein HEXIM1	Hexim1	356	40.2	5.38	160467.5	162629.9	144091.7	154444.1	182154.0	201893.6	182134.5	140613.4	165200.0	161915.6	208652.2	209217.9	202433.1	193149.0
P15307	Proto-oncogene c-Rel	Rel	587	64.9	6.54	400997.1	416136.8	370017.3	366490.6	385248.2	407616.1	341232.0	338434.7	390071.2	398485.1	29702.7	29313.0	21722.2	24099.2
P61460	GATOR complex protein DEPDC5	Depdc5	1591	180.3	6.74	66634.0	70647.0	64851.6	63124.4	74080.6	72162.3	63171.2	65197.5	67727.0	68372.2	26589.4	31202.6	34120.5	31741.6
Q9WUD8	Fas apoptotic inhibitory molecule 1	Faim	179	20.2	5.64	452392.0	464421.1	450826.8	460066.8	432641.8	412927.5	424969.2	378445.3	476363.7	475837.6	369281.4	382151.7	352294.3	410221.2
Q3USJ8	F-BAR and double SH3 domains protein 2	Fchsd2	740	84.2	5.66	79729.9	74833.1	76385.1	74674.9	83469.7	93693.8	81800.5	80083.2	78031.3	73230.0	84966.6	90874.3	91035.8	85751.9
Q91ZE0	Trimethyllysine dioxygenase, mitochondrial	Tmlhe	421	49.6	8.25	109894.7	83509.8	121311.3	100636.5	126477.3	110621.7	124802.5	100962.8	133487.4	118242.6	201738.8	202531.8	203881.0	191311.3
Q99KI3	ER membrane protein complex subunit 3	Emc3	261	30	6.81	424761.8	384849.3	470452.0	429343.1	481638.8	434241.2	445492.0	446476.8	494278.2	466411.4	157577.8	174020.1	173819.5	167297.1
Q9CQ54	NADH dehydrogenase [ubiquinone] 1 subunit C2	Ndufc2	120	14.2	9.2	229254.5	210675.3	240097.9	243908.7	234497.2	226223.5	233200.5	238825.2	251422.3	208400.9	436418.3	436579.0	400018.9	432994.3
Q80U70	Polycomb protein Suz12	Suz12	741	83	8.81	107102.7	125863.4	116011.6	101976.6	131314.3	134503.8	112368.1	120324.0	126049.3	119076.3	348395.5	370575.7	381542.8	347621.5
Q3UN04	Ubiquitin carboxyl-terminal hydrolase 30	Usp30	517	58.2	8.46	31077.2	41157.8	34882.0	36183.8	35515.5	39351.9	34003.0	33029.7	29821.2	33282.0	14346.1	15153.7	13973.6	13213.0
Q99M07	Cytochrome c oxidase assembly factor 5	Coa5	74	8.4	8.7	113199.5	111481.5	125563.5	122375.0	123799.1	120839.9	127742.6	117744.0	134303.2	123167.2	85501.9	84057.2	93032.1	79975.4
Q9ESJ0	Exportin-4	Xpo4	1151	129.9	5.12	72232.2	94008.5	68754.9	80134.4	81028.6	92107.1	78402.6	81683.7	81686.1	82731.6	67857.9	85150.2	70822.9	70692.3
Q8BK62	Olfactomedin-like protein 3	Olfml3	406	45.7	6.23	118808.1	109945.9	104483.6	112865.3	105662.0	100773.9	106686.7	124523.6	97536.1	102717.7	267404.3	260814.5	241943.1	283323.7
Q80ZQ9	Protein Abitram	Abitram	193	21.6	7.25	188002.8	245646.5	200679.8	213878.6	229141.9	224112.9	201528.3	204186.2	221520.3	221136.4	161096.6	155650.5	157478.9	154857.1
P70202	Latexin	Lxn	222	25.5	5.74	65135.2	54674.3	78509.1	59133.3	75763.0	70329.5	71424.2	67971.9	57660.9	68434.9	49283.0	54075.9	46727.4	48964.3
A0A2I3BR81	5'-nucleotidase domain-containing 2	Nt5dc2	553	63.8	7.59	87845.7	92860.4	84395.4	90260.8	83267.8	87473.2	85236.4	85611.6	78645.9	98955.9	220546.6	246210.3	224614.2	203449.0
O09110	Dual specificity mitogen-activated protein kinase kinase 3	Map2k3	347	39.3	7.43	147119.1	165731.9	127756.9	156090.4	139329.5	154902.0	146023.2	139956.0	153827.3	157888.6	65201.3	63151.0	56274.2	61352.3
Q8K274	Ketosamine-3-kinase	Fn3krp	309	34.4	7.87	63215.8	61291.5	61176.5	61190.0	55655.4	55849.2	67527.8	65620.7	56004.9	64973.0	117403.5	116951.2	128573.6	140149.3
Q8R001	Microtubule-associated protein RP/EB family member 2	Mapre2	326	36.9	5.38	85788.1	82270.2	87862.1	87176.6	93504.5	91321.2	99670.8	90892.0	89465.6	86697.9	61660.5	64683.3	62315.3	60524.7
O89050	Muskelin	Mkln1	735	84.8	6.34	41582.2	44461.5	47849.5	40758.9	53236.8	50430.2	48162.3	54645.5	50736.3	45742.8	34014.9	40377.7	36773.8	36786.6
Q9DBG9	Tax1-binding protein 3	Tax1bp3	124	13.7	8.48	132836.7	159427.2	125260.2	140917.7	150845.8	139448.9	139787.6	115638.5	167755.2	161783.7	24039.8	27384.1	26129.5	20569.0
Q8VD00	Sigma intracellular receptor 2	Tmem97	176	20.8	9.28	81883.4	104581.7	99397.8	123289.0	71160.1	72178.2	67836.1	70510.2	86709.8	100188.9	234124.2	333772.1	295722.4	407433.2
Q8BXL7	ADP-ribosylation factor-related protein 1	Arfrp1	201	22.6	6.55	298979.8	346741.0	293753.1	344419.0	354261.5	360694.2	309179.0	313804.3	329573.5	334724.7	178954.7	203695.8	189057.0	187055.4
Q8R502	Volume-regulated anion channel subunit LRRC8C	Lrrc8c	803	92.3	7.81	103111.7	107686.5	98757.3	102316.0	108193.3	109436.9	97796.5	110314.2	107901.4	112891.1	115416.5	112999.8	108977.6	100002.5
O89051	Integral membrane protein 2B	Itm2b	266	30.2	5.3	77838.7	76251.8	72177.1	76951.9	71583.5	78148.5	81008.8	86229.8	75151.7	70941.8	75006.9	86614.7	70757.9	85283.7
Q68ED3	Terminal nucleotidyltransferase 4B	Tent4b	633	69.7	8.78	126247.8	124158.2	116600.6	113455.0	138544.3	135043.6	109614.9	120201.6	127303.0	123676.7	49268.9	53760.5	46701.3	43642.7
Q9CZU4	GTPase Era, mitochondrial	Eral1	437	48.2	8.78	87140.0	96676.2	83536.0	85090.2	91206.5	89110.8	74017.7	84772.7	83326.9	88256.1	130314.1	164940.7	116851.9	117864.9
Q925N2	Sideroflexin-2	Sfxn2	322	36.1	8.82	37328.0	38194.5	41068.5	29903.9	30936.8	34708.2	51140.5	36516.0	37576.5	37531.3	61673.1	62498.9	65390.2	58861.4
Q9EPL9	Peroxisomal acyl-coenzyme A oxidase 3	Acox3	700	78.4	7.12	239052.6	202671.5	194213.0	232920.2	208548.4	215892.6	216583.6	212116.4	224258.8	220305.1	229868.6	233645.6	210345.6	252043.6
Q9CQ21	Malignant T-cell-amplified sequence 2	Mcts2	181	20.4	9.11	158771.5	146055.9	143718.0	140461.4	135754.1	154523.2	150929.0	142014.4	157215.5	149500.9	24833.1	30128.1	27802.6	27700.4
Q9Z0V8	Mitochondrial import inner membrane translocase subunit Tim17-A	Timm17a	171	18.1	7.9	32188.5	32269.3	31860.8	34552.9	37900.7	31674.9	30440.2	30580.8	40800.3	35533.8	#N/A	#N/A	#N/A	#N/A
Q9DAK9	14 kDa phosphohistidine phosphatase	Phpt1	124	14	5.53	96285.6	96411.2	100974.0	98054.7	101156.9	107420.6	107432.5	113676.8	102741.8	101349.7	115066.8	117558.3	122509.0	120252.3
Q9R060	Cytosolic Fe-S cluster assembly factor NUBP1	Nubp1	320	34.1	5.9	116604.8	123184.0	108896.9	104124.8	114259.6	116470.8	110699.3	112397.8	118554.9	129397.2	430024.5	384916.1	376783.6	414181.9
Q91WK0	Leucine-rich repeat flightless-interacting protein 2	Lrrfip2	415	47.1	5.68	124478.5	175885.7	155011.3	212705.4	92404.2	93686.5	91262.6	113910.1	133586.9	157999.5	504655.7	478130.1	514334.3	492107.4
Q6VN19	Ran-binding protein 10	Ranbp10	620	67.1	6.58	76968.6	76174.6	66319.2	74305.3	68262.9	82294.1	79632.6	81976.8	69210.9	77860.8	80044.3	81626.7	83247.5	95020.3
Q9CQE6	Histone chaperone ASF1A	Asf1a	204	22.9	4.41	10944.7	12575.3	14641.5	11782.4	16198.4	13155.0	14476.3	13000.7	15380.8	11671.5	29462.7	27198.7	23042.6	14214.4
Q9D8N2	DENN domain-containing protein 10	Dennd10	357	40.4	6.47	262216.0	270086.8	261476.0	283581.1	293157.4	294141.6	264830.2	266629.3	273599.4	271320.7	265568.3	225936.8	194215.4	243727.7
Q80YC5	Coagulation factor XII	F12	597	65.7	6.84	242157.5	240885.7	210595.2	217366.2	234514.1	227472.9	256694.5	225908.7	225671.2	241266.2	145581.4	140649.0	125021.6	134908.7
O88735	Ensconsin	Map7	730	82	9.36	442199.4	494847.2	412045.9	440713.5	449718.9	426711.3	406873.9	393071.6	438020.7	471998.3	100865.3	118728.5	102263.3	107032.0
Q9R0D8	WD repeat-containing protein 54	Wdr54	334	35.6	6.21	252798.4	308962.7	272780.7	362110.8	228070.7	233864.6	211163.5	241718.8	257272.1	286696.2	221942.1	274012.5	251424.9	320229.4
Q8BHJ9	Pre-mRNA-splicing factor SLU7	Slu7	585	68	7.08	62837.3	53484.0	74220.8	52171.5	67381.0	63562.1	54094.5	64167.1	56138.1	58645.2	69443.0	73815.2	68448.7	62191.2
P53569	CCAAT/enhancer-binding protein zeta	Cebpz	1052	120.2	5.74	110740.7	98038.1	91683.9	95075.1	102552.8	110779.2	119543.9	97126.9	114720.1	122754.9	72593.8	73979.8	73803.7	69462.9
F8VPX2	F-box and WD-40 domain protein 9	Fbxw9	458	50.5	6.9	73723.5	87713.9	79927.2	82333.1	66433.7	60963.6	67951.3	62681.4	68034.2	78214.8	226696.8	317210.7	292582.4	352826.8
P70451	Tyrosine-protein kinase Fer	Fer	823	94.5	7.05	316162.8	287456.8	267706.0	311505.4	280498.1	284116.7	286498.9	264538.9	293279.7	297584.5	504212.4	629427.3	410301.1	650883.5
Q80ZV0	Ribonuclease H2 subunit B	Rnaseh2b	308	34.7	9.45	206595.6	241020.2	197938.6	212481.2	229995.7	227878.3	205721.6	205926.5	216664.6	235532.0	95469.9	104225.3	104992.4	101659.1
E9PZM4	Chromodomain-helicase-DNA-binding protein 2	Chd2	1827	210.7	8.06	132391.5	125961.2	125794.0	123077.1	127519.6	132040.4	126174.4	118952.3	129379.3	132659.6	72764.9	67113.6	72467.9	67896.8
A6H619	PHD and RING finger domain-containing protein 1	Phrf1	1682	184	8.59	108348.0	112309.0	93291.1	110236.3	104833.2	92586.2	97312.2	89021.0	110702.4	111965.3	6005.0	8304.3	9060.4	8596.1
B9EJ80	PDZ domain-containing protein 8	Pdzd8	1147	127.7	6.04	120115.1	100998.7	95467.4	110410.6	111045.8	111962.9	103228.0	107078.9	114810.5	120616.8	144390.5	150089.3	135482.7	137538.7

Q5SRY7	F-box/WD repeat-containing protein 11	Fbxw11	542	62	7.12	95477.0	103357.4	103159.5	93867.9	107092.4	100368.2	94148.5	102036.8	103884.4	113040.9	#N/A	#N/A	#N/A	#N/A
Q8C5X1	DUF4745 domain-containing protein	'31406P16I	1059	115.5	7.24	156966.8	165652.1	149015.3	155086.6	177511.4	183967.5	164788.8	157243.0	160923.3	163043.3	#N/A	#N/A	#N/A	#N/A
Q7TN75	Retrotransposon-derived protein PEG10	Peg10	958	109.8	6.38	69526.8	77164.1	59886.6	63672.0	66902.2	62508.7	59992.7	62593.7	71913.3	70069.0	20816.7	27515.8	18666.2	23223.1
O88554	Poly [ADP-ribose] polymerase 2	Parp2	559	63.4	8.43	206068.4	240860.7	231989.0	225376.5	254851.1	250927.9	222243.4	199191.7	220060.9	230733.2	84684.2	92173.1	86828.2	89642.5
Q9QX60	Deoxyguanosine kinase, mitochondrial	Dguok	277	32.3	7.66	105668.5	108288.1	99671.3	93796.7	112404.2	121760.3	105575.2	95516.8	95260.4	95566.9	216090.3	232970.1	223795.1	219442.6
Q924L1	LETM1 domain-containing protein 1	Letmd1	360	41.7	10.54	143912.0	143179.5	139799.1	142903.0	142988.4	144850.8	150617.0	131564.4	143986.4	142767.9	830562.5	863285.3	781222.0	781579.7
Q8BU85	Methionine-R-sulfoxide reductase B3, mitochondrial	Msrb3	253	26.8	7.99	35900.7	30906.4	33157.3	28785.1	25558.9	32639.4	30444.1	35426.7	34430.7	33406.1	33116.0	38943.9	38607.8	36459.7
Q924T7	E3 ubiquitin-protein ligase RNF31	Rnf31	1066	119.2	6.52	122201.1	140109.7	114076.9	129052.6	138163.8	126862.4	118814.2	129592.5	122661.9	121253.6	83700.0	87852.5	83050.8	81512.2
Q149F1	RNA pseudouridylate synthase domain-containing protein 2	Rpusd2	553	61.5	6.77	153060.6	153906.2	153735.7	156131.0	152872.7	161931.0	152030.1	136349.3	158685.9	162438.2	178003.2	189316.5	205866.0	192572.6
Q7TQD2	Tubulin polymerization-promoting protein	Tppp	218	23.6	9.42	132698.7	181122.8	140796.2	176007.5	161163.4	194512.3	157019.9	137242.2	146625.8	155594.2	18054.3	28601.0	26233.1	24586.1
Q9D6K5	Synaptojanin-2-binding protein	Synj2bp	145	15.8	6.3	66754.4	73764.5	68386.9	89320.8	61775.8	61952.5	53825.3	65012.0	78996.0	70618.6	189605.7	218465.5	204858.2	222300.0
P04627	Serine/threonine-protein kinase A-Raf	Araf	604	67.5	9.11	32187.0	35579.6	26649.1	32911.4	27341.0	31784.4	26257.1	28002.6	27148.2	31682.1	58600.4	68202.8	55448.8	57037.9
Q9CR59	Growth arrest and DNA damage-inducible proteins-interacting protein 1	Gadd45gip'	222	25.8	10.27	238596.3	219056.2	204561.1	207540.6	198675.7	195342.8	226879.7	183887.3	221738.1	219143.1	66161.8	66246.6	50400.0	55541.3
O08539	Myc box-dependent-interacting protein 1	Bin1	588	64.4	5.03	112069.6	111759.4	103972.1	106839.9	116072.7	123353.7	138705.3	112596.2	109877.9	108752.3	160283.0	145833.2	132533.1	157158.5
Q9CQ56	Vesicle transport protein USE1	Use1	270	30.6	9.11	161138.7	180279.1	176294.7	185257.5	164319.8	145120.0	141740.9	138189.6	150794.9	158629.6	48329.7	56081.3	50727.9	53646.8
Q811I0	ATP synthase mitochondrial F1 complex assembly factor 1	Atpaf1	324	36.3	8.19	308442.4	329825.3	282562.8	310637.5	303751.1	283969.6	269984.3	268783.2	329669.9	336882.9	98339.1	82577.6	115850.8	97064.0
Q925T6	Glutamate receptor-interacting protein 1	Grip1	1127	122	6.55	199671.1	193173.0	166065.5	176517.0	182792.2	177436.0	190776.5	167930.6	185898.4	198787.0	243640.5	225570.7	206835.1	210552.2
Q8QZY6	Tetraspanin-14	Tspan14	270	30.7	6.51	136860.5	135301.7	139090.6	144117.8	171271.1	159016.5	161393.0	142243.9	146752.4	158261.4	168542.4	154499.0	145556.5	167554.1
Q9D2R0	Acetoacetyl-CoA synthetase	Aacs	672	75.2	6.71	401062.9	426203.5	374843.1	441098.5	407566.6	367970.2	384210.9	375913.2	438855.3	415650.7	184552.6	193222.0	170850.6	199951.6
Q9CQX6	N-acetyltransferase domain-containing protein	Gm16286	220	24.8	6.23	208292.6	229923.6	260456.2	256209.7	241817.3	203734.6	226552.8	225089.7	222510.8	265533.0	326365.9	293279.5	298134.1	287289.1
Q8C0Y0	Serine/threonine-protein phosphatase 4 regulatory subunit 4	Ppp4r4	875	99.4	7.71	69049.2	75793.0	67838.1	72464.3	64144.4	79445.4	74521.4	55148.6	70779.5	71675.7	72785.2	81613.3	71485.8	74997.1
Q99PN3	Tripartite motif-containing protein 26	Trim26	545	62.8	5.05	172567.0	208533.6	175425.5	192342.4	211497.4	214394.8	224643.9	188048.1	204963.0	201291.2	97559.8	101276.2	103614.5	101300.8
Q08122	Transducin-like enhancer protein 3	Tle3	772	83.4	7.2	362192.7	342814.3	383492.4	346549.3	379235.3	389844.8	388183.0	363541.5	360374.6	348801.0	#N/A	#N/A	#N/A	#N/A
Q9ER73	Elongator complex protein 4	Elp4	422	46.3	8.78	133322.0	121221.2	127472.8	130659.3	118955.1	127702.4	124473.5	125352.9	112510.2	120590.4	201730.5	204650.0	201220.2	196131.0
Q9D920	BLOC-1-related complex subunit 5	Borcs5	195	22.1	6.55	149460.4	142366.2	150138.6	158377.5	158017.0	143640.5	138541.0	143480.2	152867.6	145363.5	129687.9	142043.1	135410.1	143987.0
Q3U5F4	YrdC domain-containing protein, mitochondrial	Yrdc	280	29.4	6.07	347299.5	388431.8	386834.8	404371.5	350807.3	350020.9	314180.8	332652.5	349708.5	366645.2	189748.6	232298.4	203803.4	228925.7
P51163	Uroporphyrinogen-III synthase	Uros	265	28.5	6.54	142295.1	147172.7	141321.5	147891.5	160338.3	151297.3	143206.4	157301.7	154245.1	143573.6	427824.9	465722.7	468529.4	515940.8
Q9EP72	ER membrane protein complex subunit 7	Emc7	241	26.3	9.23	277455.3	332821.2	287351.5	277443.4	323367.3	312200.8	295578.7	263761.4	287212.7	301486.5	212350.3	282664.7	248151.7	220223.4
Q00560	Interleukin-6 receptor subunit beta	Il6st	917	102.4	5.52	173235.8	181961.7	161910.4	166275.5	176124.8	187190.2	171601.1	161543.5	184647.5	197279.7	167703.0	177037.4	139088.3	165529.1
Q8BWG8	Beta-arrestin-1	Arrb1	418	46.9	6.28	79646.3	87812.1	75904.7	86149.6	87121.9	89520.0	112303.3	85615.0	97299.0	88516.3	43357.2	41027.9	38643.8	39032.3
P42128	Forkhead box protein K1	Foxk1	719	74.9	9.17	189741.0	175889.7	170826.2	182106.2	170362.4	200921.7	174999.3	173545.9	178502.5	188900.0	195321.1	191842.9	188063.7	189810.4
Q8BGF7	PAN2-PAN3 deadenylation complex catalytic subunit Pan2	Pan2	1200	135.2	5.96	89152.6	98175.3	85500.2	96595.9	94257.4	100990.0	77213.8	100508.9	90948.0	92550.6	301544.4	351460.9	248229.7	304487.4
Q9D2J4	V-set and immunoglobulin domain-containing protein 1	Vsig1	407	44	4.58	79719.5	75267.7	69015.1	84818.3	77160.7	84598.8	79399.9	62756.0	77584.2	69544.9	64196.7	63247.6	66765.7	61051.0
Q8R059	UDP-glucose 4-epimerase	Gale	347	38.2	6.74	228625.9	256958.4	217044.4	226163.8	267917.5	228474.3	196599.0	223258.1	243051.5	260087.6	203591.2	242262.4	224457.9	218096.5
Q8BUY9	Geranylgeranyl transferase type-1 subunit beta	Pggt1b	377	42.3	6.83	100316.0	93308.7	97314.9	94861.3	100341.3	100340.0	91622.3	91220.1	93553.9	102158.0	127671.7	128164.9	119174.3	122497.1
Q9D8Z1	Activating signal cointegrator 1 complex subunit 1	Ascc1	356	41.3	6.54	74164.8	76180.4	71689.6	77300.5	78386.4	89119.7	78394.6	72411.0	75755.5	74420.8	93295.5	102778.2	98789.7	98805.9
Q91WV0	Protein Dr1	Dr1	176	19.4	4.75	134412.2	154458.5	163869.4	194730.0	134493.2	125346.2	133292.2	124691.3	151959.3	162564.5	129357.4	162050.9	183217.7	198856.7
O35166	Golgi SNAP receptor complex member 2	Gosr2	212	24.7	8.21	257959.2	245880.7	263875.6	244743.8	255528.5	245615.7	277170.7	217594.3	279820.9	271521.8	79737.0	83294.0	71039.7	73837.5
Q3V3N7	Bardet-Biedl syndrome 1 protein homolog	Bbs1	593	65.1	8.54	51979.6	61610.3	53983.3	62559.3	47503.0	56050.6	48909.1	48324.2	39107.4	56647.8	39067.9	52453.6	43867.8	50720.9
Q9JHS3	Ragulator complex protein LAMTOR2	Lamtor2	125	13.5	5.4	19460.6	12175.1	18689.6	14491.2	20913.0	13730.2	13835.8	18265.9	14855.5	14925.8	34505.5	40503.4	34660.2	31029.2
Q9JJ94	Sjoegren syndrome nuclear autoantigen 1 homolog	Ssna1	119	13.5	5.68	169820.9	225411.3	252886.9	265464.4	165629.9	147623.1	115971.0	185038.6	206245.7	191608.2	171156.3	237221.4	235219.7	265953.3
O35621	Phosphomannomutase 1	Pmm1	262	29.8	5.47	94972.8	94235.6	96801.5	99847.6	97090.2	103743.0	102119.8	99868.8	95052.0	95347.7	314509.1	351336.3	333902.0	306343.3
P30285	Cyclin-dependent kinase 4	Cdk4	303	33.7	6.62	66938.7	70994.9	60807.3	59444.4	60974.5	64963.0	59974.6	58753.5	54380.2	63023.9	74902.2	84553.7	64836.2	64312.0
Q61285	ATP-binding cassette sub-family D member 2	Abcd2	741	83.4	9.09	190770.9	212186.1	156842.0	182873.6	198105.5	192697.2	179396.7	175454.4	179964.4	190940.3	114764.9	110294.2	102965.6	113881.9
G5E8Z2	Transcription initiation factor TFIID subunit 4B	Taf4b	850	89.5	9.36	234994.2	451074.5	207681.8	323817.2	327699.7	329097.9	221271.0	276517.7	319581.0	369071.2	66288.3	96289.5	65874.2	77464.5
Q8BTJ4	Bis(5'-adenosyl)-triphosphatase enpp4	Enpp4	456	51.6	6.73	129654.3	126102.1	121115.1	133009.3	118190.0	120552.4	127462.6	117573.8	122892.2	131549.2	154075.4	160428.2	152876.1	175565.4
Q6P8M1	Putative deoxyribonuclease TATDN1	Tatdn1	295	33.3	6.18	185118.5	172588.3	174726.7	172649.9	205708.9	191411.0	192791.5	197293.2	196114.7	189976.5	413607.7	436732.1	414030.8	435623.6
P28843	Dipeptidyl peptidase 4	Dpp4	760	87.4	6.42	52538.8	55357.8	56643.7	58033.8	67637.3	69102.0	75178.9	70647.7	57573.1	64736.3	25134.4	28669.3	24429.3	25949.2
Q3UUJ4	STE20-related kinase adapter protein alpha	Strada	431	48.2	6.43	94613.8	114179.0	105867.3	126879.9	108758.5	112711.9	96277.7	94353.2	102414.8	113013.3	105925.0	118177.0	120514.6	126822.1
Q8BUY8	G-protein coupled receptor-associated sorting protein 2	Gprasp2	826	92.7	5.11	167765.3	181139.0	171219.6	168840.1	179681.4	179579.0	172284.5	161038.6	168634.6	195330.4	103221.8	75799.5	87502.8	75610.4
Q32Q92	Acyl-coenzyme A thioesterase 6	Acot6	419	46.7	7.02	273164.4	217375.5	225130.1	208385.4	254963.9	226384.7	276843.0	239559.6	262044.9	250170.8	#N/A	#N/A	#N/A	#N/A
Q8K400	Syntaxin-binding protein 5	Stxbp5	1152	127.6	7.21	97261.4	116522.7	110775.0	112993.1	117347.1	120599.3	104717.9	99428.7	113663.3	105566.3	27953.2	25536.4	31948.2	29725.0
Q8BVL9	Janus kinase and microtubule-interacting protein 1	Jakmip1	626	73.1	6.09	148477.3	123337.2	125814.2	107016.8	159320.3	153643.0	170247.8	135488.7	133854.3	132984.6	136627.8	87709.3	127032.3	97176.6
Q9WTU0	Lysine-specific demethylase PHF2	Phf2	1096	120.7	9.17	101680.5	101435.8	100325.0	92398.3	111063.3	123491.2	107774.4	95291.8	93966.2	94204.2	#N/A	#N/A	#N/A	#N/A
Q91VC9	Growth hormone-inducible transmembrane protein	Ghitm	346	37.3	9.8	36729.0	39411.4	39683.2	32622.2	37884.2	36725.9	35849.2	41278.8	34522.1	31341.6	125346.9	136668.1	123778.7	141646.6
Q8CCM6	Mitochondrial import inner membrane translocase subunit Tim21	Timm21	244	27.9	9.95	92861.6	86426.2	91019.2	85622.0	89297.8	88592.9	92228.7	97065.2	84552.1	89230.8	152234.8	129135.1	140239.8	147013.6
Q9Z179	SHC SH2 domain-binding protein 1	Shcbp1	668	75.9	4.97	71973.6	75597.1	69976.9	64812.6	83974.4	72613.8	60059.8	69082.2	62966.2	74624.4	202582.3	218190.3	192821.7	213344.4
Q5SF07	Insulin-like growth factor 2 mRNA-binding protein 2	Igf2bp2	592	65.5	8.03	136558.5	146937.9	104617.4	132623.5	107674.8	126246.9	105789.2	107393.5	118317.2	129823.4	31444.5	29132.6	28658.3	26867.3
Q9R112	Sulfide:quinone oxidoreductase, mitochondrial	Sqor	450	50.3	9.09	144922.1	150224.3	139713.8	151591.0	142046.7	154841.0	154828.8	145001.4	135285.0	140107.7	253296.6	287624.8	255310.9	261936.4
Q5XKN4	Protein jagunal homolog 1	Jagn1	183	21.1	9.8	41242.8	40267.4	40280.3	44556.7	44093.4	45068.7	50754.4	40595.3	48437.1	46869.4	86479.1	77475.8	93162.1	90178.9
Q5SSK3	Transcription elongation factor, mitochondrial	Tefm	364	41.8	9.51	159949.4	151107.5	162194.2	150902.9	155534.3	166488.3	142465.4	151083.0	157606.6	153206.4	88202.0	87584.4	74919.0	82823.7
P55065	Phospholipid transfer protein	Pltp	493	54.4	6.62	54171.1	51947.8	42656.4	52420.4	45900.2	48703.6	37102.1	52150.0	38792.2	42339.1	35722.8	35671.0	29896.1	33454.4
Q9QXA5	U6 snRNA-associated Sm-like protein LSm4	Lsm4	137	15.1	10.05	714592.4	725890.6	748660.6	734536.0	833048.3	804808.5	820646.0	729471.7	796987.3	783083.7	569495.6	611206.2	609827.6	589825.6
Q812A2	SLIT-ROBO Rho GTPase-activating protein 3	Srgap3	1099	124.3	6.67	72010.7	76618.4	80547.4	79530.9	74279.7	75471.3	65163.2	67781.8	66043.6	77455.7	113091.9	130481.6	103123.4	117712.5
P84309	Adenylate cyclase type 5	Adcy5	1262	139	7.06	55618.3	56308.0	57400.6	51054.6	54289.6	59995.2	50288.9	58412.2	55786.8	52755.4	38955.9	39766.2	38280.8	40145.8
Q9CZX9	ER membrane protein complex subunit 4	Emc4	183	20.1	8.62	132854.3	143568.6	151947.5	128754.9	137464.8	138023.3	130840.2	134519.0	145339.1	145729.0	96407.4	115455.3	131169.9	98088.5
Q9ER41	Torsin-1B	Tor1b	336	37.8	8.02	212217.1	235450.6	209401.6	229528.6	193423.6	229438.6	211612.7	230092.2	211207.6	220171.8	123217.5	121986.6	132223.8	130910.4
Q8BG73	SH3 domain-binding glutamic acid-rich-like protein 2	Sh3bgrl2	107	12.2	5.55	64151.9	58763.1	82663.7	66263.5	94322.1	82448.4	75581.7	76809.1	82099.6	83375.4	101550.2	101777.9	124726.8	99738.2
O55142	60S ribosomal protein L35a	Rpl35a	110	12.5	10.89	155396.0	215566.0	187469.8	209067.7	155943.1	160082.1	160812.3	162715.8	175362.6	178732.9	2837181.4	2795501.6	3193501.0	2734926.1
Q9CR11	YEATS domain-containing protein 4	Yeats4	227	26.5	8.41	336059.2	348293.7	357555.6	366038.1	370820.7	389350.2	380391.5	360323.5	353238.9	353055.7	190669.7	203504.8	211928.8	196516.2
Q8K215	LYR motif-containing protein 4	Lyrm4	91	10.8	10.13	571323.7	536124.9	586331.5	598255.9	568891.0	538659.0	542745.8	539134.5	592456.9	579415.4	779911.2	803784.5	847369.0	813165.4
Q91X21	Uncharacterized protein KIAA2013	Kiaa2013	634	69.4	7.8	238032.2	276842.5	246119.2	229696.9	241398.5	222875.3	211483.0	226400.0	234278.5	246743.3	437615.8	516036.8	468684.5	524000.0
Q8CG14	Complement C1s-A subcomponent	C1sa	688	76.8	5.08	113460.7	114562.5	103144.2	101457.0	114387.2	140384.1	142983.1	117679.9	107559.2	114118.7	96226.2	108769.2	89114.5	98205.7
Q9R0Q9	Mannose-P-dolichol utilization defect 1 protein	Mpdu1	247	26.5	8.75	229508.9	208068.9	203863.9	217543.0	244492.1	223407.0	227539.5	210308.1	216591.1	231256.2	390784.3	399620.1	337792.4	369070.1
Q9D6J3	Splicing factor YJU2	Yju2	314	36	6.07	96070.6	117991.2	96849.1	101632.8	111368.7	111077.5	94903.6	101662.0	103172.3	105097.1	63703.8	65940.1	62459.7	63575.4
Q8CC12	Codanin-1	Cdan1	1239	135.8	7.25	73489.4	81533.6	84073.0	81361.2	73496.2	79043.6	75693.5	76821.5	83852.4	84320.8	83034.2	83600.9	76515.3	74951.3
P41209	Centrin-1	Cetn1	172	19.7	4.88	171125.9	241958.1	276016.6	374102.3	127206.1	132561.8	127362.2	150147.3	206977.0	221249.0	60079.9	81684.4	72601.2	88270.2
O70494	Transcription factor Sp3	Sp3	783	82.3	5.26	47312.7	34990.9	49268.0	36442.9	44799.4	52561.6	48767.2	41266.7	46809.4	42835.8	37576.0	35374.6	35351.3	26420.8
Q8BYM8	Probable cysteine--tRNA ligase, mitochondrial	Cars2	551	61.2	8.18	57814.2	62133.9	57011.2	62182.2	63136.8	58271.9	61731.8	67129.6	53978.8	60677.3	209019.7	211937.9	188334.3	214411.4
Q8K2I1	Protein farnesyltransferase subunit beta	Fntb	437	48.8	5.8	193421.6	216259.0	184165.7	194773.2	188026.1	203364.3	208206.1	194790.9	188269.1	189016.4	52388.6	63713.0	54100.0	50355.9
Q8VC66	Afadin- and alpha-actinin-binding protein	Ssx2ip	615	70.9	6.65	255167.5	294616.9	245664.7	293001.5	271179.0	241157.4	213282.0	237705.6	260882.9	269786.4	106185.8	147263.3	115508.1	113046.1
O35711	Liprin-beta-2	Ppfibp2	882	98.7	6.33	84881.7	103407.7	86516.2	88116.4	110691.7	91636.6	97291.5	93686.3	101723.5	99557.4	197967.9	175209.2	169513.8	170398.9
Q3UMT1	Protein phosphatase 1 regulatory subunit 12C	Ppp1r12c	782	84.6	6	179379.8	181149.0	203680.6	177847.6	233303.3	225735.2	231857.7	218671.1	209591.6	188631.1	132676.6	116613.3	109556.6	110243.5
Q9CQX2	Cytochrome b5 type B	Cyb5b	146	16.3	4.89	521184.0	503132.6	519991.3	507578.6	506383.3	475589.2	436926.3	540768.4	423990.7	452495.4	90656.9	108985.6	105606.1	134395.0
Q8VCB1	Nucleoporin NDC1	Ndc1	673	75.4	8.9	174609.2	182652.7	176044.0	176701.0	182214.0	183132.1	121605.5	165462.3	124638.8	124366.8	135897.5	154724.4	123181.7	148132.2
Q6PHZ5	Putative RNA-binding protein 15B	Rbm15b	887	97	9.91	63277.2	72629.7	66591.2	76150.1	70595.9	83377.1	70525.3	65463.7	76607.2	71766.4	124592.2	116478.1	115443.8	117439.4
O70589	Peripheral plasma membrane protein CASK	Cask	926	105	6.43	93130.4	105788.0	97344.4	53359.5	58683.6	61903.4	58766.6	100151.6	94643.2	56961.2	70639.2	69956.5	53736.7	70772.9
P20065	Thymosin beta-4	Tmsb4x	50	5.7	5.06	198901.3	195016.0	223773.2	183933.7	194717.2	212985.4	227092.3	265084.6	245636.2	162485.9	210644.5	205401.9	219486.4	185155.9
Q9Z2B9	Ribosomal protein S6 kinase alpha-4	Rps6ka4	773	85.6	8.27	103586.3	117683.5	103457.3	115522.3	120199.1	126372.6	115708.6	105607.3	112110.9	115776.4	92128.0	90452.9	87036.9	88967.8
O70157	DNA topoisomerase 3-alpha	Top3a	1003	112.3	8.4	160212.9	182504.2	182535.9	175979.4	182769.2	156698.3	162418.4	146740.7	176424.8	184250.8	43626.2	44337.2	44891.0	43863.4
Q99PI5	Phosphatidate phosphatase LPIN2	Lpin2	893	99.6	5.47	187948.3	194488.6	172135.1	174895.1	181729.2	184956.9	159986.6	174182.9	172124.7	185368.2	106809.8	108465.1	102513.0	109729.3
Q921H9	Cytochrome c oxidase assembly factor 7	Coa7	231	25.6	6.29	292864.0	301011.4	320058.8	307056.9	329629.7	321566.7	319150.8	301645.4	321809.4	313686.2	462872.8	497463.2	511987.7	475127.9

P70196	TNF receptor-associated factor 6	Traf6	530	60	6.55	161520.6	160667.0	136525.6	140940.4	153749.3	168055.8	145386.1	139376.6	150585.7	145990.8	96029.4	88394.2	85519.7	96791.5
Q3TMH2	Secernin-3	Scrn3	418	47.6	5.66	56541.5	69938.3	54396.8	60595.0	56112.3	70090.3	58462.5	61368.5	61301.8	53563.7	122744.9	133163.5	124302.5	119739.0
O35648	Centrin-3	Cetn3	167	19.5	4.78	223325.3	211710.8	193112.0	205222.5	174256.4	178186.7	162465.5	186384.4	190211.5	200831.2	109050.5	108296.2	126024.6	138673.1
Q9CQ06	39S ribosomal protein L24, mitochondrial	Mrpl24	216	24.9	9.5	43873.1	60374.9	47085.1	53699.8	45972.2	50723.6	46541.6	41136.1	52633.1	55860.2	146360.3	177912.8	126672.5	150618.0
Q8K2I4	Beta-mannosidase	Manba	879	100.8	7.25	71268.1	77777.1	71281.5	74312.8	75516.5	78131.2	80075.1	77677.1	67428.9	73961.5	179623.2	177173.3	139392.3	188644.8
Q8CHS8	Vacuolar protein sorting-associated protein 37A	Vps37a	397	44.4	5.67	247609.6	237987.2	254738.0	249262.6	235889.0	240085.6	214358.1	223857.3	239184.7	249218.2	71626.0	71328.6	74755.5	73357.1
Q9CYH6	Ribosome biogenesis regulatory protein homolog	Rrs1	365	41.5	10.77	107867.1	104951.2	85514.9	100022.3	97078.2	89961.0	97328.3	78154.8	106134.5	113580.0	89800.8	89882.3	78062.5	87777.7
Q63829	COMM domain-containing protein 3	Commd3	195	22	5.59	195695.3	209240.9	193600.1	224374.5	193802.1	206239.7	190402.1	181844.4	193631.2	192618.1	331989.1	344094.8	322862.9	389576.6
Q8R242	Di-N-acetylchitobiase	Ctbs	366	41.3	5.6	686837.9	728639.6	702070.4	709863.7	752894.1	796911.8	778830.5	720083.9	758328.4	744679.9	50145.4	53976.1	54363.5	53097.6
E9Q6P5	Tetratricopeptide repeat protein 7B	Ttc7b	843	94.1	6.9	75688.6	100556.9	77751.5	90098.3	100744.9	100191.2	78404.1	73900.1	81120.5	88907.6	132362.6	127468.1	114793.2	135775.4
Q99MZ6	Unconventional myosin-VIIb	Myo7b	2113	240.7	8.92	184264.8	209119.3	141681.6	181713.2	177586.7	176440.5	159550.6	150809.5	184218.8	213062.5	#N/A	#N/A	#N/A	#N/A
Q4KML4	Costars family protein ABRACL	Abracl	81	9	5.8	480803.5	534621.3	421917.7	560465.1	390275.5	332471.9	389595.6	508680.4	454988.0	408198.2	1091914.2	1167024.8	933875.9	1236602.6
Q9R069	Basal cell adhesion molecule	Bcam	622	67.6	6.25	84645.0	84103.7	79734.2	80248.9	88147.5	101595.6	111351.4	96637.3	80319.7	85928.3	178250.2	149497.2	182108.2	172797.2
Q922G2	Protein FAM76A	Fam76a	307	35.1	9.19	78641.9	75939.9	73169.5	72606.5	75174.3	78623.1	78929.9	74812.3	79205.0	82600.1	491502.6	511334.0	487262.3	490966.9
P20664	DNA primase small subunit	Prim1	417	49.3	8.6	233749.3	252459.3	220627.8	211025.3	237552.2	240134.7	223283.6	222347.8	236451.0	244292.8	229314.0	241227.0	234344.4	240456.9
Q6EJB6	U3 small nucleolar RNA-associated protein 14 homolog B	Utp14b	756	85.9	8.85	50014.8	48664.3	53554.3	45014.1	48501.5	53625.8	51590.2	42488.7	54553.3	50628.7	9611.0	10933.5	12405.4	9166.7
Q8R2U4	N-terminal Xaa-Pro-Lys N-methyltransferase 1	Ntmt1	223	25.4	6.9	207612.4	218614.5	175075.3	212999.0	160731.3	162687.4	151425.9	161797.6	187943.3	191791.9	95757.0	115308.1	103250.1	127378.4
Q8K1Y2	Serine/threonine-protein kinase D3	Prkd3	889	100	6.81	68060.4	72942.4	68847.8	65222.8	70282.1	76316.5	75226.7	70199.3	71500.1	68031.0	37700.4	39033.2	41609.1	34588.1
Q8VDN4	Coiled-coil domain-containing protein 92	Ccdc92	314	35.2	9.38	88010.8	113245.6	97636.3	112654.6	83690.8	85825.2	70982.4	74543.9	86203.7	101813.7	219144.0	245835.4	195699.8	244772.6
P39098	Mannosyl-oligosaccharide 1,2-alpha-mannosidase IB	Man1a2	641	72.8	8.28	160489.7	164096.5	160600.1	168347.2	158973.7	157513.5	143544.4	159658.3	170831.9	152602.9	327315.7	302578.1	296940.9	298469.1
Q6VGS5	Protein Daple	Ccdc88c	2009	226.4	6.15	264494.7	270307.7	270486.6	268315.8	279662.7	272140.4	272574.8	272087.4	298945.6	275430.6	210570.6	217480.4	226907.8	224407.3
Q6ZPS6	Ankyrin repeat and IBR domain-containing protein 1	Ankib1	1085	121.8	5.16	135136.8	129388.5	121637.6	132414.5	157456.2	142188.4	126265.0	126096.8	133021.1	149532.5	256097.2	265579.8	246469.7	250034.9
Q8BGT8	Phytanoyl-CoA hydroxylase-interacting protein-like	Phyhipl	375	42.3	6.35	187295.9	238006.1	217916.9	248091.0	185876.1	202921.8	177094.5	167056.8	202113.5	219041.8	39555.1	51444.3	47432.5	50694.6
P22682	E3 ubiquitin-protein ligase CBL	Cbl	913	100.5	6.67	199882.0	182055.1	193434.0	153447.7	205991.5	201753.1	242239.1	190921.3	213227.4	218426.9	143946.6	153678.0	127135.2	142587.2
Q8VDD9	PH-interacting protein	Phip	1821	206.6	8.82	47205.2	47188.0	48114.1	45500.9	60813.3	66003.8	51909.1	56965.4	48983.1	44646.1	23697.9	26556.7	21338.1	23429.6
Q64191	N(4)-(beta-N-acetylglucosaminyl)-L-asparaginase	Aga	346	37	6.44	108759.1	99963.1	102449.3	103114.1	99249.9	108188.8	108814.0	94071.8	97973.0	106853.1	75794.2	82162.2	84634.2	82575.1
Q91YY4	ATP synthase mitochondrial F1 complex assembly factor 2	Atpaf2	289	33.3	6.32	364949.5	396034.5	332935.9	361648.7	357944.7	341909.5	329044.6	359757.8	347965.6	350077.4	40105.2	42905.3	38011.8	40739.9
Q8BYY4	Tetratricopeptide repeat protein 39B	Ttc39b	617	70.2	6.64	95605.2	106090.9	92005.4	103155.4	98025.1	116521.7	86015.3	90310.5	100817.7	105021.4	35220.0	44415.0	33846.1	45859.2
C0HKG6	Ribonuclease T2-B	Rnaset2b	259	29.6	6.37	361694.3	405970.0	375689.5	372555.4	410415.4	431407.1	396050.3	342192.9	412679.7	388969.0	193719.4	268907.3	288039.2	260021.6
Q8BLR9	Hypoxia-inducible factor 1-alpha inhibitor	Hif1an	349	40.2	5.83	73084.5	93491.1	74695.1	77066.1	86065.5	90487.9	75311.2	76584.1	79037.9	79391.2	311408.5	299178.7	289940.8	280023.8
Q8BGD8	Cytochrome c oxidase assembly factor 6 homolog	Coa6	79	9.3	8.16	29554.2	30308.0	34398.9	37745.4	33148.0	28637.9	31264.5	36149.4	37162.7	30784.9	13159.1	12070.2	15290.9	14738.0
Q80TH2	Erbin	Erbin	1402	157.2	5.69	92299.9	99775.1	78114.7	87817.8	93665.6	115027.2	97617.4	101880.7	84842.0	89668.7	127644.4	128788.7	115142.3	116836.7
Q91VN4	MICOS complex subunit Mic25	Chchd6	273	29.8	8.41	169107.5	185907.8	182258.1	197101.4	183978.0	185442.0	182797.3	178464.5	181540.7	173256.4	146919.1	203942.6	235257.5	277987.1
Q01147	Cyclic AMP-responsive element-binding protein 1	Creb1	341	36.7	5.57	208166.2	222768.7	210956.3	197357.8	249672.1	267487.7	292145.8	225254.0	240816.5	242447.6	74724.0	69982.7	71446.2	73726.6
B2RXR6	Serine/threonine-protein phosphatase 6 regulatory ankyrin repeat subunit B	Ankrd44	993	107.3	6.42	161523.9	161797.8	153376.1	152805.7	170406.7	167900.0	148793.5	180031.4	168983.4	162638.7	69174.6	69450.2	64570.6	64438.0
Q8C761	WD repeat-containing protein 60	Wdr60	999	115.3	7.24	208072.6	248674.7	260588.6	227340.3	200186.8	186520.1	185433.6	192420.1	212053.4	234650.9	30344.7	38013.2	44325.9	44406.0
P97300	Neuroplastin	Nptn	397	44.3	7.74	71154.8	68411.0	68684.7	62471.0	74511.9	84403.0	79407.3	81740.0	68932.9	68223.3	199540.6	191554.4	191348.8	168981.6
Q64362	AKT-interacting protein	Aktip	292	32.9	9.31	38311.6	44450.8	40609.0	43270.6	42765.9	47893.2	46054.7	42052.6	37502.1	40615.9	51881.5	53467.6	54021.0	50963.1
Q9CYT6	Adenylyl cyclase-associated protein 2	Cap2	476	52.8	6.43	61200.5	62507.6	60352.2	59572.0	68558.9	56357.8	59159.7	73936.0	61165.4	66903.0	#N/A	#N/A	#N/A	#N/A
O54782	Epididymis-specific alpha-mannosidase	Man2b2	1018	115.5	7.39	151401.5	139278.0	138277.0	158829.4	140139.4	146081.0	132664.6	129549.0	138126.5	142505.0	404447.5	361885.9	361707.5	360041.3
Q3UHA3	Spatacsin	Spg11	2430	273.8	5.94	159011.7	181975.7	159459.3	145483.0	172075.3	176322.4	139019.1	150396.1	156158.9	169134.8	22081.4	30345.8	23230.8	29003.9
Q9DC77	Small muscular protein	Smpx	85	9.2	9.7	137271.4	118446.3	157469.2	137638.0	158767.4	146563.8	161975.2	182735.3	137673.4	127471.9	338041.3	400565.9	401099.3	418006.9
Q8K3X4	Probable E3 ubiquitin-protein ligase IRF2BPL	Irf2bpl	775	80.5	8.24	261432.0	264494.4	240492.8	237245.0	295357.0	286866.6	326145.0	257286.9	261766.2	287080.9	230457.4	231887.8	235228.9	221496.7
F8VPQ2	AT-rich interactive domain-containing protein 4A	Arid4a	1261	142	5.07	254947.3	252639.4	232999.7	230860.4	247046.7	247313.9	296305.3	222334.2	301828.1	263747.7	192250.9	190572.4	170977.2	173623.4
Q9CR56	NF-kappa-B inhibitor-interacting Ras-like protein 2	Nkiras2	191	21.5	8.05	99042.2	102344.5	105207.9	91837.9	99089.4	97895.5	86711.5	89437.8	107403.9	100182.9	384981.8	367696.3	385750.1	381982.3
Q9D4V0	Ethanolamine kinase 1	Etnk1	363	42	5.44	270600.7	276646.4	268320.8	300091.4	246551.8	244815.2	220555.2	240817.1	270092.0	279591.8	271582.5	267985.4	247124.0	290407.6
O08856	RNA polymerase II elongation factor ELL	Ell	602	67.1	9.19	326114.2	335455.9	323993.9	334746.5	348854.3	377977.7	309888.8	355811.9	330188.6	337325.3	55241.5	64375.0	62424.2	59983.6
Q9JKP5	Muscleblind-like protein 1	Mbnl1	341	37	8.6	12454.9	14047.8	9990.8	11471.8	11396.2	11341.6	12464.4	14230.2	11296.4	9464.8	409108.8	364250.9	327482.9	383478.5
Q8CI78	Required for meiotic nuclear division protein 1 homolog	Rmnd1	450	51.8	7.99	165172.2	160919.7	141601.6	151188.1	151119.4	156524.9	172080.8	144312.2	149329.3	164984.7	279984.7	268075.6	262627.0	262109.8
Q5SSL4	Active breakpoint cluster region-related protein	Abr	859	97.6	6.58	181313.0	186884.9	177271.9	173688.6	188589.5	208611.8	181430.9	189814.2	170323.0	187980.8	427833.2	375952.7	397786.1	388745.7
Q8CIB6	Transmembrane protein 230	Tmem230	120	13.2	9.31	187715.7	215747.9	242412.5	245543.0	176946.8	166802.2	162853.4	176524.7	209547.9	204528.2	226094.5	253172.8	282893.8	253297.5
Q9Z211	Peroxisomal membrane protein 11A	Pex11a	246	28.1	9.83	66744.4	72428.0	59284.6	69020.1	58357.0	60702.1	57872.7	53970.2	58069.9	63916.2	498130.1	476324.2	400838.0	463595.5
Q5SYL3	Protein KIAA0100	Kiaa0100	2234	254.3	7.34	111696.7	131122.7	114533.1	120554.2	131014.7	128632.8	124210.2	122370.7	120448.3	116935.1	94178.8	94464.4	93102.2	92399.0
Q8K2Q0	COMM domain-containing protein 9	Commd9	198	21.8	5.82	74241.0	70091.8	77709.5	67486.4	81743.5	77858.4	94556.5	77685.8	84236.3	71058.1	46975.6	41868.4	45399.9	42146.4
Q9D009	Putative lipoyltransferase 2, mitochondrial	Lipt2	231	24.9	8.6	183289.4	165397.6	187243.7	179295.6	177708.8	182322.6	177671.7	183673.1	188715.1	192723.6	259242.7	375997.3	254826.2	287905.1
A0A1L1SQU7	FAT atypical cadherin 1	Fat1	4645	511.9	5	115236.6	111077.5	115335.4	108351.8	120878.6	117847.6	110693.5	123986.0	131634.9	114476.1	98292.7	73988.6	75856.9	80228.2
Q9QZC2	Plexin-C1	Plxnc1	1574	176.4	7.75	78538.8	63645.4	78606.1	61370.0	75221.7	76650.9	84480.8	73024.5	73193.8	74743.8	75978.9	66349.1	71957.6	66095.9
Q9JMA2	Queuine tRNA-ribosyltransferase catalytic subunit 1	Qtrt1	403	44.1	7.68	349000.5	344903.4	378836.1	357709.0	372660.4	385463.8	372049.6	335897.2	391933.6	381465.3	69104.3	70638.8	58156.0	75080.2
Q3TLS3	GDP-D-glucose phosphorylase 1	Gdpgp1	386	42.5	6.19	271356.5	284081.2	271967.6	303351.8	295386.1	329398.8	328633.5	270187.0	298193.1	304992.6	225077.4	223997.2	222834.8	224348.6
P13597	Intercellular adhesion molecule 1	Icam1	537	58.8	6.13	168803.0	204376.3	140027.1	176469.2	167729.8	170911.9	181938.9	143160.9	168413.2	191591.6	141919.6	148876.2	140711.5	151440.5
Q6NS59	Protein FAM135A	Fam135a	1506	166.8	5.47	103280.2	110112.2	97060.4	106175.6	89790.6	99240.9	93871.4	86472.0	104009.7	109469.4	95781.4	110105.6	86662.1	102774.7
Q8CEE7	Retinol dehydrogenase 13	Rdh13	334	36.4	8.85	446635.3	499141.3	478793.3	538093.1	441166.1	452163.1	431515.5	383029.4	449280.3	485265.8	45377.5	40098.5	43564.0	42170.8
Q8R1M0	Abasic site processing protein HMCES	Hmces	353	40.1	8.57	137170.3	124285.0	148923.4	127161.7	149970.1	142338.4	130202.2	129902.1	137463.7	144110.3	125446.8	118776.0	146141.5	111572.4
Q9D1F4	Proline-rich AKT1 substrate 1	Akt1s1	257	27.5	4.72	256124.4	253180.2	242329.9	234858.6	239076.5	213851.9	237740.2	230346.0	232029.0	224947.5	269192.5	276240.8	255022.2	245260.7
O54950	5'-AMP-activated protein kinase subunit gamma-1	Prkag1	330	37.5	7.14	197541.9	200973.2	173724.2	184067.6	171438.2	184335.1	176641.2	173525.1	164415.7	171911.2	150974.6	129074.2	128720.1	140362.6
P97801	Survival motor neuron protein	Smn1	288	31.2	7.62	71688.8	87236.0	69223.1	75252.5	76124.7	72888.7	65889.9	72461.7	67932.7	82481.3	74740.9	73957.5	78773.6	77290.9
Q9DBX6	Cytochrome P450 2S1	Cyp2s1	501	55.6	8.78	123912.6	72303.2	60337.6	66088.1	72555.3	71817.8	73374.6	132914.6	70579.2	77016.0	567441.3	296902.9	261899.9	247264.3
P97497	Sex hormone-binding globulin	Shbg	403	44.8	6.58	75158.7	89196.5	90366.6	79974.4	68345.1	69814.1	62529.0	76815.0	70709.2	75173.2	26895.5	37376.9	62037.8	37086.0
Q8BP78	Protein FRA10AC1 homolog	Fra10ac1	315	37.2	7.28	97754.9	112128.9	103953.7	106085.7	107189.0	91059.3	77860.7	90957.7	102897.0	109494.6	110112.0	119952.2	118565.0	133420.3
Q91V81	RNA-binding protein 42	Rbm42	478	50.2	9.63	179558.8	186967.5	152456.6	177520.1	174434.3	171420.5	154547.6	162750.5	158013.5	167114.1	127587.6	137884.0	122960.6	129945.1
P15327	Bisphosphoglycerate mutase	Bpgm	259	30	7.06	135716.2	119399.0	121958.1	127178.9	126921.4	130093.2	134608.3	127123.1	130531.6	130866.9	244368.8	230648.4	231468.2	265696.8
Q9ER64	Oxysterol-binding protein-related protein 5	Osbpl5	874	98.9	7.44	40010.3	41525.6	37965.7	40170.7	39600.5	42824.6	37686.7	37872.0	41583.9	41600.3	102948.9	92966.0	88951.4	91130.6
Q69ZZ6	Transmembrane and coiled-coil domains protein 1	Tmcc1	649	71.6	6.55	96885.1	104760.8	100398.6	103223.4	106988.2	105264.6	95075.1	88425.1	104734.7	102530.9	216559.9	232753.3	229492.9	245877.2
Q80W49	Beta/gamma crystallin domain-containing protein 3	Crybg3	1005	114.2	5.67	323742.8	346683.3	335687.3	349981.1	299111.5	302347.4	290922.9	290173.7	321154.9	310790.6	289558.4	289324.9	301795.1	330764.8
P17047	Lysosome-associated membrane glycoprotein 2	Lamp2	415	45.7	7.39	354294.4	456625.7	346633.4	381423.4	408320.6	422345.1	497341.2	352484.7	411017.2	403756.2	1229412.2	1351412.0	1172184.5	1384165.1
Q8VCM3	Zinc finger FYVE domain-containing protein 21	Zfyve21	234	26	8.34	52354.6	50967.8	55817.9	50283.6	59915.2	52250.2	53502.7	53149.9	41663.1	57919.2	158117.2	160132.6	156495.7	148486.7
Q8BPA8	Protein DPCD	Dpcd	203	23	9.03	61412.5	87923.2	88911.9	104496.1	65572.9	60265.9	63696.5	59211.8	68671.3	76963.4	127235.8	151889.6	163659.7	191220.2
O54786	DNA fragmentation factor subunit alpha	Dffa	331	36.6	4.91	66577.6	74878.9	71811.5	76495.6	73014.7	79667.7	72610.6	60454.0	78063.6	74151.3	79087.9	89842.8	89938.7	89423.1
Q9Z210	Peroxisomal membrane protein 11B	Pex11b	259	28.7	9.95	100739.6	123636.6	101794.0	122661.7	124910.8	106745.1	100739.8	97671.5	103917.3	125228.5	116249.9	122289.2	118774.4	113476.9
Q91XD7	Protein disulfide isomerase Creld1	Creld1	420	45.7	5.02	273474.8	248172.5	240794.4	250391.6	251675.8	241079.4	239645.5	255598.4	260956.9	260076.3	118844.5	135158.8	110243.0	114121.6
Q9CZX7	Type 2 phosphatidylinositol 4,5-bisphosphate 4-phosphatase	Pip4p2	257	28	8.68	41863.1	41401.6	38620.4	43321.2	46274.9	44337.8	41395.1	37100.8	44800.0	40695.6	29822.0	29552.1	32001.7	36097.9
P14231	Sodium/potassium-transporting ATPase subunit beta-2	Atp1b2	290	33.3	8.31	212115.2	192073.0	196489.8	198997.1	183413.6	176673.2	210594.4	192347.3	183813.6	186839.0	163782.6	142502.6	164919.8	160000.8
Q3V3Q7	Phosphofurin acidic cluster sorting protein 2	Pacs2	862	94.9	6.51	138465.2	130626.1	131069.7	131227.3	123980.4	123274.5	123636.6	127480.6	124953.9	131470.0	277116.9	295989.5	290800.5	293520.5
Q60936	Atypical kinase COQ8A, mitochondrial	Coq8a	645	71.7	6.54	188323.2	203749.5	209404.4	202582.3	262378.1	226659.5	236944.0	212055.7	211764.4	213029.4	88979.6	80960.8	88268.6	92313.6
Q9ESD7	Dysferlin	Dysf	2090	237.8	5.6	49126.8	39169.6	41342.0	36788.6	44868.4	41600.1	38862.5	44445.7	40872.6	42107.3	24641.8	28128.8	25712.4	25291.0
Q9CYH5	Glucose-fructose oxidoreductase domain-containing protein 2	Gfod2	385	42.2	6.25	196745.7	181075.0	201982.1	174601.4	237887.9	231102.0	218211.9	193820.9	203082.2	208302.2	96559.4	119390.5	99004.2	106102.9
Q9DCZ1	GMP reductase 1	Gmpr	345	37.5	7.09	123619.2	109294.1	122144.5	88538.9	152811.3	105637.6	135902.5	94919.3	124338.6	151244.9	30355.6	29710.9	24221.2	27561.6
Q9JI99	Sphingosine-1-phosphate phosphatase 1	Sgpp1	430	47.7	8	174455.3	194228.0	149432.9	157060.8	158051.8	163454.9	143708.3	155837.7	162747.7	157937.7	1145715.1	1146001.1	1077858.3	1152535.3
Q8BUV8	Protein GPR107	Gpr107	551	62	7.59	160036.0	170670.8	148760.9	165092.0	157029.2	166882.7	145114.1	157513.7	155039.6	162679.2	298715.3	299757.1	325390.9	326673.1
O08848	60 kDa SS-A/Ro ribonucleoprotein	RO60	538	60.1	7.9	174563.7	169345.4	175238.4	150122.7	169191.3	181022.2	192980.4	177915.8	170560.4	178112.3	185200.2	174845.3	165633.3	175481.5
Q9CXE7	Transmembrane emp24 domain-containing protein 5	Tmed5	229	26.2	4.93	208342.6	232493.6	242174.5	203038.6	292341.0	222075.1	241379.5	202512.1	218970.3	195523.3	134936.1	151804.6	159044.3	129278.8
Q6X893	Choline transporter-like protein 1	Slc44a1	653	73	8.75	121591.1	132418.5	126168.7	115654.4	122387.2	127344.7	136858.5	122682.6	120833.5	128859.6	73802.2	84403.6	66173.0	76509.2

Q8VED9	Galectin-related protein	Lgalsl	172	18.9	5.35	62057.4	63148.1	51762.7	57998.1	66756.7	69440.7	65531.2	63621.3	65377.5	69006.4	142470.5	153612.9	118193.6	139425.5
P97471	Mothers against decapentaplegic homolog 4	Smad4	551	60.3	6.99	44790.9	47041.0	41368.2	43513.3	46787.6	45586.0	45345.8	43830.6	45706.0	49377.1	192522.4	161252.6	186784.0	150245.5
Q6NS82	Reticulophagy regulator 2	Retreg2	541	57.5	4.46	106613.7	111686.5	113449.1	135190.9	93435.0	104799.2	98738.4	93664.7	110816.5	120176.5	329329.0	456663.8	291676.5	410944.5
Q6NZR2	Myb/SANT-like DNA-binding domain-containing protein 2	Msantd2	559	61.3	6.05	32685.5	33960.9	27902.7	28367.8	32340.5	42048.8	29279.1	23153.3	33443.4	34771.0	16393.3	17352.3	15723.9	16832.4
Q9D6K7	Tetratricopeptide repeat protein 33	Ttc33	262	29.4	5.35	69848.2	84917.3	76064.3	77811.9	90066.8	81200.9	71129.1	74407.3	70214.8	71780.4	124832.1	137641.0	122028.8	132323.5
Q9QXN3	Activating signal cointegrator 1	Trip4	581	66.2	7.55	95666.3	95588.5	104404.5	104429.1	101431.6	92900.4	86713.5	94261.3	97315.0	97309.1	#N/A	#N/A	#N/A	#N/A
Q8QZX2	HAUS augmin-like complex subunit 3	Haus3	570	66.3	5.29	143193.9	162672.3	172866.1	157768.5	181969.4	173960.3	145817.3	147445.4	166448.2	177401.1	145276.7	150929.6	134371.0	138886.6
P49070	Calcium signal-modulating cyclophilin ligand	Camlg	294	32.5	7.74	161719.5	158208.2	150391.1	153483.0	160804.1	160809.0	150346.2	147339.8	151403.8	163191.7	153835.6	145426.6	121627.6	124520.9
Q6P4T0	Autophagy-related protein 2 homolog A	Atg2a	1914	210.8	6.06	91956.2	101478.8	81207.4	87041.7	92688.1	84189.2	81931.8	84565.1	85664.1	92476.5	#N/A	#N/A	#N/A	#N/A
Q2VPR5	PWWP domain-containing protein	Hdgfl1	283	31.7	4.27	102937.1	159217.8	174481.7	304347.4	57599.3	59917.1	54679.9	75031.0	113313.2	125805.8	52032.7	65210.3	59997.4	63784.5
Q8BGA7	Uncharacterized protein C7orf26 homolog	MGI:244262	448	49.9	7.52	52490.0	57837.9	48530.7	51770.5	52884.8	58680.6	51746.3	56282.4	53389.2	52842.5	46304.2	46387.1	40329.5	49002.7
Q4VBD2	Transmembrane anterior posterior transformation protein 1	Tapt1	564	63.9	8.22	122197.2	144543.4	148830.7	146668.8	121093.8	104762.8	98869.5	122308.3	145812.8	128423.5	283668.9	283203.2	264789.0	277011.0
Q9CQM5	Thioredoxin domain-containing protein 17	Txndc17	123	14	4.77	701090.5	644958.7	670646.7	653031.5	724070.2	736702.2	768514.0	763591.0	723689.0	702376.9	612285.1	556801.0	534218.2	573291.0
Q8VCM5	Mitochondrial ubiquitin ligase activator of NFKB 1	Mul1	352	39.8	7.96	150263.9	136367.9	101974.5	131492.6	120837.5	113848.5	128079.8	120479.1	134417.7	138019.1	#N/A	#N/A	#N/A	#N/A
Q9CZ28	Vacuolar-sorting protein SNF8	Snf8	258	28.9	6.65	75315.6	89054.8	100953.1	92860.5	95734.9	93953.9	91466.4	78429.2	96354.7	93308.8	117471.1	144876.1	122926.9	148426.6
Q9CR09	Ubiquitin-fold modifier-conjugating enzyme 1	Ufc1	167	19.5	7.4	324903.4	288725.0	332546.3	324241.7	307980.5	308449.1	260993.5	325257.2	293982.6	315373.1	571161.0	507743.9	591918.8	510831.8
Q6GQT6	Sterol regulatory element-binding protein cleavage-activating protein	Scap	1276	139.5	7.08	135482.9	141366.6	107681.6	138690.1	111211.7	114229.0	122809.0	114101.6	130853.8	148005.5	175590.8	173412.3	147771.9	159928.0
Q9WVH4	Forkhead box protein O3	Foxo3	672	71	5.12	50552.3	55761.1	56709.8	47356.9	60220.4	63724.8	62692.9	58458.2	55968.9	58707.1	112033.6	126398.0	88931.7	104773.6
P60904	DnaJ homolog subfamily C member 5	Dnajc5	198	22.1	5.07	188129.9	183383.5	171677.3	165518.6	191120.2	188524.1	193591.7	172041.1	180666.5	178221.0	251533.2	232942.3	248657.8	231754.9
Q99PW4	EKC/KEOPS complex subunit Tp53rk	Tp53rk	244	27.4	9.23	254228.5	266787.1	257656.9	252169.7	314423.3	305962.8	274244.8	266212.7	273290.4	284192.6	163421.7	177825.8	169022.3	168795.2
Q9QZ08	N-acetyl-D-glucosamine kinase	Nagk	343	37.2	5.73	522075.0	504050.9	496515.0	500533.3	460454.7	474013.7	471391.9	444884.0	484586.0	484027.6	308833.2	270504.5	346161.2	271885.0
Q6P9N1	Hyccin	Fam126a	521	57.3	7.88	122792.2	127795.2	130050.0	133488.5	121505.3	122022.7	129153.0	124431.1	125354.6	135601.8	268182.7	289135.3	299275.0	287472.2
Q9Z0G0	PDZ domain-containing protein GIPC1	Gipc1	333	36.1	5.91	132481.2	145480.2	119919.6	129356.3	142653.0	154552.3	133411.4	132608.1	133227.7	135847.3	414506.2	396947.1	334570.3	358810.3
O70362	Phosphatidylinositol-glycan-specific phospholipase D	Gpld1	837	93.2	7.12	196440.9	175890.8	174708.8	191075.9	184033.0	186641.8	160315.0	190236.1	186504.8	198401.6	88017.2	73007.6	71698.2	69524.5
P03921	NADH-ubiquinone oxidoreductase chain 5	Mtnd5	607	68.4	9.03	408730.3	448505.1	426980.5	440170.9	424056.4	422890.3	373843.4	431527.6	388590.6	452856.6	869994.1	840959.6	868760.5	849538.3
Q61165	Sodium/hydrogen exchanger 1	Slc9a1	820	91.4	7.12	74187.8	72599.7	67606.3	69271.7	77621.1	79083.2	83337.3	66131.8	77005.3	72449.0	149503.1	194705.6	142271.8	179565.6
Q8C0P5	Coronin-2A	Coro2a	524	59.5	7.71	62082.6	56892.0	57777.5	58520.4	56235.8	60207.9	61501.7	68327.0	60948.5	50376.9	46381.5	61231.1	40652.5	46043.9
Q3TC33	Coiled-coil domain-containing protein 127	Ccdc127	260	30.5	9.13	50877.0	55627.5	46893.9	47514.4	60679.3	46492.9	51583.8	48631.9	47766.8	43352.3	65792.2	71178.7	71676.8	62443.7
Q80UY2	E3 ubiquitin-protein ligase KCMF1	Kcmf1	381	41.8	5.76	23830.5	24141.1	20518.8	22466.0	18142.1	22804.8	21967.0	18860.3	25718.5	20618.7	20845.9	21370.3	18156.1	20730.3
A2AJ88	Patatin-like phospholipase domain-containing protein 7	Pnpla7	1352	150.4	6.92	147888.9	108275.5	133185.6	114431.9	142923.1	138676.8	140748.7	130655.4	136010.4	132382.0	#N/A	#N/A	#N/A	#N/A
P46938	Transcriptional coactivator YAP1	Yap1	488	52.4	5.11	35632.8	35652.4	37460.2	33340.1	34847.6	37511.0	33418.0	33023.6	32158.3	38365.0	63710.7	61021.5	61343.5	61392.6
Q61712	DnaJ homolog subfamily C member 1	Dnajc1	552	63.8	8.97	173554.0	163897.8	150142.8	163931.0	167500.0	165805.8	161920.8	158381.0	169529.0	158002.8	59816.9	51777.9	52886.1	50935.2
Q9D0K1	Peroxisomal membrane protein PEX13	Pex13	405	44.6	7.53	91126.7	106357.2	97592.2	102873.4	106886.9	111040.8	81000.9	100625.2	106856.9	112140.4	58039.4	65442.3	68778.1	71573.6
P41234	ATP-binding cassette sub-family A member 2	Abca2	2434	270.3	6.76	48888.7	58402.3	57205.7	52773.0	73441.1	70018.8	61529.4	53106.3	63617.5	58543.9	78767.1	80913.2	81050.8	77787.3
Q8R2T8	General transcription factor 3C polypeptide 5	Gtf3c5	520	60.5	6.73	203295.6	219925.3	184547.2	195940.0	199495.5	223891.9	203103.3	200444.0	190611.2	214223.3	93875.1	91112.1	86937.2	84057.9
Q8BJ56	Patatin-like phospholipase domain-containing protein 2	Pnpla2	486	53.6	6.46	40468.2	42005.4	36508.8	42481.6	54256.8	44905.6	41917.9	40947.0	47056.1	42509.1	35593.5	36921.4	34401.8	36733.2
P70280	Vesicle-associated membrane protein 7	Vamp7	220	25	8.6	202911.3	215293.3	179192.9	208803.2	185985.8	180797.4	208061.0	199329.4	201056.7	178305.1	281710.1	291423.2	270523.7	273839.9
Q3U0B3	Dehydrogenase/reductase SDR family member 11	Dhrs11	260	28.3	6.34	200607.0	221362.3	228400.0	215930.3	222913.4	243284.1	217623.8	212000.3	229254.6	222159.4	149344.9	166942.9	143247.0	164225.7
Q9Z2E1	Methyl-CpG-binding domain protein 2	Mbd2	414	43.5	10.04	23599.0	25516.6	24751.0	21742.4	30405.7	30791.3	32248.8	27789.8	23400.1	26390.0	120073.7	138931.1	102196.0	118700.4
Q5XJE5	RNA polymerase-associated protein LEO1	Leo1	667	75.6	4.46	89146.8	93049.0	97085.9	93717.0	93422.7	87661.2	93234.8	86974.8	96003.0	95523.2	76873.1	97310.3	97524.5	93445.3
Q99J83	Autophagy protein 5	Atg5	275	32.4	5.91	288580.8	301814.2	277147.7	294408.0	321169.8	331216.6	277446.4	288672.2	301372.5	312290.5	167174.8	162384.5	149636.1	156859.5
Q80YR2	Protein FAM160B2	Fam160b2	744	82.4	5.62	420749.7	452780.8	379070.7	398517.1	426426.6	424674.0	425298.2	364897.3	407769.6	413717.8	71863.6	63813.1	70513.9	64557.9
Q02384	Son of sevenless homolog 2	Sos2	1333	153	6.76	102640.2	116890.7	103660.9	105460.7	110253.7	111743.0	107922.6	99170.9	105209.9	118025.0	46742.3	49596.3	53412.9	57424.2
P97863	Nuclear factor 1 B-type	Nfib	570	63.5	8.66	52968.0	51787.2	46766.4	42446.2	58537.8	65016.9	80385.3	56314.5	61422.0	58530.8	45788.1	47240.8	47359.7	48701.8
P29595	NEDD8	Nedd8	81	9	7.25	329229.3	372552.1	377782.6	456033.3	394629.0	370737.9	282434.8	383362.3	395425.5	379350.4	707260.7	837413.2	784948.1	1057993.4
Q9Z1J2	Serine/threonine-protein kinase Nek4	Nek4	792	88.9	7.33	234050.8	249413.6	195496.7	273170.4	202126.8	197344.2	167354.8	210066.1	191336.0	191617.2	53593.3	248871.5	48253.8	99942.8
Q9D404	3-oxoacyl-[acyl-carrier-protein] synthase, mitochondrial	Oxsm	459	48.6	7.06	57520.9	62356.3	50273.8	55960.8	59266.8	56340.3	49307.5	57259.6	56538.2	63282.6	100968.6	99407.5	101692.7	111648.3
Q9R0G8	Nik-related protein kinase	Nrk	1455	163.5	6.48	162241.5	210895.1	149528.7	172846.3	162629.5	165658.0	154504.2	156764.0	150350.6	182192.2	162223.0	180245.4	134541.5	160871.7
Q8BHG9	CGG triplet repeat-binding protein 1	Cggbp1	167	18.8	8.95	254800.7	250177.9	233455.0	241233.8	266100.0	252753.8	281186.3	216021.9	266121.2	281953.4	400576.3	386821.7	383176.6	390865.0
Q80U56	Late secretory pathway protein AVL9 homolog	Avl9	649	72.1	6.14	227077.9	238140.5	226845.3	216934.6	236069.6	226925.3	224397.9	226349.7	232199.4	221597.1	262361.0	269843.0	248756.0	247352.2
Q9QYY8	Spastin	Spast	614	66.4	9.69	69717.0	76393.4	62753.5	67961.6	69246.1	71759.8	64712.8	63744.9	70127.7	75813.6	100996.9	106294.5	92873.6	103945.0
Q920D3	Mediator of RNA polymerase II transcription subunit 28	Med28	178	19.5	5.58	72641.0	84559.0	89636.5	92295.4	87212.0	73381.4	64877.0	75405.7	81279.7	82892.4	#N/A	#N/A	#N/A	#N/A
Q8K202	DNA-directed RNA polymerase I subunit RPA49	Polr1e	482	54	8.19	156228.6	178456.6	167260.0	173660.2	176642.0	176843.8	177888.3	161307.6	188302.6	169709.5	39743.4	51797.1	49366.9	53270.2
Q9R257	Heme-binding protein 1	Hebp1	190	21.1	5.26	115616.2	135272.5	110610.3	144086.7	122943.5	117227.8	115690.8	105991.0	117111.2	139572.5	67876.8	70455.5	52887.0	69702.6
Q9EPX2	Papilin	Papln	1280	138.8	7.75	83860.9	89029.0	88004.5	78503.0	98337.3	115154.2	120235.6	91549.2	91668.8	84488.9	16198.9	25290.2	23225.0	24855.3
Q91ZZ3	Beta-synuclein	Sncb	133	14	4.37	43744.0	33812.6	44390.0	31341.3	47515.2	44321.7	37954.8	50735.5	38697.7	28790.2	57445.0	51810.3	57898.3	54163.3
Q80UZ2	Protein SDA1 homolog	Sdad1	687	79.5	9.2	88423.6	89754.8	97976.8	90366.9	98737.5	86638.9	71742.9	90106.9	89183.1	95402.0	334662.1	404624.8	384303.2	380171.4
P42703	Leukemia inhibitory factor receptor	Lifr	1092	122.5	6.04	124224.9	131627.9	105179.8	127515.0	114820.1	109408.6	116542.7	105753.9	126138.3	113465.1	34074.0	38389.5	26647.4	35520.3
O08585	Clathrin light chain A	Clta	235	25.6	4.58	796939.1	1043364.0	847811.8	1109171.4	882200.4	877498.8	1145783.3	875618.9	1047434.4	1102303.2	684164.7	666991.3	796103.2	688996.9
P58137	Acyl-coenzyme A thioesterase 8	Acot8	320	35.8	7.64	313112.5	324079.1	334626.8	315512.2	317107.6	295261.4	275314.0	326491.1	324616.6	320201.5	109416.3	116152.2	95580.1	105607.3
Q9JJ80	Ribosome production factor 2 homolog	Rpf2	306	35.3	10.04	149859.6	149166.7	146081.3	151758.4	151203.1	157909.4	130132.6	148479.7	142687.7	137294.3	197531.6	193036.5	181113.6	187687.2
Q68FD7	Folliculin-interacting protein 1	Fnip1	1165	130	5.55	252148.1	338584.0	300122.0	295810.8	285301.9	269194.2	224077.4	244339.4	285237.3	289633.9	150871.5	155154.4	159497.1	163441.1
Q61164	Transcriptional repressor CTCF	Ctcf	736	83.7	6.9	84604.1	80710.4	82507.9	72994.3	101905.7	94793.4	109211.3	75642.7	79729.6	85666.8	217809.5	205089.5	220748.8	205429.0
Q9CQ49	Nuclear cap-binding protein subunit 2	Ncbp2	156	18	7.81	96150.9	105800.0	127713.2	113068.9	121535.5	121003.2	107792.0	112279.3	112559.1	120828.2	181753.7	250160.7	242041.7	244538.9
Q9JI19	Acidic fibroblast growth factor intracellular-binding protein	Fibp	357	41.2	6.76	157839.4	126417.9	175537.3	158151.5	163617.0	152664.8	150654.1	143213.0	164670.1	157328.0	116759.1	104459.4	122289.8	115020.3
B8JK39	Integrin alpha-9	Itga9	1036	114.3	6.07	309819.1	293586.5	309516.0	276823.6	356719.5	382705.1	419378.4	558953.4	335932.3	325645.3	262602.8	245093.1	254466.4	246247.7
Q9DCF9	Translocon-associated protein subunit gamma	Ssr3	185	21.1	9.61	413724.1	434554.2	416449.3	409273.8	389689.9	370168.4	384595.6	398722.4	409789.2	430723.5	677810.5	700331.2	615967.0	672686.9
Q8BTT6	Digestive organ expansion factor homolog	Diexf	772	88.8	5.74	85079.0	102409.6	91987.9	105460.0	105680.6	107826.3	89497.4	93814.5	94278.1	112230.4	108174.2	114679.3	107335.2	98711.2
Q9CY21	Probable 18S rRNA (guanine-N(7))-methyltransferase	Bud23	281	31.6	8.88	114038.9	131593.0	100310.3	112235.8	115554.2	114241.1	101516.4	116269.5	99493.4	114655.4	46864.4	58781.6	50117.6	45226.0
Q91WT4	DnaJ homolog subfamily C member 17	Dnajc17	303	34.4	8.57	120970.3	155676.1	135318.1	134401.3	150486.1	157262.7	123975.5	127118.9	136795.9	150311.8	94581.9	98467.6	103523.1	89505.5
Q8BVF2	Phosducin-like protein 3	Pdcl3	240	27.6	4.72	117165.0	117099.4	101518.3	109373.1	99905.8	108491.6	101423.8	107095.6	105650.7	103347.7	388714.0	379660.4	394259.6	358478.7
Q6NVF0	Inositol polyphosphate 5-phosphatase OCRL	Ocrl	900	104.2	6.27	87211.1	90783.8	82007.3	95963.2	90577.6	101233.0	89447.0	85559.7	82847.1	88510.4	78877.8	90566.4	80924.2	83524.1
Q9JMD3	START domain-containing protein 10	Stard10	291	32.9	7.12	88973.9	88063.4	82175.0	79484.5	78639.0	91802.7	92277.2	86137.7	82544.2	87184.6	136362.0	129248.1	111901.6	125920.7
P58283	E3 ubiquitin-protein ligase RNF216	Rnf216	853	97.6	5.01	87316.0	87692.0	83218.7	81829.1	96534.6	102712.9	86759.4	79566.9	95531.4	99386.4	77016.0	86664.5	81737.9	95426.3
Q9CQA1	Trafficking protein particle complex subunit 5	Trappc5	188	20.8	9.66	453888.2	464788.8	456305.3	490776.4	464816.6	425911.8	406578.6	424390.0	473943.0	464397.0	219438.4	254174.0	198835.1	220203.2
Q5BLK4	Terminal uridylyltransferase 7	Tut7	1491	169	6.65	38622.1	53381.0	37064.2	45647.7	39551.7	42843.8	34329.8	41959.0	37009.7	44607.4	29696.8	27482.5	30121.2	35858.6
Q9DCB8	Iron-sulfur cluster assembly 2 homolog, mitochondrial	Isca2	154	16.7	5.6	32342.6	37173.2	50475.9	46578.0	54512.7	43575.4	48742.8	48230.0	57143.4	51952.9	71788.1	86535.8	67801.7	85616.7
P48760	Folylpolyglutamate synthase, mitochondrial	Fpgs	587	64.9	8.16	34591.6	30039.6	32299.2	32838.9	35935.7	37985.8	34308.7	39279.3	34853.9	36098.9	33438.2	38846.7	42909.1	36043.2
Q91WK5	Glycine cleavage system H protein, mitochondrial	Gcsh	170	18.6	4.75	310470.6	309492.2	317728.7	319955.0	346992.3	320439.8	326002.6	317180.1	310491.6	306049.7	430626.4	387063.2	433811.1	431613.2
Q60610	T-lymphoma invasion and metastasis-inducing protein 1	Tiam1	1591	177.4	6.67	101851.6	94216.2	83372.0	82129.0	81542.1	83915.5	72010.9	86276.4	87795.9	89616.5	59580.3	64106.6	53779.5	63274.5
Q8BW56	Glycosyltransferase-like domain-containing protein 1	Gtdc1	445	51	7.53	166209.0	197866.2	176251.7	202954.6	166607.7	164662.0	154573.7	150684.1	174860.3	184596.3	36030.3	43643.5	43416.2	43640.4
Q3TWF6	WD repeat-containing protein 70	Wdr70	657	73.1	5.97	98358.8	105704.1	109827.9	113480.1	109861.4	105873.8	101366.8	105466.3	109081.7	112562.6	414956.2	346890.8	355314.3	404409.4
A2AJ15	Endoplasmic reticulum mannosyl-oligosaccharide 1,2-alpha-mannosidase	Man1b1	658	75.1	8.48	154390.8	154189.5	134755.8	155495.1	149267.2	163815.7	141321.5	132133.4	145760.1	141877.9	60791.0	57015.6	60319.5	70860.6
Q00899	Transcriptional repressor protein YY1	Yy1	414	44.7	6.29	156184.0	169913.9	175065.3	175270.0	172409.6	195397.1	181746.7	162351.5	179862.1	190194.8	237339.6	262778.1	260677.7	259840.5
Q9ESP1	Stromal cell-derived factor 2-like protein 1	Sdf2l1	221	23.6	7.42	87579.4	98418.0	95652.7	102560.1	88352.2	92583.4	69430.2	77848.1	100053.9	95089.2	733225.9	796196.7	927410.3	969691.4
Q9DC70	NADH dehydrogenase [ubiquinone] iron-sulfur protein 7, mitochondrial	Ndufs7	224	24.7	9.92	377105.4	371419.5	382633.9	338034.5	360222.0	367441.2	357186.1	345232.2	335866.2	363881.6	766540.0	789752.3	900760.2	816626.2
Q8BG60	Thioredoxin-interacting protein	Txnip	397	44.3	6.87	148028.0	151768.0	140117.0	141471.6	177246.6	186640.4	156774.7	146023.1	159459.3	173937.1	114189.2	114007.2	111979.6	105327.7
P70353	Nuclear transcription factor Y subunit gamma	Nfyc	335	37.2	4.98	154933.2	149572.4	144234.2	140871.0	152923.5	170021.7	163538.4	138057.0	155326.8	155604.3	163952.7	190959.4	169911.3	170773.9
Q6NXY9	DNA-directed RNA polymerase III subunit RPC7	Polr3g	223	25.9	4.59	56263.7	50424.6	55483.9	59829.9	55257.3	61338.2	53025.2	50963.3	48934.2	60815.5	49167.2	59386.6	50592.7	51117.5
Q9QZL0	Receptor-interacting serine/threonine-protein kinase 3	Ripk3	486	53.3	7.53	230075.0	229918.8	207763.1	230973.4	224817.8	245278.2	239695.5	231407.7	240414.6	228943.0	194359.7	182251.6	183719.6	198033.6
Q811J3	Iron-responsive element-binding protein 2	Ireb2	963	104.9	6.93	94194.8	92645.1	89366.4	95044.7	89249.0	93966.9	82585.2	94456.4	92625.8	96216.3	186377.1	189407.0	195205.4	207497.4
P48410	ATP-binding cassette sub-family D member 1	Abcd1	736	81.8	8.7	81302.5	86810.1	77173.9	81945.9	78671.1	79832.9	73853.8	84276.5	82379.2	75185.8	119387.8	117170.1	109465.5	108057.2

Q8VC70	RNA-binding motif, single-stranded-interacting protein 2	Rbms2	383	40.7	9.07	80790.6	74102.2	79374.7	69849.5	76452.4	73419.8	76309.8	88405.8	73271.8	67994.8	606390.0	606328.1	586087.3	559180.8
Q8C147	Dedicator of cytokinesis protein 8	Dock8	2100	238.8	6.96	57295.3	99133.8	53971.2	65927.3	65903.9	84321.3	61179.7	65280.7	64449.4	70929.8	#N/A	#N/A	#N/A	#N/A
Q6A0A2	La-related protein 4B	Larp4b	741	81.6	7.49	95429.6	98129.6	99654.8	104523.6	88679.5	86723.8	81065.4	101905.6	109172.7	92866.6	#N/A	#N/A	#N/A	#N/A
A2AG50	MAP7 domain-containing protein 2	Map7d2	781	86	8.81	47752.1	58398.9	46387.3	48781.1	51766.8	54556.1	48448.2	42373.3	43479.3	48492.5	57399.7	66362.1	69806.1	68528.5
Q6PAL7	AT-hook DNA-binding motif-containing protein 1	Ahdc1	1594	168	8.97	52896.3	73497.3	58678.7	71808.2	67811.3	68283.1	62808.2	57171.2	64518.5	54866.4	108751.9	113557.2	110501.7	113448.1
Q0VGY8	Protein TANC1	Tanc1	1856	200.7	8.35	123488.0	137667.7	143828.0	124999.5	153068.4	163794.4	149981.0	136326.9	146324.2	151676.4	40407.5	41072.6	47668.4	41209.5
Q9JIK9	28S ribosomal protein S34, mitochondrial	Mrps34	218	25.8	10.43	433210.1	461490.7	371415.3	431229.0	424643.6	373092.5	375911.2	385452.9	399266.5	430689.9	162107.0	208219.3	160659.5	169969.3
Q8C1Y8	Vacuolar fusion protein CCZ1 homolog	Ccz1	480	55.5	6.02	190161.7	214796.0	196410.7	204467.8	201293.2	219086.4	190757.1	177088.5	179646.4	206055.5	428953.7	482566.2	514691.7	550999.0
Q3UWL8	Prefoldin subunit 4	Pfdn4	134	15.2	4.61	335705.6	386756.7	299565.6	328396.4	354196.2	349510.2	313249.0	327502.8	380702.8	387051.5	894059.8	988590.4	990011.6	1013366.9
Q9D0Q7	39S ribosomal protein L45, mitochondrial	Mrpl45	306	35.4	9.23	102169.6	103760.7	116919.1	102377.9	109409.9	117910.9	103673.0	109279.8	115852.1	105914.1	589574.5	592142.2	502667.9	559336.8
Q62440	Transducin-like enhancer protein 1	Tle1	770	83	7.23	358248.9	331564.7	337829.9	317574.1	336471.9	356689.8	354758.3	329631.1	327272.3	333304.0	#N/A	#N/A	#N/A	#N/A
Q922H1	Protein arginine N-methyltransferase 3	Prmt3	532	59.9	5.3	87306.0	74617.7	76193.8	57582.2	73040.4	73977.4	57921.2	84727.1	61016.8	66168.6	246885.1	252059.1	220928.9	236963.6
O88630	Golgi SNAP receptor complex member 1	Gosr1	250	28.5	9.29	278162.1	248886.4	254859.5	245569.5	369091.6	385032.7	443980.2	386751.7	332371.5	254169.4	91247.0	101154.2	93892.5	103465.5
P05531	X-linked lymphocyte-regulated protein PM1	Xlr	208	24.6	5.22	156586.2	242207.1	195393.3	245733.1	228176.1	246533.8	154784.5	195736.0	218874.5	213884.7	322029.6	392246.7	371514.9	350712.2
Q3TAS6	ER membrane protein complex subunit 10	Emc10	258	27	5.91	209467.0	248700.5	221344.2	238565.0	232234.5	227071.6	208120.9	203308.6	234455.1	236705.6	167292.8	162961.8	149129.2	160834.3
P52927	High mobility group protein HMGI-C	Hmga2	108	11.8	10.62	213686.0	182691.6	188265.3	164814.6	249724.3	293436.7	355348.4	237470.1	212656.9	203714.8	12864.2	9296.7	9354.8	7145.4
Q91XX1	Protocadherin gamma C3	Pcdhgc3	934	101	5.07	19812.3	17375.7	17715.1	15683.0	17207.7	19182.0	15399.5	17331.2	15181.1	19431.4	29542.1	25814.9	24210.3	23951.8
F8VQB6	Unconventional myosin-X	Myo10	2062	237.2	6.25	122667.6	120246.7	119161.9	124711.3	124948.9	119133.3	113191.4	126055.4	127064.9	118486.6	297153.1	287717.3	236116.1	249263.3
Q8K0F1	TBC1 domain family member 23	Tbc1d23	684	76.4	5.36	300741.7	308550.0	261682.9	274736.6	284264.1	282810.2	271319.6	243764.0	274425.4	297071.7	155565.9	161214.8	125202.5	145074.6
A2ASZ8	Calcium-binding mitochondrial carrier protein SCaMC-2	Slc25a25	469	52.6	8.54	133985.8	105092.6	124733.4	110172.9	144001.3	130570.5	138511.4	110023.6	112824.5	133055.0	20054.5	20041.7	16617.0	18707.3
Q9EQJ9	Membrane-associated guanylate kinase, WW and PDZ domain-containing protein 3	Magi3	1476	161.6	8.05	44035.9	55483.2	43601.2	49448.6	48048.3	54765.3	46643.8	41104.5	53596.7	53002.8	62870.2	63685.2	63033.5	61936.7
P63082	V-type proton ATPase 16 kDa proteolipid subunit	Atp6v0c	155	15.8	9.13	1167628.2	1097293.2	1271254.3	1209879.4	1297951.7	1141114.7	1131426.0	1185616.2	1231998.1	1244292.1	72042.7	76961.0	78367.6	80440.1
Q9CY18	Sorting nexin-7	Snx7	387	45	5.07	177947.7	199368.5	168001.9	199358.3	158349.2	183684.7	175878.9	172055.7	178331.0	178328.5	102982.6	112474.1	95347.4	107319.1
P03958	Adenosine deaminase	Ada	352	40	5.72	174268.3	170783.2	158011.2	155313.3	175013.4	177671.0	176748.4	164402.8	156617.4	152493.1	117483.3	104498.4	95419.3	96259.4
Q8R3Q6	Coiled-coil domain-containing protein 58	Ccdc58	144	16.7	8.16	135150.2	127916.6	142725.6	133809.6	128342.2	133162.5	133858.3	132684.2	130718.4	123438.4	98939.0	100863.7	116394.6	122542.4
Q31125	Zinc transporter SLC39A7	Slc39a7	476	50.6	6.87	158884.0	159911.7	140323.3	159048.9	138163.4	138190.8	157309.4	140499.0	152792.3	150121.8	71590.8	102214.2	62836.5	95980.1
Q8BYN5	FSD1-like protein	Fsd1l	507	56.9	6.7	153160.8	200286.3	183811.1	205909.0	170689.6	155581.0	149380.0	149624.2	176732.6	178540.8	82904.1	73773.8	85917.1	93365.7
Q68FF7	SLAIN motif-containing protein 1	Slain1	579	61.3	7.64	118603.0	113249.7	100811.8	106661.3	98485.9	104845.8	103725.7	103610.6	101292.6	110635.5	39693.3	32924.5	35155.2	34781.0
Q9CWS4	Integrator complex subunit 11	Ints11	600	67.8	7.94	90699.5	89436.5	98183.1	92384.6	103488.8	90229.0	86655.0	89769.8	90933.5	96996.2	75121.0	79713.3	72051.1	73576.7
P01660	Ig kappa chain V-III region PC 3741/TEPC 111	-	111	12.1	5.38	76649.1	69714.8	77227.4	85385.6	79290.7	76030.0	79528.5	75935.8	80621.7	82186.6	62049.9	60070.2	58604.2	66143.4
Q921D4	Mediator of RNA polymerase II transcription subunit 6	Med6	246	28.4	8.62	311931.2	339788.8	360405.8	340975.1	362844.4	326832.1	292177.4	323172.9	360079.6	352875.9	78841.5	82462.2	72640.1	84807.3
O88845	A-kinase anchor protein 10, mitochondrial	Akap10	662	73.6	6.79	126495.3	150879.9	141072.4	143698.3	146765.5	159412.4	129258.4	142330.9	164532.3	150727.7	54359.6	76346.3	75988.1	73823.6
Q8BGP6	Solute carrier family 25 member 40	Slc25a40	337	37.9	8.95	80711.5	77696.2	81232.5	82053.4	92736.1	85400.3	77373.7	80005.6	77952.8	78492.6	293297.7	280413.4	246830.4	269580.1
P99028	Cytochrome b-c1 complex subunit 6, mitochondrial	Uqcrh	89	10.4	4.87	92642.3	77294.2	97821.1	92053.7	92245.9	82531.1	82414.7	90106.4	104708.6	82302.6	29583.2	30451.4	38722.6	34218.2
Q9CWW7	CXXC-type zinc finger protein 1	Cxxc1	660	76.1	8.27	176956.9	169128.4	151595.6	180909.7	166489.4	171321.0	187200.5	164673.3	188889.0	179157.6	160997.9	174520.5	122833.5	177501.6
P14901	Heme oxygenase 1	Hmox1	289	32.9	6.54	140457.5	132395.9	116238.7	145107.8	124204.8	124287.5	113179.7	120134.1	126827.5	130301.9	91493.4	99680.2	80569.7	83572.0
Q8VDP3	[F-actin]-monooxygenase MICAL1	Mical1	1048	116.7	6.05	66655.7	68275.4	67813.7	60475.8	71985.1	74561.7	80817.2	65763.0	61512.9	68949.6	73448.6	85423.8	81330.0	73880.1
A0A0G2JE99	Ig-like domain-containing protein	Iglc1	106	11.7	6.27	70308.2	56880.5	72391.6	52842.9	57862.8	52208.9	52823.1	70174.8	69220.9	80264.0	73619.1	56088.1	75512.6	49532.3
O70496	H(+)/Cl(-) exchange transporter 7	Clcn7	803	88.7	7.27	219287.9	265255.1	222947.7	258994.3	221983.9	233162.4	223449.8	241413.3	240074.0	245217.7	267874.7	363654.3	266781.4	302471.5
Q9CQY6	Ubiquinol-cytochrome-c reductase complex assembly factor 2	Uqcc2	136	16.3	9.19	189311.6	185799.5	191440.4	185252.6	182465.3	197676.4	187760.4	191704.8	196796.9	194516.9	854044.5	845825.8	859166.3	911563.9
Q80U58	Pumilio homolog 2	Pum2	1066	114.2	7.08	201280.1	182974.8	210069.2	202618.3	181642.1	204142.4	193649.4	205317.4	195991.8	201632.9	#N/A	#N/A	#N/A	#N/A
Q7TNE3	Sperm-associated antigen 7	Spag7	227	25.9	7.43	136261.1	151102.5	150726.5	154148.3	138169.3	140459.8	134881.8	139948.5	149966.0	136994.4	57485.6	71518.2	71641.8	65557.3
Q78WZ7	DNA-directed RNA polymerase I subunit RPA43	Twistnb	330	36.7	6.21	60116.5	78011.4	75526.1	63964.1	76544.2	82389.2	67849.3	70458.4	71661.3	78338.5	154305.5	165541.1	159346.4	149517.2
Q8K094	Poliovirus receptor	Pvr	408	44.6	8.06	144695.0	223211.7	127813.7	154967.2	207435.6	145744.0	138568.7	130091.6	131265.9	162116.0	183509.4	209164.1	194689.9	199222.9
P54310	Hormone-sensitive lipase	Lipe	759	83.3	6.95	143913.1	131371.7	134450.5	132362.1	147018.7	132247.4	133258.3	129172.0	115931.2	120352.5	134483.7	134140.9	114970.5	129650.9
Q8BMA3	Connector enhancer of kinase suppressor of ras 3	Cnksr3	555	61.8	9.01	41160.1	52746.7	41561.8	38855.4	54280.0	44386.8	49993.0	47248.2	41153.5	52888.4	45682.8	38954.1	38445.2	42021.5
P62858	40S ribosomal protein S28	Rps28	69	7.8	10.7	1024313.8	1022830.5	1052606.2	978721.0	1022155.6	973799.4	901599.0	1042504.2	1027072.4	1001250.0	2899472.8	3148183.6	3372984.9	3244634.9
Q9D2G5	Synaptojanin-2	Synj2	1434	158.4	7.5	57627.9	57070.4	69552.1	58406.7	56641.7	58239.4	54703.6	60918.8	58287.1	57481.9	62677.3	58217.2	54217.0	62418.7
P63271	Transcription elongation factor SPT4-A	Supt4h1a	117	13.2	8.06	355377.4	363231.6	371374.0	372645.0	389712.6	384433.8	365441.0	360004.6	379974.0	399527.7	536906.4	527015.0	574210.6	576237.9
Q99LB0	Deoxynucleotidyltransferase terminal-interacting protein 1	Dnttip1	328	36.8	9.07	127366.8	146452.9	142435.0	135467.0	135645.8	132178.8	124035.9	115765.4	135372.4	140264.0	74411.3	88227.7	86985.8	86263.4
A2A654	Bromodomain PHD finger transcription factor	Bptf	3036	333	6.83	229833.4	260620.4	228105.3	244621.1	260426.1	282076.9	248958.9	241858.2	240949.3	254867.3	126582.4	127511.1	122419.5	128254.9
Q9DCU6	39S ribosomal protein L4, mitochondrial	Mrpl4	294	33.1	9.82	426732.6	420631.9	363655.7	394938.3	392570.6	390170.8	402279.2	375586.1	393219.4	396357.0	394361.4	379314.9	340065.7	338953.3
Q8BRM2	RAB6-interacting golgin	Gorab	368	41.5	7.36	66364.7	70031.1	63208.9	66915.2	69579.7	71322.2	63667.1	73428.0	64567.6	65329.0	206348.6	198388.1	194586.8	192236.8
Q9ET26	E3 ubiquitin-protein ligase RNF114	Rnf114	229	25.7	7.03	104789.6	121465.4	146637.3	138038.7	128898.1	121773.3	102584.6	119188.7	114370.5	118989.7	185300.1	266921.2	179453.5	208880.9
Q9DBU3	Serine/threonine-protein kinase RIO3	Riok3	519	58.7	5.69	128735.3	133744.4	135585.0	131249.1	125447.8	128342.9	117036.3	132772.4	125302.1	126214.3	216447.7	227489.9	215239.7	222093.1
Q922R1	UPF0183 protein C16orf70 homolog	MGI:244304	422	47.4	7.74	44931.7	48872.3	41641.0	40362.6	42837.9	48941.4	49851.2	39488.8	45606.4	43508.6	38614.6	36244.5	33410.5	39315.7
Q60855	Receptor-interacting serine/threonine-protein kinase 1	Ripk1	656	74.8	6.47	190510.5	156706.1	163634.0	165138.7	159180.8	167355.7	176282.2	170183.6	168453.2	183842.2	185740.2	187439.6	180356.6	183266.4
Q80ZS3	28S ribosomal protein S26, mitochondrial	Mrps26	200	23.4	9.96	119426.1	125416.0	92161.2	113173.6	110981.4	109475.0	116655.4	109091.4	120147.1	115420.0	185001.8	170494.8	168106.3	173050.8
Q8CFG4	Doublesex- and mab-3-related transcription factor A1	Dmrta1	490	52.6	9.44	116017.5	116343.5	96883.0	100651.2	106599.9	114015.6	105879.9	109046.6	110618.1	115209.9	16003.9	12885.3	10579.2	13611.9
P97304	DNA-directed RNA polymerases I and III subunit RPAC2	Polr1d	133	15.1	6.9	113959.4	110177.5	98833.3	104951.2	105522.0	101921.2	89525.7	92403.0	109155.7	106630.1	161774.9	159267.2	139712.0	151333.8
Q7TMY4	THO complex subunit 7 homolog	Thoc7	204	23.7	5.67	115177.3	125383.5	124799.2	120777.3	137392.1	128875.1	125454.3	119961.7	129758.0	128076.6	1040268.1	983413.6	993863.7	998987.2
Q9D8C4	Interferon-induced 35 kDa protein homolog	Ifi35	286	31.9	5.87	133702.4	120548.1	131941.4	137489.5	132795.7	147386.3	159412.0	145524.0	134958.5	136155.2	291873.3	267547.9	276261.2	279330.4
Q99JB8	Protein kinase C and casein kinase II substrate protein 3	Pacsin3	424	48.6	6.1	42438.3	41319.4	37790.0	39580.3	48961.6	49210.1	45145.2	39420.9	40647.5	38527.5	99621.2	101358.1	84363.3	91112.0
Q9JLV2	Short transient receptor potential channel 4-associated protein	Trpc4ap	797	90.7	7.59	67195.6	60937.1	61650.5	66194.5	60297.5	63412.3	62484.0	57154.9	64080.9	65624.5	250610.7	276921.2	247325.2	262664.8
Q3UG98	N-acetyltransferase 9	Nat9	241	27.6	5.27	386059.2	360661.5	401231.7	410147.0	467835.5	496957.5	411082.3	415351.8	510175.0	501835.8	100544.8	106420.3	101073.5	111728.6
P97457	Myosin regulatory light chain 2, skeletal muscle isoform	Mylpf	169	18.9	4.92	144345.8	121016.3	111169.0	133650.6	126349.9	145455.5	142064.3	602760.9	153123.8	137435.4	144403.8	147749.5	132053.1	146093.6
Q9DBB9	Carboxypeptidase N subunit 2	Cpn2	547	60.4	5.88	258179.6	232259.1	217097.5	220887.5	181848.7	220614.2	245616.6	208795.7	216408.9	224954.2	445896.9	476401.0	429329.0	428705.6
Q9QYI6	DnaJ homolog subfamily B member 9	Dnajb9	222	25.6	8.28	36273.4	49435.4	43382.7	52792.8	46513.6	41430.7	36372.6	37770.4	42142.0	39207.9	56973.3	75940.8	74335.9	77657.9
Q8R322	Nucleoporin GLE1	Gle1	699	79.5	7.61	92940.8	97701.8	80367.0	89827.0	88504.6	90976.7	92745.7	89385.5	90252.4	90459.9	31259.8	32987.9	32186.6	36174.8
Q9D5Y1	Coiled-coil domain-containing protein 39	Ccdc39	937	110	6.6	60095.2	95973.4	95015.4	130088.2	40692.1	45157.7	47136.4	52229.3	75707.2	67768.3	213743.4	263176.9	270886.0	346169.9
B2RXC2	Kinase	Itpkb	942	102.6	8	208824.9	213629.1	203228.9	209960.4	205067.4	204531.5	209543.2	205406.8	213396.1	211721.3	100557.9	105380.8	98299.7	117212.8
Q9JJ89	Coiled-coil domain-containing protein 86	Ccdc86	426	46.5	9.64	186921.2	218326.6	213975.6	210657.7	249286.6	218562.5	210156.6	207679.1	213973.0	211092.5	164234.1	172717.3	162356.7	170861.4
Q4VAE3	Transmembrane protein 65	Tmem65	234	24.9	7.78	248457.4	237408.3	235665.9	216942.2	265008.0	253621.0	253238.9	240433.9	248657.9	242975.3	263579.0	295466.7	306656.4	332289.7
A3KGV1	Outer dense fiber protein 2	Odf2	830	95.5	7.61	92199.7	90864.4	109240.2	100062.1	103491.5	105383.4	100776.9	91064.2	103332.7	104103.6	#N/A	#N/A	#N/A	#N/A
Q8CBY3	Leukocyte receptor cluster member 8 homolog	Leng8	785	86.7	9.32	89050.3	97437.8	86782.2	80467.7	97459.3	101901.9	91512.5	83234.9	93762.4	102649.3	45910.9	43893.1	41336.8	48821.4
Q64516	Glycerol kinase	Gk	559	61.2	5.87	33915.8	32133.0	35317.7	48396.3	34598.8	42372.9	56994.3	52173.0	35968.8	29063.2	132765.1	175128.7	181170.5	289101.6
Q60718	Disintegrin and metalloproteinase domain-containing protein 2	Adam2	735	82.3	6.2	114468.0	141607.8	130227.9	171211.5	92778.1	86997.9	82182.4	91051.6	114706.5	119798.2	89486.2	131385.4	126855.5	154748.7
Q505B7	Protein archease	Zbtb8os	168	19.7	4.56	403650.0	395854.3	380762.2	380730.5	407143.8	419782.4	387836.5	394316.7	381091.6	391396.5	373846.9	358406.2	353741.0	337428.0
Q3UPH7	Rho guanine nucleotide exchange factor 40	Arhgef40	1517	165	5.55	49459.6	43723.8	59143.7	57254.6	51518.9	61199.6	56932.6	55747.4	55081.5	53719.8	62473.5	60852.4	66403.4	64372.6
E9PWZ3	Ribosomal protein L3-like	Rpl3l	407	46.5	10.45	422124.0	412119.1	453551.1	425109.9	358471.0	361361.3	306629.8	365646.5	403320.9	403150.1	2474063.3	2633627.5	2645807.9	2590763.0
E9Q784	Zinc finger CCCH domain-containing protein 13	Zc3h13	1729	203.6	9.38	399913.3	409298.4	387363.9	390999.0	438388.4	446212.0	383698.0	378457.0	410709.3	425978.7	165606.9	225505.1	194189.1	218053.5
Q6GQV7	Erythroid differentiation-related factor 1	Edrf1	1239	138.7	6.14	99053.5	129432.8	101125.1	122836.7	116032.7	114239.5	104748.7	111176.3	112657.1	113676.3	62765.5	65011.9	58168.1	61007.8
Q924M5	Protein boule-like	Boll	281	30.8	7.47	314265.6	407971.5	404405.9	488113.0	281159.0	244299.4	238362.7	262044.3	341983.0	379285.9	343508.6	438352.9	462621.2	522017.0
Q9DAN8	Cystatin-12	Cst12	128	15	5.34	668712.8	659346.7	572096.3	601322.5	498876.4	430250.8	471829.7	659898.2	560609.8	556732.6	146836.9	155233.7	135938.8	154151.7
P58058	NAD kinase	Nadk	439	48.6	6.52	174503.1	173453.8	170120.9	172879.6	153307.1	160887.1	136715.9	148345.5	152664.0	171649.2	137862.3	150393.5	144966.1	147194.5
Q8BHY3	Anoctamin-1	Ano1	960	110.8	7.83	92743.8	118315.5	107838.9	106126.2	128376.6	122933.5	125000.8	109966.8	112180.6	114312.7	102036.7	113196.5	90320.2	108506.5
Q9D6M3	Mitochondrial glutamate carrier 1	Slc25a22	323	34.6	9.09	290141.1	315721.4	273308.2	293814.2	276045.2	284211.4	303811.2	282325.8	275695.3	304819.3	285984.0	284370.3	234369.5	282920.8
Q9D328	Transmembrane protein 35A	Tmem35a	167	18.5	9.99	1577762.2	1402178.3	1312694.3	1451917.9	1377641.9	1320282.8	1353915.4	1473756.6	1399924.5	1370994.6	820458.9	665800.3	669367.0	684734.9
Q6ZWQ7	Signal peptidase complex subunit 3	Spcs3	180	20.3	8.62	336571.6	353440.5	420166.7	425947.1	363774.2	358058.4	328997.3	488838.2	380019.1	379715.6	84424.8	93011.3	82494.5	95712.8
Q6PB97	F-box/LRR-repeat protein 19	Fbxl19	674	73.7	9.14	324614.7	295941.7	298614.9	308588.5	280252.0	258413.9	234706.1	295964.3	286736.7	291158.1	168857.1	173506.1	166189.7	170191.0
Q9WV98	Mitochondrial import inner membrane translocase subunit Tim9	Timm9	89	10.3	7.21	159066.3	195029.6	186209.2	171993.9	173062.5	169106.1	167172.3	178826.2	180522.9	170792.9	119058.7	135195.3	137809.2	130727.9
E9PW37	RAS protein activator-like 2	Rasal2	1286	144.5	7.46	133387.8	139114.6	126630.8	132164.0	130577.1	125856.8	121436.9	123655.5	125094.7	143168.2	188939.4	202542.0	177617.3	195727.2

Q3TLI0	Trafficking protein particle complex subunit 10	Trappc10	1259	141.5	5.96	73803.8	84617.3	61509.3	72557.0	71028.6	65081.6	59269.2	68064.6	67057.2	72135.2	207815.6	211155.7	192456.8	211259.6
G3X912	SprT-like domain-containing protein Spartan	Sprtn	497	55.3	8.9	145031.4	184307.8	175206.7	183244.9	152710.1	125653.8	125567.7	151731.1	158985.3	173105.9	45668.1	61979.6	63929.5	59238.7
Q8QZV7	Integrator complex subunit 13	IntS13	732	82.7	6.92	318861.0	327673.6	318403.3	306774.0	348652.2	366779.2	329828.5	288990.2	337204.6	324945.3	258652.1	267016.2	281408.3	262520.6
E9PWR4	DMAP-interaction domain-containing protein	Dip2c	1557	171	7.62	144485.5	161547.2	139953.1	150922.8	155282.1	154247.6	146480.3	131401.7	160850.7	152849.8	115944.2	121014.9	112027.4	108823.7
Q9CQ40	39S ribosomal protein L49, mitochondrial	Mrpl49	166	19.1	9.5	97457.0	111205.1	106960.5	109781.5	105875.1	109143.5	108140.2	110068.0	115740.4	109638.9	178172.0	175391.3	184487.4	175014.0
Q9DCH2	Ribonuclease P protein subunit p20	Pop7	140	15.8	8.69	39944.2	48337.8	41557.3	41731.9	45354.8	42392.2	41652.6	42111.0	46698.2	40031.1	#N/A	#N/A	#N/A	#N/A
Q8R0S2	IQ motif and SEC7 domain-containing protein 1	Iqsec1	961	107.9	7.14	68453.9	64767.3	59921.0	60421.8	65940.3	73058.4	54877.6	60628.0	65808.6	63316.5	170579.1	190719.4	164241.0	176955.3
Q3UCV8	Ubiquitin thioesterase otulin	Otulin	352	40.3	5.68	341628.7	390068.2	368311.4	375975.9	357435.1	353199.7	344503.8	325476.2	378635.4	401632.2	205366.3	221643.2	208230.8	205066.1
Q99LX5	Multiple myeloma tumor-associated protein 2 homolog	Mmtag2	260	29.3	9.86	33435.7	45719.7	41598.3	32336.0	37818.5	45016.0	34123.6	39227.5	36220.7	39493.2	#N/A	#N/A	#N/A	#N/A
Q80TI0	Protein Aster-B	Gramd1b	738	85.3	6.14	143980.6	142410.3	132111.9	133807.4	141467.5	129659.7	139953.8	133242.8	131389.1	140332.2	114039.1	130099.5	126146.5	127246.6
Q8BX22	Sal-like protein 4	Sall4	1067	113	7.81	172082.1	148859.5	171344.7	164668.9	205008.7	199700.3	172000.9	175836.2	173356.1	164750.6	57761.9	51717.5	50619.8	44227.2
Q6S9I3	HMW kininogen-II	Kng2	644	71.2	6.52	73375.8	103286.0	75598.7	93818.3	66465.9	96725.2	75266.3	101730.3	84984.8	80061.4	#N/A	#N/A	#N/A	#N/A
P39429	TNF receptor-associated factor 2	Traf2	501	56	6.96	90707.0	103085.3	89362.8	91906.7	91319.0	89036.9	89926.7	81238.8	86869.0	88750.0	63169.3	69238.2	68314.8	63209.7
E9PV87	Protein TALPID3	Talpid3	1520	166.6	6.32	35574.4	42532.3	34330.3	40990.7	36969.9	34953.4	32435.8	29270.3	33949.9	39031.4	43289.6	48739.3	34917.4	36488.1
P35441	Thrombospondin-1	Thbs1	1170	129.6	4.96	31794.0	38162.7	28662.5	30900.7	32307.6	34651.9	30273.1	36885.8	31220.9	30911.8	#N/A	#N/A	#N/A	#N/A
Q9JLJ4	Elongation of very long chain fatty acids protein 2	Elovl2	292	34.2	9.26	78321.2	87885.6	75506.4	87207.1	79961.9	89298.4	75797.6	70898.6	86100.7	87954.7	76017.4	102262.4	81320.8	79277.9
Q9Z0F8	Disintegrin and metalloproteinase domain-containing protein 17	Adam17	827	93	5.94	117369.0	121235.8	110811.0	112805.1	120451.1	115634.9	120926.8	113046.2	115468.7	126447.3	45572.3	50506.0	33823.4	39544.6
Q61214	Dual specificity tyrosine-phosphorylation-regulated kinase 1A	Dyrk1a	763	85.4	8.75	230633.2	241169.6	257987.5	244715.7	269055.3	261737.6	206503.2	228586.5	245852.4	239249.3	326004.2	359013.4	355718.8	318453.7
P98203	Armadillo repeat protein deleted in velo-cardio-facial syndrome homolog	Arvcf	962	105	6.64	277898.3	366254.1	299277.5	264220.0	1793161.2	404734.3	268560.8	398215.9	472341.7	260075.5	203779.8	283232.3	193256.3	249879.1
O70481	E3 ubiquitin-protein ligase UBR1	Ubr1	1757	200.1	6.05	62267.0	55240.2	64709.1	57123.1	55066.2	58942.9	51397.4	55353.9	59389.5	52798.0	264097.8	274783.5	280702.0	266156.0
P97864	Caspase-7	Casp7	303	34	6.33	17299.5	28539.3	22735.6	24799.1	30764.2	29640.2	26229.1	25893.8	25523.9	27023.0	154430.1	176421.7	157670.3	161357.0
O35704	Serine palmitoyltransferase 1	Sptlc1	473	52.5	6.4	34669.3	37967.6	35015.5	38459.4	38299.4	40668.9	36873.7	34346.8	37904.9	38156.5	70819.6	77021.9	78757.7	87053.5
Q9QY36	N-alpha-acetyltransferase 10	Naa10	235	26.5	5.64	39009.9	40623.3	37464.4	32670.8	37340.8	39680.4	40996.0	40389.2	47404.7	38867.5	#N/A	#N/A	#N/A	#N/A
O88907	E3 SUMO-protein ligase PIAS1	Pias1	651	71.6	7.28	1565.2	5001.2	4318.0	3527.4	4076.5	6947.0	4001.5	3017.8	3826.8	3889.0	7841.0	8302.7	7085.5	6671.1
P48025	Tyrosine-protein kinase SYK	Syk	629	71.3	7.88	70532.7	74303.2	70576.0	67133.2	73381.4	78694.5	80456.8	67504.8	66412.9	69964.2	108279.8	130163.9	107147.5	109129.6
Q5F201	Cilia- and flagella-associated protein 52	Cfap52	620	68.2	6.81	179212.5	239325.3	211017.5	287041.6	126406.4	110912.9	129186.3	126297.0	175239.0	193593.5	96559.6	157844.1	141386.0	211675.9
Q9CWE0	Mitochondrial fission regulator 1-like	Mtfr1l	289	31.7	6.1	233732.4	250550.1	239000.6	242798.0	230185.7	256876.9	231827.2	218028.0	238063.8	231834.0	507518.9	453075.5	415850.2	456800.6
P52633	Signal transducer and transcription activator 6	Stat6	837	93.4	6.23	133704.7	126450.1	97368.0	134597.9	124925.0	124796.6	122921.1	128368.1	135478.1	133140.1	146159.2	167830.1	145626.0	138520.6
Q8R3T5	Syntaxin-binding protein 6	Stxbp6	210	23.7	9.19	185390.3	189535.1	197146.1	154649.4	223912.6	231323.2	234418.1	192201.8	201592.4	210827.8	175860.1	166858.7	184190.3	148288.3
O08992	Syntenin-1	Sdcbp	299	32.4	7.15	48555.2	50098.3	54803.3	51514.7	52234.6	57297.4	46381.7	50581.6	52942.3	47695.7	76570.6	86300.2	76822.8	81363.5
P02802	Metallothionein-1	Mt1	61	6	7.96	81056.7	94819.4	88630.8	103883.0	47787.9	37627.0	40929.3	77021.4	69240.6	79012.3	142328.8	143568.0	143622.2	156323.9
Q9JLI0	Aldo_ket_red domain-containing protein	Akr1c12	323	37	6.64	91837.6	106223.5	96309.5	117313.3	92673.3	94595.8	126579.3	88125.6	98886.2	101909.2	#N/A	#N/A	#N/A	#N/A
Q8BP71	RNA binding protein fox-1 homolog 2	Rbfox2	449	47.3	6.55	52863.6	66222.8	63986.0	66454.0	63217.5	81465.1	61360.1	56328.9	65131.0	55410.0	32116.6	39925.5	27146.4	33612.3
Q8C460	ERI1 exoribonuclease 3	Eri3	337	37.2	8.07	222351.5	246131.8	250360.0	244252.4	246882.9	246532.8	280332.2	243551.6	256578.9	271140.2	561876.1	543786.3	565673.7	555905.5
Q8VE38	Oxidoreductase NAD-binding domain-containing protein 1	Oxnad1	311	34.7	8.13	466236.4	564248.1	445283.9	472240.0	495133.1	582015.3	443756.2	507948.1	518200.9	477695.8	233080.4	229800.5	244179.5	234696.5
Q80UY1	Carnosine N-methyltransferase	Carnmt1	400	46.3	6.16	250252.6	235678.8	239361.9	232476.8	249056.1	252268.1	221058.7	255442.4	245944.7	242809.7	213703.5	211919.9	203439.6	209655.3
Q8VE08	F-box only protein 33	Fbxo33	562	63.3	8.05	123911.2	138830.8	117686.5	126126.9	120255.3	118625.8	102896.8	111415.1	112093.9	126150.9	53183.0	62312.4	44955.6	62816.0
Q9ER99	Regulatory solute carrier protein family 1 member 1	Rsc1a1	582	61.2	4.78	18938.2	33623.4	22252.5	26344.4	27986.0	32507.8	23161.8	28197.3	29266.6	27985.8	31006.9	29667.4	29512.9	34204.5
Q9DB96	Neuroguidin	Ngdn	315	35.6	9.58	46607.4	41892.1	51963.8	43212.6	55451.8	49731.6	48116.9	54874.3	48703.1	45120.7	#N/A	#N/A	#N/A	#N/A
Q5SWT3	Solute carrier family 25 member 35	Slc25a35	300	32.6	9.52	174195.4	175993.3	155756.5	181574.4	158149.0	156253.5	138883.6	157059.2	162602.3	150304.9	191039.9	188506.6	183239.5	191760.2
Q9JIZ9	Phospholipid scramblase 3	Plscr3	296	31.8	6.38	172215.7	170980.8	164125.5	161033.8	178219.6	181791.6	182859.0	161811.8	162183.0	177952.3	65913.3	82730.3	77587.0	68451.4
Q6NZL1	DEAH (Asp-Glu-Ala-His) box polypeptide 37	Dhx37	1150	128.2	8.79	54648.6	64959.2	62135.7	59723.5	56799.0	60563.9	54325.5	54996.7	64956.5	61794.1	20460.7	30365.9	26367.4	28867.0
Q9Z1Y4	Thyroid receptor-interacting protein 6	Trip6	480	50.9	7.31	36550.8	44962.5	45297.9	42657.6	42845.6	43978.6	43994.6	43176.8	48290.6	41008.2	163138.9	170340.9	151082.0	125482.4
Q9D7B7	Probable glutathione peroxidase 8	Gpx8	209	24.1	9.36	323917.7	346192.8	299165.7	337697.5	306355.3	306069.6	312932.7	391267.6	306672.1	297262.5	128513.8	125310.1	123986.2	118665.1
Q8BJL1	F-box only protein 30	Fbxo30	746	82.6	5.26	115628.7	126413.7	122847.6	127125.2	117885.9	128181.9	120824.9	121254.7	132518.7	129252.1	106855.9	110117.6	93868.5	112018.2
Q63810	Calcineurin subunit B type 1	Ppp3r1	170	19.3	4.81	211600.1	183896.1	189850.1	171946.9	203113.1	186818.8	241818.7	190014.2	212889.2	187609.4	427548.1	313445.6	405377.1	325954.7
Q3UH53	Protein sidekick-1	Sdk1	2193	240.2	6.77	58447.0	55053.5	63443.6	69563.8	68170.1	66587.1	59346.1	51159.4	72502.2	51736.2	#N/A	#N/A	#N/A	#N/A
Q6PGH0	Ubiquitin domain-containing protein 2	Ubtd2	234	26.1	5.83	60393.0	62799.9	63333.7	57752.9	65330.1	59655.2	57506.7	61336.1	67048.4	64464.1	90657.9	90503.7	89224.8	94511.8
O88522	NF-kappa-B essential modulator	Ikbkg	412	47.9	5.85	53522.3	67132.1	55143.7	63226.7	54941.9	49128.3	66431.2	55563.8	51526.3	63858.0	159197.4	243959.1	157962.6	194051.4
Q8BMK1	tRNA N(3)-methylcytidine methyltransferase METTL2	Mettl2	389	43.9	6.2	32540.1	30868.3	39403.6	34610.2	37267.7	41894.8	33490.0	43822.2	37546.8	35730.6	116869.1	127866.5	125415.0	125251.6
Q62559	Intraflagellar transport protein 52 homolog	Ift52	426	48.2	5.2	141064.0	159130.4	164542.7	170391.2	132212.7	126259.3	137848.3	133612.8	136537.8	147168.4	181654.8	218268.3	209594.0	267565.6
Q8R3P7	Clusterin-associated protein 1	Cluap1	413	47.9	4.84	137246.1	160090.2	148979.1	172271.5	129114.2	142079.3	127187.6	120055.5	134038.9	124492.3	143259.2	120153.8	130916.7	165145.1
Q8K3Y3	Protein lin-28 homolog A	Lin28a	209	22.7	8.53	108258.2	96645.1	113529.6	93391.7	88295.1	93986.5	98279.4	99460.3	116030.6	132297.9	95702.9	89046.8	99227.7	88688.0
Q5DTT3	Protein TASOR 2	Tasor2	2382	264.1	6.44	114623.1	116196.0	110468.1	108419.5	130806.3	129602.6	108111.2	104636.9	113474.0	117083.8	182612.8	184228.1	168275.3	176636.8
Q9EPS3	D-glucuronyl C5-epimerase	Glce	618	70	8.85	65339.3	69611.3	70925.1	71415.4	61142.3	69326.1	65447.9	71279.5	66834.4	68376.6	410669.6	407215.5	388655.2	396473.6
Q6P5F6	Zinc transporter ZIP10	Slc39a10	833	94.3	6.71	84010.4	94597.0	98575.7	81138.9	92992.4	107234.3	91142.8	96365.6	99002.6	103794.1	72621.6	67383.4	71779.1	67776.4
Q9JKX6	ADP-sugar pyrophosphatase	Nudt5	218	24	5.48	375972.8	429341.7	388923.6	388581.1	416818.1	424563.4	380262.2	364767.6	404141.1	411174.6	224856.9	213162.7	243677.7	220413.1
Q5U4C3	Splicing factor, arginine/serine-rich 19	Scaf1	1256	133.8	9.45	49051.1	60334.6	53784.9	50770.7	60401.5	61551.0	58615.7	59815.2	56279.8	60906.0	233199.7	239129.8	257512.1	237166.4
A0A140LIQ5	Predicted gene 15262	Gm15262	728	80.8	4.32	14325.5	16596.4	16387.5	16406.9	20276.9	18767.0	15278.0	14459.9	18351.7	16223.0	#N/A	#N/A	#N/A	#N/A
Q8VE95	UPF0598 protein C8orf82 homolog	/IGI:192594	218	24.3	8.7	126831.3	129457.8	111651.4	131018.6	121421.7	136754.0	114463.1	122194.4	129393.4	125583.3	159252.3	170034.0	153017.0	166237.6
Q91X51	Golgi reassembly-stacking protein 1	Gorasp1	446	46.9	4.65	35769.2	45677.2	39248.9	37785.8	34182.6	37451.0	28830.0	38499.7	44479.0	42743.6	11210.0	10690.5	10134.4	5710.8
Q6P4S6	Serine/threonine-protein kinase SIK3	Sik3	1311	145.7	6.92	215539.8	229702.6	223258.8	218535.9	223784.8	226499.5	229317.6	226139.4	228755.3	222795.9	54055.4	74703.2	50656.3	53728.4
F7CD45	Predicted gene 8334	Gm8334	553	57.3	11.11	131002.1	158867.7	127340.7	127401.5	141892.4	145449.0	125762.3	114225.0	142179.8	145197.7	18697.5	22091.5	21706.9	17971.2
Q9CQF6	L-aminoadipate-semialdehyde dehydrogenase-phosphopantetheinyl transferase	Aasdhppt	309	35.7	7.14	170430.0	255201.5	182644.0	217756.4	196317.9	183495.9	161319.7	187736.1	182350.4	201604.2	175713.5	191009.4	202308.3	198927.1
P12849	cAMP-dependent protein kinase type I-beta regulatory subunit	Prkar1b	381	43.2	5.96	194288.7	248360.2	209468.4	223862.4	180187.2	212007.0	141326.5	203843.2	186770.2	193741.6	#N/A	#N/A	#N/A	#N/A
Q5PR68	Centrosomal protein of 112 kDa	Cep112	954	112.6	6.8	102014.3	106088.8	93843.5	104187.2	97464.4	96819.8	94671.2	87919.8	106139.3	107225.2	101981.3	113329.5	112808.5	113385.3
Q80VE5	Rab-GAP TBC domain-containing protein	Tbc1d22b	505	59.1	7.43	155357.8	173549.4	147701.2	167766.3	168311.6	177094.7	149148.4	153670.6	160259.6	166330.3	#N/A	#N/A	#N/A	#N/A
Q9D6U8	Protein FAM162A	Fam162a	155	17.7	9.88	246609.8	216595.4	249629.8	280359.8	255524.6	258144.8	241273.5	244572.2	238979.8	188164.3	414261.4	407731.1	438451.4	446673.9
P33215	Protein NEDD1	Nedd1	660	71.2	7.84	93736.6	91062.8	95125.6	99909.7	102721.5	102870.8	91173.9	94689.2	89510.4	95095.9	40189.4	50218.7	38005.6	47068.8
Q5RJG1	Nucleolar protein 10	Nol10	687	80	8.19	179741.3	196818.6	148149.2	159908.7	168024.4	180586.4	159401.9	183588.5	160206.1	182835.5	210184.3	240273.1	206848.4	200522.3
P62342	Thioredoxin reductase-like selenoprotein T	Selenot	195	22.3	8.6	169550.2	157832.5	168441.7	176587.7	166401.1	175042.5	182363.2	165067.4	175705.8	173315.9	76231.9	73308.3	89397.9	82977.8
Q3ULW8	Protein mono-ADP-ribosyltransferase PARP3	Parp3	533	59.9	7.12	71980.2	74812.4	65667.7	70911.1	81996.2	83381.8	85433.0	67598.2	69115.7	67264.6	81006.0	89434.8	75269.2	91715.1
P49962	Signal recognition particle 9 kDa protein	Srp9	86	10.2	7.99	236365.3	284930.2	293193.8	303495.9	278930.2	251011.6	239702.9	252447.1	292841.3	263628.0	305335.6	386119.5	369833.7	364138.0
O08919	Numb-like protein	Numbl	604	64.1	8.82	207977.2	204181.8	183110.2	187061.6	217557.5	220718.4	206743.7	197088.9	206014.1	214387.9	191019.8	233664.5	204710.7	223601.2
Q8CIH5	1-phosphatidylinositol 4,5-bisphosphate phosphodiesterase gamma-2	Plcg2	1265	147.5	6.77	94546.2	114769.6	98106.2	122247.8	109067.5	106851.1	93450.9	108627.4	108956.5	116160.4	82527.3	83112.2	86739.8	76649.6
Q6ZQ82	Rho GTPase-activating protein 26	Arhgap26	814	92	6.68	90350.8	103639.7	81784.4	92962.3	103302.8	99686.5	91949.1	104367.8	99491.3	99261.3	219788.8	218946.7	197295.1	204072.3
P61804	Dolichyl-diphosphooligosaccharide--protein glycosyltransferase subunit DAD1	Dad1	113	12.5	7.08	132074.8	124305.0	135280.0	146448.5	130582.2	126212.1	121219.9	124714.6	126012.8	134197.8	422832.5	449242.7	425287.4	503929.5
Q8C0M0	GATOR complex protein WDR59	Wdr59	992	111.7	7.85	96314.3	111350.7	109155.3	100503.1	108950.4	110244.3	90807.6	100618.4	107209.2	96946.9	107114.7	123600.1	111783.8	113202.6
Q9Z1P7	KN motif and ankyrin repeat domain-containing protein 3	Kank3	791	84.1	5.2	116895.8	121342.4	99790.8	105482.4	132691.5	123312.8	136227.7	111977.4	121610.8	121789.3	129509.0	130520.9	127031.1	121582.5
Q8K0H5	Transcription initiation factor TFIID subunit 10	Taf10	218	21.8	6.55	110017.2	127366.0	134977.1	123526.5	138893.9	138118.7	115968.9	131394.8	133548.3	126734.9	49318.2	61933.2	54191.7	69233.2
Q8CHP5	Partner of Y14 and mago	Pym1	203	22.7	9.5	55068.9	66758.4	57953.0	63623.2	54361.5	53777.0	57265.4	50097.9	52987.4	65960.1	13827.3	18969.1	13961.3	14610.1
Q9R008	Mevalonate kinase	Mvk	395	41.9	6.68	72490.5	68057.6	64052.6	74917.5	64480.6	54532.5	60798.7	63629.8	63915.3	69592.3	59181.2	53571.0	62202.0	54495.7
Q99KG5	Lipolysis-stimulated lipoprotein receptor	Lsr	594	66.1	6.54	84164.0	71055.3	68692.3	65121.4	79659.4	84057.0	92775.3	70190.9	72606.0	72965.4	29899.6	24308.6	23259.5	24667.6
Q9CR51	V-type proton ATPase subunit G 1	Atp6v1g1	118	13.7	7.97	33600.1	46587.1	42885.0	48055.6	37594.1	37019.0	44257.8	45247.5	41286.5	45831.6	178595.5	176699.2	201197.5	174298.3
Q8R3Y8	Interferon regulatory factor 2-binding protein 1	Irf2bp1	584	61.7	8.18	234340.6	222125.8	238547.7	219309.5	234518.4	254759.6	252539.7	225374.4	227151.8	223056.6	164276.8	157866.1	136935.2	158786.9
Q6A078	Centrosomal protein of 290 kDa	Cep290	2472	288.9	6.1	69218.5	73188.8	79145.0	68335.5	75781.2	70161.8	60997.5	66003.7	70081.7	76538.4	115910.4	113698.7	103227.3	114825.9
Q8R2K4	TAF6-like RNA polymerase II p300/CBP-associated factor-associated factor 65 kDa subunit 6L	Taf6l	616	67.2	8.92	83888.9	86659.2	78187.7	71655.1	84790.5	94075.4	79567.1	79427.0	84173.3	78662.0	40050.0	42138.2	41778.9	37770.7
Q9DC04	Regulator of G-protein signaling 3	Rgs3	966	106.2	5.86	278209.4	315151.9	303425.0	305793.7	286051.8	265561.2	242728.3	261938.6	305426.2	285555.6	127168.4	132949.2	112161.8	109303.8
Q9JL60	Glucocorticoid modulatory element-binding protein 1	Gmeb1	562	61	4.79	39356.5	46349.0	38086.9	44903.1	43119.5	47093.2	40186.3	39128.8	48462.9	41793.5	38986.4	61784.6	40938.5	42541.0
Q9EPV8	Ubiquitin-like protein 5	Ubl5	73	8.5	8.44	214539.4	210446.9	250247.5	233445.5	222207.7	226965.7	209708.6	232042.5	211491.7	218617.9	339920.5	364400.6	348204.2	372618.5
Q05895	Thrombospondin-3	Thbs3	956	104.1	4.64	44731.6	46997.0	51744.8	47554.6	54245.2	67579.0	54365.5	49997.9	48241.5	48576.2	50680.3	47879.2	47401.7	44530.3
Q921L3	Calcium load-activated calcium channel	Tmco1	188	21.2	9.74	469834.7	462490.3	391316.8	427820.7	452617.0	466690.9	414610.8	400924.6	453919.5	454649.2	146348.0	148109.6	136257.2	135431.7

Q99MW5	Testis expressed gene 13	Tex13b	186	21.7	8.97	191380.7	236179.0	170505.8	191165.9	211151.8	199620.1	179422.8	192919.9	198388.0	214412.2	172614.2	199497.2	193905.3	204607.7
Q8R3H7	Heparan sulfate 2-O-sulfotransferase 1	Hs2st1	356	41.8	8.57	69253.5	65001.8	69457.1	66521.1	80406.4	77512.7	70506.9	67754.8	69925.5	70673.3	144857.0	141462.3	123836.3	139161.5
O88559	Menin	Men1	611	67.5	6.39	148227.2	144798.0	148069.3	135623.8	156886.1	158991.7	144471.1	129860.7	155420.5	163970.6	98744.3	105205.0	104611.5	103210.0
Q9WTL7	Acyl-protein thioesterase 2	Lypla2	231	24.8	7.23	498829.6	444574.8	434357.8	442120.3	430737.6	450265.6	471404.2	447611.6	463267.8	436373.9	537232.6	560084.5	520325.3	554099.2
Q9JL56	Glycerophosphodiester phosphodiesterase 1	Gde1	331	37.6	6.9	405595.6	374033.9	339118.5	368332.4	355777.4	343778.6	365164.2	361685.3	329828.5	339423.4	81142.0	103105.1	90651.2	91630.1
E9Q2Z1	Cat eye syndrome critical region protein 2 homolog	Cecr2	1453	161.4	6.77	126116.4	141136.7	130523.2	119425.5	159048.5	156884.7	130187.7	133017.3	137857.0	143253.6	#N/A	#N/A	#N/A	#N/A
G3XA30	Non-structural maintenance of chromosomes element 4	Nsmce4a	381	43.7	5.3	19927.1	29588.2	27005.5	31996.0	25493.8	25176.8	19940.9	26643.1	27509.9	29554.5	62527.8	70808.9	67850.9	78462.4
Q9CWD8	Iron-sulfur protein NUBPL	Nubpl	319	34.1	9.07	251157.7	279671.8	239148.9	248619.7	276733.8	264552.7	277669.7	245213.6	257815.3	267248.1	135708.4	131111.5	119918.3	131124.9
Q8BYN3	Inositol-tetrakisphosphate 1-kinase	Itpk1	419	46.1	6.29	141609.3	133365.2	138096.1	136821.5	150788.7	163686.9	159544.7	144487.3	156352.8	140906.6	46859.2	48247.4	46794.3	47873.0
Q6NZA9	Transcription initiation factor TFIID subunit 9B	Taf9b	249	27.2	9.58	162988.2	201320.7	164327.4	148798.1	190324.8	201788.6	145790.9	168445.5	156536.1	166535.7	#N/A	#N/A	#N/A	#N/A
E9Q6H8	Pleckstrin homology domain-containing, family A member 5	Plekha5	1269	144.3	7.39	275839.4	282038.7	283717.2	293028.5	278880.7	285472.6	292755.3	269731.5	258840.8	282977.1	343356.3	338541.2	336795.1	357630.0
Q9JIB4	General transcription factor IIH subunit 2	Gtf2h2	396	44.7	6.83	215014.3	247620.9	233819.9	257819.7	223899.0	218429.0	220658.8	192435.7	279140.7	288185.6	333076.9	369533.9	380476.4	385172.1
Q61846	Maternal embryonic leucine zipper kinase	Melk	643	72.7	8.72	142577.6	117162.2	118566.8	116257.6	135698.1	136402.2	137348.2	122015.0	135080.8	135286.3	194774.6	172982.1	173221.7	178868.4
Q8CFJ5	UPAR/Ly6 domain-containing protein	Lypd11	196	21	8.13	485427.3	761117.9	790663.3	1029003.2	390133.5	272177.6	227779.9	367348.1	560194.7	687332.4	357677.8	502112.1	508095.9	643231.8
Q9CY94	DNA replication complex GINS protein PSF3	Gins3	216	24.6	5.64	176368.2	195442.0	211946.8	207012.0	222714.1	213668.0	177846.7	186999.2	205146.0	198789.4	109770.2	121784.3	109725.6	95353.2
Q78Y63	Phosducin-like protein 2	Pdcl2	240	27.8	4.89	120067.4	173872.1	129890.0	140768.7	127317.9	119986.9	109485.8	126148.1	132603.9	137095.4	89294.3	118691.4	108291.0	105651.0
Q8C341	SUN domain-containing ossification factor	Suco	1250	139.1	5.02	68379.1	69555.7	65418.4	76427.5	77469.2	60912.2	61809.6	61881.5	75841.6	77273.3	33459.4	35909.0	37257.6	36734.4
P52483	Ubiquitin-conjugating enzyme E2 E3	Ube2e3	207	22.9	7.18	168895.1	157173.1	172431.7	148352.9	177210.0	173713.4	154402.4	155875.9	180247.2	143552.3	657352.9	588605.3	608386.6	532069.2
Q8CG50	Ras-related protein Rab-43	Rab43	210	23.2	5.86	104888.9	102417.3	98031.7	112535.7	83089.5	79051.2	70538.1	78095.3	85802.3	91472.3	189455.2	202100.4	158719.1	191832.0
Q924H7	WW domain-containing adapter protein with coiled-coil	Wac	646	70.6	9.45	56805.4	49647.2	46612.0	43697.3	54699.2	52731.2	49452.1	41955.2	48462.7	42958.1	32022.3	35443.9	39312.2	31499.2
Q91VL8	Telomeric repeat-binding factor 2-interacting protein 1	Terf2ip	393	43.3	4.81	51476.0	61242.8	66523.2	67291.8	69358.4	68785.2	64986.3	61469.6	59738.2	61887.8	54298.1	52703.1	57594.6	55460.1
Q6NXW6	Cell cycle checkpoint protein RAD17	Rad17	688	77.3	6.14	100499.3	109233.8	98974.2	97421.9	105906.6	108808.8	99642.8	88481.9	103898.2	115163.7	209021.1	227873.8	210001.7	215050.0
Q7TMV3	FAST kinase domain-containing protein 5, mitochondrial	Fastkd5	762	86.5	8.38	75878.4	72690.7	69925.2	72025.7	75566.8	73472.8	68859.2	68478.1	68200.0	70755.2	80354.3	84086.3	95307.5	82273.3
P70265	6-phosphofructo-2-kinase/fructose-2,6-bisphosphatase 2	Pfkfb2	519	59.9	8.12	152214.9	143479.7	141703.7	136741.4	140497.0	134849.9	132109.6	155983.6	129010.7	135105.5	263503.0	275414.4	249838.8	243222.9
Q8R3I2	Lysophospholipid acyltransferase 2	Mboat2	519	59	8.35	99344.7	90968.6	87913.4	87568.0	85801.4	88901.5	88853.0	102850.0	86438.5	84318.5	132727.4	106416.5	112187.1	99926.1
Q9DAA6	Exosome complex component CSL4	Exosc1	195	21.4	8.24	780162.4	916904.2	766188.6	791599.2	922571.0	856397.0	880003.4	789638.3	944862.3	918388.9	367997.2	372618.0	368000.3	351197.7
Q8K0C1	Importin-13	Ipo13	963	108.2	5.3	50150.7	53906.9	49884.6	50617.7	49116.5	54269.7	44888.1	49033.4	55008.9	53300.0	67741.2	69885.6	64975.2	66404.5
Q99M04	Lipoyl synthase, mitochondrial	Lias	373	41.9	8.88	240941.3	257929.2	248917.6	227018.3	269015.6	248351.8	232403.3	241370.0	231019.0	235386.3	139502.0	138556.5	150980.3	137182.8
Q91X96	Guanine nucleotide exchange factor MSS4	Rabif	123	13.9	5.52	90540.0	84374.1	86515.9	87707.7	87917.2	92940.2	99984.7	84972.9	91130.2	83934.6	342958.9	303800.4	321393.3	326024.6
P59110	Sentrin-specific protease 1	Senp1	640	72.5	8.4	118342.3	128054.9	132062.1	121988.2	140057.5	138665.3	132284.4	123498.5	127576.7	125399.8	106458.6	125191.7	116196.1	115457.0
Q923S9	Ras-related protein Rab-30	Rab30	203	23	4.97	71975.9	77123.4	70713.4	73079.8	74664.6	75666.3	73820.1	62869.6	75421.8	77936.2	#N/A	#N/A	#N/A	#N/A
P50295	Arylamine N-acetyltransferase 2	Nat2	290	33.7	5.88	166161.5	180397.8	167987.0	178479.3	189491.5	181748.4	178889.1	167661.6	181180.9	188724.5	100233.4	104172.4	98702.0	104007.7
Q8BW49	Tetratricopeptide repeat protein 12	Ttc12	704	78.7	5.64	155388.1	234211.0	201377.9	289279.2	152932.0	154273.4	130835.6	149936.4	184089.0	208187.9	208601.1	228678.3	228664.5	286485.3
Q6NSQ9	Glucose-6-phosphatase 3	G6pc3	346	38.8	8.13	103904.3	102316.1	96147.4	106966.5	104843.1	106325.0	105594.4	102390.2	97444.7	109543.4	147118.2	170753.5	163775.7	170628.2
Q921C5	Protein bicaudal D homolog 2	Bicd2	820	93.3	5.44	99258.2	90995.8	105187.0	91823.7	108288.1	101551.6	103925.7	88926.9	90164.8	89786.4	250800.0	272182.4	220282.5	254897.3
A0A3Q4EGW0	Oxysterol-binding protein	Osbpl7	963	108.1	7.69	20597.8	18717.6	19630.2	15471.8	21038.7	18364.8	20013.9	18882.3	16598.7	19184.9	25706.6	27738.8	25790.6	25581.9
Q8BVE8	Histone-lysine N-methyltransferase NSD2	Nsd2	1365	152.2	8.51	310960.7	364784.2	337661.7	339431.1	406114.1	509705.8	301728.2	280725.0	332123.7	366538.5	98404.0	129065.2	125619.8	116859.5
Q8VCZ6	Small G protein signaling modulator 3	Sgsm3	750	85.4	5.87	21179.0	28455.1	26114.2	25204.5	25728.7	24965.4	21173.2	24799.8	22053.9	30455.0	228216.4	272608.7	270896.8	237697.7
Q9CZ62	Centrosomal protein of 97 kDa	Cep97	856	94.6	5	136957.4	143567.6	140297.8	141507.5	125015.0	125359.6	126528.6	127858.8	138508.2	148518.6	37816.0	41572.8	38148.4	44655.0
P70677	Caspase-3	Casp3	277	31.5	6.92	97558.3	103677.7	94639.9	99029.4	104233.1	100908.4	92712.2	101099.0	102224.6	110271.1	67431.8	74552.8	66530.0	68307.2
Q3TQB2	FAD-dependent oxidoreductase domain-containing protein 1	Foxred1	487	54.1	7.53	441966.4	428723.9	415596.1	380754.9	366373.7	348649.0	423204.2	364953.5	380843.5	415568.7	267127.2	284680.5	249996.1	263427.6
P05622	Platelet-derived growth factor receptor beta	Pdgfrb	1098	122.7	5.12	39315.3	42997.5	34689.6	39778.1	44502.0	49897.7	44707.5	39322.4	42003.0	43584.0	19580.9	16988.8	18927.7	18611.4
Q9QZD4	DNA repair endonuclease XPF	Ercc4	917	103.6	7.71	180737.2	238179.4	197397.2	230195.6	211759.2	232110.4	195061.2	184860.1	229526.9	210356.4	#N/A	#N/A	#N/A	#N/A
Q9CPT5	Nucleolar protein 16	Nop16	178	21.1	9.91	358485.1	391592.2	346920.7	346858.0	381328.6	411555.6	321995.4	339559.8	361000.5	409856.0	273915.2	301002.3	331029.9	313764.4
Q8BKI2	Trinucleotide repeat-containing gene 6B protein	Tnrc6b	1810	191.8	6.3	21106.2	8297.7	9591.2	11526.5	11361.2	14432.6	9988.7	11666.2	10766.3	10645.7	#N/A	#N/A	#N/A	#N/A
P61514	60S ribosomal protein L37a	Rpl37a	92	10.3	10.43	300524.2	325777.3	340233.7	327460.2	330335.2	301326.8	287100.1	329930.5	331696.7	330975.2	160492.1	183265.1	170125.0	171907.6
Q5U4F6	WD repeat-containing protein 34	Wdr34	535	57.9	6.52	59939.9	57327.3	64087.0	58170.3	59543.5	66638.2	54339.8	69704.0	55406.2	53183.5	81140.9	93444.7	84676.3	97222.6
Q9WTK2	Chromodomain Y-like protein	Cdyl	593	65.2	8.6	136343.8	144166.3	133265.6	116521.4	154185.4	141525.2	141225.6	129445.2	144079.3	139028.8	#N/A	#N/A	#N/A	#N/A
Q91W52	Transmembrane protein 19	Tmem19	336	36.3	7.72	465137.6	427252.1	451246.0	431886.0	444913.7	452728.1	478651.1	444607.1	417738.2	466593.2	382577.7	388223.6	345354.5	368299.1
Q8R3P2	Probable E3 ubiquitin-protein ligase DTX2	Dtx2	619	67.2	8.62	119130.1	143380.1	119671.4	127804.6	146736.3	140082.2	112098.1	124029.6	141282.7	143362.1	13926.0	24429.7	20702.8	20084.8
Q91YX5	Acyl-CoA:lysophosphatidylglycerol acyltransferase 1	Lpgat1	370	43.1	8.79	143592.3	139543.7	157453.0	158806.0	128312.4	142925.9	127964.7	141977.7	135314.3	138651.7	190912.2	204478.5	184882.6	214716.2
Q9DBU6	Serine/Arginine-related protein 53	Rsrc1	334	38.6	11.15	51377.1	58929.5	50541.7	53002.7	56919.8	50386.1	46054.4	47666.8	52795.0	58909.2	40299.4	57080.6	46799.3	54145.4
P28704	Retinoic acid receptor RXR-beta	Rxrb	520	55.8	8.27	260446.4	257820.2	258525.6	230618.9	277673.8	286427.1	269299.5	244029.4	265670.8	280812.5	285653.0	278616.9	283633.2	242443.7
Q8BGV4	TELO2-interacting protein 2	Tti2	512	56.7	6.96	76083.8	65672.4	75589.1	79548.6	91143.6	81444.6	77455.5	73586.3	80381.7	76412.4	52469.8	50369.3	53675.1	54335.5
Q07139	Protein ECT2	Ect2	913	103.1	7.44	144692.6	166755.7	170657.7	170029.2	164450.7	164955.9	135527.9	136693.6	157409.8	180701.3	101773.8	118897.5	105472.6	107205.2
O35309	N-myc-interactor	Nmi	314	35.2	5.05	87317.0	83745.0	77110.5	71361.4	78214.9	76773.1	74343.0	81244.4	78402.8	81842.1	#N/A	#N/A	#N/A	#N/A
Q8BMB0	BRCA2-interacting transcriptional repressor EMSY	Emsy	1264	135.2	9.38	95102.9	103344.2	100783.6	96173.4	115503.3	106307.1	107386.2	92196.9	98973.2	109583.6	64206.1	63044.3	64887.9	68713.4
Q9D2X5	MAU2 chromatid cohesion factor homolog	Mau2	619	69.5	7.25	221992.8	238117.9	195149.2	212326.3	218121.6	229090.7	207671.9	178292.5	227608.8	237691.5	197600.7	198058.8	151771.2	201042.6
Q91WQ5	TAF5-like RNA polymerase II p300/CBP-associated factor-associated factor 65 kDa subunit 5L	Taf5l	589	65.9	5.92	24259.4	31972.7	26333.3	24961.9	32703.7	33386.0	26626.7	26506.9	28589.2	29576.3	113958.1	100116.6	117788.1	95399.2
Q6IE21	OTU domain-containing protein 6A	Otud6a	290	33.7	7.34	93451.7	101131.6	94609.7	95082.6	98436.3	103009.2	86320.6	82346.1	96518.1	100059.4	127831.4	133691.1	122003.1	124790.1
Q8BVG4	Dipeptidyl peptidase 9	Dpp9	862	97.9	6.65	185638.0	172467.1	181015.6	164367.5	191720.4	178871.4	201062.6	179764.2	206357.3	204404.7	248992.4	286568.0	237910.7	281643.7
Q7TPM9	Myotubularin-related protein 10	Mtmr10	771	88.1	8.68	101552.4	103945.6	115405.1	98346.1	130416.7	121804.0	116126.4	116532.0	114518.3	110021.2	56487.4	52840.9	56755.7	50200.9
Q8BZS9	Putative pre-mRNA-splicing factor ATP-dependent RNA helicase DHX32	Dhx32	744	83.9	4.96	65064.5	62195.7	52431.4	56897.5	64721.6	62989.4	61317.0	52828.0	59878.7	68185.2	79189.1	73711.1	74042.8	77206.2
Q9D8S9	BolA-like protein 1	Bola1	137	14.4	8.76	78052.7	71061.8	65846.1	63057.6	69430.4	64603.0	73260.9	67447.6	69021.4	77252.8	179623.2	189276.7	174929.4	181964.4
O70572	Sphingomyelin phosphodiesterase 2	Smpd2	419	47.4	6.44	259240.2	254633.4	262280.8	252937.5	248921.3	238875.6	245805.0	245968.0	240345.5	262499.1	444841.1	416354.8	396975.4	428403.6
Q9CQQ4	Gem-associated protein 2	Gemin2	269	30.4	5.53	32480.4	36638.3	42334.1	40874.1	39437.9	37396.8	35381.3	39759.2	45576.5	37543.6	40491.6	50485.8	41052.9	41506.8
Q3UYH7	Beta-adrenergic receptor kinase 2	Adrbk2	688	79.6	7.69	84604.3	75672.7	76696.4	77092.9	68260.5	70302.1	65124.9	66707.2	75645.2	74635.5	#N/A	#N/A	#N/A	#N/A
Q8BLH7	HIRA-interacting protein 3	Hirip3	601	65.2	7.84	315580.1	381270.6	346611.9	385219.5	318156.9	325009.7	283260.5	313172.2	319747.1	356577.1	100339.7	102913.7	96530.7	104053.3
Q8C569	Protein FAM118B	Fam118b	351	39.5	6.86	258888.2	374769.3	304461.3	420617.2	227183.3	221806.1	213970.1	230994.3	288246.1	330066.2	248798.3	332320.1	264241.8	324514.2
Q9CXK9	RNA-binding protein 33	Rbm33	1231	137.2	6.46	58307.8	63234.9	72896.5	60654.5	71670.5	66142.9	58033.3	57001.0	60987.5	70556.0	37403.9	35520.2	42062.0	34812.7
Q08642	Protein-arginine deiminase type-2	Padi2	673	76.2	5.53	96500.5	122917.1	95162.9	105430.1	108942.2	130124.9	129187.5	104498.0	96961.1	113781.6	73208.3	78472.9	68335.8	77634.3
P55194	SH3 domain-binding protein 1	Sh3bp1	680	74.1	6.6	130220.6	134162.8	133804.0	121345.5	136986.0	152260.1	129233.4	136905.1	130617.8	118407.7	294527.4	294546.7	277113.2	260118.9
Q8BSN5	PHD finger protein 23	Phf23	401	43.5	5.8	155750.6	158941.4	134427.5	151476.9	131941.7	141453.6	124849.9	129761.4	149004.3	165119.0	204702.9	202822.7	185080.6	182516.9
Q91X88	Protein O-linked-mannose beta-1,2-N-acetylglucosaminyltransferase 1	Pomgnt1	660	75.1	6.67	127957.4	153908.0	133238.2	136182.8	134024.7	125032.5	103873.7	132621.2	122255.3	141948.4	54478.9	52369.2	52780.4	60298.1
Q9CQT2	RNA-binding protein 7	Rbm7	265	30.1	9.38	252245.3	253942.9	252704.2	227436.3	270657.1	265844.0	243021.5	238992.6	248109.3	244687.4	140077.8	154048.3	140005.7	136280.6
P70295	Ancient ubiquitous protein 1	Aup1	410	46.1	8.4	158320.2	182330.3	165906.1	187365.3	155619.7	148261.0	161245.1	157440.2	174754.7	172910.8	262414.4	269129.3	243142.9	264129.1
Q61542	StAR-related lipid transfer protein 3	Stard3	446	50.4	7.49	57384.1	55320.1	55544.6	51481.5	54253.3	57160.7	54458.5	53808.7	61065.0	59766.7	54784.8	54570.2	51424.7	49496.5
P35803	Neuronal membrane glycoprotein M6-b	Gpm6b	328	36.2	6.14	263032.6	233308.7	175338.0	251536.6	157376.4	149960.9	146550.0	162227.1	170901.8	183751.2	217333.6	224408.2	191185.8	226137.3
Q8C3X8	Lipase maturation factor 2	Lmf2	702	79.9	9.99	206983.6	213456.8	209199.1	198711.3	207010.0	217677.0	202252.9	194767.7	195510.5	191443.5	268008.3	230107.4	215698.4	241796.0
Q9DBE0	Cysteine sulfinic acid decarboxylase	Csad	493	55.1	6.61	124966.0	114692.6	97551.0	118844.0	116446.1	115959.0	131860.6	108815.9	111325.5	108674.8	139396.9	125031.1	105363.4	127355.6
Q6ZPG2	WD repeat-containing protein 90	Wdr90	1874	202.8	6.65	71810.9	74108.7	76100.3	76029.3	90962.9	79407.2	71906.3	74750.5	87089.5	86133.9	275038.5	300477.7	260310.6	272835.6
Q08274	Dystrophia myotonica WD repeat-containing protein	Dmwd	665	69.8	7.24	123608.8	120332.6	125989.0	126149.6	126852.2	126709.1	121032.9	115061.5	128367.2	119881.3	63938.5	62184.6	72842.0	57001.6
Q9JMG1	Endothelial differentiation-related factor 1	Edf1	148	16.4	9.99	327206.9	325757.9	357350.8	321748.8	309032.8	328375.0	298528.8	316931.8	327666.6	338017.8	676126.3	669401.0	711983.9	725902.8
Q9Z2C5	Myotubularin	Mtm1	603	69.5	7.75	88954.0	93989.0	73319.1	92736.4	77468.4	79573.3	80488.5	78356.3	84471.4	76031.5	39550.6	46375.5	36482.3	32785.0
Q9ER39	Torsin-1A	Tor1a	333	37.8	7.17	292088.0	270471.0	305624.8	268610.9	316684.0	307902.9	265691.1	295633.3	286499.1	290545.7	87626.4	78314.3	76964.0	75288.8
Q8R5F3	ADP-ribose glycohydrolase OARD1	Oard1	152	17.1	8.53	260678.8	325233.7	339165.8	331459.6	313533.9	272521.5	277056.0	246564.0	338442.7	377717.3	344657.6	340431.1	401514.8	348209.8
Q9D0T2	Dual specificity protein phosphatase 12	Dusp12	339	37.1	7.99	248259.0	274429.5	266301.2	251084.3	256402.5	252520.1	232790.1	239409.1	245871.7	265713.3	114635.9	111371.9	110143.9	112113.1
Q64092	Transcription factor E3	Tfe3	572	61.5	5.59	285735.1	287586.1	255489.6	264406.7	267329.3	243575.1	254770.7	250532.2	247921.5	264393.2	#N/A	#N/A	#N/A	#N/A
Q8VCI5	Peroxisomal biogenesis factor 19	Pex19	299	32.7	4.34	46548.6	46798.2	45999.2	48990.3	48397.7	45233.3	45951.7	42366.7	53580.3	51661.7	111878.2	106220.6	119954.3	124578.4
Q8R1G2	Carboxymethylenebutenolidase homolog	Cmbl	245	27.9	7.18	33589.3	31253.9	22055.9	41354.5	33830.7	30377.1	33137.4	38885.0	28404.0	28273.3	44148.4	43747.9	29095.8	48103.9
Q99N84	28S ribosomal protein S18b, mitochondrial	Mrps18b	254	28.7	8.35	117781.7	124071.4	135394.8	142393.4	134960.8	132724.2	121817.2	130515.1	129690.5	122186.7	#N/A	#N/A	#N/A	#N/A
Q9D937	Uncharacterized protein C11orf98 homolog	MGI:191352	123	14.1	11.47	38815.8	36960.9	40895.6	36983.4	35312.2	41214.6	37138.5	33283.1	40815.7	36941.3	168783.3	178292.2	184497.9	199065.9

Q9ESC8	AF4/FMR2 family member 4	Aff4	1160	126.6	9.36	223121.0	223911.8	207722.4	189521.8	227095.0	229271.6	219882.4	193313.8	187563.3	221691.7	#N/A	#N/A	#N/A	#N/A
Q9WTJ6	Tesmin	Tesmin	475	50.6	5.38	99047.9	116168.7	98792.2	120339.1	97564.0	92010.3	92388.7	91512.5	104989.7	103145.9	150829.1	153419.5	145890.9	169600.2
Q7TPQ3	E3 ubiquitin-protein ligase SHPRH	Shprh	1674	191.4	7.46	46953.7	44212.9	43260.5	41609.8	55878.8	58772.7	51371.1	49078.0	48360.8	46350.0	111306.2	130141.1	96870.6	103765.2
Q6P5D3	Putative ATP-dependent RNA helicase DHX57	Dhx57	1388	155.7	7.87	103202.1	104567.7	88449.9	97204.5	111881.3	119361.2	108920.4	97387.3	100621.4	104410.6	#N/A	#N/A	#N/A	#N/A
Q8CCT4	Transcription elongation factor A protein-like 5	Tceal5	200	22	6.2	441232.9	401969.2	485001.3	371547.3	493922.9	460460.3	527842.0	434036.1	464554.7	494365.4	#N/A	#N/A	#N/A	#N/A
Q8BKE6	Cytochrome P450 20A1	Cyp20a1	462	52.1	6.95	295308.2	302568.5	224930.9	281234.8	258512.6	264744.3	319725.3	305145.9	349363.5	381259.2	153985.2	141168.8	148224.1	153862.9
Q7TMY7	Importin-8	Ipo8	1010	117	5.16	100357.8	118370.4	111852.0	124640.6	137105.5	119941.2	124263.4	124025.5	122209.2	112986.8	172014.0	172811.1	167160.3	167100.8
Q3U0D9	E3 ubiquitin-protein ligase HACE1	Hace1	909	102	5.76	120999.7	148125.6	107648.4	118395.1	125852.6	136812.7	113828.1	115582.1	117568.9	129878.8	231682.3	254499.6	246913.8	263159.8
Q91WE3	Ribonuclease P protein subunit p25	Rpp25	199	21	9.16	37430.5	25585.6	40492.6	28034.2	57155.5	65426.9	71334.3	55656.3	55113.5	48732.3	#N/A	#N/A	#N/A	#N/A
Q9EPK2	Protein XRP2	Rp2	347	39.4	5.24	118421.1	103046.8	107716.4	106655.9	118861.9	129232.9	135672.5	113136.8	107274.1	120912.8	104267.3	104521.8	107386.3	105257.9
Q61387	Cytochrome c oxidase subunit 7A-related protein, mitochondrial	Cox7a2l	111	12.4	9.6	82138.8	82490.3	86138.4	75841.6	94019.0	76726.2	85151.0	87322.7	92855.5	84618.2	122427.4	130083.8	126426.9	119527.9
Q91VJ5	Polyglutamine-binding protein 1	Pqbp1	263	30.6	6.23	39457.9	38396.7	20736.9	21064.6	37233.7	45636.1	33307.1	27481.5	24666.1	41583.4	311144.8	311386.0	314309.3	299987.7
Q9D2V8	Major facilitator superfamily domain-containing protein 10	Mfsd10	456	49.3	9.95	108383.2	123318.3	104241.2	112848.9	107007.5	118616.8	123674.6	117869.8	114569.9	112114.0	74713.1	84951.7	87589.4	83680.1
P27106	Muellerian-inhibiting factor	Amh	555	59.7	5.77	26876.0	38823.3	26769.8	25887.7	26415.2	31710.8	23605.0	34477.1	24459.6	32414.6	169865.0	180273.8	169558.4	190737.9
Q9CQX8	28S ribosomal protein S36, mitochondrial	Mrps36	102	11.1	9.99	447715.0	419020.9	399629.5	415332.9	438642.1	465645.0	448431.2	421304.0	458536.8	411753.4	76745.2	87300.9	96483.5	88728.9
O88983	Syntaxin-8	Stx8	236	26.9	5.01	118974.3	110882.6	110191.0	129775.2	111560.1	113393.9	114321.6	107162.8	116779.1	104320.8	182977.3	192391.9	173488.8	182808.5
P47915	60S ribosomal protein L29	Rpl29	160	17.6	11.84	658219.3	679563.5	683580.5	681159.6	599683.7	553853.3	522999.8	571205.4	562243.7	606334.2	770994.8	1004141.5	821913.7	898535.5
Q8BGF0	Expressed sequence AW551984	AW551984	804	89.4	6.92	554237.1	549745.5	472973.7	507109.3	582028.1	719799.9	831887.7	620354.9	542732.5	574718.0	#N/A	#N/A	#N/A	#N/A
Q80TY0	Formin-binding protein 1	Fnbp1	616	71.3	5.67	169520.1	159055.1	165902.6	160496.0	171511.8	173478.9	177941.5	158542.9	159457.3	166516.8	114222.9	112032.8	117580.1	119469.4
P62309	Small nuclear ribonucleoprotein G	Snrpg	76	8.5	8.88	885081.1	935894.9	941190.5	970507.6	1017745.6	967510.4	903287.9	916200.8	979600.4	954845.5	327111.9	288785.8	350736.5	294416.6
Q8VDD8	WASH complex subunit 1	Washc1	475	51.6	5.44	104918.8	124731.3	94059.8	105740.0	93730.2	96844.4	99088.0	91432.1	105512.2	117684.0	152738.7	143626.8	136418.6	128444.7
Q9CZH3	Proteasome assembly chaperone 3	Psmg3	122	13.3	8.66	147439.8	154421.7	146311.3	154309.0	153265.3	145604.3	139405.2	140410.5	162208.3	163508.5	144522.1	146683.5	136931.0	153957.8
Q9D7V9	N-acylethanolamine-hydrolyzing acid amidase	Naaa	362	40.1	6.46	184536.5	193375.5	163113.4	182505.7	197037.9	198941.3	213961.5	182408.7	201488.8	200020.0	50734.9	51301.9	47691.0	53608.9
Q99MW3	Preferentially expressed antigen in melanoma-like protein 1	Pramel1	458	52.4	8.09	193827.5	177634.6	174959.3	149412.7	186928.8	184540.0	148202.3	178399.3	172700.5	189117.9	12475.3	14548.8	13456.6	13173.5
Q9DD18	D-aminoacyl-tRNA deacylase 1	Dtd1	209	23.4	7.87	36886.0	38441.7	41793.5	34265.8	44099.1	36967.3	39273.2	35187.6	37819.3	40544.9	112750.3	118098.1	108146.6	106274.6
P97792	Coxsackievirus and adenovirus receptor homolog	Cxadr	365	39.9	6.96	54166.6	58647.0	60692.0	52693.3	67129.7	68715.6	70479.4	56536.3	57177.6	55351.4	127529.4	123605.2	120412.0	113964.1
Q8JZV7	N-acetylglucosamine-6-phosphate deacetylase	Amdhd2	409	43.5	6.23	50767.3	49254.9	59777.5	55501.7	54580.2	57181.4	51168.6	55249.8	54939.9	49396.1	420984.3	534304.3	534497.9	513662.4
Q8C3W1	Uncharacterized protein C1orf198 homolog	MGI:191680	322	35.3	5.24	100339.8	110068.9	83442.0	106750.9	100204.1	116723.1	107913.8	87041.7	98449.4	104617.9	62482.8	59874.3	55226.7	60089.7
Q61146	Occludin	Ocln	521	59	6.48	49517.2	51715.7	61976.6	56535.0	59834.6	52992.7	60689.5	60311.4	52913.9	58510.7	55902.2	57126.1	62262.5	52331.1
Q8VDP2	UPF0428 protein CXorf56 homolog	MGI:192489	222	25.6	8.73	167119.1	183499.1	169636.1	170698.7	180116.4	193339.1	178947.8	160066.9	191232.6	186999.3	106017.2	103182.9	90389.7	106694.0
Q63739	Protein tyrosine phosphatase type IVA 1	Ptp4a1	173	19.8	8.97	399295.8	431856.7	460544.4	430943.5	420404.2	397011.8	406211.9	405581.8	436150.9	440135.0	15971.8	18081.1	15730.6	15765.6
Q9R0Q4	Mortality factor 4-like protein 2	Morf4l2	288	32.2	9.72	88641.7	87675.5	85420.8	81106.6	93739.7	91811.8	84825.6	100428.7	99433.8	96189.3	29812.9	26067.9	26842.6	18909.1
Q9JJQ7	Cor1 domain-containing protein	Xlr4b	215	24.7	9.17	80622.5	87533.8	88895.2	76552.8	87368.0	95613.3	60004.3	78349.9	74935.3	74505.9	#N/A	#N/A	#N/A	#N/A
Q5U4C1	G-protein coupled receptor-associated sorting protein 1	Gprasp1	1347	151.7	5.08	61644.9	62914.6	61560.9	60657.3	61926.5	56521.7	61071.4	62027.0	59650.5	63641.6	93951.4	87589.4	99782.8	86653.4
Q3U492	Kielin/chordin-like protein	Kcp	1550	166.5	6.06	110084.3	120401.3	116812.1	120646.2	122811.1	137432.6	122687.3	108414.4	111193.3	119591.5	82558.4	75972.5	80879.9	76966.3
Q3UMB5	Guanine nucleotide exchange protein SMCR8	Smcr8	935	104.9	5.4	102246.9	112818.3	115437.9	109902.6	114738.1	107285.5	109533.1	107514.3	116941.9	109317.7	95099.1	103998.4	95409.9	98236.8
Q91VZ6	Stromal membrane-associated protein 1	Smap1	440	47.6	8.51	390957.3	371403.7	346550.5	347694.1	378489.9	355443.3	352410.8	343303.7	366076.0	370405.1	172290.4	162752.4	200079.5	167380.9
Q5NCF2	Trafficking protein particle complex subunit 1	Trappc1	145	16.9	9.16	180528.9	195007.0	158943.5	186942.0	176678.6	163140.2	155606.0	158553.9	157501.7	179821.8	711517.4	735442.4	653702.4	744025.7
Q9DB85	Ribosomal RNA-processing protein 8	Rrp8	457	51	9.76	164474.4	188968.5	182557.5	177260.8	185623.0	195470.3	195887.3	183085.3	192010.1	191071.5	199693.4	220938.9	211967.8	220851.8
O35678	Monoglyceride lipase	Mgll	303	33.4	7.15	189510.5	203576.7	191765.0	204287.5	229406.4	174707.8	171355.6	202670.8	194854.2	196490.6	56822.1	57619.7	44277.8	50884.9
Q5F2C3	Meiosis-specific kinetochore protein	Meikin	434	47.4	5.96	69030.0	74722.2	68970.0	78097.4	62475.9	65859.3	51980.8	57876.0	66636.4	70258.0	#N/A	#N/A	#N/A	#N/A
Q6PAC3	DDB1- and CUL4-associated factor 13	Dcaf13	445	51.4	9.32	279397.7	284760.7	272811.2	263862.1	308969.0	277606.9	254527.2	272346.8	291648.6	303557.8	133224.1	143250.2	134638.7	145722.7
Q3U1T3	Breast cancer metastasis-suppressor 1-like protein	Brms1l	323	37.6	5.15	289030.2	279438.5	264435.9	256902.8	260530.7	277868.9	266024.1	266462.5	272570.4	271522.4	329894.8	324170.4	295290.1	323978.3
Q8K248	4-hydroxyphenylpyruvate dioxygenase-like protein	Hpdl	371	39.9	6.84	89530.5	97066.3	87821.8	95700.1	83411.7	87857.5	76780.6	84101.2	81048.8	87300.2	152558.6	163076.1	131191.9	164076.1
Q7TN02	Mediator of RNA polymerase II transcription subunit 26	Med26	588	64.6	9.19	37786.2	28849.6	38829.8	39613.0	41572.1	38344.8	30608.2	38768.1	32002.5	37504.3	137276.6	127523.9	130797.8	125030.5
Q9WV76	AP-4 complex subunit beta-1	Ap4b1	738	82.9	6.24	84001.8	95217.0	80509.8	96820.4	84237.6	78637.2	69207.7	75172.3	88580.8	93213.6	115496.5	135543.5	115449.5	120458.2
Q99KU0	Vacuole membrane protein 1	Vmp1	406	45.9	6.95	26087.7	71268.6	20672.5	25165.2	28418.4	30580.7	26752.9	28562.7	28115.6	27076.2	441767.1	500650.4	420482.2	450527.3
P57787	Monocarboxylate transporter 4	Slc16a3	470	50.3	7.96	178515.5	164725.5	182609.8	143137.4	156162.2	161588.6	176791.5	208972.4	175604.7	174870.0	183254.9	221449.6	174268.5	180659.6
Q61730	Interleukin-1 receptor accessory protein	Il1rap	570	65.7	7.77	59090.9	49456.5	57402.1	49074.6	59566.6	55853.3	50053.5	52840.6	53297.9	49449.7	268155.8	268893.2	227886.0	231049.2
P24638	Lysosomal acid phosphatase	Acp2	423	48.5	7.02	261877.0	256582.6	242431.7	264548.2	266637.1	247189.4	268819.4	272740.1	272161.2	255179.9	224289.6	238657.8	218884.8	227794.7
Q8BWT5	Disco-interacting protein 2 homolog A	Dip2a	1523	165.1	7.61	24991.9	28628.4	23273.5	23913.6	33194.3	33133.6	23856.3	25960.4	21620.8	30620.6	19027.9	20018.8	20208.9	20923.8
B1AZA5	Transmembrane protein 245	Tmem245	876	97.3	8.75	158627.8	142163.8	131864.4	131626.7	147178.3	140963.8	145278.2	131578.1	152601.6	151334.0	147225.2	149142.7	140715.8	134301.9
Q6AW69	Cingulin-like protein 1	Cgnl1	1298	148.1	5.77	56459.7	66849.1	55209.3	59193.5	73456.7	73390.5	71230.3	59122.6	56396.1	62105.8	4844.6	3898.9	5590.4	2859.7
Q3UJV1	Coiled-coil domain-containing protein 61	Ccdc61	511	57.3	10.49	174865.2	192410.9	211691.1	235165.0	165774.0	154650.7	135672.8	156624.4	186261.6	206583.7	92430.3	113971.6	116559.0	119123.9
Q5HZI9	Solute carrier family 25 member 51	Slc25a51	298	33.7	9.66	261841.4	282303.3	194972.0	247347.5	233276.7	243386.7	230033.8	235288.7	239958.5	260872.8	105415.3	111028.5	86777.3	111703.7
P0C605	cGMP-dependent protein kinase 1	Prkg1	671	76.3	6.04	158920.4	161468.1	149421.8	152740.9	179166.7	184348.0	208729.5	166525.5	181928.2	173238.4	314909.9	266130.3	262119.6	263080.4
P97480	Eyes absent homolog 3	Eya3	510	55.9	5.19	142533.6	138866.7	132166.4	133123.5	138279.8	130942.9	135987.6	130807.4	135663.8	141730.7	54423.9	51198.8	53626.2	55381.9
O35242	Protein FAN	Nsmaf	920	104.5	5.91	107800.6	117690.8	114103.7	111495.2	112661.1	118991.8	112247.5	110281.6	110530.0	110770.0	48670.0	55275.7	49007.9	47270.1
Q9D3B1	Very-long-chain (3R)-3-hydroxyacyl-CoA dehydratase 2	Hacd2	254	28.4	9.58	133403.0	157905.6	136896.5	143323.5	144327.0	147077.8	130233.8	145942.8	147914.2	142562.7	654689.3	596517.8	612454.7	598587.6
Q8VI63	MOB kinase activator 2	Mob2	235	26.8	6.52	417471.5	413524.0	393339.2	420380.3	424951.7	447864.6	410562.6	415231.1	416606.4	407083.3	689354.2	704084.4	657223.1	693730.2
P57791	CAAX prenyl protease 2	Rce1	329	35.8	7.97	52525.2	51631.0	41356.4	46601.2	47661.6	49457.6	47162.9	34436.8	57368.7	50730.9	29606.6	31225.8	34708.3	27636.4
Q8K013	GTP-binding protein 10	Gtpbp10	366	40.1	9.14	69331.7	79630.4	74874.7	85205.4	75246.6	71457.5	70114.9	70400.7	72914.0	82577.5	78466.4	73603.5	80453.0	79399.2
Q99L27	GMP reductase 2	Gmpr2	348	38	7.44	166632.1	248970.2	171009.4	181820.4	215902.3	208459.0	252637.1	183176.2	217317.0	232335.5	154688.2	161875.1	170266.3	144305.1
O08688	Calpain-5	Capn5	640	72.9	7.36	78717.2	89076.7	71156.8	78387.2	93801.0	101280.7	94380.8	84446.8	80100.9	81044.2	42752.5	47496.0	38090.5	43535.0
Q811W2	Cytochrome P450 26B1	Cyp26b1	512	57.3	8.46	145241.9	145476.1	143801.5	146846.7	147550.8	148319.7	141842.7	145528.8	146325.2	150027.7	104033.6	134535.1	101697.4	120314.1
Q9CR89	Endoplasmic reticulum-Golgi intermediate compartment protein 2	Ergic2	377	42.5	6.89	231674.4	259760.7	258209.2	245814.9	268127.8	269221.7	226370.2	266812.6	257872.1	244625.3	265305.3	293534.4	294711.9	288976.6
Q91X43	SH3 domain-containing protein 19	Sh3d19	789	86	8.53	107451.5	93218.7	102799.1	102812.0	112804.7	116764.7	126125.3	107662.5	112094.3	106267.5	397719.2	424936.2	374171.8	399019.1
Q8BRH3	Rho GTPase-activating protein 19	Arhgap19	494	55.7	9.26	26750.6	30752.8	25805.0	31374.4	24945.3	28749.2	20647.2	21835.3	23853.5	29535.2	#N/A	#N/A	#N/A	#N/A
O09161	Calsequestrin-2	Casq2	415	48.1	4.27	186745.8	179741.2	169039.9	160215.8	169881.9	183319.0	162141.2	198642.3	181170.5	166837.5	62647.3	63554.8	59222.6	51989.5
Q8R1G6	PDZ and LIM domain protein 2	Pdlim2	349	37.7	8.7	47010.9	57444.2	48435.1	47983.6	62564.9	79363.2	60729.1	62151.3	55038.4	58299.7	102007.8	104145.5	102716.1	91390.5
Q64449	C-type mannose receptor 2	Mrc2	1479	167	5.99	234163.4	234727.4	200876.9	215099.6	263453.6	276730.5	218058.8	256265.5	228555.0	218891.8	59652.4	63452.0	57234.7	62419.3
Q8VEB4	Phospholipase A2 group XV	Pla2g15	412	47.3	6.47	87884.9	92737.4	75031.1	73786.9	87322.8	87909.0	89548.7	74285.8	85316.6	89959.5	179132.3	168688.0	176747.7	180045.8
Q9D9M5	Pyridoxal phosphate phosphatase PHOSPHO2	Phospho2	241	27.5	6.54	133976.9	159737.5	152907.2	171020.2	137947.2	130720.8	123634.9	137532.1	142163.9	164825.2	137549.4	160673.7	140268.0	162893.4
P51830	Adenylate cyclase type 9	Adcy9	1353	150.9	7.21	164131.7	133723.0	155213.3	132197.4	174152.8	181860.4	157842.7	171195.5	166908.3	149370.0	311806.5	309103.9	239331.2	283455.7
Q8BZJ7	DCN1-like protein 2	Dcun1d2	259	30	5.59	107010.1	117632.4	127678.1	128594.4	111914.4	104916.9	102418.9	101419.2	117462.9	115086.0	62237.0	88210.8	57721.2	86053.1
Q9Z0H8	CAP-Gly domain-containing linker protein 2	Clip2	1047	115.8	6.48	159778.4	132754.1	128167.5	132473.3	138483.1	137219.2	159764.8	133361.6	130859.5	135671.7	#N/A	#N/A	#N/A	#N/A
O70228	Probable phospholipid-transporting ATPase IIA	Atp9a	1047	118.5	7.69	70978.8	77330.9	64159.7	69024.4	74803.4	74949.6	71013.8	73655.5	73340.5	70291.9	347064.2	361882.6	325847.6	351973.5
Q6NXY1	TBC1 domain family member 31	Tbc1d31	996	115.8	8.12	71227.9	79227.1	63950.4	80720.7	83015.1	69643.7	74893.0	72015.3	83734.9	77260.5	56835.9	66033.7	58274.8	60529.9
O08796	Eukaryotic elongation factor 2 kinase	Eef2k	724	81.7	5.38	93966.9	112412.4	117974.5	101461.4	80671.3	95958.2	86212.5	96386.6	99281.3	107791.2	267926.8	277807.9	272168.3	276951.9
O35245	Polycystin-2	Pkd2	966	108.9	5.66	289994.3	311216.8	271035.7	319214.4	302920.0	305295.2	303263.8	265503.5	308947.4	284605.4	107985.4	112192.0	118416.5	107047.1
Q8K3I9	Glucocorticoid-induced transcript 1 protein	Glcci1	537	57.4	9.47	102726.4	98844.9	101395.8	98073.9	86693.4	87655.5	85105.2	81872.4	89791.5	90262.0	219869.3	219637.4	232750.8	222582.5
Q9CQ69	Cytochrome b-c1 complex subunit 8	Uqcrq	82	9.8	10.26	172122.9	179082.2	194350.4	191710.6	197125.6	178066.3	181050.0	172165.5	181027.3	180433.3	1739469.0	1903141.4	2283612.5	2116824.3
Q571E4	N-acetylgalactosamine-6-sulfatase	Galns	520	57.6	6.52	196937.6	204725.0	177778.2	196541.8	199159.2	206562.9	244691.2	166678.6	193068.4	201913.7	238962.7	239990.4	231682.5	225943.3
Q80VC6	tRNA selenocysteine 1-associated protein 1	Trnau1ap	287	32.4	4.74	91720.7	99598.6	89561.1	84925.0	91729.1	83601.4	89823.3	86239.7	88759.3	86792.6	154271.8	161239.0	178608.8	175557.5
Q99MR1	GRB10-interacting GYF protein 1	Gigyf1	1044	116.2	5.39	293621.2	284442.4	297634.3	306165.5	329335.3	316975.5	329017.3	281958.5	306167.5	316257.1	170129.1	176880.5	168558.0	172517.9
Q61122	NGFI-A-binding protein 1	Nab1	486	54	6.61	44231.5	47458.9	36264.3	42632.3	40572.2	36636.8	43295.3	43810.9	42145.8	36863.6	87521.7	95136.6	85315.3	96054.4
O09117	Synaptophysin-like protein 1	Sypl1	261	28.9	8.79	225977.5	244317.3	271116.3	246222.1	267270.7	240890.1	266388.2	221418.8	229601.7	237141.3	83057.4	74584.8	68677.6	78790.3
Q9ESK4	Inhibitor of growth protein 2	Ing2	281	33	7.91	22246.0	23767.7	23297.5	21911.2	23431.8	26277.4	22088.4	24560.2	24048.7	26716.2	37856.9	38802.6	32486.9	32912.0
Q69ZK6	Probable JmjC domain-containing histone demethylation protein 2C	Jmjd1c	2350	260.5	8.07	21356.0	20745.8	18742.9	15633.6	18904.3	23881.6	17976.9	17772.8	19120.8	21516.8	#N/A	#N/A	#N/A	#N/A
Q921U8	Smoothelin	Smtn	923	100.2	8.95	82948.1	83111.1	77830.6	81071.6	76536.0	73019.9	91399.4	97379.3	75803.1	67765.8	58448.1	64114.1	61930.5	67951.6
Q8R035	Peptidyl-tRNA hydrolase ICT1, mitochondrial	Mrpl58	206	23.5	10.18	270627.7	326306.0	298711.9	286582.4	314133.8	323311.2	287453.7	278380.8	290628.5	306068.6	849560.7	794019.3	852337.4	829914.5
P34928	Apolipoprotein C-I	Apoc1	88	9.7	9.09	186842.7	193865.7	226183.1	142053.6	104819.5	155575.9	119523.5	225220.3	188814.2	174282.3	565729.2	410118.6	675793.0	366766.9

P46656	Adrenodoxin, mitochondrial	Fdx1	188	20.1	5.62	582393.4	632526.7	454864.1	671850.8	423628.8	372097.9	381960.1	493591.3	435265.0	438006.6	665389.2	677090.4	444994.3	661304.3
Q02614	SAP30-binding protein	Sap30bp	308	33.8	4.87	61580.0	68482.1	58233.9	59891.1	57227.2	61321.4	55977.1	50714.8	67426.4	64119.9	87645.5	79585.5	75627.1	77400.5
Q6GU68	Immunoglobulin superfamily containing leucine-rich repeat protein	Islr	428	45.6	5.38	107672.3	112790.5	101910.5	109179.7	109055.2	122563.3	109596.1	113300.0	102315.4	113990.3	160044.7	143396.6	135451.9	110623.4
P61967	AP-1 complex subunit sigma-1A	Ap1s1	158	18.7	5.73	352250.6	355063.1	414766.4	388151.1	437012.9	405439.5	400674.8	394041.5	408571.5	377346.0	334260.1	352566.7	326285.3	358305.4
Q6XUX1	Dual serine/threonine and tyrosine protein kinase	Dstyk	927	104.8	6.58	149982.3	159866.2	140729.0	146981.0	149470.7	172190.9	171393.7	143738.5	149908.9	158554.8	149977.0	142474.9	146959.9	145975.0
Q9ERV1	Probable E3 ubiquitin-protein ligase makorin-2	Mkrn2	416	46.6	7.34	63823.8	71223.5	60489.1	65482.9	68887.2	80253.6	63037.2	61130.2	69010.3	70940.3	245123.0	279534.3	222850.8	233687.7
Q9D2Q3	ATPase PAAT	Paat	444	48.7	6.48	50785.7	60140.4	53989.0	54693.6	58628.5	73008.7	49464.0	49025.5	55217.5	59836.6	228589.6	241657.7	253698.5	240426.0
Q8JZR0	Long-chain-fatty-acid--CoA ligase 5	Acsl5	683	76.2	7.09	148538.1	187130.6	140467.0	162634.4	165757.7	211007.5	164756.9	161550.6	157563.3	157753.9	181933.6	155220.9	154308.9	154431.5
Q9DAP7	Histone chaperone ASF1B	Asf1b	202	22.5	4.44	22773.4	36663.3	45716.2	39513.2	43165.0	39646.3	32866.6	35506.3	39637.1	42380.9	174721.7	204122.0	192458.5	176873.6
P70297	Signal transducing adapter molecule 1	Stam	548	59.7	4.84	90356.9	93540.1	80490.2	86256.4	91508.6	99341.7	92903.6	82159.8	88445.6	87294.1	104613.7	96018.3	78428.4	93382.2
Q8BG79	CWF19-like protein 2	Cwf19l2	887	103.1	8.5	146215.7	158801.5	159994.2	168179.1	147080.9	134866.6	155121.0	146521.6	151528.4	166330.0	148583.2	144726.2	125540.2	143909.0
Q8K4K6	Pantothenate kinase 1	Pank1	548	60.1	8.03	105214.1	111402.1	100658.5	112334.5	122690.5	103653.1	106419.0	100739.1	102836.4	101756.5	#N/A	#N/A	#N/A	#N/A
Q8BT14	CCR4-NOT transcription complex subunit 4	Cnot4	575	63.4	7.03	36070.2	35683.9	42087.8	38609.2	41747.2	32456.7	29079.6	32970.0	39162.1	37316.4	102735.2	104810.4	107357.2	110471.0
Q3U1V6	Ubiquitin-conjugating enzyme E2 variant 3	Uevld	471	51.6	7.28	256783.4	270907.0	259644.2	268404.5	269280.6	263447.4	236299.9	255022.4	254511.2	267046.7	282179.0	282941.9	282643.9	276389.1
Q6ZPR6	Inhibitor of Bruton tyrosine kinase	Ibtk	1352	149.5	7.72	112482.4	120765.2	103059.3	118478.7	108163.4	98125.0	101531.0	109608.7	112277.8	116744.8	75226.7	76844.9	66820.2	70182.9
A2A6A1	G patch domain-containing protein 8	Gpatch8	1505	164.9	7.64	115820.0	123753.1	107334.7	120004.5	119455.0	118284.4	118650.4	105976.0	117216.7	121482.2	90045.1	107210.4	90648.6	94032.3
Q9CQP2	Trafficking protein particle complex subunit 2	Trappc2	140	16.4	6.52	116931.9	143821.0	131202.0	148833.0	125869.2	129518.1	116404.1	121694.2	135657.6	146687.7	#N/A	#N/A	#N/A	#N/A
Q9JJT9	Phosphorylated adapter RNA export protein	Phax	385	43.2	5.34	66634.3	63148.5	63753.8	59520.6	73004.6	64558.4	75336.4	66426.5	63439.2	65947.0	49672.1	44080.3	60243.4	49437.4
P41230	Lysine-specific demethylase 5C	Kdm5c	1554	175.2	5.44	120646.8	125982.2	122082.1	116616.6	138629.5	146235.9	128098.3	130811.4	142471.2	130032.5	#N/A	#N/A	#N/A	#N/A
Q7TT18	Activating transcription factor 7-interacting protein 1	Atf7ip	1306	138.5	4.77	177854.3	183546.4	150520.5	157433.8	175113.8	174549.0	162069.7	161772.0	166474.5	170098.2	126107.4	144714.7	136576.0	148021.4
Q5NCR9	Nuclear speckle splicing regulatory protein 1	Nsrp1	542	63.8	8.76	21068.1	21113.8	22636.7	24527.2	28086.3	26816.7	24898.5	25377.0	24400.3	23418.2	238350.4	267602.6	225990.5	258760.0
Q5M8N0	CB1 cannabinoid receptor-interacting protein 1	Cnrip1	164	18.6	7.96	129710.4	105352.5	105748.9	100689.3	112934.6	125684.7	156224.3	123300.1	118823.6	104954.7	112565.4	96335.2	106728.2	95026.5
A2A690	Protein TANC2	Tanc2	1994	220.1	7.97	81075.5	84144.1	78152.7	81815.9	100239.0	91797.3	78594.6	79453.0	85558.3	92364.7	231410.4	272129.1	219450.7	219805.9
Q6ZPJ0	Testis-expressed protein 2	Tex2	1128	125.1	5.8	10388.5	19249.9	20602.2	12923.2	10761.6	9339.2	11084.9	17257.8	13089.1	12696.0	#N/A	#N/A	#N/A	#N/A
O55091	Protein IMPACT	Impact	318	36.3	5.05	126945.7	145507.2	134061.3	135001.6	132519.4	132904.6	129600.8	132022.8	126303.1	117981.9	86894.6	68567.0	86708.9	70197.1
Q5U5Q9	BRCA1-A complex subunit RAP80	Uimc1	727	81.4	6.51	73088.5	70513.6	79572.3	74661.6	78173.6	75113.4	66424.7	68704.2	72215.5	74353.8	101238.4	104488.5	124192.5	112132.2
Q8R1J9	Torsin-2A	Tor2a	321	35.9	6.83	71321.1	89768.0	75654.6	79456.4	77001.0	82721.6	66914.1	77060.2	74610.9	85104.7	28926.7	29615.5	26469.9	33437.3
Q80VI1	E3 ubiquitin-protein ligase TRIM56	Trim56	734	79.5	7.96	242273.4	257892.9	257507.9	258615.8	287994.6	293007.3	317113.4	265323.3	275119.6	298117.1	48354.5	54833.7	41558.4	49678.9
P40201	Chromodomain-helicase-DNA-binding protein 1	Chd1	1711	196.3	7.37	63896.0	69977.5	54546.3	56619.7	52584.1	55429.2	56078.3	52453.3	65930.1	71930.5	182076.0	159250.8	154192.2	176221.5
Q9D4V7	Rab-like protein 3	Rabl3	236	26.3	7.15	174704.8	192715.0	183211.7	194242.9	181340.6	183009.3	178041.9	179808.3	191902.2	184658.3	57953.6	62988.3	61844.4	65594.4
Q9D8S4	Oligoribonuclease, mitochondrial	Rexo2	237	26.7	7.15	804474.4	881732.1	959868.4	1030423.9	802782.4	743049.2	735605.2	859367.0	884076.9	872744.9	450026.2	509157.6	533768.2	611322.2
Q9DB42	Zinc finger protein 593	Znf593	134	15.1	9.55	139468.7	129730.0	157551.1	146026.4	162434.6	157171.6	149047.0	148992.5	135111.6	130304.1	11331.3	15192.6	13996.0	14690.6
Q921X6	DNA-directed RNA polymerase III subunit RPC6	Polr3f	316	35.6	6.11	76119.6	76056.4	75344.6	69926.2	77554.1	87164.4	80879.8	75222.1	71446.5	77543.4	175131.1	192934.3	181495.9	193029.3
Q80WR5	UPF0688 protein C1orf174 homolog	MGI:214068	230	24.8	6.04	186891.2	206789.2	194267.8	207450.4	185175.3	185694.9	176117.2	179032.4	190927.0	197096.1	19273.5	18143.6	22028.0	23503.6
Q640L5	Coiled-coil domain-containing protein 18	Ccdc18	1455	169.6	5.49	102610.6	135829.7	104918.2	115783.5	112778.5	96253.1	89255.2	100849.3	107791.6	119191.0	364790.9	390152.5	424006.8	379739.0
O88952	Protein lin-7 homolog C	Lin7c	197	21.8	8.43	186439.5	170958.9	205195.9	164846.9	239571.1	246600.3	252360.9	208779.5	213278.7	212175.9	74986.8	88576.9	93175.5	87630.4
Q8BQZ5	Cleavage and polyadenylation specificity factor subunit 4	Cpsf4	211	23.6	8.47	87832.5	76134.7	84869.6	89344.6	86982.8	82411.7	74034.9	74178.0	78008.7	88395.3	#N/A	#N/A	#N/A	#N/A
Q923U0	TOM1-like protein 1	Tom1l1	474	52.7	5.36	104496.8	99091.3	96175.6	111164.1	108057.8	99133.7	100080.9	96234.9	101509.6	94455.4	75573.7	77254.4	69988.3	92405.4
Q8C7D2	Protein cereblon	Crbn	445	50.8	5.33	57919.1	66515.7	63296.8	66375.1	75974.2	70061.5	56803.1	60756.0	62506.9	63648.7	28240.8	39185.3	35277.2	37004.0
Q8BG15	CTD small phosphatase-like protein 2	Ctdspl2	465	52.8	6.25	84441.9	78008.8	87753.6	79389.9	92826.0	90726.7	86694.9	79529.6	81306.7	82955.4	503756.3	451593.4	498381.2	440673.5
E9Q4F7	Ankyrin repeat domain-containing protein 11	Ankrd11	2643	296	6.98	29878.8	34788.2	28035.4	31399.2	32954.4	38400.9	30422.2	27481.3	27522.1	33251.6	44203.1	51535.8	38662.0	48968.7
Q8VE10	N-alpha-acetyltransferase 40	Naa40	237	27.2	7.36	95338.4	139047.0	98998.8	128395.5	95732.7	110036.9	89251.5	97300.0	97975.5	112963.2	47066.7	80981.1	59875.6	71390.9
Q9DCM0	Persulfide dioxygenase ETHE1, mitochondrial	Ethe1	254	27.7	7.23	113770.6	115307.5	109681.9	117649.4	112870.6	123546.0	101642.1	123124.4	108704.3	95137.0	148093.0	157477.2	135688.9	148259.8
Q8K2Y9	Cerebral cavernous malformations protein 2 homolog	Ccm2	453	49.9	5.62	72416.1	71837.0	66354.4	73820.7	76863.6	77428.9	79928.8	73421.0	70970.8	70672.1	#N/A	#N/A	#N/A	#N/A
Q8K4F5	Protein ABHD11	Abhd11	307	33.5	9.61	171984.7	145246.9	161164.0	144937.5	157080.1	166800.7	164621.1	160182.1	142647.7	148529.1	217987.7	175540.8	199608.2	188382.3
Q9CZT6	Protein CMSS1	Cmss1	276	31.6	8.46	79161.1	84163.3	69296.1	73610.5	75176.5	78628.9	87358.4	71636.8	79858.3	84609.0	363894.0	352641.9	327638.9	331134.1
P47226	Testin	Tes	423	48	8.31	257303.8	256874.9	237759.3	253208.5	241433.8	279798.7	255559.9	352420.7	254890.5	252921.0	#N/A	#N/A	#N/A	#N/A
Q0KL01	UBX domain-containing protein 2B	Ubxn2b	331	37.4	5.94	39979.6	43609.4	32364.1	36671.8	40254.7	37899.8	41364.3	42622.9	39153.3	44250.3	#N/A	#N/A	#N/A	#N/A
Q9Z0L8	Gamma-glutamyl hydrolase	Ggh	317	35.4	7.5	157146.9	142536.9	141691.7	138748.8	148525.3	155516.9	149085.4	133565.2	149129.9	141744.4	206505.5	267751.7	264231.8	225979.9
G3X9B4	BRD8 domain-containing	Brd8dc	273	30.7	4.48	11611.4	20555.6	21481.4	28307.6	19059.1	18312.0	17230.4	19752.8	14952.2	21763.7	#N/A	#N/A	#N/A	#N/A
O35144	Telomeric repeat-binding factor 2	Terf2	541	60.2	9.5	80611.3	68482.6	69705.5	67956.4	77212.3	76603.1	71354.1	62201.4	59721.2	64967.2	93438.6	83283.3	113084.1	73548.8
Q99K43	Protein regulator of cytokinesis 1	Prc1	603	70.2	7.58	709913.1	863791.7	728807.4	842595.1	568805.1	637904.9	575646.3	620394.7	709426.9	758749.0	201662.7	234199.7	244735.7	230387.9
Q5IRJ6	Zinc transporter 9	Slc30a9	567	62.8	8.69	157506.2	182749.7	151715.0	172493.2	168084.0	181567.0	141059.3	157382.4	152083.1	165408.0	250940.4	247852.4	232583.4	239777.9
Q3UR97	Sorting nexin-21	Snx21	363	40.3	5.36	209927.6	215271.1	199293.2	193092.9	220441.5	226111.7	202953.1	206369.0	206178.1	213039.4	275912.4	289903.8	242339.2	267361.3
Q3TDX8	Cytochrome b5 reductase 4	Cyb5r4	528	59.7	6.35	86090.9	92701.4	77316.9	82104.7	80638.6	88333.2	86114.1	83487.3	93917.1	86084.2	#N/A	#N/A	#N/A	#N/A
Q9JM98	WD repeat-containing protein WRAP73	Wrap73	462	52	7.18	65030.4	81876.1	74657.6	78489.9	67611.3	71549.5	65394.6	65302.2	93688.8	80377.4	98294.0	118136.5	102350.7	98902.7
Q6NVF4	DNA helicase B	Helb	1074	121.4	5.82	73438.4	75605.4	71586.0	66719.3	78056.3	80350.9	82077.0	67550.1	66335.9	72201.2	147319.1	147616.9	142841.8	137376.1
P20357	Microtubule-associated protein 2	Map2	1828	199	4.91	277046.7	373408.9	310070.6	260049.3	361839.8	382563.5	357670.4	322538.2	353045.2	381944.0	123884.1	143626.1	129374.6	125448.9
Q8K2I9	F-box DNA helicase 1	Fbh1	1042	118.2	8.13	103990.7	105184.9	101863.1	98535.4	107824.7	113192.0	102448.4	100070.5	102671.4	111707.2	76868.5	85287.1	75225.1	76448.6
P06537	Glucocorticoid receptor	Nr3c1	783	86	6.35	77635.6	148681.6	83365.2	101954.5	88453.4	102971.7	86493.2	98479.9	102546.4	97359.7	156210.9	163445.4	147621.8	164439.4
Q9CTN5	Protein SIX6OS1	Six6os1	574	66.8	5.62	156405.4	231815.7	191219.3	188708.8	202843.1	216738.1	128874.8	165092.3	184077.4	193297.2	163941.3	184456.0	177499.1	190326.7
Q3U0K8	Prolyl 3-hydroxylase OGFOD1	Ogfod1	545	62.7	4.91	97347.4	102033.6	101729.3	102181.8	107941.3	115580.6	116462.9	96033.6	109862.7	94659.9	118583.5	131214.3	111917.2	129811.2
P51829	Adenylate cyclase type 7	Adcy7	1099	122.6	7.87	92393.8	89947.9	89370.5	82145.0	95730.6	90017.7	103864.1	89951.6	87987.3	87902.4	5161.4	16370.4	4294.4	4811.2
O54940	BCL2/adenovirus E1B 19 kDa protein-interacting protein 2	Bnip2	326	37.7	4.97	212804.9	224418.3	214019.5	198313.6	202411.4	199304.7	184818.6	191052.4	217429.7	205258.2	448511.2	514143.5	398970.1	436092.6
Q8BGS2	BolA-like protein 2	Bola2	86	10.2	6.16	510230.6	528440.6	504818.9	552145.8	516566.7	505949.8	514459.3	501482.6	573072.6	554178.7	212385.9	227872.9	255018.1	268441.9
Q9WU65	Glycerol kinase 2	Gk2	554	60.6	5.3	137680.1	188621.5	196781.6	262376.2	134297.0	152718.2	141154.4	155269.2	151888.3	165126.8	83146.1	102456.1	88020.4	91651.5
P56375	Acylphosphatase-2	Acyp2	106	11.9	9.26	178646.0	165849.7	164626.5	159804.6	169127.5	193827.2	202623.2	166544.1	164652.6	168737.5	53813.8	52443.6	48254.5	56280.0
Q570Y9	DEP domain-containing mTOR-interacting protein	Deptor	409	46.1	7.91	147024.5	142787.0	150558.2	132473.7	216494.1	217825.0	201973.9	164509.6	173418.9	166893.9	106046.7	112086.8	108525.6	106591.5
Q9QZV9	NTF2-related export protein 1	Nxt1	140	15.8	5.03	60807.5	64541.8	73691.8	65103.0	62269.2	55059.0	53035.9	57499.5	63149.2	61066.2	224835.2	234943.8	235157.2	247144.7
A2AJL3	FGGY carbohydrate kinase domain-containing protein	Fggy	552	60.3	6.42	51207.4	54611.1	45787.0	50821.7	43630.9	45038.5	53847.4	47661.5	46653.2	50244.0	97730.5	87297.6	92302.7	92533.7
P70458	Plasma serine protease inhibitor	Serpina5	405	45.6	9.5	83133.0	93916.4	76168.6	79409.2	66514.8	69288.0	60549.3	85271.8	63044.4	68229.3	216689.0	255228.9	207841.3	255354.5
Q5EG47	5'-AMP-activated protein kinase catalytic subunit alpha-1	Prkaa1	559	63.9	8.12	20089.2	21978.0	21034.5	23226.7	23104.1	24509.0	18649.3	18448.5	20038.3	22597.0	104354.0	106943.5	105845.6	106996.2
Q7TS68	tRNA (cytosine(72)-C(5))-methyltransferase NSUN6	Nsun6	476	52.2	7.83	293934.9	230404.5	208732.5	229917.3	217681.4	232534.9	222562.3	209047.9	227336.1	302116.8	249445.6	163514.4	135740.9	166336.7
Q9WU62	Inner centromere protein	Incenp	880	101.1	9.55	110131.2	131895.0	117071.1	139643.9	118319.1	106112.6	105386.8	103233.0	117803.7	127436.7	71631.3	81664.3	81078.9	82617.4
Q3TRM4	Patatin-like phospholipase domain-containing protein 6	Pnpla6	1355	149.4	7.83	115159.0	128047.9	111157.5	135664.2	116574.6	117565.3	121567.3	109010.6	117357.2	125332.1	89961.1	106547.3	84901.1	91394.7
P97789	5'-3' exoribonuclease 1	Xrn1	1719	194.2	7.5	120444.0	131561.5	124151.2	120618.4	132307.2	133763.8	141412.1	127234.8	130353.7	141423.9	184392.7	163819.5	193897.6	158270.7
P29594	Caspase-2	Casp2	452	50.6	6.52	79001.5	61003.2	58723.5	60856.2	73824.6	65500.8	68957.2	62312.1	62549.2	64635.5	30501.6	33075.3	30053.0	30560.1
Q63961	Endoglin	Eng	653	70	6.2	14221.7	16451.9	15228.2	15587.1	16175.5	17082.8	15214.9	15967.0	12542.6	14121.6	50026.8	53548.9	44371.6	47717.2
Q91Z40	GB1/RHD3-type G domain-containing protein	Gbp7	638	72.7	6.33	265797.4	264267.1	245566.5	289397.9	257114.7	262786.6	291579.8	256996.6	245507.3	239621.5	#N/A	#N/A	#N/A	#N/A
O88746	Target of Myb protein 1	Tom1	492	54.3	4.94	13154.4	16468.4	18460.2	14033.1	14912.6	13334.3	16986.8	16519.9	17474.9	15208.9	81337.1	90702.7	78613.8	101393.7
Q6ZPL9	ATP-dependent RNA helicase DDX55	Ddx55	600	68.4	9.38	139833.3	149685.8	129764.0	137621.1	148912.8	147169.3	129009.9	128788.6	150729.8	135536.9	207387.4	176927.0	139286.5	141054.0
Q7TSH2	Phosphorylase b kinase regulatory subunit beta	Phkb	1085	123.8	6.83	120964.7	115659.6	119924.7	124672.5	125559.9	136990.1	146240.1	129020.3	140440.4	133120.2	106551.3	109866.1	113513.1	118631.4
Q9CYD3	Cartilage-associated protein	Crtap	400	46.1	5.71	92267.1	86219.1	74352.3	96670.5	92044.1	92993.6	68116.1	111377.3	76042.9	88098.1	194822.5	189010.2	169684.4	177482.4
Q80U28	MAP kinase-activating death domain protein	Madd	1577	175.1	6.04	127533.1	132981.4	133931.4	128207.6	133387.7	136016.6	118237.6	115705.6	132138.9	135129.8	126366.0	154032.8	144399.6	142055.4
Q3U2I3	FTS and Hook-interacting protein	Fam160a2	975	106.5	6.76	37782.5	53598.7	32949.0	38415.9	40291.8	44223.2	30215.8	36574.1	37827.3	44446.6	43907.0	49300.6	52121.5	52669.0
Q9WU28	Prefoldin subunit 5	Pfdn5	154	17.3	6.33	104537.8	111001.6	118504.0	109838.3	124455.5	113620.7	98362.0	106169.8	123814.2	108024.5	218575.7	244132.9	220915.0	154234.3
Q91ZF0	DnaJ homolog subfamily C member 24	Dnajc24	148	16.9	4.92	98889.3	117624.4	96258.3	114189.1	97621.7	92109.6	85903.4	91963.1	106837.2	96891.5	186799.8	202378.5	190789.7	192297.6
Q9D9C7	Uncharacterized protein C3orf62 homolog	MGI:214824	252	28.5	5.44	381195.3	451668.3	450122.5	483008.6	350832.3	328021.1	324896.5	334241.9	404874.0	380441.7	137505.6	188103.1	186711.3	201484.0
Q8VC65	Nurim	Nrm	262	29.4	8.21	496659.5	511001.3	509560.5	448052.2	640316.0	541131.9	596643.2	513518.0	516148.3	567964.6	273276.3	258814.5	250362.1	239950.3
P70412	CUB and zona pellucida-like domain-containing protein 1	Cuzd1	606	68	6.64	59470.1	64920.0	57168.8	403076.6	60958.6	92278.1	65758.6	184447.4	57850.7	71500.4	84995.5	103999.2	88076.0	202382.5
Q9ERR1	Nuclear distribution protein nudE-like 1	Ndel1	345	38.3	5.24	149000.4	166699.7	167842.7	154596.1	175290.8	162183.2	176304.0	157531.3	178746.0	161599.6	61053.5	56950.0	63810.9	55425.5
Q8R0X2	CDK2-associated and cullin domain-containing protein 1	Cacul1	377	42.1	5.27	83896.0	96555.5	95134.5	95775.3	100083.8	90586.3	94064.1	95764.3	93106.9	103623.7	43534.1	48203.3	46092.4	53580.6

Q9WVB4	Slit homolog 3 protein	Slit3	1523	167.6	7.62	162950.0	162146.8	151202.4	143380.6	172539.9	183109.7	162626.6	165425.1	148637.3	167179.5	64805.1	61331.0	60639.2	59426.9
P97473	RISC-loading complex subunit TARBP2	Tarbp2	365	38.8	6.01	277168.0	372164.3	313291.6	334789.6	311851.7	314403.4	291436.6	284717.7	303480.7	329791.4	19583.6	234545.2	22531.7	20856.9
Q5SV06	Spermatogenesis-associated protein 22	Spata22	358	40.1	9.58	40590.8	40844.4	41752.2	41934.8	42985.5	41202.5	34022.4	30148.5	42613.6	40585.3	196646.1	258527.7	221859.6	245650.0
Q9EP82	tRNA (guanine-N(7)-)-methyltransferase non-catalytic subunit WDR4	Wdr4	413	45.7	6.44	103900.1	95252.9	92902.1	94517.5	99151.3	106437.1	90495.7	94179.0	96793.2	104161.3	92468.9	96389.0	89712.7	100746.5
Q9CPR8	Non-structural maintenance of chromosomes element 3 homolog	Nsmce3	279	31.4	9.07	129646.6	147419.3	162870.4	183783.0	127341.0	118566.0	108551.5	120393.0	139662.5	144572.6	29041.8	37344.1	35729.3	46534.8
Q80TQ2	Ubiquitin carboxyl-terminal hydrolase CYLD	Cyld	952	106.5	5.63	73030.3	77646.9	79725.5	78364.8	74158.0	82376.5	76442.5	67898.0	81184.9	76494.2	#N/A	#N/A	#N/A	#N/A
Q5UAK0	Mesoderm induction early response protein 1	Mier1	511	57.9	4.45	274821.4	273187.6	294326.7	253753.6	303681.6	314179.0	291446.3	298392.8	285898.6	296925.6	190164.1	213614.8	209362.9	179244.2
Q924W7	DENN domain-containing protein 2B	Dennd2b	1134	126.8	9.11	424881.3	351248.0	324066.7	358128.5	369409.9	333608.4	312439.4	345673.9	332054.4	347674.1	154677.0	145725.4	138698.0	141724.5
Q8R1F9	Ribonuclease P protein subunit p40	Rpp40	363	41.5	6.68	63282.0	67729.0	69358.2	68724.9	83376.4	83258.1	74593.4	67914.0	71215.1	73625.1	123490.5	126405.9	128393.1	135819.1
Q8C0Q2	Zinc fingers and homeoboxes protein 3	Zhx3	951	104.3	6.57	24101.0	27956.6	23944.3	27835.6	26554.0	28128.7	21342.5	24126.3	25410.9	24418.1	#N/A	#N/A	#N/A	#N/A
Q9JI59	Junctional adhesion molecule B	Jam2	298	33	8.4	94614.2	73811.0	88080.6	78206.6	100896.5	104185.4	93728.1	90347.5	89021.9	88987.3	323037.7	284264.8	307414.7	270710.9
Q9D1N9	39S ribosomal protein L21, mitochondrial	Mrpl21	209	23.4	9.99	102890.7	97700.3	97429.1	95947.5	103117.8	97734.9	107899.0	100308.8	103227.5	101655.5	264369.6	267094.9	272693.0	275509.4
Q99PF4	Cadherin-23	Cdh23	3354	369.4	4.65	113886.3	115436.2	106043.1	112060.2	113959.3	115168.9	113912.0	115631.5	113375.2	115916.1	66379.9	83576.2	63297.8	66743.5
Q9CQY2	RNA guanine-N7 methyltransferase activating subunit	Ramac	119	14.5	8.4	179532.8	182490.1	186952.3	178009.1	189469.0	202962.8	205679.0	185252.0	186715.2	200145.1	#N/A	#N/A	#N/A	#N/A
Q9D8X5	CCR4-NOT transcription complex subunit 8	Cnot8	292	33.6	4.81	36237.3	41060.5	46708.7	39473.3	40605.1	40445.1	35962.8	38626.9	40062.0	44427.3	#N/A	#N/A	#N/A	#N/A
Q03963	Interferon-induced, double-stranded RNA-activated protein kinase	Eif2ak2	515	58.2	8.57	130406.6	123758.6	121881.2	133649.5	121407.0	114361.6	136863.5	126961.8	119707.2	134563.9	126990.8	120375.8	106871.6	115737.2
A6X8Z5	Rho GTPase-activating protein 31	Arhgap31	1425	155.2	5.77	142528.4	114968.4	104241.8	123748.6	119441.4	133173.7	141746.6	112217.2	137453.7	131988.0	#N/A	#N/A	#N/A	#N/A
Q3UMR5	Calcium uniporter protein, mitochondrial	Mcu	350	39.7	8.56	110378.5	123614.2	108681.6	97076.2	121363.5	135121.0	114984.2	101615.1	111893.0	112864.1	84528.9	80930.6	73918.8	79884.1
Q8BX90	Fibronectin type-III domain-containing protein 3A	Fndc3a	1198	131.9	6.64	117296.6	116429.5	128492.2	118792.4	133685.8	162794.1	135360.0	132961.0	119931.4	122110.6	37764.2	41995.4	43354.1	41942.9
Q9JI90	E3 ubiquitin-protein ligase RNF14	Rnf14	485	54.9	4.7	70818.5	78684.3	71663.0	74959.6	77046.1	77195.8	67843.2	76313.5	70534.0	79427.0	43400.3	47035.9	40192.8	44167.2
Q99LG0	Ubiquitin carboxyl-terminal hydrolase 16	Usp16	825	93.4	6.18	47478.8	69444.9	66010.5	62290.4	60483.8	63404.4	50356.4	56060.8	59829.8	64519.9	30266.7	30839.0	28029.8	29130.4
Q8VD62	UPF0696 protein C11orf68 homolog	Bles03	298	31.8	6.1	251267.4	265947.2	282560.1	241290.3	265729.4	267772.2	296483.8	245100.8	269588.5	286222.9	341818.5	346747.9	309471.9	341104.3
Q8BK75	Elongator complex protein 6	Elp6	266	29.3	6.61	78350.7	76630.9	80403.5	66983.2	84066.4	88190.2	77384.9	73446.4	70543.2	79509.2	65304.6	69446.3	71166.4	60552.8
Q8BKC8	Phosphatidylinositol 4-kinase beta	Pi4kb	816	91.5	6.32	57826.5	55784.1	50641.6	51600.6	54511.8	58730.9	49549.9	56532.8	54458.6	58841.3	95625.8	102707.6	94304.2	95188.3
Q91VX9	Transmembrane protein 168	Tmem168	697	79.6	7.96	112420.9	118856.6	132705.1	126211.9	129685.3	128582.7	115915.0	119710.5	124319.8	119455.7	121999.6	129755.8	135848.7	142363.3
Q7TQC5	Aprataxin	Aptx	342	38.7	9.17	135738.4	152349.7	150535.7	143528.8	152501.7	147638.7	134292.1	131533.9	140164.7	159862.0	223265.1	217257.8	222255.3	228418.1
O88502	High affinity cAMP-specific and IBMX-insensitive 3',5'-cyclic phosphodiesterase 8A	Pde8a	823	93.1	5.85	86578.7	96155.4	87073.7	101258.5	97869.4	92399.9	79443.4	74099.2	89001.2	88528.2	91263.9	93518.3	104317.0	99321.5
P98195	Probable phospholipid-transporting ATPase IIB	Atp9b	1146	128.9	7.64	158409.9	145592.2	183039.9	128144.0	139288.1	156668.4	127754.0	140844.3	147370.1	154824.5	216876.4	220335.9	240698.7	192119.1
Q63ZW6	Collagen IV NC1 domain-containing protein	Col4a5	1691	161.7	8.1	117991.6	110865.9	107240.9	125548.1	136393.9	158381.9	134159.9	137345.7	123315.2	122063.2	175402.9	166683.5	196603.5	147200.9
Q91WD1	DNA-directed RNA polymerase III subunit RPC4	Polr3d	398	44.3	6.98	80486.1	78718.0	66339.5	86494.9	77824.7	70770.3	80628.0	72473.2	72595.1	79489.0	164950.7	192897.4	166702.2	179970.6
Q8BGK6	Y+L amino acid transporter 2	Slc7a6	515	56.7	7.3	95036.7	101126.0	97034.2	103228.1	106660.5	108769.4	94662.7	101085.1	106136.7	97848.7	168839.2	172558.8	180647.9	171073.7
Q149M0	C-type lectin domain family 12 member B	Clec12b	275	31.2	5.17	241632.3	224335.2	230931.0	224588.3	233441.4	250493.9	225131.5	234847.8	234246.9	230817.6	250450.2	260377.0	211932.0	239800.2
Q9Z188	Dual specificity tyrosine-phosphorylation-regulated kinase 1B	Dyrk1b	629	69.1	9.09	86000.2	80589.0	75689.9	86743.7	78337.8	84212.5	73421.2	81845.0	78530.5	79174.0	#N/A	#N/A	#N/A	#N/A
Q60838	Segment polarity protein dishevelled homolog DVL-2	Dvl2	736	78.8	5.96	77762.1	66785.6	85412.1	75400.0	77946.8	84351.4	74710.9	78393.3	91145.6	89290.2	467574.3	533410.6	531174.0	547693.0
Q99JT1	Glutamyl-tRNA(Gln) amidotransferase subunit B, mitochondrial	Gatb	557	62.1	8.54	214471.7	233345.5	228082.4	218652.9	222098.0	223102.1	219530.2	227582.3	235572.9	231511.0	61344.1	67136.5	70787.5	63041.8
P63147	Ubiquitin-conjugating enzyme E2 B	Ube2b	152	17.3	5.01	149202.1	148067.4	151225.7	147412.5	136806.2	135892.8	142171.6	131488.5	145185.7	160618.2	79665.0	77395.8	78461.6	80908.1
Q9CWI3	BRCA2 and CDKN1A-interacting protein	Bccip	316	35.9	4.45	133705.2	139639.7	133327.9	120556.4	136175.4	149908.7	136857.2	126318.7	134119.2	127748.5	27506.8	27341.8	27096.6	22021.6
A6X919	Probable C-mannosyltransferase DPY19L1	Dpy19l1	746	84.1	9.16	29175.0	28322.8	29670.4	28768.2	30386.7	31919.4	26188.2	28133.1	27180.6	31098.2	39655.6	49598.1	50502.6	45575.2
Q9D1I6	39S ribosomal protein L14, mitochondrial	Mrpl14	145	15.9	10.99	189120.6	194536.9	170351.2	206992.9	167872.5	181613.4	175591.0	175262.3	200216.7	190130.2	156921.4	155043.7	140145.2	147309.8
Q05921	2-5A-dependent ribonuclease	Rnasel	735	83.2	6.55	107137.7	89882.3	88254.1	97754.4	109435.0	108947.2	111353.6	98435.3	103297.8	94822.6	81555.1	74611.9	81522.0	74648.6
Q04841	DNA-3-methyladenine glycosylase	Mpg	333	36.4	9.45	121699.7	137519.7	121160.5	126271.8	124613.9	120331.3	115536.5	120554.0	130438.4	117155.7	461841.2	456599.2	434497.4	490001.1
Q5NC05	Transcription termination factor 2	Ttf2	1138	125.5	8.9	73848.6	82185.4	73327.7	64490.2	79260.9	79873.6	71531.8	73261.0	73175.2	82659.5	#N/A	#N/A	#N/A	#N/A
Q9WU84	Copper chaperone for superoxide dismutase	Ccs	274	28.9	6.1	421122.1	431783.9	376733.6	460648.5	398087.0	357096.9	397613.6	388934.1	386654.1	391857.5	516994.5	500443.1	417541.3	529953.6
Q8K209	Adhesion G-protein coupled receptor G1	Adgrg1	687	77.2	8.59	149168.3	157354.8	134476.4	147318.5	169631.7	160694.3	177133.6	157902.5	161763.4	173890.9	#N/A	#N/A	#N/A	#N/A
Q8BIZ6	Smad nuclear-interacting protein 1	Snip1	383	44.4	9.89	13556.0	15381.6	18759.8	16323.4	15327.5	19166.6	14871.7	17585.1	14643.3	16887.7	#N/A	#N/A	#N/A	#N/A
O55100	Synaptogyrin-1	Syngr1	234	25.6	4.65	103402.6	115958.8	123822.3	113954.5	144342.2	142459.5	118640.5	110604.4	134391.1	119343.6	500771.6	383943.0	391988.9	612579.5
A2ALV5	Protein shortage in chiasmata 1 ortholog	Shoc1	1481	168	5.91	524481.3	606073.9	672871.9	767515.5	703482.5	628131.7	473784.5	516159.3	651970.5	649601.4	149442.3	155589.2	162840.7	144559.3
Q0EEE2	Patched domain-containing protein 3	Ptchd3	906	101.7	5.45	40122.3	54879.6	48156.0	49917.6	42375.3	37893.6	35105.0	41243.5	43719.1	50125.2	#N/A	#N/A	#N/A	#N/A
Q05909	Receptor-type tyrosine-protein phosphatase gamma	Ptprg	1442	161.1	6.46	116211.6	138091.0	97290.6	123090.1	137874.6	135912.4	122766.5	118435.5	116602.1	118972.7	85935.5	115332.4	67474.9	86243.1
Q8BSK8	Ribosomal protein S6 kinase beta-1	Rps6kb1	525	59.1	6.81	101068.5	97504.7	82932.4	102218.1	89974.1	94802.6	81617.1	84992.6	89350.8	97512.2	89187.0	88342.8	81829.2	80235.5
P56581	G/T mismatch-specific thymine DNA glycosylase	Tdg	421	46.8	6.99	70155.7	63886.8	66601.8	63931.8	81136.7	78071.2	73680.4	74665.6	73241.6	67763.4	#N/A	#N/A	#N/A	#N/A
Q9CZT5	Vasorin	Vasn	673	72.2	7.08	67002.6	87372.6	72942.1	74236.8	86924.4	76456.7	76713.8	83757.2	77185.7	71142.4	#N/A	#N/A	#N/A	#N/A
Q78HU3	Multivesicular body subunit 12A	Mvb12a	271	28.7	9.11	181953.4	200398.4	210898.7	171888.3	239614.4	272064.9	210560.2	185931.3	205621.1	204846.7	175160.9	180254.5	181526.8	196463.2
Q3URE1	Malonate--CoA ligase ACSF3, mitochondrial	Acsf3	583	65	8.02	96498.6	88558.9	95462.0	95047.7	102228.6	117737.8	112879.2	94908.2	101334.3	97833.5	38560.2	38794.5	37120.6	37411.4
Q80W71	Pleckstrin homology domain-containing family A member 8	Plekha8	519	57.9	4.91	60172.1	66017.1	57566.8	68098.1	58288.4	56597.5	57720.8	51919.3	62455.4	66847.4	80277.1	90057.4	79949.4	88591.6
Q3TFQ1	SPRY domain-containing protein 7	Spryd7	196	21.7	6.7	229578.8	216351.1	216259.4	229469.0	218826.2	227478.0	221093.1	189006.8	237388.3	228338.1	219740.8	239109.0	232759.9	236696.9
Q9DBJ3	Brain-specific angiogenesis inhibitor 1-associated protein 2-like protein 1	Baiap2l1	514	57.2	8.75	218562.1	194728.5	181755.1	220809.9	207514.4	217549.1	242777.6	203724.7	214386.3	190356.1	192368.3	204430.5	172919.4	202113.3
Q8K0G5	EARP and GARP complex-interacting protein 1	Eipr1	386	43.1	5.14	88758.6	87080.6	109769.8	89825.5	106218.8	102805.1	88430.5	90024.6	110593.5	84232.0	59604.6	58325.3	60660.9	59585.8
Q8C624	Spermatogenesis-associated protein 33	Spata33	132	15	9.28	53438.8	65220.3	75795.3	78909.7	52485.0	41801.4	42488.8	48449.4	63587.1	58909.4	289730.8	401855.0	355651.1	447522.2
Q9CQY1	Ubiquitin-like protein ATG12	Atg12	141	15.2	4.97	195264.8	203944.6	194314.3	196524.1	193067.2	178173.1	188316.7	181632.2	194702.4	192757.4	704837.9	692787.5	682636.4	715874.9
Q6PGA0	REST corepressor 3	Rcor3	451	49.7	8.44	105471.5	110858.8	105719.5	109034.8	109708.7	110766.9	104291.7	97848.1	107330.4	113174.3	#N/A	#N/A	#N/A	#N/A
Q32NY8	BHLH domain-containing protein	Tcfl5	489	51.9	6.92	154018.3	207369.8	192121.2	228455.0	145600.9	118527.9	99686.5	119062.1	145508.1	197712.8	158820.0	207311.8	198898.0	222099.2
Q8CFI2	Ubiquitin-conjugating enzyme E2 R1	Cdc34	235	26.6	4.56	196488.7	236694.6	245184.8	241931.2	226520.3	207713.1	172987.4	199714.6	209514.2	229217.1	231946.1	242814.8	242932.9	249720.1
Q8K0T4	Katanin p60 ATPase-containing subunit A-like 1	Katnal1	488	55.1	7.09	68134.1	78129.7	73457.4	79278.5	69411.7	65429.4	71321.5	58521.7	65504.0	68960.4	61945.3	70620.3	61000.8	73822.6
Q6DFW0	Guanine nucleotide exchange C9orf72 homolog	/IGI:192045	481	54.2	6.09	64125.5	71257.0	74688.4	72277.7	66459.8	72345.2	62239.5	74992.6	70384.9	66142.4	#N/A	#N/A	#N/A	#N/A
Q8BZ20	Protein mono-ADP-ribosyltransferase PARP12	Parp12	711	79.9	8.54	147780.8	149688.1	149516.1	142059.7	153056.8	158724.6	150996.5	139678.6	169508.0	160489.1	70164.1	83540.3	74812.5	73139.4
Q8C6G1	Cilia- and flagella-associated protein 410	Cfap410	249	28.2	6.93	89269.7	87711.7	79685.1	86542.8	87752.5	89730.0	86961.5	76695.2	89580.8	96672.4	97435.8	98019.9	92996.8	104642.6
Q91VU6	DDB1- and CUL4-associated factor 11	Dcaf11	549	62	6.44	123852.4	121662.3	112787.4	129248.6	117099.2	128864.2	115932.4	114617.6	122198.2	120690.7	47635.5	46453.2	59013.7	55675.8
Q61592	Growth arrest-specific protein 6	Gas6	674	74.6	5.52	127074.3	125076.3	113623.3	130421.3	115815.8	127004.0	119686.6	113664.2	114575.0	120438.9	103767.4	96543.9	81210.3	94144.2
Q91ZR2	Sorting nexin-18	Snx18	614	67.9	6.67	1263157.2	1000798.2	1194521.3	948186.7	1335377.6	1296033.4	1259066.2	1317642.4	1162132.9	1138177.3	133082.1	128384.9	137174.5	123937.1
A0A140T8M2	Ig-like domain-containing protein	Igkv12-44	115	12.5	7.87	262565.5	241395.5	223133.9	234752.9	338394.6	217966.5	246980.9	243143.7	239574.1	247265.8	48402.2	28055.3	35006.5	38972.8
Q8BS95	Golgi pH regulator	Gpr89a	455	52.7	9.22	10792.9	8333.2	9840.6	8409.0	10556.1	13494.7	9937.7	10279.1	11984.4	10543.5	24510.6	23261.0	22398.3	20219.5
E9PYP2	Neurobeachin-like 1	Nbeal1	2688	306	6.39	127589.8	143573.0	135905.7	139632.9	135287.3	138247.9	132150.5	130857.3	141598.5	142890.4	46067.0	56136.6	60988.9	61776.2
Q08297	DNA repair protein RAD51 homolog 1	Rad51	339	36.9	5.6	58675.3	58897.7	55558.9	57675.3	55562.5	59775.3	51336.7	52357.3	54010.4	62429.3	125786.4	132178.6	116479.0	128190.5
P40240	CD9 antigen	Cd9	226	25.2	7.23	127135.3	84417.1	131454.6	79032.9	150035.3	183472.4	198001.5	153874.9	103131.8	99167.1	42624.7	49982.2	45247.6	41864.7
Q68ED7	CREB-regulated transcription coactivator 1	Crtc1	630	66.9	6.05	101552.7	109571.1	105905.7	107496.6	115984.9	116947.3	101922.1	89464.2	103541.7	109937.4	114391.6	120497.6	118548.9	121230.4
P13808	Anion exchange protein 2	Slc4a2	1237	136.7	6.14	102661.6	111659.2	114837.8	106415.3	92559.2	115406.7	101154.9	99652.0	109029.7	114629.1	38045.1	40105.1	42705.0	40650.5
Q8VBW5	HMG box transcription factor BBX	Bbx	907	100.7	8.59	56269.8	58255.2	49908.1	46045.0	55731.6	59610.5	42811.5	54559.0	49502.0	57165.0	133529.6	141662.2	139556.4	132230.4
Q8BG94	COMM domain-containing protein 7	Commd7	200	22.6	5.94	43573.6	46675.3	49566.1	41203.9	51360.2	47518.8	46014.9	47381.6	51485.6	46284.8	247000.1	245831.4	243414.3	248243.2
Q9D958	Signal peptidase complex subunit 1	Spcs1	161	18.2	10.01	601167.2	549499.9	639150.8	606577.8	559036.5	607644.2	506630.9	581899.1	527361.9	504955.9	489723.2	427235.1	495559.8	508288.2
P55284	Cadherin-5	Cdh5	784	87.8	5.3	88508.2	84966.9	90050.6	80721.2	105518.4	105584.4	113494.6	99237.0	93810.7	88712.1	132589.2	164657.3	123507.9	152703.9
Q9CQ25	Mitotic-spindle organizing protein 2	Mzt2	159	16.5	10.18	171226.3	165737.8	166215.8	152567.7	174808.3	181840.9	181057.6	173254.2	152918.4	163239.5	142123.3	139378.1	148464.8	173823.3
Q9CQ37	Ubiquitin-conjugating enzyme E2 T	Ube2t	204	23	7.97	20820.3	26154.5	24350.2	24693.1	26468.1	27747.5	20540.1	24268.0	25604.8	26406.3	56768.2	64556.7	57958.2	60592.5
Q5SUC9	Protein SCO1 homolog, mitochondrial	Sco1	284	31.6	8.47	428499.2	417837.0	422685.0	431008.0	418154.4	419373.9	484970.3	449050.6	449885.2	437725.8	236617.2	205008.5	225734.8	204111.5
Q9CQR2	40S ribosomal protein S21	Rps21	83	9.1	8.51	760324.9	720687.0	768231.7	713482.3	754461.7	693656.6	608225.4	778165.1	676239.2	662907.4	571652.4	583758.9	615599.5	584081.0
Q9CQ86	Migration and invasion enhancer 1	Mien1	115	12.3	4.51	117495.9	138099.5	120190.7	136867.6	149587.4	136216.2	99259.1	142483.0	125633.1	129738.0	79663.7	95751.3	97011.8	103255.7
Q9WVS7	Dual specificity mitogen-activated protein kinase kinase 5	Map2k5	448	50.1	6.34	60959.2	76833.0	67432.5	73171.6	70443.6	59948.9	64288.6	62571.4	62328.5	67854.3	#N/A	#N/A	#N/A	#N/A
Q8VEK0	Cell cycle control protein 50A	Tmem30a	364	41	8.37	152906.9	164048.1	145723.7	177496.2	156290.1	154929.0	151452.7	137011.8	168020.2	169537.4	189963.9	206398.7	190181.5	200054.7
Q8C0P7	E3 SUMO-protein ligase ZNF451	Znf451	1056	120	6.79	76161.7	78929.9	78308.7	71959.1	84853.7	84938.8	74736.6	74729.2	69860.2	75799.4	102637.6	107874.4	106227.5	98814.9
Q8K4Q7	Ceramide kinase	Cerk	531	59.8	7.62	108483.7	97962.0	99132.1	103726.3	99324.7	107235.9	106732.6	103232.9	114340.1	118006.2	147816.7	128227.2	129142.0	125098.7
Q3UHN9	Bifunctional heparan sulfate N-deacetylase/N-sulfotransferase 1	Ndst1	882	100.7	7.77	172017.9	168570.7	165468.2	155283.3	171559.7	164883.0	160664.3	154872.4	170517.8	170302.3	41236.1	35362.0	33369.5	41889.4
Q9DCA5	Ribosome biogenesis protein BRX1 homolog	Brix1	353	41.2	10.02	131335.8	128410.4	126132.5	131018.4	114918.7	121051.8	112211.1	119778.3	124801.4	129943.9	290617.2	279447.9	266667.8	279321.8

P60487	Pyridoxal phosphate phosphatase	Pdxp	292	31.5	5.74	70562.3	82980.1	76442.3	72103.7	77250.7	82218.8	75602.9	71237.3	72686.0	82975.9	173146.7	167172.5	161279.2	143347.2
P46686	Tubby-related protein 2	Tulp2	562	62.7	6.65	15472.0	14812.8	15081.8	14366.3	15875.7	14865.0	9921.8	17467.3	16346.4	15810.7	115040.8	140872.0	99351.5	122044.2
Q80YR9	RNA-binding protein 12B-A	Rbm12b1	836	96.5	8.07	53290.8	67687.1	75948.5	73775.6	62320.2	66952.4	67238.6	70978.1	65698.3	66523.1	223320.9	242368.2	219853.4	242486.7
F6U5V1	HTH La-type RNA-binding domain-containing protein	Larp1b	541	59.7	9.13	62002.0	71353.6	67851.2	72535.3	71371.4	60120.9	56238.1	59097.2	71998.4	67601.6	56799.4	78684.7	70786.6	79035.0
Q8VDG7	Platelet-activating factor acetylhydrolase 2, cytoplasmic	Pafah2	390	43.5	7.47	226807.8	226022.1	153435.9	187846.4	147669.5	182766.6	194723.9	149083.4	185582.0	185285.1	278985.2	320814.5	266568.6	329094.3
O89090	Transcription factor Sp1	Sp1	784	80.7	7.34	41564.3	44782.1	41915.3	42548.8	49979.2	52802.5	47766.4	49201.5	43265.0	43563.8	178047.7	156297.0	175055.2	162892.5
Q8R4U7	Leucine zipper protein 1	Luzp1	1068	119.2	7.99	42237.9	43605.1	36241.0	35236.5	42363.0	47647.6	41350.2	42534.4	43882.0	43026.0	#N/A	#N/A	#N/A	#N/A
P01897	H-2 class I histocompatibility antigen, L-D alpha chain	H2-L	362	40.7	6.25	34691.8	29328.6	30892.0	34759.6	35886.5	33816.7	36691.7	31667.8	38652.7	31458.7	77336.5	85186.9	83837.2	82372.0
Q91ZW2	GDP-fucose protein O-fucosyltransferase 1	Pofut1	393	44.7	8.41	92760.4	122607.7	81601.8	109162.4	103096.0	105961.3	93967.3	94717.6	94637.4	114402.1	139735.9	152376.9	134748.2	143083.3
Q9DAM7	Transmembrane protein 263	Tmem263	115	11.5	9.32	176363.5	167657.7	154450.4	169199.4	158527.7	167596.0	156167.5	163769.7	157579.9	157891.3	#N/A	#N/A	#N/A	#N/A
Q8C407	Protein YIPF4	Yipf4	246	27.3	4.65	233367.1	275936.0	242004.8	248480.2	225133.4	234421.9	191477.4	213085.2	210348.7	223472.5	294788.7	315723.1	323927.6	322937.2
Q61624	Zinc finger protein 148	Znf148	794	88.7	6.48	79618.6	73949.6	83151.3	71392.0	96435.4	102126.8	95021.2	81998.3	81889.0	83526.9	104518.6	107418.5	105628.4	95463.4
Q99MS8	Tubulin polyglutamylase complex subunit 1	Tpgs1	303	32.6	8.94	55810.8	60688.7	65806.1	62991.2	59971.3	57461.4	53208.3	56804.7	62748.7	59532.8	81087.4	88179.7	78508.5	85369.8
Q80ZD3	Sodium-independent sulfate anion transporter	Slc26a11	593	64.1	7.64	9568.2	10088.5	8766.7	10632.7	12315.9	11142.2	9070.1	7853.0	7734.5	10518.3	159514.9	140281.0	164266.4	145380.3
P68404	Protein kinase C beta type	Prkcb	671	76.7	7.01	141204.6	128217.3	119931.8	133362.5	111976.7	118057.2	123360.0	119331.7	120467.1	124843.9	#N/A	#N/A	#N/A	#N/A
Q8BN57	Protein C3orf33 homolog	MGI:360771	294	33.6	9.89	201843.3	214769.0	206759.6	186343.6	186932.7	200790.1	188005.0	181752.1	179519.2	196889.7	203540.0	199788.4	206566.3	180337.0
Q91VJ2	Caveolae-associated protein 3	Cavin3	260	27.8	5.57	201856.7	204546.6	199663.7	210651.1	232234.8	246275.2	276608.2	224764.3	205372.4	194232.6	577317.3	575261.0	609866.1	585437.4
P56695	Wolframin	Wfs1	890	100.5	7.97	100614.0	120190.1	96075.3	104886.8	117487.5	118243.0	91934.7	122247.8	109826.7	111128.3	77941.7	99101.1	82028.0	87993.3
Q5EE38	Adrenocortical dysplasia protein	Acd	416	44.7	5.27	60497.6	68832.4	83779.1	71002.1	75369.8	68862.0	55619.0	57101.1	72822.7	64415.5	#N/A	#N/A	#N/A	#N/A
Q61084	Mitogen-activated protein kinase kinase kinase 3	Map3k3	626	70.7	8.87	88055.0	128489.9	78410.2	100315.0	104380.2	102910.2	82462.5	150783.0	96545.5	105102.3	#N/A	#N/A	#N/A	#N/A
Q9CPQ1	Cytochrome c oxidase subunit 6C	Cox6c	76	8.5	10.14	437199.3	546634.3	482618.6	637412.9	442052.2	383965.9	494582.0	537766.7	502441.2	522295.9	1899932.0	2310626.6	2336125.2	2260818.1
Q9CY62	E3 ubiquitin-protein ligase RNF181	Rnf181	165	19.1	6.04	314167.6	315190.5	287057.4	298906.0	321554.8	358937.2	333788.0	285697.4	304922.1	320184.3	93377.3	84439.7	79629.3	84740.6
Q8VD04	GRIP1-associated protein 1	Gripap1	806	92.7	5.25	34930.5	37181.4	36502.8	35852.2	36178.0	29519.1	36662.3	35602.6	41335.3	33453.5	261221.7	240572.5	244880.5	269670.6
Q9JM05	E3 SUMO-protein ligase PIAS4	Pias4	507	55.5	6.37	95792.4	83336.7	93054.7	94898.9	110314.9	114752.5	95236.1	84859.1	100705.9	94834.1	14908.0	16919.9	16765.3	15604.2
Q9CWU4	UPF0690 protein C1orf52 homolog	MGI:191367	180	20.1	5.11	104996.5	99012.7	97003.7	102064.4	102392.9	96571.0	96841.7	91771.5	102598.3	102942.8	462085.2	510498.2	450010.3	490776.0
Q9CZN8	Glutamyl-tRNA(Gln) amidotransferase subunit A, mitochondrial	Qrsl1	525	56.7	5.95	78413.5	99144.8	66362.4	88374.6	73229.1	74744.5	66902.5	61816.2	85062.2	89108.0	112307.8	124592.7	122105.3	140741.0
Q8BVD5	MAGUK p55 subfamily member 7	Mpp7	576	65.5	7.3	164195.0	169948.1	166614.0	161332.3	185424.6	190138.4	180601.3	168653.1	172757.5	167070.2	425946.3	456387.5	443754.5	452700.8
Q3TB82	Pleckstrin homology domain-containing family F member 1	Plekhf1	279	31.1	8.1	115195.4	117586.8	114916.4	121547.5	123986.9	114930.9	118302.2	109394.7	117746.3	117913.3	110526.2	104195.7	99913.1	112555.6
Q3TEW6	Myelin protein zero-like protein 1	Mpzl1	270	29.5	8.43	296497.3	297334.4	316389.6	275036.1	274546.5	290015.3	290096.3	273007.9	319511.1	341082.2	#N/A	#N/A	#N/A	#N/A
Q91W53	Golgin subfamily A member 7	Golga7	137	15.8	7.05	57003.5	58735.5	73241.5	68489.6	71823.6	71045.9	68532.3	64842.6	63689.2	58308.5	45853.8	46360.0	59171.6	54753.5
Q3TN34	MICAL-like protein 2	Micall2	1009	108.2	9.55	34503.7	35960.7	30470.6	29847.4	30596.7	35479.4	28697.4	28168.9	32173.7	30666.5	#N/A	#N/A	#N/A	#N/A
Q6AXC6	ATP-dependent DNA helicase DDX11	Ddx11	906	101.9	7.56	46281.6	58089.2	59093.0	45087.9	54983.6	56878.5	45673.0	53264.1	56287.4	53030.2	#N/A	#N/A	#N/A	#N/A
Q9CPW2	Ferredoxin-2, mitochondrial	Fdx2	174	18.8	6.04	41578.8	47157.7	45276.7	48246.0	47909.2	50803.9	43982.3	45334.3	46960.0	48257.4	#N/A	#N/A	#N/A	#N/A
Q3V050	Multidrug and toxin extrusion protein 2	Slc47a2	573	61.7	7.83	74569.4	81865.5	85334.4	68841.9	93310.4	99836.4	100333.9	106901.2	99380.1	94767.4	63055.8	60014.5	55151.4	61610.5
D3YY91	Leucine-rich repeat protein 1	Lrr1	422	47.5	8.57	120946.1	127944.7	105980.2	114043.8	120163.1	131749.6	110618.7	111594.0	124764.8	119496.5	#N/A	#N/A	#N/A	#N/A
Q8BTX9	Inactive hydroxysteroid dehydrogenase-like protein 1	Hsdl1	330	36.8	8.48	110132.1	111085.2	138166.3	111608.0	140545.1	145575.0	157327.6	122340.1	132555.7	122980.6	451355.8	474974.5	416695.5	434668.2
Q91WK1	SPRY domain-containing protein 4	Spryd4	207	23.3	9.45	329866.0	398837.7	303445.7	342096.2	370300.7	343006.5	318571.6	340730.8	330607.2	357725.3	38437.4	41607.3	40171.4	39233.3
Q8VIM9	Immunity-related GTPase family Q protein	Irgq	583	59.3	4.83	353151.6	329087.6	313608.0	353300.1	315478.6	314316.4	335709.8	328817.1	335792.5	310059.4	82575.3	87320.8	77248.9	85613.4
Q8CAM5	Ras-related protein Rab-36	Rab36	267	29.8	6.23	33925.5	41570.6	36935.5	43114.0	31605.6	42532.1	31665.0	34071.3	37384.3	39912.9	63249.7	71500.1	63228.8	65940.3
Q8BGF6	ELMO domain-containing protein 2	Elmod2	293	34.7	8.57	157942.9	165934.8	169218.3	168731.4	186561.1	182526.3	172618.4	161933.0	151466.9	156297.5	265454.3	315746.4	288205.4	253176.5
Q64735	Complement component receptor 1-like protein	Cr1l	483	53.7	6.65	206362.4	184057.0	197840.0	189332.0	195977.7	228952.6	271527.5	197779.8	188118.3	192626.1	122819.6	107929.1	103796.8	105740.7
O35206	Collagen alpha-1(XV) chain	Col15a1	1367	140.4	4.89	198361.7	199974.8	168116.2	188880.9	208162.2	202194.2	197273.8	209284.8	175171.5	188647.5	83307.6	88660.8	80523.2	79656.2
Q9DCH6	AN1-type zinc finger protein 6	Zfand6	223	24	6.51	32757.5	28374.9	23343.5	27519.5	26830.0	26081.0	23428.5	26969.1	21516.3	29465.1	57984.8	57975.8	51950.9	59845.2
P62484	Abl interactor 2	Abi2	446	49.4	6.01	107111.7	104399.8	97241.6	94229.3	101760.4	105571.7	101359.7	95385.4	98982.1	95435.0	136262.5	132876.8	118170.1	123644.6
Q99K46	Ubiquitin carboxyl-terminal hydrolase 11	Usp11	921	105.3	4.96	380466.9	376905.0	311588.5	329622.7	329736.2	320220.9	338216.8	289323.9	350994.6	377331.2	#N/A	#N/A	#N/A	#N/A
Q9JM93	ADP-ribosylation factor-like protein 6-interacting protein 4	Arl6ip4	229	25.5	11.19	60966.3	65487.7	72238.0	63513.5	65555.0	68871.3	58081.3	64160.6	68665.7	69591.6	#N/A	#N/A	#N/A	#N/A
Q9D1R1	Complex I assembly factor TMEM126B, mitochondrial	Tmem126b	230	25.4	9.51	125790.1	151863.3	124407.5	137069.0	135464.6	147207.7	129501.2	125224.1	136422.3	139166.9	219578.0	288286.5	243697.1	251367.8
E9Q7M2	TSC22 domain family, member 2	Tsc22d2	769	78.1	4.89	54634.9	56568.5	63219.3	58420.5	70012.6	64012.6	64743.4	57380.8	53054.5	59697.6	16844.8	16740.3	20057.0	18646.6
Q9WV72	Ankyrin repeat and SOCS box protein 3	Asb3	525	58.2	5.9	31319.8	33375.0	35751.7	35557.6	31269.7	32624.0	29929.9	27270.9	31293.3	30967.1	37280.0	43336.2	38223.9	34921.1
Q8C1A3	Methionine synthase reductase	Mtrr	696	77.5	6.38	66171.0	57901.4	58736.3	62491.5	53877.0	60919.9	57030.1	58876.1	58233.5	59722.1	159037.8	141061.6	152202.2	144201.3
P46662	Merlin	Nf2	596	69.7	6.35	72956.6	84841.3	61475.7	65848.6	72776.3	73740.6	70662.5	73251.4	73060.6	86800.2	#N/A	#N/A	#N/A	#N/A
Q9QWY8	Arf-GAP with SH3 domain, ANK repeat and PH domain-containing protein 1	Asap1	1147	127.3	7.64	153491.5	160775.2	128207.6	137745.6	139920.6	147466.0	133927.1	131100.0	125483.5	145007.6	30663.1	35818.3	29799.0	30981.7
Q9Z2A7	Diacylglycerol O-acyltransferase 1	Dgat1	498	56.8	9.39	126478.6	133623.3	134451.9	134770.3	123168.6	116892.6	112083.4	114797.1	132801.7	124343.5	100356.4	113242.6	99578.0	112665.4
Q9CYX7	RRP15-like protein	Rrp15	281	31	8.76	391214.4	420466.0	371626.5	378384.6	412169.8	400877.6	377815.9	359352.2	396815.1	422320.2	363447.3	395880.3	371087.4	358325.5
Q3TCJ1	BRISC complex subunit Abraxas 2	Abraxas2	415	46.9	6.18	143421.5	153487.8	129862.0	135517.5	135910.6	130822.0	138385.3	141203.8	130402.4	151494.3	#N/A	#N/A	#N/A	#N/A
Q9EPA7	Nicotinamide/nicotinic acid mononucleotide adenylyltransferase 1	Nmnat1	285	32.3	9	183979.7	235455.3	177143.7	201334.3	224782.9	241891.0	195325.5	205844.0	210462.2	212051.4	25088.6	26567.8	28619.1	24689.6
Q8BJW5	Nucleolar protein 11	Nol11	723	80.8	5.92	412650.8	359667.3	398330.2	357578.2	416389.3	395357.4	367458.6	394937.4	388666.5	364594.5	399383.4	373399.5	415281.1	378265.4
Q8CGE9	Regulator of G-protein signaling 12	Rgs12	1381	149.5	7.59	62558.0	78402.0	66064.5	67248.0	71088.5	62103.3	59767.0	56680.8	64669.5	70212.9	228645.4	242167.8	194101.6	232812.1
Q3UHH8	Glucoside xylosyltransferase 1	Gxylt1	404	46.5	8.97	44310.3	42573.6	51879.9	48683.7	54909.5	49535.6	38901.6	43682.2	55700.1	42765.4	326897.5	351945.2	309649.3	332614.6
Q8K4G5	Actin-binding LIM protein 1	Ablim1	861	96.7	8.63	166498.5	168287.8	148261.7	153780.4	180310.1	182982.1	199653.8	177838.0	168319.5	167057.1	94724.6	106401.3	106624.3	100915.7
Q3V300	Kinesin-like protein KIF22	Kif22	660	73.1	9.25	47233.1	54875.6	46121.6	51374.6	54859.2	46827.6	40316.2	46807.0	51609.0	51015.3	107066.6	106917.9	100334.1	99201.7
Q9QZZ6	Dermatopontin	Dpt	201	24	4.83	75737.7	87735.6	79163.0	82735.6	99995.9	105399.8	114628.5	103718.0	80209.6	96312.2	113344.0	108093.6	103301.0	108154.4
Q9JMF7	Dolichyldiphosphatase 1	Dolpp1	238	27.1	9.58	94145.5	95518.3	87755.8	105777.0	92922.7	89815.5	90355.9	100877.2	91174.0	97959.2	134925.4	126446.7	118672.0	144599.8
P05532	Mast/stem cell growth factor receptor Kit	Kit	979	109.3	7.21	118455.4	112834.0	118132.3	114808.9	123451.4	122355.3	130004.1	119943.3	115808.3	119970.5	548175.8	452380.5	493560.6	500644.9
A2AHM1	MAGE domain-containing protein	Gm8914	344	38.4	9.13	75770.2	84716.0	88646.3	86337.4	97845.6	87968.1	80766.0	76864.7	86834.6	93842.1	91576.1	102216.7	102395.6	90292.4
Q9DBQ7	Protein-associating with the carboxyl-terminal domain of ezrin	Scyl3	735	81.3	5.1	48856.4	64537.7	51774.7	54632.5	51194.0	55369.1	50504.4	50792.5	52497.5	54953.0	83755.9	89726.0	84243.3	87539.9
Q8CFI5	Probable proline--tRNA ligase, mitochondrial	Pars2	475	53.5	7.84	70298.0	77057.0	72731.8	72332.0	79701.2	82187.8	87177.8	67838.2	78635.2	67573.0	46067.8	51451.2	53657.6	48559.0
Q80TA6	Myotubularin-related protein 12	Mtmr12	747	85.5	6.84	26251.0	21903.2	24051.8	21611.3	39162.6	27691.7	30460.9	23782.8	28832.6	26935.9	64751.0	63734.0	59251.6	52809.6
Q9DBL9	1-acylglycerol-3-phosphate O-acyltransferase ABHD5	Abhd5	351	39.1	6.79	116775.4	113910.6	107706.1	111429.6	122962.3	103287.2	102665.2	110313.6	99375.0	98971.7	76731.2	90696.2	65200.8	75033.2
Q8K4F6	Probable 28S rRNA (cytosine-C(5))-methyltransferase	Nsun5	465	51	9.19	59881.6	71016.2	61389.2	72388.4	68088.1	67348.7	61332.9	58710.9	63434.0	78196.8	44198.2	46014.3	39335.9	39586.3
Q8C827	Zinc finger protein 62	Zfp62	914	104.7	8.85	29876.9	32777.2	26604.5	37669.2	32843.1	30593.9	27681.2	29574.0	32770.0	31436.4	#N/A	#N/A	#N/A	#N/A
Q80VY9	ATP-dependent RNA helicase DHX33	Dhx33	698	78.3	8.94	83170.1	80933.8	76756.1	82881.2	84475.7	84775.1	80941.1	72118.3	79789.2	84006.3	62396.9	59138.3	63518.0	54870.3
Q8C5L3	CCR4-NOT transcription complex subunit 2	Cnot2	540	59.7	7.66	246864.5	245702.5	233495.6	231485.9	233387.6	239219.5	231851.8	231721.0	217704.1	262857.6	207381.0	190703.5	183198.8	170767.5
Q3V009	Transmembrane emp24 domain-containing protein 1	Tmed1	227	25.2	4.5	48941.7	69593.6	47362.9	52942.4	59733.8	59352.4	42028.6	61373.5	50903.0	67467.8	100871.9	134327.1	97354.3	109410.6
Q8CII2	Cell division cycle protein 123 homolog	Cdc123	336	38.8	4.67	56982.4	62928.0	56878.8	52602.1	57623.2	63926.9	56487.5	57030.4	59118.1	55393.7	42636.1	56512.9	42138.0	51201.6
Q9QXL8	Nucleoside diphosphate kinase 7	Nme7	395	44.4	7.12	243603.8	265654.8	233056.2	262114.5	247032.0	252839.8	279234.8	220327.3	245823.6	262501.6	92307.6	104025.4	109770.9	109565.1
Q8BMG8	Mitochondrial folate transporter/carrier	Slc25a32	316	35	9.47	90434.2	107478.1	85119.2	89440.7	89420.5	96482.2	88135.3	87614.3	97080.1	99017.3	268512.3	247077.2	233866.0	223721.8
Q8CI61	BAG family molecular chaperone regulator 4	Bag4	457	49.1	5.34	6729.2	10484.9	10763.9	10639.0	9497.0	10630.2	8713.5	8166.7	14313.9	9425.3	63843.6	76337.6	67337.0	73228.1
Q80ZN9	Cytochrome c oxidase subunit 6B2	Cox6b2	88	10.5	9.74	308207.6	424905.0	506905.6	635621.8	245716.0	218213.0	190763.6	270361.1	355942.4	381201.8	655481.3	871356.1	805428.4	966383.1
O35963	Ras-related protein Rab-33B	Rab33b	229	25.8	7.69	94790.6	88288.1	87923.1	83138.7	87378.5	84002.4	91826.1	87115.1	77559.4	88915.6	77507.5	66769.7	70276.6	68415.4
Q8BXL9	Intermediate filament family orphan 1	Iffo1	562	62.4	5.02	200122.9	225024.3	192194.4	205625.8	218804.5	201735.1	204712.3	189904.8	222366.0	219012.1	#N/A	#N/A	#N/A	#N/A
Q3UJ22	INT_SG_DDX_CT_C domain-containing protein	Gm648	213	23.4	8.35	74578.5	75226.7	62600.7	68990.7	53019.0	47846.9	41790.4	61065.2	66802.1	62818.5	83501.5	71578.6	73002.5	72774.5
Q9CY52	Probable tRNA(His) guanylyltransferase	Thg1l	298	34.9	8.57	77708.3	69109.6	78346.8	61602.2	78093.9	75919.6	70970.0	73785.3	72733.9	68848.1	95356.4	83621.8	93869.2	87153.0
Q8BYH7	TBC1 domain family member 17	Tbc1d17	645	72.8	5.25	60478.7	52499.1	55412.2	51210.9	48018.4	57383.1	55092.2	55779.1	51445.0	48033.6	196434.3	213406.1	195937.3	211125.6
O35451	Cyclic AMP-dependent transcription factor ATF-6 beta	Atf6b	699	76	6.65	57048.4	71370.0	61132.7	62311.9	58848.1	73827.2	57142.4	62950.0	62930.3	67674.2	#N/A	#N/A	#N/A	#N/A
Q9DB34	Charged multivesicular body protein 2a	Chmp2a	222	25.1	5.97	143364.6	159285.5	143487.5	168771.3	150144.0	167338.5	143983.6	160038.9	144681.7	137989.5	280617.8	348973.0	286700.6	344991.1
Q9JHF5	V-type proton ATPase subunit a	Tcirg1	834	93.4	6.38	85944.1	92771.9	80883.1	83238.7	92807.9	92234.1	84778.8	90629.6	84100.9	87115.7	57582.9	54550.4	53668.7	61010.6
Q61666	Protein HIRA	Hira	1015	111.7	8.06	55233.2	48910.5	48865.8	51688.4	52400.9	57217.4	53481.8	50789.7	56021.8	53058.1	85759.9	68947.1	64289.5	70907.9
Q68EF0	Rab-3A-interacting protein	Rab3ip	428	47.1	6.71	34680.7	34987.6	34381.8	32200.1	35976.0	34836.9	26452.5	38492.9	34505.3	36213.5	#N/A	#N/A	#N/A	#N/A
Q60738	Zinc transporter 1	Slc30a1	503	54.7	6.62	114645.9	114138.4	101532.5	113331.1	109518.2	134270.1	111380.2	98534.8	105040.9	96273.5	47609.0	48425.3	47064.7	48990.5
P46737	Lys-63-specific deubiquitinase BRCC36	Brcc3	291	33.3	5.83	109046.5	92048.2	101985.4	103021.4	100068.3	100772.4	94290.1	110847.1	103237.4	99891.2	79509.1	154194.3	72763.7	122655.1
Q61884	Meiosis-specific nuclear structural protein 1	Mns1	491	60.2	6.65	60503.1	75484.8	74654.0	90161.4	48565.9	46621.2	46811.2	56186.8	57227.0	57405.9	237163.2	255467.1	240553.1	257020.8

Q9DB30	Phosphorylase b kinase gamma catalytic chain, liver/testis isoform	Phkg2	406	46.5	6.48	81986.0	94431.0	82902.6	87681.5	98183.6	99737.9	83857.2	84832.8	89460.7	88730.2	148223.8	157922.1	145249.1	149161.1
Q8BTW9	Serine/threonine-protein kinase PAK 4	Pak4	593	64.6	9.85	164825.2	181146.6	163395.4	175017.3	189033.1	189656.3	157166.5	161306.1	160831.2	163447.4	136406.8	148109.9	130813.1	138876.9
P41317	Mannose-binding protein C	Mbl2	244	25.9	5.06	123949.8	138451.6	92586.0	137701.2	84401.0	101018.5	94778.5	113417.7	85057.8	97059.0	44697.2	48319.1	39794.6	41323.3
O35047	Homologous-pairing protein 2 homolog	Psmc3ip	217	24.7	7.4	199406.6	232170.2	254567.5	250446.7	229797.9	220665.2	190067.9	206988.6	227111.7	230652.5	#N/A	#N/A	#N/A	#N/A
Q99LJ7	RCC1 and BTB domain-containing protein 2	Rcbtb2	551	60.1	5.49	25628.1	29500.6	26853.7	26187.1	27418.6	28318.7	27266.2	23045.0	28626.0	33104.2	#N/A	#N/A	#N/A	#N/A
Q76LS9	Ubiquitin carboxyl-terminal hydrolase MINDY-1	Mindy1	468	51.2	4.73	22963.8	31543.5	24508.5	27667.9	28594.5	28470.2	27611.5	26345.4	25468.1	29687.5	31268.2	32473.0	26544.3	30464.2
O70281	Protein-tyrosine sulfotransferase 1	Tpst1	370	42.1	9.09	79703.5	81010.3	72553.2	76671.6	74635.4	72975.7	62500.8	74821.2	76497.5	72912.7	45069.8	51660.2	44602.1	49584.9
Q9DCD5	Tight junction-associated protein 1	Tjap1	539	59.4	6.07	107743.8	111751.9	107188.0	107070.4	114752.2	116234.9	107447.3	111421.5	109464.0	111912.6	58740.0	59145.6	61655.4	59173.7
Q6RT24	Centromere-associated protein E	Cenpe	2474	286.3	5.31	143258.6	151120.0	137170.4	147536.3	148942.8	151448.0	131861.0	135293.0	142734.6	153555.8	37734.9	49407.2	44376.0	52469.2
Q9D720	Non-structural maintenance of chromosomes element 1 homolog	Nsmce1	266	30.7	6.89	135279.5	159336.7	175818.8	178651.4	144089.2	137947.4	103448.1	117346.7	137353.9	142798.2	39572.0	43606.1	46680.4	47283.5
Q8BMI3	ADP-ribosylation factor-binding protein GGA3	Gga3	718	77.9	5.66	92613.4	101694.8	97875.9	70413.0	75516.5	74430.9	63868.4	109262.6	102108.3	64413.0	53582.6	60383.1	46287.0	50625.3
Q99N87	28S ribosomal protein S5, mitochondrial	Mrps5	432	48.2	10.14	300443.1	320077.1	311734.3	314452.6	307600.8	288727.8	281945.6	292433.2	288538.5	327063.0	184929.4	225870.0	174416.6	194308.3
Q9QXE0	2-hydroxyacyl-CoA lyase 1	Hacl1	581	63.6	6.27	199124.6	191377.6	186106.7	201733.3	190750.0	199615.8	186330.4	197202.0	211000.8	209395.8	128242.2	127888.6	127247.5	141625.4
Q9WU00	Nuclear respiratory factor 1	Nrf1	503	53.5	5.05	126536.0	119456.4	114538.7	112682.0	119953.0	128136.7	134224.3	116948.6	122452.4	124662.4	205687.5	188856.6	182913.0	191500.6
Q9Z2T6	Keratin, type II cuticular Hb5	Krt85	507	55.7	6.42	183283.5	202611.7	162533.8	214037.3	192247.0	208594.4	172786.6	167851.9	204855.9	181097.4	#N/A	#N/A	#N/A	#N/A
P53798	Squalene synthase	Fdft1	416	48.1	6.32	70439.5	79311.8	72205.1	85486.9	77123.8	78978.1	67917.9	74763.7	129775.1	76216.9	120048.2	128916.3	113923.0	108456.0
Q8BVF7	Gamma-secretase subunit APH-1A	Aph1a	265	29	7.9	24386.7	29688.5	21805.4	25712.7	28158.5	27832.9	20636.3	29802.5	24031.2	23183.7	77407.5	82387.7	74329.1	76548.0
Q80TR1	Adhesion G protein-coupled receptor L1	Adgrl1	1466	161.6	6.48	60750.8	65129.6	61260.6	70103.6	70089.0	64404.2	66702.7	65791.3	77448.8	66852.7	82805.2	86407.3	79728.4	85968.4
O35492	Dual specificity protein kinase CLK3	Clk3	638	73.8	9.94	67151.4	56462.0	62488.4	64407.0	64258.8	63410.6	54758.3	66343.3	72325.5	72856.8	104260.8	109741.4	102370.6	112855.3
P55098	Peroxisome biogenesis factor 2	Pex2	305	34.7	8.46	139394.4	127678.5	123557.4	125349.1	121889.6	124540.8	127549.0	127382.6	136699.2	134028.8	126849.4	125033.4	103023.2	118106.4
Q3UIK4	N6-adenosine-methyltransferase non-catalytic subunit	Mettl14	456	52.1	6.21	436197.9	440200.4	447708.8	455164.7	533989.5	501621.9	587240.7	453830.8	540507.6	524280.0	279470.0	276706.4	282697.9	273749.3
Q8VI64	Protein MGARP	Mgarp	283	29.9	4.18	3067.7	7988.0	5909.7	5729.4	7106.7	6406.6	6804.6	4928.1	3536.7	4650.1	31916.1	32135.3	25509.6	31837.6
Q8BHY8	Sorting nexin-14	Snx14	964	111.8	6.83	158012.6	153057.0	130821.0	158739.7	146313.9	155097.3	139279.7	118648.8	140476.2	159417.6	122969.1	123947.2	118852.0	125393.8
Q6NXM2	RCC1 and BTB domain-containing protein 1	Rcbtb1	531	58.3	6.43	148445.7	203520.1	134319.4	180952.3	157316.4	172726.6	145981.6	164108.6	160730.6	173766.1	95167.4	124053.7	84524.4	106259.3
P53808	Phosphatidylcholine transfer protein	Pctp	214	24.8	7.03	173663.5	175000.5	177654.9	190327.6	179305.1	186837.4	186439.2	161529.4	197896.9	199636.1	130064.5	159755.1	170773.7	174406.5
Q99JW1	Alpha/beta hydrolase domain-containing protein 17A	Abhd17a	310	33.9	6.76	59912.2	58951.3	51556.1	57008.7	64581.4	53888.4	58846.4	61363.1	51982.8	60874.3	59198.0	64403.2	53576.4	57032.5
Q8VED2	Biogenesis of lysosome-related organelles complex 1 subunit 4	Bloc1s4	215	23.1	5.14	311447.2	293319.3	288539.2	319009.0	285961.9	256029.0	272253.3	263575.6	268119.0	291456.2	31606.1	30837.3	30083.3	29121.4
Q3U466	Ceroid-lipofuscinosis, neuronal 6	Cln6	308	35.8	8.9	182770.1	189447.1	183394.0	181891.9	194435.8	191490.5	170015.7	183591.5	195501.4	189614.2	84560.0	74243.5	88412.0	70130.3
Q9DCG9	Multifunctional methyltransferase subunit TRM112-like protein	Trmt112	125	14.1	5.27	112961.4	140856.0	120488.0	121703.7	137415.6	136668.3	122465.1	121262.1	130827.8	119020.2	180578.4	163344.5	158593.6	169056.4
P50608	Fibromodulin	Fmod	376	43	6.04	530416.8	504237.1	458055.6	554637.3	441001.2	531008.5	518727.0	565864.1	526987.3	482624.3	222821.7	218519.1	168253.6	216278.3
Q9CZT4	DNA-directed RNA polymerase III subunit RPC5	Polr3e	710	79.8	6.34	95070.7	88465.7	88641.2	87170.6	89547.2	94843.2	88410.9	99866.9	82854.2	80270.8	159713.8	158819.2	172449.4	168988.5
Q4JIM5	Tyrosine-protein kinase ABL2	Abl2	1182	128.1	7.84	27269.8	26764.1	26891.8	24221.7	33175.2	36395.7	26875.0	23702.3	32980.7	28798.3	24620.7	29870.8	27477.8	24923.7
Q8BG48	Serine/threonine-protein kinase 17B	Stk17b	372	42	5.31	161867.9	185564.9	192636.2	182114.5	165154.5	160798.9	149128.0	155903.1	190763.5	185194.6	#N/A	#N/A	#N/A	#N/A
O70279	Splicing factor ESS-2 homolog	Ess2	479	52.6	6.7	71716.1	68792.6	68920.7	72206.7	67127.5	71451.1	67933.2	62573.3	62918.1	66887.4	77586.8	67511.8	65009.5	78190.3
O88351	Inhibitor of nuclear factor kappa-B kinase subunit beta	Ikbkb	757	86.6	6.4	29348.2	27696.8	28432.9	24146.6	28392.8	27309.6	26875.3	28047.9	30448.6	29145.8	56036.0	66341.3	54365.8	55991.5
Q9CY16	28S ribosomal protein S28, mitochondrial	Mrps28	186	20.5	8.92	334180.7	376862.8	375046.0	346487.7	432255.0	385455.7	368125.4	383462.6	393743.0	385186.0	#N/A	#N/A	#N/A	#N/A
Q8CEI1	BolA-like protein 3	Bola3	110	12.2	8.97	311877.5	327603.3	324768.4	359968.9	304691.8	285118.5	310036.7	312695.8	336773.2	298368.4	161780.9	171691.5	166628.5	164921.8
Q9DBM2	Peroxisomal bifunctional enzyme	Ehhadh	718	78.3	9.13	158883.4	165757.1	139739.4	145984.2	149543.7	155087.5	155827.1	135048.8	154524.0	159699.3	134821.8	156653.5	119314.4	140531.5
P59281	Rho GTPase-activating protein 39	Arhgap39	1107	125.1	7.97	145221.4	163623.4	148551.1	151521.7	151770.4	148957.7	136120.2	134274.2	144020.8	148130.7	#N/A	#N/A	#N/A	#N/A
Q9DB40	Mediator of RNA polymerase II transcription subunit 27	Med27	311	35.3	9.31	86408.4	109642.7	77637.2	93915.9	84934.0	91928.6	93351.7	77404.7	86766.0	101942.8	255922.6	260851.6	246291.0	240108.6
A0A338P6S8	ABI gene family, member 3 (NESH)-binding protein	Abi3bp	1637	179.4	9.83	88736.4	89458.0	80415.3	79662.9	94545.1	128035.8	107616.6	94722.9	87952.6	88857.6	330407.8	328720.9	267132.9	327045.4
Q2TBA3	Mucosa-associated lymphoid tissue lymphoma translocation protein 1 homolog	Malt1	832	93.2	5.81	139456.6	156421.5	165233.6	152583.1	178534.0	142051.8	156642.9	142686.0	153747.0	157744.3	20442.4	27004.9	24277.0	27269.1
Q9D0L4	AarF domain-containing protein kinase 1	Adck1	525	59.7	8.05	47597.8	62184.0	48339.2	52291.4	46919.8	47759.0	45797.3	47442.1	53244.0	51527.5	259641.3	254037.2	275945.3	264208.7
Q8C1Z8	tRNA methyltransferase 10 homolog A	Trmt10a	328	37.9	7.61	259682.1	267324.2	253716.2	255647.1	260431.8	251078.7	214254.4	233031.8	262395.3	284257.5	75077.1	85331.3	83599.7	84896.4
Q62273	Sulfate transporter	Slc26a2	739	81.6	8.13	177714.2	182620.7	163914.0	166799.2	167358.1	167166.4	168489.2	170413.2	176942.0	180209.1	221817.7	187704.4	222542.7	171010.5
Q4PJX1	Protein odr-4 homolog	Odr4	447	50	6.14	75229.7	73465.1	66159.3	62189.8	68959.3	84995.9	74321.8	67069.2	68255.5	74648.4	115014.6	109873.8	108627.8	107471.4
Q8BHY2	Nucleolar complex protein 4 homolog	Noc4l	516	58.6	6.77	8463.2	8490.1	8820.2	8757.7	8718.9	12859.6	7982.4	6970.6	6482.8	11288.3	90847.3	100177.7	101750.3	99569.5
Q61009	Scavenger receptor class B member 1	Scarb1	509	56.7	8.09	120982.8	110698.1	105060.4	112011.6	97850.8	99219.7	96462.3	97815.5	97975.8	95623.9	56001.9	64427.3	56691.9	61282.6
Q921K9	B-cell CLL/lymphoma 7 protein family member B	Bcl7b	202	22.2	4.75	157664.3	170677.5	156750.6	162498.0	161052.8	165940.6	172830.0	162268.3	164716.0	174892.1	134539.2	140869.3	125569.3	141331.7
Q9ET22	Dipeptidyl peptidase 2	Dpp7	506	56.2	5.39	101730.5	115892.4	102998.6	99040.1	111025.8	115945.2	105706.2	106067.0	108279.6	106719.7	69629.3	87158.5	84796.7	87954.8
Q66T02	Pleckstrin homology domain-containing family G member 5	Plekhg5	1073	118.9	6.87	102459.5	95435.0	85016.1	98673.5	93041.9	93772.0	84992.5	85976.4	93999.3	93409.4	214169.2	219117.1	197968.0	210979.1
P25425	POU domain, class 2, transcription factor 1	Pou2f1	770	79.5	6.57	106200.7	153847.8	119208.8	126864.8	142337.6	143621.4	127738.5	132425.7	126073.9	140538.5	#N/A	#N/A	#N/A	#N/A
Q8R1I1	Cytochrome b-c1 complex subunit 9	Uqcr10	64	7.4	9.19	291735.0	305547.3	346562.1	341003.6	313744.6	286732.6	272598.9	328419.9	308070.8	297664.5	191502.2	185279.5	226491.5	220957.9
Q8R368	Dynein assembly factor 4, axonemal	Dnaaf4	420	48.1	8.69	37911.8	48205.2	48815.7	49841.3	25030.8	29241.5	30866.0	31286.4	40936.4	43465.4	29399.3	36275.8	35163.0	35418.6
Q8BR76	Meckelin	Tmem67	992	111.7	7.74	23814.8	29604.4	24768.8	25856.5	27379.6	31315.4	25481.3	23657.3	31629.9	29158.2	38129.7	33501.9	34751.2	37825.2
Q91WA6	Sharpin	Sharpin	380	39.8	5.76	28447.7	31217.5	32246.6	27631.4	37799.9	32223.9	32962.1	32902.5	31879.0	35509.3	53893.1	50290.6	50419.4	48791.1
Q810U5	Coiled-coil domain-containing protein 50	Ccdc50	305	35.3	6.89	532204.5	491100.7	512715.6	516918.6	467688.4	649908.4	633879.5	630656.7	490558.0	471570.0	291324.0	291461.8	284794.8	284268.1
Q9CQ18	Ribonuclease H2 subunit C	Rnaseh2c	166	17.8	5.8	16640.4	20439.5	16715.9	15773.1	20543.0	17671.5	18885.8	13783.0	17815.5	18126.0	91729.3	94643.3	74615.0	88264.3
Q923K4	tRNA modification GTPase GTPBP3, mitochondrial	Gtpbp3	492	52.1	6.05	30122.0	31811.9	40613.1	38760.9	31865.9	33625.8	25790.7	37330.6	34732.9	30178.5	12056.2	14678.5	18179.8	11664.9
Q9D187	Cytosolic iron-sulfur assembly component 2B	Ciao2b	163	17.7	4.98	15376.4	16040.0	17369.8	16044.7	14877.8	17146.6	16275.3	15789.5	15216.0	15063.2	152573.8	169444.2	178092.2	182786.8
Q3TAA7	Serine/threonine-protein kinase 11-interacting protein	Stk11ip	1072	117.9	4.96	24899.5	23127.4	22033.3	26938.0	27901.4	26264.7	25404.5	22325.2	24589.1	22210.4	26094.9	35850.9	24237.0	25052.2
Q8C3Q9	Caspase-9	Casp9	454	49.9	6.83	83334.9	82241.7	67307.5	80414.1	79069.3	79616.8	72289.8	77213.9	78902.6	90636.9	14417.0	16837.2	18980.2	16845.5
Q80YQ8	E3 ubiquitin-protein ligase RMND5A	Rmnd5a	391	44	6.06	233432.9	249192.3	251197.4	251341.3	256332.4	238736.7	232534.9	227117.8	258625.9	250639.1	182034.9	209558.9	193340.7	210735.7
Q9JKP7	DNA polymerase epsilon subunit 3	Pole3	145	16.6	4.74	175432.2	182534.5	160124.5	201650.9	159859.7	155549.4	158611.0	163706.9	159153.9	162253.1	765408.4	844905.6	909687.5	898268.2
Q9DBT3	Coiled-coil domain-containing protein 97	Ccdc97	340	38.7	4.6	40490.9	41052.7	33537.3	41294.3	35263.9	33160.2	36781.0	31756.9	36193.3	38940.9	59305.7	58469.2	58194.3	58115.1
Q8QZY2	Glycerate kinase	Glyctk	523	55.3	6.67	43220.0	42545.9	46597.3	49372.9	48993.9	45432.9	44513.5	46169.4	46541.3	45169.2	53811.6	59306.8	48040.2	51765.5
Q9DBN4	Putative monooxygenase p33MONOX	P33monox	303	32.7	9.42	157886.4	163532.0	164428.7	161542.8	181004.0	171183.6	162366.4	155638.2	154315.6	172980.2	226658.3	229637.9	223195.1	220905.9
Q8BHN0	Protein phosphatase 1L	Ppm1l	360	41	5.99	102985.1	96971.2	108492.8	79747.2	115522.0	113905.2	109609.6	111580.6	103299.4	103392.1	124494.9	111933.7	127208.5	120117.6
Q99KR8	Plasma alpha-L-fucosidase	Fuca2	461	53.6	6.61	48234.6	48394.1	40952.5	50154.9	45414.3	44577.3	45356.7	42696.5	46728.6	42771.0	56883.9	67326.9	51288.3	54352.3
O09111	NADH dehydrogenase [ubiquinone] 1 beta subcomplex subunit 11, mitochondrial	Ndufb11	151	17.4	5.22	35350.5	33437.4	35549.7	44098.8	38507.8	37179.5	40603.1	36596.0	40329.8	41526.6	21488.9	25828.9	23115.8	28369.4
Q9D9H8	UPF0565 protein C2orf69 homolog	MGI:192071	365	41.7	7.87	91841.4	116795.2	109892.3	115580.4	99729.5	102181.5	87049.1	89074.1	104563.0	113386.1	145120.3	166609.1	151461.0	164828.6
Q8C145	Zinc transporter ZIP6	Slc39a6	765	86.3	6.84	190061.7	196078.4	204932.2	205556.8	169474.8	175123.0	202565.8	166597.7	191462.8	219670.1	54267.5	58527.9	58089.1	62628.5
Q8BFQ8	Glutamine amidotransferase-like class 1 domain-containing protein 1	Gatd1	220	23.3	7.05	75142.4	73684.8	78611.8	71869.3	71892.3	83562.6	79401.5	83391.4	66950.6	76232.6	218977.4	202666.2	194315.0	196164.3
Q8BXG3	Intraflagellar transport protein 57 homolog	Ift57	429	48.7	4.98	34829.1	47524.2	35074.6	44406.4	37467.0	25027.8	32239.7	33475.7	38188.9	42542.7	#N/A	#N/A	#N/A	#N/A
Q9D6Y7	Mitochondrial peptide methionine sulfoxide reductase	Msra	233	26	8.41	134575.5	142501.0	138862.7	135367.3	149274.5	142125.1	142676.1	134142.1	130210.8	142603.0	138485.7	119626.1	144027.7	123696.8
P70428	Exostosin-2	Ext2	718	82	6.65	18082.3	21566.0	19495.0	19948.6	24452.5	20438.3	20739.2	19341.0	25010.6	19018.7	#N/A	#N/A	#N/A	#N/A
Q9D4H9	PHD finger protein 14	Phf14	881	99	5.34	14108.3	10809.5	13938.9	12597.1	12063.1	12776.2	13505.4	13568.6	14363.7	11368.9	346537.0	360116.3	326020.4	348216.7
Q6DTY7	6-phosphofructo-2-kinase/fructose-2,6-bisphosphatase 4	Pfkfb4	469	54	6.48	155943.3	167985.0	152888.5	180543.4	150151.7	140954.4	149886.7	137424.1	156704.6	161554.7	117252.9	121629.6	115265.6	123683.7
Q99PI4	Phosphatidate phosphatase LPIN3	Lpin3	848	94.3	5.78	9994.9	9666.8	10077.0	8237.3	9068.2	10422.6	8062.3	9560.2	11287.7	9380.3	40856.5	42051.7	37868.3	37603.7
Q8BH65	Protein DENND6A	Dennd6a	605	68.9	7.93	98415.2	102119.2	103931.5	90453.4	102545.4	108267.4	103779.9	112562.7	102064.3	105172.0	148485.6	160293.5	163804.2	154035.9
Q52KE7	Cyclin-L1	Ccnl1	532	60.1	10.7	40380.4	49216.4	43432.0	42127.8	48212.2	50185.8	44696.3	41144.2	43593.2	45824.0	63142.7	69129.5	62462.0	66249.0
Q91W67	Ubiquitin-like protein 7	Ubl7	380	40.4	5.01	9398.6	8457.0	6951.2	12308.0	12543.5	13301.7	12713.8	11208.5	8614.0	7632.8	210154.2	236329.8	242370.1	253726.9
Q9Z0S9	Prenylated Rab acceptor protein 1	Rabac1	185	20.6	7.9	302547.5	322988.0	298353.9	351276.2	306516.9	272037.9	226174.3	328657.5	344264.5	293279.5	429157.4	471403.9	417897.8	448678.5
O08739	AMP deaminase 3	Ampd3	766	88.6	7.33	124782.5	102999.3	115458.4	113467.4	102914.2	127713.4	169384.8	116568.2	112795.2	102444.3	106908.0	116269.2	101757.1	96830.4
Q6P5C5	Single-strand selective monofunctional uracil DNA glycosylase	Smug1	279	30.6	6.77	58398.3	62146.4	56696.3	57372.7	62154.4	58794.8	64568.0	59732.9	61992.7	59141.5	#N/A	#N/A	#N/A	#N/A
P03911	NADH-ubiquinone oxidoreductase chain 4	Mtnd4	459	51.8	9.38	54111.3	78763.9	56716.6	57696.5	63073.1	62113.6	63342.9	59797.8	65494.9	64412.0	102815.6	135019.5	125476.3	145792.5
Q99LZ3	DNA replication complex GINS protein SLD5	Gins4	223	25.9	5.24	385744.0	633854.6	511641.9	548193.6	554089.2	550285.9	363899.4	433536.4	524210.7	587812.5	78906.6	89681.0	94012.4	88671.7
O35671	Integrin beta-1-binding protein 1	Itgb1bp1	200	21.6	6.54	26309.5	31702.2	29006.7	28798.1	32079.0	37316.1	30275.2	26435.7	35544.9	32930.2	8736.0	10529.2	9278.3	10409.5
P15535	Beta-1,4-galactosyltransferase 1	B4galt1	399	44.4	9.38	169995.1	135875.8	150124.3	149299.7	135911.2	123809.7	138595.3	137450.9	130952.3	146269.8	115147.1	106735.1	114253.9	112753.5
Q3UDK1	TRAF-type zinc finger domain-containing protein 1	Trafd1	580	64.2	5.48	153464.0	175729.7	166416.4	184637.6	151548.6	161756.4	131828.1	134077.3	172638.1	164766.9	146698.1	170156.1	171907.0	183355.7
Q8BKT3	GC-rich sequence DNA-binding factor 2	Gcfc2	769	87.4	5.55	93658.6	37309.9	43365.1	43466.4	40705.7	46104.7	108304.3	39613.1	45358.3	44930.0	29974.2	32308.1	32643.2	37297.0
Q8BWY7	Zinc transporter ZIP11	Slc39a11	342	35.4	5.11	133294.2	136177.0	119135.3	126748.7	137845.8	132180.7	135989.8	130201.1	130462.6	132101.3	286904.5	306978.1	270374.9	294624.9

Q9D125	28S ribosomal protein S25, mitochondrial	Mrps25	171	19.9	9.23	380275.3	449075.1	364428.5	447645.1	377819.2	379860.9	383105.8	380776.4	394520.6	438574.5	129009.8	114621.3	104789.8	122365.6
Q8C7H1	Methylmalonic aciduria type A homolog, mitochondrial	Mmaa	415	45.9	9.32	482032.6	415054.0	399082.1	411849.5	427716.1	390434.1	412886.5	419464.5	404626.1	422496.7	252682.9	254457.5	224878.8	231192.4
Q9CQE3	28S ribosomal protein S17, mitochondrial	Mrps17	120	13.4	9.89	72492.2	67858.6	70646.4	62248.7	71309.1	64952.9	69405.7	63816.5	64279.2	67503.0	88631.4	83494.8	92026.8	77935.8
P54279	Mismatch repair endonuclease PMS2	Pms2	859	95.2	7.39	65811.1	73437.5	68616.5	67487.1	79351.5	71641.0	73516.8	66839.1	61002.1	76677.9	105921.3	132101.0	117945.2	119715.9
Q9D6J5	NADH dehydrogenase [ubiquinone] 1 beta subcomplex subunit 8, mitochondrial	Ndufb8	186	21.9	6.64	101120.8	105433.5	129602.9	113249.1	121172.4	136380.8	115945.5	114169.0	113127.4	109361.6	109239.7	94903.2	130886.4	118810.6
Q8BGB2	Tetratricopeptide repeat protein 7A	Ttc7a	858	96.1	6.2	84192.9	89914.6	84375.3	84670.2	95669.5	103138.4	88544.4	86515.5	93868.6	89339.1	374081.2	371342.8	375303.8	365298.9
P97364	Selenide, water dikinase 2	Sephs2	452	47.8	6.06	229303.3	267859.4	272549.0	321127.6	190421.5	201582.8	202649.6	206032.9	236940.6	235776.8	292005.0	353447.9	316600.4	374992.2
Q5SXY1	Cytospin-B	Specc1	1067	118	6.64	168004.9	161653.9	164439.8	162218.3	191465.5	184006.2	180663.5	182885.9	172181.8	180850.1	255551.1	280518.5	237519.9	265367.7
J9JI72	PAS domain-containing protein	Gm1141	758	85.2	6.07	125789.0	132639.5	144042.9	123198.3	145546.3	130502.4	117681.5	123832.5	123818.4	132103.5	#N/A	#N/A	#N/A	#N/A
Q7TSS2	Ubiquitin-conjugating enzyme E2 Q1	Ube2q1	422	46.1	5.1	279918.5	309021.7	269810.4	295388.8	298369.1	302382.5	278217.5	275413.2	284894.0	312530.6	#N/A	#N/A	#N/A	#N/A
Q9EPB5	Serine hydrolase-like protein	Serhl	311	35.3	8.1	180427.0	176049.1	165777.7	179920.4	176422.2	171472.8	170832.8	179539.4	174116.0	169803.2	161581.1	167655.0	152608.4	161185.5
Q566J8	Atypical kinase COQ8B, mitochondrial	Coq8b	533	59.2	7.93	127749.3	141589.9	114450.0	144705.3	136165.1	123262.0	119480.8	115427.3	124602.0	136124.9	150803.8	170697.1	146181.8	149917.6
Q9DAM1	Leucine-rich repeat-containing protein 34	Lrrc34	415	46.6	6.84	99933.1	113663.0	108142.9	153224.7	94256.7	85647.4	104336.7	95184.2	100126.5	108348.6	43991.7	39543.3	43327.2	54920.0
Q61738	Integrin alpha-7	Itga7	1179	129.2	5.71	222854.3	242971.5	239114.8	233230.7	205577.7	212799.8	218947.1	218168.4	228569.6	237648.3	136125.7	157742.5	177768.1	159583.5
P19258	Protein Mpv17	Mpv17	176	19.7	9.44	145972.0	176904.2	129567.8	146637.5	128745.6	120297.8	133854.4	123531.9	135804.9	144085.3	123262.1	124041.8	116992.6	125339.4
Q01279	Epidermal growth factor receptor	Egfr	1210	134.8	6.86	132871.9	123543.2	96547.4	134848.9	110190.1	118765.6	117630.8	124472.1	112091.1	107268.4	193830.9	195907.1	167401.0	197098.5
Q8BGA9	Mitochondrial inner membrane protein OXA1L	Oxa1l	433	48.2	9.61	146822.1	143664.4	139369.9	144559.2	127849.6	130869.6	132038.0	131100.5	127036.5	127559.3	129680.8	121389.6	112872.6	119221.0
Q91VT1	E3 SUMO-protein ligase NSE2	Nsmce2	247	28.2	8	258018.7	352509.0	334334.4	383544.0	290202.4	256565.9	204783.6	241520.1	292501.3	331156.2	280186.9	210067.1	195447.4	231305.0
P97819	85/88 kDa calcium-independent phospholipase A2	Pla2g6	807	89.5	7.24	44193.2	50785.3	47837.1	53783.2	48285.5	46686.4	46452.7	41024.7	47049.5	47509.9	39537.6	40909.1	35416.6	42406.6
Q3UDW8	Heparan-alpha-glucosaminide N-acetyltransferase	Hgsnat	656	72.5	8.27	105915.3	112763.1	92365.8	120236.0	87103.1	78804.0	81199.3	93994.4	86861.7	87122.4	105223.2	112430.5	89197.0	123193.9
Q7TMS5	Broad substrate specificity ATP-binding cassette transporter ABCG2	Abcg2	657	72.9	8.54	109763.3	129829.7	109822.1	118332.0	138446.4	127926.9	126312.8	107840.4	127427.4	112967.9	56898.5	67114.8	54650.9	64336.3
Q99JP0	Mitogen-activated protein kinase kinase kinase kinase 3	Map4k3	894	101.1	7.64	47105.6	35989.6	50439.2	37729.4	46514.2	46353.4	45893.4	44568.5	44287.3	47239.3	#N/A	#N/A	#N/A	#N/A
Q9WUH1	Transmembrane protein 115	Tmem115	350	38.1	7.5	23864.1	33422.5	22769.8	23543.2	21102.8	27848.0	21687.8	20670.7	24713.3	28224.6	98063.3	107022.6	95523.6	99523.2
Q8C181	Muscleblind-like protein 2	Mbnl2	373	40.1	9	609322.1	641889.3	574966.6	577925.1	720575.6	701940.2	682022.7	643309.5	563602.9	612158.0	#N/A	#N/A	#N/A	#N/A
Q8VCE2	GPN-loop GTPase 1	Gpn1	372	41.6	4.88	29331.9	36364.3	31562.1	32241.1	31676.3	38378.9	30665.1	31497.9	30895.9	38106.4	33210.6	36552.1	36392.4	32688.4
Q99N95	39S ribosomal protein L3, mitochondrial	Mrpl3	348	39.1	9.55	282542.2	294329.1	292457.8	293691.4	295655.9	295417.5	276779.6	271923.7	286945.5	293089.2	75973.5	62901.1	61732.1	58005.8
Q8CGA0	Protein phosphatase 1F	Ppm1f	452	49.6	5.3	138548.2	158318.8	140706.2	168634.6	158831.2	172591.1	151506.3	146121.0	169411.9	160062.0	#N/A	#N/A	#N/A	#N/A
Q9D8Y1	Transmembrane protein 126A	Tmem126a	196	21.5	9.41	109713.1	140793.1	114247.9	140465.1	107311.6	101024.8	105959.2	115704.8	126694.4	125143.6	#N/A	#N/A	#N/A	#N/A
Q62172	RalA-binding protein 1	Ralbp1	648	75	5.92	137959.7	146701.5	146188.1	140634.3	152650.5	147289.1	124698.2	135807.5	134926.8	132238.2	178038.9	211913.5	175349.4	198800.2
Q9DBC0	Protein adenylyltransferase SelO, mitochondrial	Selenoo	667	74.2	5.83	242321.9	231360.2	250647.6	247248.6	240360.5	214996.8	238921.6	209918.9	229285.5	243562.7	135954.9	140683.4	152723.0	150974.9
O88653	Ragulator complex protein LAMTOR3	Lamtor3	124	13.5	7.34	690610.9	695952.9	656687.4	696077.9	748822.3	696213.2	728006.5	687531.6	688146.8	677029.0	567893.8	575762.8	501530.6	527923.8
A6PWY4	WD repeat-containing protein 76	Wdr76	622	69	9.36	23234.5	22945.5	20776.4	21482.0	30818.3	25048.0	28178.8	26352.7	24085.4	23431.8	#N/A	#N/A	#N/A	#N/A
Q5HZJ0	Ribonuclease 3	Drosha	1373	158.7	7.87	72055.7	67957.1	60186.3	61717.8	99260.5	68760.3	63098.9	63727.0	68540.3	59745.4	27113.4	29852.3	34779.7	35707.0
A2AKB9	DDB1- and CUL4-associated factor 10	Dcaf10	566	61.5	8.16	44552.4	46219.7	48491.5	48657.3	51552.8	45199.9	41249.5	47271.3	45756.8	46319.3	#N/A	#N/A	#N/A	#N/A
Q9DAV9	Trimeric intracellular cation channel type B	Tmem38b	292	32.6	9.11	147657.7	169341.1	176989.7	180398.2	167152.3	171228.0	180885.1	166178.5	180340.1	178175.3	137445.9	139606.9	142301.3	152989.9
O35988	Syndecan-4	Sdc4	198	21.5	4.41	58454.2	48577.8	41769.6	56064.6	50611.3	53267.7	62259.7	50815.4	44994.8	46940.9	50421.5	48572.2	70296.6	46967.2
O54818	Tumor protein D53	Tpd52l1	204	22.5	6.21	285778.0	257894.8	273404.4	271807.3	263459.6	224142.8	228672.3	250374.7	281815.9	303863.1	232569.1	246360.4	202071.8	216422.5
Q78IS1	Transmembrane emp24 domain-containing protein 3	Tmed3	221	25.4	5.96	166788.2	135724.8	161407.8	167079.3	147073.2	145223.5	135956.9	150168.7	159175.7	144434.4	101570.6	111830.8	108378.7	124021.8
Q9CQS4	Solute carrier family 25 member 46	Slc25a46	418	46.2	7.64	115949.1	117788.9	113653.9	113277.8	121887.1	154375.9	125931.9	125413.7	112255.8	105101.1	141091.6	124333.9	131182.5	131344.5
Q60604	Adseverin	Scin	715	80.2	5.88	118283.0	148245.0	102556.2	109840.4	101467.7	106825.4	113149.5	124018.4	107386.1	112882.9	135685.4	134584.8	106168.3	114804.3
Q8R079	Bifunctional apoptosis regulator	Bfar	450	52.9	6.89	69605.7	56850.0	56347.0	60235.6	55206.3	58568.4	59177.1	58179.8	57993.2	52287.7	#N/A	#N/A	#N/A	#N/A
Q8C1E7	Transmembrane protein 120A	Tmem120a	343	40.7	8.88	938175.1	886800.0	832356.7	907770.8	760259.6	725995.2	781565.6	839477.5	769772.9	758611.4	132250.5	144911.3	123934.5	136073.1
Q8BX05	Putative glycerol kinase 5	Gk5	534	59.8	7.24	94969.3	85297.5	75481.5	89899.0	80238.9	75178.1	78051.8	66915.7	86796.0	91518.5	65022.5	67864.9	53765.1	63283.2
Q6PEE3	Ribonucleoside-diphosphate reductase subunit M2 B	Rrm2b	351	40.8	5.01	15082.7	16730.5	12721.0	18186.3	19601.5	17530.5	16719.7	16504.5	16723.0	20197.1	50278.6	50297.4	44162.7	49174.6
Q9D110	5-formyltetrahydrofolate cyclo-ligase	Mthfs	203	23.2	7.02	79900.7	82585.6	71407.3	75187.4	86977.2	91559.4	92254.8	68481.2	82851.5	83771.8	12527.8	12434.0	8724.3	10635.8
E9Q9B3	B30.2/SPRY domain-containing protein	Spryd3	442	49.7	6.16	425567.2	467462.9	441591.5	427858.6	489212.2	496437.9	435077.9	428421.5	451515.1	518085.2	#N/A	#N/A	#N/A	#N/A
Q91YS8	Calcium/calmodulin-dependent protein kinase type 1	Camk1	374	41.6	5.35	67083.5	53619.3	58748.4	58664.2	63545.1	63944.3	58750.0	66025.0	65866.8	57897.8	146355.5	139790.0	124781.9	139077.5
Q80YA7	Dipeptidyl peptidase 8	Dpp8	892	102.1	5.81	117169.4	112104.3	113820.7	122972.6	128069.8	105852.0	107076.5	112675.8	107986.9	119570.5	214195.0	200078.4	199939.0	199863.8
Q66GT5	Phosphatidylglycerophosphatase and protein-tyrosine phosphatase 1	Ptpmt1	193	21.9	9.72	149116.9	145177.7	140465.7	145684.6	140093.7	138011.1	133605.5	129241.0	145694.0	142666.4	171444.7	143758.7	151843.3	146619.5
Q8R4C2	RUN and FYVE domain-containing protein 2	Rufy2	606	70	5.83	21194.5	25497.4	22071.8	27301.8	35391.4	35982.5	32082.0	26608.9	29228.4	27574.9	#N/A	#N/A	#N/A	#N/A
Q3TML4	Cor1 domain-containing protein	Gm773	206	24	6.18	126377.3	135489.4	153061.5	136109.5	149278.1	145710.5	114700.2	114124.9	139637.0	145260.8	124306.4	135769.3	136490.9	119900.0
Q8R2Z5	von Willebrand factor A domain-containing protein 1	Vwa1	415	44.7	6.54	56011.8	68680.8	56266.0	59578.0	62139.5	64847.3	50247.0	54132.1	60595.6	59223.4	47495.7	52207.3	53410.9	58652.7
Q9D1B9	39S ribosomal protein L28, mitochondrial	Mrpl28	257	30.2	9.29	577745.3	520124.8	533597.2	585547.9	523323.2	465863.7	517374.2	594136.9	498295.7	547980.3	298458.7	311662.7	344404.2	306590.1
Q6A058	Armadillo repeat-containing X-linked protein 2	Armcx2	784	81	9.33	106787.9	103214.7	93376.3	100925.5	95631.4	119857.9	90161.7	104293.7	94008.6	91147.7	#N/A	#N/A	#N/A	#N/A
Q80ZK0	28S ribosomal protein S10, mitochondrial	Mrps10	160	18.7	8.84	260636.0	277341.5	265713.1	257588.1	284619.5	284729.9	271994.2	286352.2	271496.8	256647.5	207129.2	224899.6	232532.9	205639.4
P33611	DNA polymerase alpha subunit B	Pola2	600	66.2	5.34	261726.2	351529.4	388223.2	470190.4	116028.2	116151.4	106584.1	159482.2	270821.1	316130.6	62824.1	76165.6	65339.3	62469.2
Q9QXL1	Kinesin-like protein KIF21B	Kif21b	1668	186.1	7.08	267050.7	261877.1	284967.3	251046.6	361777.8	359325.4	316746.7	276072.6	296005.3	310826.4	432474.4	378066.6	445151.4	345026.1
Q99NG0	Helicase ARIP4	Rad54l2	1466	162.4	6.13	112630.2	112769.3	107294.0	116621.0	117154.6	117325.7	116990.1	97615.7	112342.8	117192.6	127913.6	141182.3	143374.2	137306.2
Q8C790	Protein FAM221A	Fam221a	301	33.3	7.88	79785.4	96696.9	91249.3	105123.5	88353.8	83758.1	74654.6	75098.2	90918.3	94388.6	272296.9	281634.1	297355.8	304222.4
Q9QXG9	TERF1-interacting nuclear factor 2	Tinf2	341	37.9	8.46	46209.5	70421.8	51258.9	60314.9	66978.1	82550.0	47046.5	60378.8	59813.0	63727.0	165357.8	196853.6	176513.7	176285.6
Q8VC03	Echinoderm microtubule-associated protein-like 3	Eml3	897	95.6	7.05	141349.4	151441.7	118065.6	147697.2	145654.0	138299.1	135298.5	126285.1	140410.1	158981.0	84582.8	93557.2	79627.8	81479.6
Q69ZW3	EH domain-binding protein 1	Ehbp1	1231	139	5.38	123703.5	144380.1	121078.5	117614.5	128723.9	126550.2	119917.5	123371.6	127772.9	116867.2	191201.8	199921.2	171507.0	188997.2
Q8CHY3	Dymeclin	Dym	669	75.8	5.77	212885.5	214004.3	218349.2	207053.2	213162.3	229542.0	201070.1	204730.6	200151.9	210430.6	316041.9	357375.3	340247.7	364721.9
Q9Z255	Ubiquitin-conjugating enzyme E2 A	Ube2a	152	17.3	5.15	163150.9	150275.1	153685.5	141532.8	157418.8	164919.0	148188.2	153440.1	144712.1	152844.1	267896.3	255865.8	234313.9	236014.6
Q80US4	Actin-related protein 5	Actr5	605	67.8	5.19	46322.0	51799.7	49622.7	48676.4	47824.7	45385.1	40833.1	46298.7	44074.7	47350.7	57868.2	63624.8	53996.9	58700.4
Q9EQC8	Papillary Renal Cell carcinoma (Translocation-associated)	Prcc	491	52.3	4.94	231305.0	327791.4	210225.4	256980.6	233393.8	213478.5	209659.5	216854.5	235235.9	250274.7	20621.8	23161.4	24805.8	18673.0
Q8BHT6	Beta-1,3-glucosyltransferase	B3glct	489	55.3	6.92	243067.5	221190.9	197661.7	203362.9	203495.4	236811.2	243116.7	215445.1	221794.6	227495.4	#N/A	#N/A	#N/A	#N/A
P62700	Protein yippee-like 5	Ypel5	121	13.8	7.31	119422.6	137156.2	127832.8	121107.9	122300.6	142390.5	101602.1	122451.3	123227.0	138608.9	36320.2	42427.0	39770.2	41561.6
Q8C6B9	Active regulator of SIRT1	Rps19bp1	143	16	10.83	71487.2	72766.4	77140.7	75758.0	59034.1	60983.2	56948.2	63261.9	69326.1	63713.0	79699.9	79934.4	69709.1	90249.7
Q8BN21	Serine/threonine-protein kinase VRK2	Vrk2	503	58.1	9.04	45035.8	51550.9	40103.0	44216.9	47452.0	48721.5	46138.6	43345.7	40612.8	52870.2	37547.1	42246.8	49325.0	47950.7
Q6IQX8	Zinc finger protein 219	Znf219	726	77.7	9.17	90713.9	80972.2	98189.5	90425.3	92516.8	86402.2	88779.0	93081.9	89694.7	80530.0	130941.1	155496.5	132804.8	130822.8
Q9D945	Protein LLP homolog	Llph	130	15.4	10.36	57670.6	84775.2	59714.4	77876.9	56924.9	55541.9	53358.0	52720.9	67777.3	75595.3	80609.6	101423.3	95031.1	90276.7
Q924X2	Carnitine O-palmitoyltransferase 1, muscle isoform	Cpt1b	772	88.2	8.38	23970.5	24032.6	25666.9	22769.2	28045.5	25661.6	23448.0	19380.4	25143.8	29814.9	#N/A	#N/A	#N/A	#N/A
Q8BVW3	Tripartite motif-containing protein 14	Trim14	440	49.6	7.93	68081.8	69836.0	61479.5	62834.2	75120.5	79311.2	77719.6	65467.6	65259.0	72424.5	39246.4	34879.2	34640.3	32475.7
Q8BGT9	Polypeptide N-acetylgalactosaminyltransferase 12	Galnt12	576	66.5	7.24	139725.2	162573.8	131226.7	145847.8	157882.8	152772.9	148333.5	146795.4	148058.8	151997.7	146539.4	147744.2	126554.3	147816.9
Q9CPQ5	Centromere protein Q	Cenpq	267	30.5	9.31	70146.3	83882.6	84298.1	86845.7	82917.7	83945.4	67130.4	71245.0	75875.4	75809.1	53688.9	55431.9	59958.3	66875.4
Q3UK78	Polycomb group RING finger protein 5	Pcgf5	256	29.6	6.74	146284.7	162142.1	172081.5	174793.1	193177.5	174620.7	140010.7	155694.8	168293.2	184653.7	119906.8	139528.6	131327.8	129438.3
Q9CQN6	Transmembrane protein 14C	Tmem14c	114	11.6	9.7	92790.4	108556.0	96306.1	104577.6	101642.8	103964.6	94369.5	92507.8	103853.3	102475.1	58875.0	65997.7	57009.8	70023.9
Q9CQH8	Ribonuclease P protein subunit p14	Rpp14	122	13.6	8.28	117512.4	129384.3	102405.6	114635.5	123847.2	124407.5	113715.1	103445.1	116016.5	119039.5	39045.6	42084.5	35365.2	37145.7
B1B0V2	Expressed sequence AU022751	AU022751	589	61.8	9.55	48546.5	73601.6	72082.3	87162.5	54321.1	57143.3	45038.1	46305.2	63362.2	67091.3	2039.4	1816.6	3726.5	4614.0
P28650	Adenylosuccinate synthetase isozyme 1	Adss1	457	50.2	8.43	35061.8	41463.6	32085.4	37747.3	36343.3	43590.0	38828.4	37910.8	38174.9	40735.5	33254.0	33834.9	29502.5	34633.9
Q64676	2-hydroxyacylsphingosine 1-beta-galactosyltransferase	Ugt8	541	61.2	9.42	212698.3	235657.7	180055.7	200565.6	223348.5	217920.2	205947.9	190090.7	207492.1	212138.9	271225.5	388123.5	272370.0	325668.7
Q920V1	UDP-GalNAc:beta-1,3-N-acetylgalactosaminyltransferase 1	B3galnt1	331	39.3	8.02	114785.9	119273.6	124688.3	114104.0	105149.8	97210.2	88578.2	96932.7	127509.1	126312.9	#N/A	#N/A	#N/A	#N/A
Q91XE8	Transmembrane protein 205	Tmem205	189	21.2	9.42	154214.7	180246.2	158886.6	172791.7	172070.1	175959.0	216528.6	169183.3	171276.0	161721.8	515895.4	524659.9	492740.5	558816.2
Q9ER71	Rho-related GTP-binding protein RhoJ	Rhoj	214	23.8	6.7	72589.6	77149.9	60365.2	56878.1	75752.0	81954.6	78216.4	72588.1	64673.6	65366.8	#N/A	#N/A	#N/A	#N/A
Q9Z2Q5	39S ribosomal protein L40, mitochondrial	Mrpl40	206	24.3	9.47	118852.0	121334.5	99013.2	119515.2	109990.5	102990.5	112603.9	104805.5	113929.1	114399.3	132857.2	143807.1	122061.8	116538.4
Q9WV04	Kinesin-like protein KIF9	Kif9	790	89.8	6.86	32394.3	41849.1	31776.1	41760.6	39374.5	36045.0	32074.1	27222.3	34045.3	36419.4	26040.4	35589.1	26795.4	28640.9
Q8BSI6	R3H and coiled-coil domain-containing protein 1	R3hcc1	488	54.1	4.94	84123.9	95245.6	91549.8	98733.9	97631.1	97145.1	96046.1	84800.2	96422.4	97495.3	110313.8	119740.0	102867.9	111090.4
Q8R3Q2	Serine/threonine-protein phosphatase 6 regulatory subunit 2	Ppp6r2	923	100.4	4.82	48798.8	46724.3	41299.0	42963.6	47213.1	42444.3	48365.4	43272.3	42572.9	40770.3	78302.9	74110.3	67623.9	62522.3
Q5S006	Leucine-rich repeat serine/threonine-protein kinase 2	Lrrk2	2527	284.5	6.83	30487.0	35446.4	35793.1	35969.2	35121.5	36457.0	28014.1	30813.8	34130.8	34765.4	14128.4	20858.6	16627.7	15974.9
Q9D061	Acyl-CoA-binding domain-containing protein 6	Acbd6	282	30.9	5.11	46666.8	47131.3	51224.9	48830.0	50994.8	45902.7	35454.0	44161.0	50604.5	52751.9	182558.7	195379.8	165041.1	181279.4
Q9CRA7	ATP synthase subunit s, mitochondrial	Dmac2l	200	23.3	8.06	229127.6	227146.5	242283.3	252275.7	225083.0	228627.7	213136.5	221621.6	225495.7	238975.8	159024.1	158504.6	148949.9	165632.2

Q99J27	Acetyl-coenzyme A transporter 1	Slc33a1	550	61	8.16	148125.0	161893.9	177366.5	198431.0	146060.6	145022.4	118424.0	135935.4	138707.8	159411.6	132579.9	152552.3	148720.5	163105.2
Q812A5	Proline-rich protein 5	Prr5	387	42.5	7.84	51138.4	46963.9	45042.1	45082.8	53853.3	47136.5	51471.4	47725.0	42321.2	48440.0	49963.5	44821.7	46407.5	49934.2
Q69ZJ7	RAB6A-GEF complex partner protein 1	Ric1	1422	158.7	6.47	283884.5	295794.8	269231.5	298082.1	304873.4	285498.9	283964.6	274716.4	304720.2	303331.6	198588.8	199357.2	160558.0	171598.5
Q6P5D4	Centrosomal protein of 135 kDa	Cep135	1140	133.3	6.48	69666.7	84735.9	53320.2	55408.4	76183.6	74010.0	59923.1	62070.8	70156.8	72643.8	#N/A	#N/A	#N/A	#N/A
O08908	Phosphatidylinositol 3-kinase regulatory subunit beta	Pik3r2	722	81.2	6.07	77931.5	72925.0	71389.7	71953.2	70715.3	78619.5	65503.2	71033.8	78656.2	78392.0	#N/A	#N/A	#N/A	#N/A
Q9CQV5	28S ribosomal protein S24, mitochondrial	Mrps24	167	18.9	9.76	261106.0	297853.6	260369.2	270507.0	302201.8	271988.8	243837.0	245745.5	290318.3	290437.6	243957.8	264475.7	236122.0	238938.3
Q9CQC8	Maspardin	Spg21	308	34.9	6.28	131661.4	119036.2	117191.3	121119.7	128645.0	128842.4	126090.7	121629.2	117178.2	131776.9	247103.6	236351.1	239475.7	224535.9
Q3UBZ5	MIF4G domain-containing protein	Mif4gd	222	25.5	5.12	173621.3	202814.1	209702.0	207407.4	185970.1	171981.2	167995.2	185427.1	180434.5	198292.0	67464.8	88902.6	74627.4	88449.5
Q9CQV7	Mitochondrial import inner membrane translocase subunit TIM14	Dnajc19	116	12.4	10.1	254929.9	250972.6	270082.8	249034.9	293511.1	281260.9	278598.5	284103.8	250025.0	229760.5	237989.9	277416.4	296488.6	258946.1
Q7TSZ8	Nucleus accumbens-associated protein 1	Nacc1	514	56.5	5.73	249370.5	162912.1	173684.4	168581.5	196932.9	183708.0	168513.2	173412.9	176325.9	248682.7	110031.6	120758.3	118783.4	120817.8
O35386	Phytanoyl-CoA dioxygenase, peroxisomal	Phyh	338	38.6	7.53	81861.1	72711.9	71783.4	73146.6	73981.7	72048.9	69631.4	78408.0	69503.3	69050.0	80879.6	66305.6	73118.7	65226.4
Q80U44	Zinc finger FYVE domain-containing protein 16	Zfyve16	1528	166.6	4.78	53594.5	62399.2	53817.2	62833.7	58171.1	56697.1	50616.2	56610.4	57710.8	55269.3	64160.3	66001.2	62811.0	68247.1
P56135	ATP synthase subunit f, mitochondrial	Atp5mf	88	10.3	9.95	282967.1	310008.2	287374.7	319780.3	288020.8	271522.1	336018.2	276707.3	315900.6	300060.8	307220.2	321904.9	304430.8	320331.0
Q5XJY4	Presenilins-associated rhomboid-like protein, mitochondrial	Parl	377	41.9	9.92	448607.6	456176.0	434010.9	483362.0	427636.9	405179.9	394955.3	400817.5	450066.3	458618.1	75077.4	88458.8	84789.0	83593.7
Q3UZ39	Leucine-rich repeat flightless-interacting protein 1	Lrrfip1	729	79.2	4.82	155712.5	140081.0	139803.1	137648.2	156820.2	172014.9	179046.4	153871.3	138285.1	152816.8	279730.5	243681.4	243636.7	234306.7
Q9D1E8	1-acyl-sn-glycerol-3-phosphate acyltransferase epsilon	Agpat5	365	42.2	9.38	95402.5	98697.7	91628.5	94306.3	96168.5	94031.3	87556.6	78497.2	99285.6	92309.9	244956.4	249440.6	226852.6	269082.3
Q8BGV0	Probable asparagine--tRNA ligase, mitochondrial	Nars2	477	53.9	7.36	49805.0	44888.2	56694.7	25179.0	48225.0	52509.7	43478.5	53331.2	54374.0	46460.8	216764.6	186558.2	201541.0	183329.8
Q8K450	Sperm-associated antigen 16 protein	Spag16	639	71.4	6.09	137650.2	188351.4	194668.8	244711.2	100263.2	103974.2	91287.8	110329.4	133211.3	181126.3	34556.5	32920.8	35812.4	29440.9
Q9DBY1	E3 ubiquitin-protein ligase synoviolin	Syvn1	612	67.3	6.95	471300.0	446458.2	426485.6	440532.6	343520.9	355425.6	376188.6	455072.9	438606.8	401291.7	799525.5	1363934.8	695033.3	982077.3
Q9QXC1	Fetuin-B	Fetub	388	42.7	6.61	459734.6	420878.5	380501.4	419147.8	370520.4	402918.0	408376.9	432566.5	422291.5	444457.9	731018.3	612188.2	595462.8	566532.3
P56873	Protein ZNRD2	Znrd2	199	21.3	5.12	130880.1	139422.3	124235.1	143361.9	125874.4	122784.0	112232.0	106330.9	121818.5	129571.3	11454.3	8871.8	8447.1	10215.0
Q3U0J8	TBC1 domain family member 2B	Tbc1d2b	965	109.9	6.02	88121.1	88560.5	69570.3	74965.5	89204.3	99025.7	97517.5	83599.8	87704.2	93646.0	75021.1	64514.1	74702.2	61844.4
Q9D2R6	Cytochrome c oxidase assembly factor 3 homolog, mitochondrial	Coa3	108	12	9.86	159612.0	168749.2	209216.1	174656.4	188889.9	145504.7	147888.3	154796.6	177248.1	192083.2	90637.7	109276.1	94598.0	111354.0
Q91W98	Solute carrier family 15 member 4	Slc15a4	574	62.2	9.16	122017.8	103218.2	125452.6	103578.6	100120.1	128807.3	96872.4	123188.5	117835.2	117564.1	#N/A	#N/A	#N/A	#N/A
Q99MS7	EH domain-binding protein 1-like protein 1	Ehbp1l1	1716	184.7	4.73	64992.9	64062.2	69718.2	74779.9	60792.7	67552.2	57657.3	60543.9	64712.7	65151.8	49926.8	52197.6	50820.9	54539.3
Q60716	Prolyl 4-hydroxylase subunit alpha-2	P4ha2	537	61	5.8	174241.8	161673.3	176096.5	147958.3	168119.5	170690.5	142834.0	160240.3	146026.7	153702.6	133533.9	153437.4	138401.1	149224.9
Q8R366	Immunoglobulin superfamily member 8	Igsf8	611	65	7.99	185088.6	203107.1	132806.0	211786.7	189819.7	216069.2	187934.0	188134.0	192761.1	204810.7	115689.8	137836.6	103291.9	109107.1
Q6IFT4	Rho GTPase-activating protein 20	Arhgap20	1182	131.3	7.21	43157.4	34998.5	40015.5	36392.7	33317.8	29413.0	31013.3	39986.3	38816.8	38745.5	#N/A	#N/A	#N/A	#N/A
Q4QQM4	Tumor protein p53-inducible protein 11	Trp53i11	189	20.9	9.41	168341.2	161819.4	169098.3	163799.1	185435.0	164660.1	175000.8	167154.8	162409.3	174753.1	207636.5	196755.0	192225.5	211212.5
Q8BGN2	UPF0462 protein C4orf33 homolog	D3Ertd751	192	22.4	5.58	82975.4	76629.7	73581.7	76039.0	81942.3	82736.9	90135.2	74010.1	75707.4	76713.4	67835.3	69664.1	71547.1	65804.7
Q9D1K2	V-type proton ATPase subunit F	Atp6v1f	119	13.4	5.82	262695.5	260748.1	273218.7	291757.1	280762.4	270600.2	248725.9	262361.3	280396.9	263339.2	207259.6	220729.3	201711.8	223305.4
B1ASA5	Zinc finger protein 362	Zfp362	418	45.6	9.64	20397.9	20894.6	23812.9	20615.8	28708.1	26309.9	26284.0	23098.3	20659.8	20079.2	29282.2	27126.6	30454.0	27594.9
Q9D711	Pirin	Pir	290	32	7.11	119270.6	118017.0	112489.7	106437.1	127763.3	139636.4	123122.3	108917.3	104512.0	113651.3	26751.6	21549.5	23201.5	22162.4
Q8VDY9	Caspase activity and apoptosis inhibitor 1	Caap1	356	37.8	4.72	192402.7	179145.6	174160.5	171736.6	174026.7	183289.8	190238.0	167286.1	166920.8	182509.3	77048.8	86364.9	77583.8	56587.7
A2RSX7	tRNA wybutosine-synthesizing protein 5	Tyw5	315	36.6	6.34	79226.8	91884.6	91247.2	89629.9	87725.8	98562.1	92056.4	89956.3	90067.6	95791.4	192537.7	209182.5	207288.2	205614.3
P70289	Receptor-type tyrosine-protein phosphatase V	Ptprv	1705	186.7	6.23	128533.5	125570.1	104699.2	108747.3	102594.0	109511.7	104243.8	95558.6	106857.1	116397.8	#N/A	#N/A	#N/A	#N/A
Q62504	Msx2-interacting protein	Spen	3644	398.5	8.54	77849.4	82782.7	76877.5	83240.1	84136.3	87511.9	80966.6	77098.1	76239.4	85923.4	#N/A	#N/A	#N/A	#N/A
Q8BPI1	Protein kintoun	Dnaaf2	814	88.3	4.96	43553.2	76618.0	52865.4	68294.0	55860.8	52233.5	40272.5	53402.7	50745.8	61123.6	44854.0	60092.0	56268.6	62079.0
B2RXZ1	Poly(A)-specific ribonuclease PNLDC1	Pnldc1	531	61.3	8.38	51915.6	56577.9	55415.1	51970.8	55953.7	52015.0	48918.8	49582.5	57128.5	64073.7	12407.0	12201.8	12555.6	14731.2
Q9JME7	Trafficking protein particle complex subunit 2-like protein	Trappc2l	139	16	6.77	117820.6	168376.7	104482.0	177460.0	123484.1	104232.1	146575.4	85806.1	157262.1	162643.1	76155.8	85520.8	82841.6	88430.9
Q8BVA5	Lipid droplet-associated hydrolase	Ldah	326	37.3	8.28	138568.2	123891.3	117330.6	118521.5	123711.6	111626.2	110464.0	122738.7	121373.3	122259.8	42582.2	48722.9	44922.3	47778.0
Q8BW10	RNA-binding protein NOB1	Nob1	403	45.4	7.31	243175.2	239394.9	196094.2	214558.6	208646.4	192575.9	194122.6	194921.8	225839.1	235468.9	222670.7	225301.5	213578.7	211256.2
Q8K012	Formin-binding protein 1-like	Fnbp1l	605	69.8	6.64	82492.5	76563.7	75114.0	78748.2	90061.4	89444.0	85698.8	81017.6	72201.7	75488.8	142974.2	120649.3	137167.4	117074.4
Q80VJ8	Protein KASH5	Ccdc155	648	72.4	4.83	45792.9	59541.3	60723.6	63801.7	58011.1	46664.1	38742.5	49502.6	62534.7	51223.5	36175.2	43378.0	42275.9	48748.1
Q9Z1X9	Cell division control protein 45 homolog	Cdc45	566	65.3	5.43	33367.2	39899.9	32440.8	35932.7	40044.8	38575.3	32827.7	35996.1	37870.0	36456.7	31366.6	36947.9	33284.1	33435.4
P63300	Selenoprotein W	Selenow	88	9.7	8.72	261493.0	329796.4	371195.0	433953.4	240730.2	215333.8	231015.7	228030.3	321728.5	345829.4	224012.3	309773.8	311643.1	383003.1
Q7TT28	RNA exonuclease 1 homolog	Rexo1	1213	130.7	8.97	269683.2	273218.1	273883.8	278767.1	297829.7	310428.2	270708.5	274926.2	275095.0	268420.0	209245.4	182966.2	198394.8	189192.2
Q3TC46	Protein PAT1 homolog 1	Patl1	770	86.7	6.37	34562.1	17303.0	11476.5	14509.1	18832.4	22455.6	18597.0	13481.6	12786.9	15732.9	#N/A	#N/A	#N/A	#N/A
Q4FZF3	Probable ATP-dependent RNA helicase DDX49	Ddx49	480	54.1	8.78	132258.0	135608.2	109254.8	123961.3	137239.6	135396.5	114666.1	115599.8	116596.5	127990.3	55293.7	56534.0	49462.3	56617.7
Q8C5N5	Programmed cell death protein 2-like	Pdcd2l	364	39.9	4.68	180872.4	179732.4	178071.0	199331.2	173323.1	185335.0	165751.8	166712.6	189401.5	186163.4	104191.7	145414.9	109510.0	140579.6
Q6ZQE4	Nuclear envelope integral membrane protein 1	Nemp1	437	49.8	7.01	97502.1	97662.6	96368.0	93076.1	106339.6	123110.4	113582.0	88819.2	101273.1	100825.5	125945.1	146972.6	138538.7	145436.7
Q78T54	Vacuolar ATPase assembly integral membrane protein Vma21	Vma21	101	11.4	7.24	129843.0	130838.1	127040.0	136455.5	137428.3	127220.7	113855.0	126133.2	136704.5	120896.9	61491.7	59930.4	61918.7	59290.0
Q61635	IRG-type G domain-containing protein	Ifi47	420	46.8	6.74	34766.0	35582.8	35307.5	31738.1	36702.4	34796.0	41146.3	32416.1	35537.2	31219.6	50395.9	45206.8	47782.6	42449.6
Q9D1K7	UPF0687 protein C20orf27 homolog	MGI:191457	174	19.5	6.68	65016.4	85966.3	97577.3	117225.2	63654.1	59747.6	59684.9	75772.7	78863.7	77861.3	91083.0	106618.1	114853.5	125181.0
Q3U1G5	Interferon-stimulated 20 kDa exonuclease-like 2	Isg20l2	368	41	10.01	128349.7	144864.8	128877.5	130690.2	145463.9	141547.8	126765.2	129917.5	130045.2	132306.3	165798.8	159572.3	150085.3	148865.2
Q8C142	Low density lipoprotein receptor adapter protein 1	Ldlrap1	308	34	6.58	113267.9	140614.7	101343.4	125549.7	141030.2	135708.5	112733.4	129360.9	116998.9	123667.1	#N/A	#N/A	#N/A	#N/A
Q60760	Growth factor receptor-bound protein 10	Grb10	621	70.5	9.07	99697.6	108645.7	100516.8	107806.8	113015.2	110512.4	105604.3	109893.1	98295.4	100931.2	125706.2	118299.0	121959.4	134887.6
Q61107	Guanylate-binding protein 4	Gbp4	620	70.8	6.64	124617.0	122270.8	120597.0	117899.5	118783.1	116231.6	124021.8	111964.4	120687.7	133024.2	144353.3	154419.6	137233.2	144011.7
Q9WTL4	Insulin receptor-related protein	Insrr	1300	144.8	6.64	38170.9	47257.7	37598.9	39759.9	94971.2	45109.0	40212.1	44498.6	47562.3	44338.2	34615.1	46514.5	46339.5	39777.7
Q8C7V8	Coiled-coil domain-containing protein 134	Ccdc134	229	26.5	8.53	136383.3	169493.6	141021.4	152981.8	155801.7	140392.2	134702.3	130789.6	138071.6	148238.0	154073.8	186034.7	148349.0	170529.1
P06802	Ectonucleotide pyrophosphatase/phosphodiesterase family member 1	Enpp1	906	103.1	6.6	62389.7	73950.3	58178.2	76026.3	66495.3	73495.0	64590.3	72175.8	63145.9	66644.8	96374.0	109805.4	107276.3	107355.6
O54983	Ketimine reductase mu-crystallin	Crym	313	33.5	5.67	117749.7	7958.2	79227.4	9208.5	13341.8	23495.7	40951.3	8304.6	74575.2	11119.0	124794.5	84845.0	128412.7	113486.9
A2A8U2	Transmembrane protein 201	Tmem201	664	72.5	9.2	181607.9	187121.3	171988.1	164773.6	191441.2	191569.0	197232.8	161610.8	177959.3	182715.5	245618.3	254254.9	214270.4	242849.0
P12025	Midkine	Mdk	140	15.4	9.7	25082.1	32501.7	45813.0	35466.5	33253.7	26236.9	25073.4	34481.2	36415.1	38451.4	174994.2	210530.9	244948.6	196531.9
A2A559	Post-GPI attachment to proteins factor 3	Pgap3	320	36.5	7.94	109965.4	120766.2	116774.2	122199.2	132251.4	115452.4	115668.9	115609.4	120845.1	127652.5	233484.2	268349.5	228904.7	248048.1
P26450	Phosphatidylinositol 3-kinase regulatory subunit alpha	Pik3r1	724	83.5	6.28	73949.9	72839.8	78094.9	74812.5	85618.7	82138.6	75171.9	79667.9	80598.8	76655.0	51134.0	60970.7	61835.0	47136.1
Q8BGV8	Mitochondrial dynamics protein MID51	Mief1	463	51.2	7.88	49186.6	56761.2	63562.1	55050.1	56566.6	64982.1	64845.6	59008.7	61801.3	57122.6	72861.7	77445.4	64531.3	61359.1
Q6PDX6	E3 ubiquitin-protein ligase Rnf220	Rnf220	566	62.7	6.04	120599.8	123924.9	124486.8	122968.1	129087.0	129224.2	124999.0	114748.2	133713.3	125505.9	102789.6	103991.3	94160.9	99560.7
A2ARA8	Integrin alpha-8	Itga8	1062	117.5	6.16	75094.7	75930.8	69690.3	74526.0	75539.0	79675.3	81700.7	67655.7	71014.0	70115.5	191841.1	198928.4	176569.5	196724.1
Q8JZY2	COMM domain-containing protein 10	Commd10	202	22.8	6.65	182457.0	172137.3	182613.5	180458.5	153027.1	169336.7	167200.7	157754.4	167404.8	148962.5	124268.0	127785.2	121934.1	129990.8
Q9D1C9	Ribosomal RNA-processing protein 7 homolog A	Rrp7a	280	32.4	9.48	155730.0	158463.7	166142.5	147698.7	177653.3	161521.1	160994.9	166512.3	167744.9	154398.5	100171.0	98774.2	109368.7	102954.2
Q3TAP4	AP-5 complex subunit beta-1	Ap5b1	876	94.6	6.07	54714.7	72365.7	51439.2	60311.0	54054.9	56409.0	44788.8	57618.8	58610.6	59992.7	#N/A	#N/A	#N/A	#N/A
A2AJI0	MAP7 domain-containing protein 1	Map7d1	846	93.2	10.15	94175.6	100077.8	94495.6	92196.1	95216.4	99746.9	104895.7	94522.6	92957.1	91001.0	98134.2	95898.4	94002.6	91785.0
Q80T85	DDB1- and CUL4-associated factor 5	Dcaf5	946	103.6	5.8	281587.5	310445.8	263207.0	304450.7	294346.4	295030.8	283941.6	286803.7	294930.3	298500.9	#N/A	#N/A	#N/A	#N/A
Q6PB51	Coiled-coil domain-containing protein 117	Ccdc117	277	30.4	5.88	156578.3	206641.1	179758.5	205045.7	181879.4	149747.5	141629.6	151278.2	198872.7	170417.2	78186.6	84758.2	85469.1	79746.5
Q9D8X2	Coiled-coil domain-containing protein 124	Ccdc124	217	25.3	9.64	182618.2	200770.5	214690.6	190216.1	190996.2	180932.0	142307.2	174900.1	176152.1	183849.5	144977.6	162753.2	163033.5	161923.6
Q60771	Claudin-11	Cldn11	207	22.1	7.91	364465.3	288731.0	396964.1	258984.7	291741.0	335526.4	412247.1	250205.7	338821.3	350662.7	1153788.3	1071421.2	987953.7	1022533.8
Q8BI71	Exocyst complex component 3-like protein	Exoc3l1	739	81.2	5.95	53256.4	67699.8	55878.2	65790.5	66541.7	66222.8	66503.3	55561.9	71799.5	60571.7	49854.0	55328.4	48856.1	52243.0
Q8BTI9	Phosphatidylinositol 4,5-bisphosphate 3-kinase catalytic subunit beta isoform	Pik3cb	1064	121.6	7.09	36882.1	34633.2	36954.1	37277.7	38970.2	40062.5	35927.8	35159.5	35131.2	38966.4	17638.7	18865.2	16399.9	16238.4
Q8K4K1	Centrin-4	Cetn4	168	19.2	4.64	144999.5	175792.9	167185.4	227553.7	140466.4	130916.9	130225.9	132041.0	154367.2	162274.3	26776.4	41348.2	49605.9	68978.8
Q9JK91	DNA mismatch repair protein Mlh1	Mlh1	760	84.6	5.62	52842.7	43501.4	48098.1	44628.7	49067.6	56209.2	49938.3	50280.5	49463.5	51804.1	12932.3	16728.4	19788.6	16836.4
Q9WU03	Kunitz-type protease inhibitor 2	Spint2	252	27.9	8.1	57508.3	62618.8	57226.9	64352.0	65347.9	63017.7	56166.0	53967.9	61426.1	61245.1	#N/A	#N/A	#N/A	#N/A
Q8BGC1	UPF0489 protein C5orf22 homolog	MGI:192512	442	49.4	4.81	126572.7	141373.5	129648.1	134398.0	129692.6	139748.0	121432.9	132637.9	138848.2	143896.3	77058.3	74050.4	76319.7	71974.5
Q8K2Y7	39S ribosomal protein L47, mitochondrial	Mrpl47	252	29.7	10.21	151326.1	159918.6	168311.7	180200.3	175738.4	150485.0	151298.3	171268.1	163247.4	172346.7	84539.8	89799.3	84755.7	90537.6
Q02357	Ankyrin-1	Ank1	1862	204.1	6.55	125442.6	134194.5	120934.3	139176.9	120496.5	130316.5	115684.4	118938.1	136695.6	149449.4	#N/A	#N/A	#N/A	#N/A
Q78XF5	Oligosaccharyltransferase complex subunit OSTC	Ostc	149	16.8	9.13	455127.2	456391.1	428937.9	477268.5	379583.2	395496.7	371929.6	470910.6	398898.2	376377.5	443810.6	456004.6	421356.9	525638.7
Q99N93	39S ribosomal protein L16, mitochondrial	Mrpl16	251	28.8	10.27	152273.9	170997.9	160884.2	177409.2	172605.8	160440.9	157182.6	133930.0	166842.3	166811.3	287225.6	280815.7	286380.7	296415.9
P47964	60S ribosomal protein L36	Rpl36	105	12.2	11.34	283949.7	313260.4	345779.2	314511.4	317486.0	276847.0	253847.7	330224.4	287833.3	284538.7	472191.8	481061.9	499033.9	513198.6
Q8BPZ8	BRCA1-A complex subunit Abraxas 1	Abraxas1	407	46	6.98	56562.6	59236.4	54180.2	60107.4	62144.7	53179.9	58880.4	51684.9	55923.9	64951.6	316624.2	364483.2	358439.6	352439.2
Q8R0F8	Acylpyruvase FAHD1, mitochondrial	Fahd1	227	25.2	7.69	87909.5	96662.5	92193.3	81205.8	91047.2	87591.5	88353.1	103433.9	97039.1	96887.5	99321.1	108410.9	99489.5	92927.5
Q9QXG2	Rab proteins geranylgeranyltransferase component A 1	Chm	665	73.9	4.68	44074.6	42721.9	36958.1	41706.3	40061.6	34194.3	28709.1	40094.8	38344.1	40106.5	121471.4	108431.7	138806.5	113354.6
O09000	Nuclear receptor coactivator 3	Ncoa3	1398	151.5	7.77	166762.5	165101.2	149497.6	172886.7	178139.1	159748.2	139323.3	146167.0	166420.7	169215.5	56240.0	48585.2	48779.1	47794.5

Q9WUZ7	SH3 domain-binding glutamic acid-rich protein	Sh3bgr	214	23.1	4.07	52882.6	53321.9	50432.4	45696.6	55337.1	56559.6	54433.1	58898.6	50107.8	48507.1	23741.2	25970.2	22909.5	21449.4
Q3TMV7	Pyridine nucleotide-disulfide oxidoreductase domain-containing protein 1	Pyroxd1	498	55.6	5.33	74626.0	76325.1	68598.9	70059.2	70488.9	72303.5	75501.3	72054.9	75356.7	74591.6	64846.7	71914.7	64349.7	63509.0
O54890	Integrin beta-3	Itgb3	787	86.7	5.24	50111.7	45604.9	51289.7	48010.0	53723.2	58788.0	59782.6	53023.0	56292.2	52207.3	167820.9	135219.6	128281.5	133419.9
Q6PG95	Protein cramped-like	Cramp1	1285	137	8.22	138419.4	140263.2	135210.1	127438.4	137719.8	138620.2	127144.6	132354.1	132559.0	131927.6	#N/A	#N/A	#N/A	#N/A
Q9JLV1	BAG family molecular chaperone regulator 3	Bag3	577	61.8	7.27	10885.3	11637.2	8892.0	13113.1	11105.7	11213.2	12193.8	5654.6	10631.5	10890.9	#N/A	#N/A	#N/A	#N/A
P70665	Sialate O-acetylesterase	Siae	541	60.7	6.79	20779.7	28289.8	20091.9	23630.7	22467.9	25290.2	21507.4	21665.1	23061.1	24599.5	35652.0	36835.6	40811.7	33025.4
Q99JR5	Tubulointerstitial nephritis antigen-like	Tinagl1	466	52.6	6.77	87014.2	89688.0	97092.1	97629.1	100637.6	99648.8	109622.5	100387.0	99429.6	93748.1	#N/A	#N/A	#N/A	#N/A
Q8R361	Rab11 family-interacting protein 5	Rab11fip5	645	69.5	9.07	85740.1	92474.7	67515.1	82429.2	79257.2	83154.6	82643.5	168735.3	85132.8	94182.7	53823.3	61096.1	59399.7	55994.5
Q6ZWZ2	Ubiquitin-conjugating enzyme E2 R2	Ube2r2	238	27.1	4.42	163646.0	160520.1	157006.3	169705.4	162731.9	173892.5	158537.6	155844.0	151526.8	174524.7	138143.1	132101.7	122856.1	116033.1
Q9CZG9	PDZ domain-containing protein 11	Pdzd11	140	16.2	7.14	32060.8	32387.5	25209.9	24472.9	27613.0	30662.6	29244.2	26677.5	33019.5	27258.4	130893.2	127652.5	106829.1	122280.5
Q9QZR9	Collagen alpha-4(IV) chain	Col4a4	1682	164	8.54	47176.5	48912.6	48151.7	43407.9	48017.2	51364.4	51688.2	50746.0	48496.8	49922.1	88800.3	86619.4	82237.5	83663.6
Q9D142	Uridine diphosphate glucose pyrophosphatase NUDT14	Nudt14	222	24.4	5.14	132412.8	129190.6	116324.4	119117.4	117537.9	124088.9	141810.1	116331.4	119166.0	129344.9	213082.1	231954.6	231006.2	219104.0
Q8BL86	Metallo-beta-lactamase domain-containing protein 2	Mblac2	279	31.2	6.86	82671.6	85406.6	87894.9	88135.2	80030.6	82504.5	77955.2	76500.8	79872.6	92093.5	#N/A	#N/A	#N/A	#N/A
Q8K1K4	Centromere protein I	Cenpi	746	85.3	8.05	176264.8	220044.9	181336.3	202756.3	199517.0	194334.6	178197.8	174317.3	200340.6	206809.4	94741.3	107261.6	86291.5	95703.4
O35280	Serine/threonine-protein kinase Chk1	Chek1	476	54.3	8.18	287677.8	291802.4	234550.7	264311.2	328403.7	265997.5	261219.9	247245.4	267591.2	292677.0	205832.9	210354.2	206505.6	200612.6
Q99J08	SEC14-like protein 2	Sec14l2	403	46.3	7.11	106224.8	101932.4	112569.8	100468.6	130109.9	123109.7	121883.2	107882.4	111566.8	98619.0	29341.3	29717.8	29545.0	28423.9
Q8R0A5	Transcription elongation factor A protein-like 3	Tceal3	200	22.5	5.44	415563.9	276074.4	379221.4	280133.8	370681.4	433098.9	651158.6	506316.1	372845.5	396777.3	121145.7	123062.7	113973.1	114869.2
Q80UU2	Ribonuclease P protein subunit p38	Rpp38	280	31.1	9.73	272170.5	286763.4	263324.5	242178.5	280592.6	287000.4	255858.9	277146.0	271389.3	269226.8	104113.4	105213.0	117816.3	105264.8
Q9DA08	SAGA-associated factor 29	Sgf29	293	33.3	8.1	134005.3	133130.8	128219.7	121610.5	135610.7	140533.4	135774.2	120617.7	130594.3	128191.0	107306.5	86416.0	103430.4	104779.4
O08586	Phosphatidylinositol 3,4,5-trisphosphate 3-phosphatase and dual-specificity protein phosphatase PTEN	Pten	403	47.1	6.37	66828.1	64466.2	69606.8	63770.1	63014.4	71852.2	60072.4	65235.9	59622.0	64925.3	74383.0	72397.9	71600.5	75189.4
Q3U0X6	Zinc finger protein 217	Zfp217	1041	113.5	8.15	89429.4	131644.3	86775.0	104427.2	128383.4	127247.2	123550.2	101383.1	95490.2	101451.0	147123.9	192795.6	155714.9	181036.9
Q80WT5	Aftiphilin	Aftph	931	101.1	4.5	207204.5	215720.2	230223.6	229884.4	208807.6	200265.9	192211.0	202536.3	221370.2	229953.8	39574.1	50507.8	50361.0	48252.2
P20444	Protein kinase C alpha type	Prkca	672	76.8	7.14	67591.8	68346.9	57529.5	71376.7	60235.2	66610.2	71392.4	73609.7	70736.9	57401.2	357871.0	375887.8	303803.6	331952.6
Q6DYE8	Ectonucleotide pyrophosphatase/phosphodiesterase family member 3	Enpp3	874	98.6	6.52	19521.2	21680.8	18175.5	17896.6	21251.5	24406.2	24570.0	24583.0	19720.1	19168.7	17446.6	17064.0	12565.9	16118.6
P58468	Protein FAM207A	Fam207a	219	24.8	11	45596.1	49450.5	50836.3	47405.4	55157.3	52430.6	43662.6	46642.7	48368.9	45911.1	46939.1	53124.6	44934.2	49704.1
Q5NCC3	E3 ubiquitin-protein ligase TRIM41	Trim41	630	71.8	5.05	240511.8	274959.1	232714.2	242859.7	240652.1	242569.1	218514.8	210977.0	243972.5	266406.7	227866.0	254401.9	242522.4	221921.1
E9Q8B5	Predicted gene 4788	Gm4788	879	99.4	7.44	187451.5	196701.4	179274.2	183052.7	192780.7	188104.0	191938.7	173613.0	188420.2	186911.0	190553.0	208887.5	171856.4	193942.1
Q6PIJ4	Nuclear factor related to kappa-B-binding protein	Nfrkb	1296	138.7	9.22	114192.2	112564.8	106821.1	120164.8	108935.7	108661.7	99104.8	99521.6	103681.7	121069.4	106543.4	102800.2	91155.1	106183.6
Q7SIG6	Arf-GAP with SH3 domain, ANK repeat and PH domain-containing protein 2	Asap2	958	106.7	6.65	291601.6	300717.8	293911.2	319219.1	369634.1	369549.2	300958.1	301101.3	320546.2	326103.2	600944.4	655564.7	611742.3	604588.9
Q9JLM9	Growth factor receptor-bound protein 14	Grb14	538	60.5	8.44	270447.4	256393.4	233093.4	258485.7	240435.3	301701.2	251584.4	263261.2	247112.6	226895.4	280549.0	320175.5	235625.5	267637.2
Q3UI43	BRISC and BRCA1-A complex member 1	Babam1	333	36.8	4.55	77754.0	123330.4	66391.8	92124.3	92714.2	93037.6	77874.0	81770.9	85594.7	99957.3	219527.6	199975.1	239342.1	210264.0
Q99L20	Glutathione S-transferase theta-3	Gstt3	241	27.4	7.74	168881.3	214040.9	159984.5	199189.1	167293.6	181703.0	158840.2	160689.4	195754.8	188831.1	#N/A	#N/A	#N/A	#N/A
Q9Z1E3	NF-kappa-B inhibitor alpha	Nfkbia	314	35	4.78	35224.0	29227.7	34497.0	28834.4	28282.8	33739.7	36016.9	31370.6	30163.4	29450.3	#N/A	#N/A	#N/A	#N/A
Q9R269	Periplakin	Ppl	1755	203.9	5.54	35504.7	33970.6	35665.8	33708.3	41686.2	41412.2	54087.8	40687.9	32161.8	34838.3	363056.8	340957.4	311055.9	350837.2
Q99N17	Cytochrome P450 CYP4F16	Cyp4f16	524	60.2	7.12	7806.3	6839.3	8445.0	6211.4	8287.4	10961.6	11873.8	6934.7	8700.9	8457.2	36427.1	38286.0	33978.3	30286.2
Q923B0	Gamma-glutamylaminecyclotransferase	Ggact	149	17.1	5.29	1986.8	5145.9	6426.0	7460.2	4489.6	5461.3	4678.9	953.0	1914.0	5287.7	#N/A	#N/A	#N/A	#N/A
Q5SQP1	HORMA domain-containing protein 2	Hormad2	306	34.8	7.49	180013.2	240856.6	176964.1	194922.1	207762.0	220429.2	167071.3	169186.2	201914.1	223312.4	453623.4	524071.3	463238.6	480391.1
O55102	Biogenesis of lysosome-related organelles complex 1 subunit 1	Bloc1s1	125	14.3	8.15	64605.3	74058.4	70509.2	69155.6	76592.1	68882.3	61293.3	70766.7	65952.6	74655.1	81239.4	92382.9	96469.1	81997.2
Q8BJM7	S-adenosyl-L-methionine-dependent tRNA 4-demethylwyosine synthase TYW1	Tyw1	721	81.5	7.83	174271.8	163370.9	160313.7	171268.3	141139.9	136639.3	138747.2	150769.1	171745.5	158634.1	119409.2	133096.7	89959.3	172940.4
Q9CR76	Transmembrane protein 186	Tmem186	216	24.6	10.11	178976.0	184201.2	155409.1	181699.5	160073.5	152268.4	153762.1	149196.7	177640.6	167710.3	134722.6	110130.1	98030.3	134405.0
Q3TQI7	Telomere length and silencing protein 1 homolog	MGI:238513	289	33.5	6.34	156252.9	162171.9	180882.9	164902.7	165744.8	162119.3	149452.7	147942.2	159790.6	174018.4	160229.6	176331.5	178829.5	174782.8
Q3UCQ1	Forkhead box protein K2	Foxk2	651	68.4	9.51	141047.9	171711.5	154967.5	185765.4	148015.6	152933.7	129642.3	153371.0	163314.3	164878.7	#N/A	#N/A	#N/A	#N/A
P82343	N-acylglucosamine 2-epimerase	Renbp	430	49.7	6.06	271490.0	270869.5	256199.2	259493.9	270552.4	292603.4	287378.5	256586.9	257062.6	293663.7	#N/A	#N/A	#N/A	#N/A
O88895	Histone deacetylase 3	Hdac3	424	48.3	5.26	382245.6	285911.5	319470.1	342189.6	311126.7	267992.5	289945.8	270941.0	363997.5	286414.2	181932.6	159211.8	176509.9	169079.0
O35943	Frataxin, mitochondrial	Fxn	207	22.9	8.02	79292.9	80146.3	95284.9	85368.5	93442.5	98582.8	89505.4	87748.0	88972.2	97054.8	41531.4	39501.7	38078.5	40420.6
Q7TMI3	E3 ubiquitin-protein ligase UHRF2	Uhrf2	803	90	8.03	59374.8	52744.3	54619.2	52041.4	51465.2	62175.0	50409.9	45039.9	51399.3	51393.6	#N/A	#N/A	#N/A	#N/A
Q921W0	Charged multivesicular body protein 1a	Chmp1a	196	21.6	8.06	452787.7	459984.6	421539.3	446118.4	429708.3	381063.2	444158.3	398594.6	452107.1	468594.3	168244.0	171807.1	180566.5	186640.7
P33622	Apolipoprotein C-III	Apoc3	99	11	4.75	87099.2	92276.6	85392.9	87392.0	82887.1	87172.4	81181.7	84731.9	96446.5	93565.7	208901.1	263944.0	205235.2	288330.1
Q6PDC0	RUN domain-containing protein 3B	Rundc3b	408	45.1	5.17	79778.6	85263.1	90178.1	79644.6	98818.0	83453.5	90978.7	84349.1	92348.6	92858.2	53568.7	68083.0	58862.2	58222.5
Q8CCH2	NHL repeat-containing protein 3	Nhlrc3	347	38.2	6.23	38152.0	42050.1	45347.0	38632.4	48083.7	40049.8	47398.7	41651.6	45173.4	40348.6	30575.8	26479.3	22697.8	29600.4
Q9EPQ8	Transcription factor 20	Tcf20	1987	215.6	8.98	128575.5	121479.8	133416.8	135524.5	145949.5	121945.3	127884.8	132934.4	131251.9	140126.1	#N/A	#N/A	#N/A	#N/A
Q91Z92	Beta-1,3-galactosyltransferase 6	B3galt6	325	37	8.82	82801.2	87426.8	88519.5	78909.9	91934.0	77770.5	74755.3	99481.8	72745.6	76787.7	100261.3	89758.7	86401.6	95227.0
P97478	5-demethoxyubiquinone hydroxylase, mitochondrial	Coq7	217	24	7.17	98417.5	100640.9	97766.9	109066.0	119624.0	110209.3	88705.7	108782.4	94791.1	99455.4	56658.5	48454.3	57631.1	62792.5
Q8BZ05	Arf-GAP with Rho-GAP domain, ANK repeat and PH domain-containing protein 2	Arap2	1703	193.3	7.18	155116.9	159427.4	144420.2	151312.0	162352.9	157552.0	146481.7	150239.8	147626.8	150470.3	#N/A	#N/A	#N/A	#N/A
Q8VBZ0	Dehydrogenase/reductase SDR family member on chromosome X homolog	Dhrsx	335	35.9	8.09	45232.0	61921.3	45770.1	55720.1	50428.4	56148.5	46834.4	42861.6	38377.4	49326.1	36567.1	55670.6	38033.7	47607.8
Q2HJ10	Zinc transporter 2	Slc30a2	371	40.6	7.18	229224.8	195194.4	173008.3	185998.5	182001.6	172534.9	194451.3	205135.5	191636.0	206546.1	815641.6	751654.8	791330.3	768829.8
Q7TT23	Uncharacterized protein C20orf194 homolog	MGI:192302	1179	132.1	6.43	100327.5	108813.3	103727.5	123636.0	103508.8	96968.0	95131.7	96698.5	107095.9	118812.9	69211.1	81369.7	75287.3	101900.6
Q9Z2B2	Brain mitochondrial carrier protein 1	Slc25a14	325	36.3	9.69	110738.8	114363.3	117818.0	99232.1	127532.8	112254.6	122695.3	129121.7	113050.6	117504.2	69976.6	69170.9	69535.6	65045.3
Q6A0D4	Raftlin	Rftn1	554	61.5	7.68	128655.1	132958.5	137950.9	121830.2	129396.2	152666.5	133944.1	121893.8	129207.9	125445.4	90061.8	85760.6	87612.5	74249.9
Q921I6	SH3 domain-binding protein 4	Sh3bp4	962	107.5	7.34	45222.1	53677.2	40477.1	48741.1	49800.2	47201.5	46063.4	44738.9	42982.4	46190.2	59221.4	60667.2	52906.5	59690.3
Q9D853	EEF1A lysine methyltransferase 2	Eef1akmt2	244	26.8	5.15	181889.4	242101.0	196776.0	222587.7	229992.9	228120.3	277122.8	172283.4	253117.5	275808.1	186659.1	174818.4	166840.3	164260.8
Q8K3J5	Zinc finger protein 131	Znf131	619	71	5.26	107053.9	108671.8	95851.1	102654.4	109331.6	111420.6	97638.7	96002.8	92762.3	100593.9	29625.7	33002.6	29876.1	32024.9
Q8VDI9	Alpha-1,2-mannosyltransferase ALG9	Alg9	611	69.5	8.94	247493.4	242136.7	208546.2	240267.2	207900.2	220836.8	211254.9	197314.0	214662.5	216666.1	107013.5	103671.5	104465.4	102915.0
Q9D820	Prolyl-tRNA synthetase associated domain-containing protein 1	Prorsd1	169	18.9	6.14	222952.4	226443.2	213586.0	205582.3	221314.2	219292.5	225046.5	220510.1	227922.4	226881.9	204132.5	224707.1	211929.7	215396.3
Q6ZPQ6	Membrane-associated phosphatidylinositol transfer protein 2	Pitpnm2	1335	147.9	7.93	74429.5	75050.8	79710.2	74469.1	80901.5	83980.5	84865.4	87438.5	85474.3	81407.6	#N/A	#N/A	#N/A	#N/A
Q8C080	Sorting nexin-16	Snx16	344	38.8	5.21	547836.6	549220.9	522995.7	505164.3	569009.1	556276.7	517419.3	510424.8	530340.0	555545.5	545944.3	592527.8	603906.0	581677.4
P16110	Galectin-3	Lgals3	264	27.5	8.38	174886.1	172811.6	173360.1	191942.5	191343.6	236343.8	358554.7	212000.3	187468.4	178954.5	213034.2	255282.3	229452.8	255407.9
Q06335	Amyloid-like protein 2	Aplp2	707	80.4	4.7	49620.8	60882.8	61165.2	67638.5	55046.0	56638.7	51354.5	54071.7	54468.5	64568.4	459637.1	447045.1	513836.3	536342.1
Q8BIG7	Catechol O-methyltransferase domain-containing protein 1	Comtd1	262	28.9	8.32	11591.2	12424.1	11207.1	11993.4	11448.9	14428.1	13539.1	12649.6	11072.9	11508.3	13831.5	14574.7	11233.5	12363.4
O35640	Annexin A8	Anxa8	327	36.7	5.94	39084.2	50215.1	58551.1	45323.2	47341.2	52331.9	42474.0	63441.5	46498.6	56929.1	96599.5	103750.3	83780.2	92203.5
Q9CQL4	39S ribosomal protein L20, mitochondrial	Mrpl20	149	17.6	11.11	124332.3	139996.2	125353.9	125573.2	119527.0	112751.0	117294.5	120019.5	135476.8	133315.6	113626.9	115508.7	109822.6	106527.0
Q9WUX5	Protein MRVI1	Mrvi1	899	97.4	5.62	81824.6	83539.7	88819.7	77274.7	86225.4	85115.3	95892.3	87513.6	92342.3	76381.4	#N/A	#N/A	#N/A	#N/A
Q99LB7	Sarcosine dehydrogenase, mitochondrial	Sardh	919	101.6	6.74	97831.5	96703.8	87839.1	99136.1	91739.3	93993.8	98523.0	96699.4	85382.5	97145.0	165055.7	146949.6	149217.6	147021.9
P28828	Receptor-type tyrosine-protein phosphatase mu	Ptprm	1452	163.5	6.68	199955.7	175096.5	164309.1	194016.9	169317.8	161556.1	174137.2	157769.9	201139.3	172743.4	237856.5	250341.9	228696.9	225278.7
P52482	Ubiquitin-conjugating enzyme E2 E1	Ube2e1	193	21.3	8.53	202815.0	253368.2	206720.4	211640.7	196776.0	202031.2	215101.3	212526.8	222946.3	218620.9	586991.7	574922.7	597539.0	546284.7
Q8BTF7	DNA ligase 4	Lig4	911	104.1	8.16	33314.5	38206.4	40867.4	36949.1	42244.8	37443.1	37339.2	39200.3	34697.8	37015.4	107146.9	110568.7	114869.9	111859.1
Q91YN0	Protein C12orf4 homolog	D6Wsu163	552	63.6	6.18	35576.3	38327.4	32699.5	38532.0	38473.3	35941.1	39931.4	35506.4	37292.7	36993.2	30888.7	30684.5	32805.9	27269.5
Q8C5L6	Inositol polyphosphate 5-phosphatase K	Inpp5k	468	54.1	5.88	18860.6	23192.3	21435.5	21573.0	21050.6	20088.5	17428.9	17588.3	23272.5	21167.7	221751.2	255742.9	234741.5	242524.4
P60605	Ubiquitin-conjugating enzyme E2 G2	Ube2g2	165	18.6	4.7	116462.2	150490.7	124441.3	135601.2	134882.7	132990.2	115199.4	125300.6	132142.9	129673.9	92702.1	110528.7	95264.2	107359.6
O08601	Microsomal triglyceride transfer protein large subunit	Mttp	894	99	7.62	77437.5	100787.8	81512.1	96217.2	86845.3	84937.5	81044.2	82018.8	82357.6	95530.4	#N/A	#N/A	#N/A	#N/A
Q4VAA7	Sorting nexin-33	Snx33	574	65.3	6.79	261113.2	218776.3	200272.2	235484.4	212840.3	188290.9	189866.5	210326.9	188061.5	211451.8	72229.9	70984.2	63613.7	54905.0
Q6IRU7	Centrosomal protein of 78 kDa	Cep78	790	86.8	8.54	13917.5	14054.8	11161.5	12193.0	7556.3	13207.5	9189.2	12519.0	13344.9	10224.7	17457.1	14575.2	14036.8	18350.5
Q91WB4	Pleckstrin homology domain-containing family F member 2	Plekhf2	249	27.7	8.21	87807.3	106048.0	82713.0	106519.5	86937.8	90600.2	85723.0	84842.9	105183.7	84325.3	104016.6	99594.5	107593.6	93536.1
Q8K0D0	Cyclin-dependent kinase 17	Cdk17	523	59.5	9.03	38712.1	37261.9	40312.2	37600.9	43340.8	39844.7	31724.3	40957.1	45303.9	43148.3	154344.3	161714.9	138956.4	151828.7
Q9D924	Iron-sulfur cluster assembly 1 homolog, mitochondrial	Isca1	129	14.2	9.07	91048.1	80037.0	82080.6	80316.5	93054.7	84114.6	71496.3	91145.5	79202.8	81780.2	#N/A	#N/A	#N/A	#N/A
Q91ZX6	Sentrin-specific protease 2	Senp2	588	67.5	9.61	27481.0	31501.2	33167.2	31611.2	27571.0	28855.4	22336.6	27596.7	28019.2	28228.2	#N/A	#N/A	#N/A	#N/A
Q9WUR9	Adenylate kinase 4, mitochondrial	Ak4	223	25	7.53	180845.4	191705.4	165695.3	163357.3	200311.4	209131.9	177060.5	149748.8	178565.9	195762.9	162649.5	205256.1	166169.8	168750.8
Q9R0A5	Serine/threonine-protein kinase Nek3	Nek3	511	57.4	6.87	240374.0	220173.2	199893.8	201192.9	200484.3	209007.9	200469.6	194712.1	214542.5	230773.4	839635.0	829748.5	768324.0	779141.7
Q8CGW4	Transcription factor SOX-30	Sox30	782	83.9	8.66	82382.2	113862.2	114407.6	125637.1	75453.5	66384.7	56005.8	74159.5	95901.8	99355.7	227581.7	359574.9	345063.2	379237.6
Q9ESY9	Gamma-interferon-inducible lysosomal thiol reductase	Ifi30	248	27.8	5.01	48838.8	47089.8	43698.5	47330.1	51504.9	42834.6	43449.4	51004.4	53901.8	39958.5	#N/A	#N/A	#N/A	#N/A
Q3UA16	Kinetochore protein Spc25	Spc25	226	26.4	6.95	95152.9	105560.9	115201.6	109660.6	113857.1	117193.3	90460.1	99216.8	105453.4	126221.2	69963.5	68342.2	75844.8	71284.6
Q9D2H2	Adenylate kinase 7	Ak7	614	70.6	4.96	77090.8	86560.7	89601.8	112283.4	72848.4	69783.5	68839.5	75667.7	67827.0	81946.7	#N/A	#N/A	#N/A	#N/A

Q62470	Integrin alpha-3	Itga3	1053	116.7	6.57	260390.5	284292.1	239436.5	245925.6	262895.9	277738.3	300818.8	240108.7	269350.3	263593.1	36109.9	34038.0	30411.1	30344.7
Q8BZ47	Zinc finger protein 609	Znf609	1413	151	8	151452.5	146535.2	148350.7	152747.3	150995.0	152667.9	138412.4	139006.2	150954.4	156361.6	165762.5	176999.1	168940.1	178606.0
Q9Z1E4	Glycogen [starch] synthase, muscle	Gys1	738	83.9	6.11	111551.5	120018.8	100934.1	107989.8	116394.2	116300.9	128685.0	124905.3	110459.0	110752.1	46848.7	71497.2	45624.4	54151.2
Q8K1H7	T-complex protein 11-like protein 2	Tcp11l2	517	57.9	5.08	63435.9	73131.4	58441.6	65856.0	63889.0	70650.8	55207.1	65493.1	62737.2	59338.3	108759.7	98147.9	95929.8	106425.0
A2AU37	Double-strand-break repair protein rad21-like protein 1	Rad21l1	552	62.6	5.05	74218.4	75185.3	69719.7	82418.7	74892.8	66616.0	68905.7	72272.2	75602.8	82196.5	81770.9	80788.5	80124.8	73817.8
Q9CWH5	tRNA (guanine(10)-N2)-methyltransferase homolog	Trmt11	460	52.9	8.46	215258.2	239456.4	203528.8	238630.1	235694.5	231308.3	211449.1	225010.0	215484.4	214173.8	#N/A	#N/A	#N/A	#N/A
Q9D180	Cilia- and flagella-associated protein 57	Cfap57	1249	144.8	5.74	88157.6	128625.1	122083.7	157196.5	83510.1	89018.8	90840.7	95318.2	95695.7	104689.7	#N/A	#N/A	#N/A	#N/A
Q9CQE0	E3 ubiquitin-protein ligase RNF138	Rnf138	245	28.3	7.31	87471.2	107652.4	89895.5	94427.2	102444.3	95501.1	94126.8	101479.9	97810.2	102799.2	26733.7	30052.1	26722.8	35609.9
P09803	Cadherin-1	Cdh1	884	98.2	4.83	13030.4	20350.7	17623.7	13611.8	14642.0	19925.2	23577.2	17066.5	23434.6	16077.3	24872.2	25287.5	18646.5	25582.6
O88933	Homeobox domain-containing protein	Esx1	382	43.5	4.92	31378.4	35956.0	32635.8	29663.5	37591.8	30352.1	28760.4	26840.4	36751.2	37109.4	22367.8	25982.4	21207.8	25215.8
P58929	Glucocorticoid modulatory element-binding protein 2	Gmeb2	530	56.6	5.31	358641.5	358603.2	331727.9	321658.3	423581.9	394009.5	373915.4	321384.8	359344.8	368098.2	399384.9	379180.3	537703.0	348317.9
Q9QYM5	Rho-related GTP-binding protein RhoN	Rnd2	227	25.4	8.35	56326.3	47311.9	44564.9	37822.2	52076.6	43731.6	42107.3	47961.4	48566.1	52665.8	211735.5	212561.3	218652.5	197220.9
Q9D7S7	60S ribosomal protein L22-like 1	Rpl22l1	122	14.5	9.45	327943.7	299263.0	342705.4	298630.2	267243.6	267527.5	281653.7	352552.3	291900.8	288977.6	699049.6	691912.1	651323.6	685417.1
A0A075B5U4	IGv domain-containing protein	Ighv1-18	102	11.3	8.68	300443.4	185811.0	252195.9	255875.5	240845.4	185729.7	270097.9	325514.1	264443.1	258668.6	#N/A	#N/A	#N/A	#N/A
Q99MU3	Double-stranded RNA-specific adenosine deaminase	Adar	1178	130.4	8.7	40336.0	41377.8	41261.1	31782.1	43922.4	39867.7	43843.9	37529.7	45841.2	44558.3	151192.5	141003.6	156131.7	127084.9
Q9D0A3	Arpin	Arpin	226	25.2	5.19	162529.0	161511.1	133908.3	153020.5	145451.3	176230.0	164433.1	161663.3	144118.8	146810.8	273548.5	307455.6	262379.2	299072.4
Q9D1G2	Phosphomevalonate kinase	Pmvk	192	21.9	5.99	197962.9	207440.3	170466.9	194926.0	166975.0	171624.5	180817.9	166589.8	191965.6	188973.1	163919.3	176582.8	152684.2	163478.8
P56392	Cytochrome c oxidase subunit 7A1, mitochondrial	Cox7a1	80	9	9.79	200435.9	184253.8	191757.4	170435.9	177377.4	207881.2	220640.2	218709.4	196094.8	189094.7	#N/A	#N/A	#N/A	#N/A
Q8R0K9	Transcription factor E2F4	E2f4	410	43.8	4.75	195457.4	195324.5	184848.8	190531.4	185934.5	195385.5	196305.6	166207.1	197817.6	201986.4	#N/A	#N/A	#N/A	#N/A
P09581	Macrophage colony-stimulating factor 1 receptor	Csf1r	977	109.1	6.21	139356.3	141701.0	116442.7	145551.7	106687.7	127538.3	122653.6	136337.3	113037.4	144432.1	217318.7	210318.4	174902.4	208241.6
Q6S5C2	N-acetylglucosamine-1-phosphotransferase subunit gamma	Gnptg	307	34.1	6.92	105188.0	119255.9	94819.1	106219.6	108679.1	111464.7	112925.4	106482.1	108150.3	116026.1	#N/A	#N/A	#N/A	#N/A
Q8CBG9	E3 ubiquitin-protein ligase RNF170	Rnf170	286	33.4	5.66	4519.2	9082.3	5722.9	6040.5	8565.6	6928.9	5280.6	6990.2	4574.4	6664.4	46736.5	50529.2	51183.1	43623.4
O70579	Peroxisomal membrane protein PMP34	Slc25a17	307	34.4	10.11	22702.0	34100.2	26069.6	30281.6	28357.5	27038.4	19044.6	26741.6	29710.6	29330.2	27331.1	27668.8	27822.2	35559.4
A2BFL2	Protein zyg-11 homolog A	Zyg11a	627	70.4	8.56	65085.5	75120.5	59198.2	68624.0	74882.6	72667.6	58629.0	63060.1	69367.0	70348.1	185650.1	213180.5	187847.8	191837.8
Q8C006	Tripartite motif-containing protein 35	Trim35	501	57.3	7.14	59110.7	55790.2	55246.3	62871.1	58549.7	62774.5	65813.8	58480.8	65188.1	62700.0	101210.1	120417.3	108752.6	106342.4
Q3UMF0	Cordon-bleu protein-like 1	Cobll1	1273	137.3	8.16	162022.1	157399.7	127582.6	167827.2	145031.4	141434.1	141158.8	152344.0	139816.9	151786.6	198728.7	201878.3	189418.8	208279.3
O88968	Transcobalamin-2	Tcn2	430	47.6	6.33	110106.6	120829.4	118709.2	102962.0	116883.2	127311.1	144699.1	126466.4	116649.6	112534.3	92673.5	113391.8	84054.5	94573.4
Q9CQ20	Mid1-interacting protein 1	Mid1ip1	182	20.3	5.5	19515.7	16648.5	20604.0	24417.3	22510.8	23192.7	19939.9	20850.1	19821.2	25362.3	#N/A	#N/A	#N/A	#N/A
Q99L00	HAUS augmin-like complex subunit 8	Haus8	373	41.6	6.24	141219.0	149922.7	164123.7	138253.5	157475.1	171011.5	135428.6	140448.2	151623.0	153900.2	37669.7	47915.7	46528.1	40384.5
Q61337	Bcl2-associated agonist of cell death	Bad	204	22.1	9.17	171815.1	202033.1	171489.5	190279.7	164816.5	165054.7	171127.5	171656.1	188823.9	180542.2	116968.6	125937.5	127336.0	124949.0
Q99LS1	Methylmalonic aciduria and homocystinuria type D homolog, mitochondrial	Mmadhc	296	33	5.07	80508.5	82591.0	79876.7	77932.5	79585.2	79868.3	76314.3	67244.9	77798.8	77022.1	#N/A	#N/A	#N/A	#N/A
Q9CXD6	Mitochondrial calcium uniporter regulator 1	Mcur1	340	37.8	10.2	97453.6	97876.1	102724.1	97168.5	120101.3	106826.1	124131.5	112741.6	119454.5	111303.5	260647.1	272782.3	241383.7	243975.9
Q80U63	Mitofusin-2	Mfn2	757	86.1	6.77	74495.8	79534.6	83432.1	81026.1	84402.7	84116.7	75445.0	81049.0	78423.4	84324.4	56881.8	69933.0	58530.9	56798.3
Q91XC8	Death-associated protein 1	Dap	102	11.1	9.36	39269.2	41262.4	38363.6	46378.6	48429.3	43798.4	45542.1	43945.3	39513.7	42270.0	#N/A	#N/A	#N/A	#N/A
Q8R550	SH3 domain-containing kinase-binding protein 1	Sh3kbp1	709	78.1	7.55	77381.7	89269.7	84281.7	81904.7	88238.9	93088.3	92025.1	84106.5	70873.5	77094.1	251064.5	231077.6	225128.1	225968.5
B1AY10	Transcriptional repressor NF-X1	Nfx1	1114	123.7	8.31	79874.7	77886.3	78547.9	75943.6	72376.1	78506.4	80702.5	66297.5	71310.6	75282.5	114472.1	111996.9	120555.0	109548.1
Q9CQV4	Reticulophagy regulator 3	Retreg3	466	51.6	4.97	37696.2	41359.4	43817.6	38403.0	37100.7	42035.0	31713.1	37937.5	39990.6	40830.2	38482.4	42404.8	38871.5	39342.6
Q9DCU9	4-hydroxy-2-oxoglutarate aldolase, mitochondrial	Hoga1	321	34.6	7.72	309483.3	354354.3	253948.4	323806.8	316523.4	356643.6	326502.1	292250.1	314271.8	330140.3	50389.5	39893.0	37976.4	42350.7
O08600	Endonuclease G, mitochondrial	Endog	294	32.2	9.54	166033.1	184247.8	155638.3	170156.3	180680.0	175918.7	170508.0	158150.2	149282.5	186663.1	#N/A	#N/A	#N/A	#N/A
B2RQG2	PHD finger protein 3	Phf3	2025	225.4	6.7	36841.3	40598.8	35853.0	39147.3	35949.3	40461.1	32482.7	35472.9	28425.0	37871.0	98057.2	107821.1	102028.1	102067.6
Q8R2S9	Actin-related protein 8	Actr8	624	70.5	7.77	73265.9	73544.0	52839.8	72478.8	57464.5	55700.9	52486.6	69008.2	55924.8	62781.3	188239.9	195092.1	203382.4	208641.3
Q9D864	Actin-related protein 6	Actr6	396	45.8	5.03	56958.8	66608.7	57775.3	58241.9	58041.4	57221.7	49892.5	49635.6	57002.7	70026.3	#N/A	#N/A	#N/A	#N/A
Q6PCQ0	IQ domain-containing protein E	Iqce	778	86.3	7.18	16868.7	16904.0	13653.7	15825.9	19037.0	10785.5	13754.0	15616.7	10838.0	14225.4	70324.1	84792.4	67969.5	85308.1
Q5SSN7	Vesicle transport protein SFT2A	Sft2d1	159	17.9	8.92	21517.4	21503.0	20190.9	23261.7	17420.6	20085.6	21051.1	17022.2	20170.0	20683.5	27505.3	38312.8	30271.0	29254.3
P01631	Ig kappa chain V-II region 26-10	-	113	12.3	8.88	36251.3	34087.7	26967.6	23659.2	51946.0	35395.6	36484.0	39511.3	28353.2	26438.7	#N/A	#N/A	#N/A	#N/A
Q63943	Myocyte-specific enhancer factor 2D	Mef2d	514	55	7.88	114585.1	107401.4	113148.7	110201.5	121895.2	124114.6	113494.0	115487.2	102829.3	104496.8	#N/A	#N/A	#N/A	#N/A
Q3UE17	RNA-binding protein MEX3D	Mex3d	643	65.3	5.63	104000.8	103791.6	96645.6	90908.4	97475.3	93859.1	93738.4	96744.8	100543.6	103111.3	#N/A	#N/A	#N/A	#N/A
E9PW43	Protein transport protein Sec61 subunit beta	Gm10320	113	11.8	11.58	180059.1	235165.6	182795.1	227123.7	181399.8	183149.3	202050.6	189075.0	211232.3	214437.8	246023.5	330670.2	268396.9	322038.8
Q9DBZ1	Inhibitor of nuclear factor kappa-B kinase-interacting protein	Ikbip	373	42.5	5.1	56415.0	46869.3	49405.5	43617.1	46821.4	41100.7	34990.1	46154.5	43503.5	44920.6	194871.6	218025.2	200311.8	214473.2
Q8VHZ7	U3 small nucleolar ribonucleoprotein protein IMP4	Imp4	291	33.6	9.32	45454.9	45277.4	39674.7	47693.8	41613.6	47832.1	44977.3	37701.5	46006.3	44482.8	167839.8	186248.7	148650.5	166561.2
Q9Z266	SNARE-associated protein Snapin	Snapin	136	14.9	9.31	165692.9	179581.2	209211.1	189268.9	208975.7	165336.3	173371.1	186994.4	194380.8	179312.2	148469.3	185095.4	198208.0	186221.0
Q61069	Upstream stimulatory factor 1	Usf1	310	33.6	5.54	76021.2	68535.4	77520.9	73145.4	85139.2	89756.9	82481.9	78243.7	75502.6	72340.2	195695.8	208101.9	180063.9	198734.4
P62482	Voltage-gated potassium channel subunit beta-2	Kcnab2	367	41	9	235041.6	245933.5	202468.7	237198.0	238288.5	233548.9	224517.3	225823.6	235325.7	231595.1	190174.6	185741.0	172205.2	188427.1
Q8K4R9	Disks large-associated protein 5	Dlgap5	808	90.1	9.17	49341.7	64507.1	47621.1	54075.4	58552.7	48281.3	48418.5	51131.3	49539.2	61892.1	91861.0	92356.0	107753.7	120777.4
P34884	Macrophage migration inhibitory factor	Mif	115	12.5	7.34	111217.1	120883.8	105038.6	102112.2	126005.8	118647.3	118777.1	109060.8	108937.5	114922.0	32788.1	33316.6	28155.0	27705.6
Q8BHT7	RAB6A-GEF complex partner protein 2	Rgp1	391	42.5	5.94	30396.8	28630.6	25958.7	27204.7	29147.2	43414.9	24683.8	32268.0	30535.5	30931.8	39027.5	41728.6	40302.5	43178.4
O88700	Bloom syndrome protein homolog	Blm	1416	158.3	7.27	87686.4	102701.8	96742.1	98169.4	93444.0	100430.2	83325.8	91385.8	89829.3	101878.6	202630.2	260131.2	244269.9	227610.2
Q61140	Breast cancer anti-estrogen resistance protein 1	Bcar1	874	94.2	5.68	209711.3	193246.4	196639.5	182676.3	227988.0	235629.0	240696.6	202024.0	220054.4	203942.3	74701.4	93095.1	88953.3	90539.2
Q9DBQ9	Transcriptional protein SWT1	Swt1	907	103.1	8.22	188628.9	188166.2	161721.4	180384.1	169220.2	161251.0	147298.7	146330.8	173903.7	180979.7	128811.8	130803.0	105563.8	114052.1
Q8R349	Cell division cycle protein 16 homolog	Cdc16	620	71.4	5.76	440191.2	403186.5	423158.9	391032.4	475619.1	491555.5	437509.1	427365.9	428122.7	412361.3	158838.3	136731.7	149434.1	160718.2
Q3TGW2	Endonuclease/exonuclease/phosphatase family domain-containing protein 1	Eepd1	569	62.9	8.35	45665.6	52529.4	38311.3	47997.6	43520.9	42549.4	36793.5	45027.5	45948.9	42401.7	#N/A	#N/A	#N/A	#N/A
Q64726	Zinc-alpha-2-glycoprotein	Azgp1	307	35.3	6.18	90538.4	79306.8	86285.1	77360.7	86519.7	83877.0	87504.1	90347.0	77146.6	86845.3	132696.7	119257.0	108892.0	110234.0
Q7TNL9	Coiled-coil-helix-coiled-coil-helix domain-containing 10	Chchd10	138	13.8	8.22	36424.0	28955.8	31213.6	28061.6	36822.8	30234.0	42234.7	27671.2	30034.5	33724.4	223048.2	220543.4	202604.5	216700.7
P23708	Nuclear transcription factor Y subunit alpha	Nfya	346	36.8	8.94	608419.2	549445.8	597282.4	520742.9	589207.8	603168.5	719075.6	571064.9	564594.3	581923.4	157853.0	180977.6	185749.0	164260.7
Q8R086	Sulfite oxidase, mitochondrial	Suox	546	60.7	6.54	272421.1	283912.6	272926.1	331369.5	253924.1	275036.3	263304.8	315656.8	266824.1	266335.0	104236.8	91914.3	88488.7	90932.0
Q9R194	Cryptochrome-2	Cry2	592	66.8	8.35	14510.6	19467.2	19406.1	18287.9	17474.7	19596.1	17091.2	19696.2	17688.2	17496.3	#N/A	#N/A	#N/A	#N/A
O35143	ATPase inhibitor, mitochondrial	ATP5IF1	106	12.2	9.64	1656505.2	1542352.3	1566496.0	1461271.6	1562473.2	1570254.6	1835009.4	1534404.6	1508173.8	1372491.1	1745919.5	1599462.3	1713354.1	1618129.4
Q91VS8	FERM, ARHGEF and pleckstrin domain-containing protein 2	Farp2	1065	121.2	8.44	195675.9	190107.3	210243.9	185442.3	193427.4	215600.7	196294.7	190447.7	209954.7	213419.7	95158.3	100647.8	96777.9	96671.0
Q9JHL1	Na(+)/H(+) exchange regulatory cofactor NHE-RF2	Slc9a3r2	337	37.4	7.59	36157.9	28781.8	26632.6	26599.4	33983.8	36109.7	33069.7	31487.1	35745.9	26508.2	40656.9	66561.6	42340.7	50130.0
O35929	GTP-binding protein REM 1	Rem1	297	32.9	9.1	165905.8	154615.8	163417.2	142051.5	152613.2	177451.0	196764.6	156984.6	145415.9	152164.5	#N/A	#N/A	#N/A	#N/A
Q8C0R0	Ubiquitin carboxyl-terminal hydrolase 37	Usp37	979	110	6.23	78565.1	78812.7	75452.5	83512.6	82966.9	87105.9	75734.1	77602.9	81315.9	72372.6	#N/A	#N/A	#N/A	#N/A
Q9R111	Guanine deaminase	Gda	454	51	5.53	83563.3	93563.9	78407.8	130891.0	81597.4	65651.8	75757.3	94538.2	81757.8	84520.9	#N/A	#N/A	#N/A	#N/A
Q8C0T5	Signal-induced proliferation-associated 1-like protein 1	Sipa1l1	1782	196.9	8.13	108048.3	118723.4	110291.0	124462.6	130567.0	124141.1	116095.1	119360.8	102848.2	108378.2	141834.6	150557.5	161584.0	167582.4
Q8QZZ7	EKC/KEOPS complex subunit Tprkb	Tprkb	175	19.5	7.39	148949.6	145353.5	135387.6	137712.3	154532.1	181192.2	159990.0	124251.6	153534.0	149436.9	20653.2	27839.4	23595.0	20701.4
Q924X7	Serine/threonine-protein kinase 33	Stk33	491	54.4	6.43	168199.1	183929.4	181959.3	230262.3	130496.2	117534.8	117934.1	149413.4	139235.2	178759.1	160493.3	198234.4	185374.3	246657.1
Q9EST3	Eukaryotic translation initiation factor 4E transporter	Eif4enif1	983	107.9	7.74	144858.0	138663.2	133328.2	145526.2	134367.7	128577.3	128223.9	121429.7	137877.6	144002.3	176960.0	185112.8	185664.4	206756.8
Q9QZS0	Collagen alpha-3(IV) chain	Col4a3	1669	161.6	9.48	34273.1	40339.4	38185.6	36052.0	39464.0	36585.4	35736.7	39852.3	38223.9	42379.1	306997.5	295580.6	263765.6	279445.1
Q9CVD2	Ataxin-3	Atxn3	355	40.5	4.83	58199.2	64256.3	67460.7	64435.6	74514.9	74417.5	78352.5	64678.6	74345.9	69028.0	78859.2	83811.8	77409.0	76931.0
Q9D114	Guanosine-3',5'-bis(diphosphate) 3'-pyrophosphohydrolase MESH1	Hddc3	179	20.2	5.96	49760.0	57994.4	55295.6	54359.5	54303.0	64222.9	78081.5	54893.4	67437.3	66830.3	60480.0	76958.4	67162.7	76996.3
P49769	Presenilin-1	Psen1	467	52.6	5.38	19780.5	22135.9	21837.5	20103.6	23338.5	21199.6	24460.4	21607.2	21125.9	24362.0	#N/A	#N/A	#N/A	#N/A
Q5EBG8	Uncharacterized protein C1orf50 homolog	MGI:214046	199	21.8	5.53	53606.1	70844.9	65963.8	67261.7	74160.4	70402.6	61905.1	58441.6	66298.3	61042.6	151586.0	174869.4	158755.3	190430.0
F8VPU2	FERM, ARHGEF and pleckstrin domain-containing protein 1	Farp1	1048	118.8	7.88	233204.9	217222.8	250567.0	209456.8	248441.3	255986.4	295223.2	264703.3	259038.2	249587.4	140798.7	132114.1	130152.8	132561.0
Q80SY6	Adenosine deaminase-like protein	Adal	360	41	6.35	255882.6	274914.0	242592.7	258728.4	275759.9	277270.0	258848.1	241244.5	255928.9	254288.9	142413.0	150157.1	151628.6	165338.0
Q924Z4	Ceramide synthase 2	Cers2	380	45	8.75	147412.7	142056.1	144370.2	124963.6	130426.9	120918.3	118315.0	131448.1	126843.9	146524.4	203688.3	201252.0	219408.1	190365.1
Q6ZQB6	Inositol hexakisphosphate and diphosphoinositol-pentakisphosphate kinase 2	Ppip5k2	1129	128.3	7.94	101462.3	112268.6	107343.7	104840.3	109483.7	106479.0	102969.7	96034.0	99240.4	100389.2	127863.7	135193.3	127508.5	135131.1
Q80ZJ6	Protein zer-1 homolog	Zer1	779	89	5.94	102105.8	116229.3	102727.9	114859.8	115691.4	120743.5	104745.9	103761.1	114457.5	115617.1	128504.2	131368.5	132229.9	135285.7
Q8BZ21	Histone acetyltransferase KAT6A	Kat6a	2003	224.8	5.58	135916.7	156678.6	163913.2	157331.7	167202.6	160912.3	159547.8	152193.1	155519.0	157991.0	#N/A	#N/A	#N/A	#N/A
Q7TPE5	Probable RNA polymerase II nuclear localization protein SLC7A6OS	Slc7a6os	306	35	4.58	128943.6	131981.2	134158.4	134001.5	137333.5	132503.5	126064.8	125722.3	131306.1	130337.1	#N/A	#N/A	#N/A	#N/A
P98197	Probable phospholipid-transporting ATPase IH	Atp11a	1187	135.4	6.48	27223.6	26138.4	31532.4	30459.0	27983.2	31003.4	31847.2	30793.9	28372.6	29122.7	23854.6	28299.9	26849.9	26415.7
E9PX57	Kinesin-like protein	Kif6	802	90.6	7.03	218028.6	222114.1	229447.2	199231.0	206291.4	219970.0	176755.3	242717.0	209934.1	211764.4	#N/A	#N/A	#N/A	#N/A
Q1A3B0	Ceramide synthase 3	Cers3	383	46.1	8.13	366442.9	499062.8	502163.9	544866.4	289290.1	247525.2	219988.9	317098.0	412339.7	453438.2	365903.1	544331.0	521814.4	612172.6
Q8R3G9	Tetraspanin-8	Tspan8	235	25.6	5.16	120184.3	91255.9	92868.9	86458.6	123113.6	134436.0	245505.0	139812.8	101360.2	118572.8	94204.3	75070.7	88165.8	76848.2

Q5U680	S-adenosylmethionine mitochondrial carrier protein	Slc25a26	274	29	9.8	182831.6	184033.6	173814.8	190796.6	196861.3	180516.1	168322.0	179427.8	178153.6	173559.4	92037.0	89870.0	78121.6	83934.2
Q8BGY7	Protein FAM210A	Fam210a	273	31.6	9.82	269241.7	184105.1	217955.7	199197.5	199164.9	217281.5	173153.3	268646.7	189518.0	179766.0	115994.6	96622.3	93354.8	104770.6
Q3UZ18	Little elongation complex subunit 2	Ice2	988	109.8	6.77	11364.8	12924.1	11459.1	15070.4	14828.8	18226.1	12895.1	15504.2	16293.1	18589.9	#N/A	#N/A	#N/A	#N/A
Q9D0N7	Chromatin assembly factor 1 subunit B	Chaf1b	572	63.1	6.55	86028.6	93669.0	96130.1	87169.5	100975.7	107274.2	87445.6	91254.2	105455.4	94071.3	126969.4	135184.1	137183.2	123161.2
P63166	Small ubiquitin-related modifier 1	Sumo1	101	11.6	5.52	210004.9	231254.4	204003.1	206133.1	231709.7	197950.8	197280.5	183617.3	202475.6	200165.9	2747532.0	3238432.8	3273032.2	3468942.5
Q3UFF7	Lysophospholipase-like protein 1	Lyplal1	239	26.3	7.88	157315.0	130513.1	158991.9	148528.8	155028.3	138700.7	141379.6	156869.8	138551.1	154194.2	603200.5	513180.9	482659.3	510722.3
Q9D0R9	WD repeat-containing protein 89	Wdr89	386	42.4	5.66	148474.6	135645.7	150513.8	147800.9	148880.6	156377.5	163035.0	145957.0	143987.6	147171.7	#N/A	#N/A	#N/A	#N/A
P58064	28S ribosomal protein S6, mitochondrial	Mrps6	125	14.3	9.5	36413.6	42832.2	36578.8	39136.6	34071.0	33148.7	34737.4	38207.3	30191.3	32094.1	501069.6	538327.0	518388.2	497649.1
O35387	HCLS1-associated protein X-1	Hax1	280	31.6	4.91	43450.8	43672.8	40930.0	43825.3	40167.6	41204.5	40068.2	36732.0	43354.8	46112.5	#N/A	#N/A	#N/A	#N/A
Q9JM62	Receptor expression-enhancing protein 6	Reep6	201	22.2	7.33	421572.5	500773.7	392460.6	574642.9	391032.8	371534.7	333049.3	359148.5	398224.1	358702.8	114030.3	123283.5	96383.5	110251.8
P59438	Hermansky-Pudlak syndrome 5 protein homolog	Hps5	1126	126.3	5.74	7397.3	7249.2	6707.4	6049.5	7547.5	7505.3	5071.2	10093.4	7195.3	6713.7	#N/A	#N/A	#N/A	#N/A
Q9QXV1	Chromobox protein homolog 8	Cbx8	362	39.8	9.94	270753.9	267660.9	240101.6	253590.1	259227.1	269155.7	242875.4	267909.0	257801.4	251110.3	58369.9	58225.8	49551.3	53698.3
Q9JJ66	Cell division cycle protein 20 homolog	Cdc20	499	54.8	9.16	67185.7	77798.5	67474.3	74054.9	73908.6	65648.6	61296.2	68458.8	66082.8	73666.5	26441.5	30822.0	30609.1	33852.7
A2AI05	NADPH-dependent diflavin oxidoreductase 1	Ndor1	598	67.1	6.61	69531.0	77031.3	64086.7	79459.5	66581.6	71203.6	59433.8	65687.5	71706.9	69557.4	55763.3	57415.0	50859.6	57925.7
P62257	Ubiquitin-conjugating enzyme E2 H	Ube2h	183	20.6	4.67	139689.6	140081.4	123567.6	127851.3	135791.8	130569.9	127936.3	126928.9	131666.3	140501.3	160658.2	175270.1	155690.3	183099.2
Q91VH1	Adiponectin receptor protein 1	Adipor1	375	42.3	7.02	33448.9	34819.4	35939.2	33379.4	39706.3	37798.6	37998.7	39085.7	35013.9	38531.4	49281.8	41782.4	56197.9	50757.1
A0A1L1SSC7	TPR_REGION domain-containing protein	Ttc13	844	95	6.96	313009.5	323361.6	340920.1	343277.9	341248.0	323085.6	299574.5	310644.4	370822.3	357952.0	121823.4	153274.6	135074.1	167057.3
Q920N2	Biotin--protein ligase	Hlcs	722	78.5	5.34	35742.1	43284.7	33386.3	42465.2	42111.8	40135.5	33747.7	31379.8	44391.9	41869.0	112604.3	103540.4	99316.3	108221.1
Q3UGP8	Putative Dol-P-Glc:Glc(2)Man(9)GlcNAc(2)-PP-Dol alpha-1,2-glucosyltransferase	Alg10b	474	55.4	9.54	162251.2	164779.6	150980.7	165789.5	158361.1	162557.1	156119.2	151832.4	158593.1	171193.5	196214.7	213522.2	180562.6	205411.9
Q8R5H6	Wiskott-Aldrich syndrome protein family member 1	Wasf1	559	61.5	6.37	98816.3	85122.1	73554.4	73012.0	73327.5	70671.2	83003.7	75672.2	79654.3	76263.9	73603.3	81147.3	65257.9	70168.9
Q9D773	39S ribosomal protein L2, mitochondrial	Mrpl2	306	33.3	11.06	215101.6	225594.5	239383.0	225440.5	237123.4	252665.3	226509.6	246248.7	213337.3	216166.6	#N/A	#N/A	#N/A	#N/A
Q6P073	Ubiquitin-conjugating enzyme E2 J2	Ube2j2	259	28.9	8.62	62949.3	79008.4	67404.3	73526.2	63869.4	78051.9	59749.8	65154.8	63663.9	76836.0	74227.8	80632.0	90019.2	83826.4
Q09200	Beta-1,4 N-acetylgalactosaminyltransferase 1	B4galnt1	533	59.2	8.59	207806.1	236380.9	210278.3	237206.2	203511.3	188004.6	183467.5	202506.8	190624.9	214396.3	79467.1	85394.4	71953.3	84469.8
Q99LT0	Protein dpy-30 homolog	Dpy30	99	11.2	4.88	56809.7	78654.9	53931.9	74086.3	76368.8	66190.1	57011.9	67830.9	69050.1	69350.8	35678.1	40990.1	31467.9	36886.4
O70546	Lysine-specific demethylase 6A	Kdm6a	1401	154.3	7.39	77565.3	84160.8	81056.7	78792.9	78399.6	81887.7	73720.2	69992.1	80705.6	85923.6	25114.7	24231.4	24138.2	21077.2
Q8BMG1	ATR-interacting protein	Atrip	785	85.5	6.25	10876.0	11330.2	13736.1	9007.3	14228.3	9639.7	9245.3	10904.1	12339.8	10901.9	#N/A	#N/A	#N/A	#N/A
Q9D2Y4	Mixed lineage kinase domain-like protein	Mlkl	472	54.3	8.25	188759.0	169321.6	160443.8	203643.9	176961.1	186533.4	193973.2	168535.4	154326.5	159453.2	81222.9	84762.2	77324.8	83856.4
Q61475	Complement decay-accelerating factor, GPI-anchored	Cd55	390	42.6	8.4	34912.9	40397.0	32302.3	35836.7	37249.4	44044.0	34596.2	36854.9	35987.0	35573.3	397213.1	408351.3	417488.1	365186.4
Q91YD6	Villin-like protein	Vill	859	96.4	6.35	59095.2	66711.7	65207.1	60960.8	71376.5	68751.4	60768.4	56080.8	59976.6	65387.9	#N/A	#N/A	#N/A	#N/A
Q9DB50	AP-1 complex subunit sigma-2	Ap1s2	160	18.9	5.48	377295.1	398647.8	437930.5	420153.4	452945.7	440976.1	377052.5	373344.1	452764.9	435708.3	431021.6	464411.5	429753.8	415848.5
Q9CPV7	Palmitoyltransferase ZDHHC6	Zdhhc6	413	47.5	8.54	19239.3	18111.9	20633.6	19787.0	19663.1	25494.7	19121.2	23997.7	20198.2	21749.3	#N/A	#N/A	#N/A	#N/A
Q8BH07	ADP-ribosylation factor-like protein 6-interacting protein 6	Arl6ip6	226	24.9	7.77	669071.3	703012.1	691802.4	674144.5	1297859.1	1028977.3	805606.0	619363.7	752273.7	746197.9	163038.9	138052.7	165039.6	139201.5
Q9D823	60S ribosomal protein L37	Rpl37	97	11.1	11.74	199991.0	213563.7	199021.2	196171.5	175002.7	182247.2	165585.9	181798.8	187912.4	198995.4	379520.4	380230.8	395404.9	368543.8
Q14B01	Ring finger protein 113A2	Rnf113a2	337	38	6.46	210921.5	195156.3	195213.8	186571.1	203751.6	207269.9	224831.3	195064.4	202903.9	214549.4	39007.0	36412.5	36626.5	36361.6
Q6GQS1	Calcium-binding mitochondrial carrier protein SCaMC-3	Slc25a23	467	52.5	7.59	264474.6	273057.0	247196.4	254986.2	282000.3	283198.5	286274.3	255173.7	264714.3	270716.1	142690.6	144426.3	130301.9	140424.0
P33242	Steroidogenic factor 1	Nr5a1	462	52	7.53	61082.2	68936.4	53357.1	57918.0	64661.9	66946.5	56633.8	58861.7	63377.6	57294.1	149284.3	153590.1	149551.5	147967.6
Q8R310	Transmembrane and coiled-coil domain protein 3	Tmcc3	477	53.7	8.6	48277.4	51344.0	43878.4	47923.4	52128.9	54871.2	51219.8	44355.4	53929.1	52519.2	27827.2	31259.8	28325.6	32311.7
Q9DCC7	Isochorismatase domain-containing protein 2B	Isoc2b	210	23.1	8.18	39163.4	34420.1	34441.4	40140.1	34212.3	31801.4	42060.4	35523.2	35149.0	37351.0	79837.6	80330.5	70706.2	86099.6
Q8CDJ3	Beclin 1-associated autophagy-related key regulator	Atg14	492	55.4	7.81	177591.6	194408.1	174716.5	180203.1	182362.0	166696.6	165371.9	168381.5	185090.5	181311.3	45650.6	52011.2	47361.5	46066.8
Q3TCX3	KH homology domain-containing protein 4	Khdc4	612	64.5	8.73	191274.8	199387.8	200228.8	212272.4	219931.5	214229.1	218667.7	202738.6	222440.7	215412.0	334815.4	378919.4	22779.8	392169.2
Q7TMW6	Cytosolic iron-sulfur assembly component 3	Ciao3	476	53.1	6.48	97722.1	83787.5	88816.4	77307.9	91720.0	92315.8	88441.8	84255.3	77110.5	83254.1	90849.9	94002.0	92857.0	89730.7
Q8K0P3	MTOR-associated protein MEAK7	Meak7	455	50.8	6.52	95857.0	105224.0	83518.9	96262.5	95934.8	95531.5	93291.7	86979.9	99222.4	94011.2	36467.2	32280.1	30421.7	24246.6
Q9Z1N9	Protein unc-13 homolog B	Unc13b	1602	181.7	5.95	81297.8	82386.7	79421.8	69954.3	81491.0	80174.1	68141.4	82833.0	75065.3	88415.2	263503.8	225482.0	243060.9	216770.9
P0CW02	Lymphocyte antigen 6C1	Ly6c1	131	14.2	6.01	77916.5	88299.4	81423.8	81182.5	86298.9	88396.9	89285.9	75196.0	87191.5	88512.6	64688.6	75442.7	60812.2	67258.4
Q99JY3	GTPase IMAP family member 4	Gimap4	328	38	6.23	86228.6	90810.7	78823.1	88371.4	98284.2	100044.1	103201.2	87903.5	92863.8	91759.0	30539.1	34750.5	27521.7	27384.7
Q8BKN5	Gamma-tubulin complex component 5	Tubgcp5	1024	117.9	5.99	91235.3	99077.2	80401.3	97438.1	93539.9	91028.7	95629.5	84153.3	86557.7	96545.4	#N/A	#N/A	#N/A	#N/A
O35955	Proteasome subunit beta type-10	Psmb10	273	29	6.87	57896.8	70505.3	66041.9	64032.1	61899.1	61424.5	59082.1	60022.2	51675.9	68938.8	124715.7	128945.8	123013.2	133333.6
Q9QZF2	Glypican-1	Gpc1	557	61.3	7.05	86101.7	75579.6	74453.1	73498.1	86794.2	88342.7	97235.5	96887.4	67509.1	74594.4	44609.1	44976.3	42623.7	38462.4
Q6PHS6	Sorting nexin-13	Snx13	957	110.7	6.62	68815.4	85797.9	76630.1	66594.9	74809.4	78745.9	79369.0	72607.4	78057.0	76525.3	#N/A	#N/A	#N/A	#N/A
Q9D289	Trafficking protein particle complex subunit 6B	Trappc6b	158	17.9	8.68	211920.6	239264.6	195478.7	240100.0	206590.1	199520.8	203755.0	200411.6	224825.3	236668.7	1245490.7	1262096.1	1570624.0	1460237.3
Q5SXJ3	Fanconi anemia group J protein homolog	Brip1	1174	131.3	7.5	136903.0	169623.2	131515.8	133579.9	155294.7	170785.3	132639.5	128019.5	147061.0	143175.1	165546.1	170625.6	152336.7	175478.0
O35710	Nocturnin	Noct	429	48.3	7.27	45462.4	49381.9	51461.6	50670.8	50199.3	47665.6	44186.5	46577.4	47811.0	44304.5	138909.6	148577.5	128527.2	128695.7
P28661	Septin-4	Septin4	478	54.9	5.87	185345.7	182231.3	184095.2	177712.5	206257.6	198101.3	208784.4	172766.2	180607.8	186242.1	174921.0	169077.9	161159.0	167663.0
Q8BR65	Sin3 histone deacetylase corepressor complex component SDS3	Suds3	328	38.1	5.66	81810.3	103320.1	94222.8	106577.0	100256.6	101869.2	99401.2	96400.0	102774.5	102748.5	159279.6	162053.3	166461.9	165986.1
Q9D8Z6	Autophagy-related protein 101	Atg101	218	25	6.15	14421.6	18580.2	14699.3	12210.6	12816.7	18459.6	13764.4	15481.8	18494.1	16345.8	#N/A	#N/A	#N/A	#N/A
Q3TTA7	E3 ubiquitin-protein ligase CBL-B	Cblb	982	109	7.96	45522.2	45674.9	43319.4	40267.4	44551.3	47099.3	39710.6	39696.7	47905.1	40726.0	35329.3	31036.2	32458.6	36429.1
Q810N3	UPAR/Ly6 domain-containing protein	Lypd10	246	26.5	6.81	518970.0	812076.5	822057.0	1027083.9	419539.8	277679.3	247898.7	417377.4	661594.1	710901.8	231842.0	412474.8	458146.2	549990.3
Q8R2U7	Leucine-rich repeat-containing protein 42	Lrrc42	421	47.9	7.4	392880.9	372048.1	452470.3	358740.9	444532.9	426527.0	418726.5	444784.6	423852.9	404989.4	#N/A	#N/A	#N/A	#N/A
Q05A62	Dynein light chain 1, axonemal	Dnal1	190	21.5	5.76	23106.2	21736.8	26356.9	36416.7	18552.2	16127.7	19328.5	19722.8	28305.9	20508.1	26551.5	29730.0	30346.2	41203.5
Q14BQ3	Cancer/testis antigen 55	Ct55	236	26.3	5.26	112257.1	91858.4	88386.6	105568.3	92299.2	91363.9	89079.4	92520.4	105612.6	108875.4	40050.5	44502.1	41588.5	37800.7
Q3TYD6	Serine/threonine-protein kinase LMTK2	Lmtk2	1471	160.4	4.51	34171.8	34568.2	29316.5	32992.2	37446.7	30033.3	29274.3	30878.3	29363.5	33633.6	47345.1	46275.2	44444.6	43184.0
A2A791	Zinc finger MYM-type protein 4	Zmym4	1549	172.3	6.8	15379.3	14302.2	14260.7	11139.2	10243.9	18949.7	16576.0	13447.1	9092.3	13553.2	103690.7	102549.8	106316.0	103173.5
O08609	Max-like protein X	Mlx	298	33.3	6.62	16199.4	14659.1	24532.9	18605.5	16978.7	22453.2	21633.2	20286.7	20700.2	12898.7	138256.3	138070.2	158138.4	127334.1
Q99NF3	Centrosomal protein of 41 kDa	Cep41	373	41.4	8.12	51380.0	58645.8	54236.3	59768.2	51909.5	54262.2	47907.7	50031.3	55343.2	52331.0	115064.4	112645.3	102331.9	102191.4
Q9QZJ6	Microfibrillar-associated protein 5	Mfap5	164	18.5	5.94	66659.0	62027.2	64203.8	61774.6	92697.7	116891.3	105909.5	73858.1	68081.7	76590.2	#N/A	#N/A	#N/A	#N/A
Q9CZ15	DNA replication complex GINS protein PSF1	Gins1	196	22.9	7.43	89045.6	86370.8	77721.3	85281.1	83783.9	92106.3	80430.7	80141.7	88370.5	93895.3	24058.2	28709.9	23171.2	24463.3
Q8K304	E3 ubiquitin-protein ligase TM129	Tmem129	362	40.6	7.06	20010.4	26274.6	28007.4	26258.8	27358.9	30023.0	23056.7	33127.7	25746.3	26288.8	#N/A	#N/A	#N/A	#N/A
Q9CWU6	Ubiquinol-cytochrome-c reductase complex assembly factor 1	Uqcc1	295	34.3	8.48	139478.9	143205.2	127657.9	151711.5	116718.2	114515.3	115309.3	121330.9	124965.0	129069.0	183496.2	193972.4	178361.6	201931.4
Q9DBS8	Centrosomal protein POC5	Poc5	558	60.9	8.09	92844.7	98716.8	95938.2	87519.5	107853.9	103622.7	101609.1	86338.3	92295.4	98625.6	39419.6	34937.7	39050.4	30268.2
Q9ERI5	Bifunctional arginine demethylase and lysyl-hydroxylase JMJD6	Jmjd6	403	46.5	8.9	194979.5	206294.1	214415.7	193174.4	209277.7	208491.7	197529.1	195679.6	190773.1	205472.2	#N/A	#N/A	#N/A	#N/A
P70675	Testis-specific protein TSX	Tsx	144	16	3.89	50455.3	68831.4	54540.2	73409.6	23578.4	23626.9	19240.8	60404.7	53942.5	40962.6	610176.4	41284.1	44953.2	50401.9
Q9R049	E3 ubiquitin-protein ligase AMFR	Amfr	643	73.1	6.46	50779.8	48438.3	50077.5	52952.2	53359.0	55206.6	52103.3	52798.4	58961.2	65559.6	117531.7	126556.7	120268.7	125685.4
Q8VBX6	Multiple PDZ domain protein	Mpdz	2055	218.6	5.02	270741.5	259098.6	248060.5	262908.3	268237.1	284704.7	296515.1	278204.7	277820.8	287403.1	7517.9	8043.4	7155.7	9037.0
A2AIL4	NADH dehydrogenase (ubiquinone) complex I, assembly factor 6	Ndufaf6	333	38.3	9.44	8452.5	7352.8	8335.4	9241.1	10823.9	9679.4	7741.1	8476.1	11596.9	8199.5	28959.6	27685.0	30301.5	28773.5
Q921R8	Solute carrier family 41 member 3	Slc41a3	488	53.2	7.65	15909.0	12822.0	15220.2	18226.7	18431.8	17823.1	18010.4	19786.8	18187.0	12315.7	24917.0	32036.7	27286.5	30634.7
Q7TND5	Ribosome production factor 1	Rpf1	349	40	9.99	284500.9	303925.3	282651.2	265798.2	310077.0	305086.2	271856.2	278447.9	307841.2	294120.8	#N/A	#N/A	#N/A	#N/A
Q99J56	Derlin-1	Derl1	251	28.8	9.51	171200.4	163828.4	138573.7	163482.0	158067.3	153980.5	141202.9	155937.1	151029.1	162081.0	336281.6	341516.3	317149.9	345208.5
P18826	Phosphorylase b kinase regulatory subunit alpha, skeletal muscle isoform	Phka1	1241	138.7	5.86	52633.9	58001.3	48030.9	49980.8	50426.2	46819.2	46815.7	44073.5	42851.7	54466.5	147729.8	125505.8	136487.9	151179.2
Q9Z0Z4	Hephaestin	Heph	1157	129.6	6.13	155413.8	132154.0	119361.7	141505.8	123994.2	126194.0	125019.6	129749.7	130714.7	133834.4	117880.7	97934.8	113500.7	115115.3
Q91VY5	Lysine-specific demethylase 4B	Kdm4b	1086	121.5	7.42	35087.7	49636.7	40812.5	41476.7	45576.4	46299.2	40769.8	42320.5	44303.5	48908.1	82932.9	79346.9	90736.9	88699.4
Q8BUY5	Complex I assembly factor TIMMDC1, mitochondrial	Timmdc1	285	31.8	9.29	63792.3	65134.5	65909.0	65172.9	68721.4	72992.5	68222.2	62523.5	69979.0	60651.3	92611.6	103422.9	92491.9	93279.6
Q3V140	Acrosin-binding protein	Acrbp	540	61.1	4.98	116007.7	181662.6	168844.0	373082.5	97381.8	91272.7	80152.4	91398.0	123607.1	151985.1	118424.6	192160.8	189205.7	393324.2
Q9R1Z7	6-pyruvoyl tetrahydrobiopterin synthase	Pts	144	16.2	6.52	27446.6	33527.2	29187.1	30954.1	34284.2	30358.7	33373.8	30157.3	25203.2	29063.4	68429.2	51984.2	55157.5	70252.1
Q8CFK2	Transcription factor IIIB 90 kDa subunit	Brf1	676	73.8	5.3	132166.4	138929.7	122067.0	130068.5	138993.9	140414.4	135478.6	130810.4	135188.3	140963.7	#N/A	#N/A	#N/A	#N/A
Q9DC53	Copine-8	Cpne8	577	64.6	5.97	61637.4	56178.1	56650.9	55883.9	64899.0	63310.9	54798.4	61671.4	52894.5	62899.3	27343.0	34732.4	29071.1	29130.5
Q9QXP4	Protein downstream neighbor of Son	Donson	560	61.9	8.22	70540.8	79264.6	78023.5	85653.9	85442.8	81163.1	76146.0	74054.3	72055.4	80518.8	140554.9	169999.3	148543.0	178634.7
Q9CPT3	N-acylneuraminate-9-phosphatase	Nanp	248	27.8	6.07	190940.1	167976.3	164243.3	168012.2	164024.4	155065.4	175921.1	161326.1	165688.3	186989.1	228237.1	186479.5	185513.8	205118.2
L0N7N1	Kinesin-like protein KIF14	Kif14	1674	186.3	7.84	558038.7	511978.5	345527.6	499415.0	390512.3	387189.2	420670.6	360706.2	444831.7	447416.5	309125.1	288950.1	247711.1	290707.3
Q02013	Aquaporin-1	Aqp1	269	28.8	7.43	74751.8	89402.4	69309.8	91153.4	93572.3	90295.2	107852.9	88370.5	82157.8	83661.7	141138.2	141847.8	130116.0	162731.8
Q9WTK7	Serine/threonine-protein kinase STK11	Stk11	436	49.2	6.84	138191.5	129986.0	128648.7	128253.7	126425.3	127678.8	125968.2	124763.8	147230.6	137796.8	99043.8	105372.2	100459.5	111477.1
Q8CE50	Sorting nexin-30	Snx30	437	49.5	5.35	123551.3	107194.0	103199.3	100357.0	94866.9	106216.1	105972.5	103791.1	106527.2	108832.8	77456.0	79886.1	76001.3	82604.5
Q9D7I8	Protein FAM83D	Fam83d	585	64.3	6.77	20056.8	21173.5	19029.2	21180.7	21353.0	20819.3	19805.4	21218.9	15792.6	22634.3	#N/A	#N/A	#N/A	#N/A

Q9JJ00	Phospholipid scramblase 1	Plscr1	328	35.9	5.06	50168.1	55268.1	54744.3	53854.9	58137.5	68238.1	79543.8	58495.7	58462.7	57686.0	27023.4	28709.1	22049.8	29365.1
Q6NVD0	FRAS1-related extracellular matrix protein 2	Frem2	3160	350.4	5.12	35750.2	43527.0	44344.5	45422.5	44497.3	54756.2	48404.2	49719.7	44891.2	45132.8	62922.2	66604.0	60110.0	62470.8
Q4VGL6	Roquin-1	Rc3h1	1130	125.3	7.27	23157.8	30223.6	24711.3	25177.5	26922.8	26895.2	23254.5	24632.1	24021.4	28195.5	#N/A	#N/A	#N/A	#N/A
Q9JKL4	NADH dehydrogenase [ubiquinone] 1 alpha subcomplex assembly factor 3	Ndufaf3	185	20.7	8.05	108882.8	125005.0	99423.6	110032.4	121785.9	123349.3	109738.7	105066.5	116381.4	115097.9	103982.9	117684.6	104857.3	102865.5
O08550	Histone-lysine N-methyltransferase 2B	Kmt2b	2713	294.6	8.1	174473.8	186154.4	172628.2	163639.9	190518.6	204380.1	171558.0	152708.8	178792.0	177274.3	156813.5	183798.7	150410.6	169506.7
Q9CQS2	H/ACA ribonucleoprotein complex subunit 3	Nop10	64	7.7	9.99	169148.2	178987.9	170392.9	174832.2	209808.0	194535.3	185693.8	161648.3	181020.7	176998.1	539540.3	521086.0	610701.6	556292.8
Q8K2B0	Endoplasmic reticulum protein SC65	P3h4	443	51.1	4.81	29712.7	33343.4	20698.9	27877.5	26800.7	26785.8	21456.9	27995.6	23411.4	22742.6	36160.2	33628.9	27240.4	32380.1
Q9CPR7	Suppressor of IKBKE 1	Sike1	207	23.5	5.38	151643.5	137178.4	144519.6	138449.5	144786.0	133617.8	124985.2	147435.2	148810.4	131272.2	104614.7	116812.0	128508.7	117522.4
Q9D136	2-oxoglutarate and iron-dependent oxygenase domain-containing protein 3	Ogfod3	315	35.4	6.74	39243.3	34323.4	38554.9	40246.7	42844.1	41505.0	38104.3	43414.5	39397.4	38444.1	120586.4	102462.4	111168.5	108528.3
Q8K396	Meiotic nuclear division protein 1 homolog	Mnd1	205	23.8	8.31	230594.2	294678.9	268649.7	275026.8	281195.8	263730.5	235495.6	235356.5	281534.7	286233.3	34374.9	49377.6	37295.9	43054.1
Q8BHS6	Armadillo repeat-containing X-linked protein 3	Armcx3	379	42.6	8.68	92555.0	50890.4	82987.2	91015.6	43654.5	53473.5	42885.6	42081.3	100225.8	42764.6	83407.1	82916.4	77559.9	77122.0
Q5I1X5	RelA-associated inhibitor	Ppp1r13l	824	88.9	6.86	57040.5	60449.8	59638.7	59202.6	59598.8	66480.7	63186.5	60985.6	51378.7	57713.2	105825.1	118039.2	103729.2	112035.2
Q9CWV6	PRKR-interacting protein 1	Prkrip1	186	21.5	9.42	261375.6	278505.2	246691.9	264869.4	251536.4	251502.3	257630.5	222062.6	256576.0	276861.8	263260.7	231593.6	302910.4	200779.8
Q9CX66	Uncharacterized protein C12orf45 homolog	D10Wsu102	185	20	4.84	157238.4	156069.1	145671.5	146655.5	142772.4	141828.0	145894.7	140511.5	153797.2	151583.6	141434.7	133760.5	129403.8	125192.3
Q9DCD6	Gamma-aminobutyric acid receptor-associated protein	Gabarap	117	13.9	8.79	335295.6	335248.0	346687.0	345815.7	336163.3	339031.6	300860.1	329500.5	291336.9	302495.3	#N/A	#N/A	#N/A	#N/A
Q3UMW8	Ceroid-lipofuscinosis neuronal protein 5 homolog	Cln5	341	39.3	8.68	84907.3	95330.2	74045.2	77189.8	82668.8	78785.3	78096.1	73778.8	87206.1	83027.3	150378.8	193275.0	146078.5	162149.8
Q9CPW7	Zinc finger matrin-type protein 2	Zmat2	199	23.6	9.01	381478.6	378348.6	457260.7	375403.5	406536.5	386005.6	387358.4	376521.7	408408.5	429823.4	165976.5	179932.9	184336.2	175055.1
Q9JMG3	Transmembrane and ubiquitin-like domain-containing protein 1	Tmub1	245	26.3	5.03	36215.2	34417.7	31315.7	39418.8	43621.0	34746.3	44345.4	47532.7	35907.8	35545.3	94643.1	106343.4	91880.1	94877.3
Q7TSF4	Leucine-rich repeat-containing protein 75A	Lrrc75a	339	37.6	8.76	157255.9	176523.4	163681.9	148621.7	182228.7	184429.0	182953.7	165436.9	177980.9	179561.5	197094.8	192946.4	178906.7	197355.2
G5E8L7	Glycosylphosphatidylinositol-anchored molecule-like 2	Gml2	176	19.9	5.54	608770.5	759217.9	898096.8	926204.0	579004.2	485919.3	380128.8	484193.8	677710.1	689364.7	628338.0	642629.2	577281.8	710760.7
Q9R1K9	Centrin-2	Cetn2	172	19.8	5	214608.7	192679.6	220704.0	213078.6	222245.8	224366.6	218211.8	210675.5	228366.3	200635.8	105234.6	113502.9	120777.4	108157.3
Q91VK4	Integral membrane protein 2C	Itm2c	269	30.5	8.59	65312.2	76922.7	69919.5	72195.7	74816.1	75052.2	66299.6	69663.8	72784.8	70365.4	52529.0	54561.7	50720.7	51492.8
Q9WTZ1	RING-box protein 2	Rnf7	113	12.7	5.44	220057.3	201850.2	199036.1	231217.9	214548.1	213199.3	231042.3	234710.6	218506.5	210565.0	197542.6	217694.3	201020.5	200221.3
O08740	DNA-directed RNA polymerase II subunit RPB11	Polr2j	117	13.2	5.86	130896.7	157882.0	170110.7	154892.8	164916.8	157557.0	127641.0	147544.9	184429.0	151993.1	159670.9	165025.5	193086.0	181116.5
Q9CXD9	Leucine-rich repeat-containing protein 17	Lrrc17	443	51.8	8.34	51636.2	50643.7	50634.7	51731.6	59237.1	60902.6	66094.6	50212.9	55076.0	57918.7	238991.5	209343.4	230111.5	201309.6
Q9R078	5'-AMP-activated protein kinase subunit beta-1	Prkab1	270	30.3	6.23	69891.2	63989.6	54263.6	64033.3	64498.8	59285.2	62054.1	58568.3	59344.7	66999.9	62481.5	73114.9	58782.2	53108.4
Q8K072	Receptor expression-enhancing protein 4	Reep4	257	29.7	9.79	49851.2	58338.8	49793.9	49282.0	51507.0	47070.4	34729.0	49811.8	50627.7	55874.7	#N/A	#N/A	#N/A	#N/A
O70126	Aurora kinase B	Aurkb	345	39.4	9.44	248748.3	170984.0	166387.6	231534.1	178214.0	179751.8	245957.3	217679.9	227162.1	216818.4	#N/A	#N/A	#N/A	#N/A
Q5SS80	Dehydrogenase/reductase SDR family member 13	Dhrs13	376	40.7	7.93	101011.6	112877.7	104794.1	105062.5	109869.0	120112.1	97958.7	109522.9	105000.3	119512.7	26519.8	34583.2	24041.7	27300.7
Q91W69	Epsin-3	Epn3	636	68.2	5.88	41243.3	43140.2	37095.9	38645.1	41421.7	43547.7	43008.9	39071.6	39503.8	33937.2	#N/A	#N/A	#N/A	#N/A
Q8R0F3	Formylglycine-generating enzyme	Sumf1	372	40.6	7.09	87710.9	75749.1	66807.8	64460.5	78155.2	78703.5	80635.6	76490.9	75807.9	84017.9	58978.0	51491.9	57220.1	51186.5
O88697	Serine/threonine-protein kinase 16	Stk16	305	34.4	6.46	44063.2	50970.0	45688.4	44993.4	54581.1	46383.0	43705.1	44800.8	49399.6	51073.4	16929.3	18142.8	18602.2	17746.1
Q3UMG5	Leucine-rich repeat and calponin homology domain-containing protein 2	Lrch2	773	84.9	6.58	133061.1	150270.5	132253.6	133946.6	161347.9	167258.1	131910.3	137690.5	159077.8	155160.8	89858.0	95754.9	104021.1	92590.8
Q80TQ5	Pleckstrin homology domain-containing family M member 2	Plekhm2	1018	112.7	4.96	54982.7	51594.4	54611.7	60536.2	53345.8	58532.5	70681.3	42057.9	49576.0	54791.8	#N/A	#N/A	#N/A	#N/A
Q9Z0M5	Lysosomal acid lipase/cholesteryl ester hydrolase	Lipa	397	45.3	8.07	111307.6	135151.5	103830.7	117406.1	117891.2	146536.1	117999.2	126148.6	112529.5	104792.2	148248.5	148044.1	131971.9	150376.7
Q8BI79	Coiled-coil domain-containing protein 40	Ccdc40	1192	136.7	5.05	51482.7	70431.2	65262.4	104587.9	48187.9	44982.9	49519.5	52635.4	50098.1	62937.9	202711.2	243970.0	241385.4	330676.5
Q9D7N6	39S ribosomal protein L30, mitochondrial	Mrpl30	160	18.3	10.15	211776.9	206524.6	188352.0	201760.9	200438.5	204855.6	189219.1	200159.3	206364.7	206820.0	792069.3	736303.6	774282.3	751728.6
P70663	SPARC-like protein 1	Sparcl1	650	72.2	4.6	184173.0	201970.7	157901.2	203808.1	183845.9	183708.9	177301.1	181487.4	175634.9	187408.9	47747.9	46681.8	38019.2	39070.3
O35454	Chloride transport protein 6	Clcn6	870	96.9	7.02	129084.6	132748.2	128706.2	111629.9	148645.4	153886.5	135115.5	141166.6	120864.1	137585.2	#N/A	#N/A	#N/A	#N/A
Q9CRC3	UPF0235 protein C15orf40 homolog	MGI:191454	126	13.2	9.16	48348.4	39491.7	44476.0	47672.7	48779.0	43175.2	40003.9	40431.6	41536.6	42298.2	15226.4	23680.2	17290.6	21706.5
P08414	Calcium/calmodulin-dependent protein kinase type IV	Camk4	469	52.6	4.93	96618.9	115885.1	127719.3	107844.1	115313.7	119014.7	118918.0	109177.2	111086.5	135674.0	#N/A	#N/A	#N/A	#N/A
Q924A2	Protein capicua homolog	Cic	2510	258	8.02	105077.6	117118.0	85062.1	96576.9	167346.6	122293.2	105575.8	107136.5	95369.1	111524.9	376025.6	375181.6	338561.8	354396.1
Q9CQG6	Transmembrane protein 147	Tmem147	224	25.3	7.77	126893.4	138840.9	130412.0	149576.2	136040.7	120387.8	113753.6	123504.7	135652.6	118386.0	599541.5	685134.5	570754.6	643854.8
Q9R0X0	Mediator of RNA polymerase II transcription subunit 20	Med20	212	23.2	6.87	153295.7	166342.5	143273.9	149205.2	144916.2	155909.8	151832.1	133261.2	146720.7	158420.1	289438.6	261278.9	282938.9	252973.5
Q60790	Ras GTPase-activating protein 3	Rasa3	834	95.9	7.34	174276.5	167484.3	143644.9	156824.7	150851.6	158559.9	166661.3	140890.8	160491.5	151108.4	78753.1	71936.8	62513.8	69559.7
Q6AW46	Carboxylesterase 5A	Ces5a	575	64.1	5.8	121829.3	121721.6	103105.8	194429.0	104259.7	157459.2	124660.0	204408.9	115253.5	115828.7	#N/A	#N/A	#N/A	#N/A
Q60805	Tyrosine-protein kinase Mer	Mertk	994	110.1	5.5	138422.8	122552.0	124247.7	122244.6	128696.2	123337.8	129705.1	125764.1	121144.5	144686.8	#N/A	#N/A	#N/A	#N/A
Q3UIJ9	Myocardial zonula adherens protein	Myzap	466	53.9	6.51	173996.3	147343.8	156538.6	143644.8	159563.9	170206.7	178865.1	167949.8	168670.3	149066.8	47927.6	63225.1	46146.7	43760.8
Q9D845	Testis-expressed protein 9	Tex9	387	44	7.65	109961.5	117860.5	117170.1	121861.7	120336.5	121504.8	111386.6	117923.0	118000.9	120410.2	133836.3	169984.7	152886.4	178619.3
Q9D1M7	Peptidyl-prolyl cis-trans isomerase FKBP11	Fkbp11	201	22.1	9.36	327898.4	302833.4	308874.9	307286.2	369054.5	357796.9	346197.6	316904.3	309682.3	351094.7	#N/A	#N/A	#N/A	#N/A
Q3U182	CREB-regulated transcription coactivator 2	Crtc2	692	73.2	7.01	59696.0	70450.0	55818.7	60032.3	60342.9	59294.1	55924.5	54953.8	66868.4	62624.7	#N/A	#N/A	#N/A	#N/A
Q9QX66	Zinc finger protein neuro-d4	Dpf1	387	44.2	7.09	28703.9	32186.6	25243.1	31938.1	29084.2	25485.1	27651.8	26035.2	26535.9	30866.9	75929.0	88317.6	72952.1	79225.3
Q3V0Q1	Dynein heavy chain 12, axonemal	Dnah12	3086	356	6.06	46617.6	38556.8	36708.5	33287.6	37915.4	40503.3	40431.6	44290.9	40481.0	34464.0	138808.6	171221.5	160177.2	219156.6
Q9QUG9	RAS guanyl-releasing protein 2	Rasgrp2	608	69.4	7.66	10366.0	6687.0	7730.6	9958.3	8729.8	10605.6	10277.7	8009.7	8686.0	9170.2	8405.7	10370.2	8865.6	9679.7
Q9CPR1	RWD domain-containing protein 4	Rwdd4	188	21.1	5.63	153617.4	163234.7	153132.7	171103.5	148264.6	152420.6	151128.6	148099.7	155943.4	178808.0	220911.3	279509.3	212703.0	239027.8
Q63ZW7	InaD-like protein	Patj	1834	198.4	4.97	18979.5	14774.3	19973.5	19749.3	21654.6	19816.5	23957.6	21094.0	20822.2	18276.6	#N/A	#N/A	#N/A	#N/A
Q3UL97	Activating transcription factor 7-interacting protein 2	Atf7ip2	452	50.9	9.45	238786.7	261231.4	226675.1	243180.9	238197.4	247459.3	205702.9	206106.9	231642.3	246156.8	183328.4	204986.3	183253.9	197067.0
B1AQJ2	Ubiquitin carboxyl-terminal hydrolase 36	Usp36	1098	119.8	9.82	180478.4	165389.9	179304.9	162258.6	206591.0	174235.2	178580.6	162317.8	173811.7	166682.1	#N/A	#N/A	#N/A	#N/A
P51612	DNA repair protein complementing XP-C cells homolog	Xpc	930	104.5	8.81	11752.0	15209.1	12814.8	13264.3	16192.8	13029.5	12974.1	12034.3	15709.4	14639.0	259848.0	252230.0	271213.9	230076.0
Q9WUP4	Polyprenol reductase	Srd5a3	330	37.9	9.79	104561.4	120706.7	105703.8	112037.2	122995.4	134030.5	106491.9	117146.5	116805.8	115887.2	70417.7	79334.0	75172.0	78670.1
Q7TQK0	Cyclin-T2	Ccnt2	723	80.2	8.91	90844.0	102479.7	90199.8	87069.3	92739.0	104627.5	88586.0	86681.1	95485.2	105784.6	#N/A	#N/A	#N/A	#N/A
Q8BJH1	Zinc finger C2HC domain-containing protein 1A	Zc2hc1a	324	35.1	9.88	109000.0	123358.6	107153.1	112190.4	120722.4	119795.8	113745.4	99875.9	113579.4	120592.6	201166.4	178656.2	175717.4	177624.9
Q9JMC3	DnaJ homolog subfamily A member 4	Dnaja4	397	44.9	7.58	117074.9	179254.7	95204.9	123113.9	111616.6	142456.6	85923.5	115868.4	110749.7	138591.0	125020.8	129378.2	148171.9	141582.2
Q6NXH3	Vasculin	Gpbp1	473	53.4	6.9	52873.0	60983.8	63951.8	74530.1	70579.1	62537.7	69820.9	63936.5	67470.4	66663.2	66073.5	74989.6	54089.3	65805.4
P59048	p53 and DNA damage-regulated protein 1	Pdrg1	133	15.4	8.1	191245.5	183887.9	196335.5	181136.8	196585.4	210789.6	162192.5	173526.6	195596.8	190668.4	#N/A	#N/A	#N/A	#N/A
Q8C5K5	Uncharacterized protein CXorf38 homolog	MGI:19164C	320	36.6	6.47	377508.5	371420.7	315001.1	337794.1	353602.1	361471.3	350741.9	303171.4	353138.0	342728.8	105344.0	98605.2	98719.9	102511.7
Q8VC52	RNA-binding protein with multiple splicing 2	Rbpms2	206	22.4	8.41	67980.0	73289.9	74041.7	64400.6	86840.2	88468.0	66242.2	68865.0	85154.9	83773.0	51738.5	56552.4	59137.7	48361.8
Q8BPS4	Integral membrane protein GPR180	Gpr180	441	48.9	7.8	47784.6	45528.6	50468.7	55649.7	44172.4	50454.9	48409.0	50872.6	47378.8	46918.0	84512.9	74420.5	73477.1	73209.2
Q62276	Mediator of RNA polymerase II transcription subunit 22	Med22	200	22.3	4.68	88355.3	89744.9	89312.4	93458.3	85688.7	91313.4	80682.5	72971.7	93714.9	93677.7	#N/A	#N/A	#N/A	#N/A
Q9WV85	Nucleoside diphosphate kinase 3	Nme3	169	19.1	6.68	164357.8	172416.6	155427.1	173985.2	162498.5	163830.2	157800.5	143496.6	168137.5	164753.3	269070.6	263294.6	241743.7	273709.8
Q9EQQ0	Histone-lysine N-methyltransferase SUV39H2	Suv39h2	477	54.1	9.04	81645.9	86792.9	77462.8	80970.0	84587.1	90456.1	75545.9	74238.3	76719.5	84041.3	180303.2	166016.9	148424.6	167045.2
Q9CQ02	COMM domain-containing protein 4	Commd4	199	21.8	6.67	68683.9	81740.2	62803.6	73687.9	75693.8	78499.5	72869.9	66655.0	68136.2	76365.4	36377.1	46511.3	29143.3	32024.8
O08664	B-cell CLL/lymphoma 7 protein family member C	Bcl7c	217	23.4	5.2	59739.2	49496.5	58251.7	54437.4	56873.9	70276.9	56673.6	56550.5	58902.9	59157.7	81449.2	86505.0	91596.6	85407.2
O35566	CD151 antigen	Cd151	253	28.2	7.47	109277.2	113993.8	104859.0	106840.7	89457.8	99585.8	87714.0	111510.2	107720.9	99911.9	193081.5	155927.9	110710.5	122836.5
Q9DB90	Protein SMG9	Smg9	520	57.6	7.01	69360.8	79670.8	85926.1	87349.1	77965.8	68564.8	65556.8	73874.7	79900.4	81397.4	383694.1	440753.6	404689.1	453288.2
P33609	DNA polymerase alpha catalytic subunit	Pola1	1465	167.2	5.74	75912.0	73215.7	65143.8	64276.1	71076.3	67439.2	71697.8	66499.9	70112.6	77238.8	308208.0	343877.2	289960.2	322904.0
Q99JX1	Transcription initiation factor TFIID subunit 11	Taf11	211	23.3	5.24	94997.6	110856.9	128770.2	128361.7	108090.1	117356.9	104400.0	96223.8	108120.1	114594.5	#N/A	#N/A	#N/A	#N/A
P29268	CCN family member 2	Ccn2	348	37.8	7.81	91896.3	77802.3	90315.8	72544.4	175018.7	157137.6	100520.1	82378.7	102913.7	94351.7	#N/A	#N/A	#N/A	#N/A
Q91YL2	E3 ubiquitin-protein ligase RNF126	Rnf126	313	34.1	5.17	13354.0	16663.6	15966.4	18870.4	13353.9	12564.8	13175.9	10488.6	10606.1	15291.9	12431.8	14338.9	13362.7	15387.8
O88852	Testis-specific Y-encoded-like protein 1	Tspyl1	379	43	8.16	168220.8	169554.8	170679.4	152482.7	172321.7	181455.3	184156.8	158521.0	181202.1	168843.3	89546.7	81370.9	85890.4	78682.1
P04184	Thymidine kinase, cytosolic	Tk1	233	25.8	8.59	62193.2	66766.1	54434.8	58762.7	61167.3	59627.4	54826.7	56612.1	50765.0	49582.3	60123.9	53753.1	48388.9	51327.5
P54797	Transport and Golgi organization 2 homolog	Tango2	276	30.9	5.1	15532.9	13792.0	18464.2	14001.2	17247.5	16323.0	17723.9	15374.8	13402.3	14494.2	97242.3	96803.3	96340.7	97447.1
Q9WVF8	Tumor suppressor candidate 2	Tusc2	110	12.1	9.69	36800.8	42979.3	38362.3	33359.9	43556.5	43253.9	36422.6	33741.6	38369.2	35402.9	84740.0	92468.2	80034.3	83727.5
P62046	Leucine-rich repeat and calponin homology domain-containing protein 1	Lrch1	709	79	6.13	53507.6	58724.3	55221.5	53629.2	60422.0	72573.9	55180.1	55781.4	51862.2	52440.3	57469.2	48579.3	54911.5	61746.0
Q8K273	Membrane magnesium transporter 1	Mmgt1	131	14.7	9.16	289753.7	328272.2	322959.6	321129.8	319246.0	354890.4	306771.8	296368.4	370134.6	335615.8	164662.0	163583.4	176697.6	160006.6
Q8K4S1	1-phosphatidylinositol 4,5-bisphosphate phosphodiesterase epsilon-1	Plce1	2282	254.9	6.15	61916.7	71876.2	71821.0	66173.9	69577.5	69884.1	65456.8	61949.4	64491.6	69257.9	#N/A	#N/A	#N/A	#N/A
Q99N19	Cytochrome P450 CYP4F13	Cyp4f13	523	59.8	7.69	123022.3	129070.6	101090.3	119843.8	109078.9	119376.3	131117.0	122377.0	117731.8	122025.6	121586.4	141381.7	113047.2	137500.2
D3YXK1	Atherin	Samd1	519	54.5	7.47	239186.8	249137.7	242296.2	276692.8	222701.8	245163.5	217662.7	231744.3	249600.8	244187.1	149185.3	139119.7	114584.8	137633.0
D3Z3L3	Tripartite motif-containing 12C	Trim12c	497	57.8	6.95	17588.7	11867.3	9905.0	13305.8	11275.7	18678.7	16009.3	9783.1	15050.8	16901.1	60843.0	60356.7	62207.1	57594.5
Q78YY6	DnaJ homolog subfamily C member 15	Dnajc15	149	15.9	9.99	150985.4	263402.5	169447.9	190156.0	177290.3	176146.1	125688.9	185946.6	183334.0	201010.1	157110.6	225975.8	211352.4	219395.8
Q8BSP2	Condensin-2 complex subunit H2	Ncaph2	607	68.9	4.67	59485.0	68758.7	59548.2	41278.2	71273.1	71177.0	60101.1	50937.2	52235.5	59946.3	87499.1	96039.2	85534.2	84277.0
Q8BHX1	HAUS augmin-like complex subunit 1	Haus1	278	31.4	5.69	34172.4	44741.4	37329.2	37044.1	39464.3	32070.5	36478.3	31956.3	36059.2	37139.9	120857.5	166950.4	178776.7	203737.2

Q8BX57	PX domain-containing protein kinase-like protein	Pxk	582	65.2	9.38	8687.7	11510.1	9462.4	7487.2	7865.2	8869.0	8335.9	7544.5	11110.9	6918.4	27366.4	29445.6	28261.5	25697.5
P0C7N9	Proteasome assembly chaperone 4	Psmg4	123	14	6.15	39171.9	44248.4	39344.5	36680.9	35417.6	35727.5	36163.6	38355.7	41003.5	39340.6	#N/A	#N/A	#N/A	#N/A
F8VPZ5	DNA excision repair protein ERCC-6	Ercc6	1481	165.9	7.97	195285.9	181612.2	173896.0	200572.8	173181.9	165565.5	167744.8	178336.8	170419.0	179787.0	157660.2	142059.8	134904.3	141555.7
O35615	Zinc finger protein ZFPM1	Zfpm1	995	105.9	6.89	43919.0	45313.6	49936.0	45217.8	48660.5	50709.5	47007.5	34926.0	37951.2	44804.6	#N/A	#N/A	#N/A	#N/A
Q99JY4	TraB domain-containing protein	Trabd	376	42.2	8.41	126185.3	119909.7	106529.5	112512.9	122371.5	116875.0	123927.7	103975.5	113296.0	127428.8	97256.2	106276.2	96302.2	110400.3
O88668	Protein CREG1	Creg1	220	24.4	6.42	154485.6	167716.6	157415.6	168755.1	180695.9	171369.9	232665.9	162023.3	173550.6	170666.2	146052.3	159826.3	141501.1	159484.2
Q9CR80	Protein FAM32A	Fam32a	112	13.2	9.94	77189.5	71687.9	69018.9	80377.9	71791.6	75722.3	79917.8	64414.1	86139.3	81052.9	133042.7	133928.5	133952.2	111806.1
P11688	Integrin alpha-5	Itga5	1053	115	5.95	58722.8	63097.4	67454.0	60586.1	77418.7	87848.2	82537.8	74971.4	70614.2	67822.5	210504.6	218340.1	186582.4	191302.1
Q99PQ2	E3 ubiquitin-protein ligase TRIM11	Trim11	467	52.5	5.31	93103.3	120382.3	102915.2	106113.3	103605.9	112975.4	99349.0	103637.1	100413.3	102710.9	#N/A	#N/A	#N/A	#N/A
Q07832	Serine/threonine-protein kinase PLK1	Plk1	603	68.3	8.72	108108.2	138178.7	135732.2	146407.6	120110.4	143394.1	128241.3	105163.1	142630.3	142010.8	#N/A	#N/A	#N/A	#N/A
Q8K3I8	Beta-defensin 19	Defb19	83	9.6	8.72	40286.0	44614.6	34460.7	38131.1	22119.8	20015.0	21371.8	38388.0	26284.1	27899.3	339954.0	315751.0	261072.9	310722.2
Q8BXK8	Arf-GAP with GTPase, ANK repeat and PH domain-containing protein 1	Agap1	857	94.4	7.94	93952.7	106522.3	106966.8	104518.1	115167.8	107664.6	99658.2	97869.9	117094.6	104746.4	66405.1	67005.9	66450.7	66873.3
O35972	39S ribosomal protein L23, mitochondrial	Mrpl23	146	17.1	9.76	143053.5	144270.8	120822.3	116350.4	126062.6	135713.0	133223.1	138246.2	124297.6	127431.1	21448.2	18390.7	18050.2	18502.5
Q3UFY8	tRNA methyltransferase 10 homolog C	Trmt10c	414	48.4	9.38	31237.0	33680.5	25672.5	32375.3	31489.7	27319.3	33613.4	29695.1	30307.4	32947.5	130901.4	135831.9	117859.6	132102.7
Q8R5C8	Zinc finger MYND domain-containing protein 11	Zmynd11	602	70.8	8.56	171209.0	152386.0	150566.4	162315.2	172914.1	175938.9	168426.0	142018.6	185111.3	177180.4	#N/A	#N/A	#N/A	#N/A
Q9Z2V6	Histone deacetylase 5	Hdac5	1113	120.9	6.2	569945.4	551778.4	546235.1	544811.5	612243.0	610317.2	612414.1	528800.7	551543.5	558714.1	185231.9	168159.0	170878.1	155083.2
Q8VD72	Tetratricopeptide repeat protein 8	Ttc8	515	58.4	6.92	75142.6	99062.5	88717.7	84413.8	89011.2	79579.1	80003.2	82288.9	77309.9	87078.1	68773.0	71196.6	63237.3	73552.5
Q811S7	Upstream-binding protein 1	Ubp1	540	60.2	6.27	112143.2	105240.4	95317.4	107246.4	96609.6	111675.0	108382.0	104901.2	106112.7	101844.2	119520.8	122613.1	103220.5	111816.2
Q3U1C6	Putative deoxyribonuclease TATDN3	Tatdn3	294	32.4	6.87	64613.3	75377.4	68898.7	77799.9	74603.9	69064.7	68216.4	58412.6	75527.4	78707.8	82812.4	83755.9	70934.5	85319.8
Q9WTV7	E3 ubiquitin-protein ligase RLIM	Rlim	600	66.3	6.9	73783.5	62849.5	67330.9	65535.4	74231.8	71865.3	74527.3	68463.8	71273.3	61813.3	#N/A	#N/A	#N/A	#N/A
Q8VCB2	Mediator of RNA polymerase II transcription subunit 25	Med25	745	78.1	8.34	73464.1	86481.4	75844.9	72477.4	83711.8	75517.6	72307.8	73294.8	79156.4	84438.1	134808.4	138381.0	120718.3	123230.2
Q921Z5	Tumor necrosis factor alpha-induced protein 8	Tnfaip8	198	22.9	7.93	21595.9	21501.2	15258.5	20612.0	21637.0	28608.9	20304.0	20724.4	24277.3	21722.1	213182.6	214726.7	206070.8	185220.6
Q810U4	Neuronal cell adhesion molecule	Nrcam	1256	138.4	5.91	190778.0	193194.1	197473.0	168867.1	190997.6	198210.6	188019.9	212239.4	191231.2	178410.5	54696.8	44239.6	48443.3	44508.7
A0A087WQ44	Snf2-related CREBBP activator protein	Srcap	3271	349.1	5.72	112303.8	124149.6	106418.4	125279.0	115490.7	108517.8	106057.5	100058.5	126274.7	130039.1	#N/A	#N/A	#N/A	#N/A
Q9CQW7	Cytochrome c oxidase assembly factor 8	Coa8	192	22.7	10.13	22893.5	29051.8	28220.2	23086.4	21485.8	22529.2	24714.7	25752.3	29551.1	27933.4	30920.1	30791.4	30286.2	35797.6
Q8BTE0	Succinate dehydrogenase assembly factor 4, mitochondrial	Sdhaf4	104	11.9	9.5	62281.0	74847.6	79596.1	77239.5	83790.0	67072.9	62882.4	78142.4	81401.2	73255.6	43730.9	58170.5	56451.8	53997.2
Q8R0J7	Vacuolar protein sorting-associated protein 37B	Vps37b	285	31	7.05	162774.0	164605.7	160107.4	154374.9	173358.1	168040.1	180659.0	155718.7	151286.7	165758.0	33459.6	40335.7	33691.9	34042.9
Q8K003	Translation machinery-associated protein 7	Tma7	64	7.1	9.99	1158557.3	995500.3	1101345.5	1020869.1	1029189.9	1048859.8	1117326.1	1143602.6	1095730.2	1026815.8	2159437.2	1946723.4	2047457.0	1882396.2
Q9ESJ4	NCK-interacting protein with SH3 domain	Nckipsd	714	78.5	6.05	144270.7	144731.8	118644.8	140136.5	122419.4	144303.9	128221.5	124878.9	128087.1	142509.2	#N/A	#N/A	#N/A	#N/A
P62892	60S ribosomal protein L39	Rpl39	51	6.4	12.56	986808.2	1117035.3	877438.8	964956.8	935989.2	879481.7	910685.8	960160.1	924435.9	942081.0	904647.8	898748.9	728033.7	821738.9
B1AXD8	Akirin-2	Akirin2	201	22.1	8.87	123611.9	151635.5	128328.5	152643.5	117117.1	127289.9	108632.5	130813.7	121145.5	117805.0	#N/A	#N/A	#N/A	#N/A
Q9D0F4	NF-kappa-B-activating protein	Nkap	415	47.2	10.13	76560.3	85021.8	77752.8	81028.9	77591.1	74443.2	63897.4	67943.3	81241.4	74942.2	30073.4	30943.8	35145.1	27672.9
Q6NVG5	Melanoregulin	Mreg	214	25	7.2	67060.1	70852.2	71312.4	72688.4	58722.0	57714.8	45345.9	54330.2	65795.5	70131.8	#N/A	#N/A	#N/A	#N/A
Q0GGX2	Zinc finger protein 541	Znf541	1363	148.2	7.42	96406.2	102618.1	106412.1	106426.6	96920.9	86677.0	82128.1	82725.3	99632.7	94830.8	54382.9	73131.2	65011.4	68670.9
Q80XR2	Calcium-transporting ATPase type 2C member 1	Atp2c1	918	100.2	6.83	11201.1	14235.6	20043.3	19049.3	16408.5	18465.6	16352.4	13237.8	19374.8	10748.8	31105.5	31875.5	35208.1	29365.8
Q91XS1	Myotubularin-related protein 4	Mtmr4	1190	132.8	6.19	22582.5	19817.7	20773.1	21886.7	20582.1	21642.0	18377.1	19853.2	18333.3	19463.6	#N/A	#N/A	#N/A	#N/A
O35980	Endonuclease III-like protein 1	Nthl1	300	33.6	9.61	54043.4	57181.1	57650.5	65394.0	60453.2	65859.6	58495.8	57038.7	59131.0	57593.0	39146.5	44028.9	39274.4	40113.1
O70401	Tetraspanin-6	Tspan6	245	27.3	7.74	149434.2	138589.5	147838.2	120173.0	165286.2	171115.8	160857.4	163765.0	141054.3	139918.6	#N/A	#N/A	#N/A	#N/A
Q7TNH6	Nephrocystin-3	Nphp3	1325	150.2	6.51	23200.6	26090.8	24159.5	31886.3	23924.7	20433.9	20792.2	20875.0	20022.5	26988.8	21589.2	22772.2	25071.4	30353.1
Q99KX1	Myeloid leukemia factor 2	Mlf2	247	28	6.98	47011.6	48200.3	49547.5	51083.7	48505.1	54160.1	43362.0	45987.5	40620.0	43715.5	60035.5	66738.5	69604.5	75351.0
Q8VCS0	N-acetylmuramoyl-L-alanine amidase	Pglyrp2	530	57.7	6.98	202705.6	219507.1	224380.6	226426.4	208421.6	197897.9	172918.8	215731.5	205012.6	206916.7	171211.0	166838.2	171770.6	178130.6
Q8BGM4	Methyltransf_25 domain-containing protein	Mettl27	238	25.8	5.01	281880.2	269814.4	232335.7	282857.3	255427.2	259500.1	280923.1	243009.9	276279.1	279872.1	166199.6	163811.7	169522.7	162645.6
Q9CR25	2-(3-amino-3-carboxypropyl)histidine synthase subunit 2	Dph2	489	52.3	5.53	344946.7	347966.0	259309.2	311434.4	290694.8	296034.0	295734.2	261433.7	333132.6	341989.0	#N/A	#N/A	#N/A	#N/A
Q80V03	Uncharacterized aarF domain-containing protein kinase 5	Adck5	582	66.6	8.76	142549.6	124006.8	125659.4	129636.5	124561.7	126908.9	135932.6	124407.2	132361.9	131369.2	56576.5	73428.7	52603.0	74285.6
Q60590	Alpha-1-acid glycoprotein 1	Orm1	207	23.9	5.85	231260.9	250088.2	252696.4	273943.1	212906.4	204471.2	309092.0	186706.7	321023.5	350430.6	130224.0	185858.2	200818.9	187720.4
Q91ZU1	Ankyrin repeat and SOCS box protein 6	Asb6	418	46.2	5.78	21590.1	22096.1	22410.0	25022.3	24570.2	21608.5	17019.8	18469.5	22598.1	25161.6	#N/A	#N/A	#N/A	#N/A
A2A5Z6	E3 ubiquitin-protein ligase SMURF2	Smurf2	748	86.1	7.96	111584.3	124695.2	107182.4	125781.1	116708.9	119307.3	105909.8	110728.1	119409.9	115625.4	52291.0	61441.3	66714.9	54259.8
Q8K2H1	Periphilin-1	Pphln1	381	43.8	8.54	307537.3	314814.1	295471.6	350661.6	314004.6	318581.6	309007.3	259389.8	343082.2	328334.8	438418.7	451202.9	465214.7	453771.7
P01872	Immunoglobulin heavy constant mu	Ighm	454	49.9	7.01	134482.7	163159.3	103917.6	170230.1	91040.7	119991.7	137584.3	132625.1	107175.5	110549.0	47795.2	37686.4	28825.2	31982.3
Q4ZGD8	Nuclear RNA export factor 2	Nxf2	691	80.6	6.65	127479.4	134467.4	118207.2	137664.8	127700.7	125605.7	123763.8	129712.8	127159.6	145579.4	#N/A	#N/A	#N/A	#N/A
Q7TQE6	Macoilin	Maco1	664	76	9.07	45915.1	46902.1	49850.1	42858.9	51465.2	42090.9	45524.5	41811.6	46382.3	47265.1	28744.9	28777.7	36666.4	31739.5
Q9JKV5	Secretory carrier-associated membrane protein 4	Scamp4	230	25.3	8.66	28042.8	32092.2	30123.5	31118.2	28696.7	31128.6	28412.7	28640.0	33446.5	33963.5	429358.2	485845.8	457473.3	483332.9
Q8K182	Complement component C8 alpha chain	C8a	587	66	6.54	40914.4	56586.5	40955.7	52717.2	37548.5	40947.0	40747.9	36790.0	44434.8	45563.2	91357.9	109526.0	68919.5	84902.0
Q9CX30	Protein YIF1B	Yif1b	311	34	9.14	12339.9	14646.8	14055.3	15474.7	8753.9	13362.2	13820.6	16057.0	12753.6	14206.3	#N/A	#N/A	#N/A	#N/A
Q8BJQ2	Ubiquitin carboxyl-terminal hydrolase 1	Usp1	784	87.4	5.41	38066.6	41648.1	52293.1	49498.4	42927.6	39130.9	38437.1	45981.1	38905.4	52681.7	53506.7	74575.1	58794.1	66332.9
P97352	Protein S100-A13	S100a13	98	11.2	6.13	39551.8	54768.2	44814.0	54400.6	54555.7	59493.8	59680.5	62592.7	52701.2	37731.5	83598.0	107255.5	98601.8	133329.6
O89103	Complement component C1q receptor	Cd93	644	69.3	5.07	55509.1	71576.3	45431.0	57519.5	55022.1	56788.1	55573.0	57758.8	47028.4	58553.3	206350.4	248552.3	198043.6	222279.0
Q91VF2	Histamine N-methyltransferase	Hnmt	295	33.6	5.06	202131.3	188493.3	201560.8	184924.6	232812.4	209195.2	254956.0	211309.7	214703.6	217253.1	84202.9	71944.6	83240.3	81463.7
Q7TMF2	3'-5' exoribonuclease 1	Eri1	345	39.5	8.31	160251.8	178051.2	155082.2	179148.0	174557.2	198893.1	150352.3	143493.4	167222.1	160949.7	371881.2	428215.9	379566.2	380436.6
Q9CQ79	Thioredoxin domain-containing protein 9	Txndc9	226	26.2	5.95	272049.1	360236.6	322868.9	293708.6	278804.5	344448.4	253434.5	277178.2	286831.6	264550.1	131076.3	142003.6	129415.4	135768.0
Q9CR64	Protein kish-A	Tmem167a	72	8.1	8.95	866819.1	821972.8	801456.1	857752.9	794343.7	683383.9	623540.7	832969.4	775695.6	742806.7	614716.0	637575.3	569284.8	682807.2
O08734	Bcl-2 homologous antagonist/killer	Bak1	209	23.3	6.18	30777.1	27643.6	18749.1	26017.8	29891.1	28601.7	26254.1	30420.7	27304.8	28009.0	144481.8	141392.4	136233.5	151456.9
P50429	Arylsulfatase B	Arsb	534	59.6	7.24	238316.9	248545.3	203516.9	226601.4	221861.5	234821.8	263545.0	236301.9	220233.7	213827.4	#N/A	#N/A	#N/A	#N/A
P58043	Sestrin-2	Sesn2	480	54.3	6.02	44479.2	42854.8	43293.0	42283.7	40751.6	42469.7	38859.8	39979.2	47474.4	36628.5	203458.2	193848.9	185492.2	189017.0
Q9CWX2	Complex I intermediate-associated protein 30, mitochondrial	Ndufaf1	328	37.8	8.6	160828.4	176897.0	176944.6	153599.4	181401.0	166192.2	151183.4	162397.9	161704.1	164153.6	142269.9	156159.0	148728.9	156213.9
Q9CQZ7	DNA-directed RNA polymerase III subunit RPC10	Polr3k	108	12.3	7.83	64548.0	66049.0	60565.6	64057.9	61259.3	63265.5	61635.7	67228.0	69102.0	61343.7	#N/A	#N/A	#N/A	#N/A
Q8BGW5	SOSS complex subunit B2	Nabp1	198	21.7	9.39	125454.4	138712.9	115500.3	130967.1	118278.4	126855.1	118562.0	122627.8	120067.1	144278.3	390708.6	400583.6	368856.5	371673.8
Q8CBQ5	Phosphatidylinositol 4-kinase type 2-beta	Pi4k2b	469	53.4	6.8	48690.9	55329.2	54267.6	50249.3	56050.0	72079.0	50344.8	51170.1	48054.8	53176.7	114648.9	114375.7	95642.3	101646.3
Q99LH2	Phosphatidylserine synthase 1	Ptdss1	473	55.6	8.28	85344.5	92130.7	92766.2	91864.8	91558.8	97916.8	86806.6	97417.3	95794.7	89637.5	114757.6	117798.5	123852.6	104838.8
Q3U155	Coiled-coil domain-containing protein 174	Ccdc174	467	53.9	6.21	55415.2	56399.3	57104.9	59737.6	63164.0	59501.3	54916.0	60890.4	56701.2	55973.1	91669.4	106585.5	100423.9	107105.0
Q8CFV9	Riboflavin kinase	Rfk	155	17.4	7.55	372947.9	367105.3	361055.2	366722.0	360585.8	376274.3	397553.8	383756.1	391120.8	378821.3	405901.0	434473.0	319218.8	373976.4
Q9CZE3	Ras-related protein Rab-32	Rab32	223	25.1	6.68	429264.9	438305.7	385406.1	412807.0	402388.2	420351.4	431476.8	385606.3	405644.1	412012.5	76683.9	66514.7	64816.7	67662.7
Q9Z0H7	B-cell lymphoma/leukemia 10	Bcl10	233	25.9	6.55	251934.2	244939.9	226163.2	231864.4	245726.1	248910.2	261937.2	216375.9	234238.5	254950.9	160974.2	182277.2	156767.8	160971.9
Q91XC9	Peroxisomal membrane protein PEX16	Pex16	336	38.7	9.72	172681.6	178716.8	170581.8	176829.6	188619.8	178104.8	167062.8	171334.7	163997.2	179488.4	110147.0	119095.8	123055.4	127808.1
Q9DCN1	Peroxisomal NADH pyrophosphatase NUDT12	Nudt12	462	51.5	7.12	100279.1	89382.1	100734.0	87646.2	109284.6	106810.1	112330.1	97484.1	98249.1	107195.2	55503.2	52613.7	44854.4	50138.0
Q8BVP6	Ly6/PLAUR domain-containing protein 4	Lypd4	246	27	7.66	114438.1	157566.4	131397.1	169849.6	114511.3	107795.8	105209.0	108683.8	127589.0	138273.3	#N/A	#N/A	#N/A	#N/A
Q8BU88	39S ribosomal protein L22, mitochondrial	Mrpl22	206	23.8	9.86	154803.2	150987.7	138094.8	142529.8	156116.6	147353.4	131747.2	139348.2	158752.5	149173.7	240253.7	247418.2	245447.3	254454.5
Q8CB27	Ubiquitin thioesterase OTU1	Yod1	343	37.5	5.76	41616.9	47419.4	41879.7	40792.4	43112.9	38745.9	38166.0	32532.5	41311.3	39782.6	13703.7	14587.6	14352.8	12637.9
Q5RJG7	D-ribitol-5-phosphate cytidylyltransferase	Crppa	447	49.1	6.4	43328.9	51336.0	33957.0	36794.2	41682.0	44778.6	47062.4	37911.0	43077.5	42319.9	54762.0	48716.0	43671.0	50373.7
Q80UU1	Ankyrin repeat and zinc finger domain-containing protein 1	Ankzf1	748	82.9	8.37	248836.7	259310.3	281022.1	279114.0	271796.8	267812.1	261060.0	252106.8	276053.9	277601.7	151960.3	156689.0	148218.2	153245.5
Q9D8L5	Coiled-coil domain-containing protein 91	Ccdc91	442	50	5.07	173688.6	168499.2	162643.2	174177.6	161583.2	179361.9	180187.5	164781.8	173799.0	169287.4	319944.1	307301.1	310501.9	287803.5
O70230	Zinc finger protein 143	Znf143	638	69	6.19	99625.6	100251.4	90613.9	83230.8	103805.2	103501.9	90817.6	88285.8	87758.8	88030.4	73436.7	76518.7	65850.3	59911.7
Q9D2H6	Transcription factor Sp2	Sp2	612	64.9	9.88	9575.4	8226.4	2953.3	3132.9	11292.6	3253.4	3236.4	9067.2	7842.8	3329.0	95810.6	96277.8	99603.1	105432.5
Q6ZQ06	Centrosomal protein of 162 kDa	Cep162	1403	160.8	5.53	73027.6	79580.2	73375.2	73117.0	75663.8	83605.2	74928.5	68769.8	75428.5	80195.1	#N/A	#N/A	#N/A	#N/A
Q9JIX0	Transcription and mRNA export factor ENY2	Eny2	101	11.5	9.33	281031.5	316209.6	305056.5	319086.3	337490.4	305840.3	263794.6	340892.3	603845.4	286937.7	1366398.3	1411486.3	1546618.3	1585928.9
P62311	U6 snRNA-associated Sm-like protein LSm3	Lsm3	102	11.8	4.7	227421.9	230565.2	228786.0	241330.8	250001.3	265038.0	252544.3	228882.7	244863.0	245399.2	#N/A	#N/A	#N/A	#N/A
Q9CXA2	Trans-L-3-hydroxyproline dehydratase	L3hypdh	354	37.8	6.7	192111.6	200551.2	176127.1	186422.0	190407.2	219164.5	241662.6	192395.8	197754.4	192991.9	190956.9	211434.6	186209.4	218238.0
Q922Q6	Protein ABHD14A	Abhd14a	247	27.8	8.81	50833.4	61248.5	57733.2	60088.9	55534.1	57628.3	54164.2	48106.0	55431.0	55683.5	#N/A	#N/A	#N/A	#N/A
Q6NZK5	Protein hinderin	Kiaa1328	612	69.7	8.57	166166.8	190998.8	195714.9	198746.1	179700.4	174359.8	139690.5	155123.6	182948.2	195622.5	125768.1	148405.2	122481.6	149307.7
Q6P3Z3	THAP domain-containing protein 4	Thap4	569	62.5	9.1	10003.0	9544.7	14704.4	12168.0	17895.9	15103.7	22212.2	16366.2	11169.7	11804.7	#N/A	#N/A	#N/A	#N/A
Q91YI4	Beta-arrestin-2	Arrb2	410	46.3	7.66	118881.8	121057.6	114609.4	109943.0	132029.7	127620.4	123893.3	125388.2	135103.1	131552.5	#N/A	#N/A	#N/A	#N/A
O70172	Phosphatidylinositol 5-phosphate 4-kinase type-2 alpha	Pip4k2a	405	46.1	6.99	40146.3	41397.1	43473.2	34848.0	38071.0	54625.9	42626.3	41631.6	38668.1	36169.9	29175.5	30430.5	28842.0	29084.8

Q8R3Y5	Uncharacterized protein C19orf47 homolog	MGI:191361	413	44.4	10.37	110725.0	91930.6	107968.0	101528.3	108103.8	103569.7	124428.2	98527.8	102423.2	105668.6	#N/A	#N/A	#N/A	#N/A
Q8VEB1	G protein-coupled receptor kinase 5	Grk5	590	67.7	8.19	64921.9	71978.6	103128.1	81914.3	72649.0	71551.2	64891.4	84099.3	83041.0	79092.4	31573.6	37725.0	38358.9	35156.4
A2AHA7	Preferentially-expressed antigen in melanoma-like 3	Pramel3	463	52.8	6.55	14562.0	15543.7	15699.7	14831.3	16294.4	12642.0	17416.6	16687.3	12409.4	18920.2	28139.9	35399.3	26060.2	27554.5
B2RY50	Armadillo repeat-containing protein 4	Armc4	1037	115.2	6.09	97252.2	116917.7	97654.1	148524.1	76278.3	82722.1	92287.6	83968.5	94687.0	97368.3	92563.5	116757.5	98648.9	133970.2
Q9CS74	Protein ecdysoneless homolog	Ecd	641	71.7	4.94	49263.2	55931.9	49256.1	51410.6	51602.0	51722.3	52576.7	48266.0	55848.2	54736.4	83252.1	81190.6	90265.0	88491.4
E9Q8I9	Protein furry homolog	Fry	3020	338.9	6.01	87607.3	81144.1	80268.1	78529.1	78120.9	77840.7	68507.2	75245.7	73556.4	73238.9	#N/A	#N/A	#N/A	#N/A
Q5ND52	rRNA methyltransferase 3, mitochondrial	Mrm3	418	46.8	9.39	147356.4	165379.2	145313.9	145559.7	136405.2	159754.2	129683.4	133282.1	145978.9	159367.3	#N/A	#N/A	#N/A	#N/A
Q9DCG2	CD302 antigen	Cd302	228	25.4	4.61	112700.7	110920.8	101808.1	108819.0	123391.0	118227.4	122560.4	103062.9	114151.4	123162.7	41313.1	42911.6	37045.2	36936.6
Q9D708	Protein S100-A16	S100a16	124	14.3	5.86	58734.7	62914.1	55543.2	78944.1	61788.7	63563.3	66766.3	90002.7	55206.0	56061.3	73317.2	76763.4	66551.4	76372.1
Q8C0C0	Zinc fingers and homeoboxes protein 2	Zhx2	836	92.2	7.46	51276.9	50915.3	59206.6	43386.3	52175.6	43088.1	59216.0	45865.8	51304.4	55421.5	66456.5	59206.1	62277.6	45242.7
Q9CZB0	Succinate dehydrogenase cytochrome b560 subunit, mitochondrial	Sdhc	169	18.4	9.94	478210.6	437290.1	431110.5	502555.7	392959.5	380765.9	390771.4	402942.0	427799.3	396017.4	113300.2	184134.3	121581.6	147621.3
Q8VIB3	Type 2 lactosamine alpha-2,3-sialyltransferase	St3gal6	329	37.8	9.16	112166.6	108968.1	100877.0	101634.9	106445.5	108561.4	101871.2	98997.2	108768.1	115121.5	163099.4	166328.3	157039.2	148746.6
Q9JKW0	ADP-ribosylation factor-like protein 6-interacting protein 1	Arl6ip1	203	23.4	9.32	468394.3	627740.7	607392.8	680615.3	502544.3	436923.2	425387.9	443982.4	552293.6	622456.1	359131.9	402268.2	508663.0	508967.0
Q99MB7	RING finger protein 141	Rnf141	230	25.5	5.2	99075.3	97858.9	103560.9	88030.4	102426.8	101124.7	85328.4	91572.5	94980.9	95591.3	79034.0	76372.6	82692.0	76410.2
O70139	cAMP-dependent protein kinase inhibitor gamma	Pkig	76	7.9	4.21	107357.1	115461.2	103084.6	107092.3	124652.3	118427.0	105390.3	103352.2	118612.3	115249.0	#N/A	#N/A	#N/A	#N/A
Q8VCM4	Lipoyltransferase 1, mitochondrial	Lipt1	373	42.1	8.48	66928.6	68154.8	68327.1	74416.9	61893.4	67668.0	61395.4	65165.4	66860.1	68586.9	137556.8	139005.7	139017.4	131304.6
Q61025	Intraflagellar transport protein 20 homolog	Ift20	132	15.2	5.12	195243.3	226448.9	179461.7	224940.7	216849.9	206809.2	205355.8	195033.6	215551.9	203855.8	#N/A	#N/A	#N/A	#N/A
Q8K2C8	Glycerol-3-phosphate acyltransferase 4	Gpat4	456	52.1	9.35	52432.8	44395.0	43409.9	43305.6	47967.9	48613.2	41560.1	164616.7	43495.1	40910.4	40521.4	41804.7	39959.8	38320.4
Q9D1I5	Methylmalonyl-CoA epimerase, mitochondrial	Mcee	178	19	9.09	91432.9	79484.2	79654.8	75751.4	88198.3	84189.7	95183.3	72654.9	84594.5	79318.1	182363.6	154901.7	157287.3	164970.1
Q921I2	Kelch domain-containing protein 4	Klhdc4	584	64.8	5.73	48983.8	50518.4	46596.6	44349.8	46019.4	48941.1	45415.8	46822.9	40177.9	45013.3	#N/A	#N/A	#N/A	#N/A
Q8CBA2	Schlafen family member 5	Slfn5	884	100.8	6.93	72560.9	76112.8	63764.3	71006.1	75810.6	83286.0	82025.0	75254.6	70833.5	70651.0	#N/A	#N/A	#N/A	#N/A
Q9Z1N2	Origin recognition complex subunit 1	Orc1	840	95	9.1	185301.5	184950.8	172724.9	194828.9	187202.5	179138.1	177168.2	278968.1	175393.9	190750.4	33194.5	32159.7	30266.1	30437.3
Q8R516	E3 ubiquitin-protein ligase MIB2	Mib2	973	105.9	8.09	91311.9	110894.3	105489.2	115011.6	115868.8	109841.7	110061.0	105612.0	118172.8	116066.2	#N/A	#N/A	#N/A	#N/A
Q8R3L8	Cyclin-dependent kinase 8	Cdk8	464	53.2	8.57	90971.4	101189.2	96313.8	87360.6	113116.0	116257.7	100131.5	103995.2	92887.6	99818.4	#N/A	#N/A	#N/A	#N/A
Q3TWL2	Type 1 phosphatidylinositol 4,5-bisphosphate 4-phosphatase	Pip4p1	284	30	8.82	172419.2	191718.2	153191.5	207103.0	170571.3	157160.0	160314.5	153995.6	170727.2	161427.6	38064.5	38432.0	32077.7	42524.8
Q9DBU0	Transmembrane 9 superfamily member 1	Tm9sf1	606	68.9	7.09	272509.5	267592.4	236667.9	261025.9	245398.5	237601.3	230132.5	243530.4	243350.1	236073.1	158507.5	157081.9	131626.7	143989.4
Q8K2Y0	ORC ubiquitin ligase 1	Obi1	722	79.9	6.33	48510.9	47081.7	55315.3	52524.9	45284.8	53391.3	52330.3	46525.7	62838.3	49839.6	42887.3	50151.6	49174.3	38810.5
Q9JJA7	Cyclin-L2	Ccnl2	518	58	10.18	21024.3	26652.1	23440.5	21690.1	29215.7	23925.7	18177.4	18706.2	28272.9	22280.4	#N/A	#N/A	#N/A	#N/A
Q5NCI0	Up-regulator of cell proliferation	Urgcp	926	104.6	6.32	33771.9	43355.3	33808.2	41753.3	41060.3	40957.9	41776.2	37189.8	33818.5	35833.9	#N/A	#N/A	#N/A	#N/A
Q9JHI2	tRNA-specific adenosine deaminase 1	Adat1	499	55.3	8.16	133244.7	144697.3	132131.2	136583.3	140128.4	141022.5	131236.9	126733.9	130536.1	143681.1	#N/A	#N/A	#N/A	#N/A
O35425	Bcl-2-related ovarian killer protein	Bok	213	23.4	9.11	93352.3	106312.4	99615.4	101634.9	102859.8	106996.1	92381.4	102328.8	94521.2	108962.3	184562.7	195546.0	162986.5	187991.4
P97784	Cryptochrome-1	Cry1	606	68	7.97	126028.6	147792.6	122890.5	131737.8	123201.0	158410.4	107896.7	116657.9	123990.8	150401.8	#N/A	#N/A	#N/A	#N/A
Q8K4E0	Alstrom syndrome protein 1 homolog	Alms1	3251	360	6.39	133837.7	170373.2	159017.8	170968.0	168954.4	165795.5	146588.0	132814.2	168808.9	169996.9	#N/A	#N/A	#N/A	#N/A
P30276	G2/mitotic-specific cyclin-B2	Ccnb2	398	45.4	8.92	175678.9	224254.6	201438.2	233248.0	189443.5	181970.6	161303.7	169117.1	210192.3	215652.2	45159.2	46540.9	51865.7	55176.5
Q99L28	Probable ribosome biogenesis protein RLP24	Rsl24d1	163	19.6	9.98	55835.1	54279.0	59561.3	58362.4	57719.5	56817.4	54780.2	62227.9	57081.6	52742.2	#N/A	#N/A	#N/A	#N/A
Q9EQP6	Arrestin-C	Arr3	381	41.9	6.44	124911.3	141178.3	133309.7	142457.8	132885.1	142359.9	159719.1	138328.3	132357.3	131927.9	#N/A	#N/A	#N/A	#N/A
Q9D8K3	Derlin-3	Derl3	228	26	8	38290.7	57378.0	50204.7	63951.9	58891.9	45656.6	48521.0	51274.8	47838.9	55359.2	42580.9	41757.7	43977.8	44750.7
A2AKB4	FERM and PDZ domain-containing protein 1	Frmpd1	1549	169.1	5.16	75834.5	84620.8	66051.3	74015.4	61305.6	66151.1	64661.3	62599.5	77645.3	68860.7	#N/A	#N/A	#N/A	#N/A
Q9CQT5	Proteasome maturation protein	Pomp	141	15.8	5.49	53925.5	72007.8	56704.1	51331.9	59028.5	58345.1	56422.0	54136.3	61990.6	61806.2	105206.1	101044.7	118905.1	110130.8
Q99PM3	Transcription initiation factor IIA subunit 1	Gtf2a1	378	41.6	4.55	525431.1	605719.4	624529.8	632117.9	630421.8	616889.5	622053.6	568170.1	636237.1	618327.0	427690.0	432548.0	430014.5	429112.0
Q9DB60	Prostamide/prostaglandin F synthase	Prxl2b	201	21.7	6.74	160249.7	154317.4	124380.2	149680.7	159188.3	168137.3	175625.7	161791.9	148836.8	155187.8	149253.7	150547.1	123895.2	137157.9
P70426	GTP-binding protein Rit1	Rit1	219	25.2	9.2	59234.4	61990.5	61682.8	64987.2	57782.8	65038.1	53130.4	54038.8	64908.5	66735.1	#N/A	#N/A	#N/A	#N/A
Q8CIE4	Protein mono-ADP-ribosyltransferase PARP10	Parp10	960	103.6	5.02	65633.8	68018.4	56512.8	64575.7	69646.9	68162.3	70753.2	61896.6	69583.5	77868.2	#N/A	#N/A	#N/A	#N/A
Q3TEI4	Uncharacterized protein C15orf39 homolog	MGI:192146	1023	108.2	8.25	89174.1	88975.4	89460.5	87285.2	89419.7	82699.8	88712.0	80043.4	101840.3	97663.3	#N/A	#N/A	#N/A	#N/A
Q8BHC0	Lymphatic vessel endothelial hyaluronic acid receptor 1	Lyve1	318	34.6	8.03	53842.7	47175.2	55870.1	37940.1	67551.0	109137.6	133287.0	81799.3	70003.4	45649.2	#N/A	#N/A	#N/A	#N/A
P58158	Galactosylgalactosylxylosylprotein 3-beta-glucuronosyltransferase 3	B3gat3	335	37	8.78	102625.5	99777.9	90747.5	92471.7	92799.8	94394.2	89425.9	88121.9	93270.8	89869.1	41497.5	41884.8	37366.9	37457.4
Q91WD0	Protein GPR108	Gpr108	569	63.9	7.56	31347.4	30402.9	28259.9	27952.4	24863.1	31708.1	25263.2	23448.0	24249.2	25452.3	91034.2	89928.6	82609.1	85806.5
P07091	Protein S100-A4	S100a4	101	11.7	5.31	189877.7	228286.9	169778.9	227804.2	185587.6	216188.1	222483.0	217351.2	197096.7	232573.1	#N/A	#N/A	#N/A	#N/A
A2AMW4	MAGE domain-containing protein	Magea10	325	35.8	4.74	255597.5	288285.6	289674.7	250504.3	293973.4	287383.6	242499.6	258356.7	281567.5	307497.7	#N/A	#N/A	#N/A	#N/A
Q8R2R1	Protein O-mannosyl-transferase 1	Pomt1	746	85.2	8.29	189772.9	217592.7	200065.3	219558.7	171197.2	164286.3	158763.5	184322.2	196288.2	214964.3	185778.9	252448.7	230154.0	270916.3
Q9DCA2	28S ribosomal protein S11, mitochondrial	Mrps11	191	20.2	10.77	97222.0	82781.4	82354.9	87123.7	72214.8	82023.0	75352.0	84814.2	86513.1	79426.1	234744.7	218760.0	211832.5	229184.6
Q9DAM9	Fibronectin type 3 and ankyrin repeat domains 1 protein	Fank1	344	38.2	7.43	74160.6	85738.9	72041.1	99977.4	58275.2	61468.0	60000.3	54736.4	70837.5	77539.5	79630.1	94407.4	97370.2	122481.5
A2AJT4	Arginine/serine-rich protein PNISR	Pnisr	805	92.1	10.01	236628.5	244813.9	301660.8	231725.5	280010.5	254019.9	251655.5	233505.1	245821.7	254642.2	190507.9	187227.5	221935.5	181040.8
Q91WD7	Kinesin-like protein KIF18A	Kif18a	886	100.9	9.04	113337.6	114871.2	120181.1	110385.6	121600.1	142082.9	114394.5	110334.2	114956.0	126045.0	290552.6	284619.6	286163.4	283168.9
Q99LI9	Polyribonucleotide 5'-hydroxyl-kinase Clp1	Clp1	425	47.7	6.62	159258.4	199626.8	202173.7	196053.2	208957.7	216044.3	179920.0	180345.7	219805.5	196354.1	342569.8	345059.5	322485.1	325942.8
Q62011	Podoplanin	Pdpn	172	18.2	5.53	59427.7	47123.0	54230.5	40418.9	72153.9	90881.1	107992.3	71107.2	61306.1	55366.4	#N/A	#N/A	#N/A	#N/A
Q3V124	EP300-interacting inhibitor of differentiation 3	Eid3	375	43.3	4.87	22196.7	24159.8	18540.3	27340.1	21189.6	21517.6	12671.2	16517.2	16038.1	22561.9	39898.6	73172.8	40381.0	55803.8
Q8R121	Protein Z-dependent protease inhibitor	Serpina10	448	51.8	5.67	39608.0	40589.9	29016.5	35498.0	32107.8	35407.6	36422.7	27322.2	33338.7	36289.1	42733.1	45287.8	28351.3	34250.1
Q9CRA0	Ecto-ADP-ribosyltransferase 4	Art4	300	33.1	8.47	74425.1	80954.3	60247.7	67168.0	70284.9	71595.9	81524.0	62489.7	67637.8	71701.7	#N/A	#N/A	#N/A	#N/A
P48771	Cytochrome c oxidase subunit 7A2, mitochondrial	Cox7a2	83	9.3	10.27	278620.9	270596.4	296073.8	298708.3	295071.8	250894.8	281368.9	269457.1	275435.1	266422.7	348190.9	314495.5	377480.0	383370.9
A2A9C3	KICSTOR complex protein SZT2	Szt2	3431	377.4	6.19	5234.4	4933.1	3751.6	1587.8	3299.8	1569.9	4546.2	1568.2	1846.4	3938.1	#N/A	#N/A	#N/A	#N/A
Q9CPZ6	ORM1-like protein 3	Ormdl3	153	17.5	9.64	80606.1	66024.5	87493.0	72664.6	91470.4	88319.9	77815.9	87330.3	84265.7	67019.3	#N/A	#N/A	#N/A	#N/A
Q8CGI1	Protein FAM193A	Fam193a	1231	136.6	6.47	42668.6	43092.9	43647.2	44692.8	47476.1	52628.4	48360.9	41971.0	51871.7	43381.5	49498.9	56922.1	62329.1	64591.5
Q8BMQ8	Vacuolar fusion protein MON1 homolog B	Mon1b	553	60	5.57	100490.9	108674.5	103488.0	107381.9	109704.0	101654.9	93335.8	99413.9	108783.9	107174.7	#N/A	#N/A	#N/A	#N/A
Q8VD26	Transmembrane protein 143	Tmem143	458	51.6	9.26	57770.7	54764.7	47569.2	58666.1	57345.7	56060.1	52753.2	52991.9	52011.6	55379.7	#N/A	#N/A	#N/A	#N/A
P70419	Polypeptide N-acetylgalactosaminyltransferase 3	Galnt3	633	72.9	8.25	41789.4	50802.5	50408.8	76149.0	37578.4	38416.0	32402.2	44641.7	42852.1	46347.6	92545.3	111355.4	78563.5	163011.8
P15920	V-type proton ATPase 116 kDa subunit a isoform 2	Atp6v0a2	856	98.1	6.64	135288.5	135472.9	139396.3	123315.8	132496.2	126630.2	114653.7	130221.0	124780.4	129762.3	51837.6	46333.2	42862.9	44802.2
Q9CPU4	Microsomal glutathione S-transferase 3	Mgst3	153	16.9	9.5	130300.9	125702.4	95891.7	119799.0	109693.5	99811.8	111934.7	102624.1	110870.6	122418.8	155750.3	171559.0	169455.4	156301.1
Q60870	Receptor expression-enhancing protein 5	Reep5	185	21	8.38	92833.1	76252.1	81633.8	88246.3	70810.6	72369.8	75196.0	89078.1	69524.6	71330.7	594985.8	565135.0	472998.5	558553.1
Q9D1H8	39S ribosomal protein L53, mitochondrial	Mrpl53	118	12.7	9.52	215513.5	195252.4	244390.2	211723.4	216740.2	197058.1	190610.4	209438.1	220549.6	216835.2	302601.8	258277.7	269203.6	266222.5
Q6DFV7	Nuclear receptor coactivator 7	Ncoa7	943	106.3	5.43	314874.9	329183.3	273434.5	311878.3	305487.7	288032.1	288930.5	301800.7	300965.1	308860.1	145973.9	127369.4	89334.2	108024.1
Q922T2	Microfibril-associated glycoprotein 3	Mfap3	349	38.4	5.33	130408.3	130869.2	124073.9	122467.2	136469.0	125485.4	127678.4	123437.0	121269.6	144139.2	89478.0	99148.7	87468.6	93655.7
A0A075B680	Ig-like domain-containing protein	Ighv1-62-2	100	11.2	8.43	25479.3	37319.2	24094.8	31483.3	64888.2	31146.9	21487.7	23623.6	27251.0	27372.9	#N/A	#N/A	#N/A	#N/A
B2RQE8	Rho GTPase-activating protein 42	Arhgap42	841	94.6	8	67610.4	72793.1	70572.6	65459.9	84495.2	91822.1	85379.1	69576.2	74007.7	76720.6	#N/A	#N/A	#N/A	#N/A
Q8BSF4	Phosphatidylserine decarboxylase proenzyme, mitochondrial	Pisd	406	45.9	9.57	358007.0	390543.4	386390.3	402077.3	388289.7	648580.0	336543.8	341545.9	375319.9	384319.0	#N/A	#N/A	#N/A	#N/A
Q61908	Cx9C motif-containing protein 4	Cmc4	68	7.7	8.38	86292.4	97461.0	104258.1	100251.3	98585.1	102170.0	98219.6	95873.5	115475.7	99311.9	#N/A	#N/A	#N/A	#N/A
Q8K3A0	Iron-sulfur cluster co-chaperone protein HscB	Hscb	234	26.6	7.37	122207.4	145373.9	199102.4	136916.7	160863.1	158326.3	148296.1	144502.2	124119.3	146304.8	204141.3	183754.1	187046.8	200277.6
Q8VEA8	Ras-related protein Rab-7b	Rab7b	199	22.5	6.21	123484.2	122966.7	120515.8	144350.9	138302.4	152765.2	125973.8	121164.5	125084.3	129259.8	39744.4	40441.8	42007.8	38141.8
Q9Z2X2	26S proteasome non-ATPase regulatory subunit 10	Psmd10	231	25.1	6.06	227523.5	221103.2	206813.5	223114.7	240587.3	212035.2	218522.0	216251.8	216444.7	231512.5	349428.7	352653.4	350533.1	375267.6
Q8VEH2	Matrin-type domain-containing protein	Ciz1	845	93.5	5.43	22258.4	23645.0	24406.4	24972.0	27042.4	23251.0	22712.8	22625.7	27123.4	22911.2	#N/A	#N/A	#N/A	#N/A
Q8C735	Protein lin-9 homolog	Lin9	542	61.7	9.03	19625.6	22457.6	23175.7	26369.5	19817.9	20167.4	17937.9	17239.0	19830.6	24819.0	137897.0	159786.9	155771.7	172815.0
Q9JHJ3	Glycosylated lysosomal membrane protein	Glmp	404	43.8	6.14	84854.7	98486.6	81492.9	83923.0	73446.9	78815.7	79682.4	71916.3	87922.3	94380.2	#N/A	#N/A	#N/A	#N/A
Q3UFK8	FERM domain-containing protein 8	Frmd8	466	51.8	6.44	61973.5	68776.2	72744.5	68062.8	68288.0	61348.7	77425.9	60606.8	64698.1	72645.2	102117.2	110553.8	102180.3	98850.3
Q9CY34	NEDD8-conjugating enzyme UBE2F	Ube2f	185	21.1	6.54	139308.0	142152.7	120158.9	147430.5	137503.4	169686.9	126157.9	121435.0	130439.2	116429.2	126802.7	134113.4	119047.0	136428.2
Q8K1L5	E3 ubiquitin-protein ligase PPP1R11	Ppp1r11	131	14.5	6.09	84492.4	87265.8	86390.3	94626.0	84976.0	79064.1	80814.8	85764.3	85810.2	87689.8	563868.0	587192.9	653529.0	656404.4
O35604	NPC intracellular cholesterol transporter 1	Npc1	1277	142.8	5.71	46389.9	46134.7	40967.3	38536.8	37889.7	50512.2	44480.4	42363.7	47943.2	43935.8	127639.1	138608.9	115860.4	119308.8
P00688	Pancreatic alpha-amylase	Amy2	508	57.3	7.28	74049.4	81436.3	56587.1	55513.2	79948.0	70469.6	83695.1	82890.8	64627.2	70801.2	#N/A	#N/A	#N/A	#N/A
Q99JV5	StAR-related lipid transfer protein 4	Stard4	224	25.6	5.67	9724.9	18422.0	8946.0	11525.7	9801.4	11546.6	10696.0	13678.8	10264.4	12725.0	#N/A	#N/A	#N/A	#N/A
P35295	Ras-related protein Rab-20	Rab20	233	26	5.77	81262.0	76622.6	69071.5	69337.9	83047.4	81790.3	72320.1	76212.4	87719.2	75247.0	#N/A	#N/A	#N/A	#N/A
Q8BJ34	Meiosis regulator and mRNA stability factor 1	Marf1	1730	191.9	8.22	81574.1	84198.6	89246.1	80044.2	96830.0	86973.6	78584.5	79136.4	86713.6	85948.9	#N/A	#N/A	#N/A	#N/A
Q68FH0	Plakophilin-4	Pkp4	1190	131.5	8.94	54082.9	58087.5	44368.0	51777.4	54204.4	60028.3	63794.2	52557.6	54754.5	57174.6	#N/A	#N/A	#N/A	#N/A
Q9CYK2	Glutaminyl-peptide cyclotransferase	Qpct	362	41.1	6.92	216240.1	216118.0	200094.7	201326.3	229842.6	225236.8	203409.8	205268.8	207593.9	224526.0	72868.8	103588.4	73910.4	82630.3

Q9CRC0	Vitamin K epoxide reductase complex subunit 1	Vkorc1	161	17.8	9.14	123927.7	142791.3	122712.4	132877.9	130187.3	147869.9	119276.0	134305.3	105927.6	132149.5	91623.4	164134.8	95291.5	123605.7
Q9CXK4	Sugar transporter SWEET1	Slc50a1	221	24.6	8.68	22291.8	20885.7	20178.2	24120.0	21560.4	21887.9	22033.1	23162.7	20574.5	17986.3	#N/A	#N/A	#N/A	#N/A
Q400C8	Archaemetzincin-2	Amz2	359	41.3	7.66	145356.2	176950.9	156079.0	170204.5	165442.9	171357.7	160523.1	153148.5	165542.9	157699.4	#N/A	#N/A	#N/A	#N/A
D3YXJ5	LRAT domain-containing 1	Lratd2	310	34.6	5.54	39394.5	41915.2	40922.2	45115.7	44903.7	42594.9	49361.5	39374.8	46343.3	45153.7	#N/A	#N/A	#N/A	#N/A
Q9WVT6	Carbonic anhydrase 14	Ca14	337	37.5	6.35	28647.6	46001.7	27828.9	31551.2	32412.8	30339.7	28274.6	31920.1	35626.9	39134.4	39205.2	45475.9	48569.7	46515.0
A0A075B5S9	Ig-like domain-containing protein	Ighv9-4	98	10.9	9.07	216730.8	212147.9	172640.8	215182.6	250347.2	184863.4	269137.2	184952.4	193446.4	211326.0	#N/A	#N/A	#N/A	#N/A
Q8CF20	KRAB domain-containing protein	00020N01l	87	9.8	5.31	32172.8	43974.1	51922.5	61600.5	41430.3	35776.4	26068.7	38536.1	39053.2	42775.5	112683.9	146137.9	129202.5	164996.5
B2RS91	RNA polymerase I-specific transcription initiation factor RRN3	Rrn3	656	74.5	5.5	107131.3	24870.1	23932.9	13815.7	20069.9	26662.3	21079.2	20403.0	15438.6	22463.8	4786.6	6402.0	6054.5	5653.7
Q8BHC9	Alpha-(1,3)-fucosyltransferase 11	Fut11	489	55.5	6.23	95512.1	97598.3	91579.8	101769.1	98143.0	104485.0	96159.9	104951.4	102459.0	106950.2	275213.1	250978.5	258918.8	273546.9
Q8K0V2	DCN1-like protein 3	Dcun1d3	304	34.4	5.12	12554.0	11808.6	11132.7	6875.0	13650.6	13247.2	15901.8	13856.7	11844.2	8314.7	230429.9	229974.7	216243.9	236511.4
P97931	Uracil-DNA glycosylase	Ung	306	33.9	9.61	474985.1	532931.4	517338.8	537779.8	441722.0	488502.6	392919.4	450422.3	482119.6	455386.3	#N/A	#N/A	#N/A	#N/A
Q9Z280	Phospholipase D1	Pld1	1074	123.9	8.72	50076.0	50990.5	45328.1	49739.2	58193.4	61996.5	63305.9	54024.4	55020.9	48057.2	#N/A	#N/A	#N/A	#N/A
Q99JY8	Phospholipid phosphatase 3	Plpp3	312	35.2	9.07	218631.3	188726.0	167332.0	170340.7	188880.0	215192.1	187633.0	228072.9	168939.4	184283.4	194685.4	244653.7	183158.8	176737.7
Q91WJ7	SPATS2-like protein	Spats2l	558	61.6	9.69	5780.5	6747.1	6600.5	4751.4	6243.0	5812.2	5761.2	6114.9	4583.7	7488.8	#N/A	#N/A	#N/A	#N/A
Q9D2C7	Bax inhibitor 1	Tmbim6	237	26.5	8.73	586781.6	543494.1	496554.2	543346.7	493551.6	517362.6	580480.3	532697.5	529570.3	517566.9	984847.8	825994.4	832030.1	854101.5
Q3TVA9	Coiled-coil domain-containing protein 136	Ccdc136	1136	131.8	4.84	103694.7	123041.9	113783.4	119973.0	117851.0	113393.1	103299.4	110278.6	119471.3	124046.1	#N/A	#N/A	#N/A	#N/A
P53612	Geranylgeranyl transferase type-2 subunit beta	Rabggtb	339	37.8	5.16	136960.1	139003.8	131824.6	142876.4	129065.6	144095.8	121665.7	127040.7	147854.0	133669.7	164348.9	161559.6	173945.7	148093.9
Q9EP52	Twisted gastrulation protein homolog 1	Twsg1	222	24.8	5.5	13690.3	14518.2	17792.9	15011.0	18990.8	15879.1	17298.2	17521.2	19568.5	10840.8	#N/A	#N/A	#N/A	#N/A
O08580	Steroid hormone receptor ERR1	Esrra	422	45.4	6.38	51454.2	60097.9	49794.3	50537.2	54380.2	55693.9	41833.0	46275.0	47720.6	55686.4	#N/A	#N/A	#N/A	#N/A
Q80UP8	Sodium-dependent phosphate transporter 2	Slc20a2	656	70.8	6.48	182025.6	166876.3	178331.6	163706.2	194442.5	193336.9	179773.7	179729.1	191822.5	187793.3	#N/A	#N/A	#N/A	#N/A
Q8VE42	Ankyrin repeat domain-containing protein 49	Ankrd49	238	27.1	5.16	123969.8	150121.4	129093.9	143071.2	142857.4	135875.8	115955.6	132009.9	142903.7	137990.1	150272.9	154365.4	134196.6	140636.6
P40237	CD82 antigen	Cd82	266	29.6	5.02	251974.7	229532.1	246084.8	238696.7	263374.6	288982.2	293987.5	253590.6	233186.1	237718.6	#N/A	#N/A	#N/A	#N/A
Q6PDH0	Pleckstrin homology-like domain family B member 1	Phldb1	1371	150	8.87	204291.1	241575.7	198278.4	196397.3	235438.9	283233.0	246126.5	213019.0	207747.3	226957.7	28520.1	29792.9	26202.7	26810.2
Q9CYV5	Transmembrane protein 135	Tmem135	458	52.3	9.5	87403.0	92065.9	73327.1	91366.7	86502.5	68230.0	88373.4	77653.1	90572.5	82032.7	76845.8	72723.2	75308.0	76829.5
Q9ERH4	Nucleolar and spindle-associated protein 1	Nusap1	427	48.5	9.89	4856.6	5233.4	4950.1	6494.6	4956.4	7253.9	5387.6	3468.9	6414.6	5869.0	#N/A	#N/A	#N/A	#N/A
Q5ND04	Heat shock factor protein 5	Hsf5	624	67.3	7.08	614168.3	755984.5	746350.4	856564.7	512679.8	463771.3	438280.1	553978.7	635331.3	684138.0	409149.3	620140.1	561349.1	636828.8
Q91WK7	Ankyrin repeat domain-containing protein 54	Ankrd54	299	32.5	6.68	206458.0	183398.3	197536.1	177869.7	181909.0	194244.2	196634.4	197913.8	196874.4	189840.7	#N/A	#N/A	#N/A	#N/A
Q8VHQ9	Acyl-coenzyme A thioesterase 11	Acot11	594	67.3	6.8	118204.4	122870.4	111998.2	113987.3	118543.2	127771.1	140255.5	121467.0	117258.0	112680.3	173827.4	170696.1	160635.5	172688.3
Q60952	Centrosome-associated protein CEP250	Cep250	2414	276.6	5.07	62800.6	60058.0	56615.2	59257.6	72549.3	71249.4	64545.5	64052.9	57925.6	69494.8	#N/A	#N/A	#N/A	#N/A
Q8R3K3	Pentatricopeptide repeat-containing protein 2, mitochondrial	Ptcd2	381	43.8	9.04	153714.4	151222.0	157704.1	149619.0	167410.1	168974.8	149343.7	155063.5	167669.7	164313.5	89053.2	103771.6	95562.4	89322.3
Q9QWF0	Chromatin assembly factor 1 subunit A	Chaf1a	911	101.9	5.4	46284.9	56334.5	40563.3	49196.9	45888.5	45765.1	39462.5	41558.2	47149.6	53645.1	125803.9	147216.4	119720.2	132249.3
Q3U3Q1	Serine/threonine-protein kinase ULK3	Ulk3	472	53.5	7.03	26860.5	6614.3	7770.5	6020.5	5951.8	9470.0	7585.2	4120.3	22554.1	4700.6	32155.0	37749.6	32923.2	35220.5
Q6NVE9	Protein phosphatase PTC7 homolog	Pptc7	310	33	5.27	58955.3	55087.1	51863.1	57750.0	61140.5	55933.7	55919.2	54656.7	60741.5	60905.3	28325.2	17807.6	20743.6	21001.4
Q8R2Q8	Bone marrow stromal antigen 2	Bst2	172	19.1	7.34	86308.2	81073.7	77473.6	80582.4	82649.3	83167.9	89276.1	75365.3	78477.4	90879.4	#N/A	#N/A	#N/A	#N/A
Q9DAJ2	Tektin-1	Tekt1	418	48.6	5.73	119792.7	137114.1	121917.8	150021.5	104903.2	113245.6	109902.3	101563.0	116348.0	124937.1	#N/A	#N/A	#N/A	#N/A
Q9JI08	Bridging integrator 3	Bin3	253	29.6	6.96	124726.0	150048.6	122079.3	126767.1	128115.5	142686.7	125964.6	116449.4	138682.2	140416.9	121051.9	123252.7	105275.6	112410.4
Q6PEE2	CBP80/20-dependent translation initiation factor	Ctif	600	67.8	6.65	67507.0	66400.2	65329.3	62914.3	86782.6	93734.9	85755.2	88702.4	77910.0	87632.4	27267.8	29898.7	24995.1	26043.5
Q8C0P0	Serine/threonine-protein kinase greatwall	Mastl	865	95.9	5.57	20495.8	20010.8	22625.0	19407.6	27084.3	20551.7	17847.5	18688.0	21071.1	24558.2	49686.9	52322.7	48388.5	43629.4
P49282	Natural resistance-associated macrophage protein 2	Slc11a2	568	62.3	5.99	68859.9	58786.3	53968.3	58991.5	57514.2	58700.1	50773.6	58669.2	59198.1	61819.7	#N/A	#N/A	#N/A	#N/A
Q62084	Protein phosphatase 1 regulatory subunit 14B	Ppp1r14b	147	15.9	4.86	28978.5	35526.6	29192.3	30683.6	29549.0	32116.0	27929.0	35439.8	28274.1	29357.2	31963.2	31654.0	31370.1	26563.1
Q5DU00	Doublecortin domain-containing protein 2	Dcdc2	475	51.9	6.51	32311.1	28923.4	32447.0	31807.4	30372.3	29492.7	50207.7	24896.5	25062.3	28905.6	#N/A	#N/A	#N/A	#N/A
Q9WVD4	H(+)/Cl(-) exchange transporter 5	Clcn5	746	83	7.01	53529.5	66402.9	49234.2	50109.4	54639.6	51671.7	61912.5	52929.9	54309.3	51776.1	213994.1	220395.2	226262.4	233022.8
P14069	Protein S100-A6	S100a6	89	10	5.48	485095.7	603694.2	750959.7	564253.3	679805.1	887803.7	1153192.5	883844.6	808851.6	622090.0	893416.9	965569.1	1129967.9	891524.7
B7ZNL9	TatD DNase domain-containing 2	Tatdn2	783	87.5	8.7	46662.6	46800.8	54257.5	43012.1	50036.1	53571.8	52303.5	44719.8	51992.0	54785.7	#N/A	#N/A	#N/A	#N/A
Q7TQI8	Testis-specific Y-encoded-like protein 2	Tspyl2	677	77.6	4.75	91902.7	92582.8	103647.0	93861.5	92969.1	91677.5	81870.8	91961.1	99610.6	102955.6	100573.9	94435.3	102011.5	96429.3
Q923Z3	Protein MTO1 homolog, mitochondrial	Mto1	669	74.3	8.92	98562.5	108179.2	111220.9	101885.8	117100.6	112093.1	105085.2	92704.7	92796.1	104373.5	28471.1	24621.7	21240.0	27799.1
Q62415	Apoptosis-stimulating of p53 protein 1	Ppp1r13b	1087	119.1	6.68	189538.2	180207.7	149485.7	176769.5	196735.3	227960.7	209818.4	156954.1	182682.1	171067.7	#N/A	#N/A	#N/A	#N/A
P56382	ATP synthase subunit epsilon, mitochondrial	Atp5f1e	52	5.8	10.01	1080940.1	1006725.2	1113686.7	1113442.3	1021945.1	853243.1	829537.1	1060701.8	926867.3	864407.8	1762569.8	1768597.8	2158341.2	1848550.7
Q9Z1R4	Uncharacterized protein C6orf47 homolog	D17h6s53e	293	32	6.89	333305.1	298042.4	294344.9	262324.2	285534.5	303622.8	304142.8	284328.7	315633.3	281000.2	242652.1	246967.5	241661.8	230378.6
Q8C3X4	Translation factor Guf1, mitochondrial	Guf1	651	72.4	8.76	46133.1	52273.9	42116.1	46009.3	42037.6	46698.5	43835.6	44661.4	35812.9	43617.0	77151.9	83140.4	82507.6	76675.5
Q91V04	Translocating chain-associated membrane protein 1	Tram1	374	43	9.69	334340.7	353133.9	311248.9	340508.9	312033.0	323799.9	294440.7	312819.4	341883.1	308098.2	187431.5	202223.3	209198.2	217328.4
E9Q414	Apolipoprotein B-100	Apob	4505	509.1	6.81	97710.0	91215.0	71172.3	86160.8	80016.8	69148.5	78839.8	79009.1	82005.5	89382.0	35782.7	38514.7	29330.8	34129.4
Q69ZL1	FYVE, RhoGEF and PH domain-containing protein 6	Fgd6	1399	155.1	7.87	72974.0	80488.7	64443.7	71889.1	70995.4	83521.6	66799.4	68967.3	64588.8	80008.6	#N/A	#N/A	#N/A	#N/A
Q69Z23	Dynein heavy chain 17, axonemal	Dnah17	4481	511.3	5.64	68398.3	75301.0	71980.5	93716.1	69254.7	63242.2	61521.4	67717.5	65054.9	70982.0	#N/A	#N/A	#N/A	#N/A
Q5Y5T1	Palmitoyltransferase ZDHHC20	Zdhhc20	380	43.9	8.25	44221.2	52850.9	43120.1	47406.0	42412.3	44008.1	39726.1	49773.0	47099.2	45971.7	#N/A	#N/A	#N/A	#N/A
Q810A3	Tetratricopeptide repeat protein 9C	Ttc9c	171	20	9.29	87008.8	93655.7	126614.0	96607.9	114116.4	107398.7	108407.5	122231.3	116846.8	97040.5	#N/A	#N/A	#N/A	#N/A
Q9EQM6	Microprocessor complex subunit DGCR8	Dgcr8	773	86.3	6.06	133767.6	159738.5	139015.7	152674.3	140347.3	150171.7	135982.8	131008.7	148307.0	160246.5	61349.6	62131.1	55553.2	64806.1
B2KFW1	Zinc finger and SCAN domain-containing protein 20	Zscan20	1030	115.3	6.74	388985.0	378208.3	470694.7	422469.7	426057.8	448393.0	461496.6	453192.2	451550.6	456806.2	#N/A	#N/A	#N/A	#N/A
Q60866	Phosphotriesterase-related protein	Pter	349	39.2	6.67	114603.0	103424.2	121448.4	99146.9	132006.7	147693.3	177348.4	129541.5	124392.6	117045.8	37478.7	37816.0	33193.7	41216.5
P09541	Myosin light chain 4	Myl4	193	21.1	5.03	40141.4	47692.5	46914.6	50198.2	48142.7	49278.0	41703.0	50838.9	50170.8	41791.6	35612.4	46448.0	42801.8	47769.0
P62876	DNA-directed RNA polymerases I, II, and III subunit RPABC5	Polr2l	67	7.6	7.77	204644.2	201929.8	213904.9	217408.4	245592.6	223736.3	206618.7	210734.6	225084.8	210439.7	356025.3	346739.2	363367.5	345824.1
A2ALW5	DnaJ homolog subfamily C member 25	Dnajc25	357	41.9	9.11	165661.8	158611.1	155992.3	142514.6	156070.2	141153.6	165261.4	148449.2	149861.3	159292.4	416591.7	382141.5	398050.8	371650.0
Q9CWP6	Motile sperm domain-containing protein 2	Mospd2	518	59.8	5.67	90969.7	99128.9	100755.0	86137.6	95871.6	86807.7	96763.9	94625.4	98146.8	99590.9	110074.8	103501.7	88451.4	105288.2
B0V2N1	Receptor-type tyrosine-protein phosphatase S	Ptprs	1907	211.8	7.23	38588.4	34006.2	34496.4	36123.5	41867.8	36315.9	33796.5	35419.6	35806.3	35894.1	#N/A	#N/A	#N/A	#N/A
Q9CPS6	Histidine triad nucleotide-binding protein 3	Hint3	165	18.8	6.33	101720.5	114300.8	121918.5	121107.7	109909.6	117889.7	120501.1	110398.3	117261.6	110744.5	238222.8	287309.3	259593.2	313039.2
Q60766	Immunity-related GTPase family M protein 1	Irgm1	409	46.5	8.28	16721.4	19905.8	21805.1	17478.3	22160.0	23010.6	27337.7	21832.3	16052.8	21216.9	50988.4	58449.2	56963.8	52270.8
O35623	BET1 homolog	Bet1	118	13.3	9.07	59526.5	62202.1	54883.1	61721.9	61054.2	49762.1	49325.0	55931.1	60522.7	55913.0	#N/A	#N/A	#N/A	#N/A
P70371	Telomeric repeat-binding factor 1	Terf1	421	48.2	6.8	121344.4	129808.2	122904.4	125563.4	135451.2	148283.3	119629.2	121009.5	129638.0	130887.6	52809.5	52928.0	46383.6	46741.5
D3Z4R1	Probable ATP-dependent DNA helicase HFM1	Hfm1	1434	161.3	7.85	63533.6	71622.9	72762.7	63550.1	73693.6	72895.7	60858.6	58054.7	69959.0	71238.4	#N/A	#N/A	#N/A	#N/A
Q8VEL2	Myotubularin-related protein 14	Mtmr14	648	72.4	6.76	123551.0	120638.7	104489.2	112267.8	118090.7	137268.8	130159.4	101934.6	130790.5	136592.2	#N/A	#N/A	#N/A	#N/A
Q3TMW1	Coiled-coil domain-containing protein 102A	Ccdc102a	549	62.6	5.69	73784.8	69639.3	77470.9	70797.9	80068.6	86120.3	79308.3	75606.6	74643.5	77268.1	105843.4	116202.7	98409.9	100022.5
Q8C5S7	Meiotic recombination protein REC8 homolog	Rec8	591	67.4	5.27	25697.1	30236.6	27988.5	33562.8	35672.8	35999.0	27993.2	23952.2	31826.2	30428.9	93986.2	112691.2	123387.1	118415.5
Q80ZK9	WD and tetratricopeptide repeats protein 1	Wdtc1	677	75.7	7.15	28545.9	31486.6	25210.4	23323.9	25387.0	25899.6	26679.0	30087.4	36101.1	30319.0	170686.6	190937.3	166863.6	172888.7
P17125	Transforming growth factor beta-3 proprotein	Tgfb3	410	46.9	7.78	120187.3	126617.9	133999.9	120671.5	130710.5	121120.6	110156.2	117715.5	127376.6	132675.7	172591.1	146892.3	143983.8	149427.7
Q8C3B8	Protein RFT1 homolog	Rft1	541	60.3	8.48	137608.6	145106.6	129589.1	122022.3	135112.6	130130.4	118819.9	105187.2	131107.1	145229.0	106084.1	109759.6	104981.7	104445.6
Q3TXX3	Protrudin	Zfyve27	415	46.2	5.35	39489.9	44620.8	47143.4	48528.8	41425.6	28895.6	35008.7	44769.7	39092.6	39542.4	#N/A	#N/A	#N/A	#N/A
E9Q7R9	Cilia- and flagella-associated protein 43	Cfap43	1682	193.3	5.62	2774.3	6011.3	8139.8	8748.6	4713.9	4863.3	4410.6	3523.0	2816.6	7674.7	6333.8	13184.1	10189.0	11911.2
Q9D7M8	DNA-directed RNA polymerase II subunit RPB4	Polr2d	142	16.3	4.79	129982.8	177767.0	182892.5	227832.1	114188.6	112802.5	99142.5	122967.8	138121.7	153905.7	#N/A	#N/A	#N/A	#N/A
Q8VEE0	Ribulose-phosphate 3-epimerase	Rpe	228	24.9	5.41	628012.3	630752.6	626378.0	594787.5	673357.3	615836.2	581808.9	600284.4	624512.5	688615.0	174155.8	182800.7	169991.3	173668.0
P52624	Uridine phosphorylase 1	Upp1	311	34.1	6.58	51497.0	49469.7	58690.1	46937.1	58454.9	53860.5	57651.4	56711.1	54608.3	60934.3	146163.3	123267.3	153400.0	127415.4
Q9CWG9	Biogenesis of lysosome-related organelles complex 1 subunit 2	Bloc1s2	143	16.3	4.88	22289.0	22267.3	23282.6	25102.9	21613.0	22590.7	22190.1	20846.5	20749.7	18839.0	343930.3	357007.5	328623.3	345210.6
Q99JH1	Ribonuclease P protein subunit p25-like protein	Rpp25l	163	17.7	10.46	63871.0	71054.2	60635.4	70107.3	81340.2	75226.0	66235.8	70814.6	80568.9	74852.8	170604.4	171576.8	183162.5	170691.5
Q99MV2	Testis-expressed protein 19.1	Tex19.1	351	40.4	4.69	262656.1	300231.8	251909.9	262498.6	270404.8	270427.9	223668.3	241310.5	251787.0	262224.6	139294.1	154406.5	128724.7	138074.1
Q9WVL2	Signal transducer and activator of transcription 2	Stat2	923	105.4	5.27	84148.8	76086.5	77591.0	106980.7	79508.2	79802.2	80387.3	77241.5	74823.0	81291.8	48572.4	50904.0	52788.5	61550.0
Q7TMR0	Lysosomal Pro-X carboxypeptidase	Prcp	491	55	6.79	126329.5	131792.2	115760.4	121059.3	127482.9	131624.9	114332.8	127930.3	122060.9	125655.4	#N/A	#N/A	#N/A	#N/A
Q99LM9	Transcriptional adapter 1	Tada1	335	37.4	7.62	180263.3	207970.8	164063.6	189553.4	194540.2	200748.2	162666.8	156436.2	187284.6	194162.1	#N/A	#N/A	#N/A	#N/A
Q9Z1S8	GRB2-associated-binding protein 2	Gab2	665	73.2	8.31	102568.1	100687.3	90129.5	95958.6	93763.7	99690.1	96372.2	93500.4	96674.6	99458.6	51559.8	61452.4	60654.5	60073.4
Q924X6	Low-density lipoprotein receptor-related protein 8	Lrp8	996	109.7	5.01	37729.0	57300.3	37958.8	50462.7	43587.9	42168.8	39330.0	42035.2	45131.7	48464.9	37582.9	40024.4	34805.3	38478.2
P82347	Delta-sarcoglycan	Sgcd	289	32.1	9.1	83108.8	87769.0	97553.1	75774.4	95347.8	97053.6	95999.2	88960.8	86434.9	94537.9	89127.6	84920.3	89418.9	86860.6
L7N1Y0	EF-hand domain-containing protein	Dnah7b	4068	466.4	6.28	36923.8	50220.7	52319.2	64380.5	34275.8	27577.7	28231.9	36181.8	44611.9	46758.2	#N/A	#N/A	#N/A	#N/A
Q8BTG7	Protein NDRG4	Ndrg4	352	38.5	6.32	130042.7	128879.7	157420.4	127884.6	193372.4	171954.1	172294.1	160192.7	146359.2	156548.8	162777.5	152234.6	138185.0	146384.1
Q8C635	Glycerol kinase-like 1	Gykl1	549	59.9	5.27	117475.0	188412.1	146154.1	181676.2	128930.3	127175.2	120240.3	113056.6	124320.8	163410.1	68926.7	85829.9	78017.6	90669.5

E9Q9Q2	R3H domain-containing 1	R3hdm1	1135	124.3	8.7	115090.0	126234.0	114082.1	111927.7	122335.4	124315.5	100083.5	105611.2	111405.2	108005.7	260449.9	253501.9	230672.5	240874.5
Q9DC11	Plexin domain-containing protein 2	Plxdc2	530	59.6	6.6	71461.9	61581.7	74990.5	57308.7	79878.5	72873.1	91534.3	67888.7	72195.8	62367.2	116739.6	132861.8	110685.9	121788.0
Q922M7	Ashwin	MGI:213829	232	26	9.55	50547.5	53615.6	52381.8	57741.5	52302.8	53875.0	49479.3	45307.6	50928.1	51959.2	80960.5	88720.1	68133.9	78350.1
Q6PGG6	Guanine nucleotide-binding protein-like 3-like protein	Gnl3l	577	65.2	8.6	69078.8	77377.8	72696.6	74262.0	71597.3	65952.3	68900.1	64098.2	60648.3	64604.7	58803.0	53337.8	52367.8	48295.9
P0C872	Bifunctional peptidase and (3S)-lysyl hydroxylase Jmjd7	Jmjd7	316	35.9	5.47	52538.7	49126.2	50954.8	56909.0	48393.9	55304.3	50645.5	43799.9	49243.5	53027.3	66472.5	63966.6	60745.5	59073.9
Q9JL62	Glycolipid transfer protein	Gltp	209	23.7	7.39	31841.3	35532.5	27885.0	42672.7	33013.8	34502.7	27480.5	32142.8	40952.7	38931.5	#N/A	#N/A	#N/A	#N/A
Q91VM8	Zinc finger protein 959	Zfp959	539	62.6	8.62	57027.7	63210.1	72741.2	60595.9	68129.6	78148.3	77568.8	69559.4	75749.3	75056.5	#N/A	#N/A	#N/A	#N/A
Q9JLI6	Selenocysteine lyase	Scly	432	47.1	6.8	25141.5	28642.9	22091.5	31711.0	25664.0	23116.7	27987.7	23438.9	23488.6	26352.8	48765.1	64799.0	57347.4	66279.6
Q04887	Transcription factor SOX-9	Sox9	507	56	6.81	291729.3	262139.2	290465.6	297219.8	349973.5	351418.5	407980.6	306229.8	327448.1	346723.0	133640.5	132302.4	134223.8	123484.9
Q80TJ1	Calcium-dependent secretion activator 1	Cadps	1355	153	5.74	98982.3	101033.5	84315.4	95155.9	90749.2	96995.4	94159.0	87198.5	93263.9	81841.9	#N/A	#N/A	#N/A	#N/A
Q6NZB1	Protein arginine N-methyltransferase 6	Prmt6	378	41.8	5.34	89471.7	98111.7	75276.9	86976.2	85954.7	80122.6	85385.1	75441.6	80335.0	91506.2	7840.1	8868.5	10364.8	6414.2
Q9D0V7	Receptor-binding cancer antigen expressed on SiSo cells	Ebag9	213	24.3	6.29	95044.1	112752.6	93878.4	117919.0	95567.8	91662.5	84828.2	91877.6	104377.2	97238.8	58659.0	68255.1	67042.4	89948.7
Q8JZU0	Nucleoside diphosphate-linked moiety X motif 13	Nudt13	352	39.1	8.02	70834.7	71115.8	61206.6	64875.5	60776.6	75321.7	60854.1	62173.2	63844.2	62587.4	#N/A	#N/A	#N/A	#N/A
Q9D992	Membrane-anchored junction protein	Majin	256	29.2	6.9	73750.0	87448.2	77341.0	91675.5	64838.5	74942.8	63518.4	67867.6	76273.0	74838.0	46665.8	57517.9	50591.0	53407.9
Q9JHC9	ETS-related transcription factor Elf-2	Elf2	593	63.2	6.42	131174.6	121001.1	128391.1	123458.6	137962.0	139679.0	126291.1	108728.6	125990.4	124002.9	205218.5	203411.7	215260.6	200811.5
Q8VC51	Telomerase Cajal body protein 1	Wrap53	532	58.1	4.48	124994.0	137760.9	111849.1	133608.1	122630.4	124960.4	120677.6	124131.3	125236.7	131929.2	#N/A	#N/A	#N/A	#N/A
P01921	H-2 class II histocompatibility antigen, A-D beta chain	H2-Ab1	265	29.9	7.83	64305.4	47231.6	52056.3	39717.8	60039.3	66989.1	63373.8	53904.9	58201.1	51603.8	#N/A	#N/A	#N/A	#N/A
Q9CSB4	Partitioning defective 3 homolog B	Pard3b	1203	132.7	8.56	154514.2	150675.3	129961.6	137636.9	130891.0	130003.1	122381.4	133812.9	132205.6	137653.6	172556.8	190640.4	143585.9	164569.9
Q9JHH9	Coatomer subunit zeta-2	Copz2	205	22.9	5.17	72116.0	74521.3	63658.0	70186.0	66205.5	62466.9	59606.5	62745.7	63511.1	67325.8	209155.9	250954.7	215325.7	235632.2
O35448	Lysosomal thioesterase PPT2	Ppt2	302	34.3	6.39	157524.6	187155.2	147806.0	162961.1	148546.6	153201.6	138122.0	140471.4	153010.1	151612.0	257471.5	242245.1	254528.3	231532.4
Q6PAR0	Kelch domain-containing protein 10	Klhdc10	439	49	9.32	77620.3	77735.3	94740.2	80185.7	86018.8	79973.3	70152.4	66140.6	83525.2	84137.9	#N/A	#N/A	#N/A	#N/A
Q8VEK2	Rhomboid domain-containing protein 2	Rhbdd2	361	39.1	9.48	138513.3	160297.2	134350.3	158353.6	152395.0	167813.6	144490.2	162375.0	140775.2	144318.0	113084.1	137620.9	106246.6	120596.4
Q505D7	Optic atrophy 3 protein homolog	Opa3	179	20.1	8.34	73769.1	70021.1	68946.4	73798.5	68399.3	76212.0	71647.2	79602.3	72520.0	74972.1	#N/A	#N/A	#N/A	#N/A
Q8BHL3	TBC1 domain family member 10B	Tbc1d10b	798	87.2	8.9	45567.1	48002.0	41494.7	43864.0	47943.2	42817.6	47084.7	41390.2	41982.1	46628.5	71104.0	72229.6	66575.4	63985.2
P63254	Cysteine-rich protein 1	Crip1	77	8.5	8.57	218592.8	211786.4	177877.2	212406.4	174447.8	376099.1	459837.2	472873.7	218551.9	188888.4	424027.9	456395.8	411389.1	461722.3
O88512	AP-1 complex subunit gamma-like 2	Ap1g2	791	87.8	6.51	435994.7	474309.4	542433.8	499896.6	502290.2	534588.5	505088.7	555390.0	489791.5	461525.3	#N/A	#N/A	#N/A	#N/A
Q8BFR1	Zinc finger CCCH-type antiviral protein 1-like	Zc3hav1l	296	32.8	8.44	166410.3	181085.3	152750.8	154684.3	172475.1	180804.4	174556.2	169539.3	164236.0	186372.9	7806.8	12945.3	11981.1	10578.0
Q80TY5	Vacuolar protein sorting-associated protein 13B	Vps13b	3993	443.7	6.46	102735.4	113491.0	117566.6	112197.8	111713.2	114289.2	106557.1	95852.2	107073.7	105171.5	179085.3	190098.2	180232.7	179788.2
Q5DTT8	Paraneoplastic antigen-like protein 5	Pnma5	618	69.1	5.27	44821.1	55418.5	57396.1	59222.9	54322.8	56829.3	44247.4	46278.9	48325.9	50643.9	165308.5	171076.1	174961.5	180922.1
O08760	N-glycosylase/DNA lyase	Ogg1	345	38.9	8.85	162209.5	170021.0	131223.8	156698.9	150012.9	141624.9	144945.9	144173.9	153557.6	161296.4	#N/A	#N/A	#N/A	#N/A
Q60591	Nuclear factor of activated T-cells, cytoplasmic 2	Nfatc2	927	100	6.76	109805.7	117252.8	92525.9	83526.4	101437.4	103670.6	85256.6	99148.3	101508.6	109325.3	#N/A	#N/A	#N/A	#N/A
Q8K2H3	Protein FAM13B	Fam13b	851	97	5.05	149644.3	172778.2	156187.7	158745.8	166659.3	179042.7	161570.8	154292.8	162534.4	157485.0	#N/A	#N/A	#N/A	#N/A
Q8BIX3	ARL14 effector protein	Arl14ep	276	31	8.24	86904.0	92614.5	97621.7	80356.8	102795.9	97677.6	94449.5	87331.8	95515.6	110069.6	#N/A	#N/A	#N/A	#N/A
Q9R0M5	Thiamin pyrophosphokinase 1	Tpk1	243	27.1	5.76	51002.2	49492.3	47667.9	59931.1	61324.6	61495.8	65912.6	60420.2	51052.1	54136.5	41279.1	38310.8	39235.4	41146.8
A2AR50	Ras-specific guanine nucleotide-releasing factor RalGPS1	Ralgps1	585	65.4	9.41	14714.4	15772.6	13590.0	14678.2	15063.1	15792.7	13393.5	15952.3	16014.5	14406.7	#N/A	#N/A	#N/A	#N/A
Q8VCE6	5'(3')-deoxyribonucleotidase, mitochondrial	Nt5m	220	25.6	8.35	171844.4	180510.8	199191.9	196030.4	213566.8	207436.7	203207.7	186653.6	193551.9	200039.1	#N/A	#N/A	#N/A	#N/A
D3Z623	DUF4482 domain-containing protein	30159F19I	648	73.9	8.13	31264.8	34357.4	32000.0	34433.7	37259.2	35578.6	35954.2	33386.1	36615.2	38277.2	#N/A	#N/A	#N/A	#N/A
Q80W37	Snurportin-1	Snupn	358	41	6.29	192252.6	239645.8	225800.2	222450.1	217586.9	203425.3	178182.1	192506.4	218651.6	244778.3	318941.5	401338.7	353884.6	350450.2
Q8R1S4	Protein MTSS 1	Mtss1	759	82.4	6.8	89035.5	67417.8	65195.8	68409.1	90038.3	82877.5	82400.5	65800.8	77467.1	73528.3	#N/A	#N/A	#N/A	#N/A
E9PUQ3	NKAP domain-containing 1	Nkapd1	292	33.9	9.74	87762.3	92781.8	94589.5	85823.7	107986.8	91178.1	93156.4	85384.1	93948.5	92718.7	#N/A	#N/A	#N/A	#N/A
Q9EQ31	Lysophosphatidic acid receptor 3	Lpar3	354	40.3	9.44	12659.6	13755.3	14347.5	9635.1	13994.7	12458.1	13906.8	17024.9	11254.0	11884.9	#N/A	#N/A	#N/A	#N/A
Q9QZB9	Dynactin subunit 5	Dctn5	182	20.1	8.02	34246.8	45126.0	48060.2	46131.2	42215.2	41114.8	36009.3	42759.2	45873.3	39263.4	#N/A	#N/A	#N/A	#N/A
Q8K5B2	Multiple coagulation factor deficiency protein 2 homolog	Mcfd2	145	16.2	4.72	28156.0	44314.9	38470.1	52186.2	35526.7	38515.0	36141.9	41985.8	48442.5	38492.7	#N/A	#N/A	#N/A	#N/A
Q62394	Zinc finger protein 185	Znf185	352	38.3	5.12	153842.2	160059.2	153647.0	157685.1	160231.5	174313.5	168670.6	164100.2	154212.8	155533.1	#N/A	#N/A	#N/A	#N/A
P70170	ATP-binding cassette sub-family C member 9	Abcc9	1546	174.1	7.47	45305.6	48413.2	38963.7	44227.6	53132.1	52814.6	62601.7	44479.1	46877.3	45023.6	24807.6	25842.7	28444.2	25415.2
Q9D8C6	Mediator of RNA polymerase II transcription subunit 11	Med11	117	13.1	5.96	20766.3	24753.6	20478.5	25396.0	27735.4	22014.6	16176.4	19496.0	24106.7	22241.7	#N/A	#N/A	#N/A	#N/A
Q9WTY1	Programmed cell death protein 7	Pdcd7	482	54.3	10.4	31561.5	30796.3	33557.3	29460.6	34172.0	31950.3	32743.2	33603.9	33038.9	28805.2	67725.1	78864.6	81137.5	78462.7
P61458	Pterin-4-alpha-carbinolamine dehydratase	Pcbd1	104	12	6.8	318554.4	353975.8	322136.2	373071.5	413730.4	486135.3	534302.7	415515.8	344490.7	376358.6	141582.4	152720.4	129843.0	147612.3
Q6Q783	Histone-lysine N-methyltransferase KMT5C	Kmt5c	468	53.1	9	106603.8	108488.2	93749.7	102175.2	99814.2	103936.6	90294.2	92969.5	103198.0	107444.4	122150.7	126389.4	115593.5	114768.8
Q80YS6	Actin filament-associated protein 1	Afap1	731	80.6	8.68	74648.5	86308.8	65922.8	80803.9	82895.3	80894.6	74202.3	77779.5	85101.6	87109.9	#N/A	#N/A	#N/A	#N/A
P97366	Ecotropic viral integration site 5 protein	Evi5	809	92.9	6.16	142338.3	138116.9	151364.1	121472.6	170881.6	180916.0	161871.6	165119.5	155879.0	126528.7	137356.8	143893.9	145068.4	135000.4
Q9WVS6	E3 ubiquitin-protein ligase parkin	Prkn	464	51.6	7.18	52712.9	50241.3	53820.6	53499.8	59183.7	52655.3	58706.7	51897.0	62604.6	60326.7	35013.0	40048.2	37700.9	38948.7
Q9D0K0	TBC1 domain family member 7	Tbc1d7	293	33.8	7.2	64130.5	65073.7	65427.1	68421.7	64671.9	71328.8	68848.3	61718.9	69266.4	64066.7	229171.1	241721.7	205626.4	216020.7
Q9CZG3	COMM domain-containing protein 8	Commd8	183	20.8	5.59	156115.2	170529.7	148420.6	165866.4	157116.2	174356.4	155098.0	173823.8	169181.9	158591.1	295305.5	327877.9	320487.1	317043.6
Q9Z2S7	TSC22 domain family protein 3	Tsc22d3	137	15.2	4.53	68884.4	72513.7	87370.3	71547.3	88413.5	77326.2	68712.5	70574.5	75519.5	75539.2	51317.1	52188.1	45638.5	53088.9
Q8K245	UV radiation resistance-associated protein	Uvrag	698	77.5	7.97	36745.1	40785.5	34883.7	32573.5	36219.6	37742.8	34906.1	35161.2	34329.0	28616.7	#N/A	#N/A	#N/A	#N/A
Q9DAX9	Amyloid protein-binding protein 2	Appbp2	585	66.8	6.9	456124.3	473653.1	424626.5	427510.6	456395.3	447012.4	426556.8	436806.8	442672.0	475818.9	#N/A	#N/A	#N/A	#N/A
P49222	Erythrocyte membrane protein band 4.2	Epb42	691	76.7	7.31	129343.3	127626.9	99946.2	146102.5	150828.7	101423.6	120355.9	110563.4	116304.1	111278.7	145895.9	148978.3	130196.1	153701.8
E9Q8T7	Dynein heavy chain 1, axonemal	Dnah1	4250	486.8	5.57	39321.8	51567.7	50358.0	54773.9	48025.8	46591.8	38880.2	43788.3	44174.4	47702.3	74155.8	106546.6	91320.2	125060.3
Q91ZN5	Adenosine 3'-phospho 5'-phosphosulfate transporter 1	Slc35b2	431	47.3	9.29	195921.8	200106.8	179363.8	195944.6	165185.8	179167.1	163213.7	183275.3	177168.8	176660.8	323276.7	379745.3	309368.2	363466.8
Q99JP4	Anaphase-promoting complex subunit CDC26	Cdc26	85	9.8	6.81	290189.0	289096.0	269988.8	287500.4	281972.2	304165.5	271071.7	259745.9	310798.0	318523.3	86886.8	101990.1	95149.2	89981.0
O54998	Peptidyl-prolyl cis-trans isomerase FKBP7	Fkbp7	218	24.9	6.04	38145.0	30942.1	34134.0	37878.4	32435.9	32573.3	32421.7	39403.6	32003.6	31758.7	#N/A	#N/A	#N/A	#N/A
P14847	C-reactive protein	Crp	225	25.3	6.2	194475.6	196908.2	208136.5	183003.4	208891.2	223344.4	225646.6	202187.8	189514.5	190344.8	#N/A	#N/A	#N/A	#N/A
Q9D3W4	GPN-loop GTPase 3	Gpn3	284	32.8	4.53	24780.8	26845.2	34409.1	32206.3	24764.3	32730.8	25767.9	32846.1	32469.5	29933.8	#N/A	#N/A	#N/A	#N/A
Q9D8U0	Interferon-related developmental regulator 2	Ifrd2	441	47.7	6.95	35455.3	43153.7	30528.1	41138.1	35998.6	41379.3	67477.8	31792.1	37270.0	37840.9	247757.7	217709.6	233088.9	229997.7
Q3UZA1	CapZ-interacting protein	Rcsd1	412	44.1	5.16	141384.4	131872.5	128518.4	144900.9	148586.7	135991.1	174022.6	168480.1	137617.5	158303.8	69256.1	57902.6	56247.7	57967.0
P70387	Hereditary hemochromatosis protein homolog	Hfe	359	40.5	6.24	131632.2	122499.9	117698.8	112626.4	116617.5	131599.1	152370.9	132224.1	120876.0	110613.2	#N/A	#N/A	#N/A	#N/A
Q8BH89	WAP four-disulfide core domain protein 15A	Wfdc15a	80	9.1	8.73	26235.9	30685.7	17816.1	25894.4	27232.9	27890.7	21957.5	21838.5	23989.4	31478.3	2914.2	7023.0	8490.7	4657.5
Q8CG03	cGMP-specific 3',5'-cyclic phosphodiesterase	Pde5a	865	98.3	6.05	132055.8	114650.4	94267.6	116979.0	95182.1	98084.2	104576.5	96368.6	101765.1	108763.2	104559.3	139521.0	105285.3	108615.9
Q8R015	Biogenesis of lysosome-related organelles complex 1 subunit 5	Bloc1s5	185	21.3	7.34	112332.8	98736.3	114909.2	109598.1	119491.8	123068.2	119094.2	103701.9	132052.4	115260.0	#N/A	#N/A	#N/A	#N/A
Q8VEK6	Inhibitor of growth protein 3	Ing3	421	46.8	6.27	91961.0	98615.4	93393.4	89561.3	101128.1	100626.7	93159.0	89245.1	99261.5	101769.0	322901.5	203061.4	233208.3	211106.2
Q923T7	E3 ubiquitin-protein ligase TRIM7	Trim7	510	57	8.05	138747.2	127597.9	125001.9	134735.4	126228.0	128468.9	136227.1	124859.1	137358.8	148378.0	#N/A	#N/A	#N/A	#N/A
Q99MX2	Ferritin heavy polypeptide-like 17	Fthl17	176	20.5	5.49	85977.4	88346.1	88896.2	86445.1	94732.9	92537.8	88653.8	86775.2	105711.6	100563.4	80991.5	67753.2	85423.2	77740.1
Q3TTL0	Uncharacterized protein C3orf38 homolog	MGI:191485	348	39.5	6.2	258241.0	256916.2	286106.9	255643.7	300858.5	295744.0	263319.5	270547.1	278992.0	282045.4	223588.4	241497.7	241574.5	221396.0
B1AXH1	NHS-like protein 2	Nhsl2	1219	132.3	8.48	126387.8	125951.9	119282.1	122959.9	126759.3	137613.0	141708.2	121200.2	114770.1	123392.9	63592.0	74326.9	63810.9	69793.6
P15655	Fibroblast growth factor 2	Fgf2	154	17.1	9.52	70561.1	63568.7	70032.8	63920.2	80049.4	91844.1	99562.1	70242.3	68218.2	75924.1	161200.3	149850.8	138591.7	137666.7
E9Q1Z2	ENTH domain-containing protein	Enthd1	618	68.4	6.84	38675.5	42140.1	39183.6	50663.6	24480.5	24920.8	24301.0	30470.6	37388.9	29539.3	28848.3	41478.2	33183.3	41696.9
Q8BXJ8	Zinc finger protein 385B	Znf385b	482	51.3	9.83	79509.4	77517.4	85428.9	79526.3	94792.4	86978.4	86944.2	85960.0	79582.4	90165.1	72751.2	80275.1	77266.7	83455.4
Q8R0Y8	Mitochondrial coenzyme A transporter SLC25A42	Slc25a42	318	35.2	10.05	62787.4	54892.6	45190.6	50784.4	49483.1	46915.3	48049.2	45800.0	51719.7	49815.3	#N/A	#N/A	#N/A	#N/A
Q8R3Q0	Store-operated calcium entry-associated regulatory factor	Saraf	334	35.8	8.19	100456.5	105875.0	101293.1	100902.7	122733.5	126216.1	110425.7	103903.8	114371.6	112861.3	347116.5	368403.9	340597.2	382137.4
Q9WTK5	Nuclear factor NF-kappa-B p100 subunit	Nfkb2	899	96.8	6.37	50660.7	59362.3	50704.1	49182.2	60545.7	45787.0	53126.8	44023.9	44195.2	52502.0	#N/A	#N/A	#N/A	#N/A
Q60961	Lysosomal-associated transmembrane protein 4A	Laptm4a	233	26.8	6.51	91044.4	99429.5	100167.7	96368.8	100021.9	107895.5	90129.7	100515.6	101455.1	106094.1	2753.3	4666.6	6137.6	4877.6
Q8K382	DENN domain-containing protein 1A	Dennd1a	1016	111.5	6.79	79589.9	77242.8	90494.0	77945.5	81226.9	79252.0	92228.6	65383.6	88814.3	81625.4	#N/A	#N/A	#N/A	#N/A
P14106	Complement C1q subcomponent subunit B	C1qb	253	26.7	8.15	98179.3	100374.9	89593.2	102413.5	98570.3	100782.8	96723.7	87396.8	103059.8	90486.3	#N/A	#N/A	#N/A	#N/A
P22366	Myeloid differentiation primary response protein MyD88	Myd88	296	33.7	5.52	63009.3	59220.3	70018.9	56439.2	79707.5	107304.0	111070.7	71665.7	72418.5	63883.4	#N/A	#N/A	#N/A	#N/A
Q6SJ95	TATA box-binding protein-like protein 2	Tbpl2	350	38.9	6.11	304209.2	184233.2	284321.3	368089.2	200895.7	170375.9	152778.7	148617.3	322673.1	200972.0	#N/A	#N/A	#N/A	#N/A
Q9D4G9	RecQ-mediated genome instability protein 1	Rmi1	616	68.4	5.41	153674.5	174775.3	176983.5	168760.3	182769.1	173533.2	140947.4	151469.3	169411.7	193221.6	#N/A	#N/A	#N/A	#N/A
Q2HXL6	ER degradation-enhancing alpha-mannosidase-like protein 3	Edem3	931	104.1	5.06	189031.8	231394.9	177457.9	179922.9	175047.3	181687.7	166960.7	202488.7	176385.7	177563.0	53767.6	54527.0	62747.4	57906.4
Q9D1L9	Ragulator complex protein LAMTOR5	Lamtor5	91	9.6	4.87	108050.0	129059.9	102576.5	110155.3	127849.4	137254.8	133235.9	116757.6	106936.1	103769.4	46770.5	57329.2	48985.9	47744.4
A0A0B4J1M0	Ig-like domain-containing protein	Ighv1-77	98	10.7	9.22	76154.4	86799.1	85747.1	85285.3	108918.8	80718.1	74364.7	92447.3	86703.5	96974.9	#N/A	#N/A	#N/A	#N/A
Q8K039	Uncharacterized protein KIAA1143 homolog	MGI:191345	155	17.5	8.48	137437.1	160223.9	153232.2	156292.2	157408.7	164561.6	164135.7	136097.9	160285.6	158478.7	#N/A	#N/A	#N/A	#N/A
Q7TN79	A-kinase anchor protein 7 isoform gamma	Akap7	314	35.5	7.87	52035.6	48739.3	52463.2	53281.3	52566.3	59199.7	54067.6	51336.8	52247.4	54625.1	68281.7	66198.3	58718.5	64010.9

Q9ERU3	Zinc finger protein 22	Znf22	237	27.3	10.29	329838.3	334119.0	362839.8	332885.9	424483.2	404151.8	396645.9	321312.6	345797.1	362187.4	320632.6	350809.8	364100.0	306875.5
Q8BH73	Glutaminyl-peptide cyclotransferase-like protein	Qpctl	383	42.7	9.29	50908.9	49974.0	46172.3	41347.1	41004.4	45475.0	46701.6	42120.8	46327.1	49184.1	#N/A	#N/A	#N/A	#N/A
Q9CPV5	Polyamine-modulated factor 1	Pmf1	202	23.1	5.26	43126.2	58068.7	53973.9	55992.7	57869.0	54978.3	44978.8	47217.7	60438.6	52848.1	62807.5	62132.6	70773.2	75827.9
Q60584	F-box/WD repeat-containing protein 2	Fbxw2	422	47.9	6.79	53443.1	61594.8	51288.1	64572.3	58030.7	56063.2	49830.1	51959.4	58584.2	51196.5	#N/A	#N/A	#N/A	#N/A
P59808	SAM and SH3 domain-containing protein 1	Sash1	1230	135.5	6.2	50818.4	41621.6	37024.5	40110.0	49752.2	49449.4	55274.5	38157.5	41511.5	51505.1	#N/A	#N/A	#N/A	#N/A
Q9D6I7	Divergent protein kinase domain 1A	Dipk1a	428	48.9	7.03	49223.3	49759.4	60693.3	49096.2	48849.5	53626.6	56007.2	49701.1	49049.7	59010.9	#N/A	#N/A	#N/A	#N/A
Q80XC2	tRNA (adenine(58)-N(1))-methyltransferase catalytic subunit TRMT61A	Trmt61a	290	31.6	6.79	35640.2	43842.4	32401.3	31635.8	36536.4	35986.5	32880.1	37730.2	36631.6	34236.6	16900.0	23496.3	20624.3	18077.8
O70293	G protein-coupled receptor kinase 6	Grk6	576	65.9	8.1	63815.6	69593.2	66922.9	73347.5	67133.7	62590.7	58927.5	65348.4	67410.8	69838.0	108041.0	102469.1	98864.9	87055.7
Q8CFC7	CLK4-associating serine/arginine rich protein	Clasrp	668	76.8	10.36	264461.9	281037.0	285124.2	285169.3	329996.2	308077.6	283235.8	267158.9	305285.3	310321.1	249296.5	226568.8	246897.1	246942.3
Q8VC48	Peroxisome assembly protein 12	Pex12	359	40.6	9	44926.1	42735.9	40221.7	36416.4	40545.0	43965.5	41670.0	39723.4	37890.0	41040.2	72924.7	70241.9	75843.0	73417.3
Q91V98	Endosialin	Cd248	765	81.8	5.66	280013.9	229921.2	255525.7	207522.7	263550.3	322675.0	285225.6	277962.4	251870.3	264695.8	71387.5	74117.2	80647.9	65397.9
Q8K410	Disintegrin and metalloproteinase domain-containing protein 32	Adam32	754	83.9	7.08	60066.7	70908.3	81460.3	99596.9	47607.0	46865.9	49204.8	45303.5	63833.5	74715.7	#N/A	#N/A	#N/A	#N/A
Q9Z2D0	Myotubularin-related protein 9	Mtmr9	545	62.9	6.62	121703.3	133446.4	113588.0	123400.2	131223.4	125414.7	112788.3	111646.2	125373.0	134245.3	115393.7	127824.9	128512.4	135746.8
Q91YQ3	Cold shock domain-containing protein C2	Csdc2	154	16.8	7.55	352513.5	354928.4	359944.7	296209.8	437621.5	439980.5	440307.2	369872.8	389424.7	389157.7	886737.8	764138.3	915705.9	670551.7
Q91W78	U6 snRNA phosphodiesterase	Usb1	267	30.2	5.63	33276.0	35954.8	30951.2	33325.1	33064.9	35923.4	35729.9	28500.4	33336.1	32133.3	46982.9	56868.2	40057.3	47678.4
Q3UBX0	Transmembrane protein 109	Tmem109	243	26.3	9.89	299555.4	232976.1	235881.7	236401.7	242237.4	256575.8	303349.0	266315.1	256302.2	244238.8	169234.3	147963.3	153770.5	139833.0
Q99P69	Kinetochore protein Nuf2	Nuf2	463	54.6	8.22	60284.9	58041.7	51914.6	53688.9	58756.5	58108.3	49565.3	51644.0	57078.8	59534.3	#N/A	#N/A	#N/A	#N/A
Q8CIL4	Uncharacterized protein C1orf131 homolog	MGI:191377	281	31.3	9.85	39280.2	39174.4	40727.2	33820.8	45121.3	43406.6	39350.2	44377.5	32341.5	41619.6	96657.7	94022.0	88746.1	85218.8
Q91VR7	Microtubule-associated proteins 1A/1B light chain 3A	Map1lc3a	121	14.3	8.68	201566.0	212508.8	267193.9	228738.3	204805.9	200320.2	225952.9	230890.2	208204.3	203717.7	332097.1	312910.9	378888.7	321809.8
P58059	28S ribosomal protein S21, mitochondrial	Mrps21	87	10.6	10.32	59622.5	84540.3	66734.2	73460.9	69839.0	70066.9	62274.4	68841.4	75100.1	69808.8	183651.3	229064.8	170753.3	193328.5
Q2KN98	Cytospin-A	Specc1l	1118	124.4	5.76	219953.9	209601.2	213075.2	202592.1	214507.5	230169.4	220147.0	205321.7	203079.5	204834.2	209458.2	225505.2	185510.7	205037.3
Q9D385	ADP-ribosylation factor-like protein 2-binding protein	Arl2bp	163	18.7	4.32	78251.4	94613.3	105757.4	115099.3	87434.3	91402.0	76793.5	75244.2	97641.3	95684.4	#N/A	#N/A	#N/A	#N/A
Q9CPX8	Cytochrome b-c1 complex subunit 10	Uqcr11	56	6.5	9.82	102023.9	118067.8	111306.5	121281.7	104283.5	82106.8	97151.7	111740.0	107805.1	105047.9	63386.4	61542.6	71513.6	67420.5
Q9CR88	28S ribosomal protein S14, mitochondrial	Mrps14	128	14.9	11.39	86521.1	113354.4	87884.9	104853.5	103018.6	96215.1	85628.3	87365.3	104157.9	102737.6	107388.8	109572.3	100671.3	102937.1
P01887	Beta-2-microglobulin	B2m	119	13.8	8.44	74139.8	79506.1	65024.4	73767.1	79715.5	75792.4	74168.9	82129.2	68053.3	66358.7	199425.2	183899.0	208138.7	205601.5
Q8C1M2	Zinc finger protein 428	Znf428	176	19	4.23	26430.1	26559.5	20520.3	24716.6	20647.4	24379.8	26485.2	23575.5	25955.3	25985.5	#N/A	#N/A	#N/A	#N/A
P41731	CD63 antigen	Cd63	238	25.7	6.98	1121737.9	1136216.0	1116339.2	1028168.5	1278262.2	1239916.4	1408513.3	1152063.4	1128796.7	1061553.3	626526.7	493496.4	595210.4	510180.9
Q6NXH8	Methyltransferase-like protein 25	Mettl25	597	66.7	6.64	39834.7	54680.1	42214.1	41106.6	44757.8	51089.9	43525.6	43254.3	41199.0	48507.9	#N/A	#N/A	#N/A	#N/A
Q9DC22	DDB1- and CUL4-associated factor 6	Dcaf6	876	97.5	5.22	58055.1	72146.4	57645.6	59049.9	57007.6	62430.4	51755.5	62996.5	61188.5	58978.9	#N/A	#N/A	#N/A	#N/A
Q6PHP4	Zinc finger protein 512B	Zfp512b	879	96.2	9.83	60135.3	65482.0	64391.1	75845.2	69124.0	66212.3	73466.4	66138.4	70258.7	62771.6	97758.0	95217.0	87649.3	96860.5
Q9CZQ9	Bardet-Biedl syndrome 5 protein homolog	Bbs5	341	38.8	5.6	118527.4	123221.7	123796.8	130841.0	118281.2	119078.4	109345.3	105984.2	123365.0	127757.6	73225.6	99870.0	92707.6	93132.8
P97865	Peroxisomal targeting signal 2 receptor	Pex7	318	35.5	5.58	109075.9	115784.8	120847.1	112142.4	104150.7	135035.3	120560.3	113888.5	110969.0	116513.9	100172.9	109263.0	119275.1	97384.2
Q8CIC2	Nucleoporin NUP42	Nup42	420	44.3	9.25	70146.5	49157.7	64270.1	63223.2	74967.5	71960.5	73525.9	64805.9	73350.9	52622.1	46041.6	49404.3	46361.3	43905.9
O08999	Latent-transforming growth factor beta-binding protein 2	Ltbp2	1813	195.7	5.29	23400.8	23195.3	24818.3	23663.2	25281.7	25904.1	22243.7	24707.3	22077.6	22630.4	59684.1	78768.7	58147.3	59699.7
Q91YY2	Beta-1,4-galactosyltransferase 3	B4galt3	395	44.1	9.25	49919.9	47300.7	46035.2	40143.8	45975.5	39930.4	36755.1	50486.4	44525.7	47940.3	49453.8	40958.9	37519.8	37313.3
Q6PDK2	Histone-lysine N-methyltransferase 2D	Kmt2d	5588	599.9	5.8	27574.2	29037.2	29212.6	26734.8	29408.9	29211.9	28341.1	29607.8	23721.0	28290.9	25965.8	27693.4	22059.1	26867.3
Q8VCH5	Rab9 effector protein with kelch motifs	Rabepk	380	41.1	5.95	6484.8	5179.4	10493.1	8969.5	8316.0	8824.5	7737.4	8623.1	10941.2	8848.8	7148.0	9272.2	6041.8	9257.2
Q9CZA6	Nuclear distribution protein nudE homolog 1	Nde1	344	38.5	5.34	15988.6	22105.7	15414.0	18228.6	16495.4	17639.5	15349.6	12990.2	17004.1	20193.4	#N/A	#N/A	#N/A	#N/A
Q05AH6	Spindlin interactor and repressor of chromatin-binding protein	Spindoc	381	41.2	5.17	22197.3	27890.0	18949.2	23417.0	29321.7	33454.2	23183.4	27667.8	21411.2	21504.0	38613.5	49514.6	42347.7	47321.7
Q64323	N-acetylglucosaminyl-phosphatidylinositol biosynthetic protein	Piga	485	54.4	8.27	207828.5	241847.6	276264.9	210152.3	277839.1	320199.2	206198.4	198965.0	238432.7	235273.0	23744.9	32232.9	26870.1	29005.9
Q61508	Extracellular matrix protein 1	Ecm1	559	62.8	6.8	106713.8	111169.1	91245.0	103852.5	101672.5	103013.9	111934.7	97488.6	106979.7	123517.3	254209.3	250654.5	283066.5	268990.8
A0A1Y7VNZ0	WD_REPEATS_REGION domain-containing protein	Dcaf4	532	59.3	8.24	136940.4	130875.6	140959.3	124694.7	141048.3	149547.0	141731.2	134031.0	134377.8	142301.6	154769.8	150202.3	143738.2	138512.7
O88829	Lactosylceramide alpha-2,3-sialyltransferase	St3gal5	414	47.3	8.51	131034.3	119766.5	125052.5	123073.8	123401.1	116790.1	132887.1	126337.3	115732.0	115037.9	305011.8	245034.2	265655.9	242415.6
Q920S3	GATA zinc finger domain-containing protein 1	Gatad1	266	28.5	9.41	123392.5	150029.2	139409.1	150061.2	149594.9	136448.8	125354.2	127148.0	140488.7	154357.8	49815.4	55178.9	48513.6	56439.8
P70459	ETS domain-containing transcription factor ERF	Erf	551	59	7.28	30577.0	35258.2	27447.1	26407.1	32593.4	45392.4	27210.6	33428.2	28993.1	23341.8	#N/A	#N/A	#N/A	#N/A
Q60519	Semaphorin-5B	Sema5b	1093	120.2	7.71	19611.5	22038.6	20061.3	22239.1	23358.4	17652.1	20146.8	19157.6	21631.7	19818.9	#N/A	#N/A	#N/A	#N/A
P97350	Plakophilin-1	Pkp1	728	80.8	8.91	7347.5	9207.7	10583.2	7846.2	23621.5	12589.6	7324.7	12169.9	14413.1	10899.9	4972.4	8515.4	5713.9	5858.4
Q9DAJ5	Dynein light chain roadblock-type 2	Dynlrb2	96	10.9	7.44	80934.0	130828.5	120471.2	187944.2	82214.4	71820.9	89801.9	77824.2	91724.6	87652.4	98932.5	139420.0	131344.2	189631.1
Q8R4Y8	Rotatin	Rttn	2226	248.3	6.52	115031.2	128759.5	94812.8	121268.9	102300.4	107489.0	93027.9	94737.4	111232.7	121680.2	#N/A	#N/A	#N/A	#N/A
P17897	Lysozyme C-1	Lyz1	148	16.8	9.41	273454.8	160908.1	853319.2	192996.7	317781.8	326975.5	244514.4	201175.4	212167.5	193568.2	#N/A	#N/A	#N/A	#N/A
Q69ZB0	Leucine-rich repeat and coiled-coil domain-containing protein 1	Lrrcc1	1026	119.1	5.96	74551.1	95972.3	78460.6	85546.8	96501.5	85848.2	62649.4	66709.7	74591.9	81028.5	#N/A	#N/A	#N/A	#N/A
Q45KJ6	Protein lin-28 homolog B	Lin28b	247	26.9	8.69	84614.5	84300.1	74449.3	76087.7	89701.0	100395.1	74572.7	69146.8	79155.6	77491.3	#N/A	#N/A	#N/A	#N/A
P55041	GTP-binding protein GEM	Gem	295	33.7	8.44	99009.7	95363.1	108202.5	88211.5	142774.8	142458.9	163645.7	113840.3	107054.3	110456.6	#N/A	#N/A	#N/A	#N/A
P97440	Histone RNA hairpin-binding protein	Slbp	275	31.6	6.8	207168.6	250840.6	279223.3	250310.2	256479.6	205162.2	191282.3	201573.7	259561.1	256718.6	229667.7	283189.2	290462.2	277058.8
P54761	Ephrin type-B receptor 4	Ephb4	987	108.8	6.95	8496.2	6145.7	10666.4	9186.9	9769.7	14084.5	11158.2	8931.3	9916.9	12467.2	#N/A	#N/A	#N/A	#N/A
Q9D3Z8	5' nucleotidase, ecto-like	Nt5el	585	65.4	5.4	36158.3	30025.9	27807.2	30467.4	33271.5	34783.8	33295.9	28634.8	34439.6	33290.3	#N/A	#N/A	#N/A	#N/A
P49935	Pro-cathepsin H	Ctsh	333	37.1	8.4	137990.7	181382.5	127446.2	229132.7	147775.4	230204.4	223341.5	176867.9	147545.8	131462.0	249415.4	262410.2	217043.6	314083.3
D3YXL0	Coiled-coil domain-containing 170	Ccdc170	722	82.7	6.32	133051.7	138151.2	124353.4	109791.9	112147.2	126241.8	126362.7	128475.0	132615.6	121701.7	117824.2	124560.4	126648.2	124619.8
Q9CR08	Ribonuclease P protein subunit p29	Pop4	221	25.6	9.74	183998.9	188649.2	192958.3	203056.4	216772.2	211937.5	171569.9	190226.5	195536.9	204537.5	#N/A	#N/A	#N/A	#N/A
O08746	Matrilin-2	Matn2	956	106.7	6.58	60027.7	64666.1	48593.5	62354.2	59374.4	64691.9	65277.8	58450.0	58203.6	65136.5	#N/A	#N/A	#N/A	#N/A
A0A0B4J1H8	Ig-like domain-containing protein	Igkv1-133	120	13.2	7.97	32809.4	31695.7	29915.8	33761.0	52383.5	35751.9	73863.9	27350.1	31925.6	34727.6	29700.2	34088.6	31107.1	33533.8
Q9Z0X4	cGMP-inhibited 3',5'-cyclic phosphodiesterase A	Pde3a	1141	124.4	6.3	86003.1	82335.9	75633.7	81700.2	82843.7	75511.9	82907.7	71457.7	85005.2	84892.6	126449.5	130216.5	116639.4	124764.3
Q9R0B7	Zinc finger protein 346	Znf346	294	32.7	8.88	80520.4	82670.9	75256.4	83886.4	81210.6	87680.6	78217.8	75667.6	76652.9	84257.9	#N/A	#N/A	#N/A	#N/A
Q9JMD1	Scm-like with four MBT domains protein 1	Sfmbt1	863	97.3	6.54	119667.4	137873.9	121973.2	130806.5	136019.0	131420.3	119909.1	111657.2	120473.7	133699.0	#N/A	#N/A	#N/A	#N/A
Q9DB07	Intraflagellar transport protein 46 homolog	Ift46	301	34	4.54	7970.6	11202.4	10082.0	11592.1	9832.6	10030.2	9175.8	7794.3	8937.2	9034.5	35216.3	46762.9	47184.0	56056.4
Q8K3D3	DNA repair protein SWI5 homolog	Swi5	89	10.3	4.77	157246.1	125940.7	137708.4	136971.0	111901.9	121987.2	132308.1	119853.6	151742.4	142124.4	#N/A	#N/A	#N/A	#N/A
Q80XJ3	Tetratricopeptide repeat protein 28	Ttc28	2450	267.3	7.15	47678.8	40845.8	42281.1	41217.4	43694.5	55592.2	56312.4	46406.3	45064.5	44040.7	23574.0	22956.8	25637.7	20171.9
Q9EPR4	Solute carrier family 23 member 2	Slc23a2	648	70	7.5	6877.3	8430.6	6680.7	9310.8	7665.3	5978.1	6365.3	7117.0	5295.1	10181.4	18152.3	18689.8	17325.1	17126.4
Q71FD5	E3 ubiquitin-protein ligase ZNRF2	Znrf2	238	23.7	6.76	75782.9	96402.7	99895.9	98644.9	91659.8	76030.3	74314.9	75110.9	85230.8	86630.7	#N/A	#N/A	#N/A	#N/A
Q80SY4	E3 ubiquitin-protein ligase MIB1	Mib1	1006	110	6.92	27946.3	25653.7	27250.5	23442.2	29153.3	31692.1	26533.2	28449.5	26375.2	28179.2	169718.5	177681.9	164659.8	183728.1
Q8CCI5	RING1 and YY1-binding protein	Rybp	228	24.8	9.63	113729.5	118287.9	108805.3	101227.0	114570.1	145052.4	97598.2	95076.2	103596.2	106994.9	#N/A	#N/A	#N/A	#N/A
P97314	Cysteine and glycine-rich protein 2	Csrp2	193	20.9	8.62	120257.9	132368.4	114608.5	134958.3	95844.4	91528.8	101297.1	113395.6	90028.1	102881.6	174358.6	218573.1	165957.8	197370.0
A0A075B5K6	Ig-like domain-containing protein	Igkv2-109	120	13	7.12	83413.3	114279.6	89363.6	128773.2	106829.1	133442.9	101355.7	102083.4	76118.8	106290.0	#N/A	#N/A	#N/A	#N/A
Q64701	Retinoblastoma-like protein 1	Rbl1	1063	119.4	7.49	242474.2	337614.4	299529.8	300939.2	324784.5	301808.5	249907.1	256737.2	328729.0	349435.6	#N/A	#N/A	#N/A	#N/A
Q3U4G3	Xyloside xylosyltransferase 1	Xxylt1	392	43.8	7.78	37507.6	35040.7	30421.1	36578.3	34974.4	37366.8	36960.0	33987.2	34110.3	34119.8	155688.4	147339.2	147042.8	135882.3
Q8JZX3	POC1 centriolar protein homolog A	Poc1a	405	45.1	7.81	316883.6	372161.8	314823.3	342165.6	347659.3	353681.2	308733.4	285780.6	318032.8	327055.3	#N/A	#N/A	#N/A	#N/A
Q9DD02	Protein Hikeshi	Hikeshi	197	21.6	5.45	294041.8	319902.8	314878.9	308626.4	332318.1	342445.9	320850.2	284606.7	326312.0	317290.0	406197.0	420520.9	438939.5	427737.9
Q8BGN3	Glycerophosphocholine cholinephosphodiesterase ENPP6	Enpp6	440	50.6	7.31	23922.2	27875.3	21654.1	27816.3	24001.8	25752.0	30869.3	22011.1	23745.2	23642.7	20212.8	21954.0	17679.0	15578.7
Q811W1	Alanine and arginine-rich domain-containing protein	Aard	167	18.9	10.89	166086.0	376376.5	184297.3	242132.0	235895.0	307886.4	142994.0	230563.0	196312.7	253598.7	271882.9	272770.8	251781.2	260546.9
D3YVL2	Cilia- and flagella-associated protein 70	Cfap70	1141	128.2	5.53	80781.6	106560.7	90468.6	119457.1	76253.1	66338.0	72899.4	84857.7	87165.2	86056.2	16490.6	20621.0	16786.9	16145.6
Q6ZPI0	Protein Jade-1	Jade1	834	93.8	7.69	236548.7	209671.2	192360.6	186894.5	231579.5	204071.9	210905.7	183459.8	203097.9	222878.7	#N/A	#N/A	#N/A	#N/A
Q8JZZ6	AMMECR1-like protein	Ammecr1l	310	34.5	8.98	98589.8	76116.7	77270.7	77430.2	86286.5	70314.5	67683.3	85943.8	74096.7	72783.1	#N/A	#N/A	#N/A	#N/A
Q9DB98	Leukocyte receptor cluster member 1 homolog	Leng1	261	30.5	9.95	280249.1	290761.7	249553.1	262436.2	242201.1	250019.0	253544.9	230529.2	275409.7	281320.0	21306.6	20189.2	19497.0	23475.4
Q69ZM6	Serine/threonine-protein kinase 36	Stk36	1316	144.1	5.92	30550.6	39395.1	33724.4	37683.0	30221.9	29975.7	26485.1	28976.3	33731.3	29035.3	#N/A	#N/A	#N/A	#N/A
B8JJ69	RIKEN cDNA 2310061I04 gene	2310061I04F	315	35.7	9.28	39216.4	49455.0	43698.0	46716.3	47603.3	49866.4	40275.7	43797.1	48884.4	50392.5	#N/A	#N/A	#N/A	#N/A
Q8R3E3	WD repeat domain phosphoinositide-interacting protein 1	Wipi1	446	48.7	6.46	101354.1	109172.9	100626.1	102855.4	111979.0	131770.3	113308.7	117710.4	106534.7	96977.2	359102.9	330534.3	387414.8	312074.0
Q8CI96	CAP-Gly domain-containing linker protein 4	Clip4	704	75.7	8.62	107019.1	107256.7	92146.8	89592.4	127774.0	124212.3	123970.8	89251.4	111619.7	112673.9	#N/A	#N/A	#N/A	#N/A
Q3URS9	Mitochondrial potassium channel	Ccdc51	406	45.1	8.09	164512.7	150701.0	139070.0	161773.9	145551.9	153600.6	146117.5	146888.5	145908.0	143891.3	226868.5	221974.2	198835.2	217733.1
Q6P3B9	Putative ribosome-binding factor A, mitochondrial	Rbfa	350	39.3	7.33	87689.8	83253.8	68714.9	80277.3	78871.0	80547.7	71110.5	80789.3	71197.3	79854.3	21782.3	29868.8	23767.9	26377.7
Q8CD19	LanC-like protein 3	Lancl3	420	46.4	7.05	222221.4	203526.2	178256.7	205377.8	195543.6	197675.2	195470.0	187062.3	193611.8	191610.7	#N/A	#N/A	#N/A	#N/A
Q99KU6	Bombesin receptor-activated protein C6orf89 homolog	MGI:213678	348	39.5	6.62	267058.1	261967.2	234146.7	242733.5	264624.4	255044.0	233493.4	240306.4	245923.7	249683.6	411290.6	467075.8	421703.8	435977.9
P98191	Phosphatide cytidylyltransferase 1	Cds1	461	52.8	7.72	53025.4	76274.0	54436.6	65850.3	56659.4	55884.0	55120.9	62417.2	56686.0	64796.0	#N/A	#N/A	#N/A	#N/A

Q8BM85	TBC domain-containing protein kinase-like protein	Tbck	762	86.3	6.2	422656.2	458184.7	379530.4	440505.0	461538.4	522442.7	415075.4	381429.9	433164.7	474446.4	173951.3	190277.8	179871.6	178440.3
O70338	Ribonuclease H1	Rnaseh1	285	31.8	9.29	47404.2	46601.3	35583.6	46977.3	44761.2	44947.1	39867.7	34683.4	48285.9	49699.4	#N/A	#N/A	#N/A	#N/A
Q8C025	Cholinephosphotransferase 1	Chpt1	398	44.6	7.08	139497.6	140781.9	127811.4	143884.3	132199.8	125450.5	141581.0	128985.3	128737.6	121863.7	131470.7	134095.1	133833.1	137159.1
P0C913	Overexpressed in colon carcinoma 1 protein homolog	MGI:191703	63	6.4	6.57	53024.0	52678.1	64775.9	61130.9	60647.7	58235.2	67357.9	66966.3	53280.7	50118.2	44396.9	44515.6	52699.9	45532.8
Q9CQV6	Microtubule-associated proteins 1A/1B light chain 3B	Map1lc3b	125	14.6	8.43	290388.0	309000.7	290250.3	278247.5	277870.8	263171.5	286049.9	266317.4	277709.2	290416.4	479558.4	409962.6	493462.4	470968.2
Q78RX3	Small integral membrane protein 12	Smim12	92	10.8	9.04	27605.8	29987.9	26570.0	28935.3	29583.2	28163.7	25315.2	24432.0	28322.8	31097.8	83944.4	90742.1	92759.2	94948.6
B2RUP2	Protein unc-13 homolog D	Unc13d	1085	123	6.62	73108.6	67668.2	59717.4	62593.5	62037.4	63268.7	63759.9	67347.4	71024.3	69730.6	150507.2	184310.6	141043.1	156586.6
Q3V1G4	Olfactomedin-like protein 2B	Olfml2b	746	83.5	4.86	106092.2	118407.7	106183.2	101562.2	109170.0	104816.8	103865.4	115444.2	119121.9	106059.2	#N/A	#N/A	#N/A	#N/A
Q6A037	NEDD4-binding protein 1	N4bp1	893	99.1	5.74	46620.2	43143.8	45625.7	47281.3	44850.4	46690.4	47105.7	41784.3	45160.1	42778.8	73431.1	76941.9	66384.9	71389.0
I7HJI5	SERPIN domain-containing protein	Serpinb9c	387	44.1	6.84	381254.0	373961.5	308631.6	336506.6	324321.7	340979.1	348675.1	295690.4	359177.5	377352.0	160482.3	164244.3	157069.1	153330.0
E9PVB5	Tetratricopeptide repeat protein 17	Ttc17	1198	135.4	6.47	159927.0	180109.8	161880.9	159160.9	175392.6	169928.2	153552.0	163483.6	158037.5	171200.8	#N/A	#N/A	#N/A	#N/A
Q9DBV3	Probable ATP-dependent RNA helicase DHX34	Dhx34	1145	128.4	8.16	213647.3	200928.3	177668.0	197124.2	201953.9	201584.0	190899.7	196981.7	197437.7	236140.7	193632.5	245048.7	196875.1	223680.2
Q9JJD0	THAP domain-containing protein 11	Thap11	305	33.3	8.82	152618.7	163860.9	129724.8	144223.4	155147.4	163191.6	157579.1	140832.9	167901.6	169286.7	242078.9	272335.1	205499.6	226822.6
Q8CIV2	Membralin	Tmem259	574	63.5	5.49	122418.0	149961.1	143540.6	131612.4	138947.6	143983.4	124382.4	126007.7	171629.0	142591.4	91428.0	90864.5	87842.1	86745.7
P98083	SHC-transforming protein 1	Shc1	579	62.6	6.54	165441.7	162437.3	154679.0	168893.6	181752.3	165563.9	165628.9	160046.6	162965.4	162799.4	88105.8	96619.9	87746.2	102607.9
Q91XE4	N-acyl-aromatic-L-amino acid amidohydrolase (carboxylate-forming)	Acy3	318	35.3	5.5	214949.9	200379.3	184179.1	198267.7	204709.8	217736.6	238532.8	212745.7	206305.2	204914.2	124184.0	106006.3	107185.5	112895.4
Q569L8	Centromere protein J	Cenpj	1344	153	6.55	21777.8	21749.4	24280.7	20240.3	23144.2	21180.8	18271.9	20661.2	18427.3	18472.9	56668.2	62770.8	64961.8	57531.5
E9PX14	Coiled-coil domain-containing 152	Ccdc152	254	30.4	9	126656.2	142663.9	127696.9	152759.5	154836.8	135708.3	119650.2	125774.4	136334.2	145404.0	135302.3	144122.1	133020.7	137710.9
Q3UHF3	Mesoderm induction early response protein 3	Mier3	551	61.5	4.58	70158.6	71579.4	95079.9	73835.7	81891.2	83111.3	96333.6	72779.1	82171.0	80842.1	110323.8	120900.6	134602.7	120957.0
Q922M5	Cell division cycle-associated 7-like protein	Cdca7l	438	50.2	7.65	143382.1	147122.0	155878.1	141571.8	175012.8	148748.9	146438.1	142165.4	159413.2	163623.3	#N/A	#N/A	#N/A	#N/A
P15306	Thrombomodulin	Thbd	577	61.8	4.6	45275.6	43422.3	42720.6	42843.6	40728.5	45190.1	43381.2	36861.7	48954.1	54067.0	162499.8	147144.8	172971.0	132374.1
P25916	Polycomb complex protein BMI-1	Bmi1	324	36.7	8.65	126810.1	130261.8	123789.0	124870.4	142304.8	140578.7	126800.9	127227.5	126812.8	124152.5	#N/A	#N/A	#N/A	#N/A
Q9JKF6	Nectin-1	Nectin1	515	57	6.35	132274.2	145544.3	111208.7	123293.9	152702.9	164343.5	159165.6	139930.3	130885.1	140835.1	100046.9	97035.9	90814.4	95873.6
Q80Z25	Oral-facial-digital syndrome 1 protein homolog	Ofd1	1017	117.3	5.83	1412150.9	1686164.2	1752449.9	1739745.5	1749145.1	1557387.7	1564704.5	1492958.1	2051173.2	2049899.4	1625626.5	1901123.1	2190524.6	1832797.1
Q9CY45	EEF1A lysine methyltransferase 1	Eef1akmt1	214	24.5	4.67	23066.6	24085.2	24359.5	28908.8	28134.8	27929.9	26910.2	26711.5	31770.7	26817.5	13389.4	12509.8	12067.3	11865.1
Q8R0F5	RNA-binding motif protein, X-linked 2	Rbmx2	326	37.5	9.72	29057.4	321842.4	27929.1	18294.4	29648.4	36753.8	31279.8	17587.5	39053.4	19716.8	27396.8	41152.5	41138.6	35948.4
E9Q9D5	Rab-like protein 2A	Rabl2	223	25.6	5.66	66543.6	68449.8	69172.0	74122.9	66086.4	56066.7	56453.0	64223.3	62845.1	65671.3	85338.6	90141.8	85519.0	80031.1
Q9QUG3	Prion-like protein doppel	Prnd	179	20.4	9.41	1163998.3	1797875.3	945591.6	2171411.5	912213.8	922481.3	961486.4	1027321.1	962094.2	993637.9	#N/A	#N/A	#N/A	#N/A
H7BX60	Telo_bind domain-containing protein	Pot1b	640	71.6	6.1	23754.6	33631.9	25857.3	27044.2	40504.0	36102.2	25073.6	29090.8	32430.8	32638.3	39943.2	53109.0	40135.9	54281.1
O08573	Galectin-9	Lgals9	353	40	9.31	4390.1	5663.4	4904.4	5971.3	5421.2	7997.4	7079.2	6523.5	6670.8	2858.4	#N/A	#N/A	#N/A	#N/A
Q9ESK3	Calpain-10	Capn10	666	74.5	7.28	35458.2	36723.0	30400.2	31910.3	37826.7	35796.3	28468.7	32180.7	31289.0	34219.9	126787.1	129173.0	138397.6	128514.5
Q6PAQ4	RNA exonuclease 4	Rexo4	432	47.6	9.91	235280.2	239697.8	243838.4	217815.0	252522.9	255715.1	230084.7	230648.1	238764.7	243644.7	#N/A	#N/A	#N/A	#N/A
Q8C726	BTB/POZ domain-containing protein 9	Btbd9	612	69.1	5.68	21467.9	27406.0	23461.1	19246.5	21296.3	22804.4	21850.8	25792.3	20000.7	18130.8	#N/A	#N/A	#N/A	#N/A
Q8BML1	[F-actin]-monooxygenase MICAL2	Mical2	960	110	8.1	199901.2	240602.7	183877.0	222608.9	198843.1	224135.2	218145.2	167176.9	204671.9	205388.5	#N/A	#N/A	#N/A	#N/A
Q921I9	Exosome complex component RRP41	Exosc4	245	26.2	6.15	39466.2	45871.5	34272.1	38316.9	48411.8	38294.0	36837.3	34722.9	43859.4	43081.9	94545.4	85969.4	80956.3	90341.3
Q9CZL5	Pterin-4-alpha-carbinolamine dehydratase 2	Pcbd2	136	14.8	9.16	379151.9	404764.7	374635.3	416980.7	406676.2	397319.2	440608.1	402488.0	394715.4	385374.4	282692.3	282844.9	258683.0	290888.7
Q9DAB5	Histone domain-containing protein	H2bfm	224	26.1	10.14	28163.4	37334.4	42130.1	36091.0	32375.4	32972.1	22980.5	22798.5	44181.4	41049.2	#N/A	#N/A	#N/A	#N/A
Q3V460	Gene model 561, (NCBI)	Smim26	107	12	5.19	25971.2	22466.3	24487.7	21789.0	19525.7	16775.1	22073.1	26840.0	21330.0	20237.2	#N/A	#N/A	#N/A	#N/A
P52019	Squalene monooxygenase	Sqle	572	63.7	8.47	92586.8	99946.1	84149.1	99642.7	86449.7	94548.8	82772.0	93921.5	90971.9	103385.9	#N/A	#N/A	#N/A	#N/A
Q9DCC8	Mitochondrial import receptor subunit TOM20 homolog	Tomm20	145	16.3	8.6	155242.0	152541.7	157897.1	167469.1	155611.2	147462.6	141792.8	144917.5	145221.2	153394.9	606692.5	620697.1	586330.0	589358.4
Q9QXP6	E3 ubiquitin-protein ligase makorin-1	Mkrn1	481	53	5.14	21598.6	27044.0	25850.9	21097.5	23982.7	24462.1	17692.4	15799.0	22094.9	19260.2	6160.2	6504.8	3111.2	4388.7
Q9Z2Y3	Homer protein homolog 1	Homer1	366	41.4	5.53	252315.2	248605.0	225062.1	232330.9	242146.9	228729.0	203281.7	218173.6	230517.8	237587.0	166247.7	177106.5	153961.0	171254.2
Q9CPX7	28S ribosomal protein S16, mitochondrial	Mrps16	135	15.2	9.67	126124.0	129938.8	128552.6	116939.0	137296.6	148410.5	123499.7	125648.9	132712.9	124155.0	337959.6	315225.6	345274.2	350618.8
Q8BPK2	Zinc finger CCHC domain-containing protein 3	Zcchc3	400	43.7	8.44	114778.3	111542.6	100409.8	111306.8	115070.8	109099.7	120033.4	99747.3	106356.9	116917.6	#N/A	#N/A	#N/A	#N/A
O89106	Bis(5'-adenosyl)-triphosphatase	Fhit	150	17.2	6.73	331632.8	305024.9	279445.0	292409.9	291835.5	293717.3	364441.2	300281.4	280136.4	322783.1	#N/A	#N/A	#N/A	#N/A
Q8BLG0	PHD finger protein 20	Phf20	1010	115.2	7.52	25896.5	27301.1	30186.2	29277.5	24817.3	26406.9	23716.5	29995.9	22172.9	27232.4	80559.0	100550.9	89703.9	87674.1
Q9CZH8	Coiled-coil domain-containing protein 77	Ccdc77	489	57.5	8.35	17247.9	23069.2	17990.2	25178.8	20722.0	18563.8	15742.6	16468.7	19375.8	21239.9	234621.6	270546.3	249184.1	275675.4
Q8CJ27	Abnormal spindle-like microcephaly-associated protein homolog	Aspm	3122	364	10.62	15613.3	20278.6	17283.2	16252.1	24519.2	18959.4	17919.5	10924.4	10122.5	23355.6	#N/A	#N/A	#N/A	#N/A
Q9DB70	FUN14 domain-containing protein 1	Fundc1	155	17.1	8.63	46493.0	44429.6	36952.6	37361.5	35582.9	43526.8	39554.1	40760.9	38062.3	38722.2	#N/A	#N/A	#N/A	#N/A
Q60767	Lymphocyte antigen 75	Ly75	1723	197.2	6.39	78539.8	90220.6	70274.5	70347.3	78680.8	86550.3	71099.0	77016.7	81786.4	88438.7	#N/A	#N/A	#N/A	#N/A
Q9ES97	Reticulon-3	Rtn3	964	103.8	4.92	1558239.8	1520367.2	1612882.9	1599011.3	1526893.7	1520060.4	1663257.8	1536342.2	1597678.5	1564766.4	132547.3	121309.0	99111.6	133631.0
Q8CI03	FLYWCH-type zinc finger-containing protein 1	Flywch1	673	77	9.91	90298.4	90060.1	89295.5	93859.8	88681.2	96990.0	101674.9	76152.6	80401.3	91885.2	#N/A	#N/A	#N/A	#N/A
Q8BHN7	Uncharacterized protein C12orf29 homolog	MGI:192119	327	37.4	7.33	131686.0	131251.7	139200.1	127295.1	134808.2	136537.9	135831.3	130581.9	144178.9	130317.5	#N/A	#N/A	#N/A	#N/A
Q9CZ92	Centromere protein P	Cenpp	286	33.3	5.25	87987.1	90284.1	91623.9	107752.1	87051.7	77873.1	67024.6	79194.7	87370.8	91700.6	116483.6	128250.6	120499.1	142244.6
Q9EQQ2	Protein YIPF5	Yipf5	257	27.9	4.36	222282.6	244646.1	237700.4	232015.1	222322.5	212445.7	193562.4	209765.0	208938.6	226286.6	357886.6	354210.3	427707.4	374182.7
P56213	FAD-linked sulfhydryl oxidase ALR	Gfer	198	22.9	7.93	113945.7	117726.0	112742.7	110859.5	120353.3	111976.0	122763.2	104075.6	117152.9	125110.8	443447.9	455779.7	395411.9	454910.0
Q8R179	Kelch repeat and BTB domain-containing protein 4	Kbtbd4	534	59.8	5.74	119453.7	123244.2	102413.9	127820.0	114264.7	109214.1	108022.5	113582.2	113795.7	112829.8	#N/A	#N/A	#N/A	#N/A
Q9DCX1	MAD2L1-binding protein	Mad2l1bp	276	31.1	5.64	32050.6	33273.0	33965.0	40146.7	34467.5	37525.9	34970.8	31921.9	32766.0	36328.4	25802.8	30878.4	26645.7	27726.8
Q80Y32	HMG box domain-containing protein	Hmgxb4	594	65.3	9.38	126870.5	103762.4	116242.1	101818.2	124694.1	141549.0	122678.5	112247.7	118963.1	129203.1	71214.9	82039.9	76356.2	75592.8
O88492	Perilipin-4	Plin4	1403	139.3	8.59	16859.5	12586.3	10172.4	11473.5	24556.4	12852.4	21033.1	17890.5	11489.1	7971.1	156932.1	153928.4	138429.8	135076.3
Q99L85	Elongator complex protein 5	Elp5	300	33.5	5.49	166688.2	217278.9	217508.8	218474.1	206441.8	183177.5	163876.3	179264.2	188366.7	208569.0	27681.7	30272.2	33742.6	35254.4
P06683	Complement component C9	C9	548	62	5.78	10119.1	15894.8	13035.9	16039.4	14768.5	13846.8	12398.2	14826.6	10639.7	14208.5	#N/A	#N/A	#N/A	#N/A
Q9CPW3	39S ribosomal protein L54, mitochondrial	Mrpl54	135	15.4	10.04	552900.8	504215.7	540523.6	531416.4	551726.7	506841.2	659357.8	591726.1	576704.8	530286.8	272303.1	268859.3	271191.7	273499.9
S4R2K0	Peptide deformylase	Pdf	231	25.8	9.06	191887.0	192040.7	203596.8	208860.2	168878.0	202554.2	173857.8	187727.2	183843.3	188358.2	#N/A	#N/A	#N/A	#N/A
Q4G5Y1	Kelch domain-containing protein 2	Klhdc2	406	45.9	6.61	224189.5	232261.0	207058.2	222297.6	212126.4	218170.1	208323.6	195099.0	231110.4	224760.9	259228.1	260291.0	249596.2	241506.0
Q8K4J6	Myocardin-related transcription factor A	Mrtfa	964	102.5	5.5	118122.1	105433.8	106053.4	106984.1	111476.5	103401.2	103375.8	95832.2	116027.7	122776.3	#N/A	#N/A	#N/A	#N/A
Q8CH36	Proton-coupled amino acid transporter 4	Slc36a4	500	55.4	7.05	41636.4	49141.2	36309.9	45180.4	45210.7	40799.1	33889.2	37812.6	39348.9	42689.6	67816.5	93147.0	96805.6	98231.2
Q8BY46	Zinc finger protein 574	Znf574	900	99.4	8.1	126176.6	123629.1	123495.6	118270.7	130007.8	131009.5	112720.2	107851.1	120871.6	124116.4	#N/A	#N/A	#N/A	#N/A
Q8BQ48	Centrosomal protein of 295 kDa	Cep295	2412	272.6	6.06	106309.7	121468.7	113152.7	116562.9	112434.5	119966.9	120356.0	121190.8	133397.4	122819.0	#N/A	#N/A	#N/A	#N/A
P62274	40S ribosomal protein S29	Rps29	56	6.7	10.13	935997.7	1216237.2	964952.4	1204985.2	939134.2	909208.2	1037099.0	972094.3	1128025.3	1147776.6	3320272.2	2972353.8	3000739.5	3061244.1
Q3THJ3	Probable RNA-binding protein EIF1AD	Eif1ad	170	19.5	4.87	49933.5	60796.6	33159.5	50616.6	51443.6	55400.1	42223.4	41723.2	59479.6	60243.0	#N/A	#N/A	#N/A	#N/A
O70472	Transmembrane protein 131	Tmem131	1877	204.5	8.54	70125.9	71506.5	72788.5	68683.8	74051.1	74353.9	71545.9	70002.2	74559.4	73197.1	102026.7	123436.0	107820.8	116252.0
P48381	Transcription factor RFX3	Rfx3	749	83.5	6.07	39018.3	32662.9	32734.6	36622.3	27988.4	32128.9	33617.8	38758.5	33790.6	30035.9	#N/A	#N/A	#N/A	#N/A
Q61805	Lipopolysaccharide-binding protein	Lbp	481	53	8.5	140566.8	143075.0	119809.5	131141.5	132975.1	143364.4	126381.1	130848.4	130602.9	146635.5	#N/A	#N/A	#N/A	#N/A
Q64FW2	All-trans-retinol 13,14-reductase	Retsat	609	67.3	8.95	14765.3	15203.5	11246.4	11950.5	11633.4	14370.1	11958.5	10806.8	11515.8	11874.6	22602.6	19599.8	15167.9	17308.9
Q3TQR0	Post-GPI attachment to proteins factor 2	Pgap2	254	29.4	8.18	30414.8	28920.3	21808.2	34047.3	23796.9	25657.1	21707.7	35505.9	24435.4	27151.7	198570.5	198131.1	177327.3	187154.4
Q9JIT1	RING finger protein 32	Rnf32	368	42.1	8.56	25716.1	31499.6	26116.7	33728.7	25637.2	30710.4	28174.5	27747.3	29089.8	30378.5	#N/A	#N/A	#N/A	#N/A
Q8CAE9	Podocalyxin-like protein 2	Podxl2	603	64.9	4.36	47030.9	51922.4	48188.5	41469.3	50666.3	56383.9	45865.9	37441.7	42191.7	48637.6	#N/A	#N/A	#N/A	#N/A
Q99KS6	Manganese-dependent ADP-ribose/CDP-alcohol diphosphatase	Adprm	340	38.9	5.49	34338.5	34653.6	30832.1	26796.9	35281.7	38908.8	33649.0	36151.9	40149.1	36733.5	23470.1	24261.5	20011.7	23985.5
Q8K387	Ubiquitin carboxyl-terminal hydrolase 45	Usp45	813	90.3	8.12	35318.9	38895.7	39378.0	39685.7	38153.1	42119.0	32758.3	33351.6	44973.0	37797.3	#N/A	#N/A	#N/A	#N/A
Q91XF0	Pyridoxine-5'-phosphate oxidase	Pnpo	261	30.1	8.22	626790.7	525698.4	614031.1	595317.5	707428.6	649458.4	745568.6	614589.8	680179.8	662472.2	155789.0	150929.8	150573.0	145098.9
Q8BUV6	U7 snRNA-associated Sm-like protein LSm11	Lsm11	361	39.9	10.73	178667.9	168113.3	180136.0	179067.3	175075.3	182624.4	157384.3	154615.5	166675.2	173877.4	#N/A	#N/A	#N/A	#N/A
Q9Z176	Serine/threonine-protein phosphatase 2A regulatory subunit B'' subunit delta	Ppp2r3d	491	55.7	5.1	70763.2	71193.0	64314.4	65626.5	72215.4	67202.2	67127.6	65312.9	75562.6	69565.1	34381.6	44115.9	32093.3	33277.3
Q9CZ83	39S ribosomal protein L55, mitochondrial	Mrpl55	127	15.1	10.51	122562.3	138804.2	108927.2	121881.4	122533.7	122492.7	102804.5	112738.2	120205.1	125840.3	189263.3	152734.2	202401.6	166468.4
P43883	Perilipin-2	Plin2	425	46.6	6.87	103056.9	136244.8	106937.4	109005.4	130227.6	111450.9	106673.3	112577.4	117241.0	116363.1	#N/A	#N/A	#N/A	#N/A
P53349	Mitogen-activated protein kinase kinase kinase 1	Map3k1	1493	161.2	7.9	22783.5	22002.6	25109.0	20722.6	19284.8	19844.7	21434.9	20638.4	22580.4	18516.4	26973.0	27421.8	28944.5	32896.3
Q60862	Origin recognition complex subunit 2	Orc2	576	65.9	6.81	168873.6	176570.1	158154.1	171708.0	171935.2	175299.7	169562.8	161832.9	166991.5	177119.4	144290.0	161449.1	143701.1	133554.4
Q8K4I7	Inactive serine protease 45	Prss45	317	35.7	7.18	146965.1	139835.5	152498.9	160706.0	124321.6	122930.2	113932.6	116945.7	147009.1	140845.2	#N/A	#N/A	#N/A	#N/A
P28574	Protein max	Max	160	18.2	6.3	93984.4	87061.9	75639.4	76140.1	80469.7	81557.0	93245.8	69709.4	82872.6	84461.8	#N/A	#N/A	#N/A	#N/A
P51569	Alpha-galactosidase A	Gla	419	47.6	5.72	96650.4	96079.4	99996.7	96522.6	102155.4	102859.1	106375.1	86880.1	98537.9	95192.8	340211.7	362721.2	348825.3	324379.6
Q3U1F9	Phosphoprotein associated with glycosphingolipid-enriched microdomains 1	Pag1	429	46.5	4.81	62760.7	70939.4	49651.2	61244.8	64533.9	69095.9	54721.7	62143.6	65988.3	59675.9	56888.2	59817.8	54774.5	57506.9
Q9CQW0	ER membrane protein complex subunit 6	Emc6	110	12	10.07	134317.6	152349.4	143865.5	133728.1	158960.7	151562.7	142013.3	130448.8	153212.6	144397.8	209801.7	237352.5	207072.9	207188.0

Q91WZ8	Dysbindin	Dtnbp1	352	39.6	4.69	126718.2	114599.9	116562.2	127842.8	117878.8	118933.6	109836.7	112965.7	120421.1	129747.9	293184.1	318195.7	324310.8	334358.9
P47810	Wee1-like protein kinase	Wee1	646	71.5	6.2	62410.5	70056.1	72109.2	60874.9	74375.4	72410.1	73817.2	58267.1	65184.8	69825.0	#N/A	#N/A	#N/A	#N/A
A0A075B5N7	Ig-like domain-containing protein	Igkv6-13	95	10.4	6.51	83437.6	33714.5	33054.7	31375.1	51336.8	37296.6	43732.9	74070.9	43163.0	39949.2	45687.1	26752.0	20147.9	25718.5
Q9JHJ8	ICOS ligand	Icoslg	322	35.9	7.91	131711.8	157743.8	132679.7	124611.6	166016.1	159574.4	187571.0	133206.8	125773.5	144877.7	77771.3	71311.5	62877.7	68556.5
Q9EPW0	Inositol polyphosphate-4-phosphatase type I A	Inpp4a	939	105.5	7.05	69436.6	76497.9	53910.6	70761.0	59314.8	68665.5	61427.9	50990.3	60843.9	66412.2	69440.9	75482.8	47604.9	64972.5
Q3US41	Epithelial splicing regulatory protein 1	Esrp1	680	75.5	6.58	55147.1	55492.7	47164.3	42593.9	45839.5	47904.7	41049.5	49429.7	46376.9	42315.4	6583.0	8451.5	8830.5	9558.6
Q9CQ89	Protein CutA	Cuta	177	18.9	6.77	84819.4	86300.0	89885.8	85633.7	91317.1	82701.6	72650.1	93535.1	82033.3	80014.8	#N/A	#N/A	#N/A	#N/A
A0A075B5M7	Ig-like domain-containing protein	Igkv5-39	95	10.3	6.25	67888.5	67161.6	97533.6	75679.4	86782.8	68857.4	75260.4	70613.7	80183.1	86224.9	40175.8	35734.4	61063.9	43942.6
P26187	Methylated-DNA--protein-cysteine methyltransferase	Mgmt	211	22.4	7.85	99847.2	116093.1	106623.3	121054.2	125351.6	124240.8	119962.2	112253.2	108721.2	125507.7	#N/A	#N/A	#N/A	#N/A
P70182	Phosphatidylinositol 4-phosphate 5-kinase type-1 alpha	Pip5k1a	546	60.4	8.59	246824.4	281247.9	261859.3	253803.1	283367.9	266640.8	248186.8	254497.5	251343.9	270060.1	139498.0	143278.7	107656.9	151357.5
A2A7B5	PR domain-containing 2, with ZNF domain	Prdm2	1709	187.1	8	34358.7	36273.4	34008.9	33553.1	37543.8	38935.5	32633.4	36845.1	36385.6	31576.3	#N/A	#N/A	#N/A	#N/A
Q8BSA9	tRNA wybutosine-synthesizing protein 3 homolog	Tyw3	257	28.6	7.78	104994.7	100544.4	98130.6	92440.5	104365.6	104176.1	94835.2	85366.7	108323.2	98098.8	71246.4	82131.3	72526.5	77581.2
Q8R1Q9	Ribokinase	Rbks	323	34.1	5.47	9554.4	7728.9	9894.0	9135.6	8449.6	8890.7	6397.6	7829.4	9315.8	9926.0	#N/A	#N/A	#N/A	#N/A
Q9DBW3	Protein NATD1	Natd1	110	12.7	8.47	241893.3	198150.0	241194.5	199215.9	241122.3	191750.2	208269.6	210686.7	208548.8	239981.9	#N/A	#N/A	#N/A	#N/A
Q922B1	ADP-ribose glycohydrolase MACROD1	Macrod1	323	35.3	8.85	124654.6	120084.1	126137.5	125215.8	139246.1	144668.9	205762.0	146956.8	140668.2	140675.6	67624.4	76413.6	69597.0	82329.8
Q9D2D9	Kelch domain-containing protein 8B	Klhdc8b	354	37.6	8.09	307021.0	297635.0	307284.3	303679.9	303697.7	295018.5	302595.0	335762.9	309917.9	324047.8	#N/A	#N/A	#N/A	#N/A
Q6NZQ6	Zinc finger protein 740	Znf740	180	20.9	9.19	55817.8	56753.4	55865.7	49315.6	64575.5	65550.1	80552.8	63707.3	62494.3	63926.5	#N/A	#N/A	#N/A	#N/A
Q5SS90	Uncharacterized protein C7orf57 homolog	Gm11992	291	32.9	8.68	24908.9	30498.3	34318.5	32143.5	27353.1	27749.3	25523.5	29194.7	31317.8	30394.3	#N/A	#N/A	#N/A	#N/A
P56394	Cytochrome c oxidase copper chaperone	Cox17	63	6.8	7.69	152772.3	174884.9	171747.3	182594.7	147563.6	150464.4	141797.0	154292.1	176747.6	142970.9	317507.2	369169.2	396841.9	408555.7
Q5NCQ5	2-(3-amino-3-carboxypropyl)histidine synthase subunit 1	Dph1	438	48	6.84	22019.0	27593.9	26220.2	28205.7	25605.8	27708.4	24738.1	21415.5	26016.7	24292.8	256759.7	240538.6	243445.1	270425.7
O54974	Galectin-7	Lgals7	136	15.2	7.25	46240.6	43758.9	37148.3	37918.3	48322.1	53897.5	65241.3	65820.9	42832.5	47041.3	46593.3	36205.4	37830.6	36791.4
P21180	Complement C2	C2	760	84.7	7.56	290475.9	305621.3	225525.6	255881.3	198651.8	242116.1	257532.3	260381.4	241295.8	262688.2	#N/A	#N/A	#N/A	#N/A
Q6NZQ4	PAX-interacting protein 1	Paxip1	1056	119.2	7.2	169550.1	154159.4	150355.2	150376.5	189316.1	180879.8	188820.7	152416.1	154941.7	156477.3	59188.8	52789.0	49802.0	54880.4
Q08369	Transcription factor GATA-4	Gata4	441	44.6	9.26	57622.3	49809.8	65576.9	44119.7	73579.2	55127.3	72316.8	50059.8	51939.4	64626.1	32678.1	30803.9	32075.2	31187.1
P63139	Nuclear transcription factor Y subunit beta	Nfyb	207	22.8	4.59	47827.6	47858.7	51824.6	51481.5	57949.7	57035.8	55250.7	50368.3	52086.1	51057.6	#N/A	#N/A	#N/A	#N/A
E9PUQ8	Diacylglycerol kinase	Dgkd	1220	135.1	7.81	71856.3	60865.0	62352.8	62690.8	61972.7	64912.4	61367.6	62598.4	60878.7	55897.0	20511.6	23368.5	25562.7	24163.7
G5E8P1	Bromodomain-containing protein 1	Brd1	1058	119.6	8.7	153617.4	160377.4	165770.1	160400.9	158675.7	166948.2	164263.4	142429.6	156785.4	166794.4	#N/A	#N/A	#N/A	#N/A
Q3UUG6	TBC1 domain family member 24	Tbc1d24	561	63.2	7.24	48778.6	52175.0	57977.6	55928.0	56099.8	58091.4	63462.6	61117.2	52954.3	51892.1	#N/A	#N/A	#N/A	#N/A
Q6P9P0	SMC5-SMC6 complex localization factor protein 2	Slf2	1278	143.9	9.28	13306.3	13187.0	13219.7	13454.8	18054.7	15834.8	14159.3	11270.5	16577.4	17405.4	#N/A	#N/A	#N/A	#N/A
Q9D9V4	Radial spoke head protein 9 homolog	Rsph9	276	31.3	5.41	33346.0	35575.4	28466.6	31392.4	28067.9	32330.0	27334.7	27633.5	27667.8	28304.7	186537.2	214248.0	213847.7	258561.9
O35427	DNA-directed RNA polymerase III subunit RPC9	Crcp	148	16.7	5	116992.7	126913.9	109774.7	118819.1	122354.7	125501.7	103873.4	106810.1	122634.9	111164.7	#N/A	#N/A	#N/A	#N/A
O88509	DNA (cytosine-5)-methyltransferase 3B	Dnmt3b	859	97.2	8.31	165007.9	179577.5	156649.1	152016.1	196237.5	185242.5	156887.1	169068.3	178158.9	172285.4	142792.2	159115.7	141777.0	124068.9
Q923Z0	G-protein coupled receptor family C group 5 member B	Gprc5b	410	45.9	8.38	41396.5	54078.8	44478.5	49416.9	48411.0	47887.5	46387.1	48142.6	49615.1	47815.7	#N/A	#N/A	#N/A	#N/A
Q8BXK9	Chloride intracellular channel protein 5	Clic5	251	28.3	5.94	40281.9	74841.8	36446.0	52442.4	46014.6	51621.7	46103.1	49108.7	45828.5	50737.9	#N/A	#N/A	#N/A	#N/A
B1AR13	CDGSH iron-sulfur domain-containing protein 3, mitochondrial	Cisd3	137	15.7	9.79	745054.7	670977.7	795040.9	703412.9	763949.2	769725.7	860019.8	728400.2	757019.7	699780.1	119442.6	125627.7	100958.2	103284.5
E9Q634	Unconventional myosin-Ie	Myo1e	1107	126.7	9.07	217114.3	253618.8	182149.0	230923.5	191322.8	194975.1	187189.8	211272.7	192119.2	220099.1	39337.7	50899.7	38309.8	44250.6
Q8BQX5	Transmembrane and coiled-coil domain-containing protein 6	Tmco6	494	54.9	6.11	102129.7	129199.2	98083.5	112991.3	120205.2	114361.6	104888.2	101536.8	101711.5	121736.9	145836.0	169684.7	131306.2	149851.2
Q8CFH6	Serine/threonine-protein kinase SIK2	Sik2	931	104.1	6.13	62567.8	58367.7	55024.0	53186.6	62198.1	56543.2	61632.7	46763.1	56041.8	53891.4	80270.4	97359.2	92989.8	88332.4
Q9Z0M6	Adhesion G protein-coupled receptor E5	Adgre5	818	90.4	7.39	70718.9	103423.8	88780.5	111902.2	134470.4	104133.9	92224.1	104598.9	106489.4	99547.2	92793.5	83342.5	76271.3	83985.1
E9PYK3	Protein mono-ADP-ribosyltransferase PARP4	Parp4	1969	216	6.07	112421.1	115078.5	100521.2	105803.2	116773.8	140317.7	119225.4	111413.2	103622.2	117826.4	161882.3	182410.5	146348.8	160070.1
Q9JJF9	Signal peptide peptidase-like 2A	Sppl2a	523	58.1	7.06	78385.8	86261.8	72489.8	71571.7	76831.2	81792.0	81359.6	79492.6	69184.2	81464.9	80029.7	83126.1	76762.0	70157.6
O35099	Mitogen-activated protein kinase kinase kinase 5	Map3k5	1380	154.4	5.78	76605.5	65057.0	65396.0	64846.7	66366.5	69981.4	65835.5	60103.0	72357.6	69471.6	#N/A	#N/A	#N/A	#N/A
Q9CQV1	Mitochondrial import inner membrane translocase subunit TIM16	Pam16	125	13.8	9.64	65223.4	65846.3	66719.3	68291.0	66717.7	70740.4	62729.3	65901.9	66584.4	67865.6	121382.1	132070.6	148957.3	127706.5
O55242	Sigma non-opioid intracellular receptor 1	Sigmar1	223	25.2	5.91	210457.3	248536.3	213282.5	211172.2	227531.8	232197.5	218395.9	221413.2	204105.3	221823.0	540792.4	553371.1	520698.6	510341.7
Q8BGX1	PC-esterase domain-containing protein 1B	Pced1b	433	49.9	8.28	96659.0	96319.7	108502.5	104215.7	123699.3	112534.8	104801.8	94826.1	112960.5	103704.8	85321.8	101168.0	95007.3	89813.2
Q9D273	Corrinoid adenosyltransferase	Mmab	237	26.3	9.2	193786.0	201211.6	183256.3	187250.0	217218.8	215173.8	197769.9	209124.7	199743.5	193427.2	183838.0	199387.3	190650.8	177196.5
E9Q137	Testis-expressed gene 264	Tex264	309	33.6	5.4	65862.4	62849.0	58059.1	64620.2	65202.7	65396.5	60678.3	63661.9	62973.3	60584.9	51098.4	57077.9	61030.2	46926.5
Q8VDT9	39S ribosomal protein L50, mitochondrial	Mrpl50	159	18.2	9.33	392767.3	400764.9	300342.3	397670.7	339080.3	334997.8	364356.0	302640.3	363839.4	384948.6	208794.6	229635.9	185461.8	256498.9
Q9DCB1	High mobility group nucleosome-binding domain-containing protein 3	Hmgn3	99	10.8	9.7	27766.2	21879.1	31263.7	27028.6	46330.6	45342.4	46464.1	31503.0	34513.5	32509.9	8822.9	6645.8	9040.9	9568.3
E9Q4Y4	Centrosomal protein 192	Cep192	2514	276.2	5.53	173301.6	171219.0	157508.1	162612.4	167089.5	186401.7	182839.4	147993.9	174856.4	170883.9	#N/A	#N/A	#N/A	#N/A
Q9CWY9	RPA-interacting protein	Rpain	219	24.9	4.97	51018.1	56894.8	55191.9	50401.1	62513.3	63089.8	49817.3	52665.0	51592.7	60897.8	#N/A	#N/A	#N/A	#N/A
P58006	Sestrin-1	Sesn1	492	56.6	6.06	35846.5	52806.2	39500.8	42248.6	39492.3	41734.5	39077.7	43908.4	43462.3	38068.9	100150.2	89816.1	77433.6	89860.3
Q61070	Etoposide-induced protein 2.4	Ei24	340	38.9	9.72	345831.2	357955.7	313042.0	375259.4	335694.1	299073.1	328160.0	330000.1	331074.2	352810.1	#N/A	#N/A	#N/A	#N/A
Q8BLY7	Hermansky-Pudlak syndrome 6 protein homolog	Hps6	805	87.3	6.8	25581.2	23309.9	24285.3	21431.1	22105.8	24431.7	24952.6	21235.4	26486.0	25635.9	25640.0	20966.2	24027.8	18867.2
Q9CQJ7	Securin	Pttg1	199	21.7	6.6	102048.0	100274.8	108519.0	107370.7	103028.7	102703.5	99094.6	93792.8	117486.6	106228.1	#N/A	#N/A	#N/A	#N/A
Q7TSG1	Centrosomal protein of 120 kDa	Cep120	988	112.5	5.94	64509.3	110449.6	63590.3	81361.7	78016.7	71385.9	67521.7	69461.2	81080.1	91919.4	#N/A	#N/A	#N/A	#N/A
Q6NXN1	SUZ domain-containing protein 1	Szrd1	152	17	9	52366.5	48207.5	55329.4	53463.3	44610.7	47223.2	48971.6	63218.2	63880.4	53211.8	92793.5	90578.4	85594.5	98723.4
O70404	Vesicle-associated membrane protein 8	Vamp8	101	11.4	8.19	240225.7	221976.1	206842.0	210306.9	229697.4	197934.7	289187.7	235879.1	240581.2	212011.1	137603.9	124935.7	117826.0	129178.3
Q9JKP8	Chromatin accessibility complex protein 1	Chrac1	129	14.1	5.2	81027.5	94844.5	82121.0	87488.5	84050.1	74900.2	71612.8	72873.7	81283.4	89587.1	680645.2	815973.8	883497.4	916712.1
O70131	Ninjurin-1	Ninj1	152	16.5	6.55	19807.9	19239.5	20193.0	18335.9	20263.4	20437.2	19251.5	15567.3	20053.4	20384.4	35077.4	33355.2	30844.7	34174.1
Q9DAK2	Parkin coregulated gene protein homolog	Pacrg	241	27.7	8.72	53201.8	58041.2	54625.4	76984.8	46094.6	37284.9	76270.6	47120.6	42754.5	55563.5	36423.7	39868.7	40960.0	56952.8
O08715	A-kinase anchor protein 1, mitochondrial	Akap1	857	92.1	5.02	61869.6	81075.6	73537.4	77268.0	74275.7	75319.9	79244.2	68768.7	73511.2	79526.4	#N/A	#N/A	#N/A	#N/A
P56379	ATP synthase subunit ATP5MPL, mitochondrial	Atp5mpl	58	6.7	9.99	1683225.1	1631403.3	1702471.1	1779902.2	1700653.3	1514590.3	1669510.1	1702143.8	1722972.3	1599164.1	280306.4	322843.4	316989.6	271930.4
Q8BL48	RING finger protein unkempt homolog	Unk	810	88	6.87	59084.2	50496.3	38966.5	46143.2	44666.9	44124.2	48045.4	45889.0	45168.9	44481.9	#N/A	#N/A	#N/A	#N/A
Q9R097	Kunitz-type protease inhibitor 1	Spint1	507	56.6	5.57	230618.9	269855.0	256745.2	222181.8	234045.4	252744.4	217015.1	232908.3	256839.9	249087.4	45536.4	63680.0	54560.8	52035.2
E9Q236	ATP-binding cassette, sub-family C (CFTR/MRP), member 4	Abcc4	1325	148.8	8.28	112495.0	117672.2	126665.4	122041.1	136616.8	127798.8	115221.9	117479.7	103404.6	106457.3	42463.9	49974.9	45766.6	48725.5
Q8K2M0	39S ribosomal protein L38, mitochondrial	Mrpl38	380	45	8.1	435392.1	463676.6	443559.5	435029.8	462088.4	457188.3	423624.7	434777.3	443493.9	433416.3	368417.6	346664.8	327676.8	358303.8
Q80U30	Protein CLEC16A	Clec16a	1036	116.2	6.05	387865.7	364474.3	427624.4	385741.5	401837.5	392478.9	345627.5	393132.2	389469.3	385553.6	185916.2	195440.7	199210.3	195808.3
D6MZJ6	Sphingomyelin phosphodiesterase 5	Smpd5	483	53.8	8.65	253674.6	280252.1	268826.9	283835.4	215949.1	227546.6	217361.0	221149.7	246190.7	284029.2	#N/A	#N/A	#N/A	#N/A
Q69Z98	Serine/threonine-protein kinase BRSK2	Brsk2	735	81.7	8.79	155943.2	178053.7	168700.8	155717.1	174050.4	174213.9	165344.0	147494.9	162885.9	178641.7	#N/A	#N/A	#N/A	#N/A
Q8CFT2	Histone-lysine N-methyltransferase SETD1B	Setd1b	1985	215.2	4.96	8569.4	7859.6	4464.2	5834.7	8224.5	7316.5	6226.8	4270.0	5377.7	4433.5	#N/A	#N/A	#N/A	#N/A
E9Q6K3	Progesterone immunomodulatory-binding factor 1	Pibf1	756	89.5	6.16	119076.5	125837.1	109539.1	120739.7	105551.0	111012.2	116158.3	115452.1	113433.6	122833.4	91211.0	92929.6	85957.3	94533.6
P10078	Zinc finger protein 28	Zfp28	825	93.3	9.17	74863.6	86331.8	71071.2	78405.5	86212.7	95106.3	96270.3	70330.8	88118.9	115965.3	#N/A	#N/A	#N/A	#N/A
Q3UH06	Ras-responsive element-binding protein 1	Rreb1	1700	184	6.73	68063.4	78050.6	79141.5	78760.7	82272.4	74607.8	79236.1	85162.4	78764.2	72917.0	#N/A	#N/A	#N/A	#N/A
Q9CWQ8	Cytosolic arginine sensor for mTORC1 subunit 1	Castor1	331	36.6	4.98	15187.6	16804.9	13747.6	16486.7	15550.3	15617.6	12109.5	15428.8	14858.5	17320.4	#N/A	#N/A	#N/A	#N/A
Q8BH50	Uncharacterized protein C18orf25 homolog	1GI:244495	245	26.4	5.19	30919.6	38790.3	34204.4	37031.8	41040.5	38195.1	41023.6	30974.1	45058.6	42350.4	49608.0	56078.7	52058.2	46198.8
Q91XB7	Protein YIF1A	Yif1a	293	32.1	9.09	20186.2	21372.5	19087.6	23581.6	21892.9	16092.7	20285.4	20149.6	21957.0	22752.5	#N/A	#N/A	#N/A	#N/A
P04202	Transforming growth factor beta-1 proprotein	Tgfb1	390	44.3	8.62	30742.8	39797.2	27262.3	37928.1	33570.9	29195.9	25073.6	33228.1	27557.6	34326.3	#N/A	#N/A	#N/A	#N/A
Q8CFY5	Protoheme IX farnesyltransferase, mitochondrial	Cox10	443	48.9	9.06	102181.0	125977.4	112245.9	120061.1	119590.4	116162.7	102970.8	93473.9	128593.4	103340.3	#N/A	#N/A	#N/A	#N/A
P48441	Alpha-L-iduronidase	Idua	634	71.2	6.73	14451.5	15242.4	12443.2	15039.3	17790.8	16698.6	14118.6	13065.8	17476.4	13825.2	#N/A	#N/A	#N/A	#N/A
P00397	Cytochrome c oxidase subunit 1	Mtco1	514	56.9	6.7	102352.8	113037.1	98674.3	106465.3	111632.3	104271.8	120381.5	99723.3	128267.3	114953.0	406626.4	424233.4	416882.5	452636.2
Q6ZPK0	PHD finger protein 21A	Phf21a	688	75.5	9.48	43654.0	48165.8	53632.9	49414.1	55838.7	61384.3	51173.2	45583.4	49278.7	56024.1	#N/A	#N/A	#N/A	#N/A
Q8C2A2	tRNA-splicing endonuclease subunit Sen54	Tsen54	525	59	6.16	69566.9	85954.2	70906.4	72260.8	86639.8	77271.8	72917.7	73510.1	69020.0	76342.3	36809.4	34486.6	34352.9	35790.2
Q80UG1	Fatty acid desaturase 6	Fads6	342	38.8	8.46	36557.2	34525.5	49813.4	31802.5	53126.1	52343.8	61090.7	48763.5	57628.5	49613.7	86013.7	80118.5	95013.0	89828.5
Q91ZS8	Double-stranded RNA-specific editase 1	Adarb1	711	78	8.94	37031.2	36051.9	32513.1	31731.3	36068.7	42734.0	34714.8	33264.9	42971.1	41881.4	#N/A	#N/A	#N/A	#N/A
E9Q8V6	DENN/MADD domain-containing 4A	Dennd4a	1869	209.2	7.56	5604.9	8925.6	5485.9	5039.9	5767.5	7863.6	5426.6	5480.2	2424.5	5708.3	36487.7	39066.2	37545.6	41924.1
A2AHY8	WD_REPEATS_REGION domain-containing protein	Dcaf8l	747	83.2	4.73	46808.8	59433.7	46676.7	50645.0	61960.0	59073.5	40571.5	43503.4	53869.0	63542.0	122765.1	112118.2	120355.8	107786.7
Q8VCC1	15-hydroxyprostaglandin dehydrogenase [NAD(+)]	Hpgd	269	29.2	6.54	260985.0	203725.1	161462.1	215857.4	171444.8	256054.8	201722.0	250889.6	210911.2	177210.1	#N/A	#N/A	#N/A	#N/A
P70704	Phospholipid-transporting ATPase IA	Atp8a1	1164	131.3	6.84	71050.5	69910.4	72418.6	69762.5	69037.4	72809.5	66490.5	72728.2	74246.7	71538.8	#N/A	#N/A	#N/A	#N/A
P70345	Bcl-2-like protein 2	Bcl2l2	193	20.8	5.58	112997.0	131548.7	106857.2	124633.3	115570.1	129387.0	115389.7	106514.2	114292.7	123030.8	#N/A	#N/A	#N/A	#N/A
Q499X9	Methionine--tRNA ligase, mitochondrial	Mars2	586	65.8	8.09	11287.7	14653.3	11829.8	14225.6	16203.2	14502.2	12068.5	11247.7	12006.9	12834.6	113541.7	122299.9	108071.1	112106.5
Q8BY02	NF-kappa-B-repressing factor	Nkrf	690	77.7	8.97	67656.9	63840.5	60604.0	66210.8	70848.9	64188.8	64082.5	73516.2	69339.0	58834.4	#N/A	#N/A	#N/A	#N/A

Q9D067	Nuclear protein MDM1	Mdm1	708	79.6	9.48	131758.5	133161.6	158558.0	142609.1	166634.1	172574.1	184775.3	136238.6	157610.3	161526.2	#N/A	#N/A	#N/A	#N/A
Q9CXX9	CUE domain-containing protein 2	Cuedc2	284	31.8	4.98	42259.8	44295.7	43695.5	41222.1	47892.1	46683.6	43254.6	42392.3	45334.7	46107.1	#N/A	#N/A	#N/A	#N/A
D3YVV7	NOVA alternative-splicing regulator 2	Nova2	556	55.6	8.43	530322.3	510696.5	521020.1	492438.5	639501.1	673153.3	881599.7	527419.0	665765.5	668407.9	25270.7	25233.0	23960.2	18635.6
Q8BK08	Transmembrane protein 11, mitochondrial	Tmem11	190	21.3	7.36	138424.5	161826.6	180931.3	143699.4	167748.3	157302.2	144215.9	169436.1	152498.9	151750.5	69397.7	101045.0	82376.3	80803.6
Q9D531	Nucleoredoxin-like protein 2	Nxnl2	156	17.6	7.34	41731.6	39806.8	38728.7	42196.7	36025.7	37162.8	36375.5	39451.3	42565.6	46684.3	61700.4	65763.7	50362.3	67256.4
Q62086	Serum paraoxonase/arylesterase 2	Pon2	354	39.6	5.83	256489.8	257786.4	249465.9	296246.9	248675.7	242508.6	263244.1	255144.8	252653.7	245444.2	36950.4	33189.8	31639.5	40127.2
Q91ZH7	Phospholipase ABHD3	Abhd3	411	46.2	7.49	165129.5	153712.1	162840.6	161202.3	153198.1	167386.8	145682.3	156750.1	155225.5	157588.1	190941.1	187339.7	191565.1	196855.9
Q505F4	C2H2-type domain-containing protein	Zfp280b	534	59.5	6.7	76829.2	91656.3	81701.1	75803.1	85602.1	92033.3	85271.9	66398.3	89491.5	93434.2	#N/A	#N/A	#N/A	#N/A
P43024	Cytochrome c oxidase subunit 6A1, mitochondrial	Cox6a1	111	12.3	9.98	169773.2	147027.9	156744.8	166129.8	169912.6	165650.3	154389.5	147158.1	192546.4	186362.7	83987.7	102483.6	88100.4	88643.4
Q9JJH1	Ribonuclease 4	Rnase4	148	17	8.85	73956.0	77718.9	80490.0	77118.9	101470.9	124703.6	113560.5	79505.0	78630.4	81253.9	37377.4	40049.8	45231.5	36935.6
Q8R1F0	Leydig cell tumor 10 kDa protein homolog	D8Ertd738e	94	10.2	11.63	268450.6	281291.5	263168.8	267826.7	271706.8	287537.1	312155.2	238672.9	303400.6	289146.5	#N/A	#N/A	#N/A	#N/A
Q9DBA6	Peroxisomal leader peptide-processing protease	Tysnd1	568	59	7.23	11428.4	13905.3	11676.8	13089.5	14779.1	15114.5	15176.8	14445.6	9314.6	12297.7	2868.7	5703.8	3840.5	6408.0
P01831	Thy-1 membrane glycoprotein	Thy1	162	18.1	8.97	65512.7	61033.0	64122.5	68233.4	79454.9	115216.3	144313.6	122641.1	81235.1	72344.6	#N/A	#N/A	#N/A	#N/A
Q9QXW9	Large neutral amino acids transporter small subunit 2	Slc7a8	531	57.8	6.67	278972.7	277138.8	213879.9	336037.1	220628.0	191818.6	188092.4	220417.1	212425.1	208377.0	#N/A	#N/A	#N/A	#N/A
O35450	FK506-binding protein-like	Fkbpl	347	38.3	7.74	320312.7	308745.8	320587.4	322512.3	329247.3	324686.9	286316.1	302478.9	331074.9	349887.9	108987.0	108322.6	104645.6	104137.8
Q8C4M7	Centromere protein U	Cenpu	410	46.3	9.66	128853.0	149389.6	152195.9	144886.2	140140.3	137061.1	127216.7	126376.8	141490.8	152790.2	#N/A	#N/A	#N/A	#N/A
Q9CZB3	THUMP domain-containing protein 2	Thumpd2	528	57.6	7.06	50049.6	64626.3	56082.5	50310.6	64882.9	63365.3	59628.0	55298.2	49642.9	52176.6	#N/A	#N/A	#N/A	#N/A
Q9CQI4	Intraflagellar transport-associated protein	Iftap	244	27.6	4.68	103994.1	120743.4	122835.9	136056.7	99413.9	93186.2	89450.8	94659.9	107412.6	111893.0	165507.0	234642.8	186727.9	246958.0
Q8BKJ9	NAD-dependent protein deacetylase sirtuin-7	Sirt7	402	45.1	9.58	109457.1	135173.0	114629.6	107417.1	133141.7	132295.4	124338.9	123888.6	114572.1	121982.5	#N/A	#N/A	#N/A	#N/A
Q9ERL9	Guanylate cyclase soluble subunit alpha-1	Gucy1a1	691	77.5	7.17	244232.5	271344.3	228201.9	236337.2	247602.7	272311.8	231774.9	247239.0	255956.2	247487.4	45610.5	55578.6	51158.2	49155.8
Q3U9N9	Monocarboxylate transporter 10	Slc16a10	512	55.3	8.06	31207.1	39047.3	34745.1	39153.7	43355.9	36373.6	38704.2	38851.8	36871.7	36336.1	#N/A	#N/A	#N/A	#N/A
Q7TMX5	Protein SHQ1 homolog	Shq1	569	63.4	4.83	61833.4	59809.3	59611.2	68418.8	58023.6	55510.4	56500.6	58064.3	54598.5	58124.7	23980.4	26316.6	28269.6	25152.9
Q8R0C0	DNA-directed RNA polymerase III subunit RPC7-like	Polr3gl	218	25.1	4.53	22135.3	31770.1	40961.8	32712.4	40830.0	45777.9	36567.3	32423.4	42774.5	39035.8	#N/A	#N/A	#N/A	#N/A
Q9WVG9	Male-specific lethal 3 homolog	Msl3	525	60.3	9.55	52121.9	44104.6	53700.9	49203.7	49083.5	61759.4	59625.1	46249.0	58432.3	48886.9	31887.2	29316.6	18651.7	24080.9
Q80UN1	BTB/POZ domain-containing protein KCTD9	Kctd9	339	37	6.25	45370.3	49264.4	42404.1	45293.7	46949.1	45981.9	38482.1	36327.7	40841.1	50735.7	#N/A	#N/A	#N/A	#N/A
P32848	Parvalbumin alpha	Pvalb	110	11.9	5.19	178457.5	89902.7	71918.4	72541.6	70569.1	74008.1	126252.2	376604.0	67592.8	65194.1	440852.7	282170.4	219973.3	223463.7
Q9EQJ0	Two pore calcium channel protein 1	Tpcn1	817	94.4	8.53	295330.6	353182.5	258647.5	309781.0	285328.5	298300.9	263822.1	283779.1	267581.3	274233.3	109284.6	125876.3	95585.1	106238.4
P61600	N-alpha-acetyltransferase 20	Naa20	178	20.4	5.03	87431.0	114391.3	101615.3	115817.9	108249.8	106564.3	101608.9	97987.6	101290.5	106232.2	29434.6	33855.0	31634.2	32071.2
Q91YL7	PGAP2-interacting protein	Cwh43	699	78.1	8.91	35451.2	33134.0	36003.4	80064.0	38788.9	50499.5	36185.4	94440.6	39614.1	30306.6	#N/A	#N/A	#N/A	#N/A
Q9JJN5	Carboxypeptidase N catalytic chain	Cpn1	457	51.8	8.28	105982.8	114863.3	107243.6	107537.1	109604.7	115191.0	121707.7	117106.0	92055.5	114529.4	87234.6	95246.0	78393.9	81798.6
Q9QY73	Transmembrane protein 59	Tmem59	323	36.3	4.87	103183.8	106853.2	83904.7	88656.4	97871.3	108794.3	100559.2	80789.7	111094.0	110479.8	#N/A	#N/A	#N/A	#N/A
Q91WC1	Protection of telomeres protein 1	Pot1	640	70.8	7.06	18693.1	19287.3	18537.2	15044.1	23047.4	15127.2	18350.9	15932.9	21770.5	16687.9	#N/A	#N/A	#N/A	#N/A
Q9DBU5	E3 ubiquitin-protein ligase RNF6	Rnf6	667	74	9.44	27343.9	32082.0	26361.2	29855.9	31409.3	27232.7	27483.7	25502.2	27920.4	36602.1	135003.1	146695.7	144870.9	137748.7
O88413	Tubby-related protein 3	Tulp3	460	51.2	6.24	288499.4	256588.1	267675.4	265656.4	269021.7	282996.4	281639.3	261162.6	293134.9	302181.5	11549.3	11869.1	13087.5	10216.4
Q3UFB2	Box C/D snoRNA protein 1	Znhit6	460	52.2	5.57	42057.7	39064.2	41010.5	38105.6	46438.2	44817.2	38127.3	41536.5	38036.6	46303.6	#N/A	#N/A	#N/A	#N/A
Q925N0	Sideroflexin-5	Sfxn5	342	37.3	9.44	27245.6	34823.2	30276.6	29283.3	30675.2	36350.1	34115.3	33863.8	37180.0	29099.6	#N/A	#N/A	#N/A	#N/A
O88978	Protein tilB homolog	Lrrc6	473	55	5.72	81947.0	93949.7	83223.4	97964.6	87693.0	70002.3	66105.9	67228.2	90316.7	93617.0	57036.9	81473.4	74668.4	82807.9
Q62136	Tyrosine-protein phosphatase non-receptor type 21	Ptpn21	1176	133.4	7.68	305473.6	296319.0	250624.9	285725.2	241687.7	230657.8	238628.1	257652.1	268657.8	261129.7	137529.6	152345.4	145970.6	159327.9
B5TVM2	Immunoglobulin-like domain-containing receptor 2	Ildr2	661	73.2	7.68	154279.5	130559.9	144761.0	123696.4	202534.2	203016.4	197802.8	151391.5	147330.3	144437.8	249482.1	226823.3	244796.5	182977.1
Q6DFV3	Rho GTPase-activating protein 21	Arhgap21	1944	215.6	7.64	78204.4	86126.1	68420.0	76287.3	78119.1	80372.8	77076.9	73355.1	78016.0	73961.0	173402.2	174610.7	159949.5	157129.4
P62077	Mitochondrial import inner membrane translocase subunit Tim8 B	Timm8b	83	9.3	5.12	96140.1	102098.9	90562.4	105889.6	97081.4	94874.9	86913.6	91712.0	97769.5	97819.2	#N/A	#N/A	#N/A	#N/A
Q922H9	Zinc finger protein 330	Znf330	316	35.6	6.16	130335.3	119872.4	120367.2	115441.8	126379.0	117500.8	107671.7	113007.5	113495.6	121392.6	83837.5	92533.5	92122.1	87924.2
Q60992	Guanine nucleotide exchange factor VAV2	Vav2	868	99.9	6.84	148466.8	223673.6	146947.4	192465.7	195668.8	224191.3	171697.6	165351.1	174780.9	155619.3	47960.6	54706.6	55935.1	47435.0
Q8R2H9	Phosphoethanolamine/phosphocholine phosphatase	Phospho1	267	29.9	7.94	17267.1	20231.3	15236.8	15495.4	17093.3	17092.8	15086.5	16062.1	15991.2	16862.5	38045.6	37159.9	36895.1	36438.9
Q8VE96	Solute carrier family 35 member F6	Slc35f6	372	41	7.14	53944.7	66229.7	54388.0	65287.9	58281.8	49771.1	48619.0	59261.5	54290.4	53511.1	104557.5	117151.8	113736.9	139472.8
Q9Z1B3	1-phosphatidylinositol 4,5-bisphosphate phosphodiesterase beta-1	Plcb1	1216	138.3	6.13	21129.5	28528.2	17679.2	26352.3	24220.2	29218.2	19029.9	25774.4	20453.9	24898.6	14257.0	23068.2	17820.6	19296.0
Q3U0Y2	Transmembrane protein 35B	Tmem35b	150	16.2	9	4708.8	7779.8	7624.8	9933.0	6978.9	7894.1	5268.8	8209.8	8425.3	5657.1	2408.5	3488.5	4266.4	4532.1
Q6PEV3	WAS/WASL-interacting protein family member 2	Wipf2	440	46.3	10.99	174808.9	199391.0	175471.2	173710.0	178906.0	180491.0	159925.8	166424.2	175392.5	190069.8	#N/A	#N/A	#N/A	#N/A
Q8VD57	Vesicle transport protein SFT2B	Sft2d2	159	17.5	9.09	121816.8	101806.6	98373.1	94926.9	131589.9	144400.4	116826.1	118166.1	105906.7	98231.7	#N/A	#N/A	#N/A	#N/A
Q8K394	Inactive phospholipase C-like protein 2	Plcl2	1128	125.7	6.92	107461.8	92590.7	125347.8	84608.8	127540.2	130635.7	121346.6	121700.4	115253.6	123251.2	#N/A	#N/A	#N/A	#N/A
Q99L90	Microspherule protein 1	Mcrs1	462	51.7	9.32	31919.7	37934.3	46413.2	33600.8	50748.0	44907.5	41641.4	37663.1	43214.6	39468.0	68080.1	81820.6	79811.5	64825.6
Q9DB52	Protein FAM122A	Fam122a	284	30.3	6.79	28966.8	41373.5	33142.5	38554.5	35806.7	45238.4	39489.5	35600.7	24636.2	31084.7	#N/A	#N/A	#N/A	#N/A
P16294	Coagulation factor IX	F9	471	52.9	5.44	36011.0	35075.7	34849.3	35591.4	36649.0	36902.0	32587.6	30949.1	37527.5	33910.9	#N/A	#N/A	#N/A	#N/A
Q9CZ96	Zinc finger CCHC-type and RNA-binding motif-containing protein 1	Zcrb1	217	24.6	8.54	41808.0	47610.5	47968.1	52047.3	48541.5	51659.9	47536.4	46911.1	42522.5	43277.2	82328.8	84590.7	78794.6	79067.6
Q5SV77	Gametogenetin-binding protein 2	Ggnbp2	696	78.9	6.47	35514.4	45106.8	37243.7	44600.8	41965.9	42494.1	37790.4	36928.6	32844.7	35502.9	57311.1	60102.7	60025.8	66851.0
P61080	Ubiquitin-conjugating enzyme E2 D1	Ube2d1	147	16.6	7.42	77863.0	154442.2	61598.9	88067.7	76956.6	83725.5	61591.7	97437.8	76917.8	75608.9	#N/A	#N/A	#N/A	#N/A
O35719	DNA repair protein RAD51 homolog 2	Rad51b	350	38.1	7.39	87698.3	110243.6	93582.0	92087.4	104860.0	107118.6	94290.6	91350.9	110855.5	111519.6	#N/A	#N/A	#N/A	#N/A
Q91X85	Feline leukemia virus subgroup C receptor-related protein 2	Flvcr2	551	60.1	6.42	8124.0	9577.1	8068.8	10415.9	7736.4	8787.6	9358.1	6636.6	10408.1	9507.2	34544.1	29107.9	27730.1	29848.9
Q920G9	Germ cell-less protein-like 1	Gmcl1	524	59.6	6.84	42238.5	52335.6	45163.0	48117.4	48771.6	42217.1	45280.8	47747.3	45832.5	44778.3	#N/A	#N/A	#N/A	#N/A
Q8BGD6	Sodium-coupled neutral amino acid transporter 9	Slc38a9	560	63.4	7.5	72846.4	75509.0	65135.5	81275.7	70893.7	69364.2	68022.8	66338.4	74468.4	68568.4	#N/A	#N/A	#N/A	#N/A
Q9R088	Thymidine kinase 2, mitochondrial	Tk2	270	31.2	7.21	28846.5	34373.8	39627.2	34212.3	40355.7	34809.4	36126.3	33290.9	39705.2	39081.0	#N/A	#N/A	#N/A	#N/A
Q9D8B6	Protein FAM210B, mitochondrial	Fam210b	190	20.3	10.51	256813.9	320339.2	242859.5	298534.8	267896.2	249802.4	267481.7	231516.8	243217.3	247198.4	780751.0	753192.2	625494.6	788411.0
Q8VE65	Transcription initiation factor TFIID subunit 12	Taf12	161	17.9	7.99	91261.0	95058.0	103012.8	95389.9	105551.6	122895.5	123971.0	125644.5	150039.3	94294.8	22872.8	26628.9	26562.0	20584.4
D3Z3Y1	Phosphatidylinositol glycan anchor biosynthesis, class G	Pigg	983	108.4	7.75	43424.0	48793.7	45903.5	51152.4	46333.5	43044.2	46671.7	46642.2	47240.4	52679.8	#N/A	#N/A	#N/A	#N/A
Q62083	PRKCA-binding protein	Pick1	416	46.6	5.52	115505.2	129153.2	136430.0	130328.2	133683.4	124176.4	108977.3	121341.4	127588.5	122134.7	80175.5	93679.8	89506.8	94896.9
E9Q9T0	Fibrosin-like 1	Fbrsl1	1031	110.7	8.7	50706.7	74489.5	45899.1	54568.9	54816.4	53831.9	49790.9	42712.6	57104.6	64621.0	#N/A	#N/A	#N/A	#N/A
Q8BVY4	SPT3, SAGA and STAGA complex component	Supt3	374	41.2	8.54	69445.9	61821.1	59028.5	67931.6	62316.9	66756.0	69803.9	56517.7	64536.6	61087.2	#N/A	#N/A	#N/A	#N/A
Q9CWF6	Bardet-Biedl syndrome 2 protein homolog	Bbs2	721	79.9	5.86	151935.0	160971.4	177223.2	192856.3	147037.7	158482.2	145563.8	134814.9	172580.5	184975.4	#N/A	#N/A	#N/A	#N/A
Q8VCE1	DnaJ homolog subfamily C member 28	Dnajc28	385	44.8	8.65	91310.0	79515.3	77948.9	70890.7	91143.9	97629.4	101621.8	90482.9	86631.8	92913.2	76678.9	73275.0	65094.2	67577.3
Q9DBF7	Pre-mRNA-splicing factor CWC25 homolog	Cwc25	416	48.8	10.23	280631.9	233335.5	257733.6	276108.7	277027.7	257141.8	280778.5	297681.0	281870.8	283767.9	#N/A	#N/A	#N/A	#N/A
Q8R395	COMM domain-containing protein 5	Commd5	224	24.5	8.06	330904.0	321951.8	315671.7	327039.5	341513.3	366616.5	388438.3	328933.4	376731.6	397376.8	#N/A	#N/A	#N/A	#N/A
Q9D4J7	PHD finger protein 6	Phf6	364	41.1	8.73	53887.7	41254.5	54727.0	46717.9	48527.5	48792.8	50757.2	45550.3	52871.3	47845.5	54560.4	56324.4	58130.8	53993.5
P70389	Insulin-like growth factor-binding protein complex acid labile subunit	Igfals	603	66.9	6.6	143132.7	144272.7	132914.0	130293.2	107224.0	110780.2	127714.9	129226.3	119168.4	108655.1	#N/A	#N/A	#N/A	#N/A
Q8CG79	Apoptosis-stimulating of p53 protein 2	Tp53bp2	1128	125.2	6.34	49867.4	58453.4	53029.6	48987.6	51821.3	55256.4	54490.5	52566.4	52473.3	54284.2	50114.2	47793.2	52856.4	46074.1
Q9D2P4	Ubiquitin-related modifier 1	Urm1	101	11.3	4.74	346833.2	328916.9	296082.0	339285.0	337445.3	292645.1	276089.2	281376.2	305848.6	353547.4	101425.2	93931.4	92438.3	117277.3
Q8BHL8	Proteasome inhibitor PI31 subunit	Psmf1	271	29.6	5.25	101959.9	111025.4	96265.5	97182.1	101083.5	104342.1	94299.1	89800.1	102213.2	110107.6	144175.4	167495.5	145896.6	158675.3
E9PYL2	Proline-rich protein 12	Prr12	2035	211.7	7.65	165185.7	170426.7	147306.1	154867.9	174037.2	166343.5	174909.0	155719.1	161658.2	169774.4	113130.1	115530.6	99987.8	101359.1
E9Q6E9	SUMO-interacting motifs-containing 1	Simc1	1354	146.5	6.39	184690.1	176223.5	193003.5	159554.6	174357.4	193764.5	190080.3	175068.8	193100.8	179071.8	179660.6	188089.6	202696.3	183977.0
Q99LJ0	CTTNBP2 N-terminal-like protein	Cttnbp2nl	638	69.8	7.71	77434.9	85978.8	70093.1	71818.8	89181.6	83120.7	79327.9	89532.4	71914.4	82821.0	#N/A	#N/A	#N/A	#N/A
Q9WVL1	AP-4 complex subunit sigma-1	Ap4s1	144	16.8	5.34	255049.4	346645.9	287522.6	390609.5	263717.6	273104.7	236917.6	231218.9	289438.4	302806.0	#N/A	#N/A	#N/A	#N/A
Q9CQ00	Distal membrane-arm assembly complex protein 1	Dmac1	111	11.3	9.39	250841.6	274842.7	271362.7	289669.4	199832.8	200454.7	208151.6	217210.3	238608.8	238092.0	#N/A	#N/A	#N/A	#N/A
Q9D563	Transmembrane protein 114	Tmem114	222	24.4	7.93	681745.3	621322.0	663982.7	599056.9	728695.6	701989.6	737709.8	696944.8	675368.0	680483.0	551910.5	477622.6	519562.5	508482.4
Q9D0P0	Emopamil-binding protein-like	Ebpl	206	23.3	6.89	147818.1	203883.3	133259.1	181735.4	155947.1	152222.4	182606.3	130643.7	182171.5	202014.7	#N/A	#N/A	#N/A	#N/A
Q80VA5	SH3 domain and tetratricopeptide repeat-containing protein 2	Sh3tc2	1289	145.1	5.92	95848.1	116718.8	107439.9	107311.3	114965.5	105558.9	102329.0	109153.4	107392.7	112117.1	#N/A	#N/A	#N/A	#N/A
Q9CSV6	Vesicle transport protein SFT2C	Sft2d3	212	21.9	10.26	94339.7	97402.1	109000.2	107025.0	106122.1	93982.5	98031.3	93849.3	101358.2	98151.9	283166.2	258383.1	215410.2	267175.5
P56564	Excitatory amino acid transporter 1	Slc1a3	543	59.6	8.4	16117.7	13634.4	13759.2	14225.6	14043.3	14368.8	10630.8	13597.4	13919.1	10115.2	#N/A	#N/A	#N/A	#N/A
Q8BUB4	WD repeat and FYVE domain-containing protein 2	Wdfy2	400	45.1	6.81	78175.4	72368.2	69037.4	68563.8	88680.2	80186.0	95616.2	68211.3	65700.2	78465.1	#N/A	#N/A	#N/A	#N/A
Q8BPT6	Mitochondrial inner membrane protease subunit 2	Immp2l	175	19.6	9.54	24675.3	25256.7	28314.3	24890.4	29645.2	32169.8	31150.6	25410.3	35184.7	28371.5	19248.7	27901.9	22598.5	20916.0
Q3UD01	Ataxin-7-like protein 3B	Atxn7l3b	97	10.7	4.32	84964.1	90126.2	81598.0	83872.5	94067.4	97637.5	87403.9	84954.4	87195.8	97217.8	#N/A	#N/A	#N/A	#N/A
Q9EST1	Gasdermin-A	Gsdma	446	49.6	5.57	59706.6	64975.1	71611.4	67751.7	88994.3	90459.9	63857.8	71188.0	81615.7	76290.5	#N/A	#N/A	#N/A	#N/A
Q9CPN9	Peptidase S1 domain-containing protein	10010C04I	247	26.4	7.93	143901.2	154055.7	137551.7	143065.8	148931.0	154297.3	151103.8	143127.1	151295.7	157622.1	201943.0	196370.0	188955.5	172024.9
P51912	Neutral amino acid transporter B(0)	Slc1a5	553	58.4	7.84	134601.4	137129.0	132240.4	116851.3	120223.5	123269.0	119200.2	119159.4	131008.9	123356.9	114471.9	98214.0	92549.9	105398.7

Q9DA80	Radial spoke head protein 3 homolog B	Rsph3b	389	45.1	6.1	78703.7	82715.2	77876.0	92850.5	70240.0	63827.4	79384.2	73623.0	76886.0	80553.9	212673.2	270211.3	251875.2	314143.1
Q9R0X5	X-linked retinitis pigmentosa GTPase regulator	Rpgr	1001	111.7	4.61	72361.4	74203.0	74819.8	67806.2	74015.5	81843.3	77684.2	66563.9	74037.1	66476.3	178822.6	177664.2	176778.4	187681.9
Q9WVD5	Mitochondrial ornithine transporter 1	Slc25a15	301	32.8	8.85	100687.8	113217.1	82249.5	111073.6	94441.0	103289.9	97968.9	98261.6	98456.0	108378.1	#N/A	#N/A	#N/A	#N/A
Q9JHE7	Protein TSSC4	Tssc4	317	33.5	5.14	443319.7	444368.5	450095.9	460806.5	476501.8	485784.5	457076.9	421154.7	439192.0	469505.9	1222913.4	1190646.8	1285334.7	1164772.3
A2AS37	Anaphylatoxin-like domain-containing protein	AI182371	356	40.4	6.52	33187.7	35452.3	32117.1	31402.3	26398.4	31051.9	29926.9	37811.1	34616.1	32251.4	#N/A	#N/A	#N/A	#N/A
Q7TQ48	Sarcalumenin	Srl	910	99.1	4.46	50281.2	56613.5	47179.0	49828.8	52592.9	52899.0	52335.0	114557.1	52588.7	54252.7	#N/A	#N/A	#N/A	#N/A
P70245	3-beta-hydroxysteroid-Delta(8),Delta(7)-isomerase	Ebp	230	26.2	7.56	852337.8	831230.7	816458.0	832008.6	830424.0	761238.6	842046.5	787596.5	864223.4	850924.2	#N/A	#N/A	#N/A	#N/A
Q9D0V8	Cyclin-dependent kinase 2-interacting protein	Cinp	212	24.1	6.43	169422.7	205544.1	167074.3	165330.5	192334.5	207326.4	162673.1	183148.9	182378.6	191073.6	#N/A	#N/A	#N/A	#N/A
Q9QUP5	Hyaluronan and proteoglycan link protein 1	Hapln1	356	40.5	7.8	24805.0	17735.7	14513.0	20009.9	16489.8	18963.9	13183.5	18841.3	16318.9	13475.1	69874.8	67567.5	62142.8	58914.7
Q3TB48	Transmembrane protein 104	Tmem104	496	55.8	7.12	101986.0	87620.8	95318.6	91873.2	84599.9	88287.0	90479.9	89673.5	109584.9	89870.2	#N/A	#N/A	#N/A	#N/A
O88207	Collagen alpha-1(V) chain	Col5a1	1838	183.6	4.98	2027.9	2032.3	2594.6	1564.6	3325.1	3185.6	1320.5	1960.5	40862.6	3021.8	#N/A	#N/A	#N/A	#N/A
Q9D799	Methionyl-tRNA formyltransferase, mitochondrial	Mtfmt	386	43.1	9.42	136904.3	163763.9	126442.8	145974.2	140056.7	133472.3	117908.1	120368.0	130085.4	146697.6	#N/A	#N/A	#N/A	#N/A
Q9WV03	Protein FAM50A	Fam50a	339	40.2	6.83	62869.4	69641.2	74490.1	67151.4	76734.6	86528.1	80769.3	74305.4	74374.1	76404.9	83646.2	85192.1	86378.7	97138.3
Q9D5K4	S100P-binding protein	S100pbp	396	44.4	5.6	278580.4	342994.1	301195.8	294147.3	329516.6	341117.4	289661.3	277635.0	323655.5	334928.4	357151.8	369227.2	321582.3	359090.2
Q6NZM9	Histone deacetylase 4	Hdac4	1076	118.5	6.92	329474.3	330494.8	324907.5	311324.3	358681.0	364147.6	342825.6	365388.2	372389.6	329989.1	98091.3	92327.6	92321.9	88643.9
Q8BGR2	Volume-regulated anion channel subunit LRRC8D	Lrrc8d	859	98.1	7.44	37213.2	33066.2	33016.0	32440.1	33518.9	35902.6	34188.3	35274.6	32471.7	36834.9	58083.6	54973.4	50162.1	50425.7
P30051	Transcriptional enhancer factor TEF-1	Tead1	426	47.9	8.15	94253.6	93423.1	90519.4	81703.3	102010.6	118316.8	110936.6	95638.1	98595.3	104827.6	104081.1	98018.2	100601.1	92040.0
Q8BGS3	Zinc finger protein with KRAB and SCAN domains 1	Zkscan1	561	63.4	7.36	136014.4	119245.2	122117.5	111639.5	142950.4	137757.4	151832.3	109613.9	131546.7	129641.3	#N/A	#N/A	#N/A	#N/A
P59266	Fat storage-inducing transmembrane protein 2	Fitm2	262	30	9	183710.6	189936.1	173333.0	182606.3	174287.0	180354.4	177136.7	175559.1	183358.9	189835.8	#N/A	#N/A	#N/A	#N/A
Q61169	Transcription factor GATA-6	Gata6	589	59.3	8.31	68107.0	60494.3	57138.7	60366.4	63044.5	60130.6	71062.7	75919.7	52929.9	70146.8	52136.9	60263.6	53840.2	48614.3
Q9DCM7	Nucleus accumbens-associated protein 2	Nacc2	586	63.2	5.9	35977.8	39230.6	39053.6	39587.5	38426.4	38048.9	32278.5	34551.3	44969.2	34845.5	14685.9	19059.2	13549.1	14664.0
Q8VDB2	Dol-P-Man:Man(7)GlcNAc(2)-PP-Dol alpha-1,6-mannosyltransferase	Alg12	486	54.5	9.33	157272.7	164629.3	154154.1	157051.7	160218.8	167654.9	153816.8	145387.1	156715.1	170383.6	24690.0	22794.2	25861.0	30767.0
A2AAY5	SH3 and PX domain-containing protein 2B	Sh3pxd2b	908	101.5	8.66	49461.2	67582.4	61997.6	52271.2	63963.9	83002.5	61243.2	59178.0	57093.4	61258.7	#N/A	#N/A	#N/A	#N/A
Q3UFS4	G patch domain-containing protein 11	Gpatch11	262	30.6	5.08	247020.8	313225.2	254088.3	262064.2	284854.3	271190.0	232881.6	261522.4	271983.9	283194.9	40888.4	42233.4	46429.8	34277.2
Q8R143	Pituitary tumor-transforming gene 1 protein-interacting protein	Pttg1ip	174	20	8.63	373578.2	395781.3	421740.2	410788.4	440170.6	397865.4	389365.2	413848.5	409596.4	421706.3	68458.3	64170.5	72808.9	73801.9
Q6ZQ29	Serine/threonine-protein kinase TAO2	Taok2	1240	139.2	7.06	161156.5	150107.0	164479.9	158204.7	179211.1	187894.2	155571.0	156847.0	172339.8	151126.0	99420.2	110274.3	107701.7	113534.3
P70187	Hippocampus abundant transcript 1 protein	Mfsd14a	490	53	8.4	25388.0	27543.3	24102.0	25632.1	23914.4	24012.2	19265.6	22484.4	17901.5	24594.2	#N/A	#N/A	#N/A	#N/A
Q9JM51	Prostaglandin E synthase	Ptges	153	17.3	9.39	120856.8	110844.5	94278.0	137310.5	120462.4	138634.9	124734.6	172101.2	93936.0	98297.5	203702.2	203464.4	193819.6	226686.8
Q5XPI3	E3 ubiquitin-protein ligase RNF123	Rnf123	1314	148.6	6.7	256233.6	286236.1	260224.7	282421.5	257256.9	242983.4	244302.7	229823.5	251661.3	301179.3	154951.2	167523.8	154141.1	168691.5
Q61048	WW domain-binding protein 4	Wbp4	376	42.1	7.36	66164.6	69992.5	54048.3	66313.0	55446.3	60217.4	57714.1	64099.3	66191.5	67396.6	32744.2	20383.4	15486.8	26586.3
Q9Z2C9	Myotubularin-related protein 7	Mtmr7	660	75.6	6.43	74442.9	94221.4	77130.7	84948.4	88869.4	92635.9	82070.9	78203.1	80979.7	83504.5	#N/A	#N/A	#N/A	#N/A
Q3TFD2	Lysophosphatidylcholine acyltransferase 1	Lpcat1	534	59.7	6.34	182163.4	177236.1	158521.0	175274.3	176945.0	186684.1	177856.9	172317.8	170742.7	175680.8	121409.6	139508.0	154450.3	148933.8
Q80VJ2	Steroid receptor RNA activator 1	Sra1	232	25.5	6.43	41921.7	45004.4	40260.9	47432.2	49683.2	47558.5	42173.4	48344.2	44094.5	45111.3	#N/A	#N/A	#N/A	#N/A
Q03311	Cholinesterase	Bche	603	68.4	7.25	181692.1	210523.9	190539.2	266010.7	205087.0	247813.0	190726.5	220793.7	203999.0	173519.9	136424.9	142426.2	155922.4	147835.9
A2ARZ3	Fibrous sheath-interacting protein 2	Fsip2	6995	784.4	6.51	57257.0	59798.2	59198.9	66711.7	57048.9	81204.6	54429.5	47125.7	61955.5	63885.6	116745.0	94384.8	107430.5	96541.4
P58465	CTD small phosphatase-like protein	Ctdspl	276	31.1	5.69	39355.6	43281.1	44928.6	38929.0	46113.1	48079.1	45136.8	46612.2	43244.9	41516.0	#N/A	#N/A	#N/A	#N/A
Q9CR21	Acyl carrier protein, mitochondrial	Ndufab1	156	17.4	5.21	67255.9	63358.3	70283.3	78270.4	79043.2	59443.8	71662.1	64412.5	72009.6	67165.4	#N/A	#N/A	#N/A	#N/A
E9Q286	Little elongation complex subunit 1	Ice1	2241	242.1	5.48	47190.8	44472.5	43074.1	41386.6	50657.9	50033.2	47402.3	48890.9	38157.3	53324.8	115987.6	119557.6	116258.8	119614.6
Q9D4B2	Tetratricopeptide repeat protein 25	Ttc25	624	71.5	6.65	146081.4	189130.8	177383.1	248113.2	131840.8	131077.5	157335.2	145519.5	171570.8	171713.5	#N/A	#N/A	#N/A	#N/A
P62878	E3 ubiquitin-protein ligase RBX1	Rbx1	108	12.3	6.96	878068.0	1124173.4	993502.3	1129514.2	992350.1	929768.9	958227.5	872192.6	1099604.2	1139092.6	1053202.9	1274806.4	1160239.7	1313727.8
Q66JQ7	Kinetochore scaffold 1	Knl1	1612	179.2	5.9	24220.6	42570.5	29195.8	34440.9	24816.2	39523.4	29358.6	24997.4	23901.7	38833.1	31516.7	35554.9	33578.7	38163.7
Q9WV30	Nuclear factor of activated T-cells 5	Nfat5	1534	165.7	5.19	146762.2	115037.7	118806.5	118019.0	130441.3	124043.5	149511.1	116769.2	120706.6	139592.5	#N/A	#N/A	#N/A	#N/A
O88425	Nucleoside diphosphate kinase 6	Nme6	189	21.8	8.32	53391.5	56106.7	60313.4	58818.9	68720.9	73618.0	71644.5	55426.7	67355.1	61416.3	#N/A	#N/A	#N/A	#N/A
Q8C0I4	Enhancer of polycomb homolog 2	Epc2	808	90.9	8.73	58096.5	52241.7	58787.6	49788.2	57409.7	61146.7	51826.8	49877.6	55617.6	48424.4	#N/A	#N/A	#N/A	#N/A
Q8C4X7	Major intrinsically disordered NOTCH2-binding receptor 1-like homolog	Minar2	193	21.8	9.36	29591.7	33597.5	26024.8	27704.9	26427.2	30740.2	26286.4	29715.4	25225.7	28272.9	#N/A	#N/A	#N/A	#N/A
Q8BYW9	EGF domain-specific O-linked N-acetylglucosamine transferase	Eogt	527	61.4	6.81	267260.3	286576.3	240807.3	285857.5	256596.6	324952.0	249145.2	251626.9	251820.2	256689.9	140669.2	147325.6	115294.6	134621.4
Q99JI1	Musculoskeletal embryonic nuclear protein 1	Mustn1	82	8.9	9.64	133207.6	163691.7	154383.0	155688.7	184829.4	229805.9	271878.1	151757.2	168041.2	182365.7	#N/A	#N/A	#N/A	#N/A
P70347	TRAF family member-associated NF-kappa-B activator	Tank	448	50.9	5.88	155061.6	170558.0	163182.6	180278.6	185445.2	192742.8	159453.1	150034.3	165477.2	183143.3	#N/A	#N/A	#N/A	#N/A
Q8BKU8	Transmembrane protein 87B	Tmem87b	555	62.9	6	85952.7	91690.5	84083.6	81644.8	83536.9	89396.4	74782.4	84158.5	85507.3	83304.0	316802.8	320741.8	300582.6	297868.5
Q2VPA6	Helicase POLQ-like	Helq	1069	119	6.64	7598.2	11516.7	7185.9	8864.7	7792.4	6810.5	6468.6	7249.5	6797.8	8303.4	#N/A	#N/A	#N/A	#N/A
P33587	Vitamin K-dependent protein C	Proc	460	51.8	6.35	69826.3	78332.4	55015.8	55542.9	59126.2	60873.4	60353.3	70163.0	66332.3	65018.4	#N/A	#N/A	#N/A	#N/A
P70414	Sodium/calcium exchanger 1	Slc8a1	970	108	5	72226.6	72134.6	59797.5	67129.3	62527.8	70472.3	63923.2	77288.6	75469.0	63465.0	104868.4	106597.6	93649.5	93077.7
Q61315	Adenomatous polyposis coli protein	Apc	2845	310.9	7.58	38008.0	44692.6	36026.9	35081.7	42154.7	41475.0	35857.8	39567.1	36127.7	38853.4	#N/A	#N/A	#N/A	#N/A
P01723	Ig lambda-1 chain V region	-	117	12.2	5.21	1126105.1	283951.7	1197512.7	320349.7	611178.6	262959.0	406763.0	1109302.6	1179729.2	1376002.2	#N/A	#N/A	#N/A	#N/A
Q9CQD4	Charged multivesicular body protein 1b-2	Chmp1b2	199	22.1	8.1	1176749.0	1000592.7	1013218.4	934462.6	874543.1	885143.5	1075367.0	1020221.7	940565.8	1013608.7	#N/A	#N/A	#N/A	#N/A
E5FYH1	Protein TOPAZ1	Topaz1	1653	185.4	7.72	24089.2	41220.5	30862.1	42371.1	34608.6	31949.9	24837.5	26576.8	31722.8	33681.5	36122.8	42175.3	44093.2	50446.7
Q9Z0H0	Cell division cycle 7-related protein kinase	Cdc7	564	62.7	8.6	107443.9	114445.7	113728.5	110287.0	101840.9	110182.0	102027.1	101074.7	113236.8	116421.7	45265.7	45001.6	47908.9	45275.3
O35625	Axin-1	Axin1	863	96.2	6.87	64389.1	69335.1	61190.9	61414.5	65626.0	63658.8	67333.7	51456.8	66456.3	69411.3	#N/A	#N/A	#N/A	#N/A
Q9WUU8	TNFAIP3-interacting protein 1	Tnip1	647	73	6.05	92921.6	97237.6	86163.0	96486.8	102880.1	113544.6	105878.4	97516.9	93797.2	96736.5	195359.4	203168.3	198354.8	189836.7
Q9R1A8	E3 ubiquitin-protein ligase COP1	Cop1	733	80.4	6.92	100768.8	78172.6	99372.0	79494.6	101029.3	103164.2	93152.3	100808.9	82789.4	99817.9	#N/A	#N/A	#N/A	#N/A
Q91YT8	CSC1-like protein 1	Tmem63a	804	91.8	7.93	164600.5	161825.2	156735.3	141524.4	156376.4	176110.4	155636.4	163219.1	164575.3	155855.5	145975.1	150083.5	137868.9	123390.8
Q9JHG6	Calcipressin-1	Rcan1	251	28.1	5.41	43541.1	41333.6	36529.0	36611.8	36857.1	38313.6	32074.2	38140.5	39528.2	36315.1	50987.3	56639.4	47781.1	47233.1
Q91VR8	Protein BRICK1	Brk1	75	8.8	5.45	95015.2	105298.8	107382.4	90384.0	106573.7	105173.8	85654.2	95165.3	100099.9	91690.4	143243.9	128836.9	137395.0	123859.5
O54804	Choline kinase alpha	Chka	453	52	6.87	61144.3	67289.6	63973.4	68071.3	73436.8	68866.8	62765.4	54343.0	79005.0	79081.8	127651.5	129915.8	120539.5	117156.9
Q6P2L6	Histone-lysine N-methyltransferase NSD3	Nsd3	1439	160.9	8.27	89118.3	109850.9	93129.7	87294.6	99152.2	107328.7	95557.3	88764.4	101429.8	100901.6	77195.3	77095.7	70226.5	68338.6
A0A075B5M4	Ig-like domain-containing protein	Igkv4-57-1	119	12.7	7.11	115011.5	128545.5	102429.2	123229.2	117826.1	109830.0	110086.1	107322.5	111329.1	127314.3	#N/A	#N/A	#N/A	#N/A
P62073	Mitochondrial import inner membrane translocase subunit Tim10	Timm10	90	10.3	6.29	38218.7	38192.3	44747.5	39815.2	38989.1	42424.6	36118.0	48121.9	45716.7	39915.9	#N/A	#N/A	#N/A	#N/A
Q8CFD4	Sorting nexin-8	Snx8	459	52	8.03	25705.0	36905.8	28467.0	31603.7	28670.6	42112.3	26411.2	22289.1	23173.5	29497.0	#N/A	#N/A	#N/A	#N/A
Q921N7	Transmembrane protein 70, mitochondrial	Tmem70	253	28.3	7.47	126141.5	132275.4	132244.4	130512.6	134226.9	121122.4	118492.8	119003.8	124825.6	139060.7	411075.8	472280.3	474357.8	457841.2
Q9R1B9	Slit homolog 2 protein	Slit2	1521	168.7	7.11	114441.8	120108.5	89485.3	112994.8	89898.2	92971.0	101390.1	107628.0	104310.6	100559.4	#N/A	#N/A	#N/A	#N/A
Q8CG73	Protein fantom	Rpgrip1l	1264	144.9	5.43	130199.2	160677.8	148211.2	164841.9	135064.9	155407.4	119571.7	144777.9	143581.8	136262.7	#N/A	#N/A	#N/A	#N/A
Q80UP3	Diacylglycerol kinase zeta	Dgkz	929	104	8.09	39480.4	40786.3	34362.2	34526.4	40905.6	40932.7	33185.9	40437.7	38532.4	36844.7	#N/A	#N/A	#N/A	#N/A
Q5S003	Sperm-associated antigen 17	Spag17	2320	262.9	6.16	114241.4	169422.0	199237.3	253092.9	86918.3	77061.2	72283.4	93562.1	133589.6	134437.4	50442.7	60507.9	65476.1	67639.9
F6YCR7	Homeobox protein Rhox13	Rhox13	232	25.3	4.61	404424.8	434846.0	422629.2	347625.8	409575.0	427712.9	344455.9	320262.2	437987.5	460556.3	1537692.4	1691807.3	1882225.8	1522430.4
Q91VN1	Zinc finger protein 24	Znf24	368	41.9	6.04	47510.8	59618.4	57350.3	52807.8	54237.1	53662.3	47749.4	57801.7	53107.0	51254.3	#N/A	#N/A	#N/A	#N/A
O09005	Sphingolipid delta(4)-desaturase DES1	Degs1	323	38.2	7.77	73981.4	88390.2	78668.4	79420.0	89608.8	84208.8	84027.4	81857.0	79145.4	82770.4	79264.6	83365.5	77775.9	75951.2
Q3TDK6	Protein rogdi homolog	Rogdi	287	32.1	8.18	41150.8	43880.5	36037.8	41807.3	41762.4	45899.9	41471.2	42983.2	45223.5	46354.1	97818.2	110718.5	93464.7	103346.8
P70274	Selenoprotein P	Selenop	380	42.7	7.09	91964.3	70188.6	75800.2	72155.2	51903.6	66445.8	65314.1	70672.1	67530.7	65481.7	52234.5	76561.1	63926.6	62944.5
Q9D1T5	Proline-rich protein 15	Prr15	122	13.6	8.13	36430.7	33784.3	35251.1	30871.9	34195.4	36192.3	37309.0	35754.7	34036.9	41838.2	18818.0	21715.5	20509.6	19056.0
Q9ES34	Ubiquitin-protein ligase E3B	Ube3b	1070	122.7	8.32	24450.5	26253.2	23031.9	27297.9	25026.0	25403.5	22823.1	23419.7	26388.7	25051.4	153700.0	152703.4	134911.4	153689.0
Q3UZW7	Protein-lysine N-methyltransferase EEF2KMT	Eef2kmt	335	36.9	5.5	109871.3	118515.1	110775.6	112314.3	118173.4	120725.9	108559.7	112726.9	109106.8	108535.6	#N/A	#N/A	#N/A	#N/A
B2RXC8	3222402P14Rik protein	Ppp2r3a	1150	130.2	5.2	39447.3	45495.0	42093.8	40807.9	44017.2	55128.2	43880.6	44864.3	44093.3	40858.2	#N/A	#N/A	#N/A	#N/A
Q9D2F7	Nuclear pore membrane glycoprotein 210-like	Nup210l	1881	208.6	7.23	15407.9	27278.3	25918.7	23397.0	20278.3	19756.9	19852.3	12610.6	14914.0	25041.1	#N/A	#N/A	#N/A	#N/A
Q99N91	39S ribosomal protein L34, mitochondrial	Mrpl34	92	10.5	11.81	124831.3	117140.5	105187.1	116236.1	117078.6	108635.3	114849.1	115535.7	120532.3	121743.8	163756.4	161539.3	144432.2	163424.6
Q64487	Receptor-type tyrosine-protein phosphatase delta	Ptprd	1912	214.3	6.55	6290.4	12901.9	9821.1	8942.2	10871.5	10245.1	11306.9	10654.9	8077.5	12378.3	#N/A	#N/A	#N/A	#N/A
Q8C6I2	Succinate dehydrogenase assembly factor 2, mitochondrial	Sdhaf2	164	19.4	6.35	268394.4	281426.6	273161.1	281975.2	287370.8	296887.7	278921.0	266039.3	293291.0	281889.5	116284.5	150490.3	135013.9	143746.6
Q9R1S8	Calpain-7	Capn7	813	92.5	7.97	239425.9	274481.1	247404.9	300437.8	249630.8	251028.0	223859.3	231758.1	257427.0	267041.8	#N/A	#N/A	#N/A	#N/A
Q9D3S3	Sorting nexin-29	Snx29	818	91.3	6.13	21351.7	14753.2	20034.4	25216.2	20522.7	16591.3	19645.1	18410.2	17745.9	25218.0	#N/A	#N/A	#N/A	#N/A
Q3TLR7	Denticleless protein homolog	Dtl	729	79.1	8.79	73433.1	67170.5	68362.2	76066.0	78039.7	78501.5	73116.2	63743.0	70409.4	61187.2	50960.8	49578.5	52251.0	51393.5
Q9D0U6	Repressor of RNA polymerase III transcription MAF1 homolog	Maf1	258	28.8	4.64	223504.3	196572.0	223524.4	196156.3	296099.9	268754.7	242941.0	229713.5	225365.4	235488.1	34871.5	38011.4	23836.3	36542.9
Q6A098	Selenocysteine insertion sequence-binding protein 2-like	Secisbp2l	1086	119.6	6.35	37673.5	38155.9	29249.7	27995.2	27756.9	27505.3	32596.9	25361.2	32519.3	26199.1	38087.3	39395.5	31876.6	30486.8
P17809	Solute carrier family 2, facilitated glucose transporter member 1	Slc2a1	492	53.9	8.87	82326.0	70418.6	81882.6	75487.1	87603.1	89479.0	89525.1	78780.2	90094.6	83730.6	282433.8	307180.8	309863.6	303891.5

Q9WVK8	Cholesterol 24-hydroxylase	Cyp46a1	500	56.8	8.82	54555.2	70235.1	57613.1	64967.9	53976.6	61265.4	61199.1	55633.7	66549.6	69381.0	#N/A	#N/A	#N/A	#N/A
Q9CVI2	Protein FAM133B	Fam133b	245	27.9	10.07	53786.1	58399.1	56800.9	53695.5	53075.9	64413.7	61144.3	51600.1	63173.2	57815.4	101077.3	104654.4	127463.4	117574.7
Q9D0C1	E3 ubiquitin-protein ligase RNF115	Rnf115	305	33.8	5.83	62791.6	65791.7	62623.1	70816.2	81451.9	70707.3	68369.5	75265.5	71159.0	70556.3	#N/A	#N/A	#N/A	#N/A
Q9QUM7	MutS protein homolog 5	Msh5	833	92.5	5.95	37183.9	45240.0	40535.9	43622.7	56068.7	47016.4	42792.3	39805.5	50310.0	49623.0	44166.6	41220.4	38143.1	30614.1
P0DN34	NADH dehydrogenase [ubiquinone] 1 beta subcomplex subunit 1	Ndufb1	57	7	8.21	715682.5	693884.6	784765.7	789667.0	720196.4	623245.4	655271.1	751688.2	744913.2	735167.5	#N/A	#N/A	#N/A	#N/A
P40338	von Hippel-Lindau disease tumor suppressor	Vhl	181	20.8	6.11	35992.0	36371.9	41320.4	42898.7	46972.4	40119.7	37539.1	35479.9	38813.1	40120.6	28028.7	29658.8	26499.4	28394.8
Q80WC9	Beta-alanine-activating enzyme	Aasdh	1100	121.5	6.6	41244.3	45002.9	49728.4	35976.4	50704.5	45631.2	43609.6	45328.5	45426.0	48150.1	#N/A	#N/A	#N/A	#N/A
Q64343	ATP-binding cassette sub-family G member 1	Abcg1	666	74	7.06	127070.5	123615.3	127179.4	128204.9	148137.3	154079.4	153593.6	112073.3	126823.5	124601.7	#N/A	#N/A	#N/A	#N/A
Q9CR27	WASH complex subunit 3	Washc3	194	21.1	4.46	330707.2	330804.3	343741.6	345857.0	307646.3	301844.1	297039.8	285490.4	315923.1	335104.4	452232.2	448720.4	485812.8	478974.8
Q9D8T4	Golgi apparatus membrane protein TVP23 homolog B	Tvp23b	205	23.3	8.34	11133.6	11502.1	12319.7	11026.4	13454.5	12904.9	12769.1	12276.7	11098.0	13579.0	#N/A	#N/A	#N/A	#N/A
Q8BIB6	RIKEN cDNA 2810021J22 gene	ɜ10021J22F	539	61.1	8.84	46595.7	50063.6	44117.9	47806.6	50304.0	49130.6	47528.0	46059.2	49544.0	55249.6	#N/A	#N/A	#N/A	#N/A
P0CG15	Chromosome transmission fidelity protein 8 homolog	Chtf8	121	13.2	8.66	124934.3	126444.0	138373.0	129720.9	154778.3	153016.1	132461.8	128331.8	142773.8	134956.1	#N/A	#N/A	#N/A	#N/A
Q3UDR8	Protein YIPF3	Yipf3	347	38	5.94	170783.8	174573.6	167159.7	182033.8	168962.9	172896.2	147333.9	163613.6	167195.7	169782.2	147288.5	164351.0	146951.8	159117.3
Q9D516	Coiled-coil domain-containing protein 130	Ccdc130	385	43.9	8.09	225003.9	244066.3	193345.9	235340.6	221199.5	211054.0	208164.0	195604.8	226115.4	251772.6	#N/A	#N/A	#N/A	#N/A
Q07231	Zinc finger and SCAN domain-containing protein 21	Zscan21	555	63	7.17	9253.2	10783.2	13362.3	12680.1	11101.9	14632.8	10505.9	10776.0	14081.5	12207.2	70019.6	77997.4	74357.3	70354.2
Q3TVW5	Trichoplein keratin filament-binding protein	Tchp	497	60.6	6.8	293285.0	298132.5	261161.2	262442.3	308699.9	253794.6	290388.8	258943.0	282582.1	293907.4	#N/A	#N/A	#N/A	#N/A
Q9Z184	Protein-arginine deiminase type-3	Padi3	664	75	5.68	19080.8	28593.4	18465.2	25647.6	24258.7	25681.0	24476.2	27827.7	23735.0	27624.9	#N/A	#N/A	#N/A	#N/A
Q9CY28	GTP-binding protein 8	Gtpbp8	285	31.9	9.28	30579.4	40726.8	33209.1	41870.6	41599.6	43251.5	31159.7	35497.8	39227.5	37572.5	22231.7	29172.1	30172.9	33425.9
Q80UW2	F-box only protein 2	Fbxo2	297	33.7	4.28	78796.6	93729.1	78284.3	98259.9	88440.5	91472.4	77119.1	76857.0	79806.3	76295.9	#N/A	#N/A	#N/A	#N/A
Q8JZY4	Mitochondrial ribonuclease P catalytic subunit	Prorp	584	66.8	8.79	109681.7	110730.1	110583.0	104288.3	110803.3	113644.6	99490.8	112262.5	113716.9	110245.8	120865.2	144788.7	122249.7	132720.9
P0DPE0	EEF1A lysine methyltransferase 4	Eef1akmt4	255	28.5	5.41	40747.0	48476.6	39132.7	38294.7	41641.1	38607.5	38946.3	43664.9	40720.5	45040.5	127035.0	136512.1	114845.3	126660.2
A2AGH6	Mediator of RNA polymerase II transcription subunit 12	Med12	2190	244.4	7.17	76018.3	82281.2	67907.8	72420.4	83018.8	105915.9	81617.2	79652.0	80553.7	85968.6	#N/A	#N/A	#N/A	#N/A
Q9Z1R3	Apolipoprotein M	Apom	190	21.3	6.52	119809.2	113581.6	104482.1	106973.9	88615.9	95962.6	90579.5	106535.6	103884.5	105300.4	276674.1	267844.2	232238.1	242371.9
A0A0G2JGA3	RanBP2-type domain-containing protein	Tex13c2	530	58.8	5.24	91601.6	88352.6	88309.6	101354.9	84310.8	83820.2	94891.9	84194.5	83794.9	101747.8	44245.4	50422.8	35284.4	36625.5
Q9CTH6	rRNA-processing protein FCF1 homolog	Fcf1	198	23.3	9.67	29547.7	38220.8	30769.0	29157.8	35887.1	27417.2	24819.1	28371.8	29802.8	30746.3	#N/A	#N/A	#N/A	#N/A
Q3V038	Tetratricopeptide repeat protein 9A	Ttc9	219	24.3	8.85	116261.0	143987.6	115505.1	160634.5	105163.1	123290.3	149266.0	113679.5	130970.8	114718.2	22350.5	23734.2	20452.2	26399.1
Q9CXU0	Mediator of RNA polymerase II transcription subunit 10	Med10	135	15.7	6.19	112839.1	143371.2	124039.4	118721.3	132205.7	134374.4	118998.3	116556.2	143444.0	121616.8	24569.4	36667.5	31437.5	31152.0
P70444	BH3-interacting domain death agonist	Bid	195	21.9	4.81	138704.2	145052.7	133471.9	136328.9	150905.8	150001.4	151413.3	145715.6	141220.4	146034.8	52601.3	60393.1	53546.5	56709.7
Q3ULB5	Serine/threonine-protein kinase PAK 6	Pak6	682	74.8	9.44	10049.5	11775.2	9614.3	11222.2	8435.2	11975.4	9860.5	9724.0	11351.8	11529.4	15183.0	18718.6	12498.6	16584.8
Q8BY98	Ankyrin repeat domain-containing protein SOWAHD	Sowahd	327	36.3	8.66	260481.2	255066.6	226199.6	245474.5	228945.7	226863.9	231432.4	218128.0	255364.8	232695.3	#N/A	#N/A	#N/A	#N/A
P55002	Microfibrillar-associated protein 2	Mfap2	183	20.6	4.89	86625.4	83001.7	74906.0	72589.2	87720.6	77628.5	98726.5	71577.1	78547.6	87140.8	#N/A	#N/A	#N/A	#N/A
Q8BWD8	Cyclin-dependent kinase 19	Cdk19	501	56.5	8.53	91985.4	86031.2	80374.0	81209.9	82822.3	83539.0	81639.2	78409.6	74480.0	80724.3	149980.9	135972.3	124218.0	123879.3
Q8R2Y3	Dolichol kinase	Dolk	534	59.1	8.34	66518.5	67880.9	74718.9	74587.2	62881.0	68468.1	65927.6	67640.1	69189.9	68299.5	89124.8	103354.4	90071.2	96473.1
Q8K2X2	RNA-binding protein 48	Rbm48	371	41.6	8.84	43104.2	47975.5	35052.2	40881.2	42643.9	38453.7	40280.6	36010.1	36341.4	43223.1	#N/A	#N/A	#N/A	#N/A
Q9D1Q4	Dolichol-phosphate mannosyltransferase subunit 3	Dpm3	92	10.1	7.08	103231.1	94711.6	108637.5	92259.2	106347.9	141713.9	151574.6	108410.7	103101.2	107179.4	137758.7	150594.5	165547.0	167543.9
Q9Z1T2	Thrombospondin-4	Thbs4	963	106.3	4.67	79935.7	84476.6	79416.0	58243.4	80598.7	93279.5	76134.1	76072.8	76781.4	79027.1	#N/A	#N/A	#N/A	#N/A
P33434	72 kDa type IV collagenase	Mmp2	662	74.1	5.53	8681.0	5298.2	8405.5	3286.1	8896.8	11123.8	14910.1	10582.2	8235.3	8699.4	#N/A	#N/A	#N/A	#N/A
Q80TZ9	Arginine-glutamic acid dipeptide repeats protein	Rere	1558	171.6	7.69	41652.5	47547.5	54990.6	41369.1	46124.9	49609.2	39506.5	45132.4	44818.9	50261.6	22340.8	25803.9	19950.7	19081.2
Q99J19	Small integral membrane protein 11A	Smim11a	55	6.3	10.01	35753.0	46365.6	38414.8	41588.8	42324.4	43136.0	40993.6	36008.3	43846.3	46219.2	#N/A	#N/A	#N/A	#N/A
Q8VDQ9	Protein KRI1 homolog	Kri1	704	82	5.12	569933.9	570521.2	490731.2	566116.4	434804.3	434146.6	520762.0	431266.2	541995.1	589324.9	74605.1	97862.1	95391.7	98137.6
A2ADA5	tRNA pseudouridine synthase-like 1	Pusl1	291	32	10.04	51436.3	48472.0	40229.2	47554.3	47861.5	52286.4	44095.7	47721.1	54343.5	42137.9	#N/A	#N/A	#N/A	#N/A
Q8R1R3	StAR-related lipid transfer protein 7, mitochondrial	Stard7	373	43.1	8.95	39835.8	36365.6	33622.0	37308.1	35228.0	30228.0	38426.2	29537.9	38060.9	38288.9	73860.6	65920.1	54900.1	56650.0
Q8VC34	Putative RNA polymerase II subunit B1 CTD phosphatase Rpap2	Rpap2	614	68.5	7.96	97055.6	100577.7	95250.2	92212.1	114595.5	112833.9	115011.2	95250.0	115882.4	100544.2	#N/A	#N/A	#N/A	#N/A
Q9D3A8	Carboxyl-terminal PDZ ligand of neuronal nitric oxide synthase protein	Nos1ap	503	55.8	6.09	98455.1	98628.2	93467.6	98206.6	113364.4	116297.8	119329.2	88585.6	98370.7	100061.4	#N/A	#N/A	#N/A	#N/A
Q8R2Z3	Anion exchange transporter	Slc26a7	656	71.8	8.13	77635.5	76154.0	70274.7	75993.0	70152.6	76255.3	68708.9	73019.0	66861.7	68373.8	24875.3	22342.6	24865.2	22478.5
Q91YU6	Leucine zipper putative tumor suppressor 2	Lzts2	671	72.5	7.11	194681.8	215448.9	186994.0	214993.3	207224.9	200661.7	203591.7	185973.2	194602.7	213312.8	75749.7	82989.5	77110.8	93235.2
Q6P3Y5	Zinc finger protein 280C	Znf280c	742	83.1	9.06	716187.1	688462.3	678450.9	645434.1	756643.2	729159.4	792689.5	653592.0	704782.6	739190.3	246994.4	257668.8	255022.9	262812.2
A2ASS6	Titin	Ttn	35213	3904.1	6.2	456911.1	568882.0	391733.7	641030.9	541820.1	803235.3	385188.9	809127.0	587497.6	619600.5	#N/A	#N/A	#N/A	#N/A
Q9JIG8	PRA1 family protein 2	Praf2	178	19.5	9.6	304797.4	283644.8	284815.9	269264.8	235566.0	251043.2	229307.0	290803.5	239899.1	254195.5	163699.6	165401.2	182830.6	174068.4
O88428	Bifunctional 3'-phosphoadenosine 5'-phosphosulfate synthase 2	Papss2	621	70.3	7.58	221355.3	251394.8	210659.4	252272.5	235743.2	219705.8	207978.5	202579.0	250812.6	243885.6	181630.2	182270.4	189566.2	189491.5
Q8BXC6	COMM domain-containing protein 2	Commd2	199	22.8	6.62	294582.9	285968.7	279666.6	289477.5	345812.4	336223.3	284681.5	274258.7	295301.9	325498.6	420129.3	479402.3	396366.2	448553.3
P25799	Nuclear factor NF-kappa-B p105 subunit	Nfkb1	971	105.5	5.39	105629.4	95746.8	106886.0	92329.7	111989.8	125135.8	122301.1	113632.9	95780.9	103355.0	160751.4	150368.1	147327.4	134460.2
P84102	Small EDRK-rich factor 2	Serf2	59	6.9	10.45	128259.1	88565.6	103051.0	92331.9	73420.9	90794.0	103382.6	118006.7	104252.7	81672.8	#N/A	#N/A	#N/A	#N/A
Q8BH01	Transmembrane and coiled-coil domain-containing protein 3	Tmco3	678	75.9	7.17	187287.4	179199.1	181245.6	176810.9	158418.7	159110.3	154430.6	160839.2	168403.4	177295.7	138705.9	146821.2	147315.1	152637.9
Q9D7B1	tRNA-dihydrouridine(20) synthase [NAD(P)+]-like	Dus2	493	55.3	6.34	346213.7	381432.1	357848.1	376674.0	361780.6	375333.8	361693.3	363976.3	370875.3	378358.8	66755.4	69207.5	65503.5	68854.7
Q9DAN6	Gametocyte-specific factor 1	Gtsf1	167	19.1	6.4	167803.3	210116.6	200303.9	227688.8	150141.6	149471.5	132869.1	147970.0	181182.8	188473.3	194645.2	241611.6	244638.3	260636.8
Q9Z131	SH3 domain-binding protein 5	Sh3bp5	463	51.8	5.11	53707.6	48077.6	47034.5	53748.8	53546.7	51225.3	53919.7	54208.7	52654.9	49810.0	#N/A	#N/A	#N/A	#N/A
Q8BJT9	ER degradation-enhancing alpha-mannosidase-like protein 2	Edem2	577	64.6	5.55	167290.4	154834.4	158816.0	170131.3	154654.7	170951.4	158609.3	146117.7	141880.0	171189.4	#N/A	#N/A	#N/A	#N/A
Q8K221	Arfaptin-2	Arfip2	341	37.7	5.87	133198.8	139678.4	124000.7	138606.3	133823.3	140918.1	128592.5	124807.4	132465.9	145495.3	#N/A	#N/A	#N/A	#N/A
Q91WC9	Sn1-specific diacylglycerol lipase beta	Daglb	669	73.9	6.73	43540.5	53845.9	48224.0	46012.8	45507.9	53340.1	45496.3	48467.2	42063.2	39461.1	110379.1	127769.6	116013.2	125351.7
A2AU72	Armadillo repeat-containing protein 3	Armc3	881	97.3	6.47	151735.1	184860.9	177548.3	210378.7	136103.6	127895.7	136235.1	128014.1	155611.2	165965.7	#N/A	#N/A	#N/A	#N/A
Q921N8	G protein pathway suppressor 2	Gps2	327	36.7	9.6	46053.5	50369.5	42703.8	52804.4	48395.6	46988.8	47833.1	56365.5	50966.5	48964.1	#N/A	#N/A	#N/A	#N/A
Q8BQY8	Checkpoint protein HUS1	Hus1	280	31.7	7.37	42403.6	52299.0	44156.3	42603.7	40301.0	44919.9	39600.4	39599.9	47347.7	44621.3	109990.9	115224.0	94430.6	106944.8
Q9QXV3	Inhibitor of growth protein 1	Ing1	279	32.1	7.42	68130.1	74300.7	63174.7	75397.8	65991.8	76243.0	62131.3	66573.5	80603.1	69496.5	#N/A	#N/A	#N/A	#N/A
Q80VP5	Probable peptide chain release factor C12orf65 homolog, mitochondrial	ΛGI:19199C	184	20.8	9.92	47826.6	47401.5	44348.0	47832.8	47956.2	48064.2	43858.1	47223.5	46553.4	44098.6	146883.9	139913.8	139267.1	146238.9
Q9CWR1	WD repeat-containing protein 73	Wdr73	371	40.8	5.54	33623.6	37022.5	43550.2	37151.8	40590.7	43878.7	39049.3	37149.0	46243.8	40081.3	112270.3	98378.4	113090.2	96862.6
Q3UQV5	Kelch repeat and BTB domain-containing protein 8	Kbtbd8	599	68.6	6.25	73103.5	93656.7	64558.4	76032.4	74994.7	74872.8	67255.2	69751.6	70045.0	67257.3	#N/A	#N/A	#N/A	#N/A
Q6PFE1	BTB domain-containing protein	Klhl5	708	79	6.54	145986.5	138063.2	138591.3	131579.3	131112.2	128968.9	140628.5	135023.6	142863.2	143396.1	#N/A	#N/A	#N/A	#N/A
Q8C0D7	Inhibitor of growth protein 4	Ing4	249	28.5	7.62	76274.8	65013.9	87005.4	65605.4	85613.5	77732.3	86474.0	67736.7	70437.5	67477.2	119580.8	112969.1	110743.0	106710.5
Q7TQI7	Ankyrin repeat and BTB/POZ domain-containing protein 2	Abtb2	1024	113.4	6.28	79499.5	79000.5	62601.0	84590.9	71836.3	66021.5	80523.2	68269.7	80043.6	87619.0	73950.6	97398.0	74735.8	85219.9
Q8BK06	F-box only protein 9	Fbxo9	437	50.7	6.84	464635.5	475541.1	436882.3	463871.8	438042.0	455733.0	404621.7	418678.9	442069.3	473779.3	143885.5	149869.4	120032.1	138215.7
Q9ES30	Complement C1q tumor necrosis factor-related protein 3	C1qtnf3	246	26.8	6.74	30461.3	37382.3	25667.8	31016.2	32186.1	38065.7	30233.4	28871.2	31610.7	39598.9	#N/A	#N/A	#N/A	#N/A
Q6ZQ89	E3 ubiquitin-protein ligase MARCHF6	Marchf6	909	102.2	6.3	189517.6	189741.8	144401.9	182958.3	158649.0	201006.0	167260.9	164713.5	155684.7	157236.5	#N/A	#N/A	#N/A	#N/A
Q2PMX6	DMRT-like domain-containing protein	Dmrtc1b	267	29.1	10.2	171258.2	167603.5	126663.3	175705.5	121348.1	122570.4	127185.8	130472.0	139797.4	150996.9	#N/A	#N/A	#N/A	#N/A
Q3TCJ8	Coiled-coil domain-containing protein 69	Ccdc69	202	23.3	6.99	43699.8	44968.0	39395.7	48178.3	35232.3	34817.5	34863.5	48575.8	47176.1	35541.5	#N/A	#N/A	#N/A	#N/A
Q01237	3-hydroxy-3-methylglutaryl-coenzyme A reductase	Hmgcr	887	97	6.7	56354.8	64912.3	58684.1	77875.6	51427.1	48124.7	44306.9	50861.4	50692.0	52722.3	#N/A	#N/A	#N/A	#N/A
Q9WV86	Katanin p60 ATPase-containing subunit A1	Katna1	491	55.9	7.5	82039.6	84996.7	97203.7	86246.5	101620.7	87637.0	80707.3	84048.1	91442.5	96766.3	85152.2	82180.1	88565.7	90488.5
O70443	Guanine nucleotide-binding protein G(z) subunit alpha	Gnaz	355	40.8	7.61	35372.1	40145.7	34881.8	37903.5	40414.5	39774.3	38893.8	41604.2	35365.6	38798.6	51851.1	58444.3	51205.2	53246.4
Q9DB32	Hydroxyacylglutathione hydrolase-like protein	Haghl	283	31.5	5.55	108546.1	102551.5	105044.5	101196.8	109452.9	100144.1	110102.2	117265.4	104216.9	110336.7	206463.2	188755.8	232935.4	211408.9
Q05AA6	Dystrophin-related protein 2	Drp2	957	108	6.25	8082.9	10243.9	9695.9	7904.1	9725.5	10007.1	10523.1	6215.7	10672.0	7641.6	#N/A	#N/A	#N/A	#N/A
Q497I4	Keratin, type I cuticular Ha5	Krt35	455	50.5	4.97	3411713.8	831518.6	1009016.0	895022.3	938631.2	946942.7	1078582.2	925323.3	1000552.4	907320.6	2574378.8	523085.2	647976.4	593511.6
Q8VHG0	Dimethylaniline monooxygenase [N-oxide-forming] 4	Fmo4	560	63.8	8.94	72451.1	63739.4	62915.9	55028.8	72651.5	71479.3	75671.4	66937.1	67598.7	75199.7	68516.0	90836.9	88170.1	123120.1
Q3U829	AP-5 complex subunit zeta-1	Ap5z1	807	89.4	6.95	173026.0	198931.8	194103.3	198511.2	190265.8	183441.2	173844.2	182224.2	215189.5	192848.8	#N/A	#N/A	#N/A	#N/A
Q9D4G2	Heat shock factor 2-binding protein	Hsf2bp	338	37.9	5.33	69303.3	101369.1	73187.9	90357.3	80893.3	80960.0	78151.6	75422.5	82018.9	90837.2	196769.9	225933.5	217847.0	233498.7
P70695	Fructose-1,6-bisphosphatase isozyme 2	Fbp2	339	36.9	6.16	36059.9	39701.0	33720.0	39394.5	32544.3	32830.6	34809.7	34143.7	30640.7	36433.6	37030.5	45066.6	32116.3	34807.5
Q9D7E4	UPF0449 protein C19orf25 homolog	ΛGI:191362	109	12.1	5.25	30555.5	30924.7	32217.5	31206.0	42398.1	35562.6	37538.2	35922.1	39997.2	40241.0	62597.5	65952.0	71808.9	82484.1
Q99M54	Cell division cycle-associated protein 3	Cdca3	266	28.7	5.81	23549.1	40582.8	37099.7	37850.0	25225.1	25602.7	24041.4	27909.9	32688.4	32881.5	#N/A	#N/A	#N/A	#N/A
P61219	DNA-directed RNA polymerases I, II, and III subunit RPABC2	Polr2f	127	14.5	4.22	590728.2	504285.7	582077.5	650227.4	642272.7	631805.1	702675.9	691209.7	633588.9	593538.7	475143.6	549588.4	584083.6	559059.2
Q6P5F7	Protein tweety homolog 3	Ttyh3	524	57.7	5.85	22947.3	20975.8	17902.3	19314.5	16427.3	24244.4	15590.5	17634.8	20263.5	14245.4	24506.3	22657.3	23055.3	24441.4
Q6PEM6	GRAM domain-containing protein 2B	Gramd2b	445	49.2	7.96	130443.6	153283.2	115676.0	137486.6	119909.9	115892.3	132293.8	99056.9	128737.2	127880.1	#N/A	#N/A	#N/A	#N/A
Q9EQG7	Ectonucleotide pyrophosphatase/phosphodiesterase family member 5	Enpp5	477	54.4	5.9	81199.7	94247.6	68630.0	85066.1	88267.3	91177.4	92511.7	79842.4	82220.1	88825.3	121808.5	139930.1	111084.5	132874.1
P0C6A2	Mastermind-like domain-containing protein 1	Mamld1	803	87.5	7.81	11904.1	19501.5	10337.4	11353.7	13235.5	16499.8	9741.2	19830.4	12934.0	10768.8	10945.4	12030.3	14411.4	11229.3

Q9CR23	Transmembrane protein 9	Tmem9	183	20.6	6.95	16864.8	18843.0	18740.9	14625.6	17040.6	19883.4	16201.6	16502.8	15766.0	16608.8	26750.7	20982.0	21431.2	26786.9
Q8VC31	Coiled-coil domain-containing protein 9	Ccdc9	543	61.4	5	299024.0	284275.1	323397.1	287524.6	327688.7	327980.8	279649.3	325476.8	294995.0	302780.4	77873.3	81156.7	87863.5	86167.3
Q8BQP8	Rab11 family-interacting protein 4	Rab11fip4	635	71.9	4.84	230024.1	242198.9	218371.7	226751.0	221736.8	211173.0	222614.1	218363.9	227639.6	231020.5	#N/A	#N/A	#N/A	#N/A
Q8K4Q8	Collectin-12	Colec12	742	81.3	5.49	77161.0	70289.7	70413.0	75217.0	63341.7	70292.8	77707.7	77091.7	63867.1	63767.1	#N/A	#N/A	#N/A	#N/A
Q3UNA4	NTF2-related export protein 2	Nxt2	142	16.2	5.81	41488.5	48453.2	35878.6	50252.2	46183.9	45983.2	37351.7	41133.1	42185.5	47967.0	#N/A	#N/A	#N/A	#N/A
Q8K1S6	Protein spire homolog 2	Spire2	718	80.2	7.37	19148.8	23119.8	23313.4	18820.6	20901.1	30621.6	20473.6	21914.1	24690.3	25189.1	#N/A	#N/A	#N/A	#N/A
Q9CX83	Armadillo repeat-containing X-linked protein 1	Armcx1	456	50.6	9.33	39723.9	50122.3	33618.9	46925.4	42523.6	47107.6	40635.8	40886.6	33498.7	43602.5	77185.4	77343.8	68928.1	84298.7
Q6P8Y0	Cilia- and flagella-associated protein 161	Cfap161	303	34.4	6.8	123583.5	141297.7	145971.7	187743.0	121735.4	121264.0	117334.8	118495.5	120424.7	138896.7	#N/A	#N/A	#N/A	#N/A
Q8CG70	Prolyl 3-hydroxylase 3	P3h3	732	81.7	6.65	35175.0	93110.0	39906.6	56000.4	61596.0	103334.6	38290.7	74249.9	52062.9	54957.6	#N/A	#N/A	#N/A	#N/A
Q8VCX1	Aldo-keto reductase family 1 member D1	Akr1d1	325	37.3	6.9	375418.8	298008.8	390936.2	364786.4	394073.7	360222.9	504175.1	372489.3	364195.6	437568.9	#N/A	#N/A	#N/A	#N/A
Q9EQK7	Protein-S-isoprenylcysteine O-methyltransferase	Icmt	283	31.8	8.18	171447.0	200704.5	197729.3	198836.8	172018.7	161511.5	158167.7	161676.3	185353.8	204774.5	211320.0	239760.2	251194.7	254333.5
P03893	NADH-ubiquinone oxidoreductase chain 2	Mtnd2	345	38.7	9.92	89100.6	93198.1	83275.6	88991.4	90916.3	91431.9	89790.0	89344.4	89255.2	94091.2	71714.6	74093.1	78537.7	79099.2
Q9D4C5	ELL-associated factor 1	Eaf1	268	28.9	5.4	166861.7	164504.6	164548.8	143723.2	176872.6	172608.2	155594.9	141443.0	171009.1	179872.1	#N/A	#N/A	#N/A	#N/A
O70479	BTB/POZ domain-containing adapter for CUL3-mediated RhoA degradation protein 2	Tnfaip1	316	36.1	7.84	316777.7	334654.6	307716.8	318938.0	300940.9	340273.5	339558.0	302605.6	328843.1	326207.9	179759.3	199644.9	178889.3	184120.8
Q5SXC4	Vascular endothelial zinc finger 1	Vezf1	518	56.5	9.57	12672.6	11378.0	12870.0	12271.2	13677.4	14038.6	15604.9	12748.1	10777.3	17579.9	#N/A	#N/A	#N/A	#N/A
Q6P6I6	DNA-directed RNA polymerase II subunit GRINL1A	Polr2m	366	41.2	6.55	47074.8	60109.8	44516.9	39904.1	53252.6	44579.7	46658.3	36040.3	44467.8	56447.0	#N/A	#N/A	#N/A	#N/A
O54785	LIM domain kinase 2	Limk2	638	72.2	7.5	66513.6	70764.9	63389.0	64267.8	78185.5	80061.8	64845.0	69092.6	75604.2	74613.5	#N/A	#N/A	#N/A	#N/A
Q62280	Protein SSXT	Ss18	418	45.8	6.46	113256.2	118920.6	118770.9	137336.1	110908.7	101343.5	98050.5	91579.4	115760.3	114344.0	#N/A	#N/A	#N/A	#N/A
O35149	Zinc transporter 4	Slc30a4	430	47.8	6.77	47365.0	49791.4	43222.8	41591.2	40174.4	52071.4	44877.9	44721.1	39377.3	45779.1	#N/A	#N/A	#N/A	#N/A
Q9QZN0	F-box only protein 15	Fbxo15	433	49.5	7.94	93240.9	147514.1	120146.7	121173.8	124595.2	98447.5	79051.1	95165.5	130062.5	145790.3	233402.8	273085.4	250774.2	244218.9
Q9CYC5	Kinetochore-associated protein DSN1 homolog	Dsn1	348	39.5	6.21	523136.0	426386.7	506260.1	439827.3	498089.2	544346.0	542905.6	526198.7	463920.9	433512.1	239496.3	232225.4	248927.1	244021.5
Q6P1I3	KICSTOR complex protein C12orf66 homolog	MGI:267098	445	50.2	9.09	93916.2	94754.2	86739.6	90304.3	87543.2	91135.7	89574.8	85344.4	92614.6	83247.1	#N/A	#N/A	#N/A	#N/A
Q6DI86	FAST kinase domain-containing protein 1, mitochondrial	Fastkd1	829	95.3	7.24	79709.6	79491.4	65153.6	70856.2	79684.4	77109.5	75229.1	70735.8	72444.1	88583.3	#N/A	#N/A	#N/A	#N/A
Q9CQ85	Mitochondrial import inner membrane translocase subunit Tim22	Timm22	194	20.1	8.22	31796.8	30572.1	29601.1	29429.2	30565.9	32439.7	31347.5	24816.3	26479.8	23963.5	#N/A	#N/A	#N/A	#N/A
Q3TH73	Protein tweety homolog 2	Ttyh2	532	59	6.07	98046.4	104208.9	109191.8	102820.1	97174.9	105606.7	102140.6	91580.1	100452.1	97500.6	89963.0	99131.8	91910.1	105829.5
Q6TYB5	Fasciculation and elongation protein zeta-2	Fez2	348	39.1	4.58	151223.6	155006.9	137998.6	138168.5	128791.5	150841.6	130532.1	142743.1	145330.9	143019.6	#N/A	#N/A	#N/A	#N/A
Q8BG17	Nucleolar protein 12	Nol12	217	25.3	10.13	134925.5	137810.4	149935.5	135973.9	119302.9	140551.2	120999.7	120336.0	134576.2	140465.3	#N/A	#N/A	#N/A	#N/A
Q91YE8	Synaptopodin-2	Synpo2	1087	116.5	7.37	24493.1	29018.6	16583.1	27493.1	25633.7	28571.6	24458.3	23095.1	26647.4	25575.4	#N/A	#N/A	#N/A	#N/A
Q7TPV2	E3 ubiquitin-protein ligase DZIP3	Dzip3	1204	137.9	5.36	112694.0	142383.0	109243.3	115795.8	119328.3	130272.5	103478.6	106999.2	124905.6	131036.7	155623.7	170106.0	164846.1	157267.3
Q9QWZ1	Cell cycle checkpoint protein RAD1	Rad1	280	31.6	4.84	263385.4	277600.5	255818.3	261450.9	263150.2	287646.0	255390.3	230425.3	277414.3	289243.3	237103.8	253860.3	202153.4	241215.1
Q3TXT3	SOSS complex subunit C	Inip	104	11.4	9.25	120747.8	131349.9	115075.3	120763.1	120262.7	124892.4	117908.1	141252.6	116371.6	116819.8	#N/A	#N/A	#N/A	#N/A
Q8R2U2	Nucleolus and neural progenitor protein	Nepro	564	63.4	9.94	43679.7	49908.8	50614.1	50922.4	50192.7	58680.7	52764.0	49680.9	45858.7	48542.2	231781.6	264029.5	245075.1	245043.3
Q8BG21	Calcium channel flower homolog	Cacfd1	171	18.3	5.44	425339.6	437064.7	431964.7	436140.5	395429.1	386059.1	395995.1	411464.8	436945.1	451414.2	194227.2	183332.9	192006.9	191620.9
Q6P8H8	Probable dolichyl pyrophosphate Glc1Man9GlcNAc2 alpha-1,3-glucosyltransferase	Alg8	526	59.5	9.04	309860.8	330375.3	336350.4	357458.5	306264.8	316595.6	257543.2	279155.2	284202.6	306180.1	479067.9	520150.4	547535.1	532035.7
Q8VEA4	Mitochondrial intermembrane space import and assembly protein 40	Chchd4	139	15.5	4.32	7886.3	9613.4	10016.0	11371.5	9210.2	9597.6	7011.9	7310.7	9673.4	8227.6	#N/A	#N/A	#N/A	#N/A
Q8BHZ4	Zinc finger protein 592	Znf592	1262	137.4	7.96	46397.9	45587.0	50706.8	37109.9	48824.4	57053.4	50888.5	44249.8	47519.6	47660.5	#N/A	#N/A	#N/A	#N/A
Q9D9J8	BPI fold-containing family A member 3	Bpifa3	232	25.7	8.59	120836.0	104902.3	97818.8	99735.3	110197.5	108881.8	101509.7	99881.9	106133.8	101955.8	228768.8	202780.7	229516.5	225548.8
P62322	U6 snRNA-associated Sm-like protein LSm5	Lsm5	91	9.9	4.54	28374.0	32165.8	41825.6	37432.4	30322.1	37248.6	37812.4	37909.6	41245.4	29311.3	30417.3	40229.8	30555.8	36293.0
Q78J03	Methionine-R-sulfoxide reductase B2, mitochondrial	Msrb2	175	19.1	9.06	280025.2	254900.6	235958.7	239221.0	277862.9	275358.6	295601.3	256524.6	253713.9	251708.1	70100.7	65039.7	56963.1	57801.1
Q6P3E7	Polyamine deacetylase HDAC10	Hdac10	666	72.1	5.39	83967.8	81916.4	73534.8	86335.5	79516.0	84065.8	78324.9	72900.2	84938.4	86037.7	80012.0	87553.2	75972.8	77809.0
Q71FD7	Filamin-binding LIM protein 1	Fblim1	375	41	6.51	110515.6	97070.7	85804.4	105572.4	90411.4	108201.2	117806.6	89902.2	111130.1	105871.9	#N/A	#N/A	#N/A	#N/A
Q924Y0	Gamma-butyrobetaine dioxygenase	Bbox1	387	44.7	6.92	263938.2	289063.5	284559.6	338797.6	237165.7	250975.1	231792.6	236257.2	274813.4	273509.4	219971.0	242654.5	210181.0	233279.9
Q9QUN7	Toll-like receptor 2	Tlr2	784	89.4	6.64	12993.2	14722.3	9238.1	9039.3	11960.3	13352.5	10096.0	12186.6	10716.4	10416.4	#N/A	#N/A	#N/A	#N/A
Q6PFH3	DDB1- and CUL4-associated factor 15	Dcaf15	600	66.6	6.58	26258.4	39268.9	33057.7	38517.2	31846.1	32871.9	30388.1	33581.4	36334.7	34051.1	40972.6	44384.3	42461.6	39662.0
Q8BYR2	Serine/threonine-protein kinase LATS1	Lats1	1129	126.2	8.73	83455.8	87914.3	85677.0	85264.1	79923.0	105458.8	102163.4	88033.1	76725.4	78336.0	#N/A	#N/A	#N/A	#N/A
Q3TJM4	Centromere protein T	Cenpt	515	56.2	9.57	74356.1	76185.3	65938.2	79837.3	70418.2	76178.7	79369.8	63889.7	85219.6	85261.0	125183.2	123446.4	107530.0	102807.6
Q64429	Cytochrome P450 1B1	Cyp1b1	543	60.5	8.43	75621.4	87990.4	74233.2	90517.1	82965.3	84227.6	109372.3	69207.4	87816.7	86762.2	154277.1	144404.1	128134.0	149317.9
Q3UY23	Ferredoxin-fold anticodon-binding domain-containing protein 1 homolog	Fdxacb1	622	70.1	7.58	194924.0	201852.3	172856.5	218399.5	162938.8	174022.5	165274.8	148034.4	186222.4	201028.4	168498.3	210989.5	166572.1	182790.3
Q8R0K4	Coiled-coil domain-containing protein 137	Ccdc137	290	32.9	10.68	80984.4	58835.7	68034.8	62339.5	67144.3	69981.7	61863.5	65877.8	64200.6	55915.9	32419.5	34412.2	39318.5	38083.6
Q571K4	TGF-beta-activated kinase 1 and MAP3K7-binding protein 3	Tab3	716	79	8.5	43820.6	44464.9	43052.0	44869.4	44872.8	42976.9	45155.9	40403.4	44726.6	50351.2	66234.9	71979.4	56958.3	60347.6
P97358	TATA box-binding protein-associated factor RNA polymerase I subunit B	Taf1b	586	67.9	8.03	15129.9	14201.0	13639.7	13810.4	13216.6	15395.4	12531.6	12911.8	12861.6	14572.3	#N/A	#N/A	#N/A	#N/A
Q99LU0	Charged multivesicular body protein 1b-1	Chmp1b1	199	22.1	8.1	263340.8	230240.7	255782.7	240269.7	274407.6	267515.3	275983.8	278543.6	267235.2	262203.4	225299.2	201969.1	223628.7	205957.7
Q3U4G0	Protein C15orf41 homolog	MGI:302688	281	32.1	7.14	106839.8	96880.8	97111.6	91244.7	101812.3	103432.5	102006.8	86746.9	95027.0	105791.8	#N/A	#N/A	#N/A	#N/A
Q9DBS2	Tumor protein p63-regulated gene 1-like protein	Tprg1l	266	29.8	7.37	459864.1	471663.7	453490.0	489175.7	398372.9	419277.7	406539.3	423519.9	438187.8	469885.7	639735.9	819292.5	655844.0	765070.4
P51791	H(+)/Cl(-) exchange transporter 3	Clcn3	818	90.8	6.28	106726.5	97179.6	87182.3	96974.1	108315.3	110297.8	115727.9	100158.8	105785.5	103452.5	#N/A	#N/A	#N/A	#N/A
Q6P7W5	tRNA-splicing endonuclease subunit Sen2	Tsen2	460	52.2	5.9	120809.3	118580.5	106243.4	107693.4	129036.1	115926.2	100861.1	98146.7	109775.9	106233.7	99712.9	120472.7	100405.3	107738.1
Q8K389	CDK5 regulatory subunit-associated protein 2	Cdk5rap2	1822	205.8	5.3	188709.1	192556.6	181448.1	178931.9	192273.5	202382.5	194237.1	175425.3	193038.8	200184.9	264646.1	294265.1	279127.0	302633.7
D3Z3K2	E3 ubiquitin-protein ligase CCNB1IP1	Ccnb1ip1	276	31.4	7.85	123220.1	116863.7	116524.7	119623.8	96277.4	100004.6	84932.9	94140.0	112243.2	111471.1	#N/A	#N/A	#N/A	#N/A
Q9JL19	Nuclear receptor coactivator 6	Ncoa6	2067	219.5	9.32	184411.0	164430.3	189054.1	172378.9	194398.9	203476.6	193360.0	194600.7	203694.0	191286.8	#N/A	#N/A	#N/A	#N/A
Q4KWH5	1-phosphatidylinositol 4,5-bisphosphate phosphodiesterase eta-1	Plch1	1682	187.6	7.33	376670.5	447160.4	341156.6	428667.1	298366.9	305604.9	334377.0	300516.8	398052.4	410789.6	598631.6	645964.2	616276.9	659737.6
Q8BMD6	Transmembrane protein 260	Tmem260	703	78.9	8.29	12187.2	19128.1	11883.8	13253.1	11553.7	16046.5	11571.4	16553.4	12435.0	12822.3	22746.5	23969.3	21261.8	24118.4
O08989	Ras-related protein M-Ras	Mras	208	23.9	8.78	95554.8	81977.7	80869.3	77208.6	75824.8	100995.0	93253.9	102657.0	87374.6	72507.0	#N/A	#N/A	#N/A	#N/A
Q9DBL2	Ganglioside-induced differentiation-associated protein 2	Gdap2	498	56.2	5.39	144706.5	163618.8	133116.4	155216.9	160949.9	152532.9	149798.0	129139.1	160222.4	162017.4	#N/A	#N/A	#N/A	#N/A
Q9JLM4	Zinc finger MYM-type protein 3	Zmym3	1370	152.8	6.39	181709.3	202515.1	181196.5	162870.4	195001.7	198480.3	188869.7	172957.8	186408.4	199267.0	154429.1	161393.8	163446.7	138921.1
A3KG59	Peptidase M20 domain-containing protein 2	Pm20d2	431	46.5	5.25	50530.0	52114.4	53022.7	41413.5	51012.1	64238.4	54593.0	47903.3	48208.7	48708.6	#N/A	#N/A	#N/A	#N/A
Q9D074	E3 ubiquitin-protein ligase MGRN1	Mgrn1	532	58.4	4.88	70828.3	67202.6	51815.3	61786.1	57896.5	66475.4	62996.7	62906.4	63073.6	65584.1	66363.7	51627.6	49131.2	54538.7
E9Q0B5	Fc fragment of IgG-binding protein	Fcgbp	2583	275	5.03	1650093.8	1505977.7	1617314.9	1906154.6	3059964.6	2877060.5	1886689.4	2331435.6	2210966.3	2172037.0	1619518.7	1628331.7	1619669.5	1776776.0
P23927	Alpha-crystallin B chain	Cryab	175	20.1	7.33	177266.1	172044.6	166981.7	167898.7	165447.1	171196.2	179211.1	235628.2	174393.3	168433.7	#N/A	#N/A	#N/A	#N/A
Q99LH9	SH3 domain-binding protein 5-like	Sh3bp5l	392	43.3	6	20694.9	21561.8	17590.5	16337.1	21693.6	22400.3	21001.0	20548.8	18604.7	20656.5	#N/A	#N/A	#N/A	#N/A
O55101	Synaptogyrin-2	Syngr2	224	24.8	4.78	246152.5	250838.9	240023.3	241871.1	247263.4	229394.1	220202.6	213319.4	229374.5	236094.5	233858.8	240696.1	231185.5	271757.2
P39654	Arachidonate 15-lipoxygenase	Alox15	663	75.4	6.11	13921.9	14191.4	11987.2	9616.3	10289.2	12905.0	21375.6	19824.6	12405.9	14332.7	17574.4	18638.7	13692.1	12174.8
Q9JLZ6	Hypermethylated in cancer 2 protein	Hic2	619	66.7	6.35	124431.8	142179.9	108971.2	154250.0	139317.6	122708.3	149911.7	126165.9	122475.1	123832.8	176278.3	188494.8	175558.8	214926.6
Q8VHG2	Angiomotin	Amot	1126	120.8	7.5	141919.3	169618.0	121674.9	160701.3	151697.7	148446.7	134814.3	144273.5	153280.6	145077.8	#N/A	#N/A	#N/A	#N/A
Q8K441	ATP-binding cassette sub-family A member 6	Abca6	1624	183.2	6.93	85181.5	96391.8	75230.3	87541.8	90122.5	89352.9	89374.6	89449.4	88209.9	89125.8	111103.2	118906.3	107383.3	105672.6
P09813	Apolipoprotein A-II	Apoa2	102	11.3	7.18	387337.9	355695.1	346337.6	354942.9	346174.0	372077.8	321319.9	325821.9	354547.7	320262.1	#N/A	#N/A	#N/A	#N/A
Q9DA37	Sphingomyelin synthase-related protein 1	Samd8	478	54.7	8.38	375473.0	446595.7	391037.8	415607.5	366472.9	432895.2	323414.7	436388.5	390023.3	387786.1	#N/A	#N/A	#N/A	#N/A
A2AMZ4	NADH dehydrogenase [ubiquinone] 1 alpha subcomplex assembly factor 8	Ndufaf8	74	7.8	9.55	118136.2	134488.3	134728.7	137219.8	138821.1	139595.8	142636.2	137262.2	137834.3	139590.0	#N/A	#N/A	#N/A	#N/A
Q91Y74	CMP-N-acetylneuraminate-beta-galactosamide-alpha-2,3-sialyltransferase 4	St3gal4	333	38	9.36	31564.1	28025.1	26132.4	29222.7	31513.2	30482.6	24280.6	31701.7	28255.9	30086.8	146366.5	156726.5	133935.4	133151.7
Q9D9I4	TBC1 domain family member 20	Tbc1d20	402	45.8	6.96	144980.1	141239.2	145104.4	143938.3	157003.6	159108.3	155617.2	138648.7	137418.8	135310.7	198632.6	198455.5	184682.4	177243.5
Q6R3M4	DNA polymerase iota	Poli	717	79.6	6.93	181267.1	178139.5	149406.8	184773.4	166196.4	161406.3	151761.0	148064.8	173014.0	176562.7	#N/A	#N/A	#N/A	#N/A
Q8R189	Monocyte to macrophage differentiation factor 2	Mmd2	247	28.9	8.53	405645.1	400939.9	381011.7	409997.0	371260.3	345528.9	403737.3	394566.2	415971.9	416939.8	578917.0	484789.9	495708.9	501286.5
P41778	Pre-B-cell leukemia transcription factor 1	Pbx1	430	46.6	7.03	39638.1	35430.9	40693.1	33252.0	50050.7	46111.3	49889.0	47410.5	37159.0	37045.4	76285.8	105249.0	69246.1	90594.0
Q9CX48	Zinc finger CCHC domain-containing protein 10	Zcchc10	178	19.1	9.42	154245.1	180459.5	135080.2	163891.1	137981.0	139706.7	135449.4	128150.1	156037.0	170560.1	#N/A	#N/A	#N/A	#N/A
Q80YA3	Phospholipase DDHD1	Ddhd1	547	61.8	6.61	75058.3	80149.0	80664.9	76087.0	98054.4	81267.3	74500.0	71491.7	76491.7	89453.6	62871.1	61904.8	91838.6	59513.2
Q8R4K2	Interleukin-1 receptor-associated kinase 4	Irak4	459	50.8	5.4	68564.0	62773.5	56046.1	65511.8	62993.4	56404.8	71132.5	71703.3	73005.7	63953.6	#N/A	#N/A	#N/A	#N/A
P15261	Interferon gamma receptor 1	Ifngr1	477	52.3	5.53	261018.5	240431.7	217061.6	227792.3	235034.9	235214.2	220621.2	219552.1	234179.6	257373.4	#N/A	#N/A	#N/A	#N/A
Q3UMR0	Ankyrin repeat domain-containing protein 27	Ankrd27	1048	116.7	6.65	29004.7	29244.9	20881.7	26460.7	25068.1	24919.7	25114.8	20234.7	26457.0	21539.1	92806.4	87802.8	85830.7	85883.0
Q0VBK2	Keratin, type II cytoskeletal 80	Krt80	452	50.6	6.27	129776.3	127936.3	129719.1	146823.1	165306.3	171203.5	154629.5	146317.2	131625.3	157844.1	#N/A	#N/A	#N/A	#N/A
Q9JLC8	Sacsin	Sacs	4582	520.4	7.11	25539.4	36611.1	25467.4	30302.4	28102.2	33417.2	23866.5	27895.4	29715.5	27154.3	#N/A	#N/A	#N/A	#N/A
Q78IK2	ATP synthase membrane subunit DAPIT, mitochondrial	Atp5md	58	6.4	9.83	122757.9	120109.7	129187.0	110428.8	135625.4	125375.1	128421.5	135661.1	127656.5	130376.7	160871.4	158301.8	211195.4	163688.5
Q9WTZ2	Membrane-bound transcription factor site-1 protease	Mbtps1	1052	117.4	8.76	148057.4	123964.0	136743.7	119532.1	131366.6	131288.6	123206.1	142262.7	135411.7	151768.7	59684.0	57847.6	59221.1	54319.4
Q8R0G7	Protein spinster homolog 1	Spns1	528	56.7	7.15	206769.1	210284.7	164682.5	176831.5	178863.0	200747.4	188600.5	169157.3	186142.8	194612.8	38698.7	34798.2	29238.5	34530.9

Q9CWT2	Protein BEX4	Bex4	118	13.8	9.66	119560.7	102538.8	111744.4	96192.4	95285.0	90119.5	99628.7	115205.3	106971.6	120937.7	84474.7	89212.3	86613.4	64185.6
Q9R1S0	B9 domain-containing protein 1	B9d1	204	22.6	6.57	35616.0	40607.9	40213.9	45758.0	40357.7	42632.3	37762.4	36791.3	45345.1	47718.0	#N/A	#N/A	#N/A	#N/A
Q8C172	Ceramide synthase 6	Cers6	384	44.8	7.64	1432132.4	1359554.4	1132987.2	1189000.0	1340343.5	1650186.3	1385835.5	1356553.1	1372661.1	1285909.2	247766.6	222912.8	242070.7	216792.8
Q60963	Platelet-activating factor acetylhydrolase	Pla2g7	440	49.2	7.12	57654.2	62075.3	64704.4	58560.7	65959.5	67798.6	55866.8	60049.9	66235.8	63585.7	#N/A	#N/A	#N/A	#N/A
Q04886	Transcription factor SOX-8	Sox8	464	49.8	7.15	377374.7	381857.2	387418.5	366064.6	378471.7	406194.6	322428.4	347912.4	397361.0	419538.5	66565.8	72837.6	58221.4	65545.4
Q9D8P7	General transcription factor 3C polypeptide 6	Gtf3c6	227	25.5	4.22	195109.3	163963.6	207481.4	183526.7	228526.2	253156.1	218934.9	191191.2	190282.4	177061.1	188270.5	193392.1	189577.7	192406.4
O88466	Zinc finger protein 106	Znf106	1888	208.8	7.59	119217.5	120120.5	109230.2	118519.6	118036.3	111045.1	106605.7	120918.4	106735.9	127454.9	#N/A	#N/A	#N/A	#N/A
Q91V16	Electron transfer flavoprotein regulatory factor 1	Etfrf1	86	10.4	9.73	69806.2	79084.2	76442.2	86010.6	75819.2	78370.2	65236.3	76581.0	77019.2	76335.0	#N/A	#N/A	#N/A	#N/A
Q812F8	Alpha-1,3-mannosyl-glycoprotein 4-beta-N-acetylglucosaminyltransferase B	Mgat4b	548	63.3	7.88	6984.5	9972.0	7710.1	5777.4	7600.8	6677.8	5872.3	7368.3	6707.5	4723.7	#N/A	#N/A	#N/A	#N/A
Q60974	Nuclear receptor corepressor 1	Ncor1	2453	270.5	6.93	77369.9	76341.8	78596.1	70188.6	79315.4	85653.1	85248.7	79794.6	83249.0	81057.9	78808.5	76952.6	78486.8	83442.2
Q923B6	Metalloreductase STEAP4	Steap4	470	53	9.17	145752.8	133546.3	151644.1	126525.9	138492.6	122539.3	157311.9	142144.9	155261.9	162338.4	242600.2	236624.8	215786.2	214257.5
Q6PCX9	E3 ubiquitin-protein ligase TRIM37	Trim37	961	107.6	5.21	13698.0	18291.8	16015.4	13549.8	18308.5	19291.0	15689.8	18065.1	18601.9	17336.8	#N/A	#N/A	#N/A	#N/A
P63030	Mitochondrial pyruvate carrier 1	Mpc1	109	12.4	9.61	128673.3	155923.7	146694.2	130244.4	165510.9	149347.9	145907.9	121269.9	138581.6	134225.1	313678.6	338864.8	350979.9	336493.2
Q5SWZ9	Mitochondrial cardiolipin hydrolase	Pld6	221	25	9.09	217523.2	240376.0	223312.6	241234.8	225661.9	197942.8	193918.0	200515.0	210640.8	226461.3	174868.4	204038.3	163872.5	193874.7
Q8C2L6	Transmembrane protein 161B	Tmem161b	487	55.4	8.37	94788.8	101552.3	82662.5	98491.0	84820.9	79787.0	87380.4	71371.3	97573.4	90911.4	#N/A	#N/A	#N/A	#N/A
D3Z2R5	Selenoprotein N	Selenon	557	62.5	5.55	40776.8	41083.9	34359.9	38463.5	41525.0	40192.1	40752.1	34353.5	38573.8	41977.1	35973.1	35185.9	34107.7	35989.9
Q8R1Z4	Protein phosphatase 1 regulatory subunit 42	Ppp1r42	357	41.1	8.34	254194.3	265959.6	294631.9	244523.3	296365.1	247450.1	247136.8	232948.7	256473.6	277469.6	#N/A	#N/A	#N/A	#N/A
P42337	Phosphatidylinositol 4,5-bisphosphate 3-kinase catalytic subunit alpha isoform	Pik3ca	1068	124.3	7.15	175012.3	188811.2	150648.5	166346.6	166239.0	189969.3	186214.4	153914.4	182371.5	173055.8	126958.4	140814.8	123261.4	132992.5
Q99J25	rRNA methyltransferase 1, mitochondrial	Mrm1	320	34.8	8.81	65691.0	78345.3	60060.5	86957.8	78698.1	101563.4	85029.5	69479.9	76185.0	65648.0	41120.0	52456.0	41487.5	50156.6
Q7TQG1	Pleckstrin homology domain-containing family A member 6	Plekha6	1173	131.3	8.97	31193.1	44027.5	46318.7	51092.2	56680.0	76366.3	51366.4	52490.8	38683.4	36874.9	#N/A	#N/A	#N/A	#N/A
Q7TSV9	Enkurin domain-containing protein 1	Enkd1	346	38.9	10.32	108732.8	116543.1	101224.2	102576.2	119580.2	112143.1	112634.5	108990.5	109992.5	116347.6	#N/A	#N/A	#N/A	#N/A
Q9D600	DNA replication complex GINS protein PSF2	Gins2	185	21.2	5.29	157646.8	195420.8	199635.4	218014.0	230188.1	221646.5	166524.0	191278.9	208889.0	222177.2	63498.4	73587.1	65673.8	76673.8
A2ACP1	Tetratricopeptide repeat protein 39A	Ttc39a	578	66.1	7.31	847628.5	846919.2	565404.0	862815.4	611136.4	594781.0	566168.1	535491.2	789680.4	862833.4	1675453.1	1642448.1	968125.0	1647364.3
Q8K0Z7	Translational activator of cytochrome c oxidase 1	Taco1	294	32.3	8.12	455699.5	424376.7	486272.7	430616.6	452269.1	437656.6	395919.7	442082.6	467430.4	467525.3	522907.9	484990.4	532303.6	487939.9
P11214	Tissue-type plasminogen activator	Plat	559	63.1	8.15	141562.7	128841.9	124012.1	127847.2	147848.8	137221.8	141506.2	116176.3	139662.2	138980.2	#N/A	#N/A	#N/A	#N/A
Q9QXW2	F-box/WD repeat-containing protein 5	Fbxw5	573	64.6	6.13	106140.6	133973.4	112090.0	142702.9	96539.6	105883.1	96223.8	91819.7	104116.7	110367.3	#N/A	#N/A	#N/A	#N/A
Q9D8H7	Metalloendopeptidase OMA1, mitochondrial	Oma1	521	58.8	9.39	97446.8	98536.4	94303.2	96853.0	86093.0	91683.8	91333.4	103049.8	99597.3	91443.8	53098.3	54659.7	46051.8	44295.6
Q9D0I6	WD repeat, SAM and U-box domain-containing protein 1	Wdsub1	474	51.7	6.34	242710.7	126262.3	180139.4	283091.3	179315.3	204956.0	105092.7	105744.6	99159.2	127649.3	376035.5	270954.6	328897.3	505867.0
P61264	Syntaxin-1B	Stx1b	288	33.2	5.38	155772.5	155631.8	153040.2	140470.4	165818.2	193668.6	206827.1	170484.1	143947.3	153635.3	#N/A	#N/A	#N/A	#N/A
E9Q3M9	DUF4592 domain-containing protein	10300C02l	1175	125.8	7.37	318623.8	211808.2	259103.8	230595.0	280935.6	308228.2	297408.3	288882.4	265178.1	246863.2	#N/A	#N/A	#N/A	#N/A
P11835	Integrin beta-2	Itgb2	771	85	7.12	75223.3	76562.1	63939.3	74690.6	60610.3	74229.0	74508.7	77887.8	69178.0	72331.4	#N/A	#N/A	#N/A	#N/A
O88513	Geminin	Gmnn	206	23.3	4.67	39795.3	46835.7	39243.7	36143.0	34869.9	33185.5	31426.8	32427.9	31001.9	42021.1	50305.0	86577.9	46217.5	57409.7
Q8K136	Sodium channel modifier 1	Scnm1	229	25.8	8.66	72752.3	83261.9	95538.1	90087.5	88589.9	94386.4	84506.7	87930.5	87374.5	98196.2	53266.1	55314.4	54512.5	52424.7
Q8K2A1	PTB domain-containing engulfment adapter protein 1	Gulp1	304	34.4	7.9	98645.5	94533.8	96589.1	93274.0	106262.4	115942.1	116991.5	108574.4	103642.3	100241.4	82399.4	78864.4	96901.1	86837.6
Q8CDK2	Cytosolic carboxypeptidase 2	Agbl2	862	99.2	8.59	123476.4	171923.3	150043.9	195047.7	112628.4	97342.2	93997.5	107408.4	115842.6	153006.7	#N/A	#N/A	#N/A	#N/A
P17665	Cytochrome c oxidase subunit 7C, mitochondrial	Cox7c	63	7.3	11	143880.3	131075.6	171004.4	164716.7	146576.1	116697.7	126777.5	151239.5	144808.4	137930.5	75888.9	88251.1	75300.3	89281.1
Q60989	E3 ubiquitin-protein ligase XIAP	Xiap	496	56	6.23	40962.2	47544.5	42903.4	41105.9	44013.8	48621.6	46855.5	42768.1	44582.4	48137.2	#N/A	#N/A	#N/A	#N/A
Q91YK0	Leucine-rich repeat-containing protein 49	Lrrc49	686	78.8	8.13	27579.6	39639.2	35333.7	38444.3	31885.2	33604.1	30619.6	32479.3	39139.0	36239.2	362759.4	357820.3	363945.0	353996.4
Q8BXK4	S-adenosylmethionine sensor upstream of mTORC1	Bmt2	403	45.9	6.27	44035.3	57140.6	52339.8	50027.1	52124.9	40974.7	43793.9	43361.1	50432.5	57383.8	84471.2	93726.9	84747.3	94722.4
Q9D4Y3	Homeobox domain-containing protein	Rhox2a	191	21.1	4.93	47880.2	47342.6	41578.8	48835.0	58690.5	104255.6	55360.9	51780.1	49361.4	37787.2	#N/A	#N/A	#N/A	#N/A
Q2EMV9	Protein mono-ADP-ribosyltransferase PARP14	Parp14	1817	203.7	6.79	85681.9	119946.6	89538.7	98467.1	113929.3	91358.2	82971.1	94469.2	87405.4	106435.6	#N/A	#N/A	#N/A	#N/A
Q9D1Q1	M-phase phosphoprotein 6	Mphosph6	161	19.1	5.3	202761.5	209843.4	208178.5	205870.5	238328.5	250085.8	214102.8	200336.9	202292.6	225658.8	190377.1	266466.6	272460.2	259988.9
Q9JMA9	Sodium- and chloride-dependent neutral and basic amino acid transporter B(0+)	Slc6a14	638	71.4	7.24	162031.1	149233.1	125204.8	134300.0	115692.6	109967.1	120784.3	131371.9	157415.8	120282.2	#N/A	#N/A	#N/A	#N/A
Q60775	ETS-related transcription factor Elf-1	Elf1	612	66.2	4.97	24105.8	50331.5	24126.5	36468.4	55810.5	50991.0	32861.8	33799.6	39292.5	50138.9	#N/A	#N/A	#N/A	#N/A
Q9WTM3	Semaphorin-6C	Sema6c	931	99.5	8.18	72770.3	81016.2	66138.5	65959.8	82238.1	76616.9	68347.2	68352.3	73953.5	79351.6	#N/A	#N/A	#N/A	#N/A
Q8R105	Vacuolar protein sorting-associated protein 37C	Vps37c	352	38.4	5.31	11401.9	15928.6	13602.8	18395.2	15482.4	15431.9	14979.8	17132.2	14179.1	15620.1	#N/A	#N/A	#N/A	#N/A
Q8BQS5	Adiponectin receptor protein 2	Adipor2	386	44	6.34	75834.1	97315.0	80642.8	88925.9	90874.6	97311.6	78699.7	81994.6	88432.3	83447.2	#N/A	#N/A	#N/A	#N/A
Q9CQE5	Regulator of G-protein signaling 10	Rgs10	181	21.1	6.81	580510.1	499358.4	420673.7	537494.8	548996.9	732490.3	730237.0	594779.3	504330.1	460736.6	#N/A	#N/A	#N/A	#N/A
Q80W68	Kin of IRRE-like protein 1	Kirrel1	789	87.1	5.92	112359.2	115019.3	134796.9	120579.3	123189.7	123932.0	126536.9	114463.5	115822.7	126472.7	#N/A	#N/A	#N/A	#N/A
A0A494B9V8	Protein prenyltransferase alpha subunit repeat-containing 1	Ptar1	424	48.3	6.98	213265.7	211431.6	200892.7	203872.7	221110.8	243899.6	260751.5	221479.5	250229.6	227477.3	#N/A	#N/A	#N/A	#N/A
Q9Z239	Phospholemman	Fxyd1	92	10.3	8.54	10946.9	7163.9	6605.0	8193.0	9964.3	10040.9	12569.6	9072.0	10208.2	9404.4	34813.9	32501.1	29886.1	36923.5
Q91WL6	Sorting nexin-11	Snx11	271	30.4	5.72	130245.1	161731.0	151443.9	136908.6	156725.0	146001.4	125729.3	126609.5	149696.4	164553.0	133711.9	111035.5	98126.6	109768.1
Q3TV70	Nuclear receptor 2C2-associated protein	Nr2c2ap	140	15.8	5.26	5847.4	6452.5	4970.3	6402.6	7021.6	8478.1	4525.2	3860.8	5528.4	5418.4	26381.6	25914.0	24698.9	24047.9
Q9D211	CDKN2AIP N-terminal-like protein	Cdkn2aipnl	116	13.2	5.29	109402.8	105610.7	109899.2	111308.0	127938.3	133343.6	117496.3	111227.5	124717.1	122968.0	84588.4	86762.3	88906.1	89229.7
Q922C1	Uncharacterized protein C19orf44 homolog	MGI:191950	641	69.2	5.85	7364.1	8803.7	7831.1	9388.4	5652.6	6861.9	5765.4	8315.0	4764.0	6427.5	#N/A	#N/A	#N/A	#N/A
Q9WTS6	Teneurin-3	Tenm3	2715	302.9	6.51	39185.1	43450.0	38786.1	39217.2	40508.9	41892.1	41498.9	38234.3	36951.9	38233.8	#N/A	#N/A	#N/A	#N/A
Q3V1N5	Headcase domain-containing protein	Heca	544	59.1	8.6	12099.0	18921.1	14030.0	14947.0	19065.9	18937.6	14267.5	17827.0	16704.1	20610.9	#N/A	#N/A	#N/A	#N/A
Q9QZN1	F-box/LRR-repeat protein 17	Fbxl17	701	75.6	8.1	94667.4	127844.5	101862.7	110373.1	119573.2	122675.1	107442.6	105867.5	104188.5	118902.7	163177.4	230224.3	163710.7	182404.3
Q5NCX5	Neuralized-like protein 4	Neurl4	1563	167.5	5.95	38187.8	40274.7	42014.5	41770.0	36248.2	39699.0	36987.5	30501.0	36119.8	40436.6	39773.2	39930.2	46593.4	39138.2
Q8BHN5	RNA-binding protein 45	Rbm45	476	53.3	7.69	128788.3	110579.6	104616.8	107068.7	126170.2	121709.5	113085.3	115786.1	126115.0	129804.9	300971.7	277697.8	300657.6	303158.0
Q9R062	Glycogenin-1	Gyg1	333	37.4	5.29	23773.1	20640.3	17119.5	18861.0	18695.9	20773.4	18704.7	19056.3	17064.3	20638.7	222278.0	220424.6	203938.7	221751.5
Q8BHL7	CDC42 small effector protein 1	Cdc42se1	80	9.1	9.99	90568.5	71322.6	85674.0	69572.4	73072.3	82304.0	75749.9	86591.1	75298.7	76958.3	#N/A	#N/A	#N/A	#N/A
P70385	Testosterone 17-beta-dehydrogenase 3	Hsd17b3	305	34.3	9.29	287063.6	302452.0	276510.3	273655.6	281411.8	295159.3	291707.8	260884.7	281736.7	280646.4	#N/A	#N/A	#N/A	#N/A
Q80SZ7	Guanine nucleotide-binding protein G(I)/G(S)/G(O) subunit gamma-5	Gng5	68	7.3	9.85	426981.3	616484.0	500637.4	570251.4	535847.5	524377.9	517153.9	501503.7	590706.2	568301.8	#N/A	#N/A	#N/A	#N/A
Q9CZ91	Serum response factor-binding protein 1	Srfbp1	441	48.7	9.54	369888.9	396937.8	420565.0	351593.0	409220.1	438892.1	407523.4	376939.0	406553.3	363032.5	291865.4	286166.4	284238.5	259116.1
Q6ZQ12	Ninein-like protein	Ninl	1394	157.7	5.01	209060.7	210859.4	204745.8	247110.9	205667.6	210075.3	187808.2	188570.5	205629.5	207011.1	232143.4	237279.0	188445.2	221226.7
P00848	ATP synthase subunit a	Mtatp6	226	25.1	10.43	266598.3	304057.2	245694.9	281254.7	258775.5	276451.5	263664.7	249777.7	278808.2	296012.6	77234.1	109732.2	81460.2	82519.0
Q8K4I3	Rho guanine nucleotide exchange factor 6	Arhgef6	771	87	5.87	267058.8	256081.9	226998.1	269896.6	247658.4	286350.5	289288.3	268154.0	230580.7	247954.7	#N/A	#N/A	#N/A	#N/A
Q3U4I7	Pyridine nucleotide-disulfide oxidoreductase domain-containing protein 2	Pyroxd2	581	62.9	8.29	147709.8	145948.0	149889.3	147480.1	153637.0	171590.9	159548.8	154612.3	140568.6	142566.8	29119.8	25639.4	31997.3	30425.7
Q3UHI4	Protein TMED8	Tmed8	326	35.8	4.74	212929.6	193891.3	203710.2	193481.3	222394.1	200791.9	232884.6	208822.6	215311.6	210533.7	176669.0	184821.8	159716.6	157020.9
Q3UY34	Protein CUSTOS	Custos	256	27.6	8.7	79839.0	80103.0	87545.7	85127.2	88537.5	89809.5	84246.6	84102.4	82561.1	78357.9	187173.1	204280.5	173339.9	219224.4
Q8BSU7	MOB kinase activator 3A	Mob3a	217	25.5	8.43	369970.1	361126.6	344421.7	356314.0	343615.7	366423.4	370519.1	332515.3	357998.0	385010.5	#N/A	#N/A	#N/A	#N/A
Q5SPW0	Vacuolar protein sorting-associated protein 54	Vps54	977	110.3	6.49	42641.6	62073.0	38598.6	46403.9	43237.2	46925.7	35082.3	38989.6	44648.6	47991.1	103110.8	105029.5	106593.8	104487.6
P05555	Integrin alpha-M	Itgam	1153	127.4	7.27	53147.8	64418.2	47418.9	54419.9	59192.3	65069.0	55704.5	58254.5	62411.0	59554.6	#N/A	#N/A	#N/A	#N/A
O35607	Bone morphogenetic protein receptor type-2	Bmpr2	1038	114.9	6.23	68775.6	76833.3	77438.9	54192.2	83835.3	75752.3	83274.7	66011.3	69485.8	80957.5	#N/A	#N/A	#N/A	#N/A
A2AG58	BCLAF1 and THRAP3 family member 3	Bclaf3	752	90.1	9.58	128704.2	133801.1	133096.9	142084.5	136475.1	140423.2	133986.1	143935.0	127321.4	124346.6	31118.5	32985.1	33278.6	29129.7
Q149L6	DnaJ homolog subfamily B member 14	Dnajb14	379	42.3	8.59	220443.7	235956.2	236044.6	251330.7	190901.8	202436.1	188746.0	214655.3	196054.2	221973.2	#N/A	#N/A	#N/A	#N/A
O35657	Sialidase-1	Neu1	409	44.6	6.02	300398.7	297773.8	282970.6	292136.1	317477.6	312375.9	292761.8	258372.2	282906.0	305564.1	#N/A	#N/A	#N/A	#N/A
Q9JKN1	Zinc transporter 7	Slc30a7	378	41.8	6.84	331750.8	314785.9	332105.5	321179.1	295655.1	284609.8	277542.3	281168.1	306243.0	327383.8	#N/A	#N/A	#N/A	#N/A
Q08376	Zinc finger and BTB domain-containing protein 14	Zbtb14	449	50.9	6.04	37904.6	41537.8	30542.4	36094.0	42068.1	43394.2	36550.8	37877.9	35819.5	46917.7	#N/A	#N/A	#N/A	#N/A
Q8K4L4	Protein POF1B	Pof1b	587	67.7	6.23	116463.0	129075.0	117309.3	107093.9	145075.9	126696.9	118104.3	111492.8	116565.2	121512.7	#N/A	#N/A	#N/A	#N/A
Q64311	Protein N-terminal asparagine amidohydrolase	Ntan1	310	34.6	6.19	8040.4	12398.0	8550.2	8960.8	8371.6	11140.5	11353.3	5372.5	12143.4	10123.6	82612.6	94134.1	90403.4	92602.0
Q6PEM8	Proton-coupled folate transporter	Slc46a1	459	50.1	8.37	65161.6	75778.7	67135.7	76892.9	74240.6	73973.2	75024.3	63227.3	74577.4	65451.9	#N/A	#N/A	#N/A	#N/A
P51910	Apolipoprotein D	Apod	189	21.5	4.91	157455.7	195495.0	166281.4	161207.6	183434.9	200973.0	156643.2	175701.8	163778.8	189406.0	367575.0	273946.2	305287.4	220658.8
Q9Z223	Molybdopterin synthase catalytic subunit	Mocs2	189	20.9	5.96	142644.5	132223.2	129743.9	121566.0	164813.6	149509.8	123676.2	129652.0	137552.9	140378.3	#N/A	#N/A	#N/A	#N/A
Q8R1K4	5-phosphohydroxy-L-lysine phospho-lyase	Phykpl	467	51.9	6.46	159793.0	180258.6	189070.2	195035.6	193834.3	179290.6	190156.9	161991.4	195164.5	206547.8	#N/A	#N/A	#N/A	#N/A
P22437	Prostaglandin G/H synthase 1	Ptgs1	602	69	6.83	8468.9	10528.8	9178.4	8825.5	8766.8	9923.5	7183.2	8207.0	7064.5	8595.2	16599.3	15042.4	13603.5	15302.0
P21958	Antigen peptide transporter 1	Tap1	724	78.8	8.79	13421.0	12088.8	10518.9	10504.5	11808.1	13530.2	16758.8	10694.8	11153.7	12890.9	#N/A	#N/A	#N/A	#N/A
Q9D8W7	OCIA domain-containing protein 2	Ociad2	154	16.9	9.41	162032.7	186154.9	138919.1	166976.4	154464.7	190231.9	145157.4	165770.1	162774.3	171487.8	95836.7	108298.6	75722.5	94429.6
Q6KAU4	Multivesicular body subunit 12B	Mvb12b	317	35.4	7.93	28513.9	28070.4	23712.0	29427.3	32220.5	31603.9	29534.1	27811.3	24229.0	29889.6	#N/A	#N/A	#N/A	#N/A
Q60612	Adenosine receptor A1	Adora1	326	36.6	8.48	15064.4	18537.3	19857.6	22794.8	23310.9	21586.3	26424.2	23251.4	24592.1	19634.2	#N/A	#N/A	#N/A	#N/A
Q8BTZ5	Ankyrin repeat domain-containing protein 46	Ankrd46	228	25.2	5.73	99261.5	86299.4	86259.7	89258.7	82513.4	93026.5	92252.8	80363.8	88388.0	87958.4	117911.5	129966.3	123496.8	120586.7

Q6PAT0	Probable inactive tRNA-specific adenosine deaminase-like protein 3	Adat3	349	37.5	7.23	16952.3	16337.1	14908.4	17035.2	19246.9	16641.2	21416.8	16261.3	15100.2	18301.5	74531.6	81506.5	65736.4	65750.8
Q9D1I2	Caspase recruitment domain-containing protein 19	Card19	183	20.9	8.03	154690.1	170985.5	143444.4	153369.8	172950.5	145732.4	151996.7	135764.6	139860.3	166828.4	#N/A	#N/A	#N/A	#N/A
Q9D9N8	Protease-associated domain-containing protein 1	Pradc1	188	21.1	5.72	73465.9	107912.5	71636.7	89447.3	80575.8	77105.0	68167.5	73741.5	77234.7	91281.2	#N/A	#N/A	#N/A	#N/A
Q9WTL8	Aryl hydrocarbon receptor nuclear translocator-like protein 1	Arntl	632	69.4	6.58	64458.3	55149.8	54314.7	52559.8	54321.2	56851.3	51131.4	52419.5	55111.9	55249.0	80669.1	91095.6	87158.4	85030.5
Q8BXV2	BRI3-binding protein	Bri3bp	253	28.2	9.52	110532.6	130452.2	133750.6	141877.6	117125.7	112399.4	127045.6	111040.2	128593.5	140390.3	#N/A	#N/A	#N/A	#N/A
Q8CDJ8	Stonin-1	Ston1	730	81.7	6.35	173483.4	181708.3	197251.2	159931.9	220488.9	263866.3	245320.1	200990.4	196895.7	198017.9	#N/A	#N/A	#N/A	#N/A
P42867	UDP-N-acetylglucosamine--dolichyl-phosphate N-acetylglucosaminephosphotransferase	Dpagt1	410	46.4	7.87	228866.0	214209.9	205482.3	190940.2	225193.5	228153.9	238884.1	238650.5	208939.1	202348.6	#N/A	#N/A	#N/A	#N/A
A2A7F4	Rearranged L-myc fusion sequence	Rlf	1918	217.3	6.71	15182.2	17477.9	17524.5	13182.8	12343.0	15751.1	15031.9	11845.3	14996.1	13544.6	#N/A	#N/A	#N/A	#N/A
Q8C753	Protein KIAA0556	Kiaa0556	1610	179.5	5.26	44802.3	63923.6	48962.1	56621.7	52897.8	57438.5	41806.5	52690.8	56301.8	67078.5	#N/A	#N/A	#N/A	#N/A
P81122	Insulin receptor substrate 2	Irs2	1321	136.7	8.69	156984.0	166071.1	158458.0	159202.9	186576.8	194868.4	154815.0	162668.8	165588.5	169293.2	178996.7	196155.9	171030.3	183095.8
Q8C6P5	PIH1_CS domain-containing protein	Pih1h3b	218	24.5	4.42	105685.1	133220.4	107331.3	128457.6	98041.7	101140.1	83492.4	97245.4	113079.3	114904.3	#N/A	#N/A	#N/A	#N/A
Q7TPN3	GPI mannosyltransferase 2	Pigv	493	55	8.1	33260.7	51918.8	36716.0	50062.6	46517.0	45009.3	39724.1	50589.1	43149.7	54287.0	#N/A	#N/A	#N/A	#N/A
Q9D0Y8	39S ribosomal protein L52, mitochondrial	Mrpl52	121	13.6	10.93	76070.5	100979.9	78602.6	80663.2	89752.0	89167.7	84090.4	86298.4	90693.6	87256.3	#N/A	#N/A	#N/A	#N/A
Q8CDM4	Coiled-coil domain-containing protein 73	Ccdc73	1066	120.3	5.68	54847.5	70105.8	60207.6	62883.1	59811.8	64698.9	50106.4	51140.5	65302.7	64765.0	#N/A	#N/A	#N/A	#N/A
P0CB42	Nucleic acid dioxygenase ALKBH1	Alkbh1	389	43.7	8.22	2784.7	2465.3	4179.0	3309.7	3913.9	3199.8	2587.1	4658.9	2400.0	5037.9	#N/A	#N/A	#N/A	#N/A
Q9D650	Protein LRATD1	Lratd1	292	32.7	5.6	68283.8	76918.5	61307.1	70287.7	59591.1	69739.8	60114.8	59608.9	74451.6	76185.9	#N/A	#N/A	#N/A	#N/A
Q62179	Semaphorin-4B	Sema4b	823	91.3	8.15	109909.1	125362.1	100655.3	117889.4	104683.5	122043.8	114593.1	111485.4	110139.6	107012.8	#N/A	#N/A	#N/A	#N/A
F8VPN2	Testis-expressed protein 15	Tex15	2785	311.1	6.39	31071.6	30090.0	29383.3	27158.7	34486.9	29552.7	28719.1	26836.1	23675.0	32136.6	#N/A	#N/A	#N/A	#N/A
P97762	Retinitis pigmentosa 9 protein homolog	rp9	213	25.2	9.83	496540.3	553411.0	500856.4	620495.2	499838.7	487702.1	649874.0	635005.0	557785.4	656297.9	457571.5	482587.1	490303.1	521603.8
Q9CZP7	Hsp90 co-chaperone Cdc37-like 1	Cdc37l1	335	38.4	5.39	250071.7	314758.9	243845.0	305270.5	241192.0	205032.8	262930.7	219541.8	283360.5	281468.9	52736.3	51297.8	48645.0	50288.5
Q9R1Q7	Proteolipid protein 2	Plp2	152	16.6	7.14	9937.6	11541.9	3385.8	9848.0	2667.4	4117.5	4604.4	3570.2	13391.5	10308.6	35690.0	34598.7	39872.6	37201.7
Q9WTZ0	Protein UXT	Uxt	157	18.2	5.81	82176.0	72051.5	80129.8	82177.0	98655.8	82145.3	94977.4	82975.6	79009.9	93390.6	#N/A	#N/A	#N/A	#N/A
Q6WBX7	Cell cycle checkpoint control protein RAD9B	Rad9b	403	44.8	5.49	203437.6	221613.9	184149.2	206236.6	220043.2	192860.9	183637.0	177826.8	205941.7	208391.9	51350.4	66812.8	68225.2	59376.9
Q8BGS7	Choline/ethanolaminephosphotransferase 1	Cept1	416	46.4	8.1	217286.8	219052.9	200518.2	225957.9	231208.5	240198.6	222010.6	241271.0	217568.9	211358.6	321567.2	303994.8	269439.4	285453.9
Q9CS00	Cactin	Cactin	772	90.6	9.79	95012.1	101326.1	93345.5	101679.9	101934.2	113797.7	102187.4	94497.5	95102.2	110539.8	154261.5	146118.0	145641.1	129443.9
Q8BJU9	Peptide chain release factor 1-like, mitochondrial	Mtrf1l	373	42.2	8.16	90110.4	83005.2	81566.8	93997.8	102156.6	106940.8	72865.1	80422.7	86388.9	90184.2	83232.6	86759.4	88704.6	70464.5
Q9CWV4	Melanoma-associated antigen B16	Mageb16	363	40.9	4.27	140915.4	149834.7	117703.1	134398.1	130704.4	132420.1	117553.0	127915.7	134820.1	140486.3	#N/A	#N/A	#N/A	#N/A
Q8R104	NAD-dependent protein deacetylase sirtuin-3	Sirt3	334	36.6	7.44	91026.9	85925.6	91104.9	98750.0	101818.6	109768.5	85511.7	100184.2	92525.9	88304.4	#N/A	#N/A	#N/A	#N/A
D3Z4S3	Putative peptidyl-tRNA hydrolase PTRHD1	Ptrhd1	140	16	9.32	120372.7	133531.7	134649.6	140735.2	133644.9	144544.4	163161.3	144383.1	156907.4	117003.3	69721.7	57227.7	59956.8	56936.0
Q8R1L4	ER lumen protein-retaining receptor 3	Kdelr3	214	25.1	9.06	198381.1	206129.9	164242.7	202227.3	157445.7	170659.2	156706.3	215134.4	170947.2	177327.2	201099.4	221573.1	233535.2	247689.5
Q9CZJ6	Protein Mis18-alpha	Mis18a	204	22.9	5.27	105931.5	117598.3	96667.4	110756.9	125927.7	119210.8	102559.4	99049.8	110543.5	116305.3	#N/A	#N/A	#N/A	#N/A
P17679	Erythroid transcription factor	Gata1	413	42.6	8.6	12469.8	13674.1	14361.7	14718.4	17889.1	19420.5	18716.0	16936.5	16215.5	19197.7	68661.4	70776.5	61820.6	63099.0
Q8K0D7	Guided entry of tail-anchored proteins factor 1	Get1	174	19.9	9.79	218132.7	250798.8	231600.6	236568.0	230771.1	229172.0	215146.4	212579.6	238153.3	235049.8	#N/A	#N/A	#N/A	#N/A
Q9CR47	Ribosome biogenesis protein NSA2 homolog	Nsa2	260	30	10.24	107627.1	115966.4	98214.8	101418.5	102687.9	107166.9	93874.0	94139.6	100015.3	112671.9	281070.4	288169.4	269593.9	275425.8
Q8VCA6	Transmembrane protein 161A	Tmem161a	480	54	8.38	149469.1	144316.8	154547.2	136730.1	147329.6	151153.8	140104.2	157199.2	143381.0	140819.8	139549.3	147758.8	121543.1	142245.7
P0CG14	Decreased expression in renal and prostate cancer protein	Derpc	533	52.2	12.32	18156.7	14463.1	24606.1	21049.0	22900.9	20769.5	22395.7	19348.9	20033.5	26145.0	17634.3	21765.2	22062.3	21046.0
Q9D2I5	LisH domain-containing protein ARMC9	Armc9	817	91.9	6.92	236463.4	278262.2	204472.6	262074.1	223160.3	194096.4	194304.3	197226.1	210242.4	251857.9	#N/A	#N/A	#N/A	#N/A
Q91WL8	WW domain-containing oxidoreductase	Wwox	414	46.5	7.02	384698.6	333059.6	353886.5	341691.1	314142.9	330911.4	371334.3	358773.3	320599.7	324203.5	#N/A	#N/A	#N/A	#N/A
Q9JJR8	Transmembrane protein 9B	Tmem9b	199	22.6	8.18	181777.7	183194.9	220770.3	188969.6	177246.8	196434.6	170128.7	204158.9	195503.9	185704.3	184012.0	225515.9	167707.4	219324.5
Q3TP92	CTD nuclear envelope phosphatase 1	Ctdnep1	244	28.4	9.76	260140.3	294057.7	285251.0	274231.9	293273.1	247189.0	175915.6	279569.4	243877.6	308300.8	#N/A	#N/A	#N/A	#N/A
Q8CG19	Latent-transforming growth factor beta-binding protein 1	Ltbp1	1712	186.6	6.02	122495.2	115350.1	113585.6	102172.9	104954.6	137582.3	128216.4	128171.9	122197.0	118418.3	#N/A	#N/A	#N/A	#N/A
Q9D1D1	Tetraspanin-11	Tspan11	253	28.1	7.36	119846.5	124073.1	119949.3	125897.4	126686.3	131305.6	128436.1	114982.0	128592.9	118914.5	#N/A	#N/A	#N/A	#N/A
Q99P58	Ras-related protein Rab-27B	Rab27b	218	24.5	5.54	4840.4	4137.9	5717.3	6325.6	7481.9	9085.4	10202.3	6233.2	6012.8	5121.4	#N/A	#N/A	#N/A	#N/A
P16301	Phosphatidylcholine-sterol acyltransferase	Lcat	438	49.7	6.43	143861.1	163184.2	142925.8	175646.7	140777.5	162974.6	134262.4	136045.0	157191.8	154703.1	141027.5	135182.4	134232.6	125426.4
O55028	[3-methyl-2-oxobutanoate dehydrogenase [lipoamide]] kinase, mitochondrial	Bckdk	412	46.6	8.91	177578.1	164302.4	157474.4	140006.5	171323.2	173955.0	168340.7	151593.2	171273.9	171467.6	38404.1	42648.3	36836.0	35756.4
Q6IQX7	Chondroitin sulfate synthase 2	Chpf	774	85.5	6.84	55568.6	68151.6	46619.5	57394.6	51849.2	56515.7	42462.8	49248.3	53760.9	55904.4	74857.9	82487.7	66946.2	77721.2
A0A140T8M3	Ig-like domain-containing protein	Igkv8-30	121	13.3	6.49	197509.9	183515.8	219490.3	213991.9	197709.2	210589.6	184264.1	183510.0	209484.6	214639.1	#N/A	#N/A	#N/A	#N/A
Q9WVQ0	Polyamine-modulated factor 1-binding protein 1	Pmfbp1	1022	119.3	6.58	627994.6	553891.7	665505.6	577561.8	736338.6	698330.7	721895.7	605578.6	688712.4	704970.2	#N/A	#N/A	#N/A	#N/A
B1AV60	NHS actin-remodeling regulator	Nhs	1647	178.9	7.01	94514.0	83140.8	100507.2	66060.2	105321.9	102990.2	86313.0	94961.8	88408.1	81715.7	40845.2	37667.2	34223.9	37685.7
Q9Z0H6	Cystatin-9	Cst9	137	16.1	9.03	202723.6	180884.3	211848.8	186586.1	186426.7	171956.0	176766.3	240454.6	201836.9	204062.9	#N/A	#N/A	#N/A	#N/A
P97465	Docking protein 1	Dok1	482	52.4	6.57	158020.5	172786.6	154085.8	169515.2	159721.0	164014.0	172133.1	153579.7	161869.5	169537.3	#N/A	#N/A	#N/A	#N/A
Q9DB94	WD repeat-containing protein 53	Wdr53	358	38.6	7.09	151734.2	150470.8	146839.4	180520.6	134587.8	151312.3	156570.5	157345.6	158690.4	157217.0	136459.4	156282.9	134899.4	158106.9
Q8BXN7	Protein phosphatase 1K, mitochondrial	Ppm1k	372	40.9	6.39	290451.1	294284.3	276806.4	263965.8	278341.3	301837.0	345267.2	286831.9	299137.8	274923.0	#N/A	#N/A	#N/A	#N/A
Q8BGB8	Ubiquinone biosynthesis protein COQ4 homolog, mitochondrial	Coq4	266	30.1	9.26	103057.5	192354.6	121729.7	157737.8	129457.2	168807.3	112272.2	166335.0	117136.0	146815.6	#N/A	#N/A	#N/A	#N/A
P13705	DNA mismatch repair protein Msh3	Msh3	1091	123	7.91	103746.1	109820.8	110324.8	97275.2	108874.5	114946.4	108495.4	108775.1	100250.5	98515.6	#N/A	#N/A	#N/A	#N/A
Q8JZZ7	Adhesion G protein-coupled receptor L2	Adgrl2	1487	166.5	6.71	134797.3	132524.8	134912.9	108543.2	165814.4	168899.8	169636.0	142321.3	154564.7	146005.8	122901.2	84716.7	92082.2	80948.2
Q9CZL2	Uncharacterized protein FAM241A	Fam241a	131	14.5	4.97	234342.9	250258.3	219022.6	256743.9	220204.9	227580.9	209404.4	192677.0	235717.4	259864.1	231492.4	254342.4	205876.4	255889.2
Q8BQR4	KAT8 regulatory NSL complex subunit 2	Kansl2	486	54.1	6.79	100842.2	107480.3	93093.9	95331.7	112224.2	113346.0	97228.2	90807.6	102542.5	103350.9	#N/A	#N/A	#N/A	#N/A
F6XLV1	Ciliary rootlet coiled-coil, rootletin family member 2	Crocc2	1638	186.5	6.13	12572.4	10113.1	10871.5	13178.0	12235.9	14856.0	34078.1	13697.9	11629.6	12625.1	#N/A	#N/A	#N/A	#N/A
Q8BJU2	Tetraspanin-9	Tspan9	239	26.7	7.42	125561.0	125351.9	106726.0	114856.7	135622.6	130523.2	122496.1	124829.6	119203.0	116300.3	#N/A	#N/A	#N/A	#N/A
Q8VCK5	Kelch-like protein 20	Klhl20	604	67.4	6.87	179089.6	227596.2	179243.1	170498.4	181289.0	176239.9	162700.8	165040.6	180974.0	205386.9	#N/A	#N/A	#N/A	#N/A
Q9D1F3	Uncharacterized protein CXorf40 homolog	MGI:191586	157	18	5.19	56946.2	60148.4	54690.1	66090.8	58277.3	57743.0	57466.4	58005.6	53128.3	61003.4	#N/A	#N/A	#N/A	#N/A
Q9EPR2	Group XIIA secretory phospholipase A2	Pla2g12a	192	21.3	7.15	88191.4	104895.5	113640.2	119375.1	99260.7	93748.9	94285.0	102197.1	91750.3	86199.1	#N/A	#N/A	#N/A	#N/A
Q6R2P8	Endonuclease 8-like 2	Neil2	329	36.8	8.5	65694.2	70829.3	58266.6	72267.8	76530.8	85885.5	73693.8	61395.0	70720.1	65323.6	78639.3	84622.0	84676.3	88563.5
A2AG10	Nsp1_C domain-containing protein	Nup62cl	272	31.2	4.68	36734.3	46621.6	37036.2	40511.6	42404.0	40241.9	34974.6	42836.1	43502.9	46356.4	#N/A	#N/A	#N/A	#N/A
Q09163	Protein delta homolog 1	Dlk1	385	41.3	5.91	52954.2	44318.1	53389.4	40746.0	63961.5	72800.4	85716.9	61928.6	53102.8	59276.2	#N/A	#N/A	#N/A	#N/A
Q99N20	Breast cancer metastasis-suppressor 1 homolog	Brms1	246	28.2	4.73	71348.9	76006.2	61171.2	61543.0	61622.2	62039.9	63794.8	68455.4	73465.3	67225.9	#N/A	#N/A	#N/A	#N/A
P35330	Intercellular adhesion molecule 2	Icam2	277	31.4	7.91	41982.3	38498.7	30243.4	29727.0	34911.2	35883.5	32262.6	29954.2	29825.5	41609.5	#N/A	#N/A	#N/A	#N/A
Q9CQ14	Tumor protein D52-like 3	Trpd52l3	231	24.6	4.97	208880.7	250224.7	235192.3	280556.8	196901.4	172248.1	176394.9	184437.1	242167.9	227829.4	124464.5	132959.0	121403.9	151176.4
D3YUE4	Family with sequence similarity 151, member B	Fam151b	279	31.4	6.11	78862.4	85199.6	78930.0	79287.8	84434.7	88409.8	78412.2	77800.2	86520.9	94146.5	106093.9	119350.4	109559.8	115266.2
C6KI89	Cation channel sperm-associated protein subunit gamma 2	Catsperg2	1145	131.3	7.06	139688.0	145725.4	141863.8	152088.3	116985.7	113350.4	125486.1	111367.8	128318.8	139984.9	#N/A	#N/A	#N/A	#N/A
Q3U284	Transmembrane protein 231	Tmem231	315	36.2	8.16	74839.0	75668.9	75661.8	87008.7	68171.9	73670.2	66677.8	74281.6	78906.4	73474.2	#N/A	#N/A	#N/A	#N/A
P58462	Forkhead box protein P1	Foxp1	705	78.8	6.67	159644.7	158729.8	163893.7	150385.4	201517.1	201057.1	204318.7	164003.0	161976.8	179753.8	96292.0	106947.1	102081.3	100424.4
Q8BWH0	Putative sodium-coupled neutral amino acid transporter 7	Slc38a7	463	49.9	5.29	127971.1	132977.9	117250.5	117041.4	126060.2	128918.0	116334.0	136426.2	121179.6	133871.2	#N/A	#N/A	#N/A	#N/A
P59024	Peptidyl-prolyl cis-trans isomerase FKBP14	Fkbp14	211	24.2	6.04	170761.2	151086.6	150800.9	161778.2	131324.8	135851.5	128873.1	134208.8	141453.6	157546.5	1296126.0	1184589.2	1150257.3	1207087.2
Q91VM4	Protein spinster homolog 2	Spns2	549	58.2	9.17	58043.7	50339.8	53821.9	51350.7	75276.3	75640.1	52303.5	56883.7	55517.9	63321.0	#N/A	#N/A	#N/A	#N/A
Q6P6M5	Peroxisomal membrane protein 11C	Pex11g	241	27.1	9.61	38477.1	53302.0	41766.5	52510.8	34189.2	35458.3	30693.6	40355.4	37497.9	39132.7	#N/A	#N/A	#N/A	#N/A
Q99PU7	Ubiquitin carboxyl-terminal hydrolase BAP1	Bap1	728	80.4	6.77	156102.6	125312.6	116424.0	120832.5	114826.3	119974.7	127303.2	124773.2	124756.4	116056.3	286924.6	262503.0	278372.4	294703.3
Q6PB60	Protein BHLHb9	Bhlhb9	539	59.8	8.7	89998.6	102646.7	85770.7	85586.5	85016.6	105289.6	77367.2	83594.9	91726.6	82929.3	#N/A	#N/A	#N/A	#N/A
Q3UZ01	RNA-binding region-containing protein 3	Rnpc3	514	57.9	7.9	80612.7	91216.5	88479.3	84887.2	93671.8	99135.5	73271.9	82754.6	89515.2	85803.4	#N/A	#N/A	#N/A	#N/A
E9Q8X5	Zinc finger and BTB domain-containing 10	Zbtb10	861	94	5.39	316795.7	303941.4	280931.0	301594.8	299533.3	319127.3	296344.5	277494.9	287369.4	301589.5	#N/A	#N/A	#N/A	#N/A
A0A140T8N9	Ig-like domain-containing protein	Igkv6-32	115	12.6	7.85	420565.2	256216.4	219645.0	247056.3	222756.7	242956.5	294340.2	375661.9	196874.1	208772.6	#N/A	#N/A	#N/A	#N/A
Q8BLY1	SPARC-related modular calcium-binding protein 1	Smoc1	463	51	8.37	206401.2	212236.7	186072.3	189181.3	202508.8	206050.5	207430.8	208133.2	188244.8	228723.3	#N/A	#N/A	#N/A	#N/A
Q9JJW0	Peroxisomal membrane protein 4	Pxmp4	212	24.2	9.83	1853570.5	1919223.9	1821057.8	2011999.7	1875021.1	1939386.1	1939956.5	1658562.7	1985328.5	1918409.4	233975.1	232907.0	207224.6	230418.1
A0A571BEW1	G_PROTEIN_RECEP_F1_2 domain-containing protein	Gm50457	323	36	8.4	482923.5	480617.3	419366.5	455351.5	405837.7	386550.8	448694.6	429140.6	439717.8	477542.3	606609.5	618813.5	666553.4	626035.6
Q9Z0U0	Xenotropic and polytropic retrovirus receptor 1	Xpr1	695	81.7	8.24	116783.8	142698.3	116889.1	141596.5	110467.8	122530.7	121919.2	108536.4	134526.8	128311.1	#N/A	#N/A	#N/A	#N/A
Q8BJ63	AP-5 complex subunit mu-1	Ap5m1	490	54.3	7.84	150776.0	166602.0	183083.7	157843.8	154578.8	173416.7	153451.7	153572.4	158073.8	175723.1	137383.1	160269.6	135066.3	137504.8
Q9CZP3	Zinc finger protein 655	Zfp655	541	63.3	6.81	163082.3	148941.3	148820.2	146121.4	152713.4	167896.2	168867.4	138855.8	172679.5	151011.6	#N/A	#N/A	#N/A	#N/A
Q8BL74	General transcription factor 3C polypeptide 2	Gtf3c2	907	100.2	6.57	778593.4	537220.0	707637.8	617400.1	705916.0	744957.2	832440.4	652424.4	678064.8	707153.4	56568.8	103125.5	51556.5	54624.5
Q9DB00	GON-4-like protein	Gon4l	2260	248.6	4.87	15406.7	20217.7	13492.4	17031.5	17047.3	20244.8	14540.7	17387.1	17841.7	17945.5	15408.3	12490.5	18912.7	15272.2
Q8BK30	NADH dehydrogenase [ubiquinone] flavoprotein 3, mitochondrial	Ndufv3	104	11.8	9.35	10895.7	12305.4	11306.0	16506.8	13597.1	6464.4	9998.9	13563.8	13402.0	10101.4	#N/A	#N/A	#N/A	#N/A
O09035	Diazepam-binding inhibitor-like 5	Dbil5	87	9.9	8.57	101372.9	121700.9	143012.2	160269.8	119475.3	108856.2	110728.9	99858.4	132397.5	120861.2	#N/A	#N/A	#N/A	#N/A

Q3US17	Zinc finger protein 48	Znf48	591	64.6	9.41	139249.5	151561.6	161071.2	140195.4	155328.4	163327.7	148741.4	158736.1	141272.8	142032.8	#N/A	#N/A	#N/A	#N/A
Q9EQY0	Serine/threonine-protein kinase/endoribonuclease IRE1	Ern1	977	110.1	6.51	166196.3	140482.5	166950.3	154361.5	169002.6	171119.3	136212.6	150584.2	153949.3	165619.0	#N/A	#N/A	#N/A	#N/A
Q9CQB7	LYR motif-containing protein 1	Lyrm1	122	14.2	9.6	116951.1	167213.2	152864.0	160297.7	145636.7	130635.4	122525.3	151536.3	158952.4	146965.8	59309.5	59003.9	56669.9	58043.5
Q67FY2	B-cell CLL/lymphoma 9-like protein	Bcl9l	1494	156.6	8.63	158337.9	178110.5	142746.4	157763.8	162229.7	173464.8	157670.5	151281.1	173975.2	165536.8	#N/A	#N/A	#N/A	#N/A
Q8K4R4	Cytoplasmic phosphatidylinositol transfer protein 1	Pitpnc1	332	38.4	6.32	212130.9	196825.7	195140.4	178774.7	204231.3	211423.3	249547.8	214144.7	214855.7	229099.0	#N/A	#N/A	#N/A	#N/A
Q8R0K2	E3 ubiquitin-protein ligase TRIM31	Trim31	507	57.1	7.85	72880.6	52853.2	70630.9	51332.1	53564.2	54620.2	59312.2	62361.4	44709.2	60508.5	#N/A	#N/A	#N/A	#N/A
O35491	Dual specificity protein kinase CLK2	Clk2	499	60	9.45	73906.4	86815.0	68258.9	75437.4	68702.6	70496.9	68475.7	66362.7	64442.5	73777.6	191491.2	224052.6	179450.0	185341.4
Q9CZ69	CKLF-like MARVEL transmembrane domain-containing protein 6	Cmtm6	183	19.8	6.29	243231.1	256125.7	256864.6	235482.0	253490.5	267460.9	225398.6	241654.5	252976.1	266073.3	472036.5	518222.5	523886.3	617876.9
Q8BWG9	Calcium release-activated calcium channel protein 1	Orai1	304	33	7.06	41560.2	35650.4	34714.2	37973.4	38647.9	44567.2	42809.6	40883.4	45614.0	36626.5	64105.1	67092.9	67848.4	64125.9
Q9QZ29	Immunoglobulin-binding protein 1b	Igbp1b	343	39.2	4.98	40063.5	34667.0	38328.8	46150.7	38367.9	32341.1	37251.2	38324.9	46108.3	37702.1	17904.9	18368.2	18614.3	22915.4
Q00558	40-kDa huntingtin-associated protein	F8a1	380	40.5	6.89	119955.1	104163.2	113878.5	108615.0	130255.6	111592.0	114767.4	111467.4	100700.5	121504.4	#N/A	#N/A	#N/A	#N/A
Q9CXU1	Mediator of RNA polymerase II transcription subunit 31	Med31	131	15.8	8.54	153505.6	155347.7	175666.1	167619.1	157757.2	152905.3	154377.9	149414.4	163244.5	165521.5	#N/A	#N/A	#N/A	#N/A
Q9QXX8	Nuclear fragile X mental retardation-interacting protein 1	Nufip1	484	54.7	9.47	334380.8	146315.1	125992.5	139443.7	137909.8	165490.0	139547.4	124983.1	143750.6	147641.1	#N/A	#N/A	#N/A	#N/A
Q8BKR5	Protein phosphatase 1 regulatory subunit 37	Ppp1r37	712	77.5	5.06	108561.9	100699.4	110252.9	105002.7	106282.9	113000.0	94759.8	97991.4	107098.8	101772.8	#N/A	#N/A	#N/A	#N/A
Q5RJH6	Protein SMG7	Smg7	1138	126.8	7.99	278137.3	266002.9	272235.1	247545.5	267998.9	288435.9	300982.1	263251.4	263072.2	276695.8	277318.0	358550.9	334328.2	338686.8
Q07968	Coagulation factor XIII B chain	F13b	669	76.1	6.92	25449.6	28816.7	24441.3	29560.2	24867.9	34230.3	23208.1	28179.1	28230.2	26006.4	#N/A	#N/A	#N/A	#N/A
Q8R0J8	Probable gluconokinase	Idnk	184	20	6.33	1570.9	4134.4	3260.4	3097.2	4004.2	2779.2	3146.1	3163.1	3698.8	3962.8	#N/A	#N/A	#N/A	#N/A
Q91W43	Glycine dehydrogenase (decarboxylating), mitochondrial	Gldc	1025	113.2	7.56	5013.0	5100.4	3597.0	9172.5	8883.6	11860.1	5606.9	9171.7	6020.2	5654.4	2831.6	8125.6	3707.3	12209.8
Q810P3	Cyclin-dependent kinase inhibitor 3	Cdkn3	211	23.8	6.38	82176.2	94951.7	82851.5	94784.4	83225.7	95080.3	100377.1	89504.5	106142.3	102716.6	#N/A	#N/A	#N/A	#N/A
A2A8R0	Zinc finger FYVE domain-containing protein	Zfyve9	1397	152.3	5	171864.4	149525.6	155569.5	181901.4	195355.9	257750.5	178427.9	175434.0	174242.5	186492.8	125602.1	133777.8	138937.0	140573.2
Q9R098	Hepatocyte growth factor activator	Hgfac	653	70.5	7.03	181350.8	191811.0	176167.9	177332.6	184196.3	195867.3	185977.0	178765.3	173665.1	162089.1	309995.4	305336.2	267718.0	279853.8
O70333	Cysteine-rich PDZ-binding protein	Cript	101	11.3	9.57	349366.9	386716.3	344524.4	385898.5	284769.9	346647.4	348089.1	293482.4	382572.9	405439.6	171559.3	200817.5	160771.5	188569.5
Q9CZV5	STAGA complex 65 subunit gamma	Supt7l	412	45.9	5.07	326656.5	400215.7	347330.3	376933.0	379885.3	355366.1	311777.8	329353.2	368090.9	401829.3	#N/A	#N/A	#N/A	#N/A
Q9Z2X8	Kelch-like ECH-associated protein 1	Keap1	624	69.5	6.44	394373.0	369085.8	335214.3	374512.7	337299.9	350723.8	368741.2	368992.3	389919.7	346680.6	#N/A	#N/A	#N/A	#N/A
Q4V9W2	Protein SREK1IP1	Srek1ip1	153	18.1	9.91	168255.8	162735.1	152303.0	156004.8	159600.7	168434.0	157549.5	132722.5	162896.1	168069.1	#N/A	#N/A	#N/A	#N/A
Q9D7E3	Esterase OVCA2	Ovca2	225	24.2	5.88	24406.9	30348.9	32624.6	34708.3	33431.9	29150.5	28802.7	28131.5	38064.3	37377.0	#N/A	#N/A	#N/A	#N/A
Q8BTS3	GB1/RHD3-type G domain-containing protein	Gbp9	619	71.3	5.97	112696.1	122130.4	97455.2	106124.6	126830.8	133365.3	172373.6	124890.5	110871.0	117679.5	#N/A	#N/A	#N/A	#N/A
Q61234	Alpha-1-syntrophin	Snta1	503	53.6	6.86	76010.5	78574.5	60764.9	72875.2	63936.3	79020.6	71424.8	71535.8	70817.6	74321.6	60234.4	57737.7	58102.5	58858.2
Q9D2R8	28S ribosomal protein S33, mitochondrial	Mrps33	106	12.5	10.26	116398.9	115432.8	89515.5	105481.8	99704.2	100524.8	105397.4	92688.6	105526.1	109137.5	#N/A	#N/A	#N/A	#N/A
Q9DA69	Intraflagellar transport protein 43 homolog	Ift43	206	23.5	4.69	28425.6	34967.4	35209.5	37603.6	25398.6	24750.4	23637.4	25663.5	27339.9	32117.0	20607.8	25979.8	28807.1	31946.2
Q9CXV1	Succinate dehydrogenase [ubiquinone] cytochrome b small subunit, mitochondrial	Sdhd	159	17	9.1	1681607.0	1719114.3	1794644.1	1912384.5	1758665.0	1574517.5	1578958.5	1703622.0	1649616.6	1663696.5	1542845.9	1742439.9	1570568.3	1879644.8
Q9CR84	ATP synthase F(0) complex subunit C1, mitochondrial	Atp5mc1	136	14.2	10.01	602745.9	562609.1	521922.6	578102.0	565600.5	529845.3	523873.1	530519.0	533730.4	519025.9	645407.8	854863.9	642664.2	862369.2
Q924H2	Mediator of RNA polymerase II transcription subunit 15	Med15	789	86.6	9.29	113790.8	111295.6	117656.8	97333.7	115413.8	109202.0	105094.2	102798.0	101556.2	106883.5	#N/A	#N/A	#N/A	#N/A
Q9DCK3	Tetraspanin-4	Tspan4	238	26	5.48	200980.1	180187.5	202631.5	175765.9	258631.5	275518.3	275321.3	205457.0	198114.8	190098.2	143681.5	148944.9	170031.0	150146.3
A2AFS3	UPF0577 protein KIAA1324	Kiaa1324	1009	110.6	6.38	49407.4	57707.8	61085.0	87859.8	42992.9	39418.3	41396.0	52928.2	53214.9	53499.7	58704.5	67235.4	77443.3	98559.9
Q61468	Mesothelin	Msln	625	69.4	7.59	188647.5	218758.2	151325.1	190088.9	198317.4	169680.9	163499.9	145139.9	185997.6	199691.5	#N/A	#N/A	#N/A	#N/A
Q80TS3	Adhesion G protein-coupled receptor L3	Adgrl3	1537	171	6.71	32869.9	32645.3	28423.0	33674.3	39316.3	40632.9	37619.2	35581.5	32389.4	32945.3	#N/A	#N/A	#N/A	#N/A
Q9R0N7	Synaptotagmin-7	Syt7	403	45.4	9.28	66648.9	65798.3	55179.6	61232.7	63622.7	59642.7	54776.8	54712.8	52205.2	60546.7	56881.7	67312.0	51632.6	60061.9
Q8R5F7	Interferon-induced helicase C domain-containing protein 1	Ifih1	1025	115.9	6.25	162816.9	149548.4	137794.1	130787.7	157396.9	166156.8	158039.3	155126.2	156536.9	160410.2	288907.5	265241.5	242074.0	277271.1
E9Q8N6	AKNA domain-containing 1	Aknad1	675	75.4	6.38	151406.8	170522.8	153709.4	155822.7	165283.9	160383.1	152885.8	150977.8	161666.8	179815.0	#N/A	#N/A	#N/A	#N/A
A0A5F8MPP0	Pleckstrin homology domain-containing, family G (with RhoGef domain) member 1	Plekhg1	1465	164	6.55	63457.6	52889.0	60943.5	61968.7	54084.5	61280.7	59160.0	52714.1	57976.4	59151.9	65935.4	54611.0	55560.6	57879.7
Q11204	CMP-N-acetylneuraminate-beta-galactosamide-alpha-2,3-sialyltransferase 2	St3gal2	350	40.1	8.5	50959.9	47936.3	51191.1	52940.9	55664.6	52491.8	55807.0	44391.4	51937.6	49422.1	#N/A	#N/A	#N/A	#N/A
Q8VDY4	EF-hand calcium-binding domain-containing protein 7	Efcab7	628	71.4	6.09	87008.1	98442.0	97647.9	110375.1	88178.8	93122.7	94596.3	69605.7	84894.4	101165.0	#N/A	#N/A	#N/A	#N/A
Q00262	Syntaxin-2	Stx2	289	33.2	6.39	139322.2	172326.7	171335.5	169064.1	167515.5	166229.7	180202.1	140735.3	177223.1	187230.5	208605.3	221217.6	230299.0	247292.2
Q9D6V8	Polyadenylate-binding protein-interacting protein 2	Paip2	124	14.7	4.12	456825.0	447570.4	433683.5	471715.2	512423.7	482104.2	419667.4	468550.9	477413.9	495046.3	#N/A	#N/A	#N/A	#N/A
Q922S4	cGMP-dependent 3',5'-cyclic phosphodiesterase	Pde2a	939	105.6	5.41	80097.5	86844.4	83408.2	79361.6	104651.3	95976.4	98061.6	80873.4	83239.5	91919.8	76008.1	70205.2	78874.5	69014.7
Q61469	Phospholipid phosphatase 1	Plpp1	283	31.9	7.02	47277.0	51016.8	45925.9	63570.8	48262.0	50712.0	57151.1	51224.6	45338.1	48593.8	#N/A	#N/A	#N/A	#N/A
A2ACJ2	Fanconi anemia core complex-associated protein 100	Faap100	879	94.2	5.36	115376.0	149088.9	121418.5	146266.2	125034.5	143761.3	126858.8	134625.9	133810.6	135762.6	#N/A	#N/A	#N/A	#N/A
Q61585	G0/G1 switch protein 2	G0s2	103	11.1	8.32	671558.4	651819.0	647423.5	661078.1	784358.6	709158.8	712584.5	692968.2	645811.5	576796.2	#N/A	#N/A	#N/A	#N/A
Q8CEG8	Ubiquitin carboxyl-terminal hydrolase 27	Usp27	438	49.6	7.14	334512.4	334875.7	312643.4	311908.0	310927.2	311184.6	337932.8	335188.7	311388.2	335033.1	#N/A	#N/A	#N/A	#N/A
Q5U5M8	Biogenesis of lysosome-related organelles complex 1 subunit 3	Bloc1s3	195	20.4	4.92	107797.4	102351.5	101420.6	105577.8	98186.4	98688.4	104092.6	95398.4	99547.8	113148.5	#N/A	#N/A	#N/A	#N/A
Q9CX11	rRNA-processing protein UTP23 homolog	Utp23	249	28.4	10.32	296496.6	290008.9	303328.9	258505.1	363445.5	323578.1	264999.3	304602.4	301015.6	290720.5	227305.7	309719.8	249657.2	293772.3
Q9CQP0	39S ribosomal protein L33, mitochondrial	Mrpl33	65	7.4	10.89	1129438.5	1339672.5	1154073.0	1298618.3	1078808.6	1124268.0	1143206.6	993811.4	1206116.3	1183938.2	309941.7	330871.7	336013.3	355076.2
Q80ZM7	Transcription initiation factor IIA subunit 2	Gtf2a2	109	12.5	6.62	656326.9	695799.1	739202.0	815250.0	702181.0	634970.0	609483.1	679903.1	747847.4	681839.0	#N/A	#N/A	#N/A	#N/A
A0A2R8VJU7	Septin 12	Septin12	357	40.8	7.84	108501.3	128528.4	118176.1	134741.5	119902.1	124457.4	98980.6	105369.5	106686.1	107069.0	#N/A	#N/A	#N/A	#N/A
Q8C4P0	Uncharacterized protein KIAA1958 homolog	MGI:244216	716	79.1	6.93	33706.1	34289.6	32998.6	33832.6	28708.3	31138.5	34852.1	27134.4	38456.2	28486.9	#N/A	#N/A	#N/A	#N/A
O08538	Angiopoietin-1	Angpt1	498	57.5	6.76	102237.5	98726.1	101196.6	99624.3	118484.5	123257.1	131736.3	107407.6	134710.1	103138.0	#N/A	#N/A	#N/A	#N/A
Q8VIF2	Serine protease 42	Prss42	335	36.7	8.48	149703.4	182634.8	183502.6	236952.5	143551.5	134883.8	136356.4	137017.0	156385.4	157917.7	#N/A	#N/A	#N/A	#N/A
Q9BCZ4	Selenoprotein S	Selenos	190	21.5	9.39	37140.8	41229.7	37856.0	33294.8	38137.7	39280.3	33937.2	39901.5	42381.9	36534.0	47079.8	54184.0	58550.6	54411.7
O70477	Homeobox protein PKNOX1	Pknox1	436	47.5	4.91	99405.4	94316.6	90712.0	85657.2	83588.7	103727.4	103809.4	90508.8	90509.2	84518.9	#N/A	#N/A	#N/A	#N/A
Q80X45	Protein kish-B	Tmem167b	74	8.3	9.61	522477.9	554216.1	547918.7	618290.9	487411.8	457679.3	476100.4	490130.5	527418.7	506704.9	374557.1	410806.5	390922.6	436266.3
Q9CPY6	Glucose-induced degradation protein 4 homolog	Gid4	217	24.8	6.52	13299.3	18027.1	14729.9	13854.6	14714.5	15969.8	15805.6	12303.5	15544.3	12398.5	30615.3	34905.1	29651.4	31344.8
Q8R4F0	Mucolipin-3	Mcoln3	553	63.7	6.8	407052.0	445499.0	476015.2	424576.9	436534.7	400238.9	397502.8	397583.6	449263.1	459124.8	454690.8	461956.7	474557.7	464766.1
Q09014	Neutrophil cytosol factor 1	Ncf1	390	44.6	9.01	69718.9	96845.4	64134.8	86868.0	78772.8	91002.3	66333.5	106944.9	76077.1	82947.6	#N/A	#N/A	#N/A	#N/A
Q8K1J5	Replication stress response regulator SDE2	Sde2	448	48.6	5.66	368928.6	414260.6	346332.8	346605.2	368587.7	380934.8	400382.5	386242.6	389263.6	388607.2	150891.8	156972.3	151747.8	159425.9
O55186	CD59A glycoprotein	Cd59a	123	13.6	7.47	26168.0	19423.4	23109.2	19382.3	32245.4	32864.0	44165.4	23710.0	22551.5	21789.9	25007.4	21268.5	24813.5	18901.4
A0A494BB10	Uncharacterized protein	Gm50478	99	11.1	9.23	460895.4	552486.2	534287.6	495566.6	558453.0	598513.8	510734.5	459843.0	580641.1	573140.4	#N/A	#N/A	#N/A	#N/A
Q80WS1	Beta-citrylglutamate synthase B	Rimklb	387	42.5	6	100678.8	119568.3	98542.4	101887.4	120019.9	119036.9	104532.2	97544.2	98861.3	112194.5	#N/A	#N/A	#N/A	#N/A
P15089	Mast cell carboxypeptidase A	Cpa3	417	48.8	9	140046.3	147009.8	144288.9	141785.3	137590.8	154948.6	162892.9	184790.2	132684.5	135475.6	#N/A	#N/A	#N/A	#N/A
Q99PL7	Acyl-CoA desaturase 3	Scd3	359	41.4	8.94	673694.8	556674.6	510662.3	592377.1	527695.7	455658.6	526155.9	560703.8	484826.4	536601.0	#N/A	#N/A	#N/A	#N/A
Q80YR6	DNA endonuclease RBBP8	Rbbp8	893	100.8	6.68	156197.6	169335.7	160929.5	163281.7	166971.1	159430.5	148184.8	138533.7	159106.4	175834.0	#N/A	#N/A	#N/A	#N/A
Q9D5S1	Testis-expressed protein 19.2	Tex19.2	317	35.2	4.82	1305485.5	1462032.0	1445338.1	1483529.3	1540683.1	1632389.4	1497305.8	1334321.6	1467350.1	1426092.1	95464.0	90747.2	83324.1	94074.8
Q8CI12	Smoothelin-like protein 2	Smtnl2	456	49.5	8.54	46518.5	45178.9	43306.2	37420.5	48233.0	66274.4	51600.5	42492.6	36098.9	38187.7	#N/A	#N/A	#N/A	#N/A
E9Q9F7	Coiled-coil domain-containing 146	Ccdc146	977	115	6.84	457364.4	416735.6	451024.9	446225.7	366390.6	396058.2	377117.3	391221.6	424927.9	443836.0	#N/A	#N/A	#N/A	#N/A
Q9CYF5	RCC1-like G exchanging factor-like protein	Rcc1l	461	50	8.29	172764.8	210127.1	162741.6	181548.1	176414.1	177785.2	178126.6	174675.5	185546.0	196040.7	#N/A	#N/A	#N/A	#N/A
Q62481	Vacuolar protein sorting-associated protein 72 homolog	Vps72	368	40.8	6.4	461940.5	508195.5	462336.6	492875.9	503689.8	509682.7	500413.1	504242.4	492140.6	490806.3	177725.3	169785.3	192675.2	155634.1
Q6IQY5	Centrosomal protein of 70 kDa	Cep70	616	71	6.11	78191.5	104818.4	76244.6	68672.9	87337.8	71908.5	60083.3	66990.0	73723.4	75911.2	136332.7	147043.6	130766.1	144650.3
Q80TL0	Protein phosphatase 1E	Ppm1e	749	83.4	4.97	23683.5	26157.3	22135.2	25232.9	18572.0	22432.5	24728.6	20473.8	24669.8	23028.7	33544.5	32750.3	29030.9	32400.3
Q5DTU0	Actin filament-associated protein 1-like 2	Afap1l2	825	92.1	5.44	491560.0	395721.9	290932.0	366018.3	315416.1	341938.6	292448.7	312718.4	342337.1	360916.7	259523.7	234267.2	153546.7	208194.5
Q5PRE5	Proline and serine-rich protein 1	Proser1	913	91.9	9.11	17571.7	16534.2	16706.2	14367.3	19478.1	17035.3	13207.5	17416.4	14180.9	15590.8	#N/A	#N/A	#N/A	#N/A
Q8CD91	SPARC-related modular calcium-binding protein 2	Smoc2	447	49.9	8.51	172336.6	161730.0	168334.4	150507.9	138131.7	169333.2	186302.0	197229.4	199266.6	157527.6	235382.8	216934.3	235287.1	219466.1
Q5SS00	DBF4-type zinc finger-containing protein 2 homolog	Zdbf2	2493	273.6	5.22	16157.7	19664.3	18905.7	24000.4	23892.2	20848.0	18753.6	21435.5	19192.2	17696.1	#N/A	#N/A	#N/A	#N/A
Q9CQB2	MAPK regulated corepressor interacting protein 2	Mcrip2	160	17.9	7.3	81470.3	84080.0	73146.3	79660.0	75584.5	77147.2	69735.6	74825.1	73186.4	77475.2	#N/A	#N/A	#N/A	#N/A
Q9JHD2	Histone acetyltransferase KAT2A	Kat2a	830	93.3	9.07	9875.9	9197.9	9920.7	10796.3	10611.4	11542.2	8289.8	9680.3	13639.2	12477.7	962585.2	769199.5	1146142.8	862818.6
Q0VG62	Ribosomal biogenesis factor	Rbis	97	11.2	10.49	553514.0	601335.1	572319.2	562980.9	516755.1	466485.0	404643.4	518603.0	547550.2	600638.1	#N/A	#N/A	#N/A	#N/A
B2RRD7	Peregrin	Brpf1	1212	137.2	7.93	136139.8	148536.0	151785.8	139645.7	165064.4	188519.2	147936.8	172936.7	151957.5	137252.8	86655.4	71927.5	77703.4	74375.1
Q99J72	DNA dC->dU-editing enzyme APOBEC-3	Apobec3	440	52.2	9.09	190769.2	200404.2	189528.9	184251.6	174006.7	180370.3	199499.5	181644.0	174491.5	175366.2	#N/A	#N/A	#N/A	#N/A
Q07797	Galectin-3-binding protein	Lgals3bp	577	64.5	5.14	12361.0	14937.4	13781.6	16161.9	15991.0	18392.0	16347.7	14667.5	14298.5	13290.6	44181.5	40801.3	38480.7	41733.6
Q14DH7	Acyl-CoA synthetase short-chain family member 3, mitochondrial	Acss3	682	74.5	8.63	197129.2	188211.6	182791.1	200475.2	187298.3	181120.4	233538.3	221379.1	181921.9	156726.0	#N/A	#N/A	#N/A	#N/A
D3YZG8	Probable bifunctional methylenetetrahydrofolate dehydrogenase/cyclohydrolase 2	Mthfd2l	338	36.4	9.39	135280.1	145932.0	149831.7	158713.1	139076.6	142742.1	117066.9	141795.1	139123.5	142356.9	457941.3	428273.2	380340.6	418908.9
Q9JL26	Formin-like protein 1	Fmnl1	1094	122	5.82	9646.6	8183.8	7951.1	7065.4	27100.0	12072.1	9356.1	8927.3	12284.5	7703.8	#N/A	#N/A	#N/A	#N/A
Q5HZK1	Centrosomal protein of 44 kDa	Cep44	386	43.2	5.5	111544.9	117957.7	106715.2	107909.7	118578.5	132478.6	106558.9	107085.4	115299.5	114540.6	#N/A	#N/A	#N/A	#N/A

Q91VP7	Transmembrane protein 101	Tmem101	257	28.8	9.57	87232.9	89663.2	88270.7	95505.6	87932.9	97724.9	84946.7	81370.8	85981.6	92971.0	819157.7	817069.3	761627.3	799203.9
Q9CV28	Ubiquitin carboxyl-terminal hydrolase MINDY-3	MINDY3	444	49.6	4.78	194668.8	201262.0	211310.8	223299.6	187948.1	186705.8	159175.0	165265.5	206814.7	206728.5	92247.5	87863.7	108654.0	98117.7
Q80WQ8	Mis18-binding protein 1	Mis18bp1	998	113.9	9.09	144683.2	174052.0	170137.5	162950.7	186597.4	180690.9	188354.6	164081.1	180754.1	191443.7	#N/A	#N/A	#N/A	#N/A
Q3V129	Serine/threonine-protein kinase ULK4	Ulk4	1303	145.3	6.27	170152.7	189045.4	167168.2	165329.9	155931.9	159973.0	144952.9	175445.9	178640.0	183883.4	#N/A	#N/A	#N/A	#N/A
Q9CQG3	Zinc finger MYND domain-containing protein 19	Zmynd19	227	26.4	6.86	152318.5	132391.2	162537.6	149181.9	181162.8	180083.4	203928.5	170415.0	163674.4	150153.2	43870.0	52651.5	53034.5	50323.1
Q0VFX2	Cilia- and flagella-associated protein 157	Cfap157	523	61	9.07	422816.9	433516.7	437181.7	439891.0	435860.3	425554.0	395575.4	422730.6	418295.2	421565.2	#N/A	#N/A	#N/A	#N/A
Q91X58	AN1-type zinc finger protein 2B	Zfand2b	257	27.9	6.93	216903.6	192910.9	187559.0	195747.4	189373.8	200901.7	193910.2	186195.7	184806.5	194305.3	#N/A	#N/A	#N/A	#N/A
J3QNX5	Protein shisa-8	Shisa8	399	42.4	9.96	233366.7	221623.8	219827.6	203238.9	279725.1	291730.5	254607.9	240158.9	248635.5	267947.1	#N/A	#N/A	#N/A	#N/A
Q9JK39	Butyrophilin-like protein 10	Btnl10	275	29.5	7.28	1256643.4	940775.1	1082010.0	907141.3	974031.2	886358.6	960337.8	1121828.6	1023246.4	1025455.7	636645.2	506383.6	570876.1	500972.1
P58269	Zinc finger protein DPF3	Dpf3	378	43	6.42	12308.1	10675.3	10688.3	9934.6	12993.6	15602.6	16854.9	13272.5	10066.0	12315.0	#N/A	#N/A	#N/A	#N/A
Q7TS74	Cytoskeleton-associated protein 2-like	Ckap2l	745	82.9	9.69	55267.8	59264.7	56535.4	61797.4	59574.8	58724.8	54395.6	59457.9	50297.7	53500.5	25376.8	31500.7	29190.2	29227.3
Q3V2Q8	NEDD4-binding protein 2-like 1	N4bp2l1	238	28	9.94	24938.5	25832.3	20647.0	19461.6	28614.0	23749.9	26903.5	21400.6	27816.5	25433.5	#N/A	#N/A	#N/A	#N/A
P0DP43	Cation channel sperm-associated protein subunit epsilon	Catspere	985	113.7	6.81	52832.1	66617.8	66121.2	56356.7	69470.5	66546.8	57502.7	63984.3	61979.2	69620.9	#N/A	#N/A	#N/A	#N/A
Q9JHH6	Carboxypeptidase B2	Cpb2	422	48.8	7.97	968441.0	502107.2	493912.7	500255.7	491023.4	528684.0	494478.3	634346.0	458705.4	478101.1	274599.8	342608.2	242195.4	267816.2
O54784	Death-associated protein kinase 3	Dapk3	448	51.4	8.7	76750.1	113920.2	75974.5	84493.1	87617.4	120511.3	80743.3	93597.4	90524.2	90238.7	#N/A	#N/A	#N/A	#N/A
Q5DID3	Uromodulin-like 1	Umodl1	1319	145.2	6.67	62274.8	69470.3	56726.5	75710.2	67743.1	69928.4	61404.7	61424.0	66813.0	68095.4	#N/A	#N/A	#N/A	#N/A
Q61124	Battenin	Cln3	438	47.6	5.44	8215.7	6544.6	6348.4	9450.9	6244.4	6300.0	9477.5	9445.6	6398.3	7429.2	#N/A	#N/A	#N/A	#N/A
P43346	Deoxycytidine kinase	Dck	260	30.3	5.43	214238.6	211563.7	228612.0	200448.2	243057.7	233339.0	224954.9	216258.5	223615.0	228840.6	#N/A	#N/A	#N/A	#N/A
Q9D361	U11/U12 small nuclear ribonucleoprotein 48 kDa protein	Snrnp48	337	39.4	8.65	77012.0	82189.5	96486.0	86162.6	96423.6	90170.1	85943.0	77441.9	85126.0	89454.8	#N/A	#N/A	#N/A	#N/A
Q8VFZ5	Olfactory receptor	Olfr516	314	35.6	8.57	95216.8	116913.2	98359.8	117629.0	115541.8	111748.4	96462.4	96891.7	123456.5	112248.9	135511.1	167116.4	135181.0	149870.7
Q8CAK3	Shiftless antiviral inhibitor of ribosomal frameshifting protein homolog	Shfl	290	33	7.28	200311.4	225602.7	219647.0	220125.2	260745.3	272992.0	313389.0	228953.3	220384.1	220750.1	99803.0	104516.9	90959.8	96974.0
Q91XM9	Disks large homolog 2	Dlg2	852	94.8	6.24	91897.1	113788.7	103633.6	106531.1	114485.0	122540.4	116473.4	104240.5	101873.0	106899.4	48774.8	40965.9	33519.0	45107.6
P22561	Wilms tumor protein homolog	Wt1	449	49.2	9.07	255635.5	264528.4	237982.5	200217.0	261452.6	277393.6	283378.6	234850.1	247071.7	250922.1	#N/A	#N/A	#N/A	#N/A
A0A087WPV9	RIKEN cDNA 1700019A02 gene	00019A02I	146	16.9	5.01	15465.6	15837.0	16873.7	15634.0	12332.4	9494.5	8968.9	11811.5	15233.3	17554.9	175871.5	191004.9	179591.7	204704.1
Q8CFJ9	GATOR complex protein WDR24	Wdr24	790	88.1	6.46	60240.3	73137.3	61286.9	66814.2	66557.3	76127.0	63770.8	62526.9	62145.1	70209.1	93045.6	113346.1	83091.5	77106.9
Q9WUJ8	Origin recognition complex subunit 6	Orc6	262	29.2	9.03	84179.3	80646.9	70003.1	80928.5	85759.4	94068.8	76004.9	81581.4	95169.2	81945.7	152912.3	174410.7	169320.5	165148.5
Q6PFE3	DNA repair and recombination protein RAD54B	Rad54b	886	99.3	8.22	57216.9	66564.4	52452.8	55228.6	59910.4	54500.7	45398.7	87636.7	61432.1	67021.1	119890.3	131627.6	129094.0	125806.7
Q9D0B5	Thiosulfate sulfurtransferase/rhodanese-like domain-containing protein 3	Tstd3	157	17.3	8.13	2876.1	9367.2	6490.8	5012.5	7229.4	8257.5	4629.5	8947.7	7745.6	5331.5	#N/A	#N/A	#N/A	#N/A
Q8R0I0	Angiotensin-converting enzyme 2	Ace2	805	92.3	5.54	189603.3	148977.9	138207.1	161190.6	159206.5	131731.0	151599.9	127339.4	152888.9	162799.8	139101.6	145084.5	127287.5	150021.5
Q9JLJ0	Lipopolysaccharide-induced tumor necrosis factor-alpha factor homolog	Litaf	161	16.9	5.88	1405092.9	1272148.5	1489021.2	1219535.9	1721920.8	1753922.8	2088656.9	1536478.4	1534679.6	1488191.7	688581.7	715319.6	790787.8	623713.8
Q8BHD8	Protein-L-isoaspartate O-methyltransferase domain-containing protein 2	Pcmtd2	359	40.7	6.46	342673.6	344490.7	324053.8	326381.0	397079.7	381269.6	360166.7	316789.2	331736.5	353126.6	27557.5	26005.5	20659.7	24128.7
F6VAN0	Cyclic AMP-dependent transcription factor ATF-6 alpha	Atf6	656	72.6	7.77	145305.7	131235.8	134202.2	138315.5	165759.7	151214.6	128804.5	141589.1	146356.8	144318.2	#N/A	#N/A	#N/A	#N/A
Q8BVR6	RING finger and SPRY domain-containing protein 1	Rspry1	576	64.3	5.5	147191.8	132747.9	145151.6	131723.0	142381.8	151869.8	140841.1	150862.2	149496.6	134905.5	91850.9	81894.6	73747.5	81929.8
Q9CZX5	PIN2/TERF1-interacting telomerase inhibitor 1	Pinx1	332	37.2	9.63	232777.1	252815.3	231263.7	225351.8	242911.5	256965.9	219394.1	240186.6	235564.1	256126.9	#N/A	#N/A	#N/A	#N/A
Q8BGB5	LIM domain-containing protein 2	Limd2	128	14.2	9.04	91978.0	118763.3	111708.0	119843.8	83308.4	84509.5	75578.8	88397.5	99881.3	98916.2	#N/A	#N/A	#N/A	#N/A
P55200	Histone-lysine N-methyltransferase 2A	Kmt2a	3966	429.4	9.16	137420.1	133888.3	121357.0	114840.4	128181.6	145639.1	131076.6	112872.9	126013.8	142546.8	59782.6	54717.9	57230.7	53521.5
O88551	Claudin-1	Cldn1	211	22.9	7.91	36008.1	29073.1	36304.0	34931.7	39850.9	51627.6	47384.2	38178.4	37053.0	39187.3	20017.7	25818.2	29462.8	24099.2
Q9DBX7	Hairy/enhancer-of-split related with YRPW motif-like protein	Heyl	326	34.9	9.98	201253.1	165057.5	131555.5	136948.7	95788.8	116282.2	150176.7	183708.4	126322.2	139068.2	241131.5	207736.0	172097.5	157932.3
P00184	Cytochrome P450 1A1	Cyp1a1	524	59.2	8.06	102463.0	145004.0	174522.3	185657.8	106585.3	100676.2	88349.9	104702.7	138173.7	122455.4	95560.5	148846.5	144537.0	147259.2
Q9Z2E2	Methyl-CpG-binding domain protein 1	Mbd1	636	70	8.66	75723.0	158453.2	81312.5	104773.8	84585.0	136717.2	64813.3	111214.4	73252.8	108787.1	206627.5	228616.1	205784.1	204450.2
Q922K9	Tyrosine-protein kinase FRK	Frk	512	58.8	5.99	268425.9	250107.3	246926.6	214525.3	257969.4	275200.8	245977.3	247404.3	241056.2	239995.5	#N/A	#N/A	#N/A	#N/A
Q9EQS9	Immunoglobulin superfamily DCC subclass member 4	Igdcc4	1252	134.7	6.18	35795.8	28277.4	29635.9	29802.9	40136.0	34973.3	33636.8	39693.5	30088.9	31491.9	#N/A	#N/A	#N/A	#N/A
Q60994	Adiponectin	Adipoq	247	26.8	5.57	196065.5	168991.0	150061.0	182844.4	179875.1	187133.5	262255.5	193171.6	159125.8	169089.5	58669.6	64719.5	65005.1	54270.0
Q69ZZ9	Uncharacterized protein KIAA0754	Kiaa0754	979	104.6	4.28	19307.0	16627.3	17405.4	19602.7	17447.9	15636.9	14583.5	17467.0	16219.8	16862.2	#N/A	#N/A	#N/A	#N/A
P52734	FYVE, RhoGEF and PH domain-containing protein 1	Fgd1	960	106.3	6.58	120015.4	148633.6	136016.3	134154.0	129180.7	153124.7	123047.2	129640.2	125365.7	136468.4	#N/A	#N/A	#N/A	#N/A
Q6NSQ7	Protein LTV1 homolog	Ltv1	470	54	4.92	12184.6	12497.5	13943.9	12401.0	11213.8	12814.2	8335.0	11876.2	15305.5	13213.5	50004.6	47010.2	64599.4	49631.1
Q8K0D2	Hyaluronan-binding protein 2	Habp2	558	62.3	6.37	169599.6	174814.7	153030.0	166547.2	148495.9	181646.9	196375.5	163795.7	172586.1	161545.5	314168.1	319798.2	309422.1	296718.6
Q8VCE4	Uncharacterized protein C9orf40 homolog	/IGI:358396	163	18	4.75	476824.8	294303.6	289608.5	260534.0	254217.7	268431.2	237379.3	227262.2	259872.4	281495.9	341199.2	305947.3	309713.0	311228.0
Q6PD10	Inositol hexakisphosphate kinase 1	Ip6k1	433	49.3	7.23	309529.4	322315.0	269931.5	298143.3	306710.4	307429.3	315906.9	307160.5	273700.1	294789.8	#N/A	#N/A	#N/A	#N/A
P19785	Estrogen receptor	Esr1	599	66.9	8.06	39629.7	44647.1	33684.9	37294.1	40392.5	40157.9	48232.1	44814.4	29201.3	38656.1	#N/A	#N/A	#N/A	#N/A
A8Y5H7	SEC14-like protein 1	Sec14l1	715	81.2	6.34	36745.8	31881.6	37047.7	34996.4	34090.6	39817.0	34255.3	32930.0	38559.7	34966.8	#N/A	#N/A	#N/A	#N/A
Q5XFZ0	UPF0711 protein C18orf21 homolog	/IGI:191529	217	24.1	10.52	217168.1	215125.2	149431.1	195291.7	198343.3	204191.0	191293.7	192216.6	182941.5	209202.3	#N/A	#N/A	#N/A	#N/A
Q60841	Reelin	Reln	3461	387.2	5.73	420889.7	454631.9	413449.7	418934.1	445843.1	456090.8	389749.7	394155.1	441003.4	449854.8	370875.9	424296.9	411918.2	383498.0
Q99N92	39S ribosomal protein L27, mitochondrial	Mrpl27	148	15.9	10.17	66496.9	74211.8	67777.4	64902.0	61754.5	71349.0	59483.7	61872.5	67229.2	64763.7	#N/A	#N/A	#N/A	#N/A
Q52KG4	Zinc finger and BTB domain-containing protein 45	Zbtb45	520	55	7.01	173780.4	151238.7	146433.2	147527.5	132797.6	142653.4	163010.8	163382.5	126619.5	148847.1	#N/A	#N/A	#N/A	#N/A
Q8CG16	Complement C1r-A subcomponent	C1ra	707	80	5.66	104196.1	115182.3	94325.5	114293.1	113689.6	125439.7	137970.7	112336.9	104774.6	114886.7	#N/A	#N/A	#N/A	#N/A
Q920Q2	DNA repair protein REV1	Rev1	1249	137.3	8.54	1549205.3	1568392.2	1681645.2	1614058.7	1746413.3	1676634.9	1797605.4	1514862.0	1743163.0	1675420.2	1254518.4	1175251.2	1221736.1	1149554.1
Q80UX8	Protein ABHD13	Abhd13	337	38.5	8.48	287589.4	292195.7	271962.7	298130.1	302166.7	286141.4	280702.3	290852.2	271252.4	291928.2	361239.3	372775.7	386315.9	393794.9
Q9WV74	Ankyrin repeat and SOCS box protein 1	Asb1	336	37.2	7.65	95666.9	104439.8	116553.2	111244.9	110497.1	101314.4	98354.5	92468.3	112577.5	114373.3	#N/A	#N/A	#N/A	#N/A
Q9WUC3	Lymphocyte antigen 6H	Ly6h	139	14.7	7.53	12372.2	11582.8	7693.8	11298.6	11235.6	13318.7	19156.1	16281.2	16459.6	15169.2	#N/A	#N/A	#N/A	#N/A
E9PYI4	Maestro heat-like repeat family member 8	Mroh8	1049	119.4	8.21	171725.0	163245.8	159240.7	177543.3	167189.1	163670.5	181926.9	155702.8	185104.7	163995.8	#N/A	#N/A	#N/A	#N/A
P58681	Toll-like receptor 7	Tlr7	1050	121.8	7.02	23677.9	29667.1	30773.5	31600.2	28416.3	30974.2	30394.6	30105.3	31779.2	29949.3	#N/A	#N/A	#N/A	#N/A
Q80U38	Protein KHNYN	Khnyn	671	74.5	7.87	155404.5	160479.7	156109.6	177248.1	169906.6	176108.2	162389.9	144976.1	164331.2	173976.1	21621.4	25932.8	18593.2	20809.9
P27808	Alpha-1,3-mannosyl-glycoprotein 2-beta-N-acetylglucosaminyltransferase	Mgat1	447	51.7	8.9	170006.7	148524.2	183289.3	192529.7	150628.0	203102.2	191460.2	220028.2	199434.9	175223.5	227852.3	201713.0	185892.1	200684.8
Q61176	Arginase-1	Arg1	323	34.8	7.01	677911.5	598002.3	553769.9	539746.0	559396.5	559607.5	525887.9	584920.3	631486.6	643945.5	#N/A	#N/A	#N/A	#N/A
Q922G0	Solute carrier family 25 member 36	Slc25a36	311	34.1	8.65	219085.5	219353.4	212018.0	214001.7	226823.4	228852.8	196241.2	218869.8	226681.1	211660.3	194432.4	195664.7	180062.7	187012.0
Q7TNS2	MICOS complex subunit Mic10	Micos10	76	8.6	8.94	141878.7	123708.6	171757.8	138704.5	147161.3	133834.2	155900.4	145021.1	154418.3	135098.9	175949.1	176623.7	206071.5	208605.1
Q8VBU8	Protein BANP	Banp	548	59.6	5.1	199852.2	203335.3	198552.9	196065.8	207283.9	201343.6	208545.2	184701.0	201251.2	212069.1	#N/A	#N/A	#N/A	#N/A
D0VYS2	Heat shock factor protein 3	Hsf3	492	57.3	5.2	134883.0	120153.0	132020.8	144520.3	129923.1	168937.1	147980.0	137802.6	138650.4	118641.3	#N/A	#N/A	#N/A	#N/A
Q8BJM5	Zinc transporter 6	Slc30a6	460	51	9.29	14038.3	17186.2	15910.0	17174.7	14808.0	15593.2	13879.4	14834.1	11511.0	12933.9	#N/A	#N/A	#N/A	#N/A
P03953	Complement factor D	Cfd	259	28	6.67	43472.5	46819.5	50388.1	43045.8	47017.5	50524.9	48933.2	43273.0	46003.5	41696.5	71750.3	73481.7	59855.5	56569.6
Q9D1F0	DUF4939 domain-containing protein	Rtl8b	113	13.6	8.4	153048.7	142328.2	142442.6	137239.8	138988.0	157261.8	137449.7	145750.2	148150.0	139303.8	#N/A	#N/A	#N/A	#N/A
P0DJE0	Protein PET100 homolog, mitochondrial	Pet100	76	9.4	9.13	94367.0	93874.8	96184.1	96307.7	100098.9	97722.9	103409.1	93959.9	112765.5	106003.8	#N/A	#N/A	#N/A	#N/A
Q60611	DNA-binding protein SATB1	Satb1	764	85.8	6.54	34829.4	27170.8	38704.1	25590.1	53799.9	24699.1	57958.9	19785.8	30640.2	44275.5	54077.7	62633.2	58360.0	52161.6
P81069	GA-binding protein subunit beta-2	Gabpb2	414	45.6	4.91	135118.2	140837.7	129765.1	145280.3	146714.7	155530.7	122377.0	143406.6	123537.9	133839.4	128670.7	141006.1	128618.4	138711.1
Q99K30	Epidermal growth factor receptor kinase substrate 8-like protein 2	Eps8l2	729	82.2	7.18	83926.7	79698.0	96660.3	79082.7	91240.4	103406.1	79402.3	88384.6	80664.1	79643.8	#N/A	#N/A	#N/A	#N/A
E9Q4T4	Coiled-coil domain-containing 71-like	Ccdc71l	218	24.2	11.52	283659.2	285701.3	269289.8	264287.3	286490.3	290558.3	259990.2	259911.2	265563.3	294834.8	#N/A	#N/A	#N/A	#N/A
Q9DCN7	E3 ubiquitin-protein ligase RNFT1	Rnft1	395	45.2	8.15	49973.4	65103.8	46522.4	49666.2	49020.5	55957.9	54840.2	52130.5	57753.2	51029.3	#N/A	#N/A	#N/A	#N/A
Q8R3A2	Cysteine-rich DPF motif domain-containing protein 1	Cdpf1	119	13.3	7.87	252622.6	251755.4	255627.9	256500.4	254891.4	269389.5	257797.4	228073.6	259047.3	290439.3	#N/A	#N/A	#N/A	#N/A
Q3UHC1	Rap guanine nucleotide exchange factor (GEF) 1	Rapgef1	1224	136.4	6.24	29576.9	36505.4	32105.4	34145.9	32475.3	29276.5	30005.1	24669.5	31023.4	37408.1	#N/A	#N/A	#N/A	#N/A
B2KF05	Bromodomain and PHD finger-containing protein 3	Brpf3	1204	135.2	6.48	42826.1	56556.2	47023.0	55794.4	46645.0	52842.2	46838.6	43823.6	53736.7	49560.4	34536.3	45349.8	35083.7	40505.4
A1L3P4	Sodium/hydrogen exchanger	Slc9a6	702	77.9	6.37	166942.6	173796.2	199765.2	150045.0	210909.8	223026.2	190799.7	184450.1	193831.7	173043.9	377878.0	367705.1	465212.0	382742.2
Q9QUI1	Leucine repeat adapter protein 25	Fam89b	189	20.1	5.19	152282.2	175940.0	143412.0	164718.2	155367.1	147811.4	145641.5	145226.0	155776.6	169821.5	117291.1	134574.9	109586.4	115084.3
Q8VIK2	Mpv17-like protein 2	Mpv17l2	200	22.5	9.22	47133.8	43596.4	39022.8	42203.1	42215.3	38179.6	43691.8	41370.9	46376.9	46187.8	49677.8	48668.7	51848.4	44884.3
Q7TMK6	Protein Hook homolog 2	Hook2	716	83.3	5.43	322260.3	351388.3	352316.7	331894.5	343429.6	382340.3	464623.0	431333.2	415608.3	353823.9	1463347.2	1309954.6	1655336.2	1398460.7
Q62010	Oviduct-specific glycoprotein	Ovgp1	721	78.8	9.19	93782.4	69293.8	78334.1	67981.8	74488.8	91015.6	112888.9	87086.8	89554.9	74535.8	#N/A	#N/A	#N/A	#N/A
Q71KU9	Fibrinogen-like protein 1	Fgl1	314	36.4	5.71	78864.9	95467.6	92598.4	87125.2	98147.1	102255.1	75309.2	73110.9	92029.7	92443.1	#N/A	#N/A	#N/A	#N/A
Q61462	Cytochrome b-245 light chain	Cyba	192	20.7	9.23	29790.4	35644.6	33433.3	34969.8	39238.4	44364.5	36403.8	39883.5	36168.1	32306.2	#N/A	#N/A	#N/A	#N/A
Q99N05	Membrane-spanning 4-domains subfamily A member 4D	Ms4a4d	225	23.8	8.84	208183.8	219337.2	218320.8	243465.9	230334.4	298658.0	283734.1	217236.0	211915.4	209038.4	#N/A	#N/A	#N/A	#N/A
Q8R422	CD109 antigen	Cd109	1442	161.6	5.57	107111.2	107744.4	100502.9	83533.5	107843.7	121779.3	113142.2	107165.3	97413.2	106840.5	12241.7	17694.9	10256.6	14284.9
Q6XZL8	Histone-lysine N-methyltransferase, H3 lysine-79 specific	Dot1l	1540	165.8	9.2	126779.7	116995.0	104946.3	113468.2	123874.5	121545.7	111998.2	106842.3	110538.7	132638.9	97355.5	116232.8	94453.7	113041.7
P37889	Fibulin-2	Fbln2	1221	131.7	4.68	50820.2	49338.3	51798.7	48680.8	54660.8	51656.0	41700.2	48623.7	49439.7	55932.7	83608.0	95347.7	81178.8	82974.3

Q6PE84	Stomatin-like protein 3	Stoml3	287	31.6	8.84	698381.0	696273.6	609037.6	702681.3	604563.6	623884.5	585124.0	606345.5	593017.2	649251.2	#N/A	#N/A	#N/A	#N/A
Q9JIB0	Ran guanine nucleotide release factor	Rangrf	185	20.4	5.45	42415.1	47423.0	43387.9	40526.4	41197.9	40973.1	46167.1	35704.4	46559.6	42378.7	#N/A	#N/A	#N/A	#N/A
Q9CPY4	Cyclin-dependent kinase 2-associated protein 2	Cdk2ap2	127	13.2	9.48	456677.0	511939.9	536272.7	459197.6	563929.6	475496.2	489019.6	534443.4	592277.3	534536.2	314917.8	400488.9	378493.0	378488.9
Q60698	Ski oncogene	Ski	725	80.1	7.85	9672.5	7292.0	10143.5	9011.5	9950.5	11993.4	14071.5	10728.4	10367.3	8085.0	#N/A	#N/A	#N/A	#N/A
Q99MK9	Ras association domain-containing protein 1	Rassf1	340	38.8	9.04	285943.6	350417.6	376926.4	343783.2	303642.8	314415.1	280708.9	294435.3	348718.4	337045.0	334326.3	412287.8	477238.2	456274.7
Q8VII3	Cystatin-14	Cst14	130	15.1	7.01	188938.5	199519.9	184234.0	200601.6	169426.4	168038.0	182458.3	186552.3	195906.1	174715.2	#N/A	#N/A	#N/A	#N/A
O35473	Nuclear nucleic acid-binding protein C1D	C1d	141	15.9	9.04	226228.7	202992.4	203114.4	219633.1	227393.3	232156.1	213833.7	197486.5	193068.4	192112.9	198110.5	186703.6	196573.9	186795.5
A2A699	Protein FAM171A2	Fam171a2	822	87.4	8.05	130265.7	133655.9	123187.5	138618.2	139481.8	151770.8	133524.8	122679.6	139086.4	137628.1	#N/A	#N/A	#N/A	#N/A
D3Z2X2	Dynein heavy chain domain 1	Dnhd1	4750	536.2	7.06	961449.7	985083.6	1098061.3	858732.0	1253409.0	1381581.2	1296657.5	1005746.5	1110241.5	1191069.1	1006898.8	953410.6	1133450.3	873924.8
O35393	Ephrin-B3	Efnb3	340	35.9	8.25	76956.4	80918.9	69659.9	68045.9	70456.9	78326.8	79712.8	67497.1	68631.1	73040.1	#N/A	#N/A	#N/A	#N/A
Q3UC65	Arginine/serine-rich protein 1	Rsrp1	298	34.5	11.77	1123342.6	976055.5	723113.0	930216.7	803337.2	744568.8	627033.2	769384.2	852560.1	882749.3	1186960.8	1206433.0	862756.9	1046528.2
P60898	DNA-directed RNA polymerase II subunit RPB9	Polr2i	125	14.5	5.14	324916.7	312496.7	350302.3	322347.2	372167.9	377749.9	333488.4	394820.8	355334.2	333165.2	#N/A	#N/A	#N/A	#N/A
Q9D115	Zinc finger protein 706	Znf706	76	8.5	10.01	533745.0	610810.2	409312.3	493396.6	431846.2	451241.8	393385.0	423529.8	460567.6	537358.1	38612.4	44913.6	47845.3	41074.2
O70258	Epsilon-sarcoglycan	Sgce	437	49.7	6.39	29193.0	29540.6	31688.7	23615.4	32365.8	31576.5	28675.9	31614.4	29982.1	30489.0	32892.2	29487.8	28648.1	25551.0
Q6P6L0	Filamin A-interacting protein 1-like	Filip1l	1131	129.7	6.37	81085.1	85584.6	72203.8	75819.3	77549.1	99423.6	80804.8	84576.0	84081.4	79260.3	#N/A	#N/A	#N/A	#N/A
Q8CG64	Fukutin-related protein	Fkrp	494	54.8	7.17	14198.4	19950.2	14942.0	15321.3	21901.3	20154.9	16672.8	17145.0	16111.5	16052.5	16420.9	27758.2	16718.1	19115.9
Q8BIF9	Zinc finger protein 787	Znf787	381	40.5	8.24	1317096.9	1160737.5	1230990.8	1132254.2	1436662.2	1425121.0	1501430.1	1136528.7	1260505.8	1305130.8	#N/A	#N/A	#N/A	#N/A
F6Y3H4	Predicted gene, 17387	Gm17387	24	2.8	10.29	335036.4	381194.9	356281.4	359083.0	386622.9	338922.5	328368.1	312502.5	328769.0	351958.3	321456.8	338953.5	333175.0	342909.4
Q9D2H1	Cytochrome c oxidase subunit 7B2	Cox7b2	82	9.4	9.66	362702.0	575180.0	712470.6	917259.4	367896.9	261363.1	212762.3	379490.8	528586.7	521721.8	257483.0	429815.3	531812.9	648690.2
Q9CZH7	Matrix-remodeling-associated protein 7	Mxra7	178	19.4	4.26	237152.8	270138.8	260044.6	263509.9	284323.1	254528.2	260480.5	286082.1	254240.6	233708.1	#N/A	#N/A	#N/A	#N/A
O88822	Lathosterol oxidase	Sc5d	299	35	8.75	336097.9	273648.3	309178.4	305285.7	302183.7	290148.7	248869.8	323496.2	290162.9	272053.0	#N/A	#N/A	#N/A	#N/A
Q63871	DNA-directed RNA polymerases I, II, and III subunit RPABC4	Polr2k	58	7	9.06	547885.3	606961.8	619948.9	575054.1	627348.4	615778.6	567874.2	547274.5	667638.0	700823.9	874181.0	917140.9	940766.5	929356.4
Q148V8	Protein FAM83H	Fam83h	1209	131	7.24	6043.4	6084.6	8324.4	7163.3	4914.4	9327.8	9181.8	7226.4	4615.6	7519.8	#N/A	#N/A	#N/A	#N/A
Q8BJS7	Microtubule-associated protein 10	Map10	891	96.1	7.39	2471.8	4268.7	2683.1	4447.5	2915.4	1143.7	2537.7	3774.4	1757.2	3409.7	#N/A	#N/A	#N/A	#N/A
Q7TST0	Butyrophilin-like protein 1	Btnl1	509	57.7	5.44	123617.4	126904.2	117810.1	119521.5	135615.5	122882.1	120242.6	108975.8	134283.8	135520.2	#N/A	#N/A	#N/A	#N/A
O88689	Protocadherin alpha-4	Pcdha4	947	103.1	5.08	457108.8	449926.3	519874.9	453504.0	508213.4	495760.4	481261.5	481886.6	471189.2	508287.1	#N/A	#N/A	#N/A	#N/A
Q9DD06	Retinoic acid receptor responder protein 2	Rarres2	162	18.3	9.2	161276.0	161833.1	150739.7	156954.6	194434.7	219550.7	205646.4	186763.6	160534.6	164295.2	#N/A	#N/A	#N/A	#N/A
Q8CHK4	Histone acetyltransferase KAT5	Kat5	513	58.6	8.48	45270.6	36803.0	36647.3	49393.9	48331.9	41740.0	44596.6	43914.2	40153.5	41157.3	#N/A	#N/A	#N/A	#N/A
A0A1D5RLM8	Predicted gene 11639	Gm11639	5808	653.8	5.4	45852.5	43841.2	50186.2	44977.3	57224.4	61091.0	46077.1	47140.8	47996.3	46622.6	#N/A	#N/A	#N/A	#N/A
Q32M07	Adenylate kinase 8	Ak8	479	55	7.08	70991.4	87384.8	68471.1	89649.4	70743.0	85947.8	79262.4	70108.0	73036.3	86381.9	#N/A	#N/A	#N/A	#N/A
Q9QXE2	DNA polymerase lambda	Poll	573	62.9	8	182183.9	128797.9	146140.2	118416.8	149099.5	159099.6	131063.2	285606.3	117621.2	135584.1	47018.3	34436.2	44411.8	37340.3
Q923D4	Splicing factor 3B subunit 5	Sf3b5	86	10.1	6.35	89286.3	93385.0	69650.5	81669.9	70495.5	80200.4	75473.0	72828.1	82624.5	91361.0	346681.2	303013.3	253478.1	306549.7
Q6W8Q3	Purkinje cell protein 4-like protein 1	Pcp4l1	68	7.5	5.52	19265.5	8093.7	8453.4	11094.4	5664.1	10384.9	17571.7	17941.2	11439.3	7868.0	#N/A	#N/A	#N/A	#N/A
P70324	T-box transcription factor TBX3	Tbx3	741	79.1	8	165979.2	146025.9	160014.1	164545.7	158490.6	167302.5	210999.4	179141.6	156449.3	159837.8	#N/A	#N/A	#N/A	#N/A
Q69ZN6	N-acetylglucosamine-1-phosphotransferase subunits alpha/beta	Gnptab	1235	140.9	7.66	263122.5	289480.7	242047.9	247249.8	275835.5	279511.8	258496.4	241216.2	268262.4	260350.0	#N/A	#N/A	#N/A	#N/A
Q61193	Ral guanine nucleotide dissociation stimulator-like 2	Rgl2	778	83.8	6.73	106323.3	151615.6	100937.2	125795.8	123310.4	149407.6	98258.5	115346.1	120040.8	126750.1	138215.0	209914.9	128508.0	142773.5
B2RX12	Canalicular multispecific organic anion transporter 2	Abcc3	1523	169	7.06	642726.0	667374.2	686489.0	642613.9	784287.5	734511.1	755081.9	673798.3	737369.8	770234.4	406481.5	431515.3	488013.2	431700.7
Q7M757	Lys-63-specific deubiquitinase BRCC36-like	C6.1al	291	33.1	6.23	161969.7	178095.0	146612.9	171273.2	136314.5	138020.9	136945.6	132706.1	148755.3	164284.6	#N/A	#N/A	#N/A	#N/A
Q91XZ4	Protocadherin beta-6	Pcdhb6	772	84.2	4.79	24363.0	22936.6	24114.9	26252.3	26144.8	25359.0	24221.4	17673.0	25230.2	24048.6	#N/A	#N/A	#N/A	#N/A
Q61626	Glutamate receptor ionotropic, kainate 5	Grik5	979	109.2	8.21	425419.7	328140.8	283311.4	323767.7	346307.1	363722.4	409965.9	326984.9	379989.7	388915.8	735904.6	577552.1	471234.2	527090.5
A6H6E9	Tetratricopeptide repeat protein 23-like	Ttc23l	458	51.5	8.13	111233.5	126182.6	121970.7	116012.3	116758.1	118801.9	105819.0	112273.7	120869.1	125303.7	#N/A	#N/A	#N/A	#N/A
P19091	Androgen receptor	Ar	899	98.1	6.79	405706.2	316936.2	343354.6	309158.9	296052.0	336306.2	376145.5	405310.1	321842.2	320574.8	21917.2	16991.0	20803.3	21965.0
Q9JJC9	Homocysteine-responsive endoplasmic reticulum-resident ubiquitin-like domain member 2 protein	Herpud2	404	44.5	5.07	229341.0	213401.9	223401.0	227294.3	238704.8	242156.6	225575.6	249852.3	220397.8	215499.7	279573.3	253538.5	249664.3	255080.4
Q6P6J4	DNA oxidative demethylase ALKBH2	Alkbh2	239	27.1	9.42	85102.8	84341.3	84549.5	75652.1	93355.1	92960.5	83798.4	75483.6	80977.3	82349.0	#N/A	#N/A	#N/A	#N/A
Q3TJ91	LLGL scribble cell polarity complex component 2	Llgl2	1027	114.3	7.46	67968.1	63327.4	66610.5	58578.2	82509.3	69221.1	70354.5	70769.4	66367.3	60536.7	#N/A	#N/A	#N/A	#N/A
Q80Y83	Dixin	Dixdc1	711	80.2	6.18	240924.7	258187.1	256202.0	250006.3	242089.4	230939.2	213756.3	229407.1	254503.4	260123.8	168827.0	168429.9	183648.9	165688.5
Q9ESW8	Pyroglutamyl-peptidase 1	Pgpep1	209	22.9	5.39	151080.5	145060.4	133420.6	120651.5	148019.6	161207.9	164130.8	149407.4	147297.3	141323.2	#N/A	#N/A	#N/A	#N/A
A0AUP1	Coiled-coil domain-containing protein 112	Ccdc112	442	52.7	9.6	130302.2	125702.0	141441.6	136711.3	146115.4	174087.2	188222.2	146003.7	156154.3	150610.5	#N/A	#N/A	#N/A	#N/A
Q9WUB7	Chloride channel protein ClC-Ka	Clcnka	687	75.6	8.98	361586.7	310122.8	340608.8	302512.7	337755.9	357059.9	362555.3	359572.7	370739.7	341740.0	#N/A	#N/A	#N/A	#N/A
P48999	Arachidonate 5-lipoxygenase	Alox5	674	77.9	6.18	209219.6	208400.6	166287.5	244401.3	163485.9	174882.0	201158.4	215507.3	168363.4	203861.3	#N/A	#N/A	#N/A	#N/A
D3YXK0	Janus kinase and microtubule-interacting protein 2	Jakmip2	820	96.3	5.97	199337.2	270428.6	174569.6	202826.9	210771.5	247460.0	193634.5	215639.3	205534.4	222103.4	#N/A	#N/A	#N/A	#N/A
Q8CHH5	BRD4-interacting chromatin-remodeling complex-associated protein-like	Bicral	1074	114.3	6.62	117314.9	125702.8	91311.1	124027.6	124280.6	95645.2	88127.4	87751.1	106735.7	107543.0	#N/A	#N/A	#N/A	#N/A
Q91XE0	Glycine N-acyltransferase	Glyat	296	34.1	8.28	94637.6	111565.7	82182.4	109453.5	83561.9	88608.0	81979.5	73281.8	86036.2	107436.7	#N/A	#N/A	#N/A	#N/A
Q9JLY0	Suppressor of cytokine signaling 6	Socs6	533	59	7.34	201548.3	192203.6	211362.3	175117.3	250872.3	218170.0	216863.7	190115.7	211033.6	213141.6	#N/A	#N/A	#N/A	#N/A
Q3URV1	Protein broad-minded	Tbc1d32	1296	148	6.15	100272.3	121502.4	126418.4	108984.7	110524.8	101896.0	91364.1	95420.9	104784.4	106954.8	71756.4	61724.2	55905.0	69289.8
Q9D173	Mitochondrial import receptor subunit TOM7 homolog	Tomm7	55	6.2	10.17	495911.6	723597.1	569089.7	640935.9	633077.9	595391.0	544312.9	607396.9	639937.4	618844.5	3932.4	6134.2	4843.2	3972.8
P12660	Purkinje cell protein 2	Pcp2	120	13	5.14	2843.2	5840.8	3572.2	4533.5	5181.3	5062.9	5348.0	6272.5	2838.7	5853.1	#N/A	#N/A	#N/A	#N/A
Q6NXH2	Glycoprotein endo-alpha-1,2-mannosidase	Manea	462	53.1	8.62	31695.6	28956.4	30419.6	27365.3	27720.8	30680.3	27590.0	28745.6	25040.9	27754.1	#N/A	#N/A	#N/A	#N/A
Q8C850	Scavenger receptor class A member 3	Scara3	606	65.4	6.44	308153.8	274523.3	260794.7	275481.8	326872.0	304685.6	333130.2	246433.3	297028.8	325416.7	125396.0	122618.4	138248.2	114454.4
Q7TN73	N-acetylneuraminate 9-O-acetyltransferase	Casd1	797	91.5	8.76	24355.5	18606.3	18820.0	22634.9	23131.4	25035.4	22191.3	18827.4	26089.8	23024.3	#N/A	#N/A	#N/A	#N/A
O54819	Tissue factor pathway inhibitor	Tfpi	306	35	8.4	92595.9	83953.5	102215.4	76718.0	93483.8	96442.5	89156.5	94806.6	94996.9	92905.6	#N/A	#N/A	#N/A	#N/A
Q76LL6	FH1/FH2 domain-containing protein 3	Fhod3	1578	175.5	5.55	45386.0	53604.2	46830.4	43933.1	48220.6	47038.8	36101.6	40990.1	44104.8	51617.9	#N/A	#N/A	#N/A	#N/A
Q9D3X5	PACRG-like protein	Pacrgl	248	27.3	9.89	176189.3	215702.0	207737.1	218873.6	194977.3	224025.2	180698.7	177078.2	190356.1	200212.6	205388.0	236305.0	219525.0	237610.0
P59054	Cysteine/serine-rich nuclear protein 1	Csrnp1	583	62.5	4.74	126232.8	155524.4	119373.7	150836.1	132458.6	137069.9	138348.9	148527.3	123756.5	140006.4	#N/A	#N/A	#N/A	#N/A
Q9JHR9	Nuclear receptor-interacting protein 2	Nrip2	270	29.3	7.68	81641.2	93053.6	74801.8	89726.8	89952.2	94278.1	81089.6	74521.0	88110.6	84747.5	#N/A	#N/A	#N/A	#N/A
Q9Z261	Claudin-7	Cldn7	211	22.3	8.7	63820.5	78992.6	65049.4	73954.4	76864.5	72083.6	68813.8	63377.3	69411.2	75134.0	#N/A	#N/A	#N/A	#N/A
Q7M732	Retrotransposon-like protein 1	Rtl1	1744	198.9	4.26	172887.1	199787.6	188939.4	202725.3	177983.3	195681.4	206862.2	158173.6	184444.5	188810.9	#N/A	#N/A	#N/A	#N/A
Q8BSL4	Heparan sulfate glucosamine 3-O-sulfotransferase 5	Hs3st5	346	40.4	9.7	19104.2	27462.3	20315.6	17576.8	21826.4	26747.1	19588.4	18138.6	19333.2	17347.1	#N/A	#N/A	#N/A	#N/A
Q9CQ66	Tctex1 domain-containing protein 2	Tctex1d2	144	16.5	5.19	199676.9	298412.8	267647.2	370048.5	196882.1	151107.2	137484.1	157959.0	223617.2	260635.3	329415.7	413983.0	438949.9	518965.0
Q9D8U2	Transmembrane protein 41A	Tmem41a	264	29.4	8.81	342089.8	325015.5	338606.9	338904.8	359280.1	361975.7	343285.9	297986.3	328297.5	315483.2	505906.2	504087.8	492683.5	507153.4
B8JJD3	FA_FANCE domain-containing protein	Fance	526	56.9	5.3	317203.9	301495.2	294132.1	270433.8	359584.7	387700.0	352753.8	279100.2	367446.8	372806.7	#N/A	#N/A	#N/A	#N/A
Q8JZL3	Thiamine-triphosphatase	Thtpa	224	24.2	4.72	207692.4	219448.2	191821.6	216523.6	204703.8	202081.0	200605.4	188259.0	192510.5	207350.1	150298.5	141365.2	124829.6	145385.5
Q9JJ48	Zinc finger CCCH domain-containing protein 8	Zc3h8	305	34.9	9.01	517949.9	608377.4	560322.9	576395.3	446526.8	500898.1	390023.7	422075.6	502714.4	556288.7	974907.8	1292198.2	1135272.6	1235053.9
Q8R2M0	Transcription elongation factor A N-terminal and central domain-containing protein 2	Tceanc2	207	24.2	10.05	42754.6	54311.0	41532.7	49324.6	41164.2	48125.6	36721.8	32636.4	36766.7	45046.3	#N/A	#N/A	#N/A	#N/A
P56528	ADP-ribosyl cyclase/cyclic ADP-ribose hydrolase 1	Cd38	304	34.4	8.29	236598.9	230743.5	214166.3	230255.5	221458.0	225069.9	237631.3	208594.8	229631.5	240728.5	#N/A	#N/A	#N/A	#N/A
Q61056	Short transient receptor potential channel 1	Trpc1	793	91.2	8.05	260259.8	268909.8	279619.5	263818.5	307347.3	271658.8	232225.5	268373.8	250304.0	277055.8	#N/A	#N/A	#N/A	#N/A
Q9D4H4	Angiomotin-like protein 1	Amotl1	968	107.9	7.39	185016.8	188897.9	177005.9	184262.5	167460.6	186425.8	177735.7	183165.1	192314.7	199066.5	#N/A	#N/A	#N/A	#N/A
D3YV89	ZnMc domain-containing protein	Mmp27	551	62.5	7.12	55110.9	46685.2	39679.2	40305.1	43496.7	43914.1	45368.4	45670.4	46908.0	51858.4	#N/A	#N/A	#N/A	#N/A
Q8K2A8	Dol-P-Man:Man(5)GlcNAc(2)-PP-Dol alpha-1,3-mannosyltransferase	Alg3	438	50.2	9.2	48827.3	44412.8	44557.2	54615.3	45283.6	43347.9	36994.8	37959.0	45793.2	51488.6	#N/A	#N/A	#N/A	#N/A
P28481	Collagen alpha-1(II) chain	Col2a1	1487	141.9	6.92	16355.0	15360.9	11223.5	16964.6	12219.3	14450.5	17113.4	19584.5	12711.8	14170.6	#N/A	#N/A	#N/A	#N/A
Q62191	E3 ubiquitin-protein ligase TRIM21	Trim21	470	54.1	7.08	129871.8	118872.6	132850.4	118529.3	142242.8	143585.8	141801.7	125122.2	133312.0	136705.1	165207.8	162545.4	164521.4	153271.2
Q9QXV0	ProSAAS	Pcsk1n	258	27.3	5.85	218587.0	246449.7	237273.7	190184.6	206703.8	223251.0	200725.3	254083.7	219745.2	224971.7	#N/A	#N/A	#N/A	#N/A
Q8BJM3	Coiled-coil domain-containing protein R3HCC1L	R3hcc1l	775	84.4	4.87	175794.0	161378.3	117639.4	166768.4	134445.4	125957.4	126451.1	126519.7	156524.9	161876.9	67555.2	72725.1	48411.4	59066.0
Q8BFR6	AN1-type zinc finger protein 1	Zfand1	268	30.2	8.1	199118.3	204282.0	158258.9	176364.6	179691.1	175635.1	170974.2	169779.8	190918.5	216880.8	234803.9	211602.5	182071.6	208898.3
Q684R7	FRAS1-related extracellular matrix protein 1	Frem1	2191	244.4	6.39	373090.2	301226.7	149780.4	274339.7	110149.4	140836.0	198772.3	176102.2	182355.5	211371.8	#N/A	#N/A	#N/A	#N/A
Q9Z100	Probable carboxypeptidase X1	Cpxm1	722	80.9	6.89	665515.4	575866.6	639570.7	548821.9	746344.9	739694.1	744001.4	608932.4	613328.3	673172.2	#N/A	#N/A	#N/A	#N/A
Q8C4U3	Secreted frizzled-related protein 1	Sfrp1	314	35.4	8.94	205706.1	196941.3	190744.1	176651.5	171114.3	178248.6	219560.5	233560.4	192037.8	212247.3	150614.1	145013.4	133061.6	132044.4
Q9QZI8	Serine incorporator 1	Serinc1	453	50.5	6.28	60084.9	69238.9	50408.5	62112.4	55358.8	64146.6	52723.3	44378.7	62246.1	62220.3	54248.0	59829.9	44457.3	57518.5
Q9JJZ4	Ubiquitin-conjugating enzyme E2 J1	Ube2j1	318	35	6.99	421168.0	444744.2	417896.5	420566.5	432101.5	467810.7	436744.8	419331.2	438006.1	441803.5	#N/A	#N/A	#N/A	#N/A
Q91Z22	Porimin	Tmem123	195	20.2	9.51	352585.9	399795.0	371051.6	389984.5	391577.6	366716.2	340714.0	339872.5	385213.7	411310.8	#N/A	#N/A	#N/A	#N/A
Q8K330	Protein phosphatase Slingshot homolog 3	Ssh3	649	72.2	5.57	86867.8	90793.7	84385.2	97229.0	79269.8	88353.8	86005.6	78827.1	84284.9	94747.7	#N/A	#N/A	#N/A	#N/A
Q03517	Secretogranin-2	Scg2	617	70.6	4.75	183324.4	182535.5	157073.0	180102.9	191233.2	213507.4	181882.2	159886.6	173940.9	192224.3	#N/A	#N/A	#N/A	#N/A

A0A0U1RPT6	DUF4629 domain-containing protein	930433I11F	723	79.4	8.54	66235.5	65448.2	60524.5	55662.5	27164.1	39847.9	56921.2	61959.0	69035.4	44494.3	#N/A	#N/A	#N/A	#N/A
Q80VN0	Cilia- and flagella-associated protein 100	Cfap100	613	72	8.51	121441.5	110894.1	126461.1	114389.7	123057.3	131725.0	147975.2	174570.3	116269.4	113615.4	656228.1	602005.5	663196.2	625855.5
Q5PR69	Capping protein inhibiting regulator of actin dynamics	Crad	1207	132.2	5.4	76539.1	72910.1	79421.1	86653.7	52952.5	52666.7	57751.3	66911.5	65293.0	67205.7	#N/A	#N/A	#N/A	#N/A
Q9CR70	EKC/KEOPS complex subunit Lage3	Lage3	148	15.8	8.13	718738.7	730234.3	674395.4	695940.0	648140.7	711044.4	689514.9	622648.9	646043.2	677621.8	#N/A	#N/A	#N/A	#N/A
Q8VC85	U6 snRNA-associated Sm-like protein LSm1	Lsm1	133	15.2	5.38	112147.6	167559.6	121735.0	128342.9	136271.3	167689.5	111963.4	125041.0	131084.6	127527.5	478999.0	474896.7	544109.3	508286.9
Q63870	Collagen alpha-1(VII) chain	Col7a1	2944	295.1	6.34	17769.4	23148.4	23225.9	26343.7	28064.4	26166.2	20529.9	24003.4	20024.1	22278.3	#N/A	#N/A	#N/A	#N/A
Q99N89	39S ribosomal protein L43, mitochondrial	Mrpl43	183	20.2	9.52	485089.3	486242.7	531913.9	489576.3	506567.9	412617.1	425656.6	491229.2	481082.9	505632.9	347562.1	354293.4	352782.8	364369.1
Q9EQX4	Allograft inflammatory factor 1-like	Aif1l	150	17	7.18	116493.4	112261.6	123023.1	108248.1	131212.9	141858.1	138130.7	128818.0	126428.5	117587.0	#N/A	#N/A	#N/A	#N/A
Q8BVR5	N-terminal Ras-GEF domain-containing protein	30474N05I	218	25.7	8.13	13390.8	9343.8	13402.3	13157.8	11931.0	13435.9	11874.0	9813.1	11201.1	15946.8	36583.8	45459.8	37275.4	38823.1
Q9JLI2	Collagen type V alpha 3 chain	Col5a3	1739	171.9	6.8	140114.6	124356.9	144359.3	133187.1	158039.6	168531.2	135025.0	143363.2	142241.3	127031.1	#N/A	#N/A	#N/A	#N/A
Q9QYS2	Metabotropic glutamate receptor 3	Grm3	879	99.1	7.75	557692.6	497639.9	455565.9	474593.8	466811.9	453554.2	498383.6	450545.1	481605.6	458093.4	459173.5	391225.0	401528.9	369550.6
A0A1Y7VMP7	MutL homolog 3	Mlh3	1443	162.4	7.05	16889.8	15613.9	19173.0	13463.3	20786.1	21567.3	18473.9	17919.9	13990.0	11524.2	73917.8	63996.8	76060.9	74328.7
Q9Z0F6	Cell cycle checkpoint control protein RAD9A	Rad9a	389	42	5.72	55147.8	53326.1	42417.7	49327.0	50282.7	51928.5	56819.8	46385.2	49855.5	53159.4	#N/A	#N/A	#N/A	#N/A
Q8K1G2	Limbin	Evc2	1220	137.6	6.2	7435.0	5781.0	4622.0	6157.7	6006.8	5409.9	6986.1	8612.5	4984.1	4646.3	#N/A	#N/A	#N/A	#N/A
Q3V037	Uncharacterized protein C6orf163 homolog	Gm136	328	38.5	7.01	44782.8	39606.8	35593.3	45740.1	49655.2	39624.6	61340.1	30286.8	36312.1	45956.6	600269.3	597926.1	472031.7	564800.3
Q9Z1M0	P2X purinoceptor 7	P2rx7	595	68.3	8.22	210154.4	337297.5	195842.6	273113.0	308904.8	426981.0	267605.0	257895.3	255920.9	295215.1	223227.5	330822.9	231340.3	273664.2
Q30D77	Collagen alpha-1(XXIV) chain	Col24a1	1733	175.6	7.34	49927.9	43115.3	40233.8	42782.4	46558.6	51447.2	69138.0	77973.3	41295.3	40070.7	#N/A	#N/A	#N/A	#N/A
P97357	TATA box-binding protein-associated factor RNA polymerase I subunit A	Taf1a	453	52.7	8.72	567923.3	539385.3	537064.2	562435.5	543809.4	527840.4	549759.4	539647.2	559184.0	594122.5	217037.7	226107.8	201794.9	209789.7
Q6P5U7	NACHT and WD repeat domain-containing protein 2	Nwd2	1742	197.3	6.15	106262.5	73181.9	87217.3	89437.7	90632.0	90895.6	117621.5	89305.7	102551.6	90613.0	170002.9	124356.1	131562.9	150832.1
Q8K445	Clarin-1	Clrn1	232	25.8	8.53	901939.8	870387.2	657122.1	853908.2	713147.3	717846.0	702385.6	643983.3	745551.1	817645.7	#N/A	#N/A	#N/A	#N/A
Q9CPW5	Translocon-associated protein subunit beta	Ssr2	183	20	8.35	224395.2	271947.0	268349.1	300338.6	247662.1	252454.7	211832.5	236412.2	289414.3	266895.8	722966.2	801642.1	846633.6	786095.9
A0A2I3BR92	Mcf.2-transforming sequence	Mcf2	1111	127.1	7.14	105951.7	115758.6	102533.8	121354.4	126335.4	115456.0	116501.4	98543.8	115807.5	106658.0	#N/A	#N/A	#N/A	#N/A
Q9CQ91	NADH dehydrogenase [ubiquinone] 1 alpha subcomplex subunit 3	Ndufa3	84	9.3	8.47	847875.1	916102.3	880056.5	1017019.6	826216.8	747737.6	755477.4	931776.6	828058.9	825914.3	772609.3	741631.2	738223.4	804174.7
Q4ACU6	SH3 and multiple ankyrin repeat domains protein 3	Shank3	1730	185.3	8.88	663486.5	913313.0	627434.8	798739.0	716046.3	769576.0	697132.5	623701.2	742799.4	798567.4	#N/A	#N/A	#N/A	#N/A
Q3U6N9	UPF0488 protein C8orf33 homolog	MGI:215233	222	24.5	10.56	216382.3	224450.9	205421.0	205865.8	214717.8	222180.4	188004.4	209151.0	217394.5	226315.3	#N/A	#N/A	#N/A	#N/A
Q8BU47	Ferritin	Ftdc2	192	22	9.32	385420.4	455355.2	409860.4	464603.4	388114.0	340501.3	333290.0	313958.2	401932.6	427597.5	449875.8	466069.9	484861.1	510584.7
Q61161	Mitogen-activated protein kinase kinase kinase kinase 2	Map4k2	821	91.2	6.46	111344.2	126102.9	106113.5	134319.6	112116.6	104772.1	92909.9	117033.8	107878.1	104167.4	30757.5	35232.8	33232.3	33280.9
Q8BH35	Complement component C8 beta chain	C8b	589	66.2	7.77	350086.5	442025.5	251508.3	349283.7	257133.4	235200.7	303092.7	287480.2	300211.3	305249.5	347412.3	416383.9	251222.7	354175.6
Q0VF58	Collagen alpha-1(XIX) chain	Col19a1	1136	114.1	8.41	96895.5	118244.7	101257.1	104073.9	112077.3	126029.5	120299.4	102802.8	106142.5	116514.2	#N/A	#N/A	#N/A	#N/A
Q61982	Neurogenic locus notch homolog protein 3	Notch3	2318	244.1	5.33	712904.7	562620.4	640065.9	575777.8	670168.4	748615.6	922500.9	712171.7	681508.6	678747.1	#N/A	#N/A	#N/A	#N/A
Q8BJA3	Homeobox-containing protein 1	Hmbox1	419	47.1	6.19	383652.8	387769.3	320703.2	369558.4	319892.9	323133.4	325076.0	352252.7	341162.1	341669.7	#N/A	#N/A	#N/A	#N/A
Q9DB28	Ribonuclease P/MRP protein subunit POP5	Pop5	169	19.3	7.9	26300.2	45431.2	25645.3	27853.1	32293.8	28373.1	25440.1	27280.5	24583.7	36378.5	#N/A	#N/A	#N/A	#N/A
P23949	mRNA decay activator protein ZFP36L2	Zfp36l2	484	50	8.24	96627.0	100339.6	78933.8	82741.3	107819.5	101259.3	99344.9	78185.2	95336.1	89992.8	#N/A	#N/A	#N/A	#N/A
E9Q3A5	G_PROTEIN_RECEP_F3_4 domain-containing protein	Vmn2r105	860	98.7	7.59	525553.0	444254.5	473790.0	425881.3	547703.9	551206.1	528893.9	538621.7	533385.4	436057.0	#N/A	#N/A	#N/A	#N/A
Q4QRL3	Coiled-coil domain-containing protein 88B	Ccdc88b	1481	166.5	5.33	991379.6	903802.3	618998.4	824781.1	985720.8	1292414.3	1398425.1	983296.7	927701.1	863635.3	533306.0	452102.4	313305.8	431356.1
P03966	N-myc proto-oncogene protein	Mycn	462	49.5	5.55	61954.2	89228.6	59727.7	78535.2	62825.8	69259.5	65429.6	60660.1	69187.5	76563.8	#N/A	#N/A	#N/A	#N/A
O35664	Interferon alpha/beta receptor 2	Ifnar2	513	56.5	4.59	213096.3	194710.2	188557.7	189932.2	193887.5	222305.0	242407.7	211482.8	192302.4	217115.7	#N/A	#N/A	#N/A	#N/A
P35991	Tyrosine-protein kinase BTK	Btk	659	76.4	7.9	347388.0	328306.8	314451.2	336815.2	358575.4	341990.3	317350.5	372808.8	336853.4	274002.3	#N/A	#N/A	#N/A	#N/A
Q8BH75	E3 ubiquitin-protein ligase NRDP1	Rnf41	317	35.9	6.14	242995.6	235051.0	230686.2	220099.5	241136.6	259624.5	246904.5	234244.4	234507.0	247067.8	#N/A	#N/A	#N/A	#N/A
Q8BY79	Solute carrier family 35 member G1	Slc35g1	368	40.2	7.84	293766.0	318348.0	313434.4	303397.3	402762.4	359895.3	349498.7	313616.5	330816.8	357433.4	#N/A	#N/A	#N/A	#N/A
Q3TVC7	Cyclin-D1-binding protein 1	Ccndbp1	356	39.3	4.87	300410.2	252795.8	222360.3	231003.3	248501.5	247527.3	278088.1	224773.7	248756.7	238584.7	407801.2	450244.4	398643.5	427816.9
Q8CB59	Protein FAM161B	Fam161b	589	66.9	9.54	418181.9	581494.2	460086.8	512517.7	484585.3	476217.2	475241.1	414980.7	474109.7	555673.0	#N/A	#N/A	#N/A	#N/A
O35633	Vesicular inhibitory amino acid transporter	Slc32a1	525	57.3	6.64	2097895.9	1425714.2	2022500.3	1526604.3	2087076.1	2295562.6	2354818.1	2384898.2	1749156.3	1664737.3	#N/A	#N/A	#N/A	#N/A
Q9QZD5	Rab proteins geranylgeranyltransferase component A 2	Chml	621	70	5.19	55080.1	79900.5	55127.3	63737.4	54384.2	65661.5	49212.0	55855.8	58100.7	60472.3	#N/A	#N/A	#N/A	#N/A
Q9CPY1	39S ribosomal protein L51, mitochondrial	Mrpl51	128	15.1	11	563693.9	536378.9	624328.5	550279.6	599037.0	549140.0	514985.5	624980.6	561581.2	529419.9	17575.1	19711.1	18536.8	19831.0
Q8BVF9	Archaemetzincin-1	Amz1	502	55.2	6.09	603635.2	604859.1	439787.7	576452.9	463331.7	456443.3	465687.7	393906.6	500010.3	577976.1	768234.7	1406206.4	663462.4	986828.3
Q80YT7	Myomegalin	Pde4dip	2224	250.5	5.53	458067.0	573603.1	412951.0	543476.8	446047.2	419959.2	361531.1	587249.8	589130.5	568585.5	279748.7	330576.9	242613.4	316819.7
Q9D8N3	Lysoplasmalogenase-like protein TMEM86A	Tmem86a	241	26.3	8.56	986303.3	1061314.0	991354.3	1106814.1	937270.4	856179.4	874464.3	908892.7	974511.4	1034009.3	#N/A	#N/A	#N/A	#N/A
A2ARV4	Low-density lipoprotein receptor-related protein 2	Lrp2	4660	518.9	5.15	208868.3	200830.0	176768.0	180139.6	180675.8	184327.3	191625.2	170834.5	191859.1	188553.6	272443.5	253908.5	276337.7	244099.2
Q08761	Vitamin K-dependent protein S	Pros1	675	74.9	5.82	202791.7	236483.8	211173.8	218749.0	218922.7	226289.0	207224.2	254342.0	203508.5	215856.0	#N/A	#N/A	#N/A	#N/A
Q61818	Retinoic acid-induced protein 1	Rai1	1889	201.4	8.75	408777.5	433121.9	356482.9	413598.7	409683.5	494889.8	462811.4	442690.4	429329.4	402841.0	#N/A	#N/A	#N/A	#N/A
Q7TNC6	Kinesin-like protein KIF26B	Kif26b	2112	225.4	8.5	562291.0	651066.2	612429.7	638739.6	540955.3	480240.3	515752.7	502334.5	600077.3	629945.6	#N/A	#N/A	#N/A	#N/A
Q9QYZ3	Sperm motility kinase 2B	Smok2b	484	54.7	9.32	452756.5	479002.8	479861.5	512899.3	490002.7	501853.5	455296.9	443744.0	494560.1	481116.1	#N/A	#N/A	#N/A	#N/A
P23440	Rod cGMP-specific 3',5'-cyclic phosphodiesterase subunit beta	Pde6b	856	98.5	5.31	53611.0	43671.2	56221.5	53917.3	60338.1	57536.4	50918.6	54735.4	52259.4	56714.9	#N/A	#N/A	#N/A	#N/A
A2ALS5	Rap1 GTPase-activating protein 1	Rap1gap	663	73.4	5.87	64150.9	67806.4	59922.1	66142.5	57980.4	57261.1	65574.8	51145.4	70953.6	61145.2	#N/A	#N/A	#N/A	#N/A
Q9Z0P7	Suppressor of fused homolog	Sufu	484	53.9	5.44	8784.1	4370.9	5010.9	7175.7	6992.3	9650.3	8607.2	5568.5	5211.1	6648.9	#N/A	#N/A	#N/A	#N/A
Q9R0U9	Testis-expressed gene 21	Tex21	575	65.4	7.72	27694.0	30094.0	26494.9	28167.4	35133.9	30028.1	31446.2	29027.0	26731.7	22899.7	#N/A	#N/A	#N/A	#N/A
Q78DX7	Proto-oncogene tyrosine-protein kinase ROS	Ros1	2340	261.8	6.07	130431.4	142681.8	155029.2	164970.6	167166.9	140893.1	143320.7	133328.8	149290.3	160877.0	#N/A	#N/A	#N/A	#N/A
Q8N7N6	SEFIR domain-containing protein	Traf3ip2	555	62.4	6.58	358827.3	370401.6	405347.3	350929.8	421352.4	427636.8	333433.5	374473.9	327666.5	344957.0	#N/A	#N/A	#N/A	#N/A
Q9CYB0	E3 ubiquitin-protein ligase TRIM13	Trim13	407	46.9	6.34	132329.2	118700.0	130981.9	125768.8	125957.0	141650.7	147762.8	122890.2	123221.1	119780.5	#N/A	#N/A	#N/A	#N/A
Q8BWQ4	Cap-specific mRNA (nucleoside-2'-O-)-methyltransferase 2	Cmtr2	767	87.1	7.08	901422.0	722650.9	581684.0	1039485.8	603564.0	588908.8	1107960.8	808604.0	678506.6	911478.6	#N/A	#N/A	#N/A	#N/A
Q499E4	Zinc finger protein DZIP1L	Dzip1l	774	87.5	8.27	152130.4	231634.2	250669.9	303492.1	157021.4	142485.2	101677.4	134129.5	185519.9	207325.2	#N/A	#N/A	#N/A	#N/A
Q6PDF3	Putative transporter SVOPL	Svopl	494	54.4	8.1	657362.3	604473.1	567069.4	613361.1	602400.1	647379.4	624944.3	685083.2	572028.7	588593.6	#N/A	#N/A	#N/A	#N/A
Q0VBD0	Integrin beta-8	Itgb8	767	84.5	7.09	1147299.9	1114972.7	1135618.9	1225126.6	974206.4	969425.1	963775.9	957702.3	1042830.9	1052613.2	#N/A	#N/A	#N/A	#N/A
P37238	Peroxisome proliferator-activated receptor gamma	Pparg	505	57.6	6.18	101249.4	80694.9	90532.0	82333.7	117610.7	124982.9	134154.2	113494.1	107850.3	105813.8	#N/A	#N/A	#N/A	#N/A
P46062	Signal-induced proliferation-associated protein 1	Sipa1	1037	112	6.35	66820.5	69343.7	59993.3	58312.2	65639.5	68103.7	67006.5	70421.5	60330.8	59733.9	#N/A	#N/A	#N/A	#N/A
A9Z1V5	von Willebrand factor A domain-containing protein 5B1	Vwa5b1	1215	133.9	7.93	250139.9	290812.1	242991.0	362112.4	287664.9	495928.8	273800.5	235195.2	287349.2	204974.7	#N/A	#N/A	#N/A	#N/A
Q9D9Z5	DET1- and DDB1-associated protein 1	Dda1	102	11.7	8.68	9776.7	13760.3	13865.0	12168.5	11371.3	13878.9	11937.5	11862.7	10490.1	10897.0	265259.2	311296.2	292023.4	320239.2
A6H690	IQ and AAA domain-containing protein 1-like	Iqca1l	825	95.9	9.51	122130.7	127147.5	110914.1	125663.4	119805.5	106731.2	110293.4	114303.1	119343.9	109710.0	120460.6	124426.9	117755.5	127281.8
P58750	Serine/threonine-protein kinase pim-3	Pim3	326	35.9	5.83	89806.5	90382.5	77987.1	78537.5	83101.8	100849.9	97974.1	80070.0	84353.7	89332.6	#N/A	#N/A	#N/A	#N/A
Q99MI6	GTPase IMAP family member 3	Gimap3	301	34.2	7.09	24914.6	30509.2	26769.5	23921.0	31519.0	24453.5	35145.4	25578.5	28799.1	22518.0	#N/A	#N/A	#N/A	#N/A
P70365	Nuclear receptor coactivator 1	Ncoa1	1447	156.9	6.14	673753.8	765492.2	728873.1	738124.9	711071.5	711390.4	752394.3	680358.4	747912.8	798699.1	118942.0	134072.7	91829.5	125895.5
Q2YFS2	Paired immunoglobulin-like type 2 receptor beta	Pilrb	224	25.2	9.58	183286.4	173627.7	206374.0	182020.9	233139.6	263693.0	142195.2	196638.7	183280.0	181311.9	#N/A	#N/A	#N/A	#N/A
Q8CGP2	Histone H2B type 1-P	Hist1h2bp	126	14	10.32	#N/A	#N/A	#N/A	#N/A	#N/A	#N/A	#N/A	#N/A	#N/A	#N/A	3257693.9	4771834.2	5068652.7	3736194.2
Q8CGP0	Histone H2B type 3-B	H2bu1	126	13.9	10.32	#N/A	#N/A	#N/A	#N/A	#N/A	#N/A	#N/A	#N/A	#N/A	#N/A	188888.6	214112.8	258454.9	172723.6
P13542	Myosin-8	Myh8	1937	222.6	5.83	#N/A	#N/A	#N/A	#N/A	#N/A	#N/A	#N/A	#N/A	#N/A	#N/A	53227.8	48418.8	49685.1	47089.5
A6ZI47	Fructose-bisphosphate aldolase	Aldoart2	364	39.3	7.44	#N/A	#N/A	#N/A	#N/A	#N/A	#N/A	#N/A	#N/A	#N/A	#N/A	298697.1	331619.0	299673.4	315100.4
Q8K370	Acyl-CoA dehydrogenase family member 10	Acad10	1069	118.9	8.24	#N/A	#N/A	#N/A	#N/A	#N/A	#N/A	#N/A	#N/A	#N/A	#N/A	14223.1	11331.4	11656.1	10577.0
P33175	Kinesin heavy chain isoform 5A	Kif5a	1027	116.9	5.94	#N/A	#N/A	#N/A	#N/A	#N/A	#N/A	#N/A	#N/A	#N/A	#N/A	33878.9	32328.5	29632.0	29995.4
P59279	Ras-related protein Rab-2B	Rab2b	216	24.2	6.68	#N/A	#N/A	#N/A	#N/A	#N/A	#N/A	#N/A	#N/A	#N/A	#N/A	42108.0	37705.7	39769.7	36249.0
Q9CXU9	Eukaryotic translation initiation factor 1b	Eif1b	113	12.8	7.37	#N/A	#N/A	#N/A	#N/A	#N/A	#N/A	#N/A	#N/A	#N/A	#N/A	254329.7	303515.1	279912.8	289414.4
Q8BGY2	Eukaryotic translation initiation factor 5A-2	Eif5a2	153	16.8	5.58	#N/A	#N/A	#N/A	#N/A	#N/A	#N/A	#N/A	#N/A	#N/A	#N/A	265120.0	282037.6	299991.0	262328.1
Q60872	Eukaryotic translation initiation factor 1A	Eif1a	144	16.5	5.24	#N/A	#N/A	#N/A	#N/A	#N/A	#N/A	#N/A	#N/A	#N/A	#N/A	289594.5	330632.3	283390.2	282949.5
Q9QXY6	EH domain-containing protein 3	Ehd3	535	60.8	6.46	#N/A	#N/A	#N/A	#N/A	#N/A	#N/A	#N/A	#N/A	#N/A	#N/A	3853.8	6505.5	6694.1	6535.3
Q9JM52	Misshapen-like kinase 1	Mink1	1308	147.2	7.44	#N/A	#N/A	#N/A	#N/A	#N/A	#N/A	#N/A	#N/A	#N/A	#N/A	987981.0	815530.5	821593.8	818921.8
Q6IFZ6	Keratin, type II cytoskeletal 1b	Krt77	572	61.3	8.02	#N/A	#N/A	#N/A	#N/A	#N/A	#N/A	#N/A	#N/A	#N/A	#N/A	288608.1	316782.0	270899.6	324709.0
Q61831	Mitogen-activated protein kinase 10	Mapk10	464	52.5	6.96	#N/A	#N/A	#N/A	#N/A	#N/A	#N/A	#N/A	#N/A	#N/A	#N/A	154608.2	170800.8	165340.8	166111.5
Q8C4X2	Casein kinase I isoform gamma-3	Csnk1g3	424	48.9	9.11	#N/A	#N/A	#N/A	#N/A	#N/A	#N/A	#N/A	#N/A	#N/A	#N/A	214068.5	240203.2	260499.4	251062.1
P62838	Ubiquitin-conjugating enzyme E2 D2	Ube2d2	147	16.7	7.83	#N/A	#N/A	#N/A	#N/A	#N/A	#N/A	#N/A	#N/A	#N/A	#N/A	245316.4	335592.2	241546.6	284105.4
Q9WUA6	RAC-gamma serine/threonine-protein kinase	Akt3	479	55.7	6.02	#N/A	#N/A	#N/A	#N/A	#N/A	#N/A	#N/A	#N/A	#N/A	#N/A	63928.3	82228.3	65311.9	64382.1
Q8R2R9	AP-3 complex subunit mu-2	Ap3m2	418	46.9	7.56	#N/A	#N/A	#N/A	#N/A	#N/A	#N/A	#N/A	#N/A	#N/A	#N/A	38845.3	41956.9	43010.0	38103.6
Q9R1R2	Tripartite motif-containing protein 3	Trim3	744	80.7	7.81	#N/A	#N/A	#N/A	#N/A	#N/A	#N/A	#N/A	#N/A	#N/A	#N/A	115788.3	122764.0	104977.4	115152.7

Q62441	Transducin-like enhancer protein 4	Tle4	773	83.7	7.5	#N/A	#N/A	#N/A	#N/A	#N/A	#N/A	#N/A	#N/A	#N/A	#N/A	252544.8	237436.1	230371.9	210189.7
Q91W82	Ubiquitin-conjugating enzyme E2 E2	Ube2e2	201	22.2	7.71	#N/A	#N/A	#N/A	#N/A	#N/A	#N/A	#N/A	#N/A	#N/A	#N/A	33591.6	40110.7	36540.7	46183.6
A2AF31	Thymosin beta	Tmsb15b2	45	5.2	6	#N/A	#N/A	#N/A	#N/A	#N/A	#N/A	#N/A	#N/A	#N/A	#N/A	188767.3	182264.1	223770.6	211854.4
Q80WG5	Volume-regulated anion channel subunit LRRC8A	Lrrc8a	810	94.1	7.94	#N/A	#N/A	#N/A	#N/A	#N/A	#N/A	#N/A	#N/A	#N/A	#N/A	98020.3	92652.2	87255.6	90882.0
Q8VE92	RNA-binding protein 4B	Rbm4b	357	40	6.74	#N/A	#N/A	#N/A	#N/A	#N/A	#N/A	#N/A	#N/A	#N/A	#N/A	271292.8	286990.0	298101.3	248633.2
P97329	Kinesin-like protein KIF20A	Kif20a	887	99.8	6.99	#N/A	#N/A	#N/A	#N/A	#N/A	#N/A	#N/A	#N/A	#N/A	#N/A	88627.8	98141.1	95357.3	84475.8
P08103	Tyrosine-protein kinase HCK	Hck	524	59.1	7.24	#N/A	#N/A	#N/A	#N/A	#N/A	#N/A	#N/A	#N/A	#N/A	#N/A	137214.6	138994.7	133118.4	134180.8
Q8VC28	Aldo-keto reductase family 1 member C13	Akr1c13	323	37	7.08	#N/A	#N/A	#N/A	#N/A	#N/A	#N/A	#N/A	#N/A	#N/A	#N/A	361534.7	397974.9	316214.3	440434.7
P40694	DNA-binding protein SMUBP-2	Ighmbp2	993	109.4	8.4	#N/A	#N/A	#N/A	#N/A	#N/A	#N/A	#N/A	#N/A	#N/A	#N/A	35007.2	35141.7	35622.2	35551.8
A2BI40	Cor1 domain-containing protein	Xlr4a	215	24.7	8.92	#N/A	#N/A	#N/A	#N/A	#N/A	#N/A	#N/A	#N/A	#N/A	#N/A	44299.5	65188.6	49022.4	56264.3
Q8VEG6	CCR4-NOT transcription complex subunit 6-like	Cnot6l	555	63	6.57	#N/A	#N/A	#N/A	#N/A	#N/A	#N/A	#N/A	#N/A	#N/A	#N/A	190640.6	202095.0	190464.8	202695.6
P70340	Mothers against decapentaplegic homolog 1	Smad1	465	52.1	7.31	#N/A	#N/A	#N/A	#N/A	#N/A	#N/A	#N/A	#N/A	#N/A	#N/A	399595.1	369968.6	393470.8	343098.4
Q9CWR0	Rho guanine nucleotide exchange factor 25	Arhgef25	618	68.2	5.29	#N/A	#N/A	#N/A	#N/A	#N/A	#N/A	#N/A	#N/A	#N/A	#N/A	40811.2	48566.2	41394.5	38708.9
Q99M73	Keratin, type II cuticular Hb4	Krt84	603	64.9	7.84	#N/A	#N/A	#N/A	#N/A	#N/A	#N/A	#N/A	#N/A	#N/A	#N/A	83762.1	88634.9	78802.9	73816.2
Q5SYH2	Transmembrane protein 199	Tmem199	208	23.1	9.1	#N/A	#N/A	#N/A	#N/A	#N/A	#N/A	#N/A	#N/A	#N/A	#N/A	88951.4	107029.2	98598.7	104687.3
Q8VEF1	Protein Aster-A	Gramd1a	722	80.6	6.86	#N/A	#N/A	#N/A	#N/A	#N/A	#N/A	#N/A	#N/A	#N/A	#N/A	58911.5	55141.9	54829.2	50532.0
Q8BKT8	HAUS augmin-like complex subunit 7	Haus7	364	40.6	5.06	#N/A	#N/A	#N/A	#N/A	#N/A	#N/A	#N/A	#N/A	#N/A	#N/A	83849.2	95828.8	92487.3	97499.2
Q3TKR2	Cor1 domain-containing protein	Xlr4c	215	24.8	9.17	#N/A	#N/A	#N/A	#N/A	#N/A	#N/A	#N/A	#N/A	#N/A	#N/A	173103.5	168365.9	150242.8	151419.4
Q6NY15	Testis-specific gene 10 protein	Tsga10	697	81.2	5.8	#N/A	#N/A	#N/A	#N/A	#N/A	#N/A	#N/A	#N/A	#N/A	#N/A	41641.5	65162.8	60211.6	65317.0
Q8BVW0	Neutral alpha-glucosidase C	Ganc	898	101.9	6.32	#N/A	#N/A	#N/A	#N/A	#N/A	#N/A	#N/A	#N/A	#N/A	#N/A	144212.9	142955.1	130904.8	155706.7
E9PYD1	Family with sequence similarity 98, member C	Fam98c	344	37.2	8.62	#N/A	#N/A	#N/A	#N/A	#N/A	#N/A	#N/A	#N/A	#N/A	#N/A	252183.6	263545.4	272284.8	267304.8
P08043	Zinc finger protein 2	Zfp2	459	52.5	8.82	#N/A	#N/A	#N/A	#N/A	#N/A	#N/A	#N/A	#N/A	#N/A	#N/A	81346.2	53076.7	98954.3	68656.5
Q6P9Z1	SWI/SNF-related matrix-associated actin-dependent regulator of chromatin subfamily D member 3	Smarcd3	483	55	9.35	#N/A	#N/A	#N/A	#N/A	#N/A	#N/A	#N/A	#N/A	#N/A	#N/A	43036.7	44558.0	30249.1	37606.6
B2RUR8	OTU domain-containing protein 7B	Otud7b	840	91.9	6.86	#N/A	#N/A	#N/A	#N/A	#N/A	#N/A	#N/A	#N/A	#N/A	#N/A	42137.0	43432.9	44961.7	40703.6
Q9QX11	Cytohesin-1	Cyth1	398	46.2	5.63	#N/A	#N/A	#N/A	#N/A	#N/A	#N/A	#N/A	#N/A	#N/A	#N/A	184848.6	177607.5	177980.3	186629.3
Q8R527	Rho-related GTP-binding protein RhoQ	Rhoq	205	22.6	6.32	#N/A	#N/A	#N/A	#N/A	#N/A	#N/A	#N/A	#N/A	#N/A	#N/A	24227.8	30580.6	21101.7	23803.6
Q9CYK1	Tryptophan--tRNA ligase, mitochondrial	Wars2	360	40.1	8.82	#N/A	#N/A	#N/A	#N/A	#N/A	#N/A	#N/A	#N/A	#N/A	#N/A	123685.2	109934.4	114207.7	110198.5
Q9R1C0	Transcription initiation factor TFIID subunit 7	Taf7	341	39.1	5.33	#N/A	#N/A	#N/A	#N/A	#N/A	#N/A	#N/A	#N/A	#N/A	#N/A	45125.0	50253.6	52263.0	44347.9
Q8BW96	Calcium/calmodulin-dependent protein kinase type 1D	Camk1d	385	42.9	7.17	#N/A	#N/A	#N/A	#N/A	#N/A	#N/A	#N/A	#N/A	#N/A	#N/A	208065.0	205315.6	188110.6	196191.8
P01656	Ig kappa chain V-III region MOPC 70	-	111	11.9	5.34	#N/A	#N/A	#N/A	#N/A	#N/A	#N/A	#N/A	#N/A	#N/A	#N/A	101293.4	119268.2	102423.8	99852.5
Q2KHK3	Aminopeptidase Q	Lvrn	559	63.3	5.55	#N/A	#N/A	#N/A	#N/A	#N/A	#N/A	#N/A	#N/A	#N/A	#N/A	47530.9	45517.2	38603.2	45794.0
P25911	Tyrosine-protein kinase Lyn	Lyn	512	58.8	7.15	#N/A	#N/A	#N/A	#N/A	#N/A	#N/A	#N/A	#N/A	#N/A	#N/A	121330.6	127945.6	117714.2	110845.4
P29037	TATA-box-binding protein	Tbp	316	34.7	9.79	#N/A	#N/A	#N/A	#N/A	#N/A	#N/A	#N/A	#N/A	#N/A	#N/A	161855.4	168790.9	140376.3	158573.0
Q62187	Transcription termination factor 1	Ttf1	859	97.7	9.38	#N/A	#N/A	#N/A	#N/A	#N/A	#N/A	#N/A	#N/A	#N/A	#N/A	57964.5	61560.9	58556.8	58409.0
Q6ZQJ5	DNA replication ATP-dependent helicase/nuclease DNA2	Dna2	1062	119.4	6.98	#N/A	#N/A	#N/A	#N/A	#N/A	#N/A	#N/A	#N/A	#N/A	#N/A	110650.9	122867.3	104378.8	126070.4
Q8K1B8	Fermitin family homolog 3	Fermt3	665	75.6	7.05	#N/A	#N/A	#N/A	#N/A	#N/A	#N/A	#N/A	#N/A	#N/A	#N/A	76426.3	82974.0	77988.9	82318.9
Q923A2	Protein Spindly	Spdl1	608	70.2	6	#N/A	#N/A	#N/A	#N/A	#N/A	#N/A	#N/A	#N/A	#N/A	#N/A	2326.8	2389.7	4001.2	4365.3
Q8K4D3	Proton-coupled amino acid transporter 1	Slc36a1	475	52.4	6.83	#N/A	#N/A	#N/A	#N/A	#N/A	#N/A	#N/A	#N/A	#N/A	#N/A	49169.1	50440.4	48968.5	43417.0
Q68FE6	Rho family-interacting cell polarization regulator 1	Ripor1	1223	132.3	5.88	#N/A	#N/A	#N/A	#N/A	#N/A	#N/A	#N/A	#N/A	#N/A	#N/A	107769.6	101300.5	88488.7	100497.4
Q8VHL1	Histone-lysine N-methyltransferase SETD7	Setd7	366	40.5	4.65	#N/A	#N/A	#N/A	#N/A	#N/A	#N/A	#N/A	#N/A	#N/A	#N/A	215781.9	226091.7	200627.3	204884.8
P30415	NK-tumor recognition protein	Nktr	1453	163.4	10.05	#N/A	#N/A	#N/A	#N/A	#N/A	#N/A	#N/A	#N/A	#N/A	#N/A	130539.2	132046.6	134410.6	138345.2
Q9D312	Keratin, type I cytoskeletal 20	Krt20	431	49	5.38	#N/A	#N/A	#N/A	#N/A	#N/A	#N/A	#N/A	#N/A	#N/A	#N/A	1560286.5	1402959.9	1049393.9	1119213.9
Q9QWT9	Kinesin-like protein KIFC1	Kifc1	674	74.1	8.72	#N/A	#N/A	#N/A	#N/A	#N/A	#N/A	#N/A	#N/A	#N/A	#N/A	13099.0	14308.3	17778.0	17003.8
O35153	BET1-like protein	Bet1l	111	12.4	8.82	#N/A	#N/A	#N/A	#N/A	#N/A	#N/A	#N/A	#N/A	#N/A	#N/A	33404.7	44928.6	43977.9	50662.1
Q8C2P3	tRNA-dihydrouridine(16/17) synthase [NAD(P)(+)]-like	Dus1l	475	53.5	8.51	#N/A	#N/A	#N/A	#N/A	#N/A	#N/A	#N/A	#N/A	#N/A	#N/A	129447.2	121459.0	130109.1	112693.4
Q8BH82	N-acyl-phosphatidylethanolamine-hydrolyzing phospholipase D	Napepld	396	45.8	5.94	#N/A	#N/A	#N/A	#N/A	#N/A	#N/A	#N/A	#N/A	#N/A	#N/A	118918.3	121821.5	92618.8	110306.1
O54990	Prominin-1	Prom1	867	97.1	6.68	#N/A	#N/A	#N/A	#N/A	#N/A	#N/A	#N/A	#N/A	#N/A	#N/A	142533.6	134823.7	107904.4	189564.2
Q8K1A5	Transmembrane protein 41B	Tmem41b	291	32.4	9.31	#N/A	#N/A	#N/A	#N/A	#N/A	#N/A	#N/A	#N/A	#N/A	#N/A	13218.8	20105.5	16960.7	14289.9
O88393	Transforming growth factor beta receptor type 3	Tgfbr3	850	93.8	6	#N/A	#N/A	#N/A	#N/A	#N/A	#N/A	#N/A	#N/A	#N/A	#N/A	108335.4	120920.4	110083.3	130051.0
Q8BFQ9	Kelch-like protein 42	Klhl42	493	55.6	6.32	#N/A	#N/A	#N/A	#N/A	#N/A	#N/A	#N/A	#N/A	#N/A	#N/A	159579.3	151487.3	146612.5	144788.1
Q9DCI9	39S ribosomal protein L32, mitochondrial	Mrpl32	187	21.7	9.7	#N/A	#N/A	#N/A	#N/A	#N/A	#N/A	#N/A	#N/A	#N/A	#N/A	104976.9	105375.6	97589.8	94475.8
Q9DBT4	Ectonucleoside triphosphate diphosphohydrolase 4	Entpd4	613	69.7	8.07	#N/A	#N/A	#N/A	#N/A	#N/A	#N/A	#N/A	#N/A	#N/A	#N/A	106408.9	124312.7	110661.0	125186.1
Q812E0	Cytoplasmic polyadenylation element-binding protein 2	Cpeb2	521	58.4	7.5	#N/A	#N/A	#N/A	#N/A	#N/A	#N/A	#N/A	#N/A	#N/A	#N/A	66943.3	75349.9	78232.8	73438.4
O35730	E3 ubiquitin-protein ligase RING1	Ring1	406	42.6	5.74	#N/A	#N/A	#N/A	#N/A	#N/A	#N/A	#N/A	#N/A	#N/A	#N/A	87913.9	112630.0	96278.3	93799.6
Q8R3W2	RIKEN cDNA 0610009B22 gene	10009B22I	140	16.4	6.61	#N/A	#N/A	#N/A	#N/A	#N/A	#N/A	#N/A	#N/A	#N/A	#N/A	16020.1	23963.7	25314.8	23293.6
Q9Z2F7	BCL2/adenovirus E1B 19 kDa protein-interacting protein 3-like	Bnip3l	218	23.8	5.85	#N/A	#N/A	#N/A	#N/A	#N/A	#N/A	#N/A	#N/A	#N/A	#N/A	358286.6	298002.5	331807.1	316471.2
Q8QZS3	Folliculin	Flcn	579	64.3	6.38	#N/A	#N/A	#N/A	#N/A	#N/A	#N/A	#N/A	#N/A	#N/A	#N/A	304989.6	311286.9	294105.4	306437.8
Q4G0F8	Ubinuclein-1	Ubn1	1135	122.3	9.31	#N/A	#N/A	#N/A	#N/A	#N/A	#N/A	#N/A	#N/A	#N/A	#N/A	165244.6	161785.3	136714.4	154812.3
Q9ES52	Phosphatidylinositol 3,4,5-trisphosphate 5-phosphatase 1	Inpp5d	1191	133.5	7.8	#N/A	#N/A	#N/A	#N/A	#N/A	#N/A	#N/A	#N/A	#N/A	#N/A	471282.6	502925.2	493606.4	522097.9
Q9JHT5	AMME syndrome candidate gene 1 protein homolog	Ammecr1	344	36	8.63	#N/A	#N/A	#N/A	#N/A	#N/A	#N/A	#N/A	#N/A	#N/A	#N/A	196781.2	222543.7	194063.1	176367.5
Q9WVC3	Caveolin-2	Cav2	162	18.2	5.63	#N/A	#N/A	#N/A	#N/A	#N/A	#N/A	#N/A	#N/A	#N/A	#N/A	37255.0	36166.2	29162.1	42564.9
Q76KJ5	DNA-directed RNA polymerase I subunit RPA34	Cd3eap	399	43.1	9.61	#N/A	#N/A	#N/A	#N/A	#N/A	#N/A	#N/A	#N/A	#N/A	#N/A	66703.5	78821.5	78061.9	77098.0
G3X9X1	BTB domain-containing protein	Kbtbd2	623	71.2	5.62	#N/A	#N/A	#N/A	#N/A	#N/A	#N/A	#N/A	#N/A	#N/A	#N/A	87488.1	86034.7	85298.4	81491.4
Q7TPW1	Nexilin	Nexn	607	72.1	5.01	#N/A	#N/A	#N/A	#N/A	#N/A	#N/A	#N/A	#N/A	#N/A	#N/A	82869.2	84493.0	90348.6	89764.2
Q8VC57	BTB/POZ domain-containing protein KCTD5	Kctd5	234	26.1	5.95	#N/A	#N/A	#N/A	#N/A	#N/A	#N/A	#N/A	#N/A	#N/A	#N/A	5206.8	5885.1	7124.4	5740.4
Q91WE1	Sorting nexin-15	Snx15	337	37.7	5.24	#N/A	#N/A	#N/A	#N/A	#N/A	#N/A	#N/A	#N/A	#N/A	#N/A	867859.1	788126.1	768636.1	836970.2
Q9CWT6	Probable ATP-dependent RNA helicase DDX28	Ddx28	540	59.5	10.04	#N/A	#N/A	#N/A	#N/A	#N/A	#N/A	#N/A	#N/A	#N/A	#N/A	41151.9	50244.2	33573.5	40561.6
Q9CQD0	Ribosomal protein L39-like	Rpl39l	51	6.3	12.41	#N/A	#N/A	#N/A	#N/A	#N/A	#N/A	#N/A	#N/A	#N/A	#N/A	314182.0	352737.4	357405.6	337473.1
B1AVY7	Kinesin-like protein KIF16B	Kif16b	1312	150	6.44	#N/A	#N/A	#N/A	#N/A	#N/A	#N/A	#N/A	#N/A	#N/A	#N/A	69900.4	70909.0	65761.9	59450.2
P49117	Nuclear receptor subfamily 2 group C member 2	Nr2c2	596	65.2	6.28	#N/A	#N/A	#N/A	#N/A	#N/A	#N/A	#N/A	#N/A	#N/A	#N/A	144410.0	158618.9	141746.1	154640.0
Q9R1S3	GPI ethanolamine phosphate transferase 1	Pign	931	105	7.58	#N/A	#N/A	#N/A	#N/A	#N/A	#N/A	#N/A	#N/A	#N/A	#N/A	41157.3	46039.8	43711.8	38445.6
Q8C1S0	Mediator of RNA polymerase II transcription subunit 19	Med19	244	26.2	9.88	#N/A	#N/A	#N/A	#N/A	#N/A	#N/A	#N/A	#N/A	#N/A	#N/A	25613.0	25969.3	23720.0	25256.3
Q6PAJ1	Breakpoint cluster region protein	Bcr	1270	143	6.92	#N/A	#N/A	#N/A	#N/A	#N/A	#N/A	#N/A	#N/A	#N/A	#N/A	38468.0	35272.2	36190.5	34895.3
Q6ZWR6	Nesprin-1	Syne1	8799	1009.3	5.59	#N/A	#N/A	#N/A	#N/A	#N/A	#N/A	#N/A	#N/A	#N/A	#N/A	11016.7	13106.8	13927.9	17101.6
Q9QWH1	Polyhomeotic-like protein 2	Phc2	850	89.7	8.75	#N/A	#N/A	#N/A	#N/A	#N/A	#N/A	#N/A	#N/A	#N/A	#N/A	139372.5	139057.0	134988.6	127840.7
Q3TZX3	Solute carrier family 25 member 33	Slc25a33	320	35	9.67	#N/A	#N/A	#N/A	#N/A	#N/A	#N/A	#N/A	#N/A	#N/A	#N/A	307136.0	322637.7	321802.7	319419.0
Q3UX61	N-alpha-acetyltransferase 11	Naa11	218	24.7	5.63	#N/A	#N/A	#N/A	#N/A	#N/A	#N/A	#N/A	#N/A	#N/A	#N/A	10628.9	12710.2	10364.7	14847.7
A2RSQ0	DENN domain-containing protein 5B	Dennd5b	1274	144.5	6.68	#N/A	#N/A	#N/A	#N/A	#N/A	#N/A	#N/A	#N/A	#N/A	#N/A	56692.2	63612.7	66873.7	73599.5
Q9QYC7	Vitamin K-dependent gamma-carboxylase	Ggcx	757	87.1	8.02	#N/A	#N/A	#N/A	#N/A	#N/A	#N/A	#N/A	#N/A	#N/A	#N/A	197731.6	209225.4	163498.2	187109.1
A2RSY1	KAT8 regulatory NSL complex subunit 3	Kansl3	903	96.1	9.41	#N/A	#N/A	#N/A	#N/A	#N/A	#N/A	#N/A	#N/A	#N/A	#N/A	57862.5	67865.2	67407.9	47293.7
Q9ESH5	WAP four-disulfide core domain protein 1	Wfdc1	211	23.1	7.53	#N/A	#N/A	#N/A	#N/A	#N/A	#N/A	#N/A	#N/A	#N/A	#N/A	1096451.7	1077755.6	764166.1	884708.9
Q32NY4	Metal transporter CNNM3	Cnnm3	713	76.2	5.58	#N/A	#N/A	#N/A	#N/A	#N/A	#N/A	#N/A	#N/A	#N/A	#N/A	226056.2	221834.3	210180.0	205824.7
O35874	Neutral amino acid transporter A	Slc1a4	532	56	5.87	#N/A	#N/A	#N/A	#N/A	#N/A	#N/A	#N/A	#N/A	#N/A	#N/A	158520.1	165896.0	158428.9	143241.3
Q3ULA2	F-box/WD repeat-containing protein 1A	Btrc	605	68.9	7.88	#N/A	#N/A	#N/A	#N/A	#N/A	#N/A	#N/A	#N/A	#N/A	#N/A	145464.5	162296.8	172134.3	174169.4
Q9DAJ4	WD repeat domain-containing protein 83	Wdr83	315	34.4	5.87	#N/A	#N/A	#N/A	#N/A	#N/A	#N/A	#N/A	#N/A	#N/A	#N/A	29948.1	32143.0	25532.4	34135.1
Q8C9M2	Coiled-coil domain-containing protein 15	Ccdc15	810	93.5	8.09	#N/A	#N/A	#N/A	#N/A	#N/A	#N/A	#N/A	#N/A	#N/A	#N/A	69604.4	83692.7	76296.5	72656.2
Q14AI0	Sister chromatid cohesion protein DCC1	DSCC1	399	45.5	4.91	#N/A	#N/A	#N/A	#N/A	#N/A	#N/A	#N/A	#N/A	#N/A	#N/A	68790.4	83769.9	69897.2	79219.7
Q6ZWY8	Thymosin beta-10	Tmsb10	44	5	5.36	#N/A	#N/A	#N/A	#N/A	#N/A	#N/A	#N/A	#N/A	#N/A	#N/A	251726.6	313684.8	308054.7	298781.5
P01663	Ig kappa chain V-III region PC 4050	-	111	12	5.06	#N/A	#N/A	#N/A	#N/A	#N/A	#N/A	#N/A	#N/A	#N/A	#N/A	163647.3	162953.6	155161.1	153925.8
Q64373	Bcl-2-like protein 1	Bcl2l1	233	26.1	4.94	#N/A	#N/A	#N/A	#N/A	#N/A	#N/A	#N/A	#N/A	#N/A	#N/A	77348.6	89636.2	76748.6	78971.2
Q01755	T-complex protein 11	Tcp11	566	61.9	6.49	#N/A	#N/A	#N/A	#N/A	#N/A	#N/A	#N/A	#N/A	#N/A	#N/A	160520.5	197941.3	200643.3	230123.2
Q7TPM1	Protein PRRC2B	Prrc2b	1486	160.8	8.16	#N/A	#N/A	#N/A	#N/A	#N/A	#N/A	#N/A	#N/A	#N/A	#N/A	115536.9	110166.7	113534.6	96451.6

Q9D826	Peroxisomal sarcosine oxidase	Pipox	390	43.8	7.55	#N/A	#N/A	#N/A	#N/A	#N/A	#N/A	#N/A	#N/A	#N/A	#N/A	119512.5	191571.9	110679.8	139971.5
P08101	Low affinity immunoglobulin gamma Fc region receptor II	Fcgr2	330	36.7	6.73	#N/A	#N/A	#N/A	#N/A	#N/A	#N/A	#N/A	#N/A	#N/A	#N/A	65821.8	69601.3	59826.1	66134.3
P60882	Multiple epidermal growth factor-like domains protein 8	Megf8	2789	297.3	6.8	#N/A	#N/A	#N/A	#N/A	#N/A	#N/A	#N/A	#N/A	#N/A	#N/A	746466.5	918874.6	719492.5	732202.0
Q8VDU5	SNF-related serine/threonine-protein kinase	Snrk	748	81.9	7.49	#N/A	#N/A	#N/A	#N/A	#N/A	#N/A	#N/A	#N/A	#N/A	#N/A	151063.4	169214.4	147644.9	145022.3
Q9CQL5	39S ribosomal protein L18, mitochondrial	Mrpl18	180	20.7	9.28	#N/A	#N/A	#N/A	#N/A	#N/A	#N/A	#N/A	#N/A	#N/A	#N/A	25578.9	28245.2	33864.3	30664.9
Q3U2V3	8-oxo-dGDP phosphatase NUDT18	Nudt18	323	35.7	6.62	#N/A	#N/A	#N/A	#N/A	#N/A	#N/A	#N/A	#N/A	#N/A	#N/A	104573.0	118419.8	119902.6	139010.8
E9Q2I4	ELM2 and Myb/SANT-like domain-containing 1	Elmsan1	1089	119.6	9.19	#N/A	#N/A	#N/A	#N/A	#N/A	#N/A	#N/A	#N/A	#N/A	#N/A	20947.4	15348.0	15550.2	17620.8
Q8BWJ3	Phosphorylase b kinase regulatory subunit alpha, liver isoform	Phka2	1235	138.4	6.37	#N/A	#N/A	#N/A	#N/A	#N/A	#N/A	#N/A	#N/A	#N/A	#N/A	156319.8	168700.6	150221.9	152264.2
Q9DCL2	Cytosolic iron-sulfur assembly component 2A	Ciao2a	160	18.4	4.82	#N/A	#N/A	#N/A	#N/A	#N/A	#N/A	#N/A	#N/A	#N/A	#N/A	150900.7	168034.3	158047.6	171161.7
Q6PCN7	Helicase-like transcription factor	Hltf	1003	113.2	8.09	#N/A	#N/A	#N/A	#N/A	#N/A	#N/A	#N/A	#N/A	#N/A	#N/A	82820.5	123911.8	78478.0	98109.9
Q8R149	BUD13 homolog	Bud13	637	72.1	9.95	#N/A	#N/A	#N/A	#N/A	#N/A	#N/A	#N/A	#N/A	#N/A	#N/A	227364.8	247355.3	245654.7	247477.1
Q149C2	TRAF3-interacting protein 1	Traf3ip1	625	71	8.38	#N/A	#N/A	#N/A	#N/A	#N/A	#N/A	#N/A	#N/A	#N/A	#N/A	49196.2	59799.5	57243.4	57584.4
Q3U3E2	Protein FAM117B	Fam117b	584	61.3	9.95	#N/A	#N/A	#N/A	#N/A	#N/A	#N/A	#N/A	#N/A	#N/A	#N/A	35652.9	37883.9	33228.3	38784.1
A0A075B5V0	Immunoglobulin heavy variable 1-26	Ighv1-26	117	12.9	8.41	#N/A	#N/A	#N/A	#N/A	#N/A	#N/A	#N/A	#N/A	#N/A	#N/A	172676.1	108140.2	148641.9	171404.3
Q61188	Histone-lysine N-methyltransferase EZH2	Ezh2	746	85.2	6.89	#N/A	#N/A	#N/A	#N/A	#N/A	#N/A	#N/A	#N/A	#N/A	#N/A	30650.5	27336.1	24466.8	31474.9
B2RX88	Centrosome and spindle pole associated protein 1	Cspp1	1205	138.2	7.37	#N/A	#N/A	#N/A	#N/A	#N/A	#N/A	#N/A	#N/A	#N/A	#N/A	18387.3	20378.2	23192.8	20758.1
Q99MB1	Toll-like receptor 3	Tlr3	905	103.6	7.93	#N/A	#N/A	#N/A	#N/A	#N/A	#N/A	#N/A	#N/A	#N/A	#N/A	120757.6	100163.0	84921.2	92374.5
Q9CR24	Nucleoside diphosphate-linked moiety X motif 8	Nudt8	210	23.2	6.52	#N/A	#N/A	#N/A	#N/A	#N/A	#N/A	#N/A	#N/A	#N/A	#N/A	15455.5	15109.1	13765.0	10172.9
Q3U4H6	Hexosaminidase D	Hexd	486	54.5	6.1	#N/A	#N/A	#N/A	#N/A	#N/A	#N/A	#N/A	#N/A	#N/A	#N/A	54542.5	57982.5	50789.9	59522.1
Q6RHR9	Membrane-associated guanylate kinase, WW and PDZ domain-containing protein 1	Magi1	1471	161.9	7.36	#N/A	#N/A	#N/A	#N/A	#N/A	#N/A	#N/A	#N/A	#N/A	#N/A	71104.2	73662.4	59316.5	60515.3
Q8BXA1	Golgi integral membrane protein 4	Golim4	655	76.7	4.83	#N/A	#N/A	#N/A	#N/A	#N/A	#N/A	#N/A	#N/A	#N/A	#N/A	80702.7	90053.5	75628.0	80031.0
O55176	E3 ubiquitin-protein ligase Praja-1	Pja1	578	63.9	4.97	#N/A	#N/A	#N/A	#N/A	#N/A	#N/A	#N/A	#N/A	#N/A	#N/A	56521.4	44185.3	53798.1	58266.3
A0A140T8N1	Ig-like domain-containing protein	Igkv1-88	120	13.2	8.02	#N/A	#N/A	#N/A	#N/A	#N/A	#N/A	#N/A	#N/A	#N/A	#N/A	48950.9	46647.5	30618.1	29905.5
Q8VCG4	Complement component C8 gamma chain	C8g	202	22.5	9.25	#N/A	#N/A	#N/A	#N/A	#N/A	#N/A	#N/A	#N/A	#N/A	#N/A	74562.4	86140.7	67428.9	81777.0
Q501J2	Adenine nucleotide translocase lysine N-methyltransferase	Antkmt	229	24.7	8.6	#N/A	#N/A	#N/A	#N/A	#N/A	#N/A	#N/A	#N/A	#N/A	#N/A	130984.1	157893.6	145853.1	139438.4
Q99P30	Peroxisomal coenzyme A diphosphatase NUDT7	Nudt7	236	26.8	6.34	#N/A	#N/A	#N/A	#N/A	#N/A	#N/A	#N/A	#N/A	#N/A	#N/A	200551.7	224374.9	201944.2	200657.3
Q9EPL2	Calsyntenin-1	Clstn1	979	108.8	4.92	#N/A	#N/A	#N/A	#N/A	#N/A	#N/A	#N/A	#N/A	#N/A	#N/A	71416.1	82656.7	61933.7	65528.8
Q8BW41	Protein O-linked-mannose beta-1,4-N-acetylglucosaminyltransferase 2	Pomgnt2	605	69.3	8.78	#N/A	#N/A	#N/A	#N/A	#N/A	#N/A	#N/A	#N/A	#N/A	#N/A	55798.0	55136.9	53662.9	57454.7
A2AH22	Activating molecule in BECN1-regulated autophagy protein 1	Ambra1	1300	142.8	7.01	#N/A	#N/A	#N/A	#N/A	#N/A	#N/A	#N/A	#N/A	#N/A	#N/A	72666.4	71845.8	70453.0	73448.0
Q8BYR8	Solute carrier family 41 member 2	Slc41a2	573	62.3	6.2	#N/A	#N/A	#N/A	#N/A	#N/A	#N/A	#N/A	#N/A	#N/A	#N/A	114351.1	113513.7	122289.1	123086.6
P49813	Tropomodulin-1	Tmod1	359	40.4	5.1	#N/A	#N/A	#N/A	#N/A	#N/A	#N/A	#N/A	#N/A	#N/A	#N/A	40744.6	94422.4	36519.0	60220.7
Q6P1E1	Zinc finger MIZ domain-containing protein 1	Zmiz1	1072	115.8	7.44	#N/A	#N/A	#N/A	#N/A	#N/A	#N/A	#N/A	#N/A	#N/A	#N/A	512128.1	531348.8	530885.3	482920.3
Q9DAT5	Mitochondrial tRNA-specific 2-thiouridylase 1	Trmu	417	47.2	8.4	#N/A	#N/A	#N/A	#N/A	#N/A	#N/A	#N/A	#N/A	#N/A	#N/A	90675.4	88837.1	70215.0	79083.7
B1AUE5	Peroxisome biogenesis factor 10	Pex10	324	37.1	9.94	#N/A	#N/A	#N/A	#N/A	#N/A	#N/A	#N/A	#N/A	#N/A	#N/A	200196.5	220791.3	194120.6	224385.2
P35689	DNA repair protein complementing XP-G cells homolog	Ercc5	1170	130.6	5.35	#N/A	#N/A	#N/A	#N/A	#N/A	#N/A	#N/A	#N/A	#N/A	#N/A	153560.6	172023.6	175765.1	169170.0
Q9EQH4	Transcription initiation factor TFIID subunit 8	Taf8	308	34	6.46	#N/A	#N/A	#N/A	#N/A	#N/A	#N/A	#N/A	#N/A	#N/A	#N/A	13162.3	19258.6	16640.1	17887.5
Q8BW70	Ubiquitin carboxyl-terminal hydrolase 38	Usp38	1042	116	5.6	#N/A	#N/A	#N/A	#N/A	#N/A	#N/A	#N/A	#N/A	#N/A	#N/A	63768.2	9335.5	7113.7	7695.5
Q8R2Q6	Tectonic-3	Tctn3	595	64.8	7.9	#N/A	#N/A	#N/A	#N/A	#N/A	#N/A	#N/A	#N/A	#N/A	#N/A	28912.3	32077.4	28475.6	31376.0
P49710	Hematopoietic lineage cell-specific protein	Hcls1	486	54.2	4.84	#N/A	#N/A	#N/A	#N/A	#N/A	#N/A	#N/A	#N/A	#N/A	#N/A	125147.7	124574.7	112056.1	132073.3
E1U8D0	Protein SOGA1	Soga1	1418	159.1	6.46	#N/A	#N/A	#N/A	#N/A	#N/A	#N/A	#N/A	#N/A	#N/A	#N/A	87149.4	84471.8	65335.5	86308.5
Q9CQI9	Mediator of RNA polymerase II transcription subunit 30	Med30	178	20.3	8.28	#N/A	#N/A	#N/A	#N/A	#N/A	#N/A	#N/A	#N/A	#N/A	#N/A	66053.8	76131.4	78655.8	78722.0
A0A0B4J1N0	Ig-like domain-containing protein	Ighv1-76	98	10.9	9.23	#N/A	#N/A	#N/A	#N/A	#N/A	#N/A	#N/A	#N/A	#N/A	#N/A	172630.5	154592.3	208415.7	212142.3
Q8BW22	Calcium-responsive transactivator	Ss18l1	402	43.7	6.58	#N/A	#N/A	#N/A	#N/A	#N/A	#N/A	#N/A	#N/A	#N/A	#N/A	91195.4	84400.9	86756.5	85033.3
Q9R1E6	Ectonucleotide pyrophosphatase/phosphodiesterase family member 2	Enpp2	862	98.8	7.27	#N/A	#N/A	#N/A	#N/A	#N/A	#N/A	#N/A	#N/A	#N/A	#N/A	85363.1	90593.9	79754.7	85313.9
Q9CZ82	Mediator of RNA polymerase II transcription subunit 18	Med18	208	23.6	6.54	#N/A	#N/A	#N/A	#N/A	#N/A	#N/A	#N/A	#N/A	#N/A	#N/A	161918.3	159004.8	154781.6	167511.3
Q9D5R2	WD repeat-containing protein 20	Wdr20	567	62.7	8.48	#N/A	#N/A	#N/A	#N/A	#N/A	#N/A	#N/A	#N/A	#N/A	#N/A	135261.6	152291.9	135703.7	149813.2
Q8K211	High affinity copper uptake protein 1	Slc31a1	196	21.9	7.28	#N/A	#N/A	#N/A	#N/A	#N/A	#N/A	#N/A	#N/A	#N/A	#N/A	53137.1	58631.4	63266.9	52982.1
A0A075B5Y2	Ig-like domain-containing protein	Ighv1-75	117	12.9	8.59	#N/A	#N/A	#N/A	#N/A	#N/A	#N/A	#N/A	#N/A	#N/A	#N/A	89282.8	99034.3	81043.7	89672.9
Q9JKS5	Intracellular hyaluronan-binding protein 4	Habp4	411	45.9	6.84	#N/A	#N/A	#N/A	#N/A	#N/A	#N/A	#N/A	#N/A	#N/A	#N/A	205766.2	185938.4	188323.9	188115.0
Q9D9T8	EF-hand domain-containing protein 1	Efhc1	648	75.1	6.05	#N/A	#N/A	#N/A	#N/A	#N/A	#N/A	#N/A	#N/A	#N/A	#N/A	276856.5	342288.3	283866.0	402041.0
Q8K2Z8	Ubiquitin-conjugating enzyme E2 Q2	Ube2q2	378	42.9	5.03	#N/A	#N/A	#N/A	#N/A	#N/A	#N/A	#N/A	#N/A	#N/A	#N/A	229263.9	265681.1	209251.2	227494.7
Q60778	NF-kappa-B inhibitor beta	Nfkbib	359	37.9	4.74	#N/A	#N/A	#N/A	#N/A	#N/A	#N/A	#N/A	#N/A	#N/A	#N/A	125827.5	133851.8	123846.0	124884.0
Q9QZL9	Dickkopf-like protein 1	Dkkl1	230	26.6	8.24	#N/A	#N/A	#N/A	#N/A	#N/A	#N/A	#N/A	#N/A	#N/A	#N/A	361172.7	455602.7	436013.0	641770.2
Q8VIJ8	GATOR complex protein NPRL3	Nprl3	569	63.6	7.03	#N/A	#N/A	#N/A	#N/A	#N/A	#N/A	#N/A	#N/A	#N/A	#N/A	47603.8	44984.6	46779.0	61387.1
Q9D2C6	DNA-directed RNA polymerase III subunit RPC8	Polr3h	204	22.9	4.58	#N/A	#N/A	#N/A	#N/A	#N/A	#N/A	#N/A	#N/A	#N/A	#N/A	148416.3	151344.6	149894.8	153257.7
E9Q743	Cilia- and flagella-associated protein 251	Wdr66	1299	148.5	4.59	#N/A	#N/A	#N/A	#N/A	#N/A	#N/A	#N/A	#N/A	#N/A	#N/A	30437.0	53033.8	54928.6	77652.9
Q64163	Transcription factor Dp-2	Tfdp2	446	49.1	6.15	#N/A	#N/A	#N/A	#N/A	#N/A	#N/A	#N/A	#N/A	#N/A	#N/A	148401.3	145587.8	145068.8	130198.4
Q8K3K7	1-acyl-sn-glycerol-3-phosphate acyltransferase beta	Agpat2	278	31	8.88	#N/A	#N/A	#N/A	#N/A	#N/A	#N/A	#N/A	#N/A	#N/A	#N/A	143921.7	135884.6	126723.1	140124.1
Q8BGQ6	EF-hand calcium-binding domain-containing protein 14	Efcab14	484	53.7	6.34	#N/A	#N/A	#N/A	#N/A	#N/A	#N/A	#N/A	#N/A	#N/A	#N/A	242466.2	268596.7	229584.6	252422.1
Q8BFX1	E3 ubiquitin-protein ligase RNF187	Rnf187	236	26.3	5.59	#N/A	#N/A	#N/A	#N/A	#N/A	#N/A	#N/A	#N/A	#N/A	#N/A	89490.0	103192.8	83724.4	87670.5
A2AEY4	MAP7 domain-containing protein 3	Map7d3	876	98.2	9.42	#N/A	#N/A	#N/A	#N/A	#N/A	#N/A	#N/A	#N/A	#N/A	#N/A	24683.5	30963.2	28574.1	27171.1
A0A075B5S3	Ig-like domain-containing protein	Ighv14-4	111	12.1	4.67	#N/A	#N/A	#N/A	#N/A	#N/A	#N/A	#N/A	#N/A	#N/A	#N/A	66307.5	65783.2	58482.8	70227.7
P35569	Insulin receptor substrate 1	Irs1	1233	130.6	8.73	#N/A	#N/A	#N/A	#N/A	#N/A	#N/A	#N/A	#N/A	#N/A	#N/A	19848.5	23555.5	21372.3	21460.5
P21844	Chymase	Cma1	247	27.6	9.39	#N/A	#N/A	#N/A	#N/A	#N/A	#N/A	#N/A	#N/A	#N/A	#N/A	48332.6	33872.1	36945.2	30859.6
Q8BJS8	Mdm2-binding protein	Mtbp	894	100.3	7.31	#N/A	#N/A	#N/A	#N/A	#N/A	#N/A	#N/A	#N/A	#N/A	#N/A	209746.9	212904.2	186552.2	203489.0
Q8VHC3	Selenoprotein M	Selenom	145	16.4	5.54	#N/A	#N/A	#N/A	#N/A	#N/A	#N/A	#N/A	#N/A	#N/A	#N/A	52822.6	56761.2	54310.6	61348.7
A2RTF1	Cation channel sperm-associated protein subunit beta	Catsperb	1109	126	8.15	#N/A	#N/A	#N/A	#N/A	#N/A	#N/A	#N/A	#N/A	#N/A	#N/A	14840.4	18181.2	14561.7	17740.4
Q8CE90	Dual specificity mitogen-activated protein kinase kinase 7	Map2k7	535	59.3	9.11	#N/A	#N/A	#N/A	#N/A	#N/A	#N/A	#N/A	#N/A	#N/A	#N/A	174492.5	184820.3	169932.7	204073.7
Q9D083	Kinetochore protein Spc24	Spc24	201	23.4	4.64	#N/A	#N/A	#N/A	#N/A	#N/A	#N/A	#N/A	#N/A	#N/A	#N/A	33804.2	35629.6	38579.1	36642.8
Q8BMC3	SHC-transforming protein 2	Shc2	573	61.7	6.7	#N/A	#N/A	#N/A	#N/A	#N/A	#N/A	#N/A	#N/A	#N/A	#N/A	72152.2	70921.8	68944.9	79281.2
Q99JG3	Annexin A13	Anxa13	317	35.9	5.99	#N/A	#N/A	#N/A	#N/A	#N/A	#N/A	#N/A	#N/A	#N/A	#N/A	191806.2	179178.1	157789.2	200297.6
Q8JZS9	39S ribosomal protein L48, mitochondrial	Mrpl48	211	23.9	9.44	#N/A	#N/A	#N/A	#N/A	#N/A	#N/A	#N/A	#N/A	#N/A	#N/A	477266.2	535324.7	406914.5	457793.2
P61025	Cyclin-dependent kinases regulatory subunit 1	Cks1b	79	9.7	8.94	#N/A	#N/A	#N/A	#N/A	#N/A	#N/A	#N/A	#N/A	#N/A	#N/A	67109.5	100324.1	80718.2	73532.1
Q9D8T0	Protein FAM3A	Fam3a	230	25.4	8.44	#N/A	#N/A	#N/A	#N/A	#N/A	#N/A	#N/A	#N/A	#N/A	#N/A	76715.9	78115.7	73745.1	75971.1
O35684	Neuroserpin	Serpini1	410	46.3	4.72	#N/A	#N/A	#N/A	#N/A	#N/A	#N/A	#N/A	#N/A	#N/A	#N/A	6711.2	5867.8	5802.6	8118.2
Q8K201	Keratinocyte-associated transmembrane protein 2	Kct2	259	28	5.07	#N/A	#N/A	#N/A	#N/A	#N/A	#N/A	#N/A	#N/A	#N/A	#N/A	140966.0	167002.8	136779.5	164284.7
B1B0X6	Predicted gene 15114	Gm15114	433	51	9.88	#N/A	#N/A	#N/A	#N/A	#N/A	#N/A	#N/A	#N/A	#N/A	#N/A	253806.6	245125.0	227586.9	228804.6
Q91XD2	LIM and senescent cell antigen-like-containing domain protein 2	Lims2	341	39	7.97	#N/A	#N/A	#N/A	#N/A	#N/A	#N/A	#N/A	#N/A	#N/A	#N/A	863463.2	882735.5	828206.9	922641.3
Q5IBH7	Speedy protein A	Spdya	310	36.1	7.88	#N/A	#N/A	#N/A	#N/A	#N/A	#N/A	#N/A	#N/A	#N/A	#N/A	41608.0	47111.3	43578.9	42921.4
Q3U1J0	Sodium-coupled neutral amino acid transporter 5	Slc38a5	479	52.6	7.59	#N/A	#N/A	#N/A	#N/A	#N/A	#N/A	#N/A	#N/A	#N/A	#N/A	267656.3	256611.3	205578.8	205919.2
G5E8P0	Gamma-tubulin complex component 6	Tubgcp6	1769	197.1	5.95	#N/A	#N/A	#N/A	#N/A	#N/A	#N/A	#N/A	#N/A	#N/A	#N/A	189030.2	182650.1	161860.8	160280.4
Q8BQ89	LIX1-like protein	Lix1l	337	36.6	8.56	#N/A	#N/A	#N/A	#N/A	#N/A	#N/A	#N/A	#N/A	#N/A	#N/A	16854.2	15492.8	13007.2	16885.9
Q52KB6	C2 domain-containing protein 3	C2cd3	2323	255.2	6.95	#N/A	#N/A	#N/A	#N/A	#N/A	#N/A	#N/A	#N/A	#N/A	#N/A	23072.0	24072.5	24277.5	27857.9
A2ALC6	Predicted gene 4985	Gm4985	410	47.1	6.65	#N/A	#N/A	#N/A	#N/A	#N/A	#N/A	#N/A	#N/A	#N/A	#N/A	97783.7	97194.8	107087.1	105934.7
Q9ESX4	Nucleolar protein of 40 kDa	Zcchc17	241	27.5	9.63	#N/A	#N/A	#N/A	#N/A	#N/A	#N/A	#N/A	#N/A	#N/A	#N/A	165377.0	186639.1	163486.5	182558.3
Q9QYL7	Activator of basal transcription 1	Abt1	269	30.6	10.01	#N/A	#N/A	#N/A	#N/A	#N/A	#N/A	#N/A	#N/A	#N/A	#N/A	81814.4	81039.7	87373.1	78814.8
Q8K3W3	Protein CASC3	Casc3	698	75.7	6.44	#N/A	#N/A	#N/A	#N/A	#N/A	#N/A	#N/A	#N/A	#N/A	#N/A	182060.0	168017.0	182655.1	150666.4
O55187	E3 SUMO-protein ligase CBX4	Cbx4	551	60.5	9.33	#N/A	#N/A	#N/A	#N/A	#N/A	#N/A	#N/A	#N/A	#N/A	#N/A	10964.1	9708.6	8465.5	7513.2
Q9QWV9	Cyclin-T1	Ccnt1	724	80.5	8.68	#N/A	#N/A	#N/A	#N/A	#N/A	#N/A	#N/A	#N/A	#N/A	#N/A	184402.4	179602.5	185682.8	159613.7
P97333	Neuropilin-1	Nrp1	923	102.9	5.9	#N/A	#N/A	#N/A	#N/A	#N/A	#N/A	#N/A	#N/A	#N/A	#N/A	61995.1	70842.2	61742.1	66125.5
Q6P7W0	Sentrin-specific protease 6	Senp6	1132	126.9	6.74	#N/A	#N/A	#N/A	#N/A	#N/A	#N/A	#N/A	#N/A	#N/A	#N/A	116272.4	118976.8	129422.5	157246.2
Q8BNI4	Derlin-2	Derl2	239	27.6	7.28	#N/A	#N/A	#N/A	#N/A	#N/A	#N/A	#N/A	#N/A	#N/A	#N/A	68279.0	65253.5	57751.4	68946.5

Q61144	Presenilin-2	Psen2	448	50	4.59	#N/A	#N/A	#N/A	#N/A	#N/A	#N/A	#N/A	#N/A	#N/A	#N/A	117765.0	113799.0	103868.0	100497.7
Q9D099	Alkaline ceramidase 3	Acer3	267	31.5	9.01	#N/A	#N/A	#N/A	#N/A	#N/A	#N/A	#N/A	#N/A	#N/A	#N/A	45791.4	50035.5	43669.7	49603.5
Q9CQ28	Diphthine--ammonia ligase	Dph6	267	29.9	5.16	#N/A	#N/A	#N/A	#N/A	#N/A	#N/A	#N/A	#N/A	#N/A	#N/A	96459.7	94313.6	90805.5	97453.1
B8JKV0	Antagonist of mitotic exit network 1	Amn1	258	28.4	7.65	#N/A	#N/A	#N/A	#N/A	#N/A	#N/A	#N/A	#N/A	#N/A	#N/A	88120.1	98871.8	84167.6	94464.6
Q8BWG4	Uncharacterized protein KIAA1755 homolog	MGI:360657	1187	129	6.52	#N/A	#N/A	#N/A	#N/A	#N/A	#N/A	#N/A	#N/A	#N/A	#N/A	40297.7	40719.5	38800.3	38768.7
Q9D270	Probable palmitoyltransferase ZDHHC21	Zdhhc21	265	31.3	8.44	#N/A	#N/A	#N/A	#N/A	#N/A	#N/A	#N/A	#N/A	#N/A	#N/A	51203.7	70913.8	57720.5	67753.5
Q8C9S8	Cysteine protease ATG4A	Atg4a	396	45.1	4.96	#N/A	#N/A	#N/A	#N/A	#N/A	#N/A	#N/A	#N/A	#N/A	#N/A	166291.5	179756.7	156658.3	182859.3
Q9D7V2	LysM and putative peptidoglycan-binding domain-containing protein 2	Lysmd2	215	23.7	5.71	#N/A	#N/A	#N/A	#N/A	#N/A	#N/A	#N/A	#N/A	#N/A	#N/A	39020.7	44821.9	43020.0	36922.1
Q9D5J6	Sedoheptulokinase	Shpk	476	51.3	6.24	#N/A	#N/A	#N/A	#N/A	#N/A	#N/A	#N/A	#N/A	#N/A	#N/A	222674.9	202654.2	166119.9	193692.3
Q9D8N6	Protein lin-37 homolog	Lin37	246	28.4	9.01	#N/A	#N/A	#N/A	#N/A	#N/A	#N/A	#N/A	#N/A	#N/A	#N/A	159554.6	174430.0	174735.1	174515.9
O70201	Baculoviral IAP repeat-containing protein 5	Birc5	140	16.3	5.6	#N/A	#N/A	#N/A	#N/A	#N/A	#N/A	#N/A	#N/A	#N/A	#N/A	38007.4	35502.5	39388.7	33337.2
Q9D1P2	Histone acetyltransferase KAT8	Kat8	458	52.5	8.38	#N/A	#N/A	#N/A	#N/A	#N/A	#N/A	#N/A	#N/A	#N/A	#N/A	91074.0	103864.0	103633.4	91143.4
Q80WR1	Tetraspanin-18	Tspan18	248	27.8	5.21	#N/A	#N/A	#N/A	#N/A	#N/A	#N/A	#N/A	#N/A	#N/A	#N/A	68914.1	63156.3	51664.5	60010.4
A2AJQ3	Probable C-mannosyltransferase DPY19L4	Dpy19l4	722	83.5	8.59	#N/A	#N/A	#N/A	#N/A	#N/A	#N/A	#N/A	#N/A	#N/A	#N/A	29583.9	35657.3	34698.3	38664.4
Q3U276	Succinate dehydrogenase assembly factor 1, mitochondrial	Sdhaf1	118	13.1	11.24	#N/A	#N/A	#N/A	#N/A	#N/A	#N/A	#N/A	#N/A	#N/A	#N/A	9418.0	13393.2	12538.3	7133.6
Q8CC35	Synaptopodin	Synpo	929	99.5	9.42	#N/A	#N/A	#N/A	#N/A	#N/A	#N/A	#N/A	#N/A	#N/A	#N/A	38757.6	44077.5	43697.6	42867.4
Q6P6J9	Thioredoxin domain-containing protein 15	Txndc15	344	38.1	4.77	#N/A	#N/A	#N/A	#N/A	#N/A	#N/A	#N/A	#N/A	#N/A	#N/A	118588.7	125461.8	112439.2	131133.6
Q80VQ0	Aldehyde dehydrogenase family 3 member B1	Aldh3b1	468	52.3	7.55	#N/A	#N/A	#N/A	#N/A	#N/A	#N/A	#N/A	#N/A	#N/A	#N/A	246665.4	220556.5	202889.6	227070.2
A0A1Y7VK00	Zinc finger protein 825	Zfp825	292	34.2	7.69	#N/A	#N/A	#N/A	#N/A	#N/A	#N/A	#N/A	#N/A	#N/A	#N/A	47225.5	58163.6	46347.3	49658.8
Q8C6C7	Protein FAM204A	Fam204a	236	27	7.91	#N/A	#N/A	#N/A	#N/A	#N/A	#N/A	#N/A	#N/A	#N/A	#N/A	56537.1	58745.4	47995.4	59431.1
Q6NVG1	Lysophospholipid acyltransferase LPCAT4	Lpcat4	524	57.1	8.75	#N/A	#N/A	#N/A	#N/A	#N/A	#N/A	#N/A	#N/A	#N/A	#N/A	8953.2	12182.1	8949.5	8817.5
Q99LV7	Phosphatidylinositol-glycan biosynthesis class X protein	Pigx	254	28.6	6.62	#N/A	#N/A	#N/A	#N/A	#N/A	#N/A	#N/A	#N/A	#N/A	#N/A	128789.2	143053.3	123057.3	128731.4
Q9CQX5	Claudin domain-containing protein 1	Cldnd1	253	28.6	6.1	#N/A	#N/A	#N/A	#N/A	#N/A	#N/A	#N/A	#N/A	#N/A	#N/A	79277.9	68213.4	77206.3	68247.0
B7ZNG0	Kinesin-like protein KIF7	Kif7	1348	151.5	6.49	#N/A	#N/A	#N/A	#N/A	#N/A	#N/A	#N/A	#N/A	#N/A	#N/A	79918.3	90976.4	82901.7	85936.2
P00158	Cytochrome b	Mt-Cyb	381	43.2	7.97	#N/A	#N/A	#N/A	#N/A	#N/A	#N/A	#N/A	#N/A	#N/A	#N/A	430035.0	748618.5	478851.6	478587.7
Q9D2U5	N-alpha-acetyltransferase 38, NatC auxiliary subunit	Naa38	125	13.4	5.5	#N/A	#N/A	#N/A	#N/A	#N/A	#N/A	#N/A	#N/A	#N/A	#N/A	150186.9	152011.9	152333.5	149097.9
Q9D5U5	Pseudouridine-5'-phosphatase	Pudp	234	26.1	6.06	#N/A	#N/A	#N/A	#N/A	#N/A	#N/A	#N/A	#N/A	#N/A	#N/A	32299.9	35022.4	32635.6	37425.1
O09114	Prostaglandin-H2 D-isomerase	Ptgds	189	21.1	8.25	#N/A	#N/A	#N/A	#N/A	#N/A	#N/A	#N/A	#N/A	#N/A	#N/A	93302.1	105362.2	93264.2	149326.0
P58466	Carboxy-terminal domain RNA polymerase II polypeptide A small phosphatase 1	Ctdsp1	261	29.2	6.13	#N/A	#N/A	#N/A	#N/A	#N/A	#N/A	#N/A	#N/A	#N/A	#N/A	96915.5	106005.5	85122.7	88875.2
Q9QYK5	Heparan-sulfate 6-O-sulfotransferase 1	Hs6st1	411	48.3	8.7	#N/A	#N/A	#N/A	#N/A	#N/A	#N/A	#N/A	#N/A	#N/A	#N/A	48652.4	50475.8	50063.0	44576.0
Q64338	Calcium/calmodulin-dependent 3',5'-cyclic nucleotide phosphodiesterase 1C	Pde1c	706	80.2	9.06	#N/A	#N/A	#N/A	#N/A	#N/A	#N/A	#N/A	#N/A	#N/A	#N/A	89463.8	97820.2	109154.1	132585.3
Q9CWQ0	Diphthine methyl ester synthase	Dph5	281	31.2	5.29	#N/A	#N/A	#N/A	#N/A	#N/A	#N/A	#N/A	#N/A	#N/A	#N/A	77040.9	94108.7	80986.1	82056.8
Q6PAM0	5'-AMP-activated protein kinase subunit beta-2	Prkab2	271	30.2	6.46	#N/A	#N/A	#N/A	#N/A	#N/A	#N/A	#N/A	#N/A	#N/A	#N/A	86695.8	92653.6	86023.4	82859.6
Q9D1C1	Ubiquitin-conjugating enzyme E2 C	Ube2c	179	19.6	7.33	#N/A	#N/A	#N/A	#N/A	#N/A	#N/A	#N/A	#N/A	#N/A	#N/A	91337.2	87053.2	82908.5	86307.8
Q3USB7	Inactive phospholipase C-like protein 1	Plcl1	1096	122.6	5.64	#N/A	#N/A	#N/A	#N/A	#N/A	#N/A	#N/A	#N/A	#N/A	#N/A	63561.1	65701.2	64469.6	72710.9
Q9CR63	Cytochrome c oxidase assembly protein COX16 homolog, mitochondrial	Cox16	106	12.3	9.82	#N/A	#N/A	#N/A	#N/A	#N/A	#N/A	#N/A	#N/A	#N/A	#N/A	176946.2	185204.2	159523.4	174747.6
Q8K305	Kinetochore-associated protein NSL1 homolog	Nsl1	281	31.7	6.13	#N/A	#N/A	#N/A	#N/A	#N/A	#N/A	#N/A	#N/A	#N/A	#N/A	88885.6	80158.9	74467.3	85157.2
Q9EPQ7	StAR-related lipid transfer protein 5	Stard5	213	23.9	6.39	#N/A	#N/A	#N/A	#N/A	#N/A	#N/A	#N/A	#N/A	#N/A	#N/A	287127.0	259246.8	229409.9	261125.1
Q3UPY5	Beta-galactosidase-1-like protein 2	Glb1l2	636	72.2	7.93	#N/A	#N/A	#N/A	#N/A	#N/A	#N/A	#N/A	#N/A	#N/A	#N/A	14005.0	12630.7	13947.8	22158.8
P03930	ATP synthase protein 8	Mtatp8	67	7.8	9.88	#N/A	#N/A	#N/A	#N/A	#N/A	#N/A	#N/A	#N/A	#N/A	#N/A	88645.7	85598.8	92169.8	73580.5
J3QMK1	Shugoshin 2B	Sgo2b	1167	130.4	8.5	#N/A	#N/A	#N/A	#N/A	#N/A	#N/A	#N/A	#N/A	#N/A	#N/A	593511.0	607205.6	592771.2	631261.6
Q9QY30	Bile salt export pump	Abcb11	1321	146.7	7.66	#N/A	#N/A	#N/A	#N/A	#N/A	#N/A	#N/A	#N/A	#N/A	#N/A	117812.3	128395.2	109216.6	112469.9
Q8CDF7	piRNA biogenesis protein EXD1	Exd1	570	63.9	5.97	#N/A	#N/A	#N/A	#N/A	#N/A	#N/A	#N/A	#N/A	#N/A	#N/A	27202.4	41583.0	31807.5	34570.7
Q9CX13	Protein cornichon homolog 4	Cnih4	139	16.1	6.49	#N/A	#N/A	#N/A	#N/A	#N/A	#N/A	#N/A	#N/A	#N/A	#N/A	30977.2	23364.4	18101.3	25297.7
Q8BFX3	BTB/POZ domain-containing protein KCTD3	Kctd3	815	88.8	7.15	#N/A	#N/A	#N/A	#N/A	#N/A	#N/A	#N/A	#N/A	#N/A	#N/A	57675.6	59730.3	59986.1	53750.3
Q6ZPV2	Chromatin-remodeling ATPase INO80	Ino80	1559	176.4	9.36	#N/A	#N/A	#N/A	#N/A	#N/A	#N/A	#N/A	#N/A	#N/A	#N/A	142910.4	149421.2	148248.6	155338.7
P53690	Matrix metalloproteinase-14	Mmp14	582	65.9	8.03	#N/A	#N/A	#N/A	#N/A	#N/A	#N/A	#N/A	#N/A	#N/A	#N/A	58883.6	69120.0	61508.7	62972.6
O54956	DNA polymerase epsilon subunit 2	Pole2	527	59.3	6.25	#N/A	#N/A	#N/A	#N/A	#N/A	#N/A	#N/A	#N/A	#N/A	#N/A	76254.1	86472.7	78440.4	81422.8
P97772	Metabotropic glutamate receptor 1	Grm1	1199	133.1	6.86	#N/A	#N/A	#N/A	#N/A	#N/A	#N/A	#N/A	#N/A	#N/A	#N/A	26766.0	42113.0	21107.2	37218.4
Q9D6W8	BLOC-1-related complex subunit 6	Borcs6	360	38	5.34	#N/A	#N/A	#N/A	#N/A	#N/A	#N/A	#N/A	#N/A	#N/A	#N/A	26546.2	31973.1	22925.8	27163.7
Q8K0Y7	Protein PAXX	Paxx	205	22	5.66	#N/A	#N/A	#N/A	#N/A	#N/A	#N/A	#N/A	#N/A	#N/A	#N/A	56548.7	63316.0	44472.4	52730.3
Q8BGP5	Transmembrane protein 127	Tmem127	238	25.8	6.62	#N/A	#N/A	#N/A	#N/A	#N/A	#N/A	#N/A	#N/A	#N/A	#N/A	58837.2	75965.8	60759.3	66283.1
E9Q2M9	WD repeat- and FYVE domain-containing protein 4	Wdfy4	3183	354.1	6.34	#N/A	#N/A	#N/A	#N/A	#N/A	#N/A	#N/A	#N/A	#N/A	#N/A	98305.1	100992.6	101516.6	102171.3
Q8K2T4	Ubiquinol-cytochrome-c reductase complex assembly factor 3	Uqcc3	89	9.6	9.52	#N/A	#N/A	#N/A	#N/A	#N/A	#N/A	#N/A	#N/A	#N/A	#N/A	113267.4	136337.0	104063.7	116591.2
P18828	Syndecan-1	Sdc1	311	32.9	4.79	#N/A	#N/A	#N/A	#N/A	#N/A	#N/A	#N/A	#N/A	#N/A	#N/A	87140.2	83451.1	88706.0	73230.6
P70207	Plexin-A2	Plxna2	1894	211.4	6.54	#N/A	#N/A	#N/A	#N/A	#N/A	#N/A	#N/A	#N/A	#N/A	#N/A	65259.7	75938.8	73387.3	68760.6
Q8BHE1	Gem-associated protein 8	Gemin8	238	28.4	7.18	#N/A	#N/A	#N/A	#N/A	#N/A	#N/A	#N/A	#N/A	#N/A	#N/A	121153.9	113273.0	108192.8	112695.7
Q8BKF1	DNA-directed RNA polymerase, mitochondrial	Polrmt	1207	136.6	9.09	#N/A	#N/A	#N/A	#N/A	#N/A	#N/A	#N/A	#N/A	#N/A	#N/A	75708.1	81144.9	85415.2	71206.8
Q9QYM8	Centromere protein H	Cenph	241	28.1	5.54	#N/A	#N/A	#N/A	#N/A	#N/A	#N/A	#N/A	#N/A	#N/A	#N/A	73686.1	88941.4	87107.7	92465.0
Q6PCZ4	Melanoma-associated antigen E1	Magee1	918	101.6	6.67	#N/A	#N/A	#N/A	#N/A	#N/A	#N/A	#N/A	#N/A	#N/A	#N/A	209196.8	202256.6	175284.9	191051.3
P97872	Dimethylaniline monooxygenase [N-oxide-forming] 5	Fmo5	533	60	8.73	#N/A	#N/A	#N/A	#N/A	#N/A	#N/A	#N/A	#N/A	#N/A	#N/A	211060.6	228519.5	189256.8	218685.4
Q3U1C4	Ribosomal_L7Ae domain-containing protein	Secisbp2	858	94.8	8.6	#N/A	#N/A	#N/A	#N/A	#N/A	#N/A	#N/A	#N/A	#N/A	#N/A	174647.9	235159.8	163665.9	193336.1
P30355	Arachidonate 5-lipoxygenase-activating protein	Alox5ap	161	18.1	8.47	#N/A	#N/A	#N/A	#N/A	#N/A	#N/A	#N/A	#N/A	#N/A	#N/A	6926.4	5032.3	7407.2	7780.9
Q8BHJ6	Serine incorporator 5	Serinc5	461	51.8	8.07	#N/A	#N/A	#N/A	#N/A	#N/A	#N/A	#N/A	#N/A	#N/A	#N/A	112609.3	108220.3	105528.3	106863.2
Q8BGZ2	Protein FAM168A	Fam168a	244	26.2	8.98	#N/A	#N/A	#N/A	#N/A	#N/A	#N/A	#N/A	#N/A	#N/A	#N/A	72796.0	73878.8	83199.8	78870.4
Q61066	Nuclear receptor subfamily 0 group B member 1	Nr0b1	472	52.5	6.1	#N/A	#N/A	#N/A	#N/A	#N/A	#N/A	#N/A	#N/A	#N/A	#N/A	231434.2	219201.1	210077.3	207057.1
Q80YF9	Rho GTPase-activating protein 33	Arhgap33	1305	139.7	9.19	#N/A	#N/A	#N/A	#N/A	#N/A	#N/A	#N/A	#N/A	#N/A	#N/A	79247.0	75826.4	87078.0	72896.9
Q922Z1	Lipid droplet assembly factor 1	Tmem159	161	17.6	7.71	#N/A	#N/A	#N/A	#N/A	#N/A	#N/A	#N/A	#N/A	#N/A	#N/A	76003.1	83501.9	63197.4	72808.4
Q07802	Transcription factor COE1	Ebf1	591	64.4	8.76	#N/A	#N/A	#N/A	#N/A	#N/A	#N/A	#N/A	#N/A	#N/A	#N/A	255610.3	235532.6	247052.1	222483.8
P01646	Ig kappa chain V-V region HP 123E6	-	108	12	7.97	#N/A	#N/A	#N/A	#N/A	#N/A	#N/A	#N/A	#N/A	#N/A	#N/A	40141.1	49680.6	41747.4	55536.3
Q8VED8	Mitochondrial fission regulator 2	Mtfr2	361	40.7	6.47	#N/A	#N/A	#N/A	#N/A	#N/A	#N/A	#N/A	#N/A	#N/A	#N/A	25723.3	24012.5	23066.0	24695.3
P70210	Transcriptional enhancer factor TEF-5	Tead3	439	48.9	8.4	#N/A	#N/A	#N/A	#N/A	#N/A	#N/A	#N/A	#N/A	#N/A	#N/A	115099.2	154205.2	111245.5	124488.3
Q9Z0F4	Calcium and integrin-binding protein 1	Cib1	191	21.8	4.65	#N/A	#N/A	#N/A	#N/A	#N/A	#N/A	#N/A	#N/A	#N/A	#N/A	12707.0	10707.1	14990.0	14151.1
Q62120	Tyrosine-protein kinase JAK2	Jak2	1129	130.2	7.52	#N/A	#N/A	#N/A	#N/A	#N/A	#N/A	#N/A	#N/A	#N/A	#N/A	7882.9	5947.2	4721.5	6615.0
Q3UQI9	Probable ubiquitin carboxyl-terminal hydrolase MINDY-4	Mindy4	744	82.9	6.18	#N/A	#N/A	#N/A	#N/A	#N/A	#N/A	#N/A	#N/A	#N/A	#N/A	61267.0	68525.4	66096.4	84960.3
Q80WC1	Ubinuclein-2	Ubn2	1314	141.7	9.17	#N/A	#N/A	#N/A	#N/A	#N/A	#N/A	#N/A	#N/A	#N/A	#N/A	217588.4	198943.5	191470.3	179026.0
Q03401	Cysteine-rich secretory protein 1	Crisp1	244	27.7	6.87	#N/A	#N/A	#N/A	#N/A	#N/A	#N/A	#N/A	#N/A	#N/A	#N/A	43446.2	44812.8	42978.2	179623.4
Q9D882	Protein FAM241B	Fam241b	120	13.1	11.41	#N/A	#N/A	#N/A	#N/A	#N/A	#N/A	#N/A	#N/A	#N/A	#N/A	181079.3	148529.2	157714.5	160224.8
Q3UTZ3	Microtubule-associated protein 11	MAP11	580	62.7	8.68	#N/A	#N/A	#N/A	#N/A	#N/A	#N/A	#N/A	#N/A	#N/A	#N/A	88964.5	91237.6	87620.5	97930.7
Q69Z66	Histone H2A deubiquitinase MYSM1	Mysm1	819	93.4	5.29	#N/A	#N/A	#N/A	#N/A	#N/A	#N/A	#N/A	#N/A	#N/A	#N/A	23498.3	24739.9	22366.1	24199.0
Q8BTK5	SET and MYND domain-containing protein 4	Smyd4	799	88.5	6.77	#N/A	#N/A	#N/A	#N/A	#N/A	#N/A	#N/A	#N/A	#N/A	#N/A	19377.7	14640.4	12558.1	16202.4
Q6PFD6	Kinesin-like protein KIF18B	Kif18b	834	91.9	8.4	#N/A	#N/A	#N/A	#N/A	#N/A	#N/A	#N/A	#N/A	#N/A	#N/A	35657.1	37860.4	30925.2	32588.6
Q923X4	Glutaredoxin-2, mitochondrial	Glrx2	156	17.3	9.95	#N/A	#N/A	#N/A	#N/A	#N/A	#N/A	#N/A	#N/A	#N/A	#N/A	38253.6	47416.7	47249.4	40814.3
Q61062	Segment polarity protein dishevelled homolog DVL-3	Dvl3	716	78.1	6.65	#N/A	#N/A	#N/A	#N/A	#N/A	#N/A	#N/A	#N/A	#N/A	#N/A	50119.2	51706.3	55643.5	42772.7
Q91ZP3	Phosphatidate phosphatase LPIN1	Lpin1	924	101.9	6.74	#N/A	#N/A	#N/A	#N/A	#N/A	#N/A	#N/A	#N/A	#N/A	#N/A	11720.8	16808.3	14823.8	16282.5
P14142	Solute carrier family 2, facilitated glucose transporter member 4	Slc2a4	509	54.7	8.13	#N/A	#N/A	#N/A	#N/A	#N/A	#N/A	#N/A	#N/A	#N/A	#N/A	92488.5	115625.8	89391.8	98233.4
Q8K442	ATP-binding cassette sub-family A member 8-A	Abca8a	1620	184.1	7.28	#N/A	#N/A	#N/A	#N/A	#N/A	#N/A	#N/A	#N/A	#N/A	#N/A	188235.1	197575.2	182918.3	193255.2
E9QA22	Zinc finger protein 644	Zfp644	1323	148.3	8.27	#N/A	#N/A	#N/A	#N/A	#N/A	#N/A	#N/A	#N/A	#N/A	#N/A	108275.8	117253.6	111017.2	114713.7
Q3UHX9	Putative methyltransferase C9orf114 homolog	Spout1	385	42.9	8.03	#N/A	#N/A	#N/A	#N/A	#N/A	#N/A	#N/A	#N/A	#N/A	#N/A	265335.5	254390.1	252551.3	261787.6
Q3TUF7	YEATS domain-containing protein 2	Yeats2	1407	148.9	9.04	#N/A	#N/A	#N/A	#N/A	#N/A	#N/A	#N/A	#N/A	#N/A	#N/A	57401.3	56984.7	51049.5	53827.7
Q8K015	Centromere protein O	Cenpo	298	34.1	8.79	#N/A	#N/A	#N/A	#N/A	#N/A	#N/A	#N/A	#N/A	#N/A	#N/A	50577.5	48395.2	57620.1	55777.3
Q3V384	AFG1-like ATPase	Afg1l	480	54.3	8.03	#N/A	#N/A	#N/A	#N/A	#N/A	#N/A	#N/A	#N/A	#N/A	#N/A	423136.6	444257.2	418321.9	446959.0

Q923W1	Trimethylguanosine synthase	Tgs1	853	96.7	4.97	#N/A	#N/A	#N/A	#N/A	#N/A	#N/A	#N/A	#N/A	#N/A	#N/A	14031.9	13345.3	15109.1	11860.5
Q9DCI3	STARD3 N-terminal-like protein	Stard3nl	235	26.8	5.02	#N/A	#N/A	#N/A	#N/A	#N/A	#N/A	#N/A	#N/A	#N/A	#N/A	141631.6	147778.6	136368.1	143512.9
Q91YL3	Uridine-cytidine kinase-like 1	Uckl1	548	60.8	7.15	#N/A	#N/A	#N/A	#N/A	#N/A	#N/A	#N/A	#N/A	#N/A	#N/A	106028.7	111238.4	90009.8	99479.9
Q8C669	E3 ubiquitin-protein ligase pellino homolog 1	Peli1	418	46.2	8.03	#N/A	#N/A	#N/A	#N/A	#N/A	#N/A	#N/A	#N/A	#N/A	#N/A	194238.2	231156.1	222478.8	245482.0
Q9CQA6	Coiled-coil-helix-coiled-coil-helix domain-containing protein 1	Chchd1	118	13.6	10.15	#N/A	#N/A	#N/A	#N/A	#N/A	#N/A	#N/A	#N/A	#N/A	#N/A	105545.7	123682.4	118690.7	118212.9
O35683	NADH dehydrogenase [ubiquinone] 1 alpha subcomplex subunit 1	Ndufa1	70	8.1	9.48	#N/A	#N/A	#N/A	#N/A	#N/A	#N/A	#N/A	#N/A	#N/A	#N/A	146718.1	174059.1	187871.8	157970.2
D3YXG0	Hemicentin-1	Hmcn1	5634	611.2	6.64	#N/A	#N/A	#N/A	#N/A	#N/A	#N/A	#N/A	#N/A	#N/A	#N/A	420944.4	393872.1	399007.9	384208.1
Q9CRG1	Transmembrane 7 superfamily member 3	Tm7sf3	565	63.2	8.25	#N/A	#N/A	#N/A	#N/A	#N/A	#N/A	#N/A	#N/A	#N/A	#N/A	90917.1	87111.7	90362.1	86570.3
Q9DBW0	Cytochrome P450 4V2	Cyp4v2	525	60.9	8.47	#N/A	#N/A	#N/A	#N/A	#N/A	#N/A	#N/A	#N/A	#N/A	#N/A	19890.2	24289.8	18931.8	24030.2
Q9CYC6	m7GpppN-mRNA hydrolase	Dcp2	422	48.3	7.94	#N/A	#N/A	#N/A	#N/A	#N/A	#N/A	#N/A	#N/A	#N/A	#N/A	94387.6	96000.0	88799.3	90681.5
Q80U57	Regulating synaptic membrane exocytosis protein 3	Rims3	307	32.6	9.29	#N/A	#N/A	#N/A	#N/A	#N/A	#N/A	#N/A	#N/A	#N/A	#N/A	38578.9	38759.1	29489.5	39155.3
Q3UIR3	E3 ubiquitin-protein ligase DTX3L	Dtx3l	748	83	8.09	#N/A	#N/A	#N/A	#N/A	#N/A	#N/A	#N/A	#N/A	#N/A	#N/A	85194.6	97262.1	83907.6	96222.7
Q7TSH3	Zinc finger protein 516	Znf516	1157	124.7	8.51	#N/A	#N/A	#N/A	#N/A	#N/A	#N/A	#N/A	#N/A	#N/A	#N/A	49176.6	73367.8	59831.8	62741.9
P12961	Neuroendocrine protein 7B2	Scg5	212	23.9	5.81	#N/A	#N/A	#N/A	#N/A	#N/A	#N/A	#N/A	#N/A	#N/A	#N/A	65858.0	81808.1	66557.4	60083.1
Q03249	Galactose-1-phosphate uridylyltransferase	Galt	379	43.2	6.77	#N/A	#N/A	#N/A	#N/A	#N/A	#N/A	#N/A	#N/A	#N/A	#N/A	102703.8	110412.2	112852.8	99358.2
Q64729	TGF-beta receptor type-1	Tgfbr1	503	56.1	7.42	#N/A	#N/A	#N/A	#N/A	#N/A	#N/A	#N/A	#N/A	#N/A	#N/A	74852.4	101011.9	85588.7	83550.8
Q64374	Regucalcin	Rgn	299	33.4	5.34	#N/A	#N/A	#N/A	#N/A	#N/A	#N/A	#N/A	#N/A	#N/A	#N/A	86634.4	100531.1	92330.0	105637.7
Q6P068	ADP-ribosylation factor-like protein 5C	Arl5c	179	20	6	#N/A	#N/A	#N/A	#N/A	#N/A	#N/A	#N/A	#N/A	#N/A	#N/A	154210.9	156595.0	148606.4	147919.5
O70622	Reticulon-2	Rtn2	471	51.3	4.88	#N/A	#N/A	#N/A	#N/A	#N/A	#N/A	#N/A	#N/A	#N/A	#N/A	143458.6	149307.9	149726.7	141870.6
Q5RL51	Glutathione S-transferase C-terminal domain-containing protein	Gstcd	634	70.6	7.61	#N/A	#N/A	#N/A	#N/A	#N/A	#N/A	#N/A	#N/A	#N/A	#N/A	92790.6	95568.9	77407.7	90274.3
Q61495	Desmoglein-1-alpha	Dsg1a	1057	114.5	4.89	#N/A	#N/A	#N/A	#N/A	#N/A	#N/A	#N/A	#N/A	#N/A	#N/A	42452.7	46668.4	46607.1	42111.6
Q9CSP9	Tetratricopeptide repeat protein 14	Ttc14	761	86.7	9	#N/A	#N/A	#N/A	#N/A	#N/A	#N/A	#N/A	#N/A	#N/A	#N/A	156803.7	172855.6	167112.5	150719.3
Q7TS72	Inositol-trisphosphate 3-kinase C	Itpkc	678	74.4	5.36	#N/A	#N/A	#N/A	#N/A	#N/A	#N/A	#N/A	#N/A	#N/A	#N/A	58022.3	72786.6	51515.4	58349.8
P58196	Phospholipid scramblase 4	Plscr4	326	36.6	6.21	#N/A	#N/A	#N/A	#N/A	#N/A	#N/A	#N/A	#N/A	#N/A	#N/A	268977.9	260625.7	253052.7	240359.8
Q9CQT9	Respirasome Complex Assembly Factor 1	Rab5if	129	14.8	4.84	#N/A	#N/A	#N/A	#N/A	#N/A	#N/A	#N/A	#N/A	#N/A	#N/A	161807.1	186344.1	168282.3	184352.7
Q9CPW9	Methionine aminopeptidase 1D, mitochondrial	Metap1d	335	37.2	6.7	#N/A	#N/A	#N/A	#N/A	#N/A	#N/A	#N/A	#N/A	#N/A	#N/A	184683.6	178153.1	194732.7	182607.0
Q8BGH7	CDC42 small effector protein 2	Cdc42se2	84	9.2	8.35	#N/A	#N/A	#N/A	#N/A	#N/A	#N/A	#N/A	#N/A	#N/A	#N/A	82345.1	74599.9	79588.1	76304.5
Q14B71	Cell division cycle-associated protein 2	Cdca2	982	106.3	8.65	#N/A	#N/A	#N/A	#N/A	#N/A	#N/A	#N/A	#N/A	#N/A	#N/A	16873.4	21407.2	22165.5	22614.2
Q8CJ26	Death domain-containing membrane protein NRADD	Nradd	228	24.7	5.01	#N/A	#N/A	#N/A	#N/A	#N/A	#N/A	#N/A	#N/A	#N/A	#N/A	62740.8	76270.6	56489.9	71180.5
Q8BHA0	INO80 complex subunit C	Ino80c	191	20.4	10.02	#N/A	#N/A	#N/A	#N/A	#N/A	#N/A	#N/A	#N/A	#N/A	#N/A	123799.2	140880.4	116469.5	124413.7
Q9QXJ1	Amyloid-beta A4 precursor protein-binding family B member 1	Apbb1	710	77.3	5	#N/A	#N/A	#N/A	#N/A	#N/A	#N/A	#N/A	#N/A	#N/A	#N/A	64476.6	66957.4	61844.0	59505.4
Q8K1A0	rRNA N6-adenosine-methyltransferase METTL5	Mettl5	209	23.6	6.4	#N/A	#N/A	#N/A	#N/A	#N/A	#N/A	#N/A	#N/A	#N/A	#N/A	85625.8	83922.6	83073.6	75051.5
Q91X46	Rho guanine nucleotide exchange factor 3	Arhgef3	524	59.5	6.14	#N/A	#N/A	#N/A	#N/A	#N/A	#N/A	#N/A	#N/A	#N/A	#N/A	108993.1	108475.4	94929.7	104284.6
Q9CQC5	Cdc42 effector protein 3	Cdc42ep3	254	27.7	5.94	#N/A	#N/A	#N/A	#N/A	#N/A	#N/A	#N/A	#N/A	#N/A	#N/A	48675.2	66919.3	52769.7	54682.1
Q6PAL0	BEN domain-containing protein 3	Bend3	825	94.1	5.55	#N/A	#N/A	#N/A	#N/A	#N/A	#N/A	#N/A	#N/A	#N/A	#N/A	5993.6	4454.4	4027.7	4257.5
Q6V4S5	Protein sidekick-2	Sdk2	2176	239.8	7.36	#N/A	#N/A	#N/A	#N/A	#N/A	#N/A	#N/A	#N/A	#N/A	#N/A	71140.0	69244.7	71973.8	63244.0
Q6NZL6	Tonsoku-like protein	Tonsl	1363	151	5.97	#N/A	#N/A	#N/A	#N/A	#N/A	#N/A	#N/A	#N/A	#N/A	#N/A	203295.0	199616.9	184534.8	199715.1
Q62511	E3 ubiquitin-protein ligase ZFP91	Zfp91	572	63.4	7.49	#N/A	#N/A	#N/A	#N/A	#N/A	#N/A	#N/A	#N/A	#N/A	#N/A	136051.3	200215.9	151017.0	150943.8
Q3USZ8	Divergent protein kinase domain 2A	Dipk2a	430	49.4	8.63	#N/A	#N/A	#N/A	#N/A	#N/A	#N/A	#N/A	#N/A	#N/A	#N/A	162537.8	157744.1	160082.4	157821.8
Q8R173	Palmitoyltransferase ZDHHC3	Zdhhc3	299	34	8.09	#N/A	#N/A	#N/A	#N/A	#N/A	#N/A	#N/A	#N/A	#N/A	#N/A	85512.8	72536.1	84535.3	76626.1
Q62356	Follistatin-related protein 1	Fstl1	306	34.5	5.85	#N/A	#N/A	#N/A	#N/A	#N/A	#N/A	#N/A	#N/A	#N/A	#N/A	102834.3	98559.6	83896.8	80277.0
Q8BSM7	Large neutral amino acids transporter small subunit 3	Slc43a1	564	62.6	8.12	#N/A	#N/A	#N/A	#N/A	#N/A	#N/A	#N/A	#N/A	#N/A	#N/A	252310.0	235564.7	203991.3	254113.8
Q5FWH2	Putative E3 ubiquitin-protein ligase UNKL	Unkl	727	79.6	7.99	#N/A	#N/A	#N/A	#N/A	#N/A	#N/A	#N/A	#N/A	#N/A	#N/A	26498.1	30987.3	23955.4	32388.1
Q9D7J4	Cytochrome c oxidase assembly protein COX20, mitochondrial	Cox20	117	13.2	9.01	#N/A	#N/A	#N/A	#N/A	#N/A	#N/A	#N/A	#N/A	#N/A	#N/A	72066.6	49819.4	46849.3	73969.4
Q9CQF8	Ribosomal protein 63, mitochondrial	Mrpl57	102	11.9	10.15	#N/A	#N/A	#N/A	#N/A	#N/A	#N/A	#N/A	#N/A	#N/A	#N/A	510534.8	543967.9	499070.0	547276.0
Q8BGT6	MICAL-like protein 1	Micall1	870	94	6.68	#N/A	#N/A	#N/A	#N/A	#N/A	#N/A	#N/A	#N/A	#N/A	#N/A	346911.3	363437.7	305973.7	336094.3
Q8VHI7	Dynein regulatory complex subunit 2	Ccdc65	493	58.1	6.98	#N/A	#N/A	#N/A	#N/A	#N/A	#N/A	#N/A	#N/A	#N/A	#N/A	563941.9	720820.3	744526.2	1234885.6
P23298	Protein kinase C eta type	Prkch	683	77.9	7.68	#N/A	#N/A	#N/A	#N/A	#N/A	#N/A	#N/A	#N/A	#N/A	#N/A	247949.0	213945.9	215956.0	242750.8
Q9D850	Transmembrane protein 68	Tmem68	329	37.8	8.46	#N/A	#N/A	#N/A	#N/A	#N/A	#N/A	#N/A	#N/A	#N/A	#N/A	49405.6	42435.8	42849.2	43879.8
Q8C1Z7	Bardet-Biedl syndrome 4 protein homolog	Bbs4	520	58.2	8.1	#N/A	#N/A	#N/A	#N/A	#N/A	#N/A	#N/A	#N/A	#N/A	#N/A	86287.3	84540.4	95131.1	105273.9
Q9JLF6	Protein-glutamine gamma-glutamyltransferase K	Tgm1	815	89.8	6.51	#N/A	#N/A	#N/A	#N/A	#N/A	#N/A	#N/A	#N/A	#N/A	#N/A	148786.0	147713.1	142164.1	161821.3
Q9JHN8	Serine/threonine-protein kinase 19	Stk19	254	28.1	9.6	#N/A	#N/A	#N/A	#N/A	#N/A	#N/A	#N/A	#N/A	#N/A	#N/A	157555.2	165459.4	157491.1	150743.9
P58544	BTB/POZ domain-containing protein 1	Btbd1	488	53.2	6.1	#N/A	#N/A	#N/A	#N/A	#N/A	#N/A	#N/A	#N/A	#N/A	#N/A	61017.2	71189.4	63616.6	60083.2
Q8K2J7	RELT-like protein 1	Rell1	272	29.3	7.85	#N/A	#N/A	#N/A	#N/A	#N/A	#N/A	#N/A	#N/A	#N/A	#N/A	84227.7	76813.5	78654.3	74275.3
B1AVP0	Phosphatase and actin regulator	Phactr2	632	69.2	7.18	#N/A	#N/A	#N/A	#N/A	#N/A	#N/A	#N/A	#N/A	#N/A	#N/A	24659.7	23924.9	26381.7	27547.3
Q9R117	Non-receptor tyrosine-protein kinase TYK2	Tyk2	1184	133.2	7.12	#N/A	#N/A	#N/A	#N/A	#N/A	#N/A	#N/A	#N/A	#N/A	#N/A	250760.0	284025.2	242364.3	274639.9
Q9EQN9	Thiamine transporter 1	Slc19a2	498	55.6	7.18	#N/A	#N/A	#N/A	#N/A	#N/A	#N/A	#N/A	#N/A	#N/A	#N/A	16598.0	15063.6	13663.4	12384.5
Q9WUF3	CASP8-associated protein 2	Casp8ap2	1962	219	6.67	#N/A	#N/A	#N/A	#N/A	#N/A	#N/A	#N/A	#N/A	#N/A	#N/A	51851.5	58589.3	53295.4	61238.0
Q3U9F6	E3 ubiquitin-protein ligase RNF166	Rnf166	237	26	8.06	#N/A	#N/A	#N/A	#N/A	#N/A	#N/A	#N/A	#N/A	#N/A	#N/A	48711.8	58727.9	52276.4	52520.0
Q9D4R2	Alpha-1,3-mannosyl-glycoprotein 4-beta-N-acetylglucosaminyltransferase-like protein MGAT4D	Mgat4d	373	43.3	9.47	#N/A	#N/A	#N/A	#N/A	#N/A	#N/A	#N/A	#N/A	#N/A	#N/A	171630.7	244145.9	242457.6	282878.6
P97360	Transcription factor ETV6	Etv6	485	56.4	7.33	#N/A	#N/A	#N/A	#N/A	#N/A	#N/A	#N/A	#N/A	#N/A	#N/A	262994.9	261195.9	226161.0	248919.9
Q8C551	RAD51-associated protein 1	Rad51ap1	337	36.2	9.38	#N/A	#N/A	#N/A	#N/A	#N/A	#N/A	#N/A	#N/A	#N/A	#N/A	42568.2	54661.0	43646.0	47661.0
Q9CWV7	Zinc finger SWIM domain-containing protein 1	Zswim1	455	51.4	6.96	#N/A	#N/A	#N/A	#N/A	#N/A	#N/A	#N/A	#N/A	#N/A	#N/A	74429.3	97662.8	85066.5	106990.7
Q9R233	Tapasin	Tapbp	465	49.7	8.5	#N/A	#N/A	#N/A	#N/A	#N/A	#N/A	#N/A	#N/A	#N/A	#N/A	239963.3	271736.8	219328.7	253374.7
P50615	Protein BTG3	Btg3	252	29	8.73	#N/A	#N/A	#N/A	#N/A	#N/A	#N/A	#N/A	#N/A	#N/A	#N/A	219677.4	244645.7	230895.3	237929.1
Q8BVI5	Syntaxin-16	Stx16	326	37.1	5.86	#N/A	#N/A	#N/A	#N/A	#N/A	#N/A	#N/A	#N/A	#N/A	#N/A	178127.8	189816.5	174127.7	189909.9
Q0P5X1	Leucine-rich repeat and IQ domain-containing protein 1	Lrriq1	1673	191.7	5.66	#N/A	#N/A	#N/A	#N/A	#N/A	#N/A	#N/A	#N/A	#N/A	#N/A	17184.8	17909.7	17619.9	20321.0
Q9JKC7	AP-4 complex subunit mu-1	Ap4m1	449	49.5	6.64	#N/A	#N/A	#N/A	#N/A	#N/A	#N/A	#N/A	#N/A	#N/A	#N/A	224132.0	231585.8	238043.4	240760.7
Q9JL15	Galectin-8	Lgals8	316	36.1	8.95	#N/A	#N/A	#N/A	#N/A	#N/A	#N/A	#N/A	#N/A	#N/A	#N/A	385670.6	366360.6	388348.5	418223.5
Q9CUX1	52 kDa repressor of the inhibitor of the protein kinase	Thap12	758	87	5.92	#N/A	#N/A	#N/A	#N/A	#N/A	#N/A	#N/A	#N/A	#N/A	#N/A	42110.8	54645.0	46718.8	52145.7
E9Q7D5	Rho guanine nucleotide exchange factor 5	Arhgef5	1581	176.6	5.83	#N/A	#N/A	#N/A	#N/A	#N/A	#N/A	#N/A	#N/A	#N/A	#N/A	139426.2	149515.0	122973.1	129532.0
Q9CQP3	Coiled-coil-helix-coiled-coil-helix domain-containing protein 5	Chchd5	110	12.3	7.31	#N/A	#N/A	#N/A	#N/A	#N/A	#N/A	#N/A	#N/A	#N/A	#N/A	19252.6	24285.6	19936.1	17737.8
D3KU66	Acetylserotonin O-methyltransferase	Asmt	387	40.9	9.88	#N/A	#N/A	#N/A	#N/A	#N/A	#N/A	#N/A	#N/A	#N/A	#N/A	19419.2	19361.1	18626.3	16448.8
B1AQ75	Keratin, type I cuticular Ha6	Krt36	473	52.8	5.05	#N/A	#N/A	#N/A	#N/A	#N/A	#N/A	#N/A	#N/A	#N/A	#N/A	14570.3	19035.2	17422.3	17874.3
Q9QUK3	Protein CLN8	Cln8	288	33.1	8.82	#N/A	#N/A	#N/A	#N/A	#N/A	#N/A	#N/A	#N/A	#N/A	#N/A	30828.6	39649.8	30476.2	30656.3
Q8R023	Polycomb group RING finger protein 1	Pcgf1	259	30.3	8.91	#N/A	#N/A	#N/A	#N/A	#N/A	#N/A	#N/A	#N/A	#N/A	#N/A	57306.9	62639.2	60442.6	56368.0
O70405	Serine/threonine-protein kinase ULK1	Ulk1	1051	112.4	8.66	#N/A	#N/A	#N/A	#N/A	#N/A	#N/A	#N/A	#N/A	#N/A	#N/A	162202.7	178629.6	152003.2	168733.3
Q9Z238	G1/S-specific cyclin-E2	Ccne2	404	46.6	7.84	#N/A	#N/A	#N/A	#N/A	#N/A	#N/A	#N/A	#N/A	#N/A	#N/A	247362.8	279576.6	278170.0	259812.4
Q6S5L9	SHC-transforming protein 4	Shc4	626	68.5	8.46	#N/A	#N/A	#N/A	#N/A	#N/A	#N/A	#N/A	#N/A	#N/A	#N/A	77595.2	110231.3	101057.1	91801.9
P54818	Galactocerebrosidase	Galc	684	77.2	6.74	#N/A	#N/A	#N/A	#N/A	#N/A	#N/A	#N/A	#N/A	#N/A	#N/A	14607.6	14836.1	13259.6	13273.2
A2ABV5	Mediator of RNA polymerase II transcription subunit 14	Med14	1459	160.9	8.53	#N/A	#N/A	#N/A	#N/A	#N/A	#N/A	#N/A	#N/A	#N/A	#N/A	22972.4	19866.4	16198.1	21874.9
Q3U3I9	Zinc finger protein 865	Znf865	1058	111.6	9.13	#N/A	#N/A	#N/A	#N/A	#N/A	#N/A	#N/A	#N/A	#N/A	#N/A	80537.6	95334.8	74588.6	82060.2
Q8BPE4	Transmembrane protein 177	Tmem177	311	34	9.77	#N/A	#N/A	#N/A	#N/A	#N/A	#N/A	#N/A	#N/A	#N/A	#N/A	361733.4	409849.1	391408.1	403178.4
Q91ZR4	Serine/threonine-protein kinase Nek8	Nek8	698	75.2	7.68	#N/A	#N/A	#N/A	#N/A	#N/A	#N/A	#N/A	#N/A	#N/A	#N/A	21126.8	19364.8	16925.6	15727.7
E9PX31	UPAR/Ly6 domain-containing protein	Gml	176	20.1	7.02	#N/A	#N/A	#N/A	#N/A	#N/A	#N/A	#N/A	#N/A	#N/A	#N/A	399449.9	564129.0	565768.1	655846.9
Q3U6U5	Putative GTP-binding protein 6	Gtpbp6	514	56.4	9.19	#N/A	#N/A	#N/A	#N/A	#N/A	#N/A	#N/A	#N/A	#N/A	#N/A	125552.7	184975.8	122271.9	150495.3
O54929	WD repeat and SOCS box-containing protein 2	Wsb2	404	45.2	8.06	#N/A	#N/A	#N/A	#N/A	#N/A	#N/A	#N/A	#N/A	#N/A	#N/A	76788.1	105233.2	83247.4	80040.1
E9Q0T8	EF-hand domain-containing protein	Dnah7a	4024	460.6	6.2	#N/A	#N/A	#N/A	#N/A	#N/A	#N/A	#N/A	#N/A	#N/A	#N/A	53624.6	64585.1	62606.6	68587.7
Q8K2Q5	Coiled-coil-helix-coiled-coil-helix domain-containing protein 7	Chchd7	85	10.1	8.51	#N/A	#N/A	#N/A	#N/A	#N/A	#N/A	#N/A	#N/A	#N/A	#N/A	183725.7	182109.3	179599.9	181181.1
P97401	Secreted frizzled-related protein 3	Frzb	323	36	8.27	#N/A	#N/A	#N/A	#N/A	#N/A	#N/A	#N/A	#N/A	#N/A	#N/A	267738.0	295289.4	239095.2	262425.3
Q8BHE0	Proline-rich protein 11	Prr11	368	40.9	10.01	#N/A	#N/A	#N/A	#N/A	#N/A	#N/A	#N/A	#N/A	#N/A	#N/A	74715.9	113803.6	83921.8	96462.1
Q91VM3	WD repeat domain phosphoinositide-interacting protein 4	Wdr45	360	39.8	6.99	#N/A	#N/A	#N/A	#N/A	#N/A	#N/A	#N/A	#N/A	#N/A	#N/A	269037.8	238662.2	249154.3	264125.0

Q8BVH9	tRNA N(3)-methylcytidine methyltransferase METTL6	Mettl6	282	32.8	7.33	#N/A	#N/A	#N/A	#N/A	#N/A	#N/A	#N/A	#N/A	#N/A	#N/A	358699.0	280313.6	257321.5	232630.3
Q8VD12	Zinc finger protein 385A	Znf385a	386	40.4	9.86	#N/A	#N/A	#N/A	#N/A	#N/A	#N/A	#N/A	#N/A	#N/A	#N/A	247663.8	268275.1	245742.8	294112.2
Q9D1Q5	SERPIN domain-containing protein	Serpinb3b	387	44.5	5.72	#N/A	#N/A	#N/A	#N/A	#N/A	#N/A	#N/A	#N/A	#N/A	#N/A	62092.0	73254.8	57503.0	77111.8
B1ARW8	Uncharacterized protein C1orf122 homolog	MGI:191617	110	11.3	8.21	#N/A	#N/A	#N/A	#N/A	#N/A	#N/A	#N/A	#N/A	#N/A	#N/A	104909.3	100687.1	98312.5	100173.9
Q6PDG8	Vacuolar fusion protein MON1 homolog A	Mon1a	556	62.1	6.1	#N/A	#N/A	#N/A	#N/A	#N/A	#N/A	#N/A	#N/A	#N/A	#N/A	32945.3	32959.5	32452.9	32238.9
Q9ERL0	AHD domain-containing protein	Mllt1	547	60.7	8.7	#N/A	#N/A	#N/A	#N/A	#N/A	#N/A	#N/A	#N/A	#N/A	#N/A	81616.9	82417.9	77384.6	81997.8
A2TJV2	Paralemmin-3	Palm3	734	78.7	4.5	#N/A	#N/A	#N/A	#N/A	#N/A	#N/A	#N/A	#N/A	#N/A	#N/A	121909.6	111486.6	114243.7	102817.5
Q9ESD6	CKLF-like MARVEL transmembrane domain-containing protein 7	Cmtm7	167	18.1	7.74	#N/A	#N/A	#N/A	#N/A	#N/A	#N/A	#N/A	#N/A	#N/A	#N/A	223167.0	262114.5	208313.9	266638.7
Q9JHE4	Galactosylceramide sulfotransferase	Gal3st1	423	48.9	8.97	#N/A	#N/A	#N/A	#N/A	#N/A	#N/A	#N/A	#N/A	#N/A	#N/A	190930.9	183030.9	174715.0	187197.1
Q91YQ7	E3 ubiquitin-protein transferase RMND5B	Rmnd5b	393	44.4	6.57	#N/A	#N/A	#N/A	#N/A	#N/A	#N/A	#N/A	#N/A	#N/A	#N/A	179500.2	177886.7	176788.6	194876.8
E9Q0B6	Dynein, axonemal, heavy chain 6	Dnah6	4144	474.5	5.63	#N/A	#N/A	#N/A	#N/A	#N/A	#N/A	#N/A	#N/A	#N/A	#N/A	216284.8	322221.5	248679.9	398754.7
Q9D6H5	Probable palmitoyltransferase ZDHHC4	Zdhhc4	343	39.5	8.46	#N/A	#N/A	#N/A	#N/A	#N/A	#N/A	#N/A	#N/A	#N/A	#N/A	73517.5	87085.3	79971.8	70729.2
Q8CDL9	Coiled-coil domain-containing protein 87	Ccdc87	855	98.4	8.53	#N/A	#N/A	#N/A	#N/A	#N/A	#N/A	#N/A	#N/A	#N/A	#N/A	2560.4	5821.7	3728.7	5094.6
Q9CQ82	Centromere protein R	Itgb3bp	176	20	9.82	#N/A	#N/A	#N/A	#N/A	#N/A	#N/A	#N/A	#N/A	#N/A	#N/A	38394.5	43062.9	48931.5	53674.6
Q8VCX6	KICSTOR complex protein kaptin	Kptn	430	47.5	5	#N/A	#N/A	#N/A	#N/A	#N/A	#N/A	#N/A	#N/A	#N/A	#N/A	203981.1	306825.0	212144.1	240234.2
P11985	Tctex1 domain-containing protein 3	Tcte3	191	22.4	8.78	#N/A	#N/A	#N/A	#N/A	#N/A	#N/A	#N/A	#N/A	#N/A	#N/A	644230.3	729656.3	752874.8	984871.1
Q9ERI2	Ras-related protein Rab-27A	Rab27a	221	25	5.36	#N/A	#N/A	#N/A	#N/A	#N/A	#N/A	#N/A	#N/A	#N/A	#N/A	141164.2	134169.7	125024.8	146234.4
Q6NXL5	ArfGAP with coiled-coil, ankyrin repeat and PH domains 3	Acap3	833	92.7	6.02	#N/A	#N/A	#N/A	#N/A	#N/A	#N/A	#N/A	#N/A	#N/A	#N/A	105022.8	115714.4	114057.7	98955.4
Q9Z104	SWI/SNF-related matrix-associated actin-dependent regulator of chromatin subfamily E member 1-related	Hmg20b	317	35.8	9.29	#N/A	#N/A	#N/A	#N/A	#N/A	#N/A	#N/A	#N/A	#N/A	#N/A	2382.7	4698.7	1747.8	1375.5
P58659	Protein eva-1 homolog C	Eva1c	440	49.3	6.84	#N/A	#N/A	#N/A	#N/A	#N/A	#N/A	#N/A	#N/A	#N/A	#N/A	86928.4	99844.6	90293.0	101567.9
Q3UHK8	Trinucleotide repeat-containing gene 6A protein	Tnrc6a	1896	203	6.99	#N/A	#N/A	#N/A	#N/A	#N/A	#N/A	#N/A	#N/A	#N/A	#N/A	74288.5	81835.7	62518.9	70898.2
Q8BYU6	Torsin-1A-interacting protein 2	Tor1aip2	502	54.5	4.86	#N/A	#N/A	#N/A	#N/A	#N/A	#N/A	#N/A	#N/A	#N/A	#N/A	667027.4	606493.4	571155.1	590138.1
Q9CPV2	Anaphase-promoting complex subunit 16	Anapc16	110	11.7	4.97	#N/A	#N/A	#N/A	#N/A	#N/A	#N/A	#N/A	#N/A	#N/A	#N/A	101859.8	95148.6	74866.5	83531.8
Q9ERQ8	Carbonic anhydrase 7	Ca7	264	29.9	6.95	#N/A	#N/A	#N/A	#N/A	#N/A	#N/A	#N/A	#N/A	#N/A	#N/A	48482.4	60986.2	42761.2	58462.2
Q06194	Coagulation factor VIII	F8	2319	266	7.34	#N/A	#N/A	#N/A	#N/A	#N/A	#N/A	#N/A	#N/A	#N/A	#N/A	139109.3	164408.4	130362.0	148867.5
Q3UN90	LYR motif-containing protein 9	Lyrm9	78	9.3	8.91	#N/A	#N/A	#N/A	#N/A	#N/A	#N/A	#N/A	#N/A	#N/A	#N/A	50912.9	46006.8	41349.9	46029.4
O35680	28S ribosomal protein S12, mitochondrial	Mrps12	139	15.4	10.71	#N/A	#N/A	#N/A	#N/A	#N/A	#N/A	#N/A	#N/A	#N/A	#N/A	624996.3	610478.7	613258.0	563008.8
Q6PCN3	Tau-tubulin kinase 1	Ttbk1	1308	141.5	5.62	#N/A	#N/A	#N/A	#N/A	#N/A	#N/A	#N/A	#N/A	#N/A	#N/A	203145.3	186992.6	169467.8	187112.4
Q61456	Cyclin-A1	Ccna1	421	47.7	5.71	#N/A	#N/A	#N/A	#N/A	#N/A	#N/A	#N/A	#N/A	#N/A	#N/A	85066.4	103863.6	95311.2	100345.0
Q8BTI7	Serine/threonine-protein phosphatase 6 regulatory ankyrin repeat subunit C	Ankrd52	1076	115	6.54	#N/A	#N/A	#N/A	#N/A	#N/A	#N/A	#N/A	#N/A	#N/A	#N/A	182553.7	198164.7	171745.4	200477.4
Q9JJC6	RILP-like protein 1	Rilpl1	406	47.3	5.16	#N/A	#N/A	#N/A	#N/A	#N/A	#N/A	#N/A	#N/A	#N/A	#N/A	19096.5	27633.2	16983.3	25793.4
Q9CWY3	N-lysine methyltransferase SETD6	Setd6	473	52.9	4.86	#N/A	#N/A	#N/A	#N/A	#N/A	#N/A	#N/A	#N/A	#N/A	#N/A	47802.9	54097.0	43215.9	45959.7
O54864	Histone-lysine N-methyltransferase SUV39H1	Suv39h1	412	47.7	8	#N/A	#N/A	#N/A	#N/A	#N/A	#N/A	#N/A	#N/A	#N/A	#N/A	63081.9	66292.0	73256.6	55208.8
A6H6E2	Multimerin-2	Mmrn2	943	105.1	5.57	#N/A	#N/A	#N/A	#N/A	#N/A	#N/A	#N/A	#N/A	#N/A	#N/A	178424.7	195906.0	172527.8	176043.5
P82349	Beta-sarcoglycan	Sgcb	320	34.9	8.6	#N/A	#N/A	#N/A	#N/A	#N/A	#N/A	#N/A	#N/A	#N/A	#N/A	133112.5	144942.6	122785.5	137722.7
B1AWL2	Zinc finger protein 462	Znf462	2495	282.5	7.33	#N/A	#N/A	#N/A	#N/A	#N/A	#N/A	#N/A	#N/A	#N/A	#N/A	141635.1	135941.5	163438.8	124611.0
Q8CID0	Cysteine-rich protein 2-binding protein	Kat14	779	88.2	6.68	#N/A	#N/A	#N/A	#N/A	#N/A	#N/A	#N/A	#N/A	#N/A	#N/A	199512.7	244115.5	195765.6	212853.4
Q8CIK8	E3 ubiquitin-protein ligase RFWD3	Rfwd3	774	84.3	5.68	#N/A	#N/A	#N/A	#N/A	#N/A	#N/A	#N/A	#N/A	#N/A	#N/A	169983.2	188709.5	251445.3	189857.1
Q6P926	Spermatogenesis-associated protein 24	Spata24	205	23.8	8.22	#N/A	#N/A	#N/A	#N/A	#N/A	#N/A	#N/A	#N/A	#N/A	#N/A	44387.7	52005.6	54653.3	56972.8
P54763	Ephrin type-B receptor 2	Ephb2	986	109.8	5.71	#N/A	#N/A	#N/A	#N/A	#N/A	#N/A	#N/A	#N/A	#N/A	#N/A	5523.5	10997.8	4871.7	9041.8
O88824	Protein JTB	Jtb	146	16.3	8.19	#N/A	#N/A	#N/A	#N/A	#N/A	#N/A	#N/A	#N/A	#N/A	#N/A	7411.0	8156.1	9069.3	8251.3
Q9D6Y4	BLOC-1-related complex subunit 8	Borcs8	120	13.6	6.54	#N/A	#N/A	#N/A	#N/A	#N/A	#N/A	#N/A	#N/A	#N/A	#N/A	120249.3	126370.2	114455.5	122900.8
Q9QUQ5	Short transient receptor potential channel 4	Trpc4	974	111.5	7.77	#N/A	#N/A	#N/A	#N/A	#N/A	#N/A	#N/A	#N/A	#N/A	#N/A	308843.9	437872.8	358971.9	430746.0
Q5ND29	Rab-interacting lysosomal protein	Rilp	369	41.1	5.33	#N/A	#N/A	#N/A	#N/A	#N/A	#N/A	#N/A	#N/A	#N/A	#N/A	545990.6	605823.5	598726.1	587788.0
Q9CZI9	Apoptosis-enhancing nuclease	Aen	336	37.3	8.75	#N/A	#N/A	#N/A	#N/A	#N/A	#N/A	#N/A	#N/A	#N/A	#N/A	85594.3	91928.0	85245.0	80140.3
P20782	Acetylcholine receptor subunit epsilon	Chrne	493	54.9	5.05	#N/A	#N/A	#N/A	#N/A	#N/A	#N/A	#N/A	#N/A	#N/A	#N/A	855367.3	857590.0	757572.5	805285.1
O35127	Protein C10	Grcc10	126	13.2	5.14	#N/A	#N/A	#N/A	#N/A	#N/A	#N/A	#N/A	#N/A	#N/A	#N/A	25559.0	22089.8	24233.6	25328.0
Q921P9	Transcription elongation factor A protein-like 1	Tceal1	165	19.3	4.54	#N/A	#N/A	#N/A	#N/A	#N/A	#N/A	#N/A	#N/A	#N/A	#N/A	96124.4	86014.4	92230.4	85095.2
A0A0J9YTR2	RIKEN cDNA 0610012G03 gene	10012G03	95	10.8	12	#N/A	#N/A	#N/A	#N/A	#N/A	#N/A	#N/A	#N/A	#N/A	#N/A	72695.7	71642.5	57705.5	68073.9
Q8BHI7	Elongation of very long chain fatty acids protein 5	Elovl5	299	35.3	9.54	#N/A	#N/A	#N/A	#N/A	#N/A	#N/A	#N/A	#N/A	#N/A	#N/A	163651.7	175352.8	188216.7	182299.8
Q9JKS4	LIM domain-binding protein 3	Ldb3	723	76.4	7.75	#N/A	#N/A	#N/A	#N/A	#N/A	#N/A	#N/A	#N/A	#N/A	#N/A	16754.2	20211.4	21437.7	22706.7
Q62230	Sialoadhesin	Siglec1	1695	182.9	6.67	#N/A	#N/A	#N/A	#N/A	#N/A	#N/A	#N/A	#N/A	#N/A	#N/A	155503.4	187770.0	153725.8	175268.3
J3QPC3	cDNA sequence BC024978	BC024978	361	38.9	5.96	#N/A	#N/A	#N/A	#N/A	#N/A	#N/A	#N/A	#N/A	#N/A	#N/A	27776.9	33773.4	28582.2	27513.2
Q8CIW5	Twinkle protein, mitochondrial	Twnk	685	76.9	9.13	#N/A	#N/A	#N/A	#N/A	#N/A	#N/A	#N/A	#N/A	#N/A	#N/A	96260.1	108542.6	110709.2	106169.3
Q9JIY2	E3 ubiquitin-protein ligase Hakai	Cbll1	491	54.4	8.51	#N/A	#N/A	#N/A	#N/A	#N/A	#N/A	#N/A	#N/A	#N/A	#N/A	131393.5	150257.0	140997.4	141092.7
Q9D8V7	Signal peptidase complex catalytic subunit SEC11C	Sec11c	192	21.6	9.23	#N/A	#N/A	#N/A	#N/A	#N/A	#N/A	#N/A	#N/A	#N/A	#N/A	259392.9	307456.7	242128.7	276675.3
Q8C0D9	Centrosomal protein of 68 kDa	Cep68	733	78.7	5.48	#N/A	#N/A	#N/A	#N/A	#N/A	#N/A	#N/A	#N/A	#N/A	#N/A	42082.4	50997.2	39436.2	38238.8
E9PZ36	Polycystic kidney and hepatic disease 1	Pkhd1	4059	444.6	6.32	#N/A	#N/A	#N/A	#N/A	#N/A	#N/A	#N/A	#N/A	#N/A	#N/A	490491.0	633614.7	606920.4	690590.4
Q6XPS7	Beta_elim_lyase domain-containing protein	Tha1	400	43.5	7.2	#N/A	#N/A	#N/A	#N/A	#N/A	#N/A	#N/A	#N/A	#N/A	#N/A	102259.1	120221.6	84989.6	90920.7
T1NXB5	V-set and transmembrane domain-containing protein 4	Vstm4	319	35.9	9.89	#N/A	#N/A	#N/A	#N/A	#N/A	#N/A	#N/A	#N/A	#N/A	#N/A	85932.9	87314.9	81160.9	74669.0
Q8CJ67	Double-stranded RNA-binding protein Staufen homolog 2	Stau2	570	62.5	9.6	#N/A	#N/A	#N/A	#N/A	#N/A	#N/A	#N/A	#N/A	#N/A	#N/A	152162.7	163113.8	149864.0	150430.3
B1AVZ0	Uracil phosphoribosyltransferase homolog	Uprt	310	34.3	6.23	#N/A	#N/A	#N/A	#N/A	#N/A	#N/A	#N/A	#N/A	#N/A	#N/A	23230.8	23156.8	19379.6	25498.0
O70445	BRCA1-associated RING domain protein 1	Bard1	765	84.2	8.37	#N/A	#N/A	#N/A	#N/A	#N/A	#N/A	#N/A	#N/A	#N/A	#N/A	152010.4	157759.5	155859.1	149912.9
A0A1B0GT42	Predicted gene, 32742	Gm32742	1604	177	6.14	#N/A	#N/A	#N/A	#N/A	#N/A	#N/A	#N/A	#N/A	#N/A	#N/A	241657.8	266427.8	299380.2	314212.0
Q9D4D2	MAGE domain-containing protein	33402E13	429	48.6	4.2	#N/A	#N/A	#N/A	#N/A	#N/A	#N/A	#N/A	#N/A	#N/A	#N/A	256513.1	210720.9	208332.1	219069.2
Q61097	Kinase suppressor of Ras 1	Ksr1	873	96.7	8.54	#N/A	#N/A	#N/A	#N/A	#N/A	#N/A	#N/A	#N/A	#N/A	#N/A	527385.9	464794.7	513604.6	466054.3
Q792Z1	Peptidase S1 domain-containing protein	Try10	246	26.2	5.83	#N/A	#N/A	#N/A	#N/A	#N/A	#N/A	#N/A	#N/A	#N/A	#N/A	34157.7	38022.9	31049.2	31282.9
Q9CR55	UPF0547 protein C16orf87 homolog	MGI:191396	154	17.8	10.07	#N/A	#N/A	#N/A	#N/A	#N/A	#N/A	#N/A	#N/A	#N/A	#N/A	418115.4	489765.4	433311.1	443469.5
Q9D113	DNL-type zinc finger protein	Dnlz	177	19.4	9.48	#N/A	#N/A	#N/A	#N/A	#N/A	#N/A	#N/A	#N/A	#N/A	#N/A	216447.1	208688.9	215582.1	212290.9
I7HPW8	Myosin XVB	Myo15b	3033	332.8	7.99	#N/A	#N/A	#N/A	#N/A	#N/A	#N/A	#N/A	#N/A	#N/A	#N/A	363748.9	301910.1	224510.7	286871.3
Q8VEJ1	GPN-loop GTPase 2	Gpn2	310	34.5	5.1	#N/A	#N/A	#N/A	#N/A	#N/A	#N/A	#N/A	#N/A	#N/A	#N/A	213135.8	223639.3	238113.7	226249.4
Q9CWQ2	Zinc finger SWIM domain-containing protein 7	Zswim7	152	16.6	6.3	#N/A	#N/A	#N/A	#N/A	#N/A	#N/A	#N/A	#N/A	#N/A	#N/A	242720.5	219874.3	225674.0	227356.2
Q8K2R5	Zinc finger protein 668	Znf668	619	68.3	8.87	#N/A	#N/A	#N/A	#N/A	#N/A	#N/A	#N/A	#N/A	#N/A	#N/A	179793.4	195940.4	155273.0	183872.8
Q7TNR9	Rho guanine nucleotide exchange factor 4	Arhgef4	484	56.6	7.94	#N/A	#N/A	#N/A	#N/A	#N/A	#N/A	#N/A	#N/A	#N/A	#N/A	67937.5	61355.1	68658.9	55555.4
E9Q649	Beta-1,3-galactosyl-O-glycosyl-glycoprotein beta-1,6-N-acetylglucosaminyltransferase 4	Gcnt4	455	52.8	7.81	#N/A	#N/A	#N/A	#N/A	#N/A	#N/A	#N/A	#N/A	#N/A	#N/A	155827.6	150046.0	146029.0	143410.6
P52850	Bifunctional heparan sulfate N-deacetylase/N-sulfotransferase 2	Ndst2	883	101.1	8.7	#N/A	#N/A	#N/A	#N/A	#N/A	#N/A	#N/A	#N/A	#N/A	#N/A	78704.5	73866.9	68549.2	75402.4
Q99LJ5	CKLF-like MARVEL transmembrane domain-containing protein 3	Cmtm3	184	20.2	4.81	#N/A	#N/A	#N/A	#N/A	#N/A	#N/A	#N/A	#N/A	#N/A	#N/A	1278152.8	1136190.6	1190762.9	1130399.3
D3Z0K6	Round spermatid basic protein 1-like	Rsbn1l	821	91.7	8.95	#N/A	#N/A	#N/A	#N/A	#N/A	#N/A	#N/A	#N/A	#N/A	#N/A	166303.7	168100.2	171125.3	155028.9
Q8K119	HMG box domain-containing protein	Pms1	917	103.5	7.05	#N/A	#N/A	#N/A	#N/A	#N/A	#N/A	#N/A	#N/A	#N/A	#N/A	83974.9	93459.3	78604.9	80968.3
Q80TN5	Palmitoyltransferase ZDHHC17	Zdhhc17	632	72.6	7.39	#N/A	#N/A	#N/A	#N/A	#N/A	#N/A	#N/A	#N/A	#N/A	#N/A	141452.6	138457.4	140687.1	130012.8
Q8VHI6	Wiskott-Aldrich syndrome protein family member 3	Wasf3	501	55.2	6.37	#N/A	#N/A	#N/A	#N/A	#N/A	#N/A	#N/A	#N/A	#N/A	#N/A	10649.0	12325.2	14293.9	10333.4
Q3UJK4	GTP-binding protein 2	Gtpbp2	602	65.7	8.05	#N/A	#N/A	#N/A	#N/A	#N/A	#N/A	#N/A	#N/A	#N/A	#N/A	190559.9	200319.1	182078.8	195939.0
Q8VIK1	U11/U12 small nuclear ribonucleoprotein 25 kDa protein	Snrnp25	123	14.3	8.78	#N/A	#N/A	#N/A	#N/A	#N/A	#N/A	#N/A	#N/A	#N/A	#N/A	331799.6	370555.9	362148.6	347955.5
E9PVA0	Olfactory receptor	Olfr653	317	35.5	8.66	#N/A	#N/A	#N/A	#N/A	#N/A	#N/A	#N/A	#N/A	#N/A	#N/A	101024.0	95089.3	98012.9	93556.0
Q148W0	Phospholipid-transporting ATPase IC	Atp8b1	1251	143.7	7.36	#N/A	#N/A	#N/A	#N/A	#N/A	#N/A	#N/A	#N/A	#N/A	#N/A	240298.4	218194.5	244616.2	243912.7
Q04519	Sphingomyelin phosphodiesterase	Smpd1	627	69.9	7.2	#N/A	#N/A	#N/A	#N/A	#N/A	#N/A	#N/A	#N/A	#N/A	#N/A	197587.5	210799.9	161200.7	196977.0
Q791N7	DNA-directed RNA polymerase I subunit RPA12	Znrd1	123	13.6	5.27	#N/A	#N/A	#N/A	#N/A	#N/A	#N/A	#N/A	#N/A	#N/A	#N/A	213077.0	235784.6	252061.6	216132.4
Q8N9S3	Activator of 90 kDa heat shock protein ATPase homolog 2	Ahsa2	331	37.6	6.44	#N/A	#N/A	#N/A	#N/A	#N/A	#N/A	#N/A	#N/A	#N/A	#N/A	204583.8	216973.4	216530.1	226782.1
Q99KR6	E3 ubiquitin-protein ligase RNF34	Rnf34	376	42	4.84	#N/A	#N/A	#N/A	#N/A	#N/A	#N/A	#N/A	#N/A	#N/A	#N/A	146879.9	154667.8	192827.1	162524.3
Q9D3N8	Growth hormone-regulated TBC protein 1	Grtp1	359	40.6	8.47	#N/A	#N/A	#N/A	#N/A	#N/A	#N/A	#N/A	#N/A	#N/A	#N/A	135796.0	152444.5	118399.7	137182.3
Q99KU1	Dehydrodolichyl diphosphate synthase complex subunit Dhdds	Dhdds	333	38.5	8.31	#N/A	#N/A	#N/A	#N/A	#N/A	#N/A	#N/A	#N/A	#N/A	#N/A	57884.7	62299.3	61477.5	52231.8
Q9WU60	Attractin	Atrn	1428	158	7.27	#N/A	#N/A	#N/A	#N/A	#N/A	#N/A	#N/A	#N/A	#N/A	#N/A	416851.8	381842.9	235278.7	292098.0

Q9EQR4	VWFA domain-containing protein	Clca3a2	902	99.8	7.17	#N/A	#N/A	#N/A	#N/A	#N/A	#N/A	#N/A	#N/A	#N/A	#N/A	297702.7	332786.9	330403.1	309345.4
Q80YY7	Zinc finger protein 618	Znf618	953	104.8	6.96	#N/A	#N/A	#N/A	#N/A	#N/A	#N/A	#N/A	#N/A	#N/A	#N/A	17334.0	18723.6	13887.0	17267.7
Q8BNJ6	Neuropilin and tolloid-like protein 2	Neto2	525	59.3	6.77	#N/A	#N/A	#N/A	#N/A	#N/A	#N/A	#N/A	#N/A	#N/A	#N/A	114923.1	130084.3	110726.3	127057.1
E9PUD6	Zinc finger and SCAN domain-containing 18	Zscan18	809	91.7	4.35	#N/A	#N/A	#N/A	#N/A	#N/A	#N/A	#N/A	#N/A	#N/A	#N/A	143693.6	146674.2	137298.3	131989.8
Q8R087	Beta-1,4-galactosyltransferase 7	B4galt7	327	37.7	8.53	#N/A	#N/A	#N/A	#N/A	#N/A	#N/A	#N/A	#N/A	#N/A	#N/A	174554.0	191522.0	152051.5	201250.6
Q8CIG9	F-box/LRR-repeat protein 8	Fbxl8	374	41.1	7.18	#N/A	#N/A	#N/A	#N/A	#N/A	#N/A	#N/A	#N/A	#N/A	#N/A	77435.8	78122.0	81388.4	69864.1
Q641K1	Cytosolic carboxypeptidase 1	Agtpbp1	1218	137.1	6.52	#N/A	#N/A	#N/A	#N/A	#N/A	#N/A	#N/A	#N/A	#N/A	#N/A	1134956.7	1145522.3	1155350.0	1082059.0
P97411	Islet cell autoantigen 1	Ica1	478	54.3	5.8	#N/A	#N/A	#N/A	#N/A	#N/A	#N/A	#N/A	#N/A	#N/A	#N/A	283803.9	267884.3	244533.2	239567.5
Q8R5A0	N-lysine methyltransferase SMYD2	Smyd2	433	49.5	6.95	#N/A	#N/A	#N/A	#N/A	#N/A	#N/A	#N/A	#N/A	#N/A	#N/A	191427.8	188942.6	194867.5	193259.8
A0A571BF98	DH domain-containing protein	Lekr1	690	80.6	7.12	#N/A	#N/A	#N/A	#N/A	#N/A	#N/A	#N/A	#N/A	#N/A	#N/A	183707.8	196708.4	195785.3	196805.2
Q9D3A9	Protein tweety homolog 1	Ttyh1	450	49	5.03	#N/A	#N/A	#N/A	#N/A	#N/A	#N/A	#N/A	#N/A	#N/A	#N/A	380087.5	378398.2	308829.6	380699.5
Q61083	Mitogen-activated protein kinase kinase kinase 2	Map3k2	619	69.5	8.46	#N/A	#N/A	#N/A	#N/A	#N/A	#N/A	#N/A	#N/A	#N/A	#N/A	230607.0	247013.2	217801.1	230567.1
Q80T69	Lysine-specific demethylase 9	Rsbn1	795	89.2	8.65	#N/A	#N/A	#N/A	#N/A	#N/A	#N/A	#N/A	#N/A	#N/A	#N/A	160112.3	170196.3	181229.9	168377.5
Q91W61	F-box/LRR-repeat protein 15	Fbxl15	300	33.1	7.43	#N/A	#N/A	#N/A	#N/A	#N/A	#N/A	#N/A	#N/A	#N/A	#N/A	73892.4	73304.9	59198.5	60196.6
Q91ZD6	ELL-associated factor 2	Eaf2	262	29.2	5.1	#N/A	#N/A	#N/A	#N/A	#N/A	#N/A	#N/A	#N/A	#N/A	#N/A	275882.2	307880.5	274907.4	295789.4
Q3UPP8	Centrosomal protein of 63 kDa	Cep63	700	80.4	5.72	#N/A	#N/A	#N/A	#N/A	#N/A	#N/A	#N/A	#N/A	#N/A	#N/A	11320.4	11477.3	11440.6	13279.2
Q9D489	Spermatogenesis- and oogenesis-specific basic helix-loop-helix-containing protein 2	Sohlh2	467	51.3	9.14	#N/A	#N/A	#N/A	#N/A	#N/A	#N/A	#N/A	#N/A	#N/A	#N/A	23351.9	24140.9	26813.5	27256.2
P98199	Phospholipid-transporting ATPase ID	Atp8b2	1209	136.9	7.02	#N/A	#N/A	#N/A	#N/A	#N/A	#N/A	#N/A	#N/A	#N/A	#N/A	11084.0	8167.8	9531.6	8491.4
Q9DA19	Corepressor interacting with RBPJ 1	Cir1	450	51.8	9.92	#N/A	#N/A	#N/A	#N/A	#N/A	#N/A	#N/A	#N/A	#N/A	#N/A	115278.0	122819.5	115654.7	107090.9
J3QPY3	Fas apoptotic inhibitory molecule-like	Faiml	178	20	7.84	#N/A	#N/A	#N/A	#N/A	#N/A	#N/A	#N/A	#N/A	#N/A	#N/A	81558.2	79197.6	92750.1	84980.8
Q9QZZ4	Unconventional myosin-XV	Myo15a	3511	395.4	9.16	#N/A	#N/A	#N/A	#N/A	#N/A	#N/A	#N/A	#N/A	#N/A	#N/A	26780.9	22041.5	22144.3	25132.2
Q9JJX6	P2X purinoceptor 4	P2rx4	388	43.4	7.99	#N/A	#N/A	#N/A	#N/A	#N/A	#N/A	#N/A	#N/A	#N/A	#N/A	238986.2	252438.7	241523.9	261028.8
Q8K190	SAYSvFN domain-containing protein 1	Saysd1	188	20.7	8.6	#N/A	#N/A	#N/A	#N/A	#N/A	#N/A	#N/A	#N/A	#N/A	#N/A	580373.7	673082.0	586536.4	628268.3
Q5RKZ7	Molybdenum cofactor biosynthesis protein 1	Mocs1	636	69.8	9.14	#N/A	#N/A	#N/A	#N/A	#N/A	#N/A	#N/A	#N/A	#N/A	#N/A	193906.8	188892.5	173162.5	182650.7
Q9CQZ0	ORM1-like protein 2	Ormdl2	153	17.4	9.64	#N/A	#N/A	#N/A	#N/A	#N/A	#N/A	#N/A	#N/A	#N/A	#N/A	13942.0	12985.3	13167.8	15044.1
P60060	Protein transport protein Sec61 subunit gamma	Sec61g	68	7.7	9.99	#N/A	#N/A	#N/A	#N/A	#N/A	#N/A	#N/A	#N/A	#N/A	#N/A	266040.5	268062.2	267887.7	256207.8
P11680	Properdin	Cfp	464	50.3	7.84	#N/A	#N/A	#N/A	#N/A	#N/A	#N/A	#N/A	#N/A	#N/A	#N/A	8595.2	6851.1	8749.7	8476.8
Q3UES3	Poly [ADP-ribose] polymerase tankyrase-2	Tnks2	1166	126.7	7.14	#N/A	#N/A	#N/A	#N/A	#N/A	#N/A	#N/A	#N/A	#N/A	#N/A	47975.4	51737.1	52515.5	41913.7
Q6RI63	Constitutive coactivator of peroxisome proliferator-activated receptor gamma	Fam120b	786	89.3	6.52	#N/A	#N/A	#N/A	#N/A	#N/A	#N/A	#N/A	#N/A	#N/A	#N/A	26026.8	41085.8	25539.9	33572.6
O54790	Transcription factor MafG	Mafg	162	17.9	10.04	#N/A	#N/A	#N/A	#N/A	#N/A	#N/A	#N/A	#N/A	#N/A	#N/A	36283.7	46415.6	45487.2	47216.7
Q922Y2	Tripartite motif-containing protein 59	Trim59	403	47.2	6.52	#N/A	#N/A	#N/A	#N/A	#N/A	#N/A	#N/A	#N/A	#N/A	#N/A	33617.3	39098.3	29032.6	26736.2
A0A075B5S5	Ig-like domain-containing protein	Ighv7-4	120	13.4	8.9	#N/A	#N/A	#N/A	#N/A	#N/A	#N/A	#N/A	#N/A	#N/A	#N/A	121709.9	165667.5	126580.5	150933.5
Q3V2K7	RanBD1 domain-containing protein	'00123L14I	440	48.1	8.38	#N/A	#N/A	#N/A	#N/A	#N/A	#N/A	#N/A	#N/A	#N/A	#N/A	52590.8	88707.7	63315.7	85969.2
Q3ULL6	Smg4_UPF3 domain-containing protein	Upf3b	472	57	9.44	#N/A	#N/A	#N/A	#N/A	#N/A	#N/A	#N/A	#N/A	#N/A	#N/A	88977.9	88377.0	82401.9	85868.9
P56671	Myc-associated zinc finger protein	Maz	477	48.7	8.95	#N/A	#N/A	#N/A	#N/A	#N/A	#N/A	#N/A	#N/A	#N/A	#N/A	683737.1	597699.2	628179.3	581289.6
Q8VDF3	Death-associated protein kinase 2	Dapk2	370	42.8	7.28	#N/A	#N/A	#N/A	#N/A	#N/A	#N/A	#N/A	#N/A	#N/A	#N/A	752706.5	744750.5	777296.1	703490.4
Q5SW45	Meckel syndrome type 1 protein homolog	Mks1	561	64.4	6.25	#N/A	#N/A	#N/A	#N/A	#N/A	#N/A	#N/A	#N/A	#N/A	#N/A	24534.2	21589.3	22630.8	26972.6
A2AJW5	Family with sequence similarity 217, member B	Fam217b	387	42.1	9.45	#N/A	#N/A	#N/A	#N/A	#N/A	#N/A	#N/A	#N/A	#N/A	#N/A	43128.7	39491.9	34311.0	38081.2
Q8CFP6	DnaJ homolog subfamily C member 27	Dnajc27	273	30.8	8.47	#N/A	#N/A	#N/A	#N/A	#N/A	#N/A	#N/A	#N/A	#N/A	#N/A	20381.7	26490.8	25476.2	25319.3
A5H0M4	ANK_REP_REGION domain-containing protein	Poteg	473	53.4	6.8	#N/A	#N/A	#N/A	#N/A	#N/A	#N/A	#N/A	#N/A	#N/A	#N/A	35590.8	37951.6	34922.4	44546.2
A2AM05	Centlein	Cntln	1397	160.6	7.99	#N/A	#N/A	#N/A	#N/A	#N/A	#N/A	#N/A	#N/A	#N/A	#N/A	821185.6	710513.3	527840.4	687035.2
Q5DTW7	Retroelement silencing factor 1	Resf1	1521	168.9	7.44	#N/A	#N/A	#N/A	#N/A	#N/A	#N/A	#N/A	#N/A	#N/A	#N/A	39573.0	35720.6	33303.4	35538.6
Q8CG80	SH2 domain-containing adapter protein F	Shf	238	26.9	5.69	#N/A	#N/A	#N/A	#N/A	#N/A	#N/A	#N/A	#N/A	#N/A	#N/A	69805.3	80096.2	84088.7	69838.8
Q6B966	NACHT, LRR and PYD domains-containing protein 14	Nlrp14	993	113.3	6.49	#N/A	#N/A	#N/A	#N/A	#N/A	#N/A	#N/A	#N/A	#N/A	#N/A	37118.1	29709.8	34374.9	29060.2
Q61488	Desert hedgehog protein	Dhh	396	43.5	9.39	#N/A	#N/A	#N/A	#N/A	#N/A	#N/A	#N/A	#N/A	#N/A	#N/A	41185.3	40088.1	42050.5	40107.9
Q32NZ6	Transmembrane channel-like protein 5	Tmc5	967	111	8.28	#N/A	#N/A	#N/A	#N/A	#N/A	#N/A	#N/A	#N/A	#N/A	#N/A	240726.0	297906.6	311579.2	244769.9
Q9R157	Disintegrin and metalloproteinase domain-containing protein 18	Adam18	719	79.2	6.18	#N/A	#N/A	#N/A	#N/A	#N/A	#N/A	#N/A	#N/A	#N/A	#N/A	6924.9	9755.8	12962.9	10796.7
P49891	Sulfotransferase 1E1	Sult1e1	295	35.6	7.01	#N/A	#N/A	#N/A	#N/A	#N/A	#N/A	#N/A	#N/A	#N/A	#N/A	190329.6	200102.8	146885.9	201319.8
Q99KN1	Arrestin domain-containing protein 1	Arrdc1	434	46.3	7.21	#N/A	#N/A	#N/A	#N/A	#N/A	#N/A	#N/A	#N/A	#N/A	#N/A	227061.1	315258.2	236982.1	271398.2
Q8BJK1	BTB/POZ domain-containing protein KCTD7	Kctd7	289	33.1	6.07	#N/A	#N/A	#N/A	#N/A	#N/A	#N/A	#N/A	#N/A	#N/A	#N/A	148493.5	122910.2	144613.2	139103.7
P0DMN7	S-adenosylmethionine decarboxylase proenzyme 1	Amd1	334	38.2	6	#N/A	#N/A	#N/A	#N/A	#N/A	#N/A	#N/A	#N/A	#N/A	#N/A	205004.6	212403.4	184584.7	198261.6
Q99J31	Oligophrenin-1	Ophn1	802	91.9	7.96	#N/A	#N/A	#N/A	#N/A	#N/A	#N/A	#N/A	#N/A	#N/A	#N/A	67279.0	63308.6	69476.9	69779.0
Q8BZN4	NUAK family SNF1-like kinase 2	Nuak2	639	70.6	9.06	#N/A	#N/A	#N/A	#N/A	#N/A	#N/A	#N/A	#N/A	#N/A	#N/A	44020.0	47482.2	59092.8	44579.7
Q499E6	Uncharacterized protein C1orf109 homolog	C1orf109	217	24.4	5.01	#N/A	#N/A	#N/A	#N/A	#N/A	#N/A	#N/A	#N/A	#N/A	#N/A	187976.7	231382.0	189281.9	199164.1
D3Z5G0	Pyrin domain-containing protein	Ifi213	586	63.4	8.1	#N/A	#N/A	#N/A	#N/A	#N/A	#N/A	#N/A	#N/A	#N/A	#N/A	13044.2	16450.7	14764.7	9816.0
Q8BL06	Inactive ubiquitin carboxyl-terminal hydrolase 54	Usp54	1588	176.6	7.62	#N/A	#N/A	#N/A	#N/A	#N/A	#N/A	#N/A	#N/A	#N/A	#N/A	111374.0	138024.0	95366.1	121891.2
Q8BZ60	Stonin-2	Ston2	895	99.5	5.26	#N/A	#N/A	#N/A	#N/A	#N/A	#N/A	#N/A	#N/A	#N/A	#N/A	6525.8	7059.6	5345.1	8774.5
O70571	[Pyruvate dehydrogenase (acetyl-transferring)] kinase isozyme 4, mitochondrial	Pdk4	412	46.6	7.08	#N/A	#N/A	#N/A	#N/A	#N/A	#N/A	#N/A	#N/A	#N/A	#N/A	245019.1	288951.8	265629.6	297169.2
E9Q5A6	Nuclear receptor subfamily 1, group H, member 5	Nr1h5	505	57.1	6.89	#N/A	#N/A	#N/A	#N/A	#N/A	#N/A	#N/A	#N/A	#N/A	#N/A	885359.3	823818.7	780881.8	736736.7
Q6P1E7	DNA-directed primase/polymerase protein	Primpol	537	61.3	6.05	#N/A	#N/A	#N/A	#N/A	#N/A	#N/A	#N/A	#N/A	#N/A	#N/A	34736.5	24712.7	26281.8	32939.5
Q9ERK0	Receptor-interacting serine/threonine-protein kinase 4	Ripk4	786	86.6	7.01	#N/A	#N/A	#N/A	#N/A	#N/A	#N/A	#N/A	#N/A	#N/A	#N/A	268914.5	273582.3	238169.3	252308.9
P35951	Low-density lipoprotein receptor	Ldlr	862	94.9	5.02	#N/A	#N/A	#N/A	#N/A	#N/A	#N/A	#N/A	#N/A	#N/A	#N/A	15387.0	16689.3	14136.7	16977.4
Q91VA3	Calpain-8	Capn8	703	79.3	5.62	#N/A	#N/A	#N/A	#N/A	#N/A	#N/A	#N/A	#N/A	#N/A	#N/A	56735.6	62956.5	56921.0	60172.4
Q3UI66	Coiled-coil domain-containing protein 34	Ccdc34	367	42.2	8.79	#N/A	#N/A	#N/A	#N/A	#N/A	#N/A	#N/A	#N/A	#N/A	#N/A	554314.2	628494.8	501124.5	519905.1
O35474	EGF-like repeat and discoidin I-like domain-containing protein 3	Edil3	480	53.7	7.58	#N/A	#N/A	#N/A	#N/A	#N/A	#N/A	#N/A	#N/A	#N/A	#N/A	40036.9	82627.9	35753.7	56502.7
Q8BI22	Centrosomal protein of 128 kDa	Cep128	1102	128.4	6.52	#N/A	#N/A	#N/A	#N/A	#N/A	#N/A	#N/A	#N/A	#N/A	#N/A	91048.4	85073.2	79969.6	78933.5
P09240	Cholecystokinin	Cck	115	12.8	9.35	#N/A	#N/A	#N/A	#N/A	#N/A	#N/A	#N/A	#N/A	#N/A	#N/A	342914.4	356586.9	326392.4	328859.2
Q3URQ7	Methenyltetrahydrofolate synthase domain-containing protein	Mthfsd	372	40.8	9.69	#N/A	#N/A	#N/A	#N/A	#N/A	#N/A	#N/A	#N/A	#N/A	#N/A	98606.1	102815.5	97841.3	88499.3
P55849	Desmocollin-1	Dsc1	886	98.9	5.47	#N/A	#N/A	#N/A	#N/A	#N/A	#N/A	#N/A	#N/A	#N/A	#N/A	303413.1	347376.7	266493.5	332014.8
Q3URK1	Coiled-coil domain-containing protein 190	Ccdc190	288	32.6	9.14	#N/A	#N/A	#N/A	#N/A	#N/A	#N/A	#N/A	#N/A	#N/A	#N/A	557725.3	482398.9	469178.2	477243.7
Q99KE3	Docking protein 4	Dok4	325	37	7.93	#N/A	#N/A	#N/A	#N/A	#N/A	#N/A	#N/A	#N/A	#N/A	#N/A	46847.0	54625.5	38736.3	42028.3
Q9CQG1	Putative glutathione-specific gamma-glutamylcyclotransferase 2	Chac2	178	20.1	5.68	#N/A	#N/A	#N/A	#N/A	#N/A	#N/A	#N/A	#N/A	#N/A	#N/A	113474.1	129406.0	118432.2	133086.2
O54824	Pro-interleukin-16	Il16	1322	141.3	7.78	#N/A	#N/A	#N/A	#N/A	#N/A	#N/A	#N/A	#N/A	#N/A	#N/A	172646.0	193746.7	162424.3	168934.9
Q8C5T8	Coiled-coil domain-containing protein 113	Ccdc113	377	44.2	8.46	#N/A	#N/A	#N/A	#N/A	#N/A	#N/A	#N/A	#N/A	#N/A	#N/A	71476.9	79427.4	83268.2	83182.3
Q9Z1K8	Y+L amino acid transporter 1	Slc7a7	510	55.6	5.74	#N/A	#N/A	#N/A	#N/A	#N/A	#N/A	#N/A	#N/A	#N/A	#N/A	271206.8	291066.3	245182.9	266830.1
Q32M21	Gasdermin-A2	Gsdma2	443	49.8	5.9	#N/A	#N/A	#N/A	#N/A	#N/A	#N/A	#N/A	#N/A	#N/A	#N/A	27576.8	37505.3	34163.8	36056.3
P28862	Stromelysin-1	Mmp3	477	53.8	6.13	#N/A	#N/A	#N/A	#N/A	#N/A	#N/A	#N/A	#N/A	#N/A	#N/A	536089.2	488197.3	390082.8	500941.4
Q2PZL6	Protocadherin Fat 4	Fat4	4981	540	4.91	#N/A	#N/A	#N/A	#N/A	#N/A	#N/A	#N/A	#N/A	#N/A	#N/A	529788.8	453010.3	455588.9	611447.9
Q64287	Interferon regulatory factor 4	Irf4	450	51.5	6.99	#N/A	#N/A	#N/A	#N/A	#N/A	#N/A	#N/A	#N/A	#N/A	#N/A	174951.8	165796.9	179381.3	177925.6
Q70KF4	Cardiomyopathy-associated protein 5	Cmya5	3739	412.8	4.75	#N/A	#N/A	#N/A	#N/A	#N/A	#N/A	#N/A	#N/A	#N/A	#N/A	432545.1	404834.7	495001.9	355253.2
A2RT91	Ankyrin and armadillo repeat-containing protein	Ankar	1465	165.2	7.68	#N/A	#N/A	#N/A	#N/A	#N/A	#N/A	#N/A	#N/A	#N/A	#N/A	282618.1	604512.8	222222.9	331482.4
Q9WVH6	Angiopoietin-4	Angpt4	509	57.8	9.04	#N/A	#N/A	#N/A	#N/A	#N/A	#N/A	#N/A	#N/A	#N/A	#N/A	65064.6	56946.1	53560.8	60793.6
O35902	Desmoglein-3	Dsg3	993	107.8	4.94	#N/A	#N/A	#N/A	#N/A	#N/A	#N/A	#N/A	#N/A	#N/A	#N/A	14616.7	15259.6	28721.1	15267.1
P98063	Bone morphogenetic protein 1	Bmp1	991	111.6	6.95	#N/A	#N/A	#N/A	#N/A	#N/A	#N/A	#N/A	#N/A	#N/A	#N/A	71831.9	87853.7	57958.0	75792.7
Q8CGY6	Protein unc-45 homolog B	Unc45b	931	103.6	7.97	#N/A	#N/A	#N/A	#N/A	#N/A	#N/A	#N/A	#N/A	#N/A	#N/A	27134.2	27238.2	23533.4	29078.5
Q9Z2G0	Protein fem-1 homolog B	Fem1b	627	70.2	6.61	#N/A	#N/A	#N/A	#N/A	#N/A	#N/A	#N/A	#N/A	#N/A	#N/A	445352.8	468407.2	456916.2	471255.8
F8VPT3	Lactase	Lct	1931	217.7	6.74	#N/A	#N/A	#N/A	#N/A	#N/A	#N/A	#N/A	#N/A	#N/A	#N/A	107693.6	105122.9	81924.7	120451.4
Q9CZD0	Methylmalonic aciduria and homocystinuria type C protein homolog	Mmachc	279	31.6	7.55	#N/A	#N/A	#N/A	#N/A	#N/A	#N/A	#N/A	#N/A	#N/A	#N/A	116184.3	147935.3	131405.0	148835.0
Q9D413	SH2 domain-containing protein 6	Sh2d6	297	32	9.92	#N/A	#N/A	#N/A	#N/A	#N/A	#N/A	#N/A	#N/A	#N/A	#N/A	435407.3	420606.4	519957.0	518047.8
Q8C636	Spermatogenesis-associated protein 16	Spata16	571	65	9.1	#N/A	#N/A	#N/A	#N/A	#N/A	#N/A	#N/A	#N/A	#N/A	#N/A	58212.9	65597.8	64607.2	45911.9
Q80TM6	R3H domain-containing protein 2	R3hdm2	1044	114.5	8.91	#N/A	#N/A	#N/A	#N/A	#N/A	#N/A	#N/A	#N/A	#N/A	#N/A	16299.4	21052.4	16113.0	18699.6
E9Q3H6	Polyketide_cyc domain-containing protein	Coq10a	259	28.9	9.82	#N/A	#N/A	#N/A	#N/A	#N/A	#N/A	#N/A	#N/A	#N/A	#N/A	76658.5	81348.6	81144.2	75477.8

A0A571BF02	Spectrin beta, non-erythrocytic 5	Sptbn5	3624	415.1	6.35	#N/A	#N/A	#N/A	#N/A	#N/A	#N/A	#N/A	#N/A	#N/A	#N/A	985595.5	851839.7	938108.3	766556.9
Q61614	Endothelin-1 receptor	Ednra	427	48.5	8.31	#N/A	#N/A	#N/A	#N/A	#N/A	#N/A	#N/A	#N/A	#N/A	#N/A	282487.0	266163.9	238770.5	263451.9
Q5JCT0	Beta-1,3-galactosyl-O-glycosyl-glycoprotein beta-1,6-N-acetylglucosaminyltransferase 3	Gcnt3	437	50.7	8.25	#N/A	#N/A	#N/A	#N/A	#N/A	#N/A	#N/A	#N/A	#N/A	#N/A	121439.3	123365.1	84953.4	97162.5
Q9CPW0	Contactin-associated protein-like 2	Cntnap2	1332	148.1	6.77	#N/A	#N/A	#N/A	#N/A	#N/A	#N/A	#N/A	#N/A	#N/A	#N/A	681240.7	720828.5	826169.3	660749.0
P45700	Mannosyl-oligosaccharide 1,2-alpha-mannosidase IA	Man1a1	655	73.2	6.81	#N/A	#N/A	#N/A	#N/A	#N/A	#N/A	#N/A	#N/A	#N/A	#N/A	88886.3	91963.8	85256.9	90467.0
Q64267	DNA repair protein complementing XP-A cells homolog	Xpa	272	31.4	7.43	#N/A	#N/A	#N/A	#N/A	#N/A	#N/A	#N/A	#N/A	#N/A	#N/A	228672.8	221125.7	221856.9	225789.5
A0A075B5Q3	Ig-like domain-containing protein	Ighv2-5	115	12.5	8.97	#N/A	#N/A	#N/A	#N/A	#N/A	#N/A	#N/A	#N/A	#N/A	#N/A	411413.3	279236.0	403686.4	234111.8
Q811B5	Proline-rich protein 3	Prr3	190	21.2	9.85	#N/A	#N/A	#N/A	#N/A	#N/A	#N/A	#N/A	#N/A	#N/A	#N/A	513644.6	452946.5	399512.3	405067.7
O88574	Histone deacetylase complex subunit SAP30	Sap30	220	23.2	9.07	#N/A	#N/A	#N/A	#N/A	#N/A	#N/A	#N/A	#N/A	#N/A	#N/A	433030.4	645184.7	455133.6	514259.5
Q8BIQ6	Predicted gene, EG210853	Zfp947	437	51.4	8.87	#N/A	#N/A	#N/A	#N/A	#N/A	#N/A	#N/A	#N/A	#N/A	#N/A	4384.5	3461.1	3867.1	4691.2
Q8BMQ3	Zinc finger protein basonuclin-2	Bnc2	1127	125.3	6.48	#N/A	#N/A	#N/A	#N/A	#N/A	#N/A	#N/A	#N/A	#N/A	#N/A	89082.9	70417.4	90356.2	79110.9
Q14B70	Highly divergent homeobox	Hdx	692	76.8	5.59	#N/A	#N/A	#N/A	#N/A	#N/A	#N/A	#N/A	#N/A	#N/A	#N/A	1066143.6	1249648.1	1185966.7	1146658.7
B1AWG4	Leucine-rich repeat-containing 37A	Lrrc37a	3298	366.5	4.88	#N/A	#N/A	#N/A	#N/A	#N/A	#N/A	#N/A	#N/A	#N/A	#N/A	692407.6	693186.4	525815.5	631536.3
Q8VGP1	Olfactory receptor	Olfr1256	306	34.7	8.73	#N/A	#N/A	#N/A	#N/A	#N/A	#N/A	#N/A	#N/A	#N/A	#N/A	15133.9	23643.5	16510.2	18049.7
Q80Y20	Alkylated DNA repair protein alkB homolog 8	Alkbh8	664	74.7	7.97	#N/A	#N/A	#N/A	#N/A	#N/A	#N/A	#N/A	#N/A	#N/A	#N/A	705833.9	769372.3	734080.2	769751.0

Supplemental Table S2b. Differentially expressed testis proteome identified by TMT labeled LC-MS/MS analysis

* **Among the identified proteins, a protein whose p-value <0.05, which is a t-test result, is less than.**

Accession	Protein name (564)	Gene name	# AAs	MW [kDa]	calc. pI	WT average	KO average	p-value (KO vs WT)	FC (KO vs WT)
Q99MW1	Serine/threonine-protein kinase 31	Stk31	1018	114.9	5.39	1341125.33	898182.60	0.013	0.670
Q8CDG1	Piwi-like protein 2	Piwil2	971	109.4	8.97	1199118.18	745239.31	0.001	0.621
Q61496	ATP-dependent RNA helicase DDX4	Ddx4	702	76.4	6.09	1395283.36	1008624.49	0.010	0.723
Q9JMB7	Piwi-like protein 1	Piwil1	862	98.5	9.38	1410759.00	643640.06	0.000	0.456
Q99MV7	RING finger protein 17	Rnf17	1640	185.5	5.48	607608.64	500355.20	0.003	0.823
Q14BI7	ATP-dependent RNA helicase TDRD9	Tdrd9	1383	155.9	6.7	502104.57	402588.62	0.030	0.802
Q99MV1	Tudor domain-containing protein 1	Tdrd1	1172	129.6	5.81	531939.48	336759.94	0.014	0.633
P61407	Tudor domain-containing protein 6	Tdrd6	2134	237.8	5.36	243346.07	146532.10	0.009	0.602
Q8K1H1	Tudor domain-containing protein 7	Tdrd7	1086	122.1	6.7	568378.11	429194.16	0.020	0.755
E9Q616	PDZ domain-containing protein	Ahnak	5656	603.9	6.3	4610599.20	5749488.01	0.001	1.247
P16546	Spectrin alpha chain, non-erythrocytic 1	Sptan1	2472	284.4	5.33	5283398.89	6819103.70	0.002	1.291
E9PWQ3	Collagen, type VI, alpha 3	Col6a3	3284	353.7	6.93	9247344.36	15950165.53	0.003	1.725
P26039	Talin-1	Tln1	2541	269.7	6.18	2886505.73	3115688.36	0.045	1.079
Q62261	Spectrin beta chain, non-erythrocytic 1	Sptbn1	2363	274.1	5.58	3450943.71	4307642.28	0.049	1.248
Q6ZWQ0	Nesprin-2	Syne2	6874	782.2	5.33	2044584.76	2156267.33	0.044	1.055
P07901	Heat shock protein HSP 90-alpha	Hsp90aa1	733	84.7	5.01	27112206.80	20494963.82	0.001	0.756
Q61879	Myosin-10	Myh10	1976	228.9	5.54	1063593.80	1212650.56	0.020	1.140
P58252	Elongation factor 2	Eef2	858	95.3	6.83	13207564.63	12177468.61	0.012	0.922
Q9WTQ5	A-kinase anchor protein 12	Akap12	1684	180.6	4.44	2353551.57	2646246.36	0.011	1.124
P08113	Endoplasmin	Hsp90b1	802	92.4	4.82	19636626.28	14329088.87	0.002	0.730
F6ZDS4	Nucleoprotein TPR	Tpr	2431	273.8	5.03	1979398.38	2250518.42	0.000	1.137
E9Q7G0	Nuclear mitotic apparatus protein 1	Numa1	2094	235.5	5.87	1412966.05	1691268.91	0.000	1.197
P20152	Vimentin	Vim	466	53.7	5.12	22654234.69	30601713.18	0.000	1.351
P01027	Complement C3	C3	1663	186.4	6.73	2999001.84	2005843.60	0.007	0.669
Q61554	Fibrillin-1	Fbn1	2873	312.1	4.92	1260066.92	2093120.43	0.000	1.661
P14733	Lamin-B1	Lmnb1	588	66.7	5.16	3961058.08	5588758.69	0.006	1.411
P80314	T-complex protein 1 subunit beta	Cct2	535	57.4	6.4	3143991.92	2837939.36	0.022	0.903
P48722	Heat shock 70 kDa protein 4L	Hspa4l	838	94.3	5.74	1954978.39	1213325.64	0.000	0.621
P17156	Heat shock-related 70 kDa protein 2	Hspa2	633	69.6	5.67	8176812.22	3765509.73	0.000	0.461
Q45VK7	Cytoplasmic dynein 2 heavy chain 1	Dync2h1	4306	492	6.6	927695.84	691987.51	0.000	0.746
Q9JKR6	Hypoxia up-regulated protein 1	Hyou1	999	111.1	5.19	3965202.17	2978305.50	0.010	0.751
P27773	Protein disulfide-isomerase A3	Pdia3	505	56.6	6.21	12759052.09	10265483.16	0.000	0.805
Q6ZQ38	Cullin-associated NEDD8-dissociated protein 1	Cand1	1230	136.2	5.78	4155351.88	3769403.36	0.019	0.907
P40142	Transketolase	Tkt	623	67.6	7.5	4324990.05	5994752.03	0.001	1.386
P27546	Microtubule-associated protein 4	Map4	1125	117.4	4.98	1205275.68	1339084.15	0.020	1.111
A2AX52	Collagen alpha-4(VI) chain	Col6a4	2309	250.6	6.81	1009888.59	1431194.27	0.003	1.417
P10126	Elongation factor 1-alpha 1	Eef1a1	462	50.1	9.01	18198559.40	15977590.46	0.001	0.878
Q9JKF1	Ras GTPase-activating-like protein IQGAP1	Iqgap1	1657	188.6	6.48	921343.46	984965.01	0.001	1.069
P06801	NADP-dependent malic enzyme	Me1	572	63.9	7.44	4871566.89	3883790.34	0.000	0.797
P16858	Glyceraldehyde-3-phosphate dehydrogenase	Gapdh	333	35.8	8.25	12313682.04	13994317.37	0.046	1.136
P09103	Protein disulfide-isomerase	P4hb	509	57	4.88	6460502.26	5715651.15	0.013	0.885
Q64514	Tripeptidyl-peptidase 2	Tpp2	1262	139.8	6.58	2607104.18	2364293.40	0.048	0.907
O70318	Band 4.1-like protein 2	Epb41l2	988	109.9	5.43	1942672.81	2395502.81	0.047	1.233
Q8CI94	Glycogen phosphorylase, brain form	Pygb	843	96.7	6.73	2796463.85	3595750.44	0.002	1.286
Q7TMM9	Tubulin beta-2A chain	Tubb2a	445	49.9	4.89	535334.86	601129.69	0.003	1.123
Q05793	Basement membrane-specific heparan sulfate proteoglycan core protein	Hspg2	3707	398	6.32	787331.59	949469.56	0.011	1.206
P14824	Annexin A6	Anxa6	673	75.8	5.5	3059989.57	3768385.54	0.015	1.232
P80316	T-complex protein 1 subunit epsilon	Cct5	541	59.6	6.02	2669357.89	2245856.05	0.006	0.841
P08003	Protein disulfide-isomerase A4	Pdia4	638	71.9	5.31	4529409.89	3981327.61	0.001	0.879
P14211	Calreticulin	Calr	416	48	4.49	13012136.70	10107183.88	0.002	0.777
Q60675	Laminin subunit alpha-2	Lama2	3118	343.6	6.09	980383.12	1076971.21	0.004	1.099
Q80X19	Collagen alpha-1(XIV) chain	Col14a1	1797	192.9	5.1	1026739.62	1223016.68	0.006	1.191
P48678	Prelamin-A/C	Lmna	665	74.2	6.98	2222864.95	3576792.38	0.001	1.609
P70168	Importin subunit beta-1	Kpnb1	876	97.1	4.78	2227270.58	2011659.68	0.040	0.903
Q920B9	FACT complex subunit SPT16	Supt16h	1047	119.7	5.66	1322950.93	1424763.64	0.037	1.077
Q04857	Collagen alpha-1(VI) chain	Col6a1	1025	108.4	5.36	3188896.18	5202374.97	0.005	1.631

P50247	Adenosylhomocysteinase	Ahcy	432	47.7	6.54	7507213.30	10941366.83	0.006	1.457
Q91ZX7	Prolow-density lipoprotein receptor-related protein 1	Lrp1	4545	504.4	5.36	1041237.48	1134226.85	0.001	1.089
P17563	Methanethiol oxidase	Selenbp1	472	52.5	6.29	1730940.02	2682998.74	0.009	1.550
P07356	Annexin A2	Anxa2	339	38.7	7.69	6724667.62	10619741.65	0.001	1.579
Q76MZ3	Serine/threonine-protein phosphatase 2A 65 kDa regulatory subunit A alpha isoform	Ppp2r1a	589	65.3	5.11	1147072.04	1237067.71	0.032	1.078
P59242	Cingulin	Cgn	1191	136.4	5.91	1077029.60	1152431.98	0.036	1.070
Q9EPU0	Regulator of nonsense transcripts 1	Upf1	1124	123.9	6.61	1493639.52	1261472.23	0.004	0.845
Q8VDM4	26S proteasome non-ATPase regulatory subunit 2	Psmd2	908	100.1	5.17	2049180.34	1836870.99	0.023	0.896
P19324	Serpin H1	Serpinh1	417	46.5	8.82	1724836.03	1188629.28	0.003	0.689
O08553	Dihydropyrimidinase-related protein 2	Dpysl2	572	62.2	6.38	1341814.26	1809018.60	0.003	1.348
P17427	AP-2 complex subunit alpha-2	Ap2a2	938	104	6.93	387072.27	410459.44	0.016	1.060
P35564	Calnexin	Canx	591	67.2	4.64	2416505.88	2079953.68	0.003	0.861
P48036	Annexin A5	Anxa5	319	35.7	4.96	5642670.60	8406267.60	0.007	1.490
P08249	Malate dehydrogenase, mitochondrial	Mdh2	338	35.6	8.68	7014224.91	7554969.61	0.007	1.077
P28665	Murinoglobulin-1	Mug1	1476	165.2	6.42	1127687.03	721722.10	0.001	0.640
P80317	T-complex protein 1 subunit zeta	Cct6a	531	58	7.08	3248095.62	2755969.82	0.032	0.848
Q922R8	Protein disulfide-isomerase A6	Pdia6	440	48.1	5.14	1796523.69	1551698.18	0.024	0.864
Q91ZW3	SWI/SNF-related matrix-associated actin-dependent regulator of chromatin subfamily A member 5	Smarca5	1051	121.6	8.15	1335452.66	1205666.46	0.041	0.903
P10649	Glutathione S-transferase Mu 1	Gstm1	218	26	7.94	11550322.11	16609552.50	0.000	1.438
P29341	Polyadenylate-binding protein 1	Pabpc1	636	70.6	9.5	3893219.88	3149345.03	0.002	0.809
Q99JF8	PC4 and SFRS1-interacting protein	Psip1	528	59.7	9.13	1278154.41	881495.71	0.002	0.690
O08709	Peroxiredoxin-6	Prdx6	224	24.9	6.01	3702997.10	4726756.69	0.004	1.276
P10518	Delta-aminolevulinic acid dehydratase	Alad	330	36	6.79	1298743.12	1592048.59	0.038	1.226
Q9CUU3	Synaptonemal complex protein 2	Sycp2	1500	172	7.93	571789.02	459728.68	0.033	0.804
Q8VC30	Triokinase/FMN cyclase	Tkfc	578	59.7	6.92	1291857.41	811002.62	0.006	0.628
P58281	Dynamin-like 120 kDa protein, mitochondrial	Opa1	960	111.3	7.55	1223570.81	1272915.56	0.012	1.040
Q9D8E6	60S ribosomal protein L4	Rpl4	419	47.1	11	2880211.56	2212267.45	0.002	0.768
Q00PI9	Heterogeneous nuclear ribonucleoprotein U-like protein 2	Hnrnpul2	745	84.9	4.89	1263457.09	1429784.39	0.001	1.132
P19137	Laminin subunit alpha-1	Lama1	3083	337.9	6.71	658757.60	702071.34	0.038	1.066
P26040	Ezrin	Ezr	586	69.4	6.1	725597.32	868585.68	0.020	1.197
Q69ZN7	Myoferlin	Myof	2048	233.2	6.16	236719.97	303829.54	0.004	1.283
O35685	Nuclear migration protein nudC	Nudc	332	38.3	5.26	2228106.42	1853368.42	0.015	0.832
P00342	L-lactate dehydrogenase C chain	Ldhc	332	35.9	8.21	2492442.87	903287.09	0.003	0.362
Q9WTM5	RuvB-like 2	Ruvbl2	463	51.1	5.64	1142989.08	981042.91	0.002	0.858
P07759	Serine protease inhibitor A3K	Serpina3k	418	46.9	5.16	1657504.98	652918.56	0.002	0.394
P11881	Inositol 1,4,5-trisphosphate receptor type 1	Itpr1	2749	313	6.04	681171.94	706153.40	0.039	1.037
Q9R0L6	Pericentriolar material 1 protein	Pcm1	2025	228.7	5.01	946305.55	739131.28	0.021	0.781
E9QA15	Caldesmon 1	Cald1	768	89.2	5.5	627075.35	779361.58	0.030	1.243
Q61838	Pregnancy zone protein	Pzp	1495	165.7	6.68	762425.78	561820.85	0.006	0.737
Q8BMF4	Dihydrolipoyllysine-residue acetyltransferase component of pyruvate dehydrogenase complex, mitochondrial	Dlat	642	67.9	8.57	1296829.13	1056741.49	0.000	0.815
P54869	Hydroxymethylglutaryl-CoA synthase, mitochondrial	Hmgcs2	508	56.8	8.41	2324153.73	1853114.92	0.009	0.797
P05202	Aspartate aminotransferase, mitochondrial	Got2	430	47.4	9	2094264.25	2311282.64	0.045	1.104
P51881	ADP/ATP translocase 2	Slc25a5	298	32.9	9.73	5524756.42	4424172.41	0.025	0.801
Q8CIB5	Fermitin family homolog 2	Fermt2	680	77.8	6.7	342384.79	421883.96	0.001	1.232
P82198	Transforming growth factor-beta-induced protein ig-h3	Tgfbi	683	74.6	7.06	690876.94	1198645.33	0.001	1.735
P17742	Peptidyl-prolyl cis-trans isomerase A	Ppia	164	18	7.9	6079091.69	5586937.24	0.003	0.919
Q91X72	Hemopexin	Hpx	460	51.3	7.8	3448739.44	2468644.54	0.009	0.716
E9Q557	Desmoplakin	Dsp	2883	332.7	6.8	312207.78	437837.10	0.040	1.402
P12970	60S ribosomal protein L7a	Rpl7a	266	30	10.56	1982016.30	1524385.90	0.000	0.769
Q02788	Collagen alpha-2(VI) chain	Col6a2	1034	110.3	6.42	2231602.40	4003014.49	0.007	1.794
Q8BH64	EH domain-containing protein 2	Ehd2	543	61.1	6.51	1019020.17	1381313.58	0.044	1.356
B2RXS4	Plexin-B2	Plxnb2	1842	206.1	5.87	308625.14	347463.29	0.007	1.126
Q61781	Keratin, type I cytoskeletal 14	Krt14	484	52.8	5.17	382350.49	634284.62	0.046	1.659
Q9Z2I8	Succinate--CoA ligase [GDP-forming] subunit beta, mitochondrial	Suclg2	433	46.8	7.02	859325.09	981150.43	0.028	1.142
P09470	Angiotensin-converting enzyme	Ace	1312	150.8	6.55	404956.98	458631.57	0.015	1.133
Q00623	Apolipoprotein A-I	Apoa1	264	30.6	5.73	4281325.73	3662786.38	0.023	0.856
Q9CZ30	Obg-like ATPase 1	Ola1	396	44.7	7.81	1166364.31	1016028.67	0.009	0.871
P28654	Decorin	Dcn	354	39.8	8.68	1259056.20	2440139.75	0.006	1.938
P62806	Histone H4	H4f16	103	11.4	11.36	10273344.39	13371500.08	0.039	1.302
Q61555	Fibrillin-2	Fbn2	2907	313.6	4.84	210543.43	270620.05	0.001	1.285
P10852	4F2 cell-surface antigen heavy chain	Slc3a2	526	58.3	5.91	621409.87	554613.93	0.016	0.893
P43277	Histone H1.3	H1-3	221	22.1	11.03	3742141.53	4958302.66	0.018	1.325
P10493	Nidogen-1	Nid1	1245	136.5	5.44	747792.26	931666.98	0.049	1.246

P18242	Cathepsin D	Ctsd	410	44.9	7.15	1732950.91	2324125.05	0.031	1.341
P61205	ADP-ribosylation factor 3	Arf3	181	20.6	7.43	4607748.94	4312360.03	0.000	0.936
P18293	Atrial natriuretic peptide receptor 1	Npr1	1057	119	6.89	275844.72	332759.45	0.000	1.206
Q5SSW2	Proteasome activator complex subunit 4	Psme4	1843	211.1	7.01	241874.97	218426.60	0.026	0.903
F8VQC1	Signal recognition particle subunit SRP72	Srp72	671	74.6	9.23	354823.88	292442.26	0.045	0.824
P14206	40S ribosomal protein SA	Rpsa	295	32.8	4.87	1872519.09	1709402.61	0.021	0.913
Q8BND3	WD repeat-containing protein 35	Wdr35	1181	133.9	6.34	453647.98	358666.58	0.026	0.791
Q9CR16	Peptidyl-prolyl cis-trans isomerase D	Ppid	370	40.7	7.43	708320.14	580335.81	0.000	0.819
Q6NWV3	Intraflagellar transport protein 122 homolog	Ift122	1182	134.7	6.98	353893.91	279932.42	0.046	0.791
Q9QYB5	Gamma-adducin	Add3	706	78.7	5.95	445395.97	506986.47	0.000	1.138
P07742	Ribonucleoside-diphosphate reductase large subunit	Rrm1	792	90.2	6.7	728575.22	849113.13	0.003	1.165
Q922U2	Keratin, type II cytoskeletal 5	Krt5	580	61.7	7.75	660430.77	939523.94	0.027	1.423
Q91Z31	Polypyrimidine tract-binding protein 2	Ptbp2	531	57.5	8.66	1254817.06	745683.82	0.000	0.594
Q99KJ8	Dynactin subunit 2	Dctn2	402	44.1	5.26	580275.06	466026.94	0.001	0.803
Q8BRF7	Sec1 family domain-containing protein 1	Scfd1	639	72.3	6.38	548650.06	477731.46	0.027	0.871
P62880	Guanine nucleotide-binding protein G(I)/G(S)/G(T) subunit beta-2	Gnb2	340	37.3	6	590167.53	692680.19	0.000	1.174
Q924M7	Mannose-6-phosphate isomerase	Mpi	423	46.5	5.95	388172.81	323455.78	0.000	0.833
O54724	Caveolae-associated protein 1	Cavin1	392	43.9	5.52	1495246.90	2030720.61	0.009	1.358
Q9QZ82	Cholesterol side-chain cleavage enzyme, mitochondrial	Cyp11a1	526	60.3	9.39	487181.68	321806.76	0.036	0.661
P27659	60S ribosomal protein L3	Rpl3	403	46.1	10.21	2735823.16	2299340.53	0.002	0.840
Q99MN1	Lysine--tRNA ligase	Kars1	595	67.8	5.94	944435.13	869932.61	0.028	0.921
Q8BJS4	SUN domain-containing protein 2	Sun2	731	81.6	7.02	364472.29	411116.87	0.005	1.128
P47740	Aldehyde dehydrogenase family 3 member A2	Aldh3a2	484	53.9	8.35	796686.62	930789.68	0.017	1.168
Q0HA38	Tetratricopeptide repeat protein 21B	Ttc21b	1315	150.7	7.14	422236.04	317382.96	0.013	0.752
P49312	Heterogeneous nuclear ribonucleoprotein A1	Hnrnpa1	320	34.2	9.23	634083.40	725440.46	0.004	1.144
P52194	Calmegin	Clgn	611	69.4	4.68	695133.14	349830.93	0.002	0.503
P51885	Lumican	Lum	338	38.2	6.43	1340131.51	2483710.30	0.003	1.853
Q9D154	Leukocyte elastase inhibitor A	Serpinb1a	379	42.5	6.21	499769.81	609049.05	0.042	1.219
Q9QYI3	DnaJ homolog subfamily C member 7	Dnajc7	494	56.4	6.49	457315.60	389069.11	0.021	0.851
O35639	Annexin A3	Anxa3	323	36.4	5.76	661427.47	967908.93	0.032	1.463
Q02257	Junction plakoglobin	Jup	745	81.7	6.14	664920.02	886774.51	0.002	1.334
P28656	Nucleosome assembly protein 1-like 1	Nap1l1	391	45.3	4.46	600265.62	539525.87	0.029	0.899
Q8BH04	Phosphoenolpyruvate carboxykinase [GTP], mitochondrial	Pck2	640	70.5	7.28	310078.72	360958.40	0.020	1.164
Q62383	Transcription elongation factor SPT6	Supt6h	1726	199	4.93	507216.12	538933.96	0.023	1.063
Q91VU7	Pseudouridylate synthase 7 homolog	Pus7	660	74.7	5.87	215101.58	240920.33	0.009	1.120
Q6NZC7	SEC23-interacting protein	Sec23ip	998	110.7	5.94	1033692.36	940634.63	0.034	0.910
P56959	RNA-binding protein FUS	Fus	518	52.6	9.36	583411.74	681785.99	0.049	1.169
Q99MD6	Thioredoxin reductase 3	Txnrd3	652	71.3	8.09	884574.96	689977.90	0.037	0.780
Q922Q8	Leucine-rich repeat-containing protein 59	Lrrc59	307	34.9	9.52	988548.68	842076.87	0.001	0.852
O35226	26S proteasome non-ATPase regulatory subunit 4	Psmd4	376	40.7	4.79	632803.10	577696.38	0.043	0.913
Q61830	Macrophage mannose receptor 1	Mrc1	1456	164.9	6.83	356693.89	273140.84	0.017	0.766
Q91W90	Thioredoxin domain-containing protein 5	Txndc5	417	46.4	5.78	742112.66	620678.30	0.000	0.836
Q8C0C7	Phenylalanine--tRNA ligase alpha subunit	Farsa	508	57.6	8.28	833992.73	741534.68	0.026	0.889
P43276	Histone H1.5	H1-5	223	22.6	10.92	1326732.85	1998133.12	0.001	1.506
P53994	Ras-related protein Rab-2A	Rab2a	212	23.5	6.54	1066758.78	880797.89	0.048	0.826
Q9JHW2	Omega-amidase NIT2	Nit2	276	30.5	6.9	684666.51	807175.08	0.001	1.179
Q80U95	Ubiquitin-protein ligase E3C	Ube3c	1083	123.9	6.39	220949.26	209990.78	0.047	0.950
Q8BUR4	Dedicator of cytokinesis protein 1	Dock1	1865	214.9	7.62	116413.95	126062.70	0.048	1.083
Q6P2K6	Serine/threonine-protein phosphatase 4 regulatory subunit 3A	Ppp4r3a	820	93.8	4.89	464280.71	510159.36	0.014	1.099
P11679	Keratin, type II cytoskeletal 8	Krt8	490	54.5	5.82	432517.95	610110.47	0.004	1.411
Q8BWF0	Succinate-semialdehyde dehydrogenase, mitochondrial	Aldh5a1	523	55.9	8.25	227649.39	245051.07	0.001	1.076
O54879	High mobility group protein B3	Hmgb3	200	23	8.37	1054369.72	1443378.61	0.034	1.369
P10107	Annexin A1	Anxa1	346	38.7	7.37	397418.80	553125.52	0.047	1.392
P48758	Carbonyl reductase [NADPH] 1	Cbr1	277	30.6	8.31	842315.66	925204.14	0.041	1.098
Q9DA79	Dipeptidase 3	Dpep3	493	54.2	6.48	1678375.43	1137234.94	0.014	0.678
Q8K0U4	Heat shock 70 kDa protein 12A	Hspa12a	675	74.8	6.77	267081.90	316326.09	0.033	1.184
Q8VBW6	NEDD8-activating enzyme E1 regulatory subunit	Nae1	534	60.2	5.52	289444.53	271236.16	0.024	0.937
Q9DAR7	m7GpppX diphosphatase	Dcps	338	39	6.48	753513.16	872595.33	0.007	1.158
P46664	Adenylosuccinate synthetase isozyme 2	Adss2	456	50	6.38	382153.02	416060.01	0.039	1.089
B2RX14	Terminal uridylyltransferase 4	Tut4	1644	184.5	8.19	172316.74	186254.70	0.034	1.081
P53395	Lipoamide acyltransferase component of branched-chain alpha-keto acid dehydrogenase complex, mitochondrial	Dbt	482	53.2	8.6	631056.13	752736.61	0.001	1.193
Q91V76	Ester hydrolase C11orf54 homolog	MGI:1918234	315	35	6.29	584260.21	684960.51	0.038	1.172
P97822	Acidic leucine-rich nuclear phosphoprotein 32 family member E	Anp32e	260	29.6	3.88	495549.84	593191.99	0.006	1.197

Q9JHQ5	Leucine zipper transcription factor-like protein 1	Lztfl1	299	34.8	5.17	422663.41	296168.26	0.028	0.701
Q99KP3	Lambda-crystallin homolog	Cryl1	319	35.2	5.86	254083.38	322180.70	0.036	1.268
Q9Z2C8	Y-box-binding protein 2	Ybx2	360	38.2	10.95	286736.77	168930.20	0.017	0.589
Q6PAR5	GTPase-activating protein and VPS9 domain-containing protein 1	Gapvd1	1458	162.3	5.19	244094.98	218350.41	0.004	0.895
Q99L47	Hsc70-interacting protein	St13	371	41.6	5.26	1792560.37	1091964.22	0.006	0.609
Q60865	Caprin-1	Caprin1	707	78.1	5.25	888263.44	765874.42	0.048	0.862
P35487	Pyruvate dehydrogenase E1 component subunit alpha, testis-specific form, mitochondrial	Pdha2	391	43.4	8.5	587127.65	310175.54	0.001	0.528
Q8CCJ3	E3 UFM1-protein ligase 1	Ufl1	793	89.5	6.67	374190.68	347379.23	0.024	0.928
Q80X68	Citrate synthase	Csl	466	52.3	8.79	180756.82	115038.81	0.018	0.636
P97384	Annexin A11	Anxa11	503	54	7.66	965588.81	1089990.44	0.006	1.129
Q8BX02	KN motif and ankyrin repeat domain-containing protein 2	Kank2	843	90.2	5.55	161576.56	203695.77	0.005	1.261
Q80XI3	Eukaryotic translation initiation factor 4 gamma 3	Eif4g3	1579	174.8	5.53	348908.85	275673.47	0.023	0.790
Q8CHP8	Glycerol-3-phosphate phosphatase	Pgp	321	34.5	5.35	716816.33	563303.22	0.000	0.786
P57759	Endoplasmic reticulum resident protein 29	Erp29	262	28.8	6.15	1230294.11	1045958.52	0.001	0.850
Q00915	Retinol-binding protein 1	Rbp1	135	15.8	5.25	751499.36	1032657.26	0.017	1.374
P97449	Aminopeptidase N	Anpep	966	109.6	5.9	188016.17	254166.97	0.016	1.352
Q78IK4	MICOS complex subunit Mic27	Apool	265	29.2	9.31	496898.34	540784.71	0.022	1.088
Q9EQQ9	Protein O-GlcNAcase	Oga	916	103.1	4.92	148139.18	182204.34	0.000	1.230
P70302	Stromal interaction molecule 1	Stim1	685	77.5	6.54	828467.80	705737.55	0.003	0.852
Q64518	Sarcoplasmic/endoplasmic reticulum calcium ATPase 3	Atp2a3	1038	113.6	5.85	65836.09	77191.57	0.007	1.172
Q61171	Peroxiredoxin-2	Prdx2	198	21.8	5.41	5503362.15	6191460.16	0.007	1.125
Q99KH8	Serine/threonine-protein kinase 24	Stk24	431	47.9	5.43	368871.52	406192.43	0.015	1.101
Q8BKG3	Inactive tyrosine-protein kinase 7	Ptk7	1062	117.5	6.84	395205.90	485254.33	0.040	1.228
Q6ZQ58	La-related protein 1	Larp1	1072	121.1	8.79	333060.23	270422.38	0.013	0.812
Q9WVA4	Transgelin-2	Tagln2	199	22.4	8.24	512578.38	644812.46	0.010	1.258
Q8CH09	SURP and G-patch domain-containing protein 2	Sugp2	1067	118	8.31	349871.55	238170.84	0.001	0.681
Q6PE01	U5 small nuclear ribonucleoprotein 40 kDa protein	Snrnp40	358	39.3	8.1	340912.95	380937.83	0.003	1.117
Q5H8C4	Vacuolar protein sorting-associated protein 13A	Vps13a	3166	359.2	6.19	356202.01	299234.88	0.013	0.840
Q9D4D4	Transketolase-like protein 2	Tktl2	627	68.4	6.96	670082.48	312457.97	0.000	0.466
Q7TQH0	Ataxin-2-like protein	Atxn2l	1049	110.6	8.85	419819.37	369865.73	0.028	0.881
Q61024	Asparagine synthetase [glutamine-hydrolyzing]	Asns	561	64.2	6.58	669759.22	607929.50	0.018	0.908
Q9WUP7	Ubiquitin carboxyl-terminal hydrolase isozyme L5	Uchl5	329	37.6	5.33	430463.58	385027.93	0.011	0.894
Q8R2G4	Ecto-ADP-ribosyltransferase 3	Art3	371	42	6.02	428956.75	345411.37	0.035	0.805
P42125	Enoyl-CoA delta isomerase 1, mitochondrial	Eci1	289	32.2	8.98	914952.50	1076365.17	0.000	1.176
E9Q4X2	UDP-glucose glycoprotein glucosyltransferase 2	Uggt2	1504	172.7	6.2	323340.07	263552.92	0.001	0.815
P62754	40S ribosomal protein S6	Rps6	249	28.7	10.84	816720.98	665718.43	0.003	0.815
P47911	60S ribosomal protein L6	Rpl6	296	33.5	10.7	2610078.74	2251758.23	0.009	0.863
Q8C0M9	Isoaspartyl peptidase/L-asparaginase	Asrgl1	326	33.9	7.65	626050.94	450083.21	0.000	0.719
O70503	Very-long-chain 3-oxoacyl-CoA reductase	Hsd17b12	312	34.7	9.52	800899.10	732932.86	0.005	0.915
Q9CZX8	40S ribosomal protein S19	Rps19	145	16.1	10.4	1319627.62	1160010.00	0.004	0.879
P62830	60S ribosomal protein L23	Rpl23	140	14.9	10.51	868141.21	716991.13	0.001	0.826
P49586	Choline-phosphate cytidylyltransferase A	Pcyt1a	367	41.6	7.03	448764.59	570931.73	0.006	1.272
Q8R4N0	Citramalyl-CoA lyase, mitochondrial	Clybl	338	37.5	8.54	171929.72	193691.45	0.006	1.127
P47963	60S ribosomal protein L13	Rpl13	211	24.3	11.55	1469739.90	1227256.78	0.032	0.835
P32037	Solute carrier family 2, facilitated glucose transporter member 3	Slc2a3	493	53.4	4.98	1121923.60	315904.87	0.002	0.282
Q9D706	RNA polymerase II-associated protein 3	Rpap3	660	74.1	7.99	325601.11	287358.00	0.007	0.883
O35350	Calpain-1 catalytic subunit	Capn1	713	82.1	5.87	200139.61	223679.41	0.012	1.118
O08716	Fatty acid-binding protein 9	Fabp9	132	15	7.74	793482.12	323940.31	0.001	0.408
Q61490	CD166 antigen	Alcam	583	65.1	6.15	229176.45	271388.76	0.026	1.184
Q9JK53	Prolargin	Prelp	378	43.3	9.54	653532.38	1017027.52	0.029	1.556
Q8R3N6	THO complex subunit 1	Thoc1	657	75.4	4.97	232282.93	256692.84	0.018	1.105
Q9R0X4	Acyl-coenzyme A thioesterase 9, mitochondrial	Acot9	439	50.5	8.59	368534.89	394132.47	0.047	1.069
O70250	Phosphoglycerate mutase 2	Pgam2	253	28.8	8.5	144300.31	77924.66	0.016	0.540
Q9CPU0	Lactoylglutathione lyase	Glo1	184	20.8	5.47	1246202.59	1542727.88	0.020	1.238
Q6NZF1	Zinc finger CCCH domain-containing protein 11A	Zc3h11a	792	86.4	8.13	257500.70	294892.23	0.001	1.145
Q9CZ42	ATP-dependent (S)-NAD(P)H-hydrate dehydratase	Naxd	343	36.7	7.77	427220.26	398684.37	0.010	0.933
Q9WVE8	Protein kinase C and casein kinase substrate in neurons protein 2	Pacsin2	486	55.8	5.2	302553.32	357248.18	0.039	1.181
Q8R127	Saccharopine dehydrogenase-like oxidoreductase	Sccpdh	429	47.1	8.6	978153.27	618752.55	0.006	0.633
Q07133	Histone H1t	H1-6	208	21.5	11.71	272354.62	153756.38	0.012	0.565
Q3U3T8	WD repeat-containing protein 62	Wdr62	1523	167.2	5.48	251056.51	210106.36	0.009	0.837
Q9D0L7	Armadillo repeat-containing protein 10	Armc10	306	33.3	7.99	347772.10	395203.57	0.000	1.136
P62748	Hippocalcin-like protein 1	Hpcal1	193	22.3	5.5	414443.51	512551.34	0.046	1.237
Q8BH69	Selenide, water dikinase 1	Sephs1	392	42.9	5.97	322744.57	363579.52	0.019	1.127

P86048	60S ribosomal protein L10-like	Rpl10l	214	24.5	10.11	515165.83	322802.07	0.039	0.627
Q8BVP2	L-lactate dehydrogenase	Ldhal6b	382	42	9.16	495255.86	172504.89	0.001	0.348
Q8BL99	Protein dopey-1	Dop1a	2399	269.1	6.24	84921.58	96904.48	0.030	1.141
P51655	Glypican-4	Gpc4	557	62.5	6.33	459454.69	513178.02	0.033	1.117
O09159	Lysosomal alpha-mannosidase	Man2b1	1013	114.6	8.13	167454.64	178412.66	0.021	1.065
Q91Z49	UAP56-interacting factor	Fyttd1	317	35.9	11.84	560820.99	420104.53	0.030	0.749
P02535	Keratin, type I cytoskeletal 10	Krt10	570	57.7	5.11	2026607.37	3110933.70	0.007	1.535
A6H630	Damage-control phosphatase ARMT1	Armt1	439	50.5	5.92	819818.52	434637.50	0.002	0.530
Q8BHC4	Dephospho-CoA kinase domain-containing protein	Dcakd	231	26.5	9.58	662176.86	699005.78	0.024	1.056
O35469	3 beta-hydroxysteroid dehydrogenase/Delta 5-->4-isomerase type 6	Hsd3b6	373	42	6.35	840852.11	396250.37	0.003	0.471
Q9JK81	UPF0160 protein MYG1, mitochondrial	Myg1	380	42.7	7.02	258102.00	277322.10	0.026	1.074
Q8K057	Intraflagellar transport protein 80 homolog	Ift80	777	87.8	7.94	248696.69	191532.43	0.001	0.770
P97447	Four and a half LIM domains protein 1	Fhl1	280	31.9	8.37	178506.54	219499.02	0.000	1.230
Q3UQ84	Threonine--tRNA ligase, mitochondrial	Tars2	723	81.6	7.87	279489.80	254994.69	0.008	0.912
Q62245	Son of sevenless homolog 1	Sos1	1319	150.8	6.89	188235.25	169369.33	0.027	0.900
Q61263	Sterol O-acyltransferase 1	Soat1	540	63.8	9.01	643843.85	480354.88	0.001	0.746
Q91V12	Cytosolic acyl coenzyme A thioester hydrolase	Acot7	381	42.5	8.68	266811.72	233176.09	0.048	0.874
Q62000	Mimecan	Ogn	298	34	5.74	470716.11	711716.89	0.025	1.512
P34022	Ran-specific GTPase-activating protein	Ranbp1	203	23.6	5.22	754664.85	570971.63	0.007	0.757
Q91XQ0	Dynein heavy chain 8, axonemal	Dnah8	4731	540.9	6.18	123875.06	85567.41	0.016	0.691
Q61001	Laminin subunit alpha-5	Lama5	3718	403.8	6.73	72354.91	88328.86	0.001	1.221
Q9Z2L6	Multiple inositol polyphosphate phosphatase 1	Minpp1	481	54.5	7.49	331585.29	274181.55	0.008	0.827
Q8VDG5	Phosphopantothenate--cysteine ligase	Ppcs	311	33.8	6.55	598682.26	557193.25	0.029	0.931
Q8CE96	tRNA (adenine(58)-N(1))-methyltransferase non-catalytic subunit TRM6	Trmt6	497	55.5	6.95	322383.77	355713.10	0.012	1.103
O09130	NFATC2-interacting protein	Nfatc2ip	412	45.1	6.67	399144.98	324725.11	0.005	0.814
P14094	Sodium/potassium-transporting ATPase subunit beta-1	Atp1b1	304	35.2	8.65	882190.51	1069111.12	0.001	1.212
Q8BXQ2	GPI transamidase component PIG-T	Pigt	582	65.7	8.4	564963.17	510464.78	0.013	0.904
Q9D1P4	Cysteine and histidine-rich domain-containing protein 1	Chordc1	331	37.3	7.9	572647.63	476972.75	0.021	0.833
Q08091	Calponin-1	Cnn1	297	33.3	8.97	189209.56	243365.00	0.032	1.286
Q99KF1	Transmembrane emp24 domain-containing protein 9	Tmed9	235	27.1	8.41	1519277.77	1372472.54	0.031	0.903
Q9D3R3	Centrosomal protein of 72 kDa	Cep72	646	72.4	6.74	321258.86	224421.12	0.002	0.699
Q99J09	Methylosome protein 50	Wdr77	342	36.9	5.27	260601.65	219290.15	0.012	0.841
P49817	Caveolin-1	Cav1	178	20.5	6.02	410093.86	562864.06	0.002	1.373
P61027	Ras-related protein Rab-10	Rab10	200	22.5	8.38	397736.45	448512.31	0.019	1.128
P28740	Kinesin-like protein KIF2A	Kif2a	705	79.7	6.73	660259.70	610752.03	0.046	0.925
A3KMP2	Tetratricopeptide repeat protein 38	Ttc38	465	52.2	6.3	182648.68	211269.38	0.041	1.157
Q920Q6	RNA-binding protein Musashi homolog 2	Msi2	346	36.9	8.47	123951.59	134973.22	0.037	1.089
P56376	Acylphosphatase-1	Acyp1	99	11.2	9.04	1223695.60	516493.07	0.000	0.422
Q922Y1	UBX domain-containing protein 1	Ubxn1	297	33.6	5.26	371730.58	333763.09	0.006	0.898
Q8BGZ7	Keratin, type II cytoskeletal 75	Krt75	551	59.7	8.31	287224.08	418945.04	0.000	1.459
Q3V089	RNA-binding protein 44	Rbm44	1013	112.2	5.86	150845.03	124674.30	0.041	0.827
P36536	GTP-binding protein SAR1a	Sar1a	198	22.4	6.93	54075.29	47172.05	0.013	0.872
Q99J99	3-mercaptopyruvate sulfurtransferase	Mpst	297	33.1	6.47	385540.92	442710.41	0.001	1.148
P10833	Ras-related protein R-Ras	Rras	218	23.7	6.79	215319.72	256244.85	0.002	1.190
Q6VH22	Intraflagellar transport protein 172 homolog	Ift172	1749	197.4	6.02	270534.85	198560.16	0.001	0.734
Q91W39	Nuclear receptor coactivator 5	Ncoa5	579	65.3	9.82	255557.46	228454.89	0.009	0.894
Q3TTP0	Testicular spindle-associated protein SHCBP1L	Shcbp1l	639	70.9	5.2	236114.71	129222.63	0.016	0.547
Q8BP40	Lysophosphatidic acid phosphatase type 6	Acp6	418	47.6	7.72	152360.25	173678.43	0.033	1.140
Q8VDL4	ADP-dependent glucokinase	Adpgk	496	53.9	5.62	300221.98	254209.33	0.014	0.847
Q6P069	Sorcin	Sri	198	21.6	5.59	384912.16	446414.44	0.001	1.160
Q8K0C4	Lanosterol 14-alpha demethylase	Cyp51a1	503	56.7	8.41	255517.31	215470.67	0.011	0.843
Q8K299	Scavenger receptor class A member 5	Scara5	491	53.6	6.76	169324.48	146511.15	0.020	0.865
Q9JJ78	Lymphokine-activated killer T-cell-originated protein kinase	Pbk	330	36.7	5.12	655914.02	419577.27	0.001	0.640
P19783	Cytochrome c oxidase subunit 4 isoform 1, mitochondrial	Cox4i1	169	19.5	9.23	1379501.41	1178403.16	0.008	0.854
Q8R164	Valacyclovir hydrolase	Bphl	291	32.8	8.94	281770.67	332155.32	0.010	1.179
Q6R0H7	Guanine nucleotide-binding protein G(s) subunit alpha isoforms XLas	Gnas	1133	121.4	4.81	436334.02	513841.90	0.046	1.178
Q60823	RAC-beta serine/threonine-protein kinase	Akt2	481	55.7	6.37	319751.96	352498.53	0.024	1.102
Q9CWH6	Proteasome subunit alpha type-8	Psma8	250	27.8	8.69	130663.23	78737.38	0.016	0.603
Q922V4	Pleiotropic regulator 1	Plrg1	513	56.9	9.17	382504.28	335322.56	0.019	0.877
Q60634	Flotillin-2	Flot2	428	47	5.2	119846.16	143836.82	0.047	1.200
Q9CZM2	60S ribosomal protein L15	Rpl15	204	24.1	11.62	811165.20	690929.31	0.019	0.852
Q9Z2A9	Glutathione hydrolase 5 proenzyme	Ggt5	573	61.6	8.5	185478.50	259394.28	0.001	1.399
Q8BPB5	EGF-containing fibulin-like extracellular matrix protein 1	Efemp1	493	54.9	5.14	136409.91	163652.81	0.044	1.200

P97820	Mitogen-activated protein kinase kinase kinase kinase 4	Map4k4	1233	140.5	7.47	281016.31	301166.44	0.007	1.072
Q70IV5	Synemin	Synm	1561	173.1	5.12	213390.92	261776.37	0.000	1.227
P00015	Cytochrome c, testis-specific	Cyct	105	11.7	9.51	510019.14	306583.86	0.000	0.601
Q9D0S9	Histidine triad nucleotide-binding protein 2, mitochondrial	Hint2	163	17.3	9.82	271828.41	300516.81	0.005	1.106
Q8VHR5	Transcriptional repressor p66-beta	Gatad2b	594	65.4	9.7	139700.60	179328.34	0.000	1.284
Q8BJ64	Choline dehydrogenase, mitochondrial	Chdh	596	66.4	8.51	205668.48	269820.54	0.040	1.312
Q80TT8	Cullin-9	Cul9	1865	209	5.63	85604.36	74804.52	0.036	0.874
Q8CI59	Metalloreductase STEAP3	Steap3	488	54.7	9.22	278882.98	367838.57	0.002	1.319
Q9Z0R9	Acyl-CoA 6-desaturase	Fads2	444	52.4	8.82	917866.29	546801.79	0.028	0.596
Q9JMG7	Hepatoma-derived growth factor-related protein 3	Hdgfl3	202	22.4	8.4	484608.19	599445.84	0.002	1.237
Q8CCP0	Nuclear export mediator factor Nemf	Nemf	1064	121.1	6.8	142736.12	130792.06	0.045	0.916
Q9Z1S0	Mitotic checkpoint serine/threonine-protein kinase BUB1 beta	Bub1b	1052	118.3	5.43	174753.71	160460.26	0.044	0.918
P67984	60S ribosomal protein L22	Rpl22	128	14.8	9.19	1197396.51	1008632.32	0.003	0.842
P62245	40S ribosomal protein S15a	Rps15a	130	14.8	10.13	1646519.89	1441271.85	0.038	0.875
O88447	Kinesin light chain 1	Klc1	541	61.4	5.68	190691.73	213741.50	0.031	1.121
Q9D2E2	Target of EGR1 protein 1	Toe1	511	56.8	6.64	150454.80	180675.35	0.018	1.201
Q811D0	Disks large homolog 1	Dlg1	905	100.1	5.8	163175.39	192705.40	0.035	1.181
O35215	D-dopachrome decarboxylase	Ddt	118	13.1	6.54	2288411.25	1280171.80	0.001	0.559
Q3UA37	Glutamine-rich protein 1	Qrich1	777	86.5	5.96	132521.33	143837.87	0.034	1.085
O89020	Afamin	Afm	608	69.3	5.78	312217.47	254973.39	0.009	0.817
Q9D1H9	Microfibril-associated glycoprotein 4	Mfap4	257	28.9	5.44	978067.26	2037154.01	0.023	2.083
Q9Z0P4	Paralemmin-1	Palm	383	41.6	4.84	114250.06	150140.45	0.010	1.314
Q8VDF2	E3 ubiquitin-protein ligase UHRF1	Uhrf1	782	88.2	8.31	197124.00	139861.20	0.000	0.710
Q69Z99	Zinc finger protein 512	Znf512	562	63.9	9.51	87652.19	121495.17	0.007	1.386
Q8C8R3	Ankyrin-2	Ank2	3898	426	5.17	121368.93	108994.66	0.014	0.898
P54116	Erythrocyte band 7 integral membrane protein	Stom	284	31.4	6.93	143649.29	122518.21	0.018	0.853
Q3UGF1	WD repeat-containing protein 19	Wdr19	1341	151.4	6.57	283112.32	229266.16	0.004	0.810
P59900	EMILIN-3	Emilin3	758	82.3	6.93	44091.57	62950.82	0.001	1.428
Q9CQU0	Thioredoxin domain-containing protein 12	Txndc12	170	19	5.26	434645.97	393471.98	0.018	0.905
Q99NB3	Ig-like domain-containing protein	Tcam1	548	60.7	5.26	212806.00	151193.89	0.020	0.710
Q5SUE7	Adenosine deaminase domain-containing protein 1	Adad1	619	67.8	8.85	160735.50	115717.46	0.008	0.720
Q9D7V1	SH2 domain-containing protein 4A	Sh2d4a	421	48.4	8.91	98535.30	113401.60	0.042	1.151
P27786	Steroid 17-alpha-hydroxylase/17,20 lyase	Cyp17a1	507	57.6	7.18	240467.79	104443.07	0.006	0.434
Q8BHG2	CXXC motif containing zinc binding protein	Czib	160	18	5.12	436435.32	409089.16	0.030	0.937
Q9D2H9	Dynein assembly factor 1, axonemal	Dnaaf1	634	69.7	4.67	163160.79	87996.82	0.007	0.539
P62488	DNA-directed RNA polymerase II subunit RPB7	Polr2g	172	19.3	5.54	393454.05	426256.69	0.033	1.083
P46412	Glutathione peroxidase 3	Gpx3	226	25.4	8.22	197457.40	251008.97	0.010	1.271
D3YUJ3	CYCLIN domain-containing protein	Ccnyl1	367	41.6	6.34	428190.79	378658.15	0.034	0.884
O88792	Junctional adhesion molecule A	F11r	300	32.4	6.77	126523.93	171351.27	0.049	1.354
A2APV2	Formin-like protein 2	Fmnl2	1086	123	7.53	190623.54	226928.14	0.039	1.190
Q9QY53	Nephrocystin-1	Nphp1	687	77	5.25	201331.93	174956.87	0.037	0.869
P07309	Transthyretin	Ttr	147	15.8	6.16	1474521.49	897085.58	0.011	0.608
Q9WU20	Methylenetetrahydrofolate reductase	Mthfr	654	74.5	5.26	152305.01	201686.34	0.012	1.324
E9PZM7	RING-type domain-containing protein	Scaf11	1456	162	6.77	102404.23	127743.49	0.005	1.247
Q60631	Growth factor receptor-bound protein 2	Grb2	217	25.2	6.32	158951.24	197308.02	0.027	1.241
P07214	SPARC	Sparc	302	34.4	4.86	334009.79	283971.60	0.042	0.850
Q9QYF1	Retinol dehydrogenase 11	Rdh11	316	35.1	8.91	367001.14	264717.32	0.031	0.721
Q8VE62	Polyadenylate-binding protein-interacting protein 1	Paip1	400	45.7	4.55	555323.63	442573.33	0.027	0.797
Q6SKR2	Methyltransferase N6AMT1	N6amt1	214	23	5.62	126359.11	144139.82	0.044	1.141
Q9DBX3	Sushi domain-containing protein 2	Susd2	820	90.6	6.62	254962.37	331269.18	0.008	1.299
P97477	Aurora kinase A	Aurka	395	44.7	9.38	296650.08	217845.01	0.010	0.734
O35638	Cohesin subunit SA-2	Stag2	1231	141.2	5.43	159522.62	213436.71	0.000	1.338
P97298	Pigment epithelium-derived factor	Serpinf1	417	46.2	6.98	207679.61	180604.78	0.034	0.870
Q3U821	WD repeat-containing protein 75	Wdr75	830	94	6.18	154088.87	177523.67	0.034	1.152
Q9CPT4	Myeloid-derived growth factor	Mydgf	166	18	6.79	631378.96	531637.32	0.000	0.842
Q3TJD7	PDZ and LIM domain protein 7	Pdlim7	457	50.1	8.47	373016.88	451345.61	0.020	1.210
Q9CY66	H/ACA ribonucleoprotein complex subunit 1	Gar1	231	23.5	11.02	474694.30	551758.06	0.000	1.162
Q9WVB0	RNA-binding protein with multiple splicing	Rbpms	197	21.8	8.07	163103.75	205694.97	0.040	1.261
E9Q1P8	Interferon regulatory factor 2-binding protein 2	Irf2bp2	570	59.3	8.69	52272.42	60190.58	0.001	1.151
P40630	Transcription factor A, mitochondrial	Tfam	243	28	9.69	576027.09	648200.97	0.031	1.125
P56546	C-terminal-binding protein 2	Ctbp2	445	48.9	6.95	164300.26	214161.97	0.041	1.303
Q8C165	N-fatty-acyl-amino acid synthase/hydrolase PM20D1	Pm20d1	503	55.6	6.43	91134.21	106945.87	0.033	1.173
Q8R4R6	Nucleoporin NUP35	Nup35	325	34.8	9.25	169478.92	185817.16	0.002	1.096

P08074	Carbonyl reductase [NADPH] 2	Cbr2	244	25.9	9.01	574574.91	445286.85	0.008	0.775
Q9R013	Cathepsin F	Ctsf	462	51.6	6.55	137703.69	168173.12	0.014	1.221
Q61704	Inter-alpha-trypsin inhibitor heavy chain H3	Itih3	889	99.3	6.05	152926.56	164066.19	0.037	1.073
Q9D869	Calcineurin B homologous protein 2	Chp2	196	22.6	5.72	198376.85	230588.74	0.014	1.162
Q6PGH2	Jupiter microtubule associated homolog 2	Jpt2	190	20	8.62	262168.23	290976.16	0.014	1.110
Q80ZX8	Sperm-associated antigen 1	Spag1	901	100.6	5.62	137470.47	102270.23	0.016	0.744
Q9CYL5	Golgi-associated plant pathogenesis-related protein 1	Glipr2	154	17.1	9.51	50480.72	75177.05	0.002	1.489
Q09143	High affinity cationic amino acid transporter 1	Slc7a1	622	67	7.06	234450.14	154146.72	0.003	0.657
Q9EQG9	Ceramide transfer protein	Cert1	624	71.1	5.44	140504.37	165090.01	0.001	1.175
Q9D5P4	Adenosine deaminase domain-containing protein 2	Adad2	478	51	8.27	168113.23	103769.72	0.000	0.617
Q9D8T2	Gasdermin-D	Gsdmdc1	487	53.2	5.1	108779.35	131598.81	0.001	1.210
Q924W5	Structural maintenance of chromosomes protein 6	Smc6	1097	127.1	7.17	73788.77	65193.04	0.007	0.884
Q9R1J0	Sterol-4-alpha-carboxylate 3-dehydrogenase, decarboxylating	Nsdhl	362	40.7	7.85	164728.69	128344.31	0.019	0.779
C0HK80	Adipocyte-related X-chromosome expressed sequence 2	Arxes2	180	20.1	9.6	804426.95	860136.13	0.027	1.069
Q80ZM5	H15 domain-containing protein	H1f10	188	20.1	11.22	484177.63	535940.29	0.024	1.107
G5E8K5	Ankyrin-3	Ank3	1961	213.9	6.7	135022.51	159216.00	0.000	1.179
Q61735	Leukocyte surface antigen CD47	Cd47	303	33.1	8.63	275354.26	332943.49	0.001	1.209
Q80XU3	Nuclear ubiquitous casein and cyclin-dependent kinase substrate 1	Nucks1	234	26.3	5.14	89324.42	121562.66	0.001	1.361
Q8BGF3	WD repeat-containing protein 92	Wdr92	357	39.8	8.15	355950.57	250230.43	0.027	0.703
P70351	Histone-lysine N-methyltransferase EZH1	Ezh1	747	85.1	7.58	171360.47	183612.32	0.042	1.071
P46414	Cyclin-dependent kinase inhibitor 1B	Cdkn1b	197	22.2	7.02	53409.70	66027.94	0.006	1.236
P08207	Protein S100-A10	S100a10	97	11.2	6.77	1320262.92	2091795.81	0.006	1.584
Q8BWR4	Ubiquitin carboxyl-terminal hydrolase 40	Usp40	1235	139.9	5.63	185932.84	164714.68	0.011	0.886
Q9D1R9	60S ribosomal protein L34	Rpl34	117	13.3	11.47	1252007.90	1088076.96	0.011	0.869
Q9D0P8	Intraflagellar transport protein 27 homolog	Ift27	186	20.8	5.2	111970.54	87356.93	0.002	0.780
Q922E4	Ethanolamine-phosphate cytidylyltransferase	Pcyt2	404	45.2	6.58	109983.68	91718.97	0.002	0.834
Q9JI75	Ribosyldihydronicotinamide dehydrogenase [quinone]	Nqo2	231	26.2	7.01	260014.51	287278.21	0.021	1.105
Q9CR39	WD repeat domain phosphoinositide-interacting protein 3	Wdr45b	344	38	7.56	187583.90	206848.00	0.019	1.103
Q91V01	Lysophospholipid acyltransferase 5	Lpcat3	487	56.1	8.56	346898.46	303899.14	0.020	0.876
D3YX43	V-set and immunoglobulin domain-containing protein 10	Vsig10	558	60.5	4.7	172297.27	200556.11	0.007	1.164
Q9QVN7	Transcription elongation factor A protein 2	Tcea2	299	33.6	9.13	188178.70	130962.19	0.015	0.696
Q80U19	Disheveled-associated activator of morphogenesis 2	Daam2	1115	128.3	6.92	235597.56	197536.37	0.013	0.838
Q923G2	DNA-directed RNA polymerases I, II, and III subunit RPABC3	Polr2h	150	17.1	4.68	119276.51	135666.38	0.009	1.137
P70255	Nuclear factor 1 C-type	Nfic	439	48.7	8.32	236732.88	311006.83	0.028	1.314
Q3TGF2	Protein FAM107B	Fam107b	131	15.6	8.31	168171.78	120120.13	0.005	0.714
Q9Z1X2	Phosphatidylserine synthase 2	Ptdss2	473	55	7.09	179102.57	160715.57	0.005	0.897
Q8R1K1	Ubiquitin-associated domain-containing protein 2	Ubac2	345	39	9.44	149917.27	129389.52	0.001	0.863
Q8CGB6	Tensin-2	Tns2	1400	151.9	8.48	156993.52	199434.46	0.002	1.270
Q6NZK8	Protein tyrosine phosphatase domain-containing protein 1	Ptpdc1	747	83.9	6.34	173496.26	133109.68	0.006	0.767
Q810Q5	Normal mucosa of esophagus-specific gene 1 protein	Nmes1	83	9.6	9.89	329546.27	218113.11	0.003	0.662
Q91VY9	Zinc finger protein 622	Znf622	476	53.4	6.1	107855.12	97754.27	0.026	0.906
Q9D710	Thioredoxin-related transmembrane protein 2	Tmx2	295	33.9	8.75	270920.48	243695.47	0.008	0.900
Q9Z0E6	Guanylate-binding protein 2	Gbp2	589	66.7	5.71	64017.62	76763.86	0.024	1.199
Q8BUN5	Mothers against decapentaplegic homolog 3	Smad3	425	48.1	7.15	71799.67	121009.39	0.006	1.685
P33610	DNA primase large subunit	Prim2	505	58.4	8.32	104130.71	125810.07	0.003	1.208
Q6Y5D8	Rho GTPase-activating protein 10	Arhgap10	786	89.3	7.18	215522.92	250095.74	0.041	1.160
Q9D0J8	Parathymosin	Ptms	101	11.4	4.22	214620.96	289994.19	0.006	1.351
Q5M8N4	Epimerase family protein SDR39U1	Sdr39u1	293	31.4	9.31	211801.05	181137.92	0.003	0.855
D3YV10	Coiled-coil domain-containing protein 13	Ccdc13	709	79.7	9.01	75429.61	61902.07	0.048	0.821
Q8BUH8	Sentrin-specific protease 7	Senp7	1037	116.3	6.52	44652.57	49842.64	0.018	1.116
Q923B1	Lariat debranching enzyme	Dbr1	550	62.3	5.22	232176.65	250950.48	0.010	1.081
Q66JX5	FGFR1 oncogene partner	Fgfr1op	399	42.7	4.81	124606.69	107940.50	0.018	0.866
Q8C0Z1	Protein FAM234A	Fam234a	555	60.5	5.68	189209.81	222146.63	0.001	1.174
Q9JK42	[Pyruvate dehydrogenase (acetyl-transferring)] kinase isozyme 2, mitochondrial	Pdk2	407	46	6.61	91564.09	105137.32	0.033	1.148
Q99MQ4	Asporin	Aspn	373	42.5	8.57	185649.01	271089.43	0.001	1.460
Q8JZV9	3-hydroxybutyrate dehydrogenase type 2	Bdh2	245	26.7	7.99	126988.93	159297.32	0.047	1.254
Q8BP56	Protein-glucosylgalactosylhydroxylysine glucosidase	Pgghg	690	76.4	5.33	146666.18	196684.96	0.016	1.341
Q8CGB3	Uveal autoantigen with coiled-coil domains and ankyrin repeats	Uaca	1411	160.7	7.2	65402.58	81412.49	0.000	1.245
Q60751	Insulin-like growth factor 1 receptor	Igf1r	1373	155.7	5.74	71960.42	87297.39	0.009	1.213
O08997	Copper transport protein ATOX1	Atox1	68	7.3	6.51	2248660.63	1649165.08	0.003	0.733
Q9DCT1	1,5-anhydro-D-fructose reductase	Akr1e2	301	34.4	7.33	792571.97	673835.40	0.012	0.850
Q61249	Immunoglobulin-binding protein 1	Igbp1	340	38.9	6.18	156783.96	144939.95	0.007	0.924
Q8BL95	Cilia- and flagella-associated protein 298	Cfap298	290	33.2	6.67	126013.91	104361.64	0.038	0.828

Q9D903	Probable rRNA-processing protein EBP2	Ebna1bp2	306	34.7	10.08	314258.60	262026.35	0.011	0.834
Q7TSY8	Shugoshin 2	Sgo2	1164	130.2	8.76	102552.91	78745.79	0.004	0.768
Q9DB43	Zinc finger protein-like 1	Zfpl1	310	34.1	8.19	332975.29	273768.43	0.012	0.822
Q3USJ8	F-BAR and double SH3 domains protein 2	Fchsd2	740	84.2	5.66	82283.94	92633.24	0.030	1.126
Q9R0D8	WD repeat-containing protein 54	Wdr54	334	35.6	6.21	283032.69	216306.41	0.004	0.764
Q8QZY6	Tetraspanin-14	Tspan14	270	30.7	6.51	148940.32	171705.82	0.003	1.153
Q91WV0	Protein Dr1	Dr1	176	19.4	4.75	165119.10	127425.99	0.004	0.772
Q9JJ94	Sjoegren syndrome nuclear autoantigen 1 homolog	Ssna1	119	13.5	5.68	227891.78	146011.67	0.000	0.641
Q8BVL9	Janus kinase and microtubule-interacting protein 1	Jakmip1	626	73.1	6.09	119148.93	148374.37	0.012	1.245
Q8CC12	Codanin-1	Cdan1	1239	135.8	7.25	79819.88	74482.23	0.020	0.933
O70494	Transcription factor Sp3	Sp3	783	82.3	5.26	37842.17	45919.19	0.038	1.213
Q91VN4	MICOS complex subunit Mic25	Chchd6	273	29.8	8.41	199810.16	152724.49	0.038	0.764
Q8CIB6	Transmembrane protein 230	Tmem230	120	13.2	9.31	238359.72	196070.04	0.029	0.823
Q6NS59	Protein FAM135A	Fam135a	1506	166.8	5.47	101494.02	90055.87	0.012	0.887
Q80W49	Beta/gamma crystallin domain-containing protein 3	Crybg3	1005	114.2	5.67	320942.20	276544.04	0.006	0.862
O08848	60 kDa SS-A/Ro ribonucleoprotein	RO60	538	60.1	7.9	171303.83	183920.42	0.037	1.074
Q8BJ56	Patatin-like phospholipase domain-containing protein 2	Pnpla2	486	53.6	6.46	38139.22	50960.87	0.045	1.336
P97863	Nuclear factor 1 B-type	Nfib	570	63.5	8.66	47882.27	61938.95	0.015	1.294
B8JK39	Integrin alpha-9	Itga9	1036	114.3	6.07	274769.41	352894.17	0.007	1.284
P58283	E3 ubiquitin-protein ligase RNF216	Rnf216	853	97.6	5.01	85112.57	95913.69	0.010	1.127
Q8BG60	Thioredoxin-interacting protein	Txnip	397	44.3	6.87	128361.06	160766.88	0.007	1.252
Q8VDP3	[F-actin]-monooxygenase MICAL1	Mical1	1048	116.7	6.05	72162.89	81782.98	0.044	1.133
A0A0G2JE99	Ig-like domain-containing protein	Iglc1	106	11.7	6.27	63396.93	51624.98	0.019	0.814
Q60718	Disintegrin and metalloproteinase domain-containing protein 2	Adam2	735	82.3	6.2	132498.88	81049.63	0.000	0.612
Q505B7	Protein archease	Zbtb8os	168	19.7	4.56	373052.41	407116.77	0.004	1.091
Q924M5	Protein boule-like	Boll	281	30.8	7.47	422656.95	273483.77	0.000	0.647
Q5F201	Cilia- and flagella-associated protein 52	Cfap52	620	68.2	6.81	190507.82	102622.21	0.004	0.539
Q8R3T5	Syntaxin-binding protein 6	Stxbp6	210	23.7	9.19	175239.79	223259.21	0.000	1.274
P02802	Metallothionein-1	Mt1	61	6	7.96	119279.10	69501.30	0.011	0.583
Q5SWT3	Solute carrier family 25 member 35	Slc25a35	300	32.6	9.52	180258.21	160094.18	0.018	0.888
Q8R3P7	Clusterin-associated protein 1	Cluap1	413	47.9	4.84	147257.70	123459.31	0.013	0.838
Q3ULW8	Protein mono-ADP-ribosyltransferase PARP3	Parp3	533	59.9	7.12	77599.55	92385.91	0.030	1.191
Q9Z1P7	KN motif and ankyrin repeat domain-containing protein 3	Kank3	791	84.1	5.2	119019.36	142718.79	0.008	1.199
Q05895	Thrombospondin-3	Thbs3	956	104.1	4.64	47689.93	58701.96	0.012	1.231
Q8CFJ5	UPAR/Ly6 domain-containing protein	Lypd11	196	21	8.13	634666.15	284355.57	0.002	0.448
P59110	Sentrin-specific protease 1	Senp1	640	72.5	8.4	120468.87	137544.16	0.000	1.142
Q8BW49	Tetratricopeptide repeat protein 12	Ttc12	704	78.7	5.64	229085.68	163950.74	0.004	0.716
P28704	Retinoic acid receptor RXR-beta	Rxrb	520	55.8	8.27	262219.74	292187.42	0.015	1.114
Q8C569	Protein FAM118B	Fam118b	351	39.5	6.86	316076.28	224929.05	0.003	0.712
P35803	Neuronal membrane glycoprotein M6-b	Gpm6b	328	36.2	6.14	222785.10	165105.11	0.001	0.741
Q9EPK2	Protein XRP2	Rp2	347	39.4	5.24	107159.18	126744.77	0.000	1.183
P47915	60S ribosomal protein L29	Rpl29	160	17.6	11.84	774763.54	633625.63	0.040	0.818
Q9D9M5	Pyridoxal phosphate phosphatase PHOSPHO2	Phospho2	241	27.5	6.54	152378.28	132277.08	0.006	0.868
P46656	Adrenodoxin, mitochondrial	Fdx1	188	20.1	5.62	598801.65	386070.21	0.000	0.645
Q5M8N0	CB1 cannabinoid receptor-interacting protein 1	Cnrip1	164	18.6	7.96	106519.54	133116.73	0.023	1.250
Q570Y9	DEP domain-containing mTOR-interacting protein	Deptor	409	46.1	7.91	125761.75	179809.78	0.014	1.430
Q32NY8	BHLH domain-containing protein	Tcfl5	489	51.9	6.92	196136.66	129475.76	0.000	0.660
F6U5V1	HTH La-type RNA-binding domain-containing protein	Larp1b	541	59.7	9.13	69880.98	57409.35	0.013	0.822
Q61624	Zinc finger protein 148	Znf148	794	88.7	6.48	90142.54	111543.29	0.024	1.237
Q9WV72	Ankyrin repeat and SOCS box protein 3	Asb3	525	58.2	5.9	36220.67	32118.02	0.025	0.887
Q9QZZ6	Dermatopontin	Dpt	201	24	4.83	94783.12	133589.79	0.026	1.409
Q3UJ22	INT_SG_DDX_CT_C domain-containing protein	Gm648	213	23.4	8.35	72781.72	55232.69	0.004	0.759
Q4JIM5	Tyrosine-protein kinase ABL2	Abl2	1182	128.1	7.84	26505.03	31139.16	0.022	1.175
Q8R368	Dynein assembly factor 4, axonemal	Dnaaf4	420	48.1	8.69	40128.83	29655.07	0.006	0.739
Q3UDK1	TRAF-type zinc finger domain-containing protein 1	Trafd1	580	64.2	5.48	169045.57	141777.35	0.003	0.839
P97364	Selenide, water dikinase 2	Sephs2	452	47.8	6.06	303485.59	237559.16	0.020	0.783
Q3UDW8	Heparan-alpha-glucosaminide N-acetyltransferase	Hgsnat	656	72.5	8.27	107665.59	76861.32	0.000	0.714
Q8C6B9	Active regulator of SIRT1	Rps19bp1	143	16	10.83	77093.16	63005.89	0.001	0.817
Q9D945	Protein LLP homolog	Llph	130	15.4	10.36	80922.24	64550.75	0.041	0.798
P28650	Adenylosuccinate synthetase isozyme 1	Adss1	457	50.2	8.43	34697.94	39102.44	0.039	1.127
Q99J27	Acetyl-coenzyme A transporter 1	Slc33a1	550	61	8.16	160346.78	128435.21	0.005	0.801
Q812A5	Proline-rich protein 5	Prr5	387	42.5	7.84	47419.26	52102.11	0.048	1.099
Q3U0J8	TBC1 domain family member 2B	Tbc1d2b	965	109.9	6.02	74662.40	90340.65	0.004	1.210

B1ASA5	Zinc finger protein 362	Zfp362	418	45.6	9.64	25022.36	29253.68	0.040	1.169
Q8BPI1	Protein kintoun	Dnaaf2	814	88.3	4.96	58078.02	45911.44	0.031	0.791
P63300	Selenoprotein W	Selenow	88	9.7	8.72	328108.76	210595.08	0.001	0.642
Q9D1K7	UPF0687 protein C20orf27 homolog	MGI:1914576	174	19.5	6.68	100440.09	66756.69	0.001	0.665
Q78XF5	Oligosaccharyltransferase complex subunit OSTC	Ostc	149	16.8	9.13	458066.94	384717.34	0.000	0.840
Q6DYE8	Ectonucleotide pyrophosphatase/phosphodiesterase family member 3	Enpp3	874	98.6	6.52	17558.63	21510.80	0.018	1.225
Q3TQI7	Telomere length and silencing protein 1 homolog	MGI:2385132	289	33.5	6.34	169297.99	156953.97	0.038	0.927
Q9WUR9	Adenylate kinase 4, mitochondrial	Ak4	223	25	7.53	175553.72	202997.24	0.010	1.156
O70579	Peroxisomal membrane protein PMP34	Slc25a17	307	34.4	10.11	28941.85	24225.64	0.046	0.837
Q924X7	Serine/threonine-protein kinase 33	Stk33	491	54.4	6.43	194388.66	133521.82	0.001	0.687
Q1A3B0	Ceramide synthase 3	Cers3	383	46.1	8.13	494594.63	248839.42	0.000	0.503
P28661	Septin-4	Septin4	478	54.9	5.87	175275.71	195531.47	0.005	1.116
Q810N3	UPAR/Ly6 domain-containing protein	Lypd10	246	26.5	6.81	604080.10	242390.44	0.005	0.401
Q05A62	Dynein light chain 1, axonemal	Dnal1	190	21.5	5.76	29430.98	21681.81	0.023	0.737
Q3V140	Acrosin-binding protein	Acrbp	540	61.1	4.98	216589.00	97246.72	0.016	0.449
Q7TSF4	Leucine-rich repeat-containing protein 75A	Lrrc75a	339	37.6	8.76	176548.24	197347.28	0.043	1.118
G5E8L7	Glycosylphosphatidylinositol-anchored molecule-like 2	Gml2	176	19.9	5.54	718912.35	521753.68	0.005	0.726
P62046	Leucine-rich repeat and calponin homology domain-containing protein 1	Lrch1	709	79	6.13	55473.56	65834.26	0.011	1.187
Q78YY6	DnaJ homolog subfamily C member 15	Dnajc15	149	15.9	9.99	198478.30	161265.29	0.044	0.813
Q7TNH6	Nephrocystin-3	Nphp3	1325	150.2	6.51	25640.37	20778.26	0.007	0.810
B2RY50	Armadillo repeat-containing protein 4	Armc4	1037	115.2	6.09	112786.03	82131.51	0.003	0.728
Q9JKW0	ADP-ribosylation factor-like protein 6-interacting protein 1	Arl6ip1	203	23.4	9.32	520396.63	372951.43	0.022	0.717
Q9DB60	Prostamide/prostaglandin F synthase	Prxl2b	201	21.7	6.74	143685.24	160450.62	0.026	1.117
Q8R2R1	Protein O-mannosyl-transferase 1	Pomt1	746	85.2	8.29	220785.94	175959.98	0.004	0.797
Q9DAM9	Fibronectin type 3 and ankyrin repeat domains 1 protein	Fank1	344	38.2	7.43	90725.89	63362.07	0.002	0.698
Q5ND04	Heat shock factor protein 5	Hsf5	624	67.3	7.08	650066.90	409816.48	0.002	0.630
E9Q7R9	Cilia- and flagella-associated protein 43	Cfap43	1682	193.3	5.62	8411.51	5472.87	0.049	0.651
P82347	Delta-sarcoglycan	Sgcd	289	32.1	9.1	86816.59	97429.05	0.008	1.122
Q9CSB4	Partitioning defective 3 homolog B	Pard3b	1203	132.7	8.56	155517.62	131590.15	0.011	0.846
P97366	Ecotropic viral integration site 5 protein	Evi5	809	92.9	6.16	139326.42	162031.70	0.004	1.163
E9Q1Z2	ENTH domain-containing protein	Enthd1	618	68.4	6.84	39483.67	25706.76	0.000	0.651
Q9ERU3	Zinc finger protein 22	Znf22	237	27.3	10.29	337762.60	441095.18	0.002	1.306
A0A1Y7VNZ0	WD_REPEATS_REGION domain-containing protein	Dcaf4	532	59.3	8.24	140086.62	149944.07	0.049	1.070
Q9DAJ5	Dynein light chain roadblock-type 2	Dynlrb2	96	10.9	7.44	134938.21	83090.76	0.006	0.616
P97440	Histone RNA hairpin-binding protein	Slbp	275	31.6	6.8	258490.07	212057.87	0.026	0.820
A0A0B4J1H8	Ig-like domain-containing protein	Igkv1-133	120	13.2	7.97	32076.45	48784.65	0.034	1.521
O54974	Galectin-7	Lgals7	136	15.2	7.25	40310.87	52243.94	0.008	1.296
Q8BGX1	PC-esterase domain-containing protein 1B	Pced1b	433	49.9	8.28	97125.90	110473.01	0.029	1.137
Q9D273	Corrinoid adenosyltransferase	Mmab	237	26.3	9.2	189572.05	207609.69	0.010	1.095
Q91ZH7	Phospholipase ABHD3	Abhd3	411	46.2	7.49	176198.30	154503.18	0.009	0.877
B5TVM2	Immunoglobulin-like domain-containing receptor 2	Ildr2	661	73.2	7.68	182171.96	281541.95	0.041	1.545
P30051	Transcriptional enhancer factor TEF-1	Tead1	426	47.9	8.15	94329.98	121242.39	0.003	1.285
Q61169	Transcription factor GATA-6	Gata6	589	59.3	8.31	57620.17	64084.67	0.043	1.112
P70274	Selenoprotein P	Selenop	380	42.7	7.09	70721.87	59119.16	0.040	0.836
P61219	DNA-directed RNA polymerases I, II, and III subunit RPABC2	Polr2f	127	14.5	4.22	561899.20	637155.02	0.044	1.134
Q9EQK7	Protein-S-isoprenylcysteine O-methyltransferase	Icmt	283	31.8	8.18	215665.73	184262.91	0.047	0.854
Q99LU0	Charged multivesicular body protein 1b-1	Chmp1b1	199	22.1	8.1	230811.08	260080.65	0.025	1.127
E9Q0B5	Fc fragment of IgG-binding protein	Fcgbp	2583	275	5.03	1665479.61	2641454.13	0.010	1.586
Q9D8P7	General transcription factor 3C polypeptide 6	Gtf3c6	227	25.5	4.22	189215.97	220780.07	0.006	1.167
Q8K2A1	PTB domain-containing engulfment adapter protein 1	Gulp1	304	34.4	7.9	91005.61	111570.98	0.000	1.226
Q8R1L4	ER lumen protein-retaining receptor 3	Kdelr3	214	25.1	9.06	209359.79	178130.33	0.031	0.851
Q9CZL2	Uncharacterized protein FAM241A	Fam241a	131	14.5	4.97	238496.01	204646.30	0.007	0.858
Q9DA69	Intraflagellar transport protein 43 homolog	Ift43	206	23.5	4.69	30443.35	21450.17	0.004	0.705
Q9CXV1	Succinate dehydrogenase [ubiquinone] cytochrome b small subunit, mitochondrial	Sdhd	159	17	9.1	1730406.10	1520655.31	0.025	0.879
Q9DCK3	Tetraspanin-4	Tspan4	238	26	5.48	171546.07	245413.68	0.000	1.431
A2AFS3	UPF0577 protein KIAA1324	Kiaa1324	1009	110.6	6.38	69750.38	44194.29	0.003	0.634
Q922S4	cGMP-dependent 3',5'-cyclic phosphodiesterase	Pde2a	939	105.6	5.41	77976.78	95523.13	0.000	1.225
O55186	CD59A glycoprotein	Cd59a	123	13.6	7.47	22259.22	35418.20	0.001	1.591
O88551	Claudin-1	Cldn1	211	22.9	7.91	29464.35	39084.33	0.048	1.326
P00184	Cytochrome P450 1A1	Cyp1a1	524	59.2	8.06	142981.27	96941.24	0.004	0.678
D3Z2X2	Dynein heavy chain domain 1	Dnhd1	4750	536.2	7.06	983876.39	1354772.18	0.000	1.377
Q3UC65	Arginine/serine-rich protein 1	Rsrp1	298	34.5	11.77	1006925.85	801239.92	0.021	0.796
Q9D2H1	Cytochrome c oxidase subunit 7B2	Cox7b2	82	9.4	9.66	554426.67	228894.81	0.003	0.413

Q9CQ66	Tctex1 domain-containing protein 2	Tctex1d2	144	16.5	5.19	354637.37	214758.50	0.009	0.606
Q9Z1M0	P2X purinoceptor 7	P2rx7	595	68.3	8.22	259432.80	356158.85	0.023	1.373
Q9CQ91	NADH dehydrogenase [ubiquinone] 1 alpha subcomplex subunit 3	Ndufa3	84	9.3	8.47	839711.52	709459.33	0.023	0.845
Q8BH35	Complement component C8 beta chain	C8b	589	66.2	7.77	345262.30	270116.09	0.018	0.782
