# Supplementary material for: Polyubiquitin gene Ubb is required for upregulation of Piwi protein level during mouse testis development
Source: Cell Death Discov. 2021 Jul 26;7:194. doi: 10.1038/s41420-021-00581-2 (PMC8313548; doi:10.1038/s41420-021-00581-2)
Supplement: Supplementary file 3 — Supplementary table 3 [file 41420_2021_581_MOESM3_ESM.pdf]

Table of Contents

Accession	UniProt identifier
FC	Ratio of Ubb K.O reporter ion average intensity versus WT reporter ion average intensity.
	#N/A is a protein that has been identified by LC-MS/MS analysis, but does not show a significant difference in expression level.
Function in stage	The spermatogeneic stage in which the gene functions based on the SpermatogenesisOnline database.
Function in cell type	The spermatogeneic cell type in which the gene functions based on the database.
Interact with 27 Ubb related proteins	A list of interacting proteins among 24 Ubb-related proteins.

Supplemental Table S3. Among the identified proteins, information on proteins that overlap with the SpermatogenesisOnline database

Accession	Protein name (403)	Gene name	FC	Function in stage	Function in cell type	Interact with 24 Ubb related proteins
P00342	L-lactate dehydrogenase C chain	Ldhc	0.36	meiotic/	spermatocyte/	#N/A
P27786	Steroid 17-alpha-hydroxylase/17,20 lyase	Cyp17a1	0.43	postmeiotic/meiotic/premeiotic/	leydig_cell/	#N/A
Q3V140	Acrosin-binding protein	Acrbp	0.45	postmeiotic/	spermatid/	#N/A
Q9JMB7	Piwi-like protein 1	Piwil1	0.46	meiotic/postmeiotic/	spermatocyte/spermatid/	Hsp90aa1
P17156	Heat shock-related 70 kDa protein 2	Hspa2	0.46	meiotic/postmeiotic/	spermatocyte/spermatid/	Hsp90aa1; Eef2
P52194	Calmegin	Clgn	0.50	meiotic/	spermatocyte/	Hsp90aa1
P35487	Pyruvate dehydrogenase E1 component subunit alpha, testis-specific form, mitochondrial	Pdha2	0.53	meiotic/postmeiotic/	spermatocyte/spermatid/	#N/A
Q07133	Histone H1t	H1-6	0.56	meiotic/postmeiotic/	spermatocyte/spermatid/	#N/A
Q9Z2C8	Y-box-binding protein 2	Ybx2	0.59	meiotic/postmeiotic/	spermatocyte/spermatid/	#N/A
Q9Z0R9	Acyl-CoA 6-desaturase	Fads2	0.60	postmeiotic/	spermatid/	#N/A
P61407	Tudor domain-containing protein 6	Tdrd6	0.60	postmeiotic/	spermatid/	#N/A
P48722	Heat shock 70 kDa protein 4L	Hspa4l	0.62	meiotic/postmeiotic/	spermatocyte/spermatid/	Hsp90aa1; Eef2; Rpsa
Q8CDG1	Piwi-like protein 2	Piwil2	0.62	meiotic/postmeiotic/	spermatocyte/spermatid/	Hsp90aa1
Q99MV1	Tudor domain-containing protein 1	Tdrd1	0.63	meiotic/postmeiotic/	spermatocyte/spermatid/	Hsp90aa1; Eef1a1; Eef2; Uchl5
Q924M5	Protein boule-like	Boll	0.65	postmeiotic/	spermatid/	#N/A
Q924X7	Serine/threonine-protein kinase 33	Stk33	0.69	meiotic/postmeiotic/	spermatocyte/spermatid/	#N/A
Q9QVN7	Transcription elongation factor A protein 2	Tcea2	0.70	meiotic/	spermatocyte/	#N/A
Q9DAM9	Fibronectin type 3 and ankyrin repeat domains 1 protein	Fank1	0.70	meiotic/postmeiotic/	spermatocyte/spermatid/	#N/A
Q8VDF2	E3 ubiquitin-protein ligase UHRF1	Uhrf1	0.71	meiotic/premeiotic/	spermatogonium/spermatocyte/spermatid/	Hist1h4j
Q61496	ATP-dependent RNA helicase DDX4	Ddx4	0.72	postmeiotic/	spermatid/	Hsp90aa1
Q8K1H1	Tudor domain-containing protein 7	Tdrd7	0.76	postmeiotic/	spermatid/	#N/A
P07901	Heat shock protein HSP 90-alpha	Hsp90aa1	0.76	meiotic/	spermatocyte/	Eef1a1; Hist1h4j; Eef2; Psmd4; Rpl3; Rps6; Rpsa
Q7TSY8	Shugoshin 2	Sgo2	0.77	meiotic/	spermatocyte/	#N/A
Q80XI3	Eukaryotic translation initiation factor 4 gamma 3	Eif4g3	0.79	meiotic/	spermatocyte/	Eef2; Rps6
Q14BI7	ATP-dependent RNA helicase TDRD9	Tdrd9	0.80	meiotic/	spermatocyte/	Hsp90aa1
Q9CUU3	Synaptonemal complex protein 2	Sycp2	0.80	meiotic/	spermatocyte/	#N/A
Q99MV7	RING finger protein 17	Rnf17	0.82	postmeiotic/	spermatid/	#N/A
Q3V089	RNA-binding protein 44	Rbm44	0.83	meiotic/	spermatocyte/	#N/A
P70274	Selenoprotein P	Selenop	0.84	postmeiotic/	leydig_cell/	#N/A
Q9QY53	Nephrocystin-1	Nphp1	0.87	postmeiotic/	spermatid/elongated_spermatids/	Hsp90aa1
Q91V12	Cytosolic acyl coenzyme A thioester hydrolase	Acot7	0.87	meiotic/postmeiotic/	spermatocyte/spermatid/	Rpl22
Q8C8R3	Ankyrin-2	Ank2	0.90	meiotic/	spermatocyte/	#N/A
Q5SSW2	Proteasome activator complex subunit 4	Psme4	0.90	meiotic/postmeiotic/	spermatocyte/spermatid/	Psmd2; Psmd4; Psma8; Uchl5
P70168	Importin subunit beta-1	Kpnb1	0.90	meiotic/premeiotic/	spermatogonium/spermatocyte/	Hsp90aa1; Eef1a1; Psmd2; Rpl6; Rpl4; Rpl15
Q9Z1S0	Mitotic checkpoint serine/threonine-protein kinase BUB1 beta	Bub1b	0.92	meiotic/	spermatocyte/	Psmd2; Psmd4; Psma8
P28740	Kinesin-like protein KIF2A	Kif2a	0.93	meiotic/postmeiotic/	spermatocyte/spermatid/	#N/A
Q8R3N6	THO complex subunit 1	Thoc1	1.11	meiotic/	spermatocyte/	#N/A
P28704	Retinoic acid receptor RXR-beta	Rxrb	1.11	postmeiotic/meiotic/premeiotic/	spermatogonium/spermatocyte/spermatid/leydig_cell/sertoli_cell/	#N/A
O35350	Calpain-1 catalytic subunit	Capn1	1.12	meiotic/postmeiotic/	spermatocyte/spermatid/elongated_spermatids/	#N/A
P40630	Transcription factor A, mitochondrial	Tfam	1.13	postmeiotic/	spermatid/	Uchl5
P56959	RNA-binding protein FUS	Fus	1.17	meiotic/	spermatocyte/	#N/A
O70494	Transcription factor Sp3	Sp3	1.21	meiotic/postmeiotic/	spermatocyte/spermatid/	#N/A
O70318	Band 4.1-like protein 2	Epb41l2	1.23	postmeiotic/meiotic/premeiotic/	spermatogonium/spermatocyte/spermatid/sertoli_cell/	#N/A
P46414	Cyclin-dependent kinase inhibitor 1B	Cdkn1b	1.24	meiotic/premeiotic/	sertoli_cell/leydig_cell/	Hsp90aa1; Psmd2; Psmd4
P62806	Histone H4	H4f16	1.30	postmeiotic/	spermatid/	#N/A
Q9WU20	Methylenetetrahydrofolate reductase	Mthfr	1.32	postmeiotic/meiotic/premeiotic/	spermatogonium/spermatocyte/spermatid/elongated_spermatids	#N/A
O88551	Claudin-1	Cldn1	1.33	postmeiotic/meiotic/premeiotic/	sertoli_cell/	#N/A
O55186	CD59A glycoprotein	Cd59a	1.59	postmeiotic/meiotic/premeiotic/	elongated_spermatids/	#N/A
P48678	Prelamin-A/C	Lmna	1.61	meiotic/	spermatocyte/	Hsp90aa1
Q8BUN5	Mothers against decapentaplegic homolog 3	Smad3	1.69	postmeiotic/meiotic/premeiotic/	sertoli_cell/other/	#N/A
P52293	Importin subunit alpha-1	Kpna2	#N/A	postmeiotic/meiotic/premeiotic/	spermatogonium/spermatocyte/spermatid/	Hsp90aa1
Q99MV5	RNA helicase Mov10l1	Mov10l1	#N/A	meiotic/	spermatocyte/	Hsp90aa1
O88643	Serine/threonine-protein kinase PAK 1	Pak1	#N/A	postmeiotic/	spermatid/	Hsp90aa1
Q8VD46	Ankyrin repeat, SAM and basic leucine zipper domain-containing protein 1	Asz1	#N/A	meiotic/	spermatocyte/	Hsp90aa1
P63037	DnaJ homolog subfamily A member 1	Dnaja1	#N/A	postmeiotic/	spermatid/sertoli_cell/	Hsp90aa1
O54946	DnaJ homolog subfamily B member 6	Dnajb6	#N/A	postmeiotic/	spermatid/	Hsp90aa1
P30416	Peptidyl-prolyl cis-trans isomerase FKBP4	Fkbp4	#N/A	meiotic/postmeiotic/	spermatocyte/spermatid/	Hsp90aa1
Q8BVN9	Protein maelstrom homolog	Mael	#N/A	meiotic/	spermatocyte/	Hsp90aa1
P63005	Platelet-activating factor acetylhydrolase IB subunit alpha	Pafah1b1	#N/A	meiotic/postmeiotic/	spermatocyte/spermatid/	Hsp90aa1
Q6WKZ8	E3 ubiquitin-protein ligase UBR2	Ubr2	#N/A	meiotic/	spermatocyte/	Hsp90aa1
Q8R5H6	Wiskott-Aldrich syndrome protein family member 1	Wasf1	#N/A	meiotic/postmeiotic/	spermatocyte/elongated_spermatids/spermatid/	Hsp90aa1
Q91YD9	Neural Wiskott-Aldrich syndrome protein	Wasl	#N/A	postmeiotic/	sertoli_cell/	Hsp90aa1
Q99NB9	Splicing factor 3B subunit 1	Sf3b1	#N/A	postmeiotic/meiotic/premeiotic/	spermatogonium/sertoli_cell/other/	Eef2
Q8BKC5	Importin-5	Ipo5	#N/A	postmeiotic/	spermatid/	Eef2
Q8K1L5	E3 ubiquitin-protein ligase PPP1R11	Ppp1r11	#N/A	postmeiotic/meiotic/premeiotic/	spermatocyte/spermatid/sertoli_cell/	Rps15a
Q8C7E9	Cleavage stimulation factor subunit 2 tau variant	Cstf2t	#N/A	meiotic/postmeiotic/	spermatocyte/spermatid/elongated_spermatids/	Rpsa
Q8K409	DNA polymerase beta	Polb	#N/A	meiotic/	spermatocyte/	Rps19
O89086	RNA-binding protein 3	Rbm3	#N/A	postmeiotic/meiotic/premeiotic/	sertoli_cell/	Rpl4
Q8BX90	Fibronectin type-III domain-containing protein 3A	Fndc3a	#N/A	postmeiotic/	spermatogonium/leydig_cell/	Rpl23
Q7TMY8	E3 ubiquitin-protein ligase HUWE1	Huwe1	#N/A	meiotic/premeiotic/	spermatogonium/spermatocyte/	Psmd4
Q3TCH7	Cullin-4A	Cul4a	#N/A	meiotic/	spermatocyte/	Nae1

Q9Z172	Small ubiquitin-related modifier 3	Sumo3	#N/A	meiotic/	spermatocyte/	Nae1
P23198	Chromobox protein homolog 3	Cbx3	#N/A	meiotic/postmeiotic/	spermatocyte/spermatid/	Hist1h4j
Q6PDK2	Histone-lysine N-methyltransferase 2D	Kmt2d	#N/A	premeiotic/	spermatogonium/	Hist1h4j
Q8K2F0	Bromodomain-containing protein 3	Brd3	#N/A	postmeiotic/	spermatid/	Hist1h4j
Q91Y44	Bromodomain testis-specific protein	Brdt	#N/A	meiotic/postmeiotic/	spermatocyte/spermatid/	Hist1h4j
Q9ESK4	Inhibitor of growth protein 2	Ing2	#N/A	meiotic/	spermatocyte/	Hist1h4j
Q80Y84	Lysine-specific demethylase 5B	Kdm5b	#N/A	meiotic/	spermatocyte/	Hist1h4j
Q9EQQ0	Histone-lysine N-methyltransferase SUV39H2	Suv39h2	#N/A	meiotic/postmeiotic/	spermatocyte/spermatid/	Hist1h4j
Q64127	Transcription intermediary factor 1-alpha	Trim24	#N/A	postmeiotic/	spermatid/	Hist1h4j
Q62318	Transcription intermediary factor 1-beta	Trim28	#N/A	postmeiotic/meiotic/premeiotic/	spermatocyte/spermatid/sertoli_cell/	Hist1h4j
P18572	Basigin	Bsg	#N/A	postmeiotic/meiotic/premeiotic/	spermatogonium/spermatocyte/spermatid/	#N/A
Q08369	Transcription factor GATA-4	Gata4	#N/A	premeiotic/	leydig_cell/sertoli_cell/	#N/A
O70325	Phospholipid hydroperoxide glutathione peroxidase	Gpx4	#N/A	meiotic/postmeiotic/	spermatocyte/spermatid/elongated_spermatids/	#N/A
P02772	Alpha-fetoprotein	Afp	#N/A	meiotic/	spermatocyte/	#N/A
P24549	Retinal dehydrogenase 1	Aldh1a1	#N/A	premeiotic/	leydig_cell/sertoli_cell/	#N/A
Q9Z1R2	Large proline-rich protein BAG6	Bag6	#N/A	meiotic/	spermatocyte/	#N/A
O08529	Calpain-2 catalytic subunit	Capn2	#N/A	postmeiotic/	spermatid/elongated_spermatids/	#N/A
P11087	Collagen alpha-1(I) chain	Col1a1	#N/A	premeiotic/	spermatogonium/	#N/A
Q01149	Collagen alpha-2(I) chain	Col1a2	#N/A	premeiotic/	spermatogonium/	#N/A
P06797	Cathepsin L1	Ctsl	#N/A	meiotic/	sertoli_cell/	#N/A
P70372	ELAV-like protein 1	Elavl1	#N/A	postmeiotic/	spermatid/	#N/A
Q69ZF3	Non-lysosomal glucosylceramidase	Gba2	#N/A	postmeiotic/	spermatid/	#N/A
P23242	Gap junction alpha-1 protein	Gja1	#N/A	postmeiotic/meiotic/premeiotic/	sertoli_cell/leydig_cell/	#N/A
P12265	Beta-glucuronidase	Gusb	#N/A	meiotic/postmeiotic/	spermatocyte/elongated_spermatids/	#N/A
P52927	High mobility group protein HMGI-C	Hmga2	#N/A	postmeiotic/	spermatocyte/spermatid/	#N/A
P09055	Integrin beta-1	Itgb1	#N/A	premeiotic/	spermatogonium/	#N/A
P54310	Hormone-sensitive lipase	Lipe	#N/A	meiotic/postmeiotic/	spermatocyte/spermatid/	#N/A
O08807	Peroxiredoxin-4	Prdx4	#N/A	postmeiotic/	spermatid/	#N/A
P35235	Tyrosine-protein phosphatase non-receptor type 11	Ptpn11	#N/A	postmeiotic/meiotic/premeiotic/	other/	#N/A
P13405	Retinoblastoma-associated protein	Rb1	#N/A	postmeiotic/meiotic/premeiotic/	sertoli_cell/	#N/A
P70458	Plasma serine protease inhibitor	Serpina5	#N/A	postmeiotic/meiotic/premeiotic/	spermatocyte/spermatid/elongated_spermatids/sertoli_cell/	#N/A
Q62432	Mothers against decapentaplegic homolog 2	Smad2	#N/A	meiotic/	spermatocyte/leydig_cell/sertoli_cell/	#N/A
Q04887	Transcription factor SOX-9	Sox9	#N/A	postmeiotic/meiotic/premeiotic/	sertoli_cell/	#N/A
P55144	Tyrosine-protein kinase receptor TYRO3	Tyro3	#N/A	postmeiotic/meiotic/premeiotic/	sertoli_cell/	#N/A
P61164	Alpha-centractin	Actr1a	#N/A	postmeiotic/	spermatid/	Hsp90aa1; Eef1a1
Q62167	ATP-dependent RNA helicase DDX3X	Ddx3x	#N/A	meiotic/	spermatocyte/	Hsp90aa1; Eef2
O88879	Apoptotic protease-activating factor 1	Apaf1	#N/A	premeiotic/	spermatogonium/	Hsp90aa1
Q00993	Tyrosine-protein kinase receptor UFO	Axl	#N/A	postmeiotic/meiotic/premeiotic/	sertoli_cell/	Hsp90aa1
P09803	Cadherin-1	Cdh1	#N/A	premeiotic/	spermatogonium/	Hsp90aa1
P49615	Cyclin-dependent-like kinase 5	Cdk5	#N/A	meiotic/	spermatocyte/sertoli_cell/	Hsp90aa1
O54833	Casein kinase II subunit alpha'	Csnk2a2	#N/A	postmeiotic/	spermatid/	Hsp90aa1
Q60598	Src substrate cortactin	Cttn	#N/A	postmeiotic/	spermatid/	Hsp90aa1
Q91XW8	Inactive peptidyl-prolyl cis-trans isomerase FKBP6	Fkbp6	#N/A	meiotic/	spermatocyte/	Hsp90aa1
P39688	Tyrosine-protein kinase Fyn	Fyn	#N/A	postmeiotic/meiotic/premeiotic/	sertoli_cell/	Hsp90aa1
P05532	Mast/stem cell growth factor receptor Kit	Kit	#N/A	postmeiotic/meiotic/premeiotic/	spermatogonium/spermatocyte/spermatid/other/	Hsp90aa1
P08228	Superoxide dismutase [Cu-Zn]	Sod1	#N/A	postmeiotic/meiotic/premeiotic/	elongated_spermatids/	Hsp90aa1
O89090	Transcription factor Sp1	Sp1	#N/A	meiotic/	spermatocyte/	Hsp90aa1
Q921F2	TAR DNA-binding protein 43	Tardbp	#N/A	meiotic/	spermatocyte/spermatid/	Hsp90aa1
Q9R0P9	Ubiquitin carboxyl-terminal hydrolase isozyme L1	Uchl1	#N/A	postmeiotic/meiotic/premeiotic/	spermatogonium/spermatocyte/elongated_spermatids/sertoli_ce	Hsp90aa1
P42859	Huntingtin	Htt	#N/A	meiotic/	spermatocyte/	Hsp90aa1
Q9WTK7	Serine/threonine-protein kinase STK11	Stk11	#N/A	postmeiotic/	spermatid/elongated_spermatids/	Hsp90aa1; Rps6
P83870	PHD finger-like domain-containing protein 5A	Phf5a	#N/A	meiotic/	spermatocyte/	Eef2; Rpl7a
Q9Z321	DNA topoisomerase 3-beta-1	Top3b	#N/A	meiotic/	spermatocyte/	Eef2; Rpl23
Q8K337	Type II inositol 1,4,5-trisphosphate 5-phosphatase	Inpp5b	#N/A	meiotic/	sertoli_cell/	Rpsa
P63280	SUMO-conjugating enzyme UBC9	Ube2i	#N/A	meiotic/	spermatocyte/	Nae1
Q7JJ13	Bromodomain-containing protein 2	Brd2	#N/A	postmeiotic/meiotic/premeiotic/	spermatogonium/spermatocyte/spermatid/	Hist1h4j
Q9ESU6	Bromodomain-containing protein 4	Brd4	#N/A	premeiotic/	spermatogonium/	Hist1h4j
O88508	DNA (cytosine-5)-methyltransferase 3A	Dnmt3a	#N/A	meiotic/premeiotic/	spermatogonium/spermatocyte/	Hist1h4j
O88509	DNA (cytosine-5)-methyltransferase 3B	Dnmt3b	#N/A	Premeiotic/	spermatogonium/	Hist1h4j
Q9Z148	Histone-lysine N-methyltransferase EHMT2	Ehmt2	#N/A	meiotic/	spermatocyte/	Hist1h4j
P14602	Heat shock protein beta-1	Hspb1	#N/A	meiotic/	spermatocyte/	Hsp90aa1; Eef1a1
Q61166	Microtubule-associated protein RP/EB family member 1	Mapre1	#N/A	postmeiotic/meiotic/premeiotic/	sertoli_cell/	Hsp90aa1; Psmd4; Rps15a
P40240	CD9 antigen	Cd9	#N/A	postmeiotic/meiotic/premeiotic/	spermatogonium/spermatocyte/spermatogonium/	Hsp90aa1; Rpl13
P63166	Small ubiquitin-related modifier 1	Sumo1	#N/A	meiotic/	spermatocyte/	Hsp90aa1; Nae1
P61957	Small ubiquitin-related modifier 2	Sumo2	#N/A	meiotic/	spermatocyte/	Hsp90aa1; Nae1
P19091	Androgen receptor	Ar	#N/A	postmeiotic/meiotic/premeiotic/	spermatogonium/spermatocyte/sertoli_cell/leydig_cell/other/	Hsp90aa1; Hist1h4j
Q62388	Serine-protein kinase ATM	Atm	#N/A	meiotic/premeiotic/	spermatogonium/spermatocyte/	Hsp90aa1; Hist1h4j
Q01147	Cyclic AMP-responsive element-binding protein 1	Creb1	#N/A	postmeiotic/meiotic/premeiotic/	spermatogonium/spermatocyte/spermatid/	Hsp90aa1; Hist1h4j
O09106	Histone deacetylase 1	Hdac1	#N/A	meiotic/	spermatocyte/	Hsp90aa1; Hist1h4j
Q923E4	NAD-dependent protein deacetylase sirtuin-1	Sirt1	#N/A	postmeiotic/meiotic/premeiotic/	spermatogonium/spermatocyte/elongated_spermatids/sertoli_ce	Hsp90aa1; Hist1h4j
P30285	Cyclin-dependent kinase 4	Cdk4	#N/A	meiotic/	spermatocyte/leydig_cell/	Hsp90aa1; Rps6
P63085	Mitogen-activated protein kinase 1	Mapk1	#N/A	meiotic/	spermatocyte/	Hsp90aa1; Rps6
P05480	Neuronal proto-oncogene tyrosine-protein kinase Src	Src	#N/A	postmeiotic/meiotic/premeiotic/	leydig_cell/	Hsp90aa1; Rps6
P42227	Signal transducer and activator of transcription 3	Stat3	#N/A	premeiotic/	spermatogonium/	Hsp90aa1; Rps6
Q6EJB6	U3 small nucleolar RNA-associated protein 14 homolog B	Utp14b	#N/A	postmeiotic/meiotic/premeiotic/	spermatogonium/spermatocyte/spermatid/	Rps6; Rpl4; Rps15a
Q80U87	Ubiquitin carboxyl-terminal hydrolase 8	Usp8	#N/A	postmeiotic/	spermatid/	Psmd4; Rpl3; Uchl5

Q00899	Transcriptional repressor protein YY1	Yy1	#N/A	meiotic/	spermatocyte/	Hist1h4j; Uchl5
Q8BSK8	Ribosomal protein S6 kinase beta-1	Rps6kb1	#N/A	meiotic/postmeiotic/	spermatocyte/elongated_spermatids/	Hsp90aa1; Eef2; Rps6
P40338	von Hippel-Lindau disease tumor suppressor	Vhl	#N/A	postmeiotic/meiotic/premeiotic/	elongated_spermatids/sertoli_cell/	Hsp90aa1; Psmd2; Psmd4; Psma8
Q02053	Ubiquitin-like modifier-activating enzyme 1	Uba1	#N/A	postmeiotic/premeiotic/	spermatogonium/spermatid/	Hsp90aa1; Psmd2; Psmd4; Uchl5
P63087	Serine/threonine-protein phosphatase PP1-gamma catalytic subunit	Ppp1cc	#N/A	postmeiotic/meiotic/premeiotic/	spermatid/elongated_spermatids/sertoli_cell/	Hsp90aa1; Rps6; Rpsa
Q3V132	ADP/ATP translocase 4	Slc25a31	#N/A	meiotic/	spermatocyte/	Rpl23; Rpl6; Rps19; Rpl15
Q61316	Heat shock 70 kDa protein 4	Hspa4	#N/A	meiotic/	other/	Hsp90aa1; Eef1a1; Eef2; Rpsa; Psmd4
P24270	Catalase	Cat	#N/A	meiotic/	spermatocyte/	Hsp90aa1; Eef1a1; Eef2; Rpsa
P31750	RAC-alpha serine/threonine-protein kinase	Akt1	#N/A	postmeiotic/premeiotic/	spermatogonium/spermatid/	Hsp90aa1; Eef2; Rps6; Hist1h4j
Q02248	Catenin beta-1	Ctnnb1	#N/A	postmeiotic/meiotic/premeiotic/	sertoli_cell/leydig_cell/	Hsp90aa1; Psmd2; Psma8; Psmd4
P25799	Nuclear factor NF-kappa-B p105 subunit	Nfkb1	#N/A	meiotic/postmeiotic/	spermatocyte/spermatid/elongated_spermatids/	Hsp90aa1; Psmd2; Psma8; Psmd4
P97377	Cyclin-dependent kinase 2	Cdk2	#N/A	meiotic/	spermatocyte/	Hsp90aa1; Hist1h4j; Psmd4; Psmd2
P20029	Endoplasmic reticulum chaperone BiP	Hspa5	#N/A	meiotic/	spermatocyte/	Hsp90aa1; Eef1a1; Eef2; Rps6; Rpsa
Q8R418	Endoribonuclease Dicer	Dicer1	#N/A	postmeiotic/meiotic/premeiotic/	spermatid/sertoli_cell/	Hsp90aa1; Rpl3; Eef2; Rpl4; Rpsa
P11440	Cyclin-dependent kinase 1	Cdk1	#N/A	meiotic/postmeiotic/	spermatocyte/elongated_spermatids/	Hsp90aa1; Psmd2; Hist1h4j; Psmd4; Psma8
Q9WV60	Glycogen synthase kinase-3 beta	Gsk3b	#N/A	meiotic/	spermatocyte/	Hsp90aa1; Psmd2; Eef2; Psmd4; Psma8; Rps6
P54728	UV excision repair protein RAD23 homolog B	Rad23b	#N/A	meiotic/premeiotic/	spermatogonium/spermatocyte/	Rpl13; Psmd2; Rpl23; Rps19; Psmd4; Uchl5; Rps6; Rpl15; Rpl22; Rps15a
P70166	Cytoplasmic polyadenylation element-binding protein 1	Cpeb1	#N/A	meiotic/	spermatocyte/	Rpl13; Rpl3; Rpl23; Rps19; Rpl4; Rpl6; Rps6; Rpl34; Rpl22; Rps15a
Q99L45	Eukaryotic translation initiation factor 2 subunit 2	Eif2s2	#N/A	premeiotic/	spermatogonium/	Rpl13; Eef2; Rpl3; Rpl6; Rps6; Rps19; Eef1a1; Rpl15; Uchl5; Psma8; Rps15a
Q8R317	Ubiquilin-1	Ubqln1	#N/A	postmeiotic/	spermatid/	Rpl13; Psmd4; Rpl23; Rpsa; Rps6; Rps19; Psmd2; Rpl15; Uchl5; Rpl22; Rps15a
Q9Z0N2	Eukaryotic translation initiation factor 2 subunit 3, Y-linked	Eif2s3y	#N/A	premeiotic/	spermatogonium/	Rpl13; Eef2; Rpl23; Rpl4; Rps6; Rpl3; Eef1a1; Rps19; Rpl6; Rpl7a; Rpsa; Psma8; Rpl15; Rps15a
P53564	Homeobox protein cut-like 1	Cux1	#N/A	postmeiotic/	spermatid/sertoli_cell/	#N/A
O88735	Ensconsin	Map7	#N/A	postmeiotic/	spermatid/	#N/A
Q4ZGD8	Nuclear RNA export factor 2	Nxf2	#N/A	meiotic/premeiotic/	spermatogonium/spermatocyte/	#N/A
Q9Z280	Phospholipase D1	Pld1	#N/A	postmeiotic/meiotic/premeiotic/	spermatocyte/spermatid/leydig_cell/	#N/A
P54279	Mismatch repair endonuclease PMS2	Pms2	#N/A	meiotic/	spermatocyte/	#N/A
Q9EQN9	Thiamine transporter 1	Slc19a2	#N/A	meiotic/	spermatocyte/	#N/A
Q00262	Syntaxin-2	Stx2	#N/A	meiotic/postmeiotic/	spermatocyte/spermatid/leydig_cell/	#N/A
Q9JJ11	Transforming acidic coiled-coil-containing protein 3	Tacc3	#N/A	meiotic/	spermatocyte/	#N/A
F8VPN2	Testis-expressed protein 15	Tex15	#N/A	meiotic/	spermatocyte/	#N/A
P22561	Wilms tumor protein homolog	Wt1	#N/A	postmeiotic/meiotic/premeiotic/	sertoli_cell/	#N/A
Q99PP2	Zinc finger protein 318	Znf318	#N/A	meiotic/	spermatocyte/	#N/A
Q5VCS6	Tudor domain-containing protein 5	Tdrd5	#N/A	meiotic/postmeiotic/	spermatocyte/spermatid/	#N/A
Q9CZU3	Exosome RNA helicase MTR4	Mtrex	#N/A	postmeiotic/	spermatid/	#N/A
Q9JKB3	Y-box-binding protein 3	Ybx3	#N/A	meiotic/postmeiotic/	spermatocyte/spermatid/elongated_spermatids/	#N/A
Q9WVP6	Poly(A) polymerase beta	Papolb	#N/A	postmeiotic/	spermatid/	#N/A
Q9D2J4	V-set and immunoglobulin domain-containing protein 1	Vsig1	#N/A	postmeiotic/meiotic/premeiotic/	other/	#N/A
Q8VCR7	Protein ABHD14B	Abhd14b	#N/A	meiotic/premeiotic/	spermatogonium/spermatocyte/	#N/A
Q62136	Tyrosine-protein phosphatase non-receptor type 21	Ptpn21	#N/A	postmeiotic/meiotic/premeiotic/	sertoli_cell/	#N/A
G5E8Z2	Transcription initiation factor TFIID subunit 4B	Taf4b	#N/A	premeiotic/	spermatogonium/	#N/A
Q61469	Phospholipid phosphatase 1	Plpp1	#N/A	premeiotic/	spermatogonium/elongated_spermatids/leydig_cell/	#N/A
Q8CD15	Ribosomal oxygenase 2	Riox2	#N/A	premeiotic/	spermatogonium/	#N/A
Q99NG0	Helicase ARIP4	Rad54l2	#N/A	postmeiotic/meiotic/premeiotic/	spermatogonium/spermatocyte/leydig_cell/	#N/A
P35569	Insulin receptor substrate 1	Irs1	#N/A	postmeiotic/meiotic/premeiotic/	sertoli_cell/	#N/A
P70281	Synaptonemal complex protein 3	Sycp3	#N/A	meiotic/	spermatocyte/	#N/A
D3Z3K2	E3 ubiquitin-protein ligase CCNB1IP1	Ccnb1ip1	#N/A	meiotic/	spermatocyte/	#N/A
Q9DAC5	Cor1 domain-containing protein	1700013H16Rik	#N/A	meiotic/	spermatocyte/	#N/A
Q8K2K6	Arf-GAP domain and FG repeat-containing protein 1	Agfg1	#N/A	meiotic/postmeiotic/	spermatocyte/spermatid/elongated_spermatids/	#N/A
Q641K1	Cytosolic carboxypeptidase 1	Agtpbp1	#N/A	meiotic/postmeiotic/	spermatocyte/spermatid/elongated_spermatids/	#N/A
P0CB42	Nucleic acid dioxygenase ALKBH1	Alkbh1	#N/A	meiotic/	spermatocyte/	#N/A
Q8K4E0	Alstrom syndrome protein 1 homolog	Alms1	#N/A	postmeiotic/	spermatid/	#N/A
Q5FWK3	Rho GTPase-activating protein 1	Arhgap1	#N/A	postmeiotic/meiotic/premeiotic/	other/	#N/A
Q6IFT4	Rho GTPase-activating protein 20	Arhgap20	#N/A	postmeiotic/meiotic/premeiotic/	other/	#N/A
Q99PT1	Rho GDP-dissociation inhibitor 1	Arhgdia	#N/A	postmeiotic/meiotic/premeiotic/	spermatogonium/spermatocyte/spermatid/	#N/A
Q9WV74	Ankyrin repeat and SOCS box protein 1	Asb1	#N/A	meiotic/	spermatocyte/	#N/A
Q91ZT8	Ankyrin repeat and SOCS box protein 9	Asb9	#N/A	postmeiotic/	spermatid/	#N/A
Q9Z2A5	Arginyl-tRNA--protein transferase 1	Ate1	#N/A	meiotic/	spermatocyte/	#N/A
O70126	Aurora kinase B	Aurkb	#N/A	meiotic/postmeiotic/	spermatocyte/spermatid/	#N/A
Q9D9K3	Cell death regulator Aven	Aven	#N/A	postmeiotic/	spermatid/	#N/A
Q09200	Beta-1,4 N-acetylgalactosaminyltransferase 1	B4galnt1	#N/A	meiotic/postmeiotic/	spermatocyte/spermatid/	#N/A
P15535	Beta-1,4-galactosyltransferase 1	B4galt1	#N/A	meiotic/	spermatocyte/spermatid/	#N/A
Q91YY2	Beta-1,4-galactosyltransferase 3	B4galt3	#N/A	meiotic/	spermatocyte/spermatid/	#N/A
Q8R087	Beta-1,4-galactosyltransferase 7	B4galt7	#N/A	meiotic/	spermatocyte/spermatid/	#N/A
Q9CWF6	Bardet-Biedl syndrome 2 protein homolog	Bbs2	#N/A	postmeiotic/	spermatid/	#N/A
P70345	Bcl-2-like protein 2	Bcl2l2	#N/A	postmeiotic/meiotic/premeiotic/	spermatogonium/spermatocyte/spermatid/leydig_cell/sertoli_cell/	#N/A
O88700	Bloom syndrome protein homolog	Blm	#N/A	meiotic/	spermatocyte/	#N/A
Q921C3	Bromodomain and WD repeat-containing protein 1	Brwd1	#N/A	postmeiotic/	spermatid/	#N/A
Q3ULA2	F-box/WD repeat-containing protein 1A	Btrc	#N/A	meiotic/	spermatocyte/	#N/A
Q8R149	BUD13 homolog	Bud13	#N/A	postmeiotic/	spermatid/	#N/A
P00920	Carbonic anhydrase 2	Ca2	#N/A	postmeiotic/meiotic/premeiotic/	elongated_spermatids/	#N/A
Q8R5M8	Cell adhesion molecule 1	Cadm1	#N/A	postmeiotic/meiotic/premeiotic/	spermatogonium/spermatocyte/spermatid/sertoli_cell/	#N/A
P08414	Calcium/calmodulin-dependent protein kinase type IV	Camk4	#N/A	postmeiotic/	spermatid/	#N/A
P22682	E3 ubiquitin-protein ligase CBL	Cbl	#N/A	meiotic/	spermatocyte/	#N/A
Q61456	Cyclin-A1	Ccna1	#N/A	postmeiotic/meiotic/premeiotic/	spermatogonium/spermatocyte/elongated_spermatids/	#N/A
Q9Z0H0	Cell division cycle 7-related protein kinase	Cdc7	#N/A	meiotic/	spermatocyte/	#N/A
P24788	Cyclin-dependent kinase 11B	Cdk11b	#N/A	meiotic/postmeiotic/	spermatocyte/spermatid/	#N/A

Q9WTK2	Chromodomain Y-like protein	Cdyl	#N/A	postmeiotic/	spermatid/	#N/A
P28659	CUGBP Elav-like family member 1	Celf1	#N/A	postmeiotic/	spermatid/	#N/A
Q8BT07	Centrosomal protein of 55 kDa	Cep55	#N/A	postmeiotic/meiotic/premeiotic/	spermatogonium/spermatocyte/spermatid/elongated_spermatids	#N/A
Q9Z0F4	Calcium and integrin-binding protein 1	Cib1	#N/A	postmeiotic/	spermatid/elongated_spermatids/sertoli_cell/	#N/A
P49025	Citron Rho-interacting kinase	Cit	#N/A	premeiotic/	spermatogonium/	#N/A
Q9R0A1	Chloride channel protein 2	Clcn2	#N/A	meiotic/	spermatocyte/	#N/A
Q60771	Claudin-11	Cldn11	#N/A	postmeiotic/meiotic/premeiotic/	sertoli_cell/	#N/A
Q922J3	CAP-Gly domain-containing linker protein 1	Clip1	#N/A	postmeiotic/	spermatid/elongated_spermatids/	#N/A
Q60809	CCR4-NOT transcription complex subunit 7	Cnot7	#N/A	postmeiotic/meiotic/premeiotic/	spermatid/sertoli_cell/leydig_cell/	#N/A
Q812E0	Cytoplasmic polyadenylation element-binding protein 2	Cpeb2	#N/A	postmeiotic/	spermatid/	#N/A
Q6NVF9	Cleavage and polyadenylation specificity factor subunit 6	Cpsf6	#N/A	postmeiotic/meiotic/premeiotic/	other/	#N/A
Q9DAN8	Cystatin-12	Cst12	#N/A	postmeiotic/meiotic/premeiotic/	elongated_spermatids/	#N/A
P97792	Coxsackievirus and adenovirus receptor homolog	Cxadr	#N/A	postmeiotic/meiotic/premeiotic/	spermatogonium/spermatocyte/spermatid/sertoli_cell/	#N/A
Q80TQ2	Ubiquitin carboxyl-terminal hydrolase CYLD	Cyld	#N/A	postmeiotic/	spermatid/	#N/A
Q811W2	Cytochrome P450 26B1	Cyp26b1	#N/A	premeiotic/	spermatogonium/	#N/A
P16381	Putative ATP-dependent RNA helicase Pl10	D1Pas1	#N/A	meiotic/postmeiotic/	spermatocyte/spermatid/	#N/A
Q9JII5	DAZ-associated protein 1	Dazap1	#N/A	postmeiotic/meiotic/premeiotic/	spermatogonium/spermatocyte/spermatid/	#N/A
Q64368	Deleted in azoospermia-like	Dazl	#N/A	postmeiotic/meiotic/premeiotic/	spermatogonium/spermatocyte/spermatid/	#N/A
Q9QY15	ATP-dependent RNA helicase DDX25	Ddx25	#N/A	postmeiotic/meiotic/premeiotic/	spermatid/leydig_cell/other/	#N/A
Q62095	ATP-dependent RNA helicase DDX3Y	Ddx3y	#N/A	meiotic/	spermatocyte/	#N/A
P54823	Probable ATP-dependent RNA helicase DDX6	Ddx6	#N/A	postmeiotic/meiotic/premeiotic/	spermatogonium/spermatocyte/spermatid/elongated_spermatids	#N/A
O09005	Sphingolipid delta(4)-desaturase DES1	Degs1	#N/A	meiotic/	spermatocyte/	#N/A
Q61488	Desert hedgehog protein	Dhh	#N/A	postmeiotic/meiotic/premeiotic/	sertoli_cell/	#N/A
Q9QZL9	Dickkopf-like protein 1	Dkkl1	#N/A	postmeiotic/	spermatocyte/spermatid/elongated_spermatids/	#N/A
Q61880	Meiotic recombination protein DMC1/LIM15 homolog	Dmc1	#N/A	meiotic/	spermatocyte/	#N/A
Q9QZ59	Doublesex- and mab-3-related transcription factor 1	Dmrt1	#N/A	meiotic/premeiotic/	spermatogonium/sertoli_cell/other/	#N/A
Q61214	Dual specificity tyrosine-phosphorylation-regulated kinase 1A	Dyrk1a	#N/A	premeiotic/	spermatogonium/sertoli_cell/	#N/A
Q91ZD6	ELL-associated factor 2	Eaf2	#N/A	postmeiotic/meiotic/premeiotic/	other/	#N/A
Q9WVK4	EH domain-containing protein 1	Ehd1	#N/A	postmeiotic/	spermatid/	#N/A
Q9JLJ4	Elongation of very long chain fatty acids protein 2	Elovl2	#N/A	meiotic/postmeiotic/	spermatocyte/spermatid/	#N/A
Q9WUZ9	Ectonucleoside triphosphate diphosphohydrolase 5	Entpd5	#N/A	postmeiotic/	spermatid/elongated_spermatids/	#N/A
Q9QZD4	DNA repair endonuclease XPF	Ercc4	#N/A	meiotic/	spermatocyte/spermatid/	#N/A
P19785	Estrogen receptor	Esr1	#N/A	postmeiotic/meiotic/premeiotic/	sertoli_cell/	#N/A
O08580	Steroid hormone receptor ERR1	Esrra	#N/A	postmeiotic/meiotic/premeiotic/	sertoli_cell/	#N/A
Q61545	RNA-binding protein EWS	Ewsr1	#N/A	meiotic/	spermatocyte/	#N/A
Q9JL70	Fanconi anemia group A protein homolog	Fanca	#N/A	meiotic/premeiotic/	spermatogonium/spermatocyte/	#N/A
Q9JHE4	Galactosylceramide sulfotransferase	Gal3st1	#N/A	meiotic/	spermatocyte/	#N/A
Q61592	Growth arrest-specific protein 6	Gas6	#N/A	postmeiotic/meiotic/premeiotic/	spermatogonium/leydig_cell/sertoli_cell/	#N/A
Q920G9	Germ cell-less protein-like 1	Gmcl1	#N/A	postmeiotic/	spermatocyte/spermatid/elongated_spermatids/	#N/A
P98192	Dihydroxyacetone phosphate acyltransferase	Gnpat	#N/A	meiotic/	spermatocyte/spermatid/	#N/A
P55937	Golgin subfamily A member 3	Golga3	#N/A	meiotic/postmeiotic/	spermatocyte/spermatid/	#N/A
Q8BH60	Golgi-associated PDZ and coiled-coil motif-containing protein	Gopc	#N/A	postmeiotic/	spermatid/	#N/A
Q61586	Glycerol-3-phosphate acyltransferase 1, mitochondrial	Gpam	#N/A	meiotic/postmeiotic/	spermatocyte/spermatid/	#N/A
Q8K2C8	Glycerol-3-phosphate acyltransferase 4	Gpat4	#N/A	meiotic/premeiotic/	spermatogonium/spermatocyte/	#N/A
Q9DAN6	Gametocyte-specific factor 1	Gtsf1	#N/A	postmeiotic/meiotic/premeiotic/	spermatocyte/other/	#N/A
P43275	Histone H1.1	H1-1	#N/A	postmeiotic/meiotic/premeiotic/	spermatogonium/spermatocyte/spermatid/	#N/A
P27661	Histone H2AX	H2ax	#N/A	meiotic/	spermatocyte/	#N/A
P70696	Histone H2B type 1-A	H2bc1	#N/A	postmeiotic/	spermatid/	#N/A
P84244	Histone H3.3	H3-3b	#N/A	meiotic/postmeiotic/	spermatocyte/spermatid/	#N/A
Q2VPR5	PWWP domain-containing protein	Hdgfl1	#N/A	meiotic/	spermatocyte/spermatid/	#N/A
Q60848	Lymphocyte-specific helicase	Hells	#N/A	meiotic/premeiotic/	spermatogonium/spermatocyte/	#N/A
Q8VD75	Huntingtin-interacting protein 1	Hip1	#N/A	postmeiotic/	spermatid/	#N/A
P17095	High mobility group protein HMG-I/HMG-Y	Hmga1	#N/A	meiotic/	spermatogonium/spermatocyte/spermatid/sertoli_cell/	#N/A
P30681	High mobility group protein B2	Hmgb2	#N/A	postmeiotic/	spermatocyte/spermatid/	#N/A
Q8BIL5	Protein Hook homolog 1	Hook1	#N/A	postmeiotic/	spermatid/elongated_spermatids/	#N/A
Q9D5T7	HORMA domain-containing protein 1	Hormad1	#N/A	meiotic/	spermatocyte/	#N/A
P51660	Peroxisomal multifunctional enzyme type 2	Hsd17b4	#N/A	postmeiotic/premeiotic/	sertoli_cell/	#N/A
Q8BKE9	Intraflagellar transport protein 74 homolog	Ift74	#N/A	premeiotic/	spermatogonium/	#N/A
Q9ES52	Phosphatidylinositol 3,4,5-trisphosphate 5-phosphatase 1	Inpp5d	#N/A	postmeiotic/	spermatocyte/spermatid/	#N/A
Q6PD10	Inositol hexakisphosphate kinase 1	Ip6k1	#N/A	postmeiotic/	spermatid/elongated_spermatids/	#N/A
Q9D8B7	Junctional adhesion molecule C	Jam3	#N/A	postmeiotic/	spermatid/	#N/A
Q6PCM1	Lysine-specific demethylase 3A	Kdm3a	#N/A	postmeiotic/	spermatid/	#N/A
Q60749	KH domain-containing, RNA-binding, signal transduction-associated protein 1	Khdrbs1	#N/A	meiotic/	spermatocyte/spermatid/	#N/A
Q8K339	DNA/RNA-binding protein KIN17	Kin	#N/A	postmeiotic/meiotic/premeiotic/	spermatogonium/spermatocyte/spermatid/sertoli_cell/	#N/A
O35344	Importin subunit alpha-4	Kpna3	#N/A	meiotic/	spermatocyte/	#N/A
O35343	Importin subunit alpha-3	Kpna4	#N/A	meiotic/premeiotic/	spermatogonium/spermatocyte/	#N/A
O35345	Importin subunit alpha-7	Kpna6	#N/A	postmeiotic/	spermatid/	#N/A
Q3U9G9	Delta(14)-sterol reductase LBR	Lbr	#N/A	postmeiotic/	spermatid/	#N/A
O54785	LIM domain kinase 2	Limk2	#N/A	meiotic/	spermatocyte/	#N/A
P21619	Lamin-B2	Lmnb2	#N/A	postmeiotic/	spermatid/	#N/A
Q3TYD6	Serine/threonine-protein kinase LMTK2	Lmtk2	#N/A	postmeiotic/	spermatid/	#N/A
O88978	Protein tilB homolog	Lrrc6	#N/A	meiotic/	spermatocyte/	#N/A
Q9ESN9	C-Jun-amino-terminal kinase-interacting protein 3	Mapk8ip3	#N/A	meiotic/premeiotic/	spermatogonium/spermatocyte/	#N/A
Q61845	Meiosis-expressed gene 1 protein	Meig1	#N/A	meiotic/postmeiotic/	spermatocyte/spermatid/	#N/A
Q60805	Tyrosine-protein kinase Mer	Mertk	#N/A	postmeiotic/meiotic/premeiotic/	sertoli_cell/leydig_cell/	#N/A
Q9QXP6	E3 ubiquitin-protein ligase makorin-1	Mkrn1	#N/A	meiotic/postmeiotic/	spermatocyte/spermatid/	#N/A

Q9JK91	DNA mismatch repair protein Mlh1	Mlh1	#N/A	meiotic/	spermatocyte/	#N/A
P33434	72 kDa type IV collagenase	Mmp2	#N/A	postmeiotic/meiotic/premeiotic/	spermatogonium/spermatocyte/spermatid/	#N/A
Q9QUM7	MutS protein homolog 5	Msh5	#N/A	meiotic/	spermatocyte/	#N/A
Q8K4B0	Metastasis-associated protein MTA1	Mta1	#N/A	postmeiotic/meiotic/premeiotic/	spermatogonium/spermatocyte/spermatid/elongated_spermatids	#N/A
Q8BJS8	Mdm2-binding protein	Mtbp	#N/A	meiotic/postmeiotic/	spermatocyte/spermatid/	#N/A
Q9Z2D1	Myotubularin-related protein 2	Mtmr2	#N/A	meiotic/	spermatocyte/sertoli_cell/	#N/A
P03966	N-myc proto-oncogene protein	Mycn	#N/A	premeiotic/	spermatogonium/	#N/A
Q3UX61	N-alpha-acetyltransferase 11	Naa11	#N/A	postmeiotic/	spermatid/	#N/A
Q9ERR1	Nuclear distribution protein nudE-like 1	Ndel1	#N/A	postmeiotic/	spermatid/	#N/A
Q9JKL4	NADH dehydrogenase [ubiquinone] 1 alpha subcomplex assembly factor 3	Ndufaf3	#N/A	meiotic/	spermatocyte/spermatid/	#N/A
Q3TKR3	NACHT, LRR and PYD domains-containing protein 4C	Nlrp4c	#N/A	premeiotic/	spermatogonium/	#N/A
Q61066	Nuclear receptor subfamily 0 group B member 1	Nr0b1	#N/A	meiotic/	leydig_cell/sertoli_cell/	#N/A
P49117	Nuclear receptor subfamily 2 group C member 2	Nr2c2	#N/A	meiotic/	spermatocyte/	#N/A
P33242	Steroidogenic factor 1	Nr5a1	#N/A	premeiotic/	leydig_cell/	#N/A
Q8BHG1	Nardilysin	Nrdc	#N/A	postmeiotic/	spermatid/	#N/A
Q8BHW2	Protein OSCP1	Oscp1	#N/A	meiotic/postmeiotic/	spermatocyte/spermatid/	#N/A
Q9DAK2	Parkin coregulated gene protein homolog	Pacrg	#N/A	postmeiotic/	spermatid/	#N/A
Q61206	Platelet-activating factor acetylhydrolase IB subunit beta	Pafah1b2	#N/A	meiotic/postmeiotic/	spermatocyte/spermatid/	#N/A
Q9D6V8	Polyadenylate-binding protein-interacting protein 2	Paip2	#N/A	postmeiotic/	spermatid/	#N/A
O88554	Poly [ADP-ribose] polymerase 2	Parp2	#N/A	meiotic/postmeiotic/	spermatocyte/spermatid/	#N/A
Q64338	Calcium/calmodulin-dependent 3',5'-cyclic nucleotide phosphodiesterase 1C	Pde1c	#N/A	meiotic/postmeiotic/	spermatocyte/spermatid/	#N/A
O88907	E3 SUMO-protein ligase PIAS1	Pias1	#N/A	postmeiotic/meiotic/premeiotic/	spermatocyte/spermatid/leydig_cell/sertoli_cell/	#N/A
Q8C5D8	E3 SUMO-protein ligase PIAS2	Pias2	#N/A	postmeiotic/meiotic/premeiotic/	other/	#N/A
Q9JM05	E3 SUMO-protein ligase PIAS4	Pias4	#N/A	meiotic/	spermatocyte/	#N/A
Q62083	PRKCA-binding protein	Pick1	#N/A	postmeiotic/	spermatid/	#N/A
Q8BTI9	Phosphatidylinositol 4,5-bisphosphate 3-kinase catalytic subunit beta isoform	Pik3cb	#N/A	premeiotic/	spermatogonium/	#N/A
P26450	Phosphatidylinositol 3-kinase regulatory subunit alpha	Pik3r1	#N/A	postmeiotic/	spermatid/	#N/A
O08908	Phosphatidylinositol 3-kinase regulatory subunit beta	Pik3r2	#N/A	postmeiotic/	spermatid/	#N/A
Q9QUR7	Peptidyl-prolyl cis-trans isomerase NIMA-interacting 1	Pin1	#N/A	Premeiotic/	spermatogonium/sertoli_cell/	#N/A
O35405	5'-3' exonuclease PLD3	Pld3	#N/A	postmeiotic/meiotic/premeiotic/	spermatocyte/spermatid/leydig_cell/	#N/A
Q5SWZ9	Mitochondrial cardiolipin hydrolase	Pld6	#N/A	postmeiotic/meiotic/premeiotic/	spermatocyte/spermatid/leydig_cell/	#N/A
A5H0M4	ANK_REP_REGION domain-containing protein	Poteg	#N/A	meiotic/postmeiotic/	spermatocyte/spermatid/	#N/A
P37238	Peroxisome proliferator-activated receptor gamma	Pparg	#N/A	postmeiotic/meiotic/premeiotic/	sertoli_cell/other/	#N/A
Q8R1Z4	Protein phosphatase 1 regulatory subunit 42	Ppp1r42	#N/A	postmeiotic/	spermatid/	#N/A
Q9DBC7	cAMP-dependent protein kinase type I-alpha regulatory subunit	Prkar1a	#N/A	meiotic/postmeiotic/	spermatocyte/spermatid/	#N/A
Q9QUG3	Prion-like protein doppel	Prnd	#N/A	postmeiotic/	spermatid/	#N/A
O35047	Homologous-pairing protein 2 homolog	Psmc3ip	#N/A	meiotic/	spermatocyte/	#N/A
Q8R326	Paraspeckle component 1	Pspc1	#N/A	postmeiotic/	spermatid/	#N/A
Q8K094	Poliovirus receptor	Pvr	#N/A	postmeiotic/meiotic/premeiotic/	sertoli_cell/	#N/A
Q9QXK2	E3 ubiquitin-protein ligase RAD18	Rad18	#N/A	meiotic/premeiotic/	spermatogonium/spermatocyte/	#N/A
Q99NF8	Ran-binding protein 17	Ranbp17	#N/A	meiotic/postmeiotic/	spermatocyte/spermatid/	#N/A
P69566	Ran-binding protein 9	Ranbp9	#N/A	meiotic/premeiotic/	spermatogonium/spermatocyte/	#N/A
P62835	Ras-related protein Rap-1A	Rap1a	#N/A	postmeiotic/	spermatid/	#N/A
Q9DAE2	RRM domain-containing protein	Rbmxl2	#N/A	postmeiotic/meiotic/premeiotic/	elongated_spermatids/	#N/A
Q8C5S7	Meiotic recombination protein REC8 homolog	Rec8	#N/A	meiotic/	spermatocyte/	#N/A
Q64374	Regucalcin	Rgn	#N/A	postmeiotic/meiotic/premeiotic/	spermatogonium/spermatocyte/spermatid/elongated_spermatids	#N/A
Q9DC04	Regulator of G-protein signaling 3	Rgs3	#N/A	meiotic/	spermatocyte/	#N/A
Q99MB7	RING finger protein 141	Rnf141	#N/A	postmeiotic/premeiotic/	spermatogonium/spermatid/elongated_spermatids/	#N/A
Q8BH75	E3 ubiquitin-protein ligase NRDP1	Rnf41	#N/A	meiotic/	spermatocyte/	#N/A
Q80T69	Lysine-specific demethylase 9	Rsbn1	#N/A	postmeiotic/	spermatid/	#N/A
Q8VIG3	Radial spoke head 1 homolog	Rsph1	#N/A	postmeiotic/	spermatid/	#N/A
P50543	Protein S100-A11	S100a11	#N/A	postmeiotic/meiotic/premeiotic/	sertoli_cell/	#N/A
Q6ZPE2	Myotubularin-related protein 5	Sbf1	#N/A	meiotic/	spermatogonium/spermatocyte/sertoli_cell/	#N/A
Q62421	Endophilin-A3	Sh3gl3	#N/A	postmeiotic/	spermatid/	#N/A
P55012	Solute carrier family 12 member 2	Slc12a2	#N/A	meiotic/	spermatogonium/spermatocyte/spermatid/elongated_spermatids	#N/A
Q60738	Zinc transporter 1	Slc30a1	#N/A	postmeiotic/meiotic/premeiotic/	elongated_spermatids/sertoli_cell/	#N/A
Q6P5F6	Zinc transporter ZIP10	Slc39a10	#N/A	meiotic/	spermatocyte/	#N/A
Q8C145	Zinc transporter ZIP6	Slc39a6	#N/A	postmeiotic/	spermatid/	#N/A
P13808	Anion exchange protein 2	Slc4a2	#N/A	postmeiotic/	spermatid/	#N/A
Q920F6	Structural maintenance of chromosomes protein 1B	Smc1b	#N/A	meiotic/premeiotic/	spermatocyte/spermatogonium/	#N/A
Q9D489	Spermatogenesis- and oogenesis-specific basic helix-loop-helix-containing protein 2	Sohlh2	#N/A	premeiotic/	spermatogonium/	#N/A
Q04886	Transcription factor SOX-8	Sox8	#N/A	postmeiotic/meiotic/premeiotic/	sertoli_cell/	#N/A
Q8K450	Sperm-associated antigen 16 protein	Spag16	#N/A	postmeiotic/meiotic/premeiotic/	spermatogonium/spermatocyte/spermatid/elongated_spermatids	#N/A
Q6P926	Spermatogenesis-associated protein 24	Spata24	#N/A	postmeiotic/	spermatid/	#N/A
Q91WM1	Spermatid perinuclear RNA-binding protein	Strbp	#N/A	postmeiotic/	spermatid/	#N/A
P11031	Activated RNA polymerase II transcriptional coactivator p15	Sub1	#N/A	meiotic/postmeiotic/	spermatocyte/spermatid/	#N/A
Q9D666	SUN domain-containing protein 1	Sun1	#N/A	meiotic/postmeiotic/	spermatocyte/spermatid/	#N/A
O54864	Histone-lysine N-methyltransferase SUV39H1	Suv39h1	#N/A	meiotic/	spermatocyte/	#N/A
Q9D495	Synaptonemal complex central element protein 1	Syce1	#N/A	meiotic/	spermatocyte/	#N/A
Q505B8	Synaptonemal complex central element protein 2	Syce2	#N/A	meiotic/	spermatocyte/	#N/A
Q62209	Synaptonemal complex protein 1	Sycp1	#N/A	meiotic/	spermatocyte/	#N/A
Q9D3R9	Transcription initiation factor TFIID subunit 7-like	Taf7l	#N/A	postmeiotic/meiotic/premeiotic/	spermatogonium/spermatocyte/spermatid/	#N/A
P97473	RISC-loading complex subunit TARBP2	Tarbp2	#N/A	postmeiotic/	spermatid/	#N/A
P29037	TATA-box-binding protein	Tbp	#N/A	meiotic/postmeiotic/	spermatocyte/spermatid/	#N/A
P62340	TATA box-binding protein-like protein 1	Tbpl1	#N/A	postmeiotic/	spermatid/	#N/A
Q9DAJ2	Tektin-1	Tekt1	#N/A	postmeiotic/	spermatid/elongated_spermatids/	#N/A

O35144	Telomeric repeat-binding factor 2	Terf2	#N/A	meiotic/postmeiotic/	spermatocyte/spermatid/	#N/A
Q9WTJ6	Tesmin	Tesmin	#N/A	meiotic/postmeiotic/	spermatocyte/spermatid/	#N/A
Q9JMI7	Testis-expressed protein 101	Tex101	#N/A	postmeiotic/meiotic/premeiotic/	spermatogonium/spermatocyte/spermatid/other/	#N/A
Q14AT2	Testis-expressed protein 11	Tex11	#N/A	meiotic/	spermatocyte/	#N/A
Q9CR81	Testis-expressed protein 12	Tex12	#N/A	meiotic/	spermatocyte/	#N/A
Q7M6U3	Inactive serine/threonine-protein kinase TEX14	Tex14	#N/A	postmeiotic/meiotic/premeiotic/	spermatocyte/other/	#N/A
Q99MV2	Testis-expressed protein 19.1	Tex19.1	#N/A	meiotic/	spermatocyte/	#N/A
Q921I1	Serotransferrin	Tf	#N/A	postmeiotic/	spermatid/	#N/A
Q6ZQF0	DNA topoisomerase 2-binding protein 1	Topbp1	#N/A	meiotic/	spermatocyte/	#N/A
Q99MS8	Tubulin polyglutamylase complex subunit 1	Tpgs1	#N/A	postmeiotic/	spermatid/	#N/A
Q3UA06	Pachytene checkpoint protein 2 homolog	Trip13	#N/A	meiotic/	spermatocyte/	#N/A
Q62348	Translin	Tsn	#N/A	meiotic/postmeiotic/	spermatocyte/spermatid/	#N/A
P63147	Ubiquitin-conjugating enzyme E2 B	Ube2b	#N/A	meiotic/postmeiotic/	spermatocyte/spermatid/	#N/A
Q64676	2-hydroxyacylsphingosine 1-beta-galactosyltransferase	Ugt8	#N/A	meiotic/	spermatocyte/	#N/A
Q65Z40	Wings apart-like protein homolog	Wapl	#N/A	meiotic/	spermatocyte/	#N/A
Q8K4P0	pre-mRNA 3' end processing protein WDR33	Wdr33	#N/A	meiotic/	spermatocyte/	#N/A
O54929	WD repeat and SOCS box-containing protein 2	Wsb2	#N/A	postmeiotic/meiotic/premeiotic/	spermatogonium/spermatocyte/sertoli_cell/	#N/A
Q64267	DNA repair protein complementing XP-A cells homolog	Xpa	#N/A	postmeiotic/meiotic/premeiotic/	elongated_spermatids/	#N/A
P0C7L0	WAS/WASL-interacting protein family member 3	Wipf3	#N/A	postmeiotic/	sertoli_cell/	#N/A
Q9D2H2	Adenylate kinase 7	Ak7	#N/A	postmeiotic/	spermatid/	#N/A
P32507	Nectin-2	Nectin2	#N/A	postmeiotic/	elongated_spermatids/sertoli_cell/	#N/A
Q9ESC8	AF4/FMR2 family member 4	Aff4	#N/A	postmeiotic/	sertoli_cell/	#N/A
P25916	Polycomb complex protein BMI-1	Bmi1	#N/A	premeiotic/	spermatogonium/	#N/A
P41241	Tyrosine-protein kinase CSK	Csk	#N/A	postmeiotic/	leydig_cell/sertoli_cell/	#N/A
F8VQB6	Unconventional myosin-X	Myo10	#N/A	postmeiotic/	sertoli_cell/	#N/A
P70365	Nuclear receptor coactivator 1	Ncoa1	#N/A	postmeiotic/	sertoli_cell/	#N/A
Q61026	Nuclear receptor coactivator 2	Ncoa2	#N/A	postmeiotic/	sertoli_cell/	#N/A
Q11011	Puromycin-sensitive aminopeptidase	Npepps	#N/A	postmeiotic/	sertoli_cell/	#N/A
