# Supplementary material for: Polyubiquitin gene Ubb is required for upregulation of Piwi protein level during mouse testis development
Source: Cell Death Discov. 2021 Jul 26;7:194. doi: 10.1038/s41420-021-00581-2 (PMC8313548; doi:10.1038/s41420-021-00581-2)
Supplement: Supplementary file 4 — Supplementary table 4 [file 41420_2021_581_MOESM4_ESM.pdf]

Table of Contents	
term_ID	Unique identifier in Gene ontology
p-value	The p-value obtained by computing multiple testing correction such as GO and pathway enrichment analysis through the g;SCS algorithm.
Gene list	Gene list corresponding to each term among DEPs

Supplemental Table S3a. Molecular function of up-regulated expressed proteins

term_ID	Molecular Function	p-value	Gene list
GO:0005201	extracellular matrix structural constituent	1.08.E-14	Col6a3 Col6a3 Fbn1 Col6a4 Col6a1 Tgfbi Col6a2 Dcn Fbn2 Nid1 Lum Prelp Ogn Lama5 Mfap4 Aspn Dpt
GO:0005198	structural molecule activity	2.23.E-13	Ahnak Ahnak Col6a3 Sptbn1 Vim Fbn1 Col6a4 Epb41l2 Col6a1 Tgfbi Dsp Col6a2 Krt14 Dcn Fbn2 Nid1 Lum Jup Prelp Ogn Lama5 Synm Mfap4 Ctbp2 Aspn Dpt Cldn1
GO:0005488	binding	2.82.E-09	Ahnak Ahnak Sptan1 Col6a3 Sptbn1 Vim Fbn1 Lmnb1 Tkt Col6a4 Epb41l2 Pygb Anxa6 Lmna Col6a1 Selenbp1 Anxa2 Dpysl2 Anxa5 Gstm1 Alad Cald1 Fermt2 Tgfbi Dsp Col6a2 Ehd2 Krt14 Dcn Fbn2 H1-3 Nid1 Ctsd Npr1 Krt5 Cavin1 Lum Serpinb1a Anxa3 Jup H1-5 Krt8 Hmgb3 Anxa1 Cryl1 Kank2 Rbp1 Anpep Oga Ptk7 Tagln2 Pcyt1a Prelp Glo1 Hpcal1 Fhl1 Ogn Lama5 Atp1b1 Cnn1 Cav1 Flot2 Synm Gatad2b Chdh Hdgfl3<br>Toe1 Palm Znf512 Emilin3 Gpx3 F11r Mthfr Scaf11 Grb2 Susd2 Stag2 Pdlim7 Rbpms Ctbp2 Glipr2 Gsdmdc1 Cd47 Nucks1 Cdkn1b S100a10 Nfic Tns2 Smad3 Prim2 Ptms Bdh2 Jakmip1 Sp3 Pnpla2 Nfib Itga9 Txnip Thbs3 Cnrip1 Deptor Znf148 Tbc1d2b Enpp3 Znf22 Fcgbp Gulp1 Tspan4 Pde2a Cd59a Cldn1 Dnhd1 P2rx7
GO:0005518	collagen binding	3.01.E-09	Col6a4 Col6a4 Col6a1 Tgfbi Col6a2 Dcn Nid1 Lum Smad3 Aspn Itga9
GO:0044877	protein-containing complex binding	4.25.E-09	Sptan1 Sptan1 Sptbn1 Vim Fbn1 Col6a4 Epb41l2 Anxa6 Col6a1 Alad Fermt2 Tgfbi Col6a2 Krt14 Dcn H1-3 Nid1 Lum Jup H1-5 Krt8 Lama5 Cav1 Synm F11r Mthfr Grb2 Ctbp2 Cdkn1b Smad3 Aspn Itga9 Tspan4
GO:0030021	extracellular matrix structural constituent conferring compression resistance	2.12.E-06	Dcn Dcn Lum Prelp Ogn Aspn
GO:0005515	protein binding	4.96.E-06	Ahnak Ahnak Sptan1 Col6a3 Sptbn1 Vim Fbn1 Lmnb1 Tkt Epb41l2 Pygb Anxa6 Lmna Col6a1 Anxa2 Dpysl2 Anxa5 Gstm1 Alad Cald1 Fermt2 Tgfbi Dsp Ehd2 Krt14 Dcn Fbn2 H1-3 Nid1 Ctsd Npr1 Krt5 Cavin1 Lum Serpinb1a Anxa3 Jup H1-5 Krt8 Hmgb3 Anxa1 Cryl1 Kank2 Oga Tagln2 Pcyt1a Prelp Hpcal1 Fhl1 Ogn Lama5 Atp1b1 Cnn1 Cav1 Flot2 Synm Gatad2b Hdgfl3 Palm Emilin3 F11r Grb2 Pdlim7 Rbpms Ctbp2 Glipr2 Cd47<br>Nucks1 Cdkn1b S100a10 Tns2 Smad3 Aspn Jakmip1 Sp3 Pnpla2 Itga9 Txnip Cnrip1 Deptor Tbc1d2b Fcgbp Gulp1 Tspan4 Pde2a Cd59a Cldn1 Dnhd1 P2rx7
GO:0019834	phospholipase A2 inhibitor activity	9.14.E-04	Anxa2 Anxa2 Anxa3 Anxa1
GO:0005544	calcium-dependent phospholipid binding	4.78.E-03	Anxa6 Anxa6 Anxa2 Anxa5 Anxa3 Anxa1
GO:0004859	phospholipase inhibitor activity	5.05.E-03	Anxa2 Anxa2 Anxa3 Anxa1
GO:0019900	kinase binding	6.09.E-03	Vim Vim Lmnb1 Dpysl2 Anxa5 Gstm1 Dsp Npr1 Jup Atp1b1 Cav1 Grb2 Ctbp2 Cdkn1b Tns2 Smad3 Jakmip1
GO:0005509	calcium ion binding	6.20.E-03	Sptan1 Sptan1 Fbn1 Anxa6 Anxa2 Anxa5 Ehd2 Fbn2 Nid1 Anxa3 Anxa1 Hpcal1 S100a10 Aspn Thbs3 Enpp3
GO:0019901	protein kinase binding	7.19.E-03	Vim Vim Lmnb1 Dpysl2 Anxa5 Gstm1 Dsp Npr1 Jup Atp1b1 Cav1 Grb2 Ctbp2 Cdkn1b Tns2 Smad3
GO:0050840	extracellular matrix binding	9.15.E-03	Anxa2 Anxa2 Tgfbi Dcn Nid1 Itga9
GO:0042802	identical protein binding	1.25.E-02	Vim Vim Fbn1 Tkt Pygb Anxa6 Lmna Anxa2 Dpysl2 Anxa5 Gstm1 Alad Ehd2 Cavin1 Anxa1 Cryl1 Pcyt1a Cav1 F11r Grb2 Rbpms Glipr2 S100a10 Smad3 Pde2a Cldn1 P2rx7
GO:0030020	extracellular matrix structural constituent conferring tensile strength	2.06.E-02	Col6a3 Col6a3 Col6a4 Col6a1 Col6a2
GO:0055102	lipase inhibitor activity	2.53.E-02	Anxa2 Anxa2 Anxa3 Anxa1
GO:0019215	intermediate filament binding	2.53.E-02	Vim Vim Krt14 Synm

Supplemental Table S3b. Biologcial process of up-regulated expressed proteins

term_ID	Biological process	p-value	Gene list
GO:0016043	cellular component organization	3.72.E-07	Ahnak Sptan1 Col6a3 Sptbn1 Vim Col6a4 Epb41l2 Anxa6 Lmna Col6a1 Anxa2 Dpysl2 Alad Myof Cald1 Fermt2 Tgfbi Dsp Col6a2 Ehd2 Krt14 Dcn H1-3 Nid1 Ctsd Lum Jup H1-5 Krt8 Hmgb3 Anxa1 Kank2 Oga Ptk7 Fhl1 Lama5 Cnn1 Cav1 Flot2 Synm Hdgfl3 Mfap4 Palm Gpx3 F11r Mthfr Scaf11 Grb2 Stag2 Pdlim7 Ctbp2 Gsdmdc1 Cd47 Nucks1 Cdkn1b S100a10 Smad3 Pnpla2 Nfib Deptor Znf148 Dpt Gulp1 Tspan4 Pde2a Cldn1 P2rx7
GO:0071840	cellular component organization or biogenesis	1.45.E-06	Ahnak Sptan1 Col6a3 Sptbn1 Vim Col6a4 Epb41l2 Anxa6 Lmna Col6a1 Anxa2 Dpysl2 Alad Myof Cald1 Fermt2 Tgfbi Dsp Col6a2 Ehd2 Krt14 Dcn H1-3 Nid1 Ctsd Lum Jup H1-5 Krt8 Hmgb3 Anxa1 Kank2 Oga Ptk7 Fhl1 Lama5 Cnn1 Cav1 Flot2 Synm Hdgfl3 Mfap4 Palm Gpx3 F11r Mthfr Scaf11 Grb2 Stag2 Pdlim7 Ctbp2 Gsdmdc1 Cd47 Nucks1 Cdkn1b S100a10 Smad3 Pnpla2 Nfib Deptor Znf148 Dpt Gulp1 Tspan4 Pde2a Cldn1 P2rx7
GO:0097435	supramolecular fiber organization	2.89.E-06	Sptan1 Sptbn1 Vim Anxa2 Cald1 Dsp Krt14 Lum Krt8 Kank2 Cav1 Hdgfl3 Mfap4 F11r Grb2 Cd47 Cdkn1b S100a10 Smad3 Dpt
GO:0048513	animal organ development	4.02.E-06	Col6a3 Vim Fbn1 Anxa6 Lmna Col6a1 Anxa2 Dpysl2 Tgfbi Dsp Col6a2 Dcn Fbn2 Nid1 Lum Anxa3 Jup H1-5 Krt8 Hmgb3 Anxa1 Kank2 Rbp1 Ptk7 Glo1 Fhl1 Lama5 Cav1 Krt75 Grb2 Pdlim7 Glipr2 Cdkn1b Nfic Tns2 Smad3 Aspn Jakmip1 Sp3 Nfib Txnip Thbs3 Znf22 Ildr2 Pde2a Cldn1 P2rx7
GO:0009888	tissue development	7.02.E-06	Col6a3 Vim Anxa6 Lmna Col6a1 Anxa2 Tgfbi Dsp Col6a2 Krt14 Dcn Fbn2 Lum Anxa1 Kank2 Ptk7 Tagln2 Lama5 Cav1 F11r Mthfr Grb2 Glipr2 Cdkn1b Smad3 Aspn Bdh2 Nfib Txnip Thbs3 Pde2a Cldn1 P2rx7
GO:0032502	developmental process	7.14.E-06	Col6a3 Vim Fbn1 Epb41l2 Anxa6 Lmna Col6a1 Selenbp1 Anxa2 Dpysl2 Myof Fermt2 Tgfbi Dsp Col6a2 Ehd2 Krt14 Dcn Fbn2 Nid1 Lum Anxa3 Jup H1-5 Krt8 Hmgb3 Anxa1 Kank2 Rbp1 Anpep Ptk7 Tagln2 Prelp Glo1 Fhl1 Lama5 Cav1 Krt75 Flot2 Ggt5 Hdgfl3 Palm F11r Mthfr Grb2 Stag2 Pdlim7 Ctbp2 Glipr2 Cdkn1b S100a10 Nfic Tns2 Smad3 Aspn Bdh2 Jakmip1 Sp3 Nfib Txnip Thbs3 Znf22 Ildr2 Pde2a Cd59a Cldn1 P2rx7
GO:0007010	cytoskeleton organization	1.08.E-05	Sptan1 Sptbn1 Vim Epb41l2 Lmna Dpysl2 Cald1 Dsp Ehd2 Krt14 Krt8 Anxa1 Kank2 Ptk7 Cnn1 Cav1 Synm Hdgfl3 Palm F11r Grb2 Stag2 Pdlim7 Cd47 Cdkn1b S100a10 Smad3
GO:0048856	anatomical structure development	3.48.E-05	Col6a3 Vim Fbn1 Epb41l2 Anxa6 Lmna Col6a1 Anxa2 Dpysl2 Myof Fermt2 Tgfbi Dsp Col6a2 Ehd2 Krt14 Dcn Fbn2 Nid1 Lum Anxa3 Jup H1-5 Krt8 Hmgb3 Anxa1 Kank2 Rbp1 Anpep Ptk7 Tagln2 Glo1 Fhl1 Lama5 Cav1 Krt75 Flot2 Hdgfl3 Palm F11r Mthfr Grb2 Pdlim7 Glipr2 Cdkn1b S100a10 Nfic Tns2 Smad3 Aspn Bdh2 Jakmip1 Sp3 Nfib Txnip Thbs3 Znf22 Ildr2 Pde2a Cd59a Cldn1 P2rx7
GO:0003417	growth plate cartilage development	4.81.E-05	Col6a3 Anxa6 Col6a1 Anxa2 Col6a2 Thbs3
GO:0009653	anatomical structure morphogenesis	5.85.E-05	Col6a3 Vim Fbn1 Epb41l2 Anxa6 Col6a1 Anxa2 Dpysl2 Myof Fermt2 Tgfbi Dsp Col6a2 Ehd2 Dcn Fbn2 Anxa3 Jup Krt8 Anxa1 Anpep Ptk7 Lama5 Cav1 Palm F11r Mthfr Grb2 S100a10 Nfic Smad3 Aspn Sp3 Nfib Thbs3 Znf22 Cd59a P2rx7
GO:0030029	actin filament-based process	9.78.E-05	Sptan1 Sptbn1 Epb41l2 Cald1 Dsp Ehd2 Jup Krt8 Anxa1 Kank2 Ptk7 Cnn1 Cav1 F11r Grb2 Pdlim7 Cd47 S100a10 Smad3
GO:0042060	wound healing	1.61.E-04	Anxa6 Anxa2 Anxa5 Myof Dsp Dcn Anxa1 Ptk7 Cav1 F11r Cdkn1b Smad3 Itga9 Cldn1
GO:0003416	endochondral bone growth	1.61.E-04	Col6a3 Anxa6 Col6a1 Anxa2 Col6a2 Thbs3
GO:0098868	bone growth	2.91.E-04	Col6a3 Anxa6 Col6a1 Anxa2 Col6a2 Thbs3
GO:0048869	cellular developmental process	3.52.E-04	Col6a3 Vim Fbn1 Epb41l2 Anxa6 Lmna Col6a1 Selenbp1 Anxa2 Dpysl2 Myof Fermt2 Tgfbi Dsp Col6a2 Ehd2 Krt14 Fbn2 Krt8 Hmgb3 Anxa1 Rbp1 Anpep Ptk7 Tagln2 Prelp Glo1 Fhl1 Lama5 Cav1 Krt75 Flot2 Hdgfl3 Palm F11r Grb2 Pdlim7 Ctbp2 Glipr2 Cdkn1b S100a10 Smad3 Bdh2 Sp3 Nfib Txnip Ildr2 Pde2a Cldn1 P2rx7
GO:0048731	system development	3.73.E-04	Col6a3 Vim Fbn1 Anxa6 Lmna Col6a1 Anxa2 Dpysl2 Tgfbi Dsp Col6a2 Dcn Fbn2 Nid1 Lum Anxa3 Jup H1-5 Krt8 Hmgb3 Anxa1 Kank2 Rbp1 Anpep Ptk7 Glo1 Fhl1 Lama5 Cav1 Krt75 Hdgfl3 Palm Mthfr Grb2 Glipr2 Cdkn1b Nfic Tns2 Smad3 Aspn Jakmip1 Sp3 Nfib Txnip Thbs3 Znf22 Ildr2 Pde2a Cd59a Cldn1 P2rx7
GO:0007155	cell adhesion	3.79.E-04	Fbn1 Col6a4 Col6a1 Fermt2 Tgfbi Dsp Col6a2 Nid1 Jup Anxa1 Ptk7 Lama5 Atp1b1 Cav1 Flot2 Mfap4 F11r Cd47 S100a10 Smad3 Itga9 Thbs3 Dpt Cldn1
GO:0060351	cartilage development involved in endochondral bone morphogenesis	4.37.E-04	Col6a3 Anxa6 Col6a1 Anxa2 Col6a2 Thbs3
GO:0022610	biological adhesion	4.43.E-04	Fbn1 Col6a4 Col6a1 Fermt2 Tgfbi Dsp Col6a2 Nid1 Jup Anxa1 Ptk7 Lama5 Atp1b1 Cav1 Flot2 Mfap4 F11r Cd47 S100a10 Smad3 Itga9 Thbs3 Dpt Cldn1
GO:0051216	cartilage development	5.56.E-04	Col6a3 Anxa6 Col6a1 Anxa2 Tgfbi Col6a2 Lum Smad3 Nfib Thbs3
GO:0002062	chondrocyte differentiation	6.54.E-04	Col6a3 Anxa6 Col6a1 Anxa2 Tgfbi Col6a2 Smad3 Nfib
GO:0030855	epithelial cell differentiation	7.30.E-04	Vim Anxa2 Dsp Krt14 Anxa1 Ptk7 Tagln2 Cav1 F11r Cdkn1b Smad3 Bdh2 Nfib Txnip Pde2a Cldn1
GO:0009628	response to abiotic stimulus	7.91.E-04	Lmna Alad Myof Krt14 Dcn Jup Krt8 Anxa1 Atp1b1 Cav1 Mfap4 Palm F11r Mthfr Grb2 Nucks1 Cdkn1b Smad3 Txnip Pde2a Cldn1 P2rx7
GO:0001765	membrane raft assembly	7.96.E-04	Anxa2 Cav1 Flot2 S100a10
GO:0061448	connective tissue development	1.03.E-03	Col6a3 Anxa6 Lmna Col6a1 Anxa2 Tgfbi Col6a2 Lum Smad3 Nfib Thbs3
GO:0071709	membrane assembly	1.90.E-03	Sptbn1 Anxa2 Cav1 Flot2 S100a10
GO:0001501	skeletal system development	1.93.E-03	Col6a3 Fbn1 Anxa6 Col6a1 Anxa2 Tgfbi Col6a2 Fbn2 Lum Smad3 Sp3 Nfib Thbs3 P2rx7
GO:0030036	actin cytoskeleton organization	2.11.E-03	Sptan1 Sptbn1 Epb41l2 Cald1 Ehd2 Krt8 Anxa1 Kank2 Ptk7 Cnn1 F11r Grb2 Pdlim7 Cd47 S100a10 Smad3
GO:0022607	cellular component assembly	2.26.E-03	Ahnak Sptan1 Sptbn1 Anxa6 Anxa2 Alad Cald1 Fermt2 Krt14 H1-3 Ctsd Jup H1-5 Krt8 Kank2 Lama5 Cav1 Flot2 Hdgfl3 Mfap4 Palm Gpx3 F11r Scaf11 Grb2 Stag2 Gsdmdc1 Cd47 Cdkn1b S100a10 Smad3 Znf148 Tspan4 Cldn1 P2rx7
GO:0048646	anatomical structure formation involved in morphogenesis	2.91.E-03	Col6a1 Anxa2 Myof Tgfbi Ehd2 Dcn Fbn2 Anxa3 Jup Krt8 Anxa1 Anpep Ptk7 Cav1 Mthfr Grb2 Smad3 Sp3 Nfib Thbs3 Cd59a
GO:0071284	cellular response to lead ion	3.00.E-03	Anxa5 Alad Cldn1
GO:0061041	regulation of wound healing	3.79.E-03	Anxa2 Anxa5 Anxa1 Cav1 F11r Cdkn1b Smad3 Cldn1
GO:0009611	response to wounding	4.12.E-03	Anxa6 Anxa2 Anxa5 Myof Dsp Dcn Anxa1 Ptk7 Cav1 F11r Cdkn1b Smad3 Itga9 Cldn1
GO:0030154	cell differentiation	4.45.E-03	Col6a3 Vim Fbn1 Anxa6 Lmna Col6a1 Selenbp1 Anxa2 Dpysl2 Myof Fermt2 Tgfbi Dsp Col6a2 Ehd2 Krt14 Fbn2 Krt8 Hmgb3 Anxa1 Rbp1 Anpep Ptk7 Tagln2 Glo1 Fhl1 Lama5 Cav1 Krt75 Flot2 Hdgfl3 F11r Grb2 Pdlim7 Ctbp2 Glipr2 Cdkn1b S100a10 Smad3 Bdh2 Sp3 Nfib Txnip Ildr2 Pde2a Cldn1
GO:0044091	membrane biogenesis	5.11.E-03	Sptbn1 Anxa2 Cav1 Flot2 S100a10
GO:0007275	multicellular organism development	5.37.E-03	Col6a3 Vim Fbn1 Anxa6 Lmna Col6a1 Anxa2 Dpysl2 Tgfbi Dsp Col6a2 Dcn Fbn2 Nid1 Lum Anxa3 Jup H1-5 Krt8 Hmgb3 Anxa1 Kank2 Rbp1 Anpep Ptk7 Glo1 Fhl1 Lama5 Cav1 Krt75 Hdgfl3 Palm F11r Mthfr Grb2 Pdlim7 Glipr2 Cdkn1b Nfic Tns2 Smad3 Aspn Jakmip1 Sp3 Nfib Txnip Thbs3 Znf22 Ildr2 Pde2a Cd59a Cldn1 P2rx7
GO:0060350	endochondral bone morphogenesis	6.15.E-03	Col6a3 Anxa6 Col6a1 Anxa2 Col6a2 Thbs3
GO:1990778	protein localization to cell periphery	7.89.E-03	Sptbn1 Epb41l2 Anxa2 Ehd2 Jup Lama5 Atp1b1 Flot2 Palm F11r S100a10
GO:0072659	protein localization to plasma membrane	7.98.E-03	Sptbn1 Anxa2 Ehd2 Jup Lama5 Atp1b1 Flot2 Palm F11r S100a10
GO:0060429	epithelium development	8.17.E-03	Vim Anxa2 Dsp Krt14 Anxa1 Kank2 Ptk7 Tagln2 Lama5 Cav1 F11r Mthfr Grb2 Cdkn1b Smad3 Bdh2 Nfib Txnip Pde2a Cldn1
GO:0009887	animal organ morphogenesis	8.37.E-03	Col6a3 Fbn1 Anxa6 Col6a1 Anxa2 Dsp Col6a2 Fbn2 Ptk7 Lama5 Cav1 Nfic Smad3 Aspn Sp3 Nfib Thbs3 Znf22 P2rx7
GO:0003418	growth plate cartilage chondrocyte differentiation	9.25.E-03	Col6a3 Anxa6 Col6a1 Col6a2
GO:0031579	membrane raft organization	1.13.E-02	Anxa2 Cav1 Flot2 S100a10
GO:1901700	response to oxygen-containing compound	1.15.E-02	Vim Fbn1 Col6a1 Dpysl2 Anxa5 Alad Col6a2 Dcn Jup Krt8 Anxa1 Kank2 Ptk7 Cav1 Ggt5 Palm Mthfr Grb2 Nucks1 Cdkn1b Tns2 Txnip Ildr2 Pde2a Cldn1 P2rx7
GO:0044085	cellular component biogenesis	1.50.E-02	Ahnak Sptan1 Sptbn1 Anxa6 Anxa2 Alad Cald1 Fermt2 Krt14 H1-3 Ctsd Jup H1-5 Krt8 Kank2 Lama5 Cav1 Flot2 Hdgfl3 Mfap4 Palm Gpx3 F11r Scaf11 Grb2 Stag2 Gsdmdc1 Cd47 Cdkn1b S100a10 Smad3 Znf148 Tspan4 Cldn1 P2rx7
GO:1902903	regulation of supramolecular fiber organization	1.52.E-02	Sptan1 Sptbn1 Kank2 Cav1 Hdgfl3 F11r Grb2 Cd47 Cdkn1b S100a10 Smad3
GO:0010616	negative regulation of cardiac muscle adaptation	1.66.E-02	Lmna Oga Smad3
GO:0007167	enzyme linked receptor protein signaling pathway	1.67.E-02	Sptbn1 Vim Fbn1 Myof Fermt2 Dcn Fbn2 Npr1 Cav1 Grb2 Rbpms Nucks1 Tns2 Smad3 Aspn Txnip Cd59a
GO:0010033	response to organic substance	1.79.E-02	Vim Fbn1 Col6a1 Anxa2 Dpysl2 Anxa5 Alad Myof Fermt2 Col6a2 Dcn Fbn2 Lum Anxa3 Jup Krt8 Anxa1 Kank2 Rbp1 Ptk7 Cav1 Ggt5 Palm Mthfr Grb2 Ctbp2 Cd47 Nucks1 Cdkn1b Tns2 Smad3 Aspn Txnip Ildr2 Pde2a Cd59a Cldn1 P2rx7
GO:1903034	regulation of response to wounding	2.01.E-02	Anxa2 Anxa5 Anxa1 Cav1 F11r Cdkn1b Smad3 Cldn1
GO:0048705	skeletal system morphogenesis	2.03.E-02	Col6a3 Anxa6 Col6a1 Anxa2 Col6a2 Fbn2 Smad3 Thbs3 P2rx7
GO:0065008	regulation of biological quality	2.24.E-02	Sptan1 Sptbn1 Vim Fbn1 Epb41l2 Anxa6 Lmna Anxa2 Dpysl2 Anxa5 Fermt2 Dsp Dcn Fbn2 Serpinb1a Jup H1-5 Anxa1 Rbp1 Anpep Oga Fhl1 Atp1b1 Cav1 Flot2 Chdh Palm F11r Grb2 Ctbp2 Cd47 Nucks1 Tns2 Smad3 Bdh2 Sp3 Pnpla2 Deptor Ildr2 Pde2a Cldn1 P2rx7
GO:0006996	organelle organization	2.37.E-02	Sptan1 Sptbn1 Vim Epb41l2 Lmna Anxa2 Dpysl2 Cald1 Dsp Ehd2 Krt14 Dcn H1-3 Ctsd H1-5 Krt8 Hmgb3 Anxa1 Kank2 Oga Ptk7 Lama5 Cnn1 Cav1 Synm Hdgfl3 Palm F11r Mthfr Grb2 Stag2 Pdlim7 Cd47 Nucks1 Cdkn1b S100a10 Smad3 Pnpla2 Pde2a P2rx7
GO:0030198	extracellular matrix organization	2.78.E-02	Col6a4 Anxa2 Tgfbi Nid1 Lum Cav1 Mfap4 Smad3 Dpt
GO:0043062	extracellular structure organization	2.95.E-02	Col6a4 Anxa2 Tgfbi Nid1 Lum Cav1 Mfap4 Smad3 Dpt
GO:0003413	chondrocyte differentiation involved in endochondral bone morphogenesis	3.07.E-02	Col6a3 Anxa6 Col6a1 Col6a2
GO:0031032	actomyosin structure organization	3.26.E-02	Epb41l2 Krt8 Kank2 Cnn1 F11r Cd47 S100a10 Smad3
GO:0090257	regulation of muscle system process	3.43.E-02	Anxa6 Lmna Cald1 Dsp Jup Oga Atp1b1 Cav1 Smad3
GO:0051128	regulation of cellular component organization	3.81.E-02	Sptan1 Sptbn1 Vim Epb41l2 Lmna Anxa2 Dpysl2 Fermt2 Ehd2 Dcn H1-3 H1-5 Anxa1 Kank2 Oga Ptk7 Fhl1 Cav1 Hdgfl3 Palm F11r Mthfr Grb2 Stag2 Cd47 Cdkn1b S100a10 Smad3 Pde2a Cldn1 P2rx7
GO:0032970	regulation of actin filament-based process	4.00.E-02	Sptan1 Sptbn1 Dsp Jup Kank2 Cav1 F11r Grb2 Cd47 S100a10 Smad3
GO:0009719	response to endogenous stimulus	4.05.E-02	Vim Fbn1 Col6a1 Anxa2 Anxa5 Alad Fermt2 Fbn2 Anxa3 Jup Anxa1 Kank2 Cav1 Ggt5 Palm Grb2 Nucks1 Cdkn1b Tns2 Smad3 Aspn Txnip Pde2a Cldn1
GO:0051901	positive regulation of mitochondrial depolarization	4.83.E-02	Dcn Oga P2rx7
GO:0062023	collagen-containing extracellular matrix	1.46.E-18	Col6a3 Fbn1 Col6a4 Anxa6 Col6a1 Anxa2 Anxa5 Tgfbi Col6a2 Dcn Fbn2 Nid1 Ctsd Lum Serpinb1a Anxa3 Anxa1 Prelp Ogn Lama5 Mfap4 S100a10 Aspn Thbs3 Dpt

Supplemental Table S3d. Pathway and reactome of up-regulated expressed proteins

term_ID	Terms	p-value	Gene list
KEGG:04512 (KEGG pathway)	ECM-receptor interaction	2.67.E-05	Col6a3 Col6a4 Col6a1 Col6a2 Lama5 Cd47 Itga9 Thbs3
KEGG:04510 (KEGG pathway)	Focal adhesion	1.53.E-03	Col6a3 Col6a4 Col6a1 Col6a2 Lama5 Cav1 Grb2 Itga9 Thbs3
REAC:R-MMU-1474244 (Reactome)	Extracellular matrix organization	1.04.E-06	Col6a3 Fbn1 Col6a1 Col6a2 Dcn Fbn2 Nid1 Ctsd Lum Lama5 Mfap4 Emilin3 F11r Cd47 Itga9
REAC:R-MMU-216083 (Reactome)	Integrin cell surface interactions	2.96.E-05	Col6a3 Fbn1 Col6a1 Col6a2 Lum F11r Cd47 Itga9
REAC:R-MMU-75153 (Reactome)	Apoptotic execution phase	4.76.E-05	Sptan1 Vim Lmnb1 Lmna Dsp H1-3 H1-5
REAC:R-MMU-375165 (Reactome)	NCAM signaling for neurite out-growth	1.18.E-04	Sptan1 Col6a3 Sptbn1 Col6a1 Col6a2 Grb2
REAC:R-MMU-1266738 (Reactome)	Developmental Biology	1.54.E-03	Sptan1 Col6a3 Sptbn1 Col6a1 Dpysl2 Dsp Col6a2 Krt14 Krt5 Jup Krt8 Krt75 Grb2 Pdlim7 Smad3 Itga9
REAC:R-MMU-1500931 (Reactome)	Cell-Cell communication	2.28.E-03	Fermt2 Krt14 Krt5 Jup F11r Grb2 Cd47
REAC:R-MMU-1474228 (Reactome)	Degradation of the extracellular matrix	2.80.E-03	Col6a3 Fbn1 Col6a1 Col6a2 Dcn Fbn2 Nid1 Ctsd
REAC:R-MMU-111465 (Reactome)	Apoptotic cleavage of cellular proteins	3.44.E-03	Sptan1 Vim Lmnb1 Lmna Dsp
REAC:R-MMU-3000178 (Reactome)	ECM proteoglycans	6.74.E-03	Col6a3 Col6a1 Col6a2 Dcn Itga9
REAC:R-MMU-109581 (Reactome)	Apoptosis	8.19.E-03	Sptan1 Vim Lmnb1 Lmna Dsp H1-3 H1-5
REAC:R-MMU-5357801 (Reactome)	Programmed Cell Death	1.26.E-02	Sptan1 Vim Lmnb1 Lmna Dsp H1-3 H1-5
REAC:R-MMU-186797 (Reactome)	Signaling by PDGF	2.45.E-02	Col6a3 Col6a1 Col6a2 Grb2 Thbs3
REAC:R-MMU-2129379 (Reactome)	Molecules associated with elastic fibres	4.12.E-02	Fbn1 Fbn2 Mfap4 Emilin3
REAC:R-MMU-2022857 (Reactome)	Keratan sulfate degradation	4.99.E-02	Lum Prelp Ogn
WP:WP374 (Wiki pathway)	Prostaglandin Synthesis and Regulation	7.62.E-04	Anxa6 Anxa2 Anxa5 Anxa3 S100a10
WP:WP730 (Wiki pathway)	Glutathione and one carbon metabolism	9.12.E-04	Anpep Ggt5 Chdh Gpx3 Mthfr
