# Supplementary material for: Polyubiquitin gene Ubb is required for upregulation of Piwi protein level during mouse testis development
Source: Cell Death Discov. 2021 Jul 26;7:194. doi: 10.1038/s41420-021-00581-2 (PMC8313548; doi:10.1038/s41420-021-00581-2)
Supplement: Supplementary file 5 — Supplementary table 5 [file 41420_2021_581_MOESM5_ESM.pdf]

Table of Contents

Accession	UniProt identifier
FC	Ratio of Ubb K.O reporter ion average intensity versus WT reporter ion average intensity.
	#N/A is a protein that has been identified by LC-MS/MS analysis, but does not show a significant difference in expression level.
p-value	The p-value obtained by performing a student t-test for all WT and K.O replicates.
	#N/A is a protein that has been identified, but is not a protein that can be quantified.
Combined score	The combined score is computed by combining the probabilities from the different evidences and corrected for the probability of randomly observing an interaction

Supplemental Table S5. 296 identified proteins that interact with Ubb.

Accession	Protein name (296)	Gene name	FC	p-value	Combined score
P61222	ATP-binding cassette sub-family E member 1	Abce1	#N/A	5.79.E-01	0.41
P60710	Actin, cytoplasmic 1	Actb	#N/A	9.44.E-01	0.43
Q9JKV1	Proteasomal ubiquitin receptor ADRM1	Adrm1	#N/A	8.41.E-01	0.95
Q9R049	E3 ubiquitin-protein ligase AMFR	Amfr	#N/A	8.38.E-01	0.82
Q3UMR0	Ankyrin repeat domain-containing protein 27	Ankrd27	#N/A	9.08.E-01	0.45
P56480	ATP synthase subunit beta, mitochondrial	Atp5f1b	#N/A	9.09.E-01	0.54
Q9Z1G4	V-type proton ATPase 116 kDa subunit a isoform 1	Atp6v0a1	#N/A	7.68.E-01	0.62
P15920	V-type proton ATPase 116 kDa subunit a isoform 2	Atp6v0a2	#N/A	9.23.E-01	0.62
Q9CVD2	Ataxin-3	Atxn3	#N/A	6.92.E-02	0.81
Q9Z1R2	Large proline-rich protein BAG6	Bag6	#N/A	8.34.E-01	0.42
O70445	BRCA1-associated RING domain protein 1	Bard1	#N/A	#N/A	0.47
Q99MP8	BRCA1-associated protein	Brap	#N/A	6.37.E-01	0.50
P46737	Lys-63-specific deubiquitinase BRCC36	Brcc3	#N/A	8.85.E-01	0.40
Q3ULA2	F-box/WD repeat-containing protein 1A	Btrc	#N/A	#N/A	0.93
P22682	E3 ubiquitin-protein ligase CBL	Cbl	#N/A	6.03.E-01	0.75
Q3TTA7	E3 ubiquitin-protein ligase CBL-B	Cblb	#N/A	9.05.E-01	0.96
Q9JLQ0	CD2-associated protein	Cd2ap	#N/A	8.99.E-01	0.96
Q9JJ66	Cell division cycle protein 20 homolog	Cdc20	#N/A	7.00.E-01	0.74
Q8CFI2	Ubiquitin-conjugating enzyme E2 R1	Cdc34	#N/A	1.47.E-01	0.95
Q60649	Caseinolytic peptidase B protein homolog	Clpb	#N/A	6.81.E-01	0.40
O35864	COP9 signalosome complex subunit 5	Cops5	#N/A	5.32.E-01	0.47
P97784	Cryptochrome-1	Cry1	#N/A	#N/A	0.90
Q9R194	Cryptochrome-2	Cry2	#N/A	#N/A	0.90
Q9WTX6	Cullin-1	Cul1	#N/A	9.25.E-01	0.95
Q9JLV5	Cullin-3	Cul3	#N/A	5.16.E-01	0.42
Q80TQ2	Ubiquitin carboxyl-terminal hydrolase CYLD	Cyld	#N/A	#N/A	0.55
Q9QZ73	DCN1-like protein 1	Dcun1d1	#N/A	9.48.E-01	0.64
Q8BZJ7	DCN1-like protein 2	Dcun1d2	#N/A	5.58.E-01	0.64
A2ADY9	Protein DDI1 homolog 2	Ddi2	#N/A	4.52.E-01	0.64
Q6Q899	Antiviral innate immune response receptor RIG-I	Ddx58	#N/A	8.23.E-01	0.68
E9PUQ8	Diacylglycerol kinase	Dgkd	#N/A	9.65.E-01	0.52
P13864	DNA (cytosine-5)-methyltransferase 1	Dnmt1	#N/A	5.38.E-01	0.79
P10126	Elongation factor 1-alpha 1	Eef1a1	0.88	1.47.E-03	0.65
P58252	Elongation factor 2	Eef2	0.92	1.25.E-02	0.47
P48024	Eukaryotic translation initiation factor 1	Eif1	#N/A	1.92.E-01	0.44
Q9DCH4	Eukaryotic translation initiation factor 3 subunit F	Eif3f	#N/A	7.25.E-01	0.42
Q80VP1	Epsin-1	Epn1	#N/A	5.75.E-01	0.44
Q8CHU3	Epsin-2	Epn2	#N/A	9.96.E-01	0.44
Q91W69	Epsin-3	Epn3	#N/A	#N/A	0.43
P42567	Epidermal growth factor receptor substrate 15	Eps15	#N/A	7.29.E-01	0.64
Q60902	Epidermal growth factor receptor substrate 15-like 1	Eps15l1	#N/A	5.38.E-01	0.64
Q3TPX4	Exocyst complex component 5	Exoc5	#N/A	8.02.E-01	0.45
P09528	Ferritin heavy chain	Fth1	#N/A	7.37.E-01	0.55
P16858	Glyceraldehyde-3-phosphate dehydrogenase	Gapdh	1.14	4.55.E-02	0.42
Q8R0H9	ADP-ribosylation factor-binding protein GGA1	Gga1	#N/A	7.51.E-01	0.69
Q6P5E6	ADP-ribosylation factor-binding protein GGA2	Gga2	#N/A	9.29.E-01	0.42
Q8BMI3	ADP-ribosylation factor-binding protein GGA3	Gga3	#N/A	3.98.E-01	0.66
Q9WU65	Glycerol kinase 2	Gk2	#N/A	2.86.E-01	0.44
E9PW43	Protein transport protein Sec61 subunit beta	Gm10320	#N/A	3.85.E-01	0.43
Q9WV60	Glycogen synthase kinase-3 beta	Gsk3b	#N/A	9.47.E-01	0.91
Q64523	Histone H2A type 2-C	H2ac20	#N/A	5.30.E-01	0.46
Q8BFU2	Histone H2A type 3	H2aw	#N/A	1.80.E-01	0.62
P27661	Histone H2AX	H2ax	#N/A	9.78.E-01	0.69

P70696	Histone H2B type 1-A	H2bc1	#N/A	7.88.E-01	0.52
P84244	Histone H3.3	H3-3b	#N/A	6.45.E-01	0.76
P68433	Histone H3.1	H3c10	#N/A	8.78.E-01	0.94
P62806	Histone H4	H4f16	1.30	3.91.E-02	0.96
Q6P3E7	Polyamine deacetylase HDAC10	Hdac10	#N/A	7.33.E-01	0.56
Q9Z2V5	Histone deacetylase 6	Hdac6	#N/A	7.82.E-01	0.74
P70387	Hereditary hemochromatosis protein homolog	Hfe	#N/A	#N/A	0.72
Q99LI8	Hepatocyte growth factor-regulated tyrosine kinase substrate	Hgs	#N/A	8.52.E-01	0.83
Q8CGP2	Histone H2B type 1-P	Hist1h2bp	#N/A	#N/A	0.56
Q64525	Histone H2B type 2-B	Hist2h2bb	#N/A	1.14.E-01	0.80
P17710	Hexokinase-1	Hk1	#N/A	8.67.E-01	0.44
Q6PCN7	Helicase-like transcription factor	Hltf	#N/A	#N/A	0.42
P07901	Heat shock protein HSP 90-alpha	Hsp90aa1	0.76	6.71.E-04	0.45
P11499	Heat shock protein HSP 90-beta	Hsp90ab1	#N/A	2.53.E-01	0.50
P17879	Heat shock 70 kDa protein 1B	Hspa1b	#N/A	#N/A	0.42
P63017	Heat shock cognate 71 kDa protein	Hspa8	#N/A	4.14.E-01	0.43
Q7TMY8	E3 ubiquitin-protein ligase HUWE1	Huwe1	#N/A	2.12.E-01	0.97
Q9JHR7	Insulin-degrading enzyme	Ide	#N/A	6.20.E-01	0.64
Q8R5F7	Interferon-induced helicase C domain-containing protein 1	Ifih1	#N/A	9.32.E-01	0.46
O88522	NF-kappa-B essential modulator	Ikbkg	#N/A	9.18.E-01	0.65
Q9ES52	Phosphatidylinositol 3,4,5-trisphosphate 5-phosphatase 1	Inpp5d	#N/A	#N/A	0.48
Q8C863	E3 ubiquitin-protein ligase Itchy	Itch	#N/A	8.94.E-01	0.95
Q8VCF0	Mitochondrial antiviral-signaling protein	Mavs	#N/A	5.83.E-02	0.65
Q76LS9	Ubiquitin carboxyl-terminal hydrolase MINDY-1	Mindy1	#N/A	7.19.E-01	0.76
Q99N96	39S ribosomal protein L1, mitochondrial	Mrpl1	#N/A	8.09.E-01	0.41
Q9D1P0	39S ribosomal protein L13, mitochondrial	Mrpl13	#N/A	8.99.E-01	0.46
Q99N93	39S ribosomal protein L16, mitochondrial	Mrpl16	#N/A	7.88.E-01	0.41
Q9D773	39S ribosomal protein L2, mitochondrial	Mrpl2	#N/A	#N/A	0.42
Q9CQ06	39S ribosomal protein L24, mitochondrial	Mrpl24	#N/A	8.29.E-01	0.41
Q80ZK0	28S ribosomal protein S10, mitochondrial	Mrps10	#N/A	1.37.E-01	0.43
Q9DC71	28S ribosomal protein S15, mitochondrial	Mrps15	#N/A	9.17.E-01	0.40
Q80X85	28S ribosomal protein S7, mitochondrial	Mrps7	#N/A	5.95.E-01	0.53
Q64331	Unconventional myosin-VI	Myo6	#N/A	8.22.E-01	0.80
Q69Z66	Histone H2A deubiquitinase MYSM1	Mysm1	#N/A	#N/A	0.42
Q8VBW6	NEDD8-activating enzyme E1 regulatory subunit	Nae1	0.94	2.43.E-02	0.48
P46935	E3 ubiquitin-protein ligase NEDD4	Nedd4	#N/A	2.11.E-01	0.80
Q8CFI0	E3 ubiquitin-protein ligase NEDD4-like	Nedd4l	#N/A	7.75.E-01	0.71
Q6PIJ4	Nuclear factor related to kappa-B-binding protein	Nfrkb	#N/A	1.17.E-01	0.76
P60670	Nuclear protein localization protein 4 homolog	Nploc4	#N/A	5.47.E-01	0.96
Q9CR47	Ribosome biogenesis protein NSA2 homolog	Nsa2	#N/A	8.57.E-01	0.54
Q9QZS3	Protein numb homolog	Numb	#N/A	7.50.E-01	0.90
Q8K3K8	Optineurin	Optn	#N/A	9.14.E-01	0.84
Q7TQI3	Ubiquitin thioesterase OTUB1	Otub1	#N/A	9.74.E-01	0.82
B2RUR8	OTU domain-containing protein 7B	Otud7b	#N/A	#N/A	0.71
Q3UCV8	Ubiquitin thioesterase otulin	Otulin	#N/A	9.72.E-01	0.68
P17918	Proliferating cell nuclear antigen	Pcna	#N/A	3.41.E-01	0.77
P27612	Phospholipase A-2-activating protein	Plaa	#N/A	8.10.E-01	0.87
Q9QXS1	Plectin	Plec	#N/A	1.07.E-01	0.54
Q6R3M4	DNA polymerase iota	Poli	#N/A	#N/A	0.88
Q9WVS6	E3 ubiquitin-protein ligase parkin	Prkn	#N/A	9.07.E-01	0.95
Q9R1P4	Proteasome subunit alpha type-1	Psma1	#N/A	7.96.E-01	0.98
P49722	Proteasome subunit alpha type-2	Psma2	#N/A	9.97.E-01	0.98
O70435	Proteasome subunit alpha type-3	Psma3	#N/A	8.74.E-01	0.98
Q9R1P0	Proteasome subunit alpha type-4	Psma4	#N/A	7.42.E-01	0.98
Q9Z2U1	Proteasome subunit alpha type-5	Psma5	#N/A	8.76.E-01	1.00
Q9QUM9	Proteasome subunit alpha type-6	Psma6	#N/A	6.77.E-01	0.98
Q9Z2U0	Proteasome subunit alpha type-7	Psma7	#N/A	8.71.E-01	0.94

Q9CWH6	Proteasome subunit alpha type-8	Psma8	0.60	1.64.E-02	0.94
O09061	Proteasome subunit beta type-1	Psmb1	#N/A	4.59.E-01	0.97
O35955	Proteasome subunit beta type-10	Psmb10	#N/A	8.24.E-01	0.93
Q9R1P3	Proteasome subunit beta type-2	Psmb2	#N/A	5.44.E-02	0.98
Q9R1P1	Proteasome subunit beta type-3	Psmb3	#N/A	9.82.E-01	0.95
P99026	Proteasome subunit beta type-4	Psmb4	#N/A	1.18.E-01	0.97
O55234	Proteasome subunit beta type-5	Psmb5	#N/A	6.11.E-01	0.97
Q60692	Proteasome subunit beta type-6	Psmb6	#N/A	7.76.E-01	0.95
P70195	Proteasome subunit beta type-7	Psmb7	#N/A	9.90.E-01	0.94
P62192	26S proteasome regulatory subunit 4	Psmc1	#N/A	4.76.E-01	0.96
P46471	26S proteasome regulatory subunit 7	Psmc2	#N/A	5.93.E-01	0.81
O88685	26S proteasome regulatory subunit 6A	Psmc3	#N/A	1.04.E-01	0.61
P54775	26S proteasome regulatory subunit 6B	Psmc4	#N/A	4.41.E-01	0.82
P62196	26S proteasome regulatory subunit 8	Psmc5	#N/A	1.00.E+00	0.97
P62334	26S proteasome regulatory subunit 10B	Psmc6	#N/A	1.51.E-01	0.96
Q3TXS7	26S proteasome non-ATPase regulatory subunit 1	Psmd1	#N/A	4.78.E-01	0.68
Q8BG32	26S proteasome non-ATPase regulatory subunit 11	Psmd11	#N/A	9.34.E-01	0.83
Q9D8W5	26S proteasome non-ATPase regulatory subunit 12	Psmd12	#N/A	5.04.E-01	0.84
Q9WVJ2	26S proteasome non-ATPase regulatory subunit 13	Psmd13	#N/A	6.19.E-01	0.50
O35593	26S proteasome non-ATPase regulatory subunit 14	Psmd14	#N/A	7.93.E-01	0.97
Q8VDM4	26S proteasome non-ATPase regulatory subunit 2	Psmd2	0.90	2.34.E-02	0.69
P14685	26S proteasome non-ATPase regulatory subunit 3	Psmd3	#N/A	6.23.E-01	0.74
O35226	26S proteasome non-ATPase regulatory subunit 4	Psmd4	0.91	4.26.E-02	0.76
Q99JI4	26S proteasome non-ATPase regulatory subunit 6	Psmd6	#N/A	5.24.E-01	0.69
P26516	26S proteasome non-ATPase regulatory subunit 7	Psmd7	#N/A	7.66.E-01	0.82
Q9CX56	26S proteasome non-ATPase regulatory subunit 8	Psmd8	#N/A	3.86.E-01	0.73
O35551	Rab GTPase-binding effector protein 1	Rabep1	#N/A	5.13.E-01	0.67
Q9JM13	Rab5 GDP/GTP exchange factor	Rabgef1	#N/A	6.22.E-01	0.81
P68040	Receptor of activated protein C kinase 1	Rack1	#N/A	6.64.E-02	0.60
Q9QXK2	E3 ubiquitin-protein ligase RAD18	Rad18	#N/A	2.99.E-01	0.84
P54726	UV excision repair protein RAD23 homolog A	Rad23a	#N/A	9.36.E-01	0.97
P54728	UV excision repair protein RAD23 homolog B	Rad23b	#N/A	5.29.E-01	0.76
Q9WUB0	RanBP-type and C3HC4-type zinc finger-containing protein 1	Rbck1	#N/A	8.70.E-01	0.70
P62878	E3 ubiquitin-protein ligase RBX1	Rbx1	#N/A	2.55.E-01	0.93
Q920Q2	DNA repair protein REV1	Rev1	#N/A	5.52.E-01	0.41
Q9WTV7	E3 ubiquitin-protein ligase RLIM	Rlim	#N/A	#N/A	0.46
Q924T7	E3 ubiquitin-protein ligase RNF31	Rnf31	#N/A	7.96.E-01	0.67
P53026	60S ribosomal protein L10a	Rpl10a	#N/A	4.06.E-01	0.63
Q9CXW4	60S ribosomal protein L11	Rpl11	#N/A	4.67.E-01	0.43
P35979	60S ribosomal protein L12	Rpl12	#N/A	3.31.E-01	0.47
P47963	60S ribosomal protein L13	Rpl13	0.84	3.19.E-02	0.52
Q9CR57	60S ribosomal protein L14	Rpl14	#N/A	6.06.E-01	0.42
Q9CZM2	60S ribosomal protein L15	Rpl15	0.85	1.95.E-02	0.53
Q9CPR4	60S ribosomal protein L17	Rpl17	#N/A	4.10.E-01	0.44
P35980	60S ribosomal protein L18	Rpl18	#N/A	4.72.E-01	0.56
P62717	60S ribosomal protein L18a	Rpl18a	#N/A	6.58.E-01	0.48
P84099	60S ribosomal protein L19	Rpl19	#N/A	8.41.E-01	0.84
O09167	60S ribosomal protein L21	Rpl21	#N/A	1.25.E-01	0.45
P67984	60S ribosomal protein L22	Rpl22	0.84	2.93.E-03	0.47
Q9D7S7	60S ribosomal protein L22-like 1	Rpl22l1	#N/A	6.25.E-01	0.47
P62830	60S ribosomal protein L23	Rpl23	0.83	1.29.E-03	0.56
P62751	60S ribosomal protein L23a	Rpl23a	#N/A	5.62.E-01	0.57
Q8BP67	60S ribosomal protein L24	Rpl24	#N/A	7.55.E-02	0.44
P61255	60S ribosomal protein L26	Rpl26	#N/A	5.70.E-01	0.64
P61358	60S ribosomal protein L27	Rpl27	#N/A	3.10.E-01	0.50
P14115	60S ribosomal protein L27a	Rpl27a	#N/A	6.37.E-01	0.42
P41105	60S ribosomal protein L28	Rpl28	#N/A	8.49.E-01	0.45

P27659	60S ribosomal protein L3	Rpl3	0.84	1.70.E-03	0.46
P62889	60S ribosomal protein L30	Rpl30	#N/A	7.76.E-01	0.56
P62900	60S ribosomal protein L31	Rpl31	#N/A	3.09.E-01	0.55
P62911	60S ribosomal protein L32	Rpl32	#N/A	9.08.E-02	0.56
Q9D1R9	60S ribosomal protein L34	Rpl34	0.87	1.08.E-02	0.48
Q6ZWV7	60S ribosomal protein L35	Rpl35	#N/A	2.78.E-01	0.59
O55142	60S ribosomal protein L35a	Rpl35a	#N/A	8.59.E-01	0.55
P47964	60S ribosomal protein L36	Rpl36	#N/A	3.83.E-01	0.44
Q9D823	60S ribosomal protein L37	Rpl37	#N/A	6.69.E-01	0.49
P61514	60S ribosomal protein L37a	Rpl37a	#N/A	7.09.E-01	0.76
Q9JJI8	60S ribosomal protein L38	Rpl38	#N/A	7.86.E-01	0.55
P62892	60S ribosomal protein L39	Rpl39	#N/A	2.63.E-01	0.50
Q9CQD0	Ribosomal protein L39-like	Rpl39l	#N/A	#N/A	0.43
E9PWZ3	Ribosomal protein L3-like	Rpl3l	#N/A	5.74.E-01	0.48
Q9D8E6	60S ribosomal protein L4	Rpl4	0.77	1.76.E-03	0.46
P47962	60S ribosomal protein L5	Rpl5	#N/A	2.53.E-01	0.51
P47911	60S ribosomal protein L6	Rpl6	0.86	8.99.E-03	0.68
P14148	60S ribosomal protein L7	Rpl7	#N/A	3.45.E-01	0.52
P12970	60S ribosomal protein L7a	Rpl7a	0.77	4.59.E-04	0.41
P62918	60S ribosomal protein L8	Rpl8	#N/A	6.88.E-01	0.85
P51410	60S ribosomal protein L9	Rpl9	#N/A	3.97.E-01	0.66
P14869	60S acidic ribosomal protein P0	Rplp0	#N/A	3.79.E-01	0.40
P47955	60S acidic ribosomal protein P1	Rplp1	#N/A	2.72.E-01	0.61
P63325	40S ribosomal protein S10	Rps10	#N/A	5.50.E-01	0.43
P62281	40S ribosomal protein S11	Rps11	#N/A	6.83.E-01	0.59
P63323	40S ribosomal protein S12	Rps12	#N/A	4.14.E-01	0.40
P62301	40S ribosomal protein S13	Rps13	#N/A	5.12.E-01	0.60
P62264	40S ribosomal protein S14	Rps14	#N/A	6.81.E-01	0.59
P62843	40S ribosomal protein S15	Rps15	#N/A	8.82.E-01	0.51
P62245	40S ribosomal protein S15a	Rps15a	0.88	3.76.E-02	0.55
P14131	40S ribosomal protein S16	Rps16	#N/A	1.54.E-01	0.73
P63276	40S ribosomal protein S17	Rps17	#N/A	8.61.E-02	0.59
P62270	40S ribosomal protein S18	Rps18	#N/A	5.81.E-01	0.56
Q9CZX8	40S ribosomal protein S19	Rps19	0.88	4.01.E-03	0.58
P25444	40S ribosomal protein S2	Rps2	#N/A	8.02.E-01	0.58
P60867	40S ribosomal protein S20	Rps20	#N/A	2.97.E-01	0.69
Q9CQR2	40S ribosomal protein S21	Rps21	#N/A	4.17.E-01	0.56
P62267	40S ribosomal protein S23	Rps23	#N/A	2.42.E-01	0.66
P62849	40S ribosomal protein S24	Rps24	#N/A	9.53.E-01	0.47
P62852	40S ribosomal protein S25	Rps25	#N/A	9.45.E-02	0.58
P62855	40S ribosomal protein S26	Rps26	#N/A	2.05.E-01	0.59
Q6ZWU9	40S ribosomal protein S27	Rps27	#N/A	#N/A	0.53
P62983	Ubiquitin-40S ribosomal protein S27a	Rps27a	#N/A	9.10.E-01	0.97
Q6ZWY3	40S ribosomal protein S27-like	Rps27l	#N/A	7.90.E-01	0.46
P62858	40S ribosomal protein S28	Rps28	#N/A	7.62.E-01	0.54
P62274	40S ribosomal protein S29	Rps29	#N/A	8.14.E-01	0.72
P62908	40S ribosomal protein S3	Rps3	#N/A	3.84.E-01	0.61
P97351	40S ribosomal protein S3a	Rps3a	#N/A	6.78.E-01	0.73
P62702	40S ribosomal protein S4, X isoform	Rps4x	#N/A	3.87.E-01	0.66
P97461	40S ribosomal protein S5	Rps5	#N/A	6.46.E-01	0.53
P62754	40S ribosomal protein S6	Rps6	0.82	3.33.E-03	0.54
P62082	40S ribosomal protein S7	Rps7	#N/A	5.17.E-01	0.54
P62242	40S ribosomal protein S8	Rps8	#N/A	1.62.E-01	0.74
Q6ZWN5	40S ribosomal protein S9	Rps9	#N/A	1.25.E-01	0.57
P14206	40S ribosomal protein SA	Rpsa	0.91	2.13.E-02	0.48
Q9DBU6	Serine/Arginine-related protein 53	Rsrc1	#N/A	2.01.E-01	0.40
P61620	Protein transport protein Sec61 subunit alpha isoform 1	Sec61a1	#N/A	6.59.E-01	0.40

Q9JLR1	Protein transport protein Sec61 subunit alpha isoform 2	Sec61a2	#N/A	7.17.E-01	0.40
P60060	Protein transport protein Sec61 subunit gamma	Sec61g	#N/A	#N/A	0.48
Q8R550	SH3 domain-containing kinase-binding protein 1	Sh3kbp1	#N/A	9.69.E-01	0.78
Q9WTX5	S-phase kinase-associated protein 1	Skp1	#N/A	2.02.E-01	0.94
A2A5Z6	E3 ubiquitin-protein ligase SMURF2	Smurf2	#N/A	9.55.E-01	0.95
P70297	Signal transducing adapter molecule 1	Stam	#N/A	4.60.E-01	0.81
O88811	Signal transducing adapter molecule 2	Stam2	#N/A	9.99.E-01	0.77
Q9CQ26	STAM-binding protein	Stambp	#N/A	7.02.E-01	0.52
Q60864	Stress-induced-phosphoprotein 1	Stip1	#N/A	1.01.E-01	0.41
P61957	Small ubiquitin-related modifier 2	Sumo2	#N/A	5.76.E-01	0.46
Q9Z172	Small ubiquitin-related modifier 3	Sumo3	#N/A	6.15.E-01	0.43
Q9DBY1	E3 ubiquitin-protein ligase synoviolin	Syvn1	#N/A	4.49.E-01	0.44
Q571K4	TGF-beta-activated kinase 1 and MAP3K7-binding protein 3	Tab3	#N/A	6.24.E-01	0.96
Q3UKC1	Tax1-binding protein 1 homolog	Tax1bp1	#N/A	2.53.E-01	0.42
O88746	Target of Myb protein 1	Tom1	#N/A	8.36.E-01	0.70
Q5SRX1	TOM1-like protein 2	Tom1l2	#N/A	8.89.E-01	0.62
P70399	TP53-binding protein 1	Tp53bp1	#N/A	7.23.E-01	0.96
P63028	Translationally-controlled tumor protein	Tpt1	#N/A	9.47.E-01	0.45
P70196	TNF receptor-associated factor 6	Traf6	#N/A	4.93.E-01	0.60
Q62191	E3 ubiquitin-protein ligase TRIM21	Trim21	#N/A	1.41.E-01	0.49
Q61510	E3 ubiquitin/ISG15 ligase TRIM25	Trim25	#N/A	7.53.E-01	0.46
G5E870	E3 ubiquitin-protein ligase TRIP12	Trip12	#N/A	2.58.E-01	0.51
Q61187	Tumor susceptibility gene 101 protein	Tsg101	#N/A	5.22.E-01	0.81
Q02053	Ubiquitin-like modifier-activating enzyme 1	Uba1	#N/A	1.55.E-01	0.93
P31254	Ubiquitin-like modifier-activating enzyme 1 Y	Uba1y	#N/A	4.69.E-01	0.92
Q9Z1F9	SUMO-activating enzyme subunit 2	Uba2	#N/A	7.36.E-01	0.43
P62984	Ubiquitin-60S ribosomal protein L40	Uba52	#N/A	#N/A	0.94
Q8C7R4	Ubiquitin-like modifier-activating enzyme 6	Uba6	#N/A	9.19.E-01	0.59
Q9Z255	Ubiquitin-conjugating enzyme E2 A	Ube2a	#N/A	5.67.E-01	0.43
P63147	Ubiquitin-conjugating enzyme E2 B	Ube2b	#N/A	7.82.E-01	0.49
Q9D1C1	Ubiquitin-conjugating enzyme E2 C	Ube2c	#N/A	#N/A	0.44
P61080	Ubiquitin-conjugating enzyme E2 D1	Ube2d1	#N/A	#N/A	0.97
P62838	Ubiquitin-conjugating enzyme E2 D2	Ube2d2	#N/A	#N/A	0.97
P61079	Ubiquitin-conjugating enzyme E2 D3	Ube2d3	#N/A	#N/A	0.97
Q91W82	Ubiquitin-conjugating enzyme E2 E2	Ube2e2	#N/A	#N/A	0.46
P52483	Ubiquitin-conjugating enzyme E2 E3	Ube2e3	#N/A	8.55.E-01	0.46
P62254	Ubiquitin-conjugating enzyme E2 G1	Ube2g1	#N/A	8.15.E-01	0.52
P60605	Ubiquitin-conjugating enzyme E2 G2	Ube2g2	#N/A	6.07.E-01	0.49
P62257	Ubiquitin-conjugating enzyme E2 H	Ube2h	#N/A	9.70.E-01	0.48
P63280	SUMO-conjugating enzyme UBC9	Ube2i	#N/A	9.45.E-01	0.46
P61087	Ubiquitin-conjugating enzyme E2 K	Ube2k	#N/A	7.94.E-01	0.98
P68037	Ubiquitin-conjugating enzyme E2 L3	Ube2l3	#N/A	7.20.E-01	0.99
P61089	Ubiquitin-conjugating enzyme E2 N	Ube2n	#N/A	6.01.E-01	0.98
Q6ZWZ2	Ubiquitin-conjugating enzyme E2 R2	Ube2r2	#N/A	7.52.E-01	0.75
Q921J4	Ubiquitin-conjugating enzyme E2 S	Ube2s	#N/A	9.08.E-01	0.82
Q9CZY3	Ubiquitin-conjugating enzyme E2 variant 1	Ube2v1	#N/A	9.80.E-01	0.74
Q9D2M8	Ubiquitin-conjugating enzyme E2 variant 2	Ube2v2	#N/A	5.65.E-01	0.81
Q8R317	Ubiquilin-1	Ubqln1	#N/A	7.14.E-01	0.62
Q9QZM0	Ubiquilin-2	Ubqln2	#N/A	4.92.E-01	0.59
Q99NB8	Ubiquilin-4	Ubqln4	#N/A	7.68.E-01	0.54
Q80TP3	E3 ubiquitin-protein ligase UBR5	Ubr5	#N/A	9.97.E-01	0.96
Q9R0P9	Ubiquitin carboxyl-terminal hydrolase isozyme L1	Uchl1	#N/A	4.37.E-01	0.72
Q9JKB1	Ubiquitin carboxyl-terminal hydrolase isozyme L3	Uchl3	#N/A	7.91.E-01	0.77
Q9WUP7	Ubiquitin carboxyl-terminal hydrolase isozyme L5	Uchl5	0.89	1.10.E-02	0.87
Q5U5Q9	BRCA1-A complex subunit RAP80	Uimc1	#N/A	9.04.E-01	0.94
Q9D2P4	Ubiquitin-related modifier 1	Urm1	#N/A	8.57.E-01	0.41
Q9JMA1	Ubiquitin carboxyl-terminal hydrolase 14	Usp14	#N/A	7.38.E-01	0.79

Q5I043	Ubiquitin carboxyl-terminal hydrolase 28	Usp28	#N/A	5.67.E-01	0.50
Q3UN04	Ubiquitin carboxyl-terminal hydrolase 30	Usp30	#N/A	9.33.E-01	0.76
Q8BWR4	Ubiquitin carboxyl-terminal hydrolase 40	Usp40	0.89	1.11.E-02	0.58
P62069	Ubiquitin carboxyl-terminal hydrolase 46	Usp46	#N/A	7.97.E-01	0.96
P56399	Ubiquitin carboxyl-terminal hydrolase 5	Usp5	#N/A	2.60.E-01	0.85
Q6A4J8	Ubiquitin carboxyl-terminal hydrolase 7	Usp7	#N/A	6.59.E-01	0.82
Q80U87	Ubiquitin carboxyl-terminal hydrolase 8	Usp8	#N/A	7.81.E-01	0.65
Q01853	Transitional endoplasmic reticulum ATPase	Vcp	#N/A	3.47.E-01	0.49
Q91XD6	Vacuolar protein-sorting-associated protein 36	Vps36	#N/A	7.78.E-01	0.87
Q8BH57	WD repeat-containing protein 48	Wdr48	#N/A	1.13.E-01	0.81
Q9DBH0	NEDD4-like E3 ubiquitin-protein ligase WWP2	Wwp2	#N/A	#N/A	0.52
Q8CB27	Ubiquitin thioesterase OTU1	Yod1	#N/A	8.99.E-01	0.78
Q91X58	AN1-type zinc finger protein 2B	Zfand2b	#N/A	#N/A	0.64
Q810J8	Zinc finger FYVE domain-containing protein 1	Zfyve1	#N/A	9.85.E-01	0.41
Q9DAZ9	Abscission/NoCut checkpoint regulator	Zfyve19	#N/A	9.74.E-01	0.41
